# Global landscape of autoimmune diseases across different lifespan: A three-decade perspective

**DOI:** 10.1097/MD.0000000000047140

**Published:** 2026-01-09

**Authors:** Xian-Pei Xiao, Ming-Yu Wu, Yan Li, Xiao Hu, Bi-Yuan Qin

**Affiliations:** a Department of Critical Care Medicine, Luojiang District People’s Hospital of Deyang City, Deyang, Sichuan Province, China; b Department of Nephrology, Luojiang District People’s Hospital of Deyang City, Deyang, Sichuan Province, China; c Department of Health Service and Management, Chengdu International Studies University, Chengdu, Sichuan, China; d Department of Science and Education, Deyang People’s Hospital, Deyang, Sichuan, China.

**Keywords:** autoimmune disease, burden of disease, prevalence rate

## Abstract

Autoimmune diseases (ADs) are immune-mediated disorders characterized by a malfunction of the immune system, leading to inflammation, organ dysfunction, and a significant impact on patients’ quality of life. This study aimed to comprehensively evaluate the dynamic trends in the prevalence of ADs across geographies, life cycles, and sex, and to project the prevalence up to 2050. We utilized data from the Global Burden of Diseases, Injuries, and Risk Factors Study 2021 to estimate the age-standardized prevalence rate of ADs. We assessed the dynamic trends from 1990 to 2021 by calculating the average annual percentage change using linkage regression analysis and evaluated age–period–cohort effects. The prevalence of ADs up to 2050 was projected using a mixed-effects model. The global age-standardized prevalence rate of ADs nearly doubled from 1990 to 2021. We observed a notable increase in type 1 diabetes, downward trends in inflammatory bowel disease, multiple sclerosis, and psoriasis, and stable trends in alopecia areata, rheumatic heart disease, and rheumatoid arthritis. The disease burden was lower in childhood but higher in young and middle-aged adults, particularly among females. Age–period–cohort analysis revealed that the risk of ADs increased with age across all regions. Our model projected an upward trend in prevalence from 2022 to 2032, followed by a gradual decline until 2050. Our findings highlight the need for tailored health policies and resource allocation to address the distinct needs of patients with ADs across different life cycles and gender groups, which is crucial for mitigating the projected future challenges to healthcare system sustainability.

## 1. Introduction

Autoimmune diseases (ADs) are a group of immune-mediated inflammatory disorders characterized by the loss of immunological tolerance to self-antigens, resulting in immune regulatory dysfunction and chronic systemic inflammation, leading to a cascade of damages involved in multiple tissues and organs.^[1]^ To date, there are >100 distinct ADs, the common types of ADs include type 1 diabetes (T1D), inflammatory bowel disease (IBD), rheumatoid arthritis (RA), etc. It has been estimated that ADs affect approximately 8% to 10% of the population worldwide.^[2,3]^ Due to the higher risks of developing infection, disabilities, and severe complications, ADs have a significant impact on the physical and mental health of patients and their families, imposing a heavy burden on society and healthcare costs worldwide.

ADs are heterogeneous conditions with various pathologies, and the exact mechanisms remain obscure. Accumulating evidence has implicated that age, genetic susceptibility, environmental modifiable factors, and the levels of hormones, and their complex interactions are thought to contribute to the onset and pathogenesis of ADs.^[4]^ Despite the complex etiology of these diseases, numerous studies have demonstrated the presence of a sex-specific pattern in the incidence and prevalence of ADs, with most cases being more prevalent in women. This pattern has been attributed, at least in part, to the influence of sex hormones, though direct causal evidence from population-level studies remains limited.^[5,6]^

The levels of sex hormones represent distinct fluctuations across life cycles, they typically reach a first peak immediately after birth, gradually decline during childhood, and then undergo a marked increase during puberty and adolescence. Following this, there is typically a period of relatively stable hormone levels during young and middle adulthood. However, in old age, there is a gradual decline in the production of sex hormones.^[7–10]^ A prior study has implied the heavy disease burden of ADs in childbearing-aged women, and the frequency and severity of disease attacks decreased significantly in middle and old age.^[11]^ Nevertheless, the prevalence and disease burden of ADs in males and females across life cycles are still undetermined. Given the postulated close relationship between sex hormones and the onset of ADs, it is imperative to gain a comprehensive understanding of the sex-specific disease burden of ADs across different life cycles from an epidemiological perspective.

The Global Burden of Diseases, Injuries, and Risk Factors Study (GBD) provides a systematic assessment of the impact of diseases, injuries, and risk factors on public health both globally and regionally. In the present study, we used data from GBD 2021 to systemically evaluate the dynamic trends in the prevalence of ADs across geographies, life cycles, and sex. We focused on 7 specific ADs (alopecia areata [AA], T1D, IBD, multiple sclerosis [MS], psoriasis, RA, and rheumatic heart disease [RHD]). These conditions were selected based on their significant contribution to the global burden of ADs, as reflected in the GBD database, considering factors such as high prevalence rates, substantial disability-adjusted life years, and their representation of diverse organ systems and pathophysiological mechanisms. Furthermore, these 7 ADs exhibit distinct patterns across age and sex, making them suitable for investigating our research objectives related to life cycles and gender disparities. Then, we calculated the age–period–cohort (APC) effects of ADs. Moreover, the age-standardized prevalence rate (ASPR) prediction of ADs up to 2050 was also conducted. This study could offer insights into the underlying causes and epidemiological characteristics of ADs, which would be helpful to inform disease management strategies, and allocate resources effectively to address the growing burden of these diseases.

## 2. Materials and methods

### 2.1. Study population and data collection

The GBD 2021 system, a reputable source of global health data, compiles estimated data on 369 diseases/injuries, and 87 health risk factors from 204 countries, regions, and globally. The data extraction regarding the prevalence and estimates of ADs from GBD 2021 was implemented by utilizing the Global Health Exchange tool. An uncertainty interval (UI) is a range of values, derived from a statistical analysis, that is likely to contain the true value of an unknown population parameter. Following the GBD estimate algorithm, all rates are reported per 100,000 population. In the present study, the study population was selected those individuals aged from 0 to 69 years, which were used to represent distinct fluctuations of sex hormones across life cycles. Given the apparent change models of sex hormones, the age of the study population was divided into 4 subgroups, including childhood (0–9 years), adolescence (10–19 years), young and middle-aged population (15–49 years), and middle-aged and elderly population (45–69 years). These age groups were defined to reflect key physiological and hormonal transition periods, despite partial overlap between the young and middle-aged (15–49 years) and middle-aged and elderly (45–69 years) groups. The overlap was intentionally retained to capture the continuum of hormonal changes and disease onset patterns across the lifespan, as these transitions are not discrete. To minimize analytical ambiguity, all statistical models were stratified by these age groups and adjusted for age as a continuous variable where appropriate. The prevalence of ADs in our study was based on the availability of data in the GBD 2021 database, which affects a significant proportion of the population, indicating a substantial disease burden.

The primary data sources of GBD 2021 consist of cross-sectional studies worldwide, hospital data for both outpatients and inpatients, community surveillance, health insurance records, health service utilization data, and additional information provided by the GBD collaborators network. Data on ADs in GBD 2021 are accessed through the Global Health Exchange tool data input source tool, which includes estimates on incidence, prevalence, mortality, disability-adjusted life years, as well as GBD demographic data and life tables. The detailed information can be found in Appendix of the GBD 2021.^[12]^

The World Health Organization (WHO) categorizes the world into 6 major regions, including Africa (47 countries), the Americas (39 countries), Southeast Asia (11 countries), Europe (53 countries), the Eastern Mediterranean (21 countries), and the Western Pacific (37 countries). These regions are categorized based on similarities in epidemiological trends, such as disease prevalence and healthcare access, as well as geographical proximity and cultural similarities, which can help monitor health status and trends at regional and global levels. This study will investigate various levels, ranging from global trends to regional and national health statuses, allowing for a comprehensive analysis of health disparities and policy impacts. Although this study exclusively utilized the GBD 2021 database, it is important to note that the GBD system itself synthesizes a wide range of data sources, including national health registries, hospital records, and survey data from over 204 countries and territories. The GBD methodology employs rigorous statistical modeling to ensure internal consistency and comparability across regions and time periods. Therefore, while external validation using independent dataset was not performed, the GBD estimates are already harmonized and validated within the GBD framework, making them suitable for cross-national and temporal trend analyses such as those conducted in this study.

### 2.2. Statistical analysis

To standardize for population age structure and compare the disease prevalence burden of ADs across different life cycles, the direct method was employed to estimate the ASPR. The number and rate of affected individuals per 100,000 population were obtained from GBD 2021 and stratified by age. These ratios are assumed to be weighted and distributed as independent Poisson random variables, which means that each ratio is treated as an independent Poisson random variable with its probability distribution, and these variables are then combined to estimate the overall ASPR. The ASPR was calculated using the direct method formula:


$$\begin{array}{l} \frac{\overset{N}{\underset{i=1}{\sum }}\alpha_{i}\omega_{i}}{\overset{N}{\underset{i=1}{\sum }}\omega_{i}} \end{array}$$

Among the age groups, N represents the number of age groups; α_*i*_ represents the prevalence of the *i*-age cycle; ω_*i*_ represents the population proportion of each age group in the GBD standard population. The 95% UI is derived from the 25th and 975th values of the 1000 ordered estimates in the GBD model, reflecting the range of uncertainty around the point estimate.

To evaluate the average change trend in ASPR of ADs over a specific period, the JoinPoint model was employed to determine the overall average annual percentage change (AAPC) along with its 95% confidence interval (CI).^[13]^ The process typically involves 3 main steps. Initially, a logarithmic linear model was utilized for piecewise regression:


$$\begin{array}{l} y=\;\alpha +\;\beta x+\;\varepsilon \end{array}$$

*y* is equal to the ln (age-standardized rate), χ refers to the calendar year.


$$\begin{array}{l} APC=\left(e^{\beta }-1\right)\times 100 \end{array}$$

The APC of ADs between each turning point was calculated by weighting the segment interval span *w* and then summing to obtain the AAPC. Subsequently, a linear regression model was utilized to estimate the 95% CI of AAPC. In general, if both the estimated AAPC value and the lower limit of the 95% CI are >0, it suggests an upward trend in the ASPR over time. Conversely, if both the AAPC value and the upper limit of the 95% CI are <0, the ASPR may be declining over time. Otherwise, the trend is deemed stable.

We applied the online APC model framework (https://analysistools.cancer.gov/apc/) to analyze epidemic trends, accounting for age, period, and cohort effects. This tool enables researchers to input data and fit statistical models that decompose trends into these 3 components. A key challenge in APC analysis is the perfect collinearity among variables (birth cohort = period ‐ age), which can bias estimates in traditional linear models.^[14]^ To mitigate this, we used the endogenous factor estimation method, which avoids arbitrary constraints and accounts for variable interdependence, thereby producing estimable functions.^[15]^ The model assumes linear effects for age, period, and cohort, and represents period–cohort effects as relative risks compared to a reference. Statistical significance was set at a two-sided *P*-value < .05. Further details are available in prior literature.^[16]^

To forecast the disease burden of ADs from 2022 to 2050, we employed the Bayesian age–period–cohort model, which uses Bayesian inference to estimate age, period, and cohort effects.^[17]^ The model incorporates prior distributions for parameters; we specified non-informative priors (e.g., normal distributions with mean zero and large variance) to minimize prior influence and allow data-driven estimates. The Bayesian age–period–cohort model approach handles collinearity through Bayesian shrinkage and assumes linear trends unless otherwise specified. We integrated the predicted prevalence with population projections to estimate global case numbers and ASPR by 2050. All analyses were performed in R software (Version 4.2.2; R Foundation for Statistical Computing, Vienna, Austria).

## 3. Results

### 3.1. Alopecia areata

#### 3.1.1. The estimate of ASPR and AAPC

**Figure 1.**
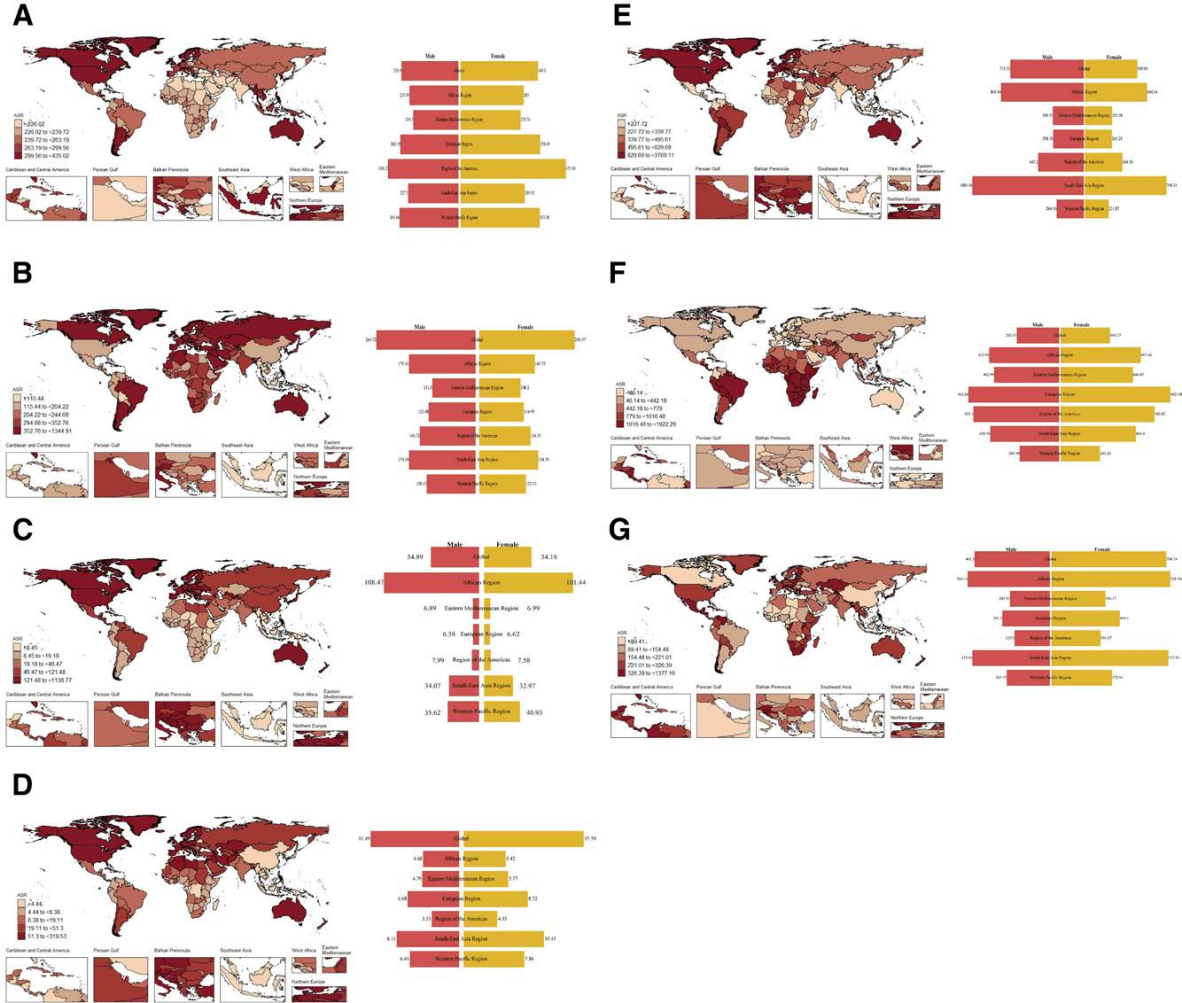
The age-standardized prevalence rate of ADs at the global in 2021. (A) AA; (B) T1D; (C) IBD; (D) MS; (E) psoriasis; (F) RHD; (G) RA. Maps were generated using R software (version 4.2.2; https://www.r-project.org/) with the ggplot2, rnaturalearth, and sf packages. AA = alopecia areata; ADs = autoimmune diseases; IBD = inflammatory bowel disease; MS = multiple sclerosis; RHD = rheumatic heart disease; RA = rheumatoid arthritis; T1D = diabetes mellitus type 1.

**Figure 2.**
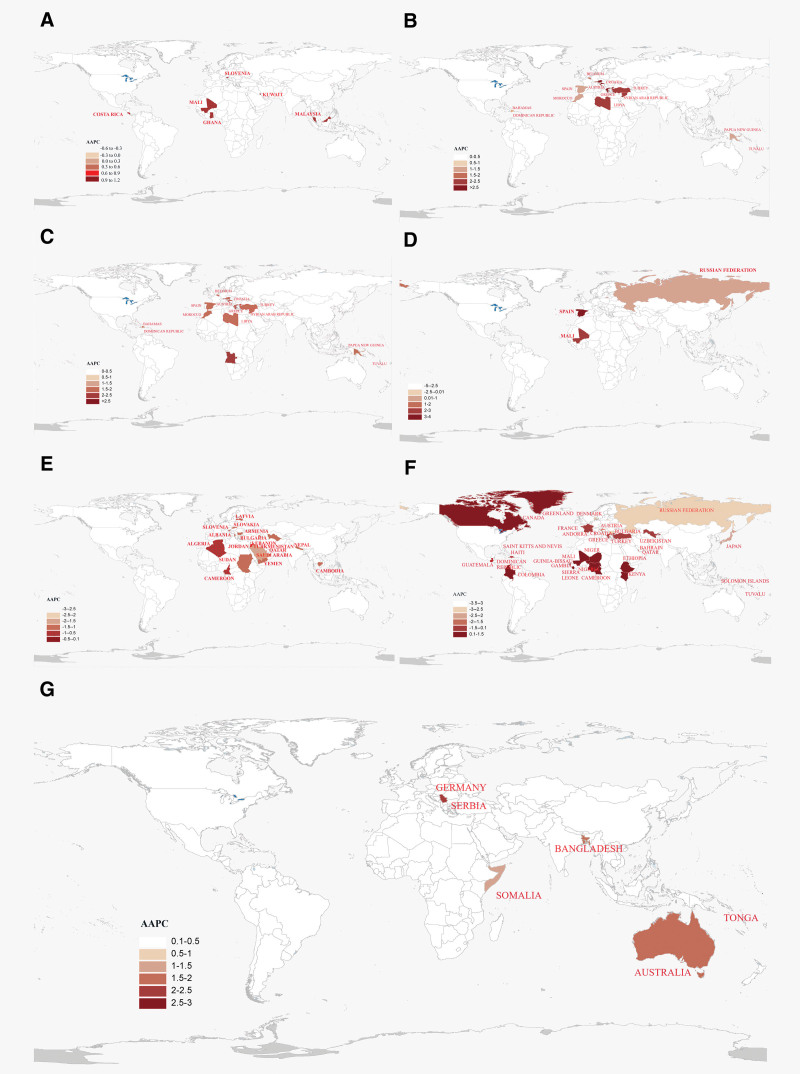
The sex-specific comparisons of global risk of ADs in different age groups from 1990 to 2021. (A) AA; (B) T1D; (C) IBD; (D) MS; (E) psoriasis; (F) RHD; (G) RA. Maps were generated using R software (version 4.2.2; https://www.r-project.org/) with the ggplot2, rnaturalearth, and sf packages. AA = alopecia areata; ADs = autoimmune diseases; IBD = inflammatory bowel disease; MS = multiple sclerosis; RA = rheumatoid arthritis; RHD = rheumatic heart disease; T1D = diabetes mellitus type 1.

In 2021, the global ASPR of AA was 131.94 (95% UI: 118.64–146.27). The highest ASPR was observed in Southeast Asia at the regional level (577.94, 95% UI: 356.11–889.92), and in the United States of America at the national level (198.51, 95% UI: 182.07–216.49). In terms of dynamic changes of AA burden across life cycles, the ASPR exhibited a “U” shape pattern from adolescence (177.46, 95% UI: 158.99–196.65) to young and middle-aged cycles (224.73, 95% UI: 201.65–248.58), and then decreased in the middle and old-aged cycles (162.81, 95% UI: 145.42–180.70). In addition, we found that the ASPR of AA was higher in males than females during adolescence (Val: 255.55 vs 0.00) (Table 1; Table S1, Supplemental Digital Content, https://links.lww.com/MD/R133; Fig. 1A). Despite population growth, the AAPC estimated trend of AA in the 0 to 69 years old remained stable globally and regionally from 1990 to 2021. However, the AAPC of other 3 life cycles all showed a downward trend, which decreased by 0.12% in childhood (95% CI: ‐0.13% to ‐0.11%), 0.14% in young and middle-aged cycles (95% CI: ‐0.15% to ‐0.14%), 0.02% in middle and old-aged years (95% CI: ‐0.02% to ‐0.03%) (Table 2; Table S2, Supplemental Digital Content, https://links.lww.com/MD/R133; Fig. 2A).

**Table 1:** Age-standardized prevalence rates of autoimmune diseases in childhood, adolescence, young and middle-age, and middle-aged and elderly period at the global and regional levels in 2021.

Measure	Sex	Location	Cause	All	Childhood	Adolescence	Young and middle-age	Middle-aged and elderly
Prevalence	Male	Global	AA	96.39 (86.56–106.8)	0 (0–0)	255.55 (229.23–282.9)	314.62 (282.55–347.65)	256.9 (230.59–284.8)
Prevalence	Female	Global	AA	169.55 (152.58–188)	0 (0–0)	0 (0–0)	406.31 (364.66–449.22)	349.3 (312.52–387.98)
Prevalence	Both	Global	AA	131.94 (118.64–146.27)	0 (0–0)	177.46 (158.99–196.65)	224.73 (201.65–248.58)	162.81 (145.42–180.7)
Prevalence	Male	African Region	AA	169.84 (115.79–232.26)	0 (0–0)	230.01 (205.73–255.26)	286.68 (256.93–317.48)	220.89 (197.17–246.21)
Prevalence	Female	African Region	AA	171.99 (117.62–233.75)	0 (0–0)	0 (0–0)	369.41 (330.46–410.1)	285 (253.77–317.36)
Prevalence	Both	African Region	AA	170.88 (116.87–233.39)	0 (0–0)	159.8 (142.57–177.43)	198.81 (177.65–220.42)	152.03 (134.97–170.09)
Prevalence	Male	Eastern Mediterranean Region	AA	6.40 (5.19–7.9)	0 (0–0)	0 (0–0)	266.02 (237.9–294.47)	204.1 (181.7–228.16)
Prevalence	Female	Eastern Mediterranean Region	AA	6.94 (5.68–8.48)	0 (0–0)	282.18 (251.17–314.67)	351.42 (312.99–390.89)	270.76 (240.17–304.08)
Prevalence	Both	Eastern Mediterranean Region	AA	6.66 (5.42–8.18)	0 (0–0)	0 (0–0)	187.82 (167.65–209.21)	144.11 (127.85–161.28)
Prevalence	Male	European Region	AA	1.24 (0.78–1.87)	0 (0–0)	294.96 (262.6–326.98)	328.34 (294.64–363.22)	262.19 (234.7–291.53)
Prevalence	Female	European Region	AA	2.18 (1.43–3.17)	0 (0–0)	321.56 (287.35–357.31)	395.18 (353.42–438.17)	358.69 (320.17–399.44)
Prevalence	Both	European Region	AA	1.70 (1.10–2.5)	0 (0–0)	203.02 (179.77–225.84)	262.55 (234.9–291.47)	159.57 (141.67–178.09)
Prevalence	Male	Region of the Americas	AA	239.85 (219.74–261.64)	0 (0–0)	296.86 (268.07–327.14)	366.31 (331.02–403.11)	318.12 (287.32–350.88)
Prevalence	Female	Region of the Americas	AA	287.69 (263.73–314.2)	0 (0–0)	484.88 (437.69–534.74)	482.09 (434.19–532.04)	475.06 (429.05–523.96)
Prevalence	Both	Region of the Americas	AA	263.09 (241.06–286.86)	0 (0–0)	187.73 (169.93–206.54)	247.54 (224.43–271.77)	149.6 (134.35–165.35)
Prevalence	Male	South-East Asia Region	AA	535.43 (330.52–822.87)	0 (0–0)	80.65 (72.09–90.57)	288.8 (257.75–321.03)	227.3 (202.12–252.69)
Prevalence	Female	South-East Asia Region	AA	622.88 (383.94–955.76)	0 (0–0)	349.35 (309.97–389.92)	371.32 (329.94–414.3)	289.83 (257.07–323.34)
Prevalence	Both	South-East Asia Region	AA	577.94 (356.11–889.92)	0 (0–0)	58.42 (51.77–66.12)	208.83 (185.74–232.66)	164.44 (146.38–183.5)
Prevalence	Male	Western Pacific Region	AA	14.95 (10.62–20.34)	0 (0–0)	271.7 (242.82–302.39)	343.54 (307.16–381.91)	265.66 (236.54–295.97)
Prevalence	Female	Western Pacific Region	AA	30.76 (22.65–40.76)	0 (0–0)	405.8 (356.04–456.45)	448.77 (398.79–501.42)	357.38 (315.81–401.48)
Prevalence	Both	Western Pacific Region	AA	22.63 (16.48–30.24)	0 (0–0)	190.46 (169.19–213.21)	243.28 (216.39–271.72)	174.39 (153.78–195.69)
Prevalence	Both	Global	T1D	89.74 (80.56–99.23)	342.56 (257.39–437.13)	284.85 (217–364.65)	303.01 (231.03–386.61)	270.84 (221.48–333.11)
Prevalence	Female	Global	T1D	159.37 (142.77–176.17)	1.23 (0.87–1.61)	0 (0–0)	290.51 (221.39–370.19)	250.97 (209.71–303.72)
Prevalence	Male	Global	T1D	124.74 (112.28–137.91)	319.83 (241.07–416.77)	296.44 (225.75–380.66)	315.31 (240.34–403.74)	260.72 (216.59–318.8)
Prevalence	Both	African Region	T1D	144.25 (95.96–203.41)	378.03 (286.99–487.53)	211.25 (154.27–279.19)	275.11 (206.59–355.93)	204.01 (157.75–259.76)
Prevalence	Female	African Region	T1D	136.48 (90.71–192.71)	0 (0–0)	370.61 (290.46–465.91)	268.59 (201.87–347.88)	145.75 (113.21–183.97)
Prevalence	Male	African Region	T1D	140.35 (93.37–197.88)	718.62 (551.16–920.9)	216.8 (158.2–286.72)	282 (211.67–365.04)	175.01 (135.48–220.75)
Prevalence	Both	Eastern Mediterranean Region	T1D	1.28 (0.87–1.81)	0 (0–0)	326.06 (242.9–423.51)	398.4 (303.78–509.25)	119.77 (93.1–149.39)
Prevalence	Female	Eastern Mediterranean Region	T1D	1.52 (1.04–2.14)	133.25 (93.47–182.48)	495.08 (395.45–611.4)	373.24 (285.29–476.21)	108.2 (85.33–135.43)
Prevalence	Male	Eastern Mediterranean Region	T1D	1.4 (0.96–1.99)	385.63 (292.85–484.79)	343.65 (256.16–446.71)	421.21 (321.15–538.86)	113.5 (89.45–140.6)
Prevalence	Both	European Region	T1D	0.63 (0.35–1.06)	152.07 (99.23–219.53)	0 (0–0)	522.71 (397.74–668.32)	130.33 (102.28–161.25)
Prevalence	Female	European Region	T1D	1.07 (0.63–1.7)	376.11 (301.25–463.08)	476.58 (362.42–609.54)	506.37 (386.08–646.59)	116.99 (92.47–145.6)
Prevalence	Male	European Region	T1D	0.85 (0.49–1.37)	611.17 (465.33–784.68)	0 (0–0)	538.95 (409.02–689.95)	123.08 (96.88–152.54)
Prevalence	Both	Region of the Americas	T1D	154.84 (140.68–170.29)	315.33 (237.33–411.66)	0 (0–0)	442.76 (340.47–562.96)	158.09 (123.48–201.52)
Prevalence	Female	Region of the Americas	T1D	163.61 (148.3–180.37)	322.46 (252.67–405.25)	435.02 (334.31–551.66)	448.55 (345.05–566.47)	134.51 (106.33–168.66)
Prevalence	Male	Region of the Americas	T1D	159.24 (144.37–175.29)	0 (0–0)	0 (0–0)	437.2 (335.55–558.97)	146.32 (114.76–183.89)
Prevalence	Both	South-East Asia Region	T1D	1175.7 (729.3–1797.04)	0 (0–0)	307.43 (237.85–387.32)	254.96 (191.59–329.78)	195.88 (153.37–247.06)
Prevalence	Female	South-East Asia Region	T1D	1244.17 (780.87–1903.79)	168.39 (126.75–218.71)	152.72 (106.68–209.6)	237.68 (178.46–307.81)	154.79 (121.99–193.07)
Prevalence	Male	South-East Asia Region	T1D	1210.19 (756.34–1850.77)	0 (0–0)	331.75 (256.29–419.43)	271.76 (203.82–352.88)	174.99 (138.99–218.29)
Prevalence	Both	Western Pacific Region	T1D	6.04 (3.55–9.42)	554.09 (415.72–713.28)	154.71 (118.71–195.16)	166.43 (128.18–209.48)	135.26 (105.42–168.53)
Prevalence	Female	Western Pacific Region	T1D	12.55 (7.9–18.81)	0 (0–0)	0 (0–0)	151.66 (117.22–190.78)	122.13 (95.91–153.03)
Prevalence	Male	Western Pacific Region	T1D	9.31 (5.71–14.17)	0 (0–0)	168.04 (128.6–213.19)	180.6 (138.44–229.12)	128.45 (100.97–159.71)
Prevalence	Both	Global	IBD	84.03 (74.71–93.4)	0.03 (0.03–0.04)	107.56 (91.05–125.93)	61.66 (51.67–72.52)	55.62 (48.07–63.97)
Prevalence	Female	Global	IBD	152.2 (135.42–169.61)	0 (0–0)	36.92 (31.06–43.22)	63.54 (53.5–74.37)	54.18 (47.48–62.06)
Prevalence	Male	Global	IBD	116.95 (104.45–129.82)	0 (0–0)	107.39 (90.39–126.48)	59.81 (49.92–70.74)	54.89 (48.09–62.23)
Prevalence	Both	African Region	IBD	224.48 (151.49–310.36)	0 (0–0)	0 (0–0)	11.01 (8.17–14.23)	115.45 (95.7–139.68)
Prevalence	Female	African Region	IBD	216.13 (144.9–298.53)	0 (0–0)	12.93 (9.75–16.68)	12.12 (8.99–15.63)	101.44 (85.18–122.18)
Prevalence	Male	African Region	IBD	220.45 (149.03–304.07)	0 (0–0)	0 (0–0)	9.83 (7.26–12.74)	108.47 (90.54–130.78)
Prevalence	Both	Eastern Mediterranean Region	IBD	3.23 (2.38–4.39)	0 (0–0)	64.31 (50–83.97)	35.08 (26.64–44.47)	6.78 (5.04–8.87)
Prevalence	Female	Eastern Mediterranean Region	IBD	3.71 (2.7–5.05)	0.03 (0.02–0.04)	37 (28.63–47.03)	37.3 (28.52–47.22)	6.99 (5.23–9.13)
Prevalence	Male	Eastern Mediterranean Region	IBD	3.46 (2.54–4.68)	0 (0–0)	66.58 (50.97–87.71)	33.07 (24.93–42.26)	6.89 (5.2–8.96)
Prevalence	Both	European Region	IBD	1.6 (0.98–2.46)	0 (0–0)	146.42 (125.04–170.29)	168.19 (143.38–196.08)	6.53 (4.85–8.58)
Prevalence	Female	European Region	IBD	3.17 (2.01–4.8)	0 (0–0)	273.95 (233.37–316.31)	178.25 (151.95–207.53)	6.62 (4.93–8.69)
Prevalence	Male	European Region	IBD	2.36 (1.49–3.59)	0.05 (0.04–0.06)	142.53 (121.07–165.96)	158.4 (134.53–184.78)	6.58 (4.95–8.58)
Prevalence	Both	Region of the Americas	IBD	192.87 (175.99–212.44)	0.05 (0.04–0.05)	107.25 (92.62–123.1)	109.11 (94.01–125.97)	8.39 (6.42–10.88)
Prevalence	Female	Region of the Americas	IBD	230.79 (210.82–252.93)	0.05 (0.04–0.05)	0 (0–0)	123.35 (106.03–142.21)	7.58 (5.67–9.93)
Prevalence	Male	Region of the Americas	IBD	211.18 (192.5–231.71)	0.05 (0.04–0.05)	97.18 (83.88–111.79)	94.49 (81.41–109.37)	7.99 (6.04–10.32)
Prevalence	Both	South-East Asia Region	IBD	520.88 (315.46–806.85)	0.03 (0.03–0.04)	38.18 (28.91–49.95)	17.46 (12.85–22.65)	35.2 (27.36–44.46)
Prevalence	Female	South-East Asia Region	IBD	588.39 (358.11–908.54)	0 (0–0)	16.33 (12.21–21.16)	18.02 (13.4–23.19)	32.97 (26.3–41.19)
Prevalence	Male	South-East Asia Region	IBD	553.48 (335.86–853.66)	0 (0–0)	37.88 (28.03–50.84)	16.91 (12.28–22.23)	34.07 (27.24–42.42)
Prevalence	Both	Western Pacific Region	IBD	15.97 (11.31–21.93)	0 (0–0)	0 (0–0)	67.96 (56.2–81.03)	29.9 (22.86–38.61)
Prevalence	Female	Western Pacific Region	IBD	26.03 (17.72–37.07)	0.01 (0.01–0.01)	61.14 (51.09–72.45)	61.72 (51.25–73.09)	40.93 (31.98–51.56)
Prevalence	Male	Western Pacific Region	IBD	20.83 (14.38–28.99)	0.03 (0.02–0.04)	0 (0–0)	73.94 (60.8–88.66)	35.62 (27.98–44.69)
Prevalence	Both	Global	MS	115.16 (103.08–127.93)	87.78 (73.54–104.6)	19.39 (16.19–23.01)	21.41 (17.53–25.89)	7.23 (6.27–8.25)
Prevalence	Female	Global	MS	166.9 (149.3–184.87)	0 (0–0)	57.59 (50.51–64.82)	28.74 (23.8–34.46)	15.59 (12.3–18.86)
Prevalence	Male	Global	MS	140.29 (126.13–155.53)	78.54 (67.05–91.79)	13.01 (10.63–15.74)	14.2 (11.32–17.55)	11.49 (9.49–13.46)
Prevalence	Both	African Region	MS	289.74 (198.64–387.26)	87.32 (71.52–105.39)	8.41 (6.38–10.81)	9.31 (6.9–12.22)	3.95 (2.84–5.22)
Prevalence	Female	African Region	MS	291.47 (199.73–388.93)	2.74 (1.91–3.71)	25.1 (19.89–29.96)	12.46 (9.35–16.18)	5.42 (3.97–7.01)
Prevalence	Male	African Region	MS	290.58 (200.25–389.07)	60.44 (49.34–74.37)	5.43 (4–7.16)	6 (4.3–8.08)	4.68 (3.41–6.08)
Prevalence	Both	Eastern Mediterranean Region	MS	20.07 (16.58–24.39)	9.65 (6.97–12.56)	0 (0–0)	30.5 (23.53–38.89)	3.63 (2.55–4.87)
Prevalence	Female	Eastern Mediterranean Region	MS	21.93 (18.28–26.52)	0 (0–0)	43.09 (34.58–53.09)	41.32 (32.14–52.67)	5.77 (4.24–7.49)
Prevalence	Male	Eastern Mediterranean Region	MS	20.97 (17.44–25.4)	44.32 (35.61–54.21)	0 (0–0)	20.69 (15.71–26.71)	4.79 (3.45–6.24)
Prevalence	Both	European Region	MS	4.43 (2.87–6.44)	61.58 (50.25–74.43)	57.82 (48.83–68.14)	64.58 (53.65–77.48)	4.73 (3.32–6.32)
Prevalence	Female	European Region	MS	7.22 (4.83–10.41)	57.41 (46.67–69.4)	162.38 (144.47–181.9)	84.56 (70.79–101.08)	8.32 (6.13–10.77)
Prevalence	Male	European Region	MS	5.78 (3.81–8.35)	168.77 (146.2–194.6)	41.15 (34.25–49.08)	44.8 (36.5–54.59)	6.68 (4.85–8.72)
Prevalence	Both	Region of the Americas	MS	578.17 (528.61–631.86)	20.04 (15.54–25.59)	47.98 (42.1–54.25)	49.56 (42.71–57.04)	2.75 (1.91–3.76)
Prevalence	Female	Region of the Americas	MS	733.98 (674.6–801.39)	27.75 (21.75–33.43)	139.37 (126.44–153.18)	69.33 (60.07–79.36)	4.35 (3.13–5.74)
Prevalence	Male	Region of the Americas	MS	653.84 (599.15–713.69)	31.4 (24.07–40.6)	28.92 (25.01–33.13)	29.07 (24.54–34.02)	3.55 (2.53–4.7)
Prevalence	Both	South-East Asia Region	MS	92 (58.21–137.07)	7.64 (5.6–10.2)	7.61 (5.57–10.06)	7.73 (5.54–10.39)	5.75 (4.14–7.58)
Prevalence	Female	South-East Asia Region	MS	104 (66.6–155.38)	15.84 (12.28–19.98)	9.56 (7.36–11.73)	9.74 (7.07–13.02)	10.41 (7.88–13.31)
Prevalence	Male	South-East Asia Region	MS	97.83 (62.64–145.46)	0.68 (0.38–1.08)	5.66 (4.06–7.63)	5.78 (4.06–7.9)	8.11 (6.04–10.49)
Prevalence	Both	Western Pacific Region	MS	19.86 (13.1–28.24)	0 (0–0)	4.36 (3.28–5.61)	4.42 (3.22–5.86)	4.85 (3.45–6.31)
Prevalence	Female	Western Pacific Region	MS	51.41 (35.73–70.52)	17.07 (13.03–21.1)	0 (0–0)	5.55 (4.11–7.28)	7.86 (5.82–10.02)
Prevalence	Male	Western Pacific Region	MS	35.18 (24.01–48.63)	7.71 (5.64–10.29)	3.29 (2.4–4.31)	3.33 (2.35–4.49)	6.41 (4.7–8.2)
Prevalence	Both	Global	Psoriasis	106.48 (96.26–117.35)	0 (0–0)	634.7 (587.82–682.97)	474.25 (437.74–512.68)	929.84 (845.73–1018.58)
Prevalence	Female	Global	Psoriasis	200.81 (181.17–221.63)	0 (0–0)	385.78 (355.77–418.4)	477.2 (440.3–515.76)	509.08 (457.73–560.72)
Prevalence	Male	Global	Psoriasis	152.78 (137.97–168.19)	0 (0–0)	650.48 (602.07–700.34)	471.58 (435.06–510.04)	715.51 (650.43–780.07)
Prevalence	Both	African Region	Psoriasis	226.63 (163.59–293.33)	0 (0–0)	254.64 (232.68–278.03)	243.6 (222.54–266.23)	1005.86 (926.78–1082.83)
Prevalence	Female	African Region	Psoriasis	264.12 (187.8–343.19)	0 (0–0)	0 (0–0)	248.51 (227.12–271.55)	604.34 (556.26–652.26)
Prevalence	Male	African Region	Psoriasis	245.02 (175.39–315.93)	0 (0–0)	249.17 (227.57–272.21)	238.43 (217.68–260.75)	806.04 (742.02–868.24)
Prevalence	Both	Eastern Mediterranean Region	Psoriasis	17.44 (14.69–20.74)	0 (0–0)	0 (0–0)	365.4 (335.3–397.64)	342.64 (307.65–380.8)
Prevalence	Female	Eastern Mediterranean Region	Psoriasis	21.04 (17.77–24.79)	0 (0–0)	392.92 (360.83–426.99)	382.22 (350.99–415.61)	265.58 (237.62–294.11)
Prevalence	Male	Eastern Mediterranean Region	Psoriasis	19.21 (16.2–22.71)	0 (0–0)	0 (0–0)	349.85 (320.53–381.47)	300.76 (271.37–330.66)
Prevalence	Both	European Region	Psoriasis	1.99 (1.4–2.75)	0 (0–0)	1145.26 (1058.56–1235.49)	1135.67 (1047.72–1227.28)	340.25 (304.86–375.73)
Prevalence	Female	European Region	Psoriasis	3.96 (2.9–5.25)	0 (0–0)	1608.61 (1493.78–1735.62)	1127.37 (1041.24–1218.47)	263.25 (236.19–293.42)
Prevalence	Male	European Region	Psoriasis	2.96 (2.15–4)	0 (0–0)	1160.88 (1072.26–1251.79)	1146.14 (1055.63–1238.25)	298.38 (269.25–328.92)
Prevalence	Both	Region of the Americas	Psoriasis	387.87 (357.94–419.28)	0 (0–0)	737.16 (686.37–789.92)	698.84 (649.15–751.52)	526 (470.94–585.02)
Prevalence	Female	Region of the Americas	Psoriasis	470.44 (433.98–508.84)	0 (0–0)	1006.69 (941.26–1079.11)	717.55 (666.69–770.94)	364.18 (325.11–405.1)
Prevalence	Male	Region of the Americas	Psoriasis	428.39 (395.22–462.92)	0 (0–0)	720.29 (670.49–772.44)	680.71 (631.44–732.88)	445.2 (401.04–491.74)
Prevalence	Both	South-East Asia Region	Psoriasis	420.84 (262.29–642.38)	0 (0–0)	0 (0–0)	308.02 (282.67–334.86)	1386.85 (1261.06–1521.52)
Prevalence	Female	South-East Asia Region	Psoriasis	509.73 (323.01–757.5)	0 (0–0)	321.96 (295.29–349.82)	312.62 (286.68–339.88)	794.24 (719.96–870.53)
Prevalence	Male	South-East Asia Region	Psoriasis	464.47 (290.89–700.81)	0 (0–0)	0 (0–0)	303.61 (278.55–330.36)	1085.08 (990.91–1183.48)
Prevalence	Both	Western Pacific Region	Psoriasis	26.29 (20.66–33.41)	0 (0–0)	393.2 (362.26–425.34)	390.99 (360.24–423.39)	299.55 (270.44–332.02)
Prevalence	Female	Western Pacific Region	Psoriasis	69.15 (56.77–84.1)	0 (0–0)	565.43 (525.26–609.32)	394.7 (363.61–427.28)	231.87 (208.01–256.34)
Prevalence	Male	Western Pacific Region	Psoriasis	47.33 (38.3–58.13)	0 (0–0)	408.76 (376.49–442.05)	388.23 (357.18–420.85)	264.36 (239.71–290.23)
Prevalence	Both	Global	RHD	89.37 (79.68–100.17)	0.42 (0.35–0.47)	336.7. (202.83–523.2)	729.04 (529.9–978.13)	217.00 (175.85–273.66)
Prevalence	Female	Global	RHD	159.33 (142.51–177.8)	0 (0–0)	720.55 (538.38–947.81)	814.95 (595.57–1088.24)	306.17 (237.5–395.91)
Prevalence	Male	Global	RHD	123.17 (110.37–137.44)	0 (0–0)	318.96 (192.44–495.8)	644.96 (466.89–870.97)	262.43 (210.91–334.18)
Prevalence	Both	African Region	RHD	145.78 (95.04–206.87)	0.2 (0.16–0.25)	0 (0–0)	1492.04 (1082.45–2007.84)	371.1 (291.51–475.13)
Prevalence	Female	African Region	RHD	145.19 (96.76–204.8)	0.19 (0.15–0.23)	1214.99 (873.33–1653.67)	1567.67 (1139.14–2105.33)	497.46 (404.59–615.21)
Prevalence	Male	African Region	RHD	145.49 (95.89–205.15)	0 (0–0)	0 (0–0)	1411.86 (1022.87–1902.79)	433.98 (347.83–542.41)
Prevalence	Both	Eastern Mediterranean Region	RHD	1.79 (1.25–2.53)	0 (0–0)	653.24 (472.27–879.72)	830.85 (602.67–1110.63)	348.73 (260.26–462.88)
Prevalence	Female	Eastern Mediterranean Region	RHD	1.91 (1.33–2.68)	0.71 (0.5–0.96)	0 (0–0)	927.93 (676.04–1235.19)	448.87 (337.35–589.27)
Prevalence	Male	Eastern Mediterranean Region	RHD	1.85 (1.29–2.6)	0 (0–0)	585.44 (420.57–793.9)	742.02 (535.95–997.58)	402.99 (306.61–520.15)
Prevalence	Both	European Region	RHD	0.72 (0.4–1.19)	0.15 (0.14–0.16)	0 (0–0)	124.02 (93.73–160.42)	419.3 (314.28–541.48)
Prevalence	Female	European Region	RHD	1.18 (0.71–1.85)	0.14 (0.13–0.15)	142.85 (110.5–182.17)	133.48 (101.24–171.94)	682.18 (515.17–891.55)
Prevalence	Male	European Region	RHD	0.94 (0.55–1.5)	0.14 (0.13–0.16)	0 (0–0)	114.76 (86.2–149.35)	562.04 (426.98–728.34)
Prevalence	Both	Region of the Americas	RHD	201.33 (183.72–220.98)	0.11 (0.1–0.12)	0 (0–0)	632.12 (466.44–834.23)	480.19 (361.77–636.61)
Prevalence	Female	Region of the Americas	RHD	214.7 (195.69–235.45)	0.11 (0.1–0.12)	575.95 (429.14–759.14)	713.72 (529.97–934.6)	582.82 (436.31–762.81)
Prevalence	Male	Region of the Americas	RHD	207.78 (189.62–227.73)	0 (0–0)	0 (0–0)	548.88 (401.19–733.46)	531.4 (404.35–697.3)
Prevalence	Both	South-East Asia Region	RHD	416.18 (247.71–648.18)	1 (0.77–1.2)	714.36 (555.64–912.52)	838.18 (605.05–1123.84)	386.21 (292.8–507.33)
Prevalence	Female	South-East Asia Region	RHD	536.06 (317.37–848.62)	0 (0–0)	736.54 (524.37–1000.08)	982.51 (710.87–1314.19)	466.4 (355.79–605.81)
Prevalence	Male	South-East Asia Region	RHD	474.12 (281.38–741.61)	0.24 (0.21–0.27)	593.57 (460.36–758.15)	698.35 (501.7–941.89)	426.96 (330.27–554.83)
Prevalence	Both	Western Pacific Region	RHD	13.6 (9.3–18.73)	0 (0–0)	0 (0–0)	452.85 (329.96–604.18)	244.76 (181.58–320.46)
Prevalence	Female	Western Pacific Region	RHD	24.56 (17.44–32.99)	0.19 (0.16–0.22)	429.13 (317.56–568.9)	492.75 (361.44–653.32)	243.26 (188.23–310.41)
Prevalence	Male	Western Pacific Region	RHD	18.9 (13.27–25.59)	0.9 (0.66–1.14)	0 (0–0)	414.92 (301.24–559.11)	243.94 (187.93–310.1)
Prevalence	Both	Global	RA	107.72 (96.2–119.89)	0.02 (0.02–0.02)	172.84 (146.45–204.68)	150.95 (124.7–182.41)	209.35 (197.52–221.36)
Prevalence	Female	Global	RA	190.47 (169.73–212.54)	0 (0–0)	638.5 (555.07–727.65)	215.86 (179.04–259.7)	704.34 (671.66–740.11)
Prevalence	Male	Global	RA	146.64 (131.38–162.77)	0.02 (0.01–0.02)	101.46 (85.14–121.5)	87.12 (71.11–106.52)	461.5 (441.66–484.1)
Prevalence	Both	African Region	RA	106.85 (74.61–143.08)	0.02 (0.01–0.02)	0 (0–0)	78.39 (62.94–98.45)	281.56 (241.33–333.2)
Prevalence	Female	African Region	RA	105.16 (74.51–138.79)	0.02 (0.01–0.02)	176.98 (147.07–213.03)	99.57 (79.67–124.88)	728.76 (626.3–855.61)
Prevalence	Male	African Region	RA	106.05 (74.71–140.9)	0.02 (0.01–0.02)	0 (0–0)	119.51 (94.99–150.27)	504.11 (434.52–591.69)
Prevalence	Both	Eastern Mediterranean Region	RA	7.67 (6.03–9.69)	0 (0–0)	143.59 (119.23–172.66)	128.71 (104.1–159.06)	141.22 (111.55–180.21)
Prevalence	Female	Eastern Mediterranean Region	RA	6.65 (5.25–8.36)	0 (0–0)	352.66 (300.16–413.88)	175.17 (141.49–216.12)	330.37 (271.19–394.71)
Prevalence	Male	Eastern Mediterranean Region	RA	7.19 (5.65–9.07)	0.01 (0.01–0.01)	97.15 (80.24–117.55)	86.62 (69.88–107.33)	243.91 (199.56–294.35)
Prevalence	Both	European Region	RA	0.51 (0.28–0.88)	0 (0–0)	198.04 (165.6–237.18)	184.87 (150.01–226.9)	139.55 (110.92–179.33)
Prevalence	Female	European Region	RA	0.77 (0.44–1.24)	0 (0–0)	703.65 (614.32–802.02)	277.08 (226.07–337.65)	419.1 (345.16–500.58)
Prevalence	Male	European Region	RA	0.63 (0.35–1.05)	0.02 (0.01–0.03)	103.38 (84.54–127)	93.46 (74.1–117.49)	291.5 (240.44–349.52)
Prevalence	Both	Region of the Americas	RA	167.55 (152.83–183.23)	0 (0–0)	295.6 (258.54–337.85)	250.5 (213.75–293.16)	131.04 (106.63–162.44)
Prevalence	Female	Region of the Americas	RA	250.24 (229.33–273.12)	0.03 (0.02–0.05)	799.16 (709.92–900.89)	369.08 (315.6–430.08)	301.07 (256.22–349.56)
Prevalence	Male	Region of the Americas	RA	206.39 (188.57–225.49)	0 (0–0)	156.96 (135.63–181.75)	127.8 (107.81–151.87)	215.9 (183.15–253.94)
Prevalence	Both	South-East Asia Region	RA	338.81 (207.92–516.15)	0 (0–0)	0 (0–0)	129.91 (105.23–158.66)	224.18 (181.25–279.9)
Prevalence	Female	South-East Asia Region	RA	380.67 (237.27–572.45)	0 (0–0)	274.36 (229.84–324.99)	182.05 (148.05–221.88)	717.07 (582.51–865.45)
Prevalence	Male	South-East Asia Region	RA	358.48 (220.96–542.64)	0 (0–0)	0 (0–0)	79.18 (63.38–97.85)	475.04 (390.03–572.62)
Prevalence	Both	Western Pacific Region	RA	16.14 (11.45–22.14)	0 (0–0)	204 (172.79–239.48)	139.43 (114.62–168.07)	146.8 (115.35–187.73)
Prevalence	Female	Western Pacific Region	RA	28.49 (20.5–38)	0.02 (0.01–0.02)	0 (0–0)	202 (165.95–243.22)	372.01 (301.27–448.69)
Prevalence	Male	Western Pacific Region	RA	21.96 (15.78–29.39)	0.02 (0.01–0.02)	116.55 (98.24–137.67)	79.4 (64.75–96.42)	263.75 (212.95–318.15)

AA = alopecia areata, CI = confidence interval, IBD = inflammatory bowel disease, MS = multiple sclerosis, RA = rheumatoid arthritis, RHD = rheumatic heart disease, T1D = diabetes mellitus type 1.

**Table 2:** Changing trends of age-standardized prevalence of autoimmune diseases in childhood, adolescence, young and middle-age, and middle-aged and elderly period at the global and regional levels in 1990–2020.

Age group	Measure	Sex	Location	AA		T1D		IBD		MS		Psoriasis		RHD		RA	
				AAPC (95% CI)	*P*	AAPC (95% CI)	*P*	AAPC (95% CI)	*P*	AAPC (95% CI)	*P*	AAPC (95% CI)	*P*	AAPC (95% CI)	*P*	AAPC (95% CI)	*P*
Childhood	Prevalence	Male	Global	0.01 (0.01 to 0.01)	<.001	NA	NA	NA	NA	‐19.59 (‐44.46 to 16.41)	.157	NA	NA	NA	NA	NA	NA
Childhood	Prevalence	Female	Global	NA	NA	NA	NA	NA	NA	NA	NA	NA	NA	‐29 (‐61.78 to 31.91)	.14	NA	NA
Childhood	Prevalence	Both	Global	NA	NA	NA	NA	NA	NA	NA	NA	NA	NA	NA	NA	‐38.38 (‐68.62 to 20.98)	.091
Childhood	Prevalence	Male	African Region	NA	NA	‐12.58 (‐51.86 to 58.77)	.435	NA	NA	5.21 (‐15.96 to 31.72)	.075	NA	NA	NA	NA	NA	NA
Childhood	Prevalence	Female	African Region	NA	NA	‐22.34 (‐56.16 to 37.56)	.15	NA	NA	NA	NA	NA	NA	NA	NA	NA	NA
Childhood	Prevalence	Both	African Region	NA	NA	‐27.97 (‐60.49 to 31.32)	.356	‐24.78 (‐51.52 to 16.73)	0.088	NA	NA	NA	NA	NA	NA	NA	NA
Childhood	Prevalence	Male	Eastern Mediterranean Region	NA	NA	‐23.71 (‐54.21 to 27.11)	.197	NA	NA	NA	NA	NA	NA	NA	NA	NA	NA
Childhood	Prevalence	Female	Eastern Mediterranean Region	NA	NA	‐27.31 (‐43.08 to ‐7.17)	.025	NA	NA	NA	NA	NA	NA	NA	NA	NA	NA
Childhood	Prevalence	Both	Eastern Mediterranean Region	NA	NA	‐19.61 (‐42.23 to 11.87)	.086	NA	NA	NA	NA	NA	NA	NA	NA	NA	NA
Childhood	Prevalence	Male	European Region	NA	NA	‐33.4 (‐97.36 to 1582.93)	.023	NA	NA	NA	NA	NA	NA	NA	NA	NA	NA
Childhood	Prevalence	Female	European Region	NA	NA	‐28.59 (‐54.66 to 12.48)	.122	‐29.6 (‐48.89 to ‐3.04)	.126	NA	NA	NA	NA	NA	NA	NA	NA
Childhood	Prevalence	Both	European Region	NA	NA	‐30.08 (‐46.26 to ‐9.02)	.143	NA	NA	NA	NA	NA	NA	NA	NA	‐42.25 (NA to NA)	.023
Childhood	Prevalence	Male	Region of the Americas	NA	NA	NA	NA	NA	NA	‐7.03 (‐99.28 to 11919.64)	0.04	NA	NA	NA	NA	NA	NA
Childhood	Prevalence	Female	Region of the Americas	NA	NA	‐26.54 (‐41.53 to ‐7.7)	.108	NA	NA	NA	NA	NA	NA	NA	NA	‐24.18 (‐81.98 to 218.98)	.033
Childhood	Prevalence	Both	Region of the Americas	NA	NA	‐26.59 (‐51.9 to 12.04)	.072	NA	NA	0 (NA to NA)	NA	NA	NA	NA	NA	NA	NA
Childhood	Prevalence	Male	South-East Asia Region	NA	NA	‐23.71 (‐45.54 to 6.87)	.001	‐70.02 (NA to NA)	.88	NA	NA	NA	NA	‐25.28 (‐72.06 to 99.79)	1.00	NA	NA
Childhood	Prevalence	Female	South-East Asia Region	NA	NA	‐20.11 (‐44.93 to 15.89)	1.00	‐25.68 (‐42.13 to ‐4.54)	.165	NA	NA	NA	NA	NA	NA	9 (NA to NA)	.494
Childhood	Prevalence	Both	South-East Asia Region	NA	NA	‐23.03 (‐44.15 to 6.07)	.213	‐26.36 (‐32.5 to ‐19.67)	1.00	NA	NA	NA	NA	NA	NA	NA	NA
Childhood	Prevalence	Male	Western Pacific Region	NA	NA	NA	NA	NA	NA	NA	NA	NA	NA	NA	NA	NA	NA
Childhood	Prevalence	Female	Western Pacific Region	NA	NA	NA	NA	NA	NA	NA	NA	NA	NA	NA	NA	NA	NA
Childhood	Prevalence	Both	Western Pacific Region	NA	NA	NA	NA	NA	NA	NA	NA	NA	NA	NA	NA	NA	NA
Adolescence	Prevalence	Male	Global	‐0.12 (‐0.13 to ‐0.11)	<.001	0.77 (0.73 to 0.82)	<.001	0 (‐0.08 to 0.07)	.958	‐0.04 (‐0.08 to 0)	.058	‐0.8 (‐0.82 to ‐0.78)	<.001	0.74 (0.64 to 0.84)	<.001	0.13 (0.07 to 0.19)	<.001
Adolescence	Prevalence	Female	Global	‐0.1 (‐0.11 to ‐0.09)	<.001	0.76 (0.72 to 0.8)	<.001	‐0.08 (‐0.13 to ‐0.03)	.003	0.01 (‐0.07 to 0.09)	.783	‐0.87 (‐0.89 to ‐0.84)	<.001	0.75 (0.65 to 0.86)	<.001	0.11 (0.04 to 0.18)	.003
Adolescence	Prevalence	Both	Global	‐0.12 (‐0.13 to ‐0.11)	<.001	0.78 (0.73 to 0.83)	<.001	0.06 (‐0.02 to 0.14)	.12	‐0.08 (‐0.12 to ‐0.03)	.001	‐0.71 (‐0.75 to ‐0.68)	<.001	0.73 (0.63 to 0.83)	<.001	0.19 (0.15 to 0.24)	<.001
Adolescence	Prevalence	Male	African Region	‐0.01 (‐0.01 to 0)	<.001	0.17 (0.16 to 0.19)	<.001	‐0.04 (‐0.09 to 0)	.067	‐0.21 (‐0.24 to ‐0.19)	<.001	‐0.68 (‐0.73 to ‐0.62)	<.001	0.31 (0.26 to 0.36)	<.001	‐0.02 (‐0.04 to 0.01)	.224
Adolescence	Prevalence	Female	African Region	0.01 (0 to 0.01)	<.001	0.18 (0.16 to 0.19)	<.001	‐0.01 (‐0.07 to 0.04)	.611	‐0.19 (‐0.21 to ‐0.16)	<.001	‐0.7 (‐0.74 to ‐0.67)	<.001	0.31 (0.23 to 0.39)	<.001	‐0.06 (‐0.08 to ‐0.04)	<.001
Adolescence	Prevalence	Both	African Region	0 (0 to 0)	.08	0.17 (0.16 to 0.18)	<.001	‐0.05 (-0.13 to 0.02)	.141	‐0.16 (‐0.24 to ‐0.07)	.001	‐0.65 (‐0.72 to ‐0.57)	<.001	0.31 (0.26 to 0.36)	<.001	0.15 (0.13 to 0.17)	<.001
Adolescence	Prevalence	Male	Eastern Mediterranean Region	0 (0 to 0.01)	.056	1.57 (1.53 to 1.6)	<.001	0.12 (0.08 to 0.17)	<.001	‐0.16 (‐0.22 to ‐0.1)	<.001	‐1.02 (‐1.04 to ‐1.01)	<.001	0.28 (0.1 to 0.46)	.003	0.53 (0.49 to 0.56)	<.001
Adolescence	Prevalence	Female	Eastern Mediterranean Region	0 (0 to 0)	<.001	1.55 (1.51 to 1.59)	<.001	0.11 (0.05 to 0.17)	<.001	‐0.19 (‐0.25 to ‐0.12)	<.001	‐1.04 (‐1.05 to ‐1.02)	<.001	0.31 (0.13 to 0.49)	.001	0.48 (0.44 to 0.52)	<.001
Adolescence	Prevalence	Both	Eastern Mediterranean Region	0.01 (0.01 to 0.01)	<.001	1.57 (1.54 to 1.61)	<.001	0.12 (0.08 to 0.16)	<.001	‐0.13 (‐0.21 to ‐0.04)	.005	‐1.01 (‐1.02 to ‐0.99)	<.001	0.25 (0.06 to 0.44)	.009	0.61 (0.57 to 0.66)	<.001
Adolescence	Prevalence	Male	European Region	0.02 (0.01 to 0.02)	<.001	1.62 (1.5 to 1.75)	<.001	0.37 (0.26 to 0.48)	<.001	0.42 (0.36 to 0.48)	<.001	‐0.31 (‐0.34 to ‐0.28)	<.001	0.94 (0.87 to 1.01)	<.001	0.64 (0.61 to 0.68)	<.001
Adolescence	Prevalence	Female	European Region	‐0.01 (‐0.01 to ‐0.01)	<.001	1.59 (1.53 to 1.65)	<.001	0.24 (0.15 to 0.34)	<.001	0.45 (0.4 to 0.51)	<.001	‐0.32 (‐0.35 to ‐0.3)	<.001	0.93 (0.87 to 1)	<.001	0.67 (0.64 to 0.7)	<.001
Adolescence	Prevalence	Both	European Region	0.07 (0.06 to 0.08)	<.001	1.66 (1.48 to 1.84)	<.001	0.5 (0.37 to 0.64)	<.001	0.39 (0.31 to 0.47)	<.001	‐0.29 (‐0.32 to ‐0.25)	<.001	0.94 (0.87 to 1.01)	<.001	0.64 (0.61 to 0.67)	<.001
Adolescence	Prevalence	Male	Region of the Americas	‐0.02 (‐0.04 to 0)	.063	0.37 (0.27 to 0.47)	<.001	0.15 (0.1 to 0.21)	<.001	‐0.06 (‐0.16 to 0.04)	.27	‐0.47 (‐0.51 to ‐0.43)	<.001	‐0.06 (‐0.09 to ‐0.04)	<.001	0.55 (0.52 to 0.57)	<.001
Adolescence	Prevalence	Female	Region of the Americas	0.04 (0 to 0.07)	.052	0.37 (0.27 to 0.46)	<.001	0.23 (0.18 to 0.28)	<.001	‐0.03 (‐0.1 to 0.04)	.43	‐0.47 (‐0.51 to ‐0.42)	<.001	‐0.05 (‐0.07 to ‐0.03)	<.001	0.59 (0.56 to 0.62)	<.001
Adolescence	Prevalence	Both	Region of the Americas	‐0.07 (‐0.08 to ‐0.06)	<.001	0.33 (0.28 to 0.38)	<.001	0.07 (0 to 0.14)	.052	‐0.1 (‐0.15 to ‐0.04)	.001	‐.46 (-.5 to -.43)	<.001	‐0.08 (‐0.11 to ‐0.04)	<.001	0.51 (0.47 to 0.54)	<.001
Adolescence	Prevalence	Male	South-East Asia Region	‐0.08 (‐0.09 to ‐0.08)	<.001	0.36 (0.33 to 0.39)	<.001	0.05 (‐0.09 to 0.19)	.49	0.27 (0.21 to 0.32)	<.001	0.01 (‐0.02 to 0.04)	.492	0.42 (0.13 to 0.72)	.005	0.54 (0.52 to 0.57)	<.001
Adolescence	Prevalence	Female	South-East Asia Region	‐0.06 (‐0.06 to ‐0.06)	<.001	0.37 (0.34 to 0.4)	<.001	0.1 (0.01 to 0.19)	.022	0.31 (0.28 to 0.33)	<.001	‐0.02 (‐0.06 to 0.03)	.495	0.47 (0.17 to 0.77)	.002	0.58 (0.53 to 0.63)	<.001
Adolescence	Prevalence	Both	South-East Asia Region	‐0.11 (‐0.11 to ‐0.1)	<.001	0.35 (0.29 to 0.41)	<.001	‐0.01 (‐0.18 to 0.15)	.861	0.31 (0.21 to 0.4)	<.001	0.04 (0 to 0.07)	.047	0.46 (0.18 to 0.75)	.001	0.52 (0.49 to 0.56)	<.001
Adolescence	Prevalence	Male	Western Pacific Region	0 (0 to 0)	.654	0.57 (0.44 to 0.7)	<.001	2.32 (2.06 to 2.58)	<.001	0.27 (0.13 to 0.4)	<.001	‐0.97 (‐0.99 to ‐0.94)	<.001	‐0.63 (‐0.7 to ‐0.56)	<.001	0.13 (0.03 to 0.24)	.012
Adolescence	Prevalence	Female	Western Pacific Region	0.01 (0.01 to 0.01)	<.001	0.6 (0.48 to 0.71)	<.001	2.17 (1.96 to 2.38)	<.001	0.3 (0.2 to 0.39)	<.001	‐0.91 (‐0.94 to ‐0.88)	<.001	‐0.56 (‐0.65 to ‐0.46)	<.001	0.14 (‐0.05 to 0.34)	.155
Adolescence	Prevalence	Both	Western Pacific Region	0.08 (0.07 to 0.08)	<.001	0.56 (0.36 to 0.76)	<.001	2.45 (2.16 to 2.75)	<.001	0.28 (0.28 to 0.28)	<.001	‐1 (‐1.04 to ‐0.95)	<.001	-0.69 (‐0.77 to ‐0.61)	<.001	0.31 (0.25 to 0.38)	<.001
Young and middle-age	Prevalence	Male	Global	‐0.12 (‐0.14 to ‐0.11)	<.001	0.88 (0.81 to 0.95)	<.001	‐0.57 (‐0.6 to ‐0.53)	<.001	‐0.24 (‐0.25 to ‐0.22)	<.001	‐0.99 (‐1.01 to ‐0.98)	<.001	0.59 (0.5 to 0.69)	<.001	0.3 (0.21 to 0.38)	<.001
Young and middle-age	Prevalence	Female	Global	‐0.12 (‐0.13 to ‐0.11)	<.001	0.84 (0.8 to 0.87)	<.001	‐0.66 (‐0.69 to ‐0.63)	<.001	‐0.24 (‐0.26 to ‐0.23)	<.001	‐0.99 (‐1.01 to ‐0.98)	<.001	0.62 (0.57 to 0.68)	<.001	0.24 (0.18 to 0.3)	<.001
Young and middle-age	Prevalence	Both	Global	‐0.14 (‐0.15 to ‐0.14)	<.001	0.93 (0.86 to 1)	<.001	‐0.45 (‐0.48 to ‐0.41)	<.001	‐0.26 (‐0.28 to ‐0.23)	<.001	‐0.98 (‐1 to ‐0.96)	<.001	0.55 (0.4 to 0.71)	<.001	0.35 (0.26 to 0.44)	<.001
Young and middle-age	Prevalence	Male	African Region	0.01 (0.01 to 0.01)	<.001	0.35 (0.35 to 0.36)	<.001	0.44 (0.4 to 0.47)	<.001	0.43 (0.41 to 0.44)	<.001	‐0.7 (‐0.75 to ‐0.64)	<.001	0.18 (0.15 to 0.22)	<.001	0.05 (0 to 0.1)	.045
Young and middle-age	Prevalence	Female	African Region	0 (0 to 0)	<.001	0.36 (0.36 to 0.36)	<.001	0.43 (0.35 to 0.51)	<.001	0.44 (0.43 to 0.46)	<.001	‐0.7 (‐0.74 to ‐0.66)	<.001	0.18 (0.13 to 0.23)	<.001	‐0.06 (‐0.13 to 0.01)	.114
Young and middle-age	Prevalence	Both	African Region	0 (0 to 0)	<.001	0.35 (0.34 to 0.36)	<.001	0.41 (0.37 to 0.46)	<.001	0.34 (0.31 to 0.38)	<.001	‐0.67 (‐0.73 to ‐0.61)	<.001	0.17 (0.14 to 0.2)	<.001	0.27 (0.18 to 0.35)	<.001
Young and middle-age	Prevalence	Male	Eastern Mediterranean Region	‐0.01 (‐0.01 to ‐0.01)	<.001	1.77 (1.73 to 1.82)	<.001	0.44 (0.42 to 0.46)	<.001	0.43 (0.37 to 0.48)	<.001	‐0.89 (‐0.89 to ‐0.88)	<.001	0.1 (0.03 to 0.18)	.006	0.39 (0.36 to 0.41)	<.001
Young and middle-age	Prevalence	Female	Eastern Mediterranean Region	0 (0 to 0)	.284	1.74 (1.71 to 1.78)	<.001	0.34 (0.32 to 0.36)	<.001	0.46 (0.37 to 0.55)	<.001	‐0.88 (‐0.89 to ‐0.87)	<.001	0.16 (0.1 to 0.23)	<.001	0.48 (0.45 to 0.5)	<.001
Young and middle-age	Prevalence	Both	Eastern Mediterranean Region	0.01 (0.01 to 0.01)	<.001	1.79 (1.76 to 1.83)	<.001	0.56 (0.52 to 0.6)	<.001	0.43 (0.4 to 0.46)	<.001	‐0.89 (‐0.91 to ‐0.87)	<.001	0.05 (‐0.03 to 0.13)	.223	0.27 (0.2 to 0.34)	<.001
Young and middle-age	Prevalence	Male	European Region	‐0.04 (‐0.04 to ‐0.03)	<.001	1.67 (1.64 to 1.71)	<.001	0.3 (0.26 to 0.35)	<.001	0.45 (0.38 to 0.52)	<.001	‐0.5 (‐0.53 to ‐0.47)	<.001	0.63 (0.56 to 0.7)	<.001	0.46 (0.43 to 0.49)	<.001
Young and middle-age	Prevalence	Female	European Region	‐0.02 (‐0.02 to ‐0.02)	<.001	1.64 (1.56 to 1.72)	<.001	0.3 (0.26 to 0.33)	<.001	0.54 (0.49 to 0.59)	<.001	‐0.49 (‐0.52 to ‐0.46)	<.001	0.65 (0.58 to 0.73)	<.001	0.48 (0.45 to 0.5)	<.001
Young and middle-age	Prevalence	Both	European Region	‐0.05 (‐0.05 to ‐0.05)	<.001	1.7 (1.63 to 1.77)	<.001	0.31 (0.24 to 0.39)	<.001	0.27 (0.21 to 0.33)	<.001	‐0.51 (‐0.54 to ‐0.47)	<.001	0.61 (0.54 to 0.68)	<.001	0.44 (0.41 to 0.47)	<.001
Young and middle-age	Prevalence	Male	Region of the Americas	‐0.22 (‐0.26 to ‐0.18)	<.001	0.13 (0.07 to 0.18)	<.001	‐1.93 (‐2.05 to ‐1.81)	<.001	‐0.42 (‐0.45 to ‐0.39)	<.001	‐0.75 (‐0.78 to ‐0.71)	<.001	0.44 (0.43 to 0.44)	<.001	0.64 (0.61 to 0.66)	<.001
Young and middle-age	Prevalence	Female	Region of the Americas	‐0.22 (‐0.26 to ‐0.18)	<.001	0.19 (0.11 to 0.27)	<.001	‐1.84 (‐1.95 to ‐1.72)	<.001	‐0.44 (‐0.48 to ‐0.4)	<.001	‐0.73 (‐0.76 to ‐0.7)	<.001	0.46 (0.42 to 0.49)	<.001	0.61 (0.59 to 0.63)	<.001
Young and middle-age	Prevalence	Both	Region of the Americas	‐0.22 (‐0.24 to ‐0.2)	<.001	0.08 (-0.06 to 0.22)	.277	‐2.13 (‐2.24 to ‐2.02)	<.001	‐0.39 (‐0.43 to ‐0.34)	<.001	‐0.76 (‐0.79 to ‐0.72)	<.001	0.4 (0.38 to 0.41)	<.001	0.7 (0.68 to 0.72)	<.001
Young and middle-age	Prevalence	Male	South-East Asia Region	‐0.05 (‐0.05 to ‐0.04)	<.001	0.76 (0.6 to 0.91)	<.001	‐0.05 (‐0.2 to 0.1)	.489	0.41 (0.38 to 0.44)	<.001	‐0.13 (‐0.15 to ‐0.11)	<.001	0.18 (‐0.18 to 0.53)	.328	0.57 (0.55 to 0.58)	<.001
Young and middle-age	Prevalence	Female	South-East Asia Region	‐0.05 (‐0.05 to ‐0.05)	<.001	0.76 (0.71 to 0.82)	<.001	0.05 (‐0.02 to 0.12)	.18	0.41 (0.4 to 0.43)	<.001	‐0.13 (‐0.16 to ‐0.1)	<.001	0.15 (‐0.06 to 0.37)	.157	0.53 (0.52 to 0.55)	<.001
Young and middle-age	Prevalence	Both	South-East Asia Region	‐0.07 (‐0.07 to ‐0.06)	<.001	0.8 (0.68 to 0.91)	<.001	‐0.15 (‐0.42 to 0.11)	.254	0.36 (0.3 to 0.42)	<.001	‐0.12 (‐0.14 to ‐0.11)	<.001	0.27 (‐0.21 to 0.74)	.272	0.56 (0.54 to 0.58)	<.001
Young and middle-age	Prevalence	Male	Western Pacific Region	0.01 (0.01 to 0.01)	<.001	1 (0.87 to 1.14)	<.001	2.09 (1.95 to 2.23)	<.001	0.38 (0.21 to 0.54)	<.001	‐0.99 (‐1.06 to ‐0.91)	<.001	‐0.46 (‐0.52 to ‐0.39)	<.001	0.21 (0.11 to 0.3)	<.001
Young and middle-age	Prevalence	Female	Western Pacific Region	‐0.01 (‐0.01 to ‐0.01)	< 0.001	0.89 (0.85 to 0.94)	<.001	2.01 (1.85 to 2.16)	<.001	0.42 (0.27 to 0.57)	<.001	‐0.96 (‐1.03 to ‐0.89)	<.001	‐0.36 (‐0.45 to ‐0.28)	<.001	0.17 (0.07 to 0.27)	.001
Young and middle-age	Prevalence	Both	Western Pacific Region	0.04 (0.04 to 0.04)	<.001	1.07 (0.95 to 1.2)	<.001	2.16 (2.03 to 2.28)	<.001	0.27 (0.11 to 0.43)	.001	‐1 (‐1.07 to ‐0.92)	<.001	‐0.57 (‐0.62 to ‐0.51)	<.001	0.23 (0.17 to 0.28)	<.001
Middle-aged and elderly	Prevalence	Male	Global	‐0.04 (‐0.05 to ‐0.02)	<.001	0.94 (0.86 to 1.02)	<.001	‐0.64 (‐0.68 to ‐0.6)	<.001	‐0.06 (‐0.19 to 0.07)	.343	‐0.87 (‐0.91 to ‐0.84)	<.001	0.05 (0.01 to 0.09)	.012	0.28 (0.18 to 0.37)	<.001
Middle-aged and elderly	Prevalence	Female	Global	‐0.1 (‐0.11 to ‐0.09)	<.001	0.84 (0.78 to 0.91)	<.001	‐0.67 (‐0.72 to ‐0.62)	<.001	‐0.05 (‐0.19 to 0.1)	.529	‐0.92 (‐0.95 to ‐0.89)	<.001	0.04 (0.01 to 0.08)	.015	0.21 (0.11 to 0.31)	<.001
Middle-aged and elderly	Prevalence	Both	Global	0.02 (0.02 to 0.03)	<.001	1.02 (0.93 to 1.1)	<.001	‐0.61 (‐0.65 to ‐0.56)	<.001	‐0.15 (‐0.26 to ‐0.03)	.01	‐0.82 (‐0.87 to ‐0.77)	<.001	0.04 (‐0.01 to 0.09)	.149	0.37 (0.31 to 0.43)	<.001
Middle-aged and elderly	Prevalence	Male	African Region	0.06 (0.05 to 0.06)	<.001	0.61 (0.6 to 0.62)	<.001	0.54 (0.42 to 0.66)	<.001	0.7 (0.67 to 0.73)	<.001	‐0.68 (‐0.72 to ‐0.64)	<.001	0.12 (0.11 to 0.13)	<.001	0.32 (0.27 to 0.37)	<.001
Middle-aged and elderly	Prevalence	Female	African Region	0 (0 to 0)	<.001	0.63 (0.62 to 0.64)	<.001	0.43 (0.3 to 0.55)	<.001	0.61 (0.58 to 0.65)	<.001	‐0.72 (‐0.78 to ‐0.66)	<.001	0.12 (0.1 to 0.14)	<.001	0.15 (0.08 to 0.22)	<.001
Middle-aged and elderly	Prevalence	Both	African Region	0 (‐0.01 to 0)	<.001	0.59 (0.58 to 0.61)	<.001	0.66 (0.53 to 0.79)	<.001	0.68 (0.63 to 0.74)	<.001	‐0.65 (‐0.71 to ‐0.59)	<.001	0.09 (0.07 to 0.11)	<.001	0.48 (0.43 to 0.54)	<.001
Middle-aged and elderly	Prevalence	Male	Eastern Mediterranean Region	0 (‐0.01 to 0)	.284	1.86 (1.8 to 1.93)	<.001	0.78 (0.69 to 0.87)	<.001	0.64 (0.51 to 0.78)	<.001	‐0.89 (‐0.9 to ‐0.87)	<.001	0.01 (‐0.02 to 0.03)	.612	0.2 (0.12 to 0.28)	<.001
																	
Middle-aged and elderly	Prevalence	Female	Eastern Mediterranean Region	0 (0 to 0)	<.001	1.86 (1.83 to 1.89)	<.001	0.78 (0.69 to 0.87)	<.001	0.7 (0.63 to 0.78)	<.001	‐0.87 (‐0.89 to ‐0.85)	<.001	0.04 (0.02 to 0.06)	<.001	0.33 (0.29 to 0.37)	<.001
Middle-aged and elderly	Prevalence	Both	Eastern Mediterranean Region	0.01 (0 to 0.01)	<.001	1.86 (1.82 to 1.9)	<.001	0.78 (0.68 to 0.87)	<.001	0.59 (0.54 to 0.64)	<.001	‐0.9 (‐0.92 to ‐0.88)	<.001	‐0.02 (‐0.05 to 0)	.045	0.01 (‐0.12 to 0.14)	.869
Middle-aged and elderly	Prevalence	Male	European Region	0.01 (0 to 0.02)	0.026	1.92 (1.7 to 2.14)	<.001	0.17 (0.11 to 0.23)	<.001	0.79 (0.69 to 0.89)	<.001	‐0.21 (‐0.28 to ‐0.14)	<.001	‐2.12 (‐2.19 to ‐2.05)	<.001	0.37 (0.3 to 0.43)	<.001
Middle-aged and elderly	Prevalence	Female	European Region	0.04 (0.03 to 0.06)	<.001	1.75 (1.62 to 1.88)	<.001	0.2 (0.14 to 0.26)	<.001	0.94 (0.85 to 1.03)	<.001	-0.21 (‐0.28 to ‐0.15)	<.001	‐2.18 (‐2.28 to ‐2.08)	<.001	0.35 (0.28 to 0.43)	<.001
Middle-aged and elderly	Prevalence	Both	European Region	‐0.01 (‐0.01 to 0)	.001	1.9 (1.79 to 2.02)	<.001	0.15 (0.08 to 0.21)	<.001	0.56 (0.5 to 0.62)	<.001	‐0.2 (‐0.28 to ‐0.12)	<.001	‐2 (‐2.08 to ‐1.91)	<.001	0.49 (0.4 to 0.58)	<.001
Middle-aged and elderly	Prevalence	Male	Region of the Americas	‐0.23 (‐0.26 to ‐0.19)	<.001	0.39 (0.27 to 0.51)	<.001	‐2.36 (‐2.46 to ‐2.26)	<.001	‐0.08 (‐0.16 to 0)	.039	‐0.74 (‐0.75 to ‐0.72)	<.001	0 (‐0.04 to 0.04)	.967	0.5 (0.47 to 0.52)	<.001
Middle-aged and elderly	Prevalence	Female	Region of the Americas	‐0.31 (‐0.35 to ‐0.26)	<.001	0.4 (0.24 to 0.57)	<.001	‐2.25 (‐2.34 to ‐2.16)	<.001	‐0.1 (‐0.18 to ‐0.02)	.018	‐0.75 (‐0.77 to ‐0.73)	<.001	0.04 (‐0.01 to 0.1)	.125	0.48 (0.44 to 0.51)	<.001
Middle-aged and elderly	Prevalence	Both	Region of the Americas	0.03 (‐0.02 to 0.07)	.217	0.39 (0.23 to 0.56)	<.001	‐2.5 (‐2.64 to ‐2.37)	<.001	‐0.05 (‐0.12 to 0.02)	.162	‐0.72 (‐0.73 to ‐0.7)	<.001	‐0.05 (‐0.1 to ‐0.01)	.022	0.56 (0.53 to 0.59)	<.001
Middle-aged and elderly	Prevalence	Male	South-East Asia Region	0.05 (0.04 to 0.05)	<.001	0.92 (0.76 to 1.08)	<.001	0.12 (‐0.07 to 0.31)	.218	0.48 (0.43 to 0.52)	<.001	‐0.31 (‐0.35 to ‐0.27)	<.001	0.13 (0.02 to 0.24)	.026	0.46 (0.42 to 0.49)	<.001
Middle-aged and elderly	Prevalence	Female	South-East Asia Region	‐0.01 (‐0.01 to ‐0.01)	<.001	0.96 (0.87 to 1.04)	<.001	0.27 (0.21 to 0.33)	<.001	0.47 (0.44 to 0.49)	<.001	‐0.29 (‐0.34 to ‐0.23)	<.001	0.11 (‐0.04 to 0.26)	.156	0.4 (0.37 to 0.43)	<.001
Middle-aged and elderly	Prevalence	Both	South-East Asia Region	0.03 (0.03 to 0.04)	<.001	0.97 (0.82 to 1.12)	<.001	0.04 (‐0.2 to 0.28)	.742	0.43 (0.36 to 0.49)	<.001	‐0.34 (‐0.37 to ‐0.3)	<.001	0.13 (‐0.02 to 0.28)	.083	0.42 (0.37 to 0.48)	<.001
Middle-aged and elderly	Prevalence	Male	Western Pacific Region	‐0.01 (‐0.02 to ‐0.01)	< 0.001	0.95 (0.82 to 1.08)	<.001	0.99 (0.83 to 1.14)	<.001	0.43 (0.28 to 0.58)	<.001	‐0.8 (‐0.84 to ‐0.76)	<.001	0.18 (0.08 to 0.27)	.001	0.2 (0.03 to 0.36)	.023
Middle-aged and elderly	Prevalence	Female	Western Pacific Region	‐0.1 (‐0.11 to ‐0.09)	<.001	0.71 (0.52 to 0.9)	<.001	1.21 (1.04 to 1.37)	<.001	0.46 (0.29 to 0.63)	<.001	‐0.8 (‐0.85 to ‐0.75)	0	0.2 (0.13 to 0.27)	<.001	0.14 (‐0.06 to 0.34)	.169
Middle-aged and elderly	Prevalence	Both	Western Pacific Region	0.04 (0.04 to 0.05)	<.001	1.1 (0.98 to 1.23)	<.001	0.84 (0.7 to 0.99)	<.001	0.31 (0.15 to 0.47)	<.001	‐0.77 (‐0.81 to ‐0.74)	<.001	0.11 (0.03 to 0.2)	.01	0.15 (0.06 to 0.24)	.001

NA represents the raw data that is missing from the GBD database to calculate the estimate.

AA = alopecia areata, CI = confidence interval, GBD = Global Burden of Diseases, Injuries, and Risk Factors Study, IBD = inflammatory bowel disease, MS = multiple sclerosis, RA = rheumatoid arthritis, RHD = rheumatic heart disease, T1D = diabetes mellitus type 1.

#### 3.1.2. The estimate of APC effects and prediction of ASPR

**Figure 3.**
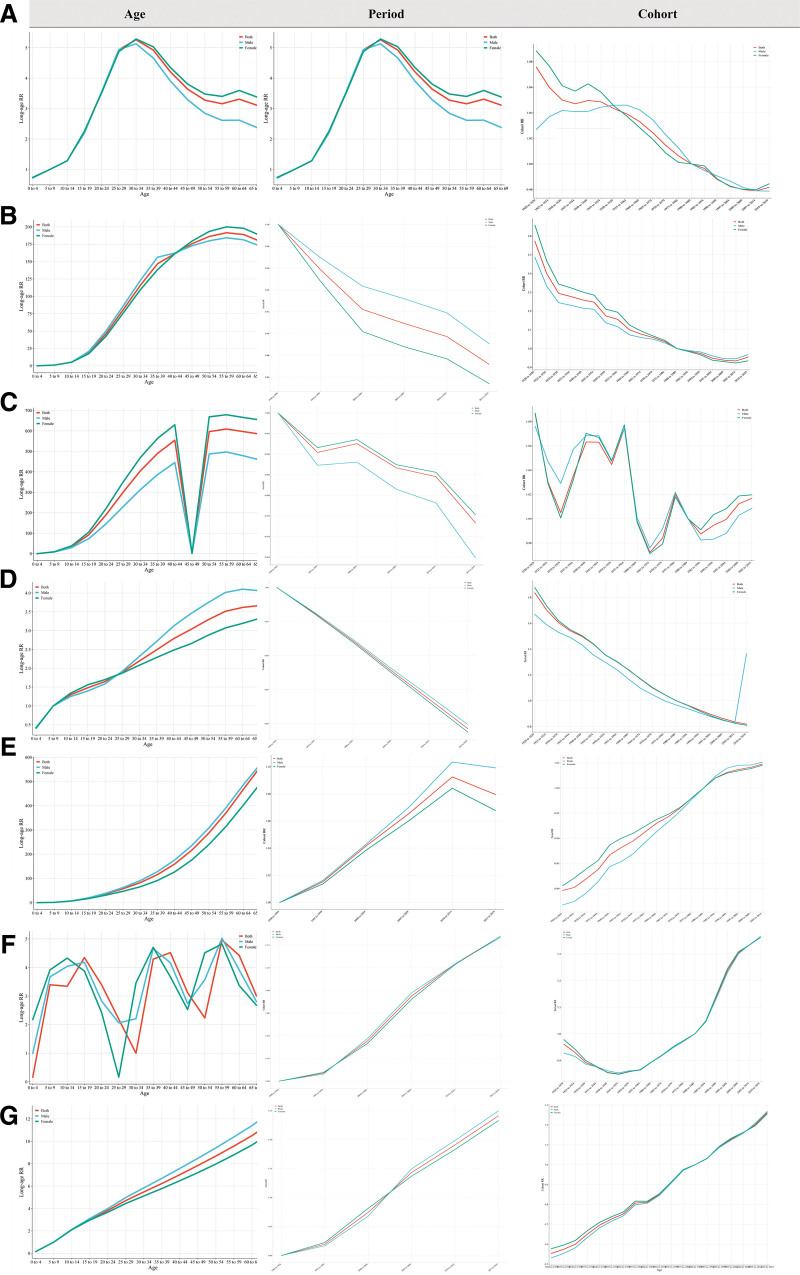
The sex-specific comparisons of age-period-cohort effects of ADs in individuals aged 0 to 69 years globally. (A) AA; (B) T1D; (C) IBD; (D) MS; (E) psoriasis; (F) RHD; (G) RA. AA = alopecia areata; ADs = autoimmune diseases; IBD = inflammatory bowel disease; MS = multiple sclerosis; RA = rheumatoid arthritis; RHD = rheumatic heart disease; T1D = diabetes mellitus type 1.

Age-related findings indicated that the global risk of AA showed a “peak” pattern in different life cycles (an increase followed by a decrease), which was also observed at regional and national levels (Fig. 3; Table S3, Supplemental Digital Content, https://links.lww.com/MD/R133). There was a more pronounced upward trend in the AAPC of AA among females compared to males, highlighting significant gender specificity. Period-related results revealed that in comparison to the individual risk during the reference period of 1990 to 1994, the individual relative risk in the period of 2015 to 2021 was 0.97 (95% CI: 0.97–0.97). Over the period from 1990 to 2021, the relative risk of AA exhibited a varying degree of decline across different periods (Table S4, Supplemental Digital Content, https://links.lww.com/MD/R133). Birth cohort effect findings demonstrated that as the birth period changes, the disease rate among consecutive birth cohorts worldwide showed a “peak” pattern, with the risk of females displaying a more stable change pattern compared to males (Table S5, Supplemental Digital Content, https://links.lww.com/MD/R133). The results of prediction also showed a continued increase ASPR globally of AA from 2020 to 2032, characterized by notable gender and age-specific trends, and the ASPR of AA was predicted to stabilize globally after 2032 (Table S6, Supplemental Digital Content, https://links.lww.com/MD/R133).

### 3.2. Type 1 diabetes

#### 3.2.1. The estimate of ASPR and AAPC

In 2021, the global ASPR of T1D in populations aged 0 to 69 years was estimated at 89.74 (95% UI: 80.56–99.23). The Eastern Mediterranean region, notably Greece, exhibited the highest ASPR regional and national (Table S7, Supplemental Digital Content, https://links.lww.com/MD/R133). Age-specific ASPR differences were evident across different life cycles and showed a continuous fluctuation, which increased to 342.56 in childhood (95% UI: 257.39–437.13), decreased to 284.85 in adolescence (95% UI: 217.00–364.65), then increased to 303.01 (95% UI: 231.03–386.61) and decreased to 270.84 (95% UI: 221.48–333.11). Notably, the ASPR of T1D was higher in males than in females across life cycles (Table 1; Table S1, Supplemental Digital Content, https://links.lww.com/MD/R133; Fig. 1B). From 1990 to 2021, the global and regional trend of T1D prevalence in populations aged 0 to 69 showed a consistent increase, with varying AAPC across life cycles (Fig. 2). The AAPC in childhood remained stable, while other life cycles saw some rise in different levels, which increased by 0.78% in adolescence (95% CI: 0.73–0.83%), 0.93% in young and middle-aged cycles (95% CI: 0.86–1.00%), 1.02% in middle and old-aged cycles (95% CI: 0.93–1.10%) (Table 2; Table S2, Supplemental Digital Content, https://links.lww.com/MD/R133; Fig. 2B).

#### 3.2.2. The estimation of APC effects and prediction of ASPR

Age effect results indicated a linear increase in global T1D risk with age, with no significant gender differences. Consistent prevalence trends were observed across regions and countries (Fig. 3; Table S3, Supplemental Digital Content, https://links.lww.com/MD/R133). The relative risk of T1D showed a steady upward trend from 1990 to 2021, with a 124% increase from 1990–1994 to 2015–2021 (95% CI: 1.23–1.25) (Table S4, Supplemental Digital Content, https://links.lww.com/MD/R133). Birth cohort effect analysis revealed a continuous increase in T1D prevalence among consecutive birth cohorts worldwide, with no gender-specific patterns observed (Table S5, Supplemental Digital Content, https://links.lww.com/MD/R133). In addition, our study also anticipated a continued increase in the global prevalence of T1D from 2020 to 2050, without showing significant age-specific effects (Table S6, Supplemental Digital Content, https://links.lww.com/MD/R133).

### 3.3. Inflammatory bowel disease

#### 3.3.1. The estimate of ASPR and AAPC

In 2021, the global ASPR of IBD in populations aged 0 to 69 years was estimated at 84.03 (95% UI: 74.71–93.40). America exhibited the highest regional ASPR, and Canada had the highest national ASPR (Table S8, Supplemental Digital Content, https://links.lww.com/MD/R133). The age-specific differences of ASPR were evident across different life cycles, and showed a “peak” pattern, which was 0.03 in childhood (95% UI: 0.03–0.04), 107.56 in adolescence (95% UI: 91.05–125.93), 61.66 in young and middle-aged cycle (95% UI: 51.67–72.52), 55.62 in middle and old-aged cycles (95% UI: 48.07–63.97). Notably, during adolescence, males showed a higher ASPR of IBD in males as compared to females (Val: 107.39 vs 36.92) (Table 1; Table S1, Supplemental Digital Content, https://links.lww.com/MD/R133; Fig. 1C). From 1990 to 2021, the global and regional AAPC of IBD among population aged 0 to 69 generally decreased (Fig. 2). Childhood and adolescent AAPC remained stable, while young and middle-aged cycle were decrease by 0.45% (‐0.48%, ‐0.41%). Middle and old-aged cycle saw a decrease by 0.61% (95% CI: ‐0.65% to ‐0.56%) (Table 2; Table S2, Supplemental Digital Content, https://links.lww.com/MD/R133; Fig. 2C).

#### 3.3.2. The estimation of APC effects and prediction of ASPR

The age-related findings suggested that the global risk of IBD was lowest in childhood, increasing with age, with both males and females showing a similar trend, lacking gender specificity. Generally, the age effect of IBD peaked in middle-aged and elderly populations across regions and countries (Fig. 3; Table S3, Supplemental Digital Content, https://links.lww.com/MD/R133). The period effect showed a continuous decline in the relative risk of IBD from 1990 to 2021, with a 15% decrease from 1990–1994 to 2015–2021 (95% CI: 0.80–0.92) (Table S4, Supplemental Digital Content, https://links.lww.com/MD/R133). Birth cohort effect findings indicated a decreasing trend in the relative risk of IBD among consecutive birth cohorts, individuals who were born between 1920 and 1929 have the highest IBD risk (Table S5, Supplemental Digital Content, https://links.lww.com/MD/R133). Our study also anticipated a continued increase in the global prevalence of IBD from 2020 to 2032, with the notable gender and age-specific trends (Table S6, Supplemental Digital Content, https://links.lww.com/MD/R133).

### 3.4. Multiple sclerosis

#### 3.4.1. The estimate of ASPR and AAPC

In 2021, the global ASPR of MS was 115.16 (95% UI: 103.08–127.93) (Table 1; Fig. 1), America and Spain showed the highest ASPR at regional and national levels, respectively (Table S9, Supplemental Digital Content, https://links.lww.com/MD/R133). The global ASPR of MS exhibited a downward trend across life cycles, which was 87.78 in childhood (95% UI: 73.54–104.6), 19.39 in adolescence (95% UI: 16.19–23.01), 21.41 in young and middle-aged cycle (95% UI: 17.53–25.89), 7.23 in middle and old-aged cycle (95% UI: 6.27–8.25). Moreover, the ASPR of MS was higher in females than males across life cycles (Table 1; Table S1, Supplemental Digital Content, https://links.lww.com/MD/R133; Fig. 1D). From 1990 to 2021, there was a declined trend of ASPR in MS for individuals aged 0 to 69 globally and regionally. The AAPC in childhood remained stable, and then decreased by ‐0.08% in adolescence (95% CI: ‐0.12% to ‐0.03%), 0.26% in middle and young-aged cycle (95% CI: ‐0.28% to ‐0.23%), 0.15% in middle and old-aged cycle (95% CI: ‐0.26% to ‐0.03%) (Table 2; Table S2, Supplemental Digital Content, https://links.lww.com/MD/R133; Fig. 2D).

#### 3.4.2. The estimation of APC effects and prediction of ASPR

The APC effects indicated that the global risk of developing MS was lowest during childhood, increased sharply in middle-aged and elderly individuals aged 54 to 59 years, and then gradually decreased with age. During adolescence, females generally have a higher risk of developing MS compared to males, suggesting a clear gender specificity of MS (Fig. 3; Table S3, Supplemental Digital Content, https://links.lww.com/MD/R133). The period effect revealed a gradual decline in the relative risk of MS from 1990 to 2021. In comparison to the 1990 to 1994 period, the risk of MS in 2015 to 2021 decreased to approximately 0.97 of the previous levels (95% CI: 0.97 to 0.98) (Table S4, Supplemental Digital Content, https://links.lww.com/MD/R133). Results from the birth cohort effect showed that the prevalence of MS in successive birth cohorts worldwide continued to decrease with changes in birth periods (Table S5, Supplemental Digital Content, https://links.lww.com/MD/R133). Our study also anticipated a continued increase in the global ASPR of MS from 2020 to 2032, characterized by notable gender and age-specific trends, and the ASPR of MS was projected to remain stable until 2050 (Table S6, Supplemental Digital Content, https://links.lww.com/MD/R133).

### 3.5. Psoriasis

#### 3.5.1. The estimate of ASPR and AAPC

In 2021, the global ASPR of psoriasis was 106.48 (95% UI: 96.26–117.35), South-East Asia and Iceland showed a highest ASPR at regional and national levels, respectively (Table S10, Supplemental Digital Content, https://links.lww.com/MD/R133). The ASPR of psoriasis varied across life cycles, negligible in childhood, with males having a higher prevalence than females in adolescence. Middle and young-aged cycle saw a slight decrease, peaking in middle and old-aged cycle (Table 1; Table S1, Supplemental Digital Content, https://links.lww.com/MD/R133; Fig. 1E). From 1990 to 2021, the AAPC varies of psoriasis for individuals aged 0 to 69 years exhibited a decreasing trend globally and regionally. Childhood AAPC remained stable, while adolescence saw a 0.71% decrease for both genders. Middle and young-aged populations decreased by 0.98% for both genders, and middle and old age decreased by 0.82%, with slightly higher decreases in males (Table 2; Table S2, Supplemental Digital Content, https://links.lww.com/MD/R133; Fig. 2E).

#### 3.5.2. The estimation of APC effects and prediction of ASPR

The APC effects on psoriasis risk globally, regionally, and nationally followed a three-stage trend: rapid rise in childhood, leveling off in adolescence, and peaking in the middle and old-aged cycle. There was no significant gender difference in psoriasis risk (Fig. 3; Table S3, Supplemental Digital Content, https://links.lww.com/MD/R133). The period effect analysis revealed a significant decrease in the relative risk of psoriasis from 1990 to 2021, decreasing to approximately 0.79 compared to the 1990 to 1994 period (Table S4, Supplemental Digital Content, https://links.lww.com/MD/R133). Birth cohort effect analysis showed a declining prevalence of psoriasis across consecutive global birth cohorts, with individuals born in the 1920 to 1929 cohort having a higher risk compared to those born in the 1980 to 1989 cohort (Table S5, Supplemental Digital Content, https://links.lww.com/MD/R133). Notably, the risk of psoriasis began to slowly increase in individuals born in the 2010 to 2021 cohort. Our study also anticipated a continued increase in the global prevalence of psoriasis from 2020 to 2032, characterized by notable gender and age-specific trends, and the ASPR of psoriasis was projected to remain stable until 2050 (Table S6, Supplemental Digital Content, https://links.lww.com/MD/R133).

### 3.6. Rheumatic heart disease

#### 3.6.1. The estimate of ASPR and AAPC

In 2021, the global ASPR of RHD was 89.37 (95% UI: 79.68–100.17). The highest ASPR was observed in the Southeast Asia region, and Uganda showed the highest ASPR at national level (Table S11, Supplemental Digital Content, https://links.lww.com/MD/R133). The prevalence of RHD showed a “peak” pattern across life cycles, where the ASPR was 0.42 in childhood (95% UI: 0.35–0.47), 336.70 in adolescence (95% UI: 202.83–523.20), 729.04 in young and middle-aged cycle (95% UI: 529.90–9978.13), and 217.00 in middle and old-aged cycles (95% UI: 175.85–273.66). In addition, the ASPR of females was similarly higher than males among different life cycles (Table 1; Table S1, Supplemental Digital Content, https://links.lww.com/MD/R133; Fig. 1F). From 1990 to 2021, the AAPC trend of RHD at ages 0 to 69 remained stable globally and regionally (Fig. 2). The AAPC remained stable globally in childhood, then increased by 0.73% in adolescence and 0.55% in the middle and young cycle, and the AAPC trend remained relatively constant in the middle and old-aged cycle (Table 2; Table S2, Supplemental Digital Content, https://links.lww.com/MD/R133; Fig. 2F).

#### 3.6.2. The estimation of APC effects and prediction of ASPR

The APC effects on the global risk of RHD varied across life cycles, where it increased in childhood, peaked in young and middle-aged individuals around 30 to 34 years old, then decreased with a notable shift among middle-aged and elderly populations aged 60 to 64 years (Fig. 3; Table S3, Supplemental Digital Content, https://links.lww.com/MD/R133). The period effect showed a stable relative risk of RHD from 1990 to 2021, with a gradual increased since 2000, reaching approximately 1.13-fold time as the original value by 2015 to 2021 (Table S4, Supplemental Digital Content, https://links.lww.com/MD/R133). The birth cohort effect revealed that the highest levels of relative risk of RHD were shown in the 1940 to 1949 and 1945 to 1954 cohorts. Moreover, populations born between 2010 and 2021 have a higher risk of RHD compared to those born in the 1980 to 1989 cohort (Table S5, Supplemental Digital Content, https://links.lww.com/MD/R133). Our study anticipated a continued increase in the global ASPR of RHD from 2020 to 2032, with the notable gender and age-specific trends, and the ASPR of psoriasis was projected to remain stable until 2050 (Table S6, Supplemental Digital Content, https://links.lww.com/MD/R133).

### 3.7. Rheumatoid arthritis

#### 3.7.1. The estimate of ASPR and AAPC

In 2021, the global ASPR of RA was 107.72 (95% UI: 96.20–119.89), where South-East Asia and the United States of America had the peak levels of ASPR of RA at regional and national levels, respectively (Table S12, Supplemental Digital Content, https://links.lww.com/MD/R133). Across life cycles, there was an increasing trend in ASPR of RA, where childhood remained low at 0.02, then increased to 172.84 in adolescence, after downward to 150.95 in the middle and young-aged cycle, the ASPR was increased to 209.35 in the middle and old-aged cycle (Table 1; Table S1, Supplemental Digital Content, https://links.lww.com/MD/R133; Fig. 1G). From 1990 to 2021, the AAPC for RA in individuals aged 0 to 69 years remained stable globally and regionally (Fig. 1). The trend increased slightly in adolescence and middle and young adulthood in both males and females. Of note, in middle and old-aged, males showed a higher increase of AAPC than females (Table 2; Table S2, Supplemental Digital Content, https://links.lww.com/MD/R133; Fig. 2G).

#### 3.7.2. The estimation of APC effects and prediction of ASPR

Unlike other ADs, the risk of developing RA follows a distinct age-related pattern. The ASPR remains stable throughout childhood but begins to rise rapidly around the age of 15 during adolescence. Notably, there is a marked increase in ASPR of RA among populations aged between 35 to 39 years in both males and females (Fig. 3; Table S3, Supplemental Digital Content, https://links.lww.com/MD/R133). The relative risk of developing RA from 1990 to 2021 initially remained stable before gradually increasing (Table S4, Supplemental Digital Content, https://links.lww.com/MD/R133). The birth cohort effect analysis revealed a continual increase in the prevalence of RA across successive birth cohorts globally, with variations based on birth period. Individuals born in the 2000 to 2009 and 2005 to 2014 cohorts had a higher risk compared to those born in the 1980 to 1989 cohort (Table S5, Supplemental Digital Content, https://links.lww.com/MD/R133). Our study also revealed a continued rise in the global ASPR of RA from 2020 to 2032, but it remained stable until 2050 (Table S6, Supplemental Digital Content, https://links.lww.com/MD/R133).

## 4. Discussion

The prevalence of ADs has been steadily increasing over the past three decades, yet effective cures remain elusive.^[18]^ Accumulating evidence has demonstrated that ADs can occur in any stage of the life cycle, with certain ADs exhibiting higher prevalence rates during specific life cycles.^[3]^ Although the majority of ADs are more prevalent in females, it has also been implied that other types of ADs, such as ankylosing spondylitis (AS), childhood T1D, and myasthenia gravis, are more common in males.^[19]^ Historically, ADs were predominantly associated with females due to observational limitations, leading to delays in treatment.^[20]^ This may be partially attributed to the changes in sex hormones across life cycles. Along with hormonal fluctuations, estrogen and progesterone in females, and androgens in males are hypothesized to have different effects on autoimmunity.^[21]^ This study categorized life cycles based on several key points regarding the dynamic changes of hormone levels to examine the age-specific and gender-specific disease burden of ADs across childhood, adolescence, young and middle-aged cycle, and middle and older aged cycle.

The global ASPR of ADs in populations aged 0 to 69 years has nearly doubled since 1990, indicating that population growth and shifts in social behaviors are driving an increase in the risk of ADs. Global ASPR for AA, T1D, IBD, psoriasis, RHD, and RA have all shown significant increases, highlighting the ongoing challenge of ADs to global health, which may strain healthcare systems. Moreover, we found that Southeast Asia and America undergo the highest risk of ADs. The substantial burden in developing regions may be attributed not only to economic transitions and lifestyle factors but also to underlying disparities in healthcare access and diagnostic capacity.^[22–24]^ Limited healthcare infrastructure in some areas could lead to under-diagnosis or delayed diagnosis, thereby influencing reported prevalence rates. Furthermore, variations in environmental exposures, such as air pollution, infectious agents, and dietary patterns, may also contribute to the heterogeneous geographical distribution of ADs.^[25]^ Nationally, there were some significant variations in ASPR among 204 countries, with developing nations showing the highest rates, and sedentary habits and inactive lifestyles could be contributing factors.^[26]^ It is essential to develop tailored health policies that address the changing trends in ADs prevalence across regions and countries to effectively fulfill the health needs of residents and reduce the burden of this disease.

The marked regional disparities highlighted in our study underscore the influence of a complex interplay of factors beyond genetics. Differences in diagnostic capabilities and healthcare infrastructure likely play a critical role; regions with more robust healthcare systems may detect ADs more efficiently, potentially inflating prevalence estimates, while under-diagnosis might obscure the true burden in resource-limited settings. Concurrently, environmental exposures (including ultraviolet radiation, pollution, and microbial diversity) vary greatly by geography and have been implicated in the pathogenesis of various ADs. Socioeconomic status, which correlates with nutrition, stress, and access to care, further modulates these risks. Therefore, the distinct patterns observed across WHO regions likely reflect a composite picture of biological susceptibility, environmental triggers, and systemic health determinants. Future research should aim to disentangle these drivers through more granular, individual-level data.

During childhood, there was a significant increase in the global ASPR of T1D and MS from 1990 to 2021, with a noticeable clustering in males. However, there were no significant changes observed in ASPR and its sex-specificity in other ADs. These observations suggest a potential role for sex hormones in shaping the immune system early in life. On a regional scale, Europe and Africa showed the highest disease burden in this context and continue to exhibit clear gender biases. It suggested that the risk of ADs in childhood may be influenced by socioeconomic factors and access to healthcare. In response to this, WHO introduced the Children’s Health Redesign strategy in 2021, with a focus on preventing and providing healthcare for chronic noncommunicable diseases among children and adolescents in Africa. This strategy relies on the involvement of various sectors such as healthcare, community, schools, and social security to enhance child and adolescent health.^[27]^ At the national level, there were significant disparities among the 204 countries and regions, with the ASPR of countries like Iraq and Saudi Arabia increasing substantially, aligning with previous research findings.^[28]^ This highlights the considerable heterogeneity in the burden of ADs across different countries and regions, emphasizing the need for adaptable health policies to specific contexts and goals to address health disparities between regions.

In adolescence, the global ASPR of ADs has doubled compared to childhood from 1990 to 2021. Females tended to face more severe disease risks during this period, particularly for rheumatic diseases like RA and RHD, this results were in line with the findings of Malagon et al.^[29]^ On a regional scale, the European and American regions exhibited the fastest-growing ASPR in psoriasis, while the escalating prevalence of RHD was imposing a significant economic burden on regions like southeast Asia and the Eastern Mediterranean. These disparities in disease trends and regional variations may be attributed to variations in pathogenic mechanisms among different diseases, as well as environmental and socioeconomic factors. Nationally, there were notable differences in ASPR in ADs across countries, indicating varying disease burdens and treatment management strategies globally. It is recommended to enhance disease management for ADs and develop tailored health policies based on specific prevalence patterns in different countries and regions.

During the young and middle-aged cycle, there was a rapid increase in the global ASPR of ADs from 1990 to 2021. Globally, seven ADs experienced varying degrees of growth during this cycle, with AA, psoriasis, and RHD showing the largest growth rates. Providing females of childbearing age with pregnancy planning and targeted care is essential to address their healthcare needs for ADs. Regionally, the European and American regions had the highest ASPR growth rates, indicating a clustering of ADs in certain countries during this life cycle. Nationally, there were significant differences in ASPR changes in ADs among different countries, with an obvious gender specificity.

In the middle and old-aged cycle, the global burden of ADs and chronic inflammatory responses tended to increase due to aging and fluctuating hormone levels. Interestingly, gender did not seem to play a significant role in affecting the disease burden of ADs in this cycle. This suggests that factors associated with aging, rather than hormonal effects alone, may have a more prominent influence on immune disorders and inflammation in this life cycle. On a regional scale, the prevalence of ADs in the African region has surged over tenfold. These differences may be attributed to various factors such as social, cultural, and behavioral variations, and other modifiable risk factors.^[30]^ Nationally, significant variations in the burden of ADs existed across different countries, emphasizing the need for tailored health policies to address these disparities effectively.

Population growth and aging are key contributors to the rising global prevalence of ADs, with variations in hormone levels across different life cycles also impacting disease occurrence. Using data from GBD 2021, this study assessed global, regional, and national disparities in ADs prevalence and changes in trends from 1990 to 2021. The analysis delved into the effects of age, period, and birth cohort on these trends. Overall, the risk of ADs rises with age, particularly in middle and old age, showing clear sex-specific patterns that are consistent with a potential role for hormonal influences. The life cycle segmented by hormone levels demonstrates the intricate connection between immunity and aging.^[2]^ The lower ADs risk in childhood, a period with distinct hormone levels compared to other life cycles, is consistent with the theorized immunomodulatory role of sex hormones. In females, estrogen levels rise from adolescence, potentially triggering immune disorders and responses, peaking during reproductive years and perimenopause. Conversely, males exhibit higher childhood ADs risk compared to females, but lower risk in subsequent life cycles, possibly due to sex hormone-mediated immune regulation. As age increases, the level of testosterone hormone in males continues to increase, which greatly promotes the body’s immunosuppressive effect.^[31]^ In short terms, it is proposed that the rise in female estrogen may trigger an inflammatory immune response, while male androgen might dampen the immune response.^[32,33]^ Furthermore, it is important to consider the potential influence of sex chromosomes and gut microbiota in the development of sex-biased autoimmunity.^[34–36]^

From 1990 to 2021, the AAPC of ADs rose by almost half, indicating a significant increase in the population affected by these conditions. Our findings, which span the entire lifespan, complement and extend recent studies focusing on younger populations. For instance, a study on adolescents and young adults (AYAs, 15–39 years) also using GBD 2021 data reported increasing incidence trends for RA, IBD, MS, T1D, and psoriasis, mirroring our observations in corresponding age subgroups.^[37]^ Furthermore, the substantial regional and national disparities in AD burden highlighted in our analysis are consistent with research in children and adolescents (0–24 years), which also identified a significant correlation between AD incidence and socioeconomic development indices.^[38]^ Despite the availability of various effective biologics, our projections suggest the overall risk and burden have not been curtailed. Most countries will likely face a substantial disease and economic burden in the future. Due to the chronic nature of ADs and the complex interplay of factors including genetics, environment, and aging, we anticipate that the ASPR of ADs will continue to rise in many countries until around 2032. Therefore, early and targeted interventions are crucial to mitigate the future risk and disease burden of ADs.

In comparison with previous studies, this is the first GBD analysis to comprehensively investigate the temporal trends, sex- and gender-specific ASPR of ADs on a global, regional, and national scale, from the perspectives of life cycle changes on sex hormone levels.^[39]^ Our study had several advantages. First, individuals aged 0 to 69 are categorized into distinct life cycles based on variations in sex hormones. This exploration of complex life cycles can enhance our understanding of the relationship between gender-specificity in ADs and disease epidemic patterns. Second, this study evaluates the AAPC of ADs across the life cycle, offering valuable insights for the development of cost-effective disease prevention and health promotion strategies. Specifically, the ASPR estimates account for differences in age distribution among countries, facilitating comparisons of disease burden across regions and countries. Moreover, analyzing ADs AAPC in various life cycles can provide a deep insight into the overall disease progression and enable a more detailed analysis of the factors contributing to differential outcomes in each life cycle. Subsequently, APC effects are examined, with age-related physiological changes identified through age effects. Period effects are investigated to understand the impact of socioeconomic and environmental factors on disease epidemic trends, while birth cohort effects are studied to evaluate age-specific exposures and susceptibility to ADs. Additionally, conducting predictive analyses can aid in understanding the necessity of enhancing interventions to reduce the burden of ADs.

Nevertheless, there are some limitations to consider. First, this study relied exclusively on the GBD 2021 database. Although the GBD integrates a wide array of global data sources and employs robust modeling techniques, independent cross-validation with other databases (e.g., WHO annual reports or national registries) was not conducted. However, the GBD is widely recognized as a comprehensive and consistent source for global burden estimates, and its use here ensures comparability across regions and time. Second, the disease burden estimates are age-standardized and reported with UIs rather than CIs, therefore, caution is advised when interpreting the findings. Third, and most critically in the context of this study’s objectives, our ecological approach and the lack of individual-level data (e.g., direct measurements of sex hormones or comorbidities) preclude any definitive causal inferences regarding the role of hormones in ADs. While we discuss patterns consistent with hormonal influence across life cycles and sex, these associations should be interpreted with caution and are subject to unmeasured confounding.

## 5. Conclusions

This study demonstrates a significant increase in the global prevalence of ADs from 1990 to 2021 across different life stages, underscoring the need for comprehensive and tailored health strategies. Our findings highlight a concerning growth trend in childhood and adolescence and reveal a substantial disease burden not only in females but also in males, calling for future health policies and resource allocation to address the needs of both genders. The significant burden of ADs among younger populations in underdeveloped regions, such as Africa, necessitates targeted interventions. Furthermore, longitudinal studies are warranted to validate the projected prevalence trends up to 2050 and to assess the effectiveness of targeted interventions in high-risk populations and regions.

## Author contributions

**Conceptualization:** Xian-Pei Xiao.

**Methodology:** Yan Li.

**Validation:** Ming-Yu Wu.

**Writing – original draft:** Xian-Pei Xiao.

**Writing – review & editing:** Xiao Hu, Bi-Yuan Qin.

## Supplementary Material


