# Supplementary material for: Global landscape of autoimmune diseases across different lifespan: A three-decade perspective
Source: Medicine (Baltimore). 2026 Jan 9;105(2):e47140. doi: 10.1097/MD.0000000000047140 (PMC12795021; doi:10.1097/MD.0000000000047140)
Supplement: Supplementary file 1 [file medi-105-e47140-s001.pdf]

**Table S1 Age-standardized prevalence rates of AA in childhood, adolescence, young and middle-age, middle and old-aged period at the global and regional levels in 2021.**

| Measure | Sex | Location | AA | | | | |
|---|---|---|---|---|---|---|---|
| | | | All | Childhood | Adolescence | Young and middle age | Middle and elderly age |
| Prevalence | Male | Afghanistan | 84.13 (73.9 to 94.89) | 212.58 (188.87 to 237.54) | 212.58 (188.87 to 237.54) | 350.05 (311.18 to 391.32) | 207.7 (183.17 to 233.88) |
| Prevalence | Female | Afghanistan | 152.29 (133.8 to 170.57) | 299.45 (263.35 to 337.85) | 299.45 (263.35 to 337.85) | 449.82 (394.99 to 506.15) | 270.89 (236.53 to 307.89) |
| Prevalence | Both | Afghanistan | 117.22 (104.35 to 130.95) | 148.92 (130.8 to 168.18) | 148.92 (130.8 to 168.18) | 249.31 (219.62 to 280.59) | 143.11 (125.25 to 162.9) |
| Prevalence | Male | Albania | 89.17 (78.54 to 100.33) | 240.48 (213.37 to 269.6) | 240.48 (213.37 to 269.6) | 302.61 (268.93 to 338.06) | 233.43 (205.02 to 264.39) |
| Prevalence | Female | Albania | 170.51 (150.08 to 191.81) | 0 (0 to 0) | 0 (0 to 0) | 394.39 (347.4 to 444.33) | 303.52 (264.36 to 346.74) |
| Prevalence | Both | Albania | 127.91 (113.51 to 142.95) | 165.2 (145.61 to 186.82) | 165.2 (145.61 to 186.82) | 205.86 (181.89 to 232.35) | 158.65 (139.41 to 180.49) |
| Prevalence | Male | Algeria | 84.13 (73.9 to 94.89) | 0 (0 to 0) | 0 (0 to 0) | 0 (0 to 0) | 206.49 (182.13 to 232.32) |
| Prevalence | Female | Algeria | 152.29 (133.8 to 170.57) | 282.14 (247.99 to 318.06) | 282.14 (247.99 to 318.06) | 0 (0 to 0) | 270.89 (236.53 to 307.89) |
| Prevalence | Both | Algeria | 117.46 (104.56 to 131.2) | 0 (0 to 0) | 0 (0 to 0) | 0 (0 to 0) | 143.11 (125.25 to 162.9) |
| Prevalence | Male | American Samoa | 108.19 (95.55 to 121.83) | 276.42 (245.38 to 309.42) | 276.42 (245.38 to 309.42) | 0 (0 to 0) | 269.9 (237.38 to 302.58) |
| Prevalence | Female | American Samoa | 192.69 (168.27 to 216.69) | 399.88 (349.06 to 449.99) | 399.88 (349.06 to 449.99) | 0 (0 to 0) | 345.82 (302.1 to 391.48) |
| Prevalence | Both | American Samoa | 149.16 (132.61 to 166.95) | 197.08 (173.41 to 222.18) | 197.08 (173.41 to 222.18) | 0 (0 to 0) | 192.03 (168.22 to 217.97) |
| Prevalence | Male | Andorra | 151.04 (133.89 to 169.5) | 325.2 (287.62 to 364.11) | 325.2 (287.62 to 364.11) | 392.93 (348.38 to 438.15) | 285.56 (252.42 to 321.17) |
| Prevalence | Female | Andorra | 166.54 (146.31 to 187.58) | 337.26 (296.8 to 381.08) | 337.26 (296.8 to 381.08) | 463.13 (407.47 to 521.36) | 418.49 (366.83 to 476.04) |

| | | | | | | | |
|---|---|---|---|---|---|---|---|
| Prevalence | Both | Andorra | 158.51<br>(141.31 to 176.19) | 229.91<br>(202.25 to 262.56) | 229.91<br>(202.25 to 262.56) | 321.37<br>(282.84 to 361.52) | 163.59<br>(142.91 to 185.21) |
| Prevalence | Male | Angola | 88.23<br>(77.51 to 99.4) | 228.7<br>(203.16 to 255.82) | 228.7<br>(203.16 to 255.82) | 302.13<br>(268.51 to 337.51) | 223.09<br>(197.18 to 250.42) |
| Prevalence | Female | Angola | 160.01<br>(141.54 to 178.93) | 327.25<br>(287.41 to 368.24) | 327.25<br>(287.41 to 368.24) | 394.39<br>(347.4 to 444.33) | 285.8<br>(249.35 to 323.09) |
| Prevalence | Both | Angola | 124.78<br>(111.02 to 138.36) | 156.91<br>(137.83 to 176.93) | 156.91<br>(137.83 to 176.93) | 205.86<br>(181.89 to 232.35) | 151.72<br>(132.53 to 172.91) |
| Prevalence | Male | Antigua and Barbuda | 89.17<br>(78.54 to 100.33) | 189.21<br>(167.63 to 212.82) | 189.21<br>(167.63 to 212.82) | 302.05<br>(268.45 to 337.38) | 234.17<br>(205.66 to 265.23) |
| Prevalence | Female | Antigua and Barbuda | 170.51<br>(150.08 to 191.81) | 469.92<br>(413.29 to 529.01) | 469.92<br>(413.29 to 529.01) | 394.39<br>(347.4 to 444.33) | 303.52<br>(264.36 to 346.74) |
| Prevalence | Both | Antigua and Barbuda | 129.04<br>(114.53 to 144.27) | 128.99<br>(113.48 to 146.02) | 128.99<br>(113.48 to 146.02) | 205.86<br>(181.89 to 232.35) | 158.65<br>(139.41 to 180.49) |
| Prevalence | Male | Argentina | 140.81<br>(124.13 to 158.33) | 0<br>(0 to 0) | 0<br>(0 to 0) | 301.18<br>(267.73 to 336.29) | 322.25<br>(284.36 to 362.68) |
| Prevalence | Female | Argentina | 191.88<br>(168.76 to 214.53) | 399.84<br>(351.57 to 450.49) | 399.84<br>(351.57 to 450.49) | 394.39<br>(347.4 to 444.33) | 479.89<br>(421.83 to 544.47) |
| Prevalence | Both | Argentina | 166.01<br>(147.42 to 184.95) | 0<br>(0 to 0) | 0<br>(0 to 0) | 205.86<br>(181.89 to 232.35) | 152.59<br>(133.17 to 173.27) |
| Prevalence | Male | Armenia | 89.17<br>(78.54 to 100.33) | 0<br>(0 to 0) | 0<br>(0 to 0) | 0<br>(0 to 0) | 237.39<br>(208.42 to 268.95) |
| Prevalence | Female | Armenia | 170.51<br>(150.08 to 191.81) | 316.6<br>(278.53 to 358.19) | 316.6<br>(278.53 to 358.19) | 0<br>(0 to 0) | 303.52<br>(264.36 to 346.74) |
| Prevalence | Both | Armenia | 127.12<br>(112.82 to 142.04) | 0<br>(0 to 0) | 0<br>(0 to 0) | 0<br>(0 to 0) | 158.65<br>(139.41 to 180.49) |
| Prevalence | Male | Australia | 140.81<br>(124.13 to 158.33) | 0<br>(0 to 0) | 0<br>(0 to 0) | 299.27<br>(266.1 to 334.27) | 319.75<br>(282.14 to 359.8) |
| Prevalence | Female | Australia | 191.88<br>(168.76 to 214.53) | 399.84<br>(351.57 to 450.49) | 399.84<br>(351.57 to 450.49) | 394.39<br>(347.4 to 444.33) | 479.89<br>(421.83 to 544.47) |
| Prevalence | Both | Australia | 165.68<br>(147.13 to 184.61) | 0<br>(0 to 0) | 0<br>(0 to 0) | 205.86<br>(181.89 to 232.35) | 152.59<br>(133.17 to 173.27) |
| Prevalence | Male | Austria | 151.04 | 389.97 | 389.97 | 385.89 | 291.06 |

| | | | | | | | |
|---|---|---|---|---|---|---|---|
| | | | (133.89 to 169.5) | (346.04 to 435.99) | (346.04 to 435.99) | (342.97 to 431.88) | (257.21 to 327.39) |
| Prevalence | Female | Austria | 166.54<br>(146.31 to 187.58) | 238.34<br>(209.37 to 268.49) | 238.34<br>(209.37 to 268.49) | 459.5<br>(404.27 to 519.73) | 418.49<br>(366.83 to 476.04) |
| Prevalence | Both | Austria | 158.48<br>(141.22 to 176.21) | 314.58<br>(276.87 to 355.54) | 314.58<br>(276.87 to 355.54) | 321.37<br>(282.84 to 361.52) | 163.59<br>(142.91 to 185.21) |
| Prevalence | Male | Azerbaijan | 89.17<br>(78.54 to 100.33) | 108.07<br>(95.98 to 120.99) | 108.07<br>(95.98 to 120.99) | 290.43<br>(258.07 to 324.55) | 235.16<br>(206.5 to 266.38) |
| Prevalence | Female | Azerbaijan | 170.51<br>(150.08 to 191.81) | 390.08<br>(343.05 to 441.47) | 390.08<br>(343.05 to 441.47) | 370.63<br>(326.56 to 417.54) | 303.52<br>(264.36 to 346.74) |
| Prevalence | Both | Azerbaijan | 127.2<br>(112.88 to 142.11) | 75.61<br>(66.54 to 85.44) | 75.61<br>(66.54 to 85.44) | 198.07<br>(174.09 to 222.9) | 158.65<br>(139.41 to 180.49) |
| Prevalence | Male | Bahrain | 84.13<br>(73.9 to 94.89) | 241.19<br>(214.11 to 269.65) | 241.19<br>(214.11 to 269.65) | 451.38<br>(401.7 to 503.02) | 234.57<br>(206.01 to 265.71) |
| Prevalence | Female | Bahrain | 152.29<br>(133.8 to 170.57) | 323<br>(283.88 to 369.88) | 323<br>(283.88 to 369.88) | 612.22<br>(539.97 to 690.04) | 303.52<br>(264.36 to 346.74) |
| Prevalence | Both | Bahrain | 117.31<br>(104.42 to 131.04) | 164.16<br>(144.8 to 185.48) | 164.16<br>(144.8 to 185.48) | 288.36<br>(253.38 to 324.29) | 158.65<br>(139.41 to 180.49) |
| Prevalence | Male | Bangladesh | 79.97<br>(70.53 to 90.14) | 243.38<br>(216.39 to 271.61) | 243.38<br>(216.39 to 271.61) | 287.49<br>(255.62 to 321.21) | 187.18<br>(165.54 to 210.54) |
| Prevalence | Female | Bangladesh | 150.23<br>(132.53 to 169.2) | 129.82<br>(114.27 to 145.77) | 129.82<br>(114.27 to 145.77) | 370.63<br>(326.56 to 417.54) | 270.89<br>(236.53 to 307.89) |
| Prevalence | Both | Bangladesh | 115.85<br>(103.21 to 129.3) | 183.89<br>(161.46 to 207.58) | 183.89<br>(161.46 to 207.58) | 198.07<br>(174.09 to 222.9) | 143.11<br>(125.25 to 162.9) |
| Prevalence | Male | Barbados | 89.17<br>(78.54 to 100.33) | 233.36<br>(206.55 to 261.45) | 233.36<br>(206.55 to 261.45) | 0<br>(0 to 0) | 204.49<br>(180.1 to 229.68) |
| Prevalence | Female | Barbados | 170.51<br>(150.08 to 191.81) | 87.28<br>(77.44 to 99.11) | 87.28<br>(77.44 to 99.11) | 0<br>(0 to 0) | 267.36<br>(233.33 to 302.32) |
| Prevalence | Both | Barbados | 128.95<br>(114.44 to 144.16) | 160.7<br>(141.14 to 181.75) | 160.7<br>(141.14 to 181.75) | 0<br>(0 to 0) | 141.85<br>(123.93 to 161.48) |
| Prevalence | Male | Belarus | 89.17<br>(78.54 to 100.33) | 269.34<br>(239.1 to 304.4) | 269.34<br>(239.1 to 304.4) | 0<br>(0 to 0) | 235.46<br>(206.76 to 266.73) |
| Prevalence | Female | Belarus | 170.51<br>(150.08 to 191.81) | 312.06<br>(274.47 to 352.23) | 312.06<br>(274.47 to 352.23) | 0<br>(0 to 0) | 303.52<br>(264.36 to 346.74) |

| | | | | | | | |
|---|---|---|---|---|---|---|---|
| Prevalence | Both | Belarus | 128.62<br>(114.15 to 143.78) | 181.89<br>(160.92 to 205.71) | 181.89<br>(160.92 to 205.71) | 0<br>(0 to 0) | 158.65<br>(139.41 to 180.49) |
| Prevalence | Male | Belgium | 151.04<br>(133.89 to 169.5) | 242.21<br>(214.86 to 271.58) | 242.21<br>(214.86 to 271.58) | 356.29<br>(316.6 to 398.45) | 237.02<br>(208.09 to 268.55) |
| Prevalence | Female | Belgium | 166.54<br>(146.31 to 187.58) | 0<br>(0 to 0) | 0<br>(0 to 0) | 449.82<br>(394.99 to 506.15) | 303.52<br>(264.36 to 346.74) |
| Prevalence | Both | Belgium | 158.62<br>(141.4 to 176.36) | 165.2<br>(145.61 to 186.82) | 165.2<br>(145.61 to 186.82) | 249.31<br>(219.62 to 280.59) | 158.65<br>(139.41 to 180.49) |
| Prevalence | Male | Belize | 89.17<br>(78.54 to 100.33) | 0<br>(0 to 0) | 0<br>(0 to 0) | 0<br>(0 to 0) | 290.36<br>(256.61 to 326.62) |
| Prevalence | Female | Belize | 170.51<br>(150.08 to 191.81) | 348.54<br>(306.54 to 393.51) | 348.54<br>(306.54 to 393.51) | 0<br>(0 to 0) | 418.49<br>(366.83 to 476.04) |
| Prevalence | Both | Belize | 129.98<br>(115.39 to 145.36) | 0<br>(0 to 0) | 0<br>(0 to 0) | 0<br>(0 to 0) | 163.59<br>(142.91 to 185.21) |
| Prevalence | Male | Benin | 88.23<br>(77.51 to 99.4) | 244.79<br>(217.43 to 273.96) | 244.79<br>(217.43 to 273.96) | 326.94<br>(290.77 to 364.73) | 230.85<br>(202.81 to 261.46) |
| Prevalence | Female | Benin | 160.01<br>(141.54 to 178.93) | 301.24<br>(263.67 to 343.57) | 301.24<br>(263.67 to 343.57) | 444.13<br>(391.43 to 500.23) | 303.52<br>(264.36 to 346.74) |
| Prevalence | Both | Benin | 124.56<br>(110.83 to 138.12) | 166.91<br>(147.31 to 188.6) | 166.91<br>(147.31 to 188.6) | 221.77<br>(195.33 to 250.02) | 158.65<br>(139.41 to 180.49) |
| Prevalence | Male | Bermuda | 89.17<br>(78.54 to 100.33) | 0<br>(0 to 0) | 0<br>(0 to 0) | 0<br>(0 to 0) | 219.48<br>(194.03 to 246.31) |
| Prevalence | Female | Bermuda | 170.51<br>(150.08 to 191.81) | 297.31<br>(261.55 to 335.2) | 297.31<br>(261.55 to 335.2) | 0<br>(0 to 0) | 285.8<br>(249.35 to 323.09) |
| Prevalence | Both | Bermuda | 129.46<br>(114.89 to 144.75) | 0<br>(0 to 0) | 0<br>(0 to 0) | 0<br>(0 to 0) | 151.72<br>(132.53 to 172.91) |
| Prevalence | Male | Bhutan | 79.97<br>(70.53 to 90.14) | 241.75<br>(214.5 to 270.87) | 241.75<br>(214.5 to 270.87) | 283.43<br>(252.01 to 316.55) | 231.86<br>(203.71 to 262.56) |
| Prevalence | Female | Bhutan | 150.23<br>(132.53 to 169.2) | 0<br>(0 to 0) | 0<br>(0 to 0) | 370.63<br>(326.56 to 417.54) | 303.52<br>(264.36 to 346.74) |
| Prevalence | Both | Bhutan | 114.79<br>(102.23 to 128.13) | 165.2<br>(145.61 to 186.82) | 165.2<br>(145.61 to 186.82) | 198.07<br>(174.09 to 222.9) | 158.65<br>(139.41 to 180.49) |
| Prevalence | Male | Bolivarian Republic of Venezuela | 0 | 207.21 | 207.21 | 0 | 201.67 |

| | | | | | | | |
|---|---|---|---|---|---|---|---|
| | | | (0 to 0) | (183.83 to 232.16) | (183.83 to 232.16) | (0 to 0) | (177.54 to 226.6) |
| Prevalence | Female | Bolivarian Republic of Venezuela | 0<br>(0 to 0) | 307.51<br>(266.75 to 347.13) | 307.51<br>(266.75 to 347.13) | 0<br>(0 to 0) | 267.36<br>(233.33 to 302.32) |
| Prevalence | Both | Bolivarian Republic of Venezuela | 0<br>(0 to 0) | 146.18<br>(128.45 to 165.4) | 146.18<br>(128.45 to 165.4) | 0<br>(0 to 0) | 141.85<br>(123.93 to 161.48) |
| Prevalence | Male | Bosnia and Herzegovina | 89.17<br>(78.54 to 100.33) | 237.65<br>(210.83 to 266.05) | 237.65<br>(210.83 to 266.05) | 0<br>(0 to 0) | 232.07<br>(203.9 to 262.82) |
| Prevalence | Female | Bosnia and Herzegovina | 170.51<br>(150.08 to 191.81) | 349.03<br>(307.48 to 400.67) | 349.03<br>(307.48 to 400.67) | 0<br>(0 to 0) | 303.52<br>(264.36 to 346.74) |
| Prevalence | Both | Bosnia and Herzegovina | 128.83<br>(114.33 to 144.02) | 162.86<br>(143.47 to 184.17) | 162.86<br>(143.47 to 184.17) | 0<br>(0 to 0) | 158.65<br>(139.41 to 180.49) |
| Prevalence | Male | Botswana | 88.23<br>(77.51 to 99.4) | 242.28<br>(215.19 to 271.27) | 242.28<br>(215.19 to 271.27) | 298.95<br>(265.88 to 333.82) | 232.42<br>(204.2 to 263.21) |
| Prevalence | Female | Botswana | 160.01<br>(141.54 to 178.93) | 296.98<br>(260.29 to 339.1) | 296.98<br>(260.29 to 339.1) | 394.39<br>(347.4 to 444.33) | 303.52<br>(264.36 to 346.74) |
| Prevalence | Both | Botswana | 123.83<br>(110.15 to 137.35) | 166.64<br>(147.06 to 188.32) | 166.64<br>(147.06 to 188.32) | 205.86<br>(181.89 to 232.35) | 158.65<br>(139.41 to 180.49) |
| Prevalence | Male | Brazil | 89.75<br>(79.66 to 101.18) | 0<br>(0 to 0) | 0<br>(0 to 0) | 0<br>(0 to 0) | 224.18<br>(198.06 to 251.67) |
| Prevalence | Female | Brazil | 171.11<br>(151.12 to 192.76) | 297.31<br>(261.55 to 335.2) | 297.31<br>(261.55 to 335.2) | 0<br>(0 to 0) | 285.8<br>(249.35 to 323.09) |
| Prevalence | Both | Brazil | 129.81<br>(116.07 to 145.09) | 0<br>(0 to 0) | 0<br>(0 to 0) | 0<br>(0 to 0) | 151.72<br>(132.53 to 172.91) |
| Prevalence | Male | Brunei Darussalam | 140.81<br>(124.13 to 158.33) | 0<br>(0 to 0) | 0<br>(0 to 0) | 0<br>(0 to 0) | 235.76<br>(208.86 to 263.71) |
| Prevalence | Female | Brunei Darussalam | 190.29<br>(168.4 to 214.44) | 318.24<br>(279.63 to 359.55) | 318.24<br>(279.63 to 359.55) | 0<br>(0 to 0) | 304.86<br>(267.67 to 343.84) |
| Prevalence | Both | Brunei Darussalam | 164.08<br>(146.69 to 183.12) | 0<br>(0 to 0) | 0<br>(0 to 0) | 0<br>(0 to 0) | 159.48<br>(139.18 to 180.85) |
| Prevalence | Male | Bulgaria | 89.17<br>(78.54 to 100.33) | 95.13<br>(85.48 to 106.86) | 95.13<br>(85.48 to 106.86) | 0<br>(0 to 0) | 311.3<br>(273.36 to 350.38) |
| Prevalence | Female | Bulgaria | 170.51<br>(150.08 to 191.81) | 431.99<br>(379.46 to 488.69) | 431.99<br>(379.46 to 488.69) | 0<br>(0 to 0) | 475.68<br>(415.44 to 539.73) |

| | | | | | | | |
|---|---|---|---|---|---|---|---|
| Prevalence | Both | Bulgaria | 128.61<br>(114.14 to 143.76) | 87.51<br>(77.66 to 98.69) | 87.51<br>(77.66 to 98.69) | 0<br>(0 to 0) | 152.59<br>(133.17 to 173.27) |
| Prevalence | Male | Burkina Faso | 88.23<br>(77.51 to 99.4) | 237.16<br>(210.81 to 264.85) | 237.16<br>(210.81 to 264.85) | 0<br>(0 to 0) | 231.31<br>(203.24 to 261.94) |
| Prevalence | Female | Burkina Faso | 160.01<br>(141.54 to 178.93) | 320.77<br>(281.58 to 366.91) | 320.77<br>(281.58 to 366.91) | 0<br>(0 to 0) | 303.52<br>(264.36 to 346.74) |
| Prevalence | Both | Burkina Faso | 124.18<br>(110.48 to 137.71) | 164.28<br>(144.91 to 185.6) | 164.28<br>(144.91 to 185.6) | 0<br>(0 to 0) | 158.65<br>(139.41 to 180.49) |
| Prevalence | Male | Burundi | 91.6<br>(81.03 to 103.15) | 256.68<br>(225.76 to 287.83) | 256.68<br>(225.76 to 287.83) | 0<br>(0 to 0) | 224<br>(197.84 to 251.47) |
| Prevalence | Female | Burundi | 157.76<br>(138.72 to 177.99) | 293.11<br>(257.92 to 330.57) | 293.11<br>(257.92 to 330.57) | 0<br>(0 to 0) | 285.8<br>(249.35 to 323.09) |
| Prevalence | Both | Burundi | 125.8<br>(112.29 to 140.5) | 174.5<br>(152.75 to 197.18) | 174.5<br>(152.75 to 197.18) | 0<br>(0 to 0) | 151.72<br>(132.53 to 172.91) |
| Prevalence | Male | Cambodia | 128.96<br>(113.35 to 144.69) | 291.54<br>(258.59 to 326.38) | 291.54<br>(258.59 to 326.38) | 0<br>(0 to 0) | 213.72<br>(188.91 to 241.23) |
| Prevalence | Female | Cambodia | 192.69<br>(168.27 to 216.69) | 212.34<br>(186.74 to 240.25) | 212.34<br>(186.74 to 240.25) | 0<br>(0 to 0) | 283.72<br>(247.73 to 323.19) |
| Prevalence | Both | Cambodia | 159.99<br>(142.63 to 178.59) | 206.2<br>(181.52 to 232.44) | 206.2<br>(181.52 to 232.44) | 0<br>(0 to 0) | 152.91<br>(133.23 to 174.26) |
| Prevalence | Male | Cameroon | 88.23<br>(77.51 to 99.4) | 0<br>(0 to 0) | 0<br>(0 to 0) | 0<br>(0 to 0) | 220.04<br>(194.48 to 246.93) |
| Prevalence | Female | Cameroon | 160.01<br>(141.54 to 178.93) | 297.31<br>(261.55 to 335.2) | 297.31<br>(261.55 to 335.2) | 0<br>(0 to 0) | 285.8<br>(249.35 to 323.09) |
| Prevalence | Both | Cameroon | 123.99<br>(110.3 to 137.52) | 0<br>(0 to 0) | 0<br>(0 to 0) | 0<br>(0 to 0) | 151.72<br>(132.53 to 172.91) |
| Prevalence | Male | Canada | 127<br>(112.11 to 143.18) | 296.53<br>(263.31 to 332.27) | 296.53<br>(263.31 to 332.27) | 0<br>(0 to 0) | 292.26<br>(256.97 to 327.85) |
| Prevalence | Female | Canada | 250.87<br>(221.99 to 281.11) | 399.88<br>(349.06 to 449.99) | 399.88<br>(349.06 to 449.99) | 0<br>(0 to 0) | 345.82<br>(302.1 to 391.48) |
| Prevalence | Both | Canada | 187.32<br>(167.59 to 208.94) | 235.24<br>(206.94 to 265.66) | 235.24<br>(206.94 to 265.66) | 0<br>(0 to 0) | 228.88<br>(200.81 to 259.42) |
| Prevalence | Male | Central African Republic | 88.23 | 250.26 | 250.26 | 0 | 219.19 |

| | | | | | | | |
|---|---|---|---|---|---|---|---|
| | | | (77.51 to 99.4) | (220.57 to 280.69) | (220.57 to 280.69) | (0 to 0) | (193.77 to 245.98) |
| Prevalence | Female | Central African Republic | 160.01<br>(141.54 to 178.93) | 293.11<br>(257.92 to 330.57) | 293.11<br>(257.92 to 330.57) | 0<br>(0 to 0) | 285.8<br>(249.35 to 323.09) |
| Prevalence | Both | Central African Republic | 124.46<br>(110.73 to 138.02) | 174.5<br>(152.75 to 197.18) | 174.5<br>(152.75 to 197.18) | 0<br>(0 to 0) | 151.72<br>(132.53 to 172.91) |
| Prevalence | Male | Chad | 88.23<br>(77.51 to 99.4) | 427.53<br>(376.83 to 477.82) | 427.53<br>(376.83 to 477.82) | 0<br>(0 to 0) | 397.07<br>(348.43 to 446.4) |
| Prevalence | Female | Chad | 160.01<br>(141.54 to 178.93) | 514.72<br>(452.67 to 581.62) | 514.72<br>(452.67 to 581.62) | 0<br>(0 to 0) | 654.16<br>(570.44 to 740.12) |
| Prevalence | Both | Chad | 124.52<br>(110.79 to 138.08) | 187.54<br>(163.5 to 212.65) | 187.54<br>(163.5 to 212.65) | 0<br>(0 to 0) | 130.78<br>(113.79 to 148.56) |
| Prevalence | Male | Chile | 140.81<br>(124.13 to 158.33) | 227.7<br>(202.3 to 254.68) | 227.7<br>(202.3 to 254.68) | 299.05<br>(265.86 to 334.11) | 218.66<br>(193.27 to 245.36) |
| Prevalence | Female | Chile | 191.88<br>(168.76 to 214.53) | 327.25<br>(287.41 to 368.24) | 327.25<br>(287.41 to 368.24) | 394.39<br>(347.4 to 444.33) | 285.8<br>(249.35 to 323.09) |
| Prevalence | Both | Chile | 165.78<br>(147.22 to 184.72) | 156.91<br>(137.83 to 176.93) | 156.91<br>(137.83 to 176.93) | 205.86<br>(181.89 to 232.35) | 151.72<br>(132.53 to 172.91) |
| Prevalence | Male | China | 96.92<br>(85.25 to 108.92) | 226.64<br>(201.35 to 253.49) | 226.64<br>(201.35 to 253.49) | 0<br>(0 to 0) | 213.98<br>(189.08 to 240.05) |
| Prevalence | Female | China | 189.53<br>(166.94 to 213.52) | 327.25<br>(287.41 to 368.24) | 327.25<br>(287.41 to 368.24) | 0<br>(0 to 0) | 285.8<br>(249.35 to 323.09) |
| Prevalence | Both | China | 139.82<br>(124.07 to 155.88) | 156.91<br>(137.83 to 176.93) | 156.91<br>(137.83 to 176.93) | 0<br>(0 to 0) | 151.72<br>(132.53 to 172.91) |
| Prevalence | Male | Colombia | 89.17<br>(78.54 to 100.33) | 0<br>(0 to 0) | 0<br>(0 to 0) | 442.39<br>(393.74 to 493.07) | 323.14<br>(285.15 to 363.7) |
| Prevalence | Female | Colombia | 170.51<br>(150.08 to 191.81) | 399.84<br>(351.57 to 450.49) | 399.84<br>(351.57 to 450.49) | 612.22<br>(539.97 to 690.04) | 479.89<br>(421.83 to 544.47) |
| Prevalence | Both | Colombia | 128.91<br>(114.41 to 144.12) | 0<br>(0 to 0) | 0<br>(0 to 0) | 288.36<br>(253.38 to 324.29) | 152.59<br>(133.17 to 173.27) |
| Prevalence | Male | Commonwealth of the Bahamas | 0<br>(0 to 0) | 261.69<br>(232.38 to 293.05) | 261.69<br>(232.38 to 293.05) | 296.89<br>(264.09 to 331.51) | 256.4<br>(226.73 to 287.37) |
| Prevalence | Female | Commonwealth of the Bahamas | 0<br>(0 to 0) | 394.99<br>(344.05 to 448.4) | 394.99<br>(344.05 to 448.4) | 394.39<br>(347.4 to 444.33) | 342.39<br>(300.4 to 387) |

| | | | | | | | |
|---|---|---|---|---|---|---|---|
| Prevalence | Both | Commonwealth of the Bahamas | 0<br>(0 to 0) | 176.36<br>(155.07 to 199.14) | 176.36<br>(155.07 to 199.14) | 205.86<br>(181.89 to 232.35) | 171.22<br>(149.51 to 193.32) |
| Prevalence | Male | Comoros | 91.6<br>(81.03 to 103.15) | 232.55<br>(205.93 to 261.48) | 232.55<br>(205.93 to 261.48) | 344.92<br>(306.72 to 385.62) | 236.07<br>(207.27 to 267.43) |
| Prevalence | Female | Comoros | 157.76<br>(138.72 to 177.99) | 318.1<br>(279.8 to 359.72) | 318.1<br>(279.8 to 359.72) | 449.82<br>(394.99 to 506.15) | 303.52<br>(264.36 to 346.74) |
| Prevalence | Both | Comoros | 124.37<br>(111.02 to 138.91) | 155.34<br>(135.55 to 175.85) | 155.34<br>(135.55 to 175.85) | 249.31<br>(219.62 to 280.59) | 158.65<br>(139.41 to 180.49) |
| Prevalence | Male | Congo | 88.23<br>(77.51 to 99.4) | 224.63<br>(199.39 to 251.19) | 224.63<br>(199.39 to 251.19) | 0<br>(0 to 0) | 219.71<br>(194.07 to 247.98) |
| Prevalence | Female | Congo | 160.01<br>(141.54 to 178.93) | 325.9<br>(285.78 to 371.71) | 325.9<br>(285.78 to 371.71) | 0<br>(0 to 0) | 283.72<br>(247.73 to 323.19) |
| Prevalence | Both | Congo | 124.51<br>(110.78 to 138.07) | 158.77<br>(139.9 to 178.86) | 158.77<br>(139.9 to 178.86) | 0<br>(0 to 0) | 152.91<br>(133.23 to 174.26) |
| Prevalence | Male | Cook Islands | 108.19<br>(95.55 to 121.83) | 226.17<br>(200.96 to 252.97) | 226.17<br>(200.96 to 252.97) | 0<br>(0 to 0) | 215.5<br>(190.45 to 241.79) |
| Prevalence | Female | Cook Islands | 192.69<br>(168.27 to 216.69) | 327.25<br>(287.41 to 368.24) | 327.25<br>(287.41 to 368.24) | 0<br>(0 to 0) | 285.8<br>(249.35 to 323.09) |
| Prevalence | Both | Cook Islands | 151.13<br>(134.27 to 169.18) | 156.91<br>(137.83 to 176.93) | 156.91<br>(137.83 to 176.93) | 0<br>(0 to 0) | 151.72<br>(132.53 to 172.91) |
| Prevalence | Male | Costa Rica | 89.17<br>(78.54 to 100.33) | 313.8<br>(276.07 to 351.79) | 313.8<br>(276.07 to 351.79) | 298.65<br>(265.58 to 333.58) | 268.45<br>(236.2 to 300.98) |
| Prevalence | Female | Costa Rica | 170.51<br>(150.08 to 191.81) | 354.94<br>(311.47 to 400.07) | 354.94<br>(311.47 to 400.07) | 394.39<br>(347.4 to 444.33) | 345.82<br>(302.1 to 391.48) |
| Prevalence | Both | Costa Rica | 129.62<br>(115.04 to 144.93) | 221.87<br>(195.92 to 251.76) | 221.87<br>(195.92 to 251.76) | 205.86<br>(181.89 to 232.35) | 192.03<br>(168.22 to 217.97) |
| Prevalence | Male | Croatia | 89.17<br>(78.54 to 100.33) | 240.09<br>(212.92 to 268.77) | 240.09<br>(212.92 to 268.77) | 0<br>(0 to 0) | 235.36<br>(206.67 to 266.61) |
| Prevalence | Female | Croatia | 170.51<br>(150.08 to 191.81) | 349.03<br>(307.48 to 400.67) | 349.03<br>(307.48 to 400.67) | 0<br>(0 to 0) | 303.52<br>(264.36 to 346.74) |
| Prevalence | Both | Croatia | 128.73<br>(114.25 to 143.91) | 162.86<br>(143.47 to 184.17) | 162.86<br>(143.47 to 184.17) | 0<br>(0 to 0) | 158.65<br>(139.41 to 180.49) |
| Prevalence | Male | Cuba | 89.17 | 0 | 0 | 0 | 213.49 |

| | | | | | | | |
|---|---|---|---|---|---|---|---|
| | | | (78.54 to 100.33) | (0 to 0) | (0 to 0) | (0 to 0) | (188.69 to 239.6) |
| Prevalence | Female | Cuba | 170.51<br>(150.08 to 191.81) | 297.31<br>(261.55 to 335.2) | 297.31<br>(261.55 to 335.2) | 0<br>(0 to 0) | 285.8<br>(249.35 to 323.09) |
| Prevalence | Both | Cuba | 128.52<br>(114.06 to 143.66) | 0<br>(0 to 0) | 0<br>(0 to 0) | 0<br>(0 to 0) | 151.72<br>(132.53 to 172.91) |
| Prevalence | Male | Cyprus | 151.04<br>(133.89 to 169.5) | 0<br>(0 to 0) | 0<br>(0 to 0) | 375.06<br>(333.53 to 418.12) | 232.27<br>(204.07 to 263.04) |
| Prevalence | Female | Cyprus | 166.54<br>(146.31 to 187.58) | 316.6<br>(278.53 to 358.19) | 316.6<br>(278.53 to 358.19) | 403.71<br>(355.63 to 454.2) | 303.52<br>(264.36 to 346.74) |
| Prevalence | Both | Cyprus | 158.53<br>(141.3 to 176.24) | 0<br>(0 to 0) | 0<br>(0 to 0) | 344.97<br>(304.18 to 389.49) | 158.65<br>(139.41 to 180.49) |
| Prevalence | Male | Czech Republic | 0<br>(0 to 0) | 0<br>(0 to 0) | 0<br>(0 to 0) | 0<br>(0 to 0) | 232.87<br>(204.57 to 263.73) |
| Prevalence | Female | Czech Republic | 0<br>(0 to 0) | 316.6<br>(278.53 to 358.19) | 316.6<br>(278.53 to 358.19) | 0<br>(0 to 0) | 303.52<br>(264.36 to 346.74) |
| Prevalence | Both | Czech Republic | 0<br>(0 to 0) | 0<br>(0 to 0) | 0<br>(0 to 0) | 0<br>(0 to 0) | 158.65<br>(139.41 to 180.49) |
| Prevalence | Male | Democratic People's Republic of Korea | 96.66<br>(84.69 to 109.13) | 337.84<br>(298.75 to 377.85) | 337.84<br>(298.75 to 377.85) | 0<br>(0 to 0) | 296.11<br>(261.54 to 332.84) |
| Prevalence | Female | Democratic People's Republic of Korea | 188.5<br>(166.43 to 211.17) | 337.26<br>(296.8 to 381.08) | 337.26<br>(296.8 to 381.08) | 0<br>(0 to 0) | 418.49<br>(366.83 to 476.04) |
| Prevalence | Both | Democratic People's Republic of Korea | 141.66<br>(126.21 to 157.62) | 229.91<br>(202.25 to 262.56) | 229.91<br>(202.25 to 262.56) | 0<br>(0 to 0) | 163.59<br>(142.91 to 185.21) |
| Prevalence | Male | Democratic Republic of the Congo | 88.23<br>(77.51 to 99.4) | 0<br>(0 to 0) | 0<br>(0 to 0) | 0<br>(0 to 0) | 230.17<br>(202.23 to 260.59) |
| Prevalence | Female | Democratic Republic of the Congo | 160.01<br>(141.54 to 178.93) | 316.6<br>(278.53 to 358.19) | 316.6<br>(278.53 to 358.19) | 0<br>(0 to 0) | 303.52<br>(264.36 to 346.74) |
| Prevalence | Both | Democratic Republic of the Congo | 123.91<br>(110.22 to 137.44) | 0<br>(0 to 0) | 0<br>(0 to 0) | 0<br>(0 to 0) | 158.65<br>(139.41 to 180.49) |
| Prevalence | Male | Denmark | 151.04<br>(133.89 to 169.5) | 0<br>(0 to 0) | 0<br>(0 to 0) | 0<br>(0 to 0) | 256.99<br>(226.91 to 289.22) |
| Prevalence | Female | Denmark | 166.54<br>(146.31 to 187.58) | 354.98<br>(312.5 to 400.45) | 354.98<br>(312.5 to 400.45) | 0<br>(0 to 0) | 340.89<br>(298.01 to 386.04) |

| | | | | | | | |
|---|---|---|---|---|---|---|---|
| Prevalence | Both | Denmark | 158.6 (141.38 to 176.34) | 0 (0 to 0) | 0 (0 to 0) | 0 (0 to 0) | 170.3 (149 to 193.18) |
| Prevalence | Male | Djibouti | 91.6 (81.03 to 103.15) | 228.04 (202.39 to 255.11) | 228.04 (202.39 to 255.11) | 301.58 (268.06 to 336.87) | 219.51 (194.04 to 246.35) |
| Prevalence | Female | Djibouti | 157.76 (138.72 to 177.99) | 0 (0 to 0) | 0 (0 to 0) | 394.39 (347.4 to 444.33) | 285.8 (249.35 to 323.09) |
| Prevalence | Both | Djibouti | 120.74 (107.85 to 134.81) | 159.07 (139.67 to 179.42) | 159.07 (139.67 to 179.42) | 205.86 (181.89 to 232.35) | 151.72 (132.53 to 172.91) |
| Prevalence | Male | Dominica | 89.17 (78.54 to 100.33) | 329.01 (291.1 to 368.5) | 329.01 (291.1 to 368.5) | 390.95 (347.76 to 435.3) | 290.42 (256.65 to 326.66) |
| Prevalence | Female | Dominica | 170.51 (150.08 to 191.81) | 337.26 (296.8 to 381.08) | 337.26 (296.8 to 381.08) | 461.29 (405.14 to 520.16) | 418.49 (366.83 to 476.04) |
| Prevalence | Both | Dominica | 128.68 (114.2 to 143.85) | 229.91 (202.25 to 262.56) | 229.91 (202.25 to 262.56) | 322.79 (284.02 to 362.41) | 163.59 (142.91 to 185.21) |
| Prevalence | Male | Dominican Republic | 89.17 (78.54 to 100.33) | 222.68 (196.5 to 251.22) | 222.68 (196.5 to 251.22) | 0 (0 to 0) | 209.16 (184.78 to 236.24) |
| Prevalence | Female | Dominican Republic | 170.51 (150.08 to 191.81) | 292.84 (257.37 to 330.41) | 292.84 (257.37 to 330.41) | 0 (0 to 0) | 283.72 (247.73 to 323.19) |
| Prevalence | Both | Dominican Republic | 129.45 (114.89 to 144.74) | 161.89 (141.3 to 184.69) | 161.89 (141.3 to 184.69) | 0 (0 to 0) | 152.91 (133.23 to 174.26) |
| Prevalence | Male | Ecuador | 89.17 (78.54 to 100.33) | 235.29 (208.78 to 263.31) | 235.29 (208.78 to 263.31) | 0 (0 to 0) | 227.2 (199.65 to 257.06) |
| Prevalence | Female | Ecuador | 170.51 (150.08 to 191.81) | 349.03 (307.48 to 400.67) | 349.03 (307.48 to 400.67) | 0 (0 to 0) | 303.52 (264.36 to 346.74) |
| Prevalence | Both | Ecuador | 129.18 (114.65 to 144.43) | 162.86 (143.47 to 184.17) | 162.86 (143.47 to 184.17) | 0 (0 to 0) | 158.65 (139.41 to 180.49) |
| Prevalence | Male | Egypt | 86.12 (75 to 97.97) | 236.84 (210.17 to 265.07) | 236.84 (210.17 to 265.07) | 0 (0 to 0) | 231.42 (203.33 to 262.08) |
| Prevalence | Female | Egypt | 152.29 (133.8 to 170.57) | 349.03 (307.48 to 400.67) | 349.03 (307.48 to 400.67) | 0 (0 to 0) | 303.52 (264.36 to 346.74) |
| Prevalence | Both | Egypt | 117.83 (104.49 to 131.54) | 162.86 (143.47 to 184.17) | 162.86 (143.47 to 184.17) | 0 (0 to 0) | 158.65 (139.41 to 180.49) |
| Prevalence | Male | El Salvador | 89.17 | 238.1 | 238.1 | 0 | 232.97 |

| | | | | | | | |
|---|---|---|---|---|---|---|---|
| | | | (78.54 to 100.33) | (211.2 to 266.54) | (211.2 to 266.54) | (0 to 0) | (204.65 to 263.85) |
| Prevalence | Female | El Salvador | 170.51<br>(150.08 to 191.81) | 349.03<br>(307.48 to 400.67) | 349.03<br>(307.48 to 400.67) | 0<br>(0 to 0) | 303.52<br>(264.36 to 346.74) |
| Prevalence | Both | El Salvador | 129.27<br>(114.73 to 144.53) | 162.86<br>(143.47 to 184.17) | 162.86<br>(143.47 to 184.17) | 0<br>(0 to 0) | 158.65<br>(139.41 to 180.49) |
| Prevalence | Male | Equatorial Guinea | 88.23<br>(77.51 to 99.4) | 211.43<br>(187.03 to 236.98) | 211.43<br>(187.03 to 236.98) | 0<br>(0 to 0) | 204.64<br>(178.96 to 230.69) |
| Prevalence | Female | Equatorial Guinea | 160.01<br>(141.54 to 178.93) | 311.39<br>(273.7 to 351.19) | 311.39<br>(273.7 to 351.19) | 0<br>(0 to 0) | 270.89<br>(236.53 to 307.89) |
| Prevalence | Both | Equatorial Guinea | 118.1<br>(105.15 to 130.96) | 148.98<br>(129.25 to 170.46) | 148.98<br>(129.25 to 170.46) | 0<br>(0 to 0) | 143.51<br>(123.79 to 165.99) |
| Prevalence | Male | Eritrea | 91.6<br>(81.03 to 103.15) | 0<br>(0 to 0) | 0<br>(0 to 0) | 0<br>(0 to 0) | 242.84<br>(213.3 to 275.36) |
| Prevalence | Female | Eritrea | 157.76<br>(138.72 to 177.99) | 316.6<br>(278.53 to 358.19) | 316.6<br>(278.53 to 358.19) | 0<br>(0 to 0) | 303.52<br>(264.36 to 346.74) |
| Prevalence | Both | Eritrea | 124.07<br>(110.75 to 138.58) | 0<br>(0 to 0) | 0<br>(0 to 0) | 0<br>(0 to 0) | 158.65<br>(139.41 to 180.49) |
| Prevalence | Male | Estonia | 89.17<br>(78.54 to 100.33) | 246.35<br>(216.8 to 276.96) | 246.35<br>(216.8 to 276.96) | 0<br>(0 to 0) | 228.36<br>(201.52 to 256.63) |
| Prevalence | Female | Estonia | 170.51<br>(150.08 to 191.81) | 293.23<br>(258.57 to 330.29) | 293.23<br>(258.57 to 330.29) | 0<br>(0 to 0) | 285.8<br>(249.35 to 323.09) |
| Prevalence | Both | Estonia | 128.75<br>(114.26 to 143.93) | 165.62<br>(144.72 to 187.82) | 165.62<br>(144.72 to 187.82) | 0<br>(0 to 0) | 151.72<br>(132.53 to 172.91) |
| Prevalence | Male | Ethiopia | 91.9<br>(81.37 to 102.87) | 229.04<br>(203.31 to 256.2) | 229.04<br>(203.31 to 256.2) | 0<br>(0 to 0) | 221.13<br>(195.28 to 249.39) |
| Prevalence | Female | Ethiopia | 158.71<br>(140.07 to 178.02) | 279.8<br>(244.28 to 317.46) | 279.8<br>(244.28 to 317.46) | 0<br>(0 to 0) | 283.72<br>(247.73 to 323.19) |
| Prevalence | Both | Ethiopia | 124.62<br>(110.97 to 138.9) | 162.91<br>(143.46 to 183.66) | 162.91<br>(143.46 to 183.66) | 0<br>(0 to 0) | 152.91<br>(133.23 to 174.26) |
| Prevalence | Male | Federated States of Micronesia | 0<br>(0 to 0) | 0<br>(0 to 0) | 0<br>(0 to 0) | 0<br>(0 to 0) | 234.2<br>(205.71 to 265.29) |
| Prevalence | Female | Federated States of Micronesia | 0<br>(0 to 0) | 316.6<br>(278.53 to 358.19) | 316.6<br>(278.53 to 358.19) | 0<br>(0 to 0) | 303.52<br>(264.36 to 346.74) |

| | | | | | | | |
|---|---|---|---|---|---|---|---|
| Prevalence | Both | Federated States of Micronesia | 0<br>(0 to 0) | 0<br>(0 to 0) | 0<br>(0 to 0) | 0<br>(0 to 0) | 158.65<br>(139.41 to 180.49) |
| Prevalence | Male | Fiji | 108.19<br>(95.55 to 121.83) | 292.77<br>(259.95 to 327.8) | 292.77<br>(259.95 to 327.8) | 0<br>(0 to 0) | 225.71<br>(199.27 to 253.5) |
| Prevalence | Female | Fiji | 192.69<br>(168.27 to 216.69) | 191.69<br>(168.88 to 215.39) | 191.69<br>(168.88 to 215.39) | 0<br>(0 to 0) | 285.8<br>(249.35 to 323.09) |
| Prevalence | Both | Fiji | 149.45<br>(132.86 to 167.28) | 199.72<br>(175.38 to 225.23) | 199.72<br>(175.38 to 225.23) | 0<br>(0 to 0) | 151.72<br>(132.53 to 172.91) |
| Prevalence | Male | Finland | 151.04<br>(133.89 to 169.5) | 0<br>(0 to 0) | 0<br>(0 to 0) | 0<br>(0 to 0) | 217.73<br>(192.49 to 244.81) |
| Prevalence | Female | Finland | 166.54<br>(146.31 to 187.58) | 296.32<br>(260.87 to 334.34) | 296.32<br>(260.87 to 334.34) | 0<br>(0 to 0) | 285.37<br>(250.14 to 322.6) |
| Prevalence | Both | Finland | 158.6<br>(141.39 to 176.35) | 0<br>(0 to 0) | 0<br>(0 to 0) | 0<br>(0 to 0) | 153.31<br>(133.62 to 174.31) |
| Prevalence | Male | France | 151.04<br>(133.89 to 169.5) | 274.63<br>(243.85 to 307.41) | 274.63<br>(243.85 to 307.41) | 0<br>(0 to 0) | 267.91<br>(235.67 to 300.2) |
| Prevalence | Female | France | 166.54<br>(146.31 to 187.58) | 399.88<br>(349.06 to 449.99) | 399.88<br>(349.06 to 449.99) | 0<br>(0 to 0) | 345.82<br>(302.1 to 391.48) |
| Prevalence | Both | France | 158.6<br>(141.39 to 176.35) | 197.08<br>(173.41 to 222.18) | 197.08<br>(173.41 to 222.18) | 0<br>(0 to 0) | 192.03<br>(168.22 to 217.97) |
| Prevalence | Male | Gabon | 88.23<br>(77.51 to 99.4) | 0<br>(0 to 0) | 0<br>(0 to 0) | 0<br>(0 to 0) | 290.95<br>(257.11 to 327.26) |
| Prevalence | Female | Gabon | 160.01<br>(141.54 to 178.93) | 348.54<br>(306.54 to 393.51) | 348.54<br>(306.54 to 393.51) | 0<br>(0 to 0) | 418.49<br>(366.83 to 476.04) |
| Prevalence | Both | Gabon | 125.54<br>(111.73 to 139.19) | 0<br>(0 to 0) | 0<br>(0 to 0) | 0<br>(0 to 0) | 163.59<br>(142.91 to 185.21) |
| Prevalence | Male | Georgia | 89.17<br>(78.54 to 100.33) | 297.55<br>(264.28 to 332.63) | 297.55<br>(264.28 to 332.63) | 0<br>(0 to 0) | 293.73<br>(259.49 to 330.35) |
| Prevalence | Female | Georgia | 170.51<br>(150.08 to 191.81) | 429.02<br>(376.04 to 482.2) | 429.02<br>(376.04 to 482.2) | 0<br>(0 to 0) | 418.49<br>(366.83 to 476.04) |
| Prevalence | Both | Georgia | 127.2<br>(112.89 to 142.14) | 255.4<br>(225.02 to 288.39) | 255.4<br>(225.02 to 288.39) | 0<br>(0 to 0) | 163.59<br>(142.91 to 185.21) |
| Prevalence | Male | Germany | 151.04 | 211.95 | 211.95 | 0 | 218.73 |

| | | | | | | | |
|---|---|---|---|---|---|---|---|
| | | | (133.89 to 169.5) | (186.68 to 238.52) | (186.68 to 238.52) | (0 to 0) | (193.39 to 245.47) |
| Prevalence | Female | Germany | 166.54<br>(146.31 to 187.58) | 300.05<br>(264.25 to 338.06) | 300.05<br>(264.25 to 338.06) | 0<br>(0 to 0) | 285.8<br>(249.35 to 323.09) |
| Prevalence | Both | Germany | 158.45<br>(141.17 to 176.17) | 148.97<br>(130.3 to 168.69) | 148.97<br>(130.3 to 168.69) | 0<br>(0 to 0) | 151.72<br>(132.53 to 172.91) |
| Prevalence | Male | Ghana | 88.23<br>(77.51 to 99.4) | 250.36<br>(220.67 to 280.79) | 250.36<br>(220.67 to 280.79) | 0<br>(0 to 0) | 216.24<br>(191.17 to 242.61) |
| Prevalence | Female | Ghana | 160.01<br>(141.54 to 178.93) | 293.11<br>(257.92 to 330.57) | 293.11<br>(257.92 to 330.57) | 0<br>(0 to 0) | 285.8<br>(249.35 to 323.09) |
| Prevalence | Both | Ghana | 123.78<br>(110.11 to 137.3) | 174.5<br>(152.75 to 197.18) | 174.5<br>(152.75 to 197.18) | 0<br>(0 to 0) | 151.72<br>(132.53 to 172.91) |
| Prevalence | Male | Greece | 151.04<br>(133.89 to 169.5) | 237.72<br>(210.85 to 266.19) | 237.72<br>(210.85 to 266.19) | 373.16<br>(331.73 to 417.14) | 235.96<br>(207.2 to 267.33) |
| Prevalence | Female | Greece | 166.54<br>(146.31 to 187.58) | 349.03<br>(307.48 to 400.67) | 349.03<br>(307.48 to 400.67) | 449.82<br>(394.99 to 506.15) | 303.52<br>(264.36 to 346.74) |
| Prevalence | Both | Greece | 158.62<br>(141.42 to 176.38) | 162.86<br>(143.47 to 184.17) | 162.86<br>(143.47 to 184.17) | 297.56<br>(261.6 to 335.42) | 158.65<br>(139.41 to 180.49) |
| Prevalence | Male | Greenland | 127<br>(112.11 to 143.18) | 296.54<br>(263.35 to 331.51) | 296.54<br>(263.35 to 331.51) | 0<br>(0 to 0) | 290.29<br>(256.54 to 326.53) |
| Prevalence | Female | Greenland | 250.87<br>(221.99 to 281.11) | 429.02<br>(376.04 to 482.2) | 429.02<br>(376.04 to 482.2) | 0<br>(0 to 0) | 418.49<br>(366.83 to 476.04) |
| Prevalence | Both | Greenland | 188.56<br>(168.64 to 210.41) | 255.4<br>(225.02 to 288.39) | 255.4<br>(225.02 to 288.39) | 0<br>(0 to 0) | 163.59<br>(142.91 to 185.21) |
| Prevalence | Male | Grenada | 89.17<br>(78.54 to 100.33) | 228.16<br>(202.96 to 254.91) | 228.16<br>(202.96 to 254.91) | 369.73<br>(328.86 to 413.23) | 224.78<br>(198.45 to 252.39) |
| Prevalence | Female | Grenada | 170.51<br>(150.08 to 191.81) | 309.77<br>(270.66 to 350.22) | 309.77<br>(270.66 to 350.22) | 449.82<br>(394.99 to 506.15) | 285.8<br>(249.35 to 323.09) |
| Prevalence | Both | Grenada | 127.89<br>(113.5 to 142.93) | 156.93<br>(138.01 to 176.68) | 156.93<br>(138.01 to 176.68) | 297.56<br>(261.6 to 335.42) | 151.72<br>(132.53 to 172.91) |
| Prevalence | Male | Guam | 108.19<br>(95.55 to 121.83) | 297.53<br>(264.26 to 332.61) | 297.53<br>(264.26 to 332.61) | 300.42<br>(267.08 to 335.62) | 295.33<br>(260.91 to 332.13) |
| Prevalence | Female | Guam | 192.69<br>(168.27 to 216.69) | 429.02<br>(376.04 to 482.2) | 429.02<br>(376.04 to 482.2) | 394.39<br>(347.4 to 444.33) | 418.49<br>(366.83 to 476.04) |

| | | | | | | | |
|---|---|---|---|---|---|---|---|
| Prevalence | Both | Guam | 148.88<br>(132.36 to 166.62) | 255.4<br>(225.02 to 288.39) | 255.4<br>(225.02 to 288.39) | 205.86<br>(181.89 to 232.35) | 163.59<br>(142.91 to 185.21) |
| Prevalence | Male | Guatemala | 89.17<br>(78.54 to 100.33) | 353.25<br>(314.03 to 394.3) | 353.25<br>(314.03 to 394.3) | 298.82<br>(265.7 to 333.82) | 371.11<br>(325.77 to 416.51) |
| Prevalence | Female | Guatemala | 170.51<br>(150.08 to 191.81) | 677.92<br>(596.01 to 769.84) | 677.92<br>(596.01 to 769.84) | 394.39<br>(347.4 to 444.33) | 654.16<br>(570.44 to 740.12) |
| Prevalence | Both | Guatemala | 129.06<br>(114.54 to 144.29) | 223.69<br>(196.65 to 251.87) | 223.69<br>(196.65 to 251.87) | 205.86<br>(181.89 to 232.35) | 130.78<br>(113.79 to 148.56) |
| Prevalence | Male | Guinea | 88.23<br>(77.51 to 99.4) | 278.54<br>(247.36 to 311.81) | 278.54<br>(247.36 to 311.81) | 397.57<br>(354.16 to 442.81) | 228.06<br>(200.41 to 258.04) |
| Prevalence | Female | Guinea | 160.01<br>(141.54 to 178.93) | 112.23<br>(98.58 to 127.84) | 112.23<br>(98.58 to 127.84) | 466.1<br>(410.98 to 523.46) | 303.52<br>(264.36 to 346.74) |
| Prevalence | Both | Guinea | 124.75<br>(111.02 to 138.34) | 193.1<br>(170.37 to 218.22) | 193.1<br>(170.37 to 218.22) | 322.79<br>(284.02 to 362.41) | 158.65<br>(139.41 to 180.49) |
| Prevalence | Male | Guinea-Bissau | 88.23<br>(77.51 to 99.4) | 0<br>(0 to 0) | 0<br>(0 to 0) | 296.5<br>(263.77 to 331.04) | 264.06<br>(232.64 to 295.87) |
| Prevalence | Female | Guinea-Bissau | 160.01<br>(141.54 to 178.93) | 360.47<br>(316.1 to 406.21) | 360.47<br>(316.1 to 406.21) | 394.39<br>(347.4 to 444.33) | 345.82<br>(302.1 to 391.48) |
| Prevalence | Both | Guinea-Bissau | 124.33<br>(110.61 to 137.88) | 0<br>(0 to 0) | 0<br>(0 to 0) | 205.86<br>(181.89 to 232.35) | 192.03<br>(168.22 to 217.97) |
| Prevalence | Male | Guyana | 89.17<br>(78.54 to 100.33) | 241.26<br>(213.9 to 270.15) | 241.26<br>(213.9 to 270.15) | 343.1<br>(304.92 to 383.22) | 239.1<br>(209.94 to 271) |
| Prevalence | Female | Guyana | 170.51<br>(150.08 to 191.81) | 349.03<br>(307.48 to 400.67) | 349.03<br>(307.48 to 400.67) | 449.82<br>(394.99 to 506.15) | 303.52<br>(264.36 to 346.74) |
| Prevalence | Both | Guyana | 129.23<br>(114.7 to 144.5) | 162.86<br>(143.47 to 184.17) | 162.86<br>(143.47 to 184.17) | 249.31<br>(219.62 to 280.59) | 158.65<br>(139.41 to 180.49) |
| Prevalence | Male | Haiti | 89.17<br>(78.54 to 100.33) | 232.73<br>(206.45 to 260.51) | 232.73<br>(206.45 to 260.51) | 267.31<br>(237.92 to 297.94) | 218.61<br>(193.29 to 245.33) |
| Prevalence | Female | Haiti | 170.51<br>(150.08 to 191.81) | 0<br>(0 to 0) | 0<br>(0 to 0) | 351.28<br>(309.28 to 395.11) | 285.8<br>(249.35 to 323.09) |
| Prevalence | Both | Haiti | 129.9<br>(115.31 to 145.27) | 159.07<br>(139.67 to 179.42) | 159.07<br>(139.67 to 179.42) | 185.99<br>(163.53 to 209.66) | 151.72<br>(132.53 to 172.91) |
| Prevalence | Male | Honduras | 89.17 | 267.8 | 267.8 | 0 | 222.1 |

| | | | | | | | |
|---|---|---|---|---|---|---|---|
| | | | (78.54 to 100.33) | (236.17 to 301.23) | (236.17 to 301.23) | (0 to 0) | (196.32 to 249.3) |
| Prevalence | Female | Honduras | 170.51<br>(150.08 to 191.81) | 275.76<br>(243.5 to 309.84) | 275.76<br>(243.5 to 309.84) | 0<br>(0 to 0) | 285.8<br>(249.35 to 323.09) |
| Prevalence | Both | Honduras | 129.6<br>(115.02 to 144.91) | 182.88<br>(159.88 to 207.27) | 182.88<br>(159.88 to 207.27) | 0<br>(0 to 0) | 151.72<br>(132.53 to 172.91) |
| Prevalence | Male | Hungary | 89.17<br>(78.54 to 100.33) | 238.69<br>(211.7 to 267.18) | 238.69<br>(211.7 to 267.18) | 0<br>(0 to 0) | 232.2<br>(204.01 to 262.96) |
| Prevalence | Female | Hungary | 170.51<br>(150.08 to 191.81) | 349.03<br>(307.48 to 400.67) | 349.03<br>(307.48 to 400.67) | 0<br>(0 to 0) | 303.52<br>(264.36 to 346.74) |
| Prevalence | Both | Hungary | 128.7<br>(114.22 to 143.87) | 162.86<br>(143.47 to 184.17) | 162.86<br>(143.47 to 184.17) | 0<br>(0 to 0) | 158.65<br>(139.41 to 180.49) |
| Prevalence | Male | Iceland | 151.04<br>(133.89 to 169.5) | 0<br>(0 to 0) | 0<br>(0 to 0) | 0<br>(0 to 0) | 234.77<br>(206.18 to 265.94) |
| Prevalence | Female | Iceland | 166.54<br>(146.31 to 187.58) | 316.6<br>(278.53 to 358.19) | 316.6<br>(278.53 to 358.19) | 0<br>(0 to 0) | 303.52<br>(264.36 to 346.74) |
| Prevalence | Both | Iceland | 158.68<br>(141.45 to 176.42) | 0<br>(0 to 0) | 0<br>(0 to 0) | 0<br>(0 to 0) | 158.65<br>(139.41 to 180.49) |
| Prevalence | Male | India | 80.54<br>(70.9 to 91.06) | 317.51<br>(281.35 to 355.57) | 317.51<br>(281.35 to 355.57) | 0<br>(0 to 0) | 234.9<br>(206.28 to 266.08) |
| Prevalence | Female | India | 151.33<br>(133.19 to 170.08) | 228.46<br>(201.52 to 259.1) | 228.46<br>(201.52 to 259.1) | 0<br>(0 to 0) | 303.52<br>(264.36 to 346.74) |
| Prevalence | Both | India | 114.35<br>(101.81 to 128.31) | 213.27<br>(187.99 to 241.21) | 213.27<br>(187.99 to 241.21) | 0<br>(0 to 0) | 158.65<br>(139.41 to 180.49) |
| Prevalence | Male | Indonesia | 129.56<br>(113.99 to 146.06) | 303.75<br>(269.44 to 340.07) | 303.75<br>(269.44 to 340.07) | 0<br>(0 to 0) | 233.1<br>(204.78 to 264) |
| Prevalence | Female | Indonesia | 193.98<br>(170.95 to 218.3) | 204.41<br>(180.13 to 232.67) | 204.41<br>(180.13 to 232.67) | 0<br>(0 to 0) | 303.52<br>(264.36 to 346.74) |
| Prevalence | Both | Indonesia | 161.05<br>(143.53 to 180.09) | 208.21<br>(183.45 to 235.48) | 208.21<br>(183.45 to 235.48) | 0<br>(0 to 0) | 158.65<br>(139.41 to 180.49) |
| Prevalence | Male | Iraq | 94.3<br>(83.03 to 105.44) | 295.94<br>(262.87 to 330.81) | 295.94<br>(262.87 to 330.81) | 0<br>(0 to 0) | 291.29<br>(257.37 to 327.58) |
| Prevalence | Female | Iraq | 152.29<br>(133.8 to 170.57) | 429.02<br>(376.04 to 482.2) | 429.02<br>(376.04 to 482.2) | 0<br>(0 to 0) | 418.49<br>(366.83 to 476.04) |

| | | | | | | | |
|---|---|---|---|---|---|---|---|
| Prevalence | Both | Iraq | 122.52<br>(109.14 to 136.8) | 255.4<br>(225.02 to 288.39) | 255.4<br>(225.02 to 288.39) | 0<br>(0 to 0) | 163.59<br>(142.91 to 185.21) |
| Prevalence | Male | Ireland | 151.04<br>(133.89 to 169.5) | 213.22<br>(188.81 to 238.78) | 213.22<br>(188.81 to 238.78) | 0<br>(0 to 0) | 205.21<br>(181.01 to 230.08) |
| Prevalence | Female | Ireland | 166.54<br>(146.31 to 187.58) | 0<br>(0 to 0) | 0<br>(0 to 0) | 0<br>(0 to 0) | 268.64<br>(235.65 to 303.65) |
| Prevalence | Both | Ireland | 158.62<br>(141.41 to 176.37) | 149.15<br>(130.64 to 168.5) | 149.15<br>(130.64 to 168.5) | 0<br>(0 to 0) | 142.4<br>(124.55 to 161.31) |
| Prevalence | Male | Islamic Republic of Iran | 0<br>(0 to 0) | 286.1<br>(253.27 to 321.08) | 286.1<br>(253.27 to 321.08) | 302.93<br>(269.21 to 338.37) | 288.28<br>(254.65 to 322.93) |
| Prevalence | Female | Islamic Republic of Iran | 0<br>(0 to 0) | 366.45<br>(322.76 to 412.7) | 366.45<br>(322.76 to 412.7) | 394.39<br>(347.4 to 444.33) | 346.77<br>(302.07 to 393.39) |
| Prevalence | Both | Islamic Republic of Iran | 0<br>(0 to 0) | 227.71<br>(199.52 to 258.54) | 227.71<br>(199.52 to 258.54) | 205.86<br>(181.89 to 232.35) | 230.23<br>(200.87 to 261.2) |
| Prevalence | Male | Israel | 151.04<br>(133.89 to 169.5) | 0<br>(0 to 0) | 0<br>(0 to 0) | 0<br>(0 to 0) | 207.56<br>(183.16 to 232.93) |
| Prevalence | Female | Israel | 166.54<br>(146.31 to 187.58) | 283.9<br>(249.68 to 320.15) | 283.9<br>(249.68 to 320.15) | 0<br>(0 to 0) | 272.32<br>(238.09 to 307.56) |
| Prevalence | Both | Israel | 158.6<br>(141.38 to 176.34) | 0<br>(0 to 0) | 0<br>(0 to 0) | 0<br>(0 to 0) | 143.58<br>(125.8 to 162.86) |
| Prevalence | Male | Italy | 151.8<br>(133.88 to 170.69) | 221.02<br>(196.25 to 246.71) | 221.02<br>(196.25 to 246.71) | 0<br>(0 to 0) | 214.81<br>(189.79 to 241.1) |
| Prevalence | Female | Italy | 167.33<br>(148.44 to 188.74) | 311.39<br>(273.7 to 351.19) | 311.39<br>(273.7 to 351.19) | 0<br>(0 to 0) | 270.89<br>(236.53 to 307.89) |
| Prevalence | Both | Italy | 159.31<br>(142.51 to 177.7) | 166.73<br>(146.5 to 188.19) | 166.73<br>(146.5 to 188.19) | 0<br>(0 to 0) | 160.76<br>(140.44 to 181.96) |
| Prevalence | Male | Jamaica | 89.17<br>(78.54 to 100.33) | 330.11<br>(292.12 to 369.67) | 330.11<br>(292.12 to 369.67) | 0<br>(0 to 0) | 292.33<br>(258.28 to 328.77) |
| Prevalence | Female | Jamaica | 170.51<br>(150.08 to 191.81) | 337.26<br>(296.8 to 381.08) | 337.26<br>(296.8 to 381.08) | 0<br>(0 to 0) | 418.49<br>(366.83 to 476.04) |
| Prevalence | Both | Jamaica | 129.06<br>(114.54 to 144.29) | 229.91<br>(202.25 to 262.56) | 229.91<br>(202.25 to 262.56) | 0<br>(0 to 0) | 163.59<br>(142.91 to 185.21) |
| Prevalence | Male | Japan | 141.63 | 0 | 0 | 0 | 293.63 |

| | | | | | | | |
|---|---|---|---|---|---|---|---|
| | | | (124.79 to 158.88) | (0 to 0) | (0 to 0) | (0 to 0) | (259.42 to 330.26) |
| Prevalence | Female | Japan | 191.45<br>(170.43 to 213.95) | 348.54<br>(306.54 to 393.51) | 348.54<br>(306.54 to 393.51) | 0<br>(0 to 0) | 418.49<br>(366.83 to 476.04) |
| Prevalence | Both | Japan | 165.83<br>(148.11 to 184.44) | 0<br>(0 to 0) | 0<br>(0 to 0) | 0<br>(0 to 0) | 163.59<br>(142.91 to 185.21) |
| Prevalence | Male | Jordan | 84.13<br>(73.9 to 94.89) | 302.68<br>(269.19 to 337.87) | 302.68<br>(269.19 to 337.87) | 0<br>(0 to 0) | 295.17<br>(260.57 to 330.61) |
| Prevalence | Female | Jordan | 152.29<br>(133.8 to 170.57) | 0<br>(0 to 0) | 0<br>(0 to 0) | 0<br>(0 to 0) | 420.39<br>(367.78 to 472.41) |
| Prevalence | Both | Jordan | 116.75<br>(103.93 to 130.44) | 253.18<br>(223.41 to 284.33) | 253.18<br>(223.41 to 284.33) | 0<br>(0 to 0) | 164.4<br>(142.95 to 184.94) |
| Prevalence | Male | Kazakhstan | 89.17<br>(78.54 to 100.33) | 265.95<br>(236.28 to 300.24) | 265.95<br>(236.28 to 300.24) | 0<br>(0 to 0) | 230.15<br>(202.22 to 260.6) |
| Prevalence | Female | Kazakhstan | 170.51<br>(150.08 to 191.81) | 312.06<br>(274.47 to 352.23) | 312.06<br>(274.47 to 352.23) | 0<br>(0 to 0) | 303.52<br>(264.36 to 346.74) |
| Prevalence | Both | Kazakhstan | 129.14<br>(114.61 to 144.38) | 181.89<br>(160.92 to 205.71) | 181.89<br>(160.92 to 205.71) | 0<br>(0 to 0) | 158.65<br>(139.41 to 180.49) |
| Prevalence | Male | Kenya | 91.9<br>(81.37 to 102.87) | 237.7<br>(212.47 to 263.82) | 237.7<br>(212.47 to 263.82) | 0<br>(0 to 0) | 315.54<br>(277.71 to 355.21) |
| Prevalence | Female | Kenya | 158.71<br>(140.07 to 178.02) | 527.8<br>(460.54 to 598.04) | 527.8<br>(460.54 to 598.04) | 0<br>(0 to 0) | 478.18<br>(418.19 to 540.49) |
| Prevalence | Both | Kenya | 124.88<br>(111.2 to 139.19) | 220.53<br>(194.67 to 246.17) | 220.53<br>(194.67 to 246.17) | 0<br>(0 to 0) | 153.72<br>(134.98 to 173.56) |
| Prevalence | Male | Kingdom of Eswatini | 0<br>(0 to 0) | 0<br>(0 to 0) | 0<br>(0 to 0) | 0<br>(0 to 0) | 201.56<br>(177.79 to 226.82) |
| Prevalence | Female | Kingdom of Eswatini | 0<br>(0 to 0) | 282.14<br>(247.99 to 318.06) | 282.14<br>(247.99 to 318.06) | 0<br>(0 to 0) | 270.89<br>(236.53 to 307.89) |
| Prevalence | Both | Kingdom of Eswatini | 0<br>(0 to 0) | 0<br>(0 to 0) | 0<br>(0 to 0) | 0<br>(0 to 0) | 143.11<br>(125.25 to 162.9) |
| Prevalence | Male | Kiribati | 108.19<br>(95.55 to 121.83) | 0<br>(0 to 0) | 0<br>(0 to 0) | 0<br>(0 to 0) | 235.88<br>(207.13 to 267.24) |
| Prevalence | Female | Kiribati | 192.69<br>(168.27 to 216.69) | 316.6<br>(278.53 to 358.19) | 316.6<br>(278.53 to 358.19) | 0<br>(0 to 0) | 303.52<br>(264.36 to 346.74) |

| | | | | | | | |
|---|---|---|---|---|---|---|---|
| Prevalence | Both | Kiribati | 150.32<br>(133.59 to 168.25) | 0<br>(0 to 0) | 0<br>(0 to 0) | 0<br>(0 to 0) | 158.65<br>(139.41 to 180.49) |
| Prevalence | Male | Kuwait | 84.13<br>(73.9 to 94.89) | 0<br>(0 to 0) | 0<br>(0 to 0) | 0<br>(0 to 0) | 219<br>(193.61 to 246.21) |
| Prevalence | Female | Kuwait | 152.29<br>(133.8 to 170.57) | 296.32<br>(260.87 to 334.34) | 296.32<br>(260.87 to 334.34) | 0<br>(0 to 0) | 285.37<br>(250.14 to 322.6) |
| Prevalence | Both | Kuwait | 117.18<br>(104.31 to 130.9) | 0<br>(0 to 0) | 0<br>(0 to 0) | 0<br>(0 to 0) | 153.31<br>(133.62 to 174.31) |
| Prevalence | Male | Kyrgyzstan | 89.17<br>(78.54 to 100.33) | 85.22<br>(75.77 to 95.49) | 85.22<br>(75.77 to 95.49) | 0<br>(0 to 0) | 272.37<br>(239.52 to 305.32) |
| Prevalence | Female | Kyrgyzstan | 170.51<br>(150.08 to 191.81) | 390.36<br>(342.2 to 439.89) | 390.36<br>(342.2 to 439.89) | 0<br>(0 to 0) | 345.82<br>(302.1 to 391.48) |
| Prevalence | Both | Kyrgyzstan | 129.13<br>(114.6 to 144.36) | 61.57<br>(54.45 to 69.63) | 61.57<br>(54.45 to 69.63) | 0<br>(0 to 0) | 192.03<br>(168.22 to 217.97) |
| Prevalence | Male | Lao People's Democratic Republic | 128.96<br>(113.35 to 144.69) | 229.53<br>(203.52 to 257.09) | 229.53<br>(203.52 to 257.09) | 0<br>(0 to 0) | 196.56<br>(173.52 to 221.31) |
| Prevalence | Female | Lao People's Democratic Republic | 192.69<br>(168.27 to 216.69) | 278.04<br>(244.39 to 313.42) | 278.04<br>(244.39 to 313.42) | 0<br>(0 to 0) | 270.89<br>(236.53 to 307.89) |
| Prevalence | Both | Lao People's Democratic Republic | 160.47<br>(143.05 to 179.16) | 164.39<br>(144.23 to 185.67) | 164.39<br>(144.23 to 185.67) | 0<br>(0 to 0) | 143.11<br>(125.25 to 162.9) |
| Prevalence | Male | Latvia | 89.17<br>(78.54 to 100.33) | 238.8<br>(211.87 to 267.28) | 238.8<br>(211.87 to 267.28) | 0<br>(0 to 0) | 235.35<br>(206.68 to 266.62) |
| Prevalence | Female | Latvia | 170.51<br>(150.08 to 191.81) | 329.8<br>(290.59 to 377.47) | 329.8<br>(290.59 to 377.47) | 0<br>(0 to 0) | 303.52<br>(264.36 to 346.74) |
| Prevalence | Both | Latvia | 128.85<br>(114.35 to 144.05) | 163.56<br>(144.2 to 184.94) | 163.56<br>(144.2 to 184.94) | 0<br>(0 to 0) | 158.65<br>(139.41 to 180.49) |
| Prevalence | Male | Lebanon | 84.13<br>(73.9 to 94.89) | 0<br>(0 to 0) | 0<br>(0 to 0) | 0<br>(0 to 0) | 288.34<br>(253.74 to 323.49) |
| Prevalence | Female | Lebanon | 152.29<br>(133.8 to 170.57) | 360.47<br>(316.1 to 406.21) | 360.47<br>(316.1 to 406.21) | 0<br>(0 to 0) | 345.82<br>(302.1 to 391.48) |
| Prevalence | Both | Lebanon | 116.27<br>(103.52 to 129.96) | 0<br>(0 to 0) | 0<br>(0 to 0) | 0<br>(0 to 0) | 228.88<br>(200.81 to 259.42) |
| Prevalence | Male | Lesotho | 88.23 | 0 | 0 | 0 | 236.09 |

| | | | | | | | |
|---|---|---|---|---|---|---|---|
| | | | (77.51 to 99.4) | (0 to 0) | (0 to 0) | (0 to 0) | (207.32 to 267.49) |
| Prevalence | Female | Lesotho | 160.01 (141.54 to 178.93) | 316.6 (278.53 to 358.19) | 316.6 (278.53 to 358.19) | 0 (0 to 0) | 303.52 (264.36 to 346.74) |
| Prevalence | Both | Lesotho | 123.91 (110.21 to 137.44) | 0 (0 to 0) | 0 (0 to 0) | 0 (0 to 0) | 158.65 (139.41 to 180.49) |
| Prevalence | Male | Liberia | 88.23 (77.51 to 99.4) | 218.14 (193.72 to 243.98) | 218.14 (193.72 to 243.98) | 0 (0 to 0) | 213.06 (187.88 to 240.29) |
| Prevalence | Female | Liberia | 160.01 (141.54 to 178.93) | 0 (0 to 0) | 0 (0 to 0) | 0 (0 to 0) | 270.89 (236.53 to 307.89) |
| Prevalence | Both | Liberia | 124.04 (110.35 to 137.58) | 150.39 (132.07 to 169.81) | 150.39 (132.07 to 169.81) | 0 (0 to 0) | 143.11 (125.25 to 162.9) |
| Prevalence | Male | Libya | 84.13 (73.9 to 94.89) | 251.45 (221.67 to 281.92) | 251.45 (221.67 to 281.92) | 0 (0 to 0) | 225.71 (199.27 to 253.5) |
| Prevalence | Female | Libya | 152.29 (133.8 to 170.57) | 293.11 (257.92 to 330.57) | 293.11 (257.92 to 330.57) | 0 (0 to 0) | 285.8 (249.35 to 323.09) |
| Prevalence | Both | Libya | 117.35 (104.46 to 131.08) | 174.5 (152.75 to 197.18) | 174.5 (152.75 to 197.18) | 0 (0 to 0) | 151.72 (132.53 to 172.91) |
| Prevalence | Male | Lithuania | 89.17 (78.54 to 100.33) | 226.1 (199.28 to 253.93) | 226.1 (199.28 to 253.93) | 0 (0 to 0) | 214.24 (189.33 to 240.41) |
| Prevalence | Female | Lithuania | 170.51 (150.08 to 191.81) | 295.72 (260.75 to 333.26) | 295.72 (260.75 to 333.26) | 0 (0 to 0) | 285.8 (249.35 to 323.09) |
| Prevalence | Both | Lithuania | 128.79 (114.3 to 143.98) | 160.46 (140.36 to 181.89) | 160.46 (140.36 to 181.89) | 0 (0 to 0) | 151.72 (132.53 to 172.91) |
| Prevalence | Male | Luxembourg | 151.04 (133.89 to 169.5) | 139.35 (123.88 to 156.07) | 139.35 (123.88 to 156.07) | 0 (0 to 0) | 205.54 (181.27 to 231.21) |
| Prevalence | Female | Luxembourg | 166.54 (146.31 to 187.58) | 355.07 (312.02 to 400.55) | 355.07 (312.02 to 400.55) | 0 (0 to 0) | 270.89 (236.53 to 307.89) |
| Prevalence | Both | Luxembourg | 158.56 (141.34 to 176.27) | 99.01 (86.97 to 111.89) | 99.01 (86.97 to 111.89) | 0 (0 to 0) | 143.11 (125.25 to 162.9) |
| Prevalence | Male | Madagascar | 91.6 (81.03 to 103.15) | 109.47 (97.22 to 122.58) | 109.47 (97.22 to 122.58) | 0 (0 to 0) | 236.26 (207.46 to 267.68) |
| Prevalence | Female | Madagascar | 157.76 (138.72 to 177.99) | 390.08 (343.05 to 441.47) | 390.08 (343.05 to 441.47) | 0 (0 to 0) | 303.52 (264.36 to 346.74) |

| | | | | | | | |
|---|---|---|---|---|---|---|---|
| Prevalence | Both | Madagascar | 124.43<br>(111.07 to 138.97) | 75.61<br>(66.54 to 85.44) | 75.61<br>(66.54 to 85.44) | 0<br>(0 to 0) | 158.65<br>(139.41 to 180.49) |
| Prevalence | Male | Malawi | 91.6<br>(81.03 to 103.15) | 295.53<br>(262.51 to 330.32) | 295.53<br>(262.51 to 330.32) | 0<br>(0 to 0) | 286.09<br>(252.9 to 321.79) |
| Prevalence | Female | Malawi | 157.76<br>(138.72 to 177.99) | 429.02<br>(376.04 to 482.2) | 429.02<br>(376.04 to 482.2) | 0<br>(0 to 0) | 418.49<br>(366.83 to 476.04) |
| Prevalence | Both | Malawi | 125.17<br>(111.74 to 139.81) | 255.4<br>(225.02 to 288.39) | 255.4<br>(225.02 to 288.39) | 0<br>(0 to 0) | 163.59<br>(142.91 to 185.21) |
| Prevalence | Male | Malaysia | 128.96<br>(113.35 to 144.69) | 252.63<br>(222.47 to 285.91) | 252.63<br>(222.47 to 285.91) | 0<br>(0 to 0) | 218.67<br>(193.09 to 246.83) |
| Prevalence | Female | Malaysia | 192.69<br>(168.27 to 216.69) | 290.2<br>(254.72 to 327.79) | 290.2<br>(254.72 to 327.79) | 0<br>(0 to 0) | 283.72<br>(247.73 to 323.19) |
| Prevalence | Both | Malaysia | 159.98<br>(142.62 to 178.58) | 176.44<br>(153.51 to 202.04) | 176.44<br>(153.51 to 202.04) | 0<br>(0 to 0) | 152.91<br>(133.23 to 174.26) |
| Prevalence | Male | Maldives | 128.96<br>(113.35 to 144.69) | 191.68<br>(170.24 to 214.5) | 191.68<br>(170.24 to 214.5) | 0<br>(0 to 0) | 220.74<br>(194.95 to 249.02) |
| Prevalence | Female | Maldives | 192.69<br>(168.27 to 216.69) | 367.11<br>(321.49 to 415.62) | 367.11<br>(321.49 to 415.62) | 0<br>(0 to 0) | 283.72<br>(247.73 to 323.19) |
| Prevalence | Both | Maldives | 158.95<br>(141.71 to 177.52) | 135.45<br>(119.31 to 152.78) | 135.45<br>(119.31 to 152.78) | 0<br>(0 to 0) | 152.91<br>(133.23 to 174.26) |
| Prevalence | Male | Mali | 88.23<br>(77.51 to 99.4) | 292.57<br>(259.97 to 327.73) | 292.57<br>(259.97 to 327.73) | 0<br>(0 to 0) | 286.43<br>(252.3 to 321.28) |
| Prevalence | Female | Mali | 160.01<br>(141.54 to 178.93) | 399.88<br>(349.06 to 449.99) | 399.88<br>(349.06 to 449.99) | 0<br>(0 to 0) | 345.82<br>(302.1 to 391.48) |
| Prevalence | Both | Mali | 124.05<br>(110.36 to 137.58) | 235.24<br>(206.94 to 265.66) | 235.24<br>(206.94 to 265.66) | 0<br>(0 to 0) | 228.88<br>(200.81 to 259.42) |
| Prevalence | Male | Malta | 151.04<br>(133.89 to 169.5) | 0<br>(0 to 0) | 0<br>(0 to 0) | 0<br>(0 to 0) | 279.85<br>(246.83 to 313.73) |
| Prevalence | Female | Malta | 166.54<br>(146.31 to 187.58) | 360.47<br>(316.1 to 406.21) | 360.47<br>(316.1 to 406.21) | 0<br>(0 to 0) | 345.82<br>(302.1 to 391.48) |
| Prevalence | Both | Malta | 158.61<br>(141.39 to 176.32) | 0<br>(0 to 0) | 0<br>(0 to 0) | 0<br>(0 to 0) | 228.88<br>(200.81 to 259.42) |
| Prevalence | Male | Marshall Islands | 108.19 | 227.07 | 227.07 | 0 | 216.24 |

| | | | | | | | |
|---|---|---|---|---|---|---|---|
| | | | (95.55 to 121.83) | (201.76 to 254.01) | (201.76 to 254.01) | (0 to 0) | (191.15 to 242.61) |
| Prevalence | Female | Marshall Islands | 192.69<br>(168.27 to 216.69) | 327.25<br>(287.41 to 368.24) | 327.25<br>(287.41 to 368.24) | 0<br>(0 to 0) | 285.8<br>(249.35 to 323.09) |
| Prevalence | Both | Marshall Islands | 149.83<br>(133.18 to 167.71) | 156.91<br>(137.83 to 176.93) | 156.91<br>(137.83 to 176.93) | 0<br>(0 to 0) | 151.72<br>(132.53 to 172.91) |
| Prevalence | Male | Mauritania | 88.23<br>(77.51 to 99.4) | 0<br>(0 to 0) | 0<br>(0 to 0) | 0<br>(0 to 0) | 290.23<br>(256.48 to 326.42) |
| Prevalence | Female | Mauritania | 160.01<br>(141.54 to 178.93) | 348.54<br>(306.54 to 393.51) | 348.54<br>(306.54 to 393.51) | 0<br>(0 to 0) | 418.49<br>(366.83 to 476.04) |
| Prevalence | Both | Mauritania | 124.72<br>(110.98 to 138.3) | 0<br>(0 to 0) | 0<br>(0 to 0) | 0<br>(0 to 0) | 163.59<br>(142.91 to 185.21) |
| Prevalence | Male | Mauritius | 128.96<br>(113.35 to 144.69) | 275.98<br>(245.36 to 308.62) | 275.98<br>(245.36 to 308.62) | 0<br>(0 to 0) | 268.77<br>(236.42 to 301.26) |
| Prevalence | Female | Mauritius | 192.69<br>(168.27 to 216.69) | 368.76<br>(321.61 to 416.53) | 368.76<br>(321.61 to 416.53) | 0<br>(0 to 0) | 345.82<br>(302.1 to 391.48) |
| Prevalence | Both | Mauritius | 160.51<br>(143.09 to 179.22) | 198.62<br>(174.84 to 223.57) | 198.62<br>(174.84 to 223.57) | 0<br>(0 to 0) | 192.03<br>(168.22 to 217.97) |
| Prevalence | Male | Mexico | 89.75<br>(79.66 to 101.18) | 0<br>(0 to 0) | 0<br>(0 to 0) | 0<br>(0 to 0) | 220.17<br>(194.65 to 247.11) |
| Prevalence | Female | Mexico | 171.11<br>(151.12 to 192.76) | 297.31<br>(261.55 to 335.2) | 297.31<br>(261.55 to 335.2) | 0<br>(0 to 0) | 285.8<br>(249.35 to 323.09) |
| Prevalence | Both | Mexico | 130.04<br>(116.27 to 145.34) | 0<br>(0 to 0) | 0<br>(0 to 0) | 0<br>(0 to 0) | 151.72<br>(132.53 to 172.91) |
| Prevalence | Male | Mongolia | 89.17<br>(78.54 to 100.33) | 331.24<br>(291.92 to 368.88) | 331.24<br>(291.92 to 368.88) | 0<br>(0 to 0) | 287.92<br>(253.37 to 323.05) |
| Prevalence | Female | Mongolia | 170.51<br>(150.08 to 191.81) | 354.94<br>(311.47 to 400.07) | 354.94<br>(311.47 to 400.07) | 0<br>(0 to 0) | 345.82<br>(302.1 to 391.48) |
| Prevalence | Both | Mongolia | 129.06<br>(114.54 to 144.29) | 263.26<br>(230.43 to 296.01) | 263.26<br>(230.43 to 296.01) | 0<br>(0 to 0) | 228.88<br>(200.81 to 259.42) |
| Prevalence | Male | Montenegro | 89.17<br>(78.54 to 100.33) | 240.77<br>(213.59 to 269.55) | 240.77<br>(213.59 to 269.55) | 0<br>(0 to 0) | 236.14<br>(209.21 to 264.11) |
| Prevalence | Female | Montenegro | 170.51<br>(150.08 to 191.81) | 350.36<br>(307.59 to 398.42) | 350.36<br>(307.59 to 398.42) | 0<br>(0 to 0) | 304.86<br>(267.67 to 343.84) |

| | | | | | | | |
|---|---|---|---|---|---|---|---|
| Prevalence | Both | Montenegro | 128.21<br>(113.78 to 143.3) | 163.71<br>(144.31 to 184.46) | 163.71<br>(144.31 to 184.46) | 0<br>(0 to 0) | 159.48<br>(139.18 to 180.85) |
| Prevalence | Male | Morocco | 84.13<br>(73.9 to 94.89) | 256.71<br>(228.02 to 289.59) | 256.71<br>(228.02 to 289.59) | 0<br>(0 to 0) | 269.2<br>(236.78 to 301.81) |
| Prevalence | Female | Morocco | 152.29<br>(133.8 to 170.57) | 361.67<br>(317.16 to 407.48) | 361.67<br>(317.16 to 407.48) | 0<br>(0 to 0) | 345.82<br>(302.1 to 391.48) |
| Prevalence | Both | Morocco | 117.5<br>(104.6 to 131.25) | 181.55<br>(159.64 to 205.84) | 181.55<br>(159.64 to 205.84) | 0<br>(0 to 0) | 192.03<br>(168.22 to 217.97) |
| Prevalence | Male | Mozambique | 91.6<br>(81.03 to 103.15) | 390.75<br>(346.78 to 436.85) | 390.75<br>(346.78 to 436.85) | 0<br>(0 to 0) | 289.09<br>(255.42 to 325) |
| Prevalence | Female | Mozambique | 157.76<br>(138.72 to 177.99) | 238.34<br>(209.37 to 268.49) | 238.34<br>(209.37 to 268.49) | 0<br>(0 to 0) | 418.49<br>(366.83 to 476.04) |
| Prevalence | Both | Mozambique | 125.8<br>(112.29 to 140.46) | 314.58<br>(276.87 to 355.54) | 314.58<br>(276.87 to 355.54) | 0<br>(0 to 0) | 163.59<br>(142.91 to 185.21) |
| Prevalence | Male | Myanmar | 128.96<br>(113.35 to 144.69) | 0<br>(0 to 0) | 0<br>(0 to 0) | 0<br>(0 to 0) | 235.48<br>(206.79 to 266.77) |
| Prevalence | Female | Myanmar | 192.69<br>(168.27 to 216.69) | 316.6<br>(278.53 to 358.19) | 316.6<br>(278.53 to 358.19) | 0<br>(0 to 0) | 303.52<br>(264.36 to 346.74) |
| Prevalence | Both | Myanmar | 160.7<br>(143.25 to 179.45) | 0<br>(0 to 0) | 0<br>(0 to 0) | 0<br>(0 to 0) | 158.65<br>(139.41 to 180.49) |
| Prevalence | Male | Namibia | 88.23<br>(77.51 to 99.4) | 108.96<br>(96.77 to 121.99) | 108.96<br>(96.77 to 121.99) | 0<br>(0 to 0) | 232.09<br>(203.92 to 262.84) |
| Prevalence | Female | Namibia | 160.01<br>(141.54 to 178.93) | 390.08<br>(343.05 to 441.47) | 390.08<br>(343.05 to 441.47) | 0<br>(0 to 0) | 303.52<br>(264.36 to 346.74) |
| Prevalence | Both | Namibia | 124.43<br>(110.7 to 137.99) | 75.61<br>(66.54 to 85.44) | 75.61<br>(66.54 to 85.44) | 0<br>(0 to 0) | 158.65<br>(139.41 to 180.49) |
| Prevalence | Male | Nepal | 80.85<br>(71.48 to 90.93) | 0<br>(0 to 0) | 0<br>(0 to 0) | 0<br>(0 to 0) | 207.24<br>(182.77 to 233.18) |
| Prevalence | Female | Nepal | 150.23<br>(132.53 to 169.2) | 282.14<br>(247.99 to 318.06) | 282.14<br>(247.99 to 318.06) | 0<br>(0 to 0) | 270.89<br>(236.53 to 307.89) |
| Prevalence | Both | Nepal | 115.61<br>(102.83 to 129.12) | 0<br>(0 to 0) | 0<br>(0 to 0) | 0<br>(0 to 0) | 143.11<br>(125.25 to 162.9) |
| Prevalence | Male | Netherlands | 150.77 | 231.89 | 231.89 | 0 | 222.18 |

| | | | | | | | |
|---|---|---|---|---|---|---|---|
| | | | (132.51 to 169.65) | (205.51 to 259.85) | (205.51 to 259.85) | (0 to 0) | (196.23 to 250.63) |
| Prevalence | Female | Netherlands | 166.62<br>(146.84 to 187.97) | 0<br>(0 to 0) | 0<br>(0 to 0) | 0<br>(0 to 0) | 283.72<br>(247.73 to 323.19) |
| Prevalence | Both | Netherlands | 158.51<br>(141.44 to 176.57) | 160.94<br>(141.58 to 181.71) | 160.94<br>(141.58 to 181.71) | 0<br>(0 to 0) | 152.91<br>(133.23 to 174.26) |
| Prevalence | Male | New Zealand | 141.63<br>(124.79 to 158.88) | 0<br>(0 to 0) | 0<br>(0 to 0) | 0<br>(0 to 0) | 292.39<br>(257.06 to 327.93) |
| Prevalence | Female | New Zealand | 193.15<br>(171.79 to 216.84) | 360.47<br>(316.1 to 406.21) | 360.47<br>(316.1 to 406.21) | 0<br>(0 to 0) | 345.82<br>(302.1 to 391.48) |
| Prevalence | Both | New Zealand | 166.87<br>(149.54 to 186.11) | 0<br>(0 to 0) | 0<br>(0 to 0) | 0<br>(0 to 0) | 228.88<br>(200.81 to 259.42) |
| Prevalence | Male | Nicaragua | 89.17<br>(78.54 to 100.33) | 0<br>(0 to 0) | 0<br>(0 to 0) | 0<br>(0 to 0) | 224.49<br>(198.29 to 252.02) |
| Prevalence | Female | Nicaragua | 170.51<br>(150.08 to 191.81) | 297.31<br>(261.55 to 335.2) | 297.31<br>(261.55 to 335.2) | 0<br>(0 to 0) | 285.8<br>(249.35 to 323.09) |
| Prevalence | Both | Nicaragua | 128.95<br>(114.44 to 144.16) | 0<br>(0 to 0) | 0<br>(0 to 0) | 0<br>(0 to 0) | 151.72<br>(132.53 to 172.91) |
| Prevalence | Male | Niger | 88.23<br>(77.51 to 99.4) | 276.94<br>(245.85 to 310.05) | 276.94<br>(245.85 to 310.05) | 0<br>(0 to 0) | 271.73<br>(238.94 to 304.61) |
| Prevalence | Female | Niger | 160.01<br>(141.54 to 178.93) | 399.88<br>(349.06 to 449.99) | 399.88<br>(349.06 to 449.99) | 0<br>(0 to 0) | 345.82<br>(302.1 to 391.48) |
| Prevalence | Both | Niger | 124.63<br>(110.9 to 138.2) | 197.08<br>(173.41 to 222.18) | 197.08<br>(173.41 to 222.18) | 0<br>(0 to 0) | 192.03<br>(168.22 to 217.97) |
| Prevalence | Male | Nigeria | 88.81<br>(78.61 to 99.66) | 212.57<br>(188.75 to 237.82) | 212.57<br>(188.75 to 237.82) | 0<br>(0 to 0) | 207.43<br>(183.11 to 232.68) |
| Prevalence | Female | Nigeria | 160.94<br>(141.91 to 181.2) | 296.22<br>(258.17 to 335.32) | 296.22<br>(258.17 to 335.32) | 0<br>(0 to 0) | 267.36<br>(233.33 to 302.32) |
| Prevalence | Both | Nigeria | 126.34<br>(112.17 to 140.42) | 144.75<br>(127.51 to 162.89) | 144.75<br>(127.51 to 162.89) | 0<br>(0 to 0) | 143.37<br>(125.32 to 162.21) |
| Prevalence | Male | North Macedonia | 89.17<br>(78.54 to 100.33) | 0<br>(0 to 0) | 0<br>(0 to 0) | 0<br>(0 to 0) | 290.8<br>(257.69 to 326.27) |
| Prevalence | Female | North Macedonia | 170.51<br>(150.08 to 191.81) | 348.79<br>(306.48 to 393.38) | 348.79<br>(306.48 to 393.38) | 0<br>(0 to 0) | 419.38<br>(366.2 to 474.2) |

| | | | | | | | |
|---|---|---|---|---|---|---|---|
| Prevalence | Both | North Macedonia | 128.5<br>(114.03 to 143.63) | 0<br>(0 to 0) | 0<br>(0 to 0) | 0<br>(0 to 0) | 163.05<br>(142.77 to 184.67) |
| Prevalence | Male | Northern Mariana Islands | 108.19<br>(95.55 to 121.83) | 0<br>(0 to 0) | 0<br>(0 to 0) | 387.22<br>(344.18 to 433.44) | 325.7<br>(288.46 to 368.55) |
| Prevalence | Female | Northern Mariana Islands | 192.69<br>(168.27 to 216.69) | 402.26<br>(354.31 to 453.58) | 402.26<br>(354.31 to 453.58) | 459.5<br>(404.27 to 519.73) | 482.39<br>(423.42 to 550.1) |
| Prevalence | Both | Northern Mariana Islands | 148.97<br>(132.44 to 166.73) | 0<br>(0 to 0) | 0<br>(0 to 0) | 321.37<br>(282.84 to 361.52) | 153.72<br>(134.98 to 173.56) |
| Prevalence | Male | Norway | 151.8<br>(133.88 to 170.69) | 239.01<br>(211.98 to 267.62) | 239.01<br>(211.98 to 267.62) | 0<br>(0 to 0) | 236.87<br>(207.96 to 268.35) |
| Prevalence | Female | Norway | 167.33<br>(148.44 to 188.74) | 349.03<br>(307.48 to 400.67) | 349.03<br>(307.48 to 400.67) | 0<br>(0 to 0) | 303.52<br>(264.36 to 346.74) |
| Prevalence | Both | Norway | 159.35<br>(142.56 to 177.74) | 162.86<br>(143.47 to 184.17) | 162.86<br>(143.47 to 184.17) | 0<br>(0 to 0) | 158.65<br>(139.41 to 180.49) |
| Prevalence | Male | Oman | 84.13<br>(73.9 to 94.89) | 0<br>(0 to 0) | 0<br>(0 to 0) | 0<br>(0 to 0) | 221.02<br>(195.37 to 248.09) |
| Prevalence | Female | Oman | 152.29<br>(133.8 to 170.57) | 297.31<br>(261.55 to 335.2) | 297.31<br>(261.55 to 335.2) | 0<br>(0 to 0) | 285.8<br>(249.35 to 323.09) |
| Prevalence | Both | Oman | 117.57<br>(104.66 to 131.32) | 0<br>(0 to 0) | 0<br>(0 to 0) | 0<br>(0 to 0) | 151.72<br>(132.53 to 172.91) |
| Prevalence | Male | Pakistan | 80.54<br>(70.9 to 91.06) | 233.64<br>(207.01 to 261.26) | 233.64<br>(207.01 to 261.26) | 0<br>(0 to 0) | 226.61<br>(200.46 to 255.01) |
| Prevalence | Female | Pakistan | 151.33<br>(133.19 to 170.08) | 0<br>(0 to 0) | 0<br>(0 to 0) | 0<br>(0 to 0) | 287.19<br>(251.21 to 327.41) |
| Prevalence | Both | Pakistan | 114.79<br>(102.2 to 128.84) | 159.94<br>(140.54 to 180.65) | 159.94<br>(140.54 to 180.65) | 0<br>(0 to 0) | 152.46<br>(133.74 to 173.41) |
| Prevalence | Male | Palestine | 84.13<br>(73.9 to 94.89) | 279.3<br>(247.75 to 312.68) | 279.3<br>(247.75 to 312.68) | 0<br>(0 to 0) | 265.37<br>(233.63 to 297.3) |
| Prevalence | Female | Palestine | 152.29<br>(133.8 to 170.57) | 0<br>(0 to 0) | 0<br>(0 to 0) | 0<br>(0 to 0) | 345.82<br>(302.1 to 391.48) |
| Prevalence | Both | Palestine | 117.41<br>(104.52 to 131.15) | 200.13<br>(176.17 to 225.81) | 200.13<br>(176.17 to 225.81) | 0<br>(0 to 0) | 192.03<br>(168.22 to 217.97) |
| Prevalence | Male | Panama | 89.17 | 238.51 | 238.51 | 0 | 229.73 |

| | | | | | | | |
|---|---|---|---|---|---|---|---|
| | | | (78.54 to 100.33) | (211.74 to 267.19) | (211.74 to 267.19) | (0 to 0) | (201.84 to 260.1) |
| Prevalence | Female | Panama | 170.51<br>(150.08 to 191.81) | 0<br>(0 to 0) | 0<br>(0 to 0) | 0<br>(0 to 0) | 303.52<br>(264.36 to 346.74) |
| Prevalence | Both | Panama | 128.91<br>(114.4 to 144.11) | 165.2<br>(145.61 to 186.82) | 165.2<br>(145.61 to 186.82) | 0<br>(0 to 0) | 158.65<br>(139.41 to 180.49) |
| Prevalence | Male | Papua New Guinea | 108.19<br>(95.55 to 121.83) | 0<br>(0 to 0) | 0<br>(0 to 0) | 0<br>(0 to 0) | 269.09<br>(236.86 to 302.31) |
| Prevalence | Female | Papua New Guinea | 192.69<br>(168.27 to 216.69) | 360.47<br>(316.1 to 406.21) | 360.47<br>(316.1 to 406.21) | 0<br>(0 to 0) | 345.82<br>(302.1 to 391.48) |
| Prevalence | Both | Papua New Guinea | 148.02<br>(131.62 to 165.66) | 0<br>(0 to 0) | 0<br>(0 to 0) | 0<br>(0 to 0) | 192.03<br>(168.22 to 217.97) |
| Prevalence | Male | Paraguay | 89.17<br>(78.54 to 100.33) | 0<br>(0 to 0) | 0<br>(0 to 0) | 0<br>(0 to 0) | 289.21<br>(255.52 to 323.83) |
| Prevalence | Female | Paraguay | 170.51<br>(150.08 to 191.81) | 350.19<br>(308.18 to 394.34) | 350.19<br>(308.18 to 394.34) | 0<br>(0 to 0) | 420.39<br>(367.78 to 472.41) |
| Prevalence | Both | Paraguay | 128.91<br>(114.41 to 144.12) | 0<br>(0 to 0) | 0<br>(0 to 0) | 0<br>(0 to 0) | 164.4<br>(142.95 to 184.94) |
| Prevalence | Male | Peru | 89.17<br>(78.54 to 100.33) | 194.84<br>(173.27 to 217.7) | 194.84<br>(173.27 to 217.7) | 0<br>(0 to 0) | 186.51<br>(164.96 to 209.86) |
| Prevalence | Female | Peru | 170.51<br>(150.08 to 191.81) | 0<br>(0 to 0) | 0<br>(0 to 0) | 0<br>(0 to 0) | 270.89<br>(236.53 to 307.89) |
| Prevalence | Both | Peru | 127.67<br>(113.3 to 142.67) | 150.39<br>(132.07 to 169.81) | 150.39<br>(132.07 to 169.81) | 0<br>(0 to 0) | 143.11<br>(125.25 to 162.9) |
| Prevalence | Male | Philippines | 129.56<br>(113.99 to 146.06) | 0<br>(0 to 0) | 0<br>(0 to 0) | 0<br>(0 to 0) | 201.59<br>(177.69 to 225.92) |
| Prevalence | Female | Philippines | 193.98<br>(170.95 to 218.3) | 280.41<br>(246.03 to 316.35) | 280.41<br>(246.03 to 316.35) | 0<br>(0 to 0) | 268.64<br>(235.65 to 303.65) |
| Prevalence | Both | Philippines | 160.93<br>(143.4 to 179.96) | 0<br>(0 to 0) | 0<br>(0 to 0) | 0<br>(0 to 0) | 142.4<br>(124.55 to 161.31) |
| Prevalence | Male | Plurinational State of Bolivia | 0<br>(0 to 0) | 300.59<br>(265.05 to 336.42) | 300.59<br>(265.05 to 336.42) | 298.77<br>(265.7 to 333.7) | 265.05<br>(233.39 to 296.99) |
| Prevalence | Female | Plurinational State of Bolivia | 0<br>(0 to 0) | 354.94<br>(311.47 to 400.07) | 354.94<br>(311.47 to 400.07) | 394.39<br>(347.4 to 444.33) | 345.82<br>(302.1 to 391.48) |

| | | | | | | | |
|---|---|---|---|---|---|---|---|
| Prevalence | Both | Plurinational State of Bolivia | 0<br>(0 to 0) | 221.87<br>(195.92 to 251.76) | 221.87<br>(195.92 to 251.76) | 205.86<br>(181.89 to 232.35) | 192.03<br>(168.22 to 217.97) |
| Prevalence | Male | Poland | 89.75<br>(79.66 to 101.18) | 212.11<br>(188.46 to 237.01) | 212.11<br>(188.46 to 237.01) | 0<br>(0 to 0) | 204.77<br>(180.59 to 230.33) |
| Prevalence | Female | Poland | 171.11<br>(151.12 to 192.76) | 311.39<br>(273.7 to 351.19) | 311.39<br>(273.7 to 351.19) | 0<br>(0 to 0) | 270.89<br>(236.53 to 307.89) |
| Prevalence | Both | Poland | 129.39<br>(115.71 to 144.67) | 148.43<br>(130.37 to 167.58) | 148.43<br>(130.37 to 167.58) | 0<br>(0 to 0) | 143.11<br>(125.25 to 162.9) |
| Prevalence | Male | Portugal | 151.04<br>(133.89 to 169.5) | 0<br>(0 to 0) | 0<br>(0 to 0) | 0<br>(0 to 0) | 231.25<br>(203.18 to 261.89) |
| Prevalence | Female | Portugal | 166.54<br>(146.31 to 187.58) | 316.6<br>(278.53 to 358.19) | 316.6<br>(278.53 to 358.19) | 0<br>(0 to 0) | 303.52<br>(264.36 to 346.74) |
| Prevalence | Both | Portugal | 158.61<br>(141.4 to 176.36) | 0<br>(0 to 0) | 0<br>(0 to 0) | 0<br>(0 to 0) | 158.65<br>(139.41 to 180.49) |
| Prevalence | Male | Principality of Monaco | 0<br>(0 to 0) | 0<br>(0 to 0) | 0<br>(0 to 0) | 0<br>(0 to 0) | 263.84<br>(232.4 to 295.61) |
| Prevalence | Female | Principality of Monaco | 0<br>(0 to 0) | 360.47<br>(316.1 to 406.21) | 360.47<br>(316.1 to 406.21) | 0<br>(0 to 0) | 345.82<br>(302.1 to 391.48) |
| Prevalence | Both | Principality of Monaco | 0<br>(0 to 0) | 0<br>(0 to 0) | 0<br>(0 to 0) | 0<br>(0 to 0) | 192.03<br>(168.22 to 217.97) |
| Prevalence | Male | Puerto Rico | 89.17<br>(78.54 to 100.33) | 236.37<br>(209.75 to 264.55) | 236.37<br>(209.75 to 264.55) | 0<br>(0 to 0) | 231.04<br>(202.99 to 261.65) |
| Prevalence | Female | Puerto Rico | 170.51<br>(150.08 to 191.81) | 349.03<br>(307.48 to 400.67) | 349.03<br>(307.48 to 400.67) | 0<br>(0 to 0) | 303.52<br>(264.36 to 346.74) |
| Prevalence | Both | Puerto Rico | 129.08<br>(114.55 to 144.3) | 162.86<br>(143.47 to 184.17) | 162.86<br>(143.47 to 184.17) | 0<br>(0 to 0) | 158.65<br>(139.41 to 180.49) |
| Prevalence | Male | Qatar | 84.13<br>(73.9 to 94.89) | 0<br>(0 to 0) | 0<br>(0 to 0) | 0<br>(0 to 0) | 233.23<br>(204.86 to 264.15) |
| Prevalence | Female | Qatar | 152.29<br>(133.8 to 170.57) | 316.6<br>(278.53 to 358.19) | 316.6<br>(278.53 to 358.19) | 0<br>(0 to 0) | 303.52<br>(264.36 to 346.74) |
| Prevalence | Both | Qatar | 113.47<br>(101.07 to 126.94) | 0<br>(0 to 0) | 0<br>(0 to 0) | 0<br>(0 to 0) | 158.65<br>(139.41 to 180.49) |
| Prevalence | Male | Republic of Cabo Verde | 0 | 0 | 0 | 0 | 288.46 |

| | | | | | | | |
|---|---|---|---|---|---|---|---|
| | | | (0 to 0) | (0 to 0) | (0 to 0) | (0 to 0) | (254.8 to 323.15) |
| Prevalence | Female | Republic of Cabo Verde | 0<br>(0 to 0) | 362.48<br>(318.92 to 408.75) | 362.48<br>(318.92 to 408.75) | 0<br>(0 to 0) | 346.77<br>(302.07 to 393.39) |
| Prevalence | Both | Republic of Cabo Verde | 0<br>(0 to 0) | 0<br>(0 to 0) | 0<br>(0 to 0) | 0<br>(0 to 0) | 230.23<br>(200.87 to 261.2) |
| Prevalence | Male | Republic of Coted'Ivoire | 0<br>(0 to 0) | 238.25<br>(211.45 to 266.75) | 238.25<br>(211.45 to 266.75) | 0<br>(0 to 0) | 233.32<br>(206.61 to 261.05) |
| Prevalence | Female | Republic of Coted'Ivoire | 0<br>(0 to 0) | 350.36<br>(307.59 to 398.42) | 350.36<br>(307.59 to 398.42) | 0<br>(0 to 0) | 304.86<br>(267.67 to 343.84) |
| Prevalence | Both | Republic of Coted'Ivoire | 0<br>(0 to 0) | 163.71<br>(144.31 to 184.46) | 163.71<br>(144.31 to 184.46) | 0<br>(0 to 0) | 159.48<br>(139.18 to 180.85) |
| Prevalence | Male | Republic of Korea | 140.81<br>(124.13 to 158.33) | 304.03<br>(270.27 to 339.33) | 304.03<br>(270.27 to 339.33) | 0<br>(0 to 0) | 297.64<br>(262.91 to 334.66) |
| Prevalence | Female | Republic of Korea | 190.29<br>(168.4 to 214.44) | 431.41<br>(378.37 to 490.96) | 431.41<br>(378.37 to 490.96) | 0<br>(0 to 0) | 418.49<br>(366.83 to 476.04) |
| Prevalence | Both | Republic of Korea | 164.56<br>(147.07 to 183.74) | 275.18<br>(242.57 to 311.02) | 275.18<br>(242.57 to 311.02) | 0<br>(0 to 0) | 163.59<br>(142.91 to 185.21) |
| Prevalence | Male | Republic of Moldova | 89.17<br>(78.54 to 100.33) | 269.32<br>(239.08 to 304.37) | 269.32<br>(239.08 to 304.37) | 0<br>(0 to 0) | 235.98<br>(207.21 to 267.34) |
| Prevalence | Female | Republic of Moldova | 170.51<br>(150.08 to 191.81) | 312.06<br>(274.47 to 352.23) | 312.06<br>(274.47 to 352.23) | 0<br>(0 to 0) | 303.52<br>(264.36 to 346.74) |
| Prevalence | Both | Republic of Moldova | 128.76<br>(114.27 to 143.94) | 181.89<br>(160.92 to 205.71) | 181.89<br>(160.92 to 205.71) | 0<br>(0 to 0) | 158.65<br>(139.41 to 180.49) |
| Prevalence | Male | Republic of Nauru | 0<br>(0 to 0) | 0<br>(0 to 0) | 0<br>(0 to 0) | 0<br>(0 to 0) | 169.68<br>(150.08 to 190.93) |
| Prevalence | Female | Republic of Nauru | 0<br>(0 to 0) | 282.14<br>(247.99 to 318.06) | 282.14<br>(247.99 to 318.06) | 0<br>(0 to 0) | 270.89<br>(236.53 to 307.89) |
| Prevalence | Both | Republic of Nauru | 0<br>(0 to 0) | 0<br>(0 to 0) | 0<br>(0 to 0) | 0<br>(0 to 0) | 143.11<br>(125.25 to 162.9) |
| Prevalence | Male | Republic of Niue | 0<br>(0 to 0) | 0<br>(0 to 0) | 0<br>(0 to 0) | 0<br>(0 to 0) | 312.1<br>(274.03 to 351.3) |
| Prevalence | Female | Republic of Niue | 0<br>(0 to 0) | 396.9<br>(348.73 to 448.97) | 396.9<br>(348.73 to 448.97) | 0<br>(0 to 0) | 475.68<br>(415.44 to 539.73) |

| | | | | | | | |
|---|---|---|---|---|---|---|---|
| Prevalence | Both | Republic of Niue | 0<br>(0 to 0) | 0<br>(0 to 0) | 0<br>(0 to 0) | 0<br>(0 to 0) | 152.59<br>(133.17 to 173.27) |
| Prevalence | Male | Republic of Palau | 0<br>(0 to 0) | 230.8<br>(205.29 to 259.61) | 230.8<br>(205.29 to 259.61) | 0<br>(0 to 0) | 237.33<br>(208.37 to 268.92) |
| Prevalence | Female | Republic of Palau | 0<br>(0 to 0) | 317.63<br>(279.37 to 359.3) | 317.63<br>(279.37 to 359.3) | 0<br>(0 to 0) | 303.52<br>(264.36 to 346.74) |
| Prevalence | Both | Republic of Palau | 0<br>(0 to 0) | 149.6<br>(131.45 to 168.11) | 149.6<br>(131.45 to 168.11) | 0<br>(0 to 0) | 158.65<br>(139.41 to 180.49) |
| Prevalence | Male | Republic of San Marino | 0<br>(0 to 0) | 236.51<br>(209.88 to 264.75) | 236.51<br>(209.88 to 264.75) | 0<br>(0 to 0) | 231.12<br>(203.09 to 261.68) |
| Prevalence | Female | Republic of San Marino | 0<br>(0 to 0) | 349.03<br>(307.48 to 400.67) | 349.03<br>(307.48 to 400.67) | 0<br>(0 to 0) | 303.52<br>(264.36 to 346.74) |
| Prevalence | Both | Republic of San Marino | 0<br>(0 to 0) | 162.86<br>(143.47 to 184.17) | 162.86<br>(143.47 to 184.17) | 0<br>(0 to 0) | 158.65<br>(139.41 to 180.49) |
| Prevalence | Male | Republic of the Gambia | 0<br>(0 to 0) | 240.28<br>(213.18 to 269.03) | 240.28<br>(213.18 to 269.03) | 0<br>(0 to 0) | 238.3<br>(211.19 to 266.52) |
| Prevalence | Female | Republic of the Gambia | 0<br>(0 to 0) | 350.36<br>(307.59 to 398.42) | 350.36<br>(307.59 to 398.42) | 0<br>(0 to 0) | 304.86<br>(267.67 to 343.84) |
| Prevalence | Both | Republic of the Gambia | 0<br>(0 to 0) | 163.71<br>(144.31 to 184.46) | 163.71<br>(144.31 to 184.46) | 0<br>(0 to 0) | 159.48<br>(139.18 to 180.85) |
| Prevalence | Male | Romania | 89.17<br>(78.54 to 100.33) | 228.02<br>(202.61 to 254.69) | 228.02<br>(202.61 to 254.69) | 0<br>(0 to 0) | 224.89<br>(198.59 to 253.74) |
| Prevalence | Female | Romania | 170.51<br>(150.08 to 191.81) | 298.64<br>(261.24 to 339.63) | 298.64<br>(261.24 to 339.63) | 0<br>(0 to 0) | 283.72<br>(247.73 to 323.19) |
| Prevalence | Both | Romania | 128.68<br>(114.2 to 143.85) | 160.54<br>(141.69 to 180.43) | 160.54<br>(141.69 to 180.43) | 0<br>(0 to 0) | 152.91<br>(133.23 to 174.26) |
| Prevalence | Male | Russian Federation | 89.75<br>(79.66 to 101.18) | 0<br>(0 to 0) | 0<br>(0 to 0) | 0<br>(0 to 0) | 229.14<br>(201.32 to 259.3) |
| Prevalence | Female | Russian Federation | 171.11<br>(151.12 to 192.76) | 316.6<br>(278.53 to 358.19) | 316.6<br>(278.53 to 358.19) | 0<br>(0 to 0) | 303.52<br>(264.36 to 346.74) |
| Prevalence | Both | Russian Federation | 129.43<br>(115.74 to 144.7) | 0<br>(0 to 0) | 0<br>(0 to 0) | 0<br>(0 to 0) | 158.65<br>(139.41 to 180.49) |
| Prevalence | Male | Rwanda | 91.6 | 266.81 | 266.81 | 0 | 230.96 |

| | | | | | | | |
|---|---|---|---|---|---|---|---|
| | | | (81.03 to 103.15) | (237 to 301.35) | (237 to 301.35) | (0 to 0) | (202.92 to 261.54) |
| Prevalence | Female | Rwanda | 157.76 (138.72 to 177.99) | 312.06 (274.47 to 352.23) | 312.06 (274.47 to 352.23) | 0 (0 to 0) | 303.52 (264.36 to 346.74) |
| Prevalence | Both | Rwanda | 124.96 (111.55 to 139.58) | 181.89 (160.92 to 205.71) | 181.89 (160.92 to 205.71) | 0 (0 to 0) | 158.65 (139.41 to 180.49) |
| Prevalence | Male | Saint Kitts and Nevis | 89.17 (78.54 to 100.33) | 225.5 (198.81 to 254.18) | 225.5 (198.81 to 254.18) | 0 (0 to 0) | 227.11 (199.56 to 256.85) |
| Prevalence | Female | Saint Kitts and Nevis | 170.51 (150.08 to 191.81) | 319.93 (281.74 to 361.35) | 319.93 (281.74 to 361.35) | 0 (0 to 0) | 303.52 (264.36 to 346.74) |
| Prevalence | Both | Saint Kitts and Nevis | 129.83 (115.24 to 145.19) | 157.26 (137.79 to 178.54) | 157.26 (137.79 to 178.54) | 0 (0 to 0) | 158.65 (139.41 to 180.49) |
| Prevalence | Male | Saint Lucia | 89.17 (78.54 to 100.33) | 346.11 (306.37 to 388.71) | 346.11 (306.37 to 388.71) | 0 (0 to 0) | 264.46 (232.88 to 296.34) |
| Prevalence | Female | Saint Lucia | 170.51 (150.08 to 191.81) | 300.53 (263.81 to 337.46) | 300.53 (263.81 to 337.46) | 0 (0 to 0) | 345.82 (302.1 to 391.48) |
| Prevalence | Both | Saint Lucia | 129.1 (114.57 to 144.34) | 248.76 (218.23 to 281.04) | 248.76 (218.23 to 281.04) | 0 (0 to 0) | 192.03 (168.22 to 217.97) |
| Prevalence | Male | Saint Vincent and the Grenadines | 89.17 (78.54 to 100.33) | 357.45 (317.52 to 399.31) | 357.45 (317.52 to 399.31) | 0 (0 to 0) | 295.39 (260.92 to 332.03) |
| Prevalence | Female | Saint Vincent and the Grenadines | 170.51 (150.08 to 191.81) | 226.31 (198.43 to 255.7) | 226.31 (198.43 to 255.7) | 0 (0 to 0) | 418.49 (366.83 to 476.04) |
| Prevalence | Both | Saint Vincent and the Grenadines | 128.75 (114.26 to 143.93) | 302.8 (266.8 to 341.78) | 302.8 (266.8 to 341.78) | 0 (0 to 0) | 163.59 (142.91 to 185.21) |
| Prevalence | Male | Samoa | 108.19 (95.55 to 121.83) | 217.44 (191.79 to 244.49) | 217.44 (191.79 to 244.49) | 0 (0 to 0) | 218.91 (193.51 to 245.65) |
| Prevalence | Female | Samoa | 192.69 (168.27 to 216.69) | 300.44 (264.74 to 338.49) | 300.44 (264.74 to 338.49) | 0 (0 to 0) | 285.8 (249.35 to 323.09) |
| Prevalence | Both | Samoa | 148.85 (132.34 to 166.59) | 150.75 (131.8 to 171.32) | 150.75 (131.8 to 171.32) | 0 (0 to 0) | 151.72 (132.53 to 172.91) |
| Prevalence | Male | Sao Tome and Principe | 88.23 (77.51 to 99.4) | 201.54 (179.25 to 225.04) | 201.54 (179.25 to 225.04) | 0 (0 to 0) | 194.52 (171.66 to 218.87) |
| Prevalence | Female | Sao Tome and Principe | 160.01 (141.54 to 178.93) | 311.39 (273.7 to 351.19) | 311.39 (273.7 to 351.19) | 0 (0 to 0) | 270.89 (236.53 to 307.89) |

| | | | | | | | |
|---|---|---|---|---|---|---|---|
| Prevalence | Both | Sao Tome and Principe | 124.24 (110.53 to 137.79) | 148.43 (130.37 to 167.58) | 148.43 (130.37 to 167.58) | 0 (0 to 0) | 143.11 (125.25 to 162.9) |
| Prevalence | Male | Saudi Arabia | 84.13 (73.9 to 94.89) | 231 (205.15 to 258.3) | 231 (205.15 to 258.3) | 0 (0 to 0) | 221.11 (195.48 to 248.19) |
| Prevalence | Female | Saudi Arabia | 152.29 (133.8 to 170.57) | 278.72 (243.25 to 315.71) | 278.72 (243.25 to 315.71) | 0 (0 to 0) | 285.8 (249.35 to 323.09) |
| Prevalence | Both | Saudi Arabia | 114.81 (102.26 to 128.37) | 159.85 (140.38 to 180.25) | 159.85 (140.38 to 180.25) | 0 (0 to 0) | 151.72 (132.53 to 172.91) |
| Prevalence | Male | Senegal | 88.23 (77.51 to 99.4) | 264.68 (235.23 to 298.62) | 264.68 (235.23 to 298.62) | 0 (0 to 0) | 232.05 (203.88 to 262.78) |
| Prevalence | Female | Senegal | 160.01 (141.54 to 178.93) | 312.06 (274.47 to 352.23) | 312.06 (274.47 to 352.23) | 0 (0 to 0) | 303.52 (264.36 to 346.74) |
| Prevalence | Both | Senegal | 122.62 (109.12 to 136.01) | 181.89 (160.92 to 205.71) | 181.89 (160.92 to 205.71) | 0 (0 to 0) | 158.65 (139.41 to 180.49) |
| Prevalence | Male | Serbia | 89.17 (78.54 to 100.33) | 0 (0 to 0) | 0 (0 to 0) | 0 (0 to 0) | 285.58 (251.68 to 320.29) |
| Prevalence | Female | Serbia | 170.51 (150.08 to 191.81) | 360.47 (316.1 to 406.21) | 360.47 (316.1 to 406.21) | 0 (0 to 0) | 345.82 (302.1 to 391.48) |
| Prevalence | Both | Serbia | 127.72 (113.33 to 142.72) | 0 (0 to 0) | 0 (0 to 0) | 0 (0 to 0) | 228.88 (200.81 to 259.42) |
| Prevalence | Male | Seychelles | 128.96 (113.35 to 144.69) | 228.59 (202.91 to 255.79) | 228.59 (202.91 to 255.79) | 0 (0 to 0) | 215.6 (190.58 to 241.87) |
| Prevalence | Female | Seychelles | 192.69 (168.27 to 216.69) | 0 (0 to 0) | 0 (0 to 0) | 0 (0 to 0) | 285.8 (249.35 to 323.09) |
| Prevalence | Both | Seychelles | 160.19 (142.81 to 178.83) | 159.07 (139.67 to 179.42) | 159.07 (139.67 to 179.42) | 0 (0 to 0) | 151.72 (132.53 to 172.91) |
| Prevalence | Male | Sierra Leone | 88.23 (77.51 to 99.4) | 326.41 (288.08 to 364.96) | 326.41 (288.08 to 364.96) | 0 (0 to 0) | 307.43 (270.14 to 346.05) |
| Prevalence | Female | Sierra Leone | 160.01 (141.54 to 178.93) | 384.25 (337.38 to 434.79) | 384.25 (337.38 to 434.79) | 0 (0 to 0) | 475.68 (415.44 to 539.73) |
| Prevalence | Both | Sierra Leone | 124.68 (110.94 to 138.26) | 214.27 (187.5 to 243.11) | 214.27 (187.5 to 243.11) | 0 (0 to 0) | 152.59 (133.17 to 173.27) |
| Prevalence | Male | Singapore | 140.81 | 236.54 | 236.54 | 0 | 231.99 |

| | | | | | | | |
|---|---|---|---|---|---|---|---|
| | | | (124.13 to 158.33) | (209.9 to 264.76) | (209.9 to 264.76) | (0 to 0) | (203.83 to 262.71) |
| Prevalence | Female | Singapore | 190.29<br>(168.4 to 214.44) | 349.03<br>(307.48 to 400.67) | 349.03<br>(307.48 to 400.67) | 0<br>(0 to 0) | 303.52<br>(264.36 to 346.74) |
| Prevalence | Both | Singapore | 164.28<br>(146.88 to 183.39) | 162.86<br>(143.47 to 184.17) | 162.86<br>(143.47 to 184.17) | 0<br>(0 to 0) | 158.65<br>(139.41 to 180.49) |
| Prevalence | Male | Slovakia | 89.17<br>(78.54 to 100.33) | 243.45<br>(215.94 to 272.66) | 243.45<br>(215.94 to 272.66) | 0<br>(0 to 0) | 230.25<br>(202.3 to 260.72) |
| Prevalence | Female | Slovakia | 170.51<br>(150.08 to 191.81) | 292.85<br>(258.37 to 333.24) | 292.85<br>(258.37 to 333.24) | 0<br>(0 to 0) | 303.52<br>(264.36 to 346.74) |
| Prevalence | Both | Slovakia | 128.74<br>(114.25 to 143.92) | 168.64<br>(148.43 to 190.88) | 168.64<br>(148.43 to 190.88) | 0<br>(0 to 0) | 158.65<br>(139.41 to 180.49) |
| Prevalence | Male | Slovenia | 89.17<br>(78.54 to 100.33) | 275.7<br>(244.8 to 308.56) | 275.7<br>(244.8 to 308.56) | 0<br>(0 to 0) | 267.09<br>(235.03 to 299.23) |
| Prevalence | Female | Slovenia | 170.51<br>(150.08 to 191.81) | 399.88<br>(349.06 to 449.99) | 399.88<br>(349.06 to 449.99) | 0<br>(0 to 0) | 345.82<br>(302.1 to 391.48) |
| Prevalence | Both | Slovenia | 128.58<br>(114.11 to 143.73) | 197.08<br>(173.41 to 222.18) | 197.08<br>(173.41 to 222.18) | 0<br>(0 to 0) | 192.03<br>(168.22 to 217.97) |
| Prevalence | Male | Socialist Republic of Viet Nam | 0<br>(0 to 0) | 224.17<br>(199.02 to 250.68) | 224.17<br>(199.02 to 250.68) | 0<br>(0 to 0) | 224.07<br>(197.83 to 252.93) |
| Prevalence | Female | Socialist Republic of Viet Nam | 0<br>(0 to 0) | 325.9<br>(285.78 to 371.71) | 325.9<br>(285.78 to 371.71) | 0<br>(0 to 0) | 283.72<br>(247.73 to 323.19) |
| Prevalence | Both | Socialist Republic of Viet Nam | 0<br>(0 to 0) | 158.77<br>(139.9 to 178.86) | 158.77<br>(139.9 to 178.86) | 0<br>(0 to 0) | 152.91<br>(133.23 to 174.26) |
| Prevalence | Male | Solomon Islands | 108.19<br>(95.55 to 121.83) | 0<br>(0 to 0) | 0<br>(0 to 0) | 0<br>(0 to 0) | 226.06<br>(199.96 to 254.4) |
| Prevalence | Female | Solomon Islands | 192.69<br>(168.27 to 216.69) | 298.65<br>(262.2 to 336.79) | 298.65<br>(262.2 to 336.79) | 0<br>(0 to 0) | 287.19<br>(251.21 to 327.41) |
| Prevalence | Both | Solomon Islands | 148.79<br>(132.3 to 166.52) | 0<br>(0 to 0) | 0<br>(0 to 0) | 0<br>(0 to 0) | 152.46<br>(133.74 to 173.41) |
| Prevalence | Male | Somalia | 91.6<br>(81.03 to 103.15) | 207.59<br>(183.1 to 233.23) | 207.59<br>(183.1 to 233.23) | 0<br>(0 to 0) | 214.99<br>(189.99 to 242.68) |
| Prevalence | Female | Somalia | 157.76<br>(138.72 to 177.99) | 297.3<br>(261.16 to 336.25) | 297.3<br>(261.16 to 336.25) | 0<br>(0 to 0) | 283.72<br>(247.73 to 323.19) |

| | | | | | | | |
|---|---|---|---|---|---|---|---|
| Prevalence | Both | Somalia | 123.39<br>(110.15 to 137.79) | 150.28<br>(132.03 to 170.94) | 150.28<br>(132.03 to 170.94) | 0<br>(0 to 0) | 152.91<br>(133.23 to 174.26) |
| Prevalence | Male | South Africa | 88.81<br>(78.61 to 99.66) | 297.85<br>(264.93 to 332.23) | 297.85<br>(264.93 to 332.23) | 0<br>(0 to 0) | 292.41<br>(258.36 to 328.89) |
| Prevalence | Female | South Africa | 160.94<br>(141.91 to 181.2) | 435.26<br>(381.34 to 493.27) | 435.26<br>(381.34 to 493.27) | 0<br>(0 to 0) | 418.49<br>(366.83 to 476.04) |
| Prevalence | Both | South Africa | 124.3<br>(110.44 to 138.14) | 275.61<br>(242.92 to 311.14) | 275.61<br>(242.92 to 311.14) | 0<br>(0 to 0) | 163.59<br>(142.91 to 185.21) |
| Prevalence | Male | South Sudan | 91.6<br>(81.03 to 103.15) | 189.89<br>(167.87 to 212.21) | 189.89<br>(167.87 to 212.21) | 0<br>(0 to 0) | 290.42<br>(255.36 to 325.73) |
| Prevalence | Female | South Sudan | 157.76<br>(138.72 to 177.99) | 425.99<br>(373.81 to 480.21) | 425.99<br>(373.81 to 480.21) | 0<br>(0 to 0) | 345.82<br>(302.1 to 391.48) |
| Prevalence | Both | South Sudan | 123.14<br>(109.92 to 137.49) | 151.06<br>(132.64 to 170.37) | 151.06<br>(132.64 to 170.37) | 0<br>(0 to 0) | 228.88<br>(200.81 to 259.42) |
| Prevalence | Male | Spain | 151.04<br>(133.89 to 169.5) | 213.52<br>(189.62 to 238.59) | 213.52<br>(189.62 to 238.59) | 0<br>(0 to 0) | 202.72<br>(178.73 to 228.11) |
| Prevalence | Female | Spain | 166.54<br>(146.31 to 187.58) | 311.39<br>(273.7 to 351.19) | 311.39<br>(273.7 to 351.19) | 0<br>(0 to 0) | 270.89<br>(236.53 to 307.89) |
| Prevalence | Both | Spain | 158.58<br>(141.35 to 176.32) | 148.43<br>(130.37 to 167.58) | 148.43<br>(130.37 to 167.58) | 0<br>(0 to 0) | 143.11<br>(125.25 to 162.9) |
| Prevalence | Male | Sri Lanka | 128.96<br>(113.35 to 144.69) | 0<br>(0 to 0) | 0<br>(0 to 0) | 0<br>(0 to 0) | 232.02<br>(203.85 to 262.77) |
| Prevalence | Female | Sri Lanka | 192.69<br>(168.27 to 216.69) | 316.6<br>(278.53 to 358.19) | 316.6<br>(278.53 to 358.19) | 0<br>(0 to 0) | 303.52<br>(264.36 to 346.74) |
| Prevalence | Both | Sri Lanka | 160.61<br>(143.18 to 179.34) | 0<br>(0 to 0) | 0<br>(0 to 0) | 0<br>(0 to 0) | 158.65<br>(139.41 to 180.49) |
| Prevalence | Male | Sudan | 84.13<br>(73.9 to 94.89) | 0<br>(0 to 0) | 0<br>(0 to 0) | 0<br>(0 to 0) | 290.79<br>(256.84 to 325.66) |
| Prevalence | Female | Sudan | 152.29<br>(133.8 to 170.57) | 350.19<br>(308.18 to 394.34) | 350.19<br>(308.18 to 394.34) | 0<br>(0 to 0) | 420.39<br>(367.78 to 472.41) |
| Prevalence | Both | Sudan | 117.06<br>(104.21 to 130.79) | 0<br>(0 to 0) | 0<br>(0 to 0) | 0<br>(0 to 0) | 164.4<br>(142.95 to 184.94) |
| Prevalence | Male | Suriname | 89.17 | 379.84 | 379.84 | 297.81 | 289.51 |

| | | | | | | | |
|---|---|---|---|---|---|---|---|
| | | | (78.54 to 100.33) | (337.11 to 424.71) | (337.11 to 424.71) | (264.88 to 332.56) | (255.85 to 325.63) |
| Prevalence | Female | Suriname | 170.51<br>(150.08 to 191.81) | 223.8<br>(196.44 to 252.09) | 223.8<br>(196.44 to 252.09) | 394.39<br>(347.4 to 444.33) | 418.49<br>(366.83 to 476.04) |
| Prevalence | Both | Suriname | 128.49<br>(114.03 to 143.62) | 320.1<br>(281.75 to 361.64) | 320.1<br>(281.75 to 361.64) | 205.86<br>(181.89 to 232.35) | 163.59<br>(142.91 to 185.21) |
| Prevalence | Male | Sweden | 151.8<br>(133.88 to 170.69) | 201.13<br>(178.08 to 226.08) | 201.13<br>(178.08 to 226.08) | 301.89<br>(267.97 to 337.56) | 205.87<br>(181.56 to 231.6) |
| Prevalence | Female | Sweden | 167.33<br>(148.44 to 188.74) | 283.49<br>(249.27 to 319.45) | 283.49<br>(249.27 to 319.45) | 396.55<br>(348.57 to 447.16) | 270.89<br>(236.53 to 307.89) |
| Prevalence | Both | Sweden | 159.3<br>(142.48 to 177.69) | 140.49<br>(123 to 158.03) | 140.49<br>(123 to 158.03) | 206.83<br>(182.5 to 232.65) | 143.11<br>(125.25 to 162.9) |
| Prevalence | Male | Switzerland | 151.04<br>(133.89 to 169.5) | 287.73<br>(255.95 to 320.36) | 287.73<br>(255.95 to 320.36) | 299.62<br>(266.43 to 334.55) | 257.03<br>(227.25 to 287.8) |
| Prevalence | Female | Switzerland | 166.54<br>(146.31 to 187.58) | 102.63<br>(90.85 to 115.55) | 102.63<br>(90.85 to 115.55) | 394.39<br>(347.4 to 444.33) | 336.71<br>(293.59 to 381.18) |
| Prevalence | Both | Switzerland | 158.53<br>(141.28 to 176.26) | 197.31<br>(174.01 to 222) | 197.31<br>(174.01 to 222) | 205.86<br>(181.89 to 232.35) | 174.37<br>(153.04 to 196.92) |
| Prevalence | Male | Syrian Arab Republic | 84.13<br>(73.9 to 94.89) | 240.76<br>(213.65 to 269.85) | 240.76<br>(213.65 to 269.85) | 300.05<br>(266.76 to 335.17) | 232.63<br>(204.35 to 263.47) |
| Prevalence | Female | Syrian Arab Republic | 152.29<br>(133.8 to 170.57) | 0<br>(0 to 0) | 0<br>(0 to 0) | 394.39<br>(347.4 to 444.33) | 303.52<br>(264.36 to 346.74) |
| Prevalence | Both | Syrian Arab Republic | 117.93<br>(104.98 to 131.71) | 165.2<br>(145.61 to 186.82) | 165.2<br>(145.61 to 186.82) | 205.86<br>(181.89 to 232.35) | 158.65<br>(139.41 to 180.49) |
| Prevalence | Male | Taiwan (Province of China) | 99.81<br>(88.32 to 111.92) | 295.78<br>(262.65 to 331.39) | 295.78<br>(262.65 to 331.39) | 298.94<br>(265.79 to 333.88) | 289.83<br>(254.92 to 325.12) |
| Prevalence | Female | Taiwan (Province of China) | 183.92<br>(163.61 to 205.45) | 399.88<br>(349.06 to 449.99) | 399.88<br>(349.06 to 449.99) | 394.39<br>(347.4 to 444.33) | 345.82<br>(302.1 to 391.48) |
| Prevalence | Both | Taiwan (Province of China) | 140<br>(125.31 to 155.53) | 235.24<br>(206.94 to 265.66) | 235.24<br>(206.94 to 265.66) | 205.86<br>(181.89 to 232.35) | 228.88<br>(200.81 to 259.42) |
| Prevalence | Male | Tajikistan | 89.17<br>(78.54 to 100.33) | 299.25<br>(265.59 to 335.07) | 299.25<br>(265.59 to 335.07) | 0<br>(0 to 0) | 283.41<br>(249.92 to 317.87) |
| Prevalence | Female | Tajikistan | 170.51<br>(150.08 to 191.81) | 0<br>(0 to 0) | 0<br>(0 to 0) | 0<br>(0 to 0) | 345.82<br>(302.1 to 391.48) |

| | | | | | | | |
|---|---|---|---|---|---|---|---|
| Prevalence | Both | Tajikistan | 128.65 (114.17 to 143.81) | 238.68 (209.82 to 269.39) | 238.68 (209.82 to 269.39) | 0 (0 to 0) | 228.88 (200.81 to 259.42) |
| Prevalence | Male | Thailand | 128.96 (113.35 to 144.69) | 228.74 (203.16 to 256) | 228.74 (203.16 to 256) | 0 (0 to 0) | 221.38 (195.67 to 248.47) |
| Prevalence | Female | Thailand | 192.69 (168.27 to 216.69) | 327.25 (287.41 to 368.24) | 327.25 (287.41 to 368.24) | 0 (0 to 0) | 285.8 (249.35 to 323.09) |
| Prevalence | Both | Thailand | 160.16 (142.78 to 178.79) | 156.91 (137.83 to 176.93) | 156.91 (137.83 to 176.93) | 0 (0 to 0) | 151.72 (132.53 to 172.91) |
| Prevalence | Male | Timor-Leste | 128.96 (113.35 to 144.69) | 274.62 (243.65 to 307.21) | 274.62 (243.65 to 307.21) | 349.97 (311.13 to 391.22) | 271.72 (238.92 to 304.59) |
| Prevalence | Female | Timor-Leste | 192.69 (168.27 to 216.69) | 399.88 (349.06 to 449.99) | 399.88 (349.06 to 449.99) | 449.82 (394.99 to 506.15) | 345.82 (302.1 to 391.48) |
| Prevalence | Both | Timor-Leste | 159.96 (142.6 to 178.56) | 197.08 (173.41 to 222.18) | 197.08 (173.41 to 222.18) | 249.31 (219.62 to 280.59) | 192.03 (168.22 to 217.97) |
| Prevalence | Male | Togo | 88.23 (77.51 to 99.4) | 0 (0 to 0) | 0 (0 to 0) | 0 (0 to 0) | 268.66 (236.32 to 301.13) |
| Prevalence | Female | Togo | 160.01 (141.54 to 178.93) | 360.47 (316.1 to 406.21) | 360.47 (316.1 to 406.21) | 0 (0 to 0) | 345.82 (302.1 to 391.48) |
| Prevalence | Both | Togo | 122.83 (109.3 to 136.25) | 0 (0 to 0) | 0 (0 to 0) | 0 (0 to 0) | 192.03 (168.22 to 217.97) |
| Prevalence | Male | Tokelau | 108.19 (95.55 to 121.83) | 263.81 (234.51 to 297.55) | 263.81 (234.51 to 297.55) | 0 (0 to 0) | 230.62 (202.62 to 261.17) |
| Prevalence | Female | Tokelau | 192.69 (168.27 to 216.69) | 312.06 (274.47 to 352.23) | 312.06 (274.47 to 352.23) | 0 (0 to 0) | 303.52 (264.36 to 346.74) |
| Prevalence | Both | Tokelau | 147.12 (130.96 to 164.69) | 181.89 (160.92 to 205.71) | 181.89 (160.92 to 205.71) | 0 (0 to 0) | 158.65 (139.41 to 180.49) |
| Prevalence | Male | Tonga | 108.19 (95.55 to 121.83) | 217.12 (192.84 to 242.72) | 217.12 (192.84 to 242.72) | 0 (0 to 0) | 207.88 (183.37 to 233.93) |
| Prevalence | Female | Tonga | 192.69 (168.27 to 216.69) | 0 (0 to 0) | 0 (0 to 0) | 0 (0 to 0) | 270.89 (236.53 to 307.89) |
| Prevalence | Both | Tonga | 149.05 (132.52 to 166.83) | 150.39 (132.07 to 169.81) | 150.39 (132.07 to 169.81) | 0 (0 to 0) | 143.11 (125.25 to 162.9) |
| Prevalence | Male | Trinidad and Tobago | 89.17 | 0 | 0 | 0 | 201.37 |

| | | | | | | | |
|---|---|---|---|---|---|---|---|
| | | | (78.54 to 100.33) | (0 to 0) | (0 to 0) | (0 to 0) | (176.31 to 226.59) |
| Prevalence | Female | Trinidad and Tobago | 170.51<br>(150.08 to 191.81) | 282.14<br>(247.99 to 318.06) | 282.14<br>(247.99 to 318.06) | 0<br>(0 to 0) | 270.89<br>(236.53 to 307.89) |
| Prevalence | Both | Trinidad and Tobago | 129.08<br>(114.55 to 144.31) | 0<br>(0 to 0) | 0<br>(0 to 0) | 0<br>(0 to 0) | 131.24<br>(113.6 to 151.31) |
| Prevalence | Male | Tunisia | 84.13<br>(73.9 to 94.89) | 227.31<br>(202.15 to 253.98) | 227.31<br>(202.15 to 253.98) | 0<br>(0 to 0) | 235.11<br>(206.48 to 266.35) |
| Prevalence | Female | Tunisia | 152.29<br>(133.8 to 170.57) | 336.18<br>(295.28 to 383.67) | 336.18<br>(295.28 to 383.67) | 0<br>(0 to 0) | 303.52<br>(264.36 to 346.74) |
| Prevalence | Both | Tunisia | 117.09<br>(104.24 to 130.83) | 159.11<br>(140.46 to 179.74) | 159.11<br>(140.46 to 179.74) | 0<br>(0 to 0) | 158.65<br>(139.41 to 180.49) |
| Prevalence | Male | Turkey | 77.88<br>(67.95 to 89.15) | 276.95<br>(245.7 to 309.79) | 276.95<br>(245.7 to 309.79) | 0<br>(0 to 0) | 269.22<br>(236.8 to 301.7) |
| Prevalence | Female | Turkey | 152.29<br>(133.8 to 170.57) | 338.67<br>(295.51 to 385.69) | 338.67<br>(295.51 to 385.69) | 0<br>(0 to 0) | 345.82<br>(302.1 to 391.48) |
| Prevalence | Both | Turkey | 114.08<br>(101.46 to 127.89) | 201.2<br>(177.12 to 226.9) | 201.2<br>(177.12 to 226.9) | 0<br>(0 to 0) | 192.03<br>(168.22 to 217.97) |
| Prevalence | Male | Turkmenistan | 89.17<br>(78.54 to 100.33) | 231.03<br>(204.75 to 258.87) | 231.03<br>(204.75 to 258.87) | 0<br>(0 to 0) | 222.15<br>(196.17 to 250.55) |
| Prevalence | Female | Turkmenistan | 170.51<br>(150.08 to 191.81) | 0<br>(0 to 0) | 0<br>(0 to 0) | 0<br>(0 to 0) | 283.72<br>(247.73 to 323.19) |
| Prevalence | Both | Turkmenistan | 127.97<br>(113.56 to 143.03) | 160.94<br>(141.58 to 181.71) | 160.94<br>(141.58 to 181.71) | 0<br>(0 to 0) | 152.91<br>(133.23 to 174.26) |
| Prevalence | Male | Tuvalu | 108.19<br>(95.55 to 121.83) | 0<br>(0 to 0) | 0<br>(0 to 0) | 277.76<br>(246.93 to 309.61) | 239.84<br>(212.43 to 268.2) |
| Prevalence | Female | Tuvalu | 192.69<br>(168.27 to 216.69) | 318.24<br>(279.63 to 359.55) | 318.24<br>(279.63 to 359.55) | 351.28<br>(309.28 to 395.11) | 304.86<br>(267.67 to 343.84) |
| Prevalence | Both | Tuvalu | 147.77<br>(131.43 to 165.41) | 0<br>(0 to 0) | 0<br>(0 to 0) | 185.99<br>(163.53 to 209.66) | 159.48<br>(139.18 to 180.85) |
| Prevalence | Male | Uganda | 91.6<br>(81.03 to 103.15) | 0<br>(0 to 0) | 0<br>(0 to 0) | 331.14<br>(295.06 to 367.97) | 171.15<br>(151.54 to 192.35) |
| Prevalence | Female | Uganda | 157.76<br>(138.72 to 177.99) | 282.14<br>(247.99 to 318.06) | 282.14<br>(247.99 to 318.06) | 438.23<br>(387.43 to 490.67) | 270.89<br>(236.53 to 307.89) |

| | | | | | | | |
|---|---|---|---|---|---|---|---|
| Prevalence | Both | Uganda | 124.38 (111.03 to 138.93) | 0 (0 to 0) | 0 (0 to 0) | 227.15 (200.38 to 255.39) | 143.11 (125.25 to 162.9) |
| Prevalence | Male | Ukraine | 89.75 (79.66 to 101.18) | 331.08 (291.99 to 367.83) | 331.08 (291.99 to 367.83) | 0 (0 to 0) | 293.69 (260.71 to 327.45) |
| Prevalence | Female | Ukraine | 171.11 (151.12 to 192.76) | 338.6 (299.87 to 379.85) | 338.6 (299.87 to 379.85) | 0 (0 to 0) | 420.07 (370.02 to 469.7) |
| Prevalence | Both | Ukraine | 129.26 (115.59 to 144.52) | 229.94 (201.47 to 256.61) | 229.94 (201.47 to 256.61) | 0 (0 to 0) | 164.27 (143.58 to 184.38) |
| Prevalence | Male | United Arab Emirates | 84.13 (73.9 to 94.89) | 231.02 (205.11 to 258.47) | 231.02 (205.11 to 258.47) | 346.84 (308.04 to 387.4) | 219.36 (194.11 to 246.43) |
| Prevalence | Female | United Arab Emirates | 152.29 (133.8 to 170.57) | 0 (0 to 0) | 0 (0 to 0) | 449.82 (394.99 to 506.15) | 283.24 (249.17 to 320.65) |
| Prevalence | Both | United Arab Emirates | 117.05 (104.19 to 130.76) | 161.66 (142.01 to 182.15) | 161.66 (142.01 to 182.15) | 249.31 (219.62 to 280.59) | 152.95 (134.19 to 173.26) |
| Prevalence | Male | United Kingdom of Great Britain and Northern Ireland | 0 (0 to 0) | 0 (0 to 0) | 0 (0 to 0) | 294.42 (261.93 to 328.73) | 420.46 (381.54 to 460.1) |
| Prevalence | Female | United Kingdom of Great Britain and Northern Ireland | 0 (0 to 0) | 562.84 (511.52 to 616.28) | 562.84 (511.52 to 616.28) | 394.39 (347.4 to 444.33) | 690.74 (626.41 to 756.99) |
| Prevalence | Both | United Kingdom of Great Britain and Northern Ireland | 0 (0 to 0) | 0 (0 to 0) | 0 (0 to 0) | 205.86 (181.89 to 232.35) | 139.49 (126.64 to 152.64) |
| Prevalence | Male | United Republic of Tanzania | 94.42 (83.75 to 106) | 0 (0 to 0) | 0 (0 to 0) | 342.54 (304.56 to 382.88) | 234.97 (206.35 to 266.17) |
| Prevalence | Female | United Republic of Tanzania | 157.5 (138.96 to 177.67) | 316.6 (278.53 to 358.19) | 316.6 (278.53 to 358.19) | 449.82 (394.99 to 506.15) | 303.52 (264.36 to 346.74) |
| Prevalence | Both | United Republic of Tanzania | 126.54 (112.5 to 141.28) | 0 (0 to 0) | 0 (0 to 0) | 249.31 (219.62 to 280.59) | 158.65 (139.41 to 180.49) |
| Prevalence | Male | United States of America | 134.37 (123.23 to 146.36) | 356.9 (313.66 to 397.42) | 356.9 (313.66 to 397.42) | 301.98 (268.06 to 337.67) | 324.25 (286.14 to 364.99) |
| Prevalence | Female | United States of America | 265.6 (242.76 to 290.22) | 387.21 (340.62 to 436.84) | 387.21 (340.62 to 436.84) | 396.55 (348.57 to 447.16) | 479.89 (421.83 to 544.47) |
| Prevalence | Both | United States of America | 198.51 (182.07 to 216.49) | 214.27 (187.5 to 243.11) | 214.27 (187.5 to 243.11) | 206.83 (182.5 to 232.65) | 152.59 (133.17 to 173.27) |
| Prevalence | Male | United States Virgin Islands | 89.17 | 238.14 | 238.14 | 0 | 234.48 |

| | | | | | | | |
|---|---|---|---|---|---|---|---|
| | | | (78.54 to 100.33) | (211.25 to 266.64) | (211.25 to 266.64) | (0 to 0) | (205.94 to 265.62) |
| Prevalence | Female | United States Virgin Islands | 170.51<br>(150.08 to 191.81) | 349.03<br>(307.48 to 400.67) | 349.03<br>(307.48 to 400.67) | 0<br>(0 to 0) | 303.52<br>(264.36 to 346.74) |
| Prevalence | Both | United States Virgin Islands | 128.96<br>(114.44 to 144.17) | 162.86<br>(143.47 to 184.17) | 162.86<br>(143.47 to 184.17) | 0<br>(0 to 0) | 158.65<br>(139.41 to 180.49) |
| Prevalence | Male | Uruguay | 140.81<br>(124.13 to 158.33) | 258.69<br>(228 to 291.31) | 258.69<br>(228 to 291.31) | 234.53<br>(208.81 to 261.45) | 265.7<br>(233.94 to 297.68) |
| Prevalence | Female | Uruguay | 191.88<br>(168.76 to 214.53) | 363.66<br>(319.17 to 409.45) | 363.66<br>(319.17 to 409.45) | 351.28<br>(309.28 to 395.11) | 345.82<br>(302.1 to 391.48) |
| Prevalence | Both | Uruguay | 165.82<br>(147.25 to 184.76) | 187.63<br>(164.6 to 213.62) | 187.63<br>(164.6 to 213.62) | 185.99<br>(163.53 to 209.66) | 192.03<br>(168.22 to 217.97) |
| Prevalence | Male | Uzbekistan | 89.17<br>(78.54 to 100.33) | 242.73<br>(215.33 to 271.96) | 242.73<br>(215.33 to 271.96) | 0<br>(0 to 0) | 232.7<br>(204.44 to 263.53) |
| Prevalence | Female | Uzbekistan | 170.51<br>(150.08 to 191.81) | 0<br>(0 to 0) | 0<br>(0 to 0) | 0<br>(0 to 0) | 303.52<br>(264.36 to 346.74) |
| Prevalence | Both | Uzbekistan | 128.82<br>(114.33 to 144.01) | 165.2<br>(145.61 to 186.82) | 165.2<br>(145.61 to 186.82) | 0<br>(0 to 0) | 158.65<br>(139.41 to 180.49) |
| Prevalence | Male | Vanuatu | 108.19<br>(95.55 to 121.83) | 277.28<br>(246.65 to 309.2) | 277.28<br>(246.65 to 309.2) | 0<br>(0 to 0) | 289.5<br>(254.6 to 324.74) |
| Prevalence | Female | Vanuatu | 192.69<br>(168.27 to 216.69) | 415.05<br>(363.57 to 470.09) | 415.05<br>(363.57 to 470.09) | 0<br>(0 to 0) | 345.82<br>(302.1 to 391.48) |
| Prevalence | Both | Vanuatu | 149.68<br>(133.04 to 167.54) | 221.82<br>(195.5 to 249.48) | 221.82<br>(195.5 to 249.48) | 0<br>(0 to 0) | 228.88<br>(200.81 to 259.42) |
| Prevalence | Male | Yemen | 84.13<br>(73.9 to 94.89) | 213.28<br>(189.49 to 238.34) | 213.28<br>(189.49 to 238.34) | 288.83<br>(256.75 to 322.67) | 208.11<br>(183.49 to 234.17) |
| Prevalence | Female | Yemen | 152.29<br>(133.8 to 170.57) | 311.39<br>(273.7 to 351.19) | 311.39<br>(273.7 to 351.19) | 367.16<br>(324.07 to 412.97) | 270.89<br>(236.53 to 307.89) |
| Prevalence | Both | Yemen | 117.37<br>(104.49 to 131.11) | 148.43<br>(130.37 to 167.58) | 148.43<br>(130.37 to 167.58) | 200.77<br>(176.67 to 225.92) | 143.11<br>(125.25 to 162.9) |
| Prevalence | Male | Zambia | 91.6<br>(81.03 to 103.15) | 246.51<br>(217.31 to 278.59) | 246.51<br>(217.31 to 278.59) | 478.65<br>(436.88 to 521.47) | 216.74<br>(191.47 to 244.65) |
| Prevalence | Female | Zambia | 157.76<br>(138.72 to 177.99) | 290.2<br>(254.72 to 327.79) | 290.2<br>(254.72 to 327.79) | 647.24<br>(589.26 to 707.57) | 283.72<br>(247.73 to 323.19) |

| | | | | | | | |
|---|---|---|---|---|---|---|---|
| Prevalence | Both | Zambia | 125.28 (111.83 to 139.91) | 176.44 (153.51 to 202.04) | 176.44 (153.51 to 202.04) | 310.22 (284.14 to 336.24) | 152.91 (133.23 to 174.26) |
| Prevalence | Male | Zimbabwe | 88.23 (77.51 to 99.4) | 229.81 (204.1 to 257.11) | 229.81 (204.1 to 257.11) | 304.95 (270.94 to 340.65) | 226.24 (199.75 to 254.19) |
| Prevalence | Female | Zimbabwe | 160.01 (141.54 to 178.93) | 327.25 (287.41 to 368.24) | 327.25 (287.41 to 368.24) | 394.39 (347.4 to 444.33) | 285.8 (249.35 to 323.09) |
| Prevalence | Both | Zimbabwe | 124.52 (110.79 to 138.08) | 156.91 (137.83 to 176.93) | 156.91 (137.83 to 176.93) | 205.86 (181.89 to 232.35) | 151.72 (132.53 to 172.91) |

**Parentheses represent 95% uncertainty intervals (UIs). AA, alopecia areata.**

**Table S2 Changing trends of age-standardized prevalence of autoimmune diseases in childhood, adolescence, young and middle-age, middle-aged and elderly period at the global and regional levels in 1990-2021.**

| Age group | Measure | Sex | Location | Cause | AAPC (95% CI) | *P* |
|---|---|---|---|---|---|---|
| Childhood | Prevalence | Both | Afghanistan | AA | -0.17 (-0.39 to 0.04) | 0.109 |
| Childhood | Prevalence | Female | Afghanistan | AA | -0.03 (-0.21 to 0.15) | 0.734 |
| Childhood | Prevalence | Male | Afghanistan | AA | -0.08 (-0.24 to 0.08) | 0.331 |
| Childhood | Prevalence | Both | Afghanistan | IBD | 0.28 (-1.59 to 2.18) | 0.765 |
| Childhood | Prevalence | Female | Afghanistan | IBD | 0.91 (-1.07 to 2.94) | 0.355 |
| Childhood | Prevalence | Male | Afghanistan | IBD | 0.36 (-1.36 to 2.11) | 0.672 |
| Childhood | Prevalence | Both | Afghanistan | MS | 0.74 (-0.91 to 2.43) | 0.367 |
| Childhood | Prevalence | Female | Afghanistan | MS | 0.47 (-1.09 to 2.05) | 0.547 |
| Childhood | Prevalence | Male | Afghanistan | MS | 0.94 (-0.8 to 2.71) | 0.281 |
| Childhood | Prevalence | Both | Afghanistan | RA | 0.6 (-1 to 2.21) | 0.451 |
| Childhood | Prevalence | Female | Afghanistan | RA | 0.42 (-1.14 to 2.01) | 0.586 |
| Childhood | Prevalence | Male | Afghanistan | RA | 0.83 (-0.61 to 2.29) | 0.249 |
| Childhood | Prevalence | Both | Albania | IBD | 0.16 (-1.07 to 1.42) | 0.790 |
| Childhood | Prevalence | Female | Albania | IBD | -0.17 (-1.26 to 0.94) | 0.758 |
| Childhood | Prevalence | Male | Albania | IBD | 0.21 (-1.16 to 1.6) | 0.760 |

| | | | | | | |
|---|---|---|---|---|---|---|
| Childhood | Prevalence | Both | Albania | Psoriasis | -1.19 (-1.93 to -0.44) | 0.003 |
| Childhood | Prevalence | Female | Albania | Psoriasis | -1.03 (-1.54 to -0.51) | < 0.001 |
| Childhood | Prevalence | Male | Albania | Psoriasis | -1.15 (-2.09 to -0.19) | 0.020 |
| Childhood | Prevalence | Both | Albania | RHD | 0.02 (-0.35 to 0.39) | 0.929 |
| Childhood | Prevalence | Female | Albania | RHD | 0.13 (-0.26 to 0.53) | 0.496 |
| Childhood | Prevalence | Male | Albania | RHD | -0.05 (-0.39 to 0.29) | 0.751 |
| Childhood | Prevalence | Both | Albania | RA | 0.97 (-0.5 to 2.48) | 0.189 |
| Childhood | Prevalence | Female | Albania | RA | 1.18 (-0.35 to 2.75) | 0.126 |
| Childhood | Prevalence | Male | Albania | RA | 0.88 (-0.51 to 2.29) | 0.208 |
| Childhood | Prevalence | Both | Algeria | MS | 0.97 (-0.71 to 2.68) | 0.249 |
| Childhood | Prevalence | Female | Algeria | MS | 0.52 (-1.1 to 2.17) | 0.519 |
| Childhood | Prevalence | Male | Algeria | MS | 0.95 (-0.8 to 2.74) | 0.278 |
| Childhood | Prevalence | Both | Algeria | Psoriasis | -0.98 (-1.78 to -0.17) | 0.019 |
| Childhood | Prevalence | Female | Algeria | Psoriasis | -0.8 (-1.56 to -0.03) | 0.042 |
| Childhood | Prevalence | Male | Algeria | Psoriasis | -0.97 (-1.82 to -0.12) | 0.027 |
| Childhood | Prevalence | Both | Algeria | RHD | 0.34 (-0.18 to 0.87) | 0.194 |
| Childhood | Prevalence | Female | Algeria | RHD | 0.23 | 0.388 |

| | | | | | | |
|---|---|---|---|---|---|---|
| | | | | | (-0.31 to 0.79) | |
| Childhood | Prevalence | Male | Algeria | RHD | 0.3<br>(-0.2 to 0.8) | 0.227 |
| Childhood | Prevalence | Both | Algeria | RA | 1.19<br>(-0.36 to 2.77) | 0.127 |
| Childhood | Prevalence | Female | Algeria | RA | 0.84<br>(-0.75 to 2.46) | 0.291 |
| Childhood | Prevalence | Male | Algeria | RA | 1.1<br>(-0.35 to 2.57) | 0.133 |
| Childhood | Prevalence | Both | American Samoa | AA | 0.02<br>(-0.25 to 0.28) | 0.904 |
| Childhood | Prevalence | Female | American Samoa | AA | 0.04<br>(-0.23 to 0.31) | 0.762 |
| Childhood | Prevalence | Male | American Samoa | AA | -0.04<br>(-0.3 to 0.23) | 0.762 |
| Childhood | Prevalence | Both | American Samoa | IBD | -0.5<br>(-0.81 to -0.18) | 0.003 |
| Childhood | Prevalence | Female | American Samoa | IBD | -0.48<br>(-0.79 to -0.18) | 0.003 |
| Childhood | Prevalence | Male | American Samoa | IBD | -0.58<br>(-0.92 to -0.24) | 0.002 |
| Childhood | Prevalence | Both | American Samoa | Psoriasis | -0.41<br>(-0.98 to 0.16) | 0.149 |
| Childhood | Prevalence | Female | American Samoa | Psoriasis | -0.4<br>(-0.89 to 0.09) | 0.107 |
| Childhood | Prevalence | Male | American Samoa | Psoriasis | -0.39<br>(-1.04 to 0.26) | 0.226 |
| Childhood | Prevalence | Both | American Samoa | RHD | 0.14<br>(-0.02 to 0.3) | 0.087 |
| Childhood | Prevalence | Female | American Samoa | RHD | 0.15<br>(0.05 to 0.25) | 0.004 |
| Childhood | Prevalence | Male | American Samoa | RHD | 0.12<br>(-0.11 to 0.34) | 0.305 |

| | | | | | | |
|---|---|---|---|---|---|---|
| Childhood | Prevalence | Both | American Samoa | RA | 0.04<br>(-1.8 to 1.93) | 0.961 |
| Childhood | Prevalence | Female | American Samoa | RA | -0.42<br>(-2.22 to 1.41) | 0.639 |
| Childhood | Prevalence | Male | American Samoa | RA | -0.08<br>(-2.05 to 1.93) | 0.934 |
| Childhood | Prevalence | Both | Andorra | AA | 0.09<br>(-0.12 to 0.3) | 0.380 |
| Childhood | Prevalence | Female | Andorra | AA | -0.08<br>(-0.62 to 0.46) | 0.762 |
| Childhood | Prevalence | Male | Andorra | AA | -0.04<br>(-0.27 to 0.2) | 0.762 |
| Childhood | Prevalence | Both | Andorra | IBD | -0.04<br>(-1.54 to 1.48) | 0.957 |
| Childhood | Prevalence | Female | Andorra | IBD | 0.4<br>(-1.01 to 1.83) | 0.566 |
| Childhood | Prevalence | Male | Andorra | IBD | -0.08<br>(-1.69 to 1.56) | 0.921 |
| Childhood | Prevalence | Both | Andorra | MS | 0.38<br>(-1.25 to 2.03) | 0.641 |
| Childhood | Prevalence | Female | Andorra | MS | 0.76<br>(-0.88 to 2.43) | 0.354 |
| Childhood | Prevalence | Male | Andorra | MS | 0.17<br>(-1.51 to 1.88) | 0.835 |
| Childhood | Prevalence | Both | Andorra | RHD | -0.71<br>(-1.02 to -0.39) | < 0.001 |
| Childhood | Prevalence | Female | Andorra | RHD | -1.05<br>(-1.28 to -0.82) | < 0.001 |
| Childhood | Prevalence | Male | Andorra | RHD | -0.38<br>(-0.77 to 0) | 0.051 |
| Childhood | Prevalence | Both | Angola | AA | 0.04<br>(-0.19 to 0.28) | 0.716 |
| Childhood | Prevalence | Female | Angola | AA | 0.04 | 0.762 |

| | | | | | | |
|---|---|---|---|---|---|---|
| | | | | | (-0.21 to 0.28) | |
| Childhood | Prevalence | Male | Angola | AA | -0.04<br>(-0.27 to 0.2) | 0.762 |
| Childhood | Prevalence | Both | Angola | IBD | 0.75<br>(-0.62 to 2.15) | 0.271 |
| Childhood | Prevalence | Female | Angola | IBD | 0.4<br>(-0.85 to 1.67) | 0.519 |
| Childhood | Prevalence | Male | Angola | IBD | 0.88<br>(-0.62 to 2.41) | 0.241 |
| Childhood | Prevalence | Both | Angola | MS | 0.27<br>(-3.46 to 4.15) | 0.885 |
| Childhood | Prevalence | Female | Angola | MS | -0.86<br>(-4.57 to 3.01) | 0.649 |
| Childhood | Prevalence | Male | Angola | MS | 0.24<br>(-3.43 to 4.05) | 0.895 |
| Childhood | Prevalence | Both | Angola | Psoriasis | -1.22<br>(-2.48 to 0.06) | 0.062 |
| Childhood | Prevalence | Female | Angola | Psoriasis | -0.93<br>(-2.2 to 0.37) | 0.153 |
| Childhood | Prevalence | Male | Angola | Psoriasis | -1.22<br>(-2.49 to 0.06) | 0.061 |
| Childhood | Prevalence | Both | Angola | RHD | 0.16<br>(-0.64 to 0.97) | 0.684 |
| Childhood | Prevalence | Female | Angola | RHD | -0.03<br>(-0.76 to 0.72) | 0.944 |
| Childhood | Prevalence | Male | Angola | RHD | 0.11<br>(-0.77 to 1) | 0.794 |
| Childhood | Prevalence | Both | Antigua and Barbuda | AA | -0.16<br>(-1.41 to 1.11) | 0.795 |
| Childhood | Prevalence | Female | Antigua and Barbuda | AA | 0.23<br>(-1.02 to 1.5) | 0.710 |
| Childhood | Prevalence | Male | Antigua and Barbuda | AA | -0.16<br>(-1.38 to 1.09) | 0.797 |

| | | | | | | |
|---|---|---|---|---|---|---|
| Childhood | Prevalence | Both | Antigua and Barbuda | T1D | 0.8<br>(-0.68 to 2.29) | 0.280 |
| Childhood | Prevalence | Female | Antigua and Barbuda | T1D | 1.14<br>(-0.15 to 2.44) | 0.081 |
| Childhood | Prevalence | Male | Antigua and Barbuda | T1D | 0.83<br>(-0.88 to 2.56) | 0.331 |
| Childhood | Prevalence | Both | Antigua and Barbuda | IBD | 0.3<br>(-1.21 to 1.82) | 0.692 |
| Childhood | Prevalence | Female | Antigua and Barbuda | IBD | -0.02<br>(-1.4 to 1.38) | 0.977 |
| Childhood | Prevalence | Male | Antigua and Barbuda | IBD | 0.33<br>(-1.31 to 1.99) | 0.686 |
| Childhood | Prevalence | Both | Antigua and Barbuda | Psoriasis | -0.51<br>(-1.85 to 0.85) | 0.447 |
| Childhood | Prevalence | Female | Antigua and Barbuda | Psoriasis | -0.19<br>(-1.56 to 1.2) | 0.779 |
| Childhood | Prevalence | Male | Antigua and Barbuda | Psoriasis | -0.43<br>(-1.81 to 0.96) | 0.528 |
| Childhood | Prevalence | Both | Antigua and Barbuda | RHD | -0.04<br>(-0.1 to 0.03) | 0.228 |
| Childhood | Prevalence | Female | Antigua and Barbuda | RHD | -0.16<br>(-0.36 to 0.04) | 0.118 |
| Childhood | Prevalence | Male | Antigua and Barbuda | RHD | -0.06<br>(-0.15 to 0.02) | 0.154 |
| Childhood | Prevalence | Both | Antigua and Barbuda | RA | 1.06<br>(-0.44 to 2.58) | 0.161 |
| Childhood | Prevalence | Female | Antigua and Barbuda | RA | 0.62<br>(-0.9 to 2.16) | 0.413 |
| Childhood | Prevalence | Male | Antigua and Barbuda | RA | 1.1<br>(-0.36 to 2.58) | 0.135 |
| Childhood | Prevalence | Both | Argentina | IBD | 0.2<br>(-1.25 to 1.68) | 0.780 |
| Childhood | Prevalence | Female | Argentina | IBD | 0.52 | 0.400 |

| | | | | | | |
|---|---|---|---|---|---|---|
| | | | | | (-0.72 to 1.76) | |
| Childhood | Prevalence | Male | Argentina | IBD | 0.23<br>(-1.5 to 1.99) | 0.790 |
| Childhood | Prevalence | Both | Argentina | MS | -0.47<br>(-4.71 to 3.95) | 0.825 |
| Childhood | Prevalence | Female | Argentina | MS | -1.29<br>(-5.39 to 2.99) | 0.536 |
| Childhood | Prevalence | Male | Argentina | MS | -0.57<br>(-4.85 to 3.9) | 0.790 |
| Childhood | Prevalence | Both | Argentina | Psoriasis | -0.75<br>(-2.27 to 0.78) | 0.322 |
| Childhood | Prevalence | Female | Argentina | Psoriasis | -0.42<br>(-1.75 to 0.93) | 0.529 |
| Childhood | Prevalence | Male | Argentina | Psoriasis | -0.74<br>(-2.42 to 0.97) | 0.383 |
| Childhood | Prevalence | Both | Argentina | RA | 0.7<br>(-1.74 to 3.2) | 0.564 |
| Childhood | Prevalence | Female | Argentina | RA | 1.27<br>(-1.09 to 3.69) | 0.282 |
| Childhood | Prevalence | Male | Argentina | RA | 0.4<br>(-2.21 to 3.08) | 0.758 |
| Childhood | Prevalence | Both | Armenia | T1D | 0.79<br>(-1.23 to 2.84) | 0.434 |
| Childhood | Prevalence | Female | Armenia | T1D | 1.35<br>(-0.58 to 3.31) | 0.163 |
| Childhood | Prevalence | Male | Armenia | T1D | 0.77<br>(-1.36 to 2.96) | 0.467 |
| Childhood | Prevalence | Both | Armenia | MS | 0.59<br>(-2.07 to 3.32) | 0.656 |
| Childhood | Prevalence | Female | Armenia | MS | 0.11<br>(-2.42 to 2.69) | 0.933 |
| Childhood | Prevalence | Male | Armenia | MS | 0.23<br>(-2.66 to 3.21) | 0.874 |

| | | | | | | |
|---|---|---|---|---|---|---|
| Childhood | Prevalence | Both | Armenia | Psoriasis | -1.11<br>(-1.85 to -0.36) | 0.005 |
| Childhood | Prevalence | Female | Armenia | Psoriasis | -0.97<br>(-1.52 to -0.42) | 0.001 |
| Childhood | Prevalence | Male | Armenia | Psoriasis | -1.11<br>(-2.04 to -0.17) | 0.023 |
| Childhood | Prevalence | Both | Armenia | RHD | -0.11<br>(-0.41 to 0.19) | 0.455 |
| Childhood | Prevalence | Female | Armenia | RHD | -0.21<br>(-0.55 to 0.13) | 0.210 |
| Childhood | Prevalence | Male | Armenia | RHD | -0.13<br>(-0.37 to 0.11) | 0.268 |
| Childhood | Prevalence | Both | Armenia | RA | 0.98<br>(-0.6 to 2.58) | 0.215 |
| Childhood | Prevalence | Female | Armenia | RA | 1.4<br>(-0.18 to 3.01) | 0.080 |
| Childhood | Prevalence | Male | Armenia | RA | 0.79<br>(-0.77 to 2.38) | 0.309 |
| Childhood | Prevalence | Both | Australia | T1D | 0.95<br>(-0.71 to 2.64) | 0.252 |
| Childhood | Prevalence | Female | Australia | T1D | 1.39<br>(-0.24 to 3.05) | 0.092 |
| Childhood | Prevalence | Male | Australia | T1D | 0.98<br>(-0.74 to 2.74) | 0.255 |
| Childhood | Prevalence | Both | Australia | MS | 1.43<br>(-0.47 to 3.36) | 0.135 |
| Childhood | Prevalence | Female | Australia | MS | 1.72<br>(-0.09 to 3.56) | 0.062 |
| Childhood | Prevalence | Male | Australia | MS | 1.38<br>(-0.69 to 3.48) | 0.184 |
| Childhood | Prevalence | Both | Australia | RHD | -1.5<br>(-4.47 to 1.56) | 0.320 |
| Childhood | Prevalence | Female | Australia | RHD | -0.72 | 0.638 |

| | | | | | | |
|---|---|---|---|---|---|---|
| | | | | | (-3.78 to 2.43) | |
| Childhood | Prevalence | Male | Australia | RHD | -1.26<br>(-4.18 to 1.74) | 0.392 |
| Childhood | Prevalence | Both | Australia | RA | 1.55<br>(0.69 to 2.41) | 0.001 |
| Childhood | Prevalence | Female | Australia | RA | 1.56<br>(0.71 to 2.43) | 0.001 |
| Childhood | Prevalence | Male | Australia | RA | 1.58<br>(0.73 to 2.44) | 0.001 |
| Childhood | Prevalence | Both | Austria | AA | 0.12<br>(-1.18 to 1.44) | 0.848 |
| Childhood | Prevalence | Female | Austria | AA | -0.29<br>(-1.74 to 1.19) | 0.693 |
| Childhood | Prevalence | Male | Austria | AA | 0.11<br>(-0.95 to 1.19) | 0.829 |
| Childhood | Prevalence | Both | Austria | T1D | 2.68<br>(1.9 to 3.46) | < 0.001 |
| Childhood | Prevalence | Female | Austria | T1D | 2.89<br>(2.08 to 3.69) | < 0.001 |
| Childhood | Prevalence | Male | Austria | T1D | 2.52<br>(1.75 to 3.29) | < 0.001 |
| Childhood | Prevalence | Both | Austria | IBD | 0.15<br>(-1.39 to 1.71) | 0.845 |
| Childhood | Prevalence | Female | Austria | IBD | 0.47<br>(-0.86 to 1.81) | 0.479 |
| Childhood | Prevalence | Male | Austria | IBD | 0<br>(-1.71 to 1.74) | 0.999 |
| Childhood | Prevalence | Both | Austria | Psoriasis | -0.5<br>(-1.37 to 0.37) | 0.248 |
| Childhood | Prevalence | Female | Austria | Psoriasis | -0.31<br>(-1.04 to 0.42) | 0.389 |
| Childhood | Prevalence | Male | Austria | Psoriasis | -0.48<br>(-1.48 to 0.54) | 0.346 |

| | | | | | | |
|---|---|---|---|---|---|---|
| Childhood | Prevalence | Both | Austria | RHD | -3.03 (-4.44 to -1.61) | < 0.001 |
| Childhood | Prevalence | Female | Austria | RHD | -4.22 (-4.84 to -3.59) | < 0.001 |
| Childhood | Prevalence | Male | Austria | RHD | -2.99 (-3.54 to -2.44) | < 0.001 |
| Childhood | Prevalence | Both | Austria | RA | 0.15 (-1.13 to 1.44) | 0.812 |
| Childhood | Prevalence | Female | Austria | RA | 0.45 (-0.87 to 1.79) | 0.494 |
| Childhood | Prevalence | Male | Austria | RA | 0.21 (-1.03 to 1.46) | 0.738 |
| Childhood | Prevalence | Both | Azerbaijan | AA | -0.63 (-2.38 to 1.14) | 0.468 |
| Childhood | Prevalence | Female | Azerbaijan | AA | 0.07 (-1.6 to 1.78) | 0.929 |
| Childhood | Prevalence | Male | Azerbaijan | AA | -0.58 (-2.29 to 1.15) | 0.494 |
| Childhood | Prevalence | Both | Azerbaijan | IBD | -1.15 (-6.62 to 4.63) | 0.679 |
| Childhood | Prevalence | Female | Azerbaijan | IBD | 0.41 (-4.86 to 5.97) | 0.878 |
| Childhood | Prevalence | Male | Azerbaijan | IBD | -1.14 (-6.84 to 4.9) | 0.694 |
| Childhood | Prevalence | Both | Azerbaijan | MS | 0.29 (-1.43 to 2.04) | 0.737 |
| Childhood | Prevalence | Female | Azerbaijan | MS | 0.78 (-0.95 to 2.54) | 0.366 |
| Childhood | Prevalence | Male | Azerbaijan | MS | 0.26 (-1.45 to 1.99) | 0.762 |
| Childhood | Prevalence | Both | Azerbaijan | Psoriasis | -0.71 (-1.46 to 0.05) | 0.065 |
| Childhood | Prevalence | Female | Azerbaijan | Psoriasis | -0.85 | 0.004 |

| | | | | | | |
|---|---|---|---|---|---|---|
| | | | | | (-1.4 to -0.3) | |
| Childhood | Prevalence | Male | Azerbaijan | Psoriasis | -0.69<br>(-1.63 to 0.26) | 0.148 |
| Childhood | Prevalence | Both | Azerbaijan | RA | 0.75<br>(-0.77 to 2.3) | 0.324 |
| Childhood | Prevalence | Female | Azerbaijan | RA | 1.16<br>(-0.36 to 2.7) | 0.131 |
| Childhood | Prevalence | Male | Azerbaijan | RA | 0.79<br>(-0.77 to 2.36) | 0.311 |
| Childhood | Prevalence | Both | Bahamas | AA | -0.05<br>(-0.16 to 0.05) | 0.298 |
| Childhood | Prevalence | Female | Bahamas | AA | -0.03<br>(-0.12 to 0.06) | 0.536 |
| Childhood | Prevalence | Male | Bahamas | AA | -0.06<br>(-0.14 to 0.03) | 0.200 |
| Childhood | Prevalence | Both | Bahamas | T1D | 1.37<br>(0.07 to 2.69) | 0.040 |
| Childhood | Prevalence | Female | Bahamas | T1D | 0.99<br>(-0.15 to 2.14) | 0.086 |
| Childhood | Prevalence | Male | Bahamas | T1D | 1.45<br>(-0.03 to 2.94) | 0.054 |
| Childhood | Prevalence | Both | Bahamas | IBD | -0.24<br>(-1.71 to 1.25) | 0.742 |
| Childhood | Prevalence | Female | Bahamas | IBD | 0.22<br>(-1.17 to 1.64) | 0.747 |
| Childhood | Prevalence | Male | Bahamas | IBD | -0.32<br>(-1.89 to 1.27) | 0.679 |
| Childhood | Prevalence | Both | Bahamas | MS | 0.81<br>(-0.96 to 2.61) | 0.360 |
| Childhood | Prevalence | Female | Bahamas | MS | 0.42<br>(-1.33 to 2.21) | 0.626 |
| Childhood | Prevalence | Male | Bahamas | MS | 0.72<br>(-1.11 to 2.57) | 0.430 |

| | | | | | | |
|---|---|---|---|---|---|---|
| Childhood | Prevalence | Both | Bahamas | Psoriasis | -0.4 (-1.93 to 1.15) | 0.599 |
| Childhood | Prevalence | Female | Bahamas | Psoriasis | -0.73 (-2.24 to 0.8) | 0.337 |
| Childhood | Prevalence | Male | Bahamas | Psoriasis | -0.43 (-1.97 to 1.12) | 0.570 |
| Childhood | Prevalence | Both | Bahamas | RHD | 0.22 (-0.23 to 0.66) | 0.326 |
| Childhood | Prevalence | Female | Bahamas | RHD | 0.12 (-0.29 to 0.52) | 0.558 |
| Childhood | Prevalence | Male | Bahamas | RHD | 0.2 (-0.32 to 0.72) | 0.441 |
| Childhood | Prevalence | Both | Bahamas | RA | 0.04 (-2.88 to 3.04) | 0.981 |
| Childhood | Prevalence | Female | Bahamas | RA | 0.54 (-2.39 to 3.56) | 0.711 |
| Childhood | Prevalence | Male | Bahamas | RA | 0.17 (-2.75 to 3.17) | 0.909 |
| Childhood | Prevalence | Both | Bahrain | AA | 0.02 (-1.48 to 1.55) | 0.975 |
| Childhood | Prevalence | Female | Bahrain | AA | -0.56 (-2.26 to 1.17) | 0.509 |
| Childhood | Prevalence | Male | Bahrain | AA | 0.09 (-1.52 to 1.72) | 0.910 |
| Childhood | Prevalence | Both | Bahrain | IBD | 0.54 (0.08 to 1.02) | 0.024 |
| Childhood | Prevalence | Female | Bahrain | IBD | 0.05 (-0.45 to 0.56) | 0.834 |
| Childhood | Prevalence | Male | Bahrain | IBD | 0.81 (0.34 to 1.29) | 0.001 |
| Childhood | Prevalence | Both | Bahrain | MS | 0.73 (-0.89 to 2.37) | 0.367 |
| Childhood | Prevalence | Female | Bahrain | MS | 1.23 | 0.134 |

| | | | | | | |
|---|---|---|---|---|---|---|
| | | | | | (-0.4 to 2.89) | |
| Childhood | Prevalence | Male | Bahrain | MS | 0.83<br>(-0.89 to 2.58) | 0.335 |
| Childhood | Prevalence | Both | Bahrain | Psoriasis | -0.65<br>(-1.45 to 0.15) | 0.107 |
| Childhood | Prevalence | Female | Bahrain | Psoriasis | -0.84<br>(-1.61 to -0.07) | 0.034 |
| Childhood | Prevalence | Male | Bahrain | Psoriasis | -0.65<br>(-1.5 to 0.21) | 0.135 |
| Childhood | Prevalence | Both | Bahrain | RHD | -1.34<br>(-1.84 to -0.84) | < 0.001 |
| Childhood | Prevalence | Female | Bahrain | RHD | -1.28<br>(-1.92 to -0.63) | < 0.001 |
| Childhood | Prevalence | Male | Bahrain | RHD | -1.16<br>(-1.68 to -0.64) | < 0.001 |
| Childhood | Prevalence | Both | Bangladesh | AA | 0.05<br>(-2.68 to 2.86) | 0.972 |
| Childhood | Prevalence | Female | Bangladesh | AA | -0.75<br>(-3.45 to 2.02) | 0.578 |
| Childhood | Prevalence | Male | Bangladesh | AA | -0.05<br>(-2.8 to 2.77) | 0.970 |
| Childhood | Prevalence | Both | Bangladesh | IBD | 0.3<br>(-1.71 to 2.35) | 0.765 |
| Childhood | Prevalence | Female | Bangladesh | IBD | 0.96<br>(-1.17 to 3.15) | 0.367 |
| Childhood | Prevalence | Male | Bangladesh | IBD | 0.34<br>(-1.59 to 2.31) | 0.722 |
| Childhood | Prevalence | Both | Bangladesh | MS | 0.09<br>(-1.63 to 1.84) | 0.916 |
| Childhood | Prevalence | Female | Bangladesh | MS | 0.5<br>(-1.21 to 2.24) | 0.559 |
| Childhood | Prevalence | Male | Bangladesh | MS | -0.06<br>(-1.85 to 1.75) | 0.942 |

| | | | | | | |
|---|---|---|---|---|---|---|
| Childhood | Prevalence | Both | Bangladesh | Psoriasis | -1.03 (-2.55 to 0.52) | 0.183 |
| Childhood | Prevalence | Female | Bangladesh | Psoriasis | -0.68 (-2.21 to 0.87) | 0.374 |
| Childhood | Prevalence | Male | Bangladesh | Psoriasis | -1.01 (-2.55 to 0.55) | 0.193 |
| Childhood | Prevalence | Both | Bangladesh | RHD | 0.58 (-0.01 to 1.18) | 0.054 |
| Childhood | Prevalence | Female | Bangladesh | RHD | 0.49 (-0.03 to 1.02) | 0.065 |
| Childhood | Prevalence | Male | Bangladesh | RHD | 0.71 (0.13 to 1.3) | 0.019 |
| Childhood | Prevalence | Both | Bangladesh | RA | 1.2 (0.02 to 2.39) | 0.046 |
| Childhood | Prevalence | Female | Bangladesh | RA | 1.41 (0.2 to 2.63) | 0.024 |
| Childhood | Prevalence | Male | Bangladesh | RA | 1.06 (-0.08 to 2.21) | 0.067 |
| Childhood | Prevalence | Both | Barbados | AA | 0.04 (-0.22 to 0.3) | 0.753 |
| Childhood | Prevalence | Female | Barbados | AA | -0.04 (-0.29 to 0.21) | 0.762 |
| Childhood | Prevalence | Male | Barbados | AA | 0.04 (-0.21 to 0.28) | 0.762 |
| Childhood | Prevalence | Both | Barbados | MS | 0.79 (-1.12 to 2.73) | 0.408 |
| Childhood | Prevalence | Female | Barbados | MS | 1.3 (-0.62 to 3.25) | 0.178 |
| Childhood | Prevalence | Male | Barbados | MS | 0.41 (-1.47 to 2.34) | 0.660 |
| Childhood | Prevalence | Both | Barbados | RHD | 0 (-0.26 to 0.27) | 0.984 |
| Childhood | Prevalence | Female | Barbados | RHD | 0.1 | 0.499 |

| | | | | | | |
|---|---|---|---|---|---|---|
| | | | | | (-0.2 to 0.4) | |
| Childhood | Prevalence | Male | Barbados | RHD | -0.03<br>(-0.24 to 0.18) | 0.797 |
| Childhood | Prevalence | Both | Belarus | MS | -1.04<br>(-6.24 to 4.45) | 0.694 |
| Childhood | Prevalence | Female | Belarus | MS | 0.39<br>(-4.74 to 5.8) | 0.879 |
| Childhood | Prevalence | Male | Belarus | MS | -1.39<br>(-6.66 to 4.18) | 0.607 |
| Childhood | Prevalence | Both | Belarus | RA | 1.34<br>(-0.87 to 3.6) | 0.226 |
| Childhood | Prevalence | Female | Belarus | RA | 0.88<br>(-1.12 to 2.92) | 0.379 |
| Childhood | Prevalence | Male | Belarus | RA | 1.12<br>(-1.3 to 3.61) | 0.354 |
| Childhood | Prevalence | Both | Belgium | T1D | 1.82<br>(0.84 to 2.8) | 0.001 |
| Childhood | Prevalence | Female | Belgium | T1D | 1.99<br>(1.07 to 2.91) | < 0.001 |
| Childhood | Prevalence | Male | Belgium | T1D | 1.87<br>(0.83 to 2.92) | 0.001 |
| Childhood | Prevalence | Both | Belgium | IBD | 1.66<br>(1.05 to 2.27) | < 0.001 |
| Childhood | Prevalence | Female | Belgium | IBD | 1.86<br>(1.09 to 2.64) | < 0.001 |
| Childhood | Prevalence | Male | Belgium | IBD | 1.43<br>(0.81 to 2.05) | < 0.001 |
| Childhood | Prevalence | Both | Belgium | RHD | -0.13<br>(-5.31 to 5.33) | 0.960 |
| Childhood | Prevalence | Female | Belgium | RHD | 0.93<br>(-4.5 to 6.66) | 0.735 |
| Childhood | Prevalence | Male | Belgium | RHD | -0.55<br>(-5.48 to 4.64) | 0.826 |

| | | | | | | |
|---|---|---|---|---|---|---|
| Childhood | Prevalence | Both | Belgium | RA | 0.28<br>(-0.92 to 1.49) | 0.639 |
| Childhood | Prevalence | Female | Belgium | RA | 0.59<br>(-0.66 to 1.85) | 0.347 |
| Childhood | Prevalence | Male | Belgium | RA | 0.35<br>(-0.77 to 1.49) | 0.531 |
| Childhood | Prevalence | Both | Belize | AA | 0.01<br>(-0.03 to 0.06) | 0.545 |
| Childhood | Prevalence | Female | Belize | AA | -0.02<br>(-0.08 to 0.04) | 0.459 |
| Childhood | Prevalence | Male | Belize | AA | -0.03<br>(-0.09 to 0.03) | 0.271 |
| Childhood | Prevalence | Both | Belize | IBD | -0.24<br>(-2.66 to 2.24) | 0.843 |
| Childhood | Prevalence | Female | Belize | IBD | 0.31<br>(-1.99 to 2.66) | 0.789 |
| Childhood | Prevalence | Male | Belize | IBD | -0.08<br>(-2.65 to 2.57) | 0.953 |
| Childhood | Prevalence | Both | Belize | MS | 0.71<br>(-1.06 to 2.52) | 0.419 |
| Childhood | Prevalence | Female | Belize | MS | 0.18<br>(-1.62 to 2) | 0.844 |
| Childhood | Prevalence | Male | Belize | MS | 0.59<br>(-1.21 to 2.43) | 0.508 |
| Childhood | Prevalence | Both | Belize | RHD | 0.1<br>(-0.09 to 0.29) | 0.291 |
| Childhood | Prevalence | Female | Belize | RHD | 0.04<br>(-0.2 to 0.29) | 0.717 |
| Childhood | Prevalence | Male | Belize | RHD | 0.03<br>(-0.11 to 0.18) | 0.640 |
| Childhood | Prevalence | Both | Belize | RA | 1.08<br>(-0.46 to 2.63) | 0.162 |
| Childhood | Prevalence | Female | Belize | RA | 0.5 | 0.518 |

| | | | | | | |
|---|---|---|---|---|---|---|
| | | | | | (-1.06 to 2.1) | |
| Childhood | Prevalence | Male | Belize | RA | 1.12<br>(-0.31 to 2.56) | 0.120 |
| Childhood | Prevalence | Both | Benin | IBD | 0.63<br>(-2.63 to 4) | 0.697 |
| Childhood | Prevalence | Female | Benin | IBD | -0.25<br>(-3.35 to 2.96) | 0.875 |
| Childhood | Prevalence | Male | Benin | IBD | 0.6<br>(-2.83 to 4.15) | 0.727 |
| Childhood | Prevalence | Both | Benin | MS | 0.62<br>(-0.28 to 1.53) | 0.171 |
| Childhood | Prevalence | Female | Benin | MS | 0.53<br>(-0.4 to 1.47) | 0.255 |
| Childhood | Prevalence | Male | Benin | MS | 0.42<br>(-0.42 to 1.28) | 0.315 |
| Childhood | Prevalence | Both | Benin | Psoriasis | -0.68<br>(-1.77 to 0.42) | 0.215 |
| Childhood | Prevalence | Female | Benin | Psoriasis | -0.98<br>(-2.07 to 0.13) | 0.080 |
| Childhood | Prevalence | Male | Benin | Psoriasis | -0.64<br>(-1.74 to 0.47) | 0.245 |
| Childhood | Prevalence | Both | Benin | RHD | 0.36<br>(-0.11 to 0.83) | 0.126 |
| Childhood | Prevalence | Female | Benin | RHD | 0.26<br>(-0.15 to 0.68) | 0.206 |
| Childhood | Prevalence | Male | Benin | RHD | 0.39<br>(-0.16 to 0.95) | 0.160 |
| Childhood | Prevalence | Both | Bermuda | IBD | 1.43<br>(-1.28 to 4.21) | 0.292 |
| Childhood | Prevalence | Female | Bermuda | IBD | 0.73<br>(-1.76 to 3.28) | 0.558 |
| Childhood | Prevalence | Male | Bermuda | IBD | 1.4<br>(-1.52 to 4.41) | 0.339 |

| | | | | | | |
|---|---|---|---|---|---|---|
| Childhood | Prevalence | Both | Bermuda | MS | 0.12<br>(-1.61 to 1.88) | 0.888 |
| Childhood | Prevalence | Female | Bermuda | MS | 0.65<br>(-1.08 to 2.41) | 0.448 |
| Childhood | Prevalence | Male | Bermuda | MS | 0.08<br>(-1.69 to 1.87) | 0.929 |
| Childhood | Prevalence | Both | Bermuda | RHD | -2.65<br>(-5.53 to 0.31) | 0.077 |
| Childhood | Prevalence | Female | Bermuda | RHD | -2.06<br>(-5.06 to 1.02) | 0.180 |
| Childhood | Prevalence | Male | Bermuda | RHD | -2.39<br>(-5.2 to 0.51) | 0.102 |
| Childhood | Prevalence | Both | Bhutan | AA | -0.05<br>(-0.3 to 0.21) | 0.705 |
| Childhood | Prevalence | Female | Bhutan | AA | 0.04<br>(-0.21 to 0.29) | 0.762 |
| Childhood | Prevalence | Male | Bhutan | AA | -0.04<br>(-0.3 to 0.22) | 0.762 |
| Childhood | Prevalence | Both | Bhutan | MS | 0.54<br>(-1.19 to 2.29) | 0.530 |
| Childhood | Prevalence | Female | Bhutan | MS | 0.12<br>(-1.58 to 1.85) | 0.883 |
| Childhood | Prevalence | Male | Bhutan | MS | 0.58<br>(-1.19 to 2.38) | 0.511 |
| Childhood | Prevalence | Both | Bhutan | RHD | -0.01<br>(-0.43 to 0.4) | 0.943 |
| Childhood | Prevalence | Female | Bhutan | RHD | 0.12<br>(-0.3 to 0.54) | 0.563 |
| Childhood | Prevalence | Male | Bhutan | RHD | -0.03<br>(-0.44 to 0.38) | 0.876 |
| Childhood | Prevalence | Both | Bolivia (Plurinational State of) | AA | -0.06<br>(-0.31 to 0.19) | 0.628 |
| Childhood | Prevalence | Female | Bolivia (Plurinational State of) | AA | 0.04 | 0.762 |

| | | | | | | |
|---|---|---|---|---|---|---|
| | | | | | (-0.21 to 0.29) | |
| Childhood | Prevalence | Male | Bolivia (Plurinational State of) | AA | -0.04<br>(-0.28 to 0.21) | 0.762 |
| Childhood | Prevalence | Both | Bolivia (Plurinational State of) | T1D | 0<br>(-2.28 to 2.33) | 0.998 |
| Childhood | Prevalence | Female | Bolivia (Plurinational State of) | T1D | 0.54<br>(-1.69 to 2.82) | 0.626 |
| Childhood | Prevalence | Male | Bolivia (Plurinational State of) | T1D | 0.06<br>(-2.29 to 2.48) | 0.956 |
| Childhood | Prevalence | Both | Bolivia (Plurinational State of) | MS | 0.78<br>(-1.36 to 2.96) | 0.464 |
| Childhood | Prevalence | Female | Bolivia (Plurinational State of) | MS | 0.42<br>(-1.73 to 2.61) | 0.696 |
| Childhood | Prevalence | Male | Bolivia (Plurinational State of) | MS | 0.6<br>(-1.44 to 2.68) | 0.552 |
| Childhood | Prevalence | Both | Bolivia (Plurinational State of) | Psoriasis | -0.67<br>(-1.37 to 0.03) | 0.061 |
| Childhood | Prevalence | Female | Bolivia (Plurinational State of) | Psoriasis | -0.51<br>(-1.21 to 0.2) | 0.152 |
| Childhood | Prevalence | Male | Bolivia (Plurinational State of) | Psoriasis | -0.65<br>(-1.35 to 0.05) | 0.069 |
| Childhood | Prevalence | Both | Bolivia (Plurinational State of) | RHD | 0.25<br>(-0.67 to 1.18) | 0.584 |
| Childhood | Prevalence | Female | Bolivia (Plurinational State of) | RHD | 0.03<br>(-0.91 to 0.98) | 0.951 |
| Childhood | Prevalence | Male | Bolivia (Plurinational State of) | RHD | 0.19<br>(-0.71 to 1.09) | 0.674 |
| Childhood | Prevalence | Both | Bosnia and Herzegovina | AA | -0.02<br>(-0.12 to 0.08) | 0.692 |
| Childhood | Prevalence | Female | Bosnia and Herzegovina | AA | -0.02<br>(-0.18 to 0.14) | 0.801 |
| Childhood | Prevalence | Male | Bosnia and Herzegovina | AA | 0.03<br>(-0.13 to 0.19) | 0.733 |

| | | | | | | |
|---|---|---|---|---|---|---|
| Childhood | Prevalence | Both | Bosnia and Herzegovina | IBD | 0.17 (-1.54 to 1.92) | 0.839 |
| Childhood | Prevalence | Female | Bosnia and Herzegovina | IBD | 0.59 (-0.95 to 2.15) | 0.444 |
| Childhood | Prevalence | Male | Bosnia and Herzegovina | IBD | 0.14 (-1.73 to 2.06) | 0.877 |
| Childhood | Prevalence | Both | Bosnia and Herzegovina | MS | 0.09 (-1.6 to 1.81) | 0.915 |
| Childhood | Prevalence | Female | Bosnia and Herzegovina | MS | 0.53 (-1.15 to 2.24) | 0.525 |
| Childhood | Prevalence | Male | Bosnia and Herzegovina | MS | -0.09 (-1.8 to 1.64) | 0.911 |
| Childhood | Prevalence | Both | Bosnia and Herzegovina | RA | 1.19 (-0.24 to 2.64) | 0.099 |
| Childhood | Prevalence | Female | Bosnia and Herzegovina | RA | 0.77 (-0.74 to 2.31) | 0.306 |
| Childhood | Prevalence | Male | Bosnia and Herzegovina | RA | 1.32 (0.06 to 2.59) | 0.040 |
| Childhood | Prevalence | Both | Botswana | IBD | 0.33 (-1.43 to 2.12) | 0.707 |
| Childhood | Prevalence | Female | Botswana | IBD | 0.77 (-1.14 to 2.72) | 0.420 |
| Childhood | Prevalence | Male | Botswana | IBD | 0.51 (-1.08 to 2.12) | 0.522 |
| Childhood | Prevalence | Both | Botswana | MS | -0.03 (-1.73 to 1.7) | 0.971 |
| Childhood | Prevalence | Female | Botswana | MS | 0.5 (-1.18 to 2.21) | 0.547 |
| Childhood | Prevalence | Male | Botswana | MS | -0.04 (-1.8 to 1.75) | 0.961 |
| Childhood | Prevalence | Both | Botswana | RHD | 0.03 (-0.07 to 0.13) | 0.569 |
| Childhood | Prevalence | Female | Botswana | RHD | 0.01 | 0.785 |

| | | | | | | |
|---|---|---|---|---|---|---|
| | | | | | (-0.08 to 0.11) | |
| Childhood | Prevalence | Male | Botswana | RHD | 0.03<br>(-0.09 to 0.15) | 0.661 |
| Childhood | Prevalence | Both | Brazil | IBD | -0.68<br>(-2.42 to 1.09) | 0.435 |
| Childhood | Prevalence | Female | Brazil | IBD | -1.11<br>(-2.77 to 0.57) | 0.186 |
| Childhood | Prevalence | Male | Brazil | IBD | -0.7<br>(-2.52 to 1.15) | 0.443 |
| Childhood | Prevalence | Both | Brazil | MS | 0.41<br>(-1.4 to 2.26) | 0.647 |
| Childhood | Prevalence | Female | Brazil | MS | 0.63<br>(-1.2 to 2.49) | 0.489 |
| Childhood | Prevalence | Male | Brazil | MS | 0.89<br>(-0.95 to 2.76) | 0.333 |
| Childhood | Prevalence | Both | Brazil | Psoriasis | -0.37<br>(-1.71 to 0.98) | 0.575 |
| Childhood | Prevalence | Female | Brazil | Psoriasis | 0<br>(-1.33 to 1.36) | 0.994 |
| Childhood | Prevalence | Male | Brazil | Psoriasis | -0.37<br>(-1.72 to 0.99) | 0.581 |
| Childhood | Prevalence | Both | Brazil | RHD | 0.08<br>(-0.11 to 0.27) | 0.395 |
| Childhood | Prevalence | Female | Brazil | RHD | 0.18<br>(-0.07 to 0.42) | 0.148 |
| Childhood | Prevalence | Male | Brazil | RHD | 0.02<br>(-0.08 to 0.12) | 0.694 |
| Childhood | Prevalence | Both | Brazil | RA | -0.21<br>(-3.04 to 2.7) | 0.883 |
| Childhood | Prevalence | Female | Brazil | RA | 0.53<br>(-2.28 to 3.41) | 0.707 |
| Childhood | Prevalence | Male | Brazil | RA | -0.16<br>(-2.93 to 2.7) | 0.910 |

| | | | | | | |
|---|---|---|---|---|---|---|
| Childhood | Prevalence | Both | Brunei Darussalam | AA | -0.72<br>(-3.43 to 2.05) | 0.593 |
| Childhood | Prevalence | Female | Brunei Darussalam | AA | 0.09<br>(-2.92 to 3.19) | 0.954 |
| Childhood | Prevalence | Male | Brunei Darussalam | AA | -0.62<br>(-2.93 to 1.75) | 0.593 |
| Childhood | Prevalence | Both | Brunei Darussalam | T1D | 0<br>(-0.4 to 0.4) | 0.991 |
| Childhood | Prevalence | Female | Brunei Darussalam | T1D | 0.12<br>(-0.27 to 0.51) | 0.540 |
| Childhood | Prevalence | Male | Brunei Darussalam | T1D | 0<br>(-0.42 to 0.41) | 0.992 |
| Childhood | Prevalence | Both | Brunei Darussalam | IBD | -0.61<br>(-4.87 to 3.84) | 0.776 |
| Childhood | Prevalence | Female | Brunei Darussalam | IBD | 0.54<br>(-3.69 to 4.97) | 0.798 |
| Childhood | Prevalence | Male | Brunei Darussalam | IBD | -0.77<br>(-5.11 to 3.77) | 0.727 |
| Childhood | Prevalence | Both | Brunei Darussalam | Psoriasis | -0.39<br>(-1.2 to 0.42) | 0.332 |
| Childhood | Prevalence | Female | Brunei Darussalam | Psoriasis | -0.28<br>(-0.95 to 0.39) | 0.395 |
| Childhood | Prevalence | Male | Brunei Darussalam | Psoriasis | -0.32<br>(-1.25 to 0.62) | 0.489 |
| Childhood | Prevalence | Both | Brunei Darussalam | RHD | -1.04<br>(-5.86 to 4.03) | 0.672 |
| Childhood | Prevalence | Female | Brunei Darussalam | RHD | -2.4<br>(-7.32 to 2.79) | 0.345 |
| Childhood | Prevalence | Male | Brunei Darussalam | RHD | -1.01<br>(-5.47 to 3.66) | 0.657 |
| Childhood | Prevalence | Both | Brunei Darussalam | RA | 0.28<br>(-2.18 to 2.8) | 0.820 |
| Childhood | Prevalence | Female | Brunei Darussalam | RA | -0.43 | 0.720 |

| | | | | | | |
|---|---|---|---|---|---|---|
| | | | | | (-2.85 to 2.04) | |
| Childhood | Prevalence | Male | Brunei Darussalam | RA | 0.38<br>(-2.05 to 2.87) | 0.754 |
| Childhood | Prevalence | Both | Bulgaria | AA | 0.02<br>(-0.07 to 0.1) | 0.712 |
| Childhood | Prevalence | Female | Bulgaria | AA | 0.02<br>(-0.04 to 0.08) | 0.472 |
| Childhood | Prevalence | Male | Bulgaria | AA | 0.05<br>(-0.01 to 0.12) | 0.100 |
| Childhood | Prevalence | Both | Bulgaria | Psoriasis | -1.07<br>(-2 to -0.12) | 0.029 |
| Childhood | Prevalence | Female | Bulgaria | Psoriasis | -0.93<br>(-1.85 to -0.01) | 0.049 |
| Childhood | Prevalence | Male | Bulgaria | Psoriasis | -1.01<br>(-1.98 to -0.03) | 0.044 |
| Childhood | Prevalence | Both | Bulgaria | RHD | -2.87<br>(-3.9 to -1.84) | < 0.001 |
| Childhood | Prevalence | Female | Bulgaria | RHD | -2.81<br>(-3.8 to -1.82) | < 0.001 |
| Childhood | Prevalence | Male | Bulgaria | RHD | -2.63<br>(-3.72 to -1.54) | < 0.001 |
| Childhood | Prevalence | Both | Burkina Faso | AA | 0.01<br>(-0.23 to 0.26) | 0.908 |
| Childhood | Prevalence | Female | Burkina Faso | AA | -0.04<br>(-0.28 to 0.21) | 0.762 |
| Childhood | Prevalence | Male | Burkina Faso | AA | 0.04<br>(-0.2 to 0.27) | 0.762 |
| Childhood | Prevalence | Both | Burkina Faso | IBD | 0.47<br>(-1.38 to 2.35) | 0.611 |
| Childhood | Prevalence | Female | Burkina Faso | IBD | 0.99<br>(-0.95 to 2.97) | 0.306 |
| Childhood | Prevalence | Male | Burkina Faso | IBD | 0.44<br>(-1.27 to 2.18) | 0.607 |

| | | | | | | |
|---|---|---|---|---|---|---|
| Childhood | Prevalence | Both | Burkina Faso | MS | 0.42<br>(-1.35 to 2.22) | 0.632 |
| Childhood | Prevalence | Female | Burkina Faso | MS | -0.04<br>(-1.79 to 1.75) | 0.967 |
| Childhood | Prevalence | Male | Burkina Faso | MS | 0.51<br>(-1.29 to 2.34) | 0.572 |
| Childhood | Prevalence | Both | Burkina Faso | Psoriasis | -0.89<br>(-2.53 to 0.77) | 0.281 |
| Childhood | Prevalence | Female | Burkina Faso | Psoriasis | -0.54<br>(-2.18 to 1.13) | 0.511 |
| Childhood | Prevalence | Male | Burkina Faso | Psoriasis | -0.81<br>(-2.46 to 0.87) | 0.329 |
| Childhood | Prevalence | Both | Burkina Faso | RHD | 0.04<br>(-0.02 to 0.11) | 0.188 |
| Childhood | Prevalence | Female | Burkina Faso | RHD | 0.09<br>(0 to 0.17) | 0.044 |
| Childhood | Prevalence | Male | Burkina Faso | RHD | 0.01<br>(-0.03 to 0.06) | 0.470 |
| Childhood | Prevalence | Both | Burkina Faso | RA | 0.3<br>(-1.94 to 2.59) | 0.788 |
| Childhood | Prevalence | Female | Burkina Faso | RA | 0.91<br>(-1.17 to 3.04) | 0.380 |
| Childhood | Prevalence | Male | Burkina Faso | RA | 0.26<br>(-2.24 to 2.82) | 0.835 |
| Childhood | Prevalence | Both | Burundi | AA | -0.09<br>(-1.06 to 0.89) | 0.850 |
| Childhood | Prevalence | Female | Burundi | AA | -0.31<br>(-1.3 to 0.68) | 0.522 |
| Childhood | Prevalence | Male | Burundi | AA | 0.01<br>(-0.94 to 0.96) | 0.986 |
| Childhood | Prevalence | Both | Burundi | Psoriasis | -0.22<br>(-1.55 to 1.13) | 0.740 |
| Childhood | Prevalence | Female | Burundi | Psoriasis | 0.1 | 0.887 |

| | | | | | | |
|---|---|---|---|---|---|---|
| | | | | | (-1.25 to 1.46) | |
| Childhood | Prevalence | Male | Burundi | Psoriasis | -0.14<br>(-1.48 to 1.22) | 0.836 |
| Childhood | Prevalence | Both | Burundi | RHD | -0.11<br>(-2.38 to 2.22) | 0.924 |
| Childhood | Prevalence | Female | Burundi | RHD | 0.42<br>(-1.83 to 2.72) | 0.706 |
| Childhood | Prevalence | Male | Burundi | RHD | -0.17<br>(-2.52 to 2.23) | 0.882 |
| Childhood | Prevalence | Both | Burundi | RA | 0.95<br>(-0.35 to 2.26) | 0.145 |
| Childhood | Prevalence | Female | Burundi | RA | 0.47<br>(-0.76 to 1.71) | 0.446 |
| Childhood | Prevalence | Male | Burundi | RA | 0.89<br>(-0.44 to 2.23) | 0.182 |
| Childhood | Prevalence | Both | Cabo Verde | IBD | 1.13<br>(-0.87 to 3.16) | 0.258 |
| Childhood | Prevalence | Female | Cabo Verde | IBD | 0.68<br>(-1.15 to 2.55) | 0.456 |
| Childhood | Prevalence | Male | Cabo Verde | IBD | 1.19<br>(-1 to 3.42) | 0.278 |
| Childhood | Prevalence | Both | Cabo Verde | MS | 0.87<br>(0.16 to 1.58) | 0.018 |
| Childhood | Prevalence | Female | Cabo Verde | MS | 1.03<br>(0.24 to 1.82) | 0.012 |
| Childhood | Prevalence | Male | Cabo Verde | MS | 1.13<br>(0.44 to 1.82) | 0.002 |
| Childhood | Prevalence | Both | Cabo Verde | RHD | 0.05<br>(-0.12 to 0.23) | 0.536 |
| Childhood | Prevalence | Female | Cabo Verde | RHD | 0.09<br>(-0.17 to 0.35) | 0.471 |
| Childhood | Prevalence | Male | Cabo Verde | RHD | 0.06<br>(0 to 0.12) | 0.041 |

| | | | | | | |
|---|---|---|---|---|---|---|
| Childhood | Prevalence | Both | Cambodia | AA | -0.11 (-0.39 to 0.18) | 0.458 |
| Childhood | Prevalence | Female | Cambodia | AA | 0.04 (-0.23 to 0.31) | 0.762 |
| Childhood | Prevalence | Male | Cambodia | AA | -0.04 (-0.29 to 0.21) | 0.762 |
| Childhood | Prevalence | Both | Cambodia | IBD | 0.22 (-1.76 to 2.24) | 0.824 |
| Childhood | Prevalence | Female | Cambodia | IBD | 0.72 (-1.26 to 2.75) | 0.464 |
| Childhood | Prevalence | Male | Cambodia | IBD | 0.36 (-1.6 to 2.37) | 0.710 |
| Childhood | Prevalence | Both | Cambodia | Psoriasis | -1.03 (-1.92 to -0.13) | 0.026 |
| Childhood | Prevalence | Female | Cambodia | Psoriasis | -0.72 (-1.4 to -0.03) | 0.041 |
| Childhood | Prevalence | Male | Cambodia | Psoriasis | -1 (-2.09 to 0.1) | 0.072 |
| Childhood | Prevalence | Both | Cambodia | RHD | 0.04 (-0.16 to 0.25) | 0.674 |
| Childhood | Prevalence | Female | Cambodia | RHD | 0.07 (-0.15 to 0.29) | 0.531 |
| Childhood | Prevalence | Male | Cambodia | RHD | 0.04 (-0.18 to 0.26) | 0.695 |
| Childhood | Prevalence | Both | Cambodia | RA | 0.24 (-2.29 to 2.83) | 0.850 |
| Childhood | Prevalence | Female | Cambodia | RA | -0.24 (-2.63 to 2.21) | 0.841 |
| Childhood | Prevalence | Male | Cambodia | RA | 0.15 (-2.46 to 2.84) | 0.907 |
| Childhood | Prevalence | Both | Cameroon | AA | 0.02 (-0.21 to 0.24) | 0.886 |
| Childhood | Prevalence | Female | Cameroon | AA | -0.04 | 0.762 |

| | | | | | | |
|---|---|---|---|---|---|---|
| | | | | | (-0.28 to 0.21) | |
| Childhood | Prevalence | Male | Cameroon | AA | 0.04<br>(-0.2 to 0.27) | 0.762 |
| Childhood | Prevalence | Both | Cameroon | Psoriasis | -0.76<br>(-1.48 to -0.04) | 0.041 |
| Childhood | Prevalence | Female | Cameroon | Psoriasis | -0.64<br>(-1.36 to 0.08) | 0.080 |
| Childhood | Prevalence | Male | Cameroon | Psoriasis | -0.67<br>(-1.4 to 0.06) | 0.070 |
| Childhood | Prevalence | Both | Cameroon | RHD | 0.23<br>(0.14 to 0.33) | < 0.001 |
| Childhood | Prevalence | Female | Cameroon | RHD | 0.28<br>(0.16 to 0.41) | < 0.001 |
| Childhood | Prevalence | Male | Cameroon | RHD | 0.22<br>(0.16 to 0.28) | < 0.001 |
| Childhood | Prevalence | Both | Canada | AA | 0.07<br>(-0.27 to 0.42) | 0.665 |
| Childhood | Prevalence | Female | Canada | AA | -0.08<br>(-0.63 to 0.47) | 0.762 |
| Childhood | Prevalence | Male | Canada | AA | -0.04<br>(-0.32 to 0.24) | 0.762 |
| Childhood | Prevalence | Both | Canada | IBD | 0.18<br>(-0.82 to 1.18) | 0.721 |
| Childhood | Prevalence | Female | Canada | IBD | -0.01<br>(-0.89 to 0.88) | 0.981 |
| Childhood | Prevalence | Male | Canada | IBD | 0.14<br>(-0.94 to 1.24) | 0.788 |
| Childhood | Prevalence | Both | Canada | MS | 0.6<br>(-1.09 to 2.31) | 0.478 |
| Childhood | Prevalence | Female | Canada | MS | 0.48<br>(-1.17 to 2.17) | 0.556 |
| Childhood | Prevalence | Male | Canada | MS | 0.46<br>(-1.35 to 2.3) | 0.612 |

| | | | | | | |
|---|---|---|---|---|---|---|
| Childhood | Prevalence | Both | Canada | RHD | -0.77<br>(-1.25 to -0.29) | 0.003 |
| Childhood | Prevalence | Female | Canada | RHD | -0.85<br>(-1.42 to -0.29) | 0.005 |
| Childhood | Prevalence | Male | Canada | RHD | -0.47<br>(-0.88 to -0.07) | 0.024 |
| Childhood | Prevalence | Both | Canada | RA | 0.58<br>(-2.68 to 3.94) | 0.722 |
| Childhood | Prevalence | Female | Canada | RA | 0.76<br>(-2.69 to 4.32) | 0.661 |
| Childhood | Prevalence | Male | Canada | RA | 0.49<br>(-2.43 to 3.49) | 0.739 |
| Childhood | Prevalence | Both | Central African Republic | AA | -0.08<br>(-0.3 to 0.15) | 0.484 |
| Childhood | Prevalence | Female | Central African Republic | AA | 0.04<br>(-0.21 to 0.28) | 0.762 |
| Childhood | Prevalence | Male | Central African Republic | AA | -0.04<br>(-0.27 to 0.2) | 0.762 |
| Childhood | Prevalence | Both | Central African Republic | MS | 0.26<br>(-1.47 to 2.01) | 0.765 |
| Childhood | Prevalence | Female | Central African Republic | MS | -0.19<br>(-1.9 to 1.56) | 0.827 |
| Childhood | Prevalence | Male | Central African Republic | MS | 0.45<br>(-1.32 to 2.25) | 0.609 |
| Childhood | Prevalence | Both | Central African Republic | RA | -0.74<br>(-4.15 to 2.8) | 0.669 |
| Childhood | Prevalence | Female | Central African Republic | RA | 0.11<br>(-3.06 to 3.38) | 0.946 |
| Childhood | Prevalence | Male | Central African Republic | RA | -0.75<br>(-4.62 to 3.28) | 0.701 |
| Childhood | Prevalence | Both | Chad | AA | -0.11<br>(-0.33 to 0.11) | 0.311 |
| Childhood | Prevalence | Female | Chad | AA | 0.04 | 0.762 |

| | | | | | | |
|---|---|---|---|---|---|---|
| | | | | | (-0.21 to 0.28) | |
| Childhood | Prevalence | Male | Chad | AA | -0.04<br>(-0.27 to 0.2) | 0.762 |
| Childhood | Prevalence | Both | Chad | T1D | 0.28<br>(-1.9 to 2.52) | 0.794 |
| Childhood | Prevalence | Female | Chad | T1D | -0.22<br>(-2.41 to 2.01) | 0.837 |
| Childhood | Prevalence | Male | Chad | T1D | 0.26<br>(-1.91 to 2.48) | 0.810 |
| Childhood | Prevalence | Both | Chad | IBD | 0.42<br>(-1.42 to 2.3) | 0.644 |
| Childhood | Prevalence | Female | Chad | IBD | 0.96<br>(-1 to 2.96) | 0.329 |
| Childhood | Prevalence | Male | Chad | IBD | 0.54<br>(-1.2 to 2.31) | 0.535 |
| Childhood | Prevalence | Both | Chad | Psoriasis | -0.85<br>(-1.99 to 0.3) | 0.142 |
| Childhood | Prevalence | Female | Chad | Psoriasis | -0.64<br>(-1.8 to 0.53) | 0.271 |
| Childhood | Prevalence | Male | Chad | Psoriasis | -0.76<br>(-1.9 to 0.4) | 0.190 |
| Childhood | Prevalence | Both | Chad | RA | 0.1<br>(-1.14 to 1.36) | 0.868 |
| Childhood | Prevalence | Female | Chad | RA | 0.48<br>(-0.94 to 1.93) | 0.495 |
| Childhood | Prevalence | Male | Chad | RA | 0.23<br>(-0.76 to 1.24) | 0.636 |
| Childhood | Prevalence | Both | Chile | Psoriasis | -0.48<br>(-1.24 to 0.29) | 0.214 |
| Childhood | Prevalence | Female | Chile | Psoriasis | -0.7<br>(-1.31 to -0.08) | 0.028 |
| Childhood | Prevalence | Male | Chile | Psoriasis | -0.43<br>(-1.35 to 0.49) | 0.344 |

| | | | | | | |
|---|---|---|---|---|---|---|
| Childhood | Prevalence | Both | China | AA | -0.01 (-0.26 to 0.25) | 0.962 |
| Childhood | Prevalence | Female | China | AA | 0.04 (-0.22 to 0.3) | 0.762 |
| Childhood | Prevalence | Male | China | AA | -0.04 (-0.29 to 0.21) | 0.762 |
| Childhood | Prevalence | Both | China | MS | 0.44 (-1.35 to 2.26) | 0.623 |
| Childhood | Prevalence | Female | China | MS | 1.01 (-0.68 to 2.73) | 0.231 |
| Childhood | Prevalence | Male | China | MS | 0.33 (-1.57 to 2.27) | 0.725 |
| Childhood | Prevalence | Both | China | Psoriasis | -1.17 (-2.35 to 0.03) | 0.056 |
| Childhood | Prevalence | Female | China | Psoriasis | -0.87 (-1.74 to 0.01) | 0.052 |
| Childhood | Prevalence | Male | China | Psoriasis | -1.16 (-2.58 to 0.29) | 0.111 |
| Childhood | Prevalence | Both | China | RHD | -0.17 (-0.4 to 0.07) | 0.173 |
| Childhood | Prevalence | Female | China | RHD | -0.16 (-0.55 to 0.23) | 0.423 |
| Childhood | Prevalence | Male | China | RHD | -0.2 (-0.47 to 0.09) | 0.172 |
| Childhood | Prevalence | Both | China | RA | -0.62 (-4.83 to 3.77) | 0.770 |
| Childhood | Prevalence | Female | China | RA | 0.41 (-3.91 to 4.93) | 0.849 |
| Childhood | Prevalence | Male | China | RA | -0.44 (-4.4 to 3.67) | 0.823 |
| Childhood | Prevalence | Both | Colombia | AA | 0.09 (-0.07 to 0.25) | 0.271 |
| Childhood | Prevalence | Female | Colombia | AA | 0.12 | 0.182 |

| | | | | | | |
|---|---|---|---|---|---|---|
| | | | | | (-0.06 to 0.3) | |
| Childhood | Prevalence | Male | Colombia | AA | 0.06 (-0.11 to 0.24) | 0.477 |
| Childhood | Prevalence | Both | Colombia | IBD | -1.31 (-4.28 to 1.75) | 0.385 |
| Childhood | Prevalence | Female | Colombia | IBD | -0.53 (-3.49 to 2.53) | 0.723 |
| Childhood | Prevalence | Male | Colombia | IBD | -1.24 (-4.24 to 1.85) | 0.414 |
| Childhood | Prevalence | Both | Colombia | Psoriasis | -0.4 (-1.17 to 0.37) | 0.298 |
| Childhood | Prevalence | Female | Colombia | Psoriasis | -0.57 (-1.33 to 0.21) | 0.144 |
| Childhood | Prevalence | Male | Colombia | Psoriasis | -0.38 (-1.15 to 0.39) | 0.320 |
| Childhood | Prevalence | Both | Colombia | RHD | -0.95 (-1.77 to -0.13) | 0.025 |
| Childhood | Prevalence | Female | Colombia | RHD | -1.29 (-2.18 to -0.4) | 0.006 |
| Childhood | Prevalence | Male | Colombia | RHD | -0.94 (-1.59 to -0.29) | 0.006 |
| Childhood | Prevalence | Both | Colombia | RA | 0.82 (-0.73 to 2.4) | 0.289 |
| Childhood | Prevalence | Female | Colombia | RA | 1.15 (-0.45 to 2.77) | 0.154 |
| Childhood | Prevalence | Male | Colombia | RA | 0.71 (-0.76 to 2.2) | 0.332 |
| Childhood | Prevalence | Both | Comoros | AA | -0.09 (-0.35 to 0.18) | 0.508 |
| Childhood | Prevalence | Female | Comoros | AA | 0.04 (-0.22 to 0.3) | 0.762 |
| Childhood | Prevalence | Male | Comoros | AA | -0.03 (-0.27 to 0.2) | 0.763 |

| | | | | | | |
|---|---|---|---|---|---|---|
| Childhood | Prevalence | Both | Comoros | Psoriasis | -0.2 (-0.89 to 0.48) | 0.547 |
| Childhood | Prevalence | Female | Comoros | Psoriasis | -0.4 (-1.09 to 0.29) | 0.244 |
| Childhood | Prevalence | Male | Comoros | Psoriasis | -0.17 (-0.85 to 0.51) | 0.605 |
| Childhood | Prevalence | Both | Comoros | RA | 0.03 (-4.43 to 4.7) | 0.988 |
| Childhood | Prevalence | Female | Comoros | RA | 1.16 (-3.2 to 5.72) | 0.595 |
| Childhood | Prevalence | Male | Comoros | RA | 0.14 (-4.76 to 5.3) | 0.954 |
| Childhood | Prevalence | Both | Congo | AA | -0.11 (-0.34 to 0.12) | 0.328 |
| Childhood | Prevalence | Female | Congo | AA | 0.04 (-0.21 to 0.28) | 0.762 |
| Childhood | Prevalence | Male | Congo | AA | -0.04 (-0.27 to 0.2) | 0.762 |
| Childhood | Prevalence | Both | Congo | MS | -0.14 (-1.86 to 1.62) | 0.873 |
| Childhood | Prevalence | Female | Congo | MS | 0.51 (-1.24 to 2.28) | 0.557 |
| Childhood | Prevalence | Male | Congo | MS | -0.1 (-1.87 to 1.7) | 0.907 |
| Childhood | Prevalence | Both | Congo | RHD | -0.17 (-0.41 to 0.08) | 0.174 |
| Childhood | Prevalence | Female | Congo | RHD | -0.16 (-0.37 to 0.06) | 0.145 |
| Childhood | Prevalence | Male | Congo | RHD | -0.21 (-0.48 to 0.06) | 0.120 |
| Childhood | Prevalence | Both | Congo | RA | 0.91 (-0.69 to 2.53) | 0.257 |
| Childhood | Prevalence | Female | Congo | RA | 0.57 | 0.495 |

| | | | | | | |
|---|---|---|---|---|---|---|
| | | | | | (-1.12 to 2.29) | |
| Childhood | Prevalence | Male | Congo | RA | 0.98<br>(-0.5 to 2.48) | 0.187 |
| Childhood | Prevalence | Both | Cook Islands | AA | 0.13<br>(-0.13 to 0.39) | 0.321 |
| Childhood | Prevalence | Female | Cook Islands | AA | -0.04<br>(-0.31 to 0.23) | 0.762 |
| Childhood | Prevalence | Male | Cook Islands | AA | 0.04<br>(-0.22 to 0.3) | 0.762 |
| Childhood | Prevalence | Both | Cook Islands | T1D | 0.47<br>(-0.87 to 1.82) | 0.480 |
| Childhood | Prevalence | Female | Cook Islands | T1D | 0.84<br>(-0.41 to 2.11) | 0.180 |
| Childhood | Prevalence | Male | Cook Islands | T1D | 0.53<br>(-0.89 to 1.96) | 0.454 |
| Childhood | Prevalence | Both | Cook Islands | IBD | 0.01<br>(-1.97 to 2.03) | 0.995 |
| Childhood | Prevalence | Female | Cook Islands | IBD | -0.51<br>(-2.49 to 1.5) | 0.602 |
| Childhood | Prevalence | Male | Cook Islands | IBD | -0.03<br>(-1.99 to 1.97) | 0.973 |
| Childhood | Prevalence | Both | Cook Islands | MS | 0.48<br>(-0.49 to 1.46) | 0.322 |
| Childhood | Prevalence | Female | Cook Islands | MS | 0.11<br>(-0.93 to 1.16) | 0.833 |
| Childhood | Prevalence | Male | Cook Islands | MS | 0.4<br>(-0.52 to 1.33) | 0.383 |
| Childhood | Prevalence | Both | Cook Islands | RA | 0.78<br>(-0.63 to 2.22) | 0.270 |
| Childhood | Prevalence | Female | Cook Islands | RA | 0.32<br>(-1.13 to 1.78) | 0.659 |
| Childhood | Prevalence | Male | Cook Islands | RA | 0.59<br>(-0.78 to 1.97) | 0.390 |

| | | | | | | |
|---|---|---|---|---|---|---|
| Childhood | Prevalence | Both | Costa Rica | AA | 0.73<br>(0.16 to 1.31) | 0.014 |
| Childhood | Prevalence | Female | Costa Rica | AA | 0.61<br>(0.05 to 1.18) | 0.034 |
| Childhood | Prevalence | Male | Costa Rica | AA | 0.72<br>(0.15 to 1.29) | 0.015 |
| Childhood | Prevalence | Both | Costa Rica | IBD | 0.03<br>(-1.5 to 1.58) | 0.968 |
| Childhood | Prevalence | Female | Costa Rica | IBD | -0.25<br>(-1.67 to 1.19) | 0.725 |
| Childhood | Prevalence | Male | Costa Rica | IBD | 0.2<br>(-1.42 to 1.85) | 0.805 |
| Childhood | Prevalence | Both | Costa Rica | Psoriasis | -0.49<br>(-2.05 to 1.1) | 0.534 |
| Childhood | Prevalence | Female | Costa Rica | Psoriasis | -0.85<br>(-2.38 to 0.72) | 0.275 |
| Childhood | Prevalence | Male | Costa Rica | Psoriasis | -0.54<br>(-2.12 to 1.07) | 0.495 |
| Childhood | Prevalence | Both | Costa Rica | RHD | 0.06<br>(-0.24 to 0.37) | 0.674 |
| Childhood | Prevalence | Female | Costa Rica | RHD | -0.05<br>(-0.39 to 0.3) | 0.791 |
| Childhood | Prevalence | Male | Costa Rica | RHD | 0.03<br>(-0.22 to 0.28) | 0.812 |
| Childhood | Prevalence | Both | Coted'Ivoire | IBD | 0.64<br>(-1.12 to 2.44) | 0.462 |
| Childhood | Prevalence | Female | Coted'Ivoire | IBD | 0.19<br>(-1.68 to 2.09) | 0.841 |
| Childhood | Prevalence | Male | Coted'Ivoire | IBD | 0.68<br>(-0.98 to 2.36) | 0.410 |
| Childhood | Prevalence | Both | Coted'Ivoire | MS | 0<br>(-1.77 to 1.79) | 0.997 |
| Childhood | Prevalence | Female | Coted'Ivoire | MS | 0.52 | 0.556 |

| | | | | | | |
|---|---|---|---|---|---|---|
| | | | | | (-1.26 to 2.33) | |
| Childhood | Prevalence | Male | Coted'Ivoire | MS | -0.09 (-1.9 to 1.75) | 0.920 |
| Childhood | Prevalence | Both | Croatia | T1D | 2.78 (1.4 to 4.18) | < 0.001 |
| Childhood | Prevalence | Female | Croatia | T1D | 2.62 (1.29 to 3.97) | < 0.001 |
| Childhood | Prevalence | Male | Croatia | T1D | 2.75 (1.32 to 4.2) | < 0.001 |
| Childhood | Prevalence | Both | Croatia | Psoriasis | -0.74 (-1.49 to 0.01) | 0.054 |
| Childhood | Prevalence | Female | Croatia | Psoriasis | -0.89 (-1.41 to -0.36) | 0.002 |
| Childhood | Prevalence | Male | Croatia | Psoriasis | -0.73 (-1.68 to 0.23) | 0.128 |
| Childhood | Prevalence | Both | Croatia | RHD | -1.53 (-2.14 to -0.9) | < 0.001 |
| Childhood | Prevalence | Female | Croatia | RHD | -1.25 (-2.55 to 0.07) | 0.063 |
| Childhood | Prevalence | Male | Croatia | RHD | -1.82 (-2.95 to -0.67) | 0.002 |
| Childhood | Prevalence | Both | Croatia | RA | 0.58 (-0.82 to 2.01) | 0.403 |
| Childhood | Prevalence | Female | Croatia | RA | 0.93 (-0.52 to 2.39) | 0.201 |
| Childhood | Prevalence | Male | Croatia | RA | 0.53 (-0.94 to 2.01) | 0.469 |
| Childhood | Prevalence | Both | Cuba | MS | 0.84 (-1 to 2.7) | 0.361 |
| Childhood | Prevalence | Female | Cuba | MS | 0.47 (-1.35 to 2.32) | 0.603 |
| Childhood | Prevalence | Male | Cuba | MS | 0.67 (-1.16 to 2.53) | 0.461 |

| | | | | | | |
|---|---|---|---|---|---|---|
| Childhood | Prevalence | Both | Cuba | RHD | -0.01 (-0.34 to 0.32) | 0.958 |
| Childhood | Prevalence | Female | Cuba | RHD | 0.09 (-0.28 to 0.46) | 0.609 |
| Childhood | Prevalence | Male | Cuba | RHD | 0.01 (-0.25 to 0.26) | 0.954 |
| Childhood | Prevalence | Both | Cyprus | AA | 0.08 (-0.16 to 0.33) | 0.485 |
| Childhood | Prevalence | Female | Cyprus | AA | -0.08 (-0.62 to 0.46) | 0.762 |
| Childhood | Prevalence | Male | Cyprus | AA | -0.04 (-0.27 to 0.2) | 0.762 |
| Childhood | Prevalence | Both | Cyprus | MS | 1.42 (-0.34 to 3.21) | 0.110 |
| Childhood | Prevalence | Female | Cyprus | MS | 0.95 (-0.76 to 2.7) | 0.265 |
| Childhood | Prevalence | Male | Cyprus | MS | 1.26 (-0.54 to 3.08) | 0.164 |
| Childhood | Prevalence | Both | Cyprus | Psoriasis | -1.63 (-3.35 to 0.12) | 0.066 |
| Childhood | Prevalence | Female | Cyprus | Psoriasis | -1.88 (-3.45 to -0.3) | 0.022 |
| Childhood | Prevalence | Male | Cyprus | Psoriasis | -1.63 (-3.49 to 0.27) | 0.089 |
| Childhood | Prevalence | Both | Cyprus | RHD | -2.63 (-7.75 to 2.78) | 0.321 |
| Childhood | Prevalence | Female | Cyprus | RHD | -2.02 (-7.57 to 3.85) | 0.478 |
| Childhood | Prevalence | Male | Cyprus | RHD | -1.77 (-6.47 to 3.16) | 0.461 |
| Childhood | Prevalence | Both | Czechia | IBD | 2.43 (0.23 to 4.68) | 0.031 |
| Childhood | Prevalence | Female | Czechia | IBD | 3.01 | 0.015 |

| | | | | | | |
|---|---|---|---|---|---|---|
| | | | | | (0.62 to 5.45) | |
| Childhood | Prevalence | Male | Czechia | IBD | 2.31<br>(0.24 to 4.43) | 0.030 |
| Childhood | Prevalence | Both | Czechia | Psoriasis | -0.89<br>(-1.64 to -0.13) | 0.023 |
| Childhood | Prevalence | Female | Czechia | Psoriasis | -0.79<br>(-1.32 to -0.26) | 0.005 |
| Childhood | Prevalence | Male | Czechia | Psoriasis | -0.88<br>(-1.83 to 0.08) | 0.070 |
| Childhood | Prevalence | Both | Czechia | RHD | -2.72<br>(-8.32 to 3.23) | 0.349 |
| Childhood | Prevalence | Female | Czechia | RHD | -4.07<br>(-9.68 to 1.9) | 0.170 |
| Childhood | Prevalence | Male | Czechia | RHD | -2.15<br>(-7.5 to 3.51) | 0.436 |
| Childhood | Prevalence | Both | Democratic People's Republic of Korea | Psoriasis | -0.59<br>(-1.78 to 0.61) | 0.319 |
| Childhood | Prevalence | Female | Democratic People's Republic of Korea | Psoriasis | -0.86<br>(-1.74 to 0.02) | 0.056 |
| Childhood | Prevalence | Male | Democratic People's Republic of Korea | Psoriasis | -0.58<br>(-1.98 to 0.83) | 0.403 |
| Childhood | Prevalence | Both | Democratic People's Republic of Korea | RHD | 0.01<br>(-0.35 to 0.37) | 0.965 |
| Childhood | Prevalence | Female | Democratic People's Republic of Korea | RHD | 0.13<br>(-0.25 to 0.52) | 0.485 |
| Childhood | Prevalence | Male | Democratic People's Republic of Korea | RHD | 0.01<br>(-0.32 to 0.34) | 0.944 |
| Childhood | Prevalence | Both | Democratic People's Republic of Korea | RA | -0.67<br>(-3.88 to 2.65) | 0.680 |
| Childhood | Prevalence | Female | Democratic People's Republic of Korea | RA | 0.19<br>(-3.07 to 3.56) | 0.907 |
| Childhood | Prevalence | Male | Democratic People's Republic of Korea | RA | -0.44<br>(-3.55 to 2.76) | 0.776 |

| | | | | | | |
|---|---|---|---|---|---|---|
| Childhood | Prevalence | Both | Democratic Republic of the Congo | IBD | -0.33 (-2.53 to 1.91) | 0.760 |
| Childhood | Prevalence | Female | Democratic Republic of the Congo | IBD | -0.95 (-2.97 to 1.12) | 0.355 |
| Childhood | Prevalence | Male | Democratic Republic of the Congo | IBD | -0.12 (-2.5 to 2.31) | 0.917 |
| Childhood | Prevalence | Both | Democratic Republic of the Congo | MS | -0.16 (-1.89 to 1.59) | 0.848 |
| Childhood | Prevalence | Female | Democratic Republic of the Congo | MS | 0.45 (-1.29 to 2.21) | 0.605 |
| Childhood | Prevalence | Male | Democratic Republic of the Congo | MS | -0.08 (-1.83 to 1.71) | 0.931 |
| Childhood | Prevalence | Both | Denmark | AA | 0.03 (-0.19 to 0.26) | 0.756 |
| Childhood | Prevalence | Female | Denmark | AA | -0.08 (-0.62 to 0.46) | 0.762 |
| Childhood | Prevalence | Male | Denmark | AA | -0.04 (-0.27 to 0.2) | 0.762 |
| Childhood | Prevalence | Both | Denmark | IBD | 0.76 (-0.26 to 1.79) | 0.137 |
| Childhood | Prevalence | Female | Denmark | IBD | 0.54 (-0.36 to 1.46) | 0.230 |
| Childhood | Prevalence | Male | Denmark | IBD | 0.68 (-0.51 to 1.88) | 0.255 |
| Childhood | Prevalence | Both | Denmark | RHD | -2.11 (-3.06 to -1.14) | < 0.001 |
| Childhood | Prevalence | Female | Denmark | RHD | -1.94 (-2.53 to -1.34) | < 0.001 |
| Childhood | Prevalence | Male | Denmark | RHD | -2.09 (-2.95 to -1.22) | < 0.001 |
| Childhood | Prevalence | Both | Denmark | RA | 0.94 (-0.32 to 2.22) | 0.139 |
| Childhood | Prevalence | Female | Denmark | RA | 0.64 | 0.285 |

| | | | | | | |
|---|---|---|---|---|---|---|
| | | | | | (-0.56 to 1.87) | |
| Childhood | Prevalence | Male | Denmark | RA | 0.83<br>(-0.49 to 2.16) | 0.210 |
| Childhood | Prevalence | Both | Djibouti | AA | 0.02<br>(-0.03 to 0.07) | 0.424 |
| Childhood | Prevalence | Female | Djibouti | AA | 0.06<br>(-0.01 to 0.13) | 0.100 |
| Childhood | Prevalence | Male | Djibouti | AA | 0.02<br>(-0.03 to 0.07) | 0.433 |
| Childhood | Prevalence | Both | Djibouti | T1D | 0.46<br>(-1.07 to 2.01) | 0.547 |
| Childhood | Prevalence | Female | Djibouti | T1D | 0.01<br>(-1.52 to 1.56) | 0.993 |
| Childhood | Prevalence | Male | Djibouti | T1D | 0.43<br>(-1.06 to 1.95) | 0.560 |
| Childhood | Prevalence | Both | Djibouti | MS | 0.2<br>(-1.56 to 1.98) | 0.822 |
| Childhood | Prevalence | Female | Djibouti | MS | -0.3<br>(-2.11 to 1.54) | 0.739 |
| Childhood | Prevalence | Male | Djibouti | MS | 0.27<br>(-1.42 to 2) | 0.744 |
| Childhood | Prevalence | Both | Djibouti | RHD | 0.22<br>(-0.45 to 0.9) | 0.507 |
| Childhood | Prevalence | Female | Djibouti | RHD | -0.04<br>(-0.76 to 0.69) | 0.915 |
| Childhood | Prevalence | Male | Djibouti | RHD | 0.2<br>(-0.43 to 0.84) | 0.515 |
| Childhood | Prevalence | Both | Djibouti | RA | 0.06<br>(-4.89 to 5.27) | 0.981 |
| Childhood | Prevalence | Female | Djibouti | RA | 0.83<br>(-4.3 to 6.25) | 0.747 |
| Childhood | Prevalence | Male | Djibouti | RA | 0.15<br>(-4.87 to 5.44) | 0.953 |

| | | | | | | |
|---|---|---|---|---|---|---|
| Childhood | Prevalence | Both | Dominica | AA | -0.06 (-0.29 to 0.18) | 0.620 |
| Childhood | Prevalence | Female | Dominica | AA | 0.04 (-0.21 to 0.29) | 0.762 |
| Childhood | Prevalence | Male | Dominica | AA | -0.04 (-0.28 to 0.21) | 0.762 |
| Childhood | Prevalence | Both | Dominica | IBD | -0.3 (-1.82 to 1.26) | 0.698 |
| Childhood | Prevalence | Female | Dominica | IBD | 0.17 (-1.28 to 1.63) | 0.817 |
| Childhood | Prevalence | Male | Dominica | IBD | -0.28 (-1.91 to 1.38) | 0.730 |
| Childhood | Prevalence | Both | Dominica | MS | 0.53 (-1.23 to 2.31) | 0.545 |
| Childhood | Prevalence | Female | Dominica | MS | 0.15 (-1.62 to 1.95) | 0.868 |
| Childhood | Prevalence | Male | Dominica | MS | 0.44 (-1.32 to 2.23) | 0.617 |
| Childhood | Prevalence | Both | Dominica | RA | 0.48 (-0.97 to 1.95) | 0.505 |
| Childhood | Prevalence | Female | Dominica | RA | 0.61 (-0.92 to 2.16) | 0.423 |
| Childhood | Prevalence | Male | Dominica | RA | 1.05 (-0.41 to 2.54) | 0.153 |
| Childhood | Prevalence | Both | Dominican Republic | AA | -0.08 (-0.33 to 0.16) | 0.484 |
| Childhood | Prevalence | Female | Dominican Republic | AA | 0.04 (-0.21 to 0.29) | 0.762 |
| Childhood | Prevalence | Male | Dominican Republic | AA | -0.04 (-0.28 to 0.21) | 0.762 |
| Childhood | Prevalence | Both | Dominican Republic | T1D | 1.19 (0.21 to 2.18) | 0.019 |
| Childhood | Prevalence | Female | Dominican Republic | T1D | 0.86 | 0.044 |

| | | | | | | |
|---|---|---|---|---|---|---|
| | | | | | (0.03 to 1.7) | |
| Childhood | Prevalence | Male | Dominican Republic | T1D | 1.25<br>(0.12 to 2.39) | 0.031 |
| Childhood | Prevalence | Both | Dominican Republic | IBD | -0.35<br>(-1.99 to 1.31) | 0.664 |
| Childhood | Prevalence | Female | Dominican Republic | IBD | -0.14<br>(-1.8 to 1.55) | 0.865 |
| Childhood | Prevalence | Male | Dominican Republic | IBD | 0<br>(-1.61 to 1.63) | 0.996 |
| Childhood | Prevalence | Both | Dominican Republic | MS | 0.52<br>(-0.29 to 1.35) | 0.201 |
| Childhood | Prevalence | Female | Dominican Republic | MS | 0.53<br>(-0.08 to 1.14) | 0.084 |
| Childhood | Prevalence | Male | Dominican Republic | MS | 0.54<br>(-0.31 to 1.41) | 0.205 |
| Childhood | Prevalence | Both | Dominican Republic | RHD | 0.57<br>(0.12 to 1.02) | 0.014 |
| Childhood | Prevalence | Female | Dominican Republic | RHD | 0.42<br>(0 to 0.84) | 0.048 |
| Childhood | Prevalence | Male | Dominican Republic | RHD | 0.55<br>(0.13 to 0.98) | 0.012 |
| Childhood | Prevalence | Both | Dominican Republic | RA | 0.48<br>(-5.09 to 6.38) | 0.864 |
| Childhood | Prevalence | Female | Dominican Republic | RA | 1.42<br>(-4.25 to 7.43) | 0.619 |
| Childhood | Prevalence | Male | Dominican Republic | RA | 0.47<br>(-5.19 to 6.48) | 0.869 |
| Childhood | Prevalence | Both | Ecuador | AA | -0.03<br>(-0.28 to 0.22) | 0.815 |
| Childhood | Prevalence | Female | Ecuador | AA | 0.04<br>(-0.21 to 0.29) | 0.762 |
| Childhood | Prevalence | Male | Ecuador | AA | -0.04<br>(-0.28 to 0.21) | 0.762 |

| | | | | | | |
|---|---|---|---|---|---|---|
| Childhood | Prevalence | Both | Ecuador | IBD | 0.3 (-0.2 to 0.8) | 0.232 |
| Childhood | Prevalence | Female | Ecuador | IBD | 0.29 (-0.18 to 0.77) | 0.220 |
| Childhood | Prevalence | Male | Ecuador | IBD | 0.33 (-0.23 to 0.89) | 0.235 |
| Childhood | Prevalence | Both | Ecuador | MS | 0.59 (-1.18 to 2.39) | 0.502 |
| Childhood | Prevalence | Female | Ecuador | MS | 1.2 (-0.59 to 3.02) | 0.182 |
| Childhood | Prevalence | Male | Ecuador | MS | 0.38 (-1.38 to 2.18) | 0.661 |
| Childhood | Prevalence | Both | Ecuador | RA | 0.38 (-3.17 to 4.07) | 0.830 |
| Childhood | Prevalence | Female | Ecuador | RA | 1.07 (-2.62 to 4.89) | 0.563 |
| Childhood | Prevalence | Male | Ecuador | RA | 0.39 (-3.05 to 3.95) | 0.821 |
| Childhood | Prevalence | Both | Egypt | AA | -0.05 (-0.3 to 0.2) | 0.687 |
| Childhood | Prevalence | Female | Egypt | AA | 0.04 (-0.21 to 0.29) | 0.762 |
| Childhood | Prevalence | Male | Egypt | AA | -0.03 (-0.26 to 0.2) | 0.808 |
| Childhood | Prevalence | Both | Egypt | T1D | 1.08 (-0.94 to 3.14) | 0.287 |
| Childhood | Prevalence | Female | Egypt | T1D | 1.66 (-0.36 to 3.71) | 0.105 |
| Childhood | Prevalence | Male | Egypt | T1D | 1.06 (-0.99 to 3.15) | 0.302 |
| Childhood | Prevalence | Both | Egypt | IBD | 0.09 (-1.7 to 1.91) | 0.920 |
| Childhood | Prevalence | Female | Egypt | IBD | -0.64 | 0.470 |

| | | | | | | |
|---|---|---|---|---|---|---|
| | | | | | (-2.4 to 1.16) | |
| Childhood | Prevalence | Male | Egypt | IBD | 0.49 (-1.27 to 2.28) | 0.578 |
| Childhood | Prevalence | Both | Egypt | MS | 0.9 (-0.79 to 2.61) | 0.286 |
| Childhood | Prevalence | Female | Egypt | MS | 0.36 (-1.27 to 2.03) | 0.654 |
| Childhood | Prevalence | Male | Egypt | MS | 1.19 (-0.55 to 2.96) | 0.173 |
| Childhood | Prevalence | Both | Egypt | Psoriasis | -0.71 (-1.52 to 0.12) | 0.089 |
| Childhood | Prevalence | Female | Egypt | Psoriasis | -0.46 (-1.23 to 0.31) | 0.230 |
| Childhood | Prevalence | Male | Egypt | Psoriasis | -0.79 (-1.64 to 0.07) | 0.071 |
| Childhood | Prevalence | Both | Egypt | RHD | 0.26 (-0.23 to 0.76) | 0.288 |
| Childhood | Prevalence | Female | Egypt | RHD | 0.44 (-0.15 to 1.03) | 0.137 |
| Childhood | Prevalence | Male | Egypt | RHD | 0.5 (0 to 1) | 0.049 |
| Childhood | Prevalence | Both | Egypt | RA | 1.04 (-1.81 to 3.97) | 0.464 |
| Childhood | Prevalence | Female | Egypt | RA | 0.47 (-2.29 to 3.32) | 0.731 |
| Childhood | Prevalence | Male | Egypt | RA | 1.13 (-1.99 to 4.34) | 0.470 |
| Childhood | Prevalence | Both | El Salvador | T1D | 0.61 (-0.18 to 1.42) | 0.125 |
| Childhood | Prevalence | Female | El Salvador | T1D | 0.82 (0.17 to 1.49) | 0.016 |
| Childhood | Prevalence | Male | El Salvador | T1D | 0.56 (-0.39 to 1.53) | 0.238 |

| | | | | | | |
|---|---|---|---|---|---|---|
| Childhood | Prevalence | Both | El Salvador | IBD | 0.08 (-1.51 to 1.7) | 0.917 |
| Childhood | Prevalence | Female | El Salvador | IBD | -0.61 (-2.22 to 1.03) | 0.451 |
| Childhood | Prevalence | Male | El Salvador | IBD | 0.39 (-1.19 to 1.99) | 0.621 |
| Childhood | Prevalence | Both | El Salvador | Psoriasis | -0.72 (-1.48 to 0.04) | 0.061 |
| Childhood | Prevalence | Female | El Salvador | Psoriasis | -0.54 (-1.3 to 0.23) | 0.160 |
| Childhood | Prevalence | Male | El Salvador | Psoriasis | -0.71 (-1.46 to 0.05) | 0.068 |
| Childhood | Prevalence | Both | El Salvador | RHD | 0.09 (-0.13 to 0.31) | 0.399 |
| Childhood | Prevalence | Female | El Salvador | RHD | 0.12 (-0.16 to 0.4) | 0.390 |
| Childhood | Prevalence | Male | El Salvador | RHD | 0.05 (-0.04 to 0.14) | 0.283 |
| Childhood | Prevalence | Both | El Salvador | RA | 0.44 (-2.68 to 3.67) | 0.776 |
| Childhood | Prevalence | Female | El Salvador | RA | 1.05 (-2.01 to 4.21) | 0.493 |
| Childhood | Prevalence | Male | El Salvador | RA | 0.3 (-2.96 to 3.67) | 0.854 |
| Childhood | Prevalence | Both | Equatorial Guinea | AA | -0.01 (-0.14 to 0.12) | 0.869 |
| Childhood | Prevalence | Female | Equatorial Guinea | AA | 0.09 (-0.05 to 0.22) | 0.211 |
| Childhood | Prevalence | Male | Equatorial Guinea | AA | 0.06 (-0.09 to 0.2) | 0.440 |
| Childhood | Prevalence | Both | Equatorial Guinea | T1D | 0.54 (-1.03 to 2.14) | 0.487 |
| Childhood | Prevalence | Female | Equatorial Guinea | T1D | 0.11 | 0.894 |

| | | | | | | |
|---|---|---|---|---|---|---|
| | | | | | (-1.51 to 1.74) | |
| Childhood | Prevalence | Male | Equatorial Guinea | T1D | 0.51 (-1.06 to 2.11) | 0.510 |
| Childhood | Prevalence | Both | Equatorial Guinea | IBD | 1.88 (0.04 to 3.76) | 0.046 |
| Childhood | Prevalence | Female | Equatorial Guinea | IBD | 1.4 (-0.53 to 3.37) | 0.149 |
| Childhood | Prevalence | Male | Equatorial Guinea | IBD | 1.94 (0.25 to 3.65) | 0.026 |
| Childhood | Prevalence | Both | Equatorial Guinea | Psoriasis | -2.53 (-3.16 to -1.9) | < 0.001 |
| Childhood | Prevalence | Female | Equatorial Guinea | Psoriasis | -2.39 (-3.03 to -1.74) | < 0.001 |
| Childhood | Prevalence | Male | Equatorial Guinea | Psoriasis | -2.52 (-3.15 to -1.88) | < 0.001 |
| Childhood | Prevalence | Both | Equatorial Guinea | RA | 1.7 (-0.59 to 4.05) | 0.141 |
| Childhood | Prevalence | Female | Equatorial Guinea | RA | 2.42 (0.25 to 4.64) | 0.030 |
| Childhood | Prevalence | Male | Equatorial Guinea | RA | 1.8 (-0.62 to 4.27) | 0.140 |
| Childhood | Prevalence | Both | Eritrea | AA | -0.01 (-0.07 to 0.05) | 0.752 |
| Childhood | Prevalence | Female | Eritrea | AA | -0.02 (-0.16 to 0.12) | 0.762 |
| Childhood | Prevalence | Male | Eritrea | AA | 0.02 (-0.13 to 0.18) | 0.762 |
| Childhood | Prevalence | Both | Eritrea | IBD | -0.02 (-2.15 to 2.15) | 0.985 |
| Childhood | Prevalence | Female | Eritrea | IBD | 0.58 (-1.4 to 2.59) | 0.558 |
| Childhood | Prevalence | Male | Eritrea | IBD | 0.01 (-2.29 to 2.36) | 0.995 |

| | | | | | | |
|---|---|---|---|---|---|---|
| Childhood | Prevalence | Both | Eritrea | RHD | 0.29 (-0.38 to 0.97) | 0.396 |
| Childhood | Prevalence | Female | Eritrea | RHD | 0.3 (-0.27 to 0.89) | 0.304 |
| Childhood | Prevalence | Male | Eritrea | RHD | 0.39 (-0.04 to 0.82) | 0.073 |
| Childhood | Prevalence | Both | Eritrea | RA | 0.65 (-0.65 to 1.97) | 0.317 |
| Childhood | Prevalence | Female | Eritrea | RA | 0.93 (-0.27 to 2.14) | 0.126 |
| Childhood | Prevalence | Male | Eritrea | RA | 0.7 (-0.65 to 2.07) | 0.301 |
| Childhood | Prevalence | Both | Estonia | IBD | 0.86 (-0.88 to 2.63) | 0.325 |
| Childhood | Prevalence | Female | Estonia | IBD | 1.41 (-0.19 to 3.04) | 0.082 |
| Childhood | Prevalence | Male | Estonia | IBD | 0.8 (-1.09 to 2.73) | 0.394 |
| Childhood | Prevalence | Both | Estonia | MS | -0.04 (-1.58 to 1.52) | 0.955 |
| Childhood | Prevalence | Female | Estonia | MS | -0.31 (-1.8 to 1.21) | 0.680 |
| Childhood | Prevalence | Male | Estonia | MS | -0.18 (-1.75 to 1.41) | 0.813 |
| Childhood | Prevalence | Both | Estonia | Psoriasis | -0.96 (-2.29 to 0.39) | 0.156 |
| Childhood | Prevalence | Female | Estonia | Psoriasis | -0.71 (-1.94 to 0.54) | 0.254 |
| Childhood | Prevalence | Male | Estonia | Psoriasis | -0.92 (-2.36 to 0.54) | 0.208 |
| Childhood | Prevalence | Both | Estonia | RHD | -2.5 (-6.73 to 1.92) | 0.252 |
| Childhood | Prevalence | Female | Estonia | RHD | -3.48 | 0.116 |

| | | | | | | |
|---|---|---|---|---|---|---|
| | | | | | (-7.71 to 0.94) | |
| Childhood | Prevalence | Male | Estonia | RHD | -2.14<br>(-5.98 to 1.86) | 0.278 |
| Childhood | Prevalence | Both | Estonia | RA | 0.74<br>(-0.99 to 2.5) | 0.390 |
| Childhood | Prevalence | Female | Estonia | RA | 1.17<br>(-0.64 to 3.02) | 0.197 |
| Childhood | Prevalence | Male | Estonia | RA | 0.83<br>(-0.84 to 2.52) | 0.320 |
| Childhood | Prevalence | Both | Eswatini | AA | 0.08<br>(-1.25 to 1.43) | 0.904 |
| Childhood | Prevalence | Female | Eswatini | AA | -0.35<br>(-1.67 to 0.99) | 0.595 |
| Childhood | Prevalence | Male | Eswatini | AA | 0.11<br>(-1.18 to 1.42) | 0.861 |
| Childhood | Prevalence | Both | Eswatini | IBD | 0.25<br>(-1.53 to 2.07) | 0.777 |
| Childhood | Prevalence | Female | Eswatini | IBD | 0.7<br>(-1.25 to 2.69) | 0.472 |
| Childhood | Prevalence | Male | Eswatini | IBD | 0.44<br>(-1.16 to 2.06) | 0.583 |
| Childhood | Prevalence | Both | Eswatini | RHD | 0.01<br>(-0.11 to 0.12) | 0.906 |
| Childhood | Prevalence | Female | Eswatini | RHD | 0.04<br>(-0.06 to 0.14) | 0.443 |
| Childhood | Prevalence | Male | Eswatini | RHD | 0.01<br>(-0.12 to 0.15) | 0.841 |
| Childhood | Prevalence | Both | Eswatini | RA | 0.01<br>(-1.76 to 1.8) | 0.994 |
| Childhood | Prevalence | Female | Eswatini | RA | 0.41<br>(-1.34 to 2.19) | 0.637 |
| Childhood | Prevalence | Male | Eswatini | RA | 0.15<br>(-1.69 to 2.01) | 0.873 |

| | | | | | | |
|---|---|---|---|---|---|---|
| Childhood | Prevalence | Both | Ethiopia | T1D | -0.34 (-1.98 to 1.33) | 0.681 |
| Childhood | Prevalence | Female | Ethiopia | T1D | 0.13 (-1.52 to 1.81) | 0.872 |
| Childhood | Prevalence | Male | Ethiopia | T1D | -0.33 (-1.96 to 1.33) | 0.683 |
| Childhood | Prevalence | Both | Ethiopia | IBD | 0.19 (-1.52 to 1.94) | 0.822 |
| Childhood | Prevalence | Female | Ethiopia | IBD | 0.66 (-1.18 to 2.54) | 0.470 |
| Childhood | Prevalence | Male | Ethiopia | IBD | 0.22 (-1.38 to 1.85) | 0.780 |
| Childhood | Prevalence | Both | Ethiopia | MS | -0.14 (-1.82 to 1.58) | 0.871 |
| Childhood | Prevalence | Female | Ethiopia | MS | 0.36 (-1.33 to 2.09) | 0.665 |
| Childhood | Prevalence | Male | Ethiopia | MS | -0.12 (-1.84 to 1.63) | 0.885 |
| Childhood | Prevalence | Both | Ethiopia | RHD | 0.53 (0.16 to 0.91) | 0.007 |
| Childhood | Prevalence | Female | Ethiopia | RHD | 0.31 (-0.11 to 0.72) | 0.141 |
| Childhood | Prevalence | Male | Ethiopia | RHD | 0.59 (0.24 to 0.95) | 0.002 |
| Childhood | Prevalence | Both | Ethiopia | RA | 0.83 (-5.37 to 7.44) | 0.791 |
| Childhood | Prevalence | Female | Ethiopia | RA | -0.64 (-6.55 to 5.63) | 0.830 |
| Childhood | Prevalence | Male | Ethiopia | RA | 0.78 (-5.52 to 7.49) | 0.808 |
| Childhood | Prevalence | Both | Fiji | AA | -0.06 (-0.32 to 0.21) | 0.674 |
| Childhood | Prevalence | Female | Fiji | AA | 0.04 | 0.762 |

| | | | | | | |
|---|---|---|---|---|---|---|
| | | | | | (-0.23 to 0.31) | |
| Childhood | Prevalence | Male | Fiji | AA | -0.04<br>(-0.3 to 0.23) | 0.762 |
| Childhood | Prevalence | Both | Fiji | T1D | 0.75<br>(-0.37 to 1.88) | 0.181 |
| Childhood | Prevalence | Female | Fiji | T1D | 0.99<br>(-0.06 to 2.06) | 0.064 |
| Childhood | Prevalence | Male | Fiji | T1D | 0.78<br>(-0.4 to 1.97) | 0.187 |
| Childhood | Prevalence | Both | Fiji | IBD | -0.07<br>(-1.56 to 1.44) | 0.922 |
| Childhood | Prevalence | Female | Fiji | IBD | -0.58<br>(-1.78 to 0.63) | 0.332 |
| Childhood | Prevalence | Male | Fiji | IBD | 0.08<br>(-1.69 to 1.88) | 0.927 |
| Childhood | Prevalence | Both | Fiji | Psoriasis | -0.6<br>(-1.7 to 0.51) | 0.274 |
| Childhood | Prevalence | Female | Fiji | Psoriasis | -0.36<br>(-1.19 to 0.47) | 0.379 |
| Childhood | Prevalence | Male | Fiji | Psoriasis | -0.61<br>(-1.93 to 0.72) | 0.355 |
| Childhood | Prevalence | Both | France | AA | -0.02<br>(-0.25 to 0.21) | 0.860 |
| Childhood | Prevalence | Female | France | AA | 0.08<br>(-0.46 to 0.62) | 0.762 |
| Childhood | Prevalence | Male | France | AA | 0.04<br>(-0.2 to 0.27) | 0.762 |
| Childhood | Prevalence | Both | France | IBD | 1.52<br>(0.44 to 2.62) | 0.008 |
| Childhood | Prevalence | Female | France | IBD | 1.92<br>(0.93 to 2.92) | < 0.001 |
| Childhood | Prevalence | Male | France | IBD | 1.26<br>(0.09 to 2.45) | 0.036 |

| | | | | | | |
|---|---|---|---|---|---|---|
| Childhood | Prevalence | Both | France | RHD | -0.89 (-0.99 to -0.79) | < 0.001 |
| Childhood | Prevalence | Female | France | RHD | -1.05 (-1.21 to -0.9) | < 0.001 |
| Childhood | Prevalence | Male | France | RHD | -0.72 (-0.87 to -0.57) | < 0.001 |
| Childhood | Prevalence | Both | Gabon | AA | -0.08 (-0.23 to 0.07) | 0.300 |
| Childhood | Prevalence | Female | Gabon | AA | 0.03 (-0.14 to 0.2) | 0.748 |
| Childhood | Prevalence | Male | Gabon | AA | -0.02 (-0.18 to 0.14) | 0.803 |
| Childhood | Prevalence | Both | Gabon | T1D | 0.6 (-0.41 to 1.61) | 0.235 |
| Childhood | Prevalence | Female | Gabon | T1D | 0.89 (-0.14 to 1.92) | 0.088 |
| Childhood | Prevalence | Male | Gabon | T1D | 0.6 (-0.39 to 1.6) | 0.226 |
| Childhood | Prevalence | Both | Gabon | MS | -0.1 (-1.79 to 1.61) | 0.903 |
| Childhood | Prevalence | Female | Gabon | MS | 0.34 (-1.37 to 2.08) | 0.687 |
| Childhood | Prevalence | Male | Gabon | MS | -0.19 (-1.92 to 1.56) | 0.824 |
| Childhood | Prevalence | Both | Gabon | RA | 0.23 (-2.83 to 3.4) | 0.879 |
| Childhood | Prevalence | Female | Gabon | RA | 0.89 (-2.02 to 3.89) | 0.540 |
| Childhood | Prevalence | Male | Gabon | RA | 0.25 (-3.12 to 3.73) | 0.883 |
| Childhood | Prevalence | Both | Gambia | AA | 0.12 (-0.1 to 0.33) | 0.277 |
| Childhood | Prevalence | Female | Gambia | AA | -0.04 | 0.762 |

| | | | | | | |
|---|---|---|---|---|---|---|
| | | | | | (-0.28 to 0.21) | |
| Childhood | Prevalence | Male | Gambia | AA | 0.04<br>(-0.2 to 0.27) | 0.762 |
| Childhood | Prevalence | Both | Gambia | T1D | 0.38<br>(-1.52 to 2.32) | 0.685 |
| Childhood | Prevalence | Female | Gambia | T1D | -0.1<br>(-2.03 to 1.87) | 0.918 |
| Childhood | Prevalence | Male | Gambia | T1D | 0.34<br>(-1.53 to 2.25) | 0.711 |
| Childhood | Prevalence | Both | Gambia | IBD | -0.25<br>(-2.03 to 1.56) | 0.778 |
| Childhood | Prevalence | Female | Gambia | IBD | 0.24<br>(-1.67 to 2.19) | 0.800 |
| Childhood | Prevalence | Male | Gambia | IBD | -0.26<br>(-1.93 to 1.43) | 0.750 |
| Childhood | Prevalence | Both | Gambia | MS | 0.44<br>(-2.8 to 3.78) | 0.788 |
| Childhood | Prevalence | Female | Gambia | MS | -0.47<br>(-3.72 to 2.9) | 0.774 |
| Childhood | Prevalence | Male | Gambia | MS | 0.34<br>(-2.95 to 3.73) | 0.838 |
| Childhood | Prevalence | Both | Gambia | RHD | 0.2<br>(0.12 to 0.28) | < 0.001 |
| Childhood | Prevalence | Female | Gambia | RHD | 0.24<br>(0.12 to 0.36) | < 0.001 |
| Childhood | Prevalence | Male | Gambia | RHD | 0.15<br>(0.1 to 0.2) | < 0.001 |
| Childhood | Prevalence | Both | Gambia | RA | 0.36<br>(-0.83 to 1.56) | 0.543 |
| Childhood | Prevalence | Female | Gambia | RA | 0.66<br>(-0.71 to 2.04) | 0.337 |
| Childhood | Prevalence | Male | Gambia | RA | 0.39<br>(-0.59 to 1.37) | 0.425 |

| | | | | | | |
|---|---|---|---|---|---|---|
| Childhood | Prevalence | Both | Georgia | AA | -0.07<br>(-0.34 to 0.19) | 0.585 |
| Childhood | Prevalence | Female | Georgia | AA | 0.04<br>(-0.21 to 0.29) | 0.762 |
| Childhood | Prevalence | Male | Georgia | AA | -0.04<br>(-0.28 to 0.21) | 0.762 |
| Childhood | Prevalence | Both | Georgia | IBD | -0.12<br>(-1.59 to 1.38) | 0.873 |
| Childhood | Prevalence | Female | Georgia | IBD | 0.24<br>(-1.12 to 1.61) | 0.724 |
| Childhood | Prevalence | Male | Georgia | IBD | -0.09<br>(-1.69 to 1.54) | 0.912 |
| Childhood | Prevalence | Both | Georgia | MS | 1.26<br>(-0.48 to 3.03) | 0.150 |
| Childhood | Prevalence | Female | Georgia | MS | 1.01<br>(-0.75 to 2.8) | 0.253 |
| Childhood | Prevalence | Male | Georgia | MS | 0.94<br>(-0.76 to 2.68) | 0.268 |
| Childhood | Prevalence | Both | Georgia | RHD | 0.07<br>(-0.16 to 0.3) | 0.543 |
| Childhood | Prevalence | Female | Georgia | RHD | 0.15<br>(-0.14 to 0.43) | 0.302 |
| Childhood | Prevalence | Male | Georgia | RHD | 0.09<br>(-0.06 to 0.24) | 0.235 |
| Childhood | Prevalence | Both | Germany | AA | -0.04<br>(-0.26 to 0.19) | 0.750 |
| Childhood | Prevalence | Female | Germany | AA | 0.08<br>(-0.46 to 0.62) | 0.762 |
| Childhood | Prevalence | Male | Germany | AA | 0.04<br>(-0.2 to 0.27) | 0.762 |
| Childhood | Prevalence | Both | Germany | IBD | 0.91<br>(-0.67 to 2.51) | 0.249 |
| Childhood | Prevalence | Female | Germany | IBD | 1.34 | 0.079 |

| | | | | | | |
|---|---|---|---|---|---|---|
| | | | | | (-0.16 to 2.86) | |
| Childhood | Prevalence | Male | Germany | IBD | 0.77<br>(-0.92 to 2.49) | 0.359 |
| Childhood | Prevalence | Both | Germany | RHD | -1.32<br>(-4.38 to 1.84) | 0.394 |
| Childhood | Prevalence | Female | Germany | RHD | -2<br>(-5.16 to 1.27) | 0.218 |
| Childhood | Prevalence | Male | Germany | RHD | -1<br>(-3.9 to 2) | 0.498 |
| Childhood | Prevalence | Both | Germany | RA | 0.41<br>(0.14 to 0.68) | 0.004 |
| Childhood | Prevalence | Female | Germany | RA | 0.34<br>(0.03 to 0.65) | 0.031 |
| Childhood | Prevalence | Male | Germany | RA | 0.42<br>(0.2 to 0.64) | 0.001 |
| Childhood | Prevalence | Both | Ghana | AA | 0.15<br>(0.02 to 0.28) | 0.027 |
| Childhood | Prevalence | Female | Ghana | AA | 0.07<br>(-0.06 to 0.2) | 0.266 |
| Childhood | Prevalence | Male | Ghana | AA | 0.1<br>(-0.03 to 0.23) | 0.131 |
| Childhood | Prevalence | Both | Ghana | MS | 1.31<br>(-0.72 to 3.37) | 0.198 |
| Childhood | Prevalence | Female | Ghana | MS | 1.69<br>(-0.42 to 3.85) | 0.112 |
| Childhood | Prevalence | Male | Ghana | MS | 0.28<br>(-1.56 to 2.14) | 0.762 |
| Childhood | Prevalence | Both | Ghana | RA | 0.49<br>(-0.67 to 1.66) | 0.397 |
| Childhood | Prevalence | Female | Ghana | RA | 0.71<br>(-0.61 to 2.05) | 0.283 |
| Childhood | Prevalence | Male | Ghana | RA | 0.54<br>(-0.42 to 1.52) | 0.259 |

| | | | | | | |
|---|---|---|---|---|---|---|
| Childhood | Prevalence | Both | Greece | AA | -0.03<br>(-0.26 to 0.21) | 0.798 |
| Childhood | Prevalence | Female | Greece | AA | 0.08<br>(-0.46 to 0.62) | 0.762 |
| Childhood | Prevalence | Male | Greece | AA | 0.04<br>(-0.2 to 0.27) | 0.762 |
| Childhood | Prevalence | Both | Greece | T1D | 3.13<br>(1.65 to 4.64) | < 0.001 |
| Childhood | Prevalence | Female | Greece | T1D | 2.9<br>(1.5 to 4.32) | < 0.001 |
| Childhood | Prevalence | Male | Greece | T1D | 3.1<br>(1.49 to 4.74) | < 0.001 |
| Childhood | Prevalence | Both | Greece | IBD | -1.25<br>(-3.37 to 0.92) | 0.245 |
| Childhood | Prevalence | Female | Greece | IBD | -1.8<br>(-3.79 to 0.24) | 0.081 |
| Childhood | Prevalence | Male | Greece | IBD | -1.31<br>(-3.48 to 0.91) | 0.236 |
| Childhood | Prevalence | Both | Greece | RHD | -0.87<br>(-1.23 to -0.52) | < 0.001 |
| Childhood | Prevalence | Female | Greece | RHD | -0.77<br>(-1.38 to -0.16) | 0.013 |
| Childhood | Prevalence | Male | Greece | RHD | -0.66<br>(-1.07 to -0.24) | 0.003 |
| Childhood | Prevalence | Both | Greenland | AA | 0.03<br>(-0.17 to 0.24) | 0.742 |
| Childhood | Prevalence | Female | Greenland | AA | 0.07<br>(-0.57 to 0.71) | 0.824 |
| Childhood | Prevalence | Male | Greenland | AA | -0.01<br>(-0.73 to 0.73) | 0.986 |
| Childhood | Prevalence | Both | Greenland | IBD | 0.54<br>(-0.63 to 1.73) | 0.353 |
| Childhood | Prevalence | Female | Greenland | IBD | 0.16 | 0.757 |

| | | | | | | |
|---|---|---|---|---|---|---|
| | | | | | (-0.9 to 1.24) | |
| Childhood | Prevalence | Male | Greenland | IBD | 0.6<br>(-0.69 to 1.91) | 0.351 |
| Childhood | Prevalence | Both | Greenland | MS | 0.11<br>(-1.25 to 1.49) | 0.867 |
| Childhood | Prevalence | Female | Greenland | MS | 0.41<br>(-0.98 to 1.81) | 0.555 |
| Childhood | Prevalence | Male | Greenland | MS | 0.2<br>(-1.19 to 1.61) | 0.771 |
| Childhood | Prevalence | Both | Greenland | RHD | -1.24<br>(-1.65 to -0.84) | < 0.001 |
| Childhood | Prevalence | Female | Greenland | RHD | -1.62<br>(-2.1 to -1.14) | < 0.001 |
| Childhood | Prevalence | Male | Greenland | RHD | -0.82<br>(-1.21 to -0.43) | < 0.001 |
| Childhood | Prevalence | Both | Greenland | RA | 1.01<br>(-0.18 to 2.21) | 0.093 |
| Childhood | Prevalence | Female | Greenland | RA | 1.3<br>(-0.11 to 2.73) | 0.069 |
| Childhood | Prevalence | Male | Greenland | RA | 0.92<br>(-0.04 to 1.89) | 0.060 |
| Childhood | Prevalence | Both | Grenada | AA | 0.07<br>(-2.49 to 2.7) | 0.955 |
| Childhood | Prevalence | Female | Grenada | AA | -0.67<br>(-3.21 to 1.94) | 0.598 |
| Childhood | Prevalence | Male | Grenada | AA | 0.13<br>(-2.44 to 2.76) | 0.921 |
| Childhood | Prevalence | Both | Grenada | MS | 0.61<br>(-1.58 to 2.85) | 0.574 |
| Childhood | Prevalence | Female | Grenada | MS | 0.17<br>(-2.04 to 2.42) | 0.879 |
| Childhood | Prevalence | Male | Grenada | MS | 0.81<br>(-1.27 to 2.94) | 0.434 |

| | | | | | | |
|---|---|---|---|---|---|---|
| Childhood | Prevalence | Both | Grenada | Psoriasis | -0.92 (-2.34 to 0.53) | 0.203 |
| Childhood | Prevalence | Female | Grenada | Psoriasis | -0.6 (-2.03 to 0.85) | 0.401 |
| Childhood | Prevalence | Male | Grenada | Psoriasis | -0.88 (-2.33 to 0.58) | 0.226 |
| Childhood | Prevalence | Both | Grenada | RHD | 0.02 (-0.18 to 0.23) | 0.824 |
| Childhood | Prevalence | Female | Grenada | RHD | 0 (-0.24 to 0.25) | 0.985 |
| Childhood | Prevalence | Male | Grenada | RHD | 0.02 (-0.15 to 0.19) | 0.806 |
| Childhood | Prevalence | Both | Guam | IBD | -0.34 (-2 to 1.35) | 0.683 |
| Childhood | Prevalence | Female | Guam | IBD | 0.14 (-1.7 to 2.02) | 0.878 |
| Childhood | Prevalence | Male | Guam | IBD | -0.33 (-1.8 to 1.17) | 0.655 |
| Childhood | Prevalence | Both | Guam | RHD | 0.38 (-0.21 to 0.98) | 0.197 |
| Childhood | Prevalence | Female | Guam | RHD | 0.44 (-0.06 to 0.93) | 0.081 |
| Childhood | Prevalence | Male | Guam | RHD | 0.39 (-0.36 to 1.14) | 0.297 |
| Childhood | Prevalence | Both | Guam | RA | 0 (-1.39 to 1.41) | 0.998 |
| Childhood | Prevalence | Female | Guam | RA | 0.38 (-1.07 to 1.84) | 0.601 |
| Childhood | Prevalence | Male | Guam | RA | -0.05 (-1.4 to 1.31) | 0.941 |
| Childhood | Prevalence | Both | Guatemala | AA | 0.03 (-0.23 to 0.3) | 0.803 |
| Childhood | Prevalence | Female | Guatemala | AA | 0.04 | 0.762 |

| | | | | | | |
|---|---|---|---|---|---|---|
| | | | | | (-0.21 to 0.29) | |
| Childhood | Prevalence | Male | Guatemala | AA | -0.04 (-0.28 to 0.21) | 0.762 |
| Childhood | Prevalence | Both | Guatemala | IBD | 0.9 (0.18 to 1.62) | 0.016 |
| Childhood | Prevalence | Female | Guatemala | IBD | 0.97 (0.08 to 1.87) | 0.034 |
| Childhood | Prevalence | Male | Guatemala | IBD | 0.82 (0.18 to 1.48) | 0.015 |
| Childhood | Prevalence | Both | Guatemala | MS | 0.63 (-3.51 to 4.95) | 0.761 |
| Childhood | Prevalence | Female | Guatemala | MS | -0.01 (-4.49 to 4.68) | 0.996 |
| Childhood | Prevalence | Male | Guatemala | MS | 0.35 (-3.51 to 4.37) | 0.856 |
| Childhood | Prevalence | Both | Guatemala | Psoriasis | -0.61 (-1.37 to 0.15) | 0.113 |
| Childhood | Prevalence | Female | Guatemala | Psoriasis | -0.81 (-1.57 to -0.05) | 0.037 |
| Childhood | Prevalence | Male | Guatemala | Psoriasis | -0.59 (-1.34 to 0.17) | 0.121 |
| Childhood | Prevalence | Both | Guatemala | RHD | 0.23 (0.06 to 0.39) | 0.010 |
| Childhood | Prevalence | Female | Guatemala | RHD | 0.23 (0.03 to 0.43) | 0.029 |
| Childhood | Prevalence | Male | Guatemala | RHD | 0.2 (0.1 to 0.3) | < 0.001 |
| Childhood | Prevalence | Both | Guatemala | RA | 1.15 (-0.31 to 2.63) | 0.118 |
| Childhood | Prevalence | Female | Guatemala | RA | 1.37 (-0.1 to 2.87) | 0.068 |
| Childhood | Prevalence | Male | Guatemala | RA | 0.97 (-0.41 to 2.38) | 0.161 |

| | | | | | | |
|---|---|---|---|---|---|---|
| Childhood | Prevalence | Both | Guinea | Psoriasis | -0.64<br>(-1.35 to 0.08) | 0.080 |
| Childhood | Prevalence | Female | Guinea | Psoriasis | -0.53<br>(-1.24 to 0.19) | 0.144 |
| Childhood | Prevalence | Male | Guinea | Psoriasis | -0.54<br>(-1.26 to 0.19) | 0.140 |
| Childhood | Prevalence | Both | Guinea | RHD | 0.23<br>(-0.41 to 0.88) | 0.469 |
| Childhood | Prevalence | Female | Guinea | RHD | 0.38<br>(-0.32 to 1.07) | 0.276 |
| Childhood | Prevalence | Male | Guinea | RHD | 0.21<br>(-0.38 to 0.8) | 0.471 |
| Childhood | Prevalence | Both | Guinea | RA | -0.51<br>(-3.76 to 2.84) | 0.754 |
| Childhood | Prevalence | Female | Guinea | RA | 0.34<br>(-2.58 to 3.35) | 0.816 |
| Childhood | Prevalence | Male | Guinea | RA | -0.73<br>(-4.62 to 3.31) | 0.708 |
| Childhood | Prevalence | Both | Guinea-Bissau | AA | 0.1<br>(-0.24 to 0.44) | 0.550 |
| Childhood | Prevalence | Female | Guinea-Bissau | AA | 0.01<br>(-0.32 to 0.34) | 0.933 |
| Childhood | Prevalence | Male | Guinea-Bissau | AA | 0.12<br>(-0.21 to 0.45) | 0.459 |
| Childhood | Prevalence | Both | Guinea-Bissau | T1D | -0.09<br>(-1.16 to 1) | 0.868 |
| Childhood | Prevalence | Female | Guinea-Bissau | T1D | 0.24<br>(-0.86 to 1.36) | 0.657 |
| Childhood | Prevalence | Male | Guinea-Bissau | T1D | -0.11<br>(-1.17 to 0.96) | 0.833 |
| Childhood | Prevalence | Both | Guinea-Bissau | IBD | 0.4<br>(-1.39 to 2.22) | 0.650 |
| Childhood | Prevalence | Female | Guinea-Bissau | IBD | 0.99 | 0.291 |

| | | | | | | |
|---|---|---|---|---|---|---|
| | | | | | (-0.89 to 2.91) | |
| Childhood | Prevalence | Male | Guinea-Bissau | IBD | 0.39<br>(-1.29 to 2.11) | 0.638 |
| Childhood | Prevalence | Both | Guinea-Bissau | MS | -0.14<br>(-2 to 1.75) | 0.877 |
| Childhood | Prevalence | Female | Guinea-Bissau | MS | 0.43<br>(-1.43 to 2.32) | 0.643 |
| Childhood | Prevalence | Male | Guinea-Bissau | MS | -0.17<br>(-2.04 to 1.75) | 0.859 |
| Childhood | Prevalence | Both | Guinea-Bissau | RHD | 0.2<br>(0.17 to 0.24) | < 0.001 |
| Childhood | Prevalence | Female | Guinea-Bissau | RHD | 0.23<br>(0.18 to 0.29) | < 0.001 |
| Childhood | Prevalence | Male | Guinea-Bissau | RHD | 0.17<br>(0.14 to 0.19) | < 0.001 |
| Childhood | Prevalence | Both | Guinea-Bissau | RA | 0.6<br>(-0.7 to 1.91) | 0.357 |
| Childhood | Prevalence | Female | Guinea-Bissau | RA | 0.18<br>(-1.27 to 1.66) | 0.800 |
| Childhood | Prevalence | Male | Guinea-Bissau | RA | 0.64<br>(-0.35 to 1.63) | 0.199 |
| Childhood | Prevalence | Both | Guyana | AA | -0.03<br>(-0.27 to 0.21) | 0.780 |
| Childhood | Prevalence | Female | Guyana | AA | 0.04<br>(-0.21 to 0.29) | 0.762 |
| Childhood | Prevalence | Male | Guyana | AA | -0.04<br>(-0.28 to 0.21) | 0.762 |
| Childhood | Prevalence | Both | Guyana | IBD | 0.02<br>(-1.76 to 1.83) | 0.981 |
| Childhood | Prevalence | Female | Guyana | IBD | 0.73<br>(-1.19 to 2.69) | 0.447 |
| Childhood | Prevalence | Male | Guyana | IBD | -0.15<br>(-1.75 to 1.48) | 0.851 |

| | | | | | | |
|---|---|---|---|---|---|---|
| Childhood | Prevalence | Both | Guyana | RHD | 0.02 (-0.09 to 0.13) | 0.689 |
| Childhood | Prevalence | Female | Guyana | RHD | 0.01 (-0.14 to 0.16) | 0.887 |
| Childhood | Prevalence | Male | Guyana | RHD | -0.02 (-0.08 to 0.04) | 0.529 |
| Childhood | Prevalence | Both | Guyana | RA | 0.06 (-1.98 to 2.15) | 0.951 |
| Childhood | Prevalence | Female | Guyana | RA | 0.58 (-1.47 to 2.67) | 0.571 |
| Childhood | Prevalence | Male | Guyana | RA | 0.09 (-1.99 to 2.21) | 0.933 |
| Childhood | Prevalence | Both | Haiti | IBD | 0.46 (-1.34 to 2.29) | 0.608 |
| Childhood | Prevalence | Female | Haiti | IBD | 0.02 (-1.95 to 2.03) | 0.983 |
| Childhood | Prevalence | Male | Haiti | IBD | 0.17 (-1.43 to 1.8) | 0.831 |
| Childhood | Prevalence | Both | Haiti | MS | -0.47 (-4.31 to 3.51) | 0.806 |
| Childhood | Prevalence | Female | Haiti | MS | 0.52 (-3.44 to 4.65) | 0.793 |
| Childhood | Prevalence | Male | Haiti | MS | -0.77 (-4.53 to 3.14) | 0.686 |
| Childhood | Prevalence | Both | Haiti | Psoriasis | -0.48 (-1.18 to 0.23) | 0.178 |
| Childhood | Prevalence | Female | Haiti | Psoriasis | -0.33 (-1.05 to 0.39) | 0.351 |
| Childhood | Prevalence | Male | Haiti | Psoriasis | -0.46 (-1.17 to 0.26) | 0.199 |
| Childhood | Prevalence | Both | Haiti | RHD | 0.11 (0.07 to 0.14) | < 0.001 |
| Childhood | Prevalence | Female | Haiti | RHD | 0.13 | < 0.001 |

| | | | | | | |
|---|---|---|---|---|---|---|
| | | | | | (0.11 to 0.14) | |
| Childhood | Prevalence | Male | Haiti | RHD | 0.05<br>(-0.02 to 0.12) | 0.187 |
| Childhood | Prevalence | Both | High SDI | AA | -0.12<br>(-0.42 to 0.18) | 0.421 |
| Childhood | Prevalence | Female | High SDI | AA | -0.2<br>(-0.72 to 0.32) | 0.430 |
| Childhood | Prevalence | Male | High SDI | AA | -0.17<br>(-0.36 to 0.01) | 0.063 |
| Childhood | Prevalence | Both | High SDI | MS | 0.31<br>(-1.49 to 2.14) | 0.729 |
| Childhood | Prevalence | Female | High SDI | MS | 0.76<br>(-1.01 to 2.56) | 0.388 |
| Childhood | Prevalence | Male | High SDI | MS | 0.1<br>(-1.73 to 1.96) | 0.914 |
| Childhood | Prevalence | Both | High SDI | RHD | -1.28<br>(-5.45 to 3.08) | 0.546 |
| Childhood | Prevalence | Female | High SDI | RHD | -2.97<br>(-7.17 to 1.41) | 0.173 |
| Childhood | Prevalence | Male | High SDI | RHD | -0.34<br>(-4.3 to 3.78) | 0.864 |
| Childhood | Prevalence | Both | High-middle SDI | AA | 0.01<br>(-0.08 to 0.09) | 0.872 |
| Childhood | Prevalence | Female | High-middle SDI | AA | 0.04<br>(0 to 0.09) | 0.051 |
| Childhood | Prevalence | Male | High-middle SDI | AA | 0.06<br>(-0.38 to 0.5) | 0.783 |
| Childhood | Prevalence | Both | High-middle SDI | IBD | -0.48<br>(-1.95 to 1.01) | 0.511 |
| Childhood | Prevalence | Female | High-middle SDI | IBD | -0.15<br>(-1.53 to 1.25) | 0.823 |
| Childhood | Prevalence | Male | High-middle SDI | IBD | -0.42<br>(-1.97 to 1.16) | 0.590 |

| | | | | | | |
|---|---|---|---|---|---|---|
| Childhood | Prevalence | Both | High-middle SDI | Psoriasis | -0.65<br>(-1.59 to 0.29) | 0.165 |
| Childhood | Prevalence | Female | High-middle SDI | Psoriasis | -0.97<br>(-1.69 to -0.24) | 0.011 |
| Childhood | Prevalence | Male | High-middle SDI | Psoriasis | -0.57<br>(-1.69 to 0.55) | 0.302 |
| Childhood | Prevalence | Both | High-middle SDI | RA | 1.3<br>(-1.41 to 4.09) | 0.338 |
| Childhood | Prevalence | Female | High-middle SDI | RA | 0.88<br>(-1.79 to 3.62) | 0.510 |
| Childhood | Prevalence | Male | High-middle SDI | RA | 1.36<br>(-1.34 to 4.13) | 0.315 |
| Childhood | Prevalence | Both | Honduras | AA | -0.03<br>(-1.15 to 1.1) | 0.953 |
| Childhood | Prevalence | Female | Honduras | AA | -0.38<br>(-1.49 to 0.74) | 0.492 |
| Childhood | Prevalence | Male | Honduras | AA | -0.07<br>(-1.18 to 1.04) | 0.892 |
| Childhood | Prevalence | Both | Honduras | MS | 0.38<br>(-1.53 to 2.33) | 0.691 |
| Childhood | Prevalence | Female | Honduras | MS | 0.94<br>(-0.97 to 2.89) | 0.322 |
| Childhood | Prevalence | Male | Honduras | MS | 0.14<br>(-1.78 to 2.1) | 0.882 |
| Childhood | Prevalence | Both | Honduras | Psoriasis | -0.62<br>(-1.38 to 0.14) | 0.105 |
| Childhood | Prevalence | Female | Honduras | Psoriasis | -0.48<br>(-1.24 to 0.29) | 0.209 |
| Childhood | Prevalence | Male | Honduras | Psoriasis | -0.62<br>(-1.37 to 0.15) | 0.109 |
| Childhood | Prevalence | Both | Honduras | RA | 0.82<br>(-0.77 to 2.44) | 0.299 |
| Childhood | Prevalence | Female | Honduras | RA | 1.14 | 0.174 |

| | | | | | | |
|---|---|---|---|---|---|---|
| | | | | | (-0.53 to 2.85) | |
| Childhood | Prevalence | Male | Honduras | RA | 0.67<br>(-0.59 to 1.95) | 0.289 |
| Childhood | Prevalence | Both | Hungary | AA | -0.08<br>(-1.52 to 1.37) | 0.906 |
| Childhood | Prevalence | Female | Hungary | AA | -0.45<br>(-1.87 to 0.99) | 0.526 |
| Childhood | Prevalence | Male | Hungary | AA | -0.09<br>(-1.52 to 1.36) | 0.898 |
| Childhood | Prevalence | Both | Hungary | IBD | 3.11<br>(1.97 to 4.26) | < 0.001 |
| Childhood | Prevalence | Female | Hungary | IBD | 3.36<br>(1.35 to 5.41) | 0.001 |
| Childhood | Prevalence | Male | Hungary | IBD | 3.01<br>(1.79 to 4.25) | < 0.001 |
| Childhood | Prevalence | Both | Hungary | MS | -0.64<br>(-2.47 to 1.22) | 0.484 |
| Childhood | Prevalence | Female | Hungary | MS | -0.43<br>(-2.26 to 1.44) | 0.641 |
| Childhood | Prevalence | Male | Hungary | MS | -0.49<br>(-2.29 to 1.33) | 0.581 |
| Childhood | Prevalence | Both | Hungary | Psoriasis | -0.95<br>(-2.61 to 0.74) | 0.258 |
| Childhood | Prevalence | Female | Hungary | Psoriasis | -1.27<br>(-2.72 to 0.21) | 0.089 |
| Childhood | Prevalence | Male | Hungary | Psoriasis | -1.01<br>(-2.88 to 0.91) | 0.289 |
| Childhood | Prevalence | Both | Hungary | RHD | -2.17<br>(-6.66 to 2.52) | 0.345 |
| Childhood | Prevalence | Female | Hungary | RHD | -3.12<br>(-7.72 to 1.72) | 0.194 |
| Childhood | Prevalence | Male | Hungary | RHD | -2<br>(-6.23 to 2.42) | 0.356 |

| | | | | | | |
|---|---|---|---|---|---|---|
| Childhood | Prevalence | Both | Hungary | RA | 0.71 (-0.79 to 2.23) | 0.344 |
| Childhood | Prevalence | Female | Hungary | RA | 0.4 (-1.17 to 2) | 0.606 |
| Childhood | Prevalence | Male | Hungary | RA | 0.8 (-0.7 to 2.31) | 0.285 |
| Childhood | Prevalence | Both | Iceland | AA | -0.02 (-0.25 to 0.2) | 0.824 |
| Childhood | Prevalence | Female | Iceland | AA | 0.08 (-0.46 to 0.62) | 0.762 |
| Childhood | Prevalence | Male | Iceland | AA | 0.04 (-0.2 to 0.27) | 0.762 |
| Childhood | Prevalence | Both | Iceland | IBD | -0.53 (-4.42 to 3.51) | 0.786 |
| Childhood | Prevalence | Female | Iceland | IBD | -0.04 (-3.65 to 3.71) | 0.982 |
| Childhood | Prevalence | Male | Iceland | IBD | 0.05 (-4.12 to 4.39) | 0.982 |
| Childhood | Prevalence | Both | Iceland | MS | 0.39 (-1.63 to 2.44) | 0.700 |
| Childhood | Prevalence | Female | Iceland | MS | 0.12 (-1.83 to 2.11) | 0.901 |
| Childhood | Prevalence | Male | Iceland | MS | 0.3 (-1.7 to 2.34) | 0.764 |
| Childhood | Prevalence | Both | Iceland | Psoriasis | -0.21 (-1.2 to 0.78) | 0.662 |
| Childhood | Prevalence | Female | Iceland | Psoriasis | -0.39 (-1.24 to 0.46) | 0.357 |
| Childhood | Prevalence | Male | Iceland | Psoriasis | -0.19 (-1.3 to 0.92) | 0.724 |
| Childhood | Prevalence | Both | India | IBD | -0.25 (-2.21 to 1.74) | 0.794 |
| Childhood | Prevalence | Female | India | IBD | 0.34 | 0.732 |

| | | | | | | |
|---|---|---|---|---|---|---|
| | | | | | (-1.66 to 2.38) | |
| Childhood | Prevalence | Male | India | IBD | -0.21<br>(-2.13 to 1.74) | 0.823 |
| Childhood | Prevalence | Both | India | Psoriasis | -0.36<br>(-1.9 to 1.21) | 0.641 |
| Childhood | Prevalence | Female | India | Psoriasis | 0.07<br>(-1.46 to 1.62) | 0.928 |
| Childhood | Prevalence | Male | India | Psoriasis | -0.36<br>(-1.91 to 1.21) | 0.640 |
| Childhood | Prevalence | Both | India | RA | 0.3<br>(-0.58 to 1.2) | 0.489 |
| Childhood | Prevalence | Female | India | RA | 0.49<br>(-0.45 to 1.43) | 0.295 |
| Childhood | Prevalence | Male | India | RA | 0.27<br>(-0.61 to 1.16) | 0.532 |
| Childhood | Prevalence | Both | Indonesia | AA | -0.04<br>(-0.17 to 0.09) | 0.544 |
| Childhood | Prevalence | Female | Indonesia | AA | 0.02<br>(-0.12 to 0.16) | 0.762 |
| Childhood | Prevalence | Male | Indonesia | AA | -0.02<br>(-0.16 to 0.12) | 0.762 |
| Childhood | Prevalence | Both | Indonesia | MS | 0.17<br>(-1.38 to 1.75) | 0.825 |
| Childhood | Prevalence | Female | Indonesia | MS | -0.28<br>(-1.81 to 1.27) | 0.710 |
| Childhood | Prevalence | Male | Indonesia | MS | 0.26<br>(-1.32 to 1.85) | 0.744 |
| Childhood | Prevalence | Both | Indonesia | Psoriasis | -0.52<br>(-1.59 to 0.56) | 0.330 |
| Childhood | Prevalence | Female | Indonesia | Psoriasis | -0.2<br>(-1 to 0.6) | 0.611 |
| Childhood | Prevalence | Male | Indonesia | Psoriasis | -0.6<br>(-1.93 to 0.74) | 0.363 |

| | | | | | | |
|---|---|---|---|---|---|---|
| Childhood | Prevalence | Both | Indonesia | RA | -0.22<br>(-1.67 to 1.25) | 0.763 |
| Childhood | Prevalence | Female | Indonesia | RA | 0.19<br>(-1.33 to 1.73) | 0.804 |
| Childhood | Prevalence | Male | Indonesia | RA | -0.06<br>(-1.43 to 1.33) | 0.933 |
| Childhood | Prevalence | Both | Iran (Islamic Republic of) | IBD | -0.32<br>(-2.11 to 1.51) | 0.724 |
| Childhood | Prevalence | Female | Iran (Islamic Republic of) | IBD | -0.1<br>(-1.89 to 1.72) | 0.910 |
| Childhood | Prevalence | Male | Iran (Islamic Republic of) | IBD | 0.13<br>(-1.69 to 1.98) | 0.889 |
| Childhood | Prevalence | Both | Iran (Islamic Republic of) | Psoriasis | -1.27<br>(-2.58 to 0.07) | 0.062 |
| Childhood | Prevalence | Female | Iran (Islamic Republic of) | Psoriasis | -1.62<br>(-2.83 to -0.39) | 0.012 |
| Childhood | Prevalence | Male | Iran (Islamic Republic of) | Psoriasis | -1.25<br>(-2.67 to 0.19) | 0.087 |
| Childhood | Prevalence | Both | Iran (Islamic Republic of) | RHD | 0.11<br>(-0.33 to 0.55) | 0.626 |
| Childhood | Prevalence | Female | Iran (Islamic Republic of) | RHD | 0<br>(-0.5 to 0.5) | 0.994 |
| Childhood | Prevalence | Male | Iran (Islamic Republic of) | RHD | 0.06<br>(-0.3 to 0.43) | 0.725 |
| Childhood | Prevalence | Both | Iraq | AA | -0.03<br>(-0.27 to 0.21) | 0.802 |
| Childhood | Prevalence | Female | Iraq | AA | 0.04<br>(-0.21 to 0.29) | 0.762 |
| Childhood | Prevalence | Male | Iraq | AA | -0.03<br>(-0.26 to 0.19) | 0.762 |
| Childhood | Prevalence | Both | Iraq | IBD | 0.91<br>(0.39 to 1.42) | 0.001 |
| Childhood | Prevalence | Female | Iraq | IBD | 0.94 | 0.003 |

| | | | | | | |
|---|---|---|---|---|---|---|
| | | | | | (0.35 to 1.53) | |
| Childhood | Prevalence | Male | Iraq | IBD | 1.01<br>(0.55 to 1.47) | < 0.001 |
| Childhood | Prevalence | Both | Iraq | MS | 0.82<br>(-1.51 to 3.21) | 0.478 |
| Childhood | Prevalence | Female | Iraq | MS | 0.14<br>(-2.16 to 2.51) | 0.901 |
| Childhood | Prevalence | Male | Iraq | MS | 0.92<br>(-1.4 to 3.3) | 0.426 |
| Childhood | Prevalence | Both | Iraq | Psoriasis | -0.57<br>(-2.04 to 0.92) | 0.438 |
| Childhood | Prevalence | Female | Iraq | Psoriasis | -0.9<br>(-2.27 to 0.5) | 0.197 |
| Childhood | Prevalence | Male | Iraq | Psoriasis | -0.54<br>(-2.1 to 1.06) | 0.494 |
| Childhood | Prevalence | Both | Iraq | RHD | 0.52<br>(-0.6 to 1.66) | 0.347 |
| Childhood | Prevalence | Female | Iraq | RHD | 0.22<br>(-0.76 to 1.22) | 0.650 |
| Childhood | Prevalence | Male | Iraq | RHD | 0.58<br>(-0.7 to 1.87) | 0.362 |
| Childhood | Prevalence | Both | Iraq | RA | 0.69<br>(-0.79 to 2.19) | 0.351 |
| Childhood | Prevalence | Female | Iraq | RA | 1.07<br>(-0.43 to 2.59) | 0.157 |
| Childhood | Prevalence | Male | Iraq | RA | 0.62<br>(-0.82 to 2.07) | 0.386 |
| Childhood | Prevalence | Both | Ireland | AA | 0.05<br>(-0.19 to 0.28) | 0.687 |
| Childhood | Prevalence | Female | Ireland | AA | -0.08<br>(-0.62 to 0.46) | 0.762 |
| Childhood | Prevalence | Male | Ireland | AA | -0.04<br>(-0.27 to 0.2) | 0.762 |

| | | | | | | |
|---|---|---|---|---|---|---|
| Childhood | Prevalence | Both | Ireland | MS | 0.45<br>(-1.21 to 2.14) | 0.586 |
| Childhood | Prevalence | Female | Ireland | MS | 0.83<br>(-0.82 to 2.5) | 0.315 |
| Childhood | Prevalence | Male | Ireland | MS | 0.23<br>(-1.51 to 2) | 0.787 |
| Childhood | Prevalence | Both | Ireland | Psoriasis | -0.61<br>(-1.48 to 0.26) | 0.162 |
| Childhood | Prevalence | Female | Ireland | Psoriasis | -0.46<br>(-1.18 to 0.27) | 0.211 |
| Childhood | Prevalence | Male | Ireland | Psoriasis | -0.63<br>(-1.63 to 0.38) | 0.209 |
| Childhood | Prevalence | Both | Ireland | RHD | -1.16<br>(-4.03 to 1.79) | 0.423 |
| Childhood | Prevalence | Female | Ireland | RHD | -2.04<br>(-5.01 to 1.02) | 0.181 |
| Childhood | Prevalence | Male | Ireland | RHD | -0.7<br>(-3.37 to 2.04) | 0.600 |
| Childhood | Prevalence | Both | Ireland | RA | 0.11<br>(-3.35 to 3.69) | 0.950 |
| Childhood | Prevalence | Female | Ireland | RA | 0.75<br>(-2.81 to 4.45) | 0.672 |
| Childhood | Prevalence | Male | Ireland | RA | -0.02<br>(-3.68 to 3.77) | 0.991 |
| Childhood | Prevalence | Both | Israel | T1D | 0.1<br>(-1.95 to 2.2) | 0.920 |
| Childhood | Prevalence | Female | Israel | T1D | -0.66<br>(-2.63 to 1.35) | 0.503 |
| Childhood | Prevalence | Male | Israel | T1D | 0.82<br>(-1.33 to 3.03) | 0.445 |
| Childhood | Prevalence | Both | Israel | MS | 0.74<br>(-1.23 to 2.74) | 0.452 |
| Childhood | Prevalence | Female | Israel | MS | 0.23 | 0.807 |

| | | | | | | |
|---|---|---|---|---|---|---|
| | | | | | (-1.69 to 2.19) | |
| Childhood | Prevalence | Male | Israel | MS | 0.79<br>(-1.17 to 2.79) | 0.419 |
| Childhood | Prevalence | Both | Israel | Psoriasis | -0.21<br>(-1.08 to 0.66) | 0.623 |
| Childhood | Prevalence | Female | Israel | Psoriasis | -0.42<br>(-1.15 to 0.3) | 0.242 |
| Childhood | Prevalence | Male | Israel | Psoriasis | -0.2<br>(-1.21 to 0.81) | 0.681 |
| Childhood | Prevalence | Both | Italy | IBD | -0.44<br>(-1.93 to 1.08) | 0.560 |
| Childhood | Prevalence | Female | Italy | IBD | -0.02<br>(-1.42 to 1.39) | 0.973 |
| Childhood | Prevalence | Male | Italy | IBD | -0.4<br>(-1.97 to 1.19) | 0.607 |
| Childhood | Prevalence | Both | Italy | MS | 1.27<br>(-0.25 to 2.82) | 0.099 |
| Childhood | Prevalence | Female | Italy | MS | 0.93<br>(-0.59 to 2.47) | 0.223 |
| Childhood | Prevalence | Male | Italy | MS | 1.2<br>(-0.35 to 2.78) | 0.125 |
| Childhood | Prevalence | Both | Italy | Psoriasis | -0.03<br>(-0.87 to 0.82) | 0.943 |
| Childhood | Prevalence | Female | Italy | Psoriasis | -0.25<br>(-0.94 to 0.44) | 0.455 |
| Childhood | Prevalence | Male | Italy | Psoriasis | -0.01<br>(-1 to 0.99) | 0.980 |
| Childhood | Prevalence | Both | Italy | RA | 0.08<br>(-3.44 to 3.73) | 0.965 |
| Childhood | Prevalence | Female | Italy | RA | -0.88<br>(-4.37 to 2.73) | 0.616 |
| Childhood | Prevalence | Male | Italy | RA | 0.14<br>(-3.29 to 3.7) | 0.933 |

| | | | | | | |
|---|---|---|---|---|---|---|
| Childhood | Prevalence | Both | Jamaica | AA | 0.02 (-0.21 to 0.26) | 0.838 |
| Childhood | Prevalence | Female | Jamaica | AA | -0.04 (-0.29 to 0.21) | 0.762 |
| Childhood | Prevalence | Male | Jamaica | AA | 0.04 (-0.21 to 0.28) | 0.762 |
| Childhood | Prevalence | Both | Jamaica | IBD | 0.04 (-1.49 to 1.6) | 0.959 |
| Childhood | Prevalence | Female | Jamaica | IBD | 0.53 (-1 to 2.08) | 0.488 |
| Childhood | Prevalence | Male | Jamaica | IBD | -0.01 (-1.59 to 1.6) | 0.994 |
| Childhood | Prevalence | Both | Jamaica | Psoriasis | -0.57 (-1.93 to 0.82) | 0.408 |
| Childhood | Prevalence | Female | Jamaica | Psoriasis | -0.88 (-2.22 to 0.48) | 0.196 |
| Childhood | Prevalence | Male | Jamaica | Psoriasis | -0.57 (-1.96 to 0.84) | 0.409 |
| Childhood | Prevalence | Both | Jamaica | RHD | 0.22 (0 to 0.45) | 0.053 |
| Childhood | Prevalence | Female | Jamaica | RHD | 0.2 (-0.04 to 0.44) | 0.104 |
| Childhood | Prevalence | Male | Jamaica | RHD | 0.19 (-0.03 to 0.42) | 0.084 |
| Childhood | Prevalence | Both | Japan | AA | -0.41 (-1.25 to 0.44) | 0.335 |
| Childhood | Prevalence | Female | Japan | AA | -0.17 (-1.52 to 1.19) | 0.798 |
| Childhood | Prevalence | Male | Japan | AA | -0.04 (-0.35 to 0.27) | 0.788 |
| Childhood | Prevalence | Both | Japan | T1D | -0.02 (-0.91 to 0.88) | 0.963 |
| Childhood | Prevalence | Female | Japan | T1D | 0.26 | 0.533 |

| | | | | | | |
|---|---|---|---|---|---|---|
| | | | | | (-0.58 to 1.1) | |
| Childhood | Prevalence | Male | Japan | T1D | -0.01<br>(-0.92 to 0.9) | 0.975 |
| Childhood | Prevalence | Both | Japan | MS | -0.75<br>(-3.6 to 2.18) | 0.600 |
| Childhood | Prevalence | Female | Japan | MS | 0.23<br>(-2.69 to 3.25) | 0.873 |
| Childhood | Prevalence | Male | Japan | MS | -0.99<br>(-3.76 to 1.86) | 0.478 |
| Childhood | Prevalence | Both | Japan | Psoriasis | -0.07<br>(-0.87 to 0.73) | 0.857 |
| Childhood | Prevalence | Female | Japan | Psoriasis | 0.05<br>(-0.61 to 0.72) | 0.872 |
| Childhood | Prevalence | Male | Japan | Psoriasis | -0.01<br>(-0.93 to 0.92) | 0.988 |
| Childhood | Prevalence | Both | Japan | RHD | -1.65<br>(-2.53 to -0.78) | < 0.001 |
| Childhood | Prevalence | Female | Japan | RHD | -2.31<br>(-2.73 to -1.89) | < 0.001 |
| Childhood | Prevalence | Male | Japan | RHD | -1.56<br>(-2.28 to -0.82) | < 0.001 |
| Childhood | Prevalence | Both | Jordan | MS | 0<br>(-1.78 to 1.81) | 0.999 |
| Childhood | Prevalence | Female | Jordan | MS | -0.35<br>(-2.05 to 1.39) | 0.685 |
| Childhood | Prevalence | Male | Jordan | MS | -0.02<br>(-1.83 to 1.83) | 0.986 |
| Childhood | Prevalence | Both | Jordan | Psoriasis | -0.88<br>(-1.67 to -0.09) | 0.030 |
| Childhood | Prevalence | Female | Jordan | Psoriasis | -1.05<br>(-1.8 to -0.29) | 0.008 |
| Childhood | Prevalence | Male | Jordan | Psoriasis | -0.89<br>(-1.72 to -0.05) | 0.038 |

| | | | | | | |
|---|---|---|---|---|---|---|
| Childhood | Prevalence | Both | Jordan | RHD | -1.41 (-4.14 to 1.39) | 0.308 |
| Childhood | Prevalence | Female | Jordan | RHD | -1.27 (-3.87 to 1.41) | 0.338 |
| Childhood | Prevalence | Male | Jordan | RHD | -0.8 (-3.63 to 2.11) | 0.574 |
| Childhood | Prevalence | Both | Jordan | RA | 0.89 (-0.61 to 2.41) | 0.234 |
| Childhood | Prevalence | Female | Jordan | RA | 1.42 (-0.09 to 2.95) | 0.064 |
| Childhood | Prevalence | Male | Jordan | RA | 0.99 (-0.46 to 2.46) | 0.173 |
| Childhood | Prevalence | Both | Kazakhstan | T1D | 1.29 (-0.04 to 2.64) | 0.057 |
| Childhood | Prevalence | Female | Kazakhstan | T1D | 0.89 (-0.38 to 2.17) | 0.164 |
| Childhood | Prevalence | Male | Kazakhstan | T1D | 1.3 (-0.12 to 2.74) | 0.070 |
| Childhood | Prevalence | Both | Kazakhstan | IBD | -0.77 (-6.11 to 4.86) | 0.775 |
| Childhood | Prevalence | Female | Kazakhstan | IBD | 0.36 (-4.82 to 5.83) | 0.889 |
| Childhood | Prevalence | Male | Kazakhstan | IBD | -0.82 (-6.36 to 5.05) | 0.771 |
| Childhood | Prevalence | Both | Kazakhstan | MS | 0.63 (-1.13 to 2.42) | 0.475 |
| Childhood | Prevalence | Female | Kazakhstan | MS | 0.34 (-1.57 to 2.28) | 0.721 |
| Childhood | Prevalence | Male | Kazakhstan | MS | 0.44 (-1.12 to 2.03) | 0.568 |
| Childhood | Prevalence | Both | Kenya | IBD | 0.41 (-1.28 to 2.13) | 0.622 |
| Childhood | Prevalence | Female | Kenya | IBD | 0.49 | 0.590 |

| | | | | | | |
|---|---|---|---|---|---|---|
| | | | | | (-1.34 to 2.36) | |
| Childhood | Prevalence | Male | Kenya | IBD | 0.34<br>(-1.21 to 1.92) | 0.656 |
| Childhood | Prevalence | Both | Kenya | Psoriasis | -0.26<br>(-0.75 to 0.23) | 0.278 |
| Childhood | Prevalence | Female | Kenya | Psoriasis | -0.17<br>(-0.65 to 0.31) | 0.471 |
| Childhood | Prevalence | Male | Kenya | Psoriasis | -0.22<br>(-0.71 to 0.28) | 0.376 |
| Childhood | Prevalence | Both | Kenya | RHD | 0.19<br>(0.08 to 0.3) | 0.001 |
| Childhood | Prevalence | Female | Kenya | RHD | 0.24<br>(0.14 to 0.34) | < 0.001 |
| Childhood | Prevalence | Male | Kenya | RHD | 0.15<br>(0.04 to 0.26) | 0.009 |
| Childhood | Prevalence | Both | Kiribati | AA | -0.57<br>(-3.36 to 2.31) | 0.686 |
| Childhood | Prevalence | Female | Kiribati | AA | 0.28<br>(-2.51 to 3.15) | 0.842 |
| Childhood | Prevalence | Male | Kiribati | AA | -0.58<br>(-3.33 to 2.25) | 0.675 |
| Childhood | Prevalence | Both | Kiribati | IBD | -0.61<br>(-2.28 to 1.09) | 0.466 |
| Childhood | Prevalence | Female | Kiribati | IBD | -1.15<br>(-2.59 to 0.31) | 0.116 |
| Childhood | Prevalence | Male | Kiribati | IBD | -0.47<br>(-2.43 to 1.53) | 0.632 |
| Childhood | Prevalence | Both | Kiribati | RA | 0.36<br>(-0.99 to 1.73) | 0.590 |
| Childhood | Prevalence | Female | Kiribati | RA | 0.02<br>(-1.36 to 1.42) | 0.976 |
| Childhood | Prevalence | Male | Kiribati | RA | 0.24<br>(-1.09 to 1.58) | 0.717 |

| | | | | | | |
|---|---|---|---|---|---|---|
| Childhood | Prevalence | Both | Kuwait | AA | 0.23 (0.05 to 0.42) | 0.016 |
| Childhood | Prevalence | Female | Kuwait | AA | -0.04 (-0.29 to 0.22) | 0.762 |
| Childhood | Prevalence | Male | Kuwait | AA | 0.03 (-0.19 to 0.26) | 0.762 |
| Childhood | Prevalence | Both | Kuwait | IBD | 0.24 (-1.55 to 2.06) | 0.789 |
| Childhood | Prevalence | Female | Kuwait | IBD | -0.55 (-2.37 to 1.31) | 0.547 |
| Childhood | Prevalence | Male | Kuwait | IBD | 0.39 (-1.46 to 2.27) | 0.671 |
| Childhood | Prevalence | Both | Kuwait | RA | 0.99 (-0.46 to 2.46) | 0.172 |
| Childhood | Prevalence | Female | Kuwait | RA | 1.11 (-0.4 to 2.65) | 0.145 |
| Childhood | Prevalence | Male | Kuwait | RA | 0.71 (-0.74 to 2.18) | 0.328 |
| Childhood | Prevalence | Both | Kyrgyzstan | AA | -0.02 (-0.17 to 0.13) | 0.793 |
| Childhood | Prevalence | Female | Kyrgyzstan | AA | 0.02 (-0.1 to 0.13) | 0.762 |
| Childhood | Prevalence | Male | Kyrgyzstan | AA | -0.02 (-0.13 to 0.09) | 0.762 |
| Childhood | Prevalence | Both | Kyrgyzstan | IBD | -1.31 (-4.61 to 2.1) | 0.434 |
| Childhood | Prevalence | Female | Kyrgyzstan | IBD | -0.5 (-3.7 to 2.81) | 0.755 |
| Childhood | Prevalence | Male | Kyrgyzstan | IBD | -1.2 (-4.7 to 2.42) | 0.497 |
| Childhood | Prevalence | Both | Kyrgyzstan | Psoriasis | -0.92 (-2.51 to 0.69) | 0.249 |
| Childhood | Prevalence | Female | Kyrgyzstan | Psoriasis | -0.57 | 0.468 |

| | | | | | | |
|---|---|---|---|---|---|---|
| | | | | | (-2.15 to 1.03) | |
| Childhood | Prevalence | Male | Kyrgyzstan | Psoriasis | -0.85<br>(-2.44 to 0.77) | 0.292 |
| Childhood | Prevalence | Both | Kyrgyzstan | RA | 0.79<br>(-0.92 to 2.52) | 0.356 |
| Childhood | Prevalence | Female | Kyrgyzstan | RA | 1.13<br>(-0.68 to 2.98) | 0.213 |
| Childhood | Prevalence | Male | Kyrgyzstan | RA | 0.69<br>(-0.66 to 2.07) | 0.305 |
| Childhood | Prevalence | Both | Lao People's Democratic Republic | T1D | 0.27<br>(-1.1 to 1.65) | 0.693 |
| Childhood | Prevalence | Female | Lao People's Democratic Republic | T1D | 0.55<br>(-0.68 to 1.79) | 0.372 |
| Childhood | Prevalence | Male | Lao People's Democratic Republic | T1D | 0.28<br>(-1.21 to 1.79) | 0.703 |
| Childhood | Prevalence | Both | Lao People's Democratic Republic | Psoriasis | -0.81<br>(-1.78 to 0.17) | 0.101 |
| Childhood | Prevalence | Female | Lao People's Democratic Republic | Psoriasis | -0.58<br>(-1.32 to 0.16) | 0.116 |
| Childhood | Prevalence | Male | Lao People's Democratic Republic | Psoriasis | -0.85<br>(-2.06 to 0.36) | 0.161 |
| Childhood | Prevalence | Both | Latvia | IBD | 0.23<br>(-1.08 to 1.55) | 0.728 |
| Childhood | Prevalence | Female | Latvia | IBD | 0.58<br>(-0.63 to 1.8) | 0.334 |
| Childhood | Prevalence | Male | Latvia | IBD | 0.23<br>(-1.18 to 1.67) | 0.740 |
| Childhood | Prevalence | Both | Latvia | MS | -0.26<br>(-1.78 to 1.29) | 0.735 |
| Childhood | Prevalence | Female | Latvia | MS | 0.2<br>(-1.28 to 1.7) | 0.785 |
| Childhood | Prevalence | Male | Latvia | MS | -0.23<br>(-1.81 to 1.36) | 0.765 |

| | | | | | | |
|---|---|---|---|---|---|---|
| Childhood | Prevalence | Both | Latvia | Psoriasis | -0.82<br>(-1.55 to -0.1) | 0.028 |
| Childhood | Prevalence | Female | Latvia | Psoriasis | -0.7<br>(-1.2 to -0.19) | 0.009 |
| Childhood | Prevalence | Male | Latvia | Psoriasis | -0.85<br>(-1.81 to 0.11) | 0.081 |
| Childhood | Prevalence | Both | Latvia | RHD | -2.12<br>(-5.48 to 1.36) | 0.220 |
| Childhood | Prevalence | Female | Latvia | RHD | -2.36<br>(-5.89 to 1.3) | 0.194 |
| Childhood | Prevalence | Male | Latvia | RHD | -2.23<br>(-5.24 to 0.88) | 0.151 |
| Childhood | Prevalence | Both | Lebanon | T1D | 1.47<br>(-0.54 to 3.52) | 0.147 |
| Childhood | Prevalence | Female | Lebanon | T1D | 1.98<br>(0 to 4) | 0.050 |
| Childhood | Prevalence | Male | Lebanon | T1D | 1.53<br>(-0.52 to 3.63) | 0.139 |
| Childhood | Prevalence | Both | Lebanon | Psoriasis | -1.17<br>(-1.98 to -0.36) | 0.006 |
| Childhood | Prevalence | Female | Lebanon | Psoriasis | -1.01<br>(-1.77 to -0.24) | 0.012 |
| Childhood | Prevalence | Male | Lebanon | Psoriasis | -1.15<br>(-2 to -0.3) | 0.010 |
| Childhood | Prevalence | Both | Lebanon | RHD | -0.45<br>(-1.14 to 0.26) | 0.203 |
| Childhood | Prevalence | Female | Lebanon | RHD | -0.36<br>(-1.03 to 0.32) | 0.290 |
| Childhood | Prevalence | Male | Lebanon | RHD | -0.48<br>(-1.23 to 0.28) | 0.206 |
| Childhood | Prevalence | Both | Lebanon | RA | 0.99<br>(-0.52 to 2.53) | 0.191 |
| Childhood | Prevalence | Female | Lebanon | RA | 1.42 | 0.063 |

| | | | | | | |
|---|---|---|---|---|---|---|
| | | | | | (-0.08 to 2.95) | |
| Childhood | Prevalence | Male | Lebanon | RA | 0.91<br>(-0.55 to 2.4) | 0.213 |
| Childhood | Prevalence | Both | Lesotho | AA | 0.04<br>(-0.2 to 0.29) | 0.715 |
| Childhood | Prevalence | Female | Lesotho | AA | -0.04<br>(-0.28 to 0.21) | 0.762 |
| Childhood | Prevalence | Male | Lesotho | AA | 0.04<br>(-0.2 to 0.27) | 0.762 |
| Childhood | Prevalence | Both | Lesotho | MS | -0.02<br>(-1.73 to 1.72) | 0.981 |
| Childhood | Prevalence | Female | Lesotho | MS | 0.4<br>(-1.3 to 2.13) | 0.639 |
| Childhood | Prevalence | Male | Lesotho | MS | 0.05<br>(-1.71 to 1.83) | 0.957 |
| Childhood | Prevalence | Both | Lesotho | Psoriasis | -0.57<br>(-1.6 to 0.46) | 0.263 |
| Childhood | Prevalence | Female | Lesotho | Psoriasis | -0.84<br>(-1.86 to 0.18) | 0.102 |
| Childhood | Prevalence | Male | Lesotho | Psoriasis | -0.55<br>(-1.58 to 0.48) | 0.280 |
| Childhood | Prevalence | Both | Liberia | AA | -0.03<br>(-0.21 to 0.15) | 0.723 |
| Childhood | Prevalence | Female | Liberia | AA | 0.05<br>(-0.09 to 0.18) | 0.487 |
| Childhood | Prevalence | Male | Liberia | AA | 0.01<br>(-0.13 to 0.15) | 0.916 |
| Childhood | Prevalence | Both | Liberia | IBD | 0.9<br>(-0.94 to 2.78) | 0.325 |
| Childhood | Prevalence | Female | Liberia | IBD | 0.39<br>(-1.58 to 2.41) | 0.689 |
| Childhood | Prevalence | Male | Liberia | IBD | 0.86<br>(-0.87 to 2.63) | 0.319 |

| | | | | | | |
|---|---|---|---|---|---|---|
| Childhood | Prevalence | Both | Liberia | MS | 0.46<br>(-1.25 to 2.2) | 0.591 |
| Childhood | Prevalence | Female | Liberia | MS | 0<br>(-1.76 to 1.8) | 0.995 |
| Childhood | Prevalence | Male | Liberia | MS | 0.52<br>(-1.25 to 2.32) | 0.553 |
| Childhood | Prevalence | Both | Liberia | RHD | 0.11<br>(-0.36 to 0.59) | 0.625 |
| Childhood | Prevalence | Female | Liberia | RHD | 0.25<br>(-0.28 to 0.79) | 0.337 |
| Childhood | Prevalence | Male | Liberia | RHD | 0.11<br>(-0.31 to 0.54) | 0.582 |
| Childhood | Prevalence | Both | Liberia | RA | -0.15<br>(-4.29 to 4.16) | 0.942 |
| Childhood | Prevalence | Female | Liberia | RA | 0.7<br>(-3.25 to 4.81) | 0.724 |
| Childhood | Prevalence | Male | Liberia | RA | -0.18<br>(-4.84 to 4.7) | 0.938 |
| Childhood | Prevalence | Both | Libya | AA | -0.17<br>(-1.61 to 1.29) | 0.808 |
| Childhood | Prevalence | Female | Libya | AA | 0.22<br>(-1.25 to 1.72) | 0.762 |
| Childhood | Prevalence | Male | Libya | AA | -0.21<br>(-1.63 to 1.23) | 0.762 |
| Childhood | Prevalence | Both | Libya | T1D | 2.02<br>(0.45 to 3.61) | 0.013 |
| Childhood | Prevalence | Female | Libya | T1D | 2.44<br>(0.86 to 4.05) | 0.004 |
| Childhood | Prevalence | Male | Libya | T1D | 2.12<br>(0.52 to 3.73) | 0.011 |
| Childhood | Prevalence | Both | Libya | IBD | 0.93<br>(-0.86 to 2.75) | 0.299 |
| Childhood | Prevalence | Female | Libya | IBD | 0.5 | 0.603 |

| | | | | | | |
|---|---|---|---|---|---|---|
| | | | | | (-1.42 to 2.45) | |
| Childhood | Prevalence | Male | Libya | IBD | 0.84<br>(-0.87 to 2.57) | 0.327 |
| Childhood | Prevalence | Both | Libya | MS | 1.23<br>(-0.44 to 2.94) | 0.143 |
| Childhood | Prevalence | Female | Libya | MS | 0.63<br>(-1.02 to 2.3) | 0.445 |
| Childhood | Prevalence | Male | Libya | MS | 1.26<br>(-0.49 to 3.05) | 0.152 |
| Childhood | Prevalence | Both | Libya | Psoriasis | -1.02<br>(-2.33 to 0.31) | 0.125 |
| Childhood | Prevalence | Female | Libya | Psoriasis | -1.32<br>(-2.52 to -0.1) | 0.035 |
| Childhood | Prevalence | Male | Libya | Psoriasis | -1.04<br>(-2.46 to 0.41) | 0.151 |
| Childhood | Prevalence | Both | Libya | RHD | 0.44<br>(-0.03 to 0.91) | 0.067 |
| Childhood | Prevalence | Female | Libya | RHD | 0.3<br>(-0.21 to 0.83) | 0.240 |
| Childhood | Prevalence | Male | Libya | RHD | 0.4<br>(-0.03 to 0.83) | 0.069 |
| Childhood | Prevalence | Both | Libya | RA | 0.2<br>(-1.77 to 2.2) | 0.840 |
| Childhood | Prevalence | Female | Libya | RA | 0.61<br>(-1.35 to 2.61) | 0.533 |
| Childhood | Prevalence | Male | Libya | RA | 0.03<br>(-2 to 2.1) | 0.978 |
| Childhood | Prevalence | Both | Lithuania | AA | -1.02<br>(-2.98 to 0.97) | 0.300 |
| Childhood | Prevalence | Female | Lithuania | AA | -0.4<br>(-2.32 to 1.55) | 0.674 |
| Childhood | Prevalence | Male | Lithuania | AA | -1.01<br>(-2.93 to 0.95) | 0.296 |

| | | | | | | |
|---|---|---|---|---|---|---|
| Childhood | Prevalence | Both | Lithuania | IBD | 0.67 (-0.65 to 2.02) | 0.308 |
| Childhood | Prevalence | Female | Lithuania | IBD | 0.89 (-0.27 to 2.07) | 0.126 |
| Childhood | Prevalence | Male | Lithuania | IBD | 0.64 (-0.84 to 2.15) | 0.384 |
| Childhood | Prevalence | Both | Lithuania | MS | 0.06 (-0.12 to 0.25) | 0.496 |
| Childhood | Prevalence | Female | Lithuania | MS | 0.22 (0.15 to 0.3) | < 0.001 |
| Childhood | Prevalence | Male | Lithuania | MS | -0.12 (-0.42 to 0.19) | 0.439 |
| Childhood | Prevalence | Both | Low SDI | AA | -0.02 (-0.25 to 0.21) | 0.846 |
| Childhood | Prevalence | Female | Low SDI | AA | 0.04 (-0.2 to 0.29) | 0.723 |
| Childhood | Prevalence | Male | Low SDI | AA | -0.03 (-0.27 to 0.21) | 0.814 |
| Childhood | Prevalence | Both | Low SDI | T1D | -0.05 (-2.73 to 2.7) | 0.969 |
| Childhood | Prevalence | Female | Low SDI | T1D | 0.64 (-2 to 3.35) | 0.628 |
| Childhood | Prevalence | Male | Low SDI | T1D | -0.07 (-2.8 to 2.74) | 0.960 |
| Childhood | Prevalence | Both | Low SDI | MS | 0.58 (0.25 to 0.91) | 0.001 |
| Childhood | Prevalence | Female | Low SDI | MS | 0.61 (0.28 to 0.94) | 0.001 |
| Childhood | Prevalence | Male | Low SDI | MS | 0.6 (0.26 to 0.94) | 0.001 |
| Childhood | Prevalence | Both | Low SDI | Psoriasis | -0.36 (-1.69 to 0.98) | 0.583 |
| Childhood | Prevalence | Female | Low SDI | Psoriasis | -0.76 | 0.258 |

| | | | | | | |
|---|---|---|---|---|---|---|
| | | | | | (-2.09 to 0.59) | |
| Childhood | Prevalence | Male | Low SDI | Psoriasis | -0.36<br>(-1.68 to 0.98) | 0.587 |
| Childhood | Prevalence | Both | Low SDI | RHD | 0.23<br>(0.19 to 0.27) | < 0.001 |
| Childhood | Prevalence | Female | Low SDI | RHD | 0.22<br>(0.16 to 0.27) | < 0.001 |
| Childhood | Prevalence | Male | Low SDI | RHD | 0.21<br>(0.12 to 0.3) | < 0.001 |
| Childhood | Prevalence | Both | Low SDI | RA | 0.23<br>(-0.94 to 1.42) | 0.691 |
| Childhood | Prevalence | Female | Low SDI | RA | 0.53<br>(-0.62 to 1.69) | 0.354 |
| Childhood | Prevalence | Male | Low SDI | RA | 0.27<br>(-0.97 to 1.52) | 0.662 |
| Childhood | Prevalence | Both | Low-middle SDI | MS | 0.37<br>(-1.33 to 2.1) | 0.663 |
| Childhood | Prevalence | Female | Low-middle SDI | MS | 0.81<br>(-0.88 to 2.54) | 0.336 |
| Childhood | Prevalence | Male | Low-middle SDI | MS | 0.34<br>(-1.4 to 2.11) | 0.693 |
| Childhood | Prevalence | Both | Low-middle SDI | Psoriasis | -0.63<br>(-1.49 to 0.24) | 0.151 |
| Childhood | Prevalence | Female | Low-middle SDI | Psoriasis | -0.42<br>(-1.21 to 0.38) | 0.294 |
| Childhood | Prevalence | Male | Low-middle SDI | Psoriasis | -0.62<br>(-1.55 to 0.32) | 0.187 |
| Childhood | Prevalence | Both | Low-middle SDI | RHD | 0.15<br>(-2.13 to 2.49) | 0.895 |
| Childhood | Prevalence | Female | Low-middle SDI | RHD | -0.42<br>(-2.64 to 1.85) | 0.704 |
| Childhood | Prevalence | Male | Low-middle SDI | RHD | 0.03<br>(-2.3 to 2.41) | 0.979 |

| | | | | | | |
|---|---|---|---|---|---|---|
| Childhood | Prevalence | Both | Low-middle SDI | RA | 0.35<br>(-0.76 to 1.47) | 0.524 |
| Childhood | Prevalence | Female | Low-middle SDI | RA | 0.61<br>(-0.54 to 1.77) | 0.287 |
| Childhood | Prevalence | Male | Low-middle SDI | RA | 0.39<br>(-0.69 to 1.49) | 0.464 |
| Childhood | Prevalence | Both | Luxembourg | AA | -0.04<br>(-0.26 to 0.18) | 0.723 |
| Childhood | Prevalence | Female | Luxembourg | AA | 0.08<br>(-0.46 to 0.62) | 0.762 |
| Childhood | Prevalence | Male | Luxembourg | AA | 0.04<br>(-0.2 to 0.27) | 0.762 |
| Childhood | Prevalence | Both | Luxembourg | IBD | 0.53<br>(-0.95 to 2.04) | 0.472 |
| Childhood | Prevalence | Female | Luxembourg | IBD | 0.29<br>(-1.1 to 1.71) | 0.671 |
| Childhood | Prevalence | Male | Luxembourg | IBD | 0.47<br>(-1.13 to 2.09) | 0.558 |
| Childhood | Prevalence | Both | Luxembourg | MS | 0.94<br>(-0.72 to 2.64) | 0.257 |
| Childhood | Prevalence | Female | Luxembourg | MS | 1.22<br>(-0.44 to 2.91) | 0.143 |
| Childhood | Prevalence | Male | Luxembourg | MS | 0.72<br>(-0.98 to 2.46) | 0.395 |
| Childhood | Prevalence | Both | Luxembourg | Psoriasis | -0.42<br>(-1.56 to 0.74) | 0.466 |
| Childhood | Prevalence | Female | Luxembourg | Psoriasis | -0.17<br>(-1.12 to 0.78) | 0.712 |
| Childhood | Prevalence | Male | Luxembourg | Psoriasis | -0.4<br>(-1.72 to 0.94) | 0.545 |
| Childhood | Prevalence | Both | Luxembourg | RHD | -1.26<br>(-5.63 to 3.31) | 0.571 |
| Childhood | Prevalence | Female | Luxembourg | RHD | -2.03 | 0.373 |

| | | | | | | |
|---|---|---|---|---|---|---|
| | | | | | (-6.48 to 2.62) | |
| Childhood | Prevalence | Male | Luxembourg | RHD | -1.05<br>(-5.18 to 3.26) | 0.617 |
| Childhood | Prevalence | Both | Luxembourg | RA | 0.11<br>(-2.3 to 2.58) | 0.928 |
| Childhood | Prevalence | Female | Luxembourg | RA | -0.36<br>(-2.71 to 2.04) | 0.759 |
| Childhood | Prevalence | Male | Luxembourg | RA | 0.16<br>(-2.29 to 2.67) | 0.896 |
| Childhood | Prevalence | Both | Madagascar | AA | 0.04<br>(-0.21 to 0.28) | 0.762 |
| Childhood | Prevalence | Female | Madagascar | AA | -0.04<br>(-0.3 to 0.22) | 0.762 |
| Childhood | Prevalence | Male | Madagascar | AA | 0.03<br>(-0.2 to 0.27) | 0.763 |
| Childhood | Prevalence | Both | Madagascar | IBD | 0.21<br>(-1.62 to 2.07) | 0.817 |
| Childhood | Prevalence | Female | Madagascar | IBD | 0.75<br>(-1.18 to 2.72) | 0.434 |
| Childhood | Prevalence | Male | Madagascar | IBD | 0.21<br>(-1.5 to 1.94) | 0.808 |
| Childhood | Prevalence | Both | Madagascar | MS | -1.1<br>(-5.2 to 3.18) | 0.598 |
| Childhood | Prevalence | Female | Madagascar | MS | -0.01<br>(-4.27 to 4.44) | 0.996 |
| Childhood | Prevalence | Male | Madagascar | MS | -0.98<br>(-4.98 to 3.18) | 0.627 |
| Childhood | Prevalence | Both | Malawi | AA | -0.14<br>(-0.77 to 0.5) | 0.664 |
| Childhood | Prevalence | Female | Malawi | AA | 0.03<br>(-0.62 to 0.69) | 0.921 |
| Childhood | Prevalence | Male | Malawi | AA | -0.15<br>(-0.76 to 0.47) | 0.626 |

| | | | | | | |
|---|---|---|---|---|---|---|
| Childhood | Prevalence | Both | Malawi | T1D | -0.1<br>(-1.87 to 1.69) | 0.906 |
| Childhood | Prevalence | Female | Malawi | T1D | 0.41<br>(-1.38 to 2.23) | 0.646 |
| Childhood | Prevalence | Male | Malawi | T1D | -0.1<br>(-1.84 to 1.67) | 0.911 |
| Childhood | Prevalence | Both | Malawi | Psoriasis | -0.57<br>(-1.93 to 0.8) | 0.399 |
| Childhood | Prevalence | Female | Malawi | Psoriasis | -0.27<br>(-1.64 to 1.12) | 0.692 |
| Childhood | Prevalence | Male | Malawi | Psoriasis | -0.51<br>(-1.87 to 0.87) | 0.455 |
| Childhood | Prevalence | Both | Malawi | RHD | -1.12<br>(-2.91 to 0.7) | 0.216 |
| Childhood | Prevalence | Female | Malawi | RHD | -0.6<br>(-2.45 to 1.28) | 0.516 |
| Childhood | Prevalence | Male | Malawi | RHD | -1.08<br>(-2.8 to 0.66) | 0.212 |
| Childhood | Prevalence | Both | Malaysia | AA | 0.8<br>(0.1 to 1.51) | 0.027 |
| Childhood | Prevalence | Female | Malaysia | AA | 0.75<br>(0.06 to 1.45) | 0.035 |
| Childhood | Prevalence | Male | Malaysia | AA | 0.83<br>(0.12 to 1.54) | 0.024 |
| Childhood | Prevalence | Both | Malaysia | MS | -0.05<br>(-1.69 to 1.62) | 0.955 |
| Childhood | Prevalence | Female | Malaysia | MS | 0.38<br>(-1.26 to 2.05) | 0.641 |
| Childhood | Prevalence | Male | Malaysia | MS | 0.02<br>(-1.65 to 1.71) | 0.985 |
| Childhood | Prevalence | Both | Malaysia | Psoriasis | -0.77<br>(-2.12 to 0.6) | 0.259 |
| Childhood | Prevalence | Female | Malaysia | Psoriasis | -0.49 | 0.364 |

| | | | | | | |
|---|---|---|---|---|---|---|
| | | | | | (-1.56 to 0.6) | |
| Childhood | Prevalence | Male | Malaysia | Psoriasis | -0.79<br>(-2.42 to 0.86) | 0.331 |
| Childhood | Prevalence | Both | Malaysia | RA | 0.47<br>(-1 to 1.96) | 0.517 |
| Childhood | Prevalence | Female | Malaysia | RA | 0.94<br>(-0.6 to 2.5) | 0.223 |
| Childhood | Prevalence | Male | Malaysia | RA | 0.37<br>(-1.01 to 1.77) | 0.591 |
| Childhood | Prevalence | Both | Maldives | MS | -0.22<br>(-1.85 to 1.43) | 0.784 |
| Childhood | Prevalence | Female | Maldives | MS | 0.3<br>(-1.35 to 1.97) | 0.715 |
| Childhood | Prevalence | Male | Maldives | MS | -0.03<br>(-1.67 to 1.63) | 0.967 |
| Childhood | Prevalence | Both | Maldives | Psoriasis | -0.96<br>(-3.02 to 1.15) | 0.356 |
| Childhood | Prevalence | Female | Maldives | Psoriasis | -1.35<br>(-2.95 to 0.28) | 0.101 |
| Childhood | Prevalence | Male | Maldives | Psoriasis | -0.88<br>(-3.46 to 1.77) | 0.499 |
| Childhood | Prevalence | Both | Maldives | RA | 0.81<br>(-0.68 to 2.32) | 0.277 |
| Childhood | Prevalence | Female | Maldives | RA | 1.45<br>(-0.06 to 2.98) | 0.059 |
| Childhood | Prevalence | Male | Maldives | RA | 0.89<br>(-0.5 to 2.31) | 0.202 |
| Childhood | Prevalence | Both | Mali | AA | 0.65<br>(0.08 to 1.22) | 0.027 |
| Childhood | Prevalence | Female | Mali | AA | 0.68<br>(0.11 to 1.26) | 0.022 |
| Childhood | Prevalence | Male | Mali | AA | 0.7<br>(0.14 to 1.27) | 0.017 |

| | | | | | | |
|---|---|---|---|---|---|---|
| Childhood | Prevalence | Both | Mali | IBD | 0.62<br>(-1.67 to 2.97) | 0.584 |
| Childhood | Prevalence | Female | Mali | IBD | 0.08<br>(-2.04 to 2.26) | 0.936 |
| Childhood | Prevalence | Male | Mali | IBD | 0.64<br>(-1.84 to 3.18) | 0.607 |
| Childhood | Prevalence | Both | Mali | MS | 1.09<br>(0.16 to 2.03) | 0.023 |
| Childhood | Prevalence | Female | Mali | MS | 1.64<br>(0.64 to 2.64) | 0.002 |
| Childhood | Prevalence | Male | Mali | MS | 1.1<br>(0.14 to 2.07) | 0.026 |
| Childhood | Prevalence | Both | Mali | Psoriasis | -0.83<br>(-2.4 to 0.76) | 0.293 |
| Childhood | Prevalence | Female | Mali | Psoriasis | -1.25<br>(-2.79 to 0.32) | 0.115 |
| Childhood | Prevalence | Male | Mali | Psoriasis | -0.82<br>(-2.39 to 0.78) | 0.303 |
| Childhood | Prevalence | Both | Mali | RHD | 0.08<br>(0.01 to 0.15) | 0.027 |
| Childhood | Prevalence | Female | Mali | RHD | 0.15<br>(0.07 to 0.23) | 0.001 |
| Childhood | Prevalence | Male | Mali | RHD | 0.04<br>(-0.02 to 0.1) | 0.200 |
| Childhood | Prevalence | Both | Malta | IBD | 0.24<br>(-0.82 to 1.3) | 0.651 |
| Childhood | Prevalence | Female | Malta | IBD | -0.02<br>(-0.78 to 0.75) | 0.959 |
| Childhood | Prevalence | Male | Malta | IBD | 0.35<br>(-1.05 to 1.76) | 0.615 |
| Childhood | Prevalence | Both | Malta | MS | 1.24<br>(-0.62 to 3.13) | 0.185 |
| Childhood | Prevalence | Female | Malta | MS | 0.83 | 0.357 |

| | | | | | | |
|---|---|---|---|---|---|---|
| | | | | | (-0.97 to 2.65) | |
| Childhood | Prevalence | Male | Malta | MS | 1.39<br>(-0.5 to 3.32) | 0.143 |
| Childhood | Prevalence | Both | Malta | Psoriasis | -0.62<br>(-1.49 to 0.25) | 0.155 |
| Childhood | Prevalence | Female | Malta | Psoriasis | -0.45<br>(-1.18 to 0.27) | 0.210 |
| Childhood | Prevalence | Male | Malta | Psoriasis | -0.62<br>(-1.63 to 0.4) | 0.223 |
| Childhood | Prevalence | Both | Malta | RA | 0.32<br>(-1.86 to 2.55) | 0.770 |
| Childhood | Prevalence | Female | Malta | RA | -0.14<br>(-2.24 to 2.01) | 0.895 |
| Childhood | Prevalence | Male | Malta | RA | 0.49<br>(-1.76 to 2.79) | 0.664 |
| Childhood | Prevalence | Both | Marshall Islands | AA | -0.01<br>(-0.08 to 0.06) | 0.741 |
| Childhood | Prevalence | Female | Marshall Islands | AA | -0.01<br>(-0.09 to 0.07) | 0.825 |
| Childhood | Prevalence | Male | Marshall Islands | AA | -0.03<br>(-0.11 to 0.05) | 0.437 |
| Childhood | Prevalence | Both | Marshall Islands | IBD | -0.49<br>(-2.08 to 1.11) | 0.532 |
| Childhood | Prevalence | Female | Marshall Islands | IBD | -0.12<br>(-1.9 to 1.69) | 0.893 |
| Childhood | Prevalence | Male | Marshall Islands | IBD | -0.41<br>(-1.82 to 1.01) | 0.555 |
| Childhood | Prevalence | Both | Marshall Islands | MS | -0.13<br>(-1.71 to 1.49) | 0.874 |
| Childhood | Prevalence | Female | Marshall Islands | MS | 0.26<br>(-1.36 to 1.91) | 0.743 |
| Childhood | Prevalence | Male | Marshall Islands | MS | -0.14<br>(-1.71 to 1.45) | 0.853 |

| | | | | | | |
|---|---|---|---|---|---|---|
| Childhood | Prevalence | Both | Marshall Islands | Psoriasis | -0.49 (-1.88 to 0.92) | 0.482 |
| Childhood | Prevalence | Female | Marshall Islands | Psoriasis | -0.74 (-1.82 to 0.35) | 0.176 |
| Childhood | Prevalence | Male | Marshall Islands | Psoriasis | -0.51 (-2.18 to 1.18) | 0.538 |
| Childhood | Prevalence | Both | Marshall Islands | RHD | -0.07 (-1.18 to 1.06) | 0.904 |
| Childhood | Prevalence | Female | Marshall Islands | RHD | 0.22 (-0.86 to 1.32) | 0.679 |
| Childhood | Prevalence | Male | Marshall Islands | RHD | -0.08 (-1.23 to 1.08) | 0.891 |
| Childhood | Prevalence | Both | Marshall Islands | RA | 0.58 (-0.89 to 2.08) | 0.425 |
| Childhood | Prevalence | Female | Marshall Islands | RA | 0.14 (-1.36 to 1.67) | 0.849 |
| Childhood | Prevalence | Male | Marshall Islands | RA | 0.41 (-1 to 1.83) | 0.557 |
| Childhood | Prevalence | Both | Mauritania | T1D | 0.59 (-0.32 to 1.52) | 0.196 |
| Childhood | Prevalence | Female | Mauritania | T1D | 0.34 (-0.59 to 1.28) | 0.456 |
| Childhood | Prevalence | Male | Mauritania | T1D | 0.58 (-0.31 to 1.48) | 0.194 |
| Childhood | Prevalence | Both | Mauritania | MS | 0.55 (-0.85 to 1.97) | 0.428 |
| Childhood | Prevalence | Female | Mauritania | MS | 0.17 (-1.26 to 1.63) | 0.808 |
| Childhood | Prevalence | Male | Mauritania | MS | 0.51 (-0.93 to 1.97) | 0.478 |
| Childhood | Prevalence | Both | Mauritania | RHD | 0.02 (-0.14 to 0.17) | 0.830 |
| Childhood | Prevalence | Female | Mauritania | RHD | 0.09 | 0.266 |

| | | | | | | |
|---|---|---|---|---|---|---|
| | | | | | (-0.07 to 0.26) | |
| Childhood | Prevalence | Male | Mauritania | RHD | -0.02 (-0.16 to 0.12) | 0.800 |
| Childhood | Prevalence | Both | Mauritania | RA | 0.61 (-0.6 to 1.84) | 0.309 |
| Childhood | Prevalence | Female | Mauritania | RA | 0.91 (-0.46 to 2.3) | 0.187 |
| Childhood | Prevalence | Male | Mauritania | RA | 0.67 (-0.31 to 1.66) | 0.174 |
| Childhood | Prevalence | Both | Mauritius | AA | 0.03 (-0.23 to 0.29) | 0.806 |
| Childhood | Prevalence | Female | Mauritius | AA | -0.04 (-0.31 to 0.23) | 0.762 |
| Childhood | Prevalence | Male | Mauritius | AA | 0.04 (-0.21 to 0.29) | 0.762 |
| Childhood | Prevalence | Both | Mauritius | IBD | 0.43 (-1.05 to 1.94) | 0.558 |
| Childhood | Prevalence | Female | Mauritius | IBD | 0.77 (-0.84 to 2.4) | 0.337 |
| Childhood | Prevalence | Male | Mauritius | IBD | 0.51 (-0.81 to 1.84) | 0.441 |
| Childhood | Prevalence | Both | Mauritius | MS | -0.18 (-1.83 to 1.5) | 0.826 |
| Childhood | Prevalence | Female | Mauritius | MS | 0.31 (-1.34 to 1.99) | 0.704 |
| Childhood | Prevalence | Male | Mauritius | MS | -0.12 (-1.8 to 1.59) | 0.889 |
| Childhood | Prevalence | Both | Mauritius | Psoriasis | -0.66 (-2.74 to 1.47) | 0.530 |
| Childhood | Prevalence | Female | Mauritius | Psoriasis | -1.04 (-2.71 to 0.65) | 0.216 |
| Childhood | Prevalence | Male | Mauritius | Psoriasis | -0.65 (-3.25 to 2.02) | 0.620 |

| | | | | | | |
|---|---|---|---|---|---|---|
| Childhood | Prevalence | Both | Mauritius | RHD | -0.06 (-0.78 to 0.67) | 0.876 |
| Childhood | Prevalence | Female | Mauritius | RHD | -0.22 (-0.94 to 0.51) | 0.536 |
| Childhood | Prevalence | Male | Mauritius | RHD | -0.09 (-0.78 to 0.62) | 0.803 |
| Childhood | Prevalence | Both | Mauritius | RA | 0.71 (-0.33 to 1.77) | 0.174 |
| Childhood | Prevalence | Female | Mauritius | RA | 1.12 (0.09 to 2.16) | 0.033 |
| Childhood | Prevalence | Male | Mauritius | RA | 0.55 (-0.42 to 1.52) | 0.258 |
| Childhood | Prevalence | Both | Mexico | AA | -0.02 (-0.27 to 0.23) | 0.853 |
| Childhood | Prevalence | Female | Mexico | AA | 0.04 (-0.21 to 0.28) | 0.762 |
| Childhood | Prevalence | Male | Mexico | AA | -0.04 (-0.28 to 0.21) | 0.762 |
| Childhood | Prevalence | Both | Mexico | IBD | -0.37 (-5.39 to 4.91) | 0.883 |
| Childhood | Prevalence | Female | Mexico | IBD | -1.44 (-6.65 to 4.06) | 0.589 |
| Childhood | Prevalence | Male | Mexico | IBD | -0.47 (-5.33 to 4.64) | 0.847 |
| Childhood | Prevalence | Both | Mexico | MS | 0.86 (-1.2 to 2.96) | 0.401 |
| Childhood | Prevalence | Female | Mexico | MS | 1.38 (-0.68 to 3.47) | 0.183 |
| Childhood | Prevalence | Male | Mexico | MS | 0.88 (-1.14 to 2.94) | 0.383 |
| Childhood | Prevalence | Both | Mexico | RHD | -0.56 (-1.31 to 0.19) | 0.134 |
| Childhood | Prevalence | Female | Mexico | RHD | -0.87 | 0.053 |

| | | | | | | |
|---|---|---|---|---|---|---|
| | | | | | (-1.74 to 0.01) | |
| Childhood | Prevalence | Male | Mexico | RHD | -0.53<br>(-1.09 to 0.04) | 0.066 |
| Childhood | Prevalence | Both | Micronesia (Federated States of) | Psoriasis | -1.12<br>(-3.17 to 0.98) | 0.283 |
| Childhood | Prevalence | Female | Micronesia (Federated States of) | Psoriasis | -0.57<br>(-2.08 to 0.97) | 0.455 |
| Childhood | Prevalence | Male | Micronesia (Federated States of) | Psoriasis | -1.29<br>(-3.9 to 1.39) | 0.330 |
| Childhood | Prevalence | Both | Micronesia (Federated States of) | RHD | 0.13<br>(-0.41 to 0.67) | 0.629 |
| Childhood | Prevalence | Female | Micronesia (Federated States of) | RHD | 0.26<br>(-0.27 to 0.8) | 0.322 |
| Childhood | Prevalence | Male | Micronesia (Federated States of) | RHD | 0.15<br>(-0.41 to 0.7) | 0.591 |
| Childhood | Prevalence | Both | Micronesia (Federated States of) | RA | 0.13<br>(-0.86 to 1.14) | 0.785 |
| Childhood | Prevalence | Female | Micronesia (Federated States of) | RA | 0.48<br>(-0.45 to 1.41) | 0.301 |
| Childhood | Prevalence | Male | Micronesia (Federated States of) | RA | 0.02<br>(-1.15 to 1.19) | 0.976 |
| Childhood | Prevalence | Both | Middle SDI | RHD | 0.08<br>(-0.24 to 0.39) | 0.623 |
| Childhood | Prevalence | Female | Middle SDI | RHD | 0.09<br>(-0.26 to 0.45) | 0.596 |
| Childhood | Prevalence | Male | Middle SDI | RHD | 0.02<br>(-0.24 to 0.28) | 0.884 |
| Childhood | Prevalence | Both | Middle SDI | RA | 0.24<br>(-1.1 to 1.59) | 0.719 |
| Childhood | Prevalence | Female | Middle SDI | RA | 0.55<br>(-0.83 to 1.96) | 0.421 |
| Childhood | Prevalence | Male | Middle SDI | RA | 0.27<br>(-0.99 to 1.54) | 0.669 |

| | | | | | | |
|---|---|---|---|---|---|---|
| Childhood | Prevalence | Both | Monaco | AA | -0.02 (-1.02 to 1) | 0.973 |
| Childhood | Prevalence | Female | Monaco | AA | -0.37 (-1.67 to 0.95) | 0.572 |
| Childhood | Prevalence | Male | Monaco | AA | -0.03 (-0.63 to 0.57) | 0.909 |
| Childhood | Prevalence | Both | Monaco | IBD | -2.08 (-10.29 to 6.88) | 0.627 |
| Childhood | Prevalence | Female | Monaco | IBD | 0.44 (-8.01 to 9.68) | 0.918 |
| Childhood | Prevalence | Male | Monaco | IBD | -2.06 (-10.18 to 6.79) | 0.625 |
| Childhood | Prevalence | Both | Monaco | MS | 0.94 (-0.67 to 2.59) | 0.243 |
| Childhood | Prevalence | Female | Monaco | MS | 0.68 (-0.94 to 2.33) | 0.398 |
| Childhood | Prevalence | Male | Monaco | MS | 0.53 (-1.08 to 2.18) | 0.506 |
| Childhood | Prevalence | Both | Monaco | Psoriasis | -0.48 (-2.07 to 1.14) | 0.549 |
| Childhood | Prevalence | Female | Monaco | Psoriasis | -0.17 (-1.62 to 1.31) | 0.818 |
| Childhood | Prevalence | Male | Monaco | Psoriasis | -0.46 (-2.2 to 1.31) | 0.595 |
| Childhood | Prevalence | Both | Monaco | RHD | -0.38 (-5.15 to 4.63) | 0.875 |
| Childhood | Prevalence | Female | Monaco | RHD | -1.53 (-6.24 to 3.42) | 0.525 |
| Childhood | Prevalence | Male | Monaco | RHD | -0.52 (-5.16 to 4.35) | 0.824 |
| Childhood | Prevalence | Both | Monaco | RA | 0.22 (-1.06 to 1.53) | 0.725 |
| Childhood | Prevalence | Female | Monaco | RA | 0.64 | 0.322 |

| | | | | | | |
|---|---|---|---|---|---|---|
| | | | | | (-0.65 to 1.95) | |
| Childhood | Prevalence | Male | Monaco | RA | 0.23<br>(-1.07 to 1.54) | 0.723 |
| Childhood | Prevalence | Both | Mongolia | MS | 0.15<br>(-1.57 to 1.9) | 0.861 |
| Childhood | Prevalence | Female | Mongolia | MS | 0.68<br>(-1.04 to 2.42) | 0.428 |
| Childhood | Prevalence | Male | Mongolia | MS | 0.1<br>(-1.61 to 1.84) | 0.907 |
| Childhood | Prevalence | Both | Mongolia | Psoriasis | -1.48<br>(-3.32 to 0.39) | 0.116 |
| Childhood | Prevalence | Female | Mongolia | Psoriasis | -1.01<br>(-2.5 to 0.52) | 0.185 |
| Childhood | Prevalence | Male | Mongolia | Psoriasis | -1.52<br>(-3.75 to 0.77) | 0.183 |
| Childhood | Prevalence | Both | Mongolia | RHD | -0.01<br>(-0.16 to 0.14) | 0.863 |
| Childhood | Prevalence | Female | Mongolia | RHD | 0.03<br>(-0.16 to 0.22) | 0.769 |
| Childhood | Prevalence | Male | Mongolia | RHD | -0.02<br>(-0.12 to 0.07) | 0.640 |
| Childhood | Prevalence | Both | Mongolia | RA | 1.01<br>(-0.69 to 2.75) | 0.236 |
| Childhood | Prevalence | Female | Mongolia | RA | 1.49<br>(-0.19 to 3.19) | 0.080 |
| Childhood | Prevalence | Male | Mongolia | RA | 0.87<br>(-0.91 to 2.69) | 0.328 |
| Childhood | Prevalence | Both | Montenegro | AA | -0.34<br>(-2.54 to 1.91) | 0.754 |
| Childhood | Prevalence | Female | Montenegro | AA | 0.33<br>(-1.86 to 2.57) | 0.762 |
| Childhood | Prevalence | Male | Montenegro | AA | -0.33<br>(-2.51 to 1.9) | 0.762 |

| | | | | | | |
|---|---|---|---|---|---|---|
| Childhood | Prevalence | Both | Montenegro | MS | 0.26 (-2.49 to 3.08) | 0.850 |
| Childhood | Prevalence | Female | Montenegro | MS | 0.95 (-1.48 to 3.44) | 0.433 |
| Childhood | Prevalence | Male | Montenegro | MS | -0.29 (-3.56 to 3.1) | 0.862 |
| Childhood | Prevalence | Both | Morocco | T1D | 1.94 (0.8 to 3.08) | 0.002 |
| Childhood | Prevalence | Female | Morocco | T1D | 1.58 (0.49 to 2.68) | 0.006 |
| Childhood | Prevalence | Male | Morocco | T1D | 1.96 (0.79 to 3.14) | 0.002 |
| Childhood | Prevalence | Both | Morocco | IBD | 0.09 (-1.81 to 2.03) | 0.921 |
| Childhood | Prevalence | Female | Morocco | IBD | 0.66 (-1.1 to 2.46) | 0.451 |
| Childhood | Prevalence | Male | Morocco | IBD | 0.07 (-2.01 to 2.18) | 0.949 |
| Childhood | Prevalence | Both | Morocco | Psoriasis | -0.78 (-2.15 to 0.62) | 0.261 |
| Childhood | Prevalence | Female | Morocco | Psoriasis | -1.06 (-2.34 to 0.23) | 0.102 |
| Childhood | Prevalence | Male | Morocco | Psoriasis | -0.83 (-2.3 to 0.67) | 0.266 |
| Childhood | Prevalence | Both | Morocco | RHD | -0.03 (-0.15 to 0.09) | 0.616 |
| Childhood | Prevalence | Female | Morocco | RHD | -0.04 (-0.19 to 0.12) | 0.642 |
| Childhood | Prevalence | Male | Morocco | RHD | -0.05 (-0.15 to 0.05) | 0.281 |
| Childhood | Prevalence | Both | Morocco | RA | 0.75 (-0.73 to 2.25) | 0.310 |
| Childhood | Prevalence | Female | Morocco | RA | 1.24 | 0.106 |

| | | | | | | |
|---|---|---|---|---|---|---|
| | | | | | (-0.28 to 2.77) | |
| Childhood | Prevalence | Male | Morocco | RA | 0.74 (-0.69 to 2.19) | 0.302 |
| Childhood | Prevalence | Both | Mozambique | MS | -1.73 (-6.41 to 3.18) | 0.470 |
| Childhood | Prevalence | Female | Mozambique | MS | -0.86 (-5.45 to 3.95) | 0.712 |
| Childhood | Prevalence | Male | Mozambique | MS | -1.12 (-5.38 to 3.34) | 0.606 |
| Childhood | Prevalence | Both | Mozambique | Psoriasis | -0.32 (-1.34 to 0.72) | 0.534 |
| Childhood | Prevalence | Female | Mozambique | Psoriasis | -0.52 (-1.55 to 0.52) | 0.310 |
| Childhood | Prevalence | Male | Mozambique | Psoriasis | -0.33 (-1.35 to 0.7) | 0.518 |
| Childhood | Prevalence | Both | Mozambique | RHD | 0.11 (-0.03 to 0.24) | 0.116 |
| Childhood | Prevalence | Female | Mozambique | RHD | 0.09 (-0.03 to 0.2) | 0.122 |
| Childhood | Prevalence | Male | Mozambique | RHD | 0.1 (-0.06 to 0.25) | 0.213 |
| Childhood | Prevalence | Both | Mozambique | RA | 0.66 (-0.58 to 1.92) | 0.287 |
| Childhood | Prevalence | Female | Mozambique | RA | 0.88 (-0.32 to 2.09) | 0.146 |
| Childhood | Prevalence | Male | Mozambique | RA | 0.78 (-0.51 to 2.1) | 0.226 |
| Childhood | Prevalence | Both | Myanmar | T1D | 0.55 (-0.12 to 1.23) | 0.103 |
| Childhood | Prevalence | Female | Myanmar | T1D | 0.71 (0.09 to 1.33) | 0.026 |
| Childhood | Prevalence | Male | Myanmar | T1D | 0.6 (-0.13 to 1.34) | 0.105 |

| | | | | | | |
|---|---|---|---|---|---|---|
| Childhood | Prevalence | Both | Myanmar | MS | 0.93 (-1.26 to 3.17) | 0.395 |
| Childhood | Prevalence | Female | Myanmar | MS | 1.44 (-0.85 to 3.77) | 0.210 |
| Childhood | Prevalence | Male | Myanmar | MS | 0.83 (-1.34 to 3.04) | 0.445 |
| Childhood | Prevalence | Both | Myanmar | Psoriasis | -0.93 (-1.9 to 0.04) | 0.060 |
| Childhood | Prevalence | Female | Myanmar | Psoriasis | -0.71 (-1.45 to 0.04) | 0.061 |
| Childhood | Prevalence | Male | Myanmar | Psoriasis | -0.96 (-2.17 to 0.26) | 0.118 |
| Childhood | Prevalence | Both | Myanmar | RA | 0.51 (-2.11 to 3.2) | 0.698 |
| Childhood | Prevalence | Female | Myanmar | RA | -0.11 (-2.72 to 2.57) | 0.931 |
| Childhood | Prevalence | Male | Myanmar | RA | 0.35 (-2.2 to 2.96) | 0.786 |
| Childhood | Prevalence | Both | Namibia | T1D | 0.39 (-0.7 to 1.5) | 0.469 |
| Childhood | Prevalence | Female | Namibia | T1D | 0.1 (-1.02 to 1.22) | 0.862 |
| Childhood | Prevalence | Male | Namibia | T1D | 0.39 (-0.7 to 1.48) | 0.472 |
| Childhood | Prevalence | Both | Namibia | IBD | 0.4 (-0.68 to 1.5) | 0.455 |
| Childhood | Prevalence | Female | Namibia | IBD | 0.76 (-0.24 to 1.77) | 0.133 |
| Childhood | Prevalence | Male | Namibia | IBD | 0.54 (-0.69 to 1.8) | 0.378 |
| Childhood | Prevalence | Both | Namibia | MS | -0.14 (-1.86 to 1.61) | 0.871 |
| Childhood | Prevalence | Female | Namibia | MS | 0.33 | 0.702 |

| | | | | | | |
|---|---|---|---|---|---|---|
| | | | | | (-1.4 to 2.09) | |
| Childhood | Prevalence | Male | Namibia | MS | -0.11 (-1.78 to 1.59) | 0.895 |
| Childhood | Prevalence | Both | Namibia | Psoriasis | -0.49 (-1.19 to 0.23) | 0.172 |
| Childhood | Prevalence | Female | Namibia | Psoriasis | -0.67 (-1.37 to 0.05) | 0.066 |
| Childhood | Prevalence | Male | Namibia | Psoriasis | -0.45 (-1.15 to 0.26) | 0.208 |
| Childhood | Prevalence | Both | Namibia | RHD | -0.04 (-0.08 to 0.01) | 0.103 |
| Childhood | Prevalence | Female | Namibia | RHD | -0.06 (-0.08 to -0.03) | < 0.001 |
| Childhood | Prevalence | Male | Namibia | RHD | -0.03 (-0.1 to 0.04) | 0.384 |
| Childhood | Prevalence | Both | Namibia | RA | 1.1 (-0.6 to 2.82) | 0.198 |
| Childhood | Prevalence | Female | Namibia | RA | 0.61 (-1.07 to 2.32) | 0.463 |
| Childhood | Prevalence | Male | Namibia | RA | 1.21 (-0.53 to 2.98) | 0.166 |
| Childhood | Prevalence | Both | Nauru | AA | 0.03 (-0.25 to 0.31) | 0.842 |
| Childhood | Prevalence | Female | Nauru | AA | 0.04 (-0.23 to 0.31) | 0.762 |
| Childhood | Prevalence | Male | Nauru | AA | -0.04 (-0.3 to 0.23) | 0.762 |
| Childhood | Prevalence | Both | Nauru | IBD | -0.74 (-2.29 to 0.84) | 0.342 |
| Childhood | Prevalence | Female | Nauru | IBD | -0.31 (-2.04 to 1.44) | 0.714 |
| Childhood | Prevalence | Male | Nauru | IBD | -0.64 (-2.01 to 0.75) | 0.351 |

| | | | | | | |
|---|---|---|---|---|---|---|
| Childhood | Prevalence | Both | Nepal | AA | 0.1<br>(-0.07 to 0.27) | 0.227 |
| Childhood | Prevalence | Female | Nepal | AA | 0.03<br>(-0.17 to 0.23) | 0.769 |
| Childhood | Prevalence | Male | Nepal | AA | 0.01<br>(-0.19 to 0.21) | 0.944 |
| Childhood | Prevalence | Both | Nepal | IBD | 0.69<br>(-6.78 to 8.75) | 0.857 |
| Childhood | Prevalence | Female | Nepal | IBD | -1.25<br>(-8.63 to 6.74) | 0.744 |
| Childhood | Prevalence | Male | Nepal | IBD | 0.4<br>(-7.14 to 8.54) | 0.918 |
| Childhood | Prevalence | Both | Nepal | MS | 0.17<br>(-1.53 to 1.91) | 0.838 |
| Childhood | Prevalence | Female | Nepal | MS | 0.57<br>(-1.13 to 2.31) | 0.499 |
| Childhood | Prevalence | Male | Nepal | MS | 0.04<br>(-1.74 to 1.85) | 0.962 |
| Childhood | Prevalence | Both | Nepal | Psoriasis | -1.3<br>(-2.13 to -0.46) | 0.004 |
| Childhood | Prevalence | Female | Nepal | Psoriasis | -1.21<br>(-2.03 to -0.37) | 0.006 |
| Childhood | Prevalence | Male | Nepal | Psoriasis | -1.26<br>(-2.1 to -0.42) | 0.005 |
| Childhood | Prevalence | Both | Nepal | RHD | 0.04<br>(-0.38 to 0.46) | 0.847 |
| Childhood | Prevalence | Female | Nepal | RHD | 0.17<br>(-0.27 to 0.6) | 0.435 |
| Childhood | Prevalence | Male | Nepal | RHD | -0.03<br>(-0.45 to 0.39) | 0.883 |
| Childhood | Prevalence | Both | Netherlands | T1D | 1.33<br>(-0.55 to 3.23) | 0.159 |
| Childhood | Prevalence | Female | Netherlands | T1D | 1.79 | 0.054 |

| | | | | | | |
|---|---|---|---|---|---|---|
| | | | | | (-0.03 to 3.65) | |
| Childhood | Prevalence | Male | Netherlands | T1D | 1.24<br>(-0.7 to 3.2) | 0.202 |
| Childhood | Prevalence | Both | Netherlands | IBD | -1.54<br>(-2.93 to -0.13) | 0.033 |
| Childhood | Prevalence | Female | Netherlands | IBD | -1.73<br>(-2.92 to -0.53) | 0.006 |
| Childhood | Prevalence | Male | Netherlands | IBD | -1.64<br>(-3.24 to -0.01) | 0.048 |
| Childhood | Prevalence | Both | Netherlands | MS | -0.11<br>(-2.29 to 2.13) | 0.922 |
| Childhood | Prevalence | Female | Netherlands | MS | -0.47<br>(-2.6 to 1.71) | 0.658 |
| Childhood | Prevalence | Male | Netherlands | MS | -0.44<br>(-2.67 to 1.85) | 0.695 |
| Childhood | Prevalence | Both | Netherlands | Psoriasis | -0.26<br>(-1.13 to 0.61) | 0.543 |
| Childhood | Prevalence | Female | Netherlands | Psoriasis | -0.43<br>(-1.15 to 0.29) | 0.232 |
| Childhood | Prevalence | Male | Netherlands | Psoriasis | -0.25<br>(-1.26 to 0.76) | 0.610 |
| Childhood | Prevalence | Both | New Zealand | MS | -0.42<br>(-2.78 to 1.99) | 0.719 |
| Childhood | Prevalence | Female | New Zealand | MS | 0.19<br>(-2.14 to 2.58) | 0.869 |
| Childhood | Prevalence | Male | New Zealand | MS | -0.88<br>(-3.37 to 1.68) | 0.484 |
| Childhood | Prevalence | Both | New Zealand | Psoriasis | -0.21<br>(-0.68 to 0.27) | 0.386 |
| Childhood | Prevalence | Female | New Zealand | Psoriasis | -0.43<br>(-1 to 0.14) | 0.133 |
| Childhood | Prevalence | Male | New Zealand | Psoriasis | -0.17<br>(-0.55 to 0.22) | 0.383 |

| | | | | | | |
|---|---|---|---|---|---|---|
| Childhood | Prevalence | Both | Nicaragua | AA | -0.02<br>(-0.28 to 0.24) | 0.855 |
| Childhood | Prevalence | Female | Nicaragua | AA | 0.04<br>(-0.21 to 0.29) | 0.762 |
| Childhood | Prevalence | Male | Nicaragua | AA | -0.04<br>(-0.28 to 0.21) | 0.762 |
| Childhood | Prevalence | Both | Nicaragua | T1D | 0.67<br>(-0.23 to 1.58) | 0.141 |
| Childhood | Prevalence | Female | Nicaragua | T1D | 0.48<br>(-0.26 to 1.22) | 0.197 |
| Childhood | Prevalence | Male | Nicaragua | T1D | 0.66<br>(-0.41 to 1.75) | 0.218 |
| Childhood | Prevalence | Both | Nicaragua | IBD | 0.07<br>(-1.63 to 1.81) | 0.931 |
| Childhood | Prevalence | Female | Nicaragua | IBD | -0.76<br>(-2.52 to 1.04) | 0.392 |
| Childhood | Prevalence | Male | Nicaragua | IBD | 0.37<br>(-1.28 to 2.05) | 0.653 |
| Childhood | Prevalence | Both | Nicaragua | MS | 1.12<br>(-0.82 to 3.09) | 0.249 |
| Childhood | Prevalence | Female | Nicaragua | MS | 0.67<br>(-1.3 to 2.68) | 0.496 |
| Childhood | Prevalence | Male | Nicaragua | MS | 0.8<br>(-1.03 to 2.66) | 0.381 |
| Childhood | Prevalence | Both | Niger | IBD | -0.08<br>(-1.93 to 1.82) | 0.935 |
| Childhood | Prevalence | Female | Niger | IBD | 0.4<br>(-1.5 to 2.34) | 0.673 |
| Childhood | Prevalence | Male | Niger | IBD | 0.11<br>(-1.7 to 1.95) | 0.905 |
| Childhood | Prevalence | Both | Niger | MS | 0.88<br>(-4.45 to 6.51) | 0.742 |
| Childhood | Prevalence | Female | Niger | MS | -0.46 | 0.868 |

| | | | | | | |
|---|---|---|---|---|---|---|
| | | | | | (-5.9 to 5.29) | |
| Childhood | Prevalence | Male | Niger | MS | 0.79<br>(-4.26 to 6.09) | 0.757 |
| Childhood | Prevalence | Both | Niger | Psoriasis | -0.23<br>(-0.95 to 0.49) | 0.512 |
| Childhood | Prevalence | Female | Niger | Psoriasis | -0.47<br>(-1.17 to 0.25) | 0.191 |
| Childhood | Prevalence | Male | Niger | Psoriasis | -0.18<br>(-0.91 to 0.54) | 0.605 |
| Childhood | Prevalence | Both | Niger | RHD | 0.18<br>(0.07 to 0.28) | 0.002 |
| Childhood | Prevalence | Female | Niger | RHD | 0.23<br>(0.1 to 0.37) | 0.001 |
| Childhood | Prevalence | Male | Niger | RHD | 0.14<br>(0.06 to 0.21) | 0.001 |
| Childhood | Prevalence | Both | Niger | RA | 0.4<br>(-0.84 to 1.66) | 0.516 |
| Childhood | Prevalence | Female | Niger | RA | 0.66<br>(-0.75 to 2.09) | 0.346 |
| Childhood | Prevalence | Male | Niger | RA | 0.43<br>(-0.56 to 1.42) | 0.387 |
| Childhood | Prevalence | Both | Nigeria | T1D | 0.51<br>(-0.49 to 1.52) | 0.305 |
| Childhood | Prevalence | Female | Nigeria | T1D | 0.86<br>(-0.17 to 1.9) | 0.100 |
| Childhood | Prevalence | Male | Nigeria | T1D | 0.48<br>(-0.49 to 1.45) | 0.321 |
| Childhood | Prevalence | Both | Nigeria | RHD | 0.48<br>(0.29 to 0.68) | < 0.001 |
| Childhood | Prevalence | Female | Nigeria | RHD | 0.55<br>(0.34 to 0.76) | < 0.001 |
| Childhood | Prevalence | Male | Nigeria | RHD | 0.44<br>(0.25 to 0.63) | < 0.001 |

| | | | | | | |
|---|---|---|---|---|---|---|
| Childhood | Prevalence | Both | Nigeria | RA | 0.97 (-0.24 to 2.19) | 0.113 |
| Childhood | Prevalence | Female | Nigeria | RA | 0.12 (-1.24 to 1.5) | 0.857 |
| Childhood | Prevalence | Male | Nigeria | RA | 0.96 (-0.08 to 2.01) | 0.069 |
| Childhood | Prevalence | Both | Niue | T1D | 1.07 (-0.59 to 2.75) | 0.199 |
| Childhood | Prevalence | Female | Niue | T1D | 0.55 (-0.97 to 2.09) | 0.469 |
| Childhood | Prevalence | Male | Niue | T1D | 1.06 (-0.7 to 2.86) | 0.229 |
| Childhood | Prevalence | Both | Niue | MS | -0.17 (-1.8 to 1.48) | 0.830 |
| Childhood | Prevalence | Female | Niue | MS | 0.35 (-1.3 to 2.02) | 0.669 |
| Childhood | Prevalence | Male | Niue | MS | -0.1 (-1.72 to 1.55) | 0.901 |
| Childhood | Prevalence | Both | Niue | Psoriasis | -0.66 (-1.75 to 0.44) | 0.230 |
| Childhood | Prevalence | Female | Niue | Psoriasis | -0.45 (-1.28 to 0.39) | 0.283 |
| Childhood | Prevalence | Male | Niue | Psoriasis | -0.73 (-2.04 to 0.59) | 0.265 |
| Childhood | Prevalence | Both | Niue | RA | 0.29 (-1.14 to 1.74) | 0.686 |
| Childhood | Prevalence | Female | Niue | RA | 0.79 (-0.7 to 2.29) | 0.289 |
| Childhood | Prevalence | Male | Niue | RA | 0.3 (-1.06 to 1.68) | 0.658 |
| Childhood | Prevalence | Both | North Macedonia | RHD | -1.86 (-2.25 to -1.48) | < 0.001 |
| Childhood | Prevalence | Female | North Macedonia | RHD | -1.8 | < 0.001 |

| | | | | | | |
|---|---|---|---|---|---|---|
| | | | | | (-2.39 to -1.21) | |
| Childhood | Prevalence | Male | North Macedonia | RHD | -1.28<br>(-1.84 to -0.71) | < 0.001 |
| Childhood | Prevalence | Both | North Macedonia | RA | -0.11<br>(-3.4 to 3.28) | 0.945 |
| Childhood | Prevalence | Female | North Macedonia | RA | 0.75<br>(-2.38 to 3.99) | 0.629 |
| Childhood | Prevalence | Male | North Macedonia | RA | -0.16<br>(-3.76 to 3.57) | 0.930 |
| Childhood | Prevalence | Both | Northern Mariana Islands | T1D | 0.78<br>(-0.96 to 2.54) | 0.369 |
| Childhood | Prevalence | Female | Northern Mariana Islands | T1D | 0.34<br>(-1.25 to 1.96) | 0.663 |
| Childhood | Prevalence | Male | Northern Mariana Islands | T1D | 0.81<br>(-1.05 to 2.7) | 0.383 |
| Childhood | Prevalence | Both | Northern Mariana Islands | MS | 0.23<br>(-1.39 to 1.88) | 0.774 |
| Childhood | Prevalence | Female | Northern Mariana Islands | MS | -0.24<br>(-1.87 to 1.42) | 0.769 |
| Childhood | Prevalence | Male | Northern Mariana Islands | MS | 0.27<br>(-1.39 to 1.95) | 0.744 |
| Childhood | Prevalence | Both | Northern Mariana Islands | RHD | 0.09<br>(-0.19 to 0.36) | 0.525 |
| Childhood | Prevalence | Female | Northern Mariana Islands | RHD | 0.12<br>(-0.17 to 0.41) | 0.394 |
| Childhood | Prevalence | Male | Northern Mariana Islands | RHD | 0.06<br>(-0.19 to 0.3) | 0.637 |
| Childhood | Prevalence | Both | Northern Mariana Islands | RA | 0.3<br>(-1.1 to 1.73) | 0.664 |
| Childhood | Prevalence | Female | Northern Mariana Islands | RA | -0.04<br>(-1.49 to 1.42) | 0.953 |
| Childhood | Prevalence | Male | Northern Mariana Islands | RA | 0.26<br>(-1.14 to 1.68) | 0.711 |

| | | | | | | |
|---|---|---|---|---|---|---|
| Childhood | Prevalence | Both | Norway | IBD | 0.23<br>(-1.54 to 2.03) | 0.793 |
| Childhood | Prevalence | Female | Norway | IBD | 0.6<br>(-1.16 to 2.4) | 0.492 |
| Childhood | Prevalence | Male | Norway | IBD | 0.38<br>(-1.39 to 2.19) | 0.664 |
| Childhood | Prevalence | Both | Norway | MS | 1.17<br>(-0.65 to 3.02) | 0.201 |
| Childhood | Prevalence | Female | Norway | MS | 1.8<br>(0 to 3.63) | 0.050 |
| Childhood | Prevalence | Male | Norway | MS | 0.89<br>(-0.98 to 2.79) | 0.342 |
| Childhood | Prevalence | Both | Norway | Psoriasis | -0.05<br>(-1.41 to 1.33) | 0.942 |
| Childhood | Prevalence | Male | Norway | Psoriasis | -0.06<br>(-1.45 to 1.36) | 0.936 |
| Childhood | Prevalence | Both | Norway | RHD | -0.13<br>(-1.49 to 1.26) | 0.851 |
| Childhood | Prevalence | Female | Norway | RHD | 0.28<br>(-1.16 to 1.74) | 0.691 |
| Childhood | Prevalence | Male | Norway | RHD | -0.6<br>(-1.89 to 0.72) | 0.360 |
| Childhood | Prevalence | Both | Norway | RA | -0.65<br>(-3.71 to 2.52) | 0.674 |
| Childhood | Prevalence | Female | Norway | RA | 0.25<br>(-2.83 to 3.43) | 0.869 |
| Childhood | Prevalence | Male | Norway | RA | -0.47<br>(-3.57 to 2.72) | 0.762 |
| Childhood | Prevalence | Both | Pakistan | T1D | 0.27<br>(-0.79 to 1.33) | 0.611 |
| Childhood | Prevalence | Female | Pakistan | T1D | 0.56<br>(-0.42 to 1.56) | 0.253 |
| Childhood | Prevalence | Male | Pakistan | T1D | 0.29 | 0.596 |

| | | | | | | |
|---|---|---|---|---|---|---|
| | | | | | (-0.81 to 1.4) | |
| Childhood | Prevalence | Both | Pakistan | IBD | 0.44<br>(-1.28 to 2.19) | 0.608 |
| Childhood | Prevalence | Female | Pakistan | IBD | 0.99<br>(-0.88 to 2.9) | 0.290 |
| Childhood | Prevalence | Male | Pakistan | IBD | 0.5<br>(-1.07 to 2.1) | 0.519 |
| Childhood | Prevalence | Both | Pakistan | Psoriasis | -0.75<br>(-1.51 to 0.02) | 0.056 |
| Childhood | Prevalence | Female | Pakistan | Psoriasis | -0.56<br>(-1.32 to 0.21) | 0.148 |
| Childhood | Prevalence | Male | Pakistan | Psoriasis | -0.74<br>(-1.5 to 0.04) | 0.062 |
| Childhood | Prevalence | Both | Pakistan | RA | 0.01<br>(-1.1 to 1.14) | 0.981 |
| Childhood | Prevalence | Female | Pakistan | RA | 0.29<br>(-0.68 to 1.28) | 0.543 |
| Childhood | Prevalence | Male | Pakistan | RA | -0.05<br>(-1.32 to 1.24) | 0.941 |
| Childhood | Prevalence | Both | Palau | AA | -0.04<br>(-0.29 to 0.22) | 0.781 |
| Childhood | Prevalence | Female | Palau | AA | -0.04<br>(-0.31 to 0.23) | 0.762 |
| Childhood | Prevalence | Male | Palau | AA | 0.04<br>(-0.22 to 0.3) | 0.762 |
| Childhood | Prevalence | Both | Palau | IBD | -0.6<br>(-2.19 to 1.01) | 0.449 |
| Childhood | Prevalence | Female | Palau | IBD | -0.07<br>(-1.88 to 1.76) | 0.934 |
| Childhood | Prevalence | Male | Palau | IBD | -0.52<br>(-1.92 to 0.89) | 0.454 |
| Childhood | Prevalence | Both | Palau | Psoriasis | -0.15<br>(-0.47 to 0.17) | 0.341 |

| | | | | | | |
|---|---|---|---|---|---|---|
| Childhood | Prevalence | Female | Palau | Psoriasis | -0.34 (-0.71 to 0.04) | 0.075 |
| Childhood | Prevalence | Male | Palau | Psoriasis | -0.11 (-0.43 to 0.21) | 0.476 |
| Childhood | Prevalence | Both | Palau | RHD | -0.66 (-1.38 to 0.07) | 0.073 |
| Childhood | Prevalence | Female | Palau | RHD | -0.59 (-1.44 to 0.27) | 0.173 |
| Childhood | Prevalence | Male | Palau | RHD | -0.42 (-1 to 0.17) | 0.157 |
| Childhood | Prevalence | Both | Palau | RA | 0.56 (-1.04 to 2.19) | 0.482 |
| Childhood | Prevalence | Female | Palau | RA | 0.24 (-1.19 to 1.69) | 0.736 |
| Childhood | Prevalence | Male | Palau | RA | 0.56 (-1.17 to 2.32) | 0.514 |
| Childhood | Prevalence | Both | Palestine | AA | -0.12 (-0.37 to 0.13) | 0.345 |
| Childhood | Prevalence | Female | Palestine | AA | 0.04 (-0.21 to 0.29) | 0.762 |
| Childhood | Prevalence | Male | Palestine | AA | -0.03 (-0.26 to 0.19) | 0.762 |
| Childhood | Prevalence | Both | Palestine | Psoriasis | -1.1 (-2.01 to -0.19) | 0.019 |
| Childhood | Prevalence | Female | Palestine | Psoriasis | -1.32 (-2.16 to -0.47) | 0.004 |
| Childhood | Prevalence | Male | Palestine | Psoriasis | -1.08 (-2.05 to -0.11) | 0.031 |
| Childhood | Prevalence | Both | Palestine | RHD | 0.09 (-0.44 to 0.62) | 0.742 |
| Childhood | Prevalence | Female | Palestine | RHD | -0.02 (-0.57 to 0.54) | 0.953 |
| Childhood | Prevalence | Male | Palestine | RHD | 0.1 | 0.694 |

| | | | | | | |
|---|---|---|---|---|---|---|
| | | | | | (-0.4 to 0.6) | |
| Childhood | Prevalence | Both | Panama | IBD | -0.52<br>(-2.13 to 1.11) | 0.517 |
| Childhood | Prevalence | Female | Panama | IBD | -0.59<br>(-2.22 to 1.08) | 0.474 |
| Childhood | Prevalence | Male | Panama | IBD | -0.16<br>(-1.74 to 1.45) | 0.841 |
| Childhood | Prevalence | Both | Panama | MS | 0.23<br>(-1.54 to 2.02) | 0.796 |
| Childhood | Prevalence | Female | Panama | MS | 0.58<br>(-1.19 to 2.39) | 0.507 |
| Childhood | Prevalence | Male | Panama | MS | 0.22<br>(-1.56 to 2.04) | 0.800 |
| Childhood | Prevalence | Both | Panama | RHD | 0.06<br>(-0.26 to 0.37) | 0.721 |
| Childhood | Prevalence | Female | Panama | RHD | -0.08<br>(-0.47 to 0.3) | 0.665 |
| Childhood | Prevalence | Male | Panama | RHD | 0.02<br>(-0.21 to 0.25) | 0.853 |
| Childhood | Prevalence | Both | Panama | RA | -0.18<br>(-3.5 to 3.26) | 0.916 |
| Childhood | Prevalence | Female | Panama | RA | 0.47<br>(-3.02 to 4.08) | 0.789 |
| Childhood | Prevalence | Male | Panama | RA | -0.1<br>(-3.39 to 3.31) | 0.952 |
| Childhood | Prevalence | Both | Papua New Guinea | T1D | 0.82<br>(0.07 to 1.57) | 0.033 |
| Childhood | Prevalence | Female | Papua New Guinea | T1D | 0.57<br>(-0.14 to 1.28) | 0.113 |
| Childhood | Prevalence | Male | Papua New Guinea | T1D | 0.84<br>(0.07 to 1.62) | 0.034 |
| Childhood | Prevalence | Both | Papua New Guinea | IBD | -0.71<br>(-2.28 to 0.88) | 0.366 |

| | | | | | | |
|---|---|---|---|---|---|---|
| Childhood | Prevalence | Female | Papua New Guinea | IBD | -0.29 (-2 to 1.44) | 0.730 |
| Childhood | Prevalence | Male | Papua New Guinea | IBD | -0.68 (-2.13 to 0.79) | 0.351 |
| Childhood | Prevalence | Both | Papua New Guinea | MS | -0.77 (-3.86 to 2.43) | 0.623 |
| Childhood | Prevalence | Female | Papua New Guinea | MS | 0.08 (-3.04 to 3.31) | 0.958 |
| Childhood | Prevalence | Male | Papua New Guinea | MS | -0.73 (-3.78 to 2.41) | 0.634 |
| Childhood | Prevalence | Both | Papua New Guinea | Psoriasis | -0.46 (-1.55 to 0.65) | 0.403 |
| Childhood | Prevalence | Female | Papua New Guinea | Psoriasis | -0.22 (-1.04 to 0.62) | 0.598 |
| Childhood | Prevalence | Male | Papua New Guinea | Psoriasis | -0.5 (-1.8 to 0.82) | 0.441 |
| Childhood | Prevalence | Both | Papua New Guinea | RA | -0.09 (-1.84 to 1.69) | 0.918 |
| Childhood | Prevalence | Female | Papua New Guinea | RA | 0.41 (-1.33 to 2.18) | 0.634 |
| Childhood | Prevalence | Male | Papua New Guinea | RA | -0.13 (-2.02 to 1.79) | 0.889 |
| Childhood | Prevalence | Both | Paraguay | AA | -0.04 (-0.28 to 0.21) | 0.768 |
| Childhood | Prevalence | Female | Paraguay | AA | 0.04 (-0.21 to 0.29) | 0.762 |
| Childhood | Prevalence | Male | Paraguay | AA | -0.04 (-0.28 to 0.21) | 0.762 |
| Childhood | Prevalence | Both | Paraguay | T1D | 0.7 (-0.85 to 2.26) | 0.365 |
| Childhood | Prevalence | Female | Paraguay | T1D | 1.09 (-0.53 to 2.74) | 0.179 |
| Childhood | Prevalence | Male | Paraguay | T1D | 0.94 | 0.161 |

| | | | | | | |
|---|---|---|---|---|---|---|
| | | | | | (-0.39 to 2.29) | |
| Childhood | Prevalence | Both | Paraguay | IBD | -2.03<br>(-6.52 to 2.68) | 0.379 |
| Childhood | Prevalence | Female | Paraguay | IBD | -0.56<br>(-5.19 to 4.29) | 0.810 |
| Childhood | Prevalence | Male | Paraguay | IBD | -2.15<br>(-6.62 to 2.53) | 0.349 |
| Childhood | Prevalence | Both | Paraguay | Psoriasis | -0.04<br>(-0.75 to 0.67) | 0.903 |
| Childhood | Prevalence | Female | Paraguay | Psoriasis | -0.21<br>(-0.91 to 0.51) | 0.555 |
| Childhood | Prevalence | Male | Paraguay | Psoriasis | -0.06<br>(-0.77 to 0.64) | 0.852 |
| Childhood | Prevalence | Both | Peru | IBD | -0.32<br>(-2.07 to 1.47) | 0.718 |
| Childhood | Prevalence | Female | Peru | IBD | 0.32<br>(-1.62 to 2.29) | 0.741 |
| Childhood | Prevalence | Male | Peru | IBD | -0.4<br>(-1.97 to 1.2) | 0.611 |
| Childhood | Prevalence | Both | Peru | RHD | -0.6<br>(-2.49 to 1.32) | 0.525 |
| Childhood | Prevalence | Female | Peru | RHD | -1.04<br>(-2.92 to 0.88) | 0.275 |
| Childhood | Prevalence | Male | Peru | RHD | -0.67<br>(-2.49 to 1.19) | 0.465 |
| Childhood | Prevalence | Both | Peru | RA | 0.75<br>(-1.6 to 3.15) | 0.523 |
| Childhood | Prevalence | Female | Peru | RA | 1.36<br>(-1.04 to 3.82) | 0.258 |
| Childhood | Prevalence | Male | Peru | RA | 0.59<br>(-1.76 to 3) | 0.612 |
| Childhood | Prevalence | Both | Philippines | IBD | 0.12<br>(-1.52 to 1.79) | 0.882 |

| | | | | | | |
|---|---|---|---|---|---|---|
| Childhood | Prevalence | Female | Philippines | IBD | 0.43 (-1.36 to 2.26) | 0.627 |
| Childhood | Prevalence | Male | Philippines | IBD | 0.39 (-1.13 to 1.92) | 0.606 |
| Childhood | Prevalence | Both | Philippines | MS | 0.23 (-1.34 to 1.83) | 0.764 |
| Childhood | Prevalence | Female | Philippines | MS | -0.24 (-1.8 to 1.35) | 0.757 |
| Childhood | Prevalence | Male | Philippines | MS | 0.27 (-1.26 to 1.83) | 0.719 |
| Childhood | Prevalence | Both | Philippines | Psoriasis | -0.06 (-0.69 to 0.58) | 0.852 |
| Childhood | Prevalence | Female | Philippines | Psoriasis | -0.23 (-0.87 to 0.41) | 0.468 |
| Childhood | Prevalence | Male | Philippines | Psoriasis | -0.07 (-0.7 to 0.56) | 0.820 |
| Childhood | Prevalence | Both | Philippines | RHD | 0.23 (-0.12 to 0.58) | 0.195 |
| Childhood | Prevalence | Female | Philippines | RHD | 0.39 (0 to 0.79) | 0.051 |
| Childhood | Prevalence | Male | Philippines | RHD | 0.18 (-0.12 to 0.48) | 0.226 |
| Childhood | Prevalence | Both | Poland | AA | -0.06 (-0.31 to 0.19) | 0.624 |
| Childhood | Prevalence | Female | Poland | AA | 0.04 (-0.21 to 0.28) | 0.762 |
| Childhood | Prevalence | Male | Poland | AA | -0.04 (-0.28 to 0.21) | 0.762 |
| Childhood | Prevalence | Both | Poland | IBD | -4.24 (-6.72 to -1.7) | 0.001 |
| Childhood | Prevalence | Female | Poland | IBD | -3.81 (-6.19 to -1.37) | 0.002 |
| Childhood | Prevalence | Male | Poland | IBD | -3.87 | < 0.001 |

| | | | | | | |
|---|---|---|---|---|---|---|
| | | | | | (-5.42 to -2.3) | |
| Childhood | Prevalence | Both | Poland | MS | -0.03 (-1.67 to 1.64) | 0.972 |
| Childhood | Prevalence | Female | Poland | MS | 0.6 (-0.99 to 2.23) | 0.447 |
| Childhood | Prevalence | Male | Poland | MS | -0.39 (-2.08 to 1.34) | 0.648 |
| Childhood | Prevalence | Both | Poland | RA | -0.22 (-3.32 to 2.98) | 0.888 |
| Childhood | Prevalence | Female | Poland | RA | -0.95 (-3.96 to 2.15) | 0.529 |
| Childhood | Prevalence | Male | Poland | RA | -0.13 (-3.16 to 3) | 0.933 |
| Childhood | Prevalence | Both | Portugal | AA | -0.08 (-0.16 to 0) | 0.062 |
| Childhood | Prevalence | Female | Portugal | AA | 0.23 (-0.43 to 0.89) | 0.480 |
| Childhood | Prevalence | Male | Portugal | AA | -0.42 (-1.63 to 0.82) | 0.493 |
| Childhood | Prevalence | Both | Portugal | IBD | 0.74 (-0.49 to 1.99) | 0.228 |
| Childhood | Prevalence | Female | Portugal | IBD | 0.5 (-0.55 to 1.56) | 0.339 |
| Childhood | Prevalence | Male | Portugal | IBD | 0.76 (-0.65 to 2.19) | 0.279 |
| Childhood | Prevalence | Both | Portugal | MS | -0.11 (-1.82 to 1.64) | 0.901 |
| Childhood | Prevalence | Female | Portugal | MS | 0.29 (-1.38 to 2) | 0.725 |
| Childhood | Prevalence | Male | Portugal | MS | -0.27 (-2.04 to 1.52) | 0.755 |
| Childhood | Prevalence | Both | Portugal | Psoriasis | -0.28 (-0.92 to 0.37) | 0.381 |

| | | | | | | |
|---|---|---|---|---|---|---|
| Childhood | Prevalence | Female | Portugal | Psoriasis | -0.49 (-1.18 to 0.2) | 0.160 |
| Childhood | Prevalence | Male | Portugal | Psoriasis | -0.24 (-0.85 to 0.37) | 0.427 |
| Childhood | Prevalence | Both | Portugal | RHD | -2.43 (-5.65 to 0.89) | 0.144 |
| Childhood | Prevalence | Female | Portugal | RHD | -3.7 (-6.98 to -0.3) | 0.034 |
| Childhood | Prevalence | Male | Portugal | RHD | -1.65 (-4.67 to 1.47) | 0.285 |
| Childhood | Prevalence | Both | Portugal | RA | 1.24 (-0.95 to 3.49) | 0.259 |
| Childhood | Prevalence | Female | Portugal | RA | 0.79 (-1.39 to 3.02) | 0.469 |
| Childhood | Prevalence | Male | Portugal | RA | 1.01 (-1.24 to 3.32) | 0.368 |
| Childhood | Prevalence | Both | Puerto Rico | AA | 0.02 (-0.25 to 0.29) | 0.865 |
| Childhood | Prevalence | Female | Puerto Rico | AA | -0.04 (-0.29 to 0.21) | 0.762 |
| Childhood | Prevalence | Male | Puerto Rico | AA | 0.04 (-0.21 to 0.28) | 0.762 |
| Childhood | Prevalence | Both | Puerto Rico | IBD | -0.13 (-1.35 to 1.1) | 0.828 |
| Childhood | Prevalence | Female | Puerto Rico | IBD | 0.14 (-1 to 1.3) | 0.802 |
| Childhood | Prevalence | Male | Puerto Rico | IBD | -0.15 (-1.46 to 1.19) | 0.822 |
| Childhood | Prevalence | Both | Puerto Rico | MS | 0.93 (-0.88 to 2.78) | 0.302 |
| Childhood | Prevalence | Female | Puerto Rico | MS | 0.39 (-1.43 to 2.24) | 0.669 |
| Childhood | Prevalence | Male | Puerto Rico | MS | 1.09 | 0.209 |

| | | | | | | |
|---|---|---|---|---|---|---|
| | | | | | (-0.64 to 2.86) | |
| Childhood | Prevalence | Both | Qatar | IBD | 0.73<br>(-1.04 to 2.54) | 0.408 |
| Childhood | Prevalence | Female | Qatar | IBD | -0.06<br>(-1.89 to 1.81) | 0.951 |
| Childhood | Prevalence | Male | Qatar | IBD | 0.87<br>(-0.88 to 2.64) | 0.319 |
| Childhood | Prevalence | Both | Qatar | Psoriasis | -0.89<br>(-1.69 to -0.08) | 0.032 |
| Childhood | Prevalence | Female | Qatar | Psoriasis | -1.05<br>(-1.81 to -0.29) | 0.009 |
| Childhood | Prevalence | Male | Qatar | Psoriasis | -0.86<br>(-1.71 to -0.01) | 0.048 |
| Childhood | Prevalence | Both | Qatar | RHD | -1.89<br>(-2.61 to -1.17) | < 0.001 |
| Childhood | Prevalence | Female | Qatar | RHD | -2.01<br>(-2.75 to -1.26) | < 0.001 |
| Childhood | Prevalence | Male | Qatar | RHD | -1.91<br>(-2.62 to -1.19) | < 0.001 |
| Childhood | Prevalence | Both | Qatar | RA | 0.56<br>(-1.87 to 3.06) | 0.642 |
| Childhood | Prevalence | Female | Qatar | RA | 1.68<br>(-0.61 to 4.03) | 0.146 |
| Childhood | Prevalence | Male | Qatar | RA | 0.67<br>(-2.03 to 3.44) | 0.618 |
| Childhood | Prevalence | Both | Republic of Korea | MS | -0.13<br>(-1.75 to 1.53) | 0.877 |
| Childhood | Prevalence | Female | Republic of Korea | MS | 0.39<br>(-1.22 to 2.03) | 0.626 |
| Childhood | Prevalence | Male | Republic of Korea | MS | -0.2<br>(-1.89 to 1.51) | 0.809 |
| Childhood | Prevalence | Both | Republic of Korea | Psoriasis | -0.33<br>(-1.13 to 0.47) | 0.401 |

| | | | | | | |
|---|---|---|---|---|---|---|
| Childhood | Prevalence | Female | Republic of Korea | Psoriasis | -0.59<br>(-1.25 to 0.07) | 0.078 |
| Childhood | Prevalence | Male | Republic of Korea | Psoriasis | -0.25<br>(-1.17 to 0.68) | 0.590 |
| Childhood | Prevalence | Both | Republic of Korea | RA | 0.48<br>(-0.9 to 1.87) | 0.488 |
| Childhood | Prevalence | Female | Republic of Korea | RA | 0.88<br>(-0.57 to 2.35) | 0.223 |
| Childhood | Prevalence | Male | Republic of Korea | RA | 0.6<br>(-0.74 to 1.96) | 0.370 |
| Childhood | Prevalence | Both | Republic of Moldova | T1D | 1.01<br>(-1.04 to 3.1) | 0.324 |
| Childhood | Prevalence | Female | Republic of Moldova | T1D | 1.59<br>(-0.31 to 3.53) | 0.097 |
| Childhood | Prevalence | Male | Republic of Moldova | T1D | 1.04<br>(-1.15 to 3.28) | 0.341 |
| Childhood | Prevalence | Both | Republic of Moldova | IBD | 0.58<br>(-0.98 to 2.17) | 0.454 |
| Childhood | Prevalence | Female | Republic of Moldova | IBD | 0.27<br>(-1.14 to 1.7) | 0.702 |
| Childhood | Prevalence | Male | Republic of Moldova | IBD | 0.52<br>(-1.2 to 2.28) | 0.541 |
| Childhood | Prevalence | Both | Republic of Moldova | MS | 0.63<br>(-0.91 to 2.2) | 0.409 |
| Childhood | Prevalence | Female | Republic of Moldova | MS | 0.84<br>(-0.69 to 2.39) | 0.272 |
| Childhood | Prevalence | Male | Republic of Moldova | MS | 0.74<br>(-0.79 to 2.29) | 0.333 |
| Childhood | Prevalence | Both | Republic of Moldova | Psoriasis | -0.73<br>(-1.45 to 0) | 0.050 |
| Childhood | Prevalence | Female | Republic of Moldova | Psoriasis | -0.6<br>(-1.1 to -0.1) | 0.020 |
| Childhood | Prevalence | Male | Republic of Moldova | Psoriasis | -0.76 | 0.118 |

| | | | | | | |
|---|---|---|---|---|---|---|
| | | | | | (-1.71 to 0.21) | |
| Childhood | Prevalence | Both | Republic of Moldova | RA | 1.02<br>(-0.64 to 2.71) | 0.221 |
| Childhood | Prevalence | Female | Republic of Moldova | RA | 0.64<br>(-1.05 to 2.36) | 0.448 |
| Childhood | Prevalence | Male | Republic of Moldova | RA | 1.09<br>(-0.5 to 2.7) | 0.173 |
| Childhood | Prevalence | Both | Romania | AA | -0.07<br>(-0.32 to 0.18) | 0.588 |
| Childhood | Prevalence | Female | Romania | AA | 0.04<br>(-0.21 to 0.29) | 0.762 |
| Childhood | Prevalence | Male | Romania | AA | -0.04<br>(-0.28 to 0.21) | 0.762 |
| Childhood | Prevalence | Both | Romania | IBD | 0.2<br>(-1.3 to 1.72) | 0.791 |
| Childhood | Prevalence | Female | Romania | IBD | 0.63<br>(-0.7 to 1.98) | 0.340 |
| Childhood | Prevalence | Male | Romania | IBD | 0.12<br>(-1.55 to 1.81) | 0.885 |
| Childhood | Prevalence | Both | Romania | MS | -0.25<br>(-1.92 to 1.44) | 0.760 |
| Childhood | Prevalence | Female | Romania | MS | 0.31<br>(-1.37 to 2.01) | 0.712 |
| Childhood | Prevalence | Male | Romania | MS | -0.41<br>(-2.07 to 1.28) | 0.623 |
| Childhood | Prevalence | Both | Romania | Psoriasis | -1.21<br>(-2.9 to 0.51) | 0.158 |
| Childhood | Prevalence | Female | Romania | Psoriasis | -0.83<br>(-2.25 to 0.62) | 0.251 |
| Childhood | Prevalence | Male | Romania | Psoriasis | -1.24<br>(-3.17 to 0.72) | 0.204 |
| Childhood | Prevalence | Both | Romania | RA | 0.25<br>(-1.99 to 2.54) | 0.824 |

| | | | | | | |
|---|---|---|---|---|---|---|
| Childhood | Prevalence | Female | Romania | RA | 0.94 (-1.26 to 3.2) | 0.392 |
| Childhood | Prevalence | Male | Romania | RA | 0.33 (-2.02 to 2.73) | 0.777 |
| Childhood | Prevalence | Both | Russian Federation | AA | -0.04 (-0.29 to 0.23) | 0.784 |
| Childhood | Prevalence | Female | Russian Federation | AA | 0.04 (-0.21 to 0.28) | 0.762 |
| Childhood | Prevalence | Male | Russian Federation | AA | -0.04 (-0.28 to 0.21) | 0.762 |
| Childhood | Prevalence | Both | Russian Federation | IBD | -0.39 (-1.92 to 1.16) | 0.607 |
| Childhood | Prevalence | Female | Russian Federation | IBD | 0.03 (-1.39 to 1.48) | 0.965 |
| Childhood | Prevalence | Male | Russian Federation | IBD | -0.33 (-1.96 to 1.33) | 0.689 |
| Childhood | Prevalence | Both | Russian Federation | MS | 1.5 (0.36 to 2.65) | 0.012 |
| Childhood | Prevalence | Female | Russian Federation | MS | 1.41 (0.25 to 2.58) | 0.019 |
| Childhood | Prevalence | Male | Russian Federation | MS | 1.41 (0.26 to 2.57) | 0.018 |
| Childhood | Prevalence | Both | Russian Federation | RHD | -2.71 (-3.83 to -1.58) | < 0.001 |
| Childhood | Prevalence | Female | Russian Federation | RHD | -2.38 (-3.44 to -1.31) | < 0.001 |
| Childhood | Prevalence | Male | Russian Federation | RHD | -2.85 (-4.15 to -1.52) | < 0.001 |
| Childhood | Prevalence | Both | Rwanda | AA | -0.03 (-0.12 to 0.07) | 0.565 |
| Childhood | Prevalence | Female | Rwanda | AA | 0 (-0.06 to 0.06) | 0.981 |
| Childhood | Prevalence | Male | Rwanda | AA | -0.01 | 0.574 |

| | | | | | | |
|---|---|---|---|---|---|---|
| | | | | | (-0.05 to 0.03) | |
| Childhood | Prevalence | Both | Rwanda | IBD | 0.43<br>(-1.38 to 2.28) | 0.632 |
| Childhood | Prevalence | Female | Rwanda | IBD | 0.89<br>(-1.01 to 2.84) | 0.348 |
| Childhood | Prevalence | Male | Rwanda | IBD | 0.44<br>(-1.23 to 2.14) | 0.595 |
| Childhood | Prevalence | Both | Rwanda | MS | 0.38<br>(-0.92 to 1.7) | 0.554 |
| Childhood | Prevalence | Female | Rwanda | MS | 0.65<br>(-0.65 to 1.97) | 0.314 |
| Childhood | Prevalence | Male | Rwanda | MS | 0.48<br>(-0.63 to 1.61) | 0.385 |
| Childhood | Prevalence | Both | Rwanda | Psoriasis | -0.3<br>(-1 to 0.41) | 0.393 |
| Childhood | Prevalence | Female | Rwanda | Psoriasis | -0.52<br>(-1.22 to 0.18) | 0.142 |
| Childhood | Prevalence | Male | Rwanda | Psoriasis | -0.27<br>(-0.97 to 0.44) | 0.446 |
| Childhood | Prevalence | Both | Rwanda | RA | 1.1<br>(-0.17 to 2.38) | 0.086 |
| Childhood | Prevalence | Female | Rwanda | RA | 1.32<br>(0.1 to 2.55) | 0.034 |
| Childhood | Prevalence | Male | Rwanda | RA | 1.28<br>(0 to 2.57) | 0.049 |
| Childhood | Prevalence | Both | Saint Kitts and Nevis | IBD | 0.23<br>(-1.95 to 2.47) | 0.831 |
| Childhood | Prevalence | Female | Saint Kitts and Nevis | IBD | -0.29<br>(-2.28 to 1.74) | 0.768 |
| Childhood | Prevalence | Male | Saint Kitts and Nevis | IBD | 0.26<br>(-2.12 to 2.7) | 0.828 |
| Childhood | Prevalence | Both | Saint Kitts and Nevis | MS | 0.12<br>(-1.84 to 2.12) | 0.900 |

| | | | | | | |
|---|---|---|---|---|---|---|
| Childhood | Prevalence | Female | Saint Kitts and Nevis | MS | 0.38 (-1.52 to 2.32) | 0.688 |
| Childhood | Prevalence | Male | Saint Kitts and Nevis | MS | 0.35 (-1.77 to 2.52) | 0.739 |
| Childhood | Prevalence | Both | Saint Kitts and Nevis | RHD | -2.86 (-3.71 to -1.99) | < 0.001 |
| Childhood | Prevalence | Female | Saint Kitts and Nevis | RHD | -3.35 (-4.28 to -2.42) | < 0.001 |
| Childhood | Prevalence | Male | Saint Kitts and Nevis | RHD | -2.16 (-2.91 to -1.4) | < 0.001 |
| Childhood | Prevalence | Both | Saint Lucia | AA | 0.01 (-0.23 to 0.25) | 0.942 |
| Childhood | Prevalence | Female | Saint Lucia | AA | -0.04 (-0.29 to 0.21) | 0.762 |
| Childhood | Prevalence | Male | Saint Lucia | AA | 0.04 (-0.21 to 0.28) | 0.762 |
| Childhood | Prevalence | Both | Saint Lucia | T1D | 0.95 (-0.7 to 2.63) | 0.251 |
| Childhood | Prevalence | Female | Saint Lucia | T1D | 1.3 (-0.13 to 2.75) | 0.073 |
| Childhood | Prevalence | Male | Saint Lucia | T1D | 1.01 (-0.89 to 2.95) | 0.288 |
| Childhood | Prevalence | Both | Saint Lucia | IBD | -0.29 (-1.84 to 1.28) | 0.706 |
| Childhood | Prevalence | Female | Saint Lucia | IBD | -0.11 (-1.63 to 1.43) | 0.882 |
| Childhood | Prevalence | Male | Saint Lucia | IBD | 0.08 (-1.56 to 1.74) | 0.924 |
| Childhood | Prevalence | Both | Saint Lucia | MS | 0.25 (-1.51 to 2.05) | 0.771 |
| Childhood | Prevalence | Female | Saint Lucia | MS | 0.76 (-1.01 to 2.56) | 0.389 |
| Childhood | Prevalence | Male | Saint Lucia | MS | 0.24 | 0.790 |

| | | | | | | |
|---|---|---|---|---|---|---|
| | | | | | (-1.58 to 2.1) | |
| Childhood | Prevalence | Both | Saint Lucia | RHD | -0.17<br>(-0.98 to 0.66) | 0.682 |
| Childhood | Prevalence | Female | Saint Lucia | RHD | -0.38<br>(-1.21 to 0.47) | 0.368 |
| Childhood | Prevalence | Male | Saint Lucia | RHD | -0.19<br>(-0.94 to 0.57) | 0.610 |
| Childhood | Prevalence | Both | Saint Lucia | RA | 0.98<br>(-1.2 to 3.22) | 0.367 |
| Childhood | Prevalence | Female | Saint Lucia | RA | 1.54<br>(-0.63 to 3.76) | 0.158 |
| Childhood | Prevalence | Male | Saint Lucia | RA | 1.05<br>(-1.23 to 3.38) | 0.358 |
| Childhood | Prevalence | Both | Saint Vincent and the Grenadines | AA | -0.06<br>(-0.19 to 0.07) | 0.365 |
| Childhood | Prevalence | Female | Saint Vincent and the Grenadines | AA | 0.06<br>(-0.09 to 0.2) | 0.417 |
| Childhood | Prevalence | Male | Saint Vincent and the Grenadines | AA | 0.01<br>(-0.13 to 0.16) | 0.860 |
| Childhood | Prevalence | Both | Saint Vincent and the Grenadines | T1D | 1.25<br>(-0.02 to 2.53) | 0.054 |
| Childhood | Prevalence | Female | Saint Vincent and the Grenadines | T1D | 0.89<br>(-0.18 to 1.97) | 0.100 |
| Childhood | Prevalence | Male | Saint Vincent and the Grenadines | T1D | 1.3<br>(-0.16 to 2.78) | 0.079 |
| Childhood | Prevalence | Both | Saint Vincent and the Grenadines | IBD | 0.15<br>(-1.46 to 1.79) | 0.850 |
| Childhood | Prevalence | Female | Saint Vincent and the Grenadines | IBD | -0.65<br>(-2.26 to 0.98) | 0.420 |
| Childhood | Prevalence | Male | Saint Vincent and the Grenadines | IBD | 0.5<br>(-1.1 to 2.13) | 0.530 |
| Childhood | Prevalence | Both | Saint Vincent and the Grenadines | MS | 0.77<br>(-1.43 to 3.03) | 0.483 |

| | | | | | | |
|---|---|---|---|---|---|---|
| Childhood | Prevalence | Female | Saint Vincent and the Grenadines | MS | 0.42<br>(-1.75 to 2.63) | 0.698 |
| Childhood | Prevalence | Male | Saint Vincent and the Grenadines | MS | 0.6<br>(-1.67 to 2.91) | 0.597 |
| Childhood | Prevalence | Both | Saint Vincent and the Grenadines | RHD | -0.06<br>(-0.54 to 0.42) | 0.793 |
| Childhood | Prevalence | Female | Saint Vincent and the Grenadines | RHD | 0.1<br>(-0.43 to 0.64) | 0.702 |
| Childhood | Prevalence | Male | Saint Vincent and the Grenadines | RHD | -0.07<br>(-0.49 to 0.35) | 0.729 |
| Childhood | Prevalence | Both | Samoa | AA | 0.13<br>(-0.38 to 0.63) | 0.613 |
| Childhood | Prevalence | Female | Samoa | AA | -0.14<br>(-0.65 to 0.36) | 0.560 |
| Childhood | Prevalence | Male | Samoa | AA | 0.12<br>(-0.36 to 0.61) | 0.612 |
| Childhood | Prevalence | Both | Samoa | IBD | -1.41<br>(-6.08 to 3.49) | 0.554 |
| Childhood | Prevalence | Female | Samoa | IBD | -0.3<br>(-5.06 to 4.69) | 0.900 |
| Childhood | Prevalence | Male | Samoa | IBD | -1.45<br>(-6.13 to 3.46) | 0.543 |
| Childhood | Prevalence | Both | Samoa | MS | 0.26<br>(-1.38 to 1.94) | 0.746 |
| Childhood | Prevalence | Female | Samoa | MS | -0.14<br>(-1.79 to 1.53) | 0.862 |
| Childhood | Prevalence | Male | Samoa | MS | 0.26<br>(-1.36 to 1.92) | 0.744 |
| Childhood | Prevalence | Both | Samoa | RA | -0.2<br>(-1.63 to 1.25) | 0.777 |
| Childhood | Prevalence | Female | Samoa | RA | 0.28<br>(-1.2 to 1.78) | 0.703 |
| Childhood | Prevalence | Male | Samoa | RA | -0.16 | 0.810 |

| | | | | | | |
|---|---|---|---|---|---|---|
| | | | | | (-1.53 to 1.22) | |
| Childhood | Prevalence | Both | San Marino | AA | 0.18 (-1.25 to 1.64) | 0.795 |
| Childhood | Prevalence | Female | San Marino | AA | -0.26 (-1.59 to 1.09) | 0.698 |
| Childhood | Prevalence | Male | San Marino | AA | 0.13 (-1.43 to 1.71) | 0.871 |
| Childhood | Prevalence | Both | San Marino | IBD | 0.07 (-1.45 to 1.6) | 0.931 |
| Childhood | Prevalence | Female | San Marino | IBD | 0.47 (-0.95 to 1.92) | 0.504 |
| Childhood | Prevalence | Male | San Marino | IBD | 0.05 (-1.58 to 1.7) | 0.955 |
| Childhood | Prevalence | Both | San Marino | MS | 0.67 (-0.89 to 2.25) | 0.391 |
| Childhood | Prevalence | Female | San Marino | MS | 0.09 (-1.46 to 1.66) | 0.907 |
| Childhood | Prevalence | Male | San Marino | MS | 0.44 (-1.16 to 2.07) | 0.578 |
| Childhood | Prevalence | Both | San Marino | Psoriasis | -0.42 (-1.79 to 0.98) | 0.545 |
| Childhood | Prevalence | Female | San Marino | Psoriasis | -0.66 (-1.9 to 0.59) | 0.286 |
| Childhood | Prevalence | Male | San Marino | Psoriasis | -0.48 (-2.01 to 1.08) | 0.533 |
| Childhood | Prevalence | Both | Sao Tome and Principe | AA | -0.04 (-0.17 to 0.1) | 0.595 |
| Childhood | Prevalence | Female | Sao Tome and Principe | AA | 0.05 (-0.09 to 0.19) | 0.485 |
| Childhood | Prevalence | Male | Sao Tome and Principe | AA | 0 (-0.15 to 0.15) | 0.970 |
| Childhood | Prevalence | Both | Sao Tome and Principe | T1D | 0.48 (-0.91 to 1.89) | 0.488 |

| | | | | | | |
|---|---|---|---|---|---|---|
| Childhood | Prevalence | Female | Sao Tome and Principe | T1D | 0.09<br>(-1.3 to 1.5) | 0.895 |
| Childhood | Prevalence | Male | Sao Tome and Principe | T1D | 0.46<br>(-0.92 to 1.86) | 0.499 |
| Childhood | Prevalence | Both | Sao Tome and Principe | Psoriasis | -1.12<br>(-2.76 to 0.54) | 0.176 |
| Childhood | Prevalence | Female | Sao Tome and Principe | Psoriasis | -0.84<br>(-2.47 to 0.81) | 0.303 |
| Childhood | Prevalence | Male | Sao Tome and Principe | Psoriasis | -1.03<br>(-2.66 to 0.64) | 0.216 |
| Childhood | Prevalence | Both | Sao Tome and Principe | RHD | 0.15<br>(-0.6 to 0.9) | 0.694 |
| Childhood | Prevalence | Female | Sao Tome and Principe | RHD | -0.04<br>(-0.82 to 0.75) | 0.921 |
| Childhood | Prevalence | Male | Sao Tome and Principe | RHD | 0.12<br>(-0.56 to 0.8) | 0.725 |
| Childhood | Prevalence | Both | Saudi Arabia | AA | 0.01<br>(-0.17 to 0.2) | 0.903 |
| Childhood | Prevalence | Female | Saudi Arabia | AA | 0.04<br>(-0.21 to 0.29) | 0.762 |
| Childhood | Prevalence | Male | Saudi Arabia | AA | -0.03<br>(-0.26 to 0.19) | 0.762 |
| Childhood | Prevalence | Both | Saudi Arabia | Psoriasis | -1.77<br>(-2.57 to -0.96) | < 0.001 |
| Childhood | Prevalence | Female | Saudi Arabia | Psoriasis | -1.59<br>(-2.35 to -0.82) | < 0.001 |
| Childhood | Prevalence | Male | Saudi Arabia | Psoriasis | -1.79<br>(-2.64 to -0.93) | < 0.001 |
| Childhood | Prevalence | Both | Saudi Arabia | RHD | -1.34<br>(-4.36 to 1.79) | 0.384 |
| Childhood | Prevalence | Female | Saudi Arabia | RHD | -0.6<br>(-3.7 to 2.6) | 0.701 |
| Childhood | Prevalence | Male | Saudi Arabia | RHD | -1.36 | 0.376 |

| | | | | | | |
|---|---|---|---|---|---|---|
| | | | | | (-4.38 to 1.76) | |
| Childhood | Prevalence | Both | Saudi Arabia | RA | 1.18<br>(-1.84 to 4.29) | 0.435 |
| Childhood | Prevalence | Female | Saudi Arabia | RA | 0.44<br>(-2.42 to 3.39) | 0.757 |
| Childhood | Prevalence | Male | Saudi Arabia | RA | 1.18<br>(-2.24 to 4.72) | 0.490 |
| Childhood | Prevalence | Both | Senegal | AA | 0.18<br>(-0.06 to 0.42) | 0.131 |
| Childhood | Prevalence | Female | Senegal | AA | 0.09<br>(-0.13 to 0.32) | 0.397 |
| Childhood | Prevalence | Male | Senegal | AA | 0.14<br>(-0.06 to 0.34) | 0.170 |
| Childhood | Prevalence | Both | Senegal | MS | 0.54<br>(-1.08 to 2.18) | 0.502 |
| Childhood | Prevalence | Female | Senegal | MS | 0.13<br>(-1.5 to 1.78) | 0.876 |
| Childhood | Prevalence | Male | Senegal | MS | 0.46<br>(-1.18 to 2.14) | 0.571 |
| Childhood | Prevalence | Both | Senegal | Psoriasis | -0.65<br>(-1.37 to 0.07) | 0.074 |
| Childhood | Prevalence | Female | Senegal | Psoriasis | -0.54<br>(-1.26 to 0.19) | 0.139 |
| Childhood | Prevalence | Male | Senegal | Psoriasis | -0.56<br>(-1.29 to 0.17) | 0.125 |
| Childhood | Prevalence | Both | Senegal | RA | 0.34<br>(-0.88 to 1.58) | 0.571 |
| Childhood | Prevalence | Female | Senegal | RA | 0.67<br>(-0.72 to 2.09) | 0.332 |
| Childhood | Prevalence | Male | Senegal | RA | 0.37<br>(-0.59 to 1.35) | 0.435 |
| Childhood | Prevalence | Both | Serbia | AA | 0.03<br>(-0.22 to 0.28) | 0.818 |

| | | | | | | |
|---|---|---|---|---|---|---|
| Childhood | Prevalence | Female | Serbia | AA | -0.04 (-0.29 to 0.21) | 0.762 |
| Childhood | Prevalence | Male | Serbia | AA | 0.04 (-0.21 to 0.28) | 0.762 |
| Childhood | Prevalence | Both | Serbia | IBD | 0.6 (-0.71 to 1.93) | 0.358 |
| Childhood | Prevalence | Female | Serbia | IBD | 0.25 (-0.93 to 1.45) | 0.669 |
| Childhood | Prevalence | Male | Serbia | IBD | 0.64 (-0.75 to 2.05) | 0.354 |
| Childhood | Prevalence | Both | Serbia | RHD | 0.04 (-0.93 to 1.02) | 0.939 |
| Childhood | Prevalence | Female | Serbia | RHD | -1.09 (-1.62 to -0.55) | < 0.001 |
| Childhood | Prevalence | Male | Serbia | RHD | -0.82 (-1.36 to -0.27) | 0.005 |
| Childhood | Prevalence | Both | Serbia | RA | 2.09 (0.87 to 3.31) | 0.001 |
| Childhood | Prevalence | Female | Serbia | RA | 2.09 (0.87 to 3.33) | 0.001 |
| Childhood | Prevalence | Male | Serbia | RA | 2.45 (1.23 to 3.68) | < 0.001 |
| Childhood | Prevalence | Both | Seychelles | MS | 0.51 (-1.2 to 2.25) | 0.549 |
| Childhood | Prevalence | Female | Seychelles | MS | 0.26 (-1.47 to 2.02) | 0.766 |
| Childhood | Prevalence | Male | Seychelles | MS | 0.28 (-1.4 to 1.99) | 0.738 |
| Childhood | Prevalence | Both | Seychelles | Psoriasis | -0.26 (-1.26 to 0.75) | 0.597 |
| Childhood | Prevalence | Female | Seychelles | Psoriasis | -0.45 (-1.18 to 0.29) | 0.225 |
| Childhood | Prevalence | Male | Seychelles | Psoriasis | -0.26 | 0.674 |

| | | | | | | |
|---|---|---|---|---|---|---|
| | | | | | (-1.5 to 0.99) | |
| Childhood | Prevalence | Both | Seychelles | RHD | -0.14<br>(-0.44 to 0.15) | 0.317 |
| Childhood | Prevalence | Female | Seychelles | RHD | -0.03<br>(-0.38 to 0.33) | 0.885 |
| Childhood | Prevalence | Male | Seychelles | RHD | -0.14<br>(-0.37 to 0.08) | 0.207 |
| Childhood | Prevalence | Both | Sierra Leone | IBD | -0.47<br>(-3.43 to 2.59) | 0.754 |
| Childhood | Prevalence | Female | Sierra Leone | IBD | 0.17<br>(-2.73 to 3.15) | 0.908 |
| Childhood | Prevalence | Male | Sierra Leone | IBD | -0.28<br>(-3.43 to 2.97) | 0.858 |
| Childhood | Prevalence | Both | Sierra Leone | Psoriasis | -0.61<br>(-1.32 to 0.11) | 0.091 |
| Childhood | Prevalence | Female | Sierra Leone | Psoriasis | -0.52<br>(-1.24 to 0.2) | 0.152 |
| Childhood | Prevalence | Male | Sierra Leone | Psoriasis | -0.53<br>(-1.25 to 0.19) | 0.142 |
| Childhood | Prevalence | Both | Sierra Leone | RHD | 0.15<br>(0.07 to 0.23) | 0.001 |
| Childhood | Prevalence | Female | Sierra Leone | RHD | 0.2<br>(0.09 to 0.31) | 0.001 |
| Childhood | Prevalence | Male | Sierra Leone | RHD | 0.14<br>(0.02 to 0.27) | 0.019 |
| Childhood | Prevalence | Both | Sierra Leone | RA | 0.21<br>(-0.99 to 1.42) | 0.722 |
| Childhood | Prevalence | Female | Sierra Leone | RA | 0.6<br>(-0.79 to 2.02) | 0.384 |
| Childhood | Prevalence | Male | Sierra Leone | RA | 0.36<br>(-0.62 to 1.35) | 0.462 |
| Childhood | Prevalence | Both | Singapore | AA | -0.09<br>(-0.33 to 0.15) | 0.450 |

| | | | | | | |
|---|---|---|---|---|---|---|
| Childhood | Prevalence | Female | Singapore | AA | -0.08 (-0.61 to 0.45) | 0.762 |
| Childhood | Prevalence | Male | Singapore | AA | -0.03 (-0.27 to 0.2) | 0.762 |
| Childhood | Prevalence | Both | Singapore | IBD | -0.79 (-2.27 to 0.72) | 0.292 |
| Childhood | Prevalence | Female | Singapore | IBD | -0.38 (-1.86 to 1.12) | 0.607 |
| Childhood | Prevalence | Male | Singapore | IBD | -0.87 (-2.37 to 0.66) | 0.251 |
| Childhood | Prevalence | Both | Slovakia | AA | -0.07 (-0.33 to 0.18) | 0.562 |
| Childhood | Prevalence | Female | Slovakia | AA | 0.04 (-0.21 to 0.29) | 0.762 |
| Childhood | Prevalence | Male | Slovakia | AA | -0.04 (-0.28 to 0.21) | 0.762 |
| Childhood | Prevalence | Both | Slovakia | IBD | 0.12 (-0.79 to 1.03) | 0.793 |
| Childhood | Prevalence | Female | Slovakia | IBD | -0.15 (-1.11 to 0.82) | 0.752 |
| Childhood | Prevalence | Male | Slovakia | IBD | 0.08 (-0.83 to 1) | 0.856 |
| Childhood | Prevalence | Both | Slovakia | MS | 0.46 (-1.17 to 2.11) | 0.570 |
| Childhood | Prevalence | Female | Slovakia | MS | 1.04 (-0.6 to 2.7) | 0.206 |
| Childhood | Prevalence | Male | Slovakia | MS | 0.22 (-1.37 to 1.83) | 0.781 |
| Childhood | Prevalence | Both | Slovakia | Psoriasis | -1.04 (-1.79 to -0.28) | 0.009 |
| Childhood | Prevalence | Female | Slovakia | Psoriasis | -0.9 (-1.43 to -0.38) | 0.002 |
| Childhood | Prevalence | Male | Slovakia | Psoriasis | -1.06 | 0.031 |

| | | | | | | |
|---|---|---|---|---|---|---|
| | | | | | (-2.01 to -0.1) | |
| Childhood | Prevalence | Both | Slovakia | RHD | 0.01<br>(-0.76 to 0.77) | 0.988 |
| Childhood | Prevalence | Female | Slovakia | RHD | -0.07<br>(-0.52 to 0.39) | 0.763 |
| Childhood | Prevalence | Male | Slovakia | RHD | -0.02<br>(-1.15 to 1.13) | 0.978 |
| Childhood | Prevalence | Both | Slovakia | RA | 0.8<br>(-0.68 to 2.31) | 0.280 |
| Childhood | Prevalence | Female | Slovakia | RA | 0.43<br>(-1.09 to 1.96) | 0.570 |
| Childhood | Prevalence | Male | Slovakia | RA | 0.82<br>(-0.62 to 2.29) | 0.254 |
| Childhood | Prevalence | Both | Slovenia | AA | -0.33<br>(-0.57 to -0.09) | 0.010 |
| Childhood | Prevalence | Female | Slovenia | AA | -0.29<br>(-0.54 to -0.04) | 0.023 |
| Childhood | Prevalence | Male | Slovenia | AA | -0.33<br>(-0.58 to -0.08) | 0.012 |
| Childhood | Prevalence | Both | Slovenia | IBD | 0.24<br>(-0.85 to 1.34) | 0.660 |
| Childhood | Prevalence | Female | Slovenia | IBD | 0.02<br>(-1.02 to 1.08) | 0.962 |
| Childhood | Prevalence | Male | Slovenia | IBD | 0.19<br>(-0.93 to 1.34) | 0.729 |
| Childhood | Prevalence | Both | Slovenia | MS | 0.28<br>(-1.46 to 2.06) | 0.744 |
| Childhood | Prevalence | Female | Slovenia | MS | 0.75<br>(-0.92 to 2.45) | 0.367 |
| Childhood | Prevalence | Male | Slovenia | MS | 0.13<br>(-1.65 to 1.93) | 0.887 |
| Childhood | Prevalence | Both | Slovenia | Psoriasis | -0.89<br>(-1.64 to -0.13) | 0.023 |

| | | | | | | |
|---|---|---|---|---|---|---|
| Childhood | Prevalence | Female | Slovenia | Psoriasis | -0.76<br>(-1.29 to -0.24) | 0.006 |
| Childhood | Prevalence | Male | Slovenia | Psoriasis | -0.89<br>(-1.83 to 0.07) | 0.068 |
| Childhood | Prevalence | Both | Slovenia | RHD | -3.44<br>(-9.35 to 2.86) | 0.267 |
| Childhood | Prevalence | Female | Slovenia | RHD | -1.81<br>(-8.16 to 4.98) | 0.581 |
| Childhood | Prevalence | Male | Slovenia | RHD | -3.32<br>(-8.82 to 2.52) | 0.248 |
| Childhood | Prevalence | Both | Slovenia | RA | 0.64<br>(-0.6 to 1.9) | 0.299 |
| Childhood | Prevalence | Female | Slovenia | RA | 0.98<br>(-0.31 to 2.29) | 0.131 |
| Childhood | Prevalence | Male | Slovenia | RA | 0.71<br>(-0.61 to 2.04) | 0.281 |
| Childhood | Prevalence | Both | Solomon Islands | AA | -0.01<br>(-0.26 to 0.25) | 0.963 |
| Childhood | Prevalence | Female | Solomon Islands | AA | 0.04<br>(-0.23 to 0.31) | 0.762 |
| Childhood | Prevalence | Male | Solomon Islands | AA | -0.04<br>(-0.3 to 0.23) | 0.762 |
| Childhood | Prevalence | Both | Solomon Islands | MS | 0.31<br>(-1.21 to 1.86) | 0.680 |
| Childhood | Prevalence | Female | Solomon Islands | MS | -0.16<br>(-1.67 to 1.38) | 0.835 |
| Childhood | Prevalence | Male | Solomon Islands | MS | 0.25<br>(-1.3 to 1.83) | 0.743 |
| Childhood | Prevalence | Both | Solomon Islands | RHD | 0.07<br>(0.01 to 0.14) | 0.029 |
| Childhood | Prevalence | Female | Solomon Islands | RHD | 0.08<br>(0.05 to 0.1) | < 0.001 |
| Childhood | Prevalence | Male | Solomon Islands | RHD | 0.05 | 0.328 |

| | | | | | | |
|---|---|---|---|---|---|---|
| | | | | | (-0.06 to 0.17) | |
| Childhood | Prevalence | Both | Solomon Islands | RA | 0.17<br>(-1.2 to 1.56) | 0.805 |
| Childhood | Prevalence | Female | Solomon Islands | RA | 0.47<br>(-0.92 to 1.89) | 0.496 |
| Childhood | Prevalence | Male | Solomon Islands | RA | -0.01<br>(-1.41 to 1.41) | 0.990 |
| Childhood | Prevalence | Both | Somalia | AA | -0.04<br>(-0.31 to 0.24) | 0.787 |
| Childhood | Prevalence | Female | Somalia | AA | 0.04<br>(-0.22 to 0.3) | 0.762 |
| Childhood | Prevalence | Male | Somalia | AA | -0.03<br>(-0.27 to 0.2) | 0.763 |
| Childhood | Prevalence | Both | Somalia | T1D | 0.33<br>(-0.46 to 1.14) | 0.400 |
| Childhood | Prevalence | Female | Somalia | T1D | 0.1<br>(-0.73 to 0.93) | 0.807 |
| Childhood | Prevalence | Male | Somalia | T1D | 0.32<br>(-0.46 to 1.1) | 0.407 |
| Childhood | Prevalence | Both | Somalia | Psoriasis | 0.22<br>(-0.46 to 0.9) | 0.522 |
| Childhood | Prevalence | Female | Somalia | Psoriasis | 0.04<br>(-0.64 to 0.73) | 0.901 |
| Childhood | Prevalence | Male | Somalia | Psoriasis | 0.24<br>(-0.44 to 0.93) | 0.473 |
| Childhood | Prevalence | Both | Somalia | RA | 0.69<br>(0.09 to 1.29) | 0.026 |
| Childhood | Prevalence | Female | Somalia | RA | 0.58<br>(-0.06 to 1.23) | 0.076 |
| Childhood | Prevalence | Male | Somalia | RA | 0.76<br>(0.16 to 1.36) | 0.014 |
| Childhood | Prevalence | Both | South Africa | RA | -0.29<br>(-2.02 to 1.47) | 0.740 |

| | | | | | | |
|---|---|---|---|---|---|---|
| Childhood | Prevalence | Female | South Africa | RA | 0.03 (-1.62 to 1.71) | 0.971 |
| Childhood | Prevalence | Male | South Africa | RA | -0.13 (-1.99 to 1.75) | 0.884 |
| Childhood | Prevalence | Both | South Sudan | AA | 0.13 (-0.13 to 0.39) | 0.305 |
| Childhood | Prevalence | Female | South Sudan | AA | 0.05 (-0.13 to 0.22) | 0.586 |
| Childhood | Prevalence | Male | South Sudan | AA | -0.01 (-0.18 to 0.16) | 0.886 |
| Childhood | Prevalence | Both | South Sudan | T1D | -0.13 (-2.88 to 2.7) | 0.923 |
| Childhood | Prevalence | Female | South Sudan | T1D | 0.54 (-2.22 to 3.38) | 0.694 |
| Childhood | Prevalence | Male | South Sudan | T1D | -0.14 (-2.89 to 2.69) | 0.921 |
| Childhood | Prevalence | Both | South Sudan | IBD | -0.26 (-2.31 to 1.82) | 0.796 |
| Childhood | Prevalence | Female | South Sudan | IBD | 0.31 (-1.56 to 2.22) | 0.737 |
| Childhood | Prevalence | Male | South Sudan | IBD | -0.44 (-2.69 to 1.87) | 0.696 |
| Childhood | Prevalence | Both | South Sudan | MS | 0.44 (-1.3 to 2.2) | 0.610 |
| Childhood | Prevalence | Female | South Sudan | MS | -0.18 (-1.9 to 1.56) | 0.831 |
| Childhood | Prevalence | Male | South Sudan | MS | 0.34 (-1.4 to 2.12) | 0.691 |
| Childhood | Prevalence | Both | South Sudan | RHD | 0.12 (-0.02 to 0.26) | 0.084 |
| Childhood | Prevalence | Female | South Sudan | RHD | 0.15 (-0.04 to 0.34) | 0.116 |
| Childhood | Prevalence | Male | South Sudan | RHD | 0.08 | 0.079 |

| | | | | | | |
|---|---|---|---|---|---|---|
| | | | | | (-0.01 to 0.17) | |
| Childhood | Prevalence | Both | Spain | AA | -0.05<br>(-0.18 to 0.09) | 0.473 |
| Childhood | Prevalence | Female | Spain | AA | 0.07<br>(-0.58 to 0.73) | 0.820 |
| Childhood | Prevalence | Male | Spain | AA | 0.12<br>(-0.66 to 0.92) | 0.749 |
| Childhood | Prevalence | Both | Spain | T1D | 1.64<br>(0.28 to 3.01) | 0.019 |
| Childhood | Prevalence | Female | Spain | T1D | 1.8<br>(0.42 to 3.19) | 0.012 |
| Childhood | Prevalence | Male | Spain | T1D | 1.9<br>(0.55 to 3.26) | 0.007 |
| Childhood | Prevalence | Both | Spain | MS | 4.21<br>(2.12 to 6.34) | < 0.001 |
| Childhood | Prevalence | Female | Spain | MS | 4.22<br>(2.33 to 6.16) | < 0.001 |
| Childhood | Prevalence | Male | Spain | MS | 4.29<br>(2.15 to 6.47) | < 0.001 |
| Childhood | Prevalence | Both | Spain | Psoriasis | -0.5<br>(-1.36 to 0.37) | 0.251 |
| Childhood | Prevalence | Female | Spain | Psoriasis | -0.35<br>(-1.08 to 0.38) | 0.331 |
| Childhood | Prevalence | Male | Spain | Psoriasis | -0.46<br>(-1.46 to 0.55) | 0.358 |
| Childhood | Prevalence | Both | Spain | RA | 0.15<br>(-1.2 to 1.52) | 0.821 |
| Childhood | Prevalence | Female | Spain | RA | 0.49<br>(-0.92 to 1.92) | 0.485 |
| Childhood | Prevalence | Male | Spain | RA | 0.16<br>(-1.08 to 1.42) | 0.792 |
| Childhood | Prevalence | Both | Sri Lanka | AA | -0.35<br>(-1.81 to 1.13) | 0.626 |

| | | | | | | |
|---|---|---|---|---|---|---|
| Childhood | Prevalence | Female | Sri Lanka | AA | 0.03 (-1.42 to 1.52) | 0.962 |
| Childhood | Prevalence | Male | Sri Lanka | AA | -0.36 (-1.8 to 1.11) | 0.621 |
| Childhood | Prevalence | Both | Sri Lanka | MS | -0.12 (-1.77 to 1.55) | 0.882 |
| Childhood | Prevalence | Female | Sri Lanka | MS | 0.32 (-1.32 to 1.99) | 0.696 |
| Childhood | Prevalence | Male | Sri Lanka | MS | -0.14 (-1.82 to 1.56) | 0.867 |
| Childhood | Prevalence | Both | Sri Lanka | Psoriasis | -0.61 (-2.48 to 1.3) | 0.517 |
| Childhood | Prevalence | Female | Sri Lanka | Psoriasis | -0.99 (-2.4 to 0.43) | 0.163 |
| Childhood | Prevalence | Male | Sri Lanka | Psoriasis | -0.57 (-2.99 to 1.92) | 0.640 |
| Childhood | Prevalence | Both | Sri Lanka | RHD | -1.27 (-2.17 to -0.37) | 0.007 |
| Childhood | Prevalence | Female | Sri Lanka | RHD | -1.77 (-2.87 to -0.66) | 0.003 |
| Childhood | Prevalence | Male | Sri Lanka | RHD | -0.98 (-1.63 to -0.33) | 0.005 |
| Childhood | Prevalence | Both | Sri Lanka | RA | 0.82 (-0.66 to 2.32) | 0.268 |
| Childhood | Prevalence | Female | Sri Lanka | RA | 0.42 (-1.11 to 1.98) | 0.578 |
| Childhood | Prevalence | Male | Sri Lanka | RA | 0.69 (-0.7 to 2.09) | 0.319 |
| Childhood | Prevalence | Both | Sudan | AA | -0.05 (-0.28 to 0.17) | 0.642 |
| Childhood | Prevalence | Female | Sudan | AA | 0.04 (-0.21 to 0.29) | 0.762 |
| Childhood | Prevalence | Male | Sudan | AA | -0.03 | 0.762 |

| | | | | | | |
|---|---|---|---|---|---|---|
| | | | | | (-0.26 to 0.19) | |
| Childhood | Prevalence | Both | Sudan | MS | 0.48<br>(-1.18 to 2.17) | 0.561 |
| Childhood | Prevalence | Female | Sudan | MS | 0.88<br>(-0.73 to 2.52) | 0.275 |
| Childhood | Prevalence | Male | Sudan | MS | 0.56<br>(-1.17 to 2.33) | 0.513 |
| Childhood | Prevalence | Both | Sudan | Psoriasis | -1.08<br>(-1.89 to -0.27) | 0.011 |
| Childhood | Prevalence | Female | Sudan | Psoriasis | -1.29<br>(-2.04 to -0.52) | 0.002 |
| Childhood | Prevalence | Male | Sudan | Psoriasis | -1.05<br>(-1.9 to -0.19) | 0.019 |
| Childhood | Prevalence | Both | Sudan | RHD | 0.19<br>(-0.2 to 0.59) | 0.321 |
| Childhood | Prevalence | Female | Sudan | RHD | 0.1<br>(-0.32 to 0.53) | 0.622 |
| Childhood | Prevalence | Male | Sudan | RHD | 0.18<br>(-0.18 to 0.54) | 0.311 |
| Childhood | Prevalence | Both | Suriname | Psoriasis | -0.86<br>(-2.37 to 0.67) | 0.259 |
| Childhood | Prevalence | Female | Suriname | Psoriasis | -0.52<br>(-2.04 to 1.02) | 0.490 |
| Childhood | Prevalence | Male | Suriname | Psoriasis | -0.83<br>(-2.35 to 0.72) | 0.281 |
| Childhood | Prevalence | Both | Sweden | T1D | -0.15<br>(-1.94 to 1.67) | 0.867 |
| Childhood | Prevalence | Female | Sweden | T1D | 0.55<br>(-0.97 to 2.1) | 0.467 |
| Childhood | Prevalence | Male | Sweden | T1D | -0.4<br>(-2.37 to 1.6) | 0.681 |
| Childhood | Prevalence | Both | Sweden | IBD | -0.67<br>(-8.02 to 7.26) | 0.858 |

| | | | | | | |
|---|---|---|---|---|---|---|
| Childhood | Prevalence | Female | Sweden | IBD | -3.78 (-11.12 to 4.17) | 0.329 |
| Childhood | Prevalence | Male | Sweden | IBD | -0.21 (-7.49 to 7.64) | 0.955 |
| Childhood | Prevalence | Both | Sweden | MS | 0.76 (-0.93 to 2.48) | 0.368 |
| Childhood | Prevalence | Female | Sweden | MS | 0.45 (-1.24 to 2.16) | 0.593 |
| Childhood | Prevalence | Male | Sweden | MS | 0.59 (-1.13 to 2.34) | 0.488 |
| Childhood | Prevalence | Both | Sweden | RA | 0.15 (-1 to 1.32) | 0.790 |
| Childhood | Prevalence | Female | Sweden | RA | 0.46 (-0.74 to 1.67) | 0.443 |
| Childhood | Prevalence | Male | Sweden | RA | 0.26 (-0.85 to 1.37) | 0.638 |
| Childhood | Prevalence | Both | Switzerland | AA | -0.14 (-1.55 to 1.29) | 0.842 |
| Childhood | Prevalence | Female | Switzerland | AA | -0.49 (-1.96 to 1) | 0.503 |
| Childhood | Prevalence | Male | Switzerland | AA | -0.21 (-1.51 to 1.11) | 0.746 |
| Childhood | Prevalence | Both | Switzerland | MS | 0.39 (-1.32 to 2.12) | 0.649 |
| Childhood | Prevalence | Female | Switzerland | MS | 0.08 (-1.56 to 1.75) | 0.920 |
| Childhood | Prevalence | Male | Switzerland | MS | 0.3 (-1.5 to 2.12) | 0.741 |
| Childhood | Prevalence | Both | Switzerland | Psoriasis | -0.2 (-1.65 to 1.27) | 0.784 |
| Childhood | Prevalence | Female | Switzerland | Psoriasis | -0.52 (-1.84 to 0.81) | 0.426 |
| Childhood | Prevalence | Male | Switzerland | Psoriasis | -0.2 | 0.799 |

| | | | | | | |
|---|---|---|---|---|---|---|
| | | | | | (-1.77 to 1.4) | |
| Childhood | Prevalence | Both | Switzerland | RA | 0.1<br>(-0.98 to 1.2) | 0.846 |
| Childhood | Prevalence | Female | Switzerland | RA | 0.41<br>(-0.7 to 1.53) | 0.459 |
| Childhood | Prevalence | Male | Switzerland | RA | 0.18<br>(-0.91 to 1.27) | 0.743 |
| Childhood | Prevalence | Both | Syrian Arab Republic | AA | 0.12<br>(-0.09 to 0.34) | 0.246 |
| Childhood | Prevalence | Female | Syrian Arab Republic | AA | 0.12<br>(-0.06 to 0.3) | 0.180 |
| Childhood | Prevalence | Male | Syrian Arab Republic | AA | 0.05<br>(-0.14 to 0.24) | 0.603 |
| Childhood | Prevalence | Both | Syrian Arab Republic | T1D | 2.43<br>(1.3 to 3.56) | < 0.001 |
| Childhood | Prevalence | Female | Syrian Arab Republic | T1D | 2.08<br>(1 to 3.18) | < 0.001 |
| Childhood | Prevalence | Male | Syrian Arab Republic | T1D | 2.44<br>(1.29 to 3.61) | < 0.001 |
| Childhood | Prevalence | Both | Syrian Arab Republic | IBD | 1.78<br>(0.64 to 2.93) | 0.003 |
| Childhood | Prevalence | Female | Syrian Arab Republic | IBD | 1.49<br>(0.53 to 2.45) | 0.004 |
| Childhood | Prevalence | Male | Syrian Arab Republic | IBD | 1.87<br>(0.51 to 3.25) | 0.009 |
| Childhood | Prevalence | Both | Syrian Arab Republic | Psoriasis | -0.88<br>(-2.16 to 0.42) | 0.178 |
| Childhood | Prevalence | Female | Syrian Arab Republic | Psoriasis | -1.19<br>(-2.37 to 0) | 0.050 |
| Childhood | Prevalence | Male | Syrian Arab Republic | Psoriasis | -0.91<br>(-2.33 to 0.54) | 0.207 |
| Childhood | Prevalence | Both | Syrian Arab Republic | RHD | 0.11<br>(-0.06 to 0.28) | 0.181 |

| | | | | | | |
|---|---|---|---|---|---|---|
| Childhood | Prevalence | Female | Syrian Arab Republic | RHD | 0.15<br>(-0.07 to 0.38) | 0.177 |
| Childhood | Prevalence | Male | Syrian Arab Republic | RHD | 0.11<br>(-0.02 to 0.23) | 0.097 |
| Childhood | Prevalence | Both | Taiwan (Province of China) | AA | -0.13<br>(-2.87 to 2.69) | 0.925 |
| Childhood | Prevalence | Female | Taiwan (Province of China) | AA | -0.94<br>(-3.72 to 1.93) | 0.504 |
| Childhood | Prevalence | Male | Taiwan (Province of China) | AA | -0.05<br>(-2.65 to 2.61) | 0.967 |
| Childhood | Prevalence | Both | Taiwan (Province of China) | T1D | 2.19<br>(0.86 to 3.55) | 0.002 |
| Childhood | Prevalence | Female | Taiwan (Province of China) | T1D | 2.04<br>(0.82 to 3.27) | 0.002 |
| Childhood | Prevalence | Male | Taiwan (Province of China) | T1D | 2.04<br>(0.59 to 3.5) | 0.007 |
| Childhood | Prevalence | Both | Taiwan (Province of China) | MS | 3.8<br>(2.41 to 5.2) | < 0.001 |
| Childhood | Prevalence | Female | Taiwan (Province of China) | MS | 3.72<br>(2.25 to 5.21) | < 0.001 |
| Childhood | Prevalence | Male | Taiwan (Province of China) | MS | 3.11<br>(1.85 to 4.39) | < 0.001 |
| Childhood | Prevalence | Both | Taiwan (Province of China) | RA | 0.04<br>(-1.27 to 1.38) | 0.946 |
| Childhood | Prevalence | Female | Taiwan (Province of China) | RA | 0.29<br>(-1.13 to 1.74) | 0.679 |
| Childhood | Prevalence | Male | Taiwan (Province of China) | RA | 0.1<br>(-0.98 to 1.18) | 0.857 |
| Childhood | Prevalence | Both | Tajikistan | MS | 0.68<br>(-1.06 to 2.44) | 0.433 |
| Childhood | Prevalence | Female | Tajikistan | MS | 0.26<br>(-1.48 to 2.03) | 0.765 |
| Childhood | Prevalence | Male | Tajikistan | MS | 0.6 | 0.484 |

| | | | | | | |
|---|---|---|---|---|---|---|
| | | | | | (-1.12 to 2.34) | |
| Childhood | Prevalence | Both | Tajikistan | RA | 0.79<br>(-0.75 to 2.35) | 0.306 |
| Childhood | Prevalence | Female | Tajikistan | RA | 0.28<br>(-1.28 to 1.86) | 0.717 |
| Childhood | Prevalence | Male | Tajikistan | RA | 0.72<br>(-0.73 to 2.2) | 0.318 |
| Childhood | Prevalence | Both | Thailand | AA | -0.04<br>(-0.31 to 0.23) | 0.756 |
| Childhood | Prevalence | Female | Thailand | AA | 0.04<br>(-0.23 to 0.31) | 0.762 |
| Childhood | Prevalence | Male | Thailand | AA | -0.04<br>(-0.29 to 0.21) | 0.762 |
| Childhood | Prevalence | Both | Thailand | MS | -0.07<br>(-1.69 to 1.58) | 0.935 |
| Childhood | Prevalence | Female | Thailand | MS | 0.32<br>(-1.31 to 1.98) | 0.688 |
| Childhood | Prevalence | Male | Thailand | MS | -0.08<br>(-1.7 to 1.56) | 0.918 |
| Childhood | Prevalence | Both | Thailand | RA | 0.73<br>(-2.09 to 3.62) | 0.605 |
| Childhood | Prevalence | Female | Thailand | RA | 0.18<br>(-2.46 to 2.88) | 0.893 |
| Childhood | Prevalence | Male | Thailand | RA | 0.6<br>(-2.41 to 3.69) | 0.691 |
| Childhood | Prevalence | Both | Timor-Leste | T1D | 0.61<br>(-0.43 to 1.65) | 0.240 |
| Childhood | Prevalence | Female | Timor-Leste | T1D | 0.84<br>(-0.11 to 1.79) | 0.081 |
| Childhood | Prevalence | Male | Timor-Leste | T1D | 0.66<br>(-0.42 to 1.76) | 0.221 |
| Childhood | Prevalence | Both | Timor-Leste | IBD | 0.55<br>(-0.97 to 2.1) | 0.465 |

| | | | | | | |
|---|---|---|---|---|---|---|
| Childhood | Prevalence | Female | Timor-Leste | IBD | 1.02<br>(-0.6 to 2.67) | 0.210 |
| Childhood | Prevalence | Male | Timor-Leste | IBD | 0.57<br>(-0.84 to 2.01) | 0.416 |
| Childhood | Prevalence | Both | Timor-Leste | MS | 0<br>(-1.62 to 1.64) | 0.998 |
| Childhood | Prevalence | Female | Timor-Leste | MS | 0.46<br>(-1.17 to 2.11) | 0.570 |
| Childhood | Prevalence | Male | Timor-Leste | MS | 0.07<br>(-1.55 to 1.73) | 0.926 |
| Childhood | Prevalence | Both | Timor-Leste | Psoriasis | -1.12<br>(-2.1 to -0.13) | 0.028 |
| Childhood | Prevalence | Female | Timor-Leste | Psoriasis | -0.93<br>(-1.68 to -0.18) | 0.017 |
| Childhood | Prevalence | Male | Timor-Leste | Psoriasis | -1.15<br>(-2.36 to 0.08) | 0.066 |
| Childhood | Prevalence | Both | Timor-Leste | RHD | 0.05<br>(-0.23 to 0.34) | 0.702 |
| Childhood | Prevalence | Female | Timor-Leste | RHD | 0.15<br>(-0.14 to 0.44) | 0.296 |
| Childhood | Prevalence | Male | Timor-Leste | RHD | 0.06<br>(-0.24 to 0.35) | 0.703 |
| Childhood | Prevalence | Both | Timor-Leste | RA | 0.32<br>(-1.15 to 1.81) | 0.659 |
| Childhood | Prevalence | Female | Timor-Leste | RA | 0.8<br>(-0.73 to 2.35) | 0.293 |
| Childhood | Prevalence | Male | Timor-Leste | RA | 0.27<br>(-1.08 to 1.65) | 0.684 |
| Childhood | Prevalence | Both | Togo | AA | -0.08<br>(-0.32 to 0.16) | 0.508 |
| Childhood | Prevalence | Female | Togo | AA | 0.04<br>(-0.21 to 0.28) | 0.762 |
| Childhood | Prevalence | Male | Togo | AA | -0.04 | 0.762 |

| | | | | | | |
|---|---|---|---|---|---|---|
| | | | | | (-0.27 to 0.2) | |
| Childhood | Prevalence | Both | Togo | Psoriasis | -0.65<br>(-1.43 to 0.13) | 0.100 |
| Childhood | Prevalence | Female | Togo | Psoriasis | -0.56<br>(-1.33 to 0.23) | 0.158 |
| Childhood | Prevalence | Male | Togo | Psoriasis | -0.59<br>(-1.37 to 0.2) | 0.139 |
| Childhood | Prevalence | Both | Togo | RHD | 0.52<br>(-0.05 to 1.09) | 0.071 |
| Childhood | Prevalence | Female | Togo | RHD | 0.66<br>(0.13 to 1.2) | 0.017 |
| Childhood | Prevalence | Male | Togo | RHD | 0.5<br>(-0.11 to 1.12) | 0.106 |
| Childhood | Prevalence | Both | Tokelau | AA | -0.15<br>(-0.44 to 0.14) | 0.296 |
| Childhood | Prevalence | Female | Tokelau | AA | 0.04<br>(-0.23 to 0.31) | 0.762 |
| Childhood | Prevalence | Male | Tokelau | AA | -0.04<br>(-0.3 to 0.23) | 0.762 |
| Childhood | Prevalence | Both | Tokelau | IBD | 0.46<br>(-1.19 to 2.15) | 0.575 |
| Childhood | Prevalence | Female | Tokelau | IBD | 0.76<br>(-1.02 to 2.58) | 0.389 |
| Childhood | Prevalence | Male | Tokelau | IBD | 0.61<br>(-0.89 to 2.13) | 0.415 |
| Childhood | Prevalence | Both | Tokelau | MS | -0.19<br>(-1.8 to 1.45) | 0.815 |
| Childhood | Prevalence | Female | Tokelau | MS | 0.34<br>(-1.27 to 1.98) | 0.674 |
| Childhood | Prevalence | Male | Tokelau | MS | -0.03<br>(-1.65 to 1.62) | 0.967 |
| Childhood | Prevalence | Both | Tokelau | Psoriasis | -0.5<br>(-1.59 to 0.61) | 0.362 |

| | | | | | | |
|---|---|---|---|---|---|---|
| Childhood | Prevalence | Female | Tokelau | Psoriasis | -0.73 (-1.55 to 0.09) | 0.080 |
| Childhood | Prevalence | Male | Tokelau | Psoriasis | -0.52 (-1.84 to 0.82) | 0.430 |
| Childhood | Prevalence | Both | Tokelau | RHD | -0.69 (-2.31 to 0.95) | 0.392 |
| Childhood | Prevalence | Female | Tokelau | RHD | -1.02 (-2.58 to 0.57) | 0.197 |
| Childhood | Prevalence | Male | Tokelau | RHD | -0.48 (-2.15 to 1.23) | 0.569 |
| Childhood | Prevalence | Both | Tokelau | RA | -0.32 (-2.34 to 1.74) | 0.752 |
| Childhood | Prevalence | Female | Tokelau | RA | 0.39 (-1.67 to 2.49) | 0.705 |
| Childhood | Prevalence | Male | Tokelau | RA | -0.22 (-2.2 to 1.8) | 0.824 |
| Childhood | Prevalence | Both | Tonga | T1D | 0.9 (-0.49 to 2.32) | 0.195 |
| Childhood | Prevalence | Female | Tonga | T1D | 0.52 (-0.77 to 1.82) | 0.417 |
| Childhood | Prevalence | Male | Tonga | T1D | 0.93 (-0.57 to 2.44) | 0.216 |
| Childhood | Prevalence | Both | Tonga | IBD | -0.68 (-2.33 to 0.99) | 0.407 |
| Childhood | Prevalence | Female | Tonga | IBD | -0.19 (-2 to 1.67) | 0.838 |
| Childhood | Prevalence | Male | Tonga | IBD | -0.64 (-2.08 to 0.82) | 0.374 |
| Childhood | Prevalence | Both | Tonga | MS | -0.18 (-1.83 to 1.49) | 0.824 |
| Childhood | Prevalence | Female | Tonga | MS | 0.33 (-1.33 to 2.02) | 0.687 |
| Childhood | Prevalence | Male | Tonga | MS | -0.16 | 0.846 |

| | | | | | | |
|---|---|---|---|---|---|---|
| | | | | | (-1.79 to 1.5) | |
| Childhood | Prevalence | Both | Tonga | RA | 1.33 (0.48 to 2.19) | 0.003 |
| Childhood | Prevalence | Female | Tonga | RA | 1.68 (0.78 to 2.58) | 0.001 |
| Childhood | Prevalence | Male | Tonga | RA | 1.27 (0.43 to 2.1) | 0.004 |
| Childhood | Prevalence | Both | Trinidad and Tobago | AA | 0.03 (-0.22 to 0.27) | 0.824 |
| Childhood | Prevalence | Female | Trinidad and Tobago | AA | -0.04 (-0.29 to 0.21) | 0.762 |
| Childhood | Prevalence | Male | Trinidad and Tobago | AA | 0.04 (-0.21 to 0.28) | 0.762 |
| Childhood | Prevalence | Both | Trinidad and Tobago | MS | 0.39 (-1.36 to 2.18) | 0.655 |
| Childhood | Prevalence | Female | Trinidad and Tobago | MS | 0.92 (-0.82 to 2.7) | 0.289 |
| Childhood | Prevalence | Male | Trinidad and Tobago | MS | 0.09 (-1.72 to 1.93) | 0.921 |
| Childhood | Prevalence | Both | Trinidad and Tobago | Psoriasis | -0.43 (-1.15 to 0.3) | 0.238 |
| Childhood | Prevalence | Female | Trinidad and Tobago | Psoriasis | -0.57 (-1.28 to 0.15) | 0.114 |
| Childhood | Prevalence | Male | Trinidad and Tobago | Psoriasis | -0.4 (-1.14 to 0.34) | 0.278 |
| Childhood | Prevalence | Both | Trinidad and Tobago | RHD | 0.31 (-0.02 to 0.65) | 0.066 |
| Childhood | Prevalence | Female | Trinidad and Tobago | RHD | 0.37 (0.09 to 0.65) | 0.011 |
| Childhood | Prevalence | Male | Trinidad and Tobago | RHD | 0.3 (-0.11 to 0.72) | 0.142 |
| Childhood | Prevalence | Both | Tunisia | IBD | -0.01 (-1.6 to 1.59) | 0.986 |

| | | | | | | |
|---|---|---|---|---|---|---|
| Childhood | Prevalence | Female | Tunisia | IBD | 0.02 (-1.48 to 1.55) | 0.976 |
| Childhood | Prevalence | Male | Tunisia | IBD | 0.45 (-1.26 to 2.18) | 0.600 |
| Childhood | Prevalence | Both | Tunisia | MS | 0.83 (-1.41 to 3.12) | 0.457 |
| Childhood | Prevalence | Female | Tunisia | MS | 0.21 (-2.01 to 2.49) | 0.847 |
| Childhood | Prevalence | Male | Tunisia | MS | 0.95 (-1.23 to 3.16) | 0.383 |
| Childhood | Prevalence | Both | Tunisia | Psoriasis | -1.59 (-3.54 to 0.39) | 0.111 |
| Childhood | Prevalence | Female | Tunisia | Psoriasis | -1.14 (-2.93 to 0.68) | 0.209 |
| Childhood | Prevalence | Male | Tunisia | Psoriasis | -1.63 (-3.77 to 0.55) | 0.136 |
| Childhood | Prevalence | Both | Tunisia | RHD | -0.95 (-3.78 to 1.97) | 0.508 |
| Childhood | Prevalence | Female | Tunisia | RHD | -1.82 (-4.46 to 0.9) | 0.179 |
| Childhood | Prevalence | Male | Tunisia | RHD | -0.71 (-3.73 to 2.41) | 0.640 |
| Childhood | Prevalence | Both | Turkey | T1D | 2.31 (1.31 to 3.31) | < 0.001 |
| Childhood | Prevalence | Female | Turkey | T1D | 1.98 (1.01 to 2.96) | < 0.001 |
| Childhood | Prevalence | Male | Turkey | T1D | 2.35 (1.33 to 3.37) | < 0.001 |
| Childhood | Prevalence | Both | Turkey | MS | -0.08 (-1.71 to 1.58) | 0.924 |
| Childhood | Prevalence | Female | Turkey | MS | 0.23 (-1.36 to 1.84) | 0.771 |
| Childhood | Prevalence | Male | Turkey | MS | -0.07 | 0.930 |

| | | | | | | |
|---|---|---|---|---|---|---|
| | | | | | (-1.76 to 1.64) | |
| Childhood | Prevalence | Both | Turkey | RHD | -1.11 (-1.9 to -0.3) | 0.009 |
| Childhood | Prevalence | Female | Turkey | RHD | -1.36 (-2.22 to -0.49) | 0.003 |
| Childhood | Prevalence | Male | Turkey | RHD | -1.15 (-1.84 to -0.44) | 0.002 |
| Childhood | Prevalence | Both | Turkey | RA | 1.34 (-0.14 to 2.85) | 0.075 |
| Childhood | Prevalence | Female | Turkey | RA | 1 (-0.46 to 2.48) | 0.172 |
| Childhood | Prevalence | Male | Turkey | RA | 1.34 (-0.15 to 2.85) | 0.075 |
| Childhood | Prevalence | Both | Turkmenistan | AA | -0.05 (-0.19 to 0.08) | 0.424 |
| Childhood | Prevalence | Female | Turkmenistan | AA | 0.02 (-0.07 to 0.11) | 0.621 |
| Childhood | Prevalence | Male | Turkmenistan | AA | 0.01 (-0.08 to 0.1) | 0.881 |
| Childhood | Prevalence | Both | Turkmenistan | IBD | -0.08 (-1.6 to 1.46) | 0.914 |
| Childhood | Prevalence | Female | Turkmenistan | IBD | 0.16 (-1.27 to 1.61) | 0.822 |
| Childhood | Prevalence | Male | Turkmenistan | IBD | 0.25 (-1.4 to 1.92) | 0.762 |
| Childhood | Prevalence | Both | Turkmenistan | MS | 0.16 (-1.44 to 1.79) | 0.839 |
| Childhood | Prevalence | Female | Turkmenistan | MS | 0.44 (-1.07 to 1.96) | 0.560 |
| Childhood | Prevalence | Male | Turkmenistan | MS | 0.24 (-1.56 to 2.08) | 0.789 |
| Childhood | Prevalence | Both | Turkmenistan | Psoriasis | -1.12 (-1.86 to -0.37) | 0.005 |

| | | | | | | |
|---|---|---|---|---|---|---|
| Childhood | Prevalence | Female | Turkmenistan | Psoriasis | -0.97 (-1.51 to -0.42) | 0.001 |
| Childhood | Prevalence | Male | Turkmenistan | Psoriasis | -1.11 (-2.05 to -0.16) | 0.023 |
| Childhood | Prevalence | Both | Turkmenistan | RHD | 0.01 (-0.21 to 0.22) | 0.951 |
| Childhood | Prevalence | Female | Turkmenistan | RHD | 0.08 (-0.19 to 0.34) | 0.555 |
| Childhood | Prevalence | Male | Turkmenistan | RHD | 0.03 (-0.12 to 0.18) | 0.727 |
| Childhood | Prevalence | Both | Turkmenistan | RA | 0.62 (-1.37 to 2.65) | 0.532 |
| Childhood | Prevalence | Female | Turkmenistan | RA | 0.11 (-1.8 to 2.06) | 0.905 |
| Childhood | Prevalence | Male | Turkmenistan | RA | 0.7 (-1.34 to 2.78) | 0.490 |
| Childhood | Prevalence | Both | Tuvalu | AA | 0.06 (-0.1 to 0.23) | 0.437 |
| Childhood | Prevalence | Female | Tuvalu | AA | 0.07 (-0.12 to 0.26) | 0.461 |
| Childhood | Prevalence | Male | Tuvalu | AA | 0.1 (-0.09 to 0.29) | 0.285 |
| Childhood | Prevalence | Both | Tuvalu | T1D | 0.62 (0.16 to 1.08) | 0.010 |
| Childhood | Prevalence | Female | Tuvalu | T1D | 0.67 (0.23 to 1.11) | 0.004 |
| Childhood | Prevalence | Male | Tuvalu | T1D | 0.63 (0.14 to 1.13) | 0.014 |
| Childhood | Prevalence | Both | Tuvalu | MS | -0.65 (-3.68 to 2.48) | 0.671 |
| Childhood | Prevalence | Female | Tuvalu | MS | 0.2 (-2.87 to 3.36) | 0.896 |
| Childhood | Prevalence | Male | Tuvalu | MS | -0.47 | 0.751 |

| | | | | | | |
|---|---|---|---|---|---|---|
| | | | | | (-3.44 to 2.59) | |
| Childhood | Prevalence | Both | Tuvalu | Psoriasis | -1.13<br>(-3.23 to 1.01) | 0.286 |
| Childhood | Prevalence | Female | Tuvalu | Psoriasis | -0.59<br>(-2.17 to 1.01) | 0.452 |
| Childhood | Prevalence | Male | Tuvalu | Psoriasis | -1.27<br>(-3.94 to 1.48) | 0.349 |
| Childhood | Prevalence | Both | Tuvalu | RHD | -1.11<br>(-2.06 to -0.14) | 0.027 |
| Childhood | Prevalence | Female | Tuvalu | RHD | -0.85<br>(-1.94 to 0.25) | 0.123 |
| Childhood | Prevalence | Male | Tuvalu | RHD | -0.75<br>(-1.48 to -0.01) | 0.047 |
| Childhood | Prevalence | Both | Tuvalu | RA | 0.46<br>(-1.44 to 2.41) | 0.624 |
| Childhood | Prevalence | Female | Tuvalu | RA | 0.15<br>(-1.67 to 2.01) | 0.869 |
| Childhood | Prevalence | Male | Tuvalu | RA | 0.56<br>(-1.43 to 2.58) | 0.572 |
| Childhood | Prevalence | Both | Uganda | IBD | 1.19<br>(-0.54 to 2.95) | 0.172 |
| Childhood | Prevalence | Female | Uganda | IBD | 1.22<br>(-0.63 to 3.11) | 0.190 |
| Childhood | Prevalence | Male | Uganda | IBD | 1.21<br>(-0.35 to 2.81) | 0.124 |
| Childhood | Prevalence | Both | Uganda | MS | 0.46<br>(-1.25 to 2.2) | 0.590 |
| Childhood | Prevalence | Female | Uganda | MS | -0.09<br>(-1.78 to 1.63) | 0.917 |
| Childhood | Prevalence | Male | Uganda | MS | 0.54<br>(-1.23 to 2.33) | 0.540 |
| Childhood | Prevalence | Both | Uganda | Psoriasis | -1.08<br>(-2.51 to 0.37) | 0.136 |

| | | | | | | |
|---|---|---|---|---|---|---|
| Childhood | Prevalence | Female | Uganda | Psoriasis | -0.77<br>(-2.23 to 0.71) | 0.295 |
| Childhood | Prevalence | Male | Uganda | Psoriasis | -1.04<br>(-2.46 to 0.41) | 0.153 |
| Childhood | Prevalence | Both | Uganda | RHD | 0.22<br>(-0.12 to 0.57) | 0.196 |
| Childhood | Prevalence | Female | Uganda | RHD | 0.15<br>(-0.19 to 0.48) | 0.372 |
| Childhood | Prevalence | Male | Uganda | RHD | 0.23<br>(-0.14 to 0.59) | 0.214 |
| Childhood | Prevalence | Both | Uganda | RA | 1.19<br>(-2.95 to 5.51) | 0.566 |
| Childhood | Prevalence | Female | Uganda | RA | 0.18<br>(-3.63 to 4.14) | 0.925 |
| Childhood | Prevalence | Male | Uganda | RA | 1.14<br>(-3.62 to 6.13) | 0.634 |
| Childhood | Prevalence | Both | Ukraine | IBD | 0.05<br>(-2.52 to 2.69) | 0.969 |
| Childhood | Prevalence | Female | Ukraine | IBD | -0.6<br>(-3.16 to 2.04) | 0.644 |
| Childhood | Prevalence | Male | Ukraine | IBD | 0.04<br>(-2.54 to 2.69) | 0.976 |
| Childhood | Prevalence | Both | Ukraine | MS | -0.49<br>(-1.62 to 0.65) | 0.385 |
| Childhood | Prevalence | Female | Ukraine | MS | -0.8<br>(-1.73 to 0.13) | 0.090 |
| Childhood | Prevalence | Male | Ukraine | MS | -0.44<br>(-1.81 to 0.95) | 0.521 |
| Childhood | Prevalence | Both | Ukraine | RA | -0.12<br>(-1.73 to 1.52) | 0.881 |
| Childhood | Prevalence | Female | Ukraine | RA | 0.31<br>(-1.35 to 1.99) | 0.708 |
| Childhood | Prevalence | Male | Ukraine | RA | -0.15 | 0.846 |

| | | | | | | |
|---|---|---|---|---|---|---|
| | | | | | (-1.73 to 1.45) | |
| Childhood | Prevalence | Both | United Arab Emirates | IBD | 0.77<br>(-1.03 to 2.6) | 0.392 |
| Childhood | Prevalence | Female | United Arab Emirates | IBD | 0.15<br>(-1.77 to 2.1) | 0.878 |
| Childhood | Prevalence | Male | United Arab Emirates | IBD | 0.73<br>(-1.05 to 2.55) | 0.409 |
| Childhood | Prevalence | Both | United Arab Emirates | MS | 0.47<br>(-1.22 to 2.19) | 0.574 |
| Childhood | Prevalence | Female | United Arab Emirates | MS | -0.16<br>(-1.98 to 1.7) | 0.862 |
| Childhood | Prevalence | Male | United Arab Emirates | MS | 0.7<br>(-0.84 to 2.27) | 0.362 |
| Childhood | Prevalence | Both | United Arab Emirates | RHD | -0.11<br>(-0.6 to 0.38) | 0.648 |
| Childhood | Prevalence | Female | United Arab Emirates | RHD | 0.06<br>(-0.52 to 0.64) | 0.828 |
| Childhood | Prevalence | Male | United Arab Emirates | RHD | -0.12<br>(-0.61 to 0.38) | 0.636 |
| Childhood | Prevalence | Both | United Kingdom | AA | 0.04<br>(-0.2 to 0.27) | 0.746 |
| Childhood | Prevalence | Female | United Kingdom | AA | -0.08<br>(-0.62 to 0.46) | 0.763 |
| Childhood | Prevalence | Male | United Kingdom | AA | -0.04<br>(-0.28 to 0.21) | 0.763 |
| Childhood | Prevalence | Both | United Kingdom | IBD | -0.18<br>(-2.36 to 2.04) | 0.866 |
| Childhood | Prevalence | Female | United Kingdom | IBD | 0.28<br>(-1.96 to 2.56) | 0.805 |
| Childhood | Prevalence | Male | United Kingdom | IBD | -0.18<br>(-2.31 to 1.99) | 0.862 |
| Childhood | Prevalence | Both | United Kingdom | MS | 0.95<br>(-0.8 to 2.73) | 0.279 |

| | | | | | | |
|---|---|---|---|---|---|---|
| Childhood | Prevalence | Female | United Kingdom | MS | 1.53 (-0.16 to 3.25) | 0.074 |
| Childhood | Prevalence | Male | United Kingdom | MS | 0.78 (-1.05 to 2.65) | 0.394 |
| Childhood | Prevalence | Both | United Kingdom | Psoriasis | -0.24 (-1.68 to 1.21) | 0.734 |
| Childhood | Prevalence | Female | United Kingdom | Psoriasis | -0.63 (-2.01 to 0.77) | 0.366 |
| Childhood | Prevalence | Male | United Kingdom | Psoriasis | -0.23 (-1.73 to 1.28) | 0.754 |
| Childhood | Prevalence | Both | United Kingdom | RHD | -3.99 (-8.66 to 0.93) | 0.106 |
| Childhood | Prevalence | Female | United Kingdom | RHD | -2.89 (-7.79 to 2.26) | 0.255 |
| Childhood | Prevalence | Male | United Kingdom | RHD | -3.42 (-7.79 to 1.16) | 0.135 |
| Childhood | Prevalence | Both | United Republic of Tanzania | IBD | -0.09 (-2.12 to 1.99) | 0.931 |
| Childhood | Prevalence | Female | United Republic of Tanzania | IBD | 0.45 (-1.59 to 2.53) | 0.658 |
| Childhood | Prevalence | Male | United Republic of Tanzania | IBD | -0.03 (-2.1 to 2.08) | 0.978 |
| Childhood | Prevalence | Both | United Republic of Tanzania | RA | 0.5 (-0.7 to 1.72) | 0.403 |
| Childhood | Prevalence | Female | United Republic of Tanzania | RA | 0.75 (-0.37 to 1.89) | 0.180 |
| Childhood | Prevalence | Male | United Republic of Tanzania | RA | 0.48 (-0.81 to 1.78) | 0.456 |
| Childhood | Prevalence | Both | United States of America | T1D | -0.69 (-3.12 to 1.8) | 0.573 |
| Childhood | Prevalence | Female | United States of America | T1D | -0.11 (-2.38 to 2.22) | 0.923 |
| Childhood | Prevalence | Male | United States of America | T1D | -0.67 | 0.602 |

| | | | | | | |
|---|---|---|---|---|---|---|
| | | | | | (-3.23 to 1.96) | |
| Childhood | Prevalence | Both | United States of America | IBD | -1.34<br>(-3.01 to 0.36) | 0.116 |
| Childhood | Prevalence | Female | United States of America | IBD | -1.59<br>(-3.13 to -0.03) | 0.046 |
| Childhood | Prevalence | Male | United States of America | IBD | -1.54<br>(-3.38 to 0.34) | 0.104 |
| Childhood | Prevalence | Both | United States of America | Psoriasis | -0.86<br>(-1.54 to -0.17) | 0.017 |
| Childhood | Prevalence | Female | United States of America | Psoriasis | -0.66<br>(-1.18 to -0.13) | 0.016 |
| Childhood | Prevalence | Male | United States of America | Psoriasis | -0.91<br>(-1.76 to -0.04) | 0.040 |
| Childhood | Prevalence | Both | United States of America | RA | 0.57<br>(-0.63 to 1.79) | 0.341 |
| Childhood | Prevalence | Female | United States of America | RA | 1.04<br>(-0.22 to 2.31) | 0.104 |
| Childhood | Prevalence | Male | United States of America | RA | 0.52<br>(-0.51 to 1.56) | 0.312 |
| Childhood | Prevalence | Both | United States Virgin Islands | T1D | 1.34<br>(0.05 to 2.63) | 0.042 |
| Childhood | Prevalence | Female | United States Virgin Islands | T1D | 1.24<br>(0.05 to 2.44) | 0.041 |
| Childhood | Prevalence | Male | United States Virgin Islands | T1D | 1.14<br>(-0.25 to 2.55) | 0.106 |
| Childhood | Prevalence | Both | United States Virgin Islands | IBD | 0.1<br>(-0.25 to 0.45) | 0.566 |
| Childhood | Prevalence | Female | United States Virgin Islands | IBD | 0.44<br>(0.03 to 0.85) | 0.038 |
| Childhood | Prevalence | Male | United States Virgin Islands | IBD | 0.03<br>(-0.45 to 0.52) | 0.900 |
| Childhood | Prevalence | Both | United States Virgin Islands | MS | -0.33<br>(-5.66 to 5.29) | 0.901 |

| | | | | | | |
|---|---|---|---|---|---|---|
| Childhood | Prevalence | Female | United States Virgin Islands | MS | 0.77 (-4.86 to 6.73) | 0.787 |
| Childhood | Prevalence | Male | United States Virgin Islands | MS | -0.59 (-5.5 to 4.58) | 0.813 |
| Childhood | Prevalence | Both | United States Virgin Islands | Psoriasis | -0.61 (-1.86 to 0.66) | 0.332 |
| Childhood | Prevalence | Female | United States Virgin Islands | Psoriasis | -0.92 (-2.14 to 0.32) | 0.140 |
| Childhood | Prevalence | Male | United States Virgin Islands | Psoriasis | -0.63 (-1.9 to 0.65) | 0.318 |
| Childhood | Prevalence | Both | United States Virgin Islands | RHD | -0.93 (-3.68 to 1.9) | 0.501 |
| Childhood | Prevalence | Female | United States Virgin Islands | RHD | -0.55 (-3.45 to 2.44) | 0.705 |
| Childhood | Prevalence | Male | United States Virgin Islands | RHD | -0.29 (-2.87 to 2.36) | 0.824 |
| Childhood | Prevalence | Both | United States Virgin Islands | RA | 0.72 (-0.74 to 2.19) | 0.324 |
| Childhood | Prevalence | Female | United States Virgin Islands | RA | 1.05 (-0.43 to 2.55) | 0.157 |
| Childhood | Prevalence | Male | United States Virgin Islands | RA | 0.67 (-0.76 to 2.12) | 0.347 |
| Childhood | Prevalence | Both | Uruguay | AA | 0.05 (-0.22 to 0.32) | 0.717 |
| Childhood | Prevalence | Female | Uruguay | AA | -0.08 (-0.6 to 0.45) | 0.762 |
| Childhood | Prevalence | Male | Uruguay | AA | -0.03 (-0.27 to 0.2) | 0.762 |
| Childhood | Prevalence | Both | Uruguay | IBD | -0.01 (-1.52 to 1.52) | 0.987 |
| Childhood | Prevalence | Female | Uruguay | IBD | -0.4 (-1.78 to 1) | 0.565 |
| Childhood | Prevalence | Male | Uruguay | IBD | 0.05 | 0.949 |

| | | | | | | |
|---|---|---|---|---|---|---|
| | | | | | (-1.62 to 1.75) | |
| Childhood | Prevalence | Both | Uruguay | MS | -0.09 (-1.84 to 1.69) | 0.918 |
| Childhood | Prevalence | Female | Uruguay | MS | 0.36 (-1.36 to 2.11) | 0.675 |
| Childhood | Prevalence | Male | Uruguay | MS | -0.06 (-1.89 to 1.8) | 0.950 |
| Childhood | Prevalence | Both | Uruguay | RA | 0.61 (-0.92 to 2.17) | 0.420 |
| Childhood | Prevalence | Female | Uruguay | RA | 1.01 (-0.58 to 2.63) | 0.205 |
| Childhood | Prevalence | Male | Uruguay | RA | 0.61 (-0.83 to 2.08) | 0.395 |
| Childhood | Prevalence | Both | Uzbekistan | AA | -0.03 (-0.28 to 0.23) | 0.839 |
| Childhood | Prevalence | Female | Uzbekistan | AA | 0.04 (-0.21 to 0.29) | 0.762 |
| Childhood | Prevalence | Male | Uzbekistan | AA | -0.04 (-0.28 to 0.21) | 0.762 |
| Childhood | Prevalence | Both | Uzbekistan | T1D | 0.78 (-0.51 to 2.07) | 0.227 |
| Childhood | Prevalence | Female | Uzbekistan | T1D | 1.1 (-0.12 to 2.35) | 0.076 |
| Childhood | Prevalence | Male | Uzbekistan | T1D | 0.79 (-0.57 to 2.18) | 0.244 |
| Childhood | Prevalence | Both | Uzbekistan | IBD | 0.06 (-2.42 to 2.61) | 0.959 |
| Childhood | Prevalence | Female | Uzbekistan | IBD | -0.62 (-3.06 to 1.88) | 0.610 |
| Childhood | Prevalence | Male | Uzbekistan | IBD | -0.03 (-2.56 to 2.57) | 0.982 |
| Childhood | Prevalence | Both | Uzbekistan | MS | -0.23 (-1.96 to 1.54) | 0.790 |

| | | | | | | |
|---|---|---|---|---|---|---|
| Childhood | Prevalence | Female | Uzbekistan | MS | -0.69<br>(-2.44 to 1.09) | 0.432 |
| Childhood | Prevalence | Male | Uzbekistan | MS | -0.33<br>(-2.03 to 1.4) | 0.696 |
| Childhood | Prevalence | Both | Uzbekistan | Psoriasis | -1.51<br>(-3.03 to 0.04) | 0.055 |
| Childhood | Prevalence | Female | Uzbekistan | Psoriasis | -1.13<br>(-2.64 to 0.4) | 0.142 |
| Childhood | Prevalence | Male | Uzbekistan | Psoriasis | -1.48<br>(-3.04 to 0.11) | 0.066 |
| Childhood | Prevalence | Both | Uzbekistan | RHD | 0.33<br>(0.11 to 0.56) | 0.006 |
| Childhood | Prevalence | Female | Uzbekistan | RHD | 0.33<br>(0.06 to 0.6) | 0.017 |
| Childhood | Prevalence | Male | Uzbekistan | RHD | 0.34<br>(0.17 to 0.51) | < 0.001 |
| Childhood | Prevalence | Both | Uzbekistan | RA | 0.32<br>(-2.54 to 3.25) | 0.824 |
| Childhood | Prevalence | Female | Uzbekistan | RA | 0.66<br>(-1.97 to 3.37) | 0.614 |
| Childhood | Prevalence | Male | Uzbekistan | RA | 0.2<br>(-3.75 to 4.32) | 0.919 |
| Childhood | Prevalence | Both | Vanuatu | AA | 0.02<br>(-0.18 to 0.22) | 0.837 |
| Childhood | Prevalence | Female | Vanuatu | AA | 0.03<br>(-0.14 to 0.19) | 0.754 |
| Childhood | Prevalence | Male | Vanuatu | AA | -0.01<br>(-0.18 to 0.15) | 0.869 |
| Childhood | Prevalence | Both | Vanuatu | IBD | -0.98<br>(-3.57 to 1.67) | 0.450 |
| Childhood | Prevalence | Female | Vanuatu | IBD | -0.52<br>(-3.06 to 2.09) | 0.684 |
| Childhood | Prevalence | Male | Vanuatu | IBD | -0.86 | 0.524 |

| | | | | | | |
|---|---|---|---|---|---|---|
| | | | | | (-3.52 to 1.88) | |
| Childhood | Prevalence | Both | Vanuatu | MS | -0.13<br>(-1.72 to 1.49) | 0.871 |
| Childhood | Prevalence | Female | Vanuatu | MS | 0.27<br>(-1.37 to 1.93) | 0.742 |
| Childhood | Prevalence | Male | Vanuatu | MS | -0.11<br>(-1.68 to 1.48) | 0.887 |
| Childhood | Prevalence | Both | Vanuatu | RHD | 0.02<br>(-0.08 to 0.13) | 0.638 |
| Childhood | Prevalence | Female | Vanuatu | RHD | 0.02<br>(-0.09 to 0.14) | 0.670 |
| Childhood | Prevalence | Male | Vanuatu | RHD | 0.02<br>(-0.06 to 0.1) | 0.614 |
| Childhood | Prevalence | Both | Venezuela (Bolivarian Republic of) | T1D | 0.46<br>(-0.35 to 1.28) | 0.251 |
| Childhood | Prevalence | Female | Venezuela (Bolivarian Republic of) | T1D | 0.69<br>(0.02 to 1.37) | 0.044 |
| Childhood | Prevalence | Male | Venezuela (Bolivarian Republic of) | T1D | 0.4<br>(-0.56 to 1.38) | 0.401 |
| Childhood | Prevalence | Both | Venezuela (Bolivarian Republic of) | IBD | -0.08<br>(-2.7 to 2.6) | 0.949 |
| Childhood | Prevalence | Female | Venezuela (Bolivarian Republic of) | IBD | -0.93<br>(-3.54 to 1.75) | 0.479 |
| Childhood | Prevalence | Male | Venezuela (Bolivarian Republic of) | IBD | 0.08<br>(-2.53 to 2.76) | 0.951 |
| Childhood | Prevalence | Both | Venezuela (Bolivarian Republic of) | Psoriasis | -0.7<br>(-2.28 to 0.91) | 0.379 |
| Childhood | Prevalence | Female | Venezuela (Bolivarian Republic of) | Psoriasis | -0.34<br>(-1.91 to 1.26) | 0.667 |
| Childhood | Prevalence | Male | Venezuela (Bolivarian Republic of) | Psoriasis | -0.65<br>(-2.24 to 0.97) | 0.416 |
| Childhood | Prevalence | Both | Venezuela (Bolivarian Republic of) | RHD | -1.6<br>(-4.13 to 1) | 0.216 |

| | | | | | | |
|---|---|---|---|---|---|---|
| Childhood | Prevalence | Female | Venezuela (Bolivarian Republic of) | RHD | -2.4<br>(-4.84 to 0.1) | 0.059 |
| Childhood | Prevalence | Male | Venezuela (Bolivarian Republic of) | RHD | -1.26<br>(-3.86 to 1.42) | 0.340 |
| Childhood | Prevalence | Both | Viet Nam | AA | -0.04<br>(-0.27 to 0.19) | 0.697 |
| Childhood | Prevalence | Female | Viet Nam | AA | 0.06<br>(-0.15 to 0.27) | 0.544 |
| Childhood | Prevalence | Male | Viet Nam | AA | 0.01<br>(-0.2 to 0.21) | 0.959 |
| Childhood | Prevalence | Both | Viet Nam | T1D | 0.52<br>(-0.41 to 1.46) | 0.265 |
| Childhood | Prevalence | Female | Viet Nam | T1D | 0.71<br>(-0.19 to 1.62) | 0.117 |
| Childhood | Prevalence | Male | Viet Nam | T1D | 0.56<br>(-0.42 to 1.54) | 0.256 |
| Childhood | Prevalence | Both | Viet Nam | IBD | 1.35<br>(-3.3 to 6.22) | 0.563 |
| Childhood | Prevalence | Female | Viet Nam | IBD | 2.6<br>(-2.04 to 7.46) | 0.266 |
| Childhood | Prevalence | Male | Viet Nam | IBD | 1.37<br>(-3.23 to 6.18) | 0.554 |
| Childhood | Prevalence | Both | Viet Nam | MS | 0.75<br>(-0.09 to 1.61) | 0.079 |
| Childhood | Prevalence | Female | Viet Nam | MS | 0.69<br>(-0.18 to 1.56) | 0.115 |
| Childhood | Prevalence | Male | Viet Nam | MS | 0.88<br>(0.06 to 1.7) | 0.036 |
| Childhood | Prevalence | Both | Viet Nam | Psoriasis | -0.62<br>(-2.07 to 0.85) | 0.391 |
| Childhood | Prevalence | Female | Viet Nam | Psoriasis | -0.92<br>(-2.08 to 0.25) | 0.119 |
| Childhood | Prevalence | Male | Viet Nam | Psoriasis | -0.69 | 0.431 |

| | | | | | | |
|---|---|---|---|---|---|---|
| | | | | | (-2.44 to 1.09) | |
| Childhood | Prevalence | Both | Viet Nam | RHD | -0.36<br>(-4.7 to 4.18) | 0.869 |
| Childhood | Prevalence | Female | Viet Nam | RHD | 0.23<br>(-3.86 to 4.49) | 0.912 |
| Childhood | Prevalence | Male | Viet Nam | RHD | 0.46<br>(-4.21 to 5.35) | 0.846 |
| Childhood | Prevalence | Both | Yemen | AA | -0.01<br>(-0.26 to 0.24) | 0.934 |
| Childhood | Prevalence | Female | Yemen | AA | 0.04<br>(-0.21 to 0.29) | 0.762 |
| Childhood | Prevalence | Male | Yemen | AA | -0.03<br>(-0.26 to 0.19) | 0.762 |
| Childhood | Prevalence | Both | Yemen | IBD | 0.01<br>(-8.47 to 9.28) | 0.998 |
| Childhood | Prevalence | Female | Yemen | IBD | -2.14<br>(-10.49 to 6.99) | 0.624 |
| Childhood | Prevalence | Male | Yemen | IBD | 0.16<br>(-8.24 to 9.33) | 0.970 |
| Childhood | Prevalence | Both | Yemen | Psoriasis | -1.15<br>(-1.96 to -0.34) | 0.007 |
| Childhood | Prevalence | Female | Yemen | Psoriasis | -1.35<br>(-2.11 to -0.59) | 0.001 |
| Childhood | Prevalence | Male | Yemen | Psoriasis | -1.16<br>(-2.02 to -0.3) | 0.010 |
| Childhood | Prevalence | Both | Yemen | RHD | 0.17<br>(-0.16 to 0.49) | 0.300 |
| Childhood | Prevalence | Female | Yemen | RHD | 0.28<br>(-0.08 to 0.64) | 0.120 |
| Childhood | Prevalence | Male | Yemen | RHD | 0.15<br>(-0.14 to 0.45) | 0.289 |
| Childhood | Prevalence | Both | Yemen | RA | 0.51<br>(-1 to 2.03) | 0.497 |

| | | | | | | |
|---|---|---|---|---|---|---|
| Childhood | Prevalence | Female | Yemen | RA | 0.9<br>(-0.62 to 2.44) | 0.237 |
| Childhood | Prevalence | Male | Yemen | RA | 0.45<br>(-0.98 to 1.9) | 0.527 |
| Childhood | Prevalence | Both | Zambia | AA | -0.02<br>(-0.25 to 0.22) | 0.879 |
| Childhood | Prevalence | Female | Zambia | AA | -0.04<br>(-0.3 to 0.22) | 0.762 |
| Childhood | Prevalence | Male | Zambia | AA | 0.03<br>(-0.2 to 0.27) | 0.763 |
| Childhood | Prevalence | Both | Zambia | MS | 0.37<br>(-1.37 to 2.14) | 0.670 |
| Childhood | Prevalence | Female | Zambia | MS | -0.08<br>(-1.83 to 1.69) | 0.923 |
| Childhood | Prevalence | Male | Zambia | MS | 0.44<br>(-1.34 to 2.24) | 0.619 |
| Childhood | Prevalence | Both | Zambia | RHD | -0.04<br>(-0.19 to 0.1) | 0.569 |
| Childhood | Prevalence | Female | Zambia | RHD | 0.03<br>(-0.12 to 0.17) | 0.681 |
| Childhood | Prevalence | Male | Zambia | RHD | -0.04<br>(-0.19 to 0.1) | 0.567 |
| Childhood | Prevalence | Both | Zambia | RA | 0.38<br>(-4.13 to 5.1) | 0.867 |
| Childhood | Prevalence | Female | Zambia | RA | 1.2<br>(-3.5 to 6.12) | 0.612 |
| Childhood | Prevalence | Male | Zambia | RA | 0.35<br>(-4.35 to 5.29) | 0.882 |
| Childhood | Prevalence | Both | Zimbabwe | AA | -0.01<br>(-0.26 to 0.25) | 0.944 |
| Childhood | Prevalence | Female | Zimbabwe | AA | 0.04<br>(-0.21 to 0.28) | 0.762 |
| Childhood | Prevalence | Male | Zimbabwe | AA | -0.04 | 0.762 |

| | | | | | | |
|---|---|---|---|---|---|---|
| | | | | | (-0.27 to 0.2) | |
| Childhood | Prevalence | Both | Zimbabwe | Psoriasis | -0.11<br>(-0.83 to 0.61) | 0.750 |
| Childhood | Prevalence | Female | Zimbabwe | Psoriasis | 0.03<br>(-0.69 to 0.74) | 0.942 |
| Childhood | Prevalence | Male | Zimbabwe | Psoriasis | -0.07<br>(-0.79 to 0.65) | 0.845 |
| Childhood | Prevalence | Both | Zimbabwe | RA | -0.04<br>(-2.55 to 2.53) | 0.974 |
| Childhood | Prevalence | Female | Zimbabwe | RA | -0.65<br>(-3.01 to 1.76) | 0.581 |
| Childhood | Prevalence | Male | Zimbabwe | RA | -0.03<br>(-2.76 to 2.77) | 0.983 |
| Adolescence | Prevalence | Both | Afghanistan | AA | -0.02<br>(-0.04 to 0) | 0.055 |
| Adolescence | Prevalence | Female | Afghanistan | AA | 0<br>(0 to 0) | 0.001 |
| Adolescence | Prevalence | Male | Afghanistan | AA | 0<br>(0 to 0) | 0.011 |
| Adolescence | Prevalence | Both | Afghanistan | T1D | 2.2<br>(2.13 to 2.26) | < 0.001 |
| Adolescence | Prevalence | Female | Afghanistan | T1D | 2.18<br>(2.12 to 2.24) | < 0.001 |
| Adolescence | Prevalence | Male | Afghanistan | T1D | 2.22<br>(2.16 to 2.27) | < 0.001 |
| Adolescence | Prevalence | Both | Afghanistan | IBD | 0.22<br>(0.18 to 0.25) | < 0.001 |
| Adolescence | Prevalence | Female | Afghanistan | IBD | 0.16<br>(0.12 to 0.21) | < 0.001 |
| Adolescence | Prevalence | Male | Afghanistan | IBD | 0.27<br>(0.22 to 0.32) | < 0.001 |
| Adolescence | Prevalence | Both | Afghanistan | MS | 0.43<br>(0.36 to 0.49) | < 0.001 |

| | | | | | | |
|---|---|---|---|---|---|---|
| Adolescence | Prevalence | Female | Afghanistan | MS | 0.46 (0.4 to 0.52) | < 0.001 |
| Adolescence | Prevalence | Male | Afghanistan | MS | 0.49 (0.46 to 0.51) | < 0.001 |
| Adolescence | Prevalence | Both | Afghanistan | Psoriasis | -0.94 (-0.97 to -0.91) | < 0.001 |
| Adolescence | Prevalence | Female | Afghanistan | Psoriasis | -0.89 (-0.92 to -0.86) | < 0.001 |
| Adolescence | Prevalence | Male | Afghanistan | Psoriasis | -0.96 (-0.98 to -0.95) | < 0.001 |
| Adolescence | Prevalence | Both | Afghanistan | RHD | 0.1 (0.09 to 0.12) | < 0.001 |
| Adolescence | Prevalence | Female | Afghanistan | RHD | 0.12 (0.1 to 0.13) | < 0.001 |
| Adolescence | Prevalence | Male | Afghanistan | RHD | 0.11 (0.06 to 0.15) | < 0.001 |
| Adolescence | Prevalence | Both | Afghanistan | RA | 0.28 (0.26 to 0.3) | < 0.001 |
| Adolescence | Prevalence | Female | Afghanistan | RA | 0.28 (0.25 to 0.32) | < 0.001 |
| Adolescence | Prevalence | Male | Afghanistan | RA | 0.39 (0.32 to 0.47) | < 0.001 |
| Adolescence | Prevalence | Both | Albania | AA | -0.03 (-0.04 to -0.01) | 0.001 |
| Adolescence | Prevalence | Female | Albania | AA | 0 (0 to 0) | 1.000 |
| Adolescence | Prevalence | Male | Albania | AA | 0 (0 to 0) | 1.000 |
| Adolescence | Prevalence | Both | Albania | T1D | 1.37 (1.3 to 1.43) | < 0.001 |
| Adolescence | Prevalence | Female | Albania | T1D | 1.38 (1.32 to 1.44) | < 0.001 |
| Adolescence | Prevalence | Male | Albania | T1D | 1.36 | < 0.001 |

| | | | | | | |
|---|---|---|---|---|---|---|
| | | | | | (1.28 to 1.44) | |
| Adolescence | Prevalence | Both | Albania | IBD | 0.16 (0.13 to 0.19) | < 0.001 |
| Adolescence | Prevalence | Female | Albania | IBD | 0.16 (0.14 to 0.18) | < 0.001 |
| Adolescence | Prevalence | Male | Albania | IBD | 0.18 (0.15 to 0.21) | < 0.001 |
| Adolescence | Prevalence | Both | Albania | MS | 0.49 (0.44 to 0.55) | < 0.001 |
| Adolescence | Prevalence | Female | Albania | MS | 0.52 (0.46 to 0.59) | < 0.001 |
| Adolescence | Prevalence | Male | Albania | MS | 0.44 (0.35 to 0.52) | < 0.001 |
| Adolescence | Prevalence | Both | Albania | Psoriasis | -0.92 (-0.95 to -0.9) | < 0.001 |
| Adolescence | Prevalence | Female | Albania | Psoriasis | -0.92 (-0.96 to -0.88) | < 0.001 |
| Adolescence | Prevalence | Male | Albania | Psoriasis | -0.89 (-0.92 to -0.86) | < 0.001 |
| Adolescence | Prevalence | Both | Albania | RHD | -0.05 (-0.06 to -0.04) | < 0.001 |
| Adolescence | Prevalence | Female | Albania | RHD | 0.01 (-0.01 to 0.03) | 0.214 |
| Adolescence | Prevalence | Male | Albania | RHD | -0.1 (-0.11 to -0.1) | < 0.001 |
| Adolescence | Prevalence | Both | Albania | RA | 0.69 (0.66 to 0.73) | < 0.001 |
| Adolescence | Prevalence | Female | Albania | RA | 0.74 (0.7 to 0.78) | < 0.001 |
| Adolescence | Prevalence | Male | Albania | RA | 0.72 (0.65 to 0.79) | < 0.001 |
| Adolescence | Prevalence | Both | Algeria | AA | 0 (0 to 0) | 0.013 |

| | | | | | | |
|---|---|---|---|---|---|---|
| Adolescence | Prevalence | Female | Algeria | AA | 0<br>(0 to 0) | 0.001 |
| Adolescence | Prevalence | Male | Algeria | AA | 0<br>(0 to 0) | 0.011 |
| Adolescence | Prevalence | Both | Algeria | T1D | 2.18<br>(2.16 to 2.21) | < 0.001 |
| Adolescence | Prevalence | Female | Algeria | T1D | 2.17<br>(2.15 to 2.18) | < 0.001 |
| Adolescence | Prevalence | Male | Algeria | T1D | 2.2<br>(2.16 to 2.24) | < 0.001 |
| Adolescence | Prevalence | Both | Algeria | IBD | -0.05<br>(-0.13 to 0.02) | 0.154 |
| Adolescence | Prevalence | Female | Algeria | IBD | -0.01<br>(-0.11 to 0.09) | 0.827 |
| Adolescence | Prevalence | Male | Algeria | IBD | -0.11<br>(-0.2 to -0.02) | 0.022 |
| Adolescence | Prevalence | Both | Algeria | MS | 0.46<br>(0.39 to 0.54) | < 0.001 |
| Adolescence | Prevalence | Female | Algeria | MS | 0.45<br>(0.36 to 0.53) | < 0.001 |
| Adolescence | Prevalence | Male | Algeria | MS | 0.48<br>(0.44 to 0.52) | < 0.001 |
| Adolescence | Prevalence | Both | Algeria | Psoriasis | -1.1<br>(-1.12 to -1.08) | < 0.001 |
| Adolescence | Prevalence | Female | Algeria | Psoriasis | -1.07<br>(-1.08 to -1.05) | < 0.001 |
| Adolescence | Prevalence | Male | Algeria | Psoriasis | -1.15<br>(-1.18 to -1.12) | < 0.001 |
| Adolescence | Prevalence | Both | Algeria | RHD | 0.88<br>(0.74 to 1.01) | < 0.001 |
| Adolescence | Prevalence | Female | Algeria | RHD | 0.91<br>(0.7 to 1.12) | < 0.001 |
| Adolescence | Prevalence | Male | Algeria | RHD | 0.84 | < 0.001 |

| | | | | | | |
|---|---|---|---|---|---|---|
| | | | | | (0.76 to 0.93) | |
| Adolescence | Prevalence | Both | Algeria | RA | 0.69<br>(0.68 to 0.7) | < 0.001 |
| Adolescence | Prevalence | Female | Algeria | RA | 0.69<br>(0.67 to 0.71) | < 0.001 |
| Adolescence | Prevalence | Male | Algeria | RA | 0.71<br>(0.65 to 0.77) | < 0.001 |
| Adolescence | Prevalence | Both | American Samoa | AA | 0.01<br>(0.01 to 0.02) | < 0.001 |
| Adolescence | Prevalence | Female | American Samoa | AA | 0<br>(0 to 0) | 0.008 |
| Adolescence | Prevalence | Male | American Samoa | AA | 0<br>(0 to 0) | 1.000 |
| Adolescence | Prevalence | Both | American Samoa | T1D | 0.64<br>(0.62 to 0.66) | < 0.001 |
| Adolescence | Prevalence | Female | American Samoa | T1D | 0.6<br>(0.59 to 0.62) | < 0.001 |
| Adolescence | Prevalence | Male | American Samoa | T1D | 0.68<br>(0.65 to 0.71) | < 0.001 |
| Adolescence | Prevalence | Both | American Samoa | IBD | 0.2<br>(0.09 to 0.31) | 0.001 |
| Adolescence | Prevalence | Female | American Samoa | IBD | 0.22<br>(0.08 to 0.36) | 0.003 |
| Adolescence | Prevalence | Male | American Samoa | IBD | 0.15<br>(0.07 to 0.22) | < 0.001 |
| Adolescence | Prevalence | Both | American Samoa | MS | 0.02<br>(-0.16 to 0.2) | 0.809 |
| Adolescence | Prevalence | Female | American Samoa | MS | -0.08<br>(-0.16 to 0.01) | 0.082 |
| Adolescence | Prevalence | Male | American Samoa | MS | 0.08<br>(-0.19 to 0.36) | 0.554 |
| Adolescence | Prevalence | Both | American Samoa | Psoriasis | -0.39<br>(-0.5 to -0.28) | < 0.001 |

| | | | | | | |
|---|---|---|---|---|---|---|
| Adolescence | Prevalence | Female | American Samoa | Psoriasis | -0.38 (-0.47 to -0.3) | < 0.001 |
| Adolescence | Prevalence | Male | American Samoa | Psoriasis | -0.41 (-0.55 to -0.27) | < 0.001 |
| Adolescence | Prevalence | Both | American Samoa | RHD | 0.11 (0.1 to 0.12) | < 0.001 |
| Adolescence | Prevalence | Female | American Samoa | RHD | 0.1 (0.09 to 0.1) | < 0.001 |
| Adolescence | Prevalence | Male | American Samoa | RHD | 0.13 (0.11 to 0.15) | < 0.001 |
| Adolescence | Prevalence | Both | American Samoa | RA | 0.31 (0.27 to 0.35) | < 0.001 |
| Adolescence | Prevalence | Female | American Samoa | RA | 0.33 (0.28 to 0.37) | < 0.001 |
| Adolescence | Prevalence | Male | American Samoa | RA | 0.25 (0.18 to 0.32) | < 0.001 |
| Adolescence | Prevalence | Both | Andorra | AA | 0 (0 to 0) | 0.547 |
| Adolescence | Prevalence | Female | Andorra | AA | 0 (0 to 0) | 1.000 |
| Adolescence | Prevalence | Male | Andorra | AA | 0 (0 to 0) | 0.005 |
| Adolescence | Prevalence | Both | Andorra | T1D | 1.7 (1.63 to 1.78) | < 0.001 |
| Adolescence | Prevalence | Female | Andorra | T1D | 1.71 (1.65 to 1.76) | < 0.001 |
| Adolescence | Prevalence | Male | Andorra | T1D | 1.72 (1.67 to 1.76) | < 0.001 |
| Adolescence | Prevalence | Both | Andorra | IBD | -0.15 (-0.17 to -0.13) | < 0.001 |
| Adolescence | Prevalence | Female | Andorra | IBD | -0.39 (-0.43 to -0.36) | < 0.001 |
| Adolescence | Prevalence | Male | Andorra | IBD | 0.09 | < 0.001 |

| | | | | | | |
|---|---|---|---|---|---|---|
| | | | | | (0.05 to 0.14) | |
| Adolescence | Prevalence | Both | Andorra | MS | 0.21<br>(0.17 to 0.25) | < 0.001 |
| Adolescence | Prevalence | Female | Andorra | MS | 0.23<br>(0.16 to 0.29) | < 0.001 |
| Adolescence | Prevalence | Male | Andorra | MS | 0.17<br>(0.14 to 0.2) | < 0.001 |
| Adolescence | Prevalence | Both | Andorra | Psoriasis | -0.23<br>(-0.24 to -0.23) | < 0.001 |
| Adolescence | Prevalence | Female | Andorra | Psoriasis | -0.23<br>(-0.24 to -0.22) | < 0.001 |
| Adolescence | Prevalence | Male | Andorra | Psoriasis | -0.24<br>(-0.24 to -0.24) | < 0.001 |
| Adolescence | Prevalence | Both | Andorra | RHD | -0.15<br>(-0.18 to -0.12) | < 0.001 |
| Adolescence | Prevalence | Female | Andorra | RHD | -0.15<br>(-0.18 to -0.13) | < 0.001 |
| Adolescence | Prevalence | Male | Andorra | RHD | -0.12<br>(-0.19 to -0.05) | 0.001 |
| Adolescence | Prevalence | Both | Andorra | RA | 0.42<br>(0.38 to 0.45) | < 0.001 |
| Adolescence | Prevalence | Female | Andorra | RA | 0.38<br>(0.34 to 0.42) | < 0.001 |
| Adolescence | Prevalence | Male | Andorra | RA | 0.45<br>(0.42 to 0.47) | < 0.001 |
| Adolescence | Prevalence | Both | Angola | AA | 0.02<br>(0.02 to 0.03) | < 0.001 |
| Adolescence | Prevalence | Female | Angola | AA | 0<br>(0 to 0) | 1.000 |
| Adolescence | Prevalence | Male | Angola | AA | 0<br>(0 to 0) | 1.000 |
| Adolescence | Prevalence | Both | Angola | T1D | 0.05<br>(0.02 to 0.08) | 0.001 |

| | | | | | | |
|---|---|---|---|---|---|---|
| Adolescence | Prevalence | Female | Angola | T1D | 0.08 (0.06 to 0.1) | < 0.001 |
| Adolescence | Prevalence | Male | Angola | T1D | 0.01 (-0.01 to 0.04) | 0.345 |
| Adolescence | Prevalence | Both | Angola | IBD | 0.53 (0.48 to 0.57) | < 0.001 |
| Adolescence | Prevalence | Female | Angola | IBD | 0.52 (0.43 to 0.62) | < 0.001 |
| Adolescence | Prevalence | Male | Angola | IBD | 0.53 (0.47 to 0.59) | < 0.001 |
| Adolescence | Prevalence | Both | Angola | MS | 0.03 (-0.12 to 0.19) | 0.674 |
| Adolescence | Prevalence | Female | Angola | MS | 0.02 (-0.06 to 0.1) | 0.602 |
| Adolescence | Prevalence | Male | Angola | MS | 0.03 (-0.14 to 0.2) | 0.719 |
| Adolescence | Prevalence | Both | Angola | Psoriasis | -1.04 (-1.08 to -1) | < 0.001 |
| Adolescence | Prevalence | Female | Angola | Psoriasis | -1.06 (-1.1 to -1.01) | < 0.001 |
| Adolescence | Prevalence | Male | Angola | Psoriasis | -1.03 (-1.13 to -0.93) | < 0.001 |
| Adolescence | Prevalence | Both | Angola | RHD | 0.09 (0.08 to 0.11) | < 0.001 |
| Adolescence | Prevalence | Female | Angola | RHD | 0.16 (0.12 to 0.19) | < 0.001 |
| Adolescence | Prevalence | Male | Angola | RHD | 0.03 (-0.01 to 0.07) | 0.173 |
| Adolescence | Prevalence | Both | Angola | RA | 0.31 (0.22 to 0.4) | < 0.001 |
| Adolescence | Prevalence | Female | Angola | RA | 0.23 (0.16 to 0.31) | < 0.001 |
| Adolescence | Prevalence | Male | Angola | RA | 0.31 | < 0.001 |

| | | | | | | |
|---|---|---|---|---|---|---|
| | | | | | (0.24 to 0.37) | |
| Adolescence | Prevalence | Both | Antigua and Barbuda | AA | -0.02<br>(-0.02 to -0.01) | < 0.001 |
| Adolescence | Prevalence | Female | Antigua and Barbuda | AA | 0<br>(0 to 0) | 1.000 |
| Adolescence | Prevalence | Male | Antigua and Barbuda | AA | 0<br>(0 to 0) | 1.000 |
| Adolescence | Prevalence | Both | Antigua and Barbuda | T1D | 1.08<br>(1.06 to 1.09) | < 0.001 |
| Adolescence | Prevalence | Female | Antigua and Barbuda | T1D | 1.01<br>(0.99 to 1.03) | < 0.001 |
| Adolescence | Prevalence | Male | Antigua and Barbuda | T1D | 1.16<br>(1.11 to 1.21) | < 0.001 |
| Adolescence | Prevalence | Both | Antigua and Barbuda | IBD | 0.03<br>(-0.02 to 0.08) | 0.218 |
| Adolescence | Prevalence | Female | Antigua and Barbuda | IBD | 0.07<br>(-0.02 to 0.16) | 0.115 |
| Adolescence | Prevalence | Male | Antigua and Barbuda | IBD | 0.01<br>(-0.03 to 0.05) | 0.558 |
| Adolescence | Prevalence | Both | Antigua and Barbuda | MS | 0.21<br>(-0.01 to 0.42) | 0.060 |
| Adolescence | Prevalence | Female | Antigua and Barbuda | MS | 0.24<br>(0.12 to 0.37) | < 0.001 |
| Adolescence | Prevalence | Male | Antigua and Barbuda | MS | 0.16<br>(0.04 to 0.29) | 0.010 |
| Adolescence | Prevalence | Both | Antigua and Barbuda | Psoriasis | -0.39<br>(-0.4 to -0.38) | < 0.001 |
| Adolescence | Prevalence | Female | Antigua and Barbuda | Psoriasis | -0.39<br>(-0.4 to -0.38) | < 0.001 |
| Adolescence | Prevalence | Male | Antigua and Barbuda | Psoriasis | -0.39<br>(-0.4 to -0.38) | < 0.001 |
| Adolescence | Prevalence | Both | Antigua and Barbuda | RHD | -0.03<br>(-0.06 to 0) | 0.074 |

| | | | | | | |
|---|---|---|---|---|---|---|
| Adolescence | Prevalence | Female | Antigua and Barbuda | RHD | 0.01 (-0.03 to 0.05) | 0.757 |
| Adolescence | Prevalence | Male | Antigua and Barbuda | RHD | -0.06 (-0.09 to -0.02) | 0.002 |
| Adolescence | Prevalence | Both | Antigua and Barbuda | RA | 0.66 (0.64 to 0.67) | < 0.001 |
| Adolescence | Prevalence | Female | Antigua and Barbuda | RA | 0.67 (0.65 to 0.68) | < 0.001 |
| Adolescence | Prevalence | Male | Antigua and Barbuda | RA | 0.7 (0.69 to 0.72) | < 0.001 |
| Adolescence | Prevalence | Both | Argentina | AA | -0.01 (-0.01 to -0.01) | < 0.001 |
| Adolescence | Prevalence | Female | Argentina | AA | 0 (0 to 0) | 1.000 |
| Adolescence | Prevalence | Male | Argentina | AA | 0 (0 to 0) | 1.000 |
| Adolescence | Prevalence | Both | Argentina | T1D | 0.65 (0.61 to 0.68) | < 0.001 |
| Adolescence | Prevalence | Female | Argentina | T1D | 0.69 (0.65 to 0.73) | < 0.001 |
| Adolescence | Prevalence | Male | Argentina | T1D | 0.61 (0.56 to 0.65) | < 0.001 |
| Adolescence | Prevalence | Both | Argentina | IBD | 0.11 (0.06 to 0.16) | < 0.001 |
| Adolescence | Prevalence | Female | Argentina | IBD | 0.12 (0.05 to 0.18) | 0.001 |
| Adolescence | Prevalence | Male | Argentina | IBD | 0.13 (0.09 to 0.16) | < 0.001 |
| Adolescence | Prevalence | Both | Argentina | MS | 0.01 (-0.09 to 0.11) | 0.855 |
| Adolescence | Prevalence | Female | Argentina | MS | 0.02 (-0.12 to 0.15) | 0.821 |
| Adolescence | Prevalence | Male | Argentina | MS | 0.09 | 0.044 |

| | | | | | | |
|---|---|---|---|---|---|---|
| | | | | | (0 to 0.17) | |
| Adolescence | Prevalence | Both | Argentina | Psoriasis | -0.43<br>(-0.44 to -0.42) | < 0.001 |
| Adolescence | Prevalence | Female | Argentina | Psoriasis | -0.45<br>(-0.46 to -0.44) | < 0.001 |
| Adolescence | Prevalence | Male | Argentina | Psoriasis | -0.4<br>(-0.42 to -0.38) | < 0.001 |
| Adolescence | Prevalence | Both | Argentina | RHD | 0.02<br>(-0.04 to 0.07) | 0.542 |
| Adolescence | Prevalence | Female | Argentina | RHD | 0.02<br>(-0.01 to 0.06) | 0.226 |
| Adolescence | Prevalence | Male | Argentina | RHD | 0.02<br>(0 to 0.05) | 0.082 |
| Adolescence | Prevalence | Both | Argentina | RA | 0.65<br>(0.64 to 0.67) | < 0.001 |
| Adolescence | Prevalence | Female | Argentina | RA | 0.66<br>(0.62 to 0.71) | < 0.001 |
| Adolescence | Prevalence | Male | Argentina | RA | 0.67<br>(0.64 to 0.7) | < 0.001 |
| Adolescence | Prevalence | Both | Armenia | AA | -0.04<br>(-0.04 to -0.04) | < 0.001 |
| Adolescence | Prevalence | Female | Armenia | AA | 0<br>(0 to 0) | 1.000 |
| Adolescence | Prevalence | Male | Armenia | AA | 0<br>(0 to 0) | 1.000 |
| Adolescence | Prevalence | Both | Armenia | T1D | 1.19<br>(1.17 to 1.21) | < 0.001 |
| Adolescence | Prevalence | Female | Armenia | T1D | 1.18<br>(1.16 to 1.2) | < 0.001 |
| Adolescence | Prevalence | Male | Armenia | T1D | 1.2<br>(1.15 to 1.25) | < 0.001 |
| Adolescence | Prevalence | Both | Armenia | IBD | 0.23<br>(0.2 to 0.26) | < 0.001 |

| | | | | | | |
|---|---|---|---|---|---|---|
| Adolescence | Prevalence | Female | Armenia | IBD | 0.25 (0.2 to 0.29) | < 0.001 |
| Adolescence | Prevalence | Male | Armenia | IBD | 0.25 (0.22 to 0.27) | < 0.001 |
| Adolescence | Prevalence | Both | Armenia | MS | 0.32 (0.26 to 0.38) | < 0.001 |
| Adolescence | Prevalence | Female | Armenia | MS | 0.32 (0.28 to 0.36) | < 0.001 |
| Adolescence | Prevalence | Male | Armenia | MS | 0.37 (0.27 to 0.47) | < 0.001 |
| Adolescence | Prevalence | Both | Armenia | Psoriasis | -0.94 (-0.97 to -0.92) | < 0.001 |
| Adolescence | Prevalence | Female | Armenia | Psoriasis | -0.94 (-0.98 to -0.9) | < 0.001 |
| Adolescence | Prevalence | Male | Armenia | Psoriasis | -0.91 (-0.93 to -0.88) | < 0.001 |
| Adolescence | Prevalence | Both | Armenia | RHD | -0.18 (-0.22 to -0.14) | < 0.001 |
| Adolescence | Prevalence | Female | Armenia | RHD | -0.17 (-0.21 to -0.12) | < 0.001 |
| Adolescence | Prevalence | Male | Armenia | RHD | -0.19 (-0.23 to -0.15) | < 0.001 |
| Adolescence | Prevalence | Both | Armenia | RA | 0.66 (0.57 to 0.75) | < 0.001 |
| Adolescence | Prevalence | Female | Armenia | RA | 0.71 (0.62 to 0.8) | < 0.001 |
| Adolescence | Prevalence | Male | Armenia | RA | 0.72 (0.56 to 0.88) | < 0.001 |
| Adolescence | Prevalence | Both | Australia | AA | 0 (0 to 0) | 0.010 |
| Adolescence | Prevalence | Female | Australia | AA | 0 (0 to 0) | 1.000 |
| Adolescence | Prevalence | Male | Australia | AA | 0 | 1.000 |

| | | | | | | |
|---|---|---|---|---|---|---|
| | | | | | (0 to 0) | |
| Adolescence | Prevalence | Both | Australia | T1D | 1.49<br>(1.4 to 1.58) | < 0.001 |
| Adolescence | Prevalence | Female | Australia | T1D | 1.36<br>(1.3 to 1.42) | < 0.001 |
| Adolescence | Prevalence | Male | Australia | T1D | 1.67<br>(1.57 to 1.76) | < 0.001 |
| Adolescence | Prevalence | Both | Australia | IBD | 2.04<br>(1.96 to 2.12) | < 0.001 |
| Adolescence | Prevalence | Female | Australia | IBD | 1.95<br>(1.9 to 2.01) | < 0.001 |
| Adolescence | Prevalence | Male | Australia | IBD | 2.06<br>(1.9 to 2.23) | < 0.001 |
| Adolescence | Prevalence | Both | Australia | MS | 0.38<br>(0.28 to 0.47) | < 0.001 |
| Adolescence | Prevalence | Female | Australia | MS | 0.45<br>(0.31 to 0.6) | < 0.001 |
| Adolescence | Prevalence | Male | Australia | MS | 0.25<br>(0.17 to 0.33) | < 0.001 |
| Adolescence | Prevalence | Both | Australia | Psoriasis | -0.46<br>(-0.56 to -0.37) | < 0.001 |
| Adolescence | Prevalence | Female | Australia | Psoriasis | -0.43<br>(-0.51 to -0.35) | < 0.001 |
| Adolescence | Prevalence | Male | Australia | Psoriasis | -0.46<br>(-0.53 to -0.39) | < 0.001 |
| Adolescence | Prevalence | Both | Australia | RHD | -0.1<br>(-0.15 to -0.04) | 0.001 |
| Adolescence | Prevalence | Female | Australia | RHD | -0.08<br>(-0.17 to 0) | 0.059 |
| Adolescence | Prevalence | Male | Australia | RHD | -0.15<br>(-0.25 to -0.06) | 0.001 |
| Adolescence | Prevalence | Both | Australia | RA | 0.71<br>(0.69 to 0.74) | < 0.001 |

| | | | | | | |
|---|---|---|---|---|---|---|
| Adolescence | Prevalence | Female | Australia | RA | 0.69 (0.67 to 0.72) | < 0.001 |
| Adolescence | Prevalence | Male | Australia | RA | 0.79 (0.75 to 0.83) | < 0.001 |
| Adolescence | Prevalence | Both | Austria | AA | 0 (0 to 0) | 1.000 |
| Adolescence | Prevalence | Female | Austria | AA | 0 (0 to 0) | 1.000 |
| Adolescence | Prevalence | Male | Austria | AA | 0 (0 to 0) | 0.005 |
| Adolescence | Prevalence | Both | Austria | T1D | 2.14 (2.05 to 2.24) | < 0.001 |
| Adolescence | Prevalence | Female | Austria | T1D | 2.03 (1.92 to 2.13) | < 0.001 |
| Adolescence | Prevalence | Male | Austria | T1D | 2.25 (2.22 to 2.29) | < 0.001 |
| Adolescence | Prevalence | Both | Austria | IBD | -0.4 (-0.63 to -0.18) | < 0.001 |
| Adolescence | Prevalence | Female | Austria | IBD | -0.63 (-0.88 to -0.37) | < 0.001 |
| Adolescence | Prevalence | Male | Austria | IBD | -0.26 (-0.51 to -0.01) | 0.041 |
| Adolescence | Prevalence | Both | Austria | MS | 0.26 (0.23 to 0.29) | < 0.001 |
| Adolescence | Prevalence | Female | Austria | MS | 0.32 (0.27 to 0.37) | < 0.001 |
| Adolescence | Prevalence | Male | Austria | MS | 0.23 (0.2 to 0.27) | < 0.001 |
| Adolescence | Prevalence | Both | Austria | Psoriasis | -0.37 (-0.38 to -0.37) | < 0.001 |
| Adolescence | Prevalence | Female | Austria | Psoriasis | -0.37 (-0.38 to -0.36) | < 0.001 |
| Adolescence | Prevalence | Male | Austria | Psoriasis | -0.37 | < 0.001 |

| | | | | | | |
|---|---|---|---|---|---|---|
| | | | | | (-0.37 to -0.36) | |
| Adolescence | Prevalence | Both | Austria | RHD | -2.34 (-2.57 to -2.11) | < 0.001 |
| Adolescence | Prevalence | Female | Austria | RHD | -2.58 (-2.89 to -2.27) | < 0.001 |
| Adolescence | Prevalence | Male | Austria | RHD | -2.05 (-2.28 to -1.81) | < 0.001 |
| Adolescence | Prevalence | Both | Austria | RA | 0.39 (0.36 to 0.42) | < 0.001 |
| Adolescence | Prevalence | Female | Austria | RA | 0.38 (0.36 to 0.41) | < 0.001 |
| Adolescence | Prevalence | Male | Austria | RA | 0.49 (0.47 to 0.52) | < 0.001 |
| Adolescence | Prevalence | Both | Azerbaijan | AA | -0.03 (-0.04 to -0.02) | < 0.001 |
| Adolescence | Prevalence | Female | Azerbaijan | AA | 0 (0 to 0) | 1.000 |
| Adolescence | Prevalence | Male | Azerbaijan | AA | 0 (0 to 0) | 1.000 |
| Adolescence | Prevalence | Both | Azerbaijan | T1D | 1.17 (1.15 to 1.2) | < 0.001 |
| Adolescence | Prevalence | Female | Azerbaijan | T1D | 1.17 (1.14 to 1.2) | < 0.001 |
| Adolescence | Prevalence | Male | Azerbaijan | T1D | 1.19 (1.16 to 1.22) | < 0.001 |
| Adolescence | Prevalence | Both | Azerbaijan | IBD | 0.16 (0.11 to 0.21) | < 0.001 |
| Adolescence | Prevalence | Female | Azerbaijan | IBD | 0.15 (0.13 to 0.17) | < 0.001 |
| Adolescence | Prevalence | Male | Azerbaijan | IBD | 0.16 (0.08 to 0.25) | < 0.001 |
| Adolescence | Prevalence | Both | Azerbaijan | MS | 0.28 (0.17 to 0.39) | < 0.001 |

| | | | | | | |
|---|---|---|---|---|---|---|
| Adolescence | Prevalence | Female | Azerbaijan | MS | 0.34 (0.19 to 0.5) | < 0.001 |
| Adolescence | Prevalence | Male | Azerbaijan | MS | 0.26 (0.17 to 0.35) | < 0.001 |
| Adolescence | Prevalence | Both | Azerbaijan | Psoriasis | -0.7 (-0.75 to -0.65) | < 0.001 |
| Adolescence | Prevalence | Female | Azerbaijan | Psoriasis | -0.7 (-0.78 to -0.62) | < 0.001 |
| Adolescence | Prevalence | Male | Azerbaijan | Psoriasis | -0.66 (-0.77 to -0.55) | < 0.001 |
| Adolescence | Prevalence | Both | Azerbaijan | RHD | -0.08 (-0.09 to -0.06) | < 0.001 |
| Adolescence | Prevalence | Female | Azerbaijan | RHD | -0.08 (-0.11 to -0.05) | < 0.001 |
| Adolescence | Prevalence | Male | Azerbaijan | RHD | -0.08 (-0.11 to -0.05) | < 0.001 |
| Adolescence | Prevalence | Both | Azerbaijan | RA | 0.63 (0.57 to 0.68) | < 0.001 |
| Adolescence | Prevalence | Female | Azerbaijan | RA | 0.64 (0.62 to 0.67) | < 0.001 |
| Adolescence | Prevalence | Male | Azerbaijan | RA | 0.71 (0.63 to 0.78) | < 0.001 |
| Adolescence | Prevalence | Both | Bahrain | AA | -0.02 (-0.05 to 0.01) | 0.187 |
| Adolescence | Prevalence | Female | Bahrain | AA | 0 (0 to 0) | 0.001 |
| Adolescence | Prevalence | Male | Bahrain | AA | 0 (0 to 0) | 0.011 |
| Adolescence | Prevalence | Both | Bahrain | T1D | 2.22 (2.18 to 2.27) | < 0.001 |
| Adolescence | Prevalence | Female | Bahrain | T1D | 2.19 (2.11 to 2.27) | < 0.001 |
| Adolescence | Prevalence | Male | Bahrain | T1D | 2.25 | < 0.001 |

| | | | | | | |
|---|---|---|---|---|---|---|
| | | | | | (2.21 to 2.29) | |
| Adolescence | Prevalence | Both | Bahrain | IBD | -0.08 (-0.14 to -0.02) | 0.006 |
| Adolescence | Prevalence | Female | Bahrain | IBD | -0.03 (-0.12 to 0.06) | 0.547 |
| Adolescence | Prevalence | Male | Bahrain | IBD | -0.13 (-0.18 to -0.08) | < 0.001 |
| Adolescence | Prevalence | Both | Bahrain | MS | 0.39 (0.32 to 0.46) | < 0.001 |
| Adolescence | Prevalence | Female | Bahrain | MS | 0.41 (0.36 to 0.47) | < 0.001 |
| Adolescence | Prevalence | Male | Bahrain | MS | 0.49 (0.43 to 0.54) | < 0.001 |
| Adolescence | Prevalence | Both | Bahrain | Psoriasis | -0.96 (-0.99 to -0.93) | < 0.001 |
| Adolescence | Prevalence | Female | Bahrain | Psoriasis | -0.93 (-0.95 to -0.91) | < 0.001 |
| Adolescence | Prevalence | Male | Bahrain | Psoriasis | -1.01 (-1.03 to -0.98) | < 0.001 |
| Adolescence | Prevalence | Both | Bahrain | RHD | -0.7 (-0.79 to -0.61) | < 0.001 |
| Adolescence | Prevalence | Female | Bahrain | RHD | -0.6 (-0.7 to -0.5) | < 0.001 |
| Adolescence | Prevalence | Male | Bahrain | RHD | -0.76 (-0.81 to -0.71) | < 0.001 |
| Adolescence | Prevalence | Both | Bahrain | RA | 0.67 (0.62 to 0.73) | < 0.001 |
| Adolescence | Prevalence | Female | Bahrain | RA | 0.69 (0.67 to 0.7) | < 0.001 |
| Adolescence | Prevalence | Male | Bahrain | RA | 0.8 (0.75 to 0.85) | < 0.001 |
| Adolescence | Prevalence | Both | Bangladesh | AA | 0.01 (0.01 to 0.02) | < 0.001 |

| | | | | | | |
|---|---|---|---|---|---|---|
| Adolescence | Prevalence | Female | Bangladesh | AA | 0<br>(0 to 0) | 0.008 |
| Adolescence | Prevalence | Male | Bangladesh | AA | 0<br>(0 to 0) | 1.000 |
| Adolescence | Prevalence | Both | Bangladesh | T1D | 0.13<br>(0.1 to 0.15) | < 0.001 |
| Adolescence | Prevalence | Female | Bangladesh | T1D | 0.16<br>(0.14 to 0.18) | < 0.001 |
| Adolescence | Prevalence | Male | Bangladesh | T1D | 0.09<br>(0.04 to 0.14) | 0.001 |
| Adolescence | Prevalence | Both | Bangladesh | IBD | 0.59<br>(0.53 to 0.64) | < 0.001 |
| Adolescence | Prevalence | Female | Bangladesh | IBD | 0.57<br>(0.52 to 0.62) | < 0.001 |
| Adolescence | Prevalence | Male | Bangladesh | IBD | 0.61<br>(0.57 to 0.65) | < 0.001 |
| Adolescence | Prevalence | Both | Bangladesh | MS | 0.08<br>(-0.01 to 0.17) | 0.081 |
| Adolescence | Prevalence | Female | Bangladesh | MS | 0.04<br>(-0.04 to 0.13) | 0.326 |
| Adolescence | Prevalence | Male | Bangladesh | MS | 0.06<br>(-0.03 to 0.15) | 0.177 |
| Adolescence | Prevalence | Both | Bangladesh | Psoriasis | -0.65<br>(-0.66 to -0.64) | < 0.001 |
| Adolescence | Prevalence | Female | Bangladesh | Psoriasis | -0.65<br>(-0.66 to -0.64) | < 0.001 |
| Adolescence | Prevalence | Male | Bangladesh | Psoriasis | -0.65<br>(-0.66 to -0.64) | < 0.001 |
| Adolescence | Prevalence | Both | Bangladesh | RHD | 0.61<br>(0.56 to 0.67) | < 0.001 |
| Adolescence | Prevalence | Female | Bangladesh | RHD | 0.56<br>(0.47 to 0.65) | < 0.001 |
| Adolescence | Prevalence | Male | Bangladesh | RHD | 0.68 | < 0.001 |

| | | | | | | |
|---|---|---|---|---|---|---|
| | | | | | (0.62 to 0.74) | |
| Adolescence | Prevalence | Both | Bangladesh | RA | 0.76 (0.75 to 0.77) | < 0.001 |
| Adolescence | Prevalence | Female | Bangladesh | RA | 0.73 (0.68 to 0.79) | < 0.001 |
| Adolescence | Prevalence | Male | Bangladesh | RA | 0.77 (0.69 to 0.84) | < 0.001 |
| Adolescence | Prevalence | Both | Barbados | AA | -0.01 (-0.01 to -0.01) | < 0.001 |
| Adolescence | Prevalence | Female | Barbados | AA | 0 (0 to 0) | 1.000 |
| Adolescence | Prevalence | Male | Barbados | AA | 0 (0 to 0) | 1.000 |
| Adolescence | Prevalence | Both | Barbados | T1D | 1.01 (0.99 to 1.02) | < 0.001 |
| Adolescence | Prevalence | Female | Barbados | T1D | 0.97 (0.96 to 0.98) | < 0.001 |
| Adolescence | Prevalence | Male | Barbados | T1D | 1.06 (1 to 1.12) | < 0.001 |
| Adolescence | Prevalence | Both | Barbados | IBD | -0.15 (-0.17 to -0.12) | < 0.001 |
| Adolescence | Prevalence | Female | Barbados | IBD | -0.04 (-0.06 to -0.02) | < 0.001 |
| Adolescence | Prevalence | Male | Barbados | IBD | -0.27 (-0.31 to -0.23) | < 0.001 |
| Adolescence | Prevalence | Both | Barbados | MS | 0.24 (0.11 to 0.38) | < 0.001 |
| Adolescence | Prevalence | Female | Barbados | MS | 0.26 (0.16 to 0.35) | < 0.001 |
| Adolescence | Prevalence | Male | Barbados | MS | 0.21 (-0.05 to 0.47) | 0.119 |
| Adolescence | Prevalence | Both | Barbados | Psoriasis | -0.24 (-0.26 to -0.22) | < 0.001 |

| | | | | | | |
|---|---|---|---|---|---|---|
| Adolescence | Prevalence | Female | Barbados | Psoriasis | -0.24<br>(-0.26 to -0.22) | < 0.001 |
| Adolescence | Prevalence | Male | Barbados | Psoriasis | -0.23<br>(-0.25 to -0.21) | < 0.001 |
| Adolescence | Prevalence | Both | Barbados | RHD | 0.04<br>(0.01 to 0.06) | 0.002 |
| Adolescence | Prevalence | Female | Barbados | RHD | 0.09<br>(0.08 to 0.11) | < 0.001 |
| Adolescence | Prevalence | Male | Barbados | RHD | -0.02<br>(-0.07 to 0.03) | 0.502 |
| Adolescence | Prevalence | Both | Barbados | RA | 0.55<br>(0.52 to 0.58) | < 0.001 |
| Adolescence | Prevalence | Female | Barbados | RA | 0.55<br>(0.51 to 0.59) | < 0.001 |
| Adolescence | Prevalence | Male | Barbados | RA | 0.6<br>(0.57 to 0.63) | < 0.001 |
| Adolescence | Prevalence | Both | Belarus | AA | -0.02<br>(-0.02 to -0.02) | < 0.001 |
| Adolescence | Prevalence | Female | Belarus | AA | 0<br>(0 to 0) | 1.000 |
| Adolescence | Prevalence | Male | Belarus | AA | 0<br>(0 to 0) | 1.000 |
| Adolescence | Prevalence | Both | Belarus | T1D | 1.57<br>(1.52 to 1.63) | < 0.001 |
| Adolescence | Prevalence | Female | Belarus | T1D | 1.57<br>(1.51 to 1.63) | < 0.001 |
| Adolescence | Prevalence | Male | Belarus | T1D | 1.62<br>(1.57 to 1.66) | < 0.001 |
| Adolescence | Prevalence | Both | Belarus | IBD | 0.25<br>(0.24 to 0.26) | < 0.001 |
| Adolescence | Prevalence | Female | Belarus | IBD | 0.24<br>(0.21 to 0.26) | < 0.001 |
| Adolescence | Prevalence | Male | Belarus | IBD | 0.28 | < 0.001 |

| | | | | | | |
|---|---|---|---|---|---|---|
| | | | | | (0.26 to 0.3) | |
| Adolescence | Prevalence | Both | Belarus | MS | 0.21<br>(0.12 to 0.3) | < 0.001 |
| Adolescence | Prevalence | Female | Belarus | MS | 0.24<br>(0.18 to 0.3) | < 0.001 |
| Adolescence | Prevalence | Male | Belarus | MS | 0.13<br>(-0.06 to 0.32) | 0.190 |
| Adolescence | Prevalence | Both | Belarus | Psoriasis | -0.83<br>(-0.84 to -0.81) | < 0.001 |
| Adolescence | Prevalence | Female | Belarus | Psoriasis | -0.82<br>(-0.83 to -0.8) | < 0.001 |
| Adolescence | Prevalence | Male | Belarus | Psoriasis | -0.82<br>(-0.83 to -0.8) | < 0.001 |
| Adolescence | Prevalence | Both | Belarus | RHD | -0.46<br>(-0.55 to -0.37) | < 0.001 |
| Adolescence | Prevalence | Female | Belarus | RHD | -0.48<br>(-0.58 to -0.38) | < 0.001 |
| Adolescence | Prevalence | Male | Belarus | RHD | -0.45<br>(-0.66 to -0.24) | < 0.001 |
| Adolescence | Prevalence | Both | Belarus | RA | 0.58<br>(0.56 to 0.6) | < 0.001 |
| Adolescence | Prevalence | Female | Belarus | RA | 0.61<br>(0.58 to 0.64) | < 0.001 |
| Adolescence | Prevalence | Male | Belarus | RA | 0.6<br>(0.59 to 0.62) | < 0.001 |
| Adolescence | Prevalence | Both | Belgium | AA | 0<br>(0 to 0) | 0.105 |
| Adolescence | Prevalence | Female | Belgium | AA | 0<br>(0 to 0) | 1.000 |
| Adolescence | Prevalence | Male | Belgium | AA | 0<br>(0 to 0) | 0.005 |
| Adolescence | Prevalence | Both | Belgium | T1D | 1.8<br>(1.66 to 1.93) | < 0.001 |

| | | | | | | |
|---|---|---|---|---|---|---|
| Adolescence | Prevalence | Female | Belgium | T1D | 1.8<br>(1.71 to 1.89) | < 0.001 |
| Adolescence | Prevalence | Male | Belgium | T1D | 1.82<br>(1.67 to 1.96) | < 0.001 |
| Adolescence | Prevalence | Both | Belgium | IBD | -0.53<br>(-0.65 to -0.41) | < 0.001 |
| Adolescence | Prevalence | Female | Belgium | IBD | -0.59<br>(-0.73 to -0.45) | < 0.001 |
| Adolescence | Prevalence | Male | Belgium | IBD | -0.43<br>(-0.57 to -0.29) | < 0.001 |
| Adolescence | Prevalence | Both | Belgium | MS | 0.4<br>(0.34 to 0.47) | < 0.001 |
| Adolescence | Prevalence | Female | Belgium | MS | 0.55<br>(0.51 to 0.58) | < 0.001 |
| Adolescence | Prevalence | Male | Belgium | MS | 0.23<br>(0.17 to 0.29) | < 0.001 |
| Adolescence | Prevalence | Both | Belgium | Psoriasis | -0.39<br>(-0.4 to -0.38) | < 0.001 |
| Adolescence | Prevalence | Female | Belgium | Psoriasis | -0.39<br>(-0.4 to -0.38) | < 0.001 |
| Adolescence | Prevalence | Male | Belgium | Psoriasis | -0.4<br>(-0.41 to -0.38) | < 0.001 |
| Adolescence | Prevalence | Both | Belgium | RHD | -0.04<br>(-0.43 to 0.35) | 0.838 |
| Adolescence | Prevalence | Female | Belgium | RHD | -0.03<br>(-0.46 to 0.39) | 0.876 |
| Adolescence | Prevalence | Male | Belgium | RHD | -0.1<br>(-0.46 to 0.26) | 0.601 |
| Adolescence | Prevalence | Both | Belgium | RA | 0.46<br>(0.43 to 0.48) | < 0.001 |
| Adolescence | Prevalence | Female | Belgium | RA | 0.44<br>(0.42 to 0.47) | < 0.001 |
| Adolescence | Prevalence | Male | Belgium | RA | 0.46 | < 0.001 |

| | | | | | | |
|---|---|---|---|---|---|---|
| | | | | | (0.45 to 0.47) | |
| Adolescence | Prevalence | Both | Belize | AA | 0.01 (0.01 to 0.01) | < 0.001 |
| Adolescence | Prevalence | Female | Belize | AA | 0 (0 to 0) | 1.000 |
| Adolescence | Prevalence | Male | Belize | AA | 0 (0 to 0) | 1.000 |
| Adolescence | Prevalence | Both | Belize | T1D | 1.03 (1.02 to 1.04) | < 0.001 |
| Adolescence | Prevalence | Female | Belize | T1D | 0.99 (0.96 to 1.03) | < 0.001 |
| Adolescence | Prevalence | Male | Belize | T1D | 1.08 (1.05 to 1.1) | < 0.001 |
| Adolescence | Prevalence | Both | Belize | IBD | 0.28 (0.24 to 0.31) | < 0.001 |
| Adolescence | Prevalence | Female | Belize | IBD | 0.27 (0.24 to 0.3) | < 0.001 |
| Adolescence | Prevalence | Male | Belize | IBD | 0.24 (0.18 to 0.29) | < 0.001 |
| Adolescence | Prevalence | Both | Belize | MS | 0.25 (0.2 to 0.29) | < 0.001 |
| Adolescence | Prevalence | Female | Belize | MS | 0.26 (0.2 to 0.31) | < 0.001 |
| Adolescence | Prevalence | Male | Belize | MS | 0.25 (0.2 to 0.31) | < 0.001 |
| Adolescence | Prevalence | Both | Belize | Psoriasis | -0.64 (-0.65 to -0.62) | < 0.001 |
| Adolescence | Prevalence | Female | Belize | Psoriasis | -0.64 (-0.65 to -0.63) | < 0.001 |
| Adolescence | Prevalence | Male | Belize | Psoriasis | -0.64 (-0.65 to -0.62) | < 0.001 |
| Adolescence | Prevalence | Both | Belize | RHD | 0.02 (0 to 0.04) | 0.017 |

| | | | | | | |
|---|---|---|---|---|---|---|
| Adolescence | Prevalence | Female | Belize | RHD | 0.05<br>(0.02 to 0.08) | < 0.001 |
| Adolescence | Prevalence | Male | Belize | RHD | -0.01<br>(-0.04 to 0.03) | 0.751 |
| Adolescence | Prevalence | Both | Belize | RA | 0.76<br>(0.69 to 0.83) | < 0.001 |
| Adolescence | Prevalence | Female | Belize | RA | 0.73<br>(0.65 to 0.81) | < 0.001 |
| Adolescence | Prevalence | Male | Belize | RA | 0.79<br>(0.72 to 0.85) | < 0.001 |
| Adolescence | Prevalence | Both | Benin | AA | 0.01<br>(0.01 to 0.01) | < 0.001 |
| Adolescence | Prevalence | Female | Benin | AA | 0<br>(0 to 0) | 1.000 |
| Adolescence | Prevalence | Male | Benin | AA | 0<br>(0 to 0) | 1.000 |
| Adolescence | Prevalence | Both | Benin | T1D | 0.02<br>(0 to 0.05) | 0.032 |
| Adolescence | Prevalence | Female | Benin | T1D | 0.04<br>(0.04 to 0.05) | < 0.001 |
| Adolescence | Prevalence | Male | Benin | T1D | 0.01<br>(-0.02 to 0.05) | 0.475 |
| Adolescence | Prevalence | Both | Benin | IBD | 0.29<br>(0.14 to 0.43) | < 0.001 |
| Adolescence | Prevalence | Female | Benin | IBD | 0.3<br>(0.2 to 0.4) | < 0.001 |
| Adolescence | Prevalence | Male | Benin | IBD | 0.32<br>(0.17 to 0.47) | < 0.001 |
| Adolescence | Prevalence | Both | Benin | MS | 0.19<br>(0.11 to 0.26) | < 0.001 |
| Adolescence | Prevalence | Female | Benin | MS | 0.1<br>(-0.01 to 0.22) | 0.080 |
| Adolescence | Prevalence | Male | Benin | MS | 0.2 | < 0.001 |

| | | | | | | |
|---|---|---|---|---|---|---|
| | | | | | (0.1 to 0.3) | |
| Adolescence | Prevalence | Both | Benin | Psoriasis | -0.64 (-0.81 to -0.47) | < 0.001 |
| Adolescence | Prevalence | Female | Benin | Psoriasis | -0.7 (-0.83 to -0.57) | < 0.001 |
| Adolescence | Prevalence | Male | Benin | Psoriasis | -0.55 (-0.71 to -0.39) | < 0.001 |
| Adolescence | Prevalence | Both | Benin | RHD | 0.19 (0.17 to 0.2) | < 0.001 |
| Adolescence | Prevalence | Female | Benin | RHD | 0.18 (0.17 to 0.2) | < 0.001 |
| Adolescence | Prevalence | Male | Benin | RHD | 0.17 (0.13 to 0.22) | < 0.001 |
| Adolescence | Prevalence | Both | Benin | RA | 0.55 (0.48 to 0.62) | < 0.001 |
| Adolescence | Prevalence | Female | Benin | RA | 0.57 (0.49 to 0.65) | < 0.001 |
| Adolescence | Prevalence | Male | Benin | RA | 0.52 (0.49 to 0.56) | < 0.001 |
| Adolescence | Prevalence | Both | Bermuda | AA | 0.01 (0 to 0.01) | < 0.001 |
| Adolescence | Prevalence | Female | Bermuda | AA | 0 (0 to 0) | 1.000 |
| Adolescence | Prevalence | Male | Bermuda | AA | 0 (0 to 0) | 1.000 |
| Adolescence | Prevalence | Both | Bermuda | T1D | 1.33 (1.32 to 1.35) | < 0.001 |
| Adolescence | Prevalence | Female | Bermuda | T1D | 1.21 (1.2 to 1.23) | < 0.001 |
| Adolescence | Prevalence | Male | Bermuda | T1D | 1.46 (1.45 to 1.47) | < 0.001 |
| Adolescence | Prevalence | Both | Bermuda | IBD | 0.05 (0.01 to 0.08) | 0.023 |

| | | | | | | |
|---|---|---|---|---|---|---|
| Adolescence | Prevalence | Female | Bermuda | IBD | 0.07<br>(0.04 to 0.1) | < 0.001 |
| Adolescence | Prevalence | Male | Bermuda | IBD | 0.03<br>(-0.01 to 0.07) | 0.160 |
| Adolescence | Prevalence | Both | Bermuda | MS | 0.25<br>(0.21 to 0.29) | < 0.001 |
| Adolescence | Prevalence | Female | Bermuda | MS | 0.27<br>(0.2 to 0.33) | < 0.001 |
| Adolescence | Prevalence | Male | Bermuda | MS | 0.23<br>(0.2 to 0.27) | < 0.001 |
| Adolescence | Prevalence | Both | Bermuda | Psoriasis | -0.37<br>(-0.39 to -0.35) | < 0.001 |
| Adolescence | Prevalence | Female | Bermuda | Psoriasis | -0.38<br>(-0.4 to -0.36) | < 0.001 |
| Adolescence | Prevalence | Male | Bermuda | Psoriasis | -0.37<br>(-0.39 to -0.35) | < 0.001 |
| Adolescence | Prevalence | Both | Bermuda | RHD | -1.15<br>(-1.28 to -1.01) | < 0.001 |
| Adolescence | Prevalence | Female | Bermuda | RHD | -1.01<br>(-1.13 to -0.89) | < 0.001 |
| Adolescence | Prevalence | Male | Bermuda | RHD | -1.29<br>(-1.46 to -1.12) | < 0.001 |
| Adolescence | Prevalence | Both | Bermuda | RA | 0.63<br>(0.59 to 0.67) | < 0.001 |
| Adolescence | Prevalence | Female | Bermuda | RA | 0.6<br>(0.57 to 0.63) | < 0.001 |
| Adolescence | Prevalence | Male | Bermuda | RA | 0.65<br>(0.63 to 0.67) | < 0.001 |
| Adolescence | Prevalence | Both | Bhutan | AA | 0.06<br>(0.05 to 0.08) | < 0.001 |
| Adolescence | Prevalence | Female | Bhutan | AA | 0<br>(0 to 0) | 0.008 |
| Adolescence | Prevalence | Male | Bhutan | AA | 0 | 1.000 |

| | | | | | | |
|---|---|---|---|---|---|---|
| | | | | | (0 to 0) | |
| Adolescence | Prevalence | Both | Bhutan | T1D | 0.1<br>(0.09 to 0.11) | < 0.001 |
| Adolescence | Prevalence | Female | Bhutan | T1D | 0.14<br>(0.09 to 0.19) | < 0.001 |
| Adolescence | Prevalence | Male | Bhutan | T1D | 0.07<br>(0.06 to 0.07) | < 0.001 |
| Adolescence | Prevalence | Both | Bhutan | IBD | 0.67<br>(0.54 to 0.8) | < 0.001 |
| Adolescence | Prevalence | Female | Bhutan | IBD | 0.69<br>(0.6 to 0.79) | < 0.001 |
| Adolescence | Prevalence | Male | Bhutan | IBD | 0.67<br>(0.6 to 0.74) | < 0.001 |
| Adolescence | Prevalence | Both | Bhutan | MS | 0.13<br>(0.09 to 0.16) | < 0.001 |
| Adolescence | Prevalence | Female | Bhutan | MS | 0.09<br>(0.05 to 0.13) | < 0.001 |
| Adolescence | Prevalence | Male | Bhutan | MS | 0.1<br>(0 to 0.2) | 0.059 |
| Adolescence | Prevalence | Both | Bhutan | Psoriasis | -0.61<br>(-0.62 to -0.6) | < 0.001 |
| Adolescence | Prevalence | Female | Bhutan | Psoriasis | -0.61<br>(-0.62 to -0.6) | < 0.001 |
| Adolescence | Prevalence | Male | Bhutan | Psoriasis | -0.62<br>(-0.63 to -0.61) | < 0.001 |
| Adolescence | Prevalence | Both | Bhutan | RHD | 0.06<br>(0.05 to 0.07) | < 0.001 |
| Adolescence | Prevalence | Female | Bhutan | RHD | 0.05<br>(0 to 0.1) | 0.070 |
| Adolescence | Prevalence | Male | Bhutan | RHD | 0.02<br>(-0.02 to 0.06) | 0.319 |
| Adolescence | Prevalence | Both | Bhutan | RA | 0.98<br>(0.95 to 1.01) | < 0.001 |

| | | | | | | |
|---|---|---|---|---|---|---|
| Adolescence | Prevalence | Female | Bhutan | RA | 0.92<br>(0.89 to 0.94) | < 0.001 |
| Adolescence | Prevalence | Male | Bhutan | RA | 0.96<br>(0.92 to 1) | < 0.001 |
| Adolescence | Prevalence | Both | Bosnia and Herzegovina | AA | 0.01<br>(-0.01 to 0.02) | 0.280 |
| Adolescence | Prevalence | Female | Bosnia and Herzegovina | AA | 0<br>(0 to 0) | 1.000 |
| Adolescence | Prevalence | Male | Bosnia and Herzegovina | AA | 0<br>(0 to 0) | 1.000 |
| Adolescence | Prevalence | Both | Bosnia and Herzegovina | T1D | 1.78<br>(1.69 to 1.88) | < 0.001 |
| Adolescence | Prevalence | Female | Bosnia and Herzegovina | T1D | 1.75<br>(1.67 to 1.82) | < 0.001 |
| Adolescence | Prevalence | Male | Bosnia and Herzegovina | T1D | 1.83<br>(1.72 to 1.94) | < 0.001 |
| Adolescence | Prevalence | Both | Bosnia and Herzegovina | IBD | 0.58<br>(0.56 to 0.6) | < 0.001 |
| Adolescence | Prevalence | Female | Bosnia and Herzegovina | IBD | 0.42<br>(0.39 to 0.46) | < 0.001 |
| Adolescence | Prevalence | Male | Bosnia and Herzegovina | IBD | 0.75<br>(0.72 to 0.77) | < 0.001 |
| Adolescence | Prevalence | Both | Bosnia and Herzegovina | MS | -0.09<br>(-0.13 to -0.05) | < 0.001 |
| Adolescence | Prevalence | Female | Bosnia and Herzegovina | MS | -0.03<br>(-0.07 to 0.02) | 0.205 |
| Adolescence | Prevalence | Male | Bosnia and Herzegovina | MS | -0.2<br>(-0.26 to -0.15) | < 0.001 |
| Adolescence | Prevalence | Both | Bosnia and Herzegovina | Psoriasis | -1.07<br>(-1.11 to -1.02) | < 0.001 |
| Adolescence | Prevalence | Female | Bosnia and Herzegovina | Psoriasis | -1.08<br>(-1.15 to -1.01) | < 0.001 |
| Adolescence | Prevalence | Male | Bosnia and Herzegovina | Psoriasis | -1.05 | < 0.001 |

| | | | | | | |
|---|---|---|---|---|---|---|
| | | | | | (-1.07 to -1.03) | |
| Adolescence | Prevalence | Both | Bosnia and Herzegovina | RHD | -0.02<br>(-0.08 to 0.03) | 0.381 |
| Adolescence | Prevalence | Female | Bosnia and Herzegovina | RHD | -0.01<br>(-0.11 to 0.09) | 0.839 |
| Adolescence | Prevalence | Male | Bosnia and Herzegovina | RHD | -0.04<br>(-0.15 to 0.06) | 0.398 |
| Adolescence | Prevalence | Both | Bosnia and Herzegovina | RA | 0.68<br>(0.62 to 0.74) | < 0.001 |
| Adolescence | Prevalence | Female | Bosnia and Herzegovina | RA | 0.66<br>(0.64 to 0.69) | < 0.001 |
| Adolescence | Prevalence | Male | Bosnia and Herzegovina | RA | 0.72<br>(0.64 to 0.81) | < 0.001 |
| Adolescence | Prevalence | Both | Botswana | AA | -0.04<br>(-0.05 to -0.04) | < 0.001 |
| Adolescence | Prevalence | Female | Botswana | AA | 0<br>(0 to 0) | 1.000 |
| Adolescence | Prevalence | Male | Botswana | AA | 0<br>(0 to 0) | 1.000 |
| Adolescence | Prevalence | Both | Botswana | T1D | 0.14<br>(0.11 to 0.16) | < 0.001 |
| Adolescence | Prevalence | Female | Botswana | T1D | 0.12<br>(0.11 to 0.13) | < 0.001 |
| Adolescence | Prevalence | Male | Botswana | T1D | 0.15<br>(0.11 to 0.19) | < 0.001 |
| Adolescence | Prevalence | Both | Botswana | IBD | 0.54<br>(0.4 to 0.68) | < 0.001 |
| Adolescence | Prevalence | Female | Botswana | IBD | 0.56<br>(0.36 to 0.76) | < 0.001 |
| Adolescence | Prevalence | Male | Botswana | IBD | 0.54<br>(0.46 to 0.63) | < 0.001 |
| Adolescence | Prevalence | Both | Botswana | MS | -0.01<br>(-0.04 to 0.03) | 0.683 |

| | | | | | | |
|---|---|---|---|---|---|---|
| Adolescence | Prevalence | Female | Botswana | MS | 0.06<br>(-0.02 to 0.15) | 0.135 |
| Adolescence | Prevalence | Male | Botswana | MS | -0.05<br>(-0.19 to 0.1) | 0.551 |
| Adolescence | Prevalence | Both | Botswana | Psoriasis | -0.71<br>(-0.79 to -0.63) | < 0.001 |
| Adolescence | Prevalence | Female | Botswana | Psoriasis | -0.73<br>(-0.79 to -0.68) | < 0.001 |
| Adolescence | Prevalence | Male | Botswana | Psoriasis | -0.66<br>(-0.73 to -0.6) | < 0.001 |
| Adolescence | Prevalence | Both | Botswana | RHD | 0.03<br>(0.01 to 0.04) | < 0.001 |
| Adolescence | Prevalence | Female | Botswana | RHD | 0.02<br>(-0.01 to 0.05) | 0.135 |
| Adolescence | Prevalence | Male | Botswana | RHD | 0.07<br>(0.04 to 0.09) | < 0.001 |
| Adolescence | Prevalence | Both | Botswana | RA | 0.47<br>(0.42 to 0.53) | < 0.001 |
| Adolescence | Prevalence | Female | Botswana | RA | 0.49<br>(0.42 to 0.56) | < 0.001 |
| Adolescence | Prevalence | Male | Botswana | RA | 0.59<br>(0.55 to 0.63) | < 0.001 |
| Adolescence | Prevalence | Both | Brazil | AA | -0.02<br>(-0.02 to -0.02) | < 0.001 |
| Adolescence | Prevalence | Female | Brazil | AA | 0<br>(0 to 0) | 1.000 |
| Adolescence | Prevalence | Male | Brazil | AA | 0<br>(0 to 0) | 1.000 |
| Adolescence | Prevalence | Both | Brazil | T1D | 0.98<br>(0.9 to 1.06) | < 0.001 |
| Adolescence | Prevalence | Female | Brazil | T1D | 1.24<br>(1.18 to 1.31) | < 0.001 |
| Adolescence | Prevalence | Male | Brazil | T1D | 0.34 | 0.035 |

| | | | | | | |
|---|---|---|---|---|---|---|
| | | | | | (0.02 to 0.67) | |
| Adolescence | Prevalence | Both | Brazil | IBD | -0.45<br>(-0.53 to -0.37) | < 0.001 |
| Adolescence | Prevalence | Female | Brazil | IBD | -0.37<br>(-0.46 to -0.29) | < 0.001 |
| Adolescence | Prevalence | Male | Brazil | IBD | -0.53<br>(-0.62 to -0.43) | < 0.001 |
| Adolescence | Prevalence | Both | Brazil | MS | 0.24<br>(0.05 to 0.43) | 0.012 |
| Adolescence | Prevalence | Female | Brazil | MS | 0.23<br>(0.06 to 0.4) | 0.007 |
| Adolescence | Prevalence | Male | Brazil | MS | 0.23<br>(0.06 to 0.39) | 0.008 |
| Adolescence | Prevalence | Both | Brazil | Psoriasis | -0.2<br>(-0.21 to -0.2) | < 0.001 |
| Adolescence | Prevalence | Female | Brazil | Psoriasis | -0.2<br>(-0.21 to -0.19) | < 0.001 |
| Adolescence | Prevalence | Male | Brazil | Psoriasis | -0.2<br>(-0.22 to -0.17) | < 0.001 |
| Adolescence | Prevalence | Both | Brazil | RHD | 0.05<br>(0.03 to 0.07) | < 0.001 |
| Adolescence | Prevalence | Female | Brazil | RHD | 0.11<br>(0.09 to 0.13) | < 0.001 |
| Adolescence | Prevalence | Male | Brazil | RHD | -0.03<br>(-0.09 to 0.04) | 0.468 |
| Adolescence | Prevalence | Both | Brazil | RA | 0.46<br>(0.37 to 0.54) | < 0.001 |
| Adolescence | Prevalence | Female | Brazil | RA | 0.49<br>(0.4 to 0.58) | < 0.001 |
| Adolescence | Prevalence | Male | Brazil | RA | 0.48<br>(0.43 to 0.53) | < 0.001 |
| Adolescence | Prevalence | Both | Brunei Darussalam | AA | -0.01<br>(-0.02 to -0.01) | < 0.001 |

| | | | | | | |
|---|---|---|---|---|---|---|
| Adolescence | Prevalence | Female | Brunei Darussalam | AA | 0<br>(0 to 0) | 1.000 |
| Adolescence | Prevalence | Male | Brunei Darussalam | AA | 0<br>(0 to 0) | 1.000 |
| Adolescence | Prevalence | Both | Brunei Darussalam | T1D | -0.02<br>(-0.03 to -0.01) | < 0.001 |
| Adolescence | Prevalence | Female | Brunei Darussalam | T1D | 0.01<br>(0 to 0.02) | 0.134 |
| Adolescence | Prevalence | Male | Brunei Darussalam | T1D | -0.05<br>(-0.05 to -0.04) | < 0.001 |
| Adolescence | Prevalence | Both | Brunei Darussalam | IBD | 0.28<br>(0.25 to 0.31) | < 0.001 |
| Adolescence | Prevalence | Female | Brunei Darussalam | IBD | 0.26<br>(0.22 to 0.29) | < 0.001 |
| Adolescence | Prevalence | Male | Brunei Darussalam | IBD | 0.28<br>(0.19 to 0.36) | < 0.001 |
| Adolescence | Prevalence | Both | Brunei Darussalam | MS | 0.01<br>(-0.04 to 0.07) | 0.612 |
| Adolescence | Prevalence | Female | Brunei Darussalam | MS | 0.03<br>(-0.17 to 0.23) | 0.752 |
| Adolescence | Prevalence | Male | Brunei Darussalam | MS | 0.11<br>(-0.05 to 0.28) | 0.186 |
| Adolescence | Prevalence | Both | Brunei Darussalam | Psoriasis | -0.3<br>(-0.31 to -0.29) | < 0.001 |
| Adolescence | Prevalence | Female | Brunei Darussalam | Psoriasis | -0.35<br>(-0.36 to -0.33) | < 0.001 |
| Adolescence | Prevalence | Male | Brunei Darussalam | Psoriasis | -0.21<br>(-0.22 to -0.2) | < 0.001 |
| Adolescence | Prevalence | Both | Brunei Darussalam | RHD | -1.24<br>(-1.35 to -1.12) | < 0.001 |
| Adolescence | Prevalence | Female | Brunei Darussalam | RHD | -1.28<br>(-1.5 to -1.06) | < 0.001 |
| Adolescence | Prevalence | Male | Brunei Darussalam | RHD | -1.21 | < 0.001 |

| | | | | | | |
|---|---|---|---|---|---|---|
| | | | | | (-1.27 to -1.16) | |
| Adolescence | Prevalence | Both | Brunei Darussalam | RA | 0.38<br>(0.35 to 0.41) | < 0.001 |
| Adolescence | Prevalence | Female | Brunei Darussalam | RA | 0.4<br>(0.38 to 0.42) | < 0.001 |
| Adolescence | Prevalence | Male | Brunei Darussalam | RA | 0.45<br>(0.43 to 0.48) | < 0.001 |
| Adolescence | Prevalence | Both | Bulgaria | AA | -0.01<br>(-0.01 to -0.01) | < 0.001 |
| Adolescence | Prevalence | Female | Bulgaria | AA | 0<br>(0 to 0) | 1.000 |
| Adolescence | Prevalence | Male | Bulgaria | AA | 0<br>(0 to 0) | 1.000 |
| Adolescence | Prevalence | Both | Bulgaria | T1D | 1.47<br>(1.41 to 1.54) | < 0.001 |
| Adolescence | Prevalence | Female | Bulgaria | T1D | 1.46<br>(1.39 to 1.53) | < 0.001 |
| Adolescence | Prevalence | Male | Bulgaria | T1D | 1.48<br>(1.42 to 1.54) | < 0.001 |
| Adolescence | Prevalence | Both | Bulgaria | IBD | 0.12<br>(0.1 to 0.14) | < 0.001 |
| Adolescence | Prevalence | Female | Bulgaria | IBD | 0.11<br>(0.06 to 0.16) | < 0.001 |
| Adolescence | Prevalence | Male | Bulgaria | IBD | 0.13<br>(0.07 to 0.19) | < 0.001 |
| Adolescence | Prevalence | Both | Bulgaria | MS | 0.5<br>(0.46 to 0.53) | < 0.001 |
| Adolescence | Prevalence | Female | Bulgaria | MS | 0.61<br>(0.59 to 0.64) | < 0.001 |
| Adolescence | Prevalence | Male | Bulgaria | MS | 0.25<br>(0.15 to 0.35) | < 0.001 |
| Adolescence | Prevalence | Both | Bulgaria | Psoriasis | -0.83<br>(-0.86 to -0.79) | < 0.001 |

| | | | | | | |
|---|---|---|---|---|---|---|
| Adolescence | Prevalence | Female | Bulgaria | Psoriasis | -0.84 (-0.89 to -0.78) | < 0.001 |
| Adolescence | Prevalence | Male | Bulgaria | Psoriasis | -0.8 (-0.82 to -0.79) | < 0.001 |
| Adolescence | Prevalence | Both | Bulgaria | RHD | -1.36 (-1.52 to -1.2) | < 0.001 |
| Adolescence | Prevalence | Female | Bulgaria | RHD | -1.08 (-1.17 to -0.99) | < 0.001 |
| Adolescence | Prevalence | Male | Bulgaria | RHD | -1.53 (-1.68 to -1.38) | < 0.001 |
| Adolescence | Prevalence | Both | Bulgaria | RA | 0.31 (0.25 to 0.38) | < 0.001 |
| Adolescence | Prevalence | Female | Bulgaria | RA | 0.34 (0.29 to 0.39) | < 0.001 |
| Adolescence | Prevalence | Male | Bulgaria | RA | 0.3 (0.27 to 0.32) | < 0.001 |
| Adolescence | Prevalence | Both | Burkina Faso | AA | -0.01 (-0.01 to 0) | < 0.001 |
| Adolescence | Prevalence | Female | Burkina Faso | AA | 0 (0 to 0) | 1.000 |
| Adolescence | Prevalence | Male | Burkina Faso | AA | 0 (0 to 0) | 1.000 |
| Adolescence | Prevalence | Both | Burkina Faso | T1D | -0.03 (-0.06 to 0.01) | 0.115 |
| Adolescence | Prevalence | Female | Burkina Faso | T1D | 0.01 (-0.04 to 0.05) | 0.702 |
| Adolescence | Prevalence | Male | Burkina Faso | T1D | -0.05 (-0.08 to -0.03) | < 0.001 |
| Adolescence | Prevalence | Both | Burkina Faso | IBD | 0.31 (0.23 to 0.4) | < 0.001 |
| Adolescence | Prevalence | Female | Burkina Faso | IBD | 0.38 (0.29 to 0.47) | < 0.001 |
| Adolescence | Prevalence | Male | Burkina Faso | IBD | 0.26 | < 0.001 |

| | | | | | | |
|---|---|---|---|---|---|---|
| | | | | | (0.22 to 0.3) | |
| Adolescence | Prevalence | Both | Burkina Faso | MS | 0.12<br>(0.07 to 0.17) | < 0.001 |
| Adolescence | Prevalence | Female | Burkina Faso | MS | 0.1<br>(0.04 to 0.15) | 0.001 |
| Adolescence | Prevalence | Male | Burkina Faso | MS | 0.16<br>(0.07 to 0.25) | < 0.001 |
| Adolescence | Prevalence | Both | Burkina Faso | Psoriasis | -0.54<br>(-0.77 to -0.3) | < 0.001 |
| Adolescence | Prevalence | Female | Burkina Faso | Psoriasis | -0.6<br>(-0.79 to -0.42) | < 0.001 |
| Adolescence | Prevalence | Male | Burkina Faso | Psoriasis | -0.45<br>(-0.65 to -0.25) | < 0.001 |
| Adolescence | Prevalence | Both | Burkina Faso | RHD | 0.07<br>(0.06 to 0.09) | < 0.001 |
| Adolescence | Prevalence | Female | Burkina Faso | RHD | 0.07<br>(0.05 to 0.1) | < 0.001 |
| Adolescence | Prevalence | Male | Burkina Faso | RHD | 0.07<br>(0.06 to 0.08) | < 0.001 |
| Adolescence | Prevalence | Both | Burkina Faso | RA | 0.49<br>(0.47 to 0.52) | < 0.001 |
| Adolescence | Prevalence | Female | Burkina Faso | RA | 0.53<br>(0.51 to 0.55) | < 0.001 |
| Adolescence | Prevalence | Male | Burkina Faso | RA | 0.44<br>(0.42 to 0.47) | < 0.001 |
| Adolescence | Prevalence | Both | Burundi | AA | 0.02<br>(0.01 to 0.03) | 0.003 |
| Adolescence | Prevalence | Female | Burundi | AA | 0<br>(0 to 0) | 1.000 |
| Adolescence | Prevalence | Male | Burundi | AA | 0<br>(0 to 0) | 1.000 |
| Adolescence | Prevalence | Both | Burundi | T1D | 0.04<br>(0.01 to 0.07) | 0.004 |

| | | | | | | |
|---|---|---|---|---|---|---|
| Adolescence | Prevalence | Female | Burundi | T1D | 0.05<br>(0.01 to 0.08) | 0.005 |
| Adolescence | Prevalence | Male | Burundi | T1D | 0.05<br>(0.02 to 0.08) | < 0.001 |
| Adolescence | Prevalence | Both | Burundi | IBD | -0.19<br>(-0.27 to -0.11) | < 0.001 |
| Adolescence | Prevalence | Female | Burundi | IBD | -0.34<br>(-0.39 to -0.29) | < 0.001 |
| Adolescence | Prevalence | Male | Burundi | IBD | -0.02<br>(-0.1 to 0.07) | 0.727 |
| Adolescence | Prevalence | Both | Burundi | MS | -0.01<br>(-0.13 to 0.11) | 0.898 |
| Adolescence | Prevalence | Female | Burundi | MS | -0.08<br>(-0.22 to 0.05) | 0.237 |
| Adolescence | Prevalence | Male | Burundi | MS | 0.01<br>(-0.07 to 0.1) | 0.759 |
| Adolescence | Prevalence | Both | Burundi | Psoriasis | -0.02<br>(-0.06 to 0.03) | 0.437 |
| Adolescence | Prevalence | Female | Burundi | Psoriasis | -0.03<br>(-0.05 to -0.01) | 0.003 |
| Adolescence | Prevalence | Male | Burundi | Psoriasis | -0.01<br>(-0.1 to 0.09) | 0.894 |
| Adolescence | Prevalence | Both | Burundi | RHD | 0.16<br>(0.14 to 0.18) | < 0.001 |
| Adolescence | Prevalence | Female | Burundi | RHD | 0.16<br>(0.14 to 0.18) | < 0.001 |
| Adolescence | Prevalence | Male | Burundi | RHD | 0.15<br>(0.08 to 0.22) | < 0.001 |
| Adolescence | Prevalence | Both | Burundi | RA | 0.12<br>(0.03 to 0.21) | 0.012 |
| Adolescence | Prevalence | Female | Burundi | RA | 0.07<br>(0.02 to 0.13) | 0.004 |
| Adolescence | Prevalence | Male | Burundi | RA | 0.12 | < 0.001 |

| | | | | | | |
|---|---|---|---|---|---|---|
| | | | | | (0.08 to 0.15) | |
| Adolescence | Prevalence | Both | Cambodia | AA | -0.03<br>(-0.03 to -0.03) | < 0.001 |
| Adolescence | Prevalence | Female | Cambodia | AA | 0<br>(0 to 0) | 0.008 |
| Adolescence | Prevalence | Male | Cambodia | AA | 0<br>(0 to 0) | 1.000 |
| Adolescence | Prevalence | Both | Cambodia | T1D | 0.43<br>(0.4 to 0.46) | < 0.001 |
| Adolescence | Prevalence | Female | Cambodia | T1D | 0.42<br>(0.37 to 0.48) | < 0.001 |
| Adolescence | Prevalence | Male | Cambodia | T1D | 0.43<br>(0.33 to 0.53) | < 0.001 |
| Adolescence | Prevalence | Both | Cambodia | IBD | 0.67<br>(0.58 to 0.76) | < 0.001 |
| Adolescence | Prevalence | Female | Cambodia | IBD | 0.71<br>(0.63 to 0.78) | < 0.001 |
| Adolescence | Prevalence | Male | Cambodia | IBD | 0.68<br>(0.58 to 0.79) | < 0.001 |
| Adolescence | Prevalence | Both | Cambodia | MS | -0.07<br>(-0.07 to -0.07) | < 0.001 |
| Adolescence | Prevalence | Female | Cambodia | MS | -0.01<br>(-0.08 to 0.06) | 0.772 |
| Adolescence | Prevalence | Male | Cambodia | MS | -0.03<br>(-0.27 to 0.22) | 0.832 |
| Adolescence | Prevalence | Both | Cambodia | Psoriasis | -0.65<br>(-0.67 to -0.64) | < 0.001 |
| Adolescence | Prevalence | Female | Cambodia | Psoriasis | -0.6<br>(-0.61 to -0.59) | < 0.001 |
| Adolescence | Prevalence | Male | Cambodia | Psoriasis | -0.65<br>(-0.7 to -0.59) | < 0.001 |
| Adolescence | Prevalence | Both | Cambodia | RHD | -0.04<br>(-0.45 to 0.38) | 0.867 |

| | | | | | | |
|---|---|---|---|---|---|---|
| Adolescence | Prevalence | Female | Cambodia | RHD | 0.02<br>(-0.39 to 0.43) | 0.939 |
| Adolescence | Prevalence | Male | Cambodia | RHD | -0.07<br>(-0.5 to 0.36) | 0.747 |
| Adolescence | Prevalence | Both | Cambodia | RA | 0.32<br>(0.3 to 0.34) | < 0.001 |
| Adolescence | Prevalence | Female | Cambodia | RA | 0.38<br>(0.35 to 0.41) | < 0.001 |
| Adolescence | Prevalence | Male | Cambodia | RA | 0.27<br>(0.19 to 0.35) | < 0.001 |
| Adolescence | Prevalence | Both | Cameroon | AA | -0.01<br>(-0.01 to -0.01) | < 0.001 |
| Adolescence | Prevalence | Female | Cameroon | AA | 0<br>(0 to 0) | 1.000 |
| Adolescence | Prevalence | Male | Cameroon | AA | 0<br>(0 to 0) | 1.000 |
| Adolescence | Prevalence | Both | Cameroon | T1D | 0.07<br>(0.06 to 0.09) | < 0.001 |
| Adolescence | Prevalence | Female | Cameroon | T1D | 0.09<br>(0.07 to 0.12) | < 0.001 |
| Adolescence | Prevalence | Male | Cameroon | T1D | 0.06<br>(0.05 to 0.07) | < 0.001 |
| Adolescence | Prevalence | Both | Cameroon | IBD | 0.36<br>(0.31 to 0.42) | < 0.001 |
| Adolescence | Prevalence | Female | Cameroon | IBD | 0.36<br>(0.28 to 0.44) | < 0.001 |
| Adolescence | Prevalence | Male | Cameroon | IBD | 0.37<br>(0.31 to 0.43) | < 0.001 |
| Adolescence | Prevalence | Both | Cameroon | MS | 0.1<br>(0.02 to 0.18) | 0.015 |
| Adolescence | Prevalence | Female | Cameroon | MS | 0.13<br>(0.04 to 0.23) | 0.007 |
| Adolescence | Prevalence | Male | Cameroon | MS | 0.11 | 0.144 |

| | | | | | | |
|---|---|---|---|---|---|---|
| | | | | | (-0.04 to 0.27) | |
| Adolescence | Prevalence | Both | Cameroon | Psoriasis | -0.69<br>(-0.86 to -0.51) | < 0.001 |
| Adolescence | Prevalence | Female | Cameroon | Psoriasis | -0.73<br>(-0.82 to -0.64) | < 0.001 |
| Adolescence | Prevalence | Male | Cameroon | Psoriasis | -0.6<br>(-0.76 to -0.45) | < 0.001 |
| Adolescence | Prevalence | Both | Cameroon | RHD | 0.28<br>(0.27 to 0.29) | < 0.001 |
| Adolescence | Prevalence | Female | Cameroon | RHD | 0.27<br>(0.24 to 0.3) | < 0.001 |
| Adolescence | Prevalence | Male | Cameroon | RHD | 0.27<br>(0.21 to 0.33) | < 0.001 |
| Adolescence | Prevalence | Both | Cameroon | RA | 0.35<br>(0.3 to 0.39) | < 0.001 |
| Adolescence | Prevalence | Female | Cameroon | RA | 0.38<br>(0.33 to 0.43) | < 0.001 |
| Adolescence | Prevalence | Male | Cameroon | RA | 0.34<br>(0.28 to 0.4) | < 0.001 |
| Adolescence | Prevalence | Both | Canada | AA | 0<br>(0 to 0) | 0.499 |
| Adolescence | Prevalence | Female | Canada | AA | 0<br>(0 to 0) | 1.000 |
| Adolescence | Prevalence | Male | Canada | AA | 0<br>(0 to 0) | 1.000 |
| Adolescence | Prevalence | Both | Canada | T1D | -0.25<br>(-0.71 to 0.21) | 0.291 |
| Adolescence | Prevalence | Female | Canada | T1D | -0.44<br>(-0.99 to 0.11) | 0.120 |
| Adolescence | Prevalence | Male | Canada | T1D | -0.07<br>(-0.47 to 0.32) | 0.711 |
| Adolescence | Prevalence | Both | Canada | IBD | -0.51<br>(-0.7 to -0.32) | < 0.001 |

| | | | | | | |
|---|---|---|---|---|---|---|
| Adolescence | Prevalence | Female | Canada | IBD | -0.52 (-0.82 to -0.22) | 0.001 |
| Adolescence | Prevalence | Male | Canada | IBD | -0.52 (-0.59 to -0.45) | < 0.001 |
| Adolescence | Prevalence | Both | Canada | MS | 0.93 (0.9 to 0.95) | < 0.001 |
| Adolescence | Prevalence | Female | Canada | MS | 1 (0.94 to 1.05) | < 0.001 |
| Adolescence | Prevalence | Male | Canada | MS | 0.77 (0.66 to 0.88) | < 0.001 |
| Adolescence | Prevalence | Both | Canada | Psoriasis | -0.42 (-0.42 to -0.41) | < 0.001 |
| Adolescence | Prevalence | Female | Canada | Psoriasis | -0.41 (-0.42 to -0.41) | < 0.001 |
| Adolescence | Prevalence | Male | Canada | Psoriasis | -0.42 (-0.43 to -0.41) | < 0.001 |
| Adolescence | Prevalence | Both | Canada | RHD | -0.1 (-0.15 to -0.06) | < 0.001 |
| Adolescence | Prevalence | Female | Canada | RHD | -0.16 (-0.23 to -0.08) | < 0.001 |
| Adolescence | Prevalence | Male | Canada | RHD | -0.06 (-0.09 to -0.02) | 0.005 |
| Adolescence | Prevalence | Both | Canada | RA | 0.84 (0.82 to 0.86) | < 0.001 |
| Adolescence | Prevalence | Female | Canada | RA | 0.89 (0.85 to 0.92) | < 0.001 |
| Adolescence | Prevalence | Male | Canada | RA | 0.76 (0.75 to 0.77) | < 0.001 |
| Adolescence | Prevalence | Both | Central African Republic | AA | 0.01 (0 to 0.02) | 0.033 |
| Adolescence | Prevalence | Female | Central African Republic | AA | 0 (0 to 0) | 1.000 |
| Adolescence | Prevalence | Male | Central African Republic | AA | 0 | 1.000 |

| | | | | | | |
|---|---|---|---|---|---|---|
| | | | | | (0 to 0) | |
| Adolescence | Prevalence | Both | Central African Republic | T1D | 0.12<br>(0.1 to 0.15) | < 0.001 |
| Adolescence | Prevalence | Female | Central African Republic | T1D | 0.15<br>(0.12 to 0.19) | < 0.001 |
| Adolescence | Prevalence | Male | Central African Republic | T1D | 0.11<br>(0.08 to 0.14) | < 0.001 |
| Adolescence | Prevalence | Both | Central African Republic | IBD | -0.04<br>(-0.04 to -0.04) | < 0.001 |
| Adolescence | Prevalence | Female | Central African Republic | IBD | -0.02<br>(-0.11 to 0.06) | 0.581 |
| Adolescence | Prevalence | Male | Central African Republic | IBD | -0.08<br>(-0.21 to 0.04) | 0.198 |
| Adolescence | Prevalence | Both | Central African Republic | MS | 0.04<br>(-0.09 to 0.17) | 0.552 |
| Adolescence | Prevalence | Female | Central African Republic | MS | 0.03<br>(-0.03 to 0.08) | 0.328 |
| Adolescence | Prevalence | Male | Central African Republic | MS | 0.07<br>(-0.13 to 0.27) | 0.486 |
| Adolescence | Prevalence | Both | Central African Republic | Psoriasis | -0.47<br>(-0.49 to -0.45) | < 0.001 |
| Adolescence | Prevalence | Female | Central African Republic | Psoriasis | -0.47<br>(-0.5 to -0.45) | < 0.001 |
| Adolescence | Prevalence | Male | Central African Republic | Psoriasis | -0.46<br>(-0.49 to -0.44) | < 0.001 |
| Adolescence | Prevalence | Both | Central African Republic | RHD | 0.11<br>(0.1 to 0.12) | < 0.001 |
| Adolescence | Prevalence | Female | Central African Republic | RHD | 0.17<br>(0.16 to 0.19) | < 0.001 |
| Adolescence | Prevalence | Male | Central African Republic | RHD | 0.05<br>(0.01 to 0.08) | 0.005 |
| Adolescence | Prevalence | Both | Central African Republic | RA | 0.01<br>(-0.15 to 0.18) | 0.873 |

| | | | | | | |
|---|---|---|---|---|---|---|
| Adolescence | Prevalence | Female | Central African Republic | RA | -0.05<br>(-0.12 to 0.02) | 0.196 |
| Adolescence | Prevalence | Male | Central African Republic | RA | 0.13<br>(0.01 to 0.25) | 0.034 |
| Adolescence | Prevalence | Both | Chad | AA | -0.01<br>(-0.01 to -0.01) | < 0.001 |
| Adolescence | Prevalence | Female | Chad | AA | 0<br>(0 to 0) | 1.000 |
| Adolescence | Prevalence | Male | Chad | AA | 0<br>(0 to 0) | 1.000 |
| Adolescence | Prevalence | Both | Chad | T1D | 0.02<br>(0.01 to 0.03) | < 0.001 |
| Adolescence | Prevalence | Female | Chad | T1D | 0.04<br>(0 to 0.07) | 0.036 |
| Adolescence | Prevalence | Male | Chad | T1D | 0.02<br>(0 to 0.03) | 0.033 |
| Adolescence | Prevalence | Both | Chad | IBD | 0.27<br>(0.18 to 0.36) | < 0.001 |
| Adolescence | Prevalence | Female | Chad | IBD | 0.3<br>(0.21 to 0.39) | < 0.001 |
| Adolescence | Prevalence | Male | Chad | IBD | 0.27<br>(0.23 to 0.31) | < 0.001 |
| Adolescence | Prevalence | Both | Chad | MS | 0.1<br>(0.03 to 0.17) | 0.003 |
| Adolescence | Prevalence | Female | Chad | MS | 0.11<br>(0.04 to 0.18) | 0.002 |
| Adolescence | Prevalence | Male | Chad | MS | 0.14<br>(0.01 to 0.26) | 0.032 |
| Adolescence | Prevalence | Both | Chad | Psoriasis | -0.57<br>(-0.73 to -0.4) | < 0.001 |
| Adolescence | Prevalence | Female | Chad | Psoriasis | -0.62<br>(-0.74 to -0.5) | < 0.001 |
| Adolescence | Prevalence | Male | Chad | Psoriasis | -0.47 | < 0.001 |

| | | | | | | |
|---|---|---|---|---|---|---|
| | | | | | (-0.61 to -0.34) | |
| Adolescence | Prevalence | Both | Chad | RHD | 0.09<br>(0.07 to 0.11) | < 0.001 |
| Adolescence | Prevalence | Female | Chad | RHD | 0.11<br>(0.07 to 0.15) | < 0.001 |
| Adolescence | Prevalence | Male | Chad | RHD | 0.08<br>(0.05 to 0.1) | < 0.001 |
| Adolescence | Prevalence | Both | Chad | RA | 0.28<br>(0.26 to 0.31) | < 0.001 |
| Adolescence | Prevalence | Female | Chad | RA | 0.33<br>(0.31 to 0.35) | < 0.001 |
| Adolescence | Prevalence | Male | Chad | RA | 0.22<br>(0.18 to 0.27) | < 0.001 |
| Adolescence | Prevalence | Both | Chile | AA | -0.01<br>(-0.01 to -0.01) | < 0.001 |
| Adolescence | Prevalence | Female | Chile | AA | 0<br>(0 to 0) | 1.000 |
| Adolescence | Prevalence | Male | Chile | AA | 0<br>(0 to 0) | 1.000 |
| Adolescence | Prevalence | Both | Chile | T1D | 0.98<br>(0.96 to 0.99) | < 0.001 |
| Adolescence | Prevalence | Female | Chile | T1D | 1<br>(0.97 to 1.03) | < 0.001 |
| Adolescence | Prevalence | Male | Chile | T1D | 0.95<br>(0.88 to 1.01) | < 0.001 |
| Adolescence | Prevalence | Both | Chile | IBD | 0.4<br>(0.14 to 0.67) | 0.003 |
| Adolescence | Prevalence | Female | Chile | IBD | 0.41<br>(0.2 to 0.61) | < 0.001 |
| Adolescence | Prevalence | Male | Chile | IBD | 0.43<br>(-0.02 to 0.89) | 0.064 |
| Adolescence | Prevalence | Both | Chile | MS | 0.08<br>(0.05 to 0.12) | < 0.001 |

| | | | | | | |
|---|---|---|---|---|---|---|
| Adolescence | Prevalence | Female | Chile | MS | 0.14 (0.1 to 0.18) | < 0.001 |
| Adolescence | Prevalence | Male | Chile | MS | 0.02 (-0.03 to 0.06) | 0.515 |
| Adolescence | Prevalence | Both | Chile | Psoriasis | -0.59 (-0.6 to -0.58) | < 0.001 |
| Adolescence | Prevalence | Female | Chile | Psoriasis | -0.59 (-0.6 to -0.57) | < 0.001 |
| Adolescence | Prevalence | Male | Chile | Psoriasis | -0.57 (-0.59 to -0.55) | < 0.001 |
| Adolescence | Prevalence | Both | Chile | RHD | -0.35 (-0.43 to -0.27) | < 0.001 |
| Adolescence | Prevalence | Female | Chile | RHD | -0.37 (-0.45 to -0.3) | < 0.001 |
| Adolescence | Prevalence | Male | Chile | RHD | -0.33 (-0.44 to -0.22) | < 0.001 |
| Adolescence | Prevalence | Both | Chile | RA | 0.76 (0.73 to 0.78) | < 0.001 |
| Adolescence | Prevalence | Female | Chile | RA | 0.77 (0.74 to 0.81) | < 0.001 |
| Adolescence | Prevalence | Male | Chile | RA | 0.82 (0.8 to 0.84) | < 0.001 |
| Adolescence | Prevalence | Both | China | AA | -0.05 (-0.06 to -0.05) | < 0.001 |
| Adolescence | Prevalence | Female | China | AA | 0 (0 to 0) | 1.000 |
| Adolescence | Prevalence | Male | China | AA | 0 (0 to 0) | 0.033 |
| Adolescence | Prevalence | Both | China | T1D | 1.19 (1.05 to 1.34) | < 0.001 |
| Adolescence | Prevalence | Female | China | T1D | 1.09 (0.99 to 1.19) | < 0.001 |
| Adolescence | Prevalence | Male | China | T1D | 1.3 | < 0.001 |

| | | | | | | |
|---|---|---|---|---|---|---|
| | | | | | (1.12 to 1.47) | |
| Adolescence | Prevalence | Both | China | IBD | 2.46 (2.24 to 2.67) | < 0.001 |
| Adolescence | Prevalence | Female | China | IBD | 2.33 (2.04 to 2.63) | < 0.001 |
| Adolescence | Prevalence | Male | China | IBD | 2.54 (2.39 to 2.69) | < 0.001 |
| Adolescence | Prevalence | Both | China | MS | 0.24 (0.11 to 0.37) | < 0.001 |
| Adolescence | Prevalence | Female | China | MS | 0.25 (0.12 to 0.38) | < 0.001 |
| Adolescence | Prevalence | Male | China | MS | 0.4 (0.4 to 0.4) | < 0.001 |
| Adolescence | Prevalence | Both | China | Psoriasis | -0.94 (-1 to -0.89) | < 0.001 |
| Adolescence | Prevalence | Female | China | Psoriasis | -0.85 (-0.89 to -0.82) | < 0.001 |
| Adolescence | Prevalence | Male | China | Psoriasis | -0.99 (-1.12 to -0.87) | < 0.001 |
| Adolescence | Prevalence | Both | China | RHD | -0.75 (-0.85 to -0.64) | < 0.001 |
| Adolescence | Prevalence | Female | China | RHD | -0.7 (-0.82 to -0.58) | < 0.001 |
| Adolescence | Prevalence | Male | China | RHD | -0.8 (-0.91 to -0.68) | < 0.001 |
| Adolescence | Prevalence | Both | China | RA | 0.27 (0.07 to 0.48) | 0.009 |
| Adolescence | Prevalence | Female | China | RA | 0.2 (-0.01 to 0.42) | 0.063 |
| Adolescence | Prevalence | Male | China | RA | 0.49 (0.3 to 0.68) | < 0.001 |
| Adolescence | Prevalence | Both | Colombia | AA | -0.04 (-0.04 to -0.03) | < 0.001 |

| | | | | | | |
|---|---|---|---|---|---|---|
| Adolescence | Prevalence | Female | Colombia | AA | 0 (0 to 0) | 1.000 |
| Adolescence | Prevalence | Male | Colombia | AA | 0 (0 to 0) | 1.000 |
| Adolescence | Prevalence | Both | Colombia | T1D | 0.76 (0.74 to 0.79) | < 0.001 |
| Adolescence | Prevalence | Female | Colombia | T1D | 0.74 (0.71 to 0.77) | < 0.001 |
| Adolescence | Prevalence | Male | Colombia | T1D | 0.82 (0.81 to 0.83) | < 0.001 |
| Adolescence | Prevalence | Both | Colombia | IBD | 0.08 (0.02 to 0.13) | 0.006 |
| Adolescence | Prevalence | Female | Colombia | IBD | 0.03 (-0.03 to 0.08) | 0.363 |
| Adolescence | Prevalence | Male | Colombia | IBD | 0.06 (0.02 to 0.09) | 0.001 |
| Adolescence | Prevalence | Both | Colombia | MS | 0.23 (0.16 to 0.31) | < 0.001 |
| Adolescence | Prevalence | Female | Colombia | MS | 0.43 (0.19 to 0.67) | < 0.001 |
| Adolescence | Prevalence | Male | Colombia | MS | 0.08 (-0.02 to 0.17) | 0.111 |
| Adolescence | Prevalence | Both | Colombia | Psoriasis | -0.51 (-0.58 to -0.43) | < 0.001 |
| Adolescence | Prevalence | Female | Colombia | Psoriasis | -0.5 (-0.6 to -0.41) | < 0.001 |
| Adolescence | Prevalence | Male | Colombia | Psoriasis | -0.5 (-0.55 to -0.46) | < 0.001 |
| Adolescence | Prevalence | Both | Colombia | RHD | -0.33 (-0.45 to -0.22) | < 0.001 |
| Adolescence | Prevalence | Female | Colombia | RHD | -0.36 (-0.48 to -0.23) | < 0.001 |
| Adolescence | Prevalence | Male | Colombia | RHD | -0.26 | < 0.001 |

| | | | | | | |
|---|---|---|---|---|---|---|
| | | | | | (-0.38 to -0.14) | |
| Adolescence | Prevalence | Both | Colombia | RA | 0.71<br>(0.68 to 0.75) | < 0.001 |
| Adolescence | Prevalence | Female | Colombia | RA | 0.8<br>(0.76 to 0.83) | < 0.001 |
| Adolescence | Prevalence | Male | Colombia | RA | 0.7<br>(0.68 to 0.72) | < 0.001 |
| Adolescence | Prevalence | Both | Comoros | AA | 0<br>(-0.01 to 0) | < 0.001 |
| Adolescence | Prevalence | Female | Comoros | AA | 0<br>(0 to 0) | 1.000 |
| Adolescence | Prevalence | Male | Comoros | AA | 0<br>(0 to 0) | 1.000 |
| Adolescence | Prevalence | Both | Comoros | T1D | 0.15<br>(0.12 to 0.17) | < 0.001 |
| Adolescence | Prevalence | Female | Comoros | T1D | 0.16<br>(0.15 to 0.17) | < 0.001 |
| Adolescence | Prevalence | Male | Comoros | T1D | 0.14<br>(0.1 to 0.17) | < 0.001 |
| Adolescence | Prevalence | Both | Comoros | IBD | 0.23<br>(0.14 to 0.32) | < 0.001 |
| Adolescence | Prevalence | Female | Comoros | IBD | 0.26<br>(0.17 to 0.35) | < 0.001 |
| Adolescence | Prevalence | Male | Comoros | IBD | 0.22<br>(0.16 to 0.28) | < 0.001 |
| Adolescence | Prevalence | Both | Comoros | MS | 0.03<br>(-0.04 to 0.09) | 0.403 |
| Adolescence | Prevalence | Female | Comoros | MS | 0.05<br>(0.01 to 0.08) | 0.009 |
| Adolescence | Prevalence | Male | Comoros | MS | -0.04<br>(-0.3 to 0.23) | 0.779 |
| Adolescence | Prevalence | Both | Comoros | Psoriasis | -0.32<br>(-0.34 to -0.3) | < 0.001 |

| | | | | | | |
|---|---|---|---|---|---|---|
| Adolescence | Prevalence | Female | Comoros | Psoriasis | -0.33 (-0.35 to -0.31) | < 0.001 |
| Adolescence | Prevalence | Male | Comoros | Psoriasis | -0.31 (-0.34 to -0.28) | < 0.001 |
| Adolescence | Prevalence | Both | Comoros | RHD | 0.14 (0.09 to 0.2) | < 0.001 |
| Adolescence | Prevalence | Female | Comoros | RHD | 0.14 (0.11 to 0.17) | < 0.001 |
| Adolescence | Prevalence | Male | Comoros | RHD | 0.16 (0.12 to 0.2) | < 0.001 |
| Adolescence | Prevalence | Both | Comoros | RA | 0.23 (0.19 to 0.27) | < 0.001 |
| Adolescence | Prevalence | Female | Comoros | RA | 0.2 (0.13 to 0.27) | < 0.001 |
| Adolescence | Prevalence | Male | Comoros | RA | 0.32 (0.26 to 0.38) | < 0.001 |
| Adolescence | Prevalence | Both | Congo | AA | 0 (-0.01 to 0.01) | 0.856 |
| Adolescence | Prevalence | Female | Congo | AA | 0 (0 to 0) | 1.000 |
| Adolescence | Prevalence | Male | Congo | AA | 0 (0 to 0) | 1.000 |
| Adolescence | Prevalence | Both | Congo | T1D | 0.24 (0.22 to 0.26) | < 0.001 |
| Adolescence | Prevalence | Female | Congo | T1D | 0.26 (0.25 to 0.27) | < 0.001 |
| Adolescence | Prevalence | Male | Congo | T1D | 0.22 (0.19 to 0.25) | < 0.001 |
| Adolescence | Prevalence | Both | Congo | IBD | 0.24 (0.14 to 0.34) | < 0.001 |
| Adolescence | Prevalence | Female | Congo | IBD | 0.24 (0.09 to 0.39) | 0.002 |
| Adolescence | Prevalence | Male | Congo | IBD | 0.23 | < 0.001 |

| | | | | | | |
|---|---|---|---|---|---|---|
| | | | | | (0.16 to 0.31) | |
| Adolescence | Prevalence | Both | Congo | MS | 0.04<br>(-0.22 to 0.3) | 0.776 |
| Adolescence | Prevalence | Female | Congo | MS | 0.04<br>(-0.07 to 0.15) | 0.504 |
| Adolescence | Prevalence | Male | Congo | MS | 0.11<br>(-0.18 to 0.4) | 0.448 |
| Adolescence | Prevalence | Both | Congo | Psoriasis | -0.9<br>(-0.93 to -0.87) | < 0.001 |
| Adolescence | Prevalence | Female | Congo | Psoriasis | -0.91<br>(-0.94 to -0.88) | < 0.001 |
| Adolescence | Prevalence | Male | Congo | Psoriasis | -0.89<br>(-0.93 to -0.86) | < 0.001 |
| Adolescence | Prevalence | Both | Congo | RHD | 0.04<br>(-0.26 to 0.34) | 0.799 |
| Adolescence | Prevalence | Female | Congo | RHD | 0.12<br>(-0.26 to 0.51) | 0.537 |
| Adolescence | Prevalence | Male | Congo | RHD | 0<br>(-0.32 to 0.33) | 0.978 |
| Adolescence | Prevalence | Both | Congo | RA | 0.24<br>(0.19 to 0.29) | < 0.001 |
| Adolescence | Prevalence | Female | Congo | RA | 0.23<br>(0.2 to 0.26) | < 0.001 |
| Adolescence | Prevalence | Male | Congo | RA | 0.27<br>(0.2 to 0.34) | < 0.001 |
| Adolescence | Prevalence | Both | Cook Islands | AA | 0.05<br>(0.03 to 0.07) | < 0.001 |
| Adolescence | Prevalence | Female | Cook Islands | AA | 0<br>(0 to 0) | 0.008 |
| Adolescence | Prevalence | Male | Cook Islands | AA | 0<br>(0 to 0) | 1.000 |
| Adolescence | Prevalence | Both | Cook Islands | T1D | 0.62<br>(0.61 to 0.64) | < 0.001 |

| | | | | | | |
|---|---|---|---|---|---|---|
| Adolescence | Prevalence | Female | Cook Islands | T1D | 0.61 (0.59 to 0.63) | < 0.001 |
| Adolescence | Prevalence | Male | Cook Islands | T1D | 0.64 (0.62 to 0.67) | < 0.001 |
| Adolescence | Prevalence | Both | Cook Islands | IBD | 0.26 (0.21 to 0.3) | < 0.001 |
| Adolescence | Prevalence | Female | Cook Islands | IBD | 0.26 (0.18 to 0.34) | < 0.001 |
| Adolescence | Prevalence | Male | Cook Islands | IBD | 0.23 (0.17 to 0.28) | < 0.001 |
| Adolescence | Prevalence | Both | Cook Islands | MS | 0.08 (0.05 to 0.11) | < 0.001 |
| Adolescence | Prevalence | Female | Cook Islands | MS | 0.02 (-0.07 to 0.1) | 0.712 |
| Adolescence | Prevalence | Male | Cook Islands | MS | 0.13 (-0.09 to 0.35) | 0.259 |
| Adolescence | Prevalence | Both | Cook Islands | Psoriasis | -0.45 (-0.47 to -0.44) | < 0.001 |
| Adolescence | Prevalence | Female | Cook Islands | Psoriasis | -0.47 (-0.48 to -0.46) | < 0.001 |
| Adolescence | Prevalence | Male | Cook Islands | Psoriasis | -0.51 (-0.56 to -0.46) | < 0.001 |
| Adolescence | Prevalence | Both | Cook Islands | RHD | -0.15 (-0.25 to -0.06) | 0.001 |
| Adolescence | Prevalence | Female | Cook Islands | RHD | -0.01 (-0.1 to 0.08) | 0.799 |
| Adolescence | Prevalence | Male | Cook Islands | RHD | -0.31 (-0.4 to -0.21) | < 0.001 |
| Adolescence | Prevalence | Both | Cook Islands | RA | 0.65 (0.62 to 0.68) | < 0.001 |
| Adolescence | Prevalence | Female | Cook Islands | RA | 0.65 (0.59 to 0.71) | < 0.001 |
| Adolescence | Prevalence | Male | Cook Islands | RA | 0.49 | < 0.001 |

| | | | | | | |
|---|---|---|---|---|---|---|
| | | | | | (0.42 to 0.56) | |
| Adolescence | Prevalence | Both | Costa Rica | AA | 0.01<br>(0 to 0.01) | < 0.001 |
| Adolescence | Prevalence | Female | Costa Rica | AA | 0<br>(0 to 0) | 1.000 |
| Adolescence | Prevalence | Male | Costa Rica | AA | 0<br>(0 to 0) | 1.000 |
| Adolescence | Prevalence | Both | Costa Rica | T1D | 0.79<br>(0.78 to 0.8) | < 0.001 |
| Adolescence | Prevalence | Female | Costa Rica | T1D | 0.82<br>(0.8 to 0.83) | < 0.001 |
| Adolescence | Prevalence | Male | Costa Rica | T1D | 0.75<br>(0.73 to 0.77) | < 0.001 |
| Adolescence | Prevalence | Both | Costa Rica | IBD | 0.06<br>(0.03 to 0.09) | < 0.001 |
| Adolescence | Prevalence | Female | Costa Rica | IBD | 0.04<br>(0.01 to 0.06) | 0.002 |
| Adolescence | Prevalence | Male | Costa Rica | IBD | 0.08<br>(0.06 to 0.1) | < 0.001 |
| Adolescence | Prevalence | Both | Costa Rica | MS | 0.31<br>(0.26 to 0.36) | < 0.001 |
| Adolescence | Prevalence | Female | Costa Rica | MS | 0.45<br>(0.34 to 0.57) | < 0.001 |
| Adolescence | Prevalence | Male | Costa Rica | MS | 0.16<br>(0.03 to 0.29) | 0.013 |
| Adolescence | Prevalence | Both | Costa Rica | Psoriasis | -0.47<br>(-0.5 to -0.43) | < 0.001 |
| Adolescence | Prevalence | Female | Costa Rica | Psoriasis | -0.46<br>(-0.51 to -0.41) | < 0.001 |
| Adolescence | Prevalence | Male | Costa Rica | Psoriasis | -0.46<br>(-0.5 to -0.43) | < 0.001 |
| Adolescence | Prevalence | Both | Costa Rica | RHD | -0.05<br>(-0.1 to -0.01) | 0.016 |

| | | | | | | |
|---|---|---|---|---|---|---|
| Adolescence | Prevalence | Female | Costa Rica | RHD | -0.07<br>(-0.11 to -0.03) | < 0.001 |
| Adolescence | Prevalence | Male | Costa Rica | RHD | -0.04<br>(-0.05 to -0.02) | < 0.001 |
| Adolescence | Prevalence | Both | Costa Rica | RA | 0.69<br>(0.66 to 0.72) | < 0.001 |
| Adolescence | Prevalence | Female | Costa Rica | RA | 0.71<br>(0.67 to 0.76) | < 0.001 |
| Adolescence | Prevalence | Male | Costa Rica | RA | 0.61<br>(0.58 to 0.65) | < 0.001 |
| Adolescence | Prevalence | Both | Croatia | AA | -0.01<br>(-0.01 to 0) | < 0.001 |
| Adolescence | Prevalence | Female | Croatia | AA | 0<br>(0 to 0) | 1.000 |
| Adolescence | Prevalence | Male | Croatia | AA | 0<br>(0 to 0) | 1.000 |
| Adolescence | Prevalence | Both | Croatia | T1D | 1.78<br>(1.58 to 1.99) | < 0.001 |
| Adolescence | Prevalence | Female | Croatia | T1D | 1.78<br>(1.58 to 1.98) | < 0.001 |
| Adolescence | Prevalence | Male | Croatia | T1D | 1.79<br>(1.59 to 1.99) | < 0.001 |
| Adolescence | Prevalence | Both | Croatia | IBD | 0.19<br>(0.11 to 0.27) | < 0.001 |
| Adolescence | Prevalence | Female | Croatia | IBD | 0.07<br>(-0.03 to 0.17) | 0.184 |
| Adolescence | Prevalence | Male | Croatia | IBD | 0.34<br>(0.19 to 0.5) | < 0.001 |
| Adolescence | Prevalence | Both | Croatia | MS | 0.69<br>(0.6 to 0.79) | < 0.001 |
| Adolescence | Prevalence | Female | Croatia | MS | 0.77<br>(0.74 to 0.8) | < 0.001 |
| Adolescence | Prevalence | Male | Croatia | MS | 0.54 | < 0.001 |

| | | | | | | |
|---|---|---|---|---|---|---|
| | | | | | (0.43 to 0.64) | |
| Adolescence | Prevalence | Both | Croatia | Psoriasis | -0.77 (-0.8 to -0.74) | < 0.001 |
| Adolescence | Prevalence | Female | Croatia | Psoriasis | -0.8 (-0.84 to -0.75) | < 0.001 |
| Adolescence | Prevalence | Male | Croatia | Psoriasis | -0.76 (-0.77 to -0.75) | < 0.001 |
| Adolescence | Prevalence | Both | Croatia | RHD | -0.59 (-0.77 to -0.42) | < 0.001 |
| Adolescence | Prevalence | Female | Croatia | RHD | -0.59 (-0.84 to -0.34) | < 0.001 |
| Adolescence | Prevalence | Male | Croatia | RHD | -0.71 (-0.96 to -0.45) | < 0.001 |
| Adolescence | Prevalence | Both | Croatia | RA | 0.5 (0.47 to 0.53) | < 0.001 |
| Adolescence | Prevalence | Female | Croatia | RA | 0.54 (0.5 to 0.58) | < 0.001 |
| Adolescence | Prevalence | Male | Croatia | RA | 0.47 (0.44 to 0.5) | < 0.001 |
| Adolescence | Prevalence | Both | Cuba | AA | -0.01 (-0.01 to -0.01) | < 0.001 |
| Adolescence | Prevalence | Female | Cuba | AA | 0 (0 to 0) | 1.000 |
| Adolescence | Prevalence | Male | Cuba | AA | 0 (0 to 0) | 1.000 |
| Adolescence | Prevalence | Both | Cuba | T1D | 0.89 (0.84 to 0.93) | < 0.001 |
| Adolescence | Prevalence | Female | Cuba | T1D | 0.91 (0.88 to 0.93) | < 0.001 |
| Adolescence | Prevalence | Male | Cuba | T1D | 0.86 (0.74 to 0.98) | < 0.001 |
| Adolescence | Prevalence | Both | Cuba | IBD | -0.12 (-0.18 to -0.05) | < 0.001 |

| | | | | | | |
|---|---|---|---|---|---|---|
| Adolescence | Prevalence | Female | Cuba | IBD | -0.11<br>(-0.2 to -0.02) | 0.018 |
| Adolescence | Prevalence | Male | Cuba | IBD | -0.13<br>(-0.16 to -0.1) | < 0.001 |
| Adolescence | Prevalence | Both | Cuba | MS | 0.15<br>(0.12 to 0.19) | < 0.001 |
| Adolescence | Prevalence | Female | Cuba | MS | 0.23<br>(0.16 to 0.31) | < 0.001 |
| Adolescence | Prevalence | Male | Cuba | MS | 0.18<br>(0.01 to 0.36) | 0.039 |
| Adolescence | Prevalence | Both | Cuba | Psoriasis | -0.22<br>(-0.23 to -0.21) | < 0.001 |
| Adolescence | Prevalence | Female | Cuba | Psoriasis | -0.23<br>(-0.24 to -0.22) | < 0.001 |
| Adolescence | Prevalence | Male | Cuba | Psoriasis | -0.22<br>(-0.23 to -0.2) | < 0.001 |
| Adolescence | Prevalence | Both | Cuba | RHD | 0.04<br>(0.02 to 0.05) | < 0.001 |
| Adolescence | Prevalence | Female | Cuba | RHD | 0.09<br>(0.07 to 0.1) | < 0.001 |
| Adolescence | Prevalence | Male | Cuba | RHD | -0.02<br>(-0.06 to 0.01) | 0.170 |
| Adolescence | Prevalence | Both | Cuba | RA | 0.72<br>(0.67 to 0.77) | < 0.001 |
| Adolescence | Prevalence | Female | Cuba | RA | 0.67<br>(0.59 to 0.75) | < 0.001 |
| Adolescence | Prevalence | Male | Cuba | RA | 0.86<br>(0.84 to 0.88) | < 0.001 |
| Adolescence | Prevalence | Both | Cyprus | AA | 0<br>(0 to 0) | 0.006 |
| Adolescence | Prevalence | Female | Cyprus | AA | 0<br>(0 to 0) | 1.000 |
| Adolescence | Prevalence | Male | Cyprus | AA | 0 | 0.005 |

| | | | | | | |
|---|---|---|---|---|---|---|
| | | | | | (0 to 0) | |
| Adolescence | Prevalence | Both | Cyprus | T1D | 2.37 (2.16 to 2.58) | < 0.001 |
| Adolescence | Prevalence | Female | Cyprus | T1D | 2.2 (2.04 to 2.36) | < 0.001 |
| Adolescence | Prevalence | Male | Cyprus | T1D | 2.45 (2.19 to 2.71) | < 0.001 |
| Adolescence | Prevalence | Both | Cyprus | IBD | 1.39 (1.19 to 1.58) | < 0.001 |
| Adolescence | Prevalence | Female | Cyprus | IBD | 0.82 (0.66 to 0.99) | < 0.001 |
| Adolescence | Prevalence | Male | Cyprus | IBD | 2.03 (1.92 to 2.15) | < 0.001 |
| Adolescence | Prevalence | Both | Cyprus | MS | 0.64 (0.61 to 0.67) | < 0.001 |
| Adolescence | Prevalence | Female | Cyprus | MS | 0.63 (0.55 to 0.71) | < 0.001 |
| Adolescence | Prevalence | Male | Cyprus | MS | 0.61 (0.58 to 0.65) | < 0.001 |
| Adolescence | Prevalence | Both | Cyprus | Psoriasis | -0.63 (-0.65 to -0.62) | < 0.001 |
| Adolescence | Prevalence | Female | Cyprus | Psoriasis | -0.63 (-0.64 to -0.62) | < 0.001 |
| Adolescence | Prevalence | Male | Cyprus | Psoriasis | -0.64 (-0.66 to -0.62) | < 0.001 |
| Adolescence | Prevalence | Both | Cyprus | RHD | -0.32 (-0.39 to -0.24) | < 0.001 |
| Adolescence | Prevalence | Female | Cyprus | RHD | -0.26 (-0.36 to -0.15) | < 0.001 |
| Adolescence | Prevalence | Male | Cyprus | RHD | -0.39 (-0.46 to -0.31) | < 0.001 |
| Adolescence | Prevalence | Both | Cyprus | RA | 0.51 (0.48 to 0.54) | < 0.001 |

| | | | | | | |
|---|---|---|---|---|---|---|
| Adolescence | Prevalence | Female | Cyprus | RA | 0.48<br>(0.42 to 0.53) | < 0.001 |
| Adolescence | Prevalence | Male | Cyprus | RA | 0.54<br>(0.51 to 0.57) | < 0.001 |
| Adolescence | Prevalence | Both | Democratic People's Republic of Korea | AA | -0.1<br>(-0.11 to -0.1) | < 0.001 |
| Adolescence | Prevalence | Female | Democratic People's Republic of Korea | AA | 0<br>(0 to 0) | 1.000 |
| Adolescence | Prevalence | Male | Democratic People's Republic of Korea | AA | 0<br>(0 to 0) | 1.000 |
| Adolescence | Prevalence | Both | Democratic People's Republic of Korea | T1D | 1.28<br>(1.24 to 1.32) | < 0.001 |
| Adolescence | Prevalence | Female | Democratic People's Republic of Korea | T1D | 1.27<br>(1.23 to 1.31) | < 0.001 |
| Adolescence | Prevalence | Male | Democratic People's Republic of Korea | T1D | 1.3<br>(1.27 to 1.34) | < 0.001 |
| Adolescence | Prevalence | Both | Democratic People's Republic of Korea | IBD | 0<br>(-0.05 to 0.05) | 0.980 |
| Adolescence | Prevalence | Female | Democratic People's Republic of Korea | IBD | -0.01<br>(-0.08 to 0.06) | 0.705 |
| Adolescence | Prevalence | Male | Democratic People's Republic of Korea | IBD | 0.04<br>(-0.01 to 0.09) | 0.140 |
| Adolescence | Prevalence | Both | Democratic People's Republic of Korea | MS | -0.01<br>(-0.16 to 0.15) | 0.912 |
| Adolescence | Prevalence | Female | Democratic People's Republic of Korea | MS | 0.05<br>(-0.16 to 0.25) | 0.645 |
| Adolescence | Prevalence | Male | Democratic People's Republic of Korea | MS | 0.1<br>(0.04 to 0.17) | 0.001 |
| Adolescence | Prevalence | Both | Democratic People's Republic of Korea | Psoriasis | -0.76<br>(-0.77 to -0.76) | < 0.001 |
| Adolescence | Prevalence | Female | Democratic People's Republic of Korea | Psoriasis | -0.72<br>(-0.73 to -0.7) | < 0.001 |
| Adolescence | Prevalence | Male | Democratic People's Republic of Korea | Psoriasis | -0.7 | < 0.001 |

| | | | | | | |
|---|---|---|---|---|---|---|
| | | | | | (-0.72 to -0.68) | |
| Adolescence | Prevalence | Both | Democratic People's Republic of Korea | RHD | 0<br>(-0.02 to 0.03) | 0.956 |
| Adolescence | Prevalence | Female | Democratic People's Republic of Korea | RHD | 0.05<br>(0.02 to 0.09) | 0.006 |
| Adolescence | Prevalence | Male | Democratic People's Republic of Korea | RHD | -0.01<br>(-0.05 to 0.03) | 0.625 |
| Adolescence | Prevalence | Both | Democratic People's Republic of Korea | RA | 0.52<br>(0.45 to 0.59) | < 0.001 |
| Adolescence | Prevalence | Female | Democratic People's Republic of Korea | RA | 0.49<br>(0.44 to 0.54) | < 0.001 |
| Adolescence | Prevalence | Male | Democratic People's Republic of Korea | RA | 0.77<br>(0.7 to 0.83) | < 0.001 |
| Adolescence | Prevalence | Both | Democratic Republic of the Congo | AA | 0<br>(0 to 0) | 0.007 |
| Adolescence | Prevalence | Female | Democratic Republic of the Congo | AA | 0<br>(0 to 0) | 1.000 |
| Adolescence | Prevalence | Male | Democratic Republic of the Congo | AA | 0<br>(0 to 0) | 1.000 |
| Adolescence | Prevalence | Both | Democratic Republic of the Congo | T1D | 0.16<br>(0.14 to 0.18) | < 0.001 |
| Adolescence | Prevalence | Female | Democratic Republic of the Congo | T1D | 0.2<br>(0.17 to 0.23) | < 0.001 |
| Adolescence | Prevalence | Male | Democratic Republic of the Congo | T1D | 0.14<br>(0.1 to 0.19) | < 0.001 |
| Adolescence | Prevalence | Both | Democratic Republic of the Congo | IBD | -0.09<br>(-0.19 to 0) | 0.049 |
| Adolescence | Prevalence | Female | Democratic Republic of the Congo | IBD | -0.1<br>(-0.21 to 0) | 0.049 |
| Adolescence | Prevalence | Male | Democratic Republic of the Congo | IBD | -0.07<br>(-0.19 to 0.04) | 0.221 |
| Adolescence | Prevalence | Both | Democratic Republic of the Congo | MS | 0.05<br>(-0.05 to 0.16) | 0.336 |

| | | | | | | |
|---|---|---|---|---|---|---|
| Adolescence | Prevalence | Female | Democratic Republic of the Congo | MS | 0.05<br>(0 to 0.1) | 0.066 |
| Adolescence | Prevalence | Male | Democratic Republic of the Congo | MS | 0.09<br>(-0.04 to 0.22) | 0.184 |
| Adolescence | Prevalence | Both | Democratic Republic of the Congo | Psoriasis | -0.59<br>(-0.65 to -0.53) | < 0.001 |
| Adolescence | Prevalence | Female | Democratic Republic of the Congo | Psoriasis | -0.61<br>(-0.67 to -0.54) | < 0.001 |
| Adolescence | Prevalence | Male | Democratic Republic of the Congo | Psoriasis | -0.59<br>(-0.65 to -0.52) | < 0.001 |
| Adolescence | Prevalence | Both | Democratic Republic of the Congo | RHD | 0.06<br>(0.05 to 0.07) | < 0.001 |
| Adolescence | Prevalence | Female | Democratic Republic of the Congo | RHD | 0.1<br>(0.08 to 0.11) | < 0.001 |
| Adolescence | Prevalence | Male | Democratic Republic of the Congo | RHD | 0.03<br>(0.01 to 0.04) | < 0.001 |
| Adolescence | Prevalence | Both | Democratic Republic of the Congo | RA | 0.11<br>(0.06 to 0.16) | < 0.001 |
| Adolescence | Prevalence | Female | Democratic Republic of the Congo | RA | 0.09<br>(-0.01 to 0.2) | 0.080 |
| Adolescence | Prevalence | Male | Democratic Republic of the Congo | RA | 0.09<br>(0.06 to 0.13) | < 0.001 |
| Adolescence | Prevalence | Both | Denmark | AA | 0<br>(0 to 0) | 0.118 |
| Adolescence | Prevalence | Female | Denmark | AA | 0<br>(0 to 0) | 1.000 |
| Adolescence | Prevalence | Male | Denmark | AA | 0<br>(0 to 0) | 0.005 |
| Adolescence | Prevalence | Both | Denmark | T1D | 1.55<br>(1.5 to 1.6) | < 0.001 |
| Adolescence | Prevalence | Female | Denmark | T1D | 1.54<br>(1.45 to 1.63) | < 0.001 |
| Adolescence | Prevalence | Male | Denmark | T1D | 1.57 | < 0.001 |

| | | | | | | |
|---|---|---|---|---|---|---|
| | | | | | (1.53 to 1.6) | |
| Adolescence | Prevalence | Both | Denmark | IBD | 0.15<br>(0.09 to 0.22) | < 0.001 |
| Adolescence | Prevalence | Female | Denmark | IBD | -0.24<br>(-0.29 to -0.2) | < 0.001 |
| Adolescence | Prevalence | Male | Denmark | IBD | 0.57<br>(0.5 to 0.64) | < 0.001 |
| Adolescence | Prevalence | Both | Denmark | MS | 0.26<br>(0.07 to 0.45) | 0.008 |
| Adolescence | Prevalence | Female | Denmark | MS | 0.29<br>(0.08 to 0.5) | 0.006 |
| Adolescence | Prevalence | Male | Denmark | MS | 0.15<br>(0.03 to 0.26) | 0.016 |
| Adolescence | Prevalence | Both | Denmark | Psoriasis | -0.34<br>(-0.35 to -0.32) | < 0.001 |
| Adolescence | Prevalence | Female | Denmark | Psoriasis | -0.34<br>(-0.36 to -0.32) | < 0.001 |
| Adolescence | Prevalence | Male | Denmark | Psoriasis | -0.34<br>(-0.35 to -0.32) | < 0.001 |
| Adolescence | Prevalence | Both | Denmark | RHD | -0.12<br>(-0.41 to 0.17) | 0.412 |
| Adolescence | Prevalence | Female | Denmark | RHD | -0.13<br>(-0.36 to 0.11) | 0.289 |
| Adolescence | Prevalence | Male | Denmark | RHD | -0.14<br>(-0.52 to 0.24) | 0.460 |
| Adolescence | Prevalence | Both | Denmark | RA | 0.49<br>(0.46 to 0.51) | < 0.001 |
| Adolescence | Prevalence | Female | Denmark | RA | 0.48<br>(0.44 to 0.52) | < 0.001 |
| Adolescence | Prevalence | Male | Denmark | RA | 0.52<br>(0.5 to 0.54) | < 0.001 |
| Adolescence | Prevalence | Both | Djibouti | AA | -0.02<br>(-0.03 to -0.01) | 0.003 |

| | | | | | | |
|---|---|---|---|---|---|---|
| Adolescence | Prevalence | Female | Djibouti | AA | 0<br>(0 to 0) | 1.000 |
| Adolescence | Prevalence | Male | Djibouti | AA | 0<br>(0 to 0) | 1.000 |
| Adolescence | Prevalence | Both | Djibouti | T1D | 0.13<br>(0.12 to 0.14) | < 0.001 |
| Adolescence | Prevalence | Female | Djibouti | T1D | 0.12<br>(0.11 to 0.13) | < 0.001 |
| Adolescence | Prevalence | Male | Djibouti | T1D | 0.14<br>(0.13 to 0.14) | < 0.001 |
| Adolescence | Prevalence | Both | Djibouti | IBD | 0.5<br>(0.43 to 0.56) | < 0.001 |
| Adolescence | Prevalence | Female | Djibouti | IBD | 0.48<br>(0.42 to 0.55) | < 0.001 |
| Adolescence | Prevalence | Male | Djibouti | IBD | 0.49<br>(0.41 to 0.57) | < 0.001 |
| Adolescence | Prevalence | Both | Djibouti | MS | 0.04<br>(-0.05 to 0.13) | 0.385 |
| Adolescence | Prevalence | Female | Djibouti | MS | 0.01<br>(-0.06 to 0.08) | 0.740 |
| Adolescence | Prevalence | Male | Djibouti | MS | 0.08<br>(-0.02 to 0.18) | 0.106 |
| Adolescence | Prevalence | Both | Djibouti | Psoriasis | -0.43<br>(-0.45 to -0.4) | < 0.001 |
| Adolescence | Prevalence | Female | Djibouti | Psoriasis | -0.43<br>(-0.45 to -0.42) | < 0.001 |
| Adolescence | Prevalence | Male | Djibouti | Psoriasis | -0.41<br>(-0.45 to -0.37) | < 0.001 |
| Adolescence | Prevalence | Both | Djibouti | RHD | 0.23<br>(0.22 to 0.24) | < 0.001 |
| Adolescence | Prevalence | Female | Djibouti | RHD | 0.21<br>(0.2 to 0.23) | < 0.001 |
| Adolescence | Prevalence | Male | Djibouti | RHD | 0.23 | < 0.001 |

| | | | | | | |
|---|---|---|---|---|---|---|
| | | | | | (0.2 to 0.26) | |
| Adolescence | Prevalence | Both | Djibouti | RA | 0.32<br>(0.26 to 0.39) | < 0.001 |
| Adolescence | Prevalence | Female | Djibouti | RA | 0.33<br>(0.25 to 0.42) | < 0.001 |
| Adolescence | Prevalence | Male | Djibouti | RA | 0.45<br>(0.41 to 0.48) | < 0.001 |
| Adolescence | Prevalence | Both | Dominica | AA | 0.01<br>(0 to 0.02) | 0.033 |
| Adolescence | Prevalence | Female | Dominica | AA | 0<br>(0 to 0) | 1.000 |
| Adolescence | Prevalence | Male | Dominica | AA | 0<br>(0 to 0) | 1.000 |
| Adolescence | Prevalence | Both | Dominica | T1D | 1.06<br>(1.04 to 1.07) | < 0.001 |
| Adolescence | Prevalence | Female | Dominica | T1D | 1.02<br>(1 to 1.03) | < 0.001 |
| Adolescence | Prevalence | Male | Dominica | T1D | 1.1<br>(1.04 to 1.15) | < 0.001 |
| Adolescence | Prevalence | Both | Dominica | IBD | -0.07<br>(-0.09 to -0.06) | < 0.001 |
| Adolescence | Prevalence | Female | Dominica | IBD | -0.04<br>(-0.06 to -0.01) | 0.018 |
| Adolescence | Prevalence | Male | Dominica | IBD | -0.12<br>(-0.15 to -0.09) | < 0.001 |
| Adolescence | Prevalence | Both | Dominica | MS | 0.29<br>(0.24 to 0.33) | < 0.001 |
| Adolescence | Prevalence | Female | Dominica | MS | 0.32<br>(0.21 to 0.42) | < 0.001 |
| Adolescence | Prevalence | Male | Dominica | MS | 0.29<br>(0.15 to 0.43) | < 0.001 |
| Adolescence | Prevalence | Both | Dominica | Psoriasis | -0.47<br>(-0.49 to -0.45) | < 0.001 |

| | | | | | | |
|---|---|---|---|---|---|---|
| Adolescence | Prevalence | Female | Dominica | Psoriasis | -0.48 (-0.49 to -0.46) | < 0.001 |
| Adolescence | Prevalence | Male | Dominica | Psoriasis | -0.46 (-0.49 to -0.44) | < 0.001 |
| Adolescence | Prevalence | Both | Dominica | RHD | 0 (-0.05 to 0.06) | 0.890 |
| Adolescence | Prevalence | Female | Dominica | RHD | 0.02 (-0.01 to 0.05) | 0.210 |
| Adolescence | Prevalence | Male | Dominica | RHD | -0.04 (-0.09 to 0.02) | 0.200 |
| Adolescence | Prevalence | Both | Dominica | RA | 0.52 (0.48 to 0.57) | < 0.001 |
| Adolescence | Prevalence | Female | Dominica | RA | 0.46 (0.42 to 0.5) | < 0.001 |
| Adolescence | Prevalence | Male | Dominica | RA | 0.57 (0.52 to 0.63) | < 0.001 |
| Adolescence | Prevalence | Both | Dominican Republic | AA | -0.04 (-0.05 to -0.03) | < 0.001 |
| Adolescence | Prevalence | Female | Dominican Republic | AA | 0 (0 to 0) | 1.000 |
| Adolescence | Prevalence | Male | Dominican Republic | AA | 0 (0 to 0) | 1.000 |
| Adolescence | Prevalence | Both | Dominican Republic | T1D | 0.99 (0.98 to 0.99) | < 0.001 |
| Adolescence | Prevalence | Female | Dominican Republic | T1D | 0.94 (0.92 to 0.95) | < 0.001 |
| Adolescence | Prevalence | Male | Dominican Republic | T1D | 1.06 (1.03 to 1.1) | < 0.001 |
| Adolescence | Prevalence | Both | Dominican Republic | IBD | 0.05 (-0.03 to 0.13) | 0.191 |
| Adolescence | Prevalence | Female | Dominican Republic | IBD | 0.07 (-0.01 to 0.16) | 0.103 |
| Adolescence | Prevalence | Male | Dominican Republic | IBD | 0.07 | 0.008 |

| | | | | | | |
|---|---|---|---|---|---|---|
| | | | | | (0.02 to 0.13) | |
| Adolescence | Prevalence | Both | Dominican Republic | MS | 0.27<br>(0.21 to 0.34) | < 0.001 |
| Adolescence | Prevalence | Female | Dominican Republic | MS | 0.29<br>(0.19 to 0.4) | < 0.001 |
| Adolescence | Prevalence | Male | Dominican Republic | MS | 0.25<br>(0.14 to 0.36) | < 0.001 |
| Adolescence | Prevalence | Both | Dominican Republic | Psoriasis | -0.55<br>(-0.6 to -0.51) | < 0.001 |
| Adolescence | Prevalence | Female | Dominican Republic | Psoriasis | -0.55<br>(-0.59 to -0.5) | < 0.001 |
| Adolescence | Prevalence | Male | Dominican Republic | Psoriasis | -0.55<br>(-0.6 to -0.51) | < 0.001 |
| Adolescence | Prevalence | Both | Dominican Republic | RHD | 0.01<br>(-0.01 to 0.02) | 0.546 |
| Adolescence | Prevalence | Female | Dominican Republic | RHD | 0.05<br>(0.03 to 0.06) | < 0.001 |
| Adolescence | Prevalence | Male | Dominican Republic | RHD | -0.02<br>(-0.05 to 0.01) | 0.240 |
| Adolescence | Prevalence | Both | Dominican Republic | RA | 0.8<br>(0.78 to 0.82) | < 0.001 |
| Adolescence | Prevalence | Female | Dominican Republic | RA | 0.85<br>(0.83 to 0.88) | < 0.001 |
| Adolescence | Prevalence | Male | Dominican Republic | RA | 0.87<br>(0.84 to 0.9) | < 0.001 |
| Adolescence | Prevalence | Both | Ecuador | AA | -0.02<br>(-0.02 to -0.02) | < 0.001 |
| Adolescence | Prevalence | Female | Ecuador | AA | 0<br>(0 to 0) | 1.000 |
| Adolescence | Prevalence | Male | Ecuador | AA | 0<br>(0 to 0) | 1.000 |
| Adolescence | Prevalence | Both | Ecuador | T1D | 0.68<br>(0.64 to 0.73) | < 0.001 |

| | | | | | | |
|---|---|---|---|---|---|---|
| Adolescence | Prevalence | Female | Ecuador | T1D | 0.68 (0.61 to 0.74) | < 0.001 |
| Adolescence | Prevalence | Male | Ecuador | T1D | 0.73 (0.64 to 0.81) | < 0.001 |
| Adolescence | Prevalence | Both | Ecuador | IBD | 0.91 (0.79 to 1.03) | < 0.001 |
| Adolescence | Prevalence | Female | Ecuador | IBD | 1.06 (0.98 to 1.13) | < 0.001 |
| Adolescence | Prevalence | Male | Ecuador | IBD | 0.86 (0.76 to 0.96) | < 0.001 |
| Adolescence | Prevalence | Both | Ecuador | MS | 0.3 (0.13 to 0.48) | 0.001 |
| Adolescence | Prevalence | Female | Ecuador | MS | 0.33 (0.2 to 0.46) | < 0.001 |
| Adolescence | Prevalence | Male | Ecuador | MS | 0.32 (0.08 to 0.56) | 0.008 |
| Adolescence | Prevalence | Both | Ecuador | Psoriasis | -0.65 (-0.72 to -0.58) | < 0.001 |
| Adolescence | Prevalence | Female | Ecuador | Psoriasis | -0.65 (-0.73 to -0.58) | < 0.001 |
| Adolescence | Prevalence | Male | Ecuador | Psoriasis | -0.65 (-0.71 to -0.59) | < 0.001 |
| Adolescence | Prevalence | Both | Ecuador | RHD | 0.01 (-0.01 to 0.03) | 0.238 |
| Adolescence | Prevalence | Female | Ecuador | RHD | 0.04 (-0.01 to 0.09) | 0.122 |
| Adolescence | Prevalence | Male | Ecuador | RHD | 0 (-0.02 to 0.02) | 0.929 |
| Adolescence | Prevalence | Both | Ecuador | RA | 0.69 (0.57 to 0.81) | < 0.001 |
| Adolescence | Prevalence | Female | Ecuador | RA | 0.71 (0.61 to 0.8) | < 0.001 |
| Adolescence | Prevalence | Male | Ecuador | RA | 0.72 | < 0.001 |

| | | | | | | |
|---|---|---|---|---|---|---|
| | | | | | (0.58 to 0.86) | |
| Adolescence | Prevalence | Both | Egypt | AA | 0.02<br>(0.02 to 0.02) | < 0.001 |
| Adolescence | Prevalence | Female | Egypt | AA | 0<br>(0 to 0) | 0.001 |
| Adolescence | Prevalence | Male | Egypt | AA | 0.07<br>(0.07 to 0.07) | < 0.001 |
| Adolescence | Prevalence | Both | Egypt | T1D | 2.13<br>(2.06 to 2.2) | < 0.001 |
| Adolescence | Prevalence | Female | Egypt | T1D | 2.14<br>(2.08 to 2.2) | < 0.001 |
| Adolescence | Prevalence | Male | Egypt | T1D | 2.09<br>(1.9 to 2.28) | < 0.001 |
| Adolescence | Prevalence | Both | Egypt | IBD | -0.2<br>(-0.24 to -0.17) | < 0.001 |
| Adolescence | Prevalence | Female | Egypt | IBD | -0.11<br>(-0.16 to -0.07) | < 0.001 |
| Adolescence | Prevalence | Male | Egypt | IBD | -0.3<br>(-0.36 to -0.25) | < 0.001 |
| Adolescence | Prevalence | Both | Egypt | MS | 0.58<br>(0.53 to 0.62) | < 0.001 |
| Adolescence | Prevalence | Female | Egypt | MS | 0.55<br>(0.47 to 0.64) | < 0.001 |
| Adolescence | Prevalence | Male | Egypt | MS | 0.69<br>(0.6 to 0.79) | < 0.001 |
| Adolescence | Prevalence | Both | Egypt | Psoriasis | -1.1<br>(-1.14 to -1.06) | < 0.001 |
| Adolescence | Prevalence | Female | Egypt | Psoriasis | -1.05<br>(-1.07 to -1.03) | < 0.001 |
| Adolescence | Prevalence | Male | Egypt | Psoriasis | -1.17<br>(-1.23 to -1.12) | < 0.001 |
| Adolescence | Prevalence | Both | Egypt | RHD | -0.27<br>(-0.4 to -0.14) | < 0.001 |

| | | | | | | |
|---|---|---|---|---|---|---|
| Adolescence | Prevalence | Female | Egypt | RHD | -0.28 (-0.45 to -0.1) | 0.002 |
| Adolescence | Prevalence | Male | Egypt | RHD | -0.27 (-0.35 to -0.19) | < 0.001 |
| Adolescence | Prevalence | Both | Egypt | RA | 0.69 (0.65 to 0.74) | < 0.001 |
| Adolescence | Prevalence | Female | Egypt | RA | 0.68 (0.63 to 0.72) | < 0.001 |
| Adolescence | Prevalence | Male | Egypt | RA | 0.72 (0.63 to 0.81) | < 0.001 |
| Adolescence | Prevalence | Both | El Salvador | AA | -0.02 (-0.02 to -0.02) | < 0.001 |
| Adolescence | Prevalence | Female | El Salvador | AA | 0 (0 to 0) | 1.000 |
| Adolescence | Prevalence | Male | El Salvador | AA | 0 (0 to 0) | 1.000 |
| Adolescence | Prevalence | Both | El Salvador | T1D | 0.73 (0.72 to 0.74) | < 0.001 |
| Adolescence | Prevalence | Female | El Salvador | T1D | 0.74 (0.72 to 0.76) | < 0.001 |
| Adolescence | Prevalence | Male | El Salvador | T1D | 0.74 (0.72 to 0.75) | < 0.001 |
| Adolescence | Prevalence | Both | El Salvador | IBD | 0.21 (0.16 to 0.25) | < 0.001 |
| Adolescence | Prevalence | Female | El Salvador | IBD | 0.19 (0.13 to 0.25) | < 0.001 |
| Adolescence | Prevalence | Male | El Salvador | IBD | 0.21 (0.15 to 0.26) | < 0.001 |
| Adolescence | Prevalence | Both | El Salvador | MS | 0.3 (0.24 to 0.37) | < 0.001 |
| Adolescence | Prevalence | Female | El Salvador | MS | 0.36 (0.25 to 0.48) | < 0.001 |
| Adolescence | Prevalence | Male | El Salvador | MS | 0.23 | 0.002 |

| | | | | | | |
|---|---|---|---|---|---|---|
| | | | | | (0.09 to 0.38) | |
| Adolescence | Prevalence | Both | El Salvador | Psoriasis | -0.59 (-0.62 to -0.55) | < 0.001 |
| Adolescence | Prevalence | Female | El Salvador | Psoriasis | -0.6 (-0.64 to -0.55) | < 0.001 |
| Adolescence | Prevalence | Male | El Salvador | Psoriasis | -0.58 (-0.62 to -0.54) | < 0.001 |
| Adolescence | Prevalence | Both | El Salvador | RHD | 0.05 (0.01 to 0.09) | 0.010 |
| Adolescence | Prevalence | Female | El Salvador | RHD | 0.01 (-0.05 to 0.07) | 0.685 |
| Adolescence | Prevalence | Male | El Salvador | RHD | 0.09 (0.08 to 0.1) | < 0.001 |
| Adolescence | Prevalence | Both | El Salvador | RA | 0.81 (0.78 to 0.85) | < 0.001 |
| Adolescence | Prevalence | Female | El Salvador | RA | 0.86 (0.84 to 0.88) | < 0.001 |
| Adolescence | Prevalence | Male | El Salvador | RA | 0.77 (0.71 to 0.83) | < 0.001 |
| Adolescence | Prevalence | Both | Equatorial Guinea | AA | -0.19 (-0.19 to -0.18) | < 0.001 |
| Adolescence | Prevalence | Female | Equatorial Guinea | AA | 0 (0 to 0) | 1.000 |
| Adolescence | Prevalence | Male | Equatorial Guinea | AA | 0 (0 to 0) | 1.000 |
| Adolescence | Prevalence | Both | Equatorial Guinea | T1D | 0.05 (0.04 to 0.06) | < 0.001 |
| Adolescence | Prevalence | Female | Equatorial Guinea | T1D | 0.05 (0.03 to 0.07) | < 0.001 |
| Adolescence | Prevalence | Male | Equatorial Guinea | T1D | 0.01 (0 to 0.03) | 0.092 |
| Adolescence | Prevalence | Both | Equatorial Guinea | IBD | 1.19 (1.13 to 1.25) | < 0.001 |

| | | | | | | |
|---|---|---|---|---|---|---|
| Adolescence | Prevalence | Female | Equatorial Guinea | IBD | 1.21<br>(1.07 to 1.35) | < 0.001 |
| Adolescence | Prevalence | Male | Equatorial Guinea | IBD | 1.24<br>(1.16 to 1.32) | < 0.001 |
| Adolescence | Prevalence | Both | Equatorial Guinea | MS | -0.14<br>(-0.17 to -0.11) | < 0.001 |
| Adolescence | Prevalence | Female | Equatorial Guinea | MS | 0.04<br>(-0.11 to 0.19) | 0.603 |
| Adolescence | Prevalence | Male | Equatorial Guinea | MS | 0.03<br>(-0.16 to 0.22) | 0.753 |
| Adolescence | Prevalence | Both | Equatorial Guinea | Psoriasis | -2.11<br>(-2.16 to -2.06) | < 0.001 |
| Adolescence | Prevalence | Female | Equatorial Guinea | Psoriasis | -2.12<br>(-2.17 to -2.07) | < 0.001 |
| Adolescence | Prevalence | Male | Equatorial Guinea | Psoriasis | -2.08<br>(-2.13 to -2.02) | < 0.001 |
| Adolescence | Prevalence | Both | Equatorial Guinea | RHD | -0.24<br>(-0.26 to -0.21) | < 0.001 |
| Adolescence | Prevalence | Female | Equatorial Guinea | RHD | -0.22<br>(-0.24 to -0.2) | < 0.001 |
| Adolescence | Prevalence | Male | Equatorial Guinea | RHD | -0.27<br>(-0.29 to -0.24) | < 0.001 |
| Adolescence | Prevalence | Both | Equatorial Guinea | RA | 0.53<br>(0.47 to 0.59) | < 0.001 |
| Adolescence | Prevalence | Female | Equatorial Guinea | RA | 0.78<br>(0.72 to 0.85) | < 0.001 |
| Adolescence | Prevalence | Male | Equatorial Guinea | RA | 0.81<br>(0.76 to 0.86) | < 0.001 |
| Adolescence | Prevalence | Both | Eritrea | AA | 0.01<br>(0.01 to 0.02) | < 0.001 |
| Adolescence | Prevalence | Female | Eritrea | AA | 0<br>(0 to 0) | 1.000 |
| Adolescence | Prevalence | Male | Eritrea | AA | 0 | 1.000 |

| | | | | | | |
|---|---|---|---|---|---|---|
| | | | | | (0 to 0) | |
| Adolescence | Prevalence | Both | Eritrea | T1D | 0.02<br>(0 to 0.05) | 0.082 |
| Adolescence | Prevalence | Female | Eritrea | T1D | 0.03<br>(0.01 to 0.04) | < 0.001 |
| Adolescence | Prevalence | Male | Eritrea | T1D | 0.03<br>(0 to 0.07) | 0.077 |
| Adolescence | Prevalence | Both | Eritrea | IBD | 0.31<br>(0.21 to 0.41) | < 0.001 |
| Adolescence | Prevalence | Female | Eritrea | IBD | 0.25<br>(0.09 to 0.42) | 0.002 |
| Adolescence | Prevalence | Male | Eritrea | IBD | 0.34<br>(0.26 to 0.42) | < 0.001 |
| Adolescence | Prevalence | Both | Eritrea | MS | 0.1<br>(0.04 to 0.16) | 0.001 |
| Adolescence | Prevalence | Female | Eritrea | MS | 0.02<br>(-0.07 to 0.12) | 0.616 |
| Adolescence | Prevalence | Male | Eritrea | MS | 0.09<br>(0 to 0.17) | 0.037 |
| Adolescence | Prevalence | Both | Eritrea | Psoriasis | -0.44<br>(-0.47 to -0.41) | < 0.001 |
| Adolescence | Prevalence | Female | Eritrea | Psoriasis | -0.47<br>(-0.51 to -0.42) | < 0.001 |
| Adolescence | Prevalence | Male | Eritrea | Psoriasis | -0.43<br>(-0.49 to -0.38) | < 0.001 |
| Adolescence | Prevalence | Both | Eritrea | RHD | 0.03<br>(-0.46 to 0.52) | 0.912 |
| Adolescence | Prevalence | Female | Eritrea | RHD | 0.03<br>(-0.47 to 0.53) | 0.901 |
| Adolescence | Prevalence | Male | Eritrea | RHD | -0.02<br>(-0.53 to 0.5) | 0.947 |
| Adolescence | Prevalence | Both | Eritrea | RA | 0.22<br>(0.11 to 0.33) | < 0.001 |

| | | | | | | |
|---|---|---|---|---|---|---|
| Adolescence | Prevalence | Female | Eritrea | RA | 0.17 (0.01 to 0.33) | 0.035 |
| Adolescence | Prevalence | Male | Eritrea | RA | 0.25 (0.23 to 0.27) | < 0.001 |
| Adolescence | Prevalence | Both | Estonia | AA | 0 (-0.01 to 0.01) | 0.998 |
| Adolescence | Prevalence | Female | Estonia | AA | 0 (0 to 0) | 1.000 |
| Adolescence | Prevalence | Male | Estonia | AA | 0 (0 to 0) | 1.000 |
| Adolescence | Prevalence | Both | Estonia | T1D | 0.55 (0.35 to 0.76) | < 0.001 |
| Adolescence | Prevalence | Female | Estonia | T1D | 0.37 (0.13 to 0.61) | 0.002 |
| Adolescence | Prevalence | Male | Estonia | T1D | 0.63 (0.17 to 1.09) | 0.007 |
| Adolescence | Prevalence | Both | Estonia | IBD | 0.4 (0.36 to 0.44) | < 0.001 |
| Adolescence | Prevalence | Female | Estonia | IBD | 0.36 (0.31 to 0.41) | < 0.001 |
| Adolescence | Prevalence | Male | Estonia | IBD | 0.45 (0.42 to 0.49) | < 0.001 |
| Adolescence | Prevalence | Both | Estonia | MS | 0.05 (-0.01 to 0.11) | 0.076 |
| Adolescence | Prevalence | Female | Estonia | MS | 0.1 (0.05 to 0.14) | < 0.001 |
| Adolescence | Prevalence | Male | Estonia | MS | 0.02 (-0.06 to 0.1) | 0.614 |
| Adolescence | Prevalence | Both | Estonia | Psoriasis | -0.82 (-0.84 to -0.81) | < 0.001 |
| Adolescence | Prevalence | Female | Estonia | Psoriasis | -0.82 (-0.83 to -0.8) | < 0.001 |
| Adolescence | Prevalence | Male | Estonia | Psoriasis | -0.83 | < 0.001 |

| | | | | | | |
|---|---|---|---|---|---|---|
| | | | | | (-0.84 to -0.81) | |
| Adolescence | Prevalence | Both | Estonia | RHD | -0.22<br>(-0.32 to -0.11) | < 0.001 |
| Adolescence | Prevalence | Female | Estonia | RHD | 0.03<br>(-0.04 to 0.1) | 0.432 |
| Adolescence | Prevalence | Male | Estonia | RHD | -0.42<br>(-0.51 to -0.32) | < 0.001 |
| Adolescence | Prevalence | Both | Estonia | RA | 0.5<br>(0.45 to 0.55) | < 0.001 |
| Adolescence | Prevalence | Female | Estonia | RA | 0.53<br>(0.5 to 0.56) | < 0.001 |
| Adolescence | Prevalence | Male | Estonia | RA | 0.47<br>(0.42 to 0.52) | < 0.001 |
| Adolescence | Prevalence | Both | Ethiopia | AA | 0<br>(0 to 0) | 0.815 |
| Adolescence | Prevalence | Female | Ethiopia | AA | 0<br>(0 to 0) | 1.000 |
| Adolescence | Prevalence | Male | Ethiopia | AA | 0<br>(0 to 0) | 1.000 |
| Adolescence | Prevalence | Both | Ethiopia | T1D | 0.04<br>(0.02 to 0.05) | < 0.001 |
| Adolescence | Prevalence | Female | Ethiopia | T1D | 0.03<br>(0.01 to 0.05) | 0.001 |
| Adolescence | Prevalence | Male | Ethiopia | T1D | 0.05<br>(-0.02 to 0.11) | 0.139 |
| Adolescence | Prevalence | Both | Ethiopia | IBD | 0.31<br>(0.24 to 0.37) | < 0.001 |
| Adolescence | Prevalence | Female | Ethiopia | IBD | 0.31<br>(0.24 to 0.38) | < 0.001 |
| Adolescence | Prevalence | Male | Ethiopia | IBD | 0.32<br>(0.27 to 0.38) | < 0.001 |
| Adolescence | Prevalence | Both | Ethiopia | MS | -0.08<br>(-0.08 to -0.08) | < 0.001 |

| | | | | | | |
|---|---|---|---|---|---|---|
| Adolescence | Prevalence | Female | Ethiopia | MS | -0.13<br>(-0.16 to -0.1) | < 0.001 |
| Adolescence | Prevalence | Male | Ethiopia | MS | -0.06<br>(-0.06 to -0.06) | < 0.001 |
| Adolescence | Prevalence | Both | Ethiopia | Psoriasis | -0.39<br>(-0.42 to -0.35) | < 0.001 |
| Adolescence | Prevalence | Female | Ethiopia | Psoriasis | -0.42<br>(-0.45 to -0.38) | < 0.001 |
| Adolescence | Prevalence | Male | Ethiopia | Psoriasis | -0.36<br>(-0.48 to -0.24) | < 0.001 |
| Adolescence | Prevalence | Both | Ethiopia | RHD | 0.69<br>(0.61 to 0.76) | < 0.001 |
| Adolescence | Prevalence | Female | Ethiopia | RHD | 0.62<br>(0.52 to 0.72) | < 0.001 |
| Adolescence | Prevalence | Male | Ethiopia | RHD | 0.77<br>(0.71 to 0.82) | < 0.001 |
| Adolescence | Prevalence | Both | Ethiopia | RA | -0.02<br>(-0.11 to 0.07) | 0.704 |
| Adolescence | Prevalence | Female | Ethiopia | RA | -0.09<br>(-0.21 to 0.03) | 0.138 |
| Adolescence | Prevalence | Male | Ethiopia | RA | 0.15<br>(0.11 to 0.2) | < 0.001 |
| Adolescence | Prevalence | Both | Fiji | AA | 0<br>(-0.01 to 0) | 0.180 |
| Adolescence | Prevalence | Female | Fiji | AA | 0<br>(0 to 0) | 0.008 |
| Adolescence | Prevalence | Male | Fiji | AA | 0<br>(0 to 0) | 1.000 |
| Adolescence | Prevalence | Both | Fiji | T1D | 0.68<br>(0.66 to 0.7) | < 0.001 |
| Adolescence | Prevalence | Female | Fiji | T1D | 0.64<br>(0.61 to 0.66) | < 0.001 |
| Adolescence | Prevalence | Male | Fiji | T1D | 0.72 | < 0.001 |

| | | | | | | |
|---|---|---|---|---|---|---|
| | | | | | (0.69 to 0.75) | |
| Adolescence | Prevalence | Both | Fiji | IBD | 0.2<br>(0.1 to 0.3) | < 0.001 |
| Adolescence | Prevalence | Female | Fiji | IBD | 0.25<br>(0.18 to 0.33) | < 0.001 |
| Adolescence | Prevalence | Male | Fiji | IBD | 0.28<br>(0.19 to 0.36) | < 0.001 |
| Adolescence | Prevalence | Both | Fiji | MS | 0.07<br>(0.01 to 0.12) | 0.013 |
| Adolescence | Prevalence | Female | Fiji | MS | 0.05<br>(-0.1 to 0.2) | 0.518 |
| Adolescence | Prevalence | Male | Fiji | MS | 0.09<br>(-0.17 to 0.35) | 0.489 |
| Adolescence | Prevalence | Both | Fiji | Psoriasis | -0.5<br>(-0.52 to -0.49) | < 0.001 |
| Adolescence | Prevalence | Female | Fiji | Psoriasis | -0.48<br>(-0.5 to -0.47) | < 0.001 |
| Adolescence | Prevalence | Male | Fiji | Psoriasis | -0.51<br>(-0.53 to -0.5) | < 0.001 |
| Adolescence | Prevalence | Both | Fiji | RHD | 1.84<br>(0.97 to 2.71) | < 0.001 |
| Adolescence | Prevalence | Female | Fiji | RHD | 1.91<br>(1.31 to 2.51) | < 0.001 |
| Adolescence | Prevalence | Male | Fiji | RHD | 1.74<br>(0.29 to 3.2) | 0.018 |
| Adolescence | Prevalence | Both | Fiji | RA | 0.49<br>(0.45 to 0.52) | < 0.001 |
| Adolescence | Prevalence | Female | Fiji | RA | 0.52<br>(0.45 to 0.6) | < 0.001 |
| Adolescence | Prevalence | Male | Fiji | RA | 0.35<br>(0.31 to 0.4) | < 0.001 |
| Adolescence | Prevalence | Both | Finland | AA | 0<br>(0 to 0) | 0.022 |

| | | | | | | |
|---|---|---|---|---|---|---|
| Adolescence | Prevalence | Female | Finland | AA | 0<br>(0 to 0) | < 0.001 |
| Adolescence | Prevalence | Male | Finland | AA | 0<br>(0 to 0) | 0.005 |
| Adolescence | Prevalence | Both | Finland | T1D | -1.08<br>(-1.58 to -0.58) | < 0.001 |
| Adolescence | Prevalence | Female | Finland | T1D | -1.4<br>(-2.09 to -0.71) | < 0.001 |
| Adolescence | Prevalence | Male | Finland | T1D | -0.82<br>(-1.13 to -0.51) | < 0.001 |
| Adolescence | Prevalence | Both | Finland | IBD | -0.96<br>(-1.34 to -0.58) | < 0.001 |
| Adolescence | Prevalence | Female | Finland | IBD | -1.17<br>(-1.65 to -0.7) | < 0.001 |
| Adolescence | Prevalence | Male | Finland | IBD | -0.73<br>(-1.21 to -0.25) | 0.003 |
| Adolescence | Prevalence | Both | Finland | MS | 0.2<br>(-0.02 to 0.41) | 0.077 |
| Adolescence | Prevalence | Female | Finland | MS | 0.24<br>(0.03 to 0.44) | 0.025 |
| Adolescence | Prevalence | Male | Finland | MS | 0.07<br>(-0.17 to 0.31) | 0.583 |
| Adolescence | Prevalence | Both | Finland | Psoriasis | -0.37<br>(-0.38 to -0.36) | < 0.001 |
| Adolescence | Prevalence | Female | Finland | Psoriasis | -0.37<br>(-0.38 to -0.37) | < 0.001 |
| Adolescence | Prevalence | Male | Finland | Psoriasis | -0.37<br>(-0.37 to -0.36) | < 0.001 |
| Adolescence | Prevalence | Both | Finland | RHD | -0.39<br>(-0.62 to -0.15) | 0.001 |
| Adolescence | Prevalence | Female | Finland | RHD | -0.3<br>(-0.55 to -0.05) | 0.019 |
| Adolescence | Prevalence | Male | Finland | RHD | -0.39 | < 0.001 |

| | | | | | | |
|---|---|---|---|---|---|---|
| | | | | | (-0.51 to -0.28) | |
| Adolescence | Prevalence | Both | Finland | RA | 0.45<br>(0.4 to 0.5) | < 0.001 |
| Adolescence | Prevalence | Female | Finland | RA | 0.44<br>(0.4 to 0.48) | < 0.001 |
| Adolescence | Prevalence | Male | Finland | RA | 0.49<br>(0.48 to 0.5) | < 0.001 |
| Adolescence | Prevalence | Both | France | AA | 0<br>(0 to 0) | 0.524 |
| Adolescence | Prevalence | Female | France | AA | 0<br>(0 to 0) | 1.000 |
| Adolescence | Prevalence | Male | France | AA | 0<br>(0 to 0) | 0.005 |
| Adolescence | Prevalence | Both | France | T1D | 2.94<br>(2.87 to 3.02) | < 0.001 |
| Adolescence | Prevalence | Female | France | T1D | 2.92<br>(2.82 to 3.02) | < 0.001 |
| Adolescence | Prevalence | Male | France | T1D | 2.94<br>(2.82 to 3.05) | < 0.001 |
| Adolescence | Prevalence | Both | France | IBD | 0.25<br>(0.08 to 0.43) | 0.004 |
| Adolescence | Prevalence | Female | France | IBD | 0.27<br>(0.22 to 0.32) | < 0.001 |
| Adolescence | Prevalence | Male | France | IBD | 0.18<br>(-0.01 to 0.37) | 0.060 |
| Adolescence | Prevalence | Both | France | MS | 0.42<br>(0.25 to 0.59) | < 0.001 |
| Adolescence | Prevalence | Female | France | MS | 0.43<br>(0.22 to 0.64) | < 0.001 |
| Adolescence | Prevalence | Male | France | MS | 0.33<br>(0.3 to 0.35) | < 0.001 |
| Adolescence | Prevalence | Both | France | Psoriasis | -0.42<br>(-0.43 to -0.4) | < 0.001 |

| | | | | | | |
|---|---|---|---|---|---|---|
| Adolescence | Prevalence | Female | France | Psoriasis | -0.44 (-0.46 to -0.42) | < 0.001 |
| Adolescence | Prevalence | Male | France | Psoriasis | -0.38 (-0.42 to -0.35) | < 0.001 |
| Adolescence | Prevalence | Both | France | RHD | -0.26 (-0.29 to -0.24) | < 0.001 |
| Adolescence | Prevalence | Female | France | RHD | -0.29 (-0.34 to -0.23) | < 0.001 |
| Adolescence | Prevalence | Male | France | RHD | -0.22 (-0.25 to -0.2) | < 0.001 |
| Adolescence | Prevalence | Both | France | RA | 0.46 (0.45 to 0.47) | < 0.001 |
| Adolescence | Prevalence | Female | France | RA | 0.46 (0.45 to 0.48) | < 0.001 |
| Adolescence | Prevalence | Male | France | RA | 0.46 (0.41 to 0.51) | < 0.001 |
| Adolescence | Prevalence | Both | Gabon | AA | 0.01 (0.01 to 0.02) | < 0.001 |
| Adolescence | Prevalence | Female | Gabon | AA | 0 (0 to 0) | 1.000 |
| Adolescence | Prevalence | Male | Gabon | AA | 0 (0 to 0) | 1.000 |
| Adolescence | Prevalence | Both | Gabon | T1D | 0.24 (0.23 to 0.25) | < 0.001 |
| Adolescence | Prevalence | Female | Gabon | T1D | 0.26 (0.25 to 0.28) | < 0.001 |
| Adolescence | Prevalence | Male | Gabon | T1D | 0.23 (0.22 to 0.24) | < 0.001 |
| Adolescence | Prevalence | Both | Gabon | IBD | 0.48 (0.38 to 0.58) | < 0.001 |
| Adolescence | Prevalence | Female | Gabon | IBD | 0.46 (0.41 to 0.51) | < 0.001 |
| Adolescence | Prevalence | Male | Gabon | IBD | 0.41 | < 0.001 |

| | | | | | | |
|---|---|---|---|---|---|---|
| | | | | | (0.37 to 0.46) | |
| Adolescence | Prevalence | Both | Gabon | MS | 0.1<br>(-0.09 to 0.28) | 0.301 |
| Adolescence | Prevalence | Female | Gabon | MS | 0.1<br>(0 to 0.2) | 0.058 |
| Adolescence | Prevalence | Male | Gabon | MS | 0.11<br>(-0.01 to 0.23) | 0.078 |
| Adolescence | Prevalence | Both | Gabon | Psoriasis | -1.1<br>(-1.18 to -1.02) | < 0.001 |
| Adolescence | Prevalence | Female | Gabon | Psoriasis | -1.11<br>(-1.2 to -1.02) | < 0.001 |
| Adolescence | Prevalence | Male | Gabon | Psoriasis | -1.09<br>(-1.16 to -1.02) | < 0.001 |
| Adolescence | Prevalence | Both | Gabon | RHD | 0.12<br>(0.11 to 0.14) | < 0.001 |
| Adolescence | Prevalence | Female | Gabon | RHD | 0.16<br>(0.15 to 0.17) | < 0.001 |
| Adolescence | Prevalence | Male | Gabon | RHD | 0.08<br>(0.06 to 0.1) | < 0.001 |
| Adolescence | Prevalence | Both | Gabon | RA | 0.64<br>(0.62 to 0.66) | < 0.001 |
| Adolescence | Prevalence | Female | Gabon | RA | 0.63<br>(0.62 to 0.64) | < 0.001 |
| Adolescence | Prevalence | Male | Gabon | RA | 0.63<br>(0.59 to 0.66) | < 0.001 |
| Adolescence | Prevalence | Both | Georgia | AA | -0.04<br>(-0.05 to -0.03) | < 0.001 |
| Adolescence | Prevalence | Female | Georgia | AA | 0<br>(0 to 0) | 1.000 |
| Adolescence | Prevalence | Male | Georgia | AA | 0<br>(0 to 0) | 1.000 |
| Adolescence | Prevalence | Both | Georgia | T1D | 1.3<br>(1.23 to 1.37) | < 0.001 |

| | | | | | | |
|---|---|---|---|---|---|---|
| Adolescence | Prevalence | Female | Georgia | T1D | 1.27 (1.23 to 1.31) | < 0.001 |
| Adolescence | Prevalence | Male | Georgia | T1D | 1.34 (1.29 to 1.38) | < 0.001 |
| Adolescence | Prevalence | Both | Georgia | IBD | 0 (-0.06 to 0.05) | 0.864 |
| Adolescence | Prevalence | Female | Georgia | IBD | -0.01 (-0.05 to 0.04) | 0.784 |
| Adolescence | Prevalence | Male | Georgia | IBD | 0.01 (-0.06 to 0.09) | 0.711 |
| Adolescence | Prevalence | Both | Georgia | MS | 0.32 (0.26 to 0.39) | < 0.001 |
| Adolescence | Prevalence | Female | Georgia | MS | 0.42 (0.35 to 0.48) | < 0.001 |
| Adolescence | Prevalence | Male | Georgia | MS | 0.31 (0.15 to 0.47) | < 0.001 |
| Adolescence | Prevalence | Both | Georgia | Psoriasis | -0.54 (-0.57 to -0.51) | < 0.001 |
| Adolescence | Prevalence | Female | Georgia | Psoriasis | -0.53 (-0.59 to -0.48) | < 0.001 |
| Adolescence | Prevalence | Male | Georgia | Psoriasis | -0.5 (-0.54 to -0.46) | < 0.001 |
| Adolescence | Prevalence | Both | Georgia | RHD | 0.07 (0.03 to 0.12) | 0.001 |
| Adolescence | Prevalence | Female | Georgia | RHD | 0.08 (0.02 to 0.14) | 0.008 |
| Adolescence | Prevalence | Male | Georgia | RHD | 0.1 (0.05 to 0.14) | < 0.001 |
| Adolescence | Prevalence | Both | Georgia | RA | 0.21 (0.18 to 0.25) | < 0.001 |
| Adolescence | Prevalence | Female | Georgia | RA | 0.26 (0.2 to 0.31) | < 0.001 |
| Adolescence | Prevalence | Male | Georgia | RA | 0.29 | < 0.001 |

| | | | | | | |
|---|---|---|---|---|---|---|
| | | | | | (0.26 to 0.32) | |
| Adolescence | Prevalence | Both | Germany | AA | 0<br>(0 to 0) | 1.000 |
| Adolescence | Prevalence | Female | Germany | AA | 0<br>(0 to 0) | 1.000 |
| Adolescence | Prevalence | Male | Germany | AA | 0<br>(0 to 0) | 0.005 |
| Adolescence | Prevalence | Both | Germany | T1D | 1.87<br>(1.75 to 1.99) | < 0.001 |
| Adolescence | Prevalence | Female | Germany | T1D | 1.83<br>(1.65 to 2) | < 0.001 |
| Adolescence | Prevalence | Male | Germany | T1D | 2.01<br>(1.94 to 2.07) | < 0.001 |
| Adolescence | Prevalence | Both | Germany | IBD | 1.14<br>(0.99 to 1.29) | < 0.001 |
| Adolescence | Prevalence | Female | Germany | IBD | 0.96<br>(0.85 to 1.08) | < 0.001 |
| Adolescence | Prevalence | Male | Germany | IBD | 1.35<br>(1.17 to 1.52) | < 0.001 |
| Adolescence | Prevalence | Both | Germany | MS | 0.12<br>(0.11 to 0.13) | < 0.001 |
| Adolescence | Prevalence | Female | Germany | MS | 0.14<br>(0.11 to 0.17) | < 0.001 |
| Adolescence | Prevalence | Male | Germany | MS | 0.15<br>(0.11 to 0.18) | < 0.001 |
| Adolescence | Prevalence | Both | Germany | Psoriasis | -0.27<br>(-0.35 to -0.19) | < 0.001 |
| Adolescence | Prevalence | Female | Germany | Psoriasis | -0.26<br>(-0.35 to -0.18) | < 0.001 |
| Adolescence | Prevalence | Male | Germany | Psoriasis | -0.27<br>(-0.34 to -0.19) | < 0.001 |
| Adolescence | Prevalence | Both | Germany | RHD | -0.42<br>(-0.65 to -0.19) | < 0.001 |

| | | | | | | |
|---|---|---|---|---|---|---|
| Adolescence | Prevalence | Female | Germany | RHD | -0.39<br>(-0.63 to -0.15) | 0.001 |
| Adolescence | Prevalence | Male | Germany | RHD | -0.46<br>(-0.68 to -0.23) | < 0.001 |
| Adolescence | Prevalence | Both | Germany | RA | 0.45<br>(0.44 to 0.47) | < 0.001 |
| Adolescence | Prevalence | Female | Germany | RA | 0.48<br>(0.44 to 0.52) | < 0.001 |
| Adolescence | Prevalence | Male | Germany | RA | 0.49<br>(0.48 to 0.51) | < 0.001 |
| Adolescence | Prevalence | Both | Ghana | AA | 0<br>(0 to 0) | 0.029 |
| Adolescence | Prevalence | Female | Ghana | AA | 0<br>(0 to 0) | 1.000 |
| Adolescence | Prevalence | Male | Ghana | AA | 0<br>(0 to 0) | 1.000 |
| Adolescence | Prevalence | Both | Ghana | T1D | 0.11<br>(0.1 to 0.12) | < 0.001 |
| Adolescence | Prevalence | Female | Ghana | T1D | 0.11<br>(0.08 to 0.15) | < 0.001 |
| Adolescence | Prevalence | Male | Ghana | T1D | 0.1<br>(0.09 to 0.12) | < 0.001 |
| Adolescence | Prevalence | Both | Ghana | IBD | 0.39<br>(0.32 to 0.45) | < 0.001 |
| Adolescence | Prevalence | Female | Ghana | IBD | 0.37<br>(0.28 to 0.47) | < 0.001 |
| Adolescence | Prevalence | Male | Ghana | IBD | 0.43<br>(0.4 to 0.46) | < 0.001 |
| Adolescence | Prevalence | Both | Ghana | MS | 0.14<br>(0.07 to 0.21) | < 0.001 |
| Adolescence | Prevalence | Female | Ghana | MS | 0.07<br>(0 to 0.15) | 0.047 |
| Adolescence | Prevalence | Male | Ghana | MS | 0.25 | < 0.001 |

| | | | | | | |
|---|---|---|---|---|---|---|
| | | | | | (0.13 to 0.38) | |
| Adolescence | Prevalence | Both | Ghana | Psoriasis | -0.72<br>(-0.93 to -0.52) | < 0.001 |
| Adolescence | Prevalence | Female | Ghana | Psoriasis | -0.82<br>(-1.05 to -0.58) | < 0.001 |
| Adolescence | Prevalence | Male | Ghana | Psoriasis | -0.61<br>(-0.75 to -0.47) | < 0.001 |
| Adolescence | Prevalence | Both | Ghana | RHD | 0.05<br>(0.02 to 0.09) | 0.004 |
| Adolescence | Prevalence | Female | Ghana | RHD | 0.06<br>(0.03 to 0.09) | < 0.001 |
| Adolescence | Prevalence | Male | Ghana | RHD | 0.04<br>(-0.03 to 0.11) | 0.290 |
| Adolescence | Prevalence | Both | Ghana | RA | 0.62<br>(0.57 to 0.67) | < 0.001 |
| Adolescence | Prevalence | Female | Ghana | RA | 0.66<br>(0.59 to 0.72) | < 0.001 |
| Adolescence | Prevalence | Male | Ghana | RA | 0.57<br>(0.51 to 0.63) | < 0.001 |
| Adolescence | Prevalence | Both | Greece | AA | 0<br>(0 to 0) | 0.003 |
| Adolescence | Prevalence | Female | Greece | AA | 0<br>(0 to 0) | 1.000 |
| Adolescence | Prevalence | Male | Greece | AA | 0<br>(0 to 0) | 0.005 |
| Adolescence | Prevalence | Both | Greece | T1D | 2.2<br>(2.15 to 2.25) | < 0.001 |
| Adolescence | Prevalence | Female | Greece | T1D | 2.02<br>(1.93 to 2.11) | < 0.001 |
| Adolescence | Prevalence | Male | Greece | T1D | 2.34<br>(2.25 to 2.44) | < 0.001 |
| Adolescence | Prevalence | Both | Greece | IBD | 0.13<br>(0.07 to 0.2) | < 0.001 |

| | | | | | | |
|---|---|---|---|---|---|---|
| Adolescence | Prevalence | Female | Greece | IBD | 0.2<br>(0.12 to 0.27) | < 0.001 |
| Adolescence | Prevalence | Male | Greece | IBD | 0.03<br>(-0.04 to 0.1) | 0.363 |
| Adolescence | Prevalence | Both | Greece | MS | 0.69<br>(0.67 to 0.72) | < 0.001 |
| Adolescence | Prevalence | Female | Greece | MS | 0.81<br>(0.77 to 0.84) | < 0.001 |
| Adolescence | Prevalence | Male | Greece | MS | 0.49<br>(0.43 to 0.55) | < 0.001 |
| Adolescence | Prevalence | Both | Greece | Psoriasis | -0.41<br>(-0.43 to -0.4) | < 0.001 |
| Adolescence | Prevalence | Female | Greece | Psoriasis | -0.41<br>(-0.44 to -0.39) | < 0.001 |
| Adolescence | Prevalence | Male | Greece | Psoriasis | -0.42<br>(-0.44 to -0.41) | < 0.001 |
| Adolescence | Prevalence | Both | Greece | RHD | -0.16<br>(-0.21 to -0.1) | < 0.001 |
| Adolescence | Prevalence | Female | Greece | RHD | -0.18<br>(-0.32 to -0.03) | 0.016 |
| Adolescence | Prevalence | Male | Greece | RHD | -0.15<br>(-0.19 to -0.11) | < 0.001 |
| Adolescence | Prevalence | Both | Greece | RA | 0.37<br>(0.35 to 0.4) | < 0.001 |
| Adolescence | Prevalence | Female | Greece | RA | 0.33<br>(0.3 to 0.37) | < 0.001 |
| Adolescence | Prevalence | Male | Greece | RA | 0.42<br>(0.4 to 0.44) | < 0.001 |
| Adolescence | Prevalence | Both | Greenland | AA | 0.04<br>(0.04 to 0.05) | < 0.001 |
| Adolescence | Prevalence | Female | Greenland | AA | 0<br>(0 to 0) | 1.000 |
| Adolescence | Prevalence | Male | Greenland | AA | 0 | 1.000 |

| | | | | | | |
|---|---|---|---|---|---|---|
| | | | | | (0 to 0) | |
| Adolescence | Prevalence | Both | Greenland | T1D | -0.02<br>(-0.04 to 0.01) | 0.158 |
| Adolescence | Prevalence | Female | Greenland | T1D | -0.02<br>(-0.04 to 0) | 0.068 |
| Adolescence | Prevalence | Male | Greenland | T1D | -0.01<br>(-0.03 to 0.01) | 0.263 |
| Adolescence | Prevalence | Both | Greenland | IBD | 0.34<br>(0.26 to 0.42) | < 0.001 |
| Adolescence | Prevalence | Female | Greenland | IBD | 0.27<br>(0.18 to 0.36) | < 0.001 |
| Adolescence | Prevalence | Male | Greenland | IBD | 0.38<br>(0.3 to 0.46) | < 0.001 |
| Adolescence | Prevalence | Both | Greenland | MS | 0.08<br>(0.03 to 0.13) | 0.003 |
| Adolescence | Prevalence | Female | Greenland | MS | 0.01<br>(-0.05 to 0.08) | 0.681 |
| Adolescence | Prevalence | Male | Greenland | MS | -0.03<br>(-0.15 to 0.09) | 0.612 |
| Adolescence | Prevalence | Both | Greenland | Psoriasis | -0.47<br>(-0.49 to -0.46) | < 0.001 |
| Adolescence | Prevalence | Female | Greenland | Psoriasis | -0.49<br>(-0.51 to -0.47) | < 0.001 |
| Adolescence | Prevalence | Male | Greenland | Psoriasis | -0.5<br>(-0.52 to -0.49) | < 0.001 |
| Adolescence | Prevalence | Both | Greenland | RHD | -0.47<br>(-0.5 to -0.45) | < 0.001 |
| Adolescence | Prevalence | Female | Greenland | RHD | -0.68<br>(-0.73 to -0.63) | < 0.001 |
| Adolescence | Prevalence | Male | Greenland | RHD | -0.27<br>(-0.28 to -0.25) | < 0.001 |
| Adolescence | Prevalence | Both | Greenland | RA | 1.01<br>(0.93 to 1.08) | < 0.001 |

| | | | | | | |
|---|---|---|---|---|---|---|
| Adolescence | Prevalence | Female | Greenland | RA | 0.94<br>(0.9 to 0.98) | < 0.001 |
| Adolescence | Prevalence | Male | Greenland | RA | 0.93<br>(0.86 to 1) | < 0.001 |
| Adolescence | Prevalence | Both | Grenada | AA | -0.03<br>(-0.04 to -0.02) | < 0.001 |
| Adolescence | Prevalence | Female | Grenada | AA | 0<br>(0 to 0) | 1.000 |
| Adolescence | Prevalence | Male | Grenada | AA | 0<br>(0 to 0) | 1.000 |
| Adolescence | Prevalence | Both | Grenada | T1D | 1.07<br>(1.05 to 1.08) | < 0.001 |
| Adolescence | Prevalence | Female | Grenada | T1D | 1.01<br>(0.98 to 1.04) | < 0.001 |
| Adolescence | Prevalence | Male | Grenada | T1D | 1.15<br>(1.13 to 1.17) | < 0.001 |
| Adolescence | Prevalence | Both | Grenada | IBD | 0.14<br>(0.1 to 0.19) | < 0.001 |
| Adolescence | Prevalence | Female | Grenada | IBD | 0.16<br>(0.05 to 0.26) | 0.005 |
| Adolescence | Prevalence | Male | Grenada | IBD | 0.14<br>(0.1 to 0.18) | < 0.001 |
| Adolescence | Prevalence | Both | Grenada | MS | 0.14<br>(0.07 to 0.22) | < 0.001 |
| Adolescence | Prevalence | Female | Grenada | MS | 0.18<br>(0.13 to 0.24) | < 0.001 |
| Adolescence | Prevalence | Male | Grenada | MS | 0.22<br>(0.15 to 0.3) | < 0.001 |
| Adolescence | Prevalence | Both | Grenada | Psoriasis | -0.63<br>(-0.65 to -0.61) | < 0.001 |
| Adolescence | Prevalence | Female | Grenada | Psoriasis | -0.63<br>(-0.65 to -0.6) | < 0.001 |
| Adolescence | Prevalence | Male | Grenada | Psoriasis | -0.62 | < 0.001 |

| | | | | | | |
|---|---|---|---|---|---|---|
| | | | | | (-0.64 to -0.6) | |
| Adolescence | Prevalence | Both | Grenada | RHD | -0.03<br>(-0.06 to 0.01) | 0.128 |
| Adolescence | Prevalence | Female | Grenada | RHD | 0.01<br>(-0.03 to 0.05) | 0.569 |
| Adolescence | Prevalence | Male | Grenada | RHD | -0.04<br>(-0.08 to 0) | 0.034 |
| Adolescence | Prevalence | Both | Grenada | RA | 0.68<br>(0.67 to 0.7) | < 0.001 |
| Adolescence | Prevalence | Female | Grenada | RA | 0.71<br>(0.69 to 0.73) | < 0.001 |
| Adolescence | Prevalence | Male | Grenada | RA | 0.8<br>(0.78 to 0.81) | < 0.001 |
| Adolescence | Prevalence | Both | Guam | AA | 0.01<br>(0 to 0.02) | 0.140 |
| Adolescence | Prevalence | Female | Guam | AA | 0<br>(0 to 0) | 0.008 |
| Adolescence | Prevalence | Male | Guam | AA | 0<br>(0 to 0) | 1.000 |
| Adolescence | Prevalence | Both | Guam | T1D | 0.59<br>(0.56 to 0.61) | < 0.001 |
| Adolescence | Prevalence | Female | Guam | T1D | 0.54<br>(0.52 to 0.57) | < 0.001 |
| Adolescence | Prevalence | Male | Guam | T1D | 0.63<br>(0.58 to 0.67) | < 0.001 |
| Adolescence | Prevalence | Both | Guam | IBD | 0.16<br>(0.08 to 0.24) | < 0.001 |
| Adolescence | Prevalence | Female | Guam | IBD | 0.13<br>(0.08 to 0.18) | < 0.001 |
| Adolescence | Prevalence | Male | Guam | IBD | 0.19<br>(0.13 to 0.26) | < 0.001 |
| Adolescence | Prevalence | Both | Guam | MS | 0.02<br>(-0.15 to 0.19) | 0.850 |

| | | | | | | |
|---|---|---|---|---|---|---|
| Adolescence | Prevalence | Female | Guam | MS | -0.02 (-0.14 to 0.11) | 0.814 |
| Adolescence | Prevalence | Male | Guam | MS | 0.21 (0.02 to 0.41) | 0.032 |
| Adolescence | Prevalence | Both | Guam | Psoriasis | -0.42 (-0.45 to -0.4) | < 0.001 |
| Adolescence | Prevalence | Female | Guam | Psoriasis | -0.41 (-0.46 to -0.35) | < 0.001 |
| Adolescence | Prevalence | Male | Guam | Psoriasis | -0.45 (-0.49 to -0.42) | < 0.001 |
| Adolescence | Prevalence | Both | Guam | RHD | 0.2 (0.17 to 0.22) | < 0.001 |
| Adolescence | Prevalence | Female | Guam | RHD | 0.2 (0.15 to 0.24) | < 0.001 |
| Adolescence | Prevalence | Male | Guam | RHD | 0.21 (0.2 to 0.21) | < 0.001 |
| Adolescence | Prevalence | Both | Guam | RA | 0.43 (0.42 to 0.45) | < 0.001 |
| Adolescence | Prevalence | Female | Guam | RA | 0.46 (0.41 to 0.51) | < 0.001 |
| Adolescence | Prevalence | Male | Guam | RA | 0.35 (0.33 to 0.36) | < 0.001 |
| Adolescence | Prevalence | Both | Guatemala | AA | -0.03 (-0.04 to -0.03) | < 0.001 |
| Adolescence | Prevalence | Female | Guatemala | AA | 0 (0 to 0) | 1.000 |
| Adolescence | Prevalence | Male | Guatemala | AA | 0 (0 to 0) | 1.000 |
| Adolescence | Prevalence | Both | Guatemala | T1D | 0.67 (0.65 to 0.7) | < 0.001 |
| Adolescence | Prevalence | Female | Guatemala | T1D | 0.71 (0.68 to 0.74) | < 0.001 |
| Adolescence | Prevalence | Male | Guatemala | T1D | 0.67 | < 0.001 |

| | | | | | | |
|---|---|---|---|---|---|---|
| | | | | | (0.66 to 0.67) | |
| Adolescence | Prevalence | Both | Guatemala | IBD | 0.29<br>(0.27 to 0.32) | < 0.001 |
| Adolescence | Prevalence | Female | Guatemala | IBD | 0.26<br>(0.21 to 0.31) | < 0.001 |
| Adolescence | Prevalence | Male | Guatemala | IBD | 0.26<br>(0.23 to 0.3) | < 0.001 |
| Adolescence | Prevalence | Both | Guatemala | MS | 0.31<br>(0.07 to 0.55) | 0.011 |
| Adolescence | Prevalence | Female | Guatemala | MS | 0.46<br>(0.21 to 0.71) | < 0.001 |
| Adolescence | Prevalence | Male | Guatemala | MS | 0.32<br>(0.06 to 0.57) | 0.014 |
| Adolescence | Prevalence | Both | Guatemala | Psoriasis | -0.72<br>(-0.76 to -0.68) | < 0.001 |
| Adolescence | Prevalence | Female | Guatemala | Psoriasis | -0.72<br>(-0.75 to -0.7) | < 0.001 |
| Adolescence | Prevalence | Male | Guatemala | Psoriasis | -0.71<br>(-0.74 to -0.68) | < 0.001 |
| Adolescence | Prevalence | Both | Guatemala | RHD | 0.16<br>(0.14 to 0.19) | < 0.001 |
| Adolescence | Prevalence | Female | Guatemala | RHD | 0.15<br>(0.13 to 0.16) | < 0.001 |
| Adolescence | Prevalence | Male | Guatemala | RHD | 0.19<br>(0.17 to 0.2) | < 0.001 |
| Adolescence | Prevalence | Both | Guatemala | RA | 0.74<br>(0.71 to 0.77) | < 0.001 |
| Adolescence | Prevalence | Female | Guatemala | RA | 0.82<br>(0.8 to 0.83) | < 0.001 |
| Adolescence | Prevalence | Male | Guatemala | RA | 0.69<br>(0.63 to 0.75) | < 0.001 |
| Adolescence | Prevalence | Both | Guinea | AA | 0.02<br>(0.02 to 0.03) | < 0.001 |

| | | | | | | |
|---|---|---|---|---|---|---|
| Adolescence | Prevalence | Female | Guinea | AA | 0<br>(0 to 0) | 1.000 |
| Adolescence | Prevalence | Male | Guinea | AA | 0<br>(0 to 0) | 1.000 |
| Adolescence | Prevalence | Both | Guinea | T1D | -0.01<br>(-0.03 to 0.01) | 0.196 |
| Adolescence | Prevalence | Female | Guinea | T1D | 0<br>(-0.04 to 0.04) | 0.942 |
| Adolescence | Prevalence | Male | Guinea | T1D | 0<br>(-0.04 to 0.03) | 0.872 |
| Adolescence | Prevalence | Both | Guinea | IBD | 0.23<br>(0.17 to 0.3) | < 0.001 |
| Adolescence | Prevalence | Female | Guinea | IBD | 0.2<br>(0.14 to 0.25) | < 0.001 |
| Adolescence | Prevalence | Male | Guinea | IBD | 0.24<br>(0.13 to 0.36) | < 0.001 |
| Adolescence | Prevalence | Both | Guinea | MS | 0.14<br>(0.02 to 0.26) | 0.026 |
| Adolescence | Prevalence | Female | Guinea | MS | 0.12<br>(0.04 to 0.19) | 0.002 |
| Adolescence | Prevalence | Male | Guinea | MS | 0.13<br>(-0.11 to 0.37) | 0.299 |
| Adolescence | Prevalence | Both | Guinea | Psoriasis | -0.55<br>(-0.72 to -0.38) | < 0.001 |
| Adolescence | Prevalence | Female | Guinea | Psoriasis | -0.63<br>(-0.78 to -0.48) | < 0.001 |
| Adolescence | Prevalence | Male | Guinea | Psoriasis | -0.51<br>(-0.65 to -0.37) | < 0.001 |
| Adolescence | Prevalence | Both | Guinea | RHD | 0.8<br>(0.65 to 0.95) | < 0.001 |
| Adolescence | Prevalence | Female | Guinea | RHD | 0.79<br>(0.73 to 0.84) | < 0.001 |
| Adolescence | Prevalence | Male | Guinea | RHD | 0.79 | < 0.001 |

| | | | | | | |
|---|---|---|---|---|---|---|
| | | | | | (0.64 to 0.94) | |
| Adolescence | Prevalence | Both | Guinea | RA | 0.35 (0.32 to 0.38) | < 0.001 |
| Adolescence | Prevalence | Female | Guinea | RA | 0.34 (0.3 to 0.37) | < 0.001 |
| Adolescence | Prevalence | Male | Guinea | RA | 0.27 (0.25 to 0.29) | < 0.001 |
| Adolescence | Prevalence | Both | Guinea-Bissau | AA | -0.01 (-0.01 to 0) | < 0.001 |
| Adolescence | Prevalence | Female | Guinea-Bissau | AA | 0 (0 to 0) | 1.000 |
| Adolescence | Prevalence | Male | Guinea-Bissau | AA | 0 (0 to 0) | 1.000 |
| Adolescence | Prevalence | Both | Guinea-Bissau | T1D | 0.03 (0.02 to 0.04) | < 0.001 |
| Adolescence | Prevalence | Female | Guinea-Bissau | T1D | 0.04 (0.02 to 0.06) | < 0.001 |
| Adolescence | Prevalence | Male | Guinea-Bissau | T1D | 0.01 (0 to 0.02) | 0.085 |
| Adolescence | Prevalence | Both | Guinea-Bissau | IBD | 0.28 (0.21 to 0.35) | < 0.001 |
| Adolescence | Prevalence | Female | Guinea-Bissau | IBD | 0.29 (0.2 to 0.38) | < 0.001 |
| Adolescence | Prevalence | Male | Guinea-Bissau | IBD | 0.34 (0.25 to 0.42) | < 0.001 |
| Adolescence | Prevalence | Both | Guinea-Bissau | MS | 0.11 (0.04 to 0.17) | 0.001 |
| Adolescence | Prevalence | Female | Guinea-Bissau | MS | 0.14 (0.04 to 0.24) | 0.008 |
| Adolescence | Prevalence | Male | Guinea-Bissau | MS | 0.08 (-0.03 to 0.18) | 0.171 |
| Adolescence | Prevalence | Both | Guinea-Bissau | Psoriasis | -0.64 (-0.8 to -0.49) | < 0.001 |

| | | | | | | |
|---|---|---|---|---|---|---|
| Adolescence | Prevalence | Female | Guinea-Bissau | Psoriasis | -0.73<br>(-0.87 to -0.58) | < 0.001 |
| Adolescence | Prevalence | Male | Guinea-Bissau | Psoriasis | -0.58<br>(-0.73 to -0.42) | < 0.001 |
| Adolescence | Prevalence | Both | Guinea-Bissau | RHD | 0.2<br>(0.17 to 0.24) | < 0.001 |
| Adolescence | Prevalence | Female | Guinea-Bissau | RHD | 0.23<br>(0.18 to 0.27) | < 0.001 |
| Adolescence | Prevalence | Male | Guinea-Bissau | RHD | 0.21<br>(0.18 to 0.23) | < 0.001 |
| Adolescence | Prevalence | Both | Guinea-Bissau | RA | 0.33<br>(0.24 to 0.43) | < 0.001 |
| Adolescence | Prevalence | Female | Guinea-Bissau | RA | 0.37<br>(0.26 to 0.48) | < 0.001 |
| Adolescence | Prevalence | Male | Guinea-Bissau | RA | 0.31<br>(0.24 to 0.38) | < 0.001 |
| Adolescence | Prevalence | Both | Guyana | AA | -0.03<br>(-0.04 to -0.02) | < 0.001 |
| Adolescence | Prevalence | Female | Guyana | AA | 0<br>(0 to 0) | 1.000 |
| Adolescence | Prevalence | Male | Guyana | AA | 0<br>(0 to 0) | 1.000 |
| Adolescence | Prevalence | Both | Guyana | T1D | 0.98<br>(0.96 to 1) | < 0.001 |
| Adolescence | Prevalence | Female | Guyana | T1D | 0.94<br>(0.94 to 0.95) | < 0.001 |
| Adolescence | Prevalence | Male | Guyana | T1D | 1.04<br>(1.01 to 1.08) | < 0.001 |
| Adolescence | Prevalence | Both | Guyana | IBD | 0.06<br>(0.04 to 0.08) | < 0.001 |
| Adolescence | Prevalence | Female | Guyana | IBD | 0.09<br>(0.05 to 0.12) | < 0.001 |
| Adolescence | Prevalence | Male | Guyana | IBD | 0.05 | 0.020 |

| | | | | | | |
|---|---|---|---|---|---|---|
| | | | | | (0.01 to 0.1) | |
| Adolescence | Prevalence | Both | Guyana | MS | 0.26<br>(0.08 to 0.45) | 0.005 |
| Adolescence | Prevalence | Female | Guyana | MS | 0.3<br>(0.23 to 0.38) | < 0.001 |
| Adolescence | Prevalence | Male | Guyana | MS | 0.27<br>(-0.02 to 0.56) | 0.064 |
| Adolescence | Prevalence | Both | Guyana | Psoriasis | -0.44<br>(-0.45 to -0.42) | < 0.001 |
| Adolescence | Prevalence | Female | Guyana | Psoriasis | -0.44<br>(-0.45 to -0.42) | < 0.001 |
| Adolescence | Prevalence | Male | Guyana | Psoriasis | -0.44<br>(-0.45 to -0.42) | < 0.001 |
| Adolescence | Prevalence | Both | Guyana | RHD | -0.01<br>(-0.02 to 0) | 0.047 |
| Adolescence | Prevalence | Female | Guyana | RHD | 0.03<br>(0.02 to 0.04) | < 0.001 |
| Adolescence | Prevalence | Male | Guyana | RHD | -0.05<br>(-0.07 to -0.02) | < 0.001 |
| Adolescence | Prevalence | Both | Guyana | RA | 0.65<br>(0.62 to 0.68) | < 0.001 |
| Adolescence | Prevalence | Female | Guyana | RA | 0.67<br>(0.64 to 0.7) | < 0.001 |
| Adolescence | Prevalence | Male | Guyana | RA | 0.75<br>(0.7 to 0.8) | < 0.001 |
| Adolescence | Prevalence | Both | Haiti | AA | -0.03<br>(-0.04 to -0.03) | < 0.001 |
| Adolescence | Prevalence | Female | Haiti | AA | 0<br>(0 to 0) | 1.000 |
| Adolescence | Prevalence | Male | Haiti | AA | 0<br>(0 to 0) | 1.000 |
| Adolescence | Prevalence | Both | Haiti | T1D | 1<br>(0.99 to 1) | < 0.001 |

| | | | | | | |
|---|---|---|---|---|---|---|
| Adolescence | Prevalence | Female | Haiti | T1D | 0.96<br>(0.91 to 1) | < 0.001 |
| Adolescence | Prevalence | Male | Haiti | T1D | 1.07<br>(1.06 to 1.07) | < 0.001 |
| Adolescence | Prevalence | Both | Haiti | IBD | 0.06<br>(0 to 0.13) | 0.037 |
| Adolescence | Prevalence | Female | Haiti | IBD | 0.07<br>(0.02 to 0.12) | 0.005 |
| Adolescence | Prevalence | Male | Haiti | IBD | 0.06<br>(0.03 to 0.09) | < 0.001 |
| Adolescence | Prevalence | Both | Haiti | MS | 0.21<br>(0.18 to 0.24) | < 0.001 |
| Adolescence | Prevalence | Female | Haiti | MS | 0.29<br>(0.2 to 0.38) | < 0.001 |
| Adolescence | Prevalence | Male | Haiti | MS | 0.19<br>(0.14 to 0.23) | < 0.001 |
| Adolescence | Prevalence | Both | Haiti | Psoriasis | -0.38<br>(-0.4 to -0.36) | < 0.001 |
| Adolescence | Prevalence | Female | Haiti | Psoriasis | -0.38<br>(-0.39 to -0.36) | < 0.001 |
| Adolescence | Prevalence | Male | Haiti | Psoriasis | -0.38<br>(-0.39 to -0.36) | < 0.001 |
| Adolescence | Prevalence | Both | Haiti | RHD | 0.08<br>(0.06 to 0.11) | < 0.001 |
| Adolescence | Prevalence | Female | Haiti | RHD | 0.14<br>(0.1 to 0.18) | < 0.001 |
| Adolescence | Prevalence | Male | Haiti | RHD | 0.03<br>(0 to 0.06) | 0.102 |
| Adolescence | Prevalence | Both | Haiti | RA | 0.29<br>(0.24 to 0.35) | < 0.001 |
| Adolescence | Prevalence | Female | Haiti | RA | 0.3<br>(0.23 to 0.36) | < 0.001 |
| Adolescence | Prevalence | Male | Haiti | RA | 0.52 | < 0.001 |

| | | | | | | |
|---|---|---|---|---|---|---|
| | | | | | (0.49 to 0.55) | |
| Adolescence | Prevalence | Both | Honduras | AA | 0<br>(-0.01 to 0) | < 0.001 |
| Adolescence | Prevalence | Female | Honduras | AA | 0<br>(0 to 0) | 1.000 |
| Adolescence | Prevalence | Male | Honduras | AA | 0<br>(0 to 0) | 1.000 |
| Adolescence | Prevalence | Both | Honduras | T1D | 0.74<br>(0.73 to 0.75) | < 0.001 |
| Adolescence | Prevalence | Female | Honduras | T1D | 0.75<br>(0.74 to 0.76) | < 0.001 |
| Adolescence | Prevalence | Male | Honduras | T1D | 0.73<br>(0.71 to 0.74) | < 0.001 |
| Adolescence | Prevalence | Both | Honduras | IBD | 0.13<br>(0.09 to 0.16) | < 0.001 |
| Adolescence | Prevalence | Female | Honduras | IBD | 0.14<br>(0.1 to 0.18) | < 0.001 |
| Adolescence | Prevalence | Male | Honduras | IBD | 0.11<br>(0.08 to 0.14) | < 0.001 |
| Adolescence | Prevalence | Both | Honduras | MS | 0.29<br>(0.24 to 0.34) | < 0.001 |
| Adolescence | Prevalence | Female | Honduras | MS | 0.42<br>(0.35 to 0.48) | < 0.001 |
| Adolescence | Prevalence | Male | Honduras | MS | 0.09<br>(-0.02 to 0.2) | 0.116 |
| Adolescence | Prevalence | Both | Honduras | Psoriasis | -0.52<br>(-0.57 to -0.48) | < 0.001 |
| Adolescence | Prevalence | Female | Honduras | Psoriasis | -0.53<br>(-0.57 to -0.49) | < 0.001 |
| Adolescence | Prevalence | Male | Honduras | Psoriasis | -0.51<br>(-0.56 to -0.47) | < 0.001 |
| Adolescence | Prevalence | Both | Honduras | RHD | 0.01<br>(-0.03 to 0.05) | 0.618 |

| | | | | | | |
|---|---|---|---|---|---|---|
| Adolescence | Prevalence | Female | Honduras | RHD | 0.01 (-0.05 to 0.07) | 0.654 |
| Adolescence | Prevalence | Male | Honduras | RHD | 0 (-0.01 to 0.01) | 0.482 |
| Adolescence | Prevalence | Both | Honduras | RA | 0.57 (0.51 to 0.64) | < 0.001 |
| Adolescence | Prevalence | Female | Honduras | RA | 0.56 (0.48 to 0.65) | < 0.001 |
| Adolescence | Prevalence | Male | Honduras | RA | 0.56 (0.49 to 0.64) | < 0.001 |
| Adolescence | Prevalence | Both | Hungary | AA | 0 (0 to 0) | 1.000 |
| Adolescence | Prevalence | Female | Hungary | AA | 0 (0 to 0) | 1.000 |
| Adolescence | Prevalence | Male | Hungary | AA | 0 (0 to 0) | 1.000 |
| Adolescence | Prevalence | Both | Hungary | T1D | 1.54 (1.49 to 1.6) | < 0.001 |
| Adolescence | Prevalence | Female | Hungary | T1D | 1.49 (1.41 to 1.56) | < 0.001 |
| Adolescence | Prevalence | Male | Hungary | T1D | 1.61 (1.52 to 1.71) | < 0.001 |
| Adolescence | Prevalence | Both | Hungary | IBD | 1.2 (1.01 to 1.4) | < 0.001 |
| Adolescence | Prevalence | Female | Hungary | IBD | 1.27 (1.13 to 1.42) | < 0.001 |
| Adolescence | Prevalence | Male | Hungary | IBD | 1.16 (0.91 to 1.4) | < 0.001 |
| Adolescence | Prevalence | Both | Hungary | MS | 0.65 (0.59 to 0.72) | < 0.001 |
| Adolescence | Prevalence | Female | Hungary | MS | 0.73 (0.63 to 0.83) | < 0.001 |
| Adolescence | Prevalence | Male | Hungary | MS | 0.61 | < 0.001 |

| | | | | | | |
|---|---|---|---|---|---|---|
| | | | | | (0.58 to 0.64) | |
| Adolescence | Prevalence | Both | Hungary | Psoriasis | -0.85<br>(-0.89 to -0.82) | < 0.001 |
| Adolescence | Prevalence | Female | Hungary | Psoriasis | -0.87<br>(-0.91 to -0.83) | < 0.001 |
| Adolescence | Prevalence | Male | Hungary | Psoriasis | -0.82<br>(-0.86 to -0.78) | < 0.001 |
| Adolescence | Prevalence | Both | Hungary | RHD | -0.27<br>(-0.34 to -0.2) | < 0.001 |
| Adolescence | Prevalence | Female | Hungary | RHD | -0.07<br>(-0.13 to -0.01) | 0.025 |
| Adolescence | Prevalence | Male | Hungary | RHD | -0.41<br>(-0.53 to -0.29) | < 0.001 |
| Adolescence | Prevalence | Both | Hungary | RA | 0.47<br>(0.44 to 0.5) | < 0.001 |
| Adolescence | Prevalence | Female | Hungary | RA | 0.46<br>(0.42 to 0.5) | < 0.001 |
| Adolescence | Prevalence | Male | Hungary | RA | 0.48<br>(0.46 to 0.51) | < 0.001 |
| Adolescence | Prevalence | Both | Iceland | AA | 0<br>(0 to 0) | 0.146 |
| Adolescence | Prevalence | Female | Iceland | AA | 0<br>(0 to 0) | 1.000 |
| Adolescence | Prevalence | Male | Iceland | AA | 0<br>(0 to 0) | 0.005 |
| Adolescence | Prevalence | Both | Iceland | T1D | 1.6<br>(1.5 to 1.69) | < 0.001 |
| Adolescence | Prevalence | Female | Iceland | T1D | 1.58<br>(1.49 to 1.67) | < 0.001 |
| Adolescence | Prevalence | Male | Iceland | T1D | 1.67<br>(1.59 to 1.75) | < 0.001 |
| Adolescence | Prevalence | Both | Iceland | IBD | 0.86<br>(0.57 to 1.16) | < 0.001 |

| | | | | | | |
|---|---|---|---|---|---|---|
| Adolescence | Prevalence | Female | Iceland | IBD | 0.33<br>(-0.04 to 0.71) | 0.079 |
| Adolescence | Prevalence | Male | Iceland | IBD | 1.41<br>(1.21 to 1.62) | < 0.001 |
| Adolescence | Prevalence | Both | Iceland | MS | 0.73<br>(0.66 to 0.79) | < 0.001 |
| Adolescence | Prevalence | Female | Iceland | MS | 0.8<br>(0.73 to 0.88) | < 0.001 |
| Adolescence | Prevalence | Male | Iceland | MS | 0.57<br>(0.5 to 0.65) | < 0.001 |
| Adolescence | Prevalence | Both | Iceland | Psoriasis | -0.42<br>(-0.43 to -0.41) | < 0.001 |
| Adolescence | Prevalence | Female | Iceland | Psoriasis | -0.43<br>(-0.45 to -0.41) | < 0.001 |
| Adolescence | Prevalence | Male | Iceland | Psoriasis | -0.42<br>(-0.43 to -0.41) | < 0.001 |
| Adolescence | Prevalence | Both | Iceland | RHD | 0.18<br>(0.11 to 0.26) | < 0.001 |
| Adolescence | Prevalence | Female | Iceland | RHD | 0.18<br>(0.1 to 0.25) | < 0.001 |
| Adolescence | Prevalence | Male | Iceland | RHD | 0.21<br>(0.17 to 0.26) | < 0.001 |
| Adolescence | Prevalence | Both | Iceland | RA | 0.41<br>(0.4 to 0.43) | < 0.001 |
| Adolescence | Prevalence | Female | Iceland | RA | 0.39<br>(0.38 to 0.39) | < 0.001 |
| Adolescence | Prevalence | Male | Iceland | RA | 0.43<br>(0.4 to 0.46) | < 0.001 |
| Adolescence | Prevalence | Both | India | AA | 0<br>(-0.01 to 0) | 0.028 |
| Adolescence | Prevalence | Female | India | AA | 0<br>(0 to 0) | 1.000 |
| Adolescence | Prevalence | Male | India | AA | 0 | 1.000 |

| | | | | | | |
|---|---|---|---|---|---|---|
| | | | | | (0 to 0) | |
| Adolescence | Prevalence | Both | India | T1D | 0.14<br>(0.12 to 0.16) | < 0.001 |
| Adolescence | Prevalence | Female | India | T1D | 0.14<br>(0.1 to 0.17) | < 0.001 |
| Adolescence | Prevalence | Male | India | T1D | 0.15<br>(0.09 to 0.21) | < 0.001 |
| Adolescence | Prevalence | Both | India | IBD | -0.33<br>(-0.49 to -0.17) | < 0.001 |
| Adolescence | Prevalence | Female | India | IBD | -0.24<br>(-0.32 to -0.17) | < 0.001 |
| Adolescence | Prevalence | Male | India | IBD | -0.39<br>(-0.51 to -0.26) | < 0.001 |
| Adolescence | Prevalence | Both | India | MS | 0.26<br>(0.19 to 0.32) | < 0.001 |
| Adolescence | Prevalence | Female | India | MS | 0.23<br>(0.2 to 0.27) | < 0.001 |
| Adolescence | Prevalence | Male | India | MS | 0.25<br>(0.16 to 0.35) | < 0.001 |
| Adolescence | Prevalence | Both | India | Psoriasis | -0.03<br>(-0.06 to 0.01) | 0.121 |
| Adolescence | Prevalence | Female | India | Psoriasis | -0.02<br>(-0.06 to 0.01) | 0.199 |
| Adolescence | Prevalence | Male | India | Psoriasis | -0.03<br>(-0.06 to 0.01) | 0.103 |
| Adolescence | Prevalence | Both | India | RHD | 0.38<br>(-0.01 to 0.77) | 0.054 |
| Adolescence | Prevalence | Female | India | RHD | 0.39<br>(-0.04 to 0.81) | 0.074 |
| Adolescence | Prevalence | Male | India | RHD | 0.32<br>(-0.05 to 0.69) | 0.094 |
| Adolescence | Prevalence | Both | India | RA | 0.48<br>(0.44 to 0.52) | < 0.001 |

| | | | | | | |
|---|---|---|---|---|---|---|
| Adolescence | Prevalence | Female | India | RA | 0.49 (0.44 to 0.53) | < 0.001 |
| Adolescence | Prevalence | Male | India | RA | 0.52 (0.49 to 0.55) | < 0.001 |
| Adolescence | Prevalence | Both | Indonesia | AA | -0.01 (-0.01 to -0.01) | < 0.001 |
| Adolescence | Prevalence | Female | Indonesia | AA | 0 (0 to 0) | 1.000 |
| Adolescence | Prevalence | Male | Indonesia | AA | 0 (0 to 0) | 1.000 |
| Adolescence | Prevalence | Both | Indonesia | T1D | 0.74 (0.68 to 0.79) | < 0.001 |
| Adolescence | Prevalence | Female | Indonesia | T1D | 0.69 (0.62 to 0.76) | < 0.001 |
| Adolescence | Prevalence | Male | Indonesia | T1D | 0.76 (0.67 to 0.86) | < 0.001 |
| Adolescence | Prevalence | Both | Indonesia | IBD | 0.62 (0.55 to 0.69) | < 0.001 |
| Adolescence | Prevalence | Female | Indonesia | IBD | 0.74 (0.66 to 0.82) | < 0.001 |
| Adolescence | Prevalence | Male | Indonesia | IBD | 0.49 (0.43 to 0.56) | < 0.001 |
| Adolescence | Prevalence | Both | Indonesia | MS | -0.06 (-0.15 to 0.04) | 0.253 |
| Adolescence | Prevalence | Female | Indonesia | MS | 0 (-0.05 to 0.04) | 0.872 |
| Adolescence | Prevalence | Male | Indonesia | MS | -0.02 (-0.15 to 0.11) | 0.750 |
| Adolescence | Prevalence | Both | Indonesia | Psoriasis | -0.35 (-0.47 to -0.24) | < 0.001 |
| Adolescence | Prevalence | Female | Indonesia | Psoriasis | -0.32 (-0.39 to -0.25) | < 0.001 |
| Adolescence | Prevalence | Male | Indonesia | Psoriasis | -0.39 | < 0.001 |

| | | | | | | |
|---|---|---|---|---|---|---|
| | | | | | (-0.59 to -0.2) | |
| Adolescence | Prevalence | Both | Indonesia | RHD | 0.57<br>(0.55 to 0.59) | < 0.001 |
| Adolescence | Prevalence | Female | Indonesia | RHD | 0.57<br>(0.55 to 0.6) | < 0.001 |
| Adolescence | Prevalence | Male | Indonesia | RHD | 0.59<br>(0.58 to 0.6) | < 0.001 |
| Adolescence | Prevalence | Both | Indonesia | RA | 0.23<br>(0.22 to 0.25) | < 0.001 |
| Adolescence | Prevalence | Female | Indonesia | RA | 0.19<br>(0.17 to 0.21) | < 0.001 |
| Adolescence | Prevalence | Male | Indonesia | RA | 0.31<br>(0.29 to 0.34) | < 0.001 |
| Adolescence | Prevalence | Both | Iraq | AA | 0<br>(-0.01 to 0.01) | 0.602 |
| Adolescence | Prevalence | Female | Iraq | AA | 0<br>(0 to 0) | 1.000 |
| Adolescence | Prevalence | Male | Iraq | AA | 0<br>(0 to 0) | 1.000 |
| Adolescence | Prevalence | Both | Iraq | T1D | 2.14<br>(2.11 to 2.18) | < 0.001 |
| Adolescence | Prevalence | Female | Iraq | T1D | 2.14<br>(2.06 to 2.22) | < 0.001 |
| Adolescence | Prevalence | Male | Iraq | T1D | 2.16<br>(2.11 to 2.2) | < 0.001 |
| Adolescence | Prevalence | Both | Iraq | IBD | 0.69<br>(0.67 to 0.72) | < 0.001 |
| Adolescence | Prevalence | Female | Iraq | IBD | 0.63<br>(0.57 to 0.68) | < 0.001 |
| Adolescence | Prevalence | Male | Iraq | IBD | 0.76<br>(0.73 to 0.8) | < 0.001 |
| Adolescence | Prevalence | Both | Iraq | MS | 0.36<br>(0.29 to 0.42) | < 0.001 |

| | | | | | | |
|---|---|---|---|---|---|---|
| Adolescence | Prevalence | Female | Iraq | MS | 0.33<br>(0.28 to 0.39) | < 0.001 |
| Adolescence | Prevalence | Male | Iraq | MS | 0.4<br>(0.32 to 0.48) | < 0.001 |
| Adolescence | Prevalence | Both | Iraq | Psoriasis | -1.3<br>(-1.47 to -1.13) | < 0.001 |
| Adolescence | Prevalence | Female | Iraq | Psoriasis | -1.34<br>(-1.51 to -1.18) | < 0.001 |
| Adolescence | Prevalence | Male | Iraq | Psoriasis | -1.31<br>(-1.46 to -1.16) | < 0.001 |
| Adolescence | Prevalence | Both | Iraq | RHD | 0.08<br>(0.05 to 0.1) | < 0.001 |
| Adolescence | Prevalence | Female | Iraq | RHD | 0.08<br>(0.07 to 0.09) | < 0.001 |
| Adolescence | Prevalence | Male | Iraq | RHD | 0.06<br>(0.04 to 0.09) | < 0.001 |
| Adolescence | Prevalence | Both | Iraq | RA | 0.41<br>(0.32 to 0.51) | < 0.001 |
| Adolescence | Prevalence | Female | Iraq | RA | 0.41<br>(0.36 to 0.46) | < 0.001 |
| Adolescence | Prevalence | Male | Iraq | RA | 0.49<br>(0.38 to 0.6) | < 0.001 |
| Adolescence | Prevalence | Both | Ireland | AA | 0<br>(0 to 0) | 0.084 |
| Adolescence | Prevalence | Female | Ireland | AA | 0<br>(0 to 0) | 1.000 |
| Adolescence | Prevalence | Male | Ireland | AA | 0<br>(0 to 0) | 0.005 |
| Adolescence | Prevalence | Both | Ireland | T1D | 2.16<br>(1.93 to 2.39) | < 0.001 |
| Adolescence | Prevalence | Female | Ireland | T1D | 2.19<br>(1.98 to 2.4) | < 0.001 |
| Adolescence | Prevalence | Male | Ireland | T1D | 2.23 | < 0.001 |

| | | | | | | |
|---|---|---|---|---|---|---|
| | | | | | (1.97 to 2.48) | |
| Adolescence | Prevalence | Both | Ireland | IBD | 0.06<br>(-0.14 to 0.26) | 0.556 |
| Adolescence | Prevalence | Female | Ireland | IBD | 0<br>(-0.17 to 0.17) | 0.985 |
| Adolescence | Prevalence | Male | Ireland | IBD | 0.21<br>(0.06 to 0.37) | 0.008 |
| Adolescence | Prevalence | Both | Ireland | MS | 0.35<br>(0.28 to 0.42) | < 0.001 |
| Adolescence | Prevalence | Female | Ireland | MS | 0.3<br>(0.18 to 0.43) | < 0.001 |
| Adolescence | Prevalence | Male | Ireland | MS | 0.49<br>(0.27 to 0.7) | < 0.001 |
| Adolescence | Prevalence | Both | Ireland | Psoriasis | -0.52<br>(-0.53 to -0.51) | < 0.001 |
| Adolescence | Prevalence | Female | Ireland | Psoriasis | -0.52<br>(-0.52 to -0.51) | < 0.001 |
| Adolescence | Prevalence | Male | Ireland | Psoriasis | -0.52<br>(-0.53 to -0.51) | < 0.001 |
| Adolescence | Prevalence | Both | Ireland | RHD | -0.17<br>(-0.27 to -0.08) | < 0.001 |
| Adolescence | Prevalence | Female | Ireland | RHD | -0.19<br>(-0.28 to -0.09) | < 0.001 |
| Adolescence | Prevalence | Male | Ireland | RHD | -0.15<br>(-0.2 to -0.1) | < 0.001 |
| Adolescence | Prevalence | Both | Ireland | RA | 0.49<br>(0.49 to 0.5) | < 0.001 |
| Adolescence | Prevalence | Female | Ireland | RA | 0.47<br>(0.46 to 0.48) | < 0.001 |
| Adolescence | Prevalence | Male | Ireland | RA | 0.53<br>(0.52 to 0.55) | < 0.001 |
| Adolescence | Prevalence | Both | Israel | AA | 0<br>(0 to 0) | 1.000 |

| | | | | | | |
|---|---|---|---|---|---|---|
| Adolescence | Prevalence | Female | Israel | AA | 0<br>(0 to 0) | 1.000 |
| Adolescence | Prevalence | Male | Israel | AA | 0<br>(0 to 0) | 0.005 |
| Adolescence | Prevalence | Both | Israel | T1D | -0.55<br>(-0.86 to -0.24) | < 0.001 |
| Adolescence | Prevalence | Female | Israel | T1D | -0.38<br>(-0.61 to -0.14) | 0.001 |
| Adolescence | Prevalence | Male | Israel | T1D | -0.63<br>(-1.01 to -0.24) | 0.002 |
| Adolescence | Prevalence | Both | Israel | IBD | -0.22<br>(-0.37 to -0.07) | 0.005 |
| Adolescence | Prevalence | Female | Israel | IBD | -0.32<br>(-0.47 to -0.17) | < 0.001 |
| Adolescence | Prevalence | Male | Israel | IBD | -0.07<br>(-0.27 to 0.13) | 0.483 |
| Adolescence | Prevalence | Both | Israel | MS | 0.2<br>(0.18 to 0.22) | < 0.001 |
| Adolescence | Prevalence | Female | Israel | MS | 0.22<br>(0.2 to 0.23) | < 0.001 |
| Adolescence | Prevalence | Male | Israel | MS | 0.16<br>(0.12 to 0.21) | < 0.001 |
| Adolescence | Prevalence | Both | Israel | Psoriasis | -0.34<br>(-0.35 to -0.34) | < 0.001 |
| Adolescence | Prevalence | Female | Israel | Psoriasis | -0.34<br>(-0.34 to -0.34) | < 0.001 |
| Adolescence | Prevalence | Male | Israel | Psoriasis | -0.35<br>(-0.36 to -0.35) | < 0.001 |
| Adolescence | Prevalence | Both | Israel | RHD | -0.4<br>(-0.44 to -0.36) | < 0.001 |
| Adolescence | Prevalence | Female | Israel | RHD | -0.44<br>(-0.51 to -0.37) | < 0.001 |
| Adolescence | Prevalence | Male | Israel | RHD | -0.33 | < 0.001 |

| | | | | | | |
|---|---|---|---|---|---|---|
| | | | | | (-0.4 to -0.26) | |
| Adolescence | Prevalence | Both | Israel | RA | 0.51<br>(0.49 to 0.52) | < 0.001 |
| Adolescence | Prevalence | Female | Israel | RA | 0.51<br>(0.48 to 0.54) | < 0.001 |
| Adolescence | Prevalence | Male | Israel | RA | 0.49<br>(0.48 to 0.51) | < 0.001 |
| Adolescence | Prevalence | Both | Italy | AA | 0<br>(0 to 0) | < 0.001 |
| Adolescence | Prevalence | Female | Italy | AA | 0<br>(0 to 0) | < 0.001 |
| Adolescence | Prevalence | Male | Italy | AA | 0<br>(0 to 0) | < 0.001 |
| Adolescence | Prevalence | Both | Italy | T1D | 0.46<br>(0.25 to 0.67) | < 0.001 |
| Adolescence | Prevalence | Female | Italy | T1D | 0.49<br>(0.27 to 0.7) | < 0.001 |
| Adolescence | Prevalence | Male | Italy | T1D | 0.46<br>(0.25 to 0.68) | < 0.001 |
| Adolescence | Prevalence | Both | Italy | IBD | -0.03<br>(-0.09 to 0.04) | 0.417 |
| Adolescence | Prevalence | Female | Italy | IBD | -0.21<br>(-0.29 to -0.13) | < 0.001 |
| Adolescence | Prevalence | Male | Italy | IBD | 0.14<br>(0.09 to 0.2) | < 0.001 |
| Adolescence | Prevalence | Both | Italy | MS | 1.5<br>(1.47 to 1.53) | < 0.001 |
| Adolescence | Prevalence | Female | Italy | MS | 1.32<br>(1.24 to 1.39) | < 0.001 |
| Adolescence | Prevalence | Male | Italy | MS | 1.82<br>(1.74 to 1.9) | < 0.001 |
| Adolescence | Prevalence | Both | Italy | Psoriasis | -0.3<br>(-0.39 to -0.21) | < 0.001 |

| | | | | | | |
|---|---|---|---|---|---|---|
| Adolescence | Prevalence | Female | Italy | Psoriasis | -0.28 (-0.35 to -0.22) | < 0.001 |
| Adolescence | Prevalence | Male | Italy | Psoriasis | -0.3 (-0.41 to -0.2) | < 0.001 |
| Adolescence | Prevalence | Both | Italy | RHD | -1.44 (-1.92 to -0.97) | < 0.001 |
| Adolescence | Prevalence | Female | Italy | RHD | -1.37 (-1.88 to -0.87) | < 0.001 |
| Adolescence | Prevalence | Male | Italy | RHD | -1.52 (-1.95 to -1.09) | < 0.001 |
| Adolescence | Prevalence | Both | Italy | RA | -0.01 (-0.03 to 0.01) | 0.350 |
| Adolescence | Prevalence | Female | Italy | RA | 0 (0 to 0.01) | 0.402 |
| Adolescence | Prevalence | Male | Italy | RA | 0.03 (-0.01 to 0.07) | 0.096 |
| Adolescence | Prevalence | Both | Jamaica | AA | -0.02 (-0.03 to -0.02) | < 0.001 |
| Adolescence | Prevalence | Female | Jamaica | AA | 0 (0 to 0) | < 0.001 |
| Adolescence | Prevalence | Male | Jamaica | AA | 0 (0 to 0) | 1.000 |
| Adolescence | Prevalence | Both | Jamaica | T1D | 1.13 (1.09 to 1.16) | < 0.001 |
| Adolescence | Prevalence | Female | Jamaica | T1D | 1.05 (1 to 1.11) | < 0.001 |
| Adolescence | Prevalence | Male | Jamaica | T1D | 1.21 (1.17 to 1.24) | < 0.001 |
| Adolescence | Prevalence | Both | Jamaica | IBD | 0.03 (0 to 0.07) | 0.073 |
| Adolescence | Prevalence | Female | Jamaica | IBD | 0.06 (0 to 0.13) | 0.043 |
| Adolescence | Prevalence | Male | Jamaica | IBD | 0.03 | 0.003 |

| | | | | | | |
|---|---|---|---|---|---|---|
| | | | | | (0.01 to 0.05) | |
| Adolescence | Prevalence | Both | Jamaica | MS | 0.24<br>(0.19 to 0.28) | < 0.001 |
| Adolescence | Prevalence | Female | Jamaica | MS | 0.25<br>(0.15 to 0.36) | < 0.001 |
| Adolescence | Prevalence | Male | Jamaica | MS | 0.2<br>(-0.01 to 0.41) | 0.058 |
| Adolescence | Prevalence | Both | Jamaica | Psoriasis | -0.47<br>(-0.49 to -0.45) | < 0.001 |
| Adolescence | Prevalence | Female | Jamaica | Psoriasis | -0.48<br>(-0.49 to -0.46) | < 0.001 |
| Adolescence | Prevalence | Male | Jamaica | Psoriasis | -0.46<br>(-0.49 to -0.44) | < 0.001 |
| Adolescence | Prevalence | Both | Jamaica | RHD | 0.17<br>(0.15 to 0.19) | < 0.001 |
| Adolescence | Prevalence | Female | Jamaica | RHD | 0.21<br>(0.19 to 0.24) | < 0.001 |
| Adolescence | Prevalence | Male | Jamaica | RHD | 0.14<br>(0.12 to 0.16) | < 0.001 |
| Adolescence | Prevalence | Both | Jamaica | RA | 0.62<br>(0.61 to 0.64) | < 0.001 |
| Adolescence | Prevalence | Female | Jamaica | RA | 0.65<br>(0.63 to 0.67) | < 0.001 |
| Adolescence | Prevalence | Male | Jamaica | RA | 0.68<br>(0.66 to 0.7) | < 0.001 |
| Adolescence | Prevalence | Both | Japan | AA | 0<br>(0 to 0) | < 0.001 |
| Adolescence | Prevalence | Female | Japan | AA | 0<br>(0 to 0) | < 0.001 |
| Adolescence | Prevalence | Male | Japan | AA | 0<br>(0 to 0) | < 0.001 |
| Adolescence | Prevalence | Both | Japan | T1D | 0.15<br>(0.14 to 0.16) | < 0.001 |

| | | | | | | |
|---|---|---|---|---|---|---|
| Adolescence | Prevalence | Female | Japan | T1D | 0.22<br>(0.21 to 0.23) | < 0.001 |
| Adolescence | Prevalence | Male | Japan | T1D | 0.08<br>(0.06 to 0.11) | < 0.001 |
| Adolescence | Prevalence | Both | Japan | IBD | 3.64<br>(3.49 to 3.8) | < 0.001 |
| Adolescence | Prevalence | Female | Japan | IBD | 3.17<br>(3.02 to 3.32) | < 0.001 |
| Adolescence | Prevalence | Male | Japan | IBD | 3.97<br>(3.78 to 4.16) | < 0.001 |
| Adolescence | Prevalence | Both | Japan | MS | 0.19<br>(0.08 to 0.3) | 0.001 |
| Adolescence | Prevalence | Female | Japan | MS | 0.29<br>(0.17 to 0.41) | < 0.001 |
| Adolescence | Prevalence | Male | Japan | MS | 0.06<br>(-0.06 to 0.18) | 0.323 |
| Adolescence | Prevalence | Both | Japan | Psoriasis | -0.03<br>(-0.06 to 0.01) | 0.132 |
| Adolescence | Prevalence | Female | Japan | Psoriasis | -0.09<br>(-0.16 to -0.03) | 0.005 |
| Adolescence | Prevalence | Male | Japan | Psoriasis | 0.06<br>(0.04 to 0.07) | < 0.001 |
| Adolescence | Prevalence | Both | Japan | RHD | -0.88<br>(-1.01 to -0.74) | < 0.001 |
| Adolescence | Prevalence | Female | Japan | RHD | -0.85<br>(-1.05 to -0.64) | < 0.001 |
| Adolescence | Prevalence | Male | Japan | RHD | -0.9<br>(-1 to -0.81) | < 0.001 |
| Adolescence | Prevalence | Both | Japan | RA | 0.34<br>(0.23 to 0.45) | < 0.001 |
| Adolescence | Prevalence | Female | Japan | RA | 0.42<br>(0.29 to 0.55) | < 0.001 |
| Adolescence | Prevalence | Male | Japan | RA | 0.19 | < 0.001 |

| | | | | | | |
|---|---|---|---|---|---|---|
| | | | | | (0.11 to 0.27) | |
| Adolescence | Prevalence | Both | Jordan | AA | 0.01 (0 to 0.02) | 0.167 |
| Adolescence | Prevalence | Female | Jordan | AA | 0 (0 to 0) | 0.001 |
| Adolescence | Prevalence | Male | Jordan | AA | 0 (0 to 0) | 0.011 |
| Adolescence | Prevalence | Both | Jordan | T1D | 2.08 (2.04 to 2.12) | < 0.001 |
| Adolescence | Prevalence | Female | Jordan | T1D | 2.12 (2.08 to 2.16) | < 0.001 |
| Adolescence | Prevalence | Male | Jordan | T1D | 2.07 (2.01 to 2.12) | < 0.001 |
| Adolescence | Prevalence | Both | Jordan | IBD | 1.05 (0.98 to 1.12) | < 0.001 |
| Adolescence | Prevalence | Female | Jordan | IBD | 0.96 (0.89 to 1.03) | < 0.001 |
| Adolescence | Prevalence | Male | Jordan | IBD | 1.14 (1.08 to 1.19) | < 0.001 |
| Adolescence | Prevalence | Both | Jordan | MS | 0.16 (0.1 to 0.21) | < 0.001 |
| Adolescence | Prevalence | Female | Jordan | MS | 0.14 (0.11 to 0.17) | < 0.001 |
| Adolescence | Prevalence | Male | Jordan | MS | 0.16 (0.09 to 0.23) | < 0.001 |
| Adolescence | Prevalence | Both | Jordan | Psoriasis | -1.09 (-1.1 to -1.08) | < 0.001 |
| Adolescence | Prevalence | Female | Jordan | Psoriasis | -1.06 (-1.07 to -1.05) | < 0.001 |
| Adolescence | Prevalence | Male | Jordan | Psoriasis | -1.13 (-1.16 to -1.1) | < 0.001 |
| Adolescence | Prevalence | Both | Jordan | RHD | -0.35 (-0.42 to -0.27) | < 0.001 |

| | | | | | | |
|---|---|---|---|---|---|---|
| Adolescence | Prevalence | Female | Jordan | RHD | -0.56<br>(-0.67 to -0.45) | < 0.001 |
| Adolescence | Prevalence | Male | Jordan | RHD | -0.1<br>(-0.21 to 0) | 0.055 |
| Adolescence | Prevalence | Both | Jordan | RA | 0.81<br>(0.78 to 0.84) | < 0.001 |
| Adolescence | Prevalence | Female | Jordan | RA | 0.77<br>(0.7 to 0.84) | < 0.001 |
| Adolescence | Prevalence | Male | Jordan | RA | 0.86<br>(0.8 to 0.93) | < 0.001 |
| Adolescence | Prevalence | Both | Kazakhstan | AA | 0<br>(0 to 0) | 0.943 |
| Adolescence | Prevalence | Female | Kazakhstan | AA | 0<br>(0 to 0) | < 0.001 |
| Adolescence | Prevalence | Male | Kazakhstan | AA | 0<br>(0 to 0) | 1.000 |
| Adolescence | Prevalence | Both | Kazakhstan | T1D | 1.23<br>(1.21 to 1.25) | < 0.001 |
| Adolescence | Prevalence | Female | Kazakhstan | T1D | 1.19<br>(1.17 to 1.2) | < 0.001 |
| Adolescence | Prevalence | Male | Kazakhstan | T1D | 1.27<br>(1.24 to 1.29) | < 0.001 |
| Adolescence | Prevalence | Both | Kazakhstan | IBD | 0.14<br>(0.1 to 0.18) | < 0.001 |
| Adolescence | Prevalence | Female | Kazakhstan | IBD | 0.09<br>(0.01 to 0.18) | 0.027 |
| Adolescence | Prevalence | Male | Kazakhstan | IBD | 0.14<br>(0.12 to 0.17) | < 0.001 |
| Adolescence | Prevalence | Both | Kazakhstan | MS | 0.27<br>(0.2 to 0.34) | < 0.001 |
| Adolescence | Prevalence | Female | Kazakhstan | MS | 0.28<br>(0.21 to 0.34) | < 0.001 |
| Adolescence | Prevalence | Male | Kazakhstan | MS | 0.32 | 0.001 |

| | | | | | | |
|---|---|---|---|---|---|---|
| | | | | | (0.13 to 0.5) | |
| Adolescence | Prevalence | Both | Kazakhstan | Psoriasis | -0.8<br>(-0.83 to -0.77) | < 0.001 |
| Adolescence | Prevalence | Female | Kazakhstan | Psoriasis | -0.8<br>(-0.84 to -0.76) | < 0.001 |
| Adolescence | Prevalence | Male | Kazakhstan | Psoriasis | -0.78<br>(-0.8 to -0.76) | < 0.001 |
| Adolescence | Prevalence | Both | Kazakhstan | RHD | -1.23<br>(-1.32 to -1.15) | < 0.001 |
| Adolescence | Prevalence | Female | Kazakhstan | RHD | -1.33<br>(-1.43 to -1.23) | < 0.001 |
| Adolescence | Prevalence | Male | Kazakhstan | RHD | -1.1<br>(-1.2 to -0.99) | < 0.001 |
| Adolescence | Prevalence | Both | Kazakhstan | RA | 0.61<br>(0.6 to 0.62) | < 0.001 |
| Adolescence | Prevalence | Female | Kazakhstan | RA | 0.59<br>(0.56 to 0.62) | < 0.001 |
| Adolescence | Prevalence | Male | Kazakhstan | RA | 0.64<br>(0.6 to 0.68) | < 0.001 |
| Adolescence | Prevalence | Both | Kenya | AA | -0.02<br>(-0.02 to -0.02) | < 0.001 |
| Adolescence | Prevalence | Female | Kenya | AA | 0<br>(0 to 0) | < 0.001 |
| Adolescence | Prevalence | Male | Kenya | AA | 0<br>(0 to 0) | < 0.001 |
| Adolescence | Prevalence | Both | Kenya | T1D | 0.16<br>(0.15 to 0.18) | < 0.001 |
| Adolescence | Prevalence | Female | Kenya | T1D | 0.16<br>(0.15 to 0.17) | < 0.001 |
| Adolescence | Prevalence | Male | Kenya | T1D | 0.15<br>(0.14 to 0.17) | < 0.001 |
| Adolescence | Prevalence | Both | Kenya | IBD | 0.2<br>(0.14 to 0.27) | < 0.001 |

| | | | | | | |
|---|---|---|---|---|---|---|
| Adolescence | Prevalence | Female | Kenya | IBD | 0.21 (0.14 to 0.28) | < 0.001 |
| Adolescence | Prevalence | Male | Kenya | IBD | 0.21 (0.15 to 0.28) | < 0.001 |
| Adolescence | Prevalence | Both | Kenya | MS | 0.11 (-0.01 to 0.22) | 0.068 |
| Adolescence | Prevalence | Female | Kenya | MS | 0.07 (0.01 to 0.13) | 0.023 |
| Adolescence | Prevalence | Male | Kenya | MS | 0.06 (-0.06 to 0.18) | 0.341 |
| Adolescence | Prevalence | Both | Kenya | Psoriasis | -0.28 (-0.31 to -0.25) | < 0.001 |
| Adolescence | Prevalence | Female | Kenya | Psoriasis | -0.3 (-0.32 to -0.29) | < 0.001 |
| Adolescence | Prevalence | Male | Kenya | Psoriasis | -0.25 (-0.3 to -0.2) | < 0.001 |
| Adolescence | Prevalence | Both | Kenya | RHD | 0.64 (0.6 to 0.68) | < 0.001 |
| Adolescence | Prevalence | Female | Kenya | RHD | 0.69 (0.58 to 0.8) | < 0.001 |
| Adolescence | Prevalence | Male | Kenya | RHD | 0.6 (0.54 to 0.67) | < 0.001 |
| Adolescence | Prevalence | Both | Kenya | RA | 0.06 (0.01 to 0.1) | 0.011 |
| Adolescence | Prevalence | Female | Kenya | RA | -0.01 (-0.06 to 0.05) | 0.838 |
| Adolescence | Prevalence | Male | Kenya | RA | 0.28 (0.24 to 0.32) | < 0.001 |
| Adolescence | Prevalence | Both | Kiribati | AA | 0.02 (0.01 to 0.02) | < 0.001 |
| Adolescence | Prevalence | Female | Kiribati | AA | 0 (0 to 0) | 0.008 |
| Adolescence | Prevalence | Male | Kiribati | AA | 0 | < 0.001 |

| | | | | | | |
|---|---|---|---|---|---|---|
| | | | | | (0 to 0) | |
| Adolescence | Prevalence | Both | Kiribati | T1D | 0.61<br>(0.6 to 0.62) | < 0.001 |
| Adolescence | Prevalence | Female | Kiribati | T1D | 0.58<br>(0.51 to 0.64) | < 0.001 |
| Adolescence | Prevalence | Male | Kiribati | T1D | 0.66<br>(0.63 to 0.69) | < 0.001 |
| Adolescence | Prevalence | Both | Kiribati | IBD | -0.06<br>(-0.13 to 0) | 0.063 |
| Adolescence | Prevalence | Female | Kiribati | IBD | -0.08<br>(-0.17 to 0.01) | 0.070 |
| Adolescence | Prevalence | Male | Kiribati | IBD | -0.06<br>(-0.14 to 0.02) | 0.117 |
| Adolescence | Prevalence | Both | Kiribati | MS | -0.02<br>(-0.2 to 0.15) | 0.789 |
| Adolescence | Prevalence | Female | Kiribati | MS | 0.05<br>(-0.07 to 0.17) | 0.391 |
| Adolescence | Prevalence | Male | Kiribati | MS | -0.1<br>(-0.3 to 0.1) | 0.310 |
| Adolescence | Prevalence | Both | Kiribati | Psoriasis | -0.12<br>(-0.15 to -0.1) | < 0.001 |
| Adolescence | Prevalence | Female | Kiribati | Psoriasis | -0.12<br>(-0.16 to -0.09) | < 0.001 |
| Adolescence | Prevalence | Male | Kiribati | Psoriasis | -0.16<br>(-0.19 to -0.12) | < 0.001 |
| Adolescence | Prevalence | Both | Kiribati | RHD | 0.09<br>(0.05 to 0.12) | < 0.001 |
| Adolescence | Prevalence | Female | Kiribati | RHD | 0.06<br>(0.03 to 0.1) | < 0.001 |
| Adolescence | Prevalence | Male | Kiribati | RHD | 0.11<br>(0.08 to 0.13) | < 0.001 |
| Adolescence | Prevalence | Both | Kiribati | RA | 0.3<br>(0.25 to 0.35) | < 0.001 |

| | | | | | | |
|---|---|---|---|---|---|---|
| Adolescence | Prevalence | Female | Kiribati | RA | 0.36 (0.31 to 0.4) | < 0.001 |
| Adolescence | Prevalence | Male | Kiribati | RA | 0.17 (0.1 to 0.24) | < 0.001 |
| Adolescence | Prevalence | Both | Kuwait | AA | -0.04 (-0.06 to -0.02) | 0.001 |
| Adolescence | Prevalence | Female | Kuwait | AA | 0 (0 to 0) | 0.001 |
| Adolescence | Prevalence | Male | Kuwait | AA | 0 (0 to 0) | 0.011 |
| Adolescence | Prevalence | Both | Kuwait | T1D | 1.04 (-0.29 to 2.4) | 0.127 |
| Adolescence | Prevalence | Female | Kuwait | T1D | 0.91 (0.8 to 1.01) | < 0.001 |
| Adolescence | Prevalence | Male | Kuwait | T1D | 1.3 (0.06 to 2.55) | 0.040 |
| Adolescence | Prevalence | Both | Kuwait | IBD | -1.52 (-2.07 to -0.97) | < 0.001 |
| Adolescence | Prevalence | Female | Kuwait | IBD | -1.55 (-2.13 to -0.96) | < 0.001 |
| Adolescence | Prevalence | Male | Kuwait | IBD | -1.39 (-1.91 to -0.86) | < 0.001 |
| Adolescence | Prevalence | Both | Kuwait | MS | 1.35 (1.24 to 1.45) | < 0.001 |
| Adolescence | Prevalence | Female | Kuwait | MS | 1.43 (1.25 to 1.61) | < 0.001 |
| Adolescence | Prevalence | Male | Kuwait | MS | 1.34 (1.27 to 1.41) | < 0.001 |
| Adolescence | Prevalence | Both | Kuwait | Psoriasis | -0.94 (-1 to -0.88) | < 0.001 |
| Adolescence | Prevalence | Female | Kuwait | Psoriasis | -0.89 (-0.95 to -0.83) | < 0.001 |
| Adolescence | Prevalence | Male | Kuwait | Psoriasis | -0.97 | < 0.001 |

| | | | | | | |
|---|---|---|---|---|---|---|
| | | | | | (-1 to -0.93) | |
| Adolescence | Prevalence | Both | Kuwait | RHD | -0.93 (-1.03 to -0.83) | < 0.001 |
| Adolescence | Prevalence | Female | Kuwait | RHD | -0.84 (-1.04 to -0.64) | < 0.001 |
| Adolescence | Prevalence | Male | Kuwait | RHD | -1.06 (-1.15 to -0.96) | < 0.001 |
| Adolescence | Prevalence | Both | Kuwait | RA | 0.58 (0.54 to 0.63) | < 0.001 |
| Adolescence | Prevalence | Female | Kuwait | RA | 0.64 (0.6 to 0.67) | < 0.001 |
| Adolescence | Prevalence | Male | Kuwait | RA | 0.65 (0.55 to 0.74) | < 0.001 |
| Adolescence | Prevalence | Both | Kyrgyzstan | AA | -0.01 (-0.01 to 0) | < 0.001 |
| Adolescence | Prevalence | Female | Kyrgyzstan | AA | 0 (0 to 0) | < 0.001 |
| Adolescence | Prevalence | Male | Kyrgyzstan | AA | 0 (0 to 0) | 1.000 |
| Adolescence | Prevalence | Both | Kyrgyzstan | T1D | 1.22 (1.2 to 1.23) | < 0.001 |
| Adolescence | Prevalence | Female | Kyrgyzstan | T1D | 1.21 (1.15 to 1.26) | < 0.001 |
| Adolescence | Prevalence | Male | Kyrgyzstan | T1D | 1.24 (1.22 to 1.26) | < 0.001 |
| Adolescence | Prevalence | Both | Kyrgyzstan | IBD | -0.01 (-0.04 to 0.02) | 0.455 |
| Adolescence | Prevalence | Female | Kyrgyzstan | IBD | -0.04 (-0.06 to -0.03) | < 0.001 |
| Adolescence | Prevalence | Male | Kyrgyzstan | IBD | 0.02 (-0.01 to 0.04) | 0.261 |
| Adolescence | Prevalence | Both | Kyrgyzstan | MS | 0.22 (0.14 to 0.3) | < 0.001 |

| | | | | | | |
|---|---|---|---|---|---|---|
| Adolescence | Prevalence | Female | Kyrgyzstan | MS | 0.19 (0.1 to 0.28) | < 0.001 |
| Adolescence | Prevalence | Male | Kyrgyzstan | MS | 0.33 (0.05 to 0.6) | 0.019 |
| Adolescence | Prevalence | Both | Kyrgyzstan | Psoriasis | -0.59 (-0.62 to -0.57) | < 0.001 |
| Adolescence | Prevalence | Female | Kyrgyzstan | Psoriasis | -0.61 (-0.62 to -0.59) | < 0.001 |
| Adolescence | Prevalence | Male | Kyrgyzstan | Psoriasis | -0.55 (-0.6 to -0.51) | < 0.001 |
| Adolescence | Prevalence | Both | Kyrgyzstan | RHD | 0.03 (-0.01 to 0.08) | 0.145 |
| Adolescence | Prevalence | Female | Kyrgyzstan | RHD | 0.06 (0 to 0.12) | 0.066 |
| Adolescence | Prevalence | Male | Kyrgyzstan | RHD | 0.03 (0 to 0.07) | 0.081 |
| Adolescence | Prevalence | Both | Kyrgyzstan | RA | 0.39 (0.33 to 0.44) | < 0.001 |
| Adolescence | Prevalence | Female | Kyrgyzstan | RA | 0.37 (0.3 to 0.45) | < 0.001 |
| Adolescence | Prevalence | Male | Kyrgyzstan | RA | 0.51 (0.46 to 0.55) | < 0.001 |
| Adolescence | Prevalence | Both | Lao People's Democratic Republic | AA | -0.02 (-0.02 to -0.02) | < 0.001 |
| Adolescence | Prevalence | Female | Lao People's Democratic Republic | AA | 0 (0 to 0) | 0.008 |
| Adolescence | Prevalence | Male | Lao People's Democratic Republic | AA | 0 (0 to 0) | < 0.001 |
| Adolescence | Prevalence | Both | Lao People's Democratic Republic | T1D | 0.43 (0.39 to 0.47) | < 0.001 |
| Adolescence | Prevalence | Female | Lao People's Democratic Republic | T1D | 0.39 (0.3 to 0.49) | < 0.001 |
| Adolescence | Prevalence | Male | Lao People's Democratic Republic | T1D | 0.44 | < 0.001 |

| | | | | | | |
|---|---|---|---|---|---|---|
| | | | | | (0.41 to 0.48) | |
| Adolescence | Prevalence | Both | Lao People's Democratic Republic | IBD | 0.67<br>(0.55 to 0.78) | < 0.001 |
| Adolescence | Prevalence | Female | Lao People's Democratic Republic | IBD | 0.72<br>(0.63 to 0.8) | < 0.001 |
| Adolescence | Prevalence | Male | Lao People's Democratic Republic | IBD | 0.65<br>(0.56 to 0.75) | < 0.001 |
| Adolescence | Prevalence | Both | Lao People's Democratic Republic | MS | -0.07<br>(-0.29 to 0.16) | 0.548 |
| Adolescence | Prevalence | Female | Lao People's Democratic Republic | MS | -0.04<br>(-0.13 to 0.04) | 0.308 |
| Adolescence | Prevalence | Male | Lao People's Democratic Republic | MS | -0.02<br>(-0.28 to 0.24) | 0.862 |
| Adolescence | Prevalence | Both | Lao People's Democratic Republic | Psoriasis | -0.66<br>(-0.69 to -0.64) | < 0.001 |
| Adolescence | Prevalence | Female | Lao People's Democratic Republic | Psoriasis | -0.61<br>(-0.63 to -0.6) | < 0.001 |
| Adolescence | Prevalence | Male | Lao People's Democratic Republic | Psoriasis | -0.67<br>(-0.73 to -0.61) | < 0.001 |
| Adolescence | Prevalence | Both | Lao People's Democratic Republic | RHD | 0<br>(-0.55 to 0.56) | 0.998 |
| Adolescence | Prevalence | Female | Lao People's Democratic Republic | RHD | 0.01<br>(-0.54 to 0.57) | 0.971 |
| Adolescence | Prevalence | Male | Lao People's Democratic Republic | RHD | 0.04<br>(-0.52 to 0.6) | 0.892 |
| Adolescence | Prevalence | Both | Lao People's Democratic Republic | RA | 0.34<br>(0.31 to 0.37) | < 0.001 |
| Adolescence | Prevalence | Female | Lao People's Democratic Republic | RA | 0.4<br>(0.33 to 0.48) | < 0.001 |
| Adolescence | Prevalence | Male | Lao People's Democratic Republic | RA | 0.3<br>(0.27 to 0.33) | < 0.001 |
| Adolescence | Prevalence | Both | Latvia | AA | 0<br>(0 to 0.01) | 0.780 |

| | | | | | | |
|---|---|---|---|---|---|---|
| Adolescence | Prevalence | Female | Latvia | AA | 0<br>(0 to 0) | < 0.001 |
| Adolescence | Prevalence | Male | Latvia | AA | 0<br>(0 to 0) | 1.000 |
| Adolescence | Prevalence | Both | Latvia | T1D | 1.48<br>(1.42 to 1.54) | < 0.001 |
| Adolescence | Prevalence | Female | Latvia | T1D | 1.43<br>(1.37 to 1.48) | < 0.001 |
| Adolescence | Prevalence | Male | Latvia | T1D | 1.55<br>(1.48 to 1.62) | < 0.001 |
| Adolescence | Prevalence | Both | Latvia | IBD | 0.94<br>(0.86 to 1.02) | < 0.001 |
| Adolescence | Prevalence | Female | Latvia | IBD | 1.04<br>(0.84 to 1.24) | < 0.001 |
| Adolescence | Prevalence | Male | Latvia | IBD | 0.9<br>(0.82 to 0.98) | < 0.001 |
| Adolescence | Prevalence | Both | Latvia | MS | 0.1<br>(0.06 to 0.14) | < 0.001 |
| Adolescence | Prevalence | Female | Latvia | MS | 0.05<br>(-0.01 to 0.11) | 0.100 |
| Adolescence | Prevalence | Male | Latvia | MS | 0.19<br>(0.16 to 0.22) | < 0.001 |
| Adolescence | Prevalence | Both | Latvia | Psoriasis | -0.74<br>(-0.77 to -0.72) | < 0.001 |
| Adolescence | Prevalence | Female | Latvia | Psoriasis | -0.75<br>(-0.78 to -0.71) | < 0.001 |
| Adolescence | Prevalence | Male | Latvia | Psoriasis | -0.74<br>(-0.77 to -0.72) | < 0.001 |
| Adolescence | Prevalence | Both | Latvia | RHD | -0.7<br>(-1.04 to -0.37) | < 0.001 |
| Adolescence | Prevalence | Female | Latvia | RHD | -0.43<br>(-0.7 to -0.17) | 0.001 |
| Adolescence | Prevalence | Male | Latvia | RHD | -0.97 | < 0.001 |

| | | | | | | |
|---|---|---|---|---|---|---|
| | | | | | (-1.39 to -0.54) | |
| Adolescence | Prevalence | Both | Latvia | RA | 0.57<br>(0.53 to 0.61) | < 0.001 |
| Adolescence | Prevalence | Female | Latvia | RA | 0.56<br>(0.52 to 0.6) | < 0.001 |
| Adolescence | Prevalence | Male | Latvia | RA | 0.57<br>(0.5 to 0.65) | < 0.001 |
| Adolescence | Prevalence | Both | Lebanon | AA | 0<br>(-0.01 to 0.02) | 0.632 |
| Adolescence | Prevalence | Female | Lebanon | AA | 0<br>(0 to 0) | 0.001 |
| Adolescence | Prevalence | Male | Lebanon | AA | 0<br>(0 to 0) | 0.011 |
| Adolescence | Prevalence | Both | Lebanon | T1D | 2.14<br>(2.09 to 2.19) | < 0.001 |
| Adolescence | Prevalence | Female | Lebanon | T1D | 2.09<br>(2.05 to 2.14) | < 0.001 |
| Adolescence | Prevalence | Male | Lebanon | T1D | 2.17<br>(2.03 to 2.3) | < 0.001 |
| Adolescence | Prevalence | Both | Lebanon | IBD | 0.6<br>(0.5 to 0.7) | < 0.001 |
| Adolescence | Prevalence | Female | Lebanon | IBD | 0.29<br>(0.22 to 0.36) | < 0.001 |
| Adolescence | Prevalence | Male | Lebanon | IBD | 0.91<br>(0.74 to 1.07) | < 0.001 |
| Adolescence | Prevalence | Both | Lebanon | MS | 0.45<br>(0.38 to 0.51) | < 0.001 |
| Adolescence | Prevalence | Female | Lebanon | MS | 0.41<br>(0.25 to 0.57) | < 0.001 |
| Adolescence | Prevalence | Male | Lebanon | MS | 0.54<br>(0.5 to 0.57) | < 0.001 |
| Adolescence | Prevalence | Both | Lebanon | Psoriasis | -1.23<br>(-1.24 to -1.22) | < 0.001 |

| | | | | | | |
|---|---|---|---|---|---|---|
| Adolescence | Prevalence | Female | Lebanon | Psoriasis | -1.2 (-1.21 to -1.19) | < 0.001 |
| Adolescence | Prevalence | Male | Lebanon | Psoriasis | -1.27 (-1.28 to -1.27) | < 0.001 |
| Adolescence | Prevalence | Both | Lebanon | RHD | -1.65 (-1.77 to -1.53) | < 0.001 |
| Adolescence | Prevalence | Female | Lebanon | RHD | -0.9 (-0.97 to -0.82) | < 0.001 |
| Adolescence | Prevalence | Male | Lebanon | RHD | -2.16 (-2.43 to -1.9) | < 0.001 |
| Adolescence | Prevalence | Both | Lebanon | RA | 0.71 (0.68 to 0.74) | < 0.001 |
| Adolescence | Prevalence | Female | Lebanon | RA | 0.7 (0.68 to 0.72) | < 0.001 |
| Adolescence | Prevalence | Male | Lebanon | RA | 0.73 (0.71 to 0.75) | < 0.001 |
| Adolescence | Prevalence | Both | Lesotho | AA | -0.01 (-0.02 to -0.01) | < 0.001 |
| Adolescence | Prevalence | Female | Lesotho | AA | 0 (0 to 0) | < 0.001 |
| Adolescence | Prevalence | Male | Lesotho | AA | 0 (0 to 0) | < 0.001 |
| Adolescence | Prevalence | Both | Lesotho | T1D | 0.11 (0.09 to 0.14) | < 0.001 |
| Adolescence | Prevalence | Female | Lesotho | T1D | 0.12 (0.11 to 0.13) | < 0.001 |
| Adolescence | Prevalence | Male | Lesotho | T1D | 0.11 (0.07 to 0.14) | < 0.001 |
| Adolescence | Prevalence | Both | Lesotho | IBD | 0.34 (0.28 to 0.4) | < 0.001 |
| Adolescence | Prevalence | Female | Lesotho | IBD | 0.35 (0.24 to 0.47) | < 0.001 |
| Adolescence | Prevalence | Male | Lesotho | IBD | 0.34 | < 0.001 |

| | | | | | | |
|---|---|---|---|---|---|---|
| | | | | | (0.27 to 0.41) | |
| Adolescence | Prevalence | Both | Lesotho | MS | 0.04<br>(-0.04 to 0.13) | 0.344 |
| Adolescence | Prevalence | Female | Lesotho | MS | 0.04<br>(-0.17 to 0.25) | 0.693 |
| Adolescence | Prevalence | Male | Lesotho | MS | 0.09<br>(0.06 to 0.13) | < 0.001 |
| Adolescence | Prevalence | Both | Lesotho | Psoriasis | -0.61<br>(-0.66 to -0.56) | < 0.001 |
| Adolescence | Prevalence | Female | Lesotho | Psoriasis | -0.63<br>(-0.71 to -0.56) | < 0.001 |
| Adolescence | Prevalence | Male | Lesotho | Psoriasis | -0.58<br>(-0.65 to -0.52) | < 0.001 |
| Adolescence | Prevalence | Both | Lesotho | RHD | 0.06<br>(0.02 to 0.1) | 0.005 |
| Adolescence | Prevalence | Female | Lesotho | RHD | 0.04<br>(0.03 to 0.06) | < 0.001 |
| Adolescence | Prevalence | Male | Lesotho | RHD | 0.06<br>(0 to 0.12) | 0.067 |
| Adolescence | Prevalence | Both | Lesotho | RA | 0.38<br>(0.32 to 0.45) | < 0.001 |
| Adolescence | Prevalence | Female | Lesotho | RA | 0.36<br>(0.27 to 0.45) | < 0.001 |
| Adolescence | Prevalence | Male | Lesotho | RA | 0.49<br>(0.46 to 0.52) | < 0.001 |
| Adolescence | Prevalence | Both | Liberia | AA | -0.03<br>(-0.06 to 0) | 0.020 |
| Adolescence | Prevalence | Female | Liberia | AA | 0<br>(0 to 0) | < 0.001 |
| Adolescence | Prevalence | Male | Liberia | AA | 0<br>(0 to 0) | < 0.001 |
| Adolescence | Prevalence | Both | Liberia | T1D | 0.08<br>(0.06 to 0.1) | < 0.001 |

| | | | | | | |
|---|---|---|---|---|---|---|
| Adolescence | Prevalence | Female | Liberia | T1D | 0.07 (0.04 to 0.1) | < 0.001 |
| Adolescence | Prevalence | Male | Liberia | T1D | 0.07 (0.03 to 0.11) | 0.001 |
| Adolescence | Prevalence | Both | Liberia | IBD | 0 (-0.12 to 0.12) | 0.970 |
| Adolescence | Prevalence | Female | Liberia | IBD | -0.15 (-0.23 to -0.07) | < 0.001 |
| Adolescence | Prevalence | Male | Liberia | IBD | 0.2 (0.08 to 0.32) | 0.001 |
| Adolescence | Prevalence | Both | Liberia | MS | 0.06 (-0.07 to 0.18) | 0.356 |
| Adolescence | Prevalence | Female | Liberia | MS | 0.11 (0.01 to 0.2) | 0.033 |
| Adolescence | Prevalence | Male | Liberia | MS | 0.07 (-0.13 to 0.26) | 0.506 |
| Adolescence | Prevalence | Both | Liberia | Psoriasis | -0.5 (-0.62 to -0.38) | < 0.001 |
| Adolescence | Prevalence | Female | Liberia | Psoriasis | -0.58 (-0.71 to -0.46) | < 0.001 |
| Adolescence | Prevalence | Male | Liberia | Psoriasis | -0.47 (-0.6 to -0.35) | < 0.001 |
| Adolescence | Prevalence | Both | Liberia | RHD | 0.3 (0.28 to 0.33) | < 0.001 |
| Adolescence | Prevalence | Female | Liberia | RHD | 0.3 (0.29 to 0.32) | < 0.001 |
| Adolescence | Prevalence | Male | Liberia | RHD | 0.3 (0.26 to 0.34) | < 0.001 |
| Adolescence | Prevalence | Both | Liberia | RA | 0.49 (0.46 to 0.53) | < 0.001 |
| Adolescence | Prevalence | Female | Liberia | RA | 0.57 (0.53 to 0.61) | < 0.001 |
| Adolescence | Prevalence | Male | Liberia | RA | 0.47 | < 0.001 |

| | | | | | | |
|---|---|---|---|---|---|---|
| | | | | | (0.43 to 0.52) | |
| Adolescence | Prevalence | Both | Libya | AA | -0.01 (-0.02 to 0.01) | 0.450 |
| Adolescence | Prevalence | Female | Libya | AA | 0 (0 to 0) | 0.001 |
| Adolescence | Prevalence | Male | Libya | AA | 0 (0 to 0) | 0.011 |
| Adolescence | Prevalence | Both | Libya | T1D | 2.35 (2.29 to 2.41) | < 0.001 |
| Adolescence | Prevalence | Female | Libya | T1D | 2.18 (2.12 to 2.25) | < 0.001 |
| Adolescence | Prevalence | Male | Libya | T1D | 2.54 (2.49 to 2.59) | < 0.001 |
| Adolescence | Prevalence | Both | Libya | IBD | 0.59 (0.43 to 0.75) | < 0.001 |
| Adolescence | Prevalence | Female | Libya | IBD | 0.54 (0.38 to 0.69) | < 0.001 |
| Adolescence | Prevalence | Male | Libya | IBD | 0.57 (0.42 to 0.72) | < 0.001 |
| Adolescence | Prevalence | Both | Libya | MS | 0.48 (0.43 to 0.52) | < 0.001 |
| Adolescence | Prevalence | Female | Libya | MS | 0.44 (0.4 to 0.49) | < 0.001 |
| Adolescence | Prevalence | Male | Libya | MS | 0.56 (0.42 to 0.69) | < 0.001 |
| Adolescence | Prevalence | Both | Libya | Psoriasis | -1.22 (-1.26 to -1.18) | < 0.001 |
| Adolescence | Prevalence | Female | Libya | Psoriasis | -1.18 (-1.22 to -1.15) | < 0.001 |
| Adolescence | Prevalence | Male | Libya | Psoriasis | -1.25 (-1.29 to -1.21) | < 0.001 |
| Adolescence | Prevalence | Both | Libya | RHD | 0.4 (0.37 to 0.42) | < 0.001 |

| | | | | | | |
|---|---|---|---|---|---|---|
| Adolescence | Prevalence | Female | Libya | RHD | 0.42 (0.37 to 0.47) | < 0.001 |
| Adolescence | Prevalence | Male | Libya | RHD | 0.39 (0.37 to 0.41) | < 0.001 |
| Adolescence | Prevalence | Both | Libya | RA | 0.53 (0.49 to 0.57) | < 0.001 |
| Adolescence | Prevalence | Female | Libya | RA | 0.51 (0.45 to 0.57) | < 0.001 |
| Adolescence | Prevalence | Male | Libya | RA | 0.56 (0.47 to 0.64) | < 0.001 |
| Adolescence | Prevalence | Both | Lithuania | AA | -0.01 (-0.01 to -0.01) | < 0.001 |
| Adolescence | Prevalence | Female | Lithuania | AA | 0 (0 to 0) | < 0.001 |
| Adolescence | Prevalence | Male | Lithuania | AA | 0 (0 to 0) | 1.000 |
| Adolescence | Prevalence | Both | Lithuania | T1D | 1.62 (1.54 to 1.7) | < 0.001 |
| Adolescence | Prevalence | Female | Lithuania | T1D | 1.54 (1.47 to 1.61) | < 0.001 |
| Adolescence | Prevalence | Male | Lithuania | T1D | 1.69 (1.59 to 1.8) | < 0.001 |
| Adolescence | Prevalence | Both | Lithuania | IBD | 1.2 (1.12 to 1.28) | < 0.001 |
| Adolescence | Prevalence | Female | Lithuania | IBD | 1.12 (1.07 to 1.17) | < 0.001 |
| Adolescence | Prevalence | Male | Lithuania | IBD | 1.28 (1.14 to 1.41) | < 0.001 |
| Adolescence | Prevalence | Both | Lithuania | MS | 0.15 (0.11 to 0.19) | < 0.001 |
| Adolescence | Prevalence | Female | Lithuania | MS | 0.18 (0.12 to 0.23) | < 0.001 |
| Adolescence | Prevalence | Male | Lithuania | MS | 0.14 | 0.056 |

| | | | | | | |
|---|---|---|---|---|---|---|
| | | | | | (0 to 0.28) | |
| Adolescence | Prevalence | Both | Lithuania | Psoriasis | -0.85<br>(-0.87 to -0.84) | < 0.001 |
| Adolescence | Prevalence | Female | Lithuania | Psoriasis | -0.84<br>(-0.86 to -0.83) | < 0.001 |
| Adolescence | Prevalence | Male | Lithuania | Psoriasis | -0.85<br>(-0.86 to -0.83) | < 0.001 |
| Adolescence | Prevalence | Both | Lithuania | RHD | -0.49<br>(-0.58 to -0.41) | < 0.001 |
| Adolescence | Prevalence | Female | Lithuania | RHD | -0.47<br>(-0.56 to -0.38) | < 0.001 |
| Adolescence | Prevalence | Male | Lithuania | RHD | -0.52<br>(-0.63 to -0.42) | < 0.001 |
| Adolescence | Prevalence | Both | Lithuania | RA | 0.45<br>(0.35 to 0.56) | < 0.001 |
| Adolescence | Prevalence | Female | Lithuania | RA | 0.47<br>(0.4 to 0.54) | < 0.001 |
| Adolescence | Prevalence | Male | Lithuania | RA | 0.52<br>(0.49 to 0.55) | < 0.001 |
| Adolescence | Prevalence | Both | Luxembourg | AA | 0<br>(0 to 0) | 0.125 |
| Adolescence | Prevalence | Female | Luxembourg | AA | 0<br>(0 to 0) | < 0.001 |
| Adolescence | Prevalence | Male | Luxembourg | AA | 0<br>(0 to 0) | 0.005 |
| Adolescence | Prevalence | Both | Luxembourg | T1D | 1.66<br>(1.61 to 1.71) | < 0.001 |
| Adolescence | Prevalence | Female | Luxembourg | T1D | 1.61<br>(1.56 to 1.67) | < 0.001 |
| Adolescence | Prevalence | Male | Luxembourg | T1D | 1.7<br>(1.62 to 1.77) | < 0.001 |
| Adolescence | Prevalence | Both | Luxembourg | IBD | 0.23<br>(0.12 to 0.34) | < 0.001 |

| | | | | | | |
|---|---|---|---|---|---|---|
| Adolescence | Prevalence | Female | Luxembourg | IBD | 0.01<br>(-0.1 to 0.11) | 0.867 |
| Adolescence | Prevalence | Male | Luxembourg | IBD | 0.47<br>(0.4 to 0.53) | < 0.001 |
| Adolescence | Prevalence | Both | Luxembourg | MS | 0.22<br>(0.19 to 0.24) | < 0.001 |
| Adolescence | Prevalence | Female | Luxembourg | MS | 0.27<br>(0.23 to 0.31) | < 0.001 |
| Adolescence | Prevalence | Male | Luxembourg | MS | 0.1<br>(0.05 to 0.16) | < 0.001 |
| Adolescence | Prevalence | Both | Luxembourg | Psoriasis | -0.27<br>(-0.29 to -0.26) | < 0.001 |
| Adolescence | Prevalence | Female | Luxembourg | Psoriasis | -0.27<br>(-0.29 to -0.25) | < 0.001 |
| Adolescence | Prevalence | Male | Luxembourg | Psoriasis | -0.27<br>(-0.28 to -0.27) | < 0.001 |
| Adolescence | Prevalence | Both | Luxembourg | RHD | -0.11<br>(-0.18 to -0.04) | 0.002 |
| Adolescence | Prevalence | Female | Luxembourg | RHD | -0.09<br>(-0.22 to 0.04) | 0.177 |
| Adolescence | Prevalence | Male | Luxembourg | RHD | -0.16<br>(-0.2 to -0.12) | < 0.001 |
| Adolescence | Prevalence | Both | Luxembourg | RA | 0.34<br>(0.33 to 0.36) | < 0.001 |
| Adolescence | Prevalence | Female | Luxembourg | RA | 0.35<br>(0.33 to 0.36) | < 0.001 |
| Adolescence | Prevalence | Male | Luxembourg | RA | 0.33<br>(0.31 to 0.36) | < 0.001 |
| Adolescence | Prevalence | Both | Madagascar | AA | -0.01<br>(-0.01 to -0.01) | < 0.001 |
| Adolescence | Prevalence | Female | Madagascar | AA | 0<br>(0 to 0) | < 0.001 |
| Adolescence | Prevalence | Male | Madagascar | AA | 0 | < 0.001 |

| | | | | | | |
|---|---|---|---|---|---|---|
| | | | | | (0 to 0) | |
| Adolescence | Prevalence | Both | Madagascar | T1D | 0.08 (0.07 to 0.1) | < 0.001 |
| Adolescence | Prevalence | Female | Madagascar | T1D | 0.09 (0.05 to 0.13) | < 0.001 |
| Adolescence | Prevalence | Male | Madagascar | T1D | 0.07 (0.06 to 0.09) | < 0.001 |
| Adolescence | Prevalence | Both | Madagascar | IBD | 0.17 (0.1 to 0.24) | < 0.001 |
| Adolescence | Prevalence | Female | Madagascar | IBD | 0.19 (0.12 to 0.27) | < 0.001 |
| Adolescence | Prevalence | Male | Madagascar | IBD | 0.2 (0.09 to 0.31) | < 0.001 |
| Adolescence | Prevalence | Both | Madagascar | MS | 0.03 (-0.04 to 0.11) | 0.417 |
| Adolescence | Prevalence | Female | Madagascar | MS | 0.03 (-0.08 to 0.14) | 0.550 |
| Adolescence | Prevalence | Male | Madagascar | MS | 0.11 (0.04 to 0.19) | 0.002 |
| Adolescence | Prevalence | Both | Madagascar | Psoriasis | -0.22 (-0.25 to -0.2) | < 0.001 |
| Adolescence | Prevalence | Female | Madagascar | Psoriasis | -0.24 (-0.27 to -0.21) | < 0.001 |
| Adolescence | Prevalence | Male | Madagascar | Psoriasis | -0.21 (-0.25 to -0.17) | < 0.001 |
| Adolescence | Prevalence | Both | Madagascar | RHD | 0.23 (0.2 to 0.26) | < 0.001 |
| Adolescence | Prevalence | Female | Madagascar | RHD | 0.23 (0.17 to 0.29) | < 0.001 |
| Adolescence | Prevalence | Male | Madagascar | RHD | 0.23 (0.21 to 0.26) | < 0.001 |
| Adolescence | Prevalence | Both | Madagascar | RA | 0.08 (0.01 to 0.15) | 0.018 |

| | | | | | | |
|---|---|---|---|---|---|---|
| Adolescence | Prevalence | Female | Madagascar | RA | 0.11<br>(-0.02 to 0.23) | 0.099 |
| Adolescence | Prevalence | Male | Madagascar | RA | 0.15<br>(0.12 to 0.18) | < 0.001 |
| Adolescence | Prevalence | Both | Malawi | AA | 0<br>(-0.01 to 0) | 0.626 |
| Adolescence | Prevalence | Female | Malawi | AA | 0<br>(0 to 0) | < 0.001 |
| Adolescence | Prevalence | Male | Malawi | AA | 0<br>(0 to 0) | < 0.001 |
| Adolescence | Prevalence | Both | Malawi | T1D | 0.09<br>(0.07 to 0.1) | < 0.001 |
| Adolescence | Prevalence | Female | Malawi | T1D | 0.09<br>(0.06 to 0.11) | < 0.001 |
| Adolescence | Prevalence | Male | Malawi | T1D | 0.1<br>(0.08 to 0.12) | < 0.001 |
| Adolescence | Prevalence | Both | Malawi | IBD | 0.19<br>(0.08 to 0.29) | 0.001 |
| Adolescence | Prevalence | Female | Malawi | IBD | 0.18<br>(0.09 to 0.28) | < 0.001 |
| Adolescence | Prevalence | Male | Malawi | IBD | 0.22<br>(0.11 to 0.33) | < 0.001 |
| Adolescence | Prevalence | Both | Malawi | MS | 0.08<br>(0.08 to 0.08) | < 0.001 |
| Adolescence | Prevalence | Female | Malawi | MS | 0.03<br>(-0.03 to 0.09) | 0.384 |
| Adolescence | Prevalence | Male | Malawi | MS | 0.13<br>(0.04 to 0.23) | 0.006 |
| Adolescence | Prevalence | Both | Malawi | Psoriasis | -0.37<br>(-0.47 to -0.28) | < 0.001 |
| Adolescence | Prevalence | Female | Malawi | Psoriasis | -0.38<br>(-0.47 to -0.3) | < 0.001 |
| Adolescence | Prevalence | Male | Malawi | Psoriasis | -0.34 | < 0.001 |

| | | | | | | |
|---|---|---|---|---|---|---|
| | | | | | (-0.44 to -0.25) | |
| Adolescence | Prevalence | Both | Malawi | RHD | 0.15<br>(0.13 to 0.16) | < 0.001 |
| Adolescence | Prevalence | Female | Malawi | RHD | 0.16<br>(0.09 to 0.22) | < 0.001 |
| Adolescence | Prevalence | Male | Malawi | RHD | 0.1<br>(0.07 to 0.12) | < 0.001 |
| Adolescence | Prevalence | Both | Malawi | RA | 0.2<br>(0.13 to 0.27) | < 0.001 |
| Adolescence | Prevalence | Female | Malawi | RA | 0.17<br>(0.11 to 0.23) | < 0.001 |
| Adolescence | Prevalence | Male | Malawi | RA | 0.28<br>(0.21 to 0.36) | < 0.001 |
| Adolescence | Prevalence | Both | Malaysia | AA | -0.01<br>(-0.01 to -0.01) | < 0.001 |
| Adolescence | Prevalence | Female | Malaysia | AA | 0<br>(0 to 0) | 0.008 |
| Adolescence | Prevalence | Male | Malaysia | AA | 0<br>(0 to 0) | < 0.001 |
| Adolescence | Prevalence | Both | Malaysia | T1D | 0.61<br>(0.59 to 0.63) | < 0.001 |
| Adolescence | Prevalence | Female | Malaysia | T1D | 0.57<br>(0.49 to 0.65) | < 0.001 |
| Adolescence | Prevalence | Male | Malaysia | T1D | 0.66<br>(0.65 to 0.68) | < 0.001 |
| Adolescence | Prevalence | Both | Malaysia | IBD | 1.5<br>(1.38 to 1.61) | < 0.001 |
| Adolescence | Prevalence | Female | Malaysia | IBD | 1.48<br>(1.34 to 1.62) | < 0.001 |
| Adolescence | Prevalence | Male | Malaysia | IBD | 1.49<br>(1.45 to 1.53) | < 0.001 |
| Adolescence | Prevalence | Both | Malaysia | MS | 0.09<br>(0 to 0.17) | 0.047 |

| | | | | | | |
|---|---|---|---|---|---|---|
| Adolescence | Prevalence | Female | Malaysia | MS | 0.04 (-0.02 to 0.09) | 0.185 |
| Adolescence | Prevalence | Male | Malaysia | MS | 0.11 (-0.08 to 0.31) | 0.263 |
| Adolescence | Prevalence | Both | Malaysia | Psoriasis | -0.61 (-0.66 to -0.57) | < 0.001 |
| Adolescence | Prevalence | Female | Malaysia | Psoriasis | -0.58 (-0.62 to -0.55) | < 0.001 |
| Adolescence | Prevalence | Male | Malaysia | Psoriasis | -0.64 (-0.69 to -0.59) | < 0.001 |
| Adolescence | Prevalence | Both | Malaysia | RHD | 0.07 (0.03 to 0.1) | < 0.001 |
| Adolescence | Prevalence | Female | Malaysia | RHD | 0.07 (0.03 to 0.11) | < 0.001 |
| Adolescence | Prevalence | Male | Malaysia | RHD | 0.08 (0.05 to 0.11) | < 0.001 |
| Adolescence | Prevalence | Both | Malaysia | RA | 0.47 (0.45 to 0.5) | < 0.001 |
| Adolescence | Prevalence | Female | Malaysia | RA | 0.49 (0.47 to 0.52) | < 0.001 |
| Adolescence | Prevalence | Male | Malaysia | RA | 0.46 (0.43 to 0.49) | < 0.001 |
| Adolescence | Prevalence | Both | Maldives | AA | -0.04 (-0.05 to -0.03) | < 0.001 |
| Adolescence | Prevalence | Female | Maldives | AA | 0 (0 to 0) | 0.008 |
| Adolescence | Prevalence | Male | Maldives | AA | 0 (0 to 0) | < 0.001 |
| Adolescence | Prevalence | Both | Maldives | T1D | 0.51 (0.43 to 0.58) | < 0.001 |
| Adolescence | Prevalence | Female | Maldives | T1D | 0.48 (0.43 to 0.52) | < 0.001 |
| Adolescence | Prevalence | Male | Maldives | T1D | 0.53 | < 0.001 |

| | | | | | | |
|---|---|---|---|---|---|---|
| | | | | | (0.5 to 0.57) | |
| Adolescence | Prevalence | Both | Maldives | IBD | 0.66<br>(0.53 to 0.79) | < 0.001 |
| Adolescence | Prevalence | Female | Maldives | IBD | 0.71<br>(0.56 to 0.85) | < 0.001 |
| Adolescence | Prevalence | Male | Maldives | IBD | 0.71<br>(0.6 to 0.83) | < 0.001 |
| Adolescence | Prevalence | Both | Maldives | MS | 0.02<br>(-0.12 to 0.16) | 0.768 |
| Adolescence | Prevalence | Female | Maldives | MS | -0.1<br>(-0.21 to 0.01) | 0.069 |
| Adolescence | Prevalence | Male | Maldives | MS | -0.02<br>(-0.16 to 0.11) | 0.734 |
| Adolescence | Prevalence | Both | Maldives | Psoriasis | -0.87<br>(-0.88 to -0.85) | < 0.001 |
| Adolescence | Prevalence | Female | Maldives | Psoriasis | -0.81<br>(-0.83 to -0.79) | < 0.001 |
| Adolescence | Prevalence | Male | Maldives | Psoriasis | -0.86<br>(-0.89 to -0.82) | < 0.001 |
| Adolescence | Prevalence | Both | Maldives | RHD | -0.02<br>(-0.04 to 0) | 0.029 |
| Adolescence | Prevalence | Female | Maldives | RHD | -0.01<br>(-0.04 to 0.02) | 0.522 |
| Adolescence | Prevalence | Male | Maldives | RHD | 0.01<br>(0 to 0.03) | 0.120 |
| Adolescence | Prevalence | Both | Maldives | RA | 0.68<br>(0.63 to 0.72) | < 0.001 |
| Adolescence | Prevalence | Female | Maldives | RA | 0.74<br>(0.67 to 0.81) | < 0.001 |
| Adolescence | Prevalence | Male | Maldives | RA | 0.72<br>(0.68 to 0.77) | < 0.001 |
| Adolescence | Prevalence | Both | Mali | AA | 0<br>(-0.01 to 0) | 0.200 |

| | | | | | | |
|---|---|---|---|---|---|---|
| Adolescence | Prevalence | Female | Mali | AA | 0<br>(0 to 0) | < 0.001 |
| Adolescence | Prevalence | Male | Mali | AA | 0<br>(0 to 0) | < 0.001 |
| Adolescence | Prevalence | Both | Mali | T1D | -0.06<br>(-0.08 to -0.04) | < 0.001 |
| Adolescence | Prevalence | Female | Mali | T1D | -0.07<br>(-0.1 to -0.04) | < 0.001 |
| Adolescence | Prevalence | Male | Mali | T1D | -0.05<br>(-0.07 to -0.02) | < 0.001 |
| Adolescence | Prevalence | Both | Mali | IBD | 0.25<br>(0.17 to 0.33) | < 0.001 |
| Adolescence | Prevalence | Female | Mali | IBD | 0.28<br>(0.18 to 0.39) | < 0.001 |
| Adolescence | Prevalence | Male | Mali | IBD | 0.2<br>(0.14 to 0.27) | < 0.001 |
| Adolescence | Prevalence | Both | Mali | MS | 0.07<br>(0.02 to 0.13) | 0.012 |
| Adolescence | Prevalence | Female | Mali | MS | 0.1<br>(0.05 to 0.15) | < 0.001 |
| Adolescence | Prevalence | Male | Mali | MS | 0.1<br>(-0.03 to 0.23) | 0.125 |
| Adolescence | Prevalence | Both | Mali | Psoriasis | -0.79<br>(-1.04 to -0.54) | < 0.001 |
| Adolescence | Prevalence | Female | Mali | Psoriasis | -0.79<br>(-0.96 to -0.62) | < 0.001 |
| Adolescence | Prevalence | Male | Mali | Psoriasis | -0.73<br>(-1.03 to -0.43) | < 0.001 |
| Adolescence | Prevalence | Both | Mali | RHD | 0.09<br>(-0.06 to 0.24) | 0.221 |
| Adolescence | Prevalence | Female | Mali | RHD | 0.01<br>(-0.19 to 0.21) | 0.910 |
| Adolescence | Prevalence | Male | Mali | RHD | 0.11 | 0.099 |

| | | | | | | |
|---|---|---|---|---|---|---|
| | | | | | (-0.02 to 0.25) | |
| Adolescence | Prevalence | Both | Mali | RA | 0.47 (0.45 to 0.48) | < 0.001 |
| Adolescence | Prevalence | Female | Mali | RA | 0.5 (0.47 to 0.54) | < 0.001 |
| Adolescence | Prevalence | Male | Mali | RA | 0.39 (0.35 to 0.43) | < 0.001 |
| Adolescence | Prevalence | Both | Malta | AA | 0 (0 to 0) | < 0.001 |
| Adolescence | Prevalence | Female | Malta | AA | 0 (0 to 0) | < 0.001 |
| Adolescence | Prevalence | Male | Malta | AA | 0 (0 to 0) | 0.005 |
| Adolescence | Prevalence | Both | Malta | T1D | 1.51 (1.25 to 1.76) | < 0.001 |
| Adolescence | Prevalence | Female | Malta | T1D | 1.5 (1.2 to 1.8) | < 0.001 |
| Adolescence | Prevalence | Male | Malta | T1D | 1.51 (1.3 to 1.72) | < 0.001 |
| Adolescence | Prevalence | Both | Malta | IBD | 0.3 (0.27 to 0.34) | < 0.001 |
| Adolescence | Prevalence | Female | Malta | IBD | 0.06 (0.02 to 0.09) | 0.002 |
| Adolescence | Prevalence | Male | Malta | IBD | 0.59 (0.54 to 0.63) | < 0.001 |
| Adolescence | Prevalence | Both | Malta | MS | 0.83 (0.72 to 0.94) | < 0.001 |
| Adolescence | Prevalence | Female | Malta | MS | 0.9 (0.81 to 0.99) | < 0.001 |
| Adolescence | Prevalence | Male | Malta | MS | 0.7 (0.59 to 0.81) | < 0.001 |
| Adolescence | Prevalence | Both | Malta | Psoriasis | -0.51 (-0.52 to -0.49) | < 0.001 |

| | | | | | | |
|---|---|---|---|---|---|---|
| Adolescence | Prevalence | Female | Malta | Psoriasis | -0.51<br>(-0.52 to -0.5) | < 0.001 |
| Adolescence | Prevalence | Male | Malta | Psoriasis | -0.51<br>(-0.52 to -0.51) | < 0.001 |
| Adolescence | Prevalence | Both | Malta | RHD | -0.32<br>(-0.38 to -0.25) | < 0.001 |
| Adolescence | Prevalence | Female | Malta | RHD | -0.3<br>(-0.37 to -0.23) | < 0.001 |
| Adolescence | Prevalence | Male | Malta | RHD | -0.3<br>(-0.38 to -0.22) | < 0.001 |
| Adolescence | Prevalence | Both | Malta | RA | 0.53<br>(0.5 to 0.56) | < 0.001 |
| Adolescence | Prevalence | Female | Malta | RA | 0.47<br>(0.43 to 0.51) | < 0.001 |
| Adolescence | Prevalence | Male | Malta | RA | 0.56<br>(0.52 to 0.6) | < 0.001 |
| Adolescence | Prevalence | Both | Marshall Islands | AA | 0.01<br>(0.01 to 0.01) | < 0.001 |
| Adolescence | Prevalence | Female | Marshall Islands | AA | 0<br>(0 to 0) | 0.008 |
| Adolescence | Prevalence | Male | Marshall Islands | AA | 0<br>(0 to 0) | < 0.001 |
| Adolescence | Prevalence | Both | Marshall Islands | T1D | 0.61<br>(0.6 to 0.63) | < 0.001 |
| Adolescence | Prevalence | Female | Marshall Islands | T1D | 0.59<br>(0.55 to 0.64) | < 0.001 |
| Adolescence | Prevalence | Male | Marshall Islands | T1D | 0.64<br>(0.58 to 0.7) | < 0.001 |
| Adolescence | Prevalence | Both | Marshall Islands | IBD | 0.18<br>(0.1 to 0.26) | < 0.001 |
| Adolescence | Prevalence | Female | Marshall Islands | IBD | 0.2<br>(0.11 to 0.29) | < 0.001 |
| Adolescence | Prevalence | Male | Marshall Islands | IBD | 0.2 | < 0.001 |

| | | | | | | |
|---|---|---|---|---|---|---|
| | | | | | (0.11 to 0.29) | |
| Adolescence | Prevalence | Both | Marshall Islands | MS | 0.06<br>(0.06 to 0.06) | < 0.001 |
| Adolescence | Prevalence | Female | Marshall Islands | MS | 0.06<br>(-0.09 to 0.2) | 0.430 |
| Adolescence | Prevalence | Male | Marshall Islands | MS | 0.08<br>(-0.02 to 0.18) | 0.133 |
| Adolescence | Prevalence | Both | Marshall Islands | Psoriasis | -0.57<br>(-0.58 to -0.55) | < 0.001 |
| Adolescence | Prevalence | Female | Marshall Islands | Psoriasis | -0.55<br>(-0.56 to -0.54) | < 0.001 |
| Adolescence | Prevalence | Male | Marshall Islands | Psoriasis | -0.59<br>(-0.63 to -0.56) | < 0.001 |
| Adolescence | Prevalence | Both | Marshall Islands | RHD | 0.08<br>(0.06 to 0.09) | < 0.001 |
| Adolescence | Prevalence | Female | Marshall Islands | RHD | 0.08<br>(0.02 to 0.14) | 0.005 |
| Adolescence | Prevalence | Male | Marshall Islands | RHD | 0.09<br>(0.07 to 0.11) | < 0.001 |
| Adolescence | Prevalence | Both | Marshall Islands | RA | 0.41<br>(0.37 to 0.44) | < 0.001 |
| Adolescence | Prevalence | Female | Marshall Islands | RA | 0.48<br>(0.47 to 0.48) | < 0.001 |
| Adolescence | Prevalence | Male | Marshall Islands | RA | 0.26<br>(0.17 to 0.34) | < 0.001 |
| Adolescence | Prevalence | Both | Mauritania | AA | 0.01<br>(0.01 to 0.01) | < 0.001 |
| Adolescence | Prevalence | Female | Mauritania | AA | 0<br>(0 to 0) | < 0.001 |
| Adolescence | Prevalence | Male | Mauritania | AA | 0<br>(0 to 0) | < 0.001 |
| Adolescence | Prevalence | Both | Mauritania | T1D | 0.09<br>(0.06 to 0.11) | < 0.001 |

| | | | | | | |
|---|---|---|---|---|---|---|
| Adolescence | Prevalence | Female | Mauritania | T1D | 0.09<br>(0.02 to 0.16) | 0.013 |
| Adolescence | Prevalence | Male | Mauritania | T1D | 0.08<br>(0.05 to 0.12) | < 0.001 |
| Adolescence | Prevalence | Both | Mauritania | IBD | 0.55<br>(0.48 to 0.62) | < 0.001 |
| Adolescence | Prevalence | Female | Mauritania | IBD | 0.54<br>(0.49 to 0.6) | < 0.001 |
| Adolescence | Prevalence | Male | Mauritania | IBD | 0.56<br>(0.49 to 0.63) | < 0.001 |
| Adolescence | Prevalence | Both | Mauritania | MS | 0.14<br>(0.06 to 0.22) | 0.001 |
| Adolescence | Prevalence | Female | Mauritania | MS | 0.11<br>(-0.01 to 0.22) | 0.068 |
| Adolescence | Prevalence | Male | Mauritania | MS | 0.08<br>(-0.06 to 0.23) | 0.264 |
| Adolescence | Prevalence | Both | Mauritania | Psoriasis | -0.69<br>(-0.83 to -0.56) | < 0.001 |
| Adolescence | Prevalence | Female | Mauritania | Psoriasis | -0.73<br>(-0.83 to -0.64) | < 0.001 |
| Adolescence | Prevalence | Male | Mauritania | Psoriasis | -0.6<br>(-0.75 to -0.46) | < 0.001 |
| Adolescence | Prevalence | Both | Mauritania | RHD | 0.05<br>(0.03 to 0.08) | < 0.001 |
| Adolescence | Prevalence | Female | Mauritania | RHD | 0.06<br>(0.04 to 0.09) | < 0.001 |
| Adolescence | Prevalence | Male | Mauritania | RHD | 0.03<br>(0 to 0.06) | 0.048 |
| Adolescence | Prevalence | Both | Mauritania | RA | 0.57<br>(0.52 to 0.62) | < 0.001 |
| Adolescence | Prevalence | Female | Mauritania | RA | 0.58<br>(0.54 to 0.62) | < 0.001 |
| Adolescence | Prevalence | Male | Mauritania | RA | 0.51 | < 0.001 |

| | | | | | | |
|---|---|---|---|---|---|---|
| | | | | | (0.45 to 0.57) | |
| Adolescence | Prevalence | Both | Mauritius | AA | 0<br>(0 to 0) | 0.606 |
| Adolescence | Prevalence | Female | Mauritius | AA | 0<br>(0 to 0) | 0.008 |
| Adolescence | Prevalence | Male | Mauritius | AA | 0<br>(0 to 0) | < 0.001 |
| Adolescence | Prevalence | Both | Mauritius | T1D | 0.55<br>(0.5 to 0.59) | < 0.001 |
| Adolescence | Prevalence | Female | Mauritius | T1D | 0.49<br>(0.46 to 0.52) | < 0.001 |
| Adolescence | Prevalence | Male | Mauritius | T1D | 0.59<br>(0.52 to 0.66) | < 0.001 |
| Adolescence | Prevalence | Both | Mauritius | IBD | 0.66<br>(0.61 to 0.7) | < 0.001 |
| Adolescence | Prevalence | Female | Mauritius | IBD | 0.76<br>(0.7 to 0.83) | < 0.001 |
| Adolescence | Prevalence | Male | Mauritius | IBD | 0.55<br>(0.43 to 0.67) | < 0.001 |
| Adolescence | Prevalence | Both | Mauritius | MS | 0.07<br>(-0.01 to 0.16) | 0.092 |
| Adolescence | Prevalence | Female | Mauritius | MS | 0.12<br>(0 to 0.24) | 0.052 |
| Adolescence | Prevalence | Male | Mauritius | MS | 0<br>(-0.21 to 0.22) | 0.987 |
| Adolescence | Prevalence | Both | Mauritius | Psoriasis | -0.53<br>(-0.55 to -0.51) | < 0.001 |
| Adolescence | Prevalence | Female | Mauritius | Psoriasis | -0.5<br>(-0.51 to -0.49) | < 0.001 |
| Adolescence | Prevalence | Male | Mauritius | Psoriasis | -0.56<br>(-0.62 to -0.5) | < 0.001 |
| Adolescence | Prevalence | Both | Mauritius | RHD | -0.03<br>(-0.06 to -0.01) | 0.018 |

| | | | | | | |
|---|---|---|---|---|---|---|
| Adolescence | Prevalence | Female | Mauritius | RHD | -0.03 (-0.07 to 0.02) | 0.278 |
| Adolescence | Prevalence | Male | Mauritius | RHD | -0.05 (-0.1 to 0) | 0.054 |
| Adolescence | Prevalence | Both | Mauritius | RA | 0.55 (0.52 to 0.57) | < 0.001 |
| Adolescence | Prevalence | Female | Mauritius | RA | 0.55 (0.49 to 0.61) | < 0.001 |
| Adolescence | Prevalence | Male | Mauritius | RA | 0.53 (0.49 to 0.57) | < 0.001 |
| Adolescence | Prevalence | Both | Mexico | AA | -0.02 (-0.02 to -0.02) | < 0.001 |
| Adolescence | Prevalence | Female | Mexico | AA | 0 (0 to 0) | < 0.001 |
| Adolescence | Prevalence | Male | Mexico | AA | 0 (0 to 0) | < 0.001 |
| Adolescence | Prevalence | Both | Mexico | T1D | 0.73 (0.7 to 0.75) | < 0.001 |
| Adolescence | Prevalence | Female | Mexico | T1D | 0.76 (0.74 to 0.79) | < 0.001 |
| Adolescence | Prevalence | Male | Mexico | T1D | 0.7 (0.64 to 0.75) | < 0.001 |
| Adolescence | Prevalence | Both | Mexico | IBD | 0.14 (0.09 to 0.2) | < 0.001 |
| Adolescence | Prevalence | Female | Mexico | IBD | -0.01 (-0.08 to 0.07) | 0.814 |
| Adolescence | Prevalence | Male | Mexico | IBD | 0.2 (0.15 to 0.24) | < 0.001 |
| Adolescence | Prevalence | Both | Mexico | MS | 0.45 (0.39 to 0.51) | < 0.001 |
| Adolescence | Prevalence | Female | Mexico | MS | 0.53 (0.46 to 0.59) | < 0.001 |
| Adolescence | Prevalence | Male | Mexico | MS | 0.4 | < 0.001 |

| | | | | | | |
|---|---|---|---|---|---|---|
| | | | | | (0.35 to 0.45) | |
| Adolescence | Prevalence | Both | Mexico | Psoriasis | -0.48 (-0.5 to -0.47) | < 0.001 |
| Adolescence | Prevalence | Female | Mexico | Psoriasis | -0.48 (-0.51 to -0.46) | < 0.001 |
| Adolescence | Prevalence | Male | Mexico | Psoriasis | -0.48 (-0.5 to -0.47) | < 0.001 |
| Adolescence | Prevalence | Both | Mexico | RHD | -0.44 (-0.49 to -0.38) | < 0.001 |
| Adolescence | Prevalence | Female | Mexico | RHD | -0.44 (-0.51 to -0.37) | < 0.001 |
| Adolescence | Prevalence | Male | Mexico | RHD | -0.42 (-0.46 to -0.37) | < 0.001 |
| Adolescence | Prevalence | Both | Mexico | RA | 0.78 (0.73 to 0.83) | < 0.001 |
| Adolescence | Prevalence | Female | Mexico | RA | 0.91 (0.84 to 0.98) | < 0.001 |
| Adolescence | Prevalence | Male | Mexico | RA | 0.58 (0.52 to 0.63) | < 0.001 |
| Adolescence | Prevalence | Both | Mongolia | AA | -0.01 (-0.01 to -0.01) | < 0.001 |
| Adolescence | Prevalence | Female | Mongolia | AA | 0 (0 to 0) | < 0.001 |
| Adolescence | Prevalence | Male | Mongolia | AA | 0 (0 to 0) | 1.000 |
| Adolescence | Prevalence | Both | Mongolia | T1D | 1.21 (1.17 to 1.25) | < 0.001 |
| Adolescence | Prevalence | Female | Mongolia | T1D | 1.17 (1.13 to 1.21) | < 0.001 |
| Adolescence | Prevalence | Male | Mongolia | T1D | 1.22 (1.19 to 1.26) | < 0.001 |
| Adolescence | Prevalence | Both | Mongolia | IBD | 0.18 (0.16 to 0.19) | < 0.001 |

| | | | | | | |
|---|---|---|---|---|---|---|
| Adolescence | Prevalence | Female | Mongolia | IBD | 0.13<br>(0.1 to 0.17) | < 0.001 |
| Adolescence | Prevalence | Male | Mongolia | IBD | 0.23<br>(0.2 to 0.26) | < 0.001 |
| Adolescence | Prevalence | Both | Mongolia | MS | 0.26<br>(0.18 to 0.33) | < 0.001 |
| Adolescence | Prevalence | Female | Mongolia | MS | 0.31<br>(0.26 to 0.36) | < 0.001 |
| Adolescence | Prevalence | Male | Mongolia | MS | 0.2<br>(0.14 to 0.27) | < 0.001 |
| Adolescence | Prevalence | Both | Mongolia | Psoriasis | -0.88<br>(-0.9 to -0.86) | < 0.001 |
| Adolescence | Prevalence | Female | Mongolia | Psoriasis | -0.88<br>(-0.91 to -0.85) | < 0.001 |
| Adolescence | Prevalence | Male | Mongolia | Psoriasis | -0.87<br>(-0.88 to -0.85) | < 0.001 |
| Adolescence | Prevalence | Both | Mongolia | RHD | -0.01<br>(-0.03 to 0.01) | 0.210 |
| Adolescence | Prevalence | Female | Mongolia | RHD | -0.02<br>(-0.1 to 0.06) | 0.654 |
| Adolescence | Prevalence | Male | Mongolia | RHD | 0.01<br>(-0.01 to 0.02) | 0.338 |
| Adolescence | Prevalence | Both | Mongolia | RA | 0.55<br>(0.52 to 0.58) | < 0.001 |
| Adolescence | Prevalence | Female | Mongolia | RA | 0.58<br>(0.5 to 0.66) | < 0.001 |
| Adolescence | Prevalence | Male | Mongolia | RA | 0.49<br>(0.42 to 0.56) | < 0.001 |
| Adolescence | Prevalence | Both | Montenegro | AA | -0.01<br>(-0.02 to -0.01) | < 0.001 |
| Adolescence | Prevalence | Female | Montenegro | AA | 0<br>(0 to 0) | < 0.001 |
| Adolescence | Prevalence | Male | Montenegro | AA | 0 | 1.000 |

| | | | | | | |
|---|---|---|---|---|---|---|
| | | | | | (0 to 0) | |
| Adolescence | Prevalence | Both | Montenegro | T1D | 1.42<br>(1.17 to 1.68) | < 0.001 |
| Adolescence | Prevalence | Female | Montenegro | T1D | 1.37<br>(1.02 to 1.73) | < 0.001 |
| Adolescence | Prevalence | Male | Montenegro | T1D | 1.51<br>(1.36 to 1.67) | < 0.001 |
| Adolescence | Prevalence | Both | Montenegro | IBD | 0.01<br>(-0.03 to 0.04) | 0.701 |
| Adolescence | Prevalence | Female | Montenegro | IBD | 0<br>(-0.02 to 0.02) | 0.681 |
| Adolescence | Prevalence | Male | Montenegro | IBD | 0.02<br>(-0.03 to 0.07) | 0.398 |
| Adolescence | Prevalence | Both | Montenegro | MS | 0.4<br>(0.36 to 0.44) | < 0.001 |
| Adolescence | Prevalence | Female | Montenegro | MS | 0.53<br>(0.45 to 0.6) | < 0.001 |
| Adolescence | Prevalence | Male | Montenegro | MS | 0.27<br>(0.13 to 0.41) | < 0.001 |
| Adolescence | Prevalence | Both | Montenegro | Psoriasis | -0.7<br>(-0.74 to -0.66) | < 0.001 |
| Adolescence | Prevalence | Female | Montenegro | Psoriasis | -0.7<br>(-0.75 to -0.64) | < 0.001 |
| Adolescence | Prevalence | Male | Montenegro | Psoriasis | -0.68<br>(-0.7 to -0.66) | < 0.001 |
| Adolescence | Prevalence | Both | Montenegro | RHD | -0.09<br>(-0.18 to -0.01) | 0.032 |
| Adolescence | Prevalence | Female | Montenegro | RHD | 0.01<br>(-0.1 to 0.12) | 0.817 |
| Adolescence | Prevalence | Male | Montenegro | RHD | -0.19<br>(-0.28 to -0.1) | < 0.001 |
| Adolescence | Prevalence | Both | Montenegro | RA | 0.38<br>(0.35 to 0.41) | < 0.001 |

| | | | | | | |
|---|---|---|---|---|---|---|
| Adolescence | Prevalence | Female | Montenegro | RA | 0.42 (0.38 to 0.45) | < 0.001 |
| Adolescence | Prevalence | Male | Montenegro | RA | 0.37 (0.33 to 0.4) | < 0.001 |
| Adolescence | Prevalence | Both | Morocco | AA | -0.02 (-0.02 to -0.02) | < 0.001 |
| Adolescence | Prevalence | Female | Morocco | AA | 0 (0 to 0) | 0.001 |
| Adolescence | Prevalence | Male | Morocco | AA | 0 (0 to 0) | 0.011 |
| Adolescence | Prevalence | Both | Morocco | T1D | 2.14 (2.11 to 2.16) | < 0.001 |
| Adolescence | Prevalence | Female | Morocco | T1D | 2.11 (2.07 to 2.15) | < 0.001 |
| Adolescence | Prevalence | Male | Morocco | T1D | 2.14 (2.11 to 2.17) | < 0.001 |
| Adolescence | Prevalence | Both | Morocco | IBD | 0.35 (0.31 to 0.38) | < 0.001 |
| Adolescence | Prevalence | Female | Morocco | IBD | 0.35 (0.29 to 0.4) | < 0.001 |
| Adolescence | Prevalence | Male | Morocco | IBD | 0.38 (0.3 to 0.45) | < 0.001 |
| Adolescence | Prevalence | Both | Morocco | MS | 0.44 (0.41 to 0.47) | < 0.001 |
| Adolescence | Prevalence | Female | Morocco | MS | 0.46 (0.43 to 0.49) | < 0.001 |
| Adolescence | Prevalence | Male | Morocco | MS | 0.48 (0.42 to 0.54) | < 0.001 |
| Adolescence | Prevalence | Both | Morocco | Psoriasis | -1.1 (-1.11 to -1.08) | < 0.001 |
| Adolescence | Prevalence | Female | Morocco | Psoriasis | -1.05 (-1.06 to -1.03) | < 0.001 |
| Adolescence | Prevalence | Male | Morocco | Psoriasis | -1.12 | < 0.001 |

| | | | | | | |
|---|---|---|---|---|---|---|
| | | | | | (-1.13 to -1.11) | |
| Adolescence | Prevalence | Both | Morocco | RHD | 0.06<br>(0.04 to 0.08) | < 0.001 |
| Adolescence | Prevalence | Female | Morocco | RHD | 0.08<br>(0.05 to 0.11) | < 0.001 |
| Adolescence | Prevalence | Male | Morocco | RHD | 0.06<br>(0.04 to 0.07) | < 0.001 |
| Adolescence | Prevalence | Both | Morocco | RA | 0.68<br>(0.65 to 0.7) | < 0.001 |
| Adolescence | Prevalence | Female | Morocco | RA | 0.69<br>(0.67 to 0.7) | < 0.001 |
| Adolescence | Prevalence | Male | Morocco | RA | 0.76<br>(0.68 to 0.84) | < 0.001 |
| Adolescence | Prevalence | Both | Mozambique | AA | 0<br>(-0.02 to 0.01) | 0.450 |
| Adolescence | Prevalence | Female | Mozambique | AA | 0<br>(0 to 0) | < 0.001 |
| Adolescence | Prevalence | Male | Mozambique | AA | 0<br>(0 to 0) | < 0.001 |
| Adolescence | Prevalence | Both | Mozambique | T1D | -0.05<br>(-0.08 to -0.02) | < 0.001 |
| Adolescence | Prevalence | Female | Mozambique | T1D | -0.05<br>(-0.09 to -0.01) | 0.010 |
| Adolescence | Prevalence | Male | Mozambique | T1D | -0.05<br>(-0.06 to -0.04) | < 0.001 |
| Adolescence | Prevalence | Both | Mozambique | IBD | 0.26<br>(0.14 to 0.38) | < 0.001 |
| Adolescence | Prevalence | Female | Mozambique | IBD | 0.29<br>(0.24 to 0.34) | < 0.001 |
| Adolescence | Prevalence | Male | Mozambique | IBD | 0.32<br>(0.28 to 0.36) | < 0.001 |
| Adolescence | Prevalence | Both | Mozambique | MS | 0.02<br>(-0.03 to 0.07) | 0.381 |

| | | | | | | |
|---|---|---|---|---|---|---|
| Adolescence | Prevalence | Female | Mozambique | MS | -0.01<br>(-0.11 to 0.1) | 0.922 |
| Adolescence | Prevalence | Male | Mozambique | MS | 0.07<br>(0 to 0.14) | 0.066 |
| Adolescence | Prevalence | Both | Mozambique | Psoriasis | -0.46<br>(-0.51 to -0.41) | < 0.001 |
| Adolescence | Prevalence | Female | Mozambique | Psoriasis | -0.49<br>(-0.59 to -0.39) | < 0.001 |
| Adolescence | Prevalence | Male | Mozambique | Psoriasis | -0.43<br>(-0.53 to -0.33) | < 0.001 |
| Adolescence | Prevalence | Both | Mozambique | RHD | 0.13<br>(0.07 to 0.2) | < 0.001 |
| Adolescence | Prevalence | Female | Mozambique | RHD | 0.12<br>(0.04 to 0.2) | 0.003 |
| Adolescence | Prevalence | Male | Mozambique | RHD | 0.11<br>(0.1 to 0.12) | < 0.001 |
| Adolescence | Prevalence | Both | Mozambique | RA | 0.22<br>(0.15 to 0.3) | < 0.001 |
| Adolescence | Prevalence | Female | Mozambique | RA | 0.21<br>(0.12 to 0.3) | < 0.001 |
| Adolescence | Prevalence | Male | Mozambique | RA | 0.34<br>(0.32 to 0.37) | < 0.001 |
| Adolescence | Prevalence | Both | Myanmar | AA | 0<br>(0 to 0) | 0.021 |
| Adolescence | Prevalence | Female | Myanmar | AA | 0<br>(0 to 0) | 0.008 |
| Adolescence | Prevalence | Male | Myanmar | AA | 0<br>(0 to 0) | < 0.001 |
| Adolescence | Prevalence | Both | Myanmar | T1D | 0.49<br>(0.48 to 0.51) | < 0.001 |
| Adolescence | Prevalence | Female | Myanmar | T1D | 0.45<br>(0.4 to 0.5) | < 0.001 |
| Adolescence | Prevalence | Male | Myanmar | T1D | 0.55 | < 0.001 |

| | | | | | | |
|---|---|---|---|---|---|---|
| | | | | | (0.51 to 0.59) | |
| Adolescence | Prevalence | Both | Myanmar | IBD | 0.62 (0.54 to 0.71) | < 0.001 |
| Adolescence | Prevalence | Female | Myanmar | IBD | 0.66 (0.47 to 0.85) | < 0.001 |
| Adolescence | Prevalence | Male | Myanmar | IBD | 0.64 (0.52 to 0.76) | < 0.001 |
| Adolescence | Prevalence | Both | Myanmar | MS | -0.06 (-0.1 to -0.02) | 0.005 |
| Adolescence | Prevalence | Female | Myanmar | MS | -0.1 (-0.13 to -0.06) | < 0.001 |
| Adolescence | Prevalence | Male | Myanmar | MS | -0.08 (-0.21 to 0.05) | 0.218 |
| Adolescence | Prevalence | Both | Myanmar | Psoriasis | -0.71 (-0.73 to -0.68) | < 0.001 |
| Adolescence | Prevalence | Female | Myanmar | Psoriasis | -0.68 (-0.69 to -0.66) | < 0.001 |
| Adolescence | Prevalence | Male | Myanmar | Psoriasis | -0.74 (-0.78 to -0.7) | < 0.001 |
| Adolescence | Prevalence | Both | Myanmar | RHD | -0.08 (-0.13 to -0.03) | 0.002 |
| Adolescence | Prevalence | Female | Myanmar | RHD | -0.14 (-0.18 to -0.09) | < 0.001 |
| Adolescence | Prevalence | Male | Myanmar | RHD | 0.03 (-0.01 to 0.07) | 0.191 |
| Adolescence | Prevalence | Both | Myanmar | RA | 0.41 (0.4 to 0.43) | < 0.001 |
| Adolescence | Prevalence | Female | Myanmar | RA | 0.45 (0.41 to 0.48) | < 0.001 |
| Adolescence | Prevalence | Male | Myanmar | RA | 0.38 (0.35 to 0.4) | < 0.001 |
| Adolescence | Prevalence | Both | Namibia | AA | 0 (-0.01 to 0) | < 0.001 |

| | | | | | | |
|---|---|---|---|---|---|---|
| Adolescence | Prevalence | Female | Namibia | AA | 0<br>(0 to 0) | < 0.001 |
| Adolescence | Prevalence | Male | Namibia | AA | 0<br>(0 to 0) | < 0.001 |
| Adolescence | Prevalence | Both | Namibia | T1D | 0.04<br>(0.02 to 0.06) | < 0.001 |
| Adolescence | Prevalence | Female | Namibia | T1D | 0.06<br>(0.03 to 0.08) | < 0.001 |
| Adolescence | Prevalence | Male | Namibia | T1D | 0.03<br>(0 to 0.06) | 0.089 |
| Adolescence | Prevalence | Both | Namibia | IBD | 0.47<br>(0.42 to 0.53) | < 0.001 |
| Adolescence | Prevalence | Female | Namibia | IBD | 0.47<br>(0.41 to 0.53) | < 0.001 |
| Adolescence | Prevalence | Male | Namibia | IBD | 0.54<br>(0.47 to 0.62) | < 0.001 |
| Adolescence | Prevalence | Both | Namibia | MS | 0.01<br>(-0.02 to 0.05) | 0.523 |
| Adolescence | Prevalence | Female | Namibia | MS | 0.03<br>(-0.07 to 0.13) | 0.612 |
| Adolescence | Prevalence | Male | Namibia | MS | 0<br>(-0.04 to 0.04) | 0.999 |
| Adolescence | Prevalence | Both | Namibia | Psoriasis | -0.58<br>(-0.64 to -0.53) | < 0.001 |
| Adolescence | Prevalence | Female | Namibia | Psoriasis | -0.61<br>(-0.69 to -0.53) | < 0.001 |
| Adolescence | Prevalence | Male | Namibia | Psoriasis | -0.54<br>(-0.6 to -0.49) | < 0.001 |
| Adolescence | Prevalence | Both | Namibia | RHD | 0<br>(-0.04 to 0.05) | 0.852 |
| Adolescence | Prevalence | Female | Namibia | RHD | 0<br>(-0.02 to 0.01) | 0.805 |
| Adolescence | Prevalence | Male | Namibia | RHD | 0.04 | 0.240 |

| | | | | | | |
|---|---|---|---|---|---|---|
| | | | | | (-0.03 to 0.1) | |
| Adolescence | Prevalence | Both | Namibia | RA | 0.25 (0.1 to 0.39) | 0.001 |
| Adolescence | Prevalence | Female | Namibia | RA | 0.2 (0.02 to 0.38) | 0.029 |
| Adolescence | Prevalence | Male | Namibia | RA | 0.38 (0.31 to 0.46) | < 0.001 |
| Adolescence | Prevalence | Both | Nepal | AA | 0 (0 to 0.01) | 0.019 |
| Adolescence | Prevalence | Female | Nepal | AA | 0 (0 to 0) | 0.008 |
| Adolescence | Prevalence | Male | Nepal | AA | 0 (0 to 0) | < 0.001 |
| Adolescence | Prevalence | Both | Nepal | T1D | 0.08 (0.08 to 0.09) | < 0.001 |
| Adolescence | Prevalence | Female | Nepal | T1D | 0.13 (0.11 to 0.16) | < 0.001 |
| Adolescence | Prevalence | Male | Nepal | T1D | 0.04 (-0.01 to 0.08) | 0.150 |
| Adolescence | Prevalence | Both | Nepal | IBD | 0.41 (0.34 to 0.48) | < 0.001 |
| Adolescence | Prevalence | Female | Nepal | IBD | 0.35 (0.27 to 0.42) | < 0.001 |
| Adolescence | Prevalence | Male | Nepal | IBD | 0.46 (0.44 to 0.48) | < 0.001 |
| Adolescence | Prevalence | Both | Nepal | MS | 0.02 (-0.04 to 0.08) | 0.606 |
| Adolescence | Prevalence | Female | Nepal | MS | 0.09 (-0.01 to 0.19) | 0.088 |
| Adolescence | Prevalence | Male | Nepal | MS | -0.02 (-0.13 to 0.09) | 0.738 |
| Adolescence | Prevalence | Both | Nepal | Psoriasis | -1.06 (-1.13 to -1) | < 0.001 |

| | | | | | | |
|---|---|---|---|---|---|---|
| Adolescence | Prevalence | Female | Nepal | Psoriasis | -1.06<br>(-1.13 to -1) | < 0.001 |
| Adolescence | Prevalence | Male | Nepal | Psoriasis | -1.07<br>(-1.15 to -0.99) | < 0.001 |
| Adolescence | Prevalence | Both | Nepal | RHD | 0.61<br>(0.53 to 0.7) | < 0.001 |
| Adolescence | Prevalence | Female | Nepal | RHD | 0.55<br>(0.34 to 0.76) | < 0.001 |
| Adolescence | Prevalence | Male | Nepal | RHD | 0.69<br>(0.56 to 0.81) | < 0.001 |
| Adolescence | Prevalence | Both | Nepal | RA | 0.98<br>(0.92 to 1.04) | < 0.001 |
| Adolescence | Prevalence | Female | Nepal | RA | 1<br>(0.9 to 1.1) | < 0.001 |
| Adolescence | Prevalence | Male | Nepal | RA | 0.97<br>(0.94 to 1) | < 0.001 |
| Adolescence | Prevalence | Both | Netherlands | AA | -0.01<br>(-0.01 to -0.01) | < 0.001 |
| Adolescence | Prevalence | Female | Netherlands | AA | -0.01<br>(-0.01 to -0.01) | < 0.001 |
| Adolescence | Prevalence | Male | Netherlands | AA | -0.01<br>(-0.01 to -0.01) | < 0.001 |
| Adolescence | Prevalence | Both | Netherlands | T1D | 1.72<br>(1.62 to 1.81) | < 0.001 |
| Adolescence | Prevalence | Female | Netherlands | T1D | 1.57<br>(1.53 to 1.62) | < 0.001 |
| Adolescence | Prevalence | Male | Netherlands | T1D | 1.85<br>(1.76 to 1.95) | < 0.001 |
| Adolescence | Prevalence | Both | Netherlands | IBD | -4.29<br>(-4.51 to -4.07) | < 0.001 |
| Adolescence | Prevalence | Female | Netherlands | IBD | -4.55<br>(-4.85 to -4.24) | < 0.001 |
| Adolescence | Prevalence | Male | Netherlands | IBD | -4.01 | < 0.001 |

| | | | | | | |
|---|---|---|---|---|---|---|
| | | | | | (-4.26 to -3.77) | |
| Adolescence | Prevalence | Both | Netherlands | MS | 0.18<br>(0.15 to 0.21) | < 0.001 |
| Adolescence | Prevalence | Female | Netherlands | MS | 0.22<br>(0.17 to 0.27) | < 0.001 |
| Adolescence | Prevalence | Male | Netherlands | MS | 0.1<br>(0.05 to 0.15) | < 0.001 |
| Adolescence | Prevalence | Both | Netherlands | Psoriasis | -0.33<br>(-0.34 to -0.32) | < 0.001 |
| Adolescence | Prevalence | Female | Netherlands | Psoriasis | -0.34<br>(-0.35 to -0.33) | < 0.001 |
| Adolescence | Prevalence | Male | Netherlands | Psoriasis | -0.33<br>(-0.34 to -0.32) | < 0.001 |
| Adolescence | Prevalence | Both | Netherlands | RHD | 0.04<br>(0.01 to 0.07) | 0.023 |
| Adolescence | Prevalence | Female | Netherlands | RHD | 0.05<br>(0 to 0.1) | 0.070 |
| Adolescence | Prevalence | Male | Netherlands | RHD | 0.05<br>(0 to 0.1) | 0.062 |
| Adolescence | Prevalence | Both | Netherlands | RA | 0.39<br>(0.36 to 0.41) | < 0.001 |
| Adolescence | Prevalence | Female | Netherlands | RA | 0.38<br>(0.37 to 0.4) | < 0.001 |
| Adolescence | Prevalence | Male | Netherlands | RA | 0.42<br>(0.39 to 0.45) | < 0.001 |
| Adolescence | Prevalence | Both | New Zealand | AA | 0<br>(0 to 0) | 0.022 |
| Adolescence | Prevalence | Female | New Zealand | AA | 0<br>(0 to 0) | < 0.001 |
| Adolescence | Prevalence | Male | New Zealand | AA | 0<br>(0 to 0) | < 0.001 |
| Adolescence | Prevalence | Both | New Zealand | T1D | 0.33<br>(0.31 to 0.36) | < 0.001 |

| | | | | | | |
|---|---|---|---|---|---|---|
| Adolescence | Prevalence | Female | New Zealand | T1D | 0.29 (0.26 to 0.33) | < 0.001 |
| Adolescence | Prevalence | Male | New Zealand | T1D | 0.37 (0.34 to 0.41) | < 0.001 |
| Adolescence | Prevalence | Both | New Zealand | IBD | 0.8 (0.71 to 0.89) | < 0.001 |
| Adolescence | Prevalence | Female | New Zealand | IBD | 0.65 (0.42 to 0.89) | < 0.001 |
| Adolescence | Prevalence | Male | New Zealand | IBD | 1.04 (1 to 1.08) | < 0.001 |
| Adolescence | Prevalence | Both | New Zealand | MS | -0.08 (-0.14 to -0.01) | 0.017 |
| Adolescence | Prevalence | Female | New Zealand | MS | -0.05 (-0.11 to 0.01) | 0.104 |
| Adolescence | Prevalence | Male | New Zealand | MS | -0.11 (-0.17 to -0.05) | < 0.001 |
| Adolescence | Prevalence | Both | New Zealand | Psoriasis | -0.4 (-0.42 to -0.39) | < 0.001 |
| Adolescence | Prevalence | Female | New Zealand | Psoriasis | -0.4 (-0.41 to -0.38) | < 0.001 |
| Adolescence | Prevalence | Male | New Zealand | Psoriasis | -0.41 (-0.43 to -0.39) | < 0.001 |
| Adolescence | Prevalence | Both | New Zealand | RHD | -0.1 (-0.19 to 0) | 0.055 |
| Adolescence | Prevalence | Female | New Zealand | RHD | 0.1 (-0.03 to 0.24) | 0.127 |
| Adolescence | Prevalence | Male | New Zealand | RHD | -0.35 (-0.44 to -0.26) | < 0.001 |
| Adolescence | Prevalence | Both | New Zealand | RA | 0.57 (0.55 to 0.59) | < 0.001 |
| Adolescence | Prevalence | Female | New Zealand | RA | 0.55 (0.53 to 0.58) | < 0.001 |
| Adolescence | Prevalence | Male | New Zealand | RA | 0.63 | < 0.001 |

| | | | | | | |
|---|---|---|---|---|---|---|
| | | | | | (0.61 to 0.66) | |
| Adolescence | Prevalence | Both | Nicaragua | AA | -0.03<br>(-0.03 to -0.03) | < 0.001 |
| Adolescence | Prevalence | Female | Nicaragua | AA | 0<br>(0 to 0) | < 0.001 |
| Adolescence | Prevalence | Male | Nicaragua | AA | 0<br>(0 to 0) | 1.000 |
| Adolescence | Prevalence | Both | Nicaragua | T1D | 0.73<br>(0.71 to 0.76) | < 0.001 |
| Adolescence | Prevalence | Female | Nicaragua | T1D | 0.75<br>(0.74 to 0.76) | < 0.001 |
| Adolescence | Prevalence | Male | Nicaragua | T1D | 0.75<br>(0.73 to 0.76) | < 0.001 |
| Adolescence | Prevalence | Both | Nicaragua | IBD | 0.36<br>(0.28 to 0.45) | < 0.001 |
| Adolescence | Prevalence | Female | Nicaragua | IBD | 0.35<br>(0.25 to 0.45) | < 0.001 |
| Adolescence | Prevalence | Male | Nicaragua | IBD | 0.34<br>(0.26 to 0.41) | < 0.001 |
| Adolescence | Prevalence | Both | Nicaragua | MS | 0.32<br>(0.2 to 0.45) | < 0.001 |
| Adolescence | Prevalence | Female | Nicaragua | MS | 0.43<br>(0.31 to 0.54) | < 0.001 |
| Adolescence | Prevalence | Male | Nicaragua | MS | 0.19<br>(0.15 to 0.23) | < 0.001 |
| Adolescence | Prevalence | Both | Nicaragua | Psoriasis | -0.6<br>(-0.62 to -0.58) | < 0.001 |
| Adolescence | Prevalence | Female | Nicaragua | Psoriasis | -0.6<br>(-0.62 to -0.58) | < 0.001 |
| Adolescence | Prevalence | Male | Nicaragua | Psoriasis | -0.6<br>(-0.64 to -0.56) | < 0.001 |
| Adolescence | Prevalence | Both | Nicaragua | RHD | 0.08<br>(0.05 to 0.11) | < 0.001 |

| | | | | | | |
|---|---|---|---|---|---|---|
| Adolescence | Prevalence | Female | Nicaragua | RHD | 0.07 (0.05 to 0.08) | < 0.001 |
| Adolescence | Prevalence | Male | Nicaragua | RHD | 0.09 (0.03 to 0.14) | 0.002 |
| Adolescence | Prevalence | Both | Nicaragua | RA | 0.66 (0.63 to 0.68) | < 0.001 |
| Adolescence | Prevalence | Female | Nicaragua | RA | 0.7 (0.66 to 0.74) | < 0.001 |
| Adolescence | Prevalence | Male | Nicaragua | RA | 0.73 (0.69 to 0.76) | < 0.001 |
| Adolescence | Prevalence | Both | Niger | AA | -0.02 (-0.02 to -0.02) | < 0.001 |
| Adolescence | Prevalence | Female | Niger | AA | 0 (0 to 0) | < 0.001 |
| Adolescence | Prevalence | Male | Niger | AA | 0 (0 to 0) | < 0.001 |
| Adolescence | Prevalence | Both | Niger | T1D | -0.03 (-0.03 to -0.02) | < 0.001 |
| Adolescence | Prevalence | Female | Niger | T1D | 0 (-0.02 to 0.02) | 0.759 |
| Adolescence | Prevalence | Male | Niger | T1D | -0.05 (-0.06 to -0.03) | < 0.001 |
| Adolescence | Prevalence | Both | Niger | IBD | 0.1 (0 to 0.19) | 0.045 |
| Adolescence | Prevalence | Female | Niger | IBD | 0.12 (0.05 to 0.19) | 0.001 |
| Adolescence | Prevalence | Male | Niger | IBD | 0.12 (0.08 to 0.16) | < 0.001 |
| Adolescence | Prevalence | Both | Niger | MS | 0.1 (0.04 to 0.16) | 0.001 |
| Adolescence | Prevalence | Female | Niger | MS | 0.08 (0.02 to 0.14) | 0.008 |
| Adolescence | Prevalence | Male | Niger | MS | 0.13 | 0.006 |

| | | | | | | |
|---|---|---|---|---|---|---|
| | | | | | (0.04 to 0.22) | |
| Adolescence | Prevalence | Both | Niger | Psoriasis | -0.35<br>(-0.46 to -0.24) | < 0.001 |
| Adolescence | Prevalence | Female | Niger | Psoriasis | -0.43<br>(-0.56 to -0.3) | < 0.001 |
| Adolescence | Prevalence | Male | Niger | Psoriasis | -0.29<br>(-0.49 to -0.09) | 0.004 |
| Adolescence | Prevalence | Both | Niger | RHD | 0.23<br>(0.21 to 0.25) | < 0.001 |
| Adolescence | Prevalence | Female | Niger | RHD | 0.24<br>(0.21 to 0.28) | < 0.001 |
| Adolescence | Prevalence | Male | Niger | RHD | 0.21<br>(0.18 to 0.23) | < 0.001 |
| Adolescence | Prevalence | Both | Niger | RA | 0.29<br>(0.26 to 0.31) | < 0.001 |
| Adolescence | Prevalence | Female | Niger | RA | 0.3<br>(0.26 to 0.35) | < 0.001 |
| Adolescence | Prevalence | Male | Niger | RA | 0.3<br>(0.28 to 0.32) | < 0.001 |
| Adolescence | Prevalence | Both | Nigeria | AA | -0.02<br>(-0.03 to -0.02) | < 0.001 |
| Adolescence | Prevalence | Female | Nigeria | AA | 0<br>(0 to 0) | < 0.001 |
| Adolescence | Prevalence | Male | Nigeria | AA | 0<br>(0 to 0) | < 0.001 |
| Adolescence | Prevalence | Both | Nigeria | T1D | 0.25<br>(0.23 to 0.26) | < 0.001 |
| Adolescence | Prevalence | Female | Nigeria | T1D | 0.24<br>(0.2 to 0.28) | < 0.001 |
| Adolescence | Prevalence | Male | Nigeria | T1D | 0.27<br>(0.24 to 0.31) | < 0.001 |
| Adolescence | Prevalence | Both | Nigeria | IBD | 0.49<br>(0.41 to 0.56) | < 0.001 |

| | | | | | | |
|---|---|---|---|---|---|---|
| Adolescence | Prevalence | Female | Nigeria | IBD | 0.6<br>(0.53 to 0.68) | < 0.001 |
| Adolescence | Prevalence | Male | Nigeria | IBD | 0.34<br>(0.27 to 0.42) | < 0.001 |
| Adolescence | Prevalence | Both | Nigeria | MS | 0.12<br>(0.09 to 0.15) | < 0.001 |
| Adolescence | Prevalence | Female | Nigeria | MS | 0.14<br>(0.08 to 0.19) | < 0.001 |
| Adolescence | Prevalence | Male | Nigeria | MS | 0.14<br>(0.02 to 0.26) | 0.020 |
| Adolescence | Prevalence | Both | Nigeria | Psoriasis | -1.17<br>(-1.2 to -1.14) | < 0.001 |
| Adolescence | Prevalence | Female | Nigeria | Psoriasis | -1.18<br>(-1.21 to -1.15) | < 0.001 |
| Adolescence | Prevalence | Male | Nigeria | Psoriasis | -1.17<br>(-1.22 to -1.13) | < 0.001 |
| Adolescence | Prevalence | Both | Nigeria | RHD | 0.25<br>(0.23 to 0.27) | < 0.001 |
| Adolescence | Prevalence | Female | Nigeria | RHD | 0.31<br>(0.28 to 0.33) | < 0.001 |
| Adolescence | Prevalence | Male | Nigeria | RHD | 0.18<br>(0.13 to 0.23) | < 0.001 |
| Adolescence | Prevalence | Both | Nigeria | RA | 0.26<br>(0.23 to 0.29) | < 0.001 |
| Adolescence | Prevalence | Female | Nigeria | RA | 0.04<br>(-0.06 to 0.14) | 0.455 |
| Adolescence | Prevalence | Male | Nigeria | RA | 0.87<br>(0.82 to 0.92) | < 0.001 |
| Adolescence | Prevalence | Both | North Macedonia | AA | -0.01<br>(-0.02 to -0.01) | < 0.001 |
| Adolescence | Prevalence | Female | North Macedonia | AA | 0<br>(0 to 0) | < 0.001 |
| Adolescence | Prevalence | Male | North Macedonia | AA | 0 | 1.000 |

| | | | | | | |
|---|---|---|---|---|---|---|
| | | | | | (0 to 0) | |
| Adolescence | Prevalence | Both | North Macedonia | T1D | 1.47 (1.39 to 1.54) | < 0.001 |
| Adolescence | Prevalence | Female | North Macedonia | T1D | 1.42 (1.33 to 1.51) | < 0.001 |
| Adolescence | Prevalence | Male | North Macedonia | T1D | 1.46 (1.42 to 1.5) | < 0.001 |
| Adolescence | Prevalence | Both | North Macedonia | IBD | 0.09 (0.07 to 0.11) | < 0.001 |
| Adolescence | Prevalence | Female | North Macedonia | IBD | 0.09 (0.04 to 0.13) | < 0.001 |
| Adolescence | Prevalence | Male | North Macedonia | IBD | 0.09 (0.05 to 0.13) | < 0.001 |
| Adolescence | Prevalence | Both | North Macedonia | MS | 0.52 (0.42 to 0.62) | < 0.001 |
| Adolescence | Prevalence | Female | North Macedonia | MS | 0.58 (0.45 to 0.71) | < 0.001 |
| Adolescence | Prevalence | Male | North Macedonia | MS | 0.48 (0.44 to 0.52) | < 0.001 |
| Adolescence | Prevalence | Both | North Macedonia | Psoriasis | -0.83 (-0.86 to -0.8) | < 0.001 |
| Adolescence | Prevalence | Female | North Macedonia | Psoriasis | -0.85 (-0.89 to -0.82) | < 0.001 |
| Adolescence | Prevalence | Male | North Macedonia | Psoriasis | -0.8 (-0.82 to -0.78) | < 0.001 |
| Adolescence | Prevalence | Both | North Macedonia | RHD | -1.13 (-1.19 to -1.06) | < 0.001 |
| Adolescence | Prevalence | Female | North Macedonia | RHD | -0.72 (-0.76 to -0.69) | < 0.001 |
| Adolescence | Prevalence | Male | North Macedonia | RHD | -1.44 (-1.57 to -1.32) | < 0.001 |
| Adolescence | Prevalence | Both | North Macedonia | RA | 0.59 (0.53 to 0.65) | < 0.001 |

| | | | | | | |
|---|---|---|---|---|---|---|
| Adolescence | Prevalence | Female | North Macedonia | RA | 0.61 (0.55 to 0.66) | < 0.001 |
| Adolescence | Prevalence | Male | North Macedonia | RA | 0.58 (0.52 to 0.63) | < 0.001 |
| Adolescence | Prevalence | Both | Northern Mariana Islands | AA | -0.14 (-0.38 to 0.09) | 0.232 |
| Adolescence | Prevalence | Female | Northern Mariana Islands | AA | 0 (0 to 0) | 0.008 |
| Adolescence | Prevalence | Male | Northern Mariana Islands | AA | 0 (0 to 0) | < 0.001 |
| Adolescence | Prevalence | Both | Northern Mariana Islands | T1D | 0.55 (0.51 to 0.59) | < 0.001 |
| Adolescence | Prevalence | Female | Northern Mariana Islands | T1D | 0.53 (0.48 to 0.57) | < 0.001 |
| Adolescence | Prevalence | Male | Northern Mariana Islands | T1D | 0.57 (0.53 to 0.62) | < 0.001 |
| Adolescence | Prevalence | Both | Northern Mariana Islands | IBD | -0.1 (-0.27 to 0.08) | 0.295 |
| Adolescence | Prevalence | Female | Northern Mariana Islands | IBD | 0 (-0.07 to 0.06) | 0.898 |
| Adolescence | Prevalence | Male | Northern Mariana Islands | IBD | -0.08 (-0.12 to -0.04) | < 0.001 |
| Adolescence | Prevalence | Both | Northern Mariana Islands | MS | -0.08 (-0.35 to 0.2) | 0.581 |
| Adolescence | Prevalence | Female | Northern Mariana Islands | MS | 0.06 (-0.05 to 0.17) | 0.291 |
| Adolescence | Prevalence | Male | Northern Mariana Islands | MS | 0.05 (-0.16 to 0.26) | 0.646 |
| Adolescence | Prevalence | Both | Northern Mariana Islands | Psoriasis | -0.24 (-0.35 to -0.13) | < 0.001 |
| Adolescence | Prevalence | Female | Northern Mariana Islands | Psoriasis | -0.13 (-0.15 to -0.11) | < 0.001 |
| Adolescence | Prevalence | Male | Northern Mariana Islands | Psoriasis | -0.17 | < 0.001 |

| | | | | | | |
|---|---|---|---|---|---|---|
| | | | | | (-0.2 to -0.14) | |
| Adolescence | Prevalence | Both | Northern Mariana Islands | RHD | 0.19<br>(0.13 to 0.25) | < 0.001 |
| Adolescence | Prevalence | Female | Northern Mariana Islands | RHD | 0.21<br>(0.19 to 0.24) | < 0.001 |
| Adolescence | Prevalence | Male | Northern Mariana Islands | RHD | 0.2<br>(0.19 to 0.21) | < 0.001 |
| Adolescence | Prevalence | Both | Northern Mariana Islands | RA | 0.18<br>(-0.15 to 0.52) | 0.281 |
| Adolescence | Prevalence | Female | Northern Mariana Islands | RA | 0.42<br>(0.39 to 0.44) | < 0.001 |
| Adolescence | Prevalence | Male | Northern Mariana Islands | RA | 0.24<br>(0.11 to 0.36) | < 0.001 |
| Adolescence | Prevalence | Both | Norway | AA | 0<br>(0 to 0) | < 0.001 |
| Adolescence | Prevalence | Female | Norway | AA | 0<br>(0 to 0) | < 0.001 |
| Adolescence | Prevalence | Male | Norway | AA | 0<br>(0 to 0) | < 0.001 |
| Adolescence | Prevalence | Both | Norway | T1D | 0.61<br>(0.49 to 0.73) | < 0.001 |
| Adolescence | Prevalence | Female | Norway | T1D | 0.66<br>(0.59 to 0.73) | < 0.001 |
| Adolescence | Prevalence | Male | Norway | T1D | 0.57<br>(0.4 to 0.74) | < 0.001 |
| Adolescence | Prevalence | Both | Norway | IBD | 1.15<br>(1.01 to 1.28) | < 0.001 |
| Adolescence | Prevalence | Female | Norway | IBD | 0.44<br>(0.2 to 0.68) | < 0.001 |
| Adolescence | Prevalence | Male | Norway | IBD | 1.98<br>(1.94 to 2.02) | < 0.001 |
| Adolescence | Prevalence | Both | Norway | MS | 0.82<br>(0.66 to 0.98) | < 0.001 |

| | | | | | | |
|---|---|---|---|---|---|---|
| Adolescence | Prevalence | Female | Norway | MS | 0.85<br>(0.66 to 1.04) | < 0.001 |
| Adolescence | Prevalence | Male | Norway | MS | 0.81<br>(0.63 to 0.98) | < 0.001 |
| Adolescence | Prevalence | Both | Norway | Psoriasis | -0.23<br>(-0.28 to -0.18) | < 0.001 |
| Adolescence | Prevalence | Female | Norway | Psoriasis | -0.23<br>(-0.29 to -0.16) | < 0.001 |
| Adolescence | Prevalence | Male | Norway | Psoriasis | -0.22<br>(-0.25 to -0.18) | < 0.001 |
| Adolescence | Prevalence | Both | Norway | RHD | 0.06<br>(-0.01 to 0.12) | 0.087 |
| Adolescence | Prevalence | Female | Norway | RHD | 0.2<br>(-0.08 to 0.49) | 0.164 |
| Adolescence | Prevalence | Male | Norway | RHD | 0<br>(-0.08 to 0.09) | 0.926 |
| Adolescence | Prevalence | Both | Norway | RA | 0.31<br>(0.26 to 0.35) | < 0.001 |
| Adolescence | Prevalence | Female | Norway | RA | 0.17<br>(-0.02 to 0.36) | 0.073 |
| Adolescence | Prevalence | Male | Norway | RA | 0.66<br>(0.59 to 0.74) | < 0.001 |
| Adolescence | Prevalence | Both | Oman | AA | 0.06<br>(0.02 to 0.11) | 0.004 |
| Adolescence | Prevalence | Female | Oman | AA | 0<br>(0 to 0) | 0.001 |
| Adolescence | Prevalence | Male | Oman | AA | 0<br>(0 to 0) | 0.011 |
| Adolescence | Prevalence | Both | Oman | T1D | 2.23<br>(2.21 to 2.24) | < 0.001 |
| Adolescence | Prevalence | Female | Oman | T1D | 2.21<br>(2.16 to 2.26) | < 0.001 |
| Adolescence | Prevalence | Male | Oman | T1D | 2.24 | < 0.001 |

| | | | | | | |
|---|---|---|---|---|---|---|
| | | | | | (2.21 to 2.28) | |
| Adolescence | Prevalence | Both | Oman | IBD | 0.27 (0.23 to 0.31) | < 0.001 |
| Adolescence | Prevalence | Female | Oman | IBD | 0.21 (0.13 to 0.29) | < 0.001 |
| Adolescence | Prevalence | Male | Oman | IBD | 0.3 (0.25 to 0.36) | < 0.001 |
| Adolescence | Prevalence | Both | Oman | MS | 0.56 (0.49 to 0.64) | < 0.001 |
| Adolescence | Prevalence | Female | Oman | MS | 0.46 (0.42 to 0.5) | < 0.001 |
| Adolescence | Prevalence | Male | Oman | MS | 0.48 (0.44 to 0.52) | < 0.001 |
| Adolescence | Prevalence | Both | Oman | Psoriasis | -1.68 (-1.71 to -1.64) | < 0.001 |
| Adolescence | Prevalence | Female | Oman | Psoriasis | -1.65 (-1.66 to -1.63) | < 0.001 |
| Adolescence | Prevalence | Male | Oman | Psoriasis | -1.74 (-1.76 to -1.72) | < 0.001 |
| Adolescence | Prevalence | Both | Oman | RHD | 0.33 (0.27 to 0.39) | < 0.001 |
| Adolescence | Prevalence | Female | Oman | RHD | 0.16 (0.05 to 0.27) | 0.004 |
| Adolescence | Prevalence | Male | Oman | RHD | 0.44 (0.3 to 0.58) | < 0.001 |
| Adolescence | Prevalence | Both | Oman | RA | 1.09 (1.01 to 1.16) | < 0.001 |
| Adolescence | Prevalence | Female | Oman | RA | 0.96 (0.91 to 1.01) | < 0.001 |
| Adolescence | Prevalence | Male | Oman | RA | 1.02 (0.96 to 1.09) | < 0.001 |
| Adolescence | Prevalence | Both | Pakistan | AA | 0.02 (0.01 to 0.02) | < 0.001 |

| | | | | | | |
|---|---|---|---|---|---|---|
| Adolescence | Prevalence | Female | Pakistan | AA | 0<br>(0 to 0) | < 0.001 |
| Adolescence | Prevalence | Male | Pakistan | AA | 0<br>(0 to 0) | < 0.001 |
| Adolescence | Prevalence | Both | Pakistan | T1D | 0.29<br>(0.28 to 0.31) | < 0.001 |
| Adolescence | Prevalence | Female | Pakistan | T1D | 0.32<br>(0.3 to 0.34) | < 0.001 |
| Adolescence | Prevalence | Male | Pakistan | T1D | 0.28<br>(0.2 to 0.37) | < 0.001 |
| Adolescence | Prevalence | Both | Pakistan | IBD | 0.42<br>(0.38 to 0.45) | < 0.001 |
| Adolescence | Prevalence | Female | Pakistan | IBD | 0.43<br>(0.34 to 0.51) | < 0.001 |
| Adolescence | Prevalence | Male | Pakistan | IBD | 0.39<br>(0.34 to 0.44) | < 0.001 |
| Adolescence | Prevalence | Both | Pakistan | MS | 0.16<br>(0.09 to 0.22) | < 0.001 |
| Adolescence | Prevalence | Female | Pakistan | MS | 0.16<br>(0.09 to 0.22) | < 0.001 |
| Adolescence | Prevalence | Male | Pakistan | MS | 0.07<br>(-0.01 to 0.14) | 0.074 |
| Adolescence | Prevalence | Both | Pakistan | Psoriasis | -0.59<br>(-0.61 to -0.58) | < 0.001 |
| Adolescence | Prevalence | Female | Pakistan | Psoriasis | -0.59<br>(-0.6 to -0.58) | < 0.001 |
| Adolescence | Prevalence | Male | Pakistan | Psoriasis | -0.6<br>(-0.62 to -0.59) | < 0.001 |
| Adolescence | Prevalence | Both | Pakistan | RHD | 0.22<br>(-0.24 to 0.67) | 0.353 |
| Adolescence | Prevalence | Female | Pakistan | RHD | 0.21<br>(-0.23 to 0.66) | 0.347 |
| Adolescence | Prevalence | Male | Pakistan | RHD | 0.21 | 0.376 |

| | | | | | | |
|---|---|---|---|---|---|---|
| | | | | | (-0.26 to 0.68) | |
| Adolescence | Prevalence | Both | Pakistan | RA | 0.28<br>(0.27 to 0.3) | < 0.001 |
| Adolescence | Prevalence | Female | Pakistan | RA | 0.33<br>(0.31 to 0.35) | < 0.001 |
| Adolescence | Prevalence | Male | Pakistan | RA | 0.23<br>(0.22 to 0.25) | < 0.001 |
| Adolescence | Prevalence | Both | Palestine | AA | 0.01<br>(0 to 0.01) | < 0.001 |
| Adolescence | Prevalence | Female | Palestine | AA | 0<br>(0 to 0) | 0.001 |
| Adolescence | Prevalence | Male | Palestine | AA | 0<br>(0 to 0) | 0.011 |
| Adolescence | Prevalence | Both | Palestine | T1D | 2.14<br>(2.09 to 2.2) | < 0.001 |
| Adolescence | Prevalence | Female | Palestine | T1D | 2.12<br>(2.08 to 2.15) | < 0.001 |
| Adolescence | Prevalence | Male | Palestine | T1D | 2.17<br>(2.07 to 2.26) | < 0.001 |
| Adolescence | Prevalence | Both | Palestine | IBD | 0.48<br>(0.42 to 0.55) | < 0.001 |
| Adolescence | Prevalence | Female | Palestine | IBD | 0.47<br>(0.41 to 0.53) | < 0.001 |
| Adolescence | Prevalence | Male | Palestine | IBD | 0.5<br>(0.46 to 0.53) | < 0.001 |
| Adolescence | Prevalence | Both | Palestine | MS | 0.36<br>(0.29 to 0.43) | < 0.001 |
| Adolescence | Prevalence | Female | Palestine | MS | 0.37<br>(0.3 to 0.43) | < 0.001 |
| Adolescence | Prevalence | Male | Palestine | MS | 0.33<br>(0.29 to 0.36) | < 0.001 |
| Adolescence | Prevalence | Both | Palestine | Psoriasis | -1.46<br>(-1.54 to -1.39) | < 0.001 |

| | | | | | | |
|---|---|---|---|---|---|---|
| Adolescence | Prevalence | Female | Palestine | Psoriasis | -1.44 (-1.51 to -1.36) | < 0.001 |
| Adolescence | Prevalence | Male | Palestine | Psoriasis | -1.51 (-1.58 to -1.44) | < 0.001 |
| Adolescence | Prevalence | Both | Palestine | RHD | 0.09 (0.07 to 0.1) | < 0.001 |
| Adolescence | Prevalence | Female | Palestine | RHD | 0.09 (0.09 to 0.1) | < 0.001 |
| Adolescence | Prevalence | Male | Palestine | RHD | 0.08 (0.07 to 0.09) | < 0.001 |
| Adolescence | Prevalence | Both | Palestine | RA | 0.49 (0.45 to 0.53) | < 0.001 |
| Adolescence | Prevalence | Female | Palestine | RA | 0.46 (0.42 to 0.5) | < 0.001 |
| Adolescence | Prevalence | Male | Palestine | RA | 0.51 (0.42 to 0.61) | < 0.001 |
| Adolescence | Prevalence | Both | Panama | AA | -0.01 (-0.01 to -0.01) | < 0.001 |
| Adolescence | Prevalence | Female | Panama | AA | 0 (0 to 0) | < 0.001 |
| Adolescence | Prevalence | Male | Panama | AA | 0 (0 to 0) | 1.000 |
| Adolescence | Prevalence | Both | Panama | T1D | 0.81 (0.8 to 0.82) | < 0.001 |
| Adolescence | Prevalence | Female | Panama | T1D | 0.82 (0.79 to 0.85) | < 0.001 |
| Adolescence | Prevalence | Male | Panama | T1D | 0.81 (0.8 to 0.83) | < 0.001 |
| Adolescence | Prevalence | Both | Panama | IBD | -0.11 (-0.16 to -0.05) | < 0.001 |
| Adolescence | Prevalence | Female | Panama | IBD | -0.24 (-0.38 to -0.1) | 0.001 |
| Adolescence | Prevalence | Male | Panama | IBD | -0.04 | 0.044 |

| | | | | | | |
|---|---|---|---|---|---|---|
| | | | | | (-0.08 to 0) | |
| Adolescence | Prevalence | Both | Panama | MS | 0.29<br>(0.13 to 0.44) | < 0.001 |
| Adolescence | Prevalence | Female | Panama | MS | 0.29<br>(0.17 to 0.42) | < 0.001 |
| Adolescence | Prevalence | Male | Panama | MS | 0.28<br>(0.2 to 0.37) | < 0.001 |
| Adolescence | Prevalence | Both | Panama | Psoriasis | -0.43<br>(-0.52 to -0.35) | < 0.001 |
| Adolescence | Prevalence | Female | Panama | Psoriasis | -0.43<br>(-0.53 to -0.33) | < 0.001 |
| Adolescence | Prevalence | Male | Panama | Psoriasis | -0.43<br>(-0.49 to -0.38) | < 0.001 |
| Adolescence | Prevalence | Both | Panama | RHD | -0.06<br>(-0.09 to -0.02) | 0.001 |
| Adolescence | Prevalence | Female | Panama | RHD | -0.05<br>(-0.13 to 0.02) | 0.158 |
| Adolescence | Prevalence | Male | Panama | RHD | -0.05<br>(-0.09 to -0.02) | 0.001 |
| Adolescence | Prevalence | Both | Panama | RA | 0.72<br>(0.68 to 0.77) | < 0.001 |
| Adolescence | Prevalence | Female | Panama | RA | 0.74<br>(0.68 to 0.81) | < 0.001 |
| Adolescence | Prevalence | Male | Panama | RA | 0.72<br>(0.69 to 0.75) | < 0.001 |
| Adolescence | Prevalence | Both | Papua New Guinea | AA | -0.01<br>(-0.02 to -0.01) | < 0.001 |
| Adolescence | Prevalence | Female | Papua New Guinea | AA | 0<br>(0 to 0) | 0.008 |
| Adolescence | Prevalence | Male | Papua New Guinea | AA | 0<br>(0 to 0) | < 0.001 |
| Adolescence | Prevalence | Both | Papua New Guinea | T1D | 0.54<br>(0.51 to 0.58) | < 0.001 |

| | | | | | | |
|---|---|---|---|---|---|---|
| Adolescence | Prevalence | Female | Papua New Guinea | T1D | 0.52<br>(0.51 to 0.54) | < 0.001 |
| Adolescence | Prevalence | Male | Papua New Guinea | T1D | 0.57<br>(0.51 to 0.63) | < 0.001 |
| Adolescence | Prevalence | Both | Papua New Guinea | IBD | 0.05<br>(-0.04 to 0.15) | 0.289 |
| Adolescence | Prevalence | Female | Papua New Guinea | IBD | 0.05<br>(-0.05 to 0.15) | 0.322 |
| Adolescence | Prevalence | Male | Papua New Guinea | IBD | 0.1<br>(-0.11 to 0.31) | 0.368 |
| Adolescence | Prevalence | Both | Papua New Guinea | MS | -0.01<br>(-0.16 to 0.13) | 0.846 |
| Adolescence | Prevalence | Female | Papua New Guinea | MS | -0.08<br>(-0.23 to 0.08) | 0.328 |
| Adolescence | Prevalence | Male | Papua New Guinea | MS | 0.13<br>(0.08 to 0.17) | < 0.001 |
| Adolescence | Prevalence | Both | Papua New Guinea | Psoriasis | -0.36<br>(-0.37 to -0.35) | < 0.001 |
| Adolescence | Prevalence | Female | Papua New Guinea | Psoriasis | -0.34<br>(-0.35 to -0.32) | < 0.001 |
| Adolescence | Prevalence | Male | Papua New Guinea | Psoriasis | -0.37<br>(-0.4 to -0.34) | < 0.001 |
| Adolescence | Prevalence | Both | Papua New Guinea | RHD | 0.16<br>(0.15 to 0.18) | < 0.001 |
| Adolescence | Prevalence | Female | Papua New Guinea | RHD | 0.17<br>(0.14 to 0.2) | < 0.001 |
| Adolescence | Prevalence | Male | Papua New Guinea | RHD | 0.17<br>(0.15 to 0.19) | < 0.001 |
| Adolescence | Prevalence | Both | Papua New Guinea | RA | 0.2<br>(0.18 to 0.22) | < 0.001 |
| Adolescence | Prevalence | Female | Papua New Guinea | RA | 0.27<br>(0.25 to 0.29) | < 0.001 |
| Adolescence | Prevalence | Male | Papua New Guinea | RA | 0.15 | 0.001 |

| | | | | | | |
|---|---|---|---|---|---|---|
| | | | | | (0.06 to 0.24) | |
| Adolescence | Prevalence | Both | Paraguay | AA | -0.02<br>(-0.02 to -0.02) | < 0.001 |
| Adolescence | Prevalence | Female | Paraguay | AA | 0<br>(0 to 0) | < 0.001 |
| Adolescence | Prevalence | Male | Paraguay | AA | 0<br>(0 to 0) | 1.000 |
| Adolescence | Prevalence | Both | Paraguay | T1D | 1.22<br>(1.18 to 1.26) | < 0.001 |
| Adolescence | Prevalence | Female | Paraguay | T1D | 1.3<br>(1.26 to 1.34) | < 0.001 |
| Adolescence | Prevalence | Male | Paraguay | T1D | 1.12<br>(1.06 to 1.18) | < 0.001 |
| Adolescence | Prevalence | Both | Paraguay | IBD | -0.64<br>(-0.7 to -0.58) | < 0.001 |
| Adolescence | Prevalence | Female | Paraguay | IBD | -0.6<br>(-0.65 to -0.54) | < 0.001 |
| Adolescence | Prevalence | Male | Paraguay | IBD | -0.67<br>(-0.73 to -0.62) | < 0.001 |
| Adolescence | Prevalence | Both | Paraguay | MS | 0.11<br>(0.06 to 0.16) | < 0.001 |
| Adolescence | Prevalence | Female | Paraguay | MS | 0.13<br>(0.08 to 0.18) | < 0.001 |
| Adolescence | Prevalence | Male | Paraguay | MS | 0.17<br>(0.07 to 0.27) | 0.001 |
| Adolescence | Prevalence | Both | Paraguay | Psoriasis | -0.15<br>(-0.17 to -0.13) | < 0.001 |
| Adolescence | Prevalence | Female | Paraguay | Psoriasis | -0.14<br>(-0.16 to -0.13) | < 0.001 |
| Adolescence | Prevalence | Male | Paraguay | Psoriasis | -0.14<br>(-0.17 to -0.12) | < 0.001 |
| Adolescence | Prevalence | Both | Paraguay | RHD | 0.01<br>(-0.03 to 0.05) | 0.582 |

| | | | | | | |
|---|---|---|---|---|---|---|
| Adolescence | Prevalence | Female | Paraguay | RHD | 0.03<br>(-0.01 to 0.06) | 0.137 |
| Adolescence | Prevalence | Male | Paraguay | RHD | -0.01<br>(-0.03 to 0) | 0.147 |
| Adolescence | Prevalence | Both | Paraguay | RA | 0.71<br>(0.68 to 0.73) | < 0.001 |
| Adolescence | Prevalence | Female | Paraguay | RA | 0.73<br>(0.7 to 0.76) | < 0.001 |
| Adolescence | Prevalence | Male | Paraguay | RA | 0.73<br>(0.7 to 0.76) | < 0.001 |
| Adolescence | Prevalence | Both | Peru | AA | -0.06<br>(-0.06 to -0.06) | < 0.001 |
| Adolescence | Prevalence | Female | Peru | AA | 0<br>(0 to 0) | < 0.001 |
| Adolescence | Prevalence | Male | Peru | AA | 0<br>(0 to 0) | 1.000 |
| Adolescence | Prevalence | Both | Peru | T1D | 0.67<br>(0.64 to 0.71) | < 0.001 |
| Adolescence | Prevalence | Female | Peru | T1D | 0.71<br>(0.67 to 0.75) | < 0.001 |
| Adolescence | Prevalence | Male | Peru | T1D | 0.68<br>(0.66 to 0.71) | < 0.001 |
| Adolescence | Prevalence | Both | Peru | IBD | -0.05<br>(-0.1 to 0) | 0.069 |
| Adolescence | Prevalence | Female | Peru | IBD | -0.04<br>(-0.1 to 0.02) | 0.220 |
| Adolescence | Prevalence | Male | Peru | IBD | -0.03<br>(-0.15 to 0.1) | 0.689 |
| Adolescence | Prevalence | Both | Peru | MS | 0.33<br>(0.23 to 0.44) | < 0.001 |
| Adolescence | Prevalence | Female | Peru | MS | 0.45<br>(0.32 to 0.58) | < 0.001 |
| Adolescence | Prevalence | Male | Peru | MS | 0.25 | 0.022 |

| | | | | | | |
|---|---|---|---|---|---|---|
| | | | | | (0.03 to 0.46) | |
| Adolescence | Prevalence | Both | Peru | Psoriasis | -0.61<br>(-0.7 to -0.53) | < 0.001 |
| Adolescence | Prevalence | Female | Peru | Psoriasis | -0.61<br>(-0.68 to -0.55) | < 0.001 |
| Adolescence | Prevalence | Male | Peru | Psoriasis | -0.6<br>(-0.67 to -0.52) | < 0.001 |
| Adolescence | Prevalence | Both | Peru | RHD | 0.04<br>(0.02 to 0.07) | 0.001 |
| Adolescence | Prevalence | Female | Peru | RHD | 0.1<br>(0.07 to 0.12) | < 0.001 |
| Adolescence | Prevalence | Male | Peru | RHD | 0.01<br>(-0.08 to 0.1) | 0.760 |
| Adolescence | Prevalence | Both | Peru | RA | 0.83<br>(0.8 to 0.85) | < 0.001 |
| Adolescence | Prevalence | Female | Peru | RA | 0.92<br>(0.9 to 0.93) | < 0.001 |
| Adolescence | Prevalence | Male | Peru | RA | 0.91<br>(0.8 to 1.02) | < 0.001 |
| Adolescence | Prevalence | Both | Philippines | AA | -0.01<br>(-0.01 to -0.01) | < 0.001 |
| Adolescence | Prevalence | Female | Philippines | AA | 0<br>(0 to 0) | < 0.001 |
| Adolescence | Prevalence | Male | Philippines | AA | 0<br>(0 to 0) | < 0.001 |
| Adolescence | Prevalence | Both | Philippines | T1D | 0.62<br>(0.55 to 0.69) | < 0.001 |
| Adolescence | Prevalence | Female | Philippines | T1D | 0.59<br>(0.52 to 0.66) | < 0.001 |
| Adolescence | Prevalence | Male | Philippines | T1D | 0.64<br>(0.55 to 0.72) | < 0.001 |
| Adolescence | Prevalence | Both | Philippines | IBD | 0.48<br>(0.36 to 0.6) | < 0.001 |

| | | | | | | |
|---|---|---|---|---|---|---|
| Adolescence | Prevalence | Female | Philippines | IBD | 0.25<br>(0.16 to 0.34) | < 0.001 |
| Adolescence | Prevalence | Male | Philippines | IBD | 0.6<br>(0.45 to 0.76) | < 0.001 |
| Adolescence | Prevalence | Both | Philippines | MS | 0.05<br>(-0.13 to 0.23) | 0.560 |
| Adolescence | Prevalence | Female | Philippines | MS | 0.04<br>(-0.06 to 0.14) | 0.434 |
| Adolescence | Prevalence | Male | Philippines | MS | 0.01<br>(-0.2 to 0.22) | 0.917 |
| Adolescence | Prevalence | Both | Philippines | Psoriasis | -0.29<br>(-0.33 to -0.25) | < 0.001 |
| Adolescence | Prevalence | Female | Philippines | Psoriasis | -0.26<br>(-0.29 to -0.22) | < 0.001 |
| Adolescence | Prevalence | Male | Philippines | Psoriasis | -0.3<br>(-0.35 to -0.25) | < 0.001 |
| Adolescence | Prevalence | Both | Philippines | RHD | 0.78<br>(0.73 to 0.83) | < 0.001 |
| Adolescence | Prevalence | Female | Philippines | RHD | 0.78<br>(0.73 to 0.82) | < 0.001 |
| Adolescence | Prevalence | Male | Philippines | RHD | 0.78<br>(0.68 to 0.87) | < 0.001 |
| Adolescence | Prevalence | Both | Philippines | RA | 0.02<br>(-0.03 to 0.07) | 0.463 |
| Adolescence | Prevalence | Female | Philippines | RA | 0.36<br>(0.34 to 0.38) | < 0.001 |
| Adolescence | Prevalence | Male | Philippines | RA | -0.43<br>(-0.46 to -0.4) | < 0.001 |
| Adolescence | Prevalence | Both | Poland | AA | 0<br>(0 to 0) | < 0.001 |
| Adolescence | Prevalence | Female | Poland | AA | 0<br>(0 to 0) | < 0.001 |
| Adolescence | Prevalence | Male | Poland | AA | 0 | < 0.001 |

| | | | | | | |
|---|---|---|---|---|---|---|
| | | | | | (0 to 0) | |
| Adolescence | Prevalence | Both | Poland | T1D | 1.98<br>(1.87 to 2.09) | < 0.001 |
| Adolescence | Prevalence | Female | Poland | T1D | 1.98<br>(1.87 to 2.09) | < 0.001 |
| Adolescence | Prevalence | Male | Poland | T1D | 1.98<br>(1.85 to 2.11) | < 0.001 |
| Adolescence | Prevalence | Both | Poland | IBD | 1.01<br>(0.95 to 1.07) | < 0.001 |
| Adolescence | Prevalence | Female | Poland | IBD | 0.85<br>(0.78 to 0.91) | < 0.001 |
| Adolescence | Prevalence | Male | Poland | IBD | 1.2<br>(1.14 to 1.25) | < 0.001 |
| Adolescence | Prevalence | Both | Poland | MS | 1.21<br>(1.13 to 1.29) | < 0.001 |
| Adolescence | Prevalence | Female | Poland | MS | 1.4<br>(1.31 to 1.49) | < 0.001 |
| Adolescence | Prevalence | Male | Poland | MS | 0.86<br>(0.78 to 0.94) | < 0.001 |
| Adolescence | Prevalence | Both | Poland | Psoriasis | -0.39<br>(-0.41 to -0.36) | < 0.001 |
| Adolescence | Prevalence | Female | Poland | Psoriasis | -0.34<br>(-0.4 to -0.29) | < 0.001 |
| Adolescence | Prevalence | Male | Poland | Psoriasis | -0.44<br>(-0.45 to -0.42) | < 0.001 |
| Adolescence | Prevalence | Both | Poland | RHD | -0.87<br>(-0.99 to -0.74) | < 0.001 |
| Adolescence | Prevalence | Female | Poland | RHD | -0.5<br>(-0.56 to -0.43) | < 0.001 |
| Adolescence | Prevalence | Male | Poland | RHD | -1.2<br>(-1.36 to -1.04) | < 0.001 |
| Adolescence | Prevalence | Both | Poland | RA | 0.4<br>(0.32 to 0.48) | < 0.001 |

| | | | | | | |
|---|---|---|---|---|---|---|
| Adolescence | Prevalence | Female | Poland | RA | 0.45 (0.38 to 0.53) | < 0.001 |
| Adolescence | Prevalence | Male | Poland | RA | 0.3 (0.21 to 0.39) | < 0.001 |
| Adolescence | Prevalence | Both | Portugal | AA | 0 (0 to 0) | < 0.001 |
| Adolescence | Prevalence | Female | Portugal | AA | 0 (0 to 0) | < 0.001 |
| Adolescence | Prevalence | Male | Portugal | AA | 0 (0 to 0) | 0.005 |
| Adolescence | Prevalence | Both | Portugal | T1D | 1.76 (1.7 to 1.82) | < 0.001 |
| Adolescence | Prevalence | Female | Portugal | T1D | 1.54 (1.48 to 1.6) | < 0.001 |
| Adolescence | Prevalence | Male | Portugal | T1D | 1.93 (1.87 to 1.99) | < 0.001 |
| Adolescence | Prevalence | Both | Portugal | IBD | 1.45 (1.33 to 1.57) | < 0.001 |
| Adolescence | Prevalence | Female | Portugal | IBD | 1.31 (1.22 to 1.39) | < 0.001 |
| Adolescence | Prevalence | Male | Portugal | IBD | 1.54 (1.44 to 1.64) | < 0.001 |
| Adolescence | Prevalence | Both | Portugal | MS | -0.46 (-0.5 to -0.43) | < 0.001 |
| Adolescence | Prevalence | Female | Portugal | MS | -0.41 (-0.48 to -0.33) | < 0.001 |
| Adolescence | Prevalence | Male | Portugal | MS | -0.55 (-0.61 to -0.49) | < 0.001 |
| Adolescence | Prevalence | Both | Portugal | Psoriasis | -0.51 (-0.52 to -0.5) | < 0.001 |
| Adolescence | Prevalence | Female | Portugal | Psoriasis | -0.51 (-0.52 to -0.49) | < 0.001 |
| Adolescence | Prevalence | Male | Portugal | Psoriasis | -0.52 | < 0.001 |

| | | | | | | |
|---|---|---|---|---|---|---|
| | | | | | (-0.53 to -0.51) | |
| Adolescence | Prevalence | Both | Portugal | RHD | -0.87<br>(-0.96 to -0.78) | < 0.001 |
| Adolescence | Prevalence | Female | Portugal | RHD | -1.01<br>(-1.08 to -0.93) | < 0.001 |
| Adolescence | Prevalence | Male | Portugal | RHD | -0.78<br>(-0.9 to -0.66) | < 0.001 |
| Adolescence | Prevalence | Both | Portugal | RA | 0.49<br>(0.42 to 0.55) | < 0.001 |
| Adolescence | Prevalence | Female | Portugal | RA | 0.51<br>(0.43 to 0.59) | < 0.001 |
| Adolescence | Prevalence | Male | Portugal | RA | 0.46<br>(0.43 to 0.49) | < 0.001 |
| Adolescence | Prevalence | Both | Puerto Rico | AA | 0<br>(0 to 0) | 0.101 |
| Adolescence | Prevalence | Female | Puerto Rico | AA | 0<br>(0 to 0) | < 0.001 |
| Adolescence | Prevalence | Male | Puerto Rico | AA | 0<br>(0 to 0) | 1.000 |
| Adolescence | Prevalence | Both | Puerto Rico | T1D | 0.55<br>(0.46 to 0.64) | < 0.001 |
| Adolescence | Prevalence | Female | Puerto Rico | T1D | 0.56<br>(0.46 to 0.65) | < 0.001 |
| Adolescence | Prevalence | Male | Puerto Rico | T1D | 0.54<br>(0.43 to 0.65) | < 0.001 |
| Adolescence | Prevalence | Both | Puerto Rico | IBD | -0.57<br>(-0.65 to -0.5) | < 0.001 |
| Adolescence | Prevalence | Female | Puerto Rico | IBD | -0.56<br>(-0.59 to -0.52) | < 0.001 |
| Adolescence | Prevalence | Male | Puerto Rico | IBD | -0.61<br>(-0.68 to -0.54) | < 0.001 |
| Adolescence | Prevalence | Both | Puerto Rico | MS | 0.36<br>(0.33 to 0.38) | < 0.001 |

| | | | | | | |
|---|---|---|---|---|---|---|
| Adolescence | Prevalence | Female | Puerto Rico | MS | 0.3<br>(0.26 to 0.34) | < 0.001 |
| Adolescence | Prevalence | Male | Puerto Rico | MS | 0.41<br>(0.32 to 0.5) | < 0.001 |
| Adolescence | Prevalence | Both | Puerto Rico | Psoriasis | -0.41<br>(-0.42 to -0.39) | < 0.001 |
| Adolescence | Prevalence | Female | Puerto Rico | Psoriasis | -0.41<br>(-0.43 to -0.4) | < 0.001 |
| Adolescence | Prevalence | Male | Puerto Rico | Psoriasis | -0.4<br>(-0.42 to -0.38) | < 0.001 |
| Adolescence | Prevalence | Both | Puerto Rico | RHD | -0.19<br>(-0.23 to -0.15) | < 0.001 |
| Adolescence | Prevalence | Female | Puerto Rico | RHD | -0.29<br>(-0.34 to -0.23) | < 0.001 |
| Adolescence | Prevalence | Male | Puerto Rico | RHD | -0.04<br>(-0.1 to 0.02) | 0.194 |
| Adolescence | Prevalence | Both | Puerto Rico | RA | 0.7<br>(0.69 to 0.72) | < 0.001 |
| Adolescence | Prevalence | Female | Puerto Rico | RA | 0.68<br>(0.6 to 0.76) | < 0.001 |
| Adolescence | Prevalence | Male | Puerto Rico | RA | 0.76<br>(0.73 to 0.78) | < 0.001 |
| Adolescence | Prevalence | Both | Qatar | AA | -0.04<br>(-0.08 to -0.01) | 0.011 |
| Adolescence | Prevalence | Female | Qatar | AA | 0<br>(0 to 0) | 0.001 |
| Adolescence | Prevalence | Male | Qatar | AA | 0<br>(0 to 0) | 0.011 |
| Adolescence | Prevalence | Both | Qatar | T1D | 2.23<br>(2.15 to 2.31) | < 0.001 |
| Adolescence | Prevalence | Female | Qatar | T1D | 2.23<br>(2.16 to 2.3) | < 0.001 |
| Adolescence | Prevalence | Male | Qatar | T1D | 2.25 | < 0.001 |

| | | | | | | |
|---|---|---|---|---|---|---|
| | | | | | (2.18 to 2.31) | |
| Adolescence | Prevalence | Both | Qatar | IBD | 0.4<br>(0.38 to 0.43) | < 0.001 |
| Adolescence | Prevalence | Female | Qatar | IBD | 0.41<br>(0.36 to 0.46) | < 0.001 |
| Adolescence | Prevalence | Male | Qatar | IBD | 0.46<br>(0.38 to 0.53) | < 0.001 |
| Adolescence | Prevalence | Both | Qatar | MS | 0.66<br>(0.53 to 0.78) | < 0.001 |
| Adolescence | Prevalence | Female | Qatar | MS | 0.79<br>(0.73 to 0.86) | < 0.001 |
| Adolescence | Prevalence | Male | Qatar | MS | 0.79<br>(0.7 to 0.87) | < 0.001 |
| Adolescence | Prevalence | Both | Qatar | Psoriasis | -1.19<br>(-1.22 to -1.17) | < 0.001 |
| Adolescence | Prevalence | Female | Qatar | Psoriasis | -1.14<br>(-1.15 to -1.13) | < 0.001 |
| Adolescence | Prevalence | Male | Qatar | Psoriasis | -1.23<br>(-1.24 to -1.21) | < 0.001 |
| Adolescence | Prevalence | Both | Qatar | RHD | -1.23<br>(-1.3 to -1.16) | < 0.001 |
| Adolescence | Prevalence | Female | Qatar | RHD | -0.52<br>(-0.55 to -0.48) | < 0.001 |
| Adolescence | Prevalence | Male | Qatar | RHD | -1.83<br>(-1.93 to -1.73) | < 0.001 |
| Adolescence | Prevalence | Both | Qatar | RA | 0.54<br>(0.47 to 0.6) | < 0.001 |
| Adolescence | Prevalence | Female | Qatar | RA | 0.64<br>(0.62 to 0.67) | < 0.001 |
| Adolescence | Prevalence | Male | Qatar | RA | 0.69<br>(0.58 to 0.79) | < 0.001 |
| Adolescence | Prevalence | Both | Republic of Korea | AA | -0.01<br>(-0.01 to 0) | < 0.001 |

| | | | | | | |
|---|---|---|---|---|---|---|
| Adolescence | Prevalence | Female | Republic of Korea | AA | 0<br>(0 to 0) | < 0.001 |
| Adolescence | Prevalence | Male | Republic of Korea | AA | 0<br>(0 to 0) | < 0.001 |
| Adolescence | Prevalence | Both | Republic of Korea | T1D | 0.03<br>(0.01 to 0.06) | 0.016 |
| Adolescence | Prevalence | Female | Republic of Korea | T1D | 0.07<br>(0.04 to 0.1) | < 0.001 |
| Adolescence | Prevalence | Male | Republic of Korea | T1D | 0<br>(-0.02 to 0.02) | 0.961 |
| Adolescence | Prevalence | Both | Republic of Korea | IBD | 3.15<br>(2.82 to 3.48) | < 0.001 |
| Adolescence | Prevalence | Female | Republic of Korea | IBD | 2.98<br>(2.62 to 3.33) | < 0.001 |
| Adolescence | Prevalence | Male | Republic of Korea | IBD | 3.31<br>(2.91 to 3.71) | < 0.001 |
| Adolescence | Prevalence | Both | Republic of Korea | MS | 0.02<br>(-0.05 to 0.09) | 0.575 |
| Adolescence | Prevalence | Female | Republic of Korea | MS | 0.07<br>(-0.03 to 0.17) | 0.174 |
| Adolescence | Prevalence | Male | Republic of Korea | MS | -0.02<br>(-0.07 to 0.03) | 0.441 |
| Adolescence | Prevalence | Both | Republic of Korea | Psoriasis | -0.47<br>(-0.49 to -0.45) | < 0.001 |
| Adolescence | Prevalence | Female | Republic of Korea | Psoriasis | -0.53<br>(-0.55 to -0.51) | < 0.001 |
| Adolescence | Prevalence | Male | Republic of Korea | Psoriasis | -0.39<br>(-0.4 to -0.37) | < 0.001 |
| Adolescence | Prevalence | Both | Republic of Korea | RHD | -0.47<br>(-0.51 to -0.43) | < 0.001 |
| Adolescence | Prevalence | Female | Republic of Korea | RHD | -0.6<br>(-0.66 to -0.55) | < 0.001 |
| Adolescence | Prevalence | Male | Republic of Korea | RHD | -0.34 | < 0.001 |

| | | | | | | |
|---|---|---|---|---|---|---|
| | | | | | (-0.38 to -0.3) | |
| Adolescence | Prevalence | Both | Republic of Korea | RA | 0.26<br>(0.24 to 0.29) | < 0.001 |
| Adolescence | Prevalence | Female | Republic of Korea | RA | 0.24<br>(0.22 to 0.26) | < 0.001 |
| Adolescence | Prevalence | Male | Republic of Korea | RA | 0.43<br>(0.39 to 0.47) | < 0.001 |
| Adolescence | Prevalence | Both | Republic of Moldova | AA | -0.01<br>(-0.01 to -0.01) | < 0.001 |
| Adolescence | Prevalence | Female | Republic of Moldova | AA | 0<br>(0 to 0) | < 0.001 |
| Adolescence | Prevalence | Male | Republic of Moldova | AA | 0<br>(0 to 0) | 1.000 |
| Adolescence | Prevalence | Both | Republic of Moldova | T1D | 1.4<br>(1.37 to 1.44) | < 0.001 |
| Adolescence | Prevalence | Female | Republic of Moldova | T1D | 1.42<br>(1.29 to 1.54) | < 0.001 |
| Adolescence | Prevalence | Male | Republic of Moldova | T1D | 1.4<br>(1.36 to 1.44) | < 0.001 |
| Adolescence | Prevalence | Both | Republic of Moldova | IBD | 0.75<br>(0.7 to 0.79) | < 0.001 |
| Adolescence | Prevalence | Female | Republic of Moldova | IBD | 0.71<br>(0.64 to 0.77) | < 0.001 |
| Adolescence | Prevalence | Male | Republic of Moldova | IBD | 0.79<br>(0.77 to 0.82) | < 0.001 |
| Adolescence | Prevalence | Both | Republic of Moldova | MS | 0.8<br>(0.74 to 0.86) | < 0.001 |
| Adolescence | Prevalence | Female | Republic of Moldova | MS | 0.69<br>(0.56 to 0.82) | < 0.001 |
| Adolescence | Prevalence | Male | Republic of Moldova | MS | 0.9<br>(0.79 to 1.01) | < 0.001 |
| Adolescence | Prevalence | Both | Republic of Moldova | Psoriasis | -0.67<br>(-0.69 to -0.64) | < 0.001 |

| | | | | | | |
|---|---|---|---|---|---|---|
| Adolescence | Prevalence | Female | Republic of Moldova | Psoriasis | -0.66 (-0.69 to -0.63) | < 0.001 |
| Adolescence | Prevalence | Male | Republic of Moldova | Psoriasis | -0.66 (-0.69 to -0.64) | < 0.001 |
| Adolescence | Prevalence | Both | Republic of Moldova | RHD | -2.09 (-2.23 to -1.96) | < 0.001 |
| Adolescence | Prevalence | Female | Republic of Moldova | RHD | -1.79 (-1.89 to -1.69) | < 0.001 |
| Adolescence | Prevalence | Male | Republic of Moldova | RHD | -2.4 (-2.54 to -2.27) | < 0.001 |
| Adolescence | Prevalence | Both | Republic of Moldova | RA | 0.68 (0.64 to 0.73) | < 0.001 |
| Adolescence | Prevalence | Female | Republic of Moldova | RA | 0.69 (0.64 to 0.74) | < 0.001 |
| Adolescence | Prevalence | Male | Republic of Moldova | RA | 0.69 (0.62 to 0.76) | < 0.001 |
| Adolescence | Prevalence | Both | Romania | AA | -0.01 (-0.01 to 0) | < 0.001 |
| Adolescence | Prevalence | Female | Romania | AA | 0 (0 to 0) | < 0.001 |
| Adolescence | Prevalence | Male | Romania | AA | 0 (0 to 0) | 1.000 |
| Adolescence | Prevalence | Both | Romania | T1D | 1.71 (1.58 to 1.84) | < 0.001 |
| Adolescence | Prevalence | Female | Romania | T1D | 1.82 (1.68 to 1.96) | < 0.001 |
| Adolescence | Prevalence | Male | Romania | T1D | 1.61 (1.49 to 1.72) | < 0.001 |
| Adolescence | Prevalence | Both | Romania | IBD | 0.51 (0.45 to 0.57) | < 0.001 |
| Adolescence | Prevalence | Female | Romania | IBD | 0.58 (0.52 to 0.64) | < 0.001 |
| Adolescence | Prevalence | Male | Romania | IBD | 0.44 | < 0.001 |

| | | | | | | |
|---|---|---|---|---|---|---|
| | | | | | (0.36 to 0.52) | |
| Adolescence | Prevalence | Both | Romania | MS | 0.27<br>(0.21 to 0.33) | < 0.001 |
| Adolescence | Prevalence | Female | Romania | MS | 0.25<br>(0.21 to 0.3) | < 0.001 |
| Adolescence | Prevalence | Male | Romania | MS | 0.22<br>(0.05 to 0.39) | 0.010 |
| Adolescence | Prevalence | Both | Romania | Psoriasis | -0.89<br>(-0.95 to -0.83) | < 0.001 |
| Adolescence | Prevalence | Female | Romania | Psoriasis | -0.91<br>(-1 to -0.82) | < 0.001 |
| Adolescence | Prevalence | Male | Romania | Psoriasis | -0.86<br>(-0.88 to -0.84) | < 0.001 |
| Adolescence | Prevalence | Both | Romania | RHD | -0.91<br>(-1.15 to -0.67) | < 0.001 |
| Adolescence | Prevalence | Female | Romania | RHD | -0.89<br>(-1.1 to -0.67) | < 0.001 |
| Adolescence | Prevalence | Male | Romania | RHD | -0.98<br>(-1.22 to -0.74) | < 0.001 |
| Adolescence | Prevalence | Both | Romania | RA | 0.6<br>(0.55 to 0.65) | < 0.001 |
| Adolescence | Prevalence | Female | Romania | RA | 0.61<br>(0.57 to 0.66) | < 0.001 |
| Adolescence | Prevalence | Male | Romania | RA | 0.58<br>(0.56 to 0.59) | < 0.001 |
| Adolescence | Prevalence | Both | Russian Federation | AA | -0.01<br>(-0.01 to -0.01) | < 0.001 |
| Adolescence | Prevalence | Female | Russian Federation | AA | 0<br>(0 to 0) | < 0.001 |
| Adolescence | Prevalence | Male | Russian Federation | AA | 0<br>(0 to 0) | < 0.001 |
| Adolescence | Prevalence | Both | Russian Federation | T1D | 1.38<br>(1.35 to 1.42) | < 0.001 |

| | | | | | | |
|---|---|---|---|---|---|---|
| Adolescence | Prevalence | Female | Russian Federation | T1D | 1.54 (1.33 to 1.75) | < 0.001 |
| Adolescence | Prevalence | Male | Russian Federation | T1D | 1.16 (1.03 to 1.29) | < 0.001 |
| Adolescence | Prevalence | Both | Russian Federation | IBD | 0.02 (0 to 0.03) | 0.019 |
| Adolescence | Prevalence | Female | Russian Federation | IBD | -0.04 (-0.08 to 0) | 0.078 |
| Adolescence | Prevalence | Male | Russian Federation | IBD | 0.08 (0.06 to 0.11) | < 0.001 |
| Adolescence | Prevalence | Both | Russian Federation | MS | -0.24 (-0.28 to -0.2) | < 0.001 |
| Adolescence | Prevalence | Female | Russian Federation | MS | -0.2 (-0.24 to -0.15) | < 0.001 |
| Adolescence | Prevalence | Male | Russian Federation | MS | -0.26 (-0.39 to -0.12) | < 0.001 |
| Adolescence | Prevalence | Both | Russian Federation | Psoriasis | -0.39 (-0.42 to -0.36) | < 0.001 |
| Adolescence | Prevalence | Female | Russian Federation | Psoriasis | -0.37 (-0.4 to -0.34) | < 0.001 |
| Adolescence | Prevalence | Male | Russian Federation | Psoriasis | -0.42 (-0.44 to -0.4) | < 0.001 |
| Adolescence | Prevalence | Both | Russian Federation | RHD | -0.35 (-0.4 to -0.29) | < 0.001 |
| Adolescence | Prevalence | Female | Russian Federation | RHD | -0.18 (-0.24 to -0.12) | < 0.001 |
| Adolescence | Prevalence | Male | Russian Federation | RHD | -0.51 (-0.57 to -0.45) | < 0.001 |
| Adolescence | Prevalence | Both | Russian Federation | RA | 0.47 (0.43 to 0.5) | < 0.001 |
| Adolescence | Prevalence | Female | Russian Federation | RA | 0.5 (0.45 to 0.56) | < 0.001 |
| Adolescence | Prevalence | Male | Russian Federation | RA | 0.41 | < 0.001 |

| | | | | | | |
|---|---|---|---|---|---|---|
| | | | | | (0.37 to 0.45) | |
| Adolescence | Prevalence | Both | Rwanda | AA | -0.01<br>(-0.02 to 0.01) | 0.293 |
| Adolescence | Prevalence | Female | Rwanda | AA | 0<br>(0 to 0) | < 0.001 |
| Adolescence | Prevalence | Male | Rwanda | AA | 0<br>(0 to 0) | < 0.001 |
| Adolescence | Prevalence | Both | Rwanda | T1D | -0.01<br>(-0.04 to 0.03) | 0.705 |
| Adolescence | Prevalence | Female | Rwanda | T1D | -0.01<br>(-0.03 to 0.01) | 0.445 |
| Adolescence | Prevalence | Male | Rwanda | T1D | 0.01<br>(-0.02 to 0.04) | 0.615 |
| Adolescence | Prevalence | Both | Rwanda | IBD | 0.41<br>(0.31 to 0.51) | < 0.001 |
| Adolescence | Prevalence | Female | Rwanda | IBD | 0.43<br>(0.35 to 0.5) | < 0.001 |
| Adolescence | Prevalence | Male | Rwanda | IBD | 0.36<br>(0.27 to 0.46) | < 0.001 |
| Adolescence | Prevalence | Both | Rwanda | MS | 0<br>(-0.08 to 0.08) | 0.998 |
| Adolescence | Prevalence | Female | Rwanda | MS | 0.03<br>(-0.01 to 0.06) | 0.116 |
| Adolescence | Prevalence | Male | Rwanda | MS | 0.02<br>(0.02 to 0.02) | < 0.001 |
| Adolescence | Prevalence | Both | Rwanda | Psoriasis | -0.43<br>(-0.47 to -0.39) | < 0.001 |
| Adolescence | Prevalence | Female | Rwanda | Psoriasis | -0.47<br>(-0.51 to -0.43) | < 0.001 |
| Adolescence | Prevalence | Male | Rwanda | Psoriasis | -0.39<br>(-0.51 to -0.28) | < 0.001 |
| Adolescence | Prevalence | Both | Rwanda | RHD | 0.07<br>(0.05 to 0.09) | < 0.001 |

| | | | | | | |
|---|---|---|---|---|---|---|
| Adolescence | Prevalence | Female | Rwanda | RHD | 0.08<br>(0.02 to 0.13) | 0.006 |
| Adolescence | Prevalence | Male | Rwanda | RHD | 0.06<br>(0.05 to 0.07) | < 0.001 |
| Adolescence | Prevalence | Both | Rwanda | RA | 0.29<br>(0.23 to 0.34) | < 0.001 |
| Adolescence | Prevalence | Female | Rwanda | RA | 0.25<br>(0.17 to 0.32) | < 0.001 |
| Adolescence | Prevalence | Male | Rwanda | RA | 0.38<br>(0.25 to 0.51) | < 0.001 |
| Adolescence | Prevalence | Both | Saint Kitts and Nevis | AA | 0<br>(0 to 0.01) | < 0.001 |
| Adolescence | Prevalence | Female | Saint Kitts and Nevis | AA | 0<br>(0 to 0) | < 0.001 |
| Adolescence | Prevalence | Male | Saint Kitts and Nevis | AA | 0<br>(0 to 0) | 1.000 |
| Adolescence | Prevalence | Both | Saint Kitts and Nevis | T1D | 1.07<br>(1.04 to 1.1) | < 0.001 |
| Adolescence | Prevalence | Female | Saint Kitts and Nevis | T1D | 1<br>(0.99 to 1.01) | < 0.001 |
| Adolescence | Prevalence | Male | Saint Kitts and Nevis | T1D | 1.14<br>(1.09 to 1.19) | < 0.001 |
| Adolescence | Prevalence | Both | Saint Kitts and Nevis | IBD | 0.03<br>(-0.01 to 0.08) | 0.096 |
| Adolescence | Prevalence | Female | Saint Kitts and Nevis | IBD | 0.03<br>(-0.01 to 0.07) | 0.126 |
| Adolescence | Prevalence | Male | Saint Kitts and Nevis | IBD | 0.03<br>(-0.02 to 0.09) | 0.228 |
| Adolescence | Prevalence | Both | Saint Kitts and Nevis | MS | 0.21<br>(0.14 to 0.29) | < 0.001 |
| Adolescence | Prevalence | Female | Saint Kitts and Nevis | MS | 0.17<br>(0.13 to 0.21) | < 0.001 |
| Adolescence | Prevalence | Male | Saint Kitts and Nevis | MS | 0.17 | 0.001 |

| | | | | | | |
|---|---|---|---|---|---|---|
| | | | | | (0.07 to 0.27) | |
| Adolescence | Prevalence | Both | Saint Kitts and Nevis | Psoriasis | -0.48 (-0.49 to -0.48) | < 0.001 |
| Adolescence | Prevalence | Female | Saint Kitts and Nevis | Psoriasis | -0.49 (-0.5 to -0.48) | < 0.001 |
| Adolescence | Prevalence | Male | Saint Kitts and Nevis | Psoriasis | -0.48 (-0.48 to -0.47) | < 0.001 |
| Adolescence | Prevalence | Both | Saint Kitts and Nevis | RHD | -1.67 (-1.78 to -1.56) | < 0.001 |
| Adolescence | Prevalence | Female | Saint Kitts and Nevis | RHD | -1.96 (-2.11 to -1.81) | < 0.001 |
| Adolescence | Prevalence | Male | Saint Kitts and Nevis | RHD | -1.09 (-1.21 to -0.97) | < 0.001 |
| Adolescence | Prevalence | Both | Saint Kitts and Nevis | RA | 0.69 (0.65 to 0.72) | < 0.001 |
| Adolescence | Prevalence | Female | Saint Kitts and Nevis | RA | 0.65 (0.61 to 0.68) | < 0.001 |
| Adolescence | Prevalence | Male | Saint Kitts and Nevis | RA | 0.75 (0.72 to 0.78) | < 0.001 |
| Adolescence | Prevalence | Both | Saint Lucia | AA | -0.02 (-0.03 to -0.02) | < 0.001 |
| Adolescence | Prevalence | Female | Saint Lucia | AA | 0 (0 to 0) | < 0.001 |
| Adolescence | Prevalence | Male | Saint Lucia | AA | 0 (0 to 0) | 1.000 |
| Adolescence | Prevalence | Both | Saint Lucia | T1D | 1.09 (1.08 to 1.1) | < 0.001 |
| Adolescence | Prevalence | Female | Saint Lucia | T1D | 1.01 (1 to 1.03) | < 0.001 |
| Adolescence | Prevalence | Male | Saint Lucia | T1D | 1.2 (1.17 to 1.23) | < 0.001 |
| Adolescence | Prevalence | Both | Saint Lucia | IBD | 0.19 (0.15 to 0.24) | < 0.001 |

| | | | | | | |
|---|---|---|---|---|---|---|
| Adolescence | Prevalence | Female | Saint Lucia | IBD | 0.19<br>(0.17 to 0.21) | < 0.001 |
| Adolescence | Prevalence | Male | Saint Lucia | IBD | 0.2<br>(0.12 to 0.28) | < 0.001 |
| Adolescence | Prevalence | Both | Saint Lucia | MS | 0.25<br>(0.17 to 0.32) | < 0.001 |
| Adolescence | Prevalence | Female | Saint Lucia | MS | 0.29<br>(0.24 to 0.35) | < 0.001 |
| Adolescence | Prevalence | Male | Saint Lucia | MS | 0.31<br>(0.18 to 0.44) | < 0.001 |
| Adolescence | Prevalence | Both | Saint Lucia | Psoriasis | -0.5<br>(-0.51 to -0.48) | < 0.001 |
| Adolescence | Prevalence | Female | Saint Lucia | Psoriasis | -0.49<br>(-0.5 to -0.47) | < 0.001 |
| Adolescence | Prevalence | Male | Saint Lucia | Psoriasis | -0.5<br>(-0.51 to -0.49) | < 0.001 |
| Adolescence | Prevalence | Both | Saint Lucia | RHD | -0.01<br>(-0.02 to 0) | 0.007 |
| Adolescence | Prevalence | Female | Saint Lucia | RHD | 0.03<br>(-0.01 to 0.07) | 0.178 |
| Adolescence | Prevalence | Male | Saint Lucia | RHD | -0.04<br>(-0.05 to -0.02) | < 0.001 |
| Adolescence | Prevalence | Both | Saint Lucia | RA | 0.7<br>(0.68 to 0.73) | < 0.001 |
| Adolescence | Prevalence | Female | Saint Lucia | RA | 0.72<br>(0.68 to 0.75) | < 0.001 |
| Adolescence | Prevalence | Male | Saint Lucia | RA | 0.77<br>(0.75 to 0.79) | < 0.001 |
| Adolescence | Prevalence | Both | Saint Vincent and the Grenadines | AA | -0.02<br>(-0.02 to -0.01) | < 0.001 |
| Adolescence | Prevalence | Female | Saint Vincent and the Grenadines | AA | 0<br>(0 to 0) | < 0.001 |
| Adolescence | Prevalence | Male | Saint Vincent and the Grenadines | AA | 0 | 1.000 |

| | | | | | | |
|---|---|---|---|---|---|---|
| | | | | | (0 to 0) | |
| Adolescence | Prevalence | Both | Saint Vincent and the Grenadines | T1D | 1.1<br>(1.09 to 1.1) | < 0.001 |
| Adolescence | Prevalence | Female | Saint Vincent and the Grenadines | T1D | 1.03<br>(1.03 to 1.04) | < 0.001 |
| Adolescence | Prevalence | Male | Saint Vincent and the Grenadines | T1D | 1.17<br>(1.16 to 1.19) | < 0.001 |
| Adolescence | Prevalence | Both | Saint Vincent and the Grenadines | IBD | 0.07<br>(0.02 to 0.13) | 0.008 |
| Adolescence | Prevalence | Female | Saint Vincent and the Grenadines | IBD | 0.12<br>(0.09 to 0.15) | < 0.001 |
| Adolescence | Prevalence | Male | Saint Vincent and the Grenadines | IBD | 0.02<br>(-0.07 to 0.11) | 0.668 |
| Adolescence | Prevalence | Both | Saint Vincent and the Grenadines | MS | 0.2<br>(0.14 to 0.26) | < 0.001 |
| Adolescence | Prevalence | Female | Saint Vincent and the Grenadines | MS | 0.23<br>(0.16 to 0.3) | < 0.001 |
| Adolescence | Prevalence | Male | Saint Vincent and the Grenadines | MS | 0.12<br>(-0.05 to 0.3) | 0.170 |
| Adolescence | Prevalence | Both | Saint Vincent and the Grenadines | Psoriasis | -0.5<br>(-0.51 to -0.49) | < 0.001 |
| Adolescence | Prevalence | Female | Saint Vincent and the Grenadines | Psoriasis | -0.5<br>(-0.52 to -0.48) | < 0.001 |
| Adolescence | Prevalence | Male | Saint Vincent and the Grenadines | Psoriasis | -0.49<br>(-0.5 to -0.48) | < 0.001 |
| Adolescence | Prevalence | Both | Saint Vincent and the Grenadines | RHD | -0.06<br>(-0.07 to -0.05) | < 0.001 |
| Adolescence | Prevalence | Female | Saint Vincent and the Grenadines | RHD | -0.02<br>(-0.05 to 0) | 0.040 |
| Adolescence | Prevalence | Male | Saint Vincent and the Grenadines | RHD | -0.11<br>(-0.24 to 0.01) | 0.070 |
| Adolescence | Prevalence | Both | Saint Vincent and the Grenadines | RA | 0.7<br>(0.68 to 0.73) | < 0.001 |

| | | | | | | |
|---|---|---|---|---|---|---|
| Adolescence | Prevalence | Female | Saint Vincent and the Grenadines | RA | 0.69<br>(0.64 to 0.73) | < 0.001 |
| Adolescence | Prevalence | Male | Saint Vincent and the Grenadines | RA | 0.78<br>(0.75 to 0.82) | < 0.001 |
| Adolescence | Prevalence | Both | Samoa | AA | 0.04<br>(0.03 to 0.04) | < 0.001 |
| Adolescence | Prevalence | Female | Samoa | AA | 0<br>(0 to 0) | 0.008 |
| Adolescence | Prevalence | Male | Samoa | AA | 0<br>(0 to 0) | < 0.001 |
| Adolescence | Prevalence | Both | Samoa | T1D | 0.64<br>(0.6 to 0.67) | < 0.001 |
| Adolescence | Prevalence | Female | Samoa | T1D | 0.61<br>(0.54 to 0.69) | < 0.001 |
| Adolescence | Prevalence | Male | Samoa | T1D | 0.65<br>(0.63 to 0.68) | < 0.001 |
| Adolescence | Prevalence | Both | Samoa | IBD | -0.1<br>(-0.15 to -0.05) | < 0.001 |
| Adolescence | Prevalence | Female | Samoa | IBD | -0.08<br>(-0.13 to -0.04) | < 0.001 |
| Adolescence | Prevalence | Male | Samoa | IBD | -0.12<br>(-0.2 to -0.04) | 0.005 |
| Adolescence | Prevalence | Both | Samoa | MS | 0.06<br>(-0.11 to 0.23) | 0.493 |
| Adolescence | Prevalence | Female | Samoa | MS | -0.04<br>(-0.25 to 0.16) | 0.676 |
| Adolescence | Prevalence | Male | Samoa | MS | -0.02<br>(-0.2 to 0.16) | 0.856 |
| Adolescence | Prevalence | Both | Samoa | Psoriasis | -0.12<br>(-0.15 to -0.1) | < 0.001 |
| Adolescence | Prevalence | Female | Samoa | Psoriasis | -0.13<br>(-0.15 to -0.12) | < 0.001 |
| Adolescence | Prevalence | Male | Samoa | Psoriasis | -0.16 | < 0.001 |

| | | | | | | |
|---|---|---|---|---|---|---|
| | | | | | (-0.2 to -0.11) | |
| Adolescence | Prevalence | Both | Samoa | RHD | 0.11<br>(0.1 to 0.12) | < 0.001 |
| Adolescence | Prevalence | Female | Samoa | RHD | 0.09<br>(0.06 to 0.13) | < 0.001 |
| Adolescence | Prevalence | Male | Samoa | RHD | 0.1<br>(0.08 to 0.12) | < 0.001 |
| Adolescence | Prevalence | Both | Samoa | RA | 0.23<br>(0.18 to 0.28) | < 0.001 |
| Adolescence | Prevalence | Female | Samoa | RA | 0.28<br>(0.22 to 0.34) | < 0.001 |
| Adolescence | Prevalence | Male | Samoa | RA | 0.1<br>(0.07 to 0.14) | < 0.001 |
| Adolescence | Prevalence | Both | Sao Tome and Principe | AA | 0.01<br>(0.01 to 0.02) | < 0.001 |
| Adolescence | Prevalence | Female | Sao Tome and Principe | AA | 0<br>(0 to 0) | < 0.001 |
| Adolescence | Prevalence | Male | Sao Tome and Principe | AA | 0<br>(0 to 0) | < 0.001 |
| Adolescence | Prevalence | Both | Sao Tome and Principe | T1D | 0.17<br>(0.13 to 0.22) | < 0.001 |
| Adolescence | Prevalence | Female | Sao Tome and Principe | T1D | 0.2<br>(0.16 to 0.23) | < 0.001 |
| Adolescence | Prevalence | Male | Sao Tome and Principe | T1D | 0.17<br>(0.1 to 0.24) | < 0.001 |
| Adolescence | Prevalence | Both | Sao Tome and Principe | IBD | 0.32<br>(0.23 to 0.41) | < 0.001 |
| Adolescence | Prevalence | Female | Sao Tome and Principe | IBD | 0.31<br>(0.21 to 0.4) | < 0.001 |
| Adolescence | Prevalence | Male | Sao Tome and Principe | IBD | 0.32<br>(0.24 to 0.4) | < 0.001 |
| Adolescence | Prevalence | Both | Sao Tome and Principe | MS | 0.12<br>(0.07 to 0.18) | < 0.001 |

| | | | | | | |
|---|---|---|---|---|---|---|
| Adolescence | Prevalence | Female | Sao Tome and Principe | MS | 0.1<br>(-0.1 to 0.3) | 0.338 |
| Adolescence | Prevalence | Male | Sao Tome and Principe | MS | 0.2<br>(0.09 to 0.3) | < 0.001 |
| Adolescence | Prevalence | Both | Sao Tome and Principe | Psoriasis | -0.78<br>(-0.96 to -0.6) | < 0.001 |
| Adolescence | Prevalence | Female | Sao Tome and Principe | Psoriasis | -0.86<br>(-1.03 to -0.68) | < 0.001 |
| Adolescence | Prevalence | Male | Sao Tome and Principe | Psoriasis | -0.68<br>(-0.82 to -0.54) | < 0.001 |
| Adolescence | Prevalence | Both | Sao Tome and Principe | RHD | 0.3<br>(0.29 to 0.32) | < 0.001 |
| Adolescence | Prevalence | Female | Sao Tome and Principe | RHD | 0.3<br>(0.26 to 0.34) | < 0.001 |
| Adolescence | Prevalence | Male | Sao Tome and Principe | RHD | 0.3<br>(0.27 to 0.33) | < 0.001 |
| Adolescence | Prevalence | Both | Sao Tome and Principe | RA | 0.48<br>(0.43 to 0.53) | < 0.001 |
| Adolescence | Prevalence | Female | Sao Tome and Principe | RA | 0.51<br>(0.46 to 0.55) | < 0.001 |
| Adolescence | Prevalence | Male | Sao Tome and Principe | RA | 0.42<br>(0.39 to 0.44) | < 0.001 |
| Adolescence | Prevalence | Both | Saudi Arabia | AA | -0.04<br>(-0.05 to -0.03) | < 0.001 |
| Adolescence | Prevalence | Female | Saudi Arabia | AA | 0<br>(0 to 0) | 0.001 |
| Adolescence | Prevalence | Male | Saudi Arabia | AA | 0<br>(0 to 0) | 0.011 |
| Adolescence | Prevalence | Both | Saudi Arabia | T1D | 2.18<br>(1.82 to 2.55) | < 0.001 |
| Adolescence | Prevalence | Female | Saudi Arabia | T1D | 2.04<br>(1.74 to 2.35) | < 0.001 |
| Adolescence | Prevalence | Male | Saudi Arabia | T1D | 2.26 | < 0.001 |

| | | | | | | |
|---|---|---|---|---|---|---|
| | | | | | (1.88 to 2.64) | |
| Adolescence | Prevalence | Both | Saudi Arabia | IBD | 0.38 (0.31 to 0.45) | < 0.001 |
| Adolescence | Prevalence | Female | Saudi Arabia | IBD | 0.49 (0.34 to 0.64) | < 0.001 |
| Adolescence | Prevalence | Male | Saudi Arabia | IBD | 0.32 (0.26 to 0.39) | < 0.001 |
| Adolescence | Prevalence | Both | Saudi Arabia | MS | 0.41 (0.37 to 0.46) | < 0.001 |
| Adolescence | Prevalence | Female | Saudi Arabia | MS | 0.44 (0.39 to 0.5) | < 0.001 |
| Adolescence | Prevalence | Male | Saudi Arabia | MS | 0.51 (0.4 to 0.62) | < 0.001 |
| Adolescence | Prevalence | Both | Saudi Arabia | Psoriasis | -1.96 (-1.99 to -1.93) | < 0.001 |
| Adolescence | Prevalence | Female | Saudi Arabia | Psoriasis | -1.89 (-1.92 to -1.86) | < 0.001 |
| Adolescence | Prevalence | Male | Saudi Arabia | Psoriasis | -2 (-2.02 to -1.97) | < 0.001 |
| Adolescence | Prevalence | Both | Saudi Arabia | RHD | -1.14 (-1.24 to -1.03) | < 0.001 |
| Adolescence | Prevalence | Female | Saudi Arabia | RHD | -1.36 (-1.62 to -1.09) | < 0.001 |
| Adolescence | Prevalence | Male | Saudi Arabia | RHD | -0.82 (-0.98 to -0.67) | < 0.001 |
| Adolescence | Prevalence | Both | Saudi Arabia | RA | 0.89 (0.85 to 0.93) | < 0.001 |
| Adolescence | Prevalence | Female | Saudi Arabia | RA | 0.92 (0.9 to 0.94) | < 0.001 |
| Adolescence | Prevalence | Male | Saudi Arabia | RA | 1.01 (0.94 to 1.09) | < 0.001 |
| Adolescence | Prevalence | Both | Senegal | AA | -0.08 (-0.08 to -0.07) | < 0.001 |

| | | | | | | |
|---|---|---|---|---|---|---|
| Adolescence | Prevalence | Female | Senegal | AA | 0<br>(0 to 0) | < 0.001 |
| Adolescence | Prevalence | Male | Senegal | AA | 0<br>(0 to 0) | < 0.001 |
| Adolescence | Prevalence | Both | Senegal | T1D | 0.03<br>(0.01 to 0.05) | 0.002 |
| Adolescence | Prevalence | Female | Senegal | T1D | 0.02<br>(-0.03 to 0.06) | 0.410 |
| Adolescence | Prevalence | Male | Senegal | T1D | 0.02<br>(0.01 to 0.03) | 0.003 |
| Adolescence | Prevalence | Both | Senegal | IBD | 0.15<br>(0.02 to 0.27) | 0.024 |
| Adolescence | Prevalence | Female | Senegal | IBD | 0.16<br>(0.02 to 0.3) | 0.024 |
| Adolescence | Prevalence | Male | Senegal | IBD | 0.19<br>(0.08 to 0.3) | 0.001 |
| Adolescence | Prevalence | Both | Senegal | MS | -0.01<br>(-0.01 to -0.01) | < 0.001 |
| Adolescence | Prevalence | Female | Senegal | MS | 0.08<br>(0.02 to 0.14) | 0.006 |
| Adolescence | Prevalence | Male | Senegal | MS | 0.02<br>(0 to 0.05) | 0.035 |
| Adolescence | Prevalence | Both | Senegal | Psoriasis | -0.59<br>(-0.75 to -0.44) | < 0.001 |
| Adolescence | Prevalence | Female | Senegal | Psoriasis | -0.65<br>(-0.76 to -0.54) | < 0.001 |
| Adolescence | Prevalence | Male | Senegal | Psoriasis | -0.5<br>(-0.65 to -0.35) | < 0.001 |
| Adolescence | Prevalence | Both | Senegal | RHD | 0.21<br>(0.01 to 0.4) | 0.037 |
| Adolescence | Prevalence | Female | Senegal | RHD | 0.26<br>(0.04 to 0.49) | 0.023 |
| Adolescence | Prevalence | Male | Senegal | RHD | 0.17 | 0.020 |

| | | | | | | |
|---|---|---|---|---|---|---|
| | | | | | (0.03 to 0.32) | |
| Adolescence | Prevalence | Both | Senegal | RA | 0.26<br>(0.24 to 0.29) | < 0.001 |
| Adolescence | Prevalence | Female | Senegal | RA | 0.38<br>(0.34 to 0.41) | < 0.001 |
| Adolescence | Prevalence | Male | Senegal | RA | 0.33<br>(0.29 to 0.37) | < 0.001 |
| Adolescence | Prevalence | Both | Serbia | AA | -0.03<br>(-0.05 to -0.02) | < 0.001 |
| Adolescence | Prevalence | Female | Serbia | AA | 0<br>(0 to 0) | < 0.001 |
| Adolescence | Prevalence | Male | Serbia | AA | 0<br>(0 to 0) | 1.000 |
| Adolescence | Prevalence | Both | Serbia | T1D | 1.42<br>(1.33 to 1.52) | < 0.001 |
| Adolescence | Prevalence | Female | Serbia | T1D | 1.36<br>(1.32 to 1.4) | < 0.001 |
| Adolescence | Prevalence | Male | Serbia | T1D | 1.49<br>(1.39 to 1.6) | < 0.001 |
| Adolescence | Prevalence | Both | Serbia | IBD | 0.88<br>(0.73 to 1.03) | < 0.001 |
| Adolescence | Prevalence | Female | Serbia | IBD | 0.66<br>(0.59 to 0.73) | < 0.001 |
| Adolescence | Prevalence | Male | Serbia | IBD | 1.01<br>(0.89 to 1.13) | < 0.001 |
| Adolescence | Prevalence | Both | Serbia | MS | 0.71<br>(0.66 to 0.76) | < 0.001 |
| Adolescence | Prevalence | Female | Serbia | MS | 0.75<br>(0.65 to 0.85) | < 0.001 |
| Adolescence | Prevalence | Male | Serbia | MS | 0.67<br>(0.64 to 0.7) | < 0.001 |
| Adolescence | Prevalence | Both | Serbia | Psoriasis | -0.89<br>(-0.92 to -0.86) | < 0.001 |

| | | | | | | |
|---|---|---|---|---|---|---|
| Adolescence | Prevalence | Female | Serbia | Psoriasis | -0.89 (-0.92 to -0.86) | < 0.001 |
| Adolescence | Prevalence | Male | Serbia | Psoriasis | -0.84 (-0.86 to -0.81) | < 0.001 |
| Adolescence | Prevalence | Both | Serbia | RHD | 0.05 (0.01 to 0.1) | 0.023 |
| Adolescence | Prevalence | Female | Serbia | RHD | 0.14 (-0.14 to 0.41) | 0.333 |
| Adolescence | Prevalence | Male | Serbia | RHD | -0.05 (-0.2 to 0.11) | 0.542 |
| Adolescence | Prevalence | Both | Serbia | RA | 1.25 (1.02 to 1.48) | < 0.001 |
| Adolescence | Prevalence | Female | Serbia | RA | 1.26 (1 to 1.53) | < 0.001 |
| Adolescence | Prevalence | Male | Serbia | RA | 1.37 (1.18 to 1.56) | < 0.001 |
| Adolescence | Prevalence | Both | Seychelles | AA | -0.01 (-0.01 to -0.01) | < 0.001 |
| Adolescence | Prevalence | Female | Seychelles | AA | 0 (0 to 0) | 0.008 |
| Adolescence | Prevalence | Male | Seychelles | AA | 0 (0 to 0) | < 0.001 |
| Adolescence | Prevalence | Both | Seychelles | T1D | 0.67 (0.62 to 0.72) | < 0.001 |
| Adolescence | Prevalence | Female | Seychelles | T1D | 0.63 (0.57 to 0.68) | < 0.001 |
| Adolescence | Prevalence | Male | Seychelles | T1D | 0.74 (0.65 to 0.82) | < 0.001 |
| Adolescence | Prevalence | Both | Seychelles | IBD | 0.5 (0.46 to 0.54) | < 0.001 |
| Adolescence | Prevalence | Female | Seychelles | IBD | 0.59 (0.52 to 0.65) | < 0.001 |
| Adolescence | Prevalence | Male | Seychelles | IBD | 0.39 | < 0.001 |

| | | | | | | |
|---|---|---|---|---|---|---|
| | | | | | (0.31 to 0.46) | |
| Adolescence | Prevalence | Both | Seychelles | MS | 0.02<br>(-0.04 to 0.08) | 0.471 |
| Adolescence | Prevalence | Female | Seychelles | MS | 0.08<br>(0.02 to 0.15) | 0.012 |
| Adolescence | Prevalence | Male | Seychelles | MS | -0.14<br>(-0.14 to -0.14) | < 0.001 |
| Adolescence | Prevalence | Both | Seychelles | Psoriasis | -0.42<br>(-0.43 to -0.41) | < 0.001 |
| Adolescence | Prevalence | Female | Seychelles | Psoriasis | -0.4<br>(-0.42 to -0.37) | < 0.001 |
| Adolescence | Prevalence | Male | Seychelles | Psoriasis | -0.44<br>(-0.48 to -0.4) | < 0.001 |
| Adolescence | Prevalence | Both | Seychelles | RHD | -0.11<br>(-0.12 to -0.09) | < 0.001 |
| Adolescence | Prevalence | Female | Seychelles | RHD | -0.1<br>(-0.15 to -0.05) | < 0.001 |
| Adolescence | Prevalence | Male | Seychelles | RHD | -0.09<br>(-0.1 to -0.07) | < 0.001 |
| Adolescence | Prevalence | Both | Seychelles | RA | 0.48<br>(0.45 to 0.5) | < 0.001 |
| Adolescence | Prevalence | Female | Seychelles | RA | 0.51<br>(0.48 to 0.55) | < 0.001 |
| Adolescence | Prevalence | Male | Seychelles | RA | 0.45<br>(0.41 to 0.48) | < 0.001 |
| Adolescence | Prevalence | Both | Sierra Leone | AA | -0.02<br>(-0.04 to -0.01) | < 0.001 |
| Adolescence | Prevalence | Female | Sierra Leone | AA | 0<br>(0 to 0) | < 0.001 |
| Adolescence | Prevalence | Male | Sierra Leone | AA | 0<br>(0 to 0) | < 0.001 |
| Adolescence | Prevalence | Both | Sierra Leone | T1D | 0.01<br>(0 to 0.03) | 0.105 |

| | | | | | | |
|---|---|---|---|---|---|---|
| Adolescence | Prevalence | Female | Sierra Leone | T1D | 0.02<br>(0 to 0.04) | 0.129 |
| Adolescence | Prevalence | Male | Sierra Leone | T1D | 0.03<br>(0 to 0.06) | 0.080 |
| Adolescence | Prevalence | Both | Sierra Leone | IBD | 0.24<br>(0.15 to 0.33) | < 0.001 |
| Adolescence | Prevalence | Female | Sierra Leone | IBD | 0.26<br>(0.2 to 0.31) | < 0.001 |
| Adolescence | Prevalence | Male | Sierra Leone | IBD | 0.29<br>(0.22 to 0.36) | < 0.001 |
| Adolescence | Prevalence | Both | Sierra Leone | MS | 0.09<br>(-0.05 to 0.24) | 0.218 |
| Adolescence | Prevalence | Female | Sierra Leone | MS | 0.12<br>(0.01 to 0.24) | 0.036 |
| Adolescence | Prevalence | Male | Sierra Leone | MS | 0.15<br>(0.02 to 0.28) | 0.020 |
| Adolescence | Prevalence | Both | Sierra Leone | Psoriasis | -0.56<br>(-0.7 to -0.42) | < 0.001 |
| Adolescence | Prevalence | Female | Sierra Leone | Psoriasis | -0.61<br>(-0.72 to -0.51) | < 0.001 |
| Adolescence | Prevalence | Male | Sierra Leone | Psoriasis | -0.47<br>(-0.65 to -0.28) | < 0.001 |
| Adolescence | Prevalence | Both | Sierra Leone | RHD | 0.21<br>(0.19 to 0.24) | < 0.001 |
| Adolescence | Prevalence | Female | Sierra Leone | RHD | 0.22<br>(0.14 to 0.29) | < 0.001 |
| Adolescence | Prevalence | Male | Sierra Leone | RHD | 0.23<br>(0.19 to 0.26) | < 0.001 |
| Adolescence | Prevalence | Both | Sierra Leone | RA | 0.32<br>(0.28 to 0.36) | < 0.001 |
| Adolescence | Prevalence | Female | Sierra Leone | RA | 0.37<br>(0.32 to 0.42) | < 0.001 |
| Adolescence | Prevalence | Male | Sierra Leone | RA | 0.27 | < 0.001 |

| | | | | | | |
|---|---|---|---|---|---|---|
| | | | | | (0.18 to 0.36) | |
| Adolescence | Prevalence | Both | Singapore | AA | 0.01<br>(-0.03 to 0.05) | 0.578 |
| Adolescence | Prevalence | Female | Singapore | AA | 0<br>(0 to 0) | < 0.001 |
| Adolescence | Prevalence | Male | Singapore | AA | 0<br>(0 to 0) | < 0.001 |
| Adolescence | Prevalence | Both | Singapore | T1D | 0.04<br>(0.02 to 0.06) | < 0.001 |
| Adolescence | Prevalence | Female | Singapore | T1D | 0.09<br>(0.07 to 0.11) | < 0.001 |
| Adolescence | Prevalence | Male | Singapore | T1D | 0<br>(-0.01 to 0.01) | 0.450 |
| Adolescence | Prevalence | Both | Singapore | IBD | -0.09<br>(-0.15 to -0.02) | 0.010 |
| Adolescence | Prevalence | Female | Singapore | IBD | 0<br>(-0.06 to 0.05) | 0.900 |
| Adolescence | Prevalence | Male | Singapore | IBD | -0.15<br>(-0.26 to -0.04) | 0.007 |
| Adolescence | Prevalence | Both | Singapore | MS | -0.05<br>(-0.26 to 0.16) | 0.649 |
| Adolescence | Prevalence | Female | Singapore | MS | 0.07<br>(0 to 0.14) | 0.061 |
| Adolescence | Prevalence | Male | Singapore | MS | 0.08<br>(-0.18 to 0.35) | 0.552 |
| Adolescence | Prevalence | Both | Singapore | Psoriasis | -0.31<br>(-0.36 to -0.27) | < 0.001 |
| Adolescence | Prevalence | Female | Singapore | Psoriasis | -0.4<br>(-0.44 to -0.35) | < 0.001 |
| Adolescence | Prevalence | Male | Singapore | Psoriasis | -0.27<br>(-0.28 to -0.26) | < 0.001 |
| Adolescence | Prevalence | Both | Singapore | RHD | -1.39<br>(-1.48 to -1.3) | < 0.001 |

| | | | | | | |
|---|---|---|---|---|---|---|
| Adolescence | Prevalence | Female | Singapore | RHD | -1.56 (-1.69 to -1.43) | < 0.001 |
| Adolescence | Prevalence | Male | Singapore | RHD | -1.23 (-1.31 to -1.15) | < 0.001 |
| Adolescence | Prevalence | Both | Singapore | RA | 0.49 (0.36 to 0.62) | < 0.001 |
| Adolescence | Prevalence | Female | Singapore | RA | 0.46 (0.44 to 0.48) | < 0.001 |
| Adolescence | Prevalence | Male | Singapore | RA | 0.48 (0.47 to 0.49) | < 0.001 |
| Adolescence | Prevalence | Both | Slovakia | AA | -0.01 (-0.01 to -0.01) | < 0.001 |
| Adolescence | Prevalence | Female | Slovakia | AA | 0 (0 to 0) | < 0.001 |
| Adolescence | Prevalence | Male | Slovakia | AA | 0 (0 to 0) | 1.000 |
| Adolescence | Prevalence | Both | Slovakia | T1D | 1.81 (1.79 to 1.84) | < 0.001 |
| Adolescence | Prevalence | Female | Slovakia | T1D | 1.71 (1.66 to 1.76) | < 0.001 |
| Adolescence | Prevalence | Male | Slovakia | T1D | 1.9 (1.85 to 1.94) | < 0.001 |
| Adolescence | Prevalence | Both | Slovakia | IBD | 0.12 (0.05 to 0.18) | 0.001 |
| Adolescence | Prevalence | Female | Slovakia | IBD | 0.13 (0.04 to 0.22) | 0.003 |
| Adolescence | Prevalence | Male | Slovakia | IBD | 0.1 (0.03 to 0.17) | 0.007 |
| Adolescence | Prevalence | Both | Slovakia | MS | 0.52 (0.44 to 0.59) | < 0.001 |
| Adolescence | Prevalence | Female | Slovakia | MS | 0.63 (0.59 to 0.67) | < 0.001 |
| Adolescence | Prevalence | Male | Slovakia | MS | 0.37 | < 0.001 |

| | | | | | | |
|---|---|---|---|---|---|---|
| | | | | | (0.29 to 0.45) | |
| Adolescence | Prevalence | Both | Slovakia | Psoriasis | -0.95 (-1 to -0.91) | < 0.001 |
| Adolescence | Prevalence | Female | Slovakia | Psoriasis | -0.97 (-1.03 to -0.92) | < 0.001 |
| Adolescence | Prevalence | Male | Slovakia | Psoriasis | -0.92 (-0.94 to -0.9) | < 0.001 |
| Adolescence | Prevalence | Both | Slovakia | RHD | -0.22 (-0.64 to 0.21) | 0.318 |
| Adolescence | Prevalence | Female | Slovakia | RHD | -0.24 (-0.79 to 0.3) | 0.381 |
| Adolescence | Prevalence | Male | Slovakia | RHD | -0.2 (-0.76 to 0.35) | 0.468 |
| Adolescence | Prevalence | Both | Slovakia | RA | 0.53 (0.48 to 0.57) | < 0.001 |
| Adolescence | Prevalence | Female | Slovakia | RA | 0.55 (0.49 to 0.61) | < 0.001 |
| Adolescence | Prevalence | Male | Slovakia | RA | 0.52 (0.48 to 0.55) | < 0.001 |
| Adolescence | Prevalence | Both | Slovenia | AA | -0.01 (-0.01 to 0) | 0.013 |
| Adolescence | Prevalence | Female | Slovenia | AA | 0 (0 to 0) | < 0.001 |
| Adolescence | Prevalence | Male | Slovenia | AA | 0 (0 to 0) | 1.000 |
| Adolescence | Prevalence | Both | Slovenia | T1D | 1.62 (1.57 to 1.67) | < 0.001 |
| Adolescence | Prevalence | Female | Slovenia | T1D | 1.53 (1.51 to 1.56) | < 0.001 |
| Adolescence | Prevalence | Male | Slovenia | T1D | 1.71 (1.68 to 1.75) | < 0.001 |
| Adolescence | Prevalence | Both | Slovenia | IBD | 0.39 (0.24 to 0.54) | < 0.001 |

| | | | | | | |
|---|---|---|---|---|---|---|
| Adolescence | Prevalence | Female | Slovenia | IBD | 0.1 (-0.07 to 0.27) | 0.239 |
| Adolescence | Prevalence | Male | Slovenia | IBD | 0.7 (0.56 to 0.85) | < 0.001 |
| Adolescence | Prevalence | Both | Slovenia | MS | 0.48 (0.44 to 0.52) | < 0.001 |
| Adolescence | Prevalence | Female | Slovenia | MS | 0.49 (0.41 to 0.58) | < 0.001 |
| Adolescence | Prevalence | Male | Slovenia | MS | 0.48 (0.38 to 0.59) | < 0.001 |
| Adolescence | Prevalence | Both | Slovenia | Psoriasis | -0.79 (-0.83 to -0.75) | < 0.001 |
| Adolescence | Prevalence | Female | Slovenia | Psoriasis | -0.81 (-0.85 to -0.78) | < 0.001 |
| Adolescence | Prevalence | Male | Slovenia | Psoriasis | -0.76 (-0.77 to -0.74) | < 0.001 |
| Adolescence | Prevalence | Both | Slovenia | RHD | -0.12 (-0.35 to 0.11) | 0.318 |
| Adolescence | Prevalence | Female | Slovenia | RHD | -0.12 (-0.29 to 0.05) | 0.182 |
| Adolescence | Prevalence | Male | Slovenia | RHD | 0 (-0.29 to 0.29) | 0.998 |
| Adolescence | Prevalence | Both | Slovenia | RA | 0.6 (0.55 to 0.65) | < 0.001 |
| Adolescence | Prevalence | Female | Slovenia | RA | 0.62 (0.57 to 0.67) | < 0.001 |
| Adolescence | Prevalence | Male | Slovenia | RA | 0.56 (0.51 to 0.61) | < 0.001 |
| Adolescence | Prevalence | Both | Solomon Islands | AA | -0.01 (-0.01 to -0.01) | < 0.001 |
| Adolescence | Prevalence | Female | Solomon Islands | AA | 0 (0 to 0) | 0.008 |
| Adolescence | Prevalence | Male | Solomon Islands | AA | 0 | < 0.001 |

| | | | | | | |
|---|---|---|---|---|---|---|
| | | | | | (0 to 0) | |
| Adolescence | Prevalence | Both | Solomon Islands | T1D | 0.59<br>(0.57 to 0.61) | < 0.001 |
| Adolescence | Prevalence | Female | Solomon Islands | T1D | 0.57<br>(0.52 to 0.62) | < 0.001 |
| Adolescence | Prevalence | Male | Solomon Islands | T1D | 0.61<br>(0.6 to 0.63) | < 0.001 |
| Adolescence | Prevalence | Both | Solomon Islands | IBD | 0.07<br>(0.02 to 0.12) | 0.007 |
| Adolescence | Prevalence | Female | Solomon Islands | IBD | 0.06<br>(-0.02 to 0.14) | 0.165 |
| Adolescence | Prevalence | Male | Solomon Islands | IBD | 0.07<br>(0.02 to 0.13) | 0.007 |
| Adolescence | Prevalence | Both | Solomon Islands | MS | 0.07<br>(-0.07 to 0.21) | 0.334 |
| Adolescence | Prevalence | Female | Solomon Islands | MS | 0.06<br>(-0.1 to 0.22) | 0.452 |
| Adolescence | Prevalence | Male | Solomon Islands | MS | 0.04<br>(-0.05 to 0.13) | 0.362 |
| Adolescence | Prevalence | Both | Solomon Islands | Psoriasis | -0.46<br>(-0.47 to -0.45) | < 0.001 |
| Adolescence | Prevalence | Female | Solomon Islands | Psoriasis | -0.44<br>(-0.46 to -0.42) | < 0.001 |
| Adolescence | Prevalence | Male | Solomon Islands | Psoriasis | -0.47<br>(-0.51 to -0.43) | < 0.001 |
| Adolescence | Prevalence | Both | Solomon Islands | RHD | 0.07<br>(0.05 to 0.09) | < 0.001 |
| Adolescence | Prevalence | Female | Solomon Islands | RHD | 0.05<br>(0.02 to 0.08) | 0.003 |
| Adolescence | Prevalence | Male | Solomon Islands | RHD | 0.1<br>(0.07 to 0.13) | < 0.001 |
| Adolescence | Prevalence | Both | Solomon Islands | RA | 0.37<br>(0.3 to 0.45) | < 0.001 |

| | | | | | | |
|---|---|---|---|---|---|---|
| Adolescence | Prevalence | Female | Solomon Islands | RA | 0.46 (0.35 to 0.56) | < 0.001 |
| Adolescence | Prevalence | Male | Solomon Islands | RA | 0.26 (0.24 to 0.28) | < 0.001 |
| Adolescence | Prevalence | Both | Somalia | AA | 0.03 (0.03 to 0.04) | < 0.001 |
| Adolescence | Prevalence | Female | Somalia | AA | 0 (0 to 0) | < 0.001 |
| Adolescence | Prevalence | Male | Somalia | AA | 0 (0 to 0) | < 0.001 |
| Adolescence | Prevalence | Both | Somalia | T1D | -0.03 (-0.06 to -0.01) | 0.007 |
| Adolescence | Prevalence | Female | Somalia | T1D | -0.03 (-0.04 to -0.02) | < 0.001 |
| Adolescence | Prevalence | Male | Somalia | T1D | -0.01 (-0.05 to 0.03) | 0.668 |
| Adolescence | Prevalence | Both | Somalia | IBD | -0.14 (-0.25 to -0.02) | 0.017 |
| Adolescence | Prevalence | Female | Somalia | IBD | -0.13 (-0.34 to 0.07) | 0.196 |
| Adolescence | Prevalence | Male | Somalia | IBD | -0.13 (-0.25 to -0.02) | 0.024 |
| Adolescence | Prevalence | Both | Somalia | MS | 0.1 (0.1 to 0.1) | < 0.001 |
| Adolescence | Prevalence | Female | Somalia | MS | 0.09 (-0.01 to 0.18) | 0.072 |
| Adolescence | Prevalence | Male | Somalia | MS | 0.07 (-0.14 to 0.27) | 0.535 |
| Adolescence | Prevalence | Both | Somalia | Psoriasis | 0.15 (0.11 to 0.18) | < 0.001 |
| Adolescence | Prevalence | Female | Somalia | Psoriasis | 0.12 (0.04 to 0.2) | 0.003 |
| Adolescence | Prevalence | Male | Somalia | Psoriasis | 0.17 | < 0.001 |

| | | | | | | |
|---|---|---|---|---|---|---|
| | | | | | (0.11 to 0.23) | |
| Adolescence | Prevalence | Both | Somalia | RHD | 0.2<br>(0.19 to 0.2) | < 0.001 |
| Adolescence | Prevalence | Female | Somalia | RHD | 0.19<br>(0.17 to 0.2) | < 0.001 |
| Adolescence | Prevalence | Male | Somalia | RHD | 0.21<br>(0.18 to 0.23) | < 0.001 |
| Adolescence | Prevalence | Both | Somalia | RA | 0.1<br>(0.02 to 0.17) | 0.011 |
| Adolescence | Prevalence | Female | Somalia | RA | 0<br>(-0.06 to 0.05) | 0.943 |
| Adolescence | Prevalence | Male | Somalia | RA | 0.12<br>(0.06 to 0.18) | < 0.001 |
| Adolescence | Prevalence | Both | South Africa | AA | -0.04<br>(-0.04 to -0.04) | < 0.001 |
| Adolescence | Prevalence | Female | South Africa | AA | 0<br>(0 to 0) | < 0.001 |
| Adolescence | Prevalence | Male | South Africa | AA | 0<br>(0 to 0) | < 0.001 |
| Adolescence | Prevalence | Both | South Africa | T1D | 0.22<br>(0.18 to 0.25) | < 0.001 |
| Adolescence | Prevalence | Female | South Africa | T1D | 0.24<br>(0.21 to 0.27) | < 0.001 |
| Adolescence | Prevalence | Male | South Africa | T1D | 0.18<br>(0.14 to 0.22) | < 0.001 |
| Adolescence | Prevalence | Both | South Africa | IBD | 0.23<br>(0.18 to 0.29) | < 0.001 |
| Adolescence | Prevalence | Female | South Africa | IBD | 0.22<br>(0.17 to 0.27) | < 0.001 |
| Adolescence | Prevalence | Male | South Africa | IBD | 0.28<br>(0.22 to 0.34) | < 0.001 |
| Adolescence | Prevalence | Both | South Africa | MS | 0.01<br>(-0.09 to 0.11) | 0.871 |

| | | | | | | |
|---|---|---|---|---|---|---|
| Adolescence | Prevalence | Female | South Africa | MS | -0.01 (-0.13 to 0.11) | 0.843 |
| Adolescence | Prevalence | Male | South Africa | MS | 0.06 (-0.02 to 0.15) | 0.125 |
| Adolescence | Prevalence | Both | South Africa | Psoriasis | -0.46 (-0.58 to -0.34) | < 0.001 |
| Adolescence | Prevalence | Female | South Africa | Psoriasis | -0.49 (-0.57 to -0.4) | < 0.001 |
| Adolescence | Prevalence | Male | South Africa | Psoriasis | -0.39 (-0.5 to -0.29) | < 0.001 |
| Adolescence | Prevalence | Both | South Africa | RHD | 0.08 (0.04 to 0.12) | < 0.001 |
| Adolescence | Prevalence | Female | South Africa | RHD | 0.08 (0.04 to 0.11) | < 0.001 |
| Adolescence | Prevalence | Male | South Africa | RHD | 0.09 (0.03 to 0.16) | 0.002 |
| Adolescence | Prevalence | Both | South Africa | RA | -0.03 (-0.1 to 0.05) | 0.486 |
| Adolescence | Prevalence | Female | South Africa | RA | -0.16 (-0.27 to -0.05) | 0.004 |
| Adolescence | Prevalence | Male | South Africa | RA | 0.44 (0.37 to 0.52) | < 0.001 |
| Adolescence | Prevalence | Both | South Sudan | AA | 0.01 (0 to 0.02) | 0.066 |
| Adolescence | Prevalence | Female | South Sudan | AA | 0 (0 to 0) | < 0.001 |
| Adolescence | Prevalence | Male | South Sudan | AA | 0 (0 to 0) | < 0.001 |
| Adolescence | Prevalence | Both | South Sudan | T1D | 0.07 (0.04 to 0.1) | < 0.001 |
| Adolescence | Prevalence | Female | South Sudan | T1D | 0.07 (0.05 to 0.1) | < 0.001 |
| Adolescence | Prevalence | Male | South Sudan | T1D | 0.09 | < 0.001 |

| | | | | | | |
|---|---|---|---|---|---|---|
| | | | | | (0.06 to 0.13) | |
| Adolescence | Prevalence | Both | South Sudan | IBD | 0.15<br>(0.09 to 0.22) | < 0.001 |
| Adolescence | Prevalence | Female | South Sudan | IBD | 0.14<br>(0.05 to 0.22) | 0.003 |
| Adolescence | Prevalence | Male | South Sudan | IBD | 0.15<br>(0.12 to 0.19) | < 0.001 |
| Adolescence | Prevalence | Both | South Sudan | MS | 0.05<br>(-0.06 to 0.15) | 0.413 |
| Adolescence | Prevalence | Female | South Sudan | MS | 0.03<br>(-0.06 to 0.12) | 0.514 |
| Adolescence | Prevalence | Male | South Sudan | MS | -0.01<br>(-0.15 to 0.14) | 0.927 |
| Adolescence | Prevalence | Both | South Sudan | Psoriasis | -0.17<br>(-0.2 to -0.15) | < 0.001 |
| Adolescence | Prevalence | Female | South Sudan | Psoriasis | -0.2<br>(-0.24 to -0.16) | < 0.001 |
| Adolescence | Prevalence | Male | South Sudan | Psoriasis | -0.17<br>(-0.24 to -0.09) | < 0.001 |
| Adolescence | Prevalence | Both | South Sudan | RHD | 0.17<br>(0.16 to 0.18) | < 0.001 |
| Adolescence | Prevalence | Female | South Sudan | RHD | 0.18<br>(0.16 to 0.19) | < 0.001 |
| Adolescence | Prevalence | Male | South Sudan | RHD | 0.16<br>(0.12 to 0.19) | < 0.001 |
| Adolescence | Prevalence | Both | South Sudan | RA | 0.25<br>(0.16 to 0.34) | < 0.001 |
| Adolescence | Prevalence | Female | South Sudan | RA | 0.23<br>(0.18 to 0.29) | < 0.001 |
| Adolescence | Prevalence | Male | South Sudan | RA | 0.27<br>(0.24 to 0.3) | < 0.001 |
| Adolescence | Prevalence | Both | Spain | AA | 0<br>(0 to 0) | 0.470 |

| | | | | | | |
|---|---|---|---|---|---|---|
| Adolescence | Prevalence | Female | Spain | AA | 0<br>(0 to 0) | < 0.001 |
| Adolescence | Prevalence | Male | Spain | AA | 0<br>(0 to 0) | 0.005 |
| Adolescence | Prevalence | Both | Spain | T1D | 1.86<br>(1.84 to 1.89) | < 0.001 |
| Adolescence | Prevalence | Female | Spain | T1D | 1.75<br>(1.72 to 1.77) | < 0.001 |
| Adolescence | Prevalence | Male | Spain | T1D | 1.97<br>(1.92 to 2.02) | < 0.001 |
| Adolescence | Prevalence | Both | Spain | IBD | 1<br>(0.89 to 1.11) | < 0.001 |
| Adolescence | Prevalence | Female | Spain | IBD | 0.91<br>(0.75 to 1.07) | < 0.001 |
| Adolescence | Prevalence | Male | Spain | IBD | 1.09<br>(0.97 to 1.21) | < 0.001 |
| Adolescence | Prevalence | Both | Spain | MS | 0.59<br>(0.52 to 0.66) | < 0.001 |
| Adolescence | Prevalence | Female | Spain | MS | 0.6<br>(0.56 to 0.64) | < 0.001 |
| Adolescence | Prevalence | Male | Spain | MS | 0.49<br>(0.37 to 0.62) | < 0.001 |
| Adolescence | Prevalence | Both | Spain | Psoriasis | -0.41<br>(-0.43 to -0.39) | < 0.001 |
| Adolescence | Prevalence | Female | Spain | Psoriasis | -0.42<br>(-0.44 to -0.4) | < 0.001 |
| Adolescence | Prevalence | Male | Spain | Psoriasis | -0.42<br>(-0.45 to -0.4) | < 0.001 |
| Adolescence | Prevalence | Both | Spain | RHD | -0.38<br>(-0.42 to -0.34) | < 0.001 |
| Adolescence | Prevalence | Female | Spain | RHD | -0.33<br>(-0.38 to -0.28) | < 0.001 |
| Adolescence | Prevalence | Male | Spain | RHD | -0.43 | < 0.001 |

| | | | | | | |
|---|---|---|---|---|---|---|
| | | | | | (-0.49 to -0.37) | |
| Adolescence | Prevalence | Both | Spain | RA | 0.39<br>(0.36 to 0.43) | < 0.001 |
| Adolescence | Prevalence | Female | Spain | RA | 0.33<br>(0.3 to 0.36) | < 0.001 |
| Adolescence | Prevalence | Male | Spain | RA | 0.58<br>(0.54 to 0.61) | < 0.001 |
| Adolescence | Prevalence | Both | Sri Lanka | AA | -0.04<br>(-0.04 to -0.04) | < 0.001 |
| Adolescence | Prevalence | Female | Sri Lanka | AA | 0<br>(0 to 0) | 0.008 |
| Adolescence | Prevalence | Male | Sri Lanka | AA | -0.1<br>(-0.1 to -0.1) | < 0.001 |
| Adolescence | Prevalence | Both | Sri Lanka | T1D | 0.64<br>(0.61 to 0.67) | < 0.001 |
| Adolescence | Prevalence | Female | Sri Lanka | T1D | 0.6<br>(0.58 to 0.62) | < 0.001 |
| Adolescence | Prevalence | Male | Sri Lanka | T1D | 0.67<br>(0.65 to 0.69) | < 0.001 |
| Adolescence | Prevalence | Both | Sri Lanka | IBD | 2.03<br>(1.84 to 2.21) | < 0.001 |
| Adolescence | Prevalence | Female | Sri Lanka | IBD | 2.16<br>(2.02 to 2.3) | < 0.001 |
| Adolescence | Prevalence | Male | Sri Lanka | IBD | 1.94<br>(1.84 to 2.05) | < 0.001 |
| Adolescence | Prevalence | Both | Sri Lanka | MS | -0.1<br>(-0.19 to 0) | 0.039 |
| Adolescence | Prevalence | Female | Sri Lanka | MS | -0.08<br>(-0.2 to 0.05) | 0.231 |
| Adolescence | Prevalence | Male | Sri Lanka | MS | -0.08<br>(-0.16 to -0.01) | 0.026 |
| Adolescence | Prevalence | Both | Sri Lanka | Psoriasis | -0.87<br>(-0.9 to -0.85) | < 0.001 |

| | | | | | | |
|---|---|---|---|---|---|---|
| Adolescence | Prevalence | Female | Sri Lanka | Psoriasis | -0.84 (-0.88 to -0.81) | < 0.001 |
| Adolescence | Prevalence | Male | Sri Lanka | Psoriasis | -0.95 (-0.98 to -0.91) | < 0.001 |
| Adolescence | Prevalence | Both | Sri Lanka | RHD | -0.34 (-0.43 to -0.26) | < 0.001 |
| Adolescence | Prevalence | Female | Sri Lanka | RHD | -0.35 (-0.48 to -0.22) | < 0.001 |
| Adolescence | Prevalence | Male | Sri Lanka | RHD | -0.33 (-0.42 to -0.23) | < 0.001 |
| Adolescence | Prevalence | Both | Sri Lanka | RA | 0.33 (0.3 to 0.35) | < 0.001 |
| Adolescence | Prevalence | Female | Sri Lanka | RA | 0.34 (0.3 to 0.38) | < 0.001 |
| Adolescence | Prevalence | Male | Sri Lanka | RA | 0.31 (0.29 to 0.33) | < 0.001 |
| Adolescence | Prevalence | Both | Sudan | AA | -0.02 (-0.03 to -0.01) | < 0.001 |
| Adolescence | Prevalence | Female | Sudan | AA | 0 (0 to 0) | 0.001 |
| Adolescence | Prevalence | Male | Sudan | AA | 0 (0 to 0) | 0.011 |
| Adolescence | Prevalence | Both | Sudan | T1D | 2.14 (2.1 to 2.19) | < 0.001 |
| Adolescence | Prevalence | Female | Sudan | T1D | 2.13 (2.07 to 2.18) | < 0.001 |
| Adolescence | Prevalence | Male | Sudan | T1D | 2.17 (2.11 to 2.22) | < 0.001 |
| Adolescence | Prevalence | Both | Sudan | IBD | 0.58 (0.55 to 0.62) | < 0.001 |
| Adolescence | Prevalence | Female | Sudan | IBD | 0.56 (0.54 to 0.59) | < 0.001 |
| Adolescence | Prevalence | Male | Sudan | IBD | 0.62 | < 0.001 |

| | | | | | | |
|---|---|---|---|---|---|---|
| | | | | | (0.58 to 0.66) | |
| Adolescence | Prevalence | Both | Sudan | MS | 0.4<br>(0.35 to 0.45) | < 0.001 |
| Adolescence | Prevalence | Female | Sudan | MS | 0.43<br>(0.37 to 0.5) | < 0.001 |
| Adolescence | Prevalence | Male | Sudan | MS | 0.45<br>(0.38 to 0.51) | < 0.001 |
| Adolescence | Prevalence | Both | Sudan | Psoriasis | -1.36<br>(-1.37 to -1.36) | < 0.001 |
| Adolescence | Prevalence | Female | Sudan | Psoriasis | -1.32<br>(-1.33 to -1.31) | < 0.001 |
| Adolescence | Prevalence | Male | Sudan | Psoriasis | -1.4<br>(-1.4 to -1.39) | < 0.001 |
| Adolescence | Prevalence | Both | Sudan | RHD | 0.21<br>(0.19 to 0.23) | < 0.001 |
| Adolescence | Prevalence | Female | Sudan | RHD | 0.21<br>(0.19 to 0.22) | < 0.001 |
| Adolescence | Prevalence | Male | Sudan | RHD | 0.21<br>(0.12 to 0.3) | < 0.001 |
| Adolescence | Prevalence | Both | Sudan | RA | 0.81<br>(0.72 to 0.89) | < 0.001 |
| Adolescence | Prevalence | Female | Sudan | RA | 0.83<br>(0.78 to 0.88) | < 0.001 |
| Adolescence | Prevalence | Male | Sudan | RA | 0.85<br>(0.8 to 0.9) | < 0.001 |
| Adolescence | Prevalence | Both | Suriname | AA | -0.01<br>(-0.01 to 0) | 0.001 |
| Adolescence | Prevalence | Female | Suriname | AA | 0<br>(0 to 0) | < 0.001 |
| Adolescence | Prevalence | Male | Suriname | AA | 0<br>(0 to 0) | 1.000 |
| Adolescence | Prevalence | Both | Suriname | T1D | 1.08<br>(1.06 to 1.09) | < 0.001 |

| | | | | | | |
|---|---|---|---|---|---|---|
| Adolescence | Prevalence | Female | Suriname | T1D | 1.01<br>(0.98 to 1.03) | < 0.001 |
| Adolescence | Prevalence | Male | Suriname | T1D | 1.17<br>(1.16 to 1.18) | < 0.001 |
| Adolescence | Prevalence | Both | Suriname | IBD | 0.02<br>(-0.06 to 0.1) | 0.548 |
| Adolescence | Prevalence | Female | Suriname | IBD | 0.02<br>(-0.11 to 0.16) | 0.726 |
| Adolescence | Prevalence | Male | Suriname | IBD | 0.04<br>(-0.01 to 0.08) | 0.105 |
| Adolescence | Prevalence | Both | Suriname | MS | 0.25<br>(0.2 to 0.3) | < 0.001 |
| Adolescence | Prevalence | Female | Suriname | MS | 0.22<br>(0.13 to 0.31) | < 0.001 |
| Adolescence | Prevalence | Male | Suriname | MS | 0.25<br>(0.16 to 0.34) | < 0.001 |
| Adolescence | Prevalence | Both | Suriname | Psoriasis | -0.4<br>(-0.44 to -0.36) | < 0.001 |
| Adolescence | Prevalence | Female | Suriname | Psoriasis | -0.4<br>(-0.44 to -0.36) | < 0.001 |
| Adolescence | Prevalence | Male | Suriname | Psoriasis | -0.39<br>(-0.43 to -0.35) | < 0.001 |
| Adolescence | Prevalence | Both | Suriname | RHD | 0.01<br>(-0.02 to 0.04) | 0.403 |
| Adolescence | Prevalence | Female | Suriname | RHD | 0.04<br>(0.02 to 0.07) | 0.001 |
| Adolescence | Prevalence | Male | Suriname | RHD | 0<br>(-0.02 to 0.03) | 0.790 |
| Adolescence | Prevalence | Both | Suriname | RA | 0.68<br>(0.6 to 0.75) | < 0.001 |
| Adolescence | Prevalence | Female | Suriname | RA | 0.65<br>(0.6 to 0.7) | < 0.001 |
| Adolescence | Prevalence | Male | Suriname | RA | 0.74 | < 0.001 |

| | | | | | | |
|---|---|---|---|---|---|---|
| | | | | | (0.71 to 0.77) | |
| Adolescence | Prevalence | Both | Sweden | AA | 0 (0 to 0) | < 0.001 |
| Adolescence | Prevalence | Female | Sweden | AA | 0 (0 to 0) | < 0.001 |
| Adolescence | Prevalence | Male | Sweden | AA | 0 (0 to 0) | < 0.001 |
| Adolescence | Prevalence | Both | Sweden | T1D | 0.13 (0 to 0.27) | 0.052 |
| Adolescence | Prevalence | Female | Sweden | T1D | 0.17 (0.02 to 0.32) | 0.030 |
| Adolescence | Prevalence | Male | Sweden | T1D | 0.1 (-0.02 to 0.21) | 0.105 |
| Adolescence | Prevalence | Both | Sweden | IBD | -0.35 (-0.51 to -0.18) | < 0.001 |
| Adolescence | Prevalence | Female | Sweden | IBD | -0.74 (-0.88 to -0.61) | < 0.001 |
| Adolescence | Prevalence | Male | Sweden | IBD | -0.03 (-0.25 to 0.18) | 0.751 |
| Adolescence | Prevalence | Both | Sweden | MS | -0.21 (-0.22 to -0.2) | < 0.001 |
| Adolescence | Prevalence | Female | Sweden | MS | -0.26 (-0.3 to -0.23) | < 0.001 |
| Adolescence | Prevalence | Male | Sweden | MS | -0.03 (-0.08 to 0.01) | 0.151 |
| Adolescence | Prevalence | Both | Sweden | Psoriasis | -0.14 (-0.17 to -0.12) | < 0.001 |
| Adolescence | Prevalence | Female | Sweden | Psoriasis | -0.11 (-0.13 to -0.1) | < 0.001 |
| Adolescence | Prevalence | Male | Sweden | Psoriasis | -0.14 (-0.16 to -0.12) | < 0.001 |
| Adolescence | Prevalence | Both | Sweden | RHD | -0.32 (-0.54 to -0.1) | 0.005 |

| | | | | | | |
|---|---|---|---|---|---|---|
| Adolescence | Prevalence | Female | Sweden | RHD | -0.29<br>(-0.51 to -0.06) | 0.012 |
| Adolescence | Prevalence | Male | Sweden | RHD | -0.35<br>(-0.58 to -0.12) | 0.003 |
| Adolescence | Prevalence | Both | Sweden | RA | 0.2<br>(0.19 to 0.22) | < 0.001 |
| Adolescence | Prevalence | Female | Sweden | RA | 0.24<br>(0.23 to 0.26) | < 0.001 |
| Adolescence | Prevalence | Male | Sweden | RA | 0.16<br>(0.13 to 0.18) | < 0.001 |
| Adolescence | Prevalence | Both | Switzerland | AA | 0<br>(0 to 0) | 0.037 |
| Adolescence | Prevalence | Female | Switzerland | AA | 0<br>(0 to 0) | < 0.001 |
| Adolescence | Prevalence | Male | Switzerland | AA | 0<br>(0 to 0) | 0.005 |
| Adolescence | Prevalence | Both | Switzerland | T1D | 1.64<br>(1.59 to 1.68) | < 0.001 |
| Adolescence | Prevalence | Female | Switzerland | T1D | 1.63<br>(1.58 to 1.67) | < 0.001 |
| Adolescence | Prevalence | Male | Switzerland | T1D | 1.64<br>(1.56 to 1.72) | < 0.001 |
| Adolescence | Prevalence | Both | Switzerland | IBD | 0.42<br>(0.34 to 0.5) | < 0.001 |
| Adolescence | Prevalence | Female | Switzerland | IBD | 0.06<br>(-0.02 to 0.14) | 0.118 |
| Adolescence | Prevalence | Male | Switzerland | IBD | 0.84<br>(0.71 to 0.96) | < 0.001 |
| Adolescence | Prevalence | Both | Switzerland | MS | 0.11<br>(0.08 to 0.14) | < 0.001 |
| Adolescence | Prevalence | Female | Switzerland | MS | 0.14<br>(0.09 to 0.18) | < 0.001 |
| Adolescence | Prevalence | Male | Switzerland | MS | 0.09 | < 0.001 |

| | | | | | | |
|---|---|---|---|---|---|---|
| | | | | | (0.06 to 0.11) | |
| Adolescence | Prevalence | Both | Switzerland | Psoriasis | -0.24<br>(-0.25 to -0.23) | < 0.001 |
| Adolescence | Prevalence | Female | Switzerland | Psoriasis | -0.23<br>(-0.24 to -0.22) | < 0.001 |
| Adolescence | Prevalence | Male | Switzerland | Psoriasis | -0.24<br>(-0.25 to -0.24) | < 0.001 |
| Adolescence | Prevalence | Both | Switzerland | RHD | -0.24<br>(-0.44 to -0.03) | 0.022 |
| Adolescence | Prevalence | Female | Switzerland | RHD | -0.3<br>(-0.47 to -0.13) | 0.001 |
| Adolescence | Prevalence | Male | Switzerland | RHD | -0.4<br>(-0.79 to 0) | 0.047 |
| Adolescence | Prevalence | Both | Switzerland | RA | 0.31<br>(0.29 to 0.34) | < 0.001 |
| Adolescence | Prevalence | Female | Switzerland | RA | 0.32<br>(0.31 to 0.33) | < 0.001 |
| Adolescence | Prevalence | Male | Switzerland | RA | 0.34<br>(0.32 to 0.37) | < 0.001 |
| Adolescence | Prevalence | Both | Syrian Arab Republic | AA | 0.01<br>(0 to 0.03) | 0.029 |
| Adolescence | Prevalence | Female | Syrian Arab Republic | AA | 0<br>(0 to 0) | 0.001 |
| Adolescence | Prevalence | Male | Syrian Arab Republic | AA | 0<br>(0 to 0) | 0.011 |
| Adolescence | Prevalence | Both | Syrian Arab Republic | T1D | 2.24<br>(2.14 to 2.33) | < 0.001 |
| Adolescence | Prevalence | Female | Syrian Arab Republic | T1D | 2.23<br>(2.06 to 2.39) | < 0.001 |
| Adolescence | Prevalence | Male | Syrian Arab Republic | T1D | 2.24<br>(2.16 to 2.33) | < 0.001 |
| Adolescence | Prevalence | Both | Syrian Arab Republic | IBD | 0.47<br>(0.44 to 0.51) | < 0.001 |

| | | | | | | |
|---|---|---|---|---|---|---|
| Adolescence | Prevalence | Female | Syrian Arab Republic | IBD | 0.49 (0.45 to 0.54) | < 0.001 |
| Adolescence | Prevalence | Male | Syrian Arab Republic | IBD | 0.45 (0.4 to 0.5) | < 0.001 |
| Adolescence | Prevalence | Both | Syrian Arab Republic | MS | 0.4 (0.31 to 0.48) | < 0.001 |
| Adolescence | Prevalence | Female | Syrian Arab Republic | MS | 0.33 (0.2 to 0.47) | < 0.001 |
| Adolescence | Prevalence | Male | Syrian Arab Republic | MS | 0.41 (0.38 to 0.45) | < 0.001 |
| Adolescence | Prevalence | Both | Syrian Arab Republic | Psoriasis | -1.17 (-1.18 to -1.16) | < 0.001 |
| Adolescence | Prevalence | Female | Syrian Arab Republic | Psoriasis | -1.15 (-1.16 to -1.14) | < 0.001 |
| Adolescence | Prevalence | Male | Syrian Arab Republic | Psoriasis | -1.22 (-1.23 to -1.21) | < 0.001 |
| Adolescence | Prevalence | Both | Syrian Arab Republic | RHD | 0.14 (0.11 to 0.17) | < 0.001 |
| Adolescence | Prevalence | Female | Syrian Arab Republic | RHD | 0.1 (0.05 to 0.16) | < 0.001 |
| Adolescence | Prevalence | Male | Syrian Arab Republic | RHD | 0.14 (0.11 to 0.16) | < 0.001 |
| Adolescence | Prevalence | Both | Syrian Arab Republic | RA | 0.56 (0.46 to 0.66) | < 0.001 |
| Adolescence | Prevalence | Female | Syrian Arab Republic | RA | 0.53 (0.4 to 0.66) | < 0.001 |
| Adolescence | Prevalence | Male | Syrian Arab Republic | RA | 0.57 (0.47 to 0.68) | < 0.001 |
| Adolescence | Prevalence | Both | Taiwan (Province of China) | AA | -0.02 (-0.02 to -0.02) | < 0.001 |
| Adolescence | Prevalence | Female | Taiwan (Province of China) | AA | 0 (0 to 0) | < 0.001 |
| Adolescence | Prevalence | Male | Taiwan (Province of China) | AA | 0 | < 0.001 |

| | | | | | | |
|---|---|---|---|---|---|---|
| | | | | | (0 to 0) | |
| Adolescence | Prevalence | Both | Taiwan (Province of China) | T1D | 1.96 (1.88 to 2.03) | < 0.001 |
| Adolescence | Prevalence | Female | Taiwan (Province of China) | T1D | 2.16 (2.05 to 2.26) | < 0.001 |
| Adolescence | Prevalence | Male | Taiwan (Province of China) | T1D | 1.75 (1.69 to 1.8) | < 0.001 |
| Adolescence | Prevalence | Both | Taiwan (Province of China) | IBD | 2.68 (2.29 to 3.06) | < 0.001 |
| Adolescence | Prevalence | Female | Taiwan (Province of China) | IBD | 2.74 (2.35 to 3.13) | < 0.001 |
| Adolescence | Prevalence | Male | Taiwan (Province of China) | IBD | 2.61 (2.24 to 2.99) | < 0.001 |
| Adolescence | Prevalence | Both | Taiwan (Province of China) | MS | 3.33 (3.32 to 3.34) | < 0.001 |
| Adolescence | Prevalence | Female | Taiwan (Province of China) | MS | 3.62 (3.57 to 3.66) | < 0.001 |
| Adolescence | Prevalence | Male | Taiwan (Province of China) | MS | 2.92 (2.82 to 3.01) | < 0.001 |
| Adolescence | Prevalence | Both | Taiwan (Province of China) | Psoriasis | -0.98 (-1.05 to -0.9) | < 0.001 |
| Adolescence | Prevalence | Female | Taiwan (Province of China) | Psoriasis | -1.03 (-1.08 to -0.99) | < 0.001 |
| Adolescence | Prevalence | Male | Taiwan (Province of China) | Psoriasis | -0.93 (-1.02 to -0.84) | < 0.001 |
| Adolescence | Prevalence | Both | Taiwan (Province of China) | RHD | 1.49 (1.22 to 1.76) | < 0.001 |
| Adolescence | Prevalence | Female | Taiwan (Province of China) | RHD | 1.26 (0.96 to 1.55) | < 0.001 |
| Adolescence | Prevalence | Male | Taiwan (Province of China) | RHD | 1.76 (1.58 to 1.94) | < 0.001 |
| Adolescence | Prevalence | Both | Taiwan (Province of China) | RA | 0.3 (0.1 to 0.51) | 0.004 |

| | | | | | | |
|---|---|---|---|---|---|---|
| Adolescence | Prevalence | Female | Taiwan (Province of China) | RA | 0.36 (0.13 to 0.58) | 0.002 |
| Adolescence | Prevalence | Male | Taiwan (Province of China) | RA | 0.25 (0.06 to 0.45) | 0.012 |
| Adolescence | Prevalence | Both | Tajikistan | AA | -0.03 (-0.03 to -0.02) | < 0.001 |
| Adolescence | Prevalence | Female | Tajikistan | AA | 0 (0 to 0) | < 0.001 |
| Adolescence | Prevalence | Male | Tajikistan | AA | 0 (0 to 0) | 1.000 |
| Adolescence | Prevalence | Both | Tajikistan | T1D | 1.24 (1.18 to 1.3) | < 0.001 |
| Adolescence | Prevalence | Female | Tajikistan | T1D | 1.18 (1.1 to 1.26) | < 0.001 |
| Adolescence | Prevalence | Male | Tajikistan | T1D | 1.26 (1.2 to 1.31) | < 0.001 |
| Adolescence | Prevalence | Both | Tajikistan | IBD | 0.06 (0.01 to 0.12) | 0.028 |
| Adolescence | Prevalence | Female | Tajikistan | IBD | 0.08 (0.01 to 0.14) | 0.018 |
| Adolescence | Prevalence | Male | Tajikistan | IBD | 0.09 (-0.01 to 0.18) | 0.080 |
| Adolescence | Prevalence | Both | Tajikistan | MS | 0.25 (0.2 to 0.3) | < 0.001 |
| Adolescence | Prevalence | Female | Tajikistan | MS | 0.27 (0.21 to 0.34) | < 0.001 |
| Adolescence | Prevalence | Male | Tajikistan | MS | 0.23 (0.09 to 0.37) | 0.001 |
| Adolescence | Prevalence | Both | Tajikistan | Psoriasis | -0.68 (-0.79 to -0.57) | < 0.001 |
| Adolescence | Prevalence | Female | Tajikistan | Psoriasis | -0.68 (-0.76 to -0.6) | < 0.001 |
| Adolescence | Prevalence | Male | Tajikistan | Psoriasis | -0.65 | < 0.001 |

| | | | | | | |
|---|---|---|---|---|---|---|
| | | | | | (-0.75 to -0.55) | |
| Adolescence | Prevalence | Both | Tajikistan | RHD | 0.05<br>(0.01 to 0.1) | 0.010 |
| Adolescence | Prevalence | Female | Tajikistan | RHD | 0.07<br>(0.02 to 0.13) | 0.012 |
| Adolescence | Prevalence | Male | Tajikistan | RHD | 0.03<br>(-0.01 to 0.06) | 0.103 |
| Adolescence | Prevalence | Both | Tajikistan | RA | 0.26<br>(0.2 to 0.33) | < 0.001 |
| Adolescence | Prevalence | Female | Tajikistan | RA | 0.28<br>(0.21 to 0.35) | < 0.001 |
| Adolescence | Prevalence | Male | Tajikistan | RA | 0.38<br>(0.37 to 0.39) | < 0.001 |
| Adolescence | Prevalence | Both | Thailand | AA | -0.01<br>(-0.02 to 0) | 0.073 |
| Adolescence | Prevalence | Female | Thailand | AA | 0<br>(0 to 0) | 0.008 |
| Adolescence | Prevalence | Male | Thailand | AA | 0<br>(0 to 0) | < 0.001 |
| Adolescence | Prevalence | Both | Thailand | T1D | 0.62<br>(0.6 to 0.63) | < 0.001 |
| Adolescence | Prevalence | Female | Thailand | T1D | 0.57<br>(0.56 to 0.59) | < 0.001 |
| Adolescence | Prevalence | Male | Thailand | T1D | 0.66<br>(0.64 to 0.68) | < 0.001 |
| Adolescence | Prevalence | Both | Thailand | IBD | 1.12<br>(0.98 to 1.26) | < 0.001 |
| Adolescence | Prevalence | Female | Thailand | IBD | 1.16<br>(0.9 to 1.43) | < 0.001 |
| Adolescence | Prevalence | Male | Thailand | IBD | 1.1<br>(0.98 to 1.22) | < 0.001 |
| Adolescence | Prevalence | Both | Thailand | MS | -0.08<br>(-0.1 to -0.05) | < 0.001 |

| | | | | | | |
|---|---|---|---|---|---|---|
| Adolescence | Prevalence | Female | Thailand | MS | -0.01 (-0.09 to 0.06) | 0.747 |
| Adolescence | Prevalence | Male | Thailand | MS | -0.06 (-0.24 to 0.13) | 0.561 |
| Adolescence | Prevalence | Both | Thailand | Psoriasis | -0.5 (-0.52 to -0.47) | < 0.001 |
| Adolescence | Prevalence | Female | Thailand | Psoriasis | -0.47 (-0.49 to -0.46) | < 0.001 |
| Adolescence | Prevalence | Male | Thailand | Psoriasis | -0.53 (-0.57 to -0.48) | < 0.001 |
| Adolescence | Prevalence | Both | Thailand | RHD | -0.07 (-0.1 to -0.03) | < 0.001 |
| Adolescence | Prevalence | Female | Thailand | RHD | -0.07 (-0.08 to -0.06) | < 0.001 |
| Adolescence | Prevalence | Male | Thailand | RHD | -0.05 (-0.12 to 0.02) | 0.143 |
| Adolescence | Prevalence | Both | Thailand | RA | 0.59 (0.56 to 0.63) | < 0.001 |
| Adolescence | Prevalence | Female | Thailand | RA | 0.66 (0.64 to 0.68) | < 0.001 |
| Adolescence | Prevalence | Male | Thailand | RA | 0.52 (0.49 to 0.55) | < 0.001 |
| Adolescence | Prevalence | Both | Timor-Leste | AA | 0.03 (0.02 to 0.03) | < 0.001 |
| Adolescence | Prevalence | Female | Timor-Leste | AA | 0 (0 to 0) | 0.008 |
| Adolescence | Prevalence | Male | Timor-Leste | AA | 0 (0 to 0) | < 0.001 |
| Adolescence | Prevalence | Both | Timor-Leste | T1D | 0.52 (0.51 to 0.54) | < 0.001 |
| Adolescence | Prevalence | Female | Timor-Leste | T1D | 0.48 (0.46 to 0.5) | < 0.001 |
| Adolescence | Prevalence | Male | Timor-Leste | T1D | 0.56 | < 0.001 |

| | | | | | | |
|---|---|---|---|---|---|---|
| | | | | | (0.53 to 0.59) | |
| Adolescence | Prevalence | Both | Timor-Leste | IBD | 0.71<br>(0.66 to 0.76) | < 0.001 |
| Adolescence | Prevalence | Female | Timor-Leste | IBD | 0.73<br>(0.51 to 0.94) | < 0.001 |
| Adolescence | Prevalence | Male | Timor-Leste | IBD | 0.7<br>(0.66 to 0.75) | < 0.001 |
| Adolescence | Prevalence | Both | Timor-Leste | MS | 0.08<br>(0.08 to 0.08) | < 0.001 |
| Adolescence | Prevalence | Female | Timor-Leste | MS | -0.02<br>(-0.12 to 0.09) | 0.734 |
| Adolescence | Prevalence | Male | Timor-Leste | MS | 0.02<br>(0.02 to 0.02) | < 0.001 |
| Adolescence | Prevalence | Both | Timor-Leste | Psoriasis | -0.69<br>(-0.76 to -0.63) | < 0.001 |
| Adolescence | Prevalence | Female | Timor-Leste | Psoriasis | -0.71<br>(-0.75 to -0.67) | < 0.001 |
| Adolescence | Prevalence | Male | Timor-Leste | Psoriasis | -0.74<br>(-0.82 to -0.66) | < 0.001 |
| Adolescence | Prevalence | Both | Timor-Leste | RHD | 0.14<br>(0.12 to 0.16) | < 0.001 |
| Adolescence | Prevalence | Female | Timor-Leste | RHD | 0.11<br>(0.1 to 0.11) | < 0.001 |
| Adolescence | Prevalence | Male | Timor-Leste | RHD | 0.13<br>(0.08 to 0.19) | < 0.001 |
| Adolescence | Prevalence | Both | Timor-Leste | RA | 0.25<br>(0.23 to 0.26) | < 0.001 |
| Adolescence | Prevalence | Female | Timor-Leste | RA | 0.26<br>(0.25 to 0.28) | < 0.001 |
| Adolescence | Prevalence | Male | Timor-Leste | RA | 0.14<br>(0.08 to 0.2) | < 0.001 |
| Adolescence | Prevalence | Both | Togo | AA | -0.03<br>(-0.05 to -0.02) | < 0.001 |

| | | | | | | |
|---|---|---|---|---|---|---|
| Adolescence | Prevalence | Female | Togo | AA | 0<br>(0 to 0) | < 0.001 |
| Adolescence | Prevalence | Male | Togo | AA | 0<br>(0 to 0) | < 0.001 |
| Adolescence | Prevalence | Both | Togo | T1D | 0.12<br>(0.11 to 0.13) | < 0.001 |
| Adolescence | Prevalence | Female | Togo | T1D | 0.11<br>(0.1 to 0.11) | < 0.001 |
| Adolescence | Prevalence | Male | Togo | T1D | 0.13<br>(0.11 to 0.16) | < 0.001 |
| Adolescence | Prevalence | Both | Togo | IBD | -0.05<br>(-0.1 to 0.01) | 0.120 |
| Adolescence | Prevalence | Female | Togo | IBD | -0.07<br>(-0.16 to 0.03) | 0.171 |
| Adolescence | Prevalence | Male | Togo | IBD | 0.01<br>(-0.06 to 0.08) | 0.748 |
| Adolescence | Prevalence | Both | Togo | MS | 0.07<br>(-0.04 to 0.18) | 0.213 |
| Adolescence | Prevalence | Female | Togo | MS | 0.07<br>(0 to 0.14) | 0.049 |
| Adolescence | Prevalence | Male | Togo | MS | 0.11<br>(-0.01 to 0.23) | 0.062 |
| Adolescence | Prevalence | Both | Togo | Psoriasis | -0.58<br>(-0.71 to -0.46) | < 0.001 |
| Adolescence | Prevalence | Female | Togo | Psoriasis | -0.65<br>(-0.77 to -0.54) | < 0.001 |
| Adolescence | Prevalence | Male | Togo | Psoriasis | -0.5<br>(-0.65 to -0.34) | < 0.001 |
| Adolescence | Prevalence | Both | Togo | RHD | 0.2<br>(0.16 to 0.24) | < 0.001 |
| Adolescence | Prevalence | Female | Togo | RHD | 0.19<br>(0.15 to 0.23) | < 0.001 |
| Adolescence | Prevalence | Male | Togo | RHD | 0.21 | < 0.001 |

| | | | | | | |
|---|---|---|---|---|---|---|
| | | | | | (0.18 to 0.24) | |
| Adolescence | Prevalence | Both | Togo | RA | 0.33 (0.29 to 0.37) | < 0.001 |
| Adolescence | Prevalence | Female | Togo | RA | 0.42 (0.38 to 0.45) | < 0.001 |
| Adolescence | Prevalence | Male | Togo | RA | 0.3 (0.25 to 0.35) | < 0.001 |
| Adolescence | Prevalence | Both | Tokelau | AA | -0.06 (-0.29 to 0.18) | 0.631 |
| Adolescence | Prevalence | Female | Tokelau | AA | 0 (0 to 0) | 0.008 |
| Adolescence | Prevalence | Male | Tokelau | AA | 0 (0 to 0) | < 0.001 |
| Adolescence | Prevalence | Both | Tokelau | T1D | 0.54 (0.52 to 0.56) | < 0.001 |
| Adolescence | Prevalence | Female | Tokelau | T1D | 0.54 (0.5 to 0.58) | < 0.001 |
| Adolescence | Prevalence | Male | Tokelau | T1D | 0.55 (0.53 to 0.57) | < 0.001 |
| Adolescence | Prevalence | Both | Tokelau | IBD | 0.57 (0.46 to 0.68) | < 0.001 |
| Adolescence | Prevalence | Female | Tokelau | IBD | 0.6 (0.55 to 0.65) | < 0.001 |
| Adolescence | Prevalence | Male | Tokelau | IBD | 0.58 (0.53 to 0.63) | < 0.001 |
| Adolescence | Prevalence | Both | Tokelau | MS | 0.04 (-0.02 to 0.11) | 0.185 |
| Adolescence | Prevalence | Female | Tokelau | MS | 0.08 (-0.09 to 0.24) | 0.360 |
| Adolescence | Prevalence | Male | Tokelau | MS | 0.12 (-0.05 to 0.29) | 0.179 |
| Adolescence | Prevalence | Both | Tokelau | Psoriasis | -0.66 (-0.72 to -0.61) | < 0.001 |

| | | | | | | |
|---|---|---|---|---|---|---|
| Adolescence | Prevalence | Female | Tokelau | Psoriasis | -0.62 (-0.62 to -0.61) | < 0.001 |
| Adolescence | Prevalence | Male | Tokelau | Psoriasis | -0.66 (-0.69 to -0.63) | < 0.001 |
| Adolescence | Prevalence | Both | Tokelau | RHD | -0.44 (-0.53 to -0.35) | < 0.001 |
| Adolescence | Prevalence | Female | Tokelau | RHD | -0.31 (-0.4 to -0.22) | < 0.001 |
| Adolescence | Prevalence | Male | Tokelau | RHD | -0.56 (-0.62 to -0.5) | < 0.001 |
| Adolescence | Prevalence | Both | Tokelau | RA | 0.58 (0.26 to 0.9) | < 0.001 |
| Adolescence | Prevalence | Female | Tokelau | RA | 0.73 (0.69 to 0.77) | < 0.001 |
| Adolescence | Prevalence | Male | Tokelau | RA | 0.55 (0.51 to 0.58) | < 0.001 |
| Adolescence | Prevalence | Both | Tonga | AA | 0.01 (0 to 0.01) | < 0.001 |
| Adolescence | Prevalence | Female | Tonga | AA | 0 (0 to 0) | 0.008 |
| Adolescence | Prevalence | Male | Tonga | AA | 0 (0 to 0) | < 0.001 |
| Adolescence | Prevalence | Both | Tonga | T1D | 0.64 (0.61 to 0.68) | < 0.001 |
| Adolescence | Prevalence | Female | Tonga | T1D | 0.62 (0.58 to 0.66) | < 0.001 |
| Adolescence | Prevalence | Male | Tonga | T1D | 0.68 (0.64 to 0.72) | < 0.001 |
| Adolescence | Prevalence | Both | Tonga | IBD | 0.07 (-0.03 to 0.17) | 0.186 |
| Adolescence | Prevalence | Female | Tonga | IBD | 0.08 (0.02 to 0.13) | 0.006 |
| Adolescence | Prevalence | Male | Tonga | IBD | 0.08 | 0.007 |

| | | | | | | |
|---|---|---|---|---|---|---|
| | | | | | (0.02 to 0.15) | |
| Adolescence | Prevalence | Both | Tonga | MS | 0.02 (-0.14 to 0.18) | 0.781 |
| Adolescence | Prevalence | Female | Tonga | MS | 0.02 (-0.01 to 0.05) | 0.267 |
| Adolescence | Prevalence | Male | Tonga | MS | 0.04 (-0.11 to 0.18) | 0.624 |
| Adolescence | Prevalence | Both | Tonga | Psoriasis | -0.45 (-0.47 to -0.43) | < 0.001 |
| Adolescence | Prevalence | Female | Tonga | Psoriasis | -0.44 (-0.47 to -0.4) | < 0.001 |
| Adolescence | Prevalence | Male | Tonga | Psoriasis | -0.47 (-0.5 to -0.45) | < 0.001 |
| Adolescence | Prevalence | Both | Tonga | RHD | 0.05 (-0.64 to 0.75) | 0.887 |
| Adolescence | Prevalence | Female | Tonga | RHD | 0.08 (-0.55 to 0.72) | 0.796 |
| Adolescence | Prevalence | Male | Tonga | RHD | 0.12 (-0.6 to 0.85) | 0.745 |
| Adolescence | Prevalence | Both | Tonga | RA | 0.36 (0.3 to 0.43) | < 0.001 |
| Adolescence | Prevalence | Female | Tonga | RA | 0.41 (0.35 to 0.47) | < 0.001 |
| Adolescence | Prevalence | Male | Tonga | RA | 0.29 (0.24 to 0.34) | < 0.001 |
| Adolescence | Prevalence | Both | Trinidad and Tobago | AA | 0 (-0.01 to 0) | < 0.001 |
| Adolescence | Prevalence | Female | Trinidad and Tobago | AA | 0 (0 to 0) | < 0.001 |
| Adolescence | Prevalence | Male | Trinidad and Tobago | AA | 0 (0 to 0) | 1.000 |
| Adolescence | Prevalence | Both | Trinidad and Tobago | T1D | 1.08 (1.07 to 1.09) | < 0.001 |

| | | | | | | |
|---|---|---|---|---|---|---|
| Adolescence | Prevalence | Female | Trinidad and Tobago | T1D | 1.01 (0.99 to 1.04) | < 0.001 |
| Adolescence | Prevalence | Male | Trinidad and Tobago | T1D | 1.15 (1.13 to 1.16) | < 0.001 |
| Adolescence | Prevalence | Both | Trinidad and Tobago | IBD | 0.03 (-0.01 to 0.07) | 0.164 |
| Adolescence | Prevalence | Female | Trinidad and Tobago | IBD | 0.08 (0.03 to 0.13) | 0.001 |
| Adolescence | Prevalence | Male | Trinidad and Tobago | IBD | 0.01 (-0.02 to 0.05) | 0.409 |
| Adolescence | Prevalence | Both | Trinidad and Tobago | MS | 0.35 (0.29 to 0.42) | < 0.001 |
| Adolescence | Prevalence | Female | Trinidad and Tobago | MS | 0.46 (0.38 to 0.54) | < 0.001 |
| Adolescence | Prevalence | Male | Trinidad and Tobago | MS | 0.17 (0.01 to 0.33) | 0.034 |
| Adolescence | Prevalence | Both | Trinidad and Tobago | Psoriasis | -0.41 (-0.44 to -0.38) | < 0.001 |
| Adolescence | Prevalence | Female | Trinidad and Tobago | Psoriasis | -0.42 (-0.44 to -0.39) | < 0.001 |
| Adolescence | Prevalence | Male | Trinidad and Tobago | Psoriasis | -0.41 (-0.44 to -0.38) | < 0.001 |
| Adolescence | Prevalence | Both | Trinidad and Tobago | RHD | 0.01 (0 to 0.02) | 0.043 |
| Adolescence | Prevalence | Female | Trinidad and Tobago | RHD | 0.03 (-0.01 to 0.08) | 0.156 |
| Adolescence | Prevalence | Male | Trinidad and Tobago | RHD | -0.02 (-0.04 to 0) | 0.098 |
| Adolescence | Prevalence | Both | Trinidad and Tobago | RA | 0.66 (0.63 to 0.69) | < 0.001 |
| Adolescence | Prevalence | Female | Trinidad and Tobago | RA | 0.66 (0.61 to 0.71) | < 0.001 |
| Adolescence | Prevalence | Male | Trinidad and Tobago | RA | 0.71 | < 0.001 |

| | | | | | | |
|---|---|---|---|---|---|---|
| | | | | | (0.65 to 0.77) | |
| Adolescence | Prevalence | Both | Tunisia | AA | -0.01<br>(-0.01 to -0.01) | < 0.001 |
| Adolescence | Prevalence | Female | Tunisia | AA | 0<br>(0 to 0) | 0.001 |
| Adolescence | Prevalence | Male | Tunisia | AA | 0<br>(0 to 0) | 0.011 |
| Adolescence | Prevalence | Both | Tunisia | T1D | 2.43<br>(2.39 to 2.47) | < 0.001 |
| Adolescence | Prevalence | Female | Tunisia | T1D | 2.35<br>(2.32 to 2.38) | < 0.001 |
| Adolescence | Prevalence | Male | Tunisia | T1D | 2.48<br>(2.44 to 2.53) | < 0.001 |
| Adolescence | Prevalence | Both | Tunisia | IBD | 0.41<br>(0.39 to 0.43) | < 0.001 |
| Adolescence | Prevalence | Female | Tunisia | IBD | 0.37<br>(0.35 to 0.39) | < 0.001 |
| Adolescence | Prevalence | Male | Tunisia | IBD | 0.47<br>(0.44 to 0.49) | < 0.001 |
| Adolescence | Prevalence | Both | Tunisia | MS | 0.41<br>(0.37 to 0.45) | < 0.001 |
| Adolescence | Prevalence | Female | Tunisia | MS | 0.4<br>(0.34 to 0.46) | < 0.001 |
| Adolescence | Prevalence | Male | Tunisia | MS | 0.5<br>(0.47 to 0.53) | < 0.001 |
| Adolescence | Prevalence | Both | Tunisia | Psoriasis | -1.13<br>(-1.16 to -1.1) | < 0.001 |
| Adolescence | Prevalence | Female | Tunisia | Psoriasis | -1.1<br>(-1.11 to -1.08) | < 0.001 |
| Adolescence | Prevalence | Male | Tunisia | Psoriasis | -1.18<br>(-1.22 to -1.15) | < 0.001 |
| Adolescence | Prevalence | Both | Tunisia | RHD | -1.13<br>(-1.24 to -1.02) | < 0.001 |

| | | | | | | |
|---|---|---|---|---|---|---|
| Adolescence | Prevalence | Female | Tunisia | RHD | -1.42<br>(-1.56 to -1.28) | < 0.001 |
| Adolescence | Prevalence | Male | Tunisia | RHD | -0.7<br>(-0.76 to -0.64) | < 0.001 |
| Adolescence | Prevalence | Both | Tunisia | RA | 0.67<br>(0.66 to 0.68) | < 0.001 |
| Adolescence | Prevalence | Female | Tunisia | RA | 0.66<br>(0.64 to 0.69) | < 0.001 |
| Adolescence | Prevalence | Male | Tunisia | RA | 0.73<br>(0.68 to 0.78) | < 0.001 |
| Adolescence | Prevalence | Both | Turkey | AA | 0.01<br>(0.01 to 0.01) | < 0.001 |
| Adolescence | Prevalence | Female | Turkey | AA | 0<br>(0 to 0) | 0.001 |
| Adolescence | Prevalence | Male | Turkey | AA | 0.04<br>(0.04 to 0.04) | < 0.001 |
| Adolescence | Prevalence | Both | Turkey | T1D | 2.17<br>(2.14 to 2.2) | < 0.001 |
| Adolescence | Prevalence | Female | Turkey | T1D | 2.16<br>(2.14 to 2.18) | < 0.001 |
| Adolescence | Prevalence | Male | Turkey | T1D | 2.18<br>(2.14 to 2.21) | < 0.001 |
| Adolescence | Prevalence | Both | Turkey | IBD | 1.58<br>(1.26 to 1.89) | < 0.001 |
| Adolescence | Prevalence | Female | Turkey | IBD | 1.39<br>(1.08 to 1.7) | < 0.001 |
| Adolescence | Prevalence | Male | Turkey | IBD | 1.88<br>(1.66 to 2.1) | < 0.001 |
| Adolescence | Prevalence | Both | Turkey | MS | 0.04<br>(0.02 to 0.05) | < 0.001 |
| Adolescence | Prevalence | Female | Turkey | MS | 0.03<br>(0 to 0.06) | 0.021 |
| Adolescence | Prevalence | Male | Turkey | MS | 0.06 | < 0.001 |

| | | | | | | |
|---|---|---|---|---|---|---|
| | | | | | (0.04 to 0.09) | |
| Adolescence | Prevalence | Both | Turkey | Psoriasis | -1.15<br>(-1.17 to -1.14) | < 0.001 |
| Adolescence | Prevalence | Female | Turkey | Psoriasis | -1.14<br>(-1.16 to -1.13) | < 0.001 |
| Adolescence | Prevalence | Male | Turkey | Psoriasis | -1.17<br>(-1.18 to -1.15) | < 0.001 |
| Adolescence | Prevalence | Both | Turkey | RHD | -0.07<br>(-0.1 to -0.04) | < 0.001 |
| Adolescence | Prevalence | Female | Turkey | RHD | -0.1<br>(-0.13 to -0.06) | < 0.001 |
| Adolescence | Prevalence | Male | Turkey | RHD | -0.03<br>(-0.15 to 0.08) | 0.546 |
| Adolescence | Prevalence | Both | Turkey | RA | 0.74<br>(0.72 to 0.77) | < 0.001 |
| Adolescence | Prevalence | Female | Turkey | RA | 0.73<br>(0.69 to 0.77) | < 0.001 |
| Adolescence | Prevalence | Male | Turkey | RA | 0.82<br>(0.71 to 0.93) | < 0.001 |
| Adolescence | Prevalence | Both | Turkmenistan | AA | -0.03<br>(-0.03 to -0.03) | < 0.001 |
| Adolescence | Prevalence | Female | Turkmenistan | AA | 0<br>(0 to 0) | < 0.001 |
| Adolescence | Prevalence | Male | Turkmenistan | AA | 0<br>(0 to 0) | 1.000 |
| Adolescence | Prevalence | Both | Turkmenistan | T1D | 1.19<br>(1.15 to 1.24) | < 0.001 |
| Adolescence | Prevalence | Female | Turkmenistan | T1D | 1.16<br>(1.11 to 1.21) | < 0.001 |
| Adolescence | Prevalence | Male | Turkmenistan | T1D | 1.22<br>(1.17 to 1.27) | < 0.001 |
| Adolescence | Prevalence | Both | Turkmenistan | IBD | 0.35<br>(0.19 to 0.5) | < 0.001 |

| | | | | | | |
|---|---|---|---|---|---|---|
| Adolescence | Prevalence | Female | Turkmenistan | IBD | 0.35<br>(0.11 to 0.58) | 0.004 |
| Adolescence | Prevalence | Male | Turkmenistan | IBD | 0.35<br>(0.27 to 0.44) | < 0.001 |
| Adolescence | Prevalence | Both | Turkmenistan | MS | 0.27<br>(0.17 to 0.37) | < 0.001 |
| Adolescence | Prevalence | Female | Turkmenistan | MS | 0.34<br>(0.28 to 0.4) | < 0.001 |
| Adolescence | Prevalence | Male | Turkmenistan | MS | 0.28<br>(0.18 to 0.38) | < 0.001 |
| Adolescence | Prevalence | Both | Turkmenistan | Psoriasis | -1.01<br>(-1.11 to -0.91) | < 0.001 |
| Adolescence | Prevalence | Female | Turkmenistan | Psoriasis | -1.01<br>(-1.13 to -0.89) | < 0.001 |
| Adolescence | Prevalence | Male | Turkmenistan | Psoriasis | -0.98<br>(-1.02 to -0.93) | < 0.001 |
| Adolescence | Prevalence | Both | Turkmenistan | RHD | -0.01<br>(-0.1 to 0.08) | 0.816 |
| Adolescence | Prevalence | Female | Turkmenistan | RHD | 0<br>(-0.04 to 0.03) | 0.924 |
| Adolescence | Prevalence | Male | Turkmenistan | RHD | 0.02<br>(-0.04 to 0.07) | 0.537 |
| Adolescence | Prevalence | Both | Turkmenistan | RA | 0.68<br>(0.64 to 0.72) | < 0.001 |
| Adolescence | Prevalence | Female | Turkmenistan | RA | 0.71<br>(0.65 to 0.77) | < 0.001 |
| Adolescence | Prevalence | Male | Turkmenistan | RA | 0.74<br>(0.69 to 0.78) | < 0.001 |
| Adolescence | Prevalence | Both | Tuvalu | AA | -0.05<br>(-0.05 to -0.04) | < 0.001 |
| Adolescence | Prevalence | Female | Tuvalu | AA | 0<br>(0 to 0) | 0.008 |
| Adolescence | Prevalence | Male | Tuvalu | AA | 0 | < 0.001 |

| | | | | | | |
|---|---|---|---|---|---|---|
| | | | | | (0 to 0) | |
| Adolescence | Prevalence | Both | Tuvalu | T1D | 0.57<br>(0.53 to 0.61) | < 0.001 |
| Adolescence | Prevalence | Female | Tuvalu | T1D | 0.53<br>(0.44 to 0.62) | < 0.001 |
| Adolescence | Prevalence | Male | Tuvalu | T1D | 0.6<br>(0.56 to 0.64) | < 0.001 |
| Adolescence | Prevalence | Both | Tuvalu | IBD | 0.06<br>(-0.01 to 0.14) | 0.111 |
| Adolescence | Prevalence | Female | Tuvalu | IBD | 0.1<br>(0 to 0.19) | 0.046 |
| Adolescence | Prevalence | Male | Tuvalu | IBD | 0.06<br>(-0.08 to 0.19) | 0.427 |
| Adolescence | Prevalence | Both | Tuvalu | MS | -0.01<br>(-0.03 to 0.01) | 0.158 |
| Adolescence | Prevalence | Female | Tuvalu | MS | 0.01<br>(-0.3 to 0.33) | 0.946 |
| Adolescence | Prevalence | Male | Tuvalu | MS | -0.03<br>(-0.22 to 0.17) | 0.784 |
| Adolescence | Prevalence | Both | Tuvalu | Psoriasis | -0.57<br>(-0.59 to -0.56) | < 0.001 |
| Adolescence | Prevalence | Female | Tuvalu | Psoriasis | -0.52<br>(-0.54 to -0.51) | < 0.001 |
| Adolescence | Prevalence | Male | Tuvalu | Psoriasis | -0.56<br>(-0.59 to -0.53) | < 0.001 |
| Adolescence | Prevalence | Both | Tuvalu | RHD | -0.51<br>(-0.61 to -0.41) | < 0.001 |
| Adolescence | Prevalence | Female | Tuvalu | RHD | -0.5<br>(-0.62 to -0.37) | < 0.001 |
| Adolescence | Prevalence | Male | Tuvalu | RHD | -0.5<br>(-0.72 to -0.28) | < 0.001 |
| Adolescence | Prevalence | Both | Tuvalu | RA | 0.43<br>(0.41 to 0.45) | < 0.001 |

| | | | | | | |
|---|---|---|---|---|---|---|
| Adolescence | Prevalence | Female | Tuvalu | RA | 0.54<br>(0.52 to 0.56) | < 0.001 |
| Adolescence | Prevalence | Male | Tuvalu | RA | 0.38<br>(0.33 to 0.44) | < 0.001 |
| Adolescence | Prevalence | Both | Uganda | AA | -0.03<br>(-0.03 to -0.03) | < 0.001 |
| Adolescence | Prevalence | Female | Uganda | AA | 0<br>(0 to 0) | < 0.001 |
| Adolescence | Prevalence | Male | Uganda | AA | 0<br>(0 to 0) | < 0.001 |
| Adolescence | Prevalence | Both | Uganda | T1D | 0.1<br>(0.05 to 0.15) | < 0.001 |
| Adolescence | Prevalence | Female | Uganda | T1D | 0.12<br>(0.05 to 0.18) | 0.001 |
| Adolescence | Prevalence | Male | Uganda | T1D | 0.1<br>(0.05 to 0.15) | < 0.001 |
| Adolescence | Prevalence | Both | Uganda | IBD | 0.4<br>(0.28 to 0.53) | < 0.001 |
| Adolescence | Prevalence | Female | Uganda | IBD | 0.3<br>(0.16 to 0.44) | < 0.001 |
| Adolescence | Prevalence | Male | Uganda | IBD | 0.49<br>(0.35 to 0.63) | < 0.001 |
| Adolescence | Prevalence | Both | Uganda | MS | -0.04<br>(-0.23 to 0.16) | 0.711 |
| Adolescence | Prevalence | Female | Uganda | MS | -0.01<br>(-0.01 to -0.01) | < 0.001 |
| Adolescence | Prevalence | Male | Uganda | MS | 0.13<br>(-0.09 to 0.36) | 0.253 |
| Adolescence | Prevalence | Both | Uganda | Psoriasis | -0.59<br>(-0.64 to -0.54) | < 0.001 |
| Adolescence | Prevalence | Female | Uganda | Psoriasis | -0.6<br>(-0.65 to -0.55) | < 0.001 |
| Adolescence | Prevalence | Male | Uganda | Psoriasis | -0.57 | < 0.001 |

| | | | | | | |
|---|---|---|---|---|---|---|
| | | | | | (-0.63 to -0.52) | |
| Adolescence | Prevalence | Both | Uganda | RHD | 0.89<br>(-0.07 to 1.86) | 0.071 |
| Adolescence | Prevalence | Female | Uganda | RHD | 0.89<br>(-0.02 to 1.81) | 0.055 |
| Adolescence | Prevalence | Male | Uganda | RHD | 0.91<br>(0.13 to 1.7) | 0.022 |
| Adolescence | Prevalence | Both | Uganda | RA | 0.21<br>(0.17 to 0.24) | < 0.001 |
| Adolescence | Prevalence | Female | Uganda | RA | 0.21<br>(0.17 to 0.25) | < 0.001 |
| Adolescence | Prevalence | Male | Uganda | RA | 0.33<br>(0.29 to 0.37) | < 0.001 |
| Adolescence | Prevalence | Both | Ukraine | AA | -0.01<br>(-0.01 to -0.01) | < 0.001 |
| Adolescence | Prevalence | Female | Ukraine | AA | 0<br>(0 to 0) | < 0.001 |
| Adolescence | Prevalence | Male | Ukraine | AA | 0<br>(0 to 0) | < 0.001 |
| Adolescence | Prevalence | Both | Ukraine | T1D | 1.32<br>(1.21 to 1.43) | < 0.001 |
| Adolescence | Prevalence | Female | Ukraine | T1D | 1.38<br>(1.32 to 1.43) | < 0.001 |
| Adolescence | Prevalence | Male | Ukraine | T1D | 1.21<br>(1.1 to 1.31) | < 0.001 |
| Adolescence | Prevalence | Both | Ukraine | IBD | 0.03<br>(0 to 0.06) | 0.085 |
| Adolescence | Prevalence | Female | Ukraine | IBD | 0.06<br>(0 to 0.13) | 0.051 |
| Adolescence | Prevalence | Male | Ukraine | IBD | 0.02<br>(-0.02 to 0.06) | 0.384 |
| Adolescence | Prevalence | Both | Ukraine | MS | 0.04<br>(-0.04 to 0.13) | 0.294 |

| | | | | | | |
|---|---|---|---|---|---|---|
| Adolescence | Prevalence | Female | Ukraine | MS | 0.05 (-0.03 to 0.13) | 0.211 |
| Adolescence | Prevalence | Male | Ukraine | MS | 0.13 (0 to 0.26) | 0.058 |
| Adolescence | Prevalence | Both | Ukraine | Psoriasis | -0.54 (-0.56 to -0.51) | < 0.001 |
| Adolescence | Prevalence | Female | Ukraine | Psoriasis | -0.53 (-0.55 to -0.5) | < 0.001 |
| Adolescence | Prevalence | Male | Ukraine | Psoriasis | -0.54 (-0.57 to -0.51) | < 0.001 |
| Adolescence | Prevalence | Both | Ukraine | RHD | 0.19 (0.1 to 0.28) | < 0.001 |
| Adolescence | Prevalence | Female | Ukraine | RHD | 0.23 (0.05 to 0.4) | 0.012 |
| Adolescence | Prevalence | Male | Ukraine | RHD | 0.14 (-0.04 to 0.33) | 0.133 |
| Adolescence | Prevalence | Both | Ukraine | RA | 0.28 (0.25 to 0.3) | < 0.001 |
| Adolescence | Prevalence | Female | Ukraine | RA | 0.29 (0.28 to 0.31) | < 0.001 |
| Adolescence | Prevalence | Male | Ukraine | RA | 0.28 (0.24 to 0.33) | < 0.001 |
| Adolescence | Prevalence | Both | United Arab Emirates | AA | 0.03 (-0.06 to 0.12) | 0.523 |
| Adolescence | Prevalence | Female | United Arab Emirates | AA | 0 (0 to 0) | 0.001 |
| Adolescence | Prevalence | Male | United Arab Emirates | AA | 0 (0 to 0) | 0.011 |
| Adolescence | Prevalence | Both | United Arab Emirates | T1D | 2.63 (2.58 to 2.68) | < 0.001 |
| Adolescence | Prevalence | Female | United Arab Emirates | T1D | 2.61 (2.56 to 2.65) | < 0.001 |
| Adolescence | Prevalence | Male | United Arab Emirates | T1D | 2.66 | < 0.001 |

| | | | | | | |
|---|---|---|---|---|---|---|
| | | | | | (2.58 to 2.75) | |
| Adolescence | Prevalence | Both | United Arab Emirates | IBD | 0.51 (0.4 to 0.62) | < 0.001 |
| Adolescence | Prevalence | Female | United Arab Emirates | IBD | 0.46 (0.42 to 0.51) | < 0.001 |
| Adolescence | Prevalence | Male | United Arab Emirates | IBD | 0.54 (0.5 to 0.59) | < 0.001 |
| Adolescence | Prevalence | Both | United Arab Emirates | MS | 0.67 (0.37 to 0.97) | < 0.001 |
| Adolescence | Prevalence | Female | United Arab Emirates | MS | 0.52 (0.45 to 0.59) | < 0.001 |
| Adolescence | Prevalence | Male | United Arab Emirates | MS | 0.77 (0.5 to 1.04) | < 0.001 |
| Adolescence | Prevalence | Both | United Arab Emirates | Psoriasis | -1.22 (-1.28 to -1.16) | < 0.001 |
| Adolescence | Prevalence | Female | United Arab Emirates | Psoriasis | -1.2 (-1.22 to -1.19) | < 0.001 |
| Adolescence | Prevalence | Male | United Arab Emirates | Psoriasis | -1.29 (-1.31 to -1.26) | < 0.001 |
| Adolescence | Prevalence | Both | United Arab Emirates | RHD | 0.02 (0.01 to 0.03) | < 0.001 |
| Adolescence | Prevalence | Female | United Arab Emirates | RHD | 0.02 (-0.01 to 0.05) | 0.136 |
| Adolescence | Prevalence | Male | United Arab Emirates | RHD | 0.01 (-0.01 to 0.03) | 0.214 |
| Adolescence | Prevalence | Both | United Arab Emirates | RA | 0.89 (0.55 to 1.23) | < 0.001 |
| Adolescence | Prevalence | Female | United Arab Emirates | RA | 0.77 (0.71 to 0.83) | < 0.001 |
| Adolescence | Prevalence | Male | United Arab Emirates | RA | 0.88 (0.81 to 0.95) | < 0.001 |
| Adolescence | Prevalence | Both | United Republic of Tanzania | AA | 0.02 (0.01 to 0.03) | < 0.001 |

| | | | | | | |
|---|---|---|---|---|---|---|
| Adolescence | Prevalence | Female | United Republic of Tanzania | AA | 0.06<br>(0.05 to 0.08) | < 0.001 |
| Adolescence | Prevalence | Male | United Republic of Tanzania | AA | -0.03<br>(-0.04 to -0.02) | < 0.001 |
| Adolescence | Prevalence | Both | United Republic of Tanzania | T1D | 0.07<br>(0.05 to 0.08) | < 0.001 |
| Adolescence | Prevalence | Female | United Republic of Tanzania | T1D | 0.06<br>(0.04 to 0.07) | < 0.001 |
| Adolescence | Prevalence | Male | United Republic of Tanzania | T1D | 0.07<br>(0.06 to 0.08) | < 0.001 |
| Adolescence | Prevalence | Both | United Republic of Tanzania | IBD | 0.22<br>(0.15 to 0.28) | < 0.001 |
| Adolescence | Prevalence | Female | United Republic of Tanzania | IBD | 0.19<br>(0.13 to 0.26) | < 0.001 |
| Adolescence | Prevalence | Male | United Republic of Tanzania | IBD | 0.24<br>(0.13 to 0.35) | < 0.001 |
| Adolescence | Prevalence | Both | United Republic of Tanzania | MS | 0.02<br>(-0.13 to 0.16) | 0.834 |
| Adolescence | Prevalence | Female | United Republic of Tanzania | MS | 0.01<br>(-0.18 to 0.19) | 0.950 |
| Adolescence | Prevalence | Male | United Republic of Tanzania | MS | 0<br>(-0.09 to 0.09) | 0.998 |
| Adolescence | Prevalence | Both | United Republic of Tanzania | Psoriasis | -0.4<br>(-0.43 to -0.37) | < 0.001 |
| Adolescence | Prevalence | Female | United Republic of Tanzania | Psoriasis | -0.41<br>(-0.42 to -0.4) | < 0.001 |
| Adolescence | Prevalence | Male | United Republic of Tanzania | Psoriasis | -0.41<br>(-0.47 to -0.35) | < 0.001 |
| Adolescence | Prevalence | Both | United Republic of Tanzania | RHD | 0.2<br>(0.2 to 0.21) | < 0.001 |
| Adolescence | Prevalence | Female | United Republic of Tanzania | RHD | 0.21<br>(0.18 to 0.24) | < 0.001 |
| Adolescence | Prevalence | Male | United Republic of Tanzania | RHD | 0.2 | < 0.001 |

| | | | | | | |
|---|---|---|---|---|---|---|
| | | | | | (0.18 to 0.21) | |
| Adolescence | Prevalence | Both | United Republic of Tanzania | RA | 0.26<br>(0.22 to 0.29) | < 0.001 |
| Adolescence | Prevalence | Female | United Republic of Tanzania | RA | 0.24<br>(0.22 to 0.27) | < 0.001 |
| Adolescence | Prevalence | Male | United Republic of Tanzania | RA | 0.36<br>(0.3 to 0.42) | < 0.001 |
| Adolescence | Prevalence | Both | United States of America | AA | -0.04<br>(-0.08 to -0.01) | 0.014 |
| Adolescence | Prevalence | Female | United States of America | AA | 0.05<br>(0.03 to 0.07) | < 0.001 |
| Adolescence | Prevalence | Male | United States of America | AA | -0.21<br>(-0.22 to -0.21) | < 0.001 |
| Adolescence | Prevalence | Both | United States of America | T1D | 0.01<br>(-0.18 to 0.19) | 0.954 |
| Adolescence | Prevalence | Female | United States of America | T1D | -0.26<br>(-0.42 to -0.1) | 0.001 |
| Adolescence | Prevalence | Male | United States of America | T1D | 0.16<br>(-0.05 to 0.38) | 0.136 |
| Adolescence | Prevalence | Both | United States of America | IBD | 0.39<br>(0.3 to 0.47) | < 0.001 |
| Adolescence | Prevalence | Female | United States of America | IBD | 0.42<br>(0.32 to 0.52) | < 0.001 |
| Adolescence | Prevalence | Male | United States of America | IBD | 0.34<br>(0.27 to 0.42) | < 0.001 |
| Adolescence | Prevalence | Both | United States of America | MS | -0.35<br>(-0.37 to -0.33) | < 0.001 |
| Adolescence | Prevalence | Female | United States of America | MS | -0.34<br>(-0.38 to -0.3) | < 0.001 |
| Adolescence | Prevalence | Male | United States of America | MS | -0.34<br>(-0.37 to -0.31) | < 0.001 |
| Adolescence | Prevalence | Both | United States of America | Psoriasis | -0.66<br>(-0.74 to -0.59) | < 0.001 |

| | | | | | | |
|---|---|---|---|---|---|---|
| Adolescence | Prevalence | Female | United States of America | Psoriasis | -0.68 (-0.74 to -0.61) | < 0.001 |
| Adolescence | Prevalence | Male | United States of America | Psoriasis | -0.65 (-0.74 to -0.57) | < 0.001 |
| Adolescence | Prevalence | Both | United States of America | RHD | -1.01 (-1.1 to -0.92) | < 0.001 |
| Adolescence | Prevalence | Female | United States of America | RHD | -1.04 (-1.21 to -0.87) | < 0.001 |
| Adolescence | Prevalence | Male | United States of America | RHD | -1 (-1.04 to -0.97) | < 0.001 |
| Adolescence | Prevalence | Both | United States of America | RA | 0.35 (0.29 to 0.42) | < 0.001 |
| Adolescence | Prevalence | Female | United States of America | RA | 0.35 (0.28 to 0.41) | < 0.001 |
| Adolescence | Prevalence | Male | United States of America | RA | 0.34 (0.28 to 0.4) | < 0.001 |
| Adolescence | Prevalence | Both | United States Virgin Islands | AA | -0.02 (-0.02 to -0.01) | < 0.001 |
| Adolescence | Prevalence | Female | United States Virgin Islands | AA | 0 (0 to 0) | < 0.001 |
| Adolescence | Prevalence | Male | United States Virgin Islands | AA | 0 (0 to 0) | 1.000 |
| Adolescence | Prevalence | Both | United States Virgin Islands | T1D | 0.91 (0.71 to 1.1) | < 0.001 |
| Adolescence | Prevalence | Female | United States Virgin Islands | T1D | 0.86 (0.58 to 1.15) | < 0.001 |
| Adolescence | Prevalence | Male | United States Virgin Islands | T1D | 0.79 (0.64 to 0.94) | < 0.001 |
| Adolescence | Prevalence | Both | United States Virgin Islands | IBD | 0.15 (0.12 to 0.19) | < 0.001 |
| Adolescence | Prevalence | Female | United States Virgin Islands | IBD | 0.18 (0.16 to 0.21) | < 0.001 |
| Adolescence | Prevalence | Male | United States Virgin Islands | IBD | 0.13 | < 0.001 |

| | | | | | | |
|---|---|---|---|---|---|---|
| | | | | | (0.08 to 0.17) | |
| Adolescence | Prevalence | Both | United States Virgin Islands | MS | 0.24 (0.16 to 0.31) | < 0.001 |
| Adolescence | Prevalence | Female | United States Virgin Islands | MS | 0.2 (0.17 to 0.23) | < 0.001 |
| Adolescence | Prevalence | Male | United States Virgin Islands | MS | 0.26 (0.14 to 0.37) | < 0.001 |
| Adolescence | Prevalence | Both | United States Virgin Islands | Psoriasis | -0.53 (-0.55 to -0.51) | < 0.001 |
| Adolescence | Prevalence | Female | United States Virgin Islands | Psoriasis | -0.52 (-0.56 to -0.49) | < 0.001 |
| Adolescence | Prevalence | Male | United States Virgin Islands | Psoriasis | -0.52 (-0.54 to -0.51) | < 0.001 |
| Adolescence | Prevalence | Both | United States Virgin Islands | RHD | -0.85 (-1.05 to -0.65) | < 0.001 |
| Adolescence | Prevalence | Female | United States Virgin Islands | RHD | -0.83 (-0.92 to -0.73) | < 0.001 |
| Adolescence | Prevalence | Male | United States Virgin Islands | RHD | -0.92 (-1.06 to -0.78) | < 0.001 |
| Adolescence | Prevalence | Both | United States Virgin Islands | RA | 0.68 (0.66 to 0.69) | < 0.001 |
| Adolescence | Prevalence | Female | United States Virgin Islands | RA | 0.7 (0.69 to 0.72) | < 0.001 |
| Adolescence | Prevalence | Male | United States Virgin Islands | RA | 0.7 (0.68 to 0.72) | < 0.001 |
| Adolescence | Prevalence | Both | Uruguay | AA | 0 (0 to 0) | < 0.001 |
| Adolescence | Prevalence | Female | Uruguay | AA | 0 (0 to 0) | < 0.001 |
| Adolescence | Prevalence | Male | Uruguay | AA | 0 (0 to 0) | < 0.001 |
| Adolescence | Prevalence | Both | Uruguay | T1D | 0.09 (0.07 to 0.12) | < 0.001 |

| | | | | | | |
|---|---|---|---|---|---|---|
| Adolescence | Prevalence | Female | Uruguay | T1D | 0.08 (0.06 to 0.1) | < 0.001 |
| Adolescence | Prevalence | Male | Uruguay | T1D | 0.1 (0.06 to 0.14) | < 0.001 |
| Adolescence | Prevalence | Both | Uruguay | IBD | 0.03 (0 to 0.07) | 0.083 |
| Adolescence | Prevalence | Female | Uruguay | IBD | 0.09 (0.06 to 0.12) | < 0.001 |
| Adolescence | Prevalence | Male | Uruguay | IBD | -0.02 (-0.1 to 0.05) | 0.536 |
| Adolescence | Prevalence | Both | Uruguay | MS | 0.14 (0.11 to 0.18) | < 0.001 |
| Adolescence | Prevalence | Female | Uruguay | MS | 0.14 (0.11 to 0.17) | < 0.001 |
| Adolescence | Prevalence | Male | Uruguay | MS | 0.18 (0.12 to 0.23) | < 0.001 |
| Adolescence | Prevalence | Both | Uruguay | Psoriasis | -0.42 (-0.43 to -0.41) | < 0.001 |
| Adolescence | Prevalence | Female | Uruguay | Psoriasis | -0.43 (-0.45 to -0.42) | < 0.001 |
| Adolescence | Prevalence | Male | Uruguay | Psoriasis | -0.4 (-0.41 to -0.39) | < 0.001 |
| Adolescence | Prevalence | Both | Uruguay | RHD | 0.2 (0.13 to 0.27) | < 0.001 |
| Adolescence | Prevalence | Female | Uruguay | RHD | 0.12 (0.08 to 0.16) | < 0.001 |
| Adolescence | Prevalence | Male | Uruguay | RHD | 0.27 (0.12 to 0.41) | < 0.001 |
| Adolescence | Prevalence | Both | Uruguay | RA | 0.64 (0.61 to 0.67) | < 0.001 |
| Adolescence | Prevalence | Female | Uruguay | RA | 0.64 (0.61 to 0.66) | < 0.001 |
| Adolescence | Prevalence | Male | Uruguay | RA | 0.69 | < 0.001 |

| | | | | | | |
|---|---|---|---|---|---|---|
| | | | | | (0.67 to 0.72) | |
| Adolescence | Prevalence | Both | Uzbekistan | AA | -0.02 (-0.02 to -0.02) | < 0.001 |
| Adolescence | Prevalence | Female | Uzbekistan | AA | 0 (0 to 0) | < 0.001 |
| Adolescence | Prevalence | Male | Uzbekistan | AA | 0 (0 to 0) | 1.000 |
| Adolescence | Prevalence | Both | Uzbekistan | T1D | 1.22 (1.18 to 1.26) | < 0.001 |
| Adolescence | Prevalence | Female | Uzbekistan | T1D | 1.18 (1.12 to 1.24) | < 0.001 |
| Adolescence | Prevalence | Male | Uzbekistan | T1D | 1.25 (1.19 to 1.32) | < 0.001 |
| Adolescence | Prevalence | Both | Uzbekistan | IBD | 0.18 (0.15 to 0.21) | < 0.001 |
| Adolescence | Prevalence | Female | Uzbekistan | IBD | 0.16 (0.11 to 0.2) | < 0.001 |
| Adolescence | Prevalence | Male | Uzbekistan | IBD | 0.21 (0.2 to 0.23) | < 0.001 |
| Adolescence | Prevalence | Both | Uzbekistan | MS | 0.13 (0.09 to 0.17) | < 0.001 |
| Adolescence | Prevalence | Female | Uzbekistan | MS | 0.2 (0.13 to 0.26) | < 0.001 |
| Adolescence | Prevalence | Male | Uzbekistan | MS | 0.16 (-0.07 to 0.39) | 0.163 |
| Adolescence | Prevalence | Both | Uzbekistan | Psoriasis | -0.94 (-0.98 to -0.9) | < 0.001 |
| Adolescence | Prevalence | Female | Uzbekistan | Psoriasis | -0.94 (-1.01 to -0.87) | < 0.001 |
| Adolescence | Prevalence | Male | Uzbekistan | Psoriasis | -0.91 (-0.93 to -0.9) | < 0.001 |
| Adolescence | Prevalence | Both | Uzbekistan | RHD | 0.25 (0.21 to 0.28) | < 0.001 |

| | | | | | | |
|---|---|---|---|---|---|---|
| Adolescence | Prevalence | Female | Uzbekistan | RHD | 0.27 (0.23 to 0.31) | < 0.001 |
| Adolescence | Prevalence | Male | Uzbekistan | RHD | 0.22 (0.2 to 0.24) | < 0.001 |
| Adolescence | Prevalence | Both | Uzbekistan | RA | 0.31 (0.18 to 0.45) | < 0.001 |
| Adolescence | Prevalence | Female | Uzbekistan | RA | 0.32 (0.18 to 0.46) | < 0.001 |
| Adolescence | Prevalence | Male | Uzbekistan | RA | 0.49 (0.41 to 0.58) | < 0.001 |
| Adolescence | Prevalence | Both | Vanuatu | AA | 0.01 (0.01 to 0.01) | < 0.001 |
| Adolescence | Prevalence | Female | Vanuatu | AA | 0 (0 to 0) | 0.008 |
| Adolescence | Prevalence | Male | Vanuatu | AA | 0 (0 to 0) | < 0.001 |
| Adolescence | Prevalence | Both | Vanuatu | T1D | 0.58 (0.56 to 0.6) | < 0.001 |
| Adolescence | Prevalence | Female | Vanuatu | T1D | 0.54 (0.5 to 0.58) | < 0.001 |
| Adolescence | Prevalence | Male | Vanuatu | T1D | 0.61 (0.58 to 0.64) | < 0.001 |
| Adolescence | Prevalence | Both | Vanuatu | IBD | 0.08 (0.02 to 0.14) | 0.012 |
| Adolescence | Prevalence | Female | Vanuatu | IBD | 0.03 (-0.05 to 0.12) | 0.423 |
| Adolescence | Prevalence | Male | Vanuatu | IBD | 0.15 (0.07 to 0.23) | < 0.001 |
| Adolescence | Prevalence | Both | Vanuatu | MS | 0.05 (0.05 to 0.05) | < 0.001 |
| Adolescence | Prevalence | Female | Vanuatu | MS | -0.02 (-0.14 to 0.1) | 0.717 |
| Adolescence | Prevalence | Male | Vanuatu | MS | 0.11 | 0.196 |

| | | | | | | |
|---|---|---|---|---|---|---|
| | | | | | (-0.06 to 0.28) | |
| Adolescence | Prevalence | Both | Vanuatu | Psoriasis | -0.42<br>(-0.43 to -0.4) | < 0.001 |
| Adolescence | Prevalence | Female | Vanuatu | Psoriasis | -0.4<br>(-0.42 to -0.39) | < 0.001 |
| Adolescence | Prevalence | Male | Vanuatu | Psoriasis | -0.44<br>(-0.47 to -0.42) | < 0.001 |
| Adolescence | Prevalence | Both | Vanuatu | RHD | 0.04<br>(0.02 to 0.07) | < 0.001 |
| Adolescence | Prevalence | Female | Vanuatu | RHD | 0.04<br>(0.01 to 0.06) | 0.011 |
| Adolescence | Prevalence | Male | Vanuatu | RHD | 0.04<br>(0 to 0.08) | 0.042 |
| Adolescence | Prevalence | Both | Vanuatu | RA | 0.31<br>(0.29 to 0.33) | < 0.001 |
| Adolescence | Prevalence | Female | Vanuatu | RA | 0.34<br>(0.33 to 0.36) | < 0.001 |
| Adolescence | Prevalence | Male | Vanuatu | RA | 0.24<br>(0.22 to 0.26) | < 0.001 |
| Adolescence | Prevalence | Both | Yemen | AA | 0.02<br>(0.02 to 0.03) | < 0.001 |
| Adolescence | Prevalence | Female | Yemen | AA | 0<br>(0 to 0) | 0.001 |
| Adolescence | Prevalence | Male | Yemen | AA | 0<br>(0 to 0) | 0.011 |
| Adolescence | Prevalence | Both | Yemen | T1D | 2.18<br>(2.16 to 2.2) | < 0.001 |
| Adolescence | Prevalence | Female | Yemen | T1D | 2.16<br>(2.14 to 2.19) | < 0.001 |
| Adolescence | Prevalence | Male | Yemen | T1D | 2.2<br>(2.17 to 2.24) | < 0.001 |
| Adolescence | Prevalence | Both | Yemen | IBD | 0.49<br>(0.44 to 0.53) | < 0.001 |

| | | | | | | |
|---|---|---|---|---|---|---|
| Adolescence | Prevalence | Female | Yemen | IBD | 0.45<br>(0.41 to 0.49) | < 0.001 |
| Adolescence | Prevalence | Male | Yemen | IBD | 0.52<br>(0.45 to 0.58) | < 0.001 |
| Adolescence | Prevalence | Both | Yemen | MS | 0.41<br>(0.35 to 0.46) | < 0.001 |
| Adolescence | Prevalence | Female | Yemen | MS | 0.33<br>(0.3 to 0.35) | < 0.001 |
| Adolescence | Prevalence | Male | Yemen | MS | 0.47<br>(0.41 to 0.54) | < 0.001 |
| Adolescence | Prevalence | Both | Yemen | Psoriasis | -1.26<br>(-1.28 to -1.23) | < 0.001 |
| Adolescence | Prevalence | Female | Yemen | Psoriasis | -1.24<br>(-1.26 to -1.21) | < 0.001 |
| Adolescence | Prevalence | Male | Yemen | Psoriasis | -1.31<br>(-1.34 to -1.28) | < 0.001 |
| Adolescence | Prevalence | Both | Yemen | RHD | 0.18<br>(-1.17 to 1.55) | 0.793 |
| Adolescence | Prevalence | Female | Yemen | RHD | 0.2<br>(-1.09 to 1.5) | 0.765 |
| Adolescence | Prevalence | Male | Yemen | RHD | 0.15<br>(-1.26 to 1.59) | 0.831 |
| Adolescence | Prevalence | Both | Yemen | RA | 0.45<br>(0.41 to 0.48) | < 0.001 |
| Adolescence | Prevalence | Female | Yemen | RA | 0.39<br>(0.37 to 0.42) | < 0.001 |
| Adolescence | Prevalence | Male | Yemen | RA | 0.46<br>(0.36 to 0.56) | < 0.001 |
| Adolescence | Prevalence | Both | Zambia | AA | -0.01<br>(-0.01 to 0) | < 0.001 |
| Adolescence | Prevalence | Female | Zambia | AA | 0<br>(0 to 0) | < 0.001 |
| Adolescence | Prevalence | Male | Zambia | AA | 0 | < 0.001 |

| | | | | | | |
|---|---|---|---|---|---|---|
| | | | | | (0 to 0) | |
| Adolescence | Prevalence | Both | Zambia | T1D | 0.07 (0.04 to 0.1) | < 0.001 |
| Adolescence | Prevalence | Female | Zambia | T1D | 0.07 (0.03 to 0.12) | 0.001 |
| Adolescence | Prevalence | Male | Zambia | T1D | 0.08 (0.04 to 0.12) | < 0.001 |
| Adolescence | Prevalence | Both | Zambia | IBD | 0.61 (0.55 to 0.67) | < 0.001 |
| Adolescence | Prevalence | Female | Zambia | IBD | 0.6 (0.54 to 0.65) | < 0.001 |
| Adolescence | Prevalence | Male | Zambia | IBD | 0.65 (0.59 to 0.72) | < 0.001 |
| Adolescence | Prevalence | Both | Zambia | MS | -0.02 (-0.11 to 0.06) | 0.608 |
| Adolescence | Prevalence | Female | Zambia | MS | -0.03 (-0.16 to 0.09) | 0.592 |
| Adolescence | Prevalence | Male | Zambia | MS | 0.08 (-0.08 to 0.25) | 0.324 |
| Adolescence | Prevalence | Both | Zambia | Psoriasis | -0.5 (-0.57 to -0.42) | < 0.001 |
| Adolescence | Prevalence | Female | Zambia | Psoriasis | -0.51 (-0.56 to -0.46) | < 0.001 |
| Adolescence | Prevalence | Male | Zambia | Psoriasis | -0.45 (-0.53 to -0.37) | < 0.001 |
| Adolescence | Prevalence | Both | Zambia | RHD | -0.19 (-0.22 to -0.15) | < 0.001 |
| Adolescence | Prevalence | Female | Zambia | RHD | -0.23 (-0.27 to -0.18) | < 0.001 |
| Adolescence | Prevalence | Male | Zambia | RHD | -0.12 (-0.15 to -0.08) | < 0.001 |
| Adolescence | Prevalence | Both | Zambia | RA | 0.23 (0.12 to 0.34) | < 0.001 |

| | | | | | | |
|---|---|---|---|---|---|---|
| Adolescence | Prevalence | Female | Zambia | RA | 0.21<br>(0.1 to 0.32) | < 0.001 |
| Adolescence | Prevalence | Male | Zambia | RA | 0.29<br>(0.22 to 0.37) | < 0.001 |
| Adolescence | Prevalence | Both | Zimbabwe | AA | 0<br>(0 to 0) | 0.008 |
| Adolescence | Prevalence | Female | Zimbabwe | AA | 0<br>(0 to 0) | < 0.001 |
| Adolescence | Prevalence | Male | Zimbabwe | AA | 0<br>(0 to 0) | < 0.001 |
| Adolescence | Prevalence | Both | Zimbabwe | T1D | 0.2<br>(0.18 to 0.21) | < 0.001 |
| Adolescence | Prevalence | Female | Zimbabwe | T1D | 0.21<br>(0.19 to 0.23) | < 0.001 |
| Adolescence | Prevalence | Male | Zimbabwe | T1D | 0.19<br>(0.14 to 0.25) | < 0.001 |
| Adolescence | Prevalence | Both | Zimbabwe | IBD | 0.02<br>(-0.03 to 0.08) | 0.459 |
| Adolescence | Prevalence | Female | Zimbabwe | IBD | -0.02<br>(-0.06 to 0.03) | 0.493 |
| Adolescence | Prevalence | Male | Zimbabwe | IBD | 0.04<br>(-0.03 to 0.12) | 0.261 |
| Adolescence | Prevalence | Both | Zimbabwe | MS | 0.11<br>(0.01 to 0.21) | 0.034 |
| Adolescence | Prevalence | Female | Zimbabwe | MS | 0.13<br>(0.08 to 0.19) | < 0.001 |
| Adolescence | Prevalence | Male | Zimbabwe | MS | 0.04<br>(-0.05 to 0.12) | 0.387 |
| Adolescence | Prevalence | Both | Zimbabwe | Psoriasis | -0.2<br>(-0.25 to -0.15) | < 0.001 |
| Adolescence | Prevalence | Female | Zimbabwe | Psoriasis | -0.23<br>(-0.28 to -0.18) | < 0.001 |
| Adolescence | Prevalence | Male | Zimbabwe | Psoriasis | -0.17 | < 0.001 |

| | | | | | | |
|---|---|---|---|---|---|---|
| | | | | | (-0.24 to -0.11) | |
| Adolescence | Prevalence | Both | Zimbabwe | RHD | 0.12 (0.11 to 0.13) | < 0.001 |
| Adolescence | Prevalence | Female | Zimbabwe | RHD | 0.1 (0.07 to 0.14) | < 0.001 |
| Adolescence | Prevalence | Male | Zimbabwe | RHD | 0.15 (0.12 to 0.18) | < 0.001 |
| Adolescence | Prevalence | Both | Zimbabwe | RA | 0.07 (0.02 to 0.11) | 0.008 |
| Adolescence | Prevalence | Female | Zimbabwe | RA | 0.01 (-0.05 to 0.08) | 0.749 |
| Adolescence | Prevalence | Male | Zimbabwe | RA | 0.18 (0.12 to 0.25) | < 0.001 |
| Young and middle-age | Prevalence | Both | American Samoa | MS | 0.1 (0.04 to 0.15) | < 0.001 |
| Young and middle-age | Prevalence | Female | American Samoa | MS | 0.06 (0.03 to 0.09) | < 0.001 |
| Young and middle-age | Prevalence | Male | American Samoa | MS | 0.11 (0.09 to 0.13) | < 0.001 |
| Young and middle-age | Prevalence | Both | American Samoa | Psoriasis | -0.43 (-0.53 to -0.33) | < 0.001 |
| Young and middle-age | Prevalence | Female | American Samoa | Psoriasis | -0.4 (-0.51 to -0.3) | < 0.001 |
| Young and middle-age | Prevalence | Male | American Samoa | Psoriasis | -0.43 (-0.56 to -0.3) | < 0.001 |
| Young and middle-age | Prevalence | Both | American Samoa | RHD | 0.09 (0.07 to 0.11) | < 0.001 |
| Young and middle-age | Prevalence | Female | American Samoa | RHD | 0.09 (0.08 to 0.11) | < 0.001 |
| Young and middle-age | Prevalence | Male | American Samoa | RHD | 0.08 (0.04 to 0.12) | < 0.001 |
| Young and middle-age | Prevalence | Both | American Samoa | RA | 0.26 (0.23 to 0.29) | < 0.001 |

| | | | | | | |
|---|---|---|---|---|---|---|
| Young and middle-age | Prevalence | Female | American Samoa | RA | 0.24 (0.2 to 0.28) | < 0.001 |
| Young and middle-age | Prevalence | Male | American Samoa | RA | 0.15 (0.13 to 0.18) | < 0.001 |
| Young and middle-age | Prevalence | Both | Antigua and Barbuda | MS | 0.94 (0.88 to 1.01) | < 0.001 |
| Young and middle-age | Prevalence | Female | Antigua and Barbuda | MS | 1.07 (0.95 to 1.2) | < 0.001 |
| Young and middle-age | Prevalence | Male | Antigua and Barbuda | MS | 0.31 (0.29 to 0.32) | < 0.001 |
| Young and middle-age | Prevalence | Both | Antigua and Barbuda | Psoriasis | -0.38 (-0.39 to -0.38) | < 0.001 |
| Young and middle-age | Prevalence | Female | Antigua and Barbuda | Psoriasis | -0.38 (-0.39 to -0.37) | < 0.001 |
| Young and middle-age | Prevalence | Male | Antigua and Barbuda | Psoriasis | -0.39 (-0.4 to -0.37) | < 0.001 |
| Young and middle-age | Prevalence | Both | Antigua and Barbuda | RHD | -0.01 (-0.03 to 0.01) | 0.278 |
| Young and middle-age | Prevalence | Female | Antigua and Barbuda | RHD | 0.01 (-0.02 to 0.04) | 0.505 |
| Young and middle-age | Prevalence | Male | Antigua and Barbuda | RHD | -0.02 (-0.04 to -0.01) | 0.011 |
| Young and middle-age | Prevalence | Both | Antigua and Barbuda | RA | 0.8 (0.77 to 0.83) | < 0.001 |
| Young and middle-age | Prevalence | Female | Antigua and Barbuda | RA | 0.8 (0.78 to 0.83) | < 0.001 |
| Young and middle-age | Prevalence | Male | Antigua and Barbuda | RA | 0.81 (0.77 to 0.84) | < 0.001 |
| Young and middle-age | Prevalence | Both | Australia | MS | 1.37 (1.21 to 1.53) | < 0.001 |
| Young and middle-age | Prevalence | Female | Australia | MS | 1.41 (1.26 to 1.56) | < 0.001 |
| Young and middle-age | Prevalence | Male | Australia | MS | 1.17 | < 0.001 |

| | | | | | | |
|---|---|---|---|---|---|---|
| | | | | | (0.99 to 1.35) | |
| Young and middle-age | Prevalence | Both | Australia | Psoriasis | -0.46<br>(-0.53 to -0.38) | < 0.001 |
| Young and middle-age | Prevalence | Female | Australia | Psoriasis | -0.46<br>(-0.53 to -0.38) | < 0.001 |
| Young and middle-age | Prevalence | Male | Australia | Psoriasis | -0.47<br>(-0.53 to -0.4) | < 0.001 |
| Young and middle-age | Prevalence | Both | Australia | RHD | -0.11<br>(-0.2 to -0.02) | 0.016 |
| Young and middle-age | Prevalence | Female | Australia | RHD | -0.16<br>(-0.27 to -0.06) | 0.002 |
| Young and middle-age | Prevalence | Male | Australia | RHD | -0.06<br>(-0.13 to 0.01) | 0.098 |
| Young and middle-age | Prevalence | Both | Australia | RA | 0.68<br>(0.64 to 0.72) | < 0.001 |
| Young and middle-age | Prevalence | Female | Australia | RA | 0.63<br>(0.59 to 0.67) | < 0.001 |
| Young and middle-age | Prevalence | Male | Australia | RA | 0.7<br>(0.68 to 0.72) | < 0.001 |
| Young and middle-age | Prevalence | Both | Barbados | MS | 0.96<br>(0.86 to 1.05) | < 0.001 |
| Young and middle-age | Prevalence | Female | Barbados | MS | 1.05<br>(0.95 to 1.16) | < 0.001 |
| Young and middle-age | Prevalence | Male | Barbados | MS | 0.56<br>(0.4 to 0.72) | < 0.001 |
| Young and middle-age | Prevalence | Both | Barbados | Psoriasis | -0.23<br>(-0.25 to -0.21) | < 0.001 |
| Young and middle-age | Prevalence | Female | Barbados | Psoriasis | -0.23<br>(-0.24 to -0.21) | < 0.001 |
| Young and middle-age | Prevalence | Male | Barbados | Psoriasis | -0.23<br>(-0.25 to -0.21) | < 0.001 |
| Young and middle-age | Prevalence | Both | Barbados | RHD | 0.03<br>(0.01 to 0.04) | 0.006 |

| | | | | | | |
|---|---|---|---|---|---|---|
| Young and middle-age | Prevalence | Female | Barbados | RHD | 0.05<br>(0.04 to 0.06) | < 0.001 |
| Young and middle-age | Prevalence | Male | Barbados | RHD | -0.02<br>(-0.07 to 0.02) | 0.257 |
| Young and middle-age | Prevalence | Both | Barbados | RA | 0.68<br>(0.64 to 0.71) | < 0.001 |
| Young and middle-age | Prevalence | Female | Barbados | RA | 0.67<br>(0.63 to 0.71) | < 0.001 |
| Young and middle-age | Prevalence | Male | Barbados | RA | 0.74<br>(0.69 to 0.78) | < 0.001 |
| Young and middle-age | Prevalence | Both | Belize | MS | 0.57<br>(0.52 to 0.61) | < 0.001 |
| Young and middle-age | Prevalence | Female | Belize | MS | 0.52<br>(0.44 to 0.59) | < 0.001 |
| Young and middle-age | Prevalence | Male | Belize | MS | 0.43<br>(0.4 to 0.46) | < 0.001 |
| Young and middle-age | Prevalence | Both | Belize | Psoriasis | -0.64<br>(-0.65 to -0.63) | < 0.001 |
| Young and middle-age | Prevalence | Female | Belize | Psoriasis | -0.64<br>(-0.66 to -0.63) | < 0.001 |
| Young and middle-age | Prevalence | Male | Belize | Psoriasis | -0.64<br>(-0.65 to -0.63) | < 0.001 |
| Young and middle-age | Prevalence | Both | Belize | RHD | 0.04<br>(0.03 to 0.05) | < 0.001 |
| Young and middle-age | Prevalence | Female | Belize | RHD | 0.05<br>(0.04 to 0.06) | < 0.001 |
| Young and middle-age | Prevalence | Male | Belize | RHD | -0.02<br>(-0.04 to 0) | 0.058 |
| Young and middle-age | Prevalence | Both | Belize | RA | 0.88<br>(0.84 to 0.91) | < 0.001 |
| Young and middle-age | Prevalence | Female | Belize | RA | 0.76<br>(0.72 to 0.8) | < 0.001 |
| Young and middle-age | Prevalence | Male | Belize | RA | 0.89 | < 0.001 |

| | | | | | | |
|---|---|---|---|---|---|---|
| | | | | | (0.83 to 0.96) | |
| Young and middle-age | Prevalence | Both | Bermuda | MS | 0.37<br>(0.34 to 0.41) | < 0.001 |
| Young and middle-age | Prevalence | Female | Bermuda | MS | 0.4<br>(0.36 to 0.45) | < 0.001 |
| Young and middle-age | Prevalence | Male | Bermuda | MS | 0.32<br>(0.28 to 0.35) | < 0.001 |
| Young and middle-age | Prevalence | Both | Bermuda | Psoriasis | -0.37<br>(-0.39 to -0.35) | < 0.001 |
| Young and middle-age | Prevalence | Female | Bermuda | Psoriasis | -0.37<br>(-0.39 to -0.35) | < 0.001 |
| Young and middle-age | Prevalence | Male | Bermuda | Psoriasis | -0.37<br>(-0.39 to -0.35) | < 0.001 |
| Young and middle-age | Prevalence | Both | Bermuda | RHD | -1.52<br>(-1.63 to -1.42) | < 0.001 |
| Young and middle-age | Prevalence | Female | Bermuda | RHD | -1.47<br>(-1.56 to -1.38) | < 0.001 |
| Young and middle-age | Prevalence | Male | Bermuda | RHD | -1.52<br>(-1.71 to -1.33) | < 0.001 |
| Young and middle-age | Prevalence | Both | Bermuda | RA | 0.87<br>(0.83 to 0.91) | < 0.001 |
| Young and middle-age | Prevalence | Female | Bermuda | RA | 0.89<br>(0.86 to 0.92) | < 0.001 |
| Young and middle-age | Prevalence | Male | Bermuda | RA | 0.9<br>(0.84 to 0.96) | < 0.001 |
| Young and middle-age | Prevalence | Both | Bosnia and Herzegovina | MS | 0.01<br>(-0.03 to 0.05) | 0.657 |
| Young and middle-age | Prevalence | Female | Bosnia and Herzegovina | MS | 0.2<br>(0.17 to 0.24) | < 0.001 |
| Young and middle-age | Prevalence | Male | Bosnia and Herzegovina | MS | -0.32<br>(-0.39 to -0.25) | < 0.001 |
| Young and middle-age | Prevalence | Both | Bosnia and Herzegovina | Psoriasis | -1.08<br>(-1.12 to -1.03) | < 0.001 |

| | | | | | | |
|---|---|---|---|---|---|---|
| Young and middle-age | Prevalence | Female | Bosnia and Herzegovina | Psoriasis | -1.08<br>(-1.15 to -1.01) | < 0.001 |
| Young and middle-age | Prevalence | Male | Bosnia and Herzegovina | Psoriasis | -1.06<br>(-1.09 to -1.03) | < 0.001 |
| Young and middle-age | Prevalence | Both | Bosnia and Herzegovina | RHD | 0.79<br>(0.57 to 1) | < 0.001 |
| Young and middle-age | Prevalence | Female | Bosnia and Herzegovina | RHD | 0.69<br>(0.48 to 0.89) | < 0.001 |
| Young and middle-age | Prevalence | Male | Bosnia and Herzegovina | RHD | 0.92<br>(0.73 to 1.11) | < 0.001 |
| Young and middle-age | Prevalence | Both | Bosnia and Herzegovina | RA | 0.79<br>(0.72 to 0.85) | < 0.001 |
| Young and middle-age | Prevalence | Female | Bosnia and Herzegovina | RA | 0.75<br>(0.7 to 0.81) | < 0.001 |
| Young and middle-age | Prevalence | Male | Bosnia and Herzegovina | RA | 0.79<br>(0.72 to 0.86) | < 0.001 |
| Young and middle-age | Prevalence | Both | Brunei Darussalam | MS | 0.17<br>(0.1 to 0.24) | < 0.001 |
| Young and middle-age | Prevalence | Female | Brunei Darussalam | MS | 0.1<br>(0.06 to 0.14) | < 0.001 |
| Young and middle-age | Prevalence | Male | Brunei Darussalam | MS | 0.13<br>(0.07 to 0.19) | < 0.001 |
| Young and middle-age | Prevalence | Both | Brunei Darussalam | Psoriasis | -0.3<br>(-0.31 to -0.29) | < 0.001 |
| Young and middle-age | Prevalence | Female | Brunei Darussalam | Psoriasis | -0.37<br>(-0.38 to -0.35) | < 0.001 |
| Young and middle-age | Prevalence | Male | Brunei Darussalam | Psoriasis | -0.23<br>(-0.25 to -0.2) | < 0.001 |
| Young and middle-age | Prevalence | Both | Brunei Darussalam | RHD | -1.38<br>(-1.59 to -1.18) | < 0.001 |
| Young and middle-age | Prevalence | Female | Brunei Darussalam | RHD | -1.7<br>(-1.96 to -1.44) | < 0.001 |
| Young and middle-age | Prevalence | Male | Brunei Darussalam | RHD | -1.06 | < 0.001 |

| | | | | | | |
|---|---|---|---|---|---|---|
| | | | | | (-1.26 to -0.86) | |
| Young and middle-age | Prevalence | Both | Brunei Darussalam | RA | 0.55<br>(0.5 to 0.61) | < 0.001 |
| Young and middle-age | Prevalence | Female | Brunei Darussalam | RA | 0.45<br>(0.42 to 0.47) | < 0.001 |
| Young and middle-age | Prevalence | Male | Brunei Darussalam | RA | 0.55<br>(0.54 to 0.56) | < 0.001 |
| Young and middle-age | Prevalence | Both | Burkina Faso | MS | 0.12<br>(0.09 to 0.14) | < 0.001 |
| Young and middle-age | Prevalence | Female | Burkina Faso | MS | 0.15<br>(0.11 to 0.19) | < 0.001 |
| Young and middle-age | Prevalence | Male | Burkina Faso | MS | 0.24<br>(0.19 to 0.29) | < 0.001 |
| Young and middle-age | Prevalence | Both | Burkina Faso | Psoriasis | -0.55<br>(-0.78 to -0.31) | < 0.001 |
| Young and middle-age | Prevalence | Female | Burkina Faso | Psoriasis | -0.61<br>(-0.79 to -0.44) | < 0.001 |
| Young and middle-age | Prevalence | Male | Burkina Faso | Psoriasis | -0.45<br>(-0.64 to -0.26) | < 0.001 |
| Young and middle-age | Prevalence | Both | Burkina Faso | RHD | 0.04<br>(0.04 to 0.05) | < 0.001 |
| Young and middle-age | Prevalence | Female | Burkina Faso | RHD | 0.06<br>(0.05 to 0.07) | < 0.001 |
| Young and middle-age | Prevalence | Male | Burkina Faso | RHD | 0.05<br>(0.03 to 0.06) | < 0.001 |
| Young and middle-age | Prevalence | Both | Burkina Faso | RA | 0.5<br>(0.48 to 0.51) | < 0.001 |
| Young and middle-age | Prevalence | Female | Burkina Faso | RA | 0.54<br>(0.52 to 0.56) | < 0.001 |
| Young and middle-age | Prevalence | Male | Burkina Faso | RA | 0.54<br>(0.53 to 0.55) | < 0.001 |
| Young and middle-age | Prevalence | Both | Canada | MS | 0.64<br>(0.58 to 0.7) | < 0.001 |

| | | | | | | |
|---|---|---|---|---|---|---|
| Young and middle-age | Prevalence | Female | Canada | MS | 0.63<br>(0.56 to 0.69) | < 0.001 |
| Young and middle-age | Prevalence | Male | Canada | MS | 0.63<br>(0.58 to 0.67) | < 0.001 |
| Young and middle-age | Prevalence | Both | Canada | Psoriasis | -0.43<br>(-0.43 to -0.42) | < 0.001 |
| Young and middle-age | Prevalence | Female | Canada | Psoriasis | -0.42<br>(-0.43 to -0.42) | < 0.001 |
| Young and middle-age | Prevalence | Male | Canada | Psoriasis | -0.42<br>(-0.43 to -0.42) | < 0.001 |
| Young and middle-age | Prevalence | Both | Canada | RHD | -0.27<br>(-0.31 to -0.23) | < 0.001 |
| Young and middle-age | Prevalence | Female | Canada | RHD | -0.38<br>(-0.43 to -0.33) | < 0.001 |
| Young and middle-age | Prevalence | Male | Canada | RHD | -0.14<br>(-0.19 to -0.1) | < 0.001 |
| Young and middle-age | Prevalence | Both | Canada | RA | 0.85<br>(0.83 to 0.88) | < 0.001 |
| Young and middle-age | Prevalence | Female | Canada | RA | 0.85<br>(0.83 to 0.88) | < 0.001 |
| Young and middle-age | Prevalence | Male | Canada | RA | 0.78<br>(0.77 to 0.8) | < 0.001 |
| Young and middle-age | Prevalence | Both | Central African Republic | MS | 0.09<br>(0.07 to 0.12) | < 0.001 |
| Young and middle-age | Prevalence | Female | Central African Republic | MS | 0.11<br>(0.08 to 0.14) | < 0.001 |
| Young and middle-age | Prevalence | Male | Central African Republic | MS | 0.12<br>(0.09 to 0.14) | < 0.001 |
| Young and middle-age | Prevalence | Both | Central African Republic | Psoriasis | -0.47<br>(-0.48 to -0.46) | < 0.001 |
| Young and middle-age | Prevalence | Female | Central African Republic | Psoriasis | -0.47<br>(-0.49 to -0.46) | < 0.001 |
| Young and middle-age | Prevalence | Male | Central African Republic | Psoriasis | -0.46 | < 0.001 |

| | | | | | | |
|---|---|---|---|---|---|---|
| | | | | | (-0.47 to -0.45) | |
| Young and middle-age | Prevalence | Both | Central African Republic | RHD | 0.11<br>(0.09 to 0.12) | < 0.001 |
| Young and middle-age | Prevalence | Female | Central African Republic | RHD | 0.16<br>(0.13 to 0.2) | < 0.001 |
| Young and middle-age | Prevalence | Male | Central African Republic | RHD | 0.05<br>(0.04 to 0.06) | < 0.001 |
| Young and middle-age | Prevalence | Both | Central African Republic | RA | -0.02<br>(-0.07 to 0.03) | 0.427 |
| Young and middle-age | Prevalence | Female | Central African Republic | RA | 0<br>(-0.04 to 0.05) | 0.933 |
| Young and middle-age | Prevalence | Male | Central African Republic | RA | -0.02<br>(-0.17 to 0.12) | 0.744 |
| Young and middle-age | Prevalence | Both | Cook Islands | MS | 0.14<br>(0.1 to 0.18) | < 0.001 |
| Young and middle-age | Prevalence | Female | Cook Islands | MS | 0.04<br>(0.02 to 0.06) | 0.001 |
| Young and middle-age | Prevalence | Male | Cook Islands | MS | 0.09<br>(0.04 to 0.13) | < 0.001 |
| Young and middle-age | Prevalence | Both | Cook Islands | Psoriasis | -0.51<br>(-0.54 to -0.48) | < 0.001 |
| Young and middle-age | Prevalence | Female | Cook Islands | Psoriasis | -0.49<br>(-0.5 to -0.48) | < 0.001 |
| Young and middle-age | Prevalence | Male | Cook Islands | Psoriasis | -0.53<br>(-0.58 to -0.48) | < 0.001 |
| Young and middle-age | Prevalence | Both | Cook Islands | RHD | 0.14<br>(0.06 to 0.22) | 0.001 |
| Young and middle-age | Prevalence | Female | Cook Islands | RHD | 0.13<br>(0.04 to 0.22) | 0.006 |
| Young and middle-age | Prevalence | Male | Cook Islands | RHD | 0.05<br>(-0.02 to 0.11) | 0.149 |
| Young and middle-age | Prevalence | Both | Cook Islands | RA | 0.67<br>(0.64 to 0.7) | < 0.001 |

| | | | | | | |
|---|---|---|---|---|---|---|
| Young and middle-age | Prevalence | Female | Cook Islands | RA | 0.56 (0.53 to 0.59) | < 0.001 |
| Young and middle-age | Prevalence | Male | Cook Islands | RA | 0.51 (0.48 to 0.54) | < 0.001 |
| Young and middle-age | Prevalence | Both | Democratic People's Republic of Korea | MS | 0.02 (-0.01 to 0.05) | 0.210 |
| Young and middle-age | Prevalence | Female | Democratic People's Republic of Korea | MS | 0.08 (0.04 to 0.13) | < 0.001 |
| Young and middle-age | Prevalence | Male | Democratic People's Republic of Korea | MS | 0.16 (0.11 to 0.21) | < 0.001 |
| Young and middle-age | Prevalence | Both | Democratic People's Republic of Korea | Psoriasis | -0.73 (-0.74 to -0.71) | < 0.001 |
| Young and middle-age | Prevalence | Female | Democratic People's Republic of Korea | Psoriasis | -0.73 (-0.74 to -0.72) | < 0.001 |
| Young and middle-age | Prevalence | Male | Democratic People's Republic of Korea | Psoriasis | -0.73 (-0.75 to -0.7) | < 0.001 |
| Young and middle-age | Prevalence | Both | Democratic People's Republic of Korea | RHD | 0.04 (0.03 to 0.05) | < 0.001 |
| Young and middle-age | Prevalence | Female | Democratic People's Republic of Korea | RHD | 0.09 (0.07 to 0.12) | < 0.001 |
| Young and middle-age | Prevalence | Male | Democratic People's Republic of Korea | RHD | 0.02 (-0.02 to 0.07) | 0.319 |
| Young and middle-age | Prevalence | Both | Democratic People's Republic of Korea | RA | -0.29 (-0.36 to -0.22) | < 0.001 |
| Young and middle-age | Prevalence | Female | Democratic People's Republic of Korea | RA | -0.22 (-0.3 to -0.14) | < 0.001 |
| Young and middle-age | Prevalence | Male | Democratic People's Republic of Korea | RA | 0.12 (0.03 to 0.22) | 0.013 |
| Young and middle-age | Prevalence | Both | Democratic Republic of the Congo | MS | 0.09 (0.07 to 0.11) | < 0.001 |
| Young and middle-age | Prevalence | Female | Democratic Republic of the Congo | MS | 0.12 (0.1 to 0.13) | < 0.001 |
| Young and middle-age | Prevalence | Male | Democratic Republic of the Congo | MS | 0.19 | < 0.001 |

| | | | | | | |
|---|---|---|---|---|---|---|
| | | | | | (0.13 to 0.24) | |
| Young and middle-age | Prevalence | Both | Democratic Republic of the Congo | Psoriasis | -0.62 (-0.69 to -0.54) | < 0.001 |
| Young and middle-age | Prevalence | Female | Democratic Republic of the Congo | Psoriasis | -0.61 (-0.69 to -0.54) | < 0.001 |
| Young and middle-age | Prevalence | Male | Democratic Republic of the Congo | Psoriasis | -0.61 (-0.67 to -0.55) | < 0.001 |
| Young and middle-age | Prevalence | Both | Democratic Republic of the Congo | RHD | 0.03 (0.02 to 0.05) | < 0.001 |
| Young and middle-age | Prevalence | Female | Democratic Republic of the Congo | RHD | 0.07 (0.04 to 0.1) | < 0.001 |
| Young and middle-age | Prevalence | Male | Democratic Republic of the Congo | RHD | 0 (-0.02 to 0.01) | 0.528 |
| Young and middle-age | Prevalence | Both | Democratic Republic of the Congo | RA | 0.27 (0.12 to 0.42) | < 0.001 |
| Young and middle-age | Prevalence | Female | Democratic Republic of the Congo | RA | 0.33 (0.22 to 0.43) | < 0.001 |
| Young and middle-age | Prevalence | Male | Democratic Republic of the Congo | RA | 0.3 (0.2 to 0.39) | < 0.001 |
| Young and middle-age | Prevalence | Both | Dominican Republic | MS | 0.48 (0.45 to 0.51) | < 0.001 |
| Young and middle-age | Prevalence | Female | Dominican Republic | MS | 0.55 (0.5 to 0.61) | < 0.001 |
| Young and middle-age | Prevalence | Male | Dominican Republic | MS | 0.48 (0.45 to 0.51) | < 0.001 |
| Young and middle-age | Prevalence | Both | Dominican Republic | Psoriasis | -0.55 (-0.58 to -0.51) | < 0.001 |
| Young and middle-age | Prevalence | Female | Dominican Republic | Psoriasis | -0.54 (-0.57 to -0.51) | < 0.001 |
| Young and middle-age | Prevalence | Male | Dominican Republic | Psoriasis | -0.54 (-0.58 to -0.51) | < 0.001 |
| Young and middle-age | Prevalence | Both | Dominican Republic | RHD | -0.02 (-0.03 to -0.01) | < 0.001 |

| | | | | | | |
|---|---|---|---|---|---|---|
| Young and middle-age | Prevalence | Female | Dominican Republic | RHD | 0.01<br>(0 to 0.02) | 0.002 |
| Young and middle-age | Prevalence | Male | Dominican Republic | RHD | -0.02<br>(-0.04 to -0.01) | < 0.001 |
| Young and middle-age | Prevalence | Both | Dominican Republic | RA | 0.95<br>(0.92 to 0.99) | < 0.001 |
| Young and middle-age | Prevalence | Female | Dominican Republic | RA | 1.05<br>(0.99 to 1.11) | < 0.001 |
| Young and middle-age | Prevalence | Male | Dominican Republic | RA | 0.97<br>(0.95 to 0.98) | < 0.001 |
| Young and middle-age | Prevalence | Both | Georgia | MS | 0.82<br>(0.62 to 1.03) | < 0.001 |
| Young and middle-age | Prevalence | Female | Georgia | MS | 1.07<br>(0.92 to 1.21) | < 0.001 |
| Young and middle-age | Prevalence | Male | Georgia | MS | 0.59<br>(0.37 to 0.8) | < 0.001 |
| Young and middle-age | Prevalence | Both | Georgia | Psoriasis | -0.5<br>(-0.53 to -0.46) | < 0.001 |
| Young and middle-age | Prevalence | Female | Georgia | Psoriasis | -0.53<br>(-0.55 to -0.5) | < 0.001 |
| Young and middle-age | Prevalence | Male | Georgia | Psoriasis | -0.48<br>(-0.54 to -0.42) | < 0.001 |
| Young and middle-age | Prevalence | Both | Georgia | RHD | 0.07<br>(0.05 to 0.09) | < 0.001 |
| Young and middle-age | Prevalence | Female | Georgia | RHD | 0.09<br>(0.05 to 0.14) | < 0.001 |
| Young and middle-age | Prevalence | Male | Georgia | RHD | 0.07<br>(0.05 to 0.1) | < 0.001 |
| Young and middle-age | Prevalence | Both | Georgia | RA | 0.29<br>(0.2 to 0.38) | < 0.001 |
| Young and middle-age | Prevalence | Female | Georgia | RA | 0.33<br>(0.22 to 0.44) | < 0.001 |
| Young and middle-age | Prevalence | Male | Georgia | RA | 0.39 | < 0.001 |

| | | | | | | |
|---|---|---|---|---|---|---|
| | | | | | (0.35 to 0.42) | |
| Young and middle-age | Prevalence | Both | Greenland | MS | 0.38<br>(0.34 to 0.42) | < 0.001 |
| Young and middle-age | Prevalence | Female | Greenland | MS | 0.25<br>(0.11 to 0.39) | 0.001 |
| Young and middle-age | Prevalence | Male | Greenland | MS | 0.43<br>(0.4 to 0.46) | < 0.001 |
| Young and middle-age | Prevalence | Both | Greenland | Psoriasis | -0.48<br>(-0.5 to -0.46) | < 0.001 |
| Young and middle-age | Prevalence | Female | Greenland | Psoriasis | -0.48<br>(-0.49 to -0.46) | < 0.001 |
| Young and middle-age | Prevalence | Male | Greenland | Psoriasis | -0.48<br>(-0.5 to -0.46) | < 0.001 |
| Young and middle-age | Prevalence | Both | Greenland | RHD | -0.77<br>(-0.84 to -0.71) | < 0.001 |
| Young and middle-age | Prevalence | Female | Greenland | RHD | -1.16<br>(-1.25 to -1.07) | < 0.001 |
| Young and middle-age | Prevalence | Male | Greenland | RHD | -0.48<br>(-0.52 to -0.45) | < 0.001 |
| Young and middle-age | Prevalence | Both | Greenland | RA | 1.16<br>(1.14 to 1.18) | < 0.001 |
| Young and middle-age | Prevalence | Female | Greenland | RA | 1.03<br>(1 to 1.06) | < 0.001 |
| Young and middle-age | Prevalence | Male | Greenland | RA | 1.02<br>(0.99 to 1.05) | < 0.001 |
| Young and middle-age | Prevalence | Both | Grenada | MS | 0.52<br>(0.46 to 0.59) | < 0.001 |
| Young and middle-age | Prevalence | Female | Grenada | MS | 0.5<br>(0.44 to 0.57) | < 0.001 |
| Young and middle-age | Prevalence | Male | Grenada | MS | 0.7<br>(0.6 to 0.79) | < 0.001 |
| Young and middle-age | Prevalence | Both | Grenada | Psoriasis | -0.63<br>(-0.65 to -0.6) | < 0.001 |

| | | | | | | |
|---|---|---|---|---|---|---|
| Young and middle-age | Prevalence | Female | Grenada | Psoriasis | -0.62 (-0.65 to -0.6) | < 0.001 |
| Young and middle-age | Prevalence | Male | Grenada | Psoriasis | -0.63 (-0.65 to -0.61) | < 0.001 |
| Young and middle-age | Prevalence | Both | Grenada | RHD | -0.03 (-0.05 to -0.01) | 0.007 |
| Young and middle-age | Prevalence | Female | Grenada | RHD | -0.01 (-0.03 to 0.01) | 0.285 |
| Young and middle-age | Prevalence | Male | Grenada | RHD | -0.03 (-0.06 to 0.01) | 0.151 |
| Young and middle-age | Prevalence | Both | Grenada | RA | 0.83 (0.81 to 0.86) | < 0.001 |
| Young and middle-age | Prevalence | Female | Grenada | RA | 0.87 (0.85 to 0.9) | < 0.001 |
| Young and middle-age | Prevalence | Male | Grenada | RA | 0.93 (0.88 to 0.98) | < 0.001 |
| Young and middle-age | Prevalence | Both | Guam | MS | 0.09 (0.03 to 0.16) | 0.004 |
| Young and middle-age | Prevalence | Female | Guam | MS | 0.04 (0.01 to 0.06) | 0.002 |
| Young and middle-age | Prevalence | Male | Guam | MS | 0.08 (0.01 to 0.15) | 0.019 |
| Young and middle-age | Prevalence | Both | Guam | Psoriasis | -0.45 (-0.51 to -0.39) | < 0.001 |
| Young and middle-age | Prevalence | Female | Guam | Psoriasis | -0.44 (-0.47 to -0.41) | < 0.001 |
| Young and middle-age | Prevalence | Male | Guam | Psoriasis | -0.47 (-0.51 to -0.43) | < 0.001 |
| Young and middle-age | Prevalence | Both | Guam | RHD | 0.19 (0.18 to 0.2) | < 0.001 |
| Young and middle-age | Prevalence | Female | Guam | RHD | 0.18 (0.15 to 0.22) | < 0.001 |
| Young and middle-age | Prevalence | Male | Guam | RHD | 0.15 | < 0.001 |

| | | | | | | |
|---|---|---|---|---|---|---|
| | | | | | (0.13 to 0.16) | |
| Young and middle-age | Prevalence | Both | Guam | RA | 0.3<br>(0.26 to 0.35) | < 0.001 |
| Young and middle-age | Prevalence | Female | Guam | RA | 0.24<br>(0.14 to 0.35) | < 0.001 |
| Young and middle-age | Prevalence | Male | Guam | RA | 0.21<br>(0.16 to 0.26) | < 0.001 |
| Young and middle-age | Prevalence | Both | Hungary | MS | -0.55<br>(-0.72 to -0.39) | < 0.001 |
| Young and middle-age | Prevalence | Female | Hungary | MS | -0.65<br>(-0.9 to -0.4) | < 0.001 |
| Young and middle-age | Prevalence | Male | Hungary | MS | -0.35<br>(-0.54 to -0.16) | < 0.001 |
| Young and middle-age | Prevalence | Both | Hungary | Psoriasis | -0.84<br>(-0.88 to -0.8) | < 0.001 |
| Young and middle-age | Prevalence | Female | Hungary | Psoriasis | -0.87<br>(-0.92 to -0.82) | < 0.001 |
| Young and middle-age | Prevalence | Male | Hungary | Psoriasis | -0.81<br>(-0.84 to -0.77) | < 0.001 |
| Young and middle-age | Prevalence | Both | Hungary | RHD | -0.99<br>(-1.14 to -0.84) | < 0.001 |
| Young and middle-age | Prevalence | Female | Hungary | RHD | -0.9<br>(-1.07 to -0.73) | < 0.001 |
| Young and middle-age | Prevalence | Male | Hungary | RHD | -1.07<br>(-1.2 to -0.94) | < 0.001 |
| Young and middle-age | Prevalence | Both | Hungary | RA | 0.41<br>(0.38 to 0.43) | < 0.001 |
| Young and middle-age | Prevalence | Female | Hungary | RA | 0.39<br>(0.37 to 0.41) | < 0.001 |
| Young and middle-age | Prevalence | Male | Hungary | RA | 0.55<br>(0.53 to 0.57) | < 0.001 |
| Young and middle-age | Prevalence | Both | Ireland | MS | 0.29<br>(0.21 to 0.36) | < 0.001 |

| | | | | | | |
|---|---|---|---|---|---|---|
| Young and middle-age | Prevalence | Female | Ireland | MS | 0.27 (0.18 to 0.36) | < 0.001 |
| Young and middle-age | Prevalence | Male | Ireland | MS | 0.21 (0.19 to 0.23) | < 0.001 |
| Young and middle-age | Prevalence | Both | Ireland | Psoriasis | -0.54 (-0.55 to -0.53) | < 0.001 |
| Young and middle-age | Prevalence | Female | Ireland | Psoriasis | -0.54 (-0.54 to -0.53) | < 0.001 |
| Young and middle-age | Prevalence | Male | Ireland | Psoriasis | -0.53 (-0.54 to -0.52) | < 0.001 |
| Young and middle-age | Prevalence | Both | Ireland | RHD | -0.47 (-0.54 to -0.4) | < 0.001 |
| Young and middle-age | Prevalence | Female | Ireland | RHD | -0.76 (-0.83 to -0.69) | < 0.001 |
| Young and middle-age | Prevalence | Male | Ireland | RHD | -0.14 (-0.19 to -0.09) | < 0.001 |
| Young and middle-age | Prevalence | Both | Ireland | RA | 0.5 (0.46 to 0.53) | < 0.001 |
| Young and middle-age | Prevalence | Female | Ireland | RA | 0.44 (0.39 to 0.48) | < 0.001 |
| Young and middle-age | Prevalence | Male | Ireland | RA | 0.53 (0.51 to 0.54) | < 0.001 |
| Young and middle-age | Prevalence | Both | Jamaica | MS | 0.46 (0.38 to 0.54) | < 0.001 |
| Young and middle-age | Prevalence | Female | Jamaica | MS | 0.48 (0.4 to 0.56) | < 0.001 |
| Young and middle-age | Prevalence | Male | Jamaica | MS | 0.39 (0.35 to 0.43) | < 0.001 |
| Young and middle-age | Prevalence | Both | Jamaica | Psoriasis | -0.47 (-0.49 to -0.45) | < 0.001 |
| Young and middle-age | Prevalence | Female | Jamaica | Psoriasis | -0.47 (-0.49 to -0.45) | < 0.001 |
| Young and middle-age | Prevalence | Male | Jamaica | Psoriasis | -0.47 | < 0.001 |

| | | | | | | |
|---|---|---|---|---|---|---|
| | | | | | (-0.49 to -0.45) | |
| Young and middle-age | Prevalence | Both | Jamaica | RHD | 0.16<br>(0.15 to 0.17) | < 0.001 |
| Young and middle-age | Prevalence | Female | Jamaica | RHD | 0.2<br>(0.18 to 0.23) | < 0.001 |
| Young and middle-age | Prevalence | Male | Jamaica | RHD | 0.12<br>(0.1 to 0.14) | < 0.001 |
| Young and middle-age | Prevalence | Both | Jamaica | RA | 0.78<br>(0.73 to 0.82) | < 0.001 |
| Young and middle-age | Prevalence | Female | Jamaica | RA | 0.78<br>(0.73 to 0.82) | < 0.001 |
| Young and middle-age | Prevalence | Male | Jamaica | RA | 0.78<br>(0.73 to 0.83) | < 0.001 |
| Young and middle-age | Prevalence | Both | Japan | MS | 0.43<br>(0.34 to 0.52) | < 0.001 |
| Young and middle-age | Prevalence | Female | Japan | MS | 0.63<br>(0.55 to 0.7) | < 0.001 |
| Young and middle-age | Prevalence | Male | Japan | MS | 0.06<br>(-0.05 to 0.18) | 0.281 |
| Young and middle-age | Prevalence | Both | Japan | Psoriasis | -0.02<br>(-0.05 to 0.01) | 0.291 |
| Young and middle-age | Prevalence | Female | Japan | Psoriasis | -0.09<br>(-0.15 to -0.04) | 0.001 |
| Young and middle-age | Prevalence | Male | Japan | Psoriasis | 0.06<br>(0.05 to 0.07) | < 0.001 |
| Young and middle-age | Prevalence | Both | Japan | RHD | -1.74<br>(-2 to -1.48) | < 0.001 |
| Young and middle-age | Prevalence | Female | Japan | RHD | -1.87<br>(-2.09 to -1.65) | < 0.001 |
| Young and middle-age | Prevalence | Male | Japan | RHD | -1.5<br>(-1.69 to -1.31) | < 0.001 |
| Young and middle-age | Prevalence | Both | Japan | RA | 0.42<br>(0.19 to 0.66) | < 0.001 |

| | | | | | | |
|---|---|---|---|---|---|---|
| Young and middle-age | Prevalence | Female | Japan | RA | 0.46<br>(0.16 to 0.77) | 0.003 |
| Young and middle-age | Prevalence | Male | Japan | RA | 0.36<br>(0.27 to 0.46) | < 0.001 |
| Young and middle-age | Prevalence | Both | Lao People's Democratic Republic | MS | 0.03<br>(-0.02 to 0.09) | 0.232 |
| Young and middle-age | Prevalence | Female | Lao People's Democratic Republic | MS | 0.08<br>(0.02 to 0.15) | 0.011 |
| Young and middle-age | Prevalence | Male | Lao People's Democratic Republic | MS | 0.05<br>(0 to 0.11) | 0.047 |
| Young and middle-age | Prevalence | Both | Lao People's Democratic Republic | Psoriasis | -0.68<br>(-0.7 to -0.66) | < 0.001 |
| Young and middle-age | Prevalence | Female | Lao People's Democratic Republic | Psoriasis | -0.64<br>(-0.66 to -0.62) | < 0.001 |
| Young and middle-age | Prevalence | Male | Lao People's Democratic Republic | Psoriasis | -0.68<br>(-0.73 to -0.63) | < 0.001 |
| Young and middle-age | Prevalence | Both | Lao People's Democratic Republic | RHD | -0.03<br>(-0.23 to 0.17) | 0.766 |
| Young and middle-age | Prevalence | Female | Lao People's Democratic Republic | RHD | -0.02<br>(-0.2 to 0.16) | 0.833 |
| Young and middle-age | Prevalence | Male | Lao People's Democratic Republic | RHD | -0.02<br>(-0.21 to 0.17) | 0.818 |
| Young and middle-age | Prevalence | Both | Lao People's Democratic Republic | RA | 0.42<br>(0.4 to 0.44) | < 0.001 |
| Young and middle-age | Prevalence | Female | Lao People's Democratic Republic | RA | 0.51<br>(0.49 to 0.53) | < 0.001 |
| Young and middle-age | Prevalence | Male | Lao People's Democratic Republic | RA | 0.4<br>(0.37 to 0.43) | < 0.001 |
| Young and middle-age | Prevalence | Both | Malaysia | MS | 0.08<br>(0.04 to 0.11) | < 0.001 |
| Young and middle-age | Prevalence | Female | Malaysia | MS | 0.11<br>(0.1 to 0.12) | < 0.001 |
| Young and middle-age | Prevalence | Male | Malaysia | MS | 0.15 | < 0.001 |

| | | | | | | |
|---|---|---|---|---|---|---|
| | | | | | (0.11 to 0.19) | |
| Young and middle-age | Prevalence | Both | Malaysia | Psoriasis | -0.64<br>(-0.68 to -0.6) | < 0.001 |
| Young and middle-age | Prevalence | Female | Malaysia | Psoriasis | -0.61<br>(-0.64 to -0.58) | < 0.001 |
| Young and middle-age | Prevalence | Male | Malaysia | Psoriasis | -0.67<br>(-0.74 to -0.61) | < 0.001 |
| Young and middle-age | Prevalence | Both | Malaysia | RHD | 0.05<br>(0.03 to 0.07) | < 0.001 |
| Young and middle-age | Prevalence | Female | Malaysia | RHD | 0.08<br>(0.07 to 0.09) | < 0.001 |
| Young and middle-age | Prevalence | Male | Malaysia | RHD | 0.05<br>(0.04 to 0.07) | < 0.001 |
| Young and middle-age | Prevalence | Both | Malaysia | RA | 0.48<br>(0.46 to 0.5) | < 0.001 |
| Young and middle-age | Prevalence | Female | Malaysia | RA | 0.53<br>(0.5 to 0.56) | < 0.001 |
| Young and middle-age | Prevalence | Male | Malaysia | RA | 0.53<br>(0.48 to 0.57) | < 0.001 |
| Young and middle-age | Prevalence | Both | Mongolia | MS | 0.37<br>(0.24 to 0.5) | < 0.001 |
| Young and middle-age | Prevalence | Female | Mongolia | MS | 0.38<br>(0.23 to 0.52) | < 0.001 |
| Young and middle-age | Prevalence | Male | Mongolia | MS | 0.4<br>(0.21 to 0.6) | < 0.001 |
| Young and middle-age | Prevalence | Both | Mongolia | Psoriasis | -0.88<br>(-0.91 to -0.86) | < 0.001 |
| Young and middle-age | Prevalence | Female | Mongolia | Psoriasis | -0.9<br>(-0.93 to -0.87) | < 0.001 |
| Young and middle-age | Prevalence | Male | Mongolia | Psoriasis | -0.87<br>(-0.89 to -0.85) | < 0.001 |
| Young and middle-age | Prevalence | Both | Mongolia | RHD | -0.04<br>(-0.05 to -0.02) | < 0.001 |

| | | | | | | |
|---|---|---|---|---|---|---|
| Young and middle-age | Prevalence | Female | Mongolia | RHD | -0.04 (-0.06 to -0.02) | < 0.001 |
| Young and middle-age | Prevalence | Male | Mongolia | RHD | -0.03 (-0.05 to -0.02) | < 0.001 |
| Young and middle-age | Prevalence | Both | Mongolia | RA | 0.87 (0.81 to 0.93) | < 0.001 |
| Young and middle-age | Prevalence | Female | Mongolia | RA | 0.95 (0.91 to 0.99) | < 0.001 |
| Young and middle-age | Prevalence | Male | Mongolia | RA | 0.64 (0.46 to 0.83) | < 0.001 |
| Young and middle-age | Prevalence | Both | Montenegro | MS | 0.42 (0.35 to 0.49) | < 0.001 |
| Young and middle-age | Prevalence | Female | Montenegro | MS | 0.6 (0.52 to 0.68) | < 0.001 |
| Young and middle-age | Prevalence | Male | Montenegro | MS | 0.13 (0.09 to 0.18) | < 0.001 |
| Young and middle-age | Prevalence | Both | Montenegro | Psoriasis | -0.68 (-0.72 to -0.65) | < 0.001 |
| Young and middle-age | Prevalence | Female | Montenegro | Psoriasis | -0.7 (-0.76 to -0.65) | < 0.001 |
| Young and middle-age | Prevalence | Male | Montenegro | Psoriasis | -0.67 (-0.68 to -0.65) | < 0.001 |
| Young and middle-age | Prevalence | Both | Montenegro | RHD | -0.13 (-0.2 to -0.06) | < 0.001 |
| Young and middle-age | Prevalence | Female | Montenegro | RHD | -0.11 (-0.18 to -0.04) | 0.001 |
| Young and middle-age | Prevalence | Male | Montenegro | RHD | -0.15 (-0.24 to -0.06) | 0.001 |
| Young and middle-age | Prevalence | Both | Montenegro | RA | 0.46 (0.42 to 0.5) | < 0.001 |
| Young and middle-age | Prevalence | Female | Montenegro | RA | 0.44 (0.41 to 0.48) | < 0.001 |
| Young and middle-age | Prevalence | Male | Montenegro | RA | 0.46 | < 0.001 |

| | | | | | | |
|---|---|---|---|---|---|---|
| | | | | | (0.44 to 0.48) | |
| Young and middle-age | Prevalence | Both | New Zealand | MS | -0.17 (-0.22 to -0.12) | < 0.001 |
| Young and middle-age | Prevalence | Female | New Zealand | MS | -0.15 (-0.19 to -0.11) | < 0.001 |
| Young and middle-age | Prevalence | Male | New Zealand | MS | -0.57 (-0.66 to -0.49) | < 0.001 |
| Young and middle-age | Prevalence | Both | New Zealand | Psoriasis | -0.38 (-0.4 to -0.37) | < 0.001 |
| Young and middle-age | Prevalence | Female | New Zealand | Psoriasis | -0.38 (-0.39 to -0.36) | < 0.001 |
| Young and middle-age | Prevalence | Male | New Zealand | Psoriasis | -0.4 (-0.42 to -0.38) | < 0.001 |
| Young and middle-age | Prevalence | Both | New Zealand | RHD | -1.04 (-1.13 to -0.96) | < 0.001 |
| Young and middle-age | Prevalence | Female | New Zealand | RHD | -1.03 (-1.09 to -0.96) | < 0.001 |
| Young and middle-age | Prevalence | Male | New Zealand | RHD | -1.16 (-1.33 to -0.99) | < 0.001 |
| Young and middle-age | Prevalence | Both | New Zealand | RA | 0.35 (0.33 to 0.37) | < 0.001 |
| Young and middle-age | Prevalence | Female | New Zealand | RA | 0.23 (0.19 to 0.28) | < 0.001 |
| Young and middle-age | Prevalence | Male | New Zealand | RA | 0.44 (0.4 to 0.48) | < 0.001 |
| Young and middle-age | Prevalence | Both | North Macedonia | MS | 0.86 (0.82 to 0.91) | < 0.001 |
| Young and middle-age | Prevalence | Female | North Macedonia | MS | 0.95 (0.9 to 1.01) | < 0.001 |
| Young and middle-age | Prevalence | Male | North Macedonia | MS | 0.75 (0.72 to 0.78) | < 0.001 |
| Young and middle-age | Prevalence | Both | North Macedonia | Psoriasis | -0.82 (-0.84 to -0.8) | < 0.001 |

| | | | | | | |
|---|---|---|---|---|---|---|
| Young and middle-age | Prevalence | Female | North Macedonia | Psoriasis | -0.86<br>(-0.9 to -0.82) | < 0.001 |
| Young and middle-age | Prevalence | Male | North Macedonia | Psoriasis | -0.81<br>(-0.83 to -0.79) | < 0.001 |
| Young and middle-age | Prevalence | Both | North Macedonia | RHD | -1.07<br>(-1.16 to -0.97) | < 0.001 |
| Young and middle-age | Prevalence | Female | North Macedonia | RHD | -1.6<br>(-1.7 to -1.49) | < 0.001 |
| Young and middle-age | Prevalence | Male | North Macedonia | RHD | -0.56<br>(-0.68 to -0.44) | < 0.001 |
| Young and middle-age | Prevalence | Both | North Macedonia | RA | 0.69<br>(0.64 to 0.75) | < 0.001 |
| Young and middle-age | Prevalence | Female | North Macedonia | RA | 0.71<br>(0.66 to 0.77) | < 0.001 |
| Young and middle-age | Prevalence | Male | North Macedonia | RA | 0.72<br>(0.69 to 0.75) | < 0.001 |
| Young and middle-age | Prevalence | Both | Northern Mariana Islands | MS | 0.04<br>(0 to 0.08) | 0.028 |
| Young and middle-age | Prevalence | Female | Northern Mariana Islands | MS | 0.03<br>(0.01 to 0.05) | 0.003 |
| Young and middle-age | Prevalence | Male | Northern Mariana Islands | MS | 0.06<br>(0 to 0.13) | 0.053 |
| Young and middle-age | Prevalence | Both | Northern Mariana Islands | Psoriasis | -0.17<br>(-0.28 to -0.05) | 0.004 |
| Young and middle-age | Prevalence | Female | Northern Mariana Islands | Psoriasis | -0.15<br>(-0.17 to -0.12) | < 0.001 |
| Young and middle-age | Prevalence | Male | Northern Mariana Islands | Psoriasis | -0.18<br>(-0.21 to -0.15) | < 0.001 |
| Young and middle-age | Prevalence | Both | Northern Mariana Islands | RHD | 0.14<br>(0.1 to 0.18) | < 0.001 |
| Young and middle-age | Prevalence | Female | Northern Mariana Islands | RHD | 0.16<br>(0.14 to 0.18) | < 0.001 |
| Young and middle-age | Prevalence | Male | Northern Mariana Islands | RHD | 0.14 | < 0.001 |

| | | | | | | |
|---|---|---|---|---|---|---|
| | | | | | (0.12 to 0.15) | |
| Young and middle-age | Prevalence | Both | Northern Mariana Islands | RA | 0.32<br>(0.19 to 0.46) | < 0.001 |
| Young and middle-age | Prevalence | Female | Northern Mariana Islands | RA | 0.29<br>(0.23 to 0.36) | < 0.001 |
| Young and middle-age | Prevalence | Male | Northern Mariana Islands | RA | 0.2<br>(0.18 to 0.23) | < 0.001 |
| Young and middle-age | Prevalence | Both | Palestine | MS | 0.54<br>(0.48 to 0.6) | < 0.001 |
| Young and middle-age | Prevalence | Female | Palestine | MS | 0.57<br>(0.53 to 0.61) | < 0.001 |
| Young and middle-age | Prevalence | Male | Palestine | MS | 0.64<br>(0.62 to 0.66) | < 0.001 |
| Young and middle-age | Prevalence | Both | Palestine | Psoriasis | -1.28<br>(-1.33 to -1.23) | < 0.001 |
| Young and middle-age | Prevalence | Female | Palestine | Psoriasis | -1.27<br>(-1.34 to -1.2) | < 0.001 |
| Young and middle-age | Prevalence | Male | Palestine | Psoriasis | -1.28<br>(-1.33 to -1.23) | < 0.001 |
| Young and middle-age | Prevalence | Both | Palestine | RHD | 0.05<br>(0.04 to 0.06) | < 0.001 |
| Young and middle-age | Prevalence | Female | Palestine | RHD | 0.06<br>(0.06 to 0.07) | < 0.001 |
| Young and middle-age | Prevalence | Male | Palestine | RHD | 0.06<br>(0.04 to 0.07) | < 0.001 |
| Young and middle-age | Prevalence | Both | Palestine | RA | 0.54<br>(0.5 to 0.59) | < 0.001 |
| Young and middle-age | Prevalence | Female | Palestine | RA | 0.61<br>(0.57 to 0.65) | < 0.001 |
| Young and middle-age | Prevalence | Male | Palestine | RA | 0.63<br>(0.55 to 0.71) | < 0.001 |
| Young and middle-age | Prevalence | Both | Puerto Rico | MS | 0.63<br>(0.54 to 0.72) | < 0.001 |

| | | | | | | |
|---|---|---|---|---|---|---|
| Young and middle-age | Prevalence | Female | Puerto Rico | MS | 0.57<br>(0.49 to 0.64) | < 0.001 |
| Young and middle-age | Prevalence | Male | Puerto Rico | MS | 0.83<br>(0.8 to 0.86) | < 0.001 |
| Young and middle-age | Prevalence | Both | Puerto Rico | Psoriasis | -0.4<br>(-0.41 to -0.39) | < 0.001 |
| Young and middle-age | Prevalence | Female | Puerto Rico | Psoriasis | -0.4<br>(-0.41 to -0.39) | < 0.001 |
| Young and middle-age | Prevalence | Male | Puerto Rico | Psoriasis | -0.41<br>(-0.43 to -0.39) | < 0.001 |
| Young and middle-age | Prevalence | Both | Puerto Rico | RHD | -0.33<br>(-0.39 to -0.26) | < 0.001 |
| Young and middle-age | Prevalence | Female | Puerto Rico | RHD | -0.48<br>(-0.55 to -0.4) | < 0.001 |
| Young and middle-age | Prevalence | Male | Puerto Rico | RHD | -0.04<br>(-0.09 to 0.01) | 0.094 |
| Young and middle-age | Prevalence | Both | Puerto Rico | RA | 0.91<br>(0.88 to 0.93) | < 0.001 |
| Young and middle-age | Prevalence | Female | Puerto Rico | RA | 0.94<br>(0.92 to 0.96) | < 0.001 |
| Young and middle-age | Prevalence | Male | Puerto Rico | RA | 0.91<br>(0.87 to 0.94) | < 0.001 |
| Young and middle-age | Prevalence | Both | Republic of Korea | MS | 0.04<br>(0.02 to 0.06) | < 0.001 |
| Young and middle-age | Prevalence | Female | Republic of Korea | MS | 0.12<br>(0.1 to 0.13) | < 0.001 |
| Young and middle-age | Prevalence | Male | Republic of Korea | MS | 0.01<br>(-0.02 to 0.04) | 0.526 |
| Young and middle-age | Prevalence | Both | Republic of Korea | Psoriasis | -0.47<br>(-0.49 to -0.45) | < 0.001 |
| Young and middle-age | Prevalence | Female | Republic of Korea | Psoriasis | -0.55<br>(-0.57 to -0.53) | < 0.001 |
| Young and middle-age | Prevalence | Male | Republic of Korea | Psoriasis | -0.4 | < 0.001 |

| | | | | | | |
|---|---|---|---|---|---|---|
| | | | | | (-0.42 to -0.38) | |
| Young and middle-age | Prevalence | Both | Republic of Korea | RHD | -0.47 (-0.51 to -0.43) | < 0.001 |
| Young and middle-age | Prevalence | Female | Republic of Korea | RHD | -0.71 (-0.79 to -0.64) | < 0.001 |
| Young and middle-age | Prevalence | Male | Republic of Korea | RHD | -0.16 (-0.21 to -0.12) | < 0.001 |
| Young and middle-age | Prevalence | Both | Republic of Korea | RA | 0.48 (0.47 to 0.5) | < 0.001 |
| Young and middle-age | Prevalence | Female | Republic of Korea | RA | 0.49 (0.48 to 0.49) | < 0.001 |
| Young and middle-age | Prevalence | Male | Republic of Korea | RA | 0.62 (0.59 to 0.66) | < 0.001 |
| Young and middle-age | Prevalence | Both | Republic of Moldova | MS | 0.46 (0.33 to 0.59) | < 0.001 |
| Young and middle-age | Prevalence | Female | Republic of Moldova | MS | 0.51 (0.33 to 0.68) | < 0.001 |
| Young and middle-age | Prevalence | Male | Republic of Moldova | MS | 0.45 (0.3 to 0.6) | < 0.001 |
| Young and middle-age | Prevalence | Both | Republic of Moldova | Psoriasis | -0.66 (-0.68 to -0.63) | < 0.001 |
| Young and middle-age | Prevalence | Female | Republic of Moldova | Psoriasis | -0.65 (-0.68 to -0.63) | < 0.001 |
| Young and middle-age | Prevalence | Male | Republic of Moldova | Psoriasis | -0.67 (-0.69 to -0.64) | < 0.001 |
| Young and middle-age | Prevalence | Both | Republic of Moldova | RHD | -3.48 (-3.67 to -3.29) | < 0.001 |
| Young and middle-age | Prevalence | Female | Republic of Moldova | RHD | -3.39 (-3.56 to -3.22) | < 0.001 |
| Young and middle-age | Prevalence | Male | Republic of Moldova | RHD | -3.5 (-3.76 to -3.24) | < 0.001 |
| Young and middle-age | Prevalence | Both | Republic of Moldova | RA | 0.67 (0.62 to 0.73) | < 0.001 |

| | | | | | | |
|---|---|---|---|---|---|---|
| Young and middle-age | Prevalence | Female | Republic of Moldova | RA | 0.75 (0.68 to 0.82) | < 0.001 |
| Young and middle-age | Prevalence | Male | Republic of Moldova | RA | 0.74 (0.72 to 0.76) | < 0.001 |
| Young and middle-age | Prevalence | Both | Romania | MS | -0.06 (-0.15 to 0.03) | 0.188 |
| Young and middle-age | Prevalence | Female | Romania | MS | 0.02 (-0.04 to 0.09) | 0.519 |
| Young and middle-age | Prevalence | Male | Romania | MS | -0.15 (-0.28 to -0.02) | 0.025 |
| Young and middle-age | Prevalence | Both | Romania | Psoriasis | -0.85 (-0.91 to -0.8) | < 0.001 |
| Young and middle-age | Prevalence | Female | Romania | Psoriasis | -0.89 (-1 to -0.79) | < 0.001 |
| Young and middle-age | Prevalence | Male | Romania | Psoriasis | -0.83 (-0.86 to -0.81) | < 0.001 |
| Young and middle-age | Prevalence | Both | Romania | RHD | -2.52 (-2.65 to -2.4) | < 0.001 |
| Young and middle-age | Prevalence | Female | Romania | RHD | -2.61 (-2.77 to -2.45) | < 0.001 |
| Young and middle-age | Prevalence | Male | Romania | RHD | -2.42 (-2.65 to -2.19) | < 0.001 |
| Young and middle-age | Prevalence | Both | Romania | RA | 0.72 (0.67 to 0.77) | < 0.001 |
| Young and middle-age | Prevalence | Female | Romania | RA | 0.74 (0.68 to 0.81) | < 0.001 |
| Young and middle-age | Prevalence | Male | Romania | RA | 0.76 (0.73 to 0.79) | < 0.001 |
| Young and middle-age | Prevalence | Both | Russian Federation | MS | -0.53 (-0.6 to -0.46) | < 0.001 |
| Young and middle-age | Prevalence | Female | Russian Federation | MS | -0.48 (-0.54 to -0.43) | < 0.001 |
| Young and middle-age | Prevalence | Male | Russian Federation | MS | -0.61 | < 0.001 |

| | | | | | | |
|---|---|---|---|---|---|---|
| | | | | | (-0.69 to -0.52) | |
| Young and middle-age | Prevalence | Both | Russian Federation | Psoriasis | -0.44<br>(-0.46 to -0.42) | < 0.001 |
| Young and middle-age | Prevalence | Female | Russian Federation | Psoriasis | -0.43<br>(-0.45 to -0.41) | < 0.001 |
| Young and middle-age | Prevalence | Male | Russian Federation | Psoriasis | -0.44<br>(-0.46 to -0.42) | < 0.001 |
| Young and middle-age | Prevalence | Both | Russian Federation | RHD | -1.92<br>(-2 to -1.84) | < 0.001 |
| Young and middle-age | Prevalence | Female | Russian Federation | RHD | -1.76<br>(-1.83 to -1.68) | < 0.001 |
| Young and middle-age | Prevalence | Male | Russian Federation | RHD | -2.13<br>(-2.22 to -2.04) | < 0.001 |
| Young and middle-age | Prevalence | Both | Russian Federation | RA | 0.21<br>(0.16 to 0.25) | < 0.001 |
| Young and middle-age | Prevalence | Female | Russian Federation | RA | 0.21<br>(0.17 to 0.25) | < 0.001 |
| Young and middle-age | Prevalence | Male | Russian Federation | RA | 0.18<br>(0.1 to 0.27) | < 0.001 |
| Young and middle-age | Prevalence | Both | Saint Kitts and Nevis | MS | 0.34<br>(0.27 to 0.41) | < 0.001 |
| Young and middle-age | Prevalence | Female | Saint Kitts and Nevis | MS | 0.25<br>(0.17 to 0.33) | < 0.001 |
| Young and middle-age | Prevalence | Male | Saint Kitts and Nevis | MS | 0.54<br>(0.42 to 0.66) | < 0.001 |
| Young and middle-age | Prevalence | Both | Saint Kitts and Nevis | Psoriasis | -0.48<br>(-0.48 to -0.47) | < 0.001 |
| Young and middle-age | Prevalence | Female | Saint Kitts and Nevis | Psoriasis | -0.48<br>(-0.48 to -0.47) | < 0.001 |
| Young and middle-age | Prevalence | Male | Saint Kitts and Nevis | Psoriasis | -0.47<br>(-0.48 to -0.47) | < 0.001 |
| Young and middle-age | Prevalence | Both | Saint Kitts and Nevis | RHD | -2.76<br>(-2.89 to -2.63) | < 0.001 |

| | | | | | | |
|---|---|---|---|---|---|---|
| Young and middle-age | Prevalence | Female | Saint Kitts and Nevis | RHD | -3.29 (-3.47 to -3.11) | < 0.001 |
| Young and middle-age | Prevalence | Male | Saint Kitts and Nevis | RHD | -1.72 (-1.81 to -1.62) | < 0.001 |
| Young and middle-age | Prevalence | Both | Saint Kitts and Nevis | RA | 0.71 (0.68 to 0.73) | < 0.001 |
| Young and middle-age | Prevalence | Female | Saint Kitts and Nevis | RA | 0.72 (0.7 to 0.74) | < 0.001 |
| Young and middle-age | Prevalence | Male | Saint Kitts and Nevis | RA | 0.83 (0.8 to 0.86) | < 0.001 |
| Young and middle-age | Prevalence | Both | Saint Lucia | MS | 0.49 (0.47 to 0.52) | < 0.001 |
| Young and middle-age | Prevalence | Female | Saint Lucia | MS | 0.52 (0.49 to 0.55) | < 0.001 |
| Young and middle-age | Prevalence | Male | Saint Lucia | MS | 0.51 (0.44 to 0.57) | < 0.001 |
| Young and middle-age | Prevalence | Both | Saint Lucia | Psoriasis | -0.5 (-0.5 to -0.49) | < 0.001 |
| Young and middle-age | Prevalence | Female | Saint Lucia | Psoriasis | -0.5 (-0.51 to -0.49) | < 0.001 |
| Young and middle-age | Prevalence | Male | Saint Lucia | Psoriasis | -0.5 (-0.51 to -0.49) | < 0.001 |
| Young and middle-age | Prevalence | Both | Saint Lucia | RHD | 0 (-0.03 to 0.03) | 0.958 |
| Young and middle-age | Prevalence | Female | Saint Lucia | RHD | 0.04 (0.01 to 0.07) | 0.022 |
| Young and middle-age | Prevalence | Male | Saint Lucia | RHD | -0.04 (-0.08 to 0.01) | 0.126 |
| Young and middle-age | Prevalence | Both | Saint Lucia | RA | 0.8 (0.77 to 0.83) | < 0.001 |
| Young and middle-age | Prevalence | Female | Saint Lucia | RA | 0.82 (0.77 to 0.86) | < 0.001 |
| Young and middle-age | Prevalence | Male | Saint Lucia | RA | 0.91 | < 0.001 |

| | | | | | | |
|---|---|---|---|---|---|---|
| | | | | | (0.88 to 0.94) | |
| Young and middle-age | Prevalence | Both | Saint Vincent and the Grenadines | MS | 0.57<br>(0.55 to 0.59) | < 0.001 |
| Young and middle-age | Prevalence | Female | Saint Vincent and the Grenadines | MS | 0.68<br>(0.64 to 0.71) | < 0.001 |
| Young and middle-age | Prevalence | Male | Saint Vincent and the Grenadines | MS | 0.28<br>(0.25 to 0.3) | < 0.001 |
| Young and middle-age | Prevalence | Both | Saint Vincent and the Grenadines | Psoriasis | -0.49<br>(-0.5 to -0.48) | < 0.001 |
| Young and middle-age | Prevalence | Female | Saint Vincent and the Grenadines | Psoriasis | -0.49<br>(-0.5 to -0.48) | < 0.001 |
| Young and middle-age | Prevalence | Male | Saint Vincent and the Grenadines | Psoriasis | -0.49<br>(-0.5 to -0.48) | < 0.001 |
| Young and middle-age | Prevalence | Both | Saint Vincent and the Grenadines | RHD | -0.01<br>(-0.06 to 0.04) | 0.575 |
| Young and middle-age | Prevalence | Female | Saint Vincent and the Grenadines | RHD | 0.01<br>(-0.03 to 0.05) | 0.675 |
| Young and middle-age | Prevalence | Male | Saint Vincent and the Grenadines | RHD | -0.07<br>(-0.13 to -0.01) | 0.022 |
| Young and middle-age | Prevalence | Both | Saint Vincent and the Grenadines | RA | 0.77<br>(0.73 to 0.81) | < 0.001 |
| Young and middle-age | Prevalence | Female | Saint Vincent and the Grenadines | RA | 0.75<br>(0.71 to 0.78) | < 0.001 |
| Young and middle-age | Prevalence | Male | Saint Vincent and the Grenadines | RA | 0.84<br>(0.8 to 0.88) | < 0.001 |
| Young and middle-age | Prevalence | Both | Solomon Islands | MS | 0.08<br>(0.05 to 0.11) | < 0.001 |
| Young and middle-age | Prevalence | Female | Solomon Islands | MS | 0.03<br>(-0.03 to 0.09) | 0.272 |
| Young and middle-age | Prevalence | Male | Solomon Islands | MS | 0.07<br>(0.01 to 0.13) | 0.016 |
| Young and middle-age | Prevalence | Both | Solomon Islands | Psoriasis | -0.48<br>(-0.51 to -0.45) | < 0.001 |

| | | | | | | |
|---|---|---|---|---|---|---|
| Young and middle-age | Prevalence | Female | Solomon Islands | Psoriasis | -0.46 (-0.5 to -0.42) | < 0.001 |
| Young and middle-age | Prevalence | Male | Solomon Islands | Psoriasis | -0.48 (-0.51 to -0.45) | < 0.001 |
| Young and middle-age | Prevalence | Both | Solomon Islands | RHD | 0.06 (0.03 to 0.09) | < 0.001 |
| Young and middle-age | Prevalence | Female | Solomon Islands | RHD | 0.08 (0.07 to 0.09) | < 0.001 |
| Young and middle-age | Prevalence | Male | Solomon Islands | RHD | 0.04 (0.02 to 0.06) | < 0.001 |
| Young and middle-age | Prevalence | Both | Solomon Islands | RA | 0.39 (0.31 to 0.47) | < 0.001 |
| Young and middle-age | Prevalence | Female | Solomon Islands | RA | 0.33 (0.22 to 0.45) | < 0.001 |
| Young and middle-age | Prevalence | Male | Solomon Islands | RA | 0.32 (0.26 to 0.39) | < 0.001 |
| Young and middle-age | Prevalence | Both | Syrian Arab Republic | MS | 0.74 (0.68 to 0.81) | < 0.001 |
| Young and middle-age | Prevalence | Female | Syrian Arab Republic | MS | 0.55 (0.46 to 0.64) | < 0.001 |
| Young and middle-age | Prevalence | Male | Syrian Arab Republic | MS | 0.66 (0.6 to 0.71) | < 0.001 |
| Young and middle-age | Prevalence | Both | Syrian Arab Republic | Psoriasis | -0.96 (-0.97 to -0.94) | < 0.001 |
| Young and middle-age | Prevalence | Female | Syrian Arab Republic | Psoriasis | -0.98 (-0.99 to -0.96) | < 0.001 |
| Young and middle-age | Prevalence | Male | Syrian Arab Republic | Psoriasis | -0.98 (-0.99 to -0.97) | < 0.001 |
| Young and middle-age | Prevalence | Both | Syrian Arab Republic | RHD | 0.13 (0.12 to 0.14) | < 0.001 |
| Young and middle-age | Prevalence | Female | Syrian Arab Republic | RHD | 0.11 (0.1 to 0.12) | < 0.001 |
| Young and middle-age | Prevalence | Male | Syrian Arab Republic | RHD | 0.11 | < 0.001 |

| | | | | | | |
|---|---|---|---|---|---|---|
| | | | | | (0.09 to 0.14) | |
| Young and middle-age | Prevalence | Both | Syrian Arab Republic | RA | 0.95<br>(0.87 to 1.03) | < 0.001 |
| Young and middle-age | Prevalence | Female | Syrian Arab Republic | RA | 0.78<br>(0.67 to 0.88) | < 0.001 |
| Young and middle-age | Prevalence | Male | Syrian Arab Republic | RA | 0.76<br>(0.71 to 0.81) | < 0.001 |
| Young and middle-age | Prevalence | Both | Taiwan (Province of China) | MS | 3.51<br>(3.45 to 3.57) | < 0.001 |
| Young and middle-age | Prevalence | Female | Taiwan (Province of China) | MS | 3.77<br>(3.7 to 3.83) | < 0.001 |
| Young and middle-age | Prevalence | Male | Taiwan (Province of China) | MS | 3.1<br>(3.04 to 3.16) | < 0.001 |
| Young and middle-age | Prevalence | Both | Taiwan (Province of China) | Psoriasis | -0.97<br>(-1.03 to -0.92) | < 0.001 |
| Young and middle-age | Prevalence | Female | Taiwan (Province of China) | Psoriasis | -0.96<br>(-1.02 to -0.9) | < 0.001 |
| Young and middle-age | Prevalence | Male | Taiwan (Province of China) | Psoriasis | -0.96<br>(-1.02 to -0.9) | < 0.001 |
| Young and middle-age | Prevalence | Both | Taiwan (Province of China) | RHD | 0.46<br>(0.32 to 0.6) | < 0.001 |
| Young and middle-age | Prevalence | Female | Taiwan (Province of China) | RHD | 0.09<br>(0 to 0.18) | 0.052 |
| Young and middle-age | Prevalence | Male | Taiwan (Province of China) | RHD | 1.04<br>(0.95 to 1.13) | < 0.001 |
| Young and middle-age | Prevalence | Both | Taiwan (Province of China) | RA | 0.11<br>(0.06 to 0.16) | < 0.001 |
| Young and middle-age | Prevalence | Female | Taiwan (Province of China) | RA | 0.05<br>(0.02 to 0.08) | 0.003 |
| Young and middle-age | Prevalence | Male | Taiwan (Province of China) | RA | 0.11<br>(0.04 to 0.19) | 0.003 |
| Young and middle-age | Prevalence | Both | Tokelau | MS | 0.02<br>(-0.06 to 0.1) | 0.589 |

| | | | | | | |
|---|---|---|---|---|---|---|
| Young and middle-age | Prevalence | Female | Tokelau | MS | 0.08 (0.04 to 0.12) | < 0.001 |
| Young and middle-age | Prevalence | Male | Tokelau | MS | 0.09 (0.03 to 0.16) | 0.003 |
| Young and middle-age | Prevalence | Both | Tokelau | Psoriasis | -0.65 (-0.69 to -0.62) | < 0.001 |
| Young and middle-age | Prevalence | Female | Tokelau | Psoriasis | -0.64 (-0.65 to -0.62) | < 0.001 |
| Young and middle-age | Prevalence | Male | Tokelau | Psoriasis | -0.68 (-0.71 to -0.66) | < 0.001 |
| Young and middle-age | Prevalence | Both | Tokelau | RHD | -0.48 (-0.54 to -0.42) | < 0.001 |
| Young and middle-age | Prevalence | Female | Tokelau | RHD | -0.42 (-0.53 to -0.31) | < 0.001 |
| Young and middle-age | Prevalence | Male | Tokelau | RHD | -0.44 (-0.48 to -0.4) | < 0.001 |
| Young and middle-age | Prevalence | Both | Tokelau | RA | 0.54 (0.49 to 0.59) | < 0.001 |
| Young and middle-age | Prevalence | Female | Tokelau | RA | 0.68 (0.66 to 0.7) | < 0.001 |
| Young and middle-age | Prevalence | Male | Tokelau | RA | 0.54 (0.52 to 0.56) | < 0.001 |
| Young and middle-age | Prevalence | Both | Turkmenistan | MS | 0.42 (0.31 to 0.52) | < 0.001 |
| Young and middle-age | Prevalence | Female | Turkmenistan | MS | 0.48 (0.37 to 0.6) | < 0.001 |
| Young and middle-age | Prevalence | Male | Turkmenistan | MS | 0.47 (0.38 to 0.56) | < 0.001 |
| Young and middle-age | Prevalence | Both | Turkmenistan | Psoriasis | -0.99 (-1.07 to -0.91) | < 0.001 |
| Young and middle-age | Prevalence | Female | Turkmenistan | Psoriasis | -1.01 (-1.09 to -0.92) | < 0.001 |
| Young and middle-age | Prevalence | Male | Turkmenistan | Psoriasis | -0.95 | < 0.001 |

| | | | | | | |
|---|---|---|---|---|---|---|
| | | | | | (-1.01 to -0.9) | |
| Young and middle-age | Prevalence | Both | Turkmenistan | RHD | -0.01<br>(-0.05 to 0.03) | 0.613 |
| Young and middle-age | Prevalence | Female | Turkmenistan | RHD | 0.01<br>(-0.02 to 0.05) | 0.466 |
| Young and middle-age | Prevalence | Male | Turkmenistan | RHD | 0.02<br>(-0.02 to 0.05) | 0.365 |
| Young and middle-age | Prevalence | Both | Turkmenistan | RA | 0.8<br>(0.74 to 0.86) | < 0.001 |
| Young and middle-age | Prevalence | Female | Turkmenistan | RA | 0.9<br>(0.81 to 0.99) | < 0.001 |
| Young and middle-age | Prevalence | Male | Turkmenistan | RA | 0.88<br>(0.8 to 0.96) | < 0.001 |
| Young and middle-age | Prevalence | Both | Tuvalu | MS | -0.05<br>(-0.11 to 0.02) | 0.146 |
| Young and middle-age | Prevalence | Female | Tuvalu | MS | 0.09<br>(0.05 to 0.14) | < 0.001 |
| Young and middle-age | Prevalence | Male | Tuvalu | MS | 0.05<br>(-0.01 to 0.1) | 0.088 |
| Young and middle-age | Prevalence | Both | Tuvalu | Psoriasis | -0.57<br>(-0.59 to -0.54) | < 0.001 |
| Young and middle-age | Prevalence | Female | Tuvalu | Psoriasis | -0.55<br>(-0.56 to -0.53) | < 0.001 |
| Young and middle-age | Prevalence | Male | Tuvalu | Psoriasis | -0.59<br>(-0.63 to -0.54) | < 0.001 |
| Young and middle-age | Prevalence | Both | Tuvalu | RHD | -0.68<br>(-0.74 to -0.62) | < 0.001 |
| Young and middle-age | Prevalence | Female | Tuvalu | RHD | -0.61<br>(-0.68 to -0.55) | < 0.001 |
| Young and middle-age | Prevalence | Male | Tuvalu | RHD | -0.33<br>(-0.38 to -0.27) | < 0.001 |
| Young and middle-age | Prevalence | Both | Tuvalu | RA | 0.23<br>(0.21 to 0.24) | < 0.001 |

| | | | | | | |
|---|---|---|---|---|---|---|
| Young and middle-age | Prevalence | Female | Tuvalu | RA | 0.5<br>(0.47 to 0.52) | < 0.001 |
| Young and middle-age | Prevalence | Male | Tuvalu | RA | 0.39<br>(0.35 to 0.42) | < 0.001 |
| Young and middle-age | Prevalence | Both | Ukraine | MS | -0.49<br>(-0.59 to -0.39) | < 0.001 |
| Young and middle-age | Prevalence | Female | Ukraine | MS | -0.54<br>(-0.64 to -0.43) | < 0.001 |
| Young and middle-age | Prevalence | Male | Ukraine | MS | -0.44<br>(-0.56 to -0.32) | < 0.001 |
| Young and middle-age | Prevalence | Both | Ukraine | Psoriasis | -0.53<br>(-0.55 to -0.51) | < 0.001 |
| Young and middle-age | Prevalence | Female | Ukraine | Psoriasis | -0.53<br>(-0.55 to -0.51) | < 0.001 |
| Young and middle-age | Prevalence | Male | Ukraine | Psoriasis | -0.53<br>(-0.54 to -0.51) | < 0.001 |
| Young and middle-age | Prevalence | Both | Ukraine | RHD | -0.59<br>(-0.82 to -0.36) | < 0.001 |
| Young and middle-age | Prevalence | Female | Ukraine | RHD | -0.57<br>(-0.83 to -0.3) | < 0.001 |
| Young and middle-age | Prevalence | Male | Ukraine | RHD | -0.59<br>(-0.74 to -0.45) | < 0.001 |
| Young and middle-age | Prevalence | Both | Ukraine | RA | 0.19<br>(0.11 to 0.27) | < 0.001 |
| Young and middle-age | Prevalence | Female | Ukraine | RA | 0.22<br>(0.12 to 0.31) | < 0.001 |
| Young and middle-age | Prevalence | Male | Ukraine | RA | 0.18<br>(0.1 to 0.26) | < 0.001 |
| Young and middle-age | Prevalence | Both | United Arab Emirates | MS | -0.03<br>(-0.11 to 0.06) | 0.532 |
| Young and middle-age | Prevalence | Female | United Arab Emirates | MS | 0.25<br>(0.21 to 0.3) | < 0.001 |
| Young and middle-age | Prevalence | Male | United Arab Emirates | MS | -0.2 | < 0.001 |

| | | | | | | |
|---|---|---|---|---|---|---|
| | | | | | (-0.3 to -0.09) | |
| Young and middle-age | Prevalence | Both | United Arab Emirates | Psoriasis | -1<br>(-1.04 to -0.95) | < 0.001 |
| Young and middle-age | Prevalence | Female | United Arab Emirates | Psoriasis | -0.99<br>(-1 to -0.97) | < 0.001 |
| Young and middle-age | Prevalence | Male | United Arab Emirates | Psoriasis | -1.03<br>(-1.05 to -1.02) | < 0.001 |
| Young and middle-age | Prevalence | Both | United Arab Emirates | RHD | 0.02<br>(0 to 0.03) | 0.111 |
| Young and middle-age | Prevalence | Female | United Arab Emirates | RHD | 0.02<br>(0.01 to 0.03) | < 0.001 |
| Young and middle-age | Prevalence | Male | United Arab Emirates | RHD | 0.01<br>(-0.01 to 0.04) | 0.171 |
| Young and middle-age | Prevalence | Both | United Arab Emirates | RA | 0.86<br>(0.79 to 0.93) | < 0.001 |
| Young and middle-age | Prevalence | Female | United Arab Emirates | RA | 0.85<br>(0.82 to 0.89) | < 0.001 |
| Young and middle-age | Prevalence | Male | United Arab Emirates | RA | 0.9<br>(0.86 to 0.95) | < 0.001 |
| Young and middle-age | Prevalence | Both | United Republic of Tanzania | MS | 0.12<br>(0.11 to 0.14) | < 0.001 |
| Young and middle-age | Prevalence | Female | United Republic of Tanzania | MS | 0.11<br>(0.1 to 0.12) | < 0.001 |
| Young and middle-age | Prevalence | Male | United Republic of Tanzania | MS | 0.17<br>(0.14 to 0.19) | < 0.001 |
| Young and middle-age | Prevalence | Both | United Republic of Tanzania | Psoriasis | -0.42<br>(-0.45 to -0.39) | < 0.001 |
| Young and middle-age | Prevalence | Female | United Republic of Tanzania | Psoriasis | -0.43<br>(-0.44 to -0.41) | < 0.001 |
| Young and middle-age | Prevalence | Male | United Republic of Tanzania | Psoriasis | -0.43<br>(-0.49 to -0.38) | < 0.001 |
| Young and middle-age | Prevalence | Both | United Republic of Tanzania | RHD | 0.2<br>(0.17 to 0.22) | < 0.001 |

| | | | | | | |
|---|---|---|---|---|---|---|
| Young and middle-age | Prevalence | Female | United Republic of Tanzania | RHD | 0.21 (0.2 to 0.22) | < 0.001 |
| Young and middle-age | Prevalence | Male | United Republic of Tanzania | RHD | 0.18 (0.14 to 0.23) | < 0.001 |
| Young and middle-age | Prevalence | Both | United Republic of Tanzania | RA | 0.5 (0.46 to 0.53) | < 0.001 |
| Young and middle-age | Prevalence | Female | United Republic of Tanzania | RA | 0.45 (0.41 to 0.49) | < 0.001 |
| Young and middle-age | Prevalence | Male | United Republic of Tanzania | RA | 0.56 (0.52 to 0.61) | < 0.001 |
| Young and middle-age | Prevalence | Both | United States of America | MS | 0.25 (0.21 to 0.29) | < 0.001 |
| Young and middle-age | Prevalence | Female | United States of America | MS | 0.28 (0.24 to 0.33) | < 0.001 |
| Young and middle-age | Prevalence | Male | United States of America | MS | 0.2 (0.18 to 0.22) | < 0.001 |
| Young and middle-age | Prevalence | Both | United States of America | Psoriasis | -0.58 (-0.63 to -0.52) | < 0.001 |
| Young and middle-age | Prevalence | Female | United States of America | Psoriasis | -0.56 (-0.61 to -0.51) | < 0.001 |
| Young and middle-age | Prevalence | Male | United States of America | Psoriasis | -0.6 (-0.66 to -0.54) | < 0.001 |
| Young and middle-age | Prevalence | Both | United States of America | RHD | -1.09 (-1.24 to -0.94) | < 0.001 |
| Young and middle-age | Prevalence | Female | United States of America | RHD | -1.09 (-1.22 to -0.96) | < 0.001 |
| Young and middle-age | Prevalence | Male | United States of America | RHD | -1.11 (-1.21 to -1.01) | < 0.001 |
| Young and middle-age | Prevalence | Both | United States of America | RA | 0.58 (0.54 to 0.62) | < 0.001 |
| Young and middle-age | Prevalence | Female | United States of America | RA | 0.61 (0.57 to 0.65) | < 0.001 |
| Young and middle-age | Prevalence | Male | United States of America | RA | 0.51 | < 0.001 |

| | | | | | | |
|---|---|---|---|---|---|---|
| | | | | | (0.47 to 0.55) | |
| Young and middle-age | Prevalence | Both | United States Virgin Islands | MS | 0.56<br>(0.53 to 0.59) | < 0.001 |
| Young and middle-age | Prevalence | Female | United States Virgin Islands | MS | 0.6<br>(0.55 to 0.65) | < 0.001 |
| Young and middle-age | Prevalence | Male | United States Virgin Islands | MS | 0.43<br>(0.4 to 0.45) | < 0.001 |
| Young and middle-age | Prevalence | Both | United States Virgin Islands | Psoriasis | -0.53<br>(-0.54 to -0.51) | < 0.001 |
| Young and middle-age | Prevalence | Female | United States Virgin Islands | Psoriasis | -0.52<br>(-0.54 to -0.5) | < 0.001 |
| Young and middle-age | Prevalence | Male | United States Virgin Islands | Psoriasis | -0.53<br>(-0.54 to -0.51) | < 0.001 |
| Young and middle-age | Prevalence | Both | United States Virgin Islands | RHD | -0.7<br>(-0.82 to -0.58) | < 0.001 |
| Young and middle-age | Prevalence | Female | United States Virgin Islands | RHD | -0.9<br>(-1.03 to -0.77) | < 0.001 |
| Young and middle-age | Prevalence | Male | United States Virgin Islands | RHD | -0.34<br>(-0.45 to -0.24) | < 0.001 |
| Young and middle-age | Prevalence | Both | United States Virgin Islands | RA | 0.83<br>(0.8 to 0.87) | < 0.001 |
| Young and middle-age | Prevalence | Female | United States Virgin Islands | RA | 0.82<br>(0.8 to 0.84) | < 0.001 |
| Young and middle-age | Prevalence | Male | United States Virgin Islands | RA | 0.85<br>(0.81 to 0.89) | < 0.001 |
| Middle-aged and elderly age | Prevalence | Both | Afghanistan | AA | 0.01<br>(-0.02 to 0.04) | 0.669 |
| Middle-aged and elderly age | Prevalence | Female | Afghanistan | AA | 0<br>(0 to 0) | < 0.001 |
| Middle-aged and elderly age | Prevalence | Male | Afghanistan | AA | 0<br>(0 to 0) | 0.015 |
| Middle-aged and elderly age | Prevalence | Both | Afghanistan | T1D | 1.66<br>(1.58 to 1.74) | < 0.001 |

| | | | | | | |
|---|---|---|---|---|---|---|
| Middle-aged and elderly age | Prevalence | Female | Afghanistan | T1D | 1.69 (1.62 to 1.76) | < 0.001 |
| Middle-aged and elderly age | Prevalence | Male | Afghanistan | T1D | 1.63 (1.55 to 1.72) | < 0.001 |
| Middle-aged and elderly age | Prevalence | Both | Afghanistan | IBD | 0.29 (0.25 to 0.33) | < 0.001 |
| Middle-aged and elderly age | Prevalence | Female | Afghanistan | IBD | 0.25 (0.19 to 0.31) | < 0.001 |
| Middle-aged and elderly age | Prevalence | Male | Afghanistan | IBD | 0.3 (0.24 to 0.36) | < 0.001 |
| Middle-aged and elderly age | Prevalence | Both | Afghanistan | MS | 0.71 (0.68 to 0.74) | < 0.001 |
| Middle-aged and elderly age | Prevalence | Female | Afghanistan | MS | 0.66 (0.63 to 0.69) | < 0.001 |
| Middle-aged and elderly age | Prevalence | Male | Afghanistan | MS | 0.82 (0.8 to 0.84) | < 0.001 |
| Middle-aged and elderly age | Prevalence | Both | Afghanistan | Psoriasis | -0.7 (-0.73 to -0.67) | < 0.001 |
| Middle-aged and elderly age | Prevalence | Female | Afghanistan | Psoriasis | -0.74 (-0.77 to -0.7) | < 0.001 |
| Middle-aged and elderly age | Prevalence | Male | Afghanistan | Psoriasis | -0.66 (-0.71 to -0.61) | < 0.001 |
| Middle-aged and elderly age | Prevalence | Both | Afghanistan | RHD | 0.06 (0.04 to 0.07) | < 0.001 |
| Middle-aged and elderly age | Prevalence | Female | Afghanistan | RHD | 0.08 (0.06 to 0.1) | < 0.001 |
| Middle-aged and elderly age | Prevalence | Male | Afghanistan | RHD | 0.03 (-0.02 to 0.07) | 0.244 |
| Middle-aged and elderly age | Prevalence | Both | Afghanistan | RA | 0.6 (0.52 to 0.67) | < 0.001 |
| Middle-aged and elderly age | Prevalence | Female | Afghanistan | RA | 0.59 (0.57 to 0.6) | < 0.001 |
| Middle-aged and elderly age | Prevalence | Male | Afghanistan | RA | 0.57 | < 0.001 |

| | | | | | | |
|---|---|---|---|---|---|---|
| | | | | | (0.52 to 0.61) | |
| Middle-aged and elderly age | Prevalence | Both | Albania | AA | 0.13<br>(0.12 to 0.13) | < 0.001 |
| Middle-aged and elderly age | Prevalence | Female | Albania | AA | 0<br>(0 to 0) | < 0.001 |
| Middle-aged and elderly age | Prevalence | Male | Albania | AA | 0<br>(0 to 0) | < 0.001 |
| Middle-aged and elderly age | Prevalence | Both | Albania | T1D | 1.17<br>(1.15 to 1.19) | < 0.001 |
| Middle-aged and elderly age | Prevalence | Female | Albania | T1D | 1.2<br>(1.18 to 1.23) | < 0.001 |
| Middle-aged and elderly age | Prevalence | Male | Albania | T1D | 1.2<br>(1.16 to 1.23) | < 0.001 |
| Middle-aged and elderly age | Prevalence | Both | Albania | IBD | -0.12<br>(-0.2 to -0.05) | 0.002 |
| Middle-aged and elderly age | Prevalence | Female | Albania | IBD | -0.05<br>(-0.11 to 0.01) | 0.079 |
| Middle-aged and elderly age | Prevalence | Male | Albania | IBD | -0.13<br>(-0.23 to -0.02) | 0.016 |
| Middle-aged and elderly age | Prevalence | Both | Albania | MS | -1.05<br>(-1.38 to -0.73) | < 0.001 |
| Middle-aged and elderly age | Prevalence | Female | Albania | MS | -1<br>(-1.43 to -0.56) | < 0.001 |
| Middle-aged and elderly age | Prevalence | Male | Albania | MS | -1.09<br>(-1.29 to -0.88) | < 0.001 |
| Middle-aged and elderly age | Prevalence | Both | Albania | Psoriasis | -0.91<br>(-0.94 to -0.88) | < 0.001 |
| Middle-aged and elderly age | Prevalence | Female | Albania | Psoriasis | -0.94<br>(-0.98 to -0.9) | < 0.001 |
| Middle-aged and elderly age | Prevalence | Male | Albania | Psoriasis | -0.89<br>(-0.93 to -0.86) | < 0.001 |
| Middle-aged and elderly age | Prevalence | Both | Albania | RHD | 0.1<br>(0.07 to 0.12) | < 0.001 |

| | | | | | | |
|---|---|---|---|---|---|---|
| Middle-aged and elderly age | Prevalence | Female | Albania | RHD | 0.1 (0.08 to 0.13) | < 0.001 |
| Middle-aged and elderly age | Prevalence | Male | Albania | RHD | -0.01 (-0.05 to 0.03) | 0.573 |
| Middle-aged and elderly age | Prevalence | Both | Albania | RA | 1.29 (1.18 to 1.41) | < 0.001 |
| Middle-aged and elderly age | Prevalence | Female | Albania | RA | 1.17 (1.05 to 1.3) | < 0.001 |
| Middle-aged and elderly age | Prevalence | Male | Albania | RA | 1.18 (1.08 to 1.29) | < 0.001 |
| Middle-aged and elderly age | Prevalence | Both | Algeria | AA | -0.05 (-0.06 to -0.04) | < 0.001 |
| Middle-aged and elderly age | Prevalence | Female | Algeria | AA | 0 (0 to 0) | < 0.001 |
| Middle-aged and elderly age | Prevalence | Male | Algeria | AA | 0 (0 to 0) | 0.015 |
| Middle-aged and elderly age | Prevalence | Both | Algeria | T1D | 2.55 (2.5 to 2.6) | < 0.001 |
| Middle-aged and elderly age | Prevalence | Female | Algeria | T1D | 2.48 (2.44 to 2.53) | < 0.001 |
| Middle-aged and elderly age | Prevalence | Male | Algeria | T1D | 2.62 (2.56 to 2.69) | < 0.001 |
| Middle-aged and elderly age | Prevalence | Both | Algeria | IBD | 0.7 (0.44 to 0.96) | < 0.001 |
| Middle-aged and elderly age | Prevalence | Female | Algeria | IBD | 0.68 (0.48 to 0.88) | < 0.001 |
| Middle-aged and elderly age | Prevalence | Male | Algeria | IBD | 0.64 (0.5 to 0.79) | < 0.001 |
| Middle-aged and elderly age | Prevalence | Both | Algeria | MS | 0.78 (0.77 to 0.8) | < 0.001 |
| Middle-aged and elderly age | Prevalence | Female | Algeria | MS | 0.83 (0.82 to 0.84) | < 0.001 |
| Middle-aged and elderly age | Prevalence | Male | Algeria | MS | 0.83 | < 0.001 |

| | | | | | | |
|---|---|---|---|---|---|---|
| | | | | | (0.8 to 0.85) | |
| Middle-aged and elderly age | Prevalence | Both | Algeria | Psoriasis | -0.88<br>(-0.89 to -0.87) | < 0.001 |
| Middle-aged and elderly age | Prevalence | Female | Algeria | Psoriasis | -0.88<br>(-0.89 to -0.88) | < 0.001 |
| Middle-aged and elderly age | Prevalence | Male | Algeria | Psoriasis | -0.87<br>(-0.88 to -0.85) | < 0.001 |
| Middle-aged and elderly age | Prevalence | Both | Algeria | RHD | 0.15<br>(0.12 to 0.18) | < 0.001 |
| Middle-aged and elderly age | Prevalence | Female | Algeria | RHD | 0.2<br>(0.16 to 0.23) | < 0.001 |
| Middle-aged and elderly age | Prevalence | Male | Algeria | RHD | 0.14<br>(0.09 to 0.19) | < 0.001 |
| Middle-aged and elderly age | Prevalence | Both | Algeria | RA | 0.97<br>(0.89 to 1.05) | < 0.001 |
| Middle-aged and elderly age | Prevalence | Female | Algeria | RA | 1.05<br>(1.01 to 1.1) | < 0.001 |
| Middle-aged and elderly age | Prevalence | Male | Algeria | RA | 0.9<br>(0.85 to 0.95) | < 0.001 |
| Middle-aged and elderly age | Prevalence | Both | American Samoa | AA | 0.11<br>(0.11 to 0.12) | < 0.001 |
| Middle-aged and elderly age | Prevalence | Female | American Samoa | AA | 0<br>(0 to 0) | < 0.001 |
| Middle-aged and elderly age | Prevalence | Male | American Samoa | AA | 0<br>(0 to 0) | < 0.001 |
| Middle-aged and elderly age | Prevalence | Both | American Samoa | T1D | 0.83<br>(0.82 to 0.84) | < 0.001 |
| Middle-aged and elderly age | Prevalence | Female | American Samoa | T1D | 0.83<br>(0.82 to 0.85) | < 0.001 |
| Middle-aged and elderly age | Prevalence | Male | American Samoa | T1D | 0.88<br>(0.87 to 0.9) | < 0.001 |
| Middle-aged and elderly age | Prevalence | Both | American Samoa | IBD | -0.16<br>(-0.28 to -0.04) | 0.012 |

| | | | | | | |
|---|---|---|---|---|---|---|
| Middle-aged and elderly age | Prevalence | Female | American Samoa | IBD | -0.14<br>(-0.28 to -0.01) | 0.040 |
| Middle-aged and elderly age | Prevalence | Male | American Samoa | IBD | -0.17<br>(-0.34 to 0.01) | 0.063 |
| Middle-aged and elderly age | Prevalence | Both | American Samoa | MS | 0.21<br>(0.17 to 0.25) | < 0.001 |
| Middle-aged and elderly age | Prevalence | Female | American Samoa | MS | 0.1<br>(0.07 to 0.13) | < 0.001 |
| Middle-aged and elderly age | Prevalence | Male | American Samoa | MS | 0.12<br>(0.09 to 0.15) | < 0.001 |
| Middle-aged and elderly age | Prevalence | Both | American Samoa | Psoriasis | -0.49<br>(-0.62 to -0.36) | < 0.001 |
| Middle-aged and elderly age | Prevalence | Female | American Samoa | Psoriasis | -0.41<br>(-0.54 to -0.28) | < 0.001 |
| Middle-aged and elderly age | Prevalence | Male | American Samoa | Psoriasis | -0.42<br>(-0.56 to -0.28) | < 0.001 |
| Middle-aged and elderly age | Prevalence | Both | American Samoa | RHD | 0.11<br>(0.09 to 0.13) | < 0.001 |
| Middle-aged and elderly age | Prevalence | Female | American Samoa | RHD | 0.07<br>(0.04 to 0.1) | < 0.001 |
| Middle-aged and elderly age | Prevalence | Male | American Samoa | RHD | 0.06<br>(0.02 to 0.1) | 0.004 |
| Middle-aged and elderly age | Prevalence | Both | American Samoa | RA | 0.37<br>(0.3 to 0.44) | < 0.001 |
| Middle-aged and elderly age | Prevalence | Female | American Samoa | RA | 0.26<br>(0.21 to 0.3) | < 0.001 |
| Middle-aged and elderly age | Prevalence | Male | American Samoa | RA | 0.21<br>(0.14 to 0.28) | < 0.001 |
| Middle-aged and elderly age | Prevalence | Both | Andorra | AA | 0.01<br>(-0.09 to 0.12) | 0.789 |
| Middle-aged and elderly age | Prevalence | Female | Andorra | AA | 0<br>(0 to 0) | < 0.001 |
| Middle-aged and elderly age | Prevalence | Male | Andorra | AA | 0 | < 0.001 |

| | | | | | | |
|---|---|---|---|---|---|---|
| | | | | | (0 to 0) | |
| Middle-aged and elderly age | Prevalence | Both | Andorra | T1D | 1.9<br>(1.86 to 1.95) | < 0.001 |
| Middle-aged and elderly age | Prevalence | Female | Andorra | T1D | 1.89<br>(1.77 to 2.02) | < 0.001 |
| Middle-aged and elderly age | Prevalence | Male | Andorra | T1D | 1.92<br>(1.81 to 2.03) | < 0.001 |
| Middle-aged and elderly age | Prevalence | Both | Andorra | IBD | 0.09<br>(0.08 to 0.11) | < 0.001 |
| Middle-aged and elderly age | Prevalence | Female | Andorra | IBD | 0.15<br>(0.12 to 0.18) | < 0.001 |
| Middle-aged and elderly age | Prevalence | Male | Andorra | IBD | 0.05<br>(0.04 to 0.05) | < 0.001 |
| Middle-aged and elderly age | Prevalence | Both | Andorra | MS | 0.52<br>(0.48 to 0.55) | < 0.001 |
| Middle-aged and elderly age | Prevalence | Female | Andorra | MS | 0.58<br>(0.54 to 0.61) | < 0.001 |
| Middle-aged and elderly age | Prevalence | Male | Andorra | MS | 0.36<br>(0.36 to 0.37) | < 0.001 |
| Middle-aged and elderly age | Prevalence | Both | Andorra | Psoriasis | -0.25<br>(-0.27 to -0.24) | < 0.001 |
| Middle-aged and elderly age | Prevalence | Female | Andorra | Psoriasis | -0.25<br>(-0.27 to -0.23) | < 0.001 |
| Middle-aged and elderly age | Prevalence | Male | Andorra | Psoriasis | -0.25<br>(-0.25 to -0.24) | < 0.001 |
| Middle-aged and elderly age | Prevalence | Both | Andorra | RHD | -0.75<br>(-0.84 to -0.67) | < 0.001 |
| Middle-aged and elderly age | Prevalence | Female | Andorra | RHD | -1.08<br>(-1.14 to -1.02) | < 0.001 |
| Middle-aged and elderly age | Prevalence | Male | Andorra | RHD | -0.28<br>(-0.37 to -0.2) | < 0.001 |
| Middle-aged and elderly age | Prevalence | Both | Andorra | RA | 0.34<br>(0.24 to 0.45) | < 0.001 |

| | | | | | | |
|---|---|---|---|---|---|---|
| Middle-aged and elderly age | Prevalence | Female | Andorra | RA | 0.3<br>(0.24 to 0.36) | < 0.001 |
| Middle-aged and elderly age | Prevalence | Male | Andorra | RA | 0.36<br>(0.32 to 0.41) | < 0.001 |
| Middle-aged and elderly age | Prevalence | Both | Angola | AA | 0.11<br>(0.1 to 0.11) | < 0.001 |
| Middle-aged and elderly age | Prevalence | Female | Angola | AA | 0<br>(0 to 0) | 0.027 |
| Middle-aged and elderly age | Prevalence | Male | Angola | AA | 0<br>(0 to 0) | < 0.001 |
| Middle-aged and elderly age | Prevalence | Both | Angola | T1D | 0.54<br>(0.52 to 0.56) | < 0.001 |
| Middle-aged and elderly age | Prevalence | Female | Angola | T1D | 0.55<br>(0.52 to 0.58) | < 0.001 |
| Middle-aged and elderly age | Prevalence | Male | Angola | T1D | 0.54<br>(0.52 to 0.56) | < 0.001 |
| Middle-aged and elderly age | Prevalence | Both | Angola | IBD | 0.61<br>(0.56 to 0.65) | < 0.001 |
| Middle-aged and elderly age | Prevalence | Female | Angola | IBD | 0.4<br>(0.37 to 0.44) | < 0.001 |
| Middle-aged and elderly age | Prevalence | Male | Angola | IBD | 0.8<br>(0.76 to 0.83) | < 0.001 |
| Middle-aged and elderly age | Prevalence | Both | Angola | MS | 0.36<br>(0.34 to 0.39) | < 0.001 |
| Middle-aged and elderly age | Prevalence | Female | Angola | MS | 0.2<br>(0.18 to 0.22) | < 0.001 |
| Middle-aged and elderly age | Prevalence | Male | Angola | MS | 0.32<br>(0.28 to 0.36) | < 0.001 |
| Middle-aged and elderly age | Prevalence | Both | Angola | Psoriasis | -1.11<br>(-1.17 to -1.05) | < 0.001 |
| Middle-aged and elderly age | Prevalence | Female | Angola | Psoriasis | -1.12<br>(-1.17 to -1.08) | < 0.001 |
| Middle-aged and elderly age | Prevalence | Male | Angola | Psoriasis | -1.12 | < 0.001 |

| | | | | | | |
|---|---|---|---|---|---|---|
| | | | | | (-1.19 to -1.05) | |
| Middle-aged and elderly age | Prevalence | Both | Angola | RHD | 0.12<br>(0.12 to 0.13) | < 0.001 |
| Middle-aged and elderly age | Prevalence | Female | Angola | RHD | 0.14<br>(0.13 to 0.15) | < 0.001 |
| Middle-aged and elderly age | Prevalence | Male | Angola | RHD | 0.06<br>(0.05 to 0.07) | < 0.001 |
| Middle-aged and elderly age | Prevalence | Both | Angola | RA | 0.88<br>(0.77 to 0.99) | < 0.001 |
| Middle-aged and elderly age | Prevalence | Female | Angola | RA | 0.83<br>(0.74 to 0.92) | < 0.001 |
| Middle-aged and elderly age | Prevalence | Male | Angola | RA | 0.86<br>(0.74 to 0.98) | < 0.001 |
| Middle-aged and elderly age | Prevalence | Both | Antigua and Barbuda | AA | 0<br>(-0.01 to 0.01) | 0.977 |
| Middle-aged and elderly age | Prevalence | Female | Antigua and Barbuda | AA | 0<br>(0 to 0) | < 0.001 |
| Middle-aged and elderly age | Prevalence | Male | Antigua and Barbuda | AA | 0<br>(0 to 0) | < 0.001 |
| Middle-aged and elderly age | Prevalence | Both | Antigua and Barbuda | T1D | 1.25<br>(1.24 to 1.26) | < 0.001 |
| Middle-aged and elderly age | Prevalence | Female | Antigua and Barbuda | T1D | 1.17<br>(1.16 to 1.18) | < 0.001 |
| Middle-aged and elderly age | Prevalence | Male | Antigua and Barbuda | T1D | 1.32<br>(1.32 to 1.33) | < 0.001 |
| Middle-aged and elderly age | Prevalence | Both | Antigua and Barbuda | IBD | 0.22<br>(0.2 to 0.23) | < 0.001 |
| Middle-aged and elderly age | Prevalence | Female | Antigua and Barbuda | IBD | 0.23<br>(0.2 to 0.27) | < 0.001 |
| Middle-aged and elderly age | Prevalence | Male | Antigua and Barbuda | IBD | 0.21<br>(0.19 to 0.23) | < 0.001 |
| Middle-aged and elderly age | Prevalence | Both | Antigua and Barbuda | MS | 1.11<br>(1.09 to 1.13) | < 0.001 |

| | | | | | | |
|---|---|---|---|---|---|---|
| Middle-aged and elderly age | Prevalence | Female | Antigua and Barbuda | MS | 1.24 (1.2 to 1.28) | < 0.001 |
| Middle-aged and elderly age | Prevalence | Male | Antigua and Barbuda | MS | 0.33 (0.3 to 0.37) | < 0.001 |
| Middle-aged and elderly age | Prevalence | Both | Antigua and Barbuda | Psoriasis | -0.36 (-0.38 to -0.35) | < 0.001 |
| Middle-aged and elderly age | Prevalence | Female | Antigua and Barbuda | Psoriasis | -0.36 (-0.37 to -0.35) | < 0.001 |
| Middle-aged and elderly age | Prevalence | Male | Antigua and Barbuda | Psoriasis | -0.37 (-0.39 to -0.35) | < 0.001 |
| Middle-aged and elderly age | Prevalence | Both | Antigua and Barbuda | RHD | 0.05 (0.02 to 0.08) | 0.001 |
| Middle-aged and elderly age | Prevalence | Female | Antigua and Barbuda | RHD | 0.06 (0.03 to 0.09) | < 0.001 |
| Middle-aged and elderly age | Prevalence | Male | Antigua and Barbuda | RHD | 0.02 (0.01 to 0.04) | < 0.001 |
| Middle-aged and elderly age | Prevalence | Both | Antigua and Barbuda | RA | 0.88 (0.84 to 0.92) | < 0.001 |
| Middle-aged and elderly age | Prevalence | Female | Antigua and Barbuda | RA | 0.88 (0.86 to 0.89) | < 0.001 |
| Middle-aged and elderly age | Prevalence | Male | Antigua and Barbuda | RA | 0.92 (0.88 to 0.96) | < 0.001 |
| Middle-aged and elderly age | Prevalence | Both | Argentina | AA | 0 (0 to 0) | 0.512 |
| Middle-aged and elderly age | Prevalence | Female | Argentina | AA | 0 (0 to 0) | 0.000 |
| Middle-aged and elderly age | Prevalence | Male | Argentina | AA | 0 (0 to 0) | 0.002 |
| Middle-aged and elderly age | Prevalence | Both | Argentina | T1D | 1.27 (1.22 to 1.32) | < 0.001 |
| Middle-aged and elderly age | Prevalence | Female | Argentina | T1D | 1.28 (1.23 to 1.33) | < 0.001 |
| Middle-aged and elderly age | Prevalence | Male | Argentina | T1D | 1.25 | < 0.001 |

| | | | | | | |
|---|---|---|---|---|---|---|
| | | | | | (1.19 to 1.31) | |
| Middle-aged and elderly age | Prevalence | Both | Argentina | IBD | 0.25 (0.22 to 0.28) | < 0.001 |
| Middle-aged and elderly age | Prevalence | Female | Argentina | IBD | 0.28 (0.27 to 0.3) | < 0.001 |
| Middle-aged and elderly age | Prevalence | Male | Argentina | IBD | 0.21 (0.16 to 0.26) | < 0.001 |
| Middle-aged and elderly age | Prevalence | Both | Argentina | MS | 0.14 (0.11 to 0.17) | < 0.001 |
| Middle-aged and elderly age | Prevalence | Female | Argentina | MS | 0.18 (0.15 to 0.21) | < 0.001 |
| Middle-aged and elderly age | Prevalence | Male | Argentina | MS | 0.08 (0.01 to 0.15) | 0.028 |
| Middle-aged and elderly age | Prevalence | Both | Argentina | Psoriasis | -0.45 (-0.46 to -0.44) | < 0.001 |
| Middle-aged and elderly age | Prevalence | Female | Argentina | Psoriasis | -0.47 (-0.48 to -0.46) | < 0.001 |
| Middle-aged and elderly age | Prevalence | Male | Argentina | Psoriasis | -0.43 (-0.43 to -0.42) | < 0.001 |
| Middle-aged and elderly age | Prevalence | Both | Argentina | RHD | 0.15 (0.13 to 0.16) | < 0.001 |
| Middle-aged and elderly age | Prevalence | Female | Argentina | RHD | 0.14 (0.1 to 0.18) | < 0.001 |
| Middle-aged and elderly age | Prevalence | Male | Argentina | RHD | 0.14 (0.11 to 0.16) | < 0.001 |
| Middle-aged and elderly age | Prevalence | Both | Argentina | RA | 0.73 (0.64 to 0.82) | < 0.001 |
| Middle-aged and elderly age | Prevalence | Female | Argentina | RA | 0.8 (0.73 to 0.88) | < 0.001 |
| Middle-aged and elderly age | Prevalence | Male | Argentina | RA | 0.47 (0.36 to 0.59) | < 0.001 |
| Middle-aged and elderly age | Prevalence | Both | Armenia | AA | 0.05 (0.05 to 0.06) | < 0.001 |

| | | | | | | |
|---|---|---|---|---|---|---|
| Middle-aged and elderly age | Prevalence | Female | Armenia | AA | 0<br>(0 to 0) | < 0.001 |
| Middle-aged and elderly age | Prevalence | Male | Armenia | AA | 0<br>(0 to 0) | < 0.001 |
| Middle-aged and elderly age | Prevalence | Both | Armenia | T1D | 1.12<br>(1.1 to 1.14) | < 0.001 |
| Middle-aged and elderly age | Prevalence | Female | Armenia | T1D | 1.12<br>(1.1 to 1.13) | < 0.001 |
| Middle-aged and elderly age | Prevalence | Male | Armenia | T1D | 1.15<br>(1.12 to 1.18) | < 0.001 |
| Middle-aged and elderly age | Prevalence | Both | Armenia | IBD | 0.24<br>(0.17 to 0.31) | < 0.001 |
| Middle-aged and elderly age | Prevalence | Female | Armenia | IBD | 0.23<br>(0.15 to 0.31) | < 0.001 |
| Middle-aged and elderly age | Prevalence | Male | Armenia | IBD | 0.26<br>(0.18 to 0.34) | < 0.001 |
| Middle-aged and elderly age | Prevalence | Both | Armenia | MS | 0.98<br>(0.95 to 1) | < 0.001 |
| Middle-aged and elderly age | Prevalence | Female | Armenia | MS | 1.17<br>(1.12 to 1.21) | < 0.001 |
| Middle-aged and elderly age | Prevalence | Male | Armenia | MS | 0.69<br>(0.61 to 0.78) | < 0.001 |
| Middle-aged and elderly age | Prevalence | Both | Armenia | Psoriasis | -0.93<br>(-0.98 to -0.88) | < 0.001 |
| Middle-aged and elderly age | Prevalence | Female | Armenia | Psoriasis | -0.93<br>(-0.97 to -0.89) | < 0.001 |
| Middle-aged and elderly age | Prevalence | Male | Armenia | Psoriasis | -0.92<br>(-0.96 to -0.88) | < 0.001 |
| Middle-aged and elderly age | Prevalence | Both | Armenia | RHD | -0.06<br>(-0.07 to -0.04) | < 0.001 |
| Middle-aged and elderly age | Prevalence | Female | Armenia | RHD | -0.06<br>(-0.08 to -0.04) | < 0.001 |
| Middle-aged and elderly age | Prevalence | Male | Armenia | RHD | -0.11 | < 0.001 |

| | | | | | | |
|---|---|---|---|---|---|---|
| | | | | | (-0.13 to -0.1) | |
| Middle-aged and elderly age | Prevalence | Both | Armenia | RA | 1.21<br>(1.12 to 1.31) | < 0.001 |
| Middle-aged and elderly age | Prevalence | Female | Armenia | RA | 1.22<br>(1.12 to 1.31) | < 0.001 |
| Middle-aged and elderly age | Prevalence | Male | Armenia | RA | 0.99<br>(0.9 to 1.08) | < 0.001 |
| Middle-aged and elderly age | Prevalence | Both | Australia | AA | 0.06<br>(0.06 to 0.07) | < 0.001 |
| Middle-aged and elderly age | Prevalence | Female | Australia | AA | 0<br>(0 to 0) | 0.000 |
| Middle-aged and elderly age | Prevalence | Male | Australia | AA | 0<br>(0 to 0) | 0.002 |
| Middle-aged and elderly age | Prevalence | Both | Australia | T1D | 1.62<br>(1.57 to 1.67) | < 0.001 |
| Middle-aged and elderly age | Prevalence | Female | Australia | T1D | 1.5<br>(1.4 to 1.6) | < 0.001 |
| Middle-aged and elderly age | Prevalence | Male | Australia | T1D | 1.68<br>(1.57 to 1.78) | < 0.001 |
| Middle-aged and elderly age | Prevalence | Both | Australia | IBD | 1.06<br>(0.95 to 1.17) | < 0.001 |
| Middle-aged and elderly age | Prevalence | Female | Australia | IBD | 1.13<br>(1.04 to 1.22) | < 0.001 |
| Middle-aged and elderly age | Prevalence | Male | Australia | IBD | 1.02<br>(0.88 to 1.17) | < 0.001 |
| Middle-aged and elderly age | Prevalence | Both | Australia | MS | 1.38<br>(1.26 to 1.51) | < 0.001 |
| Middle-aged and elderly age | Prevalence | Female | Australia | MS | 1.39<br>(1.3 to 1.47) | < 0.001 |
| Middle-aged and elderly age | Prevalence | Male | Australia | MS | 1.25<br>(1.02 to 1.48) | < 0.001 |
| Middle-aged and elderly age | Prevalence | Both | Australia | Psoriasis | -0.47<br>(-0.5 to -0.44) | < 0.001 |

| | | | | | | |
|---|---|---|---|---|---|---|
| Middle-aged and elderly age | Prevalence | Female | Australia | Psoriasis | -0.46 (-0.5 to -0.43) | < 0.001 |
| Middle-aged and elderly age | Prevalence | Male | Australia | Psoriasis | -0.48 (-0.52 to -0.45) | < 0.001 |
| Middle-aged and elderly age | Prevalence | Both | Australia | RHD | -0.77 (-0.85 to -0.68) | < 0.001 |
| Middle-aged and elderly age | Prevalence | Female | Australia | RHD | -0.89 (-0.95 to -0.82) | < 0.001 |
| Middle-aged and elderly age | Prevalence | Male | Australia | RHD | -0.66 (-0.78 to -0.53) | < 0.001 |
| Middle-aged and elderly age | Prevalence | Both | Australia | RA | 0.55 (0.45 to 0.65) | < 0.001 |
| Middle-aged and elderly age | Prevalence | Female | Australia | RA | 0.44 (0.37 to 0.51) | < 0.001 |
| Middle-aged and elderly age | Prevalence | Male | Australia | RA | 0.55 (0.46 to 0.63) | < 0.001 |
| Middle-aged and elderly age | Prevalence | Both | Austria | AA | -0.03 (-0.03 to -0.02) | < 0.001 |
| Middle-aged and elderly age | Prevalence | Female | Austria | AA | 0 (0 to 0) | < 0.001 |
| Middle-aged and elderly age | Prevalence | Male | Austria | AA | 0 (0 to 0) | < 0.001 |
| Middle-aged and elderly age | Prevalence | Both | Austria | T1D | 2.73 (2.67 to 2.79) | < 0.001 |
| Middle-aged and elderly age | Prevalence | Female | Austria | T1D | 2.9 (2.82 to 2.97) | < 0.001 |
| Middle-aged and elderly age | Prevalence | Male | Austria | T1D | 2.61 (2.48 to 2.74) | < 0.001 |
| Middle-aged and elderly age | Prevalence | Both | Austria | IBD | 0.24 (0.07 to 0.4) | 0.006 |
| Middle-aged and elderly age | Prevalence | Female | Austria | IBD | 0.28 (0.03 to 0.53) | 0.029 |
| Middle-aged and elderly age | Prevalence | Male | Austria | IBD | 0.09 | 0.215 |

| | | | | | | |
|---|---|---|---|---|---|---|
| | | | | | (-0.05 to 0.23) | |
| Middle-aged and elderly age | Prevalence | Both | Austria | MS | 1.04 (0.98 to 1.11) | < 0.001 |
| Middle-aged and elderly age | Prevalence | Female | Austria | MS | 1.11 (1.04 to 1.19) | < 0.001 |
| Middle-aged and elderly age | Prevalence | Male | Austria | MS | 0.96 (0.89 to 1.02) | < 0.001 |
| Middle-aged and elderly age | Prevalence | Both | Austria | Psoriasis | -0.37 (-0.38 to -0.36) | < 0.001 |
| Middle-aged and elderly age | Prevalence | Female | Austria | Psoriasis | -0.37 (-0.38 to -0.36) | < 0.001 |
| Middle-aged and elderly age | Prevalence | Male | Austria | Psoriasis | -0.38 (-0.39 to -0.37) | < 0.001 |
| Middle-aged and elderly age | Prevalence | Both | Austria | RHD | -3.33 (-3.69 to -2.97) | < 0.001 |
| Middle-aged and elderly age | Prevalence | Female | Austria | RHD | -3.27 (-3.74 to -2.8) | < 0.001 |
| Middle-aged and elderly age | Prevalence | Male | Austria | RHD | -3.31 (-3.6 to -3.01) | < 0.001 |
| Middle-aged and elderly age | Prevalence | Both | Austria | RA | 0.21 (0.15 to 0.26) | < 0.001 |
| Middle-aged and elderly age | Prevalence | Female | Austria | RA | 0.14 (0.02 to 0.26) | 0.026 |
| Middle-aged and elderly age | Prevalence | Male | Austria | RA | 0.41 (0.36 to 0.45) | < 0.001 |
| Middle-aged and elderly age | Prevalence | Both | Azerbaijan | AA | 0.01 (0.01 to 0.01) | < 0.001 |
| Middle-aged and elderly age | Prevalence | Female | Azerbaijan | AA | 0 (0 to 0) | < 0.001 |
| Middle-aged and elderly age | Prevalence | Male | Azerbaijan | AA | 0 (0 to 0) | < 0.001 |
| Middle-aged and elderly age | Prevalence | Both | Azerbaijan | T1D | 1.05 (1.04 to 1.06) | < 0.001 |

| | | | | | | |
|---|---|---|---|---|---|---|
| Middle-aged and elderly age | Prevalence | Female | Azerbaijan | T1D | 1.03<br>(1.02 to 1.05) | < 0.001 |
| Middle-aged and elderly age | Prevalence | Male | Azerbaijan | T1D | 1.08<br>(1.06 to 1.09) | < 0.001 |
| Middle-aged and elderly age | Prevalence | Both | Azerbaijan | IBD | 0.24<br>(0.16 to 0.32) | < 0.001 |
| Middle-aged and elderly age | Prevalence | Female | Azerbaijan | IBD | 0.21<br>(0.13 to 0.3) | < 0.001 |
| Middle-aged and elderly age | Prevalence | Male | Azerbaijan | IBD | 0.25<br>(0.1 to 0.39) | 0.001 |
| Middle-aged and elderly age | Prevalence | Both | Azerbaijan | MS | 0.44<br>(0.38 to 0.51) | < 0.001 |
| Middle-aged and elderly age | Prevalence | Female | Azerbaijan | MS | 0.42<br>(0.27 to 0.57) | < 0.001 |
| Middle-aged and elderly age | Prevalence | Male | Azerbaijan | MS | 0.4<br>(0.29 to 0.5) | < 0.001 |
| Middle-aged and elderly age | Prevalence | Both | Azerbaijan | Psoriasis | -0.67<br>(-0.77 to -0.57) | < 0.001 |
| Middle-aged and elderly age | Prevalence | Female | Azerbaijan | Psoriasis | -0.69<br>(-0.76 to -0.61) | < 0.001 |
| Middle-aged and elderly age | Prevalence | Male | Azerbaijan | Psoriasis | -0.65<br>(-0.78 to -0.51) | < 0.001 |
| Middle-aged and elderly age | Prevalence | Both | Azerbaijan | RHD | 0<br>(-0.02 to 0.03) | 0.747 |
| Middle-aged and elderly age | Prevalence | Female | Azerbaijan | RHD | 0.01<br>(-0.02 to 0.05) | 0.414 |
| Middle-aged and elderly age | Prevalence | Male | Azerbaijan | RHD | -0.03<br>(-0.05 to -0.01) | 0.002 |
| Middle-aged and elderly age | Prevalence | Both | Azerbaijan | RA | 0.89<br>(0.76 to 1.02) | < 0.001 |
| Middle-aged and elderly age | Prevalence | Female | Azerbaijan | RA | 0.85<br>(0.7 to 1) | < 0.001 |
| Middle-aged and elderly age | Prevalence | Male | Azerbaijan | RA | 0.9 | < 0.001 |

| | | | | | | |
|---|---|---|---|---|---|---|
| | | | | | (0.81 to 0.99) | |
| Middle-aged and elderly age | Prevalence | Both | Bahamas | AA | 0<br>(-0.01 to 0.01) | 0.770 |
| Middle-aged and elderly age | Prevalence | Female | Bahamas | AA | 0<br>(0 to 0) | < 0.001 |
| Middle-aged and elderly age | Prevalence | Male | Bahamas | AA | 0<br>(0 to 0) | < 0.001 |
| Middle-aged and elderly age | Prevalence | Both | Bahamas | T1D | 1.43<br>(1.4 to 1.46) | < 0.001 |
| Middle-aged and elderly age | Prevalence | Female | Bahamas | T1D | 1.35<br>(1.3 to 1.4) | < 0.001 |
| Middle-aged and elderly age | Prevalence | Male | Bahamas | T1D | 1.5<br>(1.49 to 1.52) | < 0.001 |
| Middle-aged and elderly age | Prevalence | Both | Bahamas | IBD | -0.2<br>(-0.25 to -0.15) | < 0.001 |
| Middle-aged and elderly age | Prevalence | Female | Bahamas | IBD | -0.11<br>(-0.15 to -0.06) | < 0.001 |
| Middle-aged and elderly age | Prevalence | Male | Bahamas | IBD | -0.29<br>(-0.36 to -0.21) | < 0.001 |
| Middle-aged and elderly age | Prevalence | Both | Bahamas | MS | 0.58<br>(0.55 to 0.61) | < 0.001 |
| Middle-aged and elderly age | Prevalence | Female | Bahamas | MS | 0.63<br>(0.58 to 0.68) | < 0.001 |
| Middle-aged and elderly age | Prevalence | Male | Bahamas | MS | 0.48<br>(0.43 to 0.53) | < 0.001 |
| Middle-aged and elderly age | Prevalence | Both | Bahamas | Psoriasis | -0.25<br>(-0.3 to -0.19) | < 0.001 |
| Middle-aged and elderly age | Prevalence | Female | Bahamas | Psoriasis | -0.25<br>(-0.31 to -0.19) | < 0.001 |
| Middle-aged and elderly age | Prevalence | Male | Bahamas | Psoriasis | -0.24<br>(-0.29 to -0.2) | < 0.001 |
| Middle-aged and elderly age | Prevalence | Both | Bahamas | RHD | 0.02<br>(-0.01 to 0.05) | 0.111 |

| | | | | | | |
|---|---|---|---|---|---|---|
| Middle-aged and elderly age | Prevalence | Female | Bahamas | RHD | 0.04 (0 to 0.09) | 0.069 |
| Middle-aged and elderly age | Prevalence | Male | Bahamas | RHD | -0.02 (-0.03 to 0) | 0.037 |
| Middle-aged and elderly age | Prevalence | Both | Bahamas | RA | 0.61 (0.57 to 0.64) | < 0.001 |
| Middle-aged and elderly age | Prevalence | Female | Bahamas | RA | 0.56 (0.5 to 0.62) | < 0.001 |
| Middle-aged and elderly age | Prevalence | Male | Bahamas | RA | 0.79 (0.69 to 0.89) | < 0.001 |
| Middle-aged and elderly age | Prevalence | Both | Bahrain | AA | -0.17 (-0.24 to -0.1) | < 0.001 |
| Middle-aged and elderly age | Prevalence | Female | Bahrain | AA | 0 (0 to 0) | < 0.001 |
| Middle-aged and elderly age | Prevalence | Male | Bahrain | AA | 0 (0 to 0) | 0.015 |
| Middle-aged and elderly age | Prevalence | Both | Bahrain | T1D | 2.41 (2.37 to 2.45) | < 0.001 |
| Middle-aged and elderly age | Prevalence | Female | Bahrain | T1D | 2.36 (2.27 to 2.45) | < 0.001 |
| Middle-aged and elderly age | Prevalence | Male | Bahrain | T1D | 2.39 (2.34 to 2.43) | < 0.001 |
| Middle-aged and elderly age | Prevalence | Both | Bahrain | IBD | 0.45 (0.32 to 0.59) | < 0.001 |
| Middle-aged and elderly age | Prevalence | Female | Bahrain | IBD | 0.43 (0.28 to 0.58) | < 0.001 |
| Middle-aged and elderly age | Prevalence | Male | Bahrain | IBD | 0.46 (0.32 to 0.61) | < 0.001 |
| Middle-aged and elderly age | Prevalence | Both | Bahrain | MS | 0.84 (0.79 to 0.9) | < 0.001 |
| Middle-aged and elderly age | Prevalence | Female | Bahrain | MS | 0.97 (0.92 to 1.02) | < 0.001 |
| Middle-aged and elderly age | Prevalence | Male | Bahrain | MS | 1.09 | < 0.001 |

| | | | | | | |
|---|---|---|---|---|---|---|
| | | | | | (1.05 to 1.13) | |
| Middle-aged and elderly age | Prevalence | Both | Bahrain | Psoriasis | -0.73<br>(-0.75 to -0.72) | < 0.001 |
| Middle-aged and elderly age | Prevalence | Female | Bahrain | Psoriasis | -0.72<br>(-0.75 to -0.7) | < 0.001 |
| Middle-aged and elderly age | Prevalence | Male | Bahrain | Psoriasis | -0.71<br>(-0.73 to -0.69) | < 0.001 |
| Middle-aged and elderly age | Prevalence | Both | Bahrain | RHD | -1.17<br>(-1.27 to -1.06) | < 0.001 |
| Middle-aged and elderly age | Prevalence | Female | Bahrain | RHD | -1.27<br>(-1.36 to -1.19) | < 0.001 |
| Middle-aged and elderly age | Prevalence | Male | Bahrain | RHD | -0.87<br>(-1.03 to -0.71) | < 0.001 |
| Middle-aged and elderly age | Prevalence | Both | Bahrain | RA | 1.07<br>(0.98 to 1.17) | < 0.001 |
| Middle-aged and elderly age | Prevalence | Female | Bahrain | RA | 1.5<br>(1.28 to 1.71) | < 0.001 |
| Middle-aged and elderly age | Prevalence | Male | Bahrain | RA | 1.19<br>(1.14 to 1.25) | < 0.001 |
| Middle-aged and elderly age | Prevalence | Both | Bangladesh | AA | 0.09<br>(0.08 to 0.09) | < 0.001 |
| Middle-aged and elderly age | Prevalence | Female | Bangladesh | AA | 0<br>(0 to 0) | < 0.001 |
| Middle-aged and elderly age | Prevalence | Male | Bangladesh | AA | 0<br>(0 to 0) | 0.161 |
| Middle-aged and elderly age | Prevalence | Both | Bangladesh | T1D | 0.71<br>(0.68 to 0.73) | < 0.001 |
| Middle-aged and elderly age | Prevalence | Female | Bangladesh | T1D | 0.66<br>(0.62 to 0.69) | < 0.001 |
| Middle-aged and elderly age | Prevalence | Male | Bangladesh | T1D | 0.77<br>(0.75 to 0.8) | < 0.001 |
| Middle-aged and elderly age | Prevalence | Both | Bangladesh | IBD | 0.81<br>(0.73 to 0.89) | < 0.001 |

| | | | | | | |
|---|---|---|---|---|---|---|
| Middle-aged and elderly age | Prevalence | Female | Bangladesh | IBD | 0.83 (0.76 to 0.89) | < 0.001 |
| Middle-aged and elderly age | Prevalence | Male | Bangladesh | IBD | 0.85 (0.74 to 0.96) | < 0.001 |
| Middle-aged and elderly age | Prevalence | Both | Bangladesh | MS | 0.33 (0.29 to 0.36) | < 0.001 |
| Middle-aged and elderly age | Prevalence | Female | Bangladesh | MS | 0.28 (0.25 to 0.3) | < 0.001 |
| Middle-aged and elderly age | Prevalence | Male | Bangladesh | MS | 0.21 (0.15 to 0.26) | < 0.001 |
| Middle-aged and elderly age | Prevalence | Both | Bangladesh | Psoriasis | -0.65 (-0.66 to -0.64) | < 0.001 |
| Middle-aged and elderly age | Prevalence | Female | Bangladesh | Psoriasis | -0.66 (-0.68 to -0.64) | < 0.001 |
| Middle-aged and elderly age | Prevalence | Male | Bangladesh | Psoriasis | -0.66 (-0.68 to -0.65) | < 0.001 |
| Middle-aged and elderly age | Prevalence | Both | Bangladesh | RHD | 0.16 (0.1 to 0.21) | < 0.001 |
| Middle-aged and elderly age | Prevalence | Female | Bangladesh | RHD | 0.01 (-0.03 to 0.05) | 0.616 |
| Middle-aged and elderly age | Prevalence | Male | Bangladesh | RHD | 0.2 (0.13 to 0.26) | < 0.001 |
| Middle-aged and elderly age | Prevalence | Both | Bangladesh | RA | 1.36 (1.32 to 1.4) | < 0.001 |
| Middle-aged and elderly age | Prevalence | Female | Bangladesh | RA | 1.3 (1.26 to 1.34) | < 0.001 |
| Middle-aged and elderly age | Prevalence | Male | Bangladesh | RA | 1.18 (1.13 to 1.22) | < 0.001 |
| Middle-aged and elderly age | Prevalence | Both | Barbados | AA | -0.04 (-0.04 to -0.03) | < 0.001 |
| Middle-aged and elderly age | Prevalence | Female | Barbados | AA | 0 (0 to 0) | < 0.001 |
| Middle-aged and elderly age | Prevalence | Male | Barbados | AA | 0 | < 0.001 |

| | | | | | | |
|---|---|---|---|---|---|---|
| | | | | | (0 to 0) | |
| Middle-aged and elderly age | Prevalence | Both | Barbados | T1D | 1.37<br>(1.34 to 1.41) | < 0.001 |
| Middle-aged and elderly age | Prevalence | Female | Barbados | T1D | 1.22<br>(1.17 to 1.27) | < 0.001 |
| Middle-aged and elderly age | Prevalence | Male | Barbados | T1D | 1.51<br>(1.48 to 1.54) | < 0.001 |
| Middle-aged and elderly age | Prevalence | Both | Barbados | IBD | 0.1<br>(0.06 to 0.14) | < 0.001 |
| Middle-aged and elderly age | Prevalence | Female | Barbados | IBD | 0.13<br>(0.08 to 0.18) | < 0.001 |
| Middle-aged and elderly age | Prevalence | Male | Barbados | IBD | 0.07<br>(0.06 to 0.09) | < 0.001 |
| Middle-aged and elderly age | Prevalence | Both | Barbados | MS | 0.99<br>(0.96 to 1.02) | < 0.001 |
| Middle-aged and elderly age | Prevalence | Female | Barbados | MS | 1.16<br>(1.13 to 1.19) | < 0.001 |
| Middle-aged and elderly age | Prevalence | Male | Barbados | MS | 0.61<br>(0.57 to 0.66) | < 0.001 |
| Middle-aged and elderly age | Prevalence | Both | Barbados | Psoriasis | -0.21<br>(-0.24 to -0.19) | < 0.001 |
| Middle-aged and elderly age | Prevalence | Female | Barbados | Psoriasis | -0.22<br>(-0.23 to -0.2) | < 0.001 |
| Middle-aged and elderly age | Prevalence | Male | Barbados | Psoriasis | -0.2<br>(-0.25 to -0.16) | < 0.001 |
| Middle-aged and elderly age | Prevalence | Both | Barbados | RHD | 0.01<br>(-0.01 to 0.03) | 0.392 |
| Middle-aged and elderly age | Prevalence | Female | Barbados | RHD | 0.05<br>(-0.01 to 0.11) | 0.085 |
| Middle-aged and elderly age | Prevalence | Male | Barbados | RHD | -0.01<br>(-0.03 to 0.01) | 0.200 |
| Middle-aged and elderly age | Prevalence | Both | Barbados | RA | 0.72<br>(0.69 to 0.74) | < 0.001 |

| | | | | | | |
|---|---|---|---|---|---|---|
| Middle-aged and elderly age | Prevalence | Female | Barbados | RA | 0.76 (0.71 to 0.82) | < 0.001 |
| Middle-aged and elderly age | Prevalence | Male | Barbados | RA | 0.85 (0.81 to 0.88) | < 0.001 |
| Middle-aged and elderly age | Prevalence | Both | Belarus | AA | -0.01 (-0.02 to -0.01) | < 0.001 |
| Middle-aged and elderly age | Prevalence | Female | Belarus | AA | 0 (0 to 0) | < 0.001 |
| Middle-aged and elderly age | Prevalence | Male | Belarus | AA | 0 (0 to 0) | < 0.001 |
| Middle-aged and elderly age | Prevalence | Both | Belarus | T1D | 1.62 (1.56 to 1.67) | < 0.001 |
| Middle-aged and elderly age | Prevalence | Female | Belarus | T1D | 1.62 (1.59 to 1.65) | < 0.001 |
| Middle-aged and elderly age | Prevalence | Male | Belarus | T1D | 1.58 (1.53 to 1.64) | < 0.001 |
| Middle-aged and elderly age | Prevalence | Both | Belarus | IBD | 0.27 (0.25 to 0.3) | < 0.001 |
| Middle-aged and elderly age | Prevalence | Female | Belarus | IBD | 0.3 (0.28 to 0.31) | < 0.001 |
| Middle-aged and elderly age | Prevalence | Male | Belarus | IBD | 0.23 (0.18 to 0.29) | < 0.001 |
| Middle-aged and elderly age | Prevalence | Both | Belarus | MS | 0.03 (-0.03 to 0.1) | 0.347 |
| Middle-aged and elderly age | Prevalence | Female | Belarus | MS | 0.28 (0.22 to 0.34) | < 0.001 |
| Middle-aged and elderly age | Prevalence | Male | Belarus | MS | -0.31 (-0.4 to -0.22) | < 0.001 |
| Middle-aged and elderly age | Prevalence | Both | Belarus | Psoriasis | -0.83 (-0.85 to -0.81) | < 0.001 |
| Middle-aged and elderly age | Prevalence | Female | Belarus | Psoriasis | -0.82 (-0.84 to -0.8) | < 0.001 |
| Middle-aged and elderly age | Prevalence | Male | Belarus | Psoriasis | -0.84 | < 0.001 |

| | | | | | | |
|---|---|---|---|---|---|---|
| | | | | | (-0.86 to -0.82) | |
| Middle-aged and elderly age | Prevalence | Both | Belarus | RHD | -2.09<br>(-2.31 to -1.88) | < 0.001 |
| Middle-aged and elderly age | Prevalence | Female | Belarus | RHD | -2.06<br>(-2.36 to -1.77) | < 0.001 |
| Middle-aged and elderly age | Prevalence | Male | Belarus | RHD | -2.11<br>(-2.44 to -1.78) | < 0.001 |
| Middle-aged and elderly age | Prevalence | Both | Belarus | RA | 0.99<br>(0.89 to 1.09) | < 0.001 |
| Middle-aged and elderly age | Prevalence | Female | Belarus | RA | 1.05<br>(0.96 to 1.13) | < 0.001 |
| Middle-aged and elderly age | Prevalence | Male | Belarus | RA | 0.82<br>(0.74 to 0.91) | < 0.001 |
| Middle-aged and elderly age | Prevalence | Both | Belgium | AA | 0<br>(-0.01 to 0) | 0.004 |
| Middle-aged and elderly age | Prevalence | Female | Belgium | AA | 0<br>(0 to 0) | < 0.001 |
| Middle-aged and elderly age | Prevalence | Male | Belgium | AA | 0<br>(0 to 0) | < 0.001 |
| Middle-aged and elderly age | Prevalence | Both | Belgium | T1D | 2.06<br>(1.93 to 2.2) | < 0.001 |
| Middle-aged and elderly age | Prevalence | Female | Belgium | T1D | 1.96<br>(1.76 to 2.16) | < 0.001 |
| Middle-aged and elderly age | Prevalence | Male | Belgium | T1D | 2.15<br>(1.98 to 2.33) | < 0.001 |
| Middle-aged and elderly age | Prevalence | Both | Belgium | IBD | 2.36<br>(2 to 2.72) | < 0.001 |
| Middle-aged and elderly age | Prevalence | Female | Belgium | IBD | 2.52<br>(2.09 to 2.94) | < 0.001 |
| Middle-aged and elderly age | Prevalence | Male | Belgium | IBD | 2.19<br>(1.91 to 2.47) | < 0.001 |
| Middle-aged and elderly age | Prevalence | Both | Belgium | MS | 0.4<br>(0.37 to 0.43) | < 0.001 |

| | | | | | | |
|---|---|---|---|---|---|---|
| Middle-aged and elderly age | Prevalence | Female | Belgium | MS | 0.61 (0.57 to 0.64) | < 0.001 |
| Middle-aged and elderly age | Prevalence | Male | Belgium | MS | 0.1 (0.04 to 0.16) | 0.001 |
| Middle-aged and elderly age | Prevalence | Both | Belgium | Psoriasis | -0.41 (-0.43 to -0.39) | < 0.001 |
| Middle-aged and elderly age | Prevalence | Female | Belgium | Psoriasis | -0.41 (-0.43 to -0.38) | < 0.001 |
| Middle-aged and elderly age | Prevalence | Male | Belgium | Psoriasis | -0.42 (-0.43 to -0.4) | < 0.001 |
| Middle-aged and elderly age | Prevalence | Both | Belgium | RHD | -0.07 (-0.92 to 0.8) | 0.881 |
| Middle-aged and elderly age | Prevalence | Female | Belgium | RHD | 0.1 (-0.4 to 0.6) | 0.698 |
| Middle-aged and elderly age | Prevalence | Male | Belgium | RHD | 0.31 (-1.39 to 2.04) | 0.722 |
| Middle-aged and elderly age | Prevalence | Both | Belgium | RA | 0.46 (0.43 to 0.48) | < 0.001 |
| Middle-aged and elderly age | Prevalence | Female | Belgium | RA | 0.47 (0.43 to 0.5) | < 0.001 |
| Middle-aged and elderly age | Prevalence | Male | Belgium | RA | 0.45 (0.42 to 0.48) | < 0.001 |
| Middle-aged and elderly age | Prevalence | Both | Belize | AA | 0.05 (0.05 to 0.05) | < 0.001 |
| Middle-aged and elderly age | Prevalence | Female | Belize | AA | 0 (0 to 0) | < 0.001 |
| Middle-aged and elderly age | Prevalence | Male | Belize | AA | 0 (0 to 0) | < 0.001 |
| Middle-aged and elderly age | Prevalence | Both | Belize | T1D | 1.1 (1.08 to 1.11) | < 0.001 |
| Middle-aged and elderly age | Prevalence | Female | Belize | T1D | 1.05 (1.01 to 1.09) | < 0.001 |
| Middle-aged and elderly age | Prevalence | Male | Belize | T1D | 1.16 | < 0.001 |

| | | | | | | |
|---|---|---|---|---|---|---|
| | | | | | (1.15 to 1.17) | |
| Middle-aged and elderly age | Prevalence | Both | Belize | IBD | 0.33 (0.31 to 0.35) | < 0.001 |
| Middle-aged and elderly age | Prevalence | Female | Belize | IBD | 0.33 (0.27 to 0.39) | < 0.001 |
| Middle-aged and elderly age | Prevalence | Male | Belize | IBD | 0.28 (0.23 to 0.33) | < 0.001 |
| Middle-aged and elderly age | Prevalence | Both | Belize | MS | 0.58 (0.53 to 0.63) | < 0.001 |
| Middle-aged and elderly age | Prevalence | Female | Belize | MS | 0.58 (0.51 to 0.64) | < 0.001 |
| Middle-aged and elderly age | Prevalence | Male | Belize | MS | 0.42 (0.41 to 0.44) | < 0.001 |
| Middle-aged and elderly age | Prevalence | Both | Belize | Psoriasis | -0.64 (-0.66 to -0.62) | < 0.001 |
| Middle-aged and elderly age | Prevalence | Female | Belize | Psoriasis | -0.64 (-0.66 to -0.62) | < 0.001 |
| Middle-aged and elderly age | Prevalence | Male | Belize | Psoriasis | -0.65 (-0.66 to -0.63) | < 0.001 |
| Middle-aged and elderly age | Prevalence | Both | Belize | RHD | 0.11 (0.1 to 0.11) | < 0.001 |
| Middle-aged and elderly age | Prevalence | Female | Belize | RHD | 0.12 (0.1 to 0.14) | < 0.001 |
| Middle-aged and elderly age | Prevalence | Male | Belize | RHD | 0.05 (0.04 to 0.06) | < 0.001 |
| Middle-aged and elderly age | Prevalence | Both | Belize | RA | 0.88 (0.77 to 0.98) | < 0.001 |
| Middle-aged and elderly age | Prevalence | Female | Belize | RA | 0.75 (0.67 to 0.83) | < 0.001 |
| Middle-aged and elderly age | Prevalence | Male | Belize | RA | 0.96 (0.85 to 1.07) | < 0.001 |
| Middle-aged and elderly age | Prevalence | Both | Benin | AA | -0.05 (-0.06 to -0.03) | < 0.001 |

| | | | | | | |
|---|---|---|---|---|---|---|
| Middle-aged and elderly age | Prevalence | Female | Benin | AA | 0<br>(0 to 0) | 0.027 |
| Middle-aged and elderly age | Prevalence | Male | Benin | AA | 0<br>(0 to 0) | < 0.001 |
| Middle-aged and elderly age | Prevalence | Both | Benin | T1D | 0.28<br>(0.27 to 0.3) | < 0.001 |
| Middle-aged and elderly age | Prevalence | Female | Benin | T1D | 0.28<br>(0.27 to 0.28) | < 0.001 |
| Middle-aged and elderly age | Prevalence | Male | Benin | T1D | 0.28<br>(0.27 to 0.29) | < 0.001 |
| Middle-aged and elderly age | Prevalence | Both | Benin | IBD | 0.51<br>(0.41 to 0.61) | < 0.001 |
| Middle-aged and elderly age | Prevalence | Female | Benin | IBD | 0.44<br>(0.32 to 0.56) | < 0.001 |
| Middle-aged and elderly age | Prevalence | Male | Benin | IBD | 0.59<br>(0.52 to 0.66) | < 0.001 |
| Middle-aged and elderly age | Prevalence | Both | Benin | MS | 0.16<br>(0.12 to 0.19) | < 0.001 |
| Middle-aged and elderly age | Prevalence | Female | Benin | MS | 0.2<br>(0.17 to 0.24) | < 0.001 |
| Middle-aged and elderly age | Prevalence | Male | Benin | MS | 0.25<br>(0.19 to 0.31) | < 0.001 |
| Middle-aged and elderly age | Prevalence | Both | Benin | Psoriasis | -0.64<br>(-0.8 to -0.47) | < 0.001 |
| Middle-aged and elderly age | Prevalence | Female | Benin | Psoriasis | -0.69<br>(-0.82 to -0.56) | < 0.001 |
| Middle-aged and elderly age | Prevalence | Male | Benin | Psoriasis | -0.55<br>(-0.69 to -0.4) | < 0.001 |
| Middle-aged and elderly age | Prevalence | Both | Benin | RHD | 0.13<br>(0.1 to 0.15) | < 0.001 |
| Middle-aged and elderly age | Prevalence | Female | Benin | RHD | 0.16<br>(0.15 to 0.18) | < 0.001 |
| Middle-aged and elderly age | Prevalence | Male | Benin | RHD | 0.12 | < 0.001 |

| | | | | | | |
|---|---|---|---|---|---|---|
| | | | | | (0.11 to 0.13) | |
| Middle-aged and elderly age | Prevalence | Both | Benin | RA | 0.61<br>(0.58 to 0.64) | < 0.001 |
| Middle-aged and elderly age | Prevalence | Female | Benin | RA | 0.6<br>(0.59 to 0.62) | < 0.001 |
| Middle-aged and elderly age | Prevalence | Male | Benin | RA | 0.68<br>(0.65 to 0.71) | < 0.001 |
| Middle-aged and elderly age | Prevalence | Both | Bermuda | AA | -0.03<br>(-0.04 to -0.02) | < 0.001 |
| Middle-aged and elderly age | Prevalence | Female | Bermuda | AA | 0<br>(0 to 0) | < 0.001 |
| Middle-aged and elderly age | Prevalence | Male | Bermuda | AA | 0<br>(0 to 0) | < 0.001 |
| Middle-aged and elderly age | Prevalence | Both | Bermuda | T1D | 1.85<br>(1.84 to 1.86) | < 0.001 |
| Middle-aged and elderly age | Prevalence | Female | Bermuda | T1D | 1.75<br>(1.74 to 1.75) | < 0.001 |
| Middle-aged and elderly age | Prevalence | Male | Bermuda | T1D | 1.93<br>(1.92 to 1.94) | < 0.001 |
| Middle-aged and elderly age | Prevalence | Both | Bermuda | IBD | 0.11<br>(0.1 to 0.12) | < 0.001 |
| Middle-aged and elderly age | Prevalence | Female | Bermuda | IBD | 0.16<br>(0.13 to 0.19) | < 0.001 |
| Middle-aged and elderly age | Prevalence | Male | Bermuda | IBD | 0.04<br>(0 to 0.09) | 0.049 |
| Middle-aged and elderly age | Prevalence | Both | Bermuda | MS | 0.42<br>(0.38 to 0.45) | < 0.001 |
| Middle-aged and elderly age | Prevalence | Female | Bermuda | MS | 0.47<br>(0.42 to 0.52) | < 0.001 |
| Middle-aged and elderly age | Prevalence | Male | Bermuda | MS | 0.4<br>(0.39 to 0.42) | < 0.001 |
| Middle-aged and elderly age | Prevalence | Both | Bermuda | Psoriasis | -0.36<br>(-0.38 to -0.34) | < 0.001 |

| | | | | | | |
|---|---|---|---|---|---|---|
| Middle-aged and elderly age | Prevalence | Female | Bermuda | Psoriasis | -0.35 (-0.37 to -0.33) | < 0.001 |
| Middle-aged and elderly age | Prevalence | Male | Bermuda | Psoriasis | -0.37 (-0.39 to -0.35) | < 0.001 |
| Middle-aged and elderly age | Prevalence | Both | Bermuda | RHD | -1.58 (-1.66 to -1.5) | < 0.001 |
| Middle-aged and elderly age | Prevalence | Female | Bermuda | RHD | -1.55 (-1.63 to -1.46) | < 0.001 |
| Middle-aged and elderly age | Prevalence | Male | Bermuda | RHD | -1.52 (-1.69 to -1.35) | < 0.001 |
| Middle-aged and elderly age | Prevalence | Both | Bermuda | RA | 1.01 (0.98 to 1.05) | < 0.001 |
| Middle-aged and elderly age | Prevalence | Female | Bermuda | RA | 1.04 (1 to 1.09) | < 0.001 |
| Middle-aged and elderly age | Prevalence | Male | Bermuda | RA | 1.07 (1 to 1.15) | < 0.001 |
| Middle-aged and elderly age | Prevalence | Both | Bhutan | AA | 0.01 (0.01 to 0.01) | < 0.001 |
| Middle-aged and elderly age | Prevalence | Female | Bhutan | AA | 0 (0 to 0) | < 0.001 |
| Middle-aged and elderly age | Prevalence | Male | Bhutan | AA | 0 (0 to 0) | 0.161 |
| Middle-aged and elderly age | Prevalence | Both | Bhutan | T1D | 0.64 (0.61 to 0.66) | < 0.001 |
| Middle-aged and elderly age | Prevalence | Female | Bhutan | T1D | 0.59 (0.57 to 0.61) | < 0.001 |
| Middle-aged and elderly age | Prevalence | Male | Bhutan | T1D | 0.68 (0.67 to 0.69) | < 0.001 |
| Middle-aged and elderly age | Prevalence | Both | Bhutan | IBD | 0.99 (0.93 to 1.04) | < 0.001 |
| Middle-aged and elderly age | Prevalence | Female | Bhutan | IBD | 0.95 (0.87 to 1.03) | < 0.001 |
| Middle-aged and elderly age | Prevalence | Male | Bhutan | IBD | 1.01 | < 0.001 |

| | | | | | | |
|---|---|---|---|---|---|---|
| | | | | | (0.95 to 1.08) | |
| Middle-aged and elderly age | Prevalence | Both | Bhutan | MS | 0.44<br>(0.4 to 0.47) | < 0.001 |
| Middle-aged and elderly age | Prevalence | Female | Bhutan | MS | 0.39<br>(0.3 to 0.47) | < 0.001 |
| Middle-aged and elderly age | Prevalence | Male | Bhutan | MS | 0.45<br>(0.36 to 0.54) | < 0.001 |
| Middle-aged and elderly age | Prevalence | Both | Bhutan | Psoriasis | -0.62<br>(-0.64 to -0.61) | < 0.001 |
| Middle-aged and elderly age | Prevalence | Female | Bhutan | Psoriasis | -0.62<br>(-0.64 to -0.6) | < 0.001 |
| Middle-aged and elderly age | Prevalence | Male | Bhutan | Psoriasis | -0.62<br>(-0.63 to -0.61) | < 0.001 |
| Middle-aged and elderly age | Prevalence | Both | Bhutan | RHD | 0.11<br>(0.1 to 0.12) | < 0.001 |
| Middle-aged and elderly age | Prevalence | Female | Bhutan | RHD | 0.15<br>(0.1 to 0.19) | < 0.001 |
| Middle-aged and elderly age | Prevalence | Male | Bhutan | RHD | 0.07<br>(0.07 to 0.08) | < 0.001 |
| Middle-aged and elderly age | Prevalence | Both | Bhutan | RA | 1.45<br>(1.41 to 1.48) | < 0.001 |
| Middle-aged and elderly age | Prevalence | Female | Bhutan | RA | 1.45<br>(1.41 to 1.49) | < 0.001 |
| Middle-aged and elderly age | Prevalence | Male | Bhutan | RA | 1.41<br>(1.39 to 1.44) | < 0.001 |
| Middle-aged and elderly age | Prevalence | Both | Bolivia (Plurinational State of) | AA | -0.03<br>(-0.04 to -0.02) | < 0.001 |
| Middle-aged and elderly age | Prevalence | Female | Bolivia (Plurinational State of) | AA | 0<br>(0 to 0) | < 0.001 |
| Middle-aged and elderly age | Prevalence | Male | Bolivia (Plurinational State of) | AA | 0<br>(0 to 0) | < 0.001 |
| Middle-aged and elderly age | Prevalence | Both | Bolivia (Plurinational State of) | T1D | 0.85<br>(0.83 to 0.87) | < 0.001 |

| | | | | | | |
|---|---|---|---|---|---|---|
| Middle-aged and elderly age | Prevalence | Female | Bolivia (Plurinational State of) | T1D | 0.85<br>(0.84 to 0.87) | < 0.001 |
| Middle-aged and elderly age | Prevalence | Male | Bolivia (Plurinational State of) | T1D | 0.85<br>(0.81 to 0.89) | < 0.001 |
| Middle-aged and elderly age | Prevalence | Both | Bolivia (Plurinational State of) | IBD | -0.01<br>(-0.06 to 0.04) | 0.740 |
| Middle-aged and elderly age | Prevalence | Female | Bolivia (Plurinational State of) | IBD | 0.03<br>(-0.02 to 0.08) | 0.183 |
| Middle-aged and elderly age | Prevalence | Male | Bolivia (Plurinational State of) | IBD | -0.06<br>(-0.11 to -0.01) | 0.030 |
| Middle-aged and elderly age | Prevalence | Both | Bolivia (Plurinational State of) | MS | 0.8<br>(0.76 to 0.84) | < 0.001 |
| Middle-aged and elderly age | Prevalence | Female | Bolivia (Plurinational State of) | MS | 0.93<br>(0.87 to 0.99) | < 0.001 |
| Middle-aged and elderly age | Prevalence | Male | Bolivia (Plurinational State of) | MS | 0.63<br>(0.59 to 0.66) | < 0.001 |
| Middle-aged and elderly age | Prevalence | Both | Bolivia (Plurinational State of) | Psoriasis | -0.66<br>(-0.68 to -0.64) | < 0.001 |
| Middle-aged and elderly age | Prevalence | Female | Bolivia (Plurinational State of) | Psoriasis | -0.65<br>(-0.67 to -0.63) | < 0.001 |
| Middle-aged and elderly age | Prevalence | Male | Bolivia (Plurinational State of) | Psoriasis | -0.67<br>(-0.69 to -0.64) | < 0.001 |
| Middle-aged and elderly age | Prevalence | Both | Bolivia (Plurinational State of) | RHD | 0.13<br>(0.13 to 0.14) | < 0.001 |
| Middle-aged and elderly age | Prevalence | Female | Bolivia (Plurinational State of) | RHD | 0.17<br>(0.16 to 0.17) | < 0.001 |
| Middle-aged and elderly age | Prevalence | Male | Bolivia (Plurinational State of) | RHD | 0.11<br>(0.1 to 0.11) | < 0.001 |
| Middle-aged and elderly age | Prevalence | Both | Bolivia (Plurinational State of) | RA | 1.42<br>(1.39 to 1.45) | < 0.001 |
| Middle-aged and elderly age | Prevalence | Female | Bolivia (Plurinational State of) | RA | 1.49<br>(1.43 to 1.54) | < 0.001 |
| Middle-aged and elderly age | Prevalence | Male | Bolivia (Plurinational State of) | RA | 1.39 | < 0.001 |

| | | | | | | |
|---|---|---|---|---|---|---|
| | | | | | (1.35 to 1.44) | |
| Middle-aged and elderly age | Prevalence | Both | Bosnia and Herzegovina | AA | 0<br>(-0.01 to 0) | 0.161 |
| Middle-aged and elderly age | Prevalence | Female | Bosnia and Herzegovina | AA | 0<br>(0 to 0) | < 0.001 |
| Middle-aged and elderly age | Prevalence | Male | Bosnia and Herzegovina | AA | 0<br>(0 to 0) | < 0.001 |
| Middle-aged and elderly age | Prevalence | Both | Bosnia and Herzegovina | T1D | 1.99<br>(1.93 to 2.04) | < 0.001 |
| Middle-aged and elderly age | Prevalence | Female | Bosnia and Herzegovina | T1D | 1.96<br>(1.9 to 2.01) | < 0.001 |
| Middle-aged and elderly age | Prevalence | Male | Bosnia and Herzegovina | T1D | 2<br>(1.95 to 2.05) | < 0.001 |
| Middle-aged and elderly age | Prevalence | Both | Bosnia and Herzegovina | IBD | 0.61<br>(0.57 to 0.65) | < 0.001 |
| Middle-aged and elderly age | Prevalence | Female | Bosnia and Herzegovina | IBD | 0.65<br>(0.57 to 0.73) | < 0.001 |
| Middle-aged and elderly age | Prevalence | Male | Bosnia and Herzegovina | IBD | 0.57<br>(0.5 to 0.65) | < 0.001 |
| Middle-aged and elderly age | Prevalence | Both | Bosnia and Herzegovina | MS | 0.26<br>(0.26 to 0.27) | < 0.001 |
| Middle-aged and elderly age | Prevalence | Female | Bosnia and Herzegovina | MS | 0.46<br>(0.42 to 0.5) | < 0.001 |
| Middle-aged and elderly age | Prevalence | Male | Bosnia and Herzegovina | MS | 0.01<br>(-0.02 to 0.05) | 0.452 |
| Middle-aged and elderly age | Prevalence | Both | Bosnia and Herzegovina | Psoriasis | -1.08<br>(-1.13 to -1.02) | < 0.001 |
| Middle-aged and elderly age | Prevalence | Female | Bosnia and Herzegovina | Psoriasis | -1.09<br>(-1.17 to -1.01) | < 0.001 |
| Middle-aged and elderly age | Prevalence | Male | Bosnia and Herzegovina | Psoriasis | -1.07<br>(-1.09 to -1.04) | < 0.001 |
| Middle-aged and elderly age | Prevalence | Both | Bosnia and Herzegovina | RHD | -0.33<br>(-0.57 to -0.1) | 0.005 |

| | | | | | | |
|---|---|---|---|---|---|---|
| Middle-aged and elderly age | Prevalence | Female | Bosnia and Herzegovina | RHD | -0.68<br>(-0.9 to -0.46) | < 0.001 |
| Middle-aged and elderly age | Prevalence | Male | Bosnia and Herzegovina | RHD | 0.16<br>(-0.04 to 0.35) | 0.111 |
| Middle-aged and elderly age | Prevalence | Both | Bosnia and Herzegovina | RA | 0.93<br>(0.82 to 1.04) | < 0.001 |
| Middle-aged and elderly age | Prevalence | Female | Bosnia and Herzegovina | RA | 0.99<br>(0.89 to 1.09) | < 0.001 |
| Middle-aged and elderly age | Prevalence | Male | Bosnia and Herzegovina | RA | 0.8<br>(0.62 to 0.98) | < 0.001 |
| Middle-aged and elderly age | Prevalence | Both | Botswana | AA | 0.02<br>(0.01 to 0.03) | < 0.001 |
| Middle-aged and elderly age | Prevalence | Female | Botswana | AA | 0<br>(0 to 0) | 0.027 |
| Middle-aged and elderly age | Prevalence | Male | Botswana | AA | 0<br>(0 to 0) | < 0.001 |
| Middle-aged and elderly age | Prevalence | Both | Botswana | T1D | 0.53<br>(0.51 to 0.56) | < 0.001 |
| Middle-aged and elderly age | Prevalence | Female | Botswana | T1D | 0.55<br>(0.54 to 0.56) | < 0.001 |
| Middle-aged and elderly age | Prevalence | Male | Botswana | T1D | 0.53<br>(0.5 to 0.55) | < 0.001 |
| Middle-aged and elderly age | Prevalence | Both | Botswana | IBD | 0.79<br>(0.66 to 0.92) | < 0.001 |
| Middle-aged and elderly age | Prevalence | Female | Botswana | IBD | 0.72<br>(0.57 to 0.88) | < 0.001 |
| Middle-aged and elderly age | Prevalence | Male | Botswana | IBD | 0.87<br>(0.77 to 0.97) | < 0.001 |
| Middle-aged and elderly age | Prevalence | Both | Botswana | MS | 0.35<br>(0.32 to 0.37) | < 0.001 |
| Middle-aged and elderly age | Prevalence | Female | Botswana | MS | 0.35<br>(0.32 to 0.38) | < 0.001 |
| Middle-aged and elderly age | Prevalence | Male | Botswana | MS | 0.32 | < 0.001 |

| | | | | | | |
|---|---|---|---|---|---|---|
| | | | | | (0.27 to 0.38) | |
| Middle-aged and elderly age | Prevalence | Both | Botswana | Psoriasis | -0.7<br>(-0.74 to -0.66) | < 0.001 |
| Middle-aged and elderly age | Prevalence | Female | Botswana | Psoriasis | -0.73<br>(-0.77 to -0.69) | < 0.001 |
| Middle-aged and elderly age | Prevalence | Male | Botswana | Psoriasis | -0.66<br>(-0.73 to -0.6) | < 0.001 |
| Middle-aged and elderly age | Prevalence | Both | Botswana | RHD | 0.03<br>(0.02 to 0.05) | < 0.001 |
| Middle-aged and elderly age | Prevalence | Female | Botswana | RHD | 0.05<br>(0.02 to 0.08) | 0.001 |
| Middle-aged and elderly age | Prevalence | Male | Botswana | RHD | 0.01<br>(-0.01 to 0.03) | 0.334 |
| Middle-aged and elderly age | Prevalence | Both | Botswana | RA | 0.84<br>(0.69 to 0.98) | < 0.001 |
| Middle-aged and elderly age | Prevalence | Female | Botswana | RA | 0.83<br>(0.67 to 1) | < 0.001 |
| Middle-aged and elderly age | Prevalence | Male | Botswana | RA | 0.8<br>(0.68 to 0.92) | < 0.001 |
| Middle-aged and elderly age | Prevalence | Both | Brazil | AA | 0.02<br>(0.02 to 0.02) | < 0.001 |
| Middle-aged and elderly age | Prevalence | Female | Brazil | AA | 0<br>(0 to 0) | < 0.001 |
| Middle-aged and elderly age | Prevalence | Male | Brazil | AA | 0<br>(0 to 0) | < 0.001 |
| Middle-aged and elderly age | Prevalence | Both | Brazil | T1D | 1.37<br>(1.31 to 1.44) | < 0.001 |
| Middle-aged and elderly age | Prevalence | Female | Brazil | T1D | 1.66<br>(1.57 to 1.74) | < 0.001 |
| Middle-aged and elderly age | Prevalence | Male | Brazil | T1D | 0.85<br>(0.53 to 1.18) | < 0.001 |
| Middle-aged and elderly age | Prevalence | Both | Brazil | IBD | -1.17<br>(-1.41 to -0.93) | < 0.001 |

| | | | | | | |
|---|---|---|---|---|---|---|
| Middle-aged and elderly age | Prevalence | Female | Brazil | IBD | -1.04 (-1.42 to -0.66) | < 0.001 |
| Middle-aged and elderly age | Prevalence | Male | Brazil | IBD | -1.4 (-1.55 to -1.24) | < 0.001 |
| Middle-aged and elderly age | Prevalence | Both | Brazil | MS | 0.34 (0.05 to 0.63) | 0.023 |
| Middle-aged and elderly age | Prevalence | Female | Brazil | MS | 0.04 (-0.25 to 0.34) | 0.770 |
| Middle-aged and elderly age | Prevalence | Male | Brazil | MS | 0.96 (0.73 to 1.18) | < 0.001 |
| Middle-aged and elderly age | Prevalence | Both | Brazil | Psoriasis | -0.17 (-0.19 to -0.15) | < 0.001 |
| Middle-aged and elderly age | Prevalence | Female | Brazil | Psoriasis | -0.17 (-0.19 to -0.15) | < 0.001 |
| Middle-aged and elderly age | Prevalence | Male | Brazil | Psoriasis | -0.17 (-0.19 to -0.16) | < 0.001 |
| Middle-aged and elderly age | Prevalence | Both | Brazil | RHD | 0.14 (0.11 to 0.16) | < 0.001 |
| Middle-aged and elderly age | Prevalence | Female | Brazil | RHD | 0.18 (0.14 to 0.21) | < 0.001 |
| Middle-aged and elderly age | Prevalence | Male | Brazil | RHD | 0.05 (0.03 to 0.06) | < 0.001 |
| Middle-aged and elderly age | Prevalence | Both | Brazil | RA | 0.24 (0.19 to 0.28) | < 0.001 |
| Middle-aged and elderly age | Prevalence | Female | Brazil | RA | 0.2 (0.16 to 0.23) | < 0.001 |
| Middle-aged and elderly age | Prevalence | Male | Brazil | RA | 0.27 (0.21 to 0.32) | < 0.001 |
| Middle-aged and elderly age | Prevalence | Both | Brunei Darussalam | AA | 0.05 (0.03 to 0.07) | < 0.001 |
| Middle-aged and elderly age | Prevalence | Female | Brunei Darussalam | AA | 0 (0 to 0) | < 0.001 |
| Middle-aged and elderly age | Prevalence | Male | Brunei Darussalam | AA | 0 | 0.002 |

| | | | | | | |
|---|---|---|---|---|---|---|
| | | | | | (0 to 0) | |
| Middle-aged and elderly age | Prevalence | Both | Brunei Darussalam | T1D | 0.17<br>(0.16 to 0.18) | < 0.001 |
| Middle-aged and elderly age | Prevalence | Female | Brunei Darussalam | T1D | 0.17<br>(0.16 to 0.18) | < 0.001 |
| Middle-aged and elderly age | Prevalence | Male | Brunei Darussalam | T1D | 0.18<br>(0.17 to 0.19) | < 0.001 |
| Middle-aged and elderly age | Prevalence | Both | Brunei Darussalam | IBD | 0.25<br>(0.23 to 0.27) | < 0.001 |
| Middle-aged and elderly age | Prevalence | Female | Brunei Darussalam | IBD | 0.31<br>(0.27 to 0.35) | < 0.001 |
| Middle-aged and elderly age | Prevalence | Male | Brunei Darussalam | IBD | 0.24<br>(0.23 to 0.25) | < 0.001 |
| Middle-aged and elderly age | Prevalence | Both | Brunei Darussalam | MS | 0.24<br>(0.14 to 0.34) | < 0.001 |
| Middle-aged and elderly age | Prevalence | Female | Brunei Darussalam | MS | 0.22<br>(0.14 to 0.3) | < 0.001 |
| Middle-aged and elderly age | Prevalence | Male | Brunei Darussalam | MS | 0.18<br>(0.13 to 0.23) | < 0.001 |
| Middle-aged and elderly age | Prevalence | Both | Brunei Darussalam | Psoriasis | -0.28<br>(-0.3 to -0.27) | < 0.001 |
| Middle-aged and elderly age | Prevalence | Female | Brunei Darussalam | Psoriasis | -0.35<br>(-0.37 to -0.33) | < 0.001 |
| Middle-aged and elderly age | Prevalence | Male | Brunei Darussalam | Psoriasis | -0.22<br>(-0.23 to -0.21) | < 0.001 |
| Middle-aged and elderly age | Prevalence | Both | Brunei Darussalam | RHD | -1<br>(-1.18 to -0.82) | < 0.001 |
| Middle-aged and elderly age | Prevalence | Female | Brunei Darussalam | RHD | -1.05<br>(-1.24 to -0.86) | < 0.001 |
| Middle-aged and elderly age | Prevalence | Male | Brunei Darussalam | RHD | -1<br>(-1.16 to -0.84) | < 0.001 |
| Middle-aged and elderly age | Prevalence | Both | Brunei Darussalam | RA | 0.55<br>(0.48 to 0.62) | < 0.001 |

| | | | | | | |
|---|---|---|---|---|---|---|
| Middle-aged and elderly age | Prevalence | Female | Brunei Darussalam | RA | 0.5<br>(0.47 to 0.54) | < 0.001 |
| Middle-aged and elderly age | Prevalence | Male | Brunei Darussalam | RA | 0.57<br>(0.53 to 0.6) | < 0.001 |
| Middle-aged and elderly age | Prevalence | Both | Bulgaria | AA | -0.02<br>(-0.03 to -0.02) | < 0.001 |
| Middle-aged and elderly age | Prevalence | Female | Bulgaria | AA | 0<br>(0 to 0) | < 0.001 |
| Middle-aged and elderly age | Prevalence | Male | Bulgaria | AA | 0<br>(0 to 0) | < 0.001 |
| Middle-aged and elderly age | Prevalence | Both | Bulgaria | T1D | 1.75<br>(1.7 to 1.8) | < 0.001 |
| Middle-aged and elderly age | Prevalence | Female | Bulgaria | T1D | 1.73<br>(1.7 to 1.75) | < 0.001 |
| Middle-aged and elderly age | Prevalence | Male | Bulgaria | T1D | 1.77<br>(1.68 to 1.87) | < 0.001 |
| Middle-aged and elderly age | Prevalence | Both | Bulgaria | IBD | -0.23<br>(-0.26 to -0.2) | < 0.001 |
| Middle-aged and elderly age | Prevalence | Female | Bulgaria | IBD | -0.23<br>(-0.28 to -0.19) | < 0.001 |
| Middle-aged and elderly age | Prevalence | Male | Bulgaria | IBD | -0.24<br>(-0.26 to -0.21) | < 0.001 |
| Middle-aged and elderly age | Prevalence | Both | Bulgaria | MS | -0.49<br>(-0.7 to -0.28) | < 0.001 |
| Middle-aged and elderly age | Prevalence | Female | Bulgaria | MS | -0.31<br>(-0.51 to -0.11) | 0.002 |
| Middle-aged and elderly age | Prevalence | Male | Bulgaria | MS | -0.68<br>(-0.9 to -0.46) | < 0.001 |
| Middle-aged and elderly age | Prevalence | Both | Bulgaria | Psoriasis | -0.81<br>(-0.84 to -0.77) | < 0.001 |
| Middle-aged and elderly age | Prevalence | Female | Bulgaria | Psoriasis | -0.82<br>(-0.88 to -0.76) | < 0.001 |
| Middle-aged and elderly age | Prevalence | Male | Bulgaria | Psoriasis | -0.79 | < 0.001 |

| | | | | | | |
|---|---|---|---|---|---|---|
| | | | | | (-0.81 to -0.77) | |
| Middle-aged and elderly age | Prevalence | Both | Bulgaria | RHD | -2.42<br>(-2.57 to -2.27) | < 0.001 |
| Middle-aged and elderly age | Prevalence | Female | Bulgaria | RHD | -2.67<br>(-2.79 to -2.55) | < 0.001 |
| Middle-aged and elderly age | Prevalence | Male | Bulgaria | RHD | -2.06<br>(-2.18 to -1.95) | < 0.001 |
| Middle-aged and elderly age | Prevalence | Both | Bulgaria | RA | 0.46<br>(0.4 to 0.52) | < 0.001 |
| Middle-aged and elderly age | Prevalence | Female | Bulgaria | RA | 0.48<br>(0.42 to 0.55) | < 0.001 |
| Middle-aged and elderly age | Prevalence | Male | Bulgaria | RA | 0.48<br>(0.4 to 0.55) | < 0.001 |
| Middle-aged and elderly age | Prevalence | Both | Burkina Faso | AA | 0.03<br>(0.01 to 0.05) | 0.010 |
| Middle-aged and elderly age | Prevalence | Female | Burkina Faso | AA | 0<br>(0 to 0) | 0.027 |
| Middle-aged and elderly age | Prevalence | Male | Burkina Faso | AA | 0<br>(0 to 0) | < 0.001 |
| Middle-aged and elderly age | Prevalence | Both | Burkina Faso | T1D | 0.15<br>(0.13 to 0.16) | < 0.001 |
| Middle-aged and elderly age | Prevalence | Female | Burkina Faso | T1D | 0.15<br>(0.14 to 0.16) | < 0.001 |
| Middle-aged and elderly age | Prevalence | Male | Burkina Faso | T1D | 0.15<br>(0.14 to 0.15) | < 0.001 |
| Middle-aged and elderly age | Prevalence | Both | Burkina Faso | IBD | 0.41<br>(0.37 to 0.46) | < 0.001 |
| Middle-aged and elderly age | Prevalence | Female | Burkina Faso | IBD | 0.29<br>(0.25 to 0.33) | < 0.001 |
| Middle-aged and elderly age | Prevalence | Male | Burkina Faso | IBD | 0.51<br>(0.35 to 0.66) | < 0.001 |
| Middle-aged and elderly age | Prevalence | Both | Burkina Faso | MS | 0.2<br>(0.16 to 0.23) | < 0.001 |

| | | | | | | |
|---|---|---|---|---|---|---|
| Middle-aged and elderly age | Prevalence | Female | Burkina Faso | MS | 0.11<br>(0.06 to 0.16) | < 0.001 |
| Middle-aged and elderly age | Prevalence | Male | Burkina Faso | MS | 0.31<br>(0.27 to 0.34) | < 0.001 |
| Middle-aged and elderly age | Prevalence | Both | Burkina Faso | Psoriasis | -0.54<br>(-0.77 to -0.3) | < 0.001 |
| Middle-aged and elderly age | Prevalence | Female | Burkina Faso | Psoriasis | -0.6<br>(-0.8 to -0.41) | < 0.001 |
| Middle-aged and elderly age | Prevalence | Male | Burkina Faso | Psoriasis | -0.45<br>(-0.64 to -0.27) | < 0.001 |
| Middle-aged and elderly age | Prevalence | Both | Burkina Faso | RHD | 0.05<br>(0.04 to 0.06) | < 0.001 |
| Middle-aged and elderly age | Prevalence | Female | Burkina Faso | RHD | 0.08<br>(0.06 to 0.1) | < 0.001 |
| Middle-aged and elderly age | Prevalence | Male | Burkina Faso | RHD | 0<br>(-0.01 to 0.01) | 0.497 |
| Middle-aged and elderly age | Prevalence | Both | Burkina Faso | RA | 0.61<br>(0.59 to 0.64) | < 0.001 |
| Middle-aged and elderly age | Prevalence | Female | Burkina Faso | RA | 0.58<br>(0.55 to 0.6) | < 0.001 |
| Middle-aged and elderly age | Prevalence | Male | Burkina Faso | RA | 0.63<br>(0.58 to 0.67) | < 0.001 |
| Middle-aged and elderly age | Prevalence | Both | Burundi | AA | -0.17<br>(-0.18 to -0.16) | < 0.001 |
| Middle-aged and elderly age | Prevalence | Female | Burundi | AA | 0<br>(0 to 0) | 1.000 |
| Middle-aged and elderly age | Prevalence | Male | Burundi | AA | 0<br>(0 to 0) | < 0.001 |
| Middle-aged and elderly age | Prevalence | Both | Burundi | T1D | 0.39<br>(0.37 to 0.42) | < 0.001 |
| Middle-aged and elderly age | Prevalence | Female | Burundi | T1D | 0.39<br>(0.37 to 0.42) | < 0.001 |
| Middle-aged and elderly age | Prevalence | Male | Burundi | T1D | 0.38 | < 0.001 |

| | | | | | | |
|---|---|---|---|---|---|---|
| | | | | | (0.35 to 0.41) | |
| Middle-aged and elderly age | Prevalence | Both | Burundi | IBD | -0.1<br>(-0.17 to -0.02) | 0.011 |
| Middle-aged and elderly age | Prevalence | Female | Burundi | IBD | -0.16<br>(-0.2 to -0.12) | < 0.001 |
| Middle-aged and elderly age | Prevalence | Male | Burundi | IBD | -0.04<br>(-0.15 to 0.08) | 0.524 |
| Middle-aged and elderly age | Prevalence | Both | Burundi | MS | -0.01<br>(-0.06 to 0.04) | 0.806 |
| Middle-aged and elderly age | Prevalence | Female | Burundi | MS | 0.16<br>(0.09 to 0.23) | < 0.001 |
| Middle-aged and elderly age | Prevalence | Male | Burundi | MS | 0.17<br>(0.11 to 0.23) | < 0.001 |
| Middle-aged and elderly age | Prevalence | Both | Burundi | Psoriasis | -0.05<br>(-0.06 to -0.03) | < 0.001 |
| Middle-aged and elderly age | Prevalence | Female | Burundi | Psoriasis | -0.03<br>(-0.05 to -0.02) | < 0.001 |
| Middle-aged and elderly age | Prevalence | Male | Burundi | Psoriasis | -0.04<br>(-0.08 to 0) | 0.070 |
| Middle-aged and elderly age | Prevalence | Both | Burundi | RHD | 0.03<br>(0.01 to 0.06) | 0.015 |
| Middle-aged and elderly age | Prevalence | Female | Burundi | RHD | 0.1<br>(0.09 to 0.1) | < 0.001 |
| Middle-aged and elderly age | Prevalence | Male | Burundi | RHD | 0.05<br>(-0.03 to 0.12) | 0.224 |
| Middle-aged and elderly age | Prevalence | Both | Burundi | RA | 0.64<br>(0.53 to 0.76) | < 0.001 |
| Middle-aged and elderly age | Prevalence | Female | Burundi | RA | 0.46<br>(0.37 to 0.56) | < 0.001 |
| Middle-aged and elderly age | Prevalence | Male | Burundi | RA | 0.5<br>(0.38 to 0.61) | < 0.001 |
| Middle-aged and elderly age | Prevalence | Both | Cabo Verde | AA | -0.19<br>(-0.2 to -0.18) | < 0.001 |

| | | | | | | |
|---|---|---|---|---|---|---|
| Middle-aged and elderly age | Prevalence | Female | Cabo Verde | AA | 0<br>(0 to 0) | 0.027 |
| Middle-aged and elderly age | Prevalence | Male | Cabo Verde | AA | 0<br>(0 to 0) | < 0.001 |
| Middle-aged and elderly age | Prevalence | Both | Cabo Verde | T1D | 0.61<br>(0.58 to 0.64) | < 0.001 |
| Middle-aged and elderly age | Prevalence | Female | Cabo Verde | T1D | 0.6<br>(0.58 to 0.63) | < 0.001 |
| Middle-aged and elderly age | Prevalence | Male | Cabo Verde | T1D | 0.59<br>(0.57 to 0.61) | < 0.001 |
| Middle-aged and elderly age | Prevalence | Both | Cabo Verde | IBD | 0.63<br>(0.55 to 0.71) | < 0.001 |
| Middle-aged and elderly age | Prevalence | Female | Cabo Verde | IBD | 0.62<br>(0.57 to 0.68) | < 0.001 |
| Middle-aged and elderly age | Prevalence | Male | Cabo Verde | IBD | 0.69<br>(0.63 to 0.75) | < 0.001 |
| Middle-aged and elderly age | Prevalence | Both | Cabo Verde | MS | 0.14<br>(0.12 to 0.16) | < 0.001 |
| Middle-aged and elderly age | Prevalence | Female | Cabo Verde | MS | 0.35<br>(0.29 to 0.41) | < 0.001 |
| Middle-aged and elderly age | Prevalence | Male | Cabo Verde | MS | 0.39<br>(0.29 to 0.48) | < 0.001 |
| Middle-aged and elderly age | Prevalence | Both | Cabo Verde | Psoriasis | -0.91<br>(-1.08 to -0.73) | < 0.001 |
| Middle-aged and elderly age | Prevalence | Female | Cabo Verde | Psoriasis | -0.97<br>(-1.17 to -0.77) | < 0.001 |
| Middle-aged and elderly age | Prevalence | Male | Cabo Verde | Psoriasis | -0.79<br>(-0.92 to -0.67) | < 0.001 |
| Middle-aged and elderly age | Prevalence | Both | Cabo Verde | RHD | 0.05<br>(0.04 to 0.06) | < 0.001 |
| Middle-aged and elderly age | Prevalence | Female | Cabo Verde | RHD | 0.15<br>(0.14 to 0.17) | < 0.001 |
| Middle-aged and elderly age | Prevalence | Male | Cabo Verde | RHD | 0.08 | < 0.001 |

| | | | | | | |
|---|---|---|---|---|---|---|
| | | | | | (0.07 to 0.08) | |
| Middle-aged and elderly age | Prevalence | Both | Cabo Verde | RA | 0.77 (0.72 to 0.81) | < 0.001 |
| Middle-aged and elderly age | Prevalence | Female | Cabo Verde | RA | 0.91 (0.86 to 0.95) | < 0.001 |
| Middle-aged and elderly age | Prevalence | Male | Cabo Verde | RA | 0.9 (0.86 to 0.93) | < 0.001 |
| Middle-aged and elderly age | Prevalence | Both | Cambodia | AA | -0.04 (-0.04 to -0.03) | < 0.001 |
| Middle-aged and elderly age | Prevalence | Female | Cambodia | AA | 0 (0 to 0) | < 0.001 |
| Middle-aged and elderly age | Prevalence | Male | Cambodia | AA | 0 (0 to 0) | < 0.001 |
| Middle-aged and elderly age | Prevalence | Both | Cambodia | T1D | 0.59 (0.57 to 0.61) | < 0.001 |
| Middle-aged and elderly age | Prevalence | Female | Cambodia | T1D | 0.54 (0.53 to 0.55) | < 0.001 |
| Middle-aged and elderly age | Prevalence | Male | Cambodia | T1D | 0.65 (0.61 to 0.68) | < 0.001 |
| Middle-aged and elderly age | Prevalence | Both | Cambodia | IBD | 0.62 (0.47 to 0.78) | < 0.001 |
| Middle-aged and elderly age | Prevalence | Female | Cambodia | IBD | 0.44 (0.28 to 0.61) | < 0.001 |
| Middle-aged and elderly age | Prevalence | Male | Cambodia | IBD | 0.92 (0.79 to 1.04) | < 0.001 |
| Middle-aged and elderly age | Prevalence | Both | Cambodia | MS | 0.37 (0.33 to 0.4) | < 0.001 |
| Middle-aged and elderly age | Prevalence | Female | Cambodia | MS | 0.38 (0.32 to 0.44) | < 0.001 |
| Middle-aged and elderly age | Prevalence | Male | Cambodia | MS | 0.45 (0.4 to 0.49) | < 0.001 |
| Middle-aged and elderly age | Prevalence | Both | Cambodia | Psoriasis | -0.62 (-0.64 to -0.6) | < 0.001 |

| | | | | | | |
|---|---|---|---|---|---|---|
| Middle-aged and elderly age | Prevalence | Female | Cambodia | Psoriasis | -0.63 (-0.63 to -0.62) | < 0.001 |
| Middle-aged and elderly age | Prevalence | Male | Cambodia | Psoriasis | -0.65 (-0.69 to -0.61) | < 0.001 |
| Middle-aged and elderly age | Prevalence | Both | Cambodia | RHD | 0 (-0.01 to 0.01) | 0.694 |
| Middle-aged and elderly age | Prevalence | Female | Cambodia | RHD | 0.06 (0.03 to 0.09) | < 0.001 |
| Middle-aged and elderly age | Prevalence | Male | Cambodia | RHD | -0.04 (-0.06 to -0.02) | < 0.001 |
| Middle-aged and elderly age | Prevalence | Both | Cambodia | RA | 0.7 (0.63 to 0.77) | < 0.001 |
| Middle-aged and elderly age | Prevalence | Female | Cambodia | RA | 0.8 (0.72 to 0.89) | < 0.001 |
| Middle-aged and elderly age | Prevalence | Male | Cambodia | RA | 0.62 (0.57 to 0.68) | < 0.001 |
| Middle-aged and elderly age | Prevalence | Both | Cameroon | AA | -0.01 (-0.01 to 0) | 0.040 |
| Middle-aged and elderly age | Prevalence | Female | Cameroon | AA | 0 (0 to 0) | 0.027 |
| Middle-aged and elderly age | Prevalence | Male | Cameroon | AA | 0 (0 to 0) | < 0.001 |
| Middle-aged and elderly age | Prevalence | Both | Cameroon | T1D | 0.47 (0.44 to 0.49) | < 0.001 |
| Middle-aged and elderly age | Prevalence | Female | Cameroon | T1D | 0.47 (0.45 to 0.5) | < 0.001 |
| Middle-aged and elderly age | Prevalence | Male | Cameroon | T1D | 0.46 (0.44 to 0.47) | < 0.001 |
| Middle-aged and elderly age | Prevalence | Both | Cameroon | IBD | 0.73 (0.61 to 0.85) | < 0.001 |
| Middle-aged and elderly age | Prevalence | Female | Cameroon | IBD | 0.66 (0.55 to 0.77) | < 0.001 |
| Middle-aged and elderly age | Prevalence | Male | Cameroon | IBD | 0.8 | < 0.001 |

| | | | | | | |
|---|---|---|---|---|---|---|
| | | | | | (0.69 to 0.91) | |
| Middle-aged and elderly age | Prevalence | Both | Cameroon | MS | 0.27<br>(0.22 to 0.33) | < 0.001 |
| Middle-aged and elderly age | Prevalence | Female | Cameroon | MS | 0.25<br>(0.23 to 0.27) | < 0.001 |
| Middle-aged and elderly age | Prevalence | Male | Cameroon | MS | 0.38<br>(0.17 to 0.58) | < 0.001 |
| Middle-aged and elderly age | Prevalence | Both | Cameroon | Psoriasis | -0.68<br>(-0.83 to -0.53) | < 0.001 |
| Middle-aged and elderly age | Prevalence | Female | Cameroon | Psoriasis | -0.72<br>(-0.8 to -0.64) | < 0.001 |
| Middle-aged and elderly age | Prevalence | Male | Cameroon | Psoriasis | -0.59<br>(-0.74 to -0.45) | < 0.001 |
| Middle-aged and elderly age | Prevalence | Both | Cameroon | RHD | 0.22<br>(0.2 to 0.24) | < 0.001 |
| Middle-aged and elderly age | Prevalence | Female | Cameroon | RHD | 0.25<br>(0.23 to 0.27) | < 0.001 |
| Middle-aged and elderly age | Prevalence | Male | Cameroon | RHD | 0.18<br>(0.15 to 0.21) | < 0.001 |
| Middle-aged and elderly age | Prevalence | Both | Cameroon | RA | 0.59<br>(0.52 to 0.66) | < 0.001 |
| Middle-aged and elderly age | Prevalence | Female | Cameroon | RA | 0.56<br>(0.49 to 0.63) | < 0.001 |
| Middle-aged and elderly age | Prevalence | Male | Cameroon | RA | 0.64<br>(0.57 to 0.71) | < 0.001 |
| Middle-aged and elderly age | Prevalence | Both | Canada | AA | 0.04<br>(0.03 to 0.04) | < 0.001 |
| Middle-aged and elderly age | Prevalence | Female | Canada | AA | 0<br>(0 to 0) | < 0.001 |
| Middle-aged and elderly age | Prevalence | Male | Canada | AA | 0<br>(0 to 0) | < 0.001 |
| Middle-aged and elderly age | Prevalence | Both | Canada | T1D | 0.83<br>(0.54 to 1.13) | < 0.001 |

| | | | | | | |
|---|---|---|---|---|---|---|
| Middle-aged and elderly age | Prevalence | Female | Canada | T1D | 0.69<br>(0.21 to 1.17) | 0.005 |
| Middle-aged and elderly age | Prevalence | Male | Canada | T1D | 0.98<br>(0.84 to 1.13) | < 0.001 |
| Middle-aged and elderly age | Prevalence | Both | Canada | IBD | -0.61<br>(-0.82 to -0.4) | < 0.001 |
| Middle-aged and elderly age | Prevalence | Female | Canada | IBD | -0.38<br>(-0.52 to -0.24) | < 0.001 |
| Middle-aged and elderly age | Prevalence | Male | Canada | IBD | -0.9<br>(-1.11 to -0.7) | < 0.001 |
| Middle-aged and elderly age | Prevalence | Both | Canada | MS | 0.65<br>(0.59 to 0.7) | < 0.001 |
| Middle-aged and elderly age | Prevalence | Female | Canada | MS | 0.66<br>(0.59 to 0.72) | < 0.001 |
| Middle-aged and elderly age | Prevalence | Male | Canada | MS | 0.56<br>(0.52 to 0.59) | < 0.001 |
| Middle-aged and elderly age | Prevalence | Both | Canada | Psoriasis | -0.41<br>(-0.41 to -0.41) | < 0.001 |
| Middle-aged and elderly age | Prevalence | Female | Canada | Psoriasis | -0.41<br>(-0.42 to -0.4) | < 0.001 |
| Middle-aged and elderly age | Prevalence | Male | Canada | Psoriasis | -0.41<br>(-0.41 to -0.4) | < 0.001 |
| Middle-aged and elderly age | Prevalence | Both | Canada | RHD | -0.65<br>(-0.69 to -0.6) | < 0.001 |
| Middle-aged and elderly age | Prevalence | Female | Canada | RHD | -0.88<br>(-0.96 to -0.81) | < 0.001 |
| Middle-aged and elderly age | Prevalence | Male | Canada | RHD | -0.4<br>(-0.46 to -0.34) | < 0.001 |
| Middle-aged and elderly age | Prevalence | Both | Canada | RA | 0.88<br>(0.86 to 0.89) | < 0.001 |
| Middle-aged and elderly age | Prevalence | Female | Canada | RA | 0.87<br>(0.85 to 0.89) | < 0.001 |
| Middle-aged and elderly age | Prevalence | Male | Canada | RA | 0.79 | < 0.001 |

| | | | | | | |
|---|---|---|---|---|---|---|
| | | | | | (0.76 to 0.81) | |
| Middle-aged and elderly age | Prevalence | Both | Central African Republic | AA | -0.07<br>(-0.08 to -0.06) | < 0.001 |
| Middle-aged and elderly age | Prevalence | Female | Central African Republic | AA | 0<br>(0 to 0) | 0.027 |
| Middle-aged and elderly age | Prevalence | Male | Central African Republic | AA | 0<br>(0 to 0) | < 0.001 |
| Middle-aged and elderly age | Prevalence | Both | Central African Republic | T1D | 0.37<br>(0.36 to 0.38) | < 0.001 |
| Middle-aged and elderly age | Prevalence | Female | Central African Republic | T1D | 0.38<br>(0.37 to 0.38) | < 0.001 |
| Middle-aged and elderly age | Prevalence | Male | Central African Republic | T1D | 0.36<br>(0.35 to 0.38) | < 0.001 |
| Middle-aged and elderly age | Prevalence | Both | Central African Republic | IBD | 0.1<br>(0.06 to 0.15) | < 0.001 |
| Middle-aged and elderly age | Prevalence | Female | Central African Republic | IBD | 0<br>(-0.07 to 0.06) | 0.902 |
| Middle-aged and elderly age | Prevalence | Male | Central African Republic | IBD | 0.25<br>(0.21 to 0.29) | < 0.001 |
| Middle-aged and elderly age | Prevalence | Both | Central African Republic | MS | 0.01<br>(-0.01 to 0.02) | 0.343 |
| Middle-aged and elderly age | Prevalence | Female | Central African Republic | MS | 0.07<br>(0.05 to 0.09) | < 0.001 |
| Middle-aged and elderly age | Prevalence | Male | Central African Republic | MS | 0.11<br>(0.09 to 0.12) | < 0.001 |
| Middle-aged and elderly age | Prevalence | Both | Central African Republic | Psoriasis | -0.49<br>(-0.51 to -0.47) | < 0.001 |
| Middle-aged and elderly age | Prevalence | Female | Central African Republic | Psoriasis | -0.49<br>(-0.51 to -0.47) | < 0.001 |
| Middle-aged and elderly age | Prevalence | Male | Central African Republic | Psoriasis | -0.48<br>(-0.52 to -0.44) | < 0.001 |
| Middle-aged and elderly age | Prevalence | Both | Central African Republic | RHD | 0.02<br>(0.01 to 0.03) | < 0.001 |

| | | | | | | |
|---|---|---|---|---|---|---|
| Middle-aged and elderly age | Prevalence | Female | Central African Republic | RHD | 0.06<br>(0.04 to 0.09) | < 0.001 |
| Middle-aged and elderly age | Prevalence | Male | Central African Republic | RHD | -0.01<br>(-0.03 to 0) | 0.163 |
| Middle-aged and elderly age | Prevalence | Both | Central African Republic | RA | -0.03<br>(-0.1 to 0.04) | 0.402 |
| Middle-aged and elderly age | Prevalence | Female | Central African Republic | RA | 0.03<br>(-0.04 to 0.09) | 0.400 |
| Middle-aged and elderly age | Prevalence | Male | Central African Republic | RA | -0.04<br>(-0.13 to 0.04) | 0.321 |
| Middle-aged and elderly age | Prevalence | Both | Chad | AA | -0.14<br>(-0.15 to -0.13) | < 0.001 |
| Middle-aged and elderly age | Prevalence | Female | Chad | AA | 0<br>(0 to 0) | 0.027 |
| Middle-aged and elderly age | Prevalence | Male | Chad | AA | 0<br>(0 to 0) | < 0.001 |
| Middle-aged and elderly age | Prevalence | Both | Chad | T1D | 0.2<br>(0.2 to 0.21) | < 0.001 |
| Middle-aged and elderly age | Prevalence | Female | Chad | T1D | 0.2<br>(0.18 to 0.21) | < 0.001 |
| Middle-aged and elderly age | Prevalence | Male | Chad | T1D | 0.19<br>(0.17 to 0.2) | < 0.001 |
| Middle-aged and elderly age | Prevalence | Both | Chad | IBD | 0.53<br>(0.48 to 0.57) | < 0.001 |
| Middle-aged and elderly age | Prevalence | Female | Chad | IBD | 0.52<br>(0.49 to 0.54) | < 0.001 |
| Middle-aged and elderly age | Prevalence | Male | Chad | IBD | 0.56<br>(0.49 to 0.63) | < 0.001 |
| Middle-aged and elderly age | Prevalence | Both | Chad | MS | 0.01<br>(-0.02 to 0.04) | 0.522 |
| Middle-aged and elderly age | Prevalence | Female | Chad | MS | 0.16<br>(0.13 to 0.19) | < 0.001 |
| Middle-aged and elderly age | Prevalence | Male | Chad | MS | 0.28 | < 0.001 |

| | | | | | | |
|---|---|---|---|---|---|---|
| | | | | | (0.2 to 0.35) | |
| Middle-aged and elderly age | Prevalence | Both | Chad | Psoriasis | -0.58<br>(-0.73 to -0.42) | < 0.001 |
| Middle-aged and elderly age | Prevalence | Female | Chad | Psoriasis | -0.62<br>(-0.75 to -0.5) | < 0.001 |
| Middle-aged and elderly age | Prevalence | Male | Chad | Psoriasis | -0.48<br>(-0.63 to -0.34) | < 0.001 |
| Middle-aged and elderly age | Prevalence | Both | Chad | RHD | 0.04<br>(0.03 to 0.05) | < 0.001 |
| Middle-aged and elderly age | Prevalence | Female | Chad | RHD | 0.09<br>(0.07 to 0.12) | < 0.001 |
| Middle-aged and elderly age | Prevalence | Male | Chad | RHD | 0.03<br>(0.02 to 0.04) | < 0.001 |
| Middle-aged and elderly age | Prevalence | Both | Chad | RA | 0.32<br>(0.3 to 0.35) | < 0.001 |
| Middle-aged and elderly age | Prevalence | Female | Chad | RA | 0.38<br>(0.33 to 0.44) | < 0.001 |
| Middle-aged and elderly age | Prevalence | Male | Chad | RA | 0.41<br>(0.38 to 0.45) | < 0.001 |
| Middle-aged and elderly age | Prevalence | Both | Chile | AA | -0.02<br>(-0.02 to -0.01) | < 0.001 |
| Middle-aged and elderly age | Prevalence | Female | Chile | AA | 0<br>(0 to 0) | 0.000 |
| Middle-aged and elderly age | Prevalence | Male | Chile | AA | 0<br>(0 to 0) | 0.002 |
| Middle-aged and elderly age | Prevalence | Both | Chile | T1D | 1.33<br>(1.29 to 1.36) | < 0.001 |
| Middle-aged and elderly age | Prevalence | Female | Chile | T1D | 1.25<br>(1.22 to 1.29) | < 0.001 |
| Middle-aged and elderly age | Prevalence | Male | Chile | T1D | 1.41<br>(1.36 to 1.45) | < 0.001 |
| Middle-aged and elderly age | Prevalence | Both | Chile | IBD | 0.16<br>(0.07 to 0.24) | < 0.001 |

| | | | | | | |
|---|---|---|---|---|---|---|
| Middle-aged and elderly age | Prevalence | Female | Chile | IBD | 0.25 (0.16 to 0.34) | < 0.001 |
| Middle-aged and elderly age | Prevalence | Male | Chile | IBD | 0.05 (-0.08 to 0.19) | 0.423 |
| Middle-aged and elderly age | Prevalence | Both | Chile | MS | 0.31 (0.24 to 0.39) | < 0.001 |
| Middle-aged and elderly age | Prevalence | Female | Chile | MS | 0.35 (0.3 to 0.41) | < 0.001 |
| Middle-aged and elderly age | Prevalence | Male | Chile | MS | 0.26 (0.15 to 0.37) | < 0.001 |
| Middle-aged and elderly age | Prevalence | Both | Chile | Psoriasis | -0.62 (-0.63 to -0.61) | < 0.001 |
| Middle-aged and elderly age | Prevalence | Female | Chile | Psoriasis | -0.63 (-0.65 to -0.61) | < 0.001 |
| Middle-aged and elderly age | Prevalence | Male | Chile | Psoriasis | -0.6 (-0.62 to -0.59) | < 0.001 |
| Middle-aged and elderly age | Prevalence | Both | Chile | RHD | -1.97 (-2.06 to -1.88) | < 0.001 |
| Middle-aged and elderly age | Prevalence | Female | Chile | RHD | -2.18 (-2.23 to -2.13) | < 0.001 |
| Middle-aged and elderly age | Prevalence | Male | Chile | RHD | -1.69 (-1.82 to -1.57) | < 0.001 |
| Middle-aged and elderly age | Prevalence | Both | Chile | RA | 1.44 (1.41 to 1.46) | < 0.001 |
| Middle-aged and elderly age | Prevalence | Female | Chile | RA | 1.48 (1.46 to 1.5) | < 0.001 |
| Middle-aged and elderly age | Prevalence | Male | Chile | RA | 1.26 (1.21 to 1.3) | < 0.001 |
| Middle-aged and elderly age | Prevalence | Both | China | AA | 0.06 (0.05 to 0.06) | < 0.001 |
| Middle-aged and elderly age | Prevalence | Female | China | AA | 0 (0 to 0) | 0.001 |
| Middle-aged and elderly age | Prevalence | Male | China | AA | 0 | < 0.001 |

| | | | | | | |
|---|---|---|---|---|---|---|
| | | | | | (0 to 0) | |
| Middle-aged and elderly age | Prevalence | Both | China | T1D | 1.93<br>(1.73 to 2.14) | < 0.001 |
| Middle-aged and elderly age | Prevalence | Female | China | T1D | 1.83<br>(1.66 to 2) | < 0.001 |
| Middle-aged and elderly age | Prevalence | Male | China | T1D | 2.02<br>(1.81 to 2.22) | < 0.001 |
| Middle-aged and elderly age | Prevalence | Both | China | IBD | 2.62<br>(2.5 to 2.74) | < 0.001 |
| Middle-aged and elderly age | Prevalence | Female | China | IBD | 2.95<br>(2.82 to 3.08) | < 0.001 |
| Middle-aged and elderly age | Prevalence | Male | China | IBD | 2.4<br>(2.27 to 2.52) | < 0.001 |
| Middle-aged and elderly age | Prevalence | Both | China | MS | 1.1<br>(1.03 to 1.17) | < 0.001 |
| Middle-aged and elderly age | Prevalence | Female | China | MS | 1.29<br>(1.11 to 1.47) | < 0.001 |
| Middle-aged and elderly age | Prevalence | Male | China | MS | 0.85<br>(0.8 to 0.89) | < 0.001 |
| Middle-aged and elderly age | Prevalence | Both | China | Psoriasis | -0.99<br>(-1.02 to -0.96) | < 0.001 |
| Middle-aged and elderly age | Prevalence | Female | China | Psoriasis | -0.97<br>(-1.03 to -0.9) | < 0.001 |
| Middle-aged and elderly age | Prevalence | Male | China | Psoriasis | -0.92<br>(-0.94 to -0.9) | < 0.001 |
| Middle-aged and elderly age | Prevalence | Both | China | RHD | 0.01<br>(-0.06 to 0.08) | 0.860 |
| Middle-aged and elderly age | Prevalence | Female | China | RHD | 0.04<br>(-0.02 to 0.09) | 0.171 |
| Middle-aged and elderly age | Prevalence | Male | China | RHD | -0.07<br>(-0.16 to 0.02) | 0.140 |
| Middle-aged and elderly age | Prevalence | Both | China | RA | 0.24<br>(0.14 to 0.34) | < 0.001 |

| | | | | | | |
|---|---|---|---|---|---|---|
| Middle-aged and elderly age | Prevalence | Female | China | RA | 0.14<br>(0.04 to 0.23) | 0.005 |
| Middle-aged and elderly age | Prevalence | Male | China | RA | 0.2<br>(0.17 to 0.23) | < 0.001 |
| Middle-aged and elderly age | Prevalence | Both | Colombia | AA | 0.06<br>(0.05 to 0.06) | < 0.001 |
| Middle-aged and elderly age | Prevalence | Female | Colombia | AA | 0<br>(0 to 0) | < 0.001 |
| Middle-aged and elderly age | Prevalence | Male | Colombia | AA | 0<br>(0 to 0) | < 0.001 |
| Middle-aged and elderly age | Prevalence | Both | Colombia | T1D | 0.97<br>(0.96 to 0.98) | < 0.001 |
| Middle-aged and elderly age | Prevalence | Female | Colombia | T1D | 1.04<br>(1.02 to 1.06) | < 0.001 |
| Middle-aged and elderly age | Prevalence | Male | Colombia | T1D | 0.95<br>(0.93 to 0.96) | < 0.001 |
| Middle-aged and elderly age | Prevalence | Both | Colombia | IBD | 0.01<br>(-0.01 to 0.03) | 0.374 |
| Middle-aged and elderly age | Prevalence | Female | Colombia | IBD | 0.06<br>(0.04 to 0.09) | < 0.001 |
| Middle-aged and elderly age | Prevalence | Male | Colombia | IBD | 0.02<br>(-0.01 to 0.04) | 0.247 |
| Middle-aged and elderly age | Prevalence | Both | Colombia | MS | 0.9<br>(0.81 to 0.98) | < 0.001 |
| Middle-aged and elderly age | Prevalence | Female | Colombia | MS | 0.9<br>(0.81 to 0.99) | < 0.001 |
| Middle-aged and elderly age | Prevalence | Male | Colombia | MS | 0.6<br>(0.54 to 0.67) | < 0.001 |
| Middle-aged and elderly age | Prevalence | Both | Colombia | Psoriasis | -0.48<br>(-0.52 to -0.44) | < 0.001 |
| Middle-aged and elderly age | Prevalence | Female | Colombia | Psoriasis | -0.5<br>(-0.55 to -0.45) | < 0.001 |
| Middle-aged and elderly age | Prevalence | Male | Colombia | Psoriasis | -0.47 | < 0.001 |

| | | | | | | |
|---|---|---|---|---|---|---|
| | | | | | (-0.52 to -0.43) | |
| Middle-aged and elderly age | Prevalence | Both | Colombia | RHD | -0.79<br>(-1.04 to -0.55) | < 0.001 |
| Middle-aged and elderly age | Prevalence | Female | Colombia | RHD | -0.85<br>(-1.13 to -0.56) | < 0.001 |
| Middle-aged and elderly age | Prevalence | Male | Colombia | RHD | -0.8<br>(-0.97 to -0.63) | < 0.001 |
| Middle-aged and elderly age | Prevalence | Both | Colombia | RA | 0.9<br>(0.76 to 1.05) | < 0.001 |
| Middle-aged and elderly age | Prevalence | Female | Colombia | RA | 0.84<br>(0.73 to 0.95) | < 0.001 |
| Middle-aged and elderly age | Prevalence | Male | Colombia | RA | 0.72<br>(0.56 to 0.88) | < 0.001 |
| Middle-aged and elderly age | Prevalence | Both | Comoros | AA | 0.01<br>(0.01 to 0.02) | < 0.001 |
| Middle-aged and elderly age | Prevalence | Female | Comoros | AA | 0<br>(0 to 0) | 1.000 |
| Middle-aged and elderly age | Prevalence | Male | Comoros | AA | 0<br>(0 to 0) | < 0.001 |
| Middle-aged and elderly age | Prevalence | Both | Comoros | T1D | 0.64<br>(0.63 to 0.65) | < 0.001 |
| Middle-aged and elderly age | Prevalence | Female | Comoros | T1D | 0.65<br>(0.64 to 0.67) | < 0.001 |
| Middle-aged and elderly age | Prevalence | Male | Comoros | T1D | 0.63<br>(0.62 to 0.65) | < 0.001 |
| Middle-aged and elderly age | Prevalence | Both | Comoros | IBD | -0.01<br>(-0.12 to 0.1) | 0.835 |
| Middle-aged and elderly age | Prevalence | Female | Comoros | IBD | -0.27<br>(-0.4 to -0.13) | < 0.001 |
| Middle-aged and elderly age | Prevalence | Male | Comoros | IBD | 0.23<br>(0.13 to 0.34) | < 0.001 |
| Middle-aged and elderly age | Prevalence | Both | Comoros | MS | 0.25<br>(0.22 to 0.28) | < 0.001 |

| | | | | | | |
|---|---|---|---|---|---|---|
| Middle-aged and elderly age | Prevalence | Female | Comoros | MS | 0.23 (0.2 to 0.27) | < 0.001 |
| Middle-aged and elderly age | Prevalence | Male | Comoros | MS | 0.23 (0.15 to 0.3) | < 0.001 |
| Middle-aged and elderly age | Prevalence | Both | Comoros | Psoriasis | -0.33 (-0.35 to -0.31) | < 0.001 |
| Middle-aged and elderly age | Prevalence | Female | Comoros | Psoriasis | -0.34 (-0.36 to -0.32) | < 0.001 |
| Middle-aged and elderly age | Prevalence | Male | Comoros | Psoriasis | -0.32 (-0.34 to -0.3) | < 0.001 |
| Middle-aged and elderly age | Prevalence | Both | Comoros | RHD | 0.06 (0.04 to 0.07) | < 0.001 |
| Middle-aged and elderly age | Prevalence | Female | Comoros | RHD | 0.08 (0.04 to 0.11) | < 0.001 |
| Middle-aged and elderly age | Prevalence | Male | Comoros | RHD | 0.02 (-0.03 to 0.07) | 0.387 |
| Middle-aged and elderly age | Prevalence | Both | Comoros | RA | 0.72 (0.64 to 0.79) | < 0.001 |
| Middle-aged and elderly age | Prevalence | Female | Comoros | RA | 0.72 (0.69 to 0.75) | < 0.001 |
| Middle-aged and elderly age | Prevalence | Male | Comoros | RA | 0.73 (0.62 to 0.84) | < 0.001 |
| Middle-aged and elderly age | Prevalence | Both | Congo | AA | -0.13 (-0.15 to -0.12) | < 0.001 |
| Middle-aged and elderly age | Prevalence | Female | Congo | AA | 0 (0 to 0) | 0.027 |
| Middle-aged and elderly age | Prevalence | Male | Congo | AA | 0 (0 to 0) | < 0.001 |
| Middle-aged and elderly age | Prevalence | Both | Congo | T1D | 0.85 (0.84 to 0.86) | < 0.001 |
| Middle-aged and elderly age | Prevalence | Female | Congo | T1D | 0.87 (0.86 to 0.87) | < 0.001 |
| Middle-aged and elderly age | Prevalence | Male | Congo | T1D | 0.82 | < 0.001 |

| | | | | | | |
|---|---|---|---|---|---|---|
| | | | | | (0.81 to 0.83) | |
| Middle-aged and elderly age | Prevalence | Both | Congo | IBD | 0.15<br>(0.07 to 0.23) | < 0.001 |
| Middle-aged and elderly age | Prevalence | Female | Congo | IBD | -0.01<br>(-0.06 to 0.04) | 0.610 |
| Middle-aged and elderly age | Prevalence | Male | Congo | IBD | 0.3<br>(0.15 to 0.46) | < 0.001 |
| Middle-aged and elderly age | Prevalence | Both | Congo | MS | 0.11<br>(0.07 to 0.14) | < 0.001 |
| Middle-aged and elderly age | Prevalence | Female | Congo | MS | 0.22<br>(0.18 to 0.27) | < 0.001 |
| Middle-aged and elderly age | Prevalence | Male | Congo | MS | 0.23<br>(0.19 to 0.28) | < 0.001 |
| Middle-aged and elderly age | Prevalence | Both | Congo | Psoriasis | -0.98<br>(-1 to -0.96) | < 0.001 |
| Middle-aged and elderly age | Prevalence | Female | Congo | Psoriasis | -0.98<br>(-1 to -0.96) | < 0.001 |
| Middle-aged and elderly age | Prevalence | Male | Congo | Psoriasis | -0.97<br>(-0.98 to -0.96) | < 0.001 |
| Middle-aged and elderly age | Prevalence | Both | Congo | RHD | -0.03<br>(-0.04 to -0.01) | < 0.001 |
| Middle-aged and elderly age | Prevalence | Female | Congo | RHD | 0.03<br>(0.01 to 0.04) | < 0.001 |
| Middle-aged and elderly age | Prevalence | Male | Congo | RHD | -0.06<br>(-0.09 to -0.03) | < 0.001 |
| Middle-aged and elderly age | Prevalence | Both | Congo | RA | 0.53<br>(0.4 to 0.67) | < 0.001 |
| Middle-aged and elderly age | Prevalence | Female | Congo | RA | 0.65<br>(0.53 to 0.77) | < 0.001 |
| Middle-aged and elderly age | Prevalence | Male | Congo | RA | 0.56<br>(0.42 to 0.7) | < 0.001 |
| Middle-aged and elderly age | Prevalence | Both | Cook Islands | AA | 0.06<br>(0.02 to 0.09) | 0.002 |

| | | | | | | |
|---|---|---|---|---|---|---|
| Middle-aged and elderly age | Prevalence | Female | Cook Islands | AA | 0<br>(0 to 0) | < 0.001 |
| Middle-aged and elderly age | Prevalence | Male | Cook Islands | AA | 0<br>(0 to 0) | < 0.001 |
| Middle-aged and elderly age | Prevalence | Both | Cook Islands | T1D | 0.82<br>(0.78 to 0.85) | < 0.001 |
| Middle-aged and elderly age | Prevalence | Female | Cook Islands | T1D | 0.8<br>(0.78 to 0.81) | < 0.001 |
| Middle-aged and elderly age | Prevalence | Male | Cook Islands | T1D | 0.86<br>(0.77 to 0.95) | < 0.001 |
| Middle-aged and elderly age | Prevalence | Both | Cook Islands | IBD | -0.27<br>(-0.32 to -0.22) | < 0.001 |
| Middle-aged and elderly age | Prevalence | Female | Cook Islands | IBD | -0.21<br>(-0.29 to -0.13) | < 0.001 |
| Middle-aged and elderly age | Prevalence | Male | Cook Islands | IBD | -0.28<br>(-0.34 to -0.23) | < 0.001 |
| Middle-aged and elderly age | Prevalence | Both | Cook Islands | MS | 0.2<br>(0.15 to 0.25) | < 0.001 |
| Middle-aged and elderly age | Prevalence | Female | Cook Islands | MS | 0.14<br>(0.11 to 0.16) | < 0.001 |
| Middle-aged and elderly age | Prevalence | Male | Cook Islands | MS | 0.14<br>(0.11 to 0.17) | < 0.001 |
| Middle-aged and elderly age | Prevalence | Both | Cook Islands | Psoriasis | -0.55<br>(-0.59 to -0.52) | < 0.001 |
| Middle-aged and elderly age | Prevalence | Female | Cook Islands | Psoriasis | -0.51<br>(-0.52 to -0.5) | < 0.001 |
| Middle-aged and elderly age | Prevalence | Male | Cook Islands | Psoriasis | -0.53<br>(-0.57 to -0.48) | < 0.001 |
| Middle-aged and elderly age | Prevalence | Both | Cook Islands | RHD | -0.13<br>(-0.17 to -0.09) | < 0.001 |
| Middle-aged and elderly age | Prevalence | Female | Cook Islands | RHD | -0.2<br>(-0.27 to -0.13) | < 0.001 |
| Middle-aged and elderly age | Prevalence | Male | Cook Islands | RHD | -0.07 | < 0.001 |

| | | | | | | |
|---|---|---|---|---|---|---|
| | | | | | (-0.1 to -0.03) | |
| Middle-aged and elderly age | Prevalence | Both | Cook Islands | RA | 0.62<br>(0.57 to 0.67) | < 0.001 |
| Middle-aged and elderly age | Prevalence | Female | Cook Islands | RA | 0.57<br>(0.53 to 0.61) | < 0.001 |
| Middle-aged and elderly age | Prevalence | Male | Cook Islands | RA | 0.51<br>(0.46 to 0.57) | < 0.001 |
| Middle-aged and elderly age | Prevalence | Both | Costa Rica | AA | 0.05<br>(0.05 to 0.05) | < 0.001 |
| Middle-aged and elderly age | Prevalence | Female | Costa Rica | AA | 0<br>(0 to 0) | < 0.001 |
| Middle-aged and elderly age | Prevalence | Male | Costa Rica | AA | 0<br>(0 to 0) | < 0.001 |
| Middle-aged and elderly age | Prevalence | Both | Costa Rica | T1D | 0.68<br>(0.66 to 0.7) | < 0.001 |
| Middle-aged and elderly age | Prevalence | Female | Costa Rica | T1D | 0.7<br>(0.68 to 0.72) | < 0.001 |
| Middle-aged and elderly age | Prevalence | Male | Costa Rica | T1D | 0.66<br>(0.64 to 0.69) | < 0.001 |
| Middle-aged and elderly age | Prevalence | Both | Costa Rica | IBD | 0.02<br>(0 to 0.03) | 0.026 |
| Middle-aged and elderly age | Prevalence | Female | Costa Rica | IBD | 0.06<br>(0 to 0.12) | 0.037 |
| Middle-aged and elderly age | Prevalence | Male | Costa Rica | IBD | 0.02<br>(0 to 0.04) | 0.066 |
| Middle-aged and elderly age | Prevalence | Both | Costa Rica | MS | 0.78<br>(0.7 to 0.86) | < 0.001 |
| Middle-aged and elderly age | Prevalence | Female | Costa Rica | MS | 0.69<br>(0.62 to 0.77) | < 0.001 |
| Middle-aged and elderly age | Prevalence | Male | Costa Rica | MS | 0.64<br>(0.58 to 0.7) | < 0.001 |
| Middle-aged and elderly age | Prevalence | Both | Costa Rica | Psoriasis | -0.44<br>(-0.46 to -0.42) | < 0.001 |

| | | | | | | |
|---|---|---|---|---|---|---|
| Middle-aged and elderly age | Prevalence | Female | Costa Rica | Psoriasis | -0.46<br>(-0.49 to -0.43) | < 0.001 |
| Middle-aged and elderly age | Prevalence | Male | Costa Rica | Psoriasis | -0.43<br>(-0.45 to -0.41) | < 0.001 |
| Middle-aged and elderly age | Prevalence | Both | Costa Rica | RHD | 0.07<br>(0.05 to 0.1) | < 0.001 |
| Middle-aged and elderly age | Prevalence | Female | Costa Rica | RHD | 0.06<br>(0.03 to 0.08) | < 0.001 |
| Middle-aged and elderly age | Prevalence | Male | Costa Rica | RHD | 0.03<br>(0.01 to 0.06) | 0.010 |
| Middle-aged and elderly age | Prevalence | Both | Costa Rica | RA | 0.98<br>(0.95 to 1.01) | < 0.001 |
| Middle-aged and elderly age | Prevalence | Female | Costa Rica | RA | 0.91<br>(0.86 to 0.96) | < 0.001 |
| Middle-aged and elderly age | Prevalence | Male | Costa Rica | RA | 0.75<br>(0.71 to 0.78) | < 0.001 |
| Middle-aged and elderly age | Prevalence | Both | Coted'Ivoire | AA | 0.01<br>(0 to 0.01) | 0.004 |
| Middle-aged and elderly age | Prevalence | Female | Coted'Ivoire | AA | 0<br>(0 to 0) | 0.027 |
| Middle-aged and elderly age | Prevalence | Male | Coted'Ivoire | AA | 0<br>(0 to 0) | < 0.001 |
| Middle-aged and elderly age | Prevalence | Both | Coted'Ivoire | T1D | 0.36<br>(0.35 to 0.37) | < 0.001 |
| Middle-aged and elderly age | Prevalence | Female | Coted'Ivoire | T1D | 0.37<br>(0.35 to 0.38) | < 0.001 |
| Middle-aged and elderly age | Prevalence | Male | Coted'Ivoire | T1D | 0.36<br>(0.35 to 0.37) | < 0.001 |
| Middle-aged and elderly age | Prevalence | Both | Coted'Ivoire | IBD | 0.09<br>(0.06 to 0.13) | < 0.001 |
| Middle-aged and elderly age | Prevalence | Female | Coted'Ivoire | IBD | -0.05<br>(-0.11 to 0) | 0.049 |
| Middle-aged and elderly age | Prevalence | Male | Coted'Ivoire | IBD | 0.24 | < 0.001 |

| | | | | | | |
|---|---|---|---|---|---|---|
| | | | | | (0.17 to 0.31) | |
| Middle-aged and elderly age | Prevalence | Both | Coted'Ivoire | MS | 0.22<br>(0.2 to 0.24) | < 0.001 |
| Middle-aged and elderly age | Prevalence | Female | Coted'Ivoire | MS | 0.18<br>(0.14 to 0.22) | < 0.001 |
| Middle-aged and elderly age | Prevalence | Male | Coted'Ivoire | MS | 0.22<br>(0.09 to 0.35) | 0.001 |
| Middle-aged and elderly age | Prevalence | Both | Coted'Ivoire | Psoriasis | -0.59<br>(-0.68 to -0.51) | < 0.001 |
| Middle-aged and elderly age | Prevalence | Female | Coted'Ivoire | Psoriasis | -0.67<br>(-0.77 to -0.58) | < 0.001 |
| Middle-aged and elderly age | Prevalence | Male | Coted'Ivoire | Psoriasis | -0.56<br>(-0.7 to -0.41) | < 0.001 |
| Middle-aged and elderly age | Prevalence | Both | Coted'Ivoire | RHD | 0.06<br>(0.05 to 0.08) | < 0.001 |
| Middle-aged and elderly age | Prevalence | Female | Coted'Ivoire | RHD | 0.1<br>(0.07 to 0.14) | < 0.001 |
| Middle-aged and elderly age | Prevalence | Male | Coted'Ivoire | RHD | 0.03<br>(0.02 to 0.05) | < 0.001 |
| Middle-aged and elderly age | Prevalence | Both | Coted'Ivoire | RA | 0.53<br>(0.5 to 0.55) | < 0.001 |
| Middle-aged and elderly age | Prevalence | Female | Coted'Ivoire | RA | 0.49<br>(0.46 to 0.51) | < 0.001 |
| Middle-aged and elderly age | Prevalence | Male | Coted'Ivoire | RA | 0.57<br>(0.54 to 0.6) | < 0.001 |
| Middle-aged and elderly age | Prevalence | Both | Croatia | AA | -0.01<br>(-0.01 to 0) | 0.008 |
| Middle-aged and elderly age | Prevalence | Female | Croatia | AA | 0<br>(0 to 0) | < 0.001 |
| Middle-aged and elderly age | Prevalence | Male | Croatia | AA | 0<br>(0 to 0) | < 0.001 |
| Middle-aged and elderly age | Prevalence | Both | Croatia | T1D | 2.35<br>(1.78 to 2.91) | < 0.001 |

| | | | | | | |
|---|---|---|---|---|---|---|
| Middle-aged and elderly age | Prevalence | Female | Croatia | T1D | 2.3<br>(1.69 to 2.91) | < 0.001 |
| Middle-aged and elderly age | Prevalence | Male | Croatia | T1D | 2.39<br>(1.88 to 2.91) | < 0.001 |
| Middle-aged and elderly age | Prevalence | Both | Croatia | IBD | 0.68<br>(0.64 to 0.73) | < 0.001 |
| Middle-aged and elderly age | Prevalence | Female | Croatia | IBD | 0.73<br>(0.68 to 0.79) | < 0.001 |
| Middle-aged and elderly age | Prevalence | Male | Croatia | IBD | 0.65<br>(0.61 to 0.7) | < 0.001 |
| Middle-aged and elderly age | Prevalence | Both | Croatia | MS | 0.97<br>(0.94 to 0.99) | < 0.001 |
| Middle-aged and elderly age | Prevalence | Female | Croatia | MS | 1.19<br>(1.13 to 1.24) | < 0.001 |
| Middle-aged and elderly age | Prevalence | Male | Croatia | MS | 0.59<br>(0.54 to 0.64) | < 0.001 |
| Middle-aged and elderly age | Prevalence | Both | Croatia | Psoriasis | -0.76<br>(-0.79 to -0.72) | < 0.001 |
| Middle-aged and elderly age | Prevalence | Female | Croatia | Psoriasis | -0.79<br>(-0.87 to -0.71) | < 0.001 |
| Middle-aged and elderly age | Prevalence | Male | Croatia | Psoriasis | -0.74<br>(-0.75 to -0.72) | < 0.001 |
| Middle-aged and elderly age | Prevalence | Both | Croatia | RHD | -1.86<br>(-2.29 to -1.42) | < 0.001 |
| Middle-aged and elderly age | Prevalence | Female | Croatia | RHD | -1.72<br>(-1.92 to -1.53) | < 0.001 |
| Middle-aged and elderly age | Prevalence | Male | Croatia | RHD | -2.26<br>(-2.63 to -1.88) | < 0.001 |
| Middle-aged and elderly age | Prevalence | Both | Croatia | RA | 0.78<br>(0.73 to 0.83) | < 0.001 |
| Middle-aged and elderly age | Prevalence | Female | Croatia | RA | 0.82<br>(0.79 to 0.85) | < 0.001 |
| Middle-aged and elderly age | Prevalence | Male | Croatia | RA | 0.75 | < 0.001 |

| | | | | | | |
|---|---|---|---|---|---|---|
| | | | | | (0.69 to 0.81) | |
| Middle-aged and elderly age | Prevalence | Both | Cuba | AA | 0.02<br>(0.02 to 0.03) | < 0.001 |
| Middle-aged and elderly age | Prevalence | Female | Cuba | AA | 0<br>(0 to 0) | < 0.001 |
| Middle-aged and elderly age | Prevalence | Male | Cuba | AA | 0<br>(0 to 0) | < 0.001 |
| Middle-aged and elderly age | Prevalence | Both | Cuba | T1D | 1.52<br>(1.41 to 1.62) | < 0.001 |
| Middle-aged and elderly age | Prevalence | Female | Cuba | T1D | 1.37<br>(1.28 to 1.46) | < 0.001 |
| Middle-aged and elderly age | Prevalence | Male | Cuba | T1D | 1.67<br>(1.55 to 1.78) | < 0.001 |
| Middle-aged and elderly age | Prevalence | Both | Cuba | IBD | 0.01<br>(-0.03 to 0.04) | 0.786 |
| Middle-aged and elderly age | Prevalence | Female | Cuba | IBD | 0.05<br>(-0.01 to 0.11) | 0.128 |
| Middle-aged and elderly age | Prevalence | Male | Cuba | IBD | -0.04<br>(-0.06 to -0.02) | < 0.001 |
| Middle-aged and elderly age | Prevalence | Both | Cuba | MS | 0.69<br>(0.66 to 0.71) | < 0.001 |
| Middle-aged and elderly age | Prevalence | Female | Cuba | MS | 0.71<br>(0.68 to 0.73) | < 0.001 |
| Middle-aged and elderly age | Prevalence | Male | Cuba | MS | 0.51<br>(0.47 to 0.55) | < 0.001 |
| Middle-aged and elderly age | Prevalence | Both | Cuba | Psoriasis | -0.2<br>(-0.22 to -0.18) | < 0.001 |
| Middle-aged and elderly age | Prevalence | Female | Cuba | Psoriasis | -0.2<br>(-0.23 to -0.17) | < 0.001 |
| Middle-aged and elderly age | Prevalence | Male | Cuba | Psoriasis | -0.2<br>(-0.21 to -0.19) | < 0.001 |
| Middle-aged and elderly age | Prevalence | Both | Cuba | RHD | 0.03<br>(0.02 to 0.05) | < 0.001 |

| | | | | | | |
|---|---|---|---|---|---|---|
| Middle-aged and elderly age | Prevalence | Female | Cuba | RHD | 0.05 (0.04 to 0.06) | < 0.001 |
| Middle-aged and elderly age | Prevalence | Male | Cuba | RHD | -0.01 (-0.04 to 0.02) | 0.451 |
| Middle-aged and elderly age | Prevalence | Both | Cuba | RA | 0.92 (0.91 to 0.94) | < 0.001 |
| Middle-aged and elderly age | Prevalence | Female | Cuba | RA | 0.84 (0.81 to 0.88) | < 0.001 |
| Middle-aged and elderly age | Prevalence | Male | Cuba | RA | 1.03 (0.94 to 1.12) | < 0.001 |
| Middle-aged and elderly age | Prevalence | Both | Cyprus | AA | 0.02 (0.01 to 0.02) | < 0.001 |
| Middle-aged and elderly age | Prevalence | Female | Cyprus | AA | 0 (0 to 0) | < 0.001 |
| Middle-aged and elderly age | Prevalence | Male | Cyprus | AA | 0 (0 to 0) | < 0.001 |
| Middle-aged and elderly age | Prevalence | Both | Cyprus | T1D | 2.89 (2.74 to 3.05) | < 0.001 |
| Middle-aged and elderly age | Prevalence | Female | Cyprus | T1D | 2.75 (2.58 to 2.92) | < 0.001 |
| Middle-aged and elderly age | Prevalence | Male | Cyprus | T1D | 3.1 (2.92 to 3.29) | < 0.001 |
| Middle-aged and elderly age | Prevalence | Both | Cyprus | IBD | 0.81 (0.73 to 0.89) | < 0.001 |
| Middle-aged and elderly age | Prevalence | Female | Cyprus | IBD | 0.72 (0.42 to 1.03) | < 0.001 |
| Middle-aged and elderly age | Prevalence | Male | Cyprus | IBD | 0.9 (0.83 to 0.97) | < 0.001 |
| Middle-aged and elderly age | Prevalence | Both | Cyprus | MS | 0.99 (0.96 to 1.02) | < 0.001 |
| Middle-aged and elderly age | Prevalence | Female | Cyprus | MS | 1.04 (1.01 to 1.07) | < 0.001 |
| Middle-aged and elderly age | Prevalence | Male | Cyprus | MS | 0.85 | < 0.001 |

| | | | | | | |
|---|---|---|---|---|---|---|
| | | | | | (0.77 to 0.93) | |
| Middle-aged and elderly age | Prevalence | Both | Cyprus | Psoriasis | -0.66 (-0.67 to -0.65) | < 0.001 |
| Middle-aged and elderly age | Prevalence | Female | Cyprus | Psoriasis | -0.66 (-0.67 to -0.64) | < 0.001 |
| Middle-aged and elderly age | Prevalence | Male | Cyprus | Psoriasis | -0.66 (-0.67 to -0.65) | < 0.001 |
| Middle-aged and elderly age | Prevalence | Both | Cyprus | RHD | -1.94 (-2.38 to -1.5) | < 0.001 |
| Middle-aged and elderly age | Prevalence | Female | Cyprus | RHD | -2.33 (-2.77 to -1.89) | < 0.001 |
| Middle-aged and elderly age | Prevalence | Male | Cyprus | RHD | -1.17 (-1.33 to -1.01) | < 0.001 |
| Middle-aged and elderly age | Prevalence | Both | Cyprus | RA | 0.77 (0.67 to 0.86) | < 0.001 |
| Middle-aged and elderly age | Prevalence | Female | Cyprus | RA | 0.74 (0.63 to 0.85) | < 0.001 |
| Middle-aged and elderly age | Prevalence | Male | Cyprus | RA | 0.77 (0.73 to 0.81) | < 0.001 |
| Middle-aged and elderly age | Prevalence | Both | Czechia | AA | -0.06 (-0.06 to -0.05) | < 0.001 |
| Middle-aged and elderly age | Prevalence | Female | Czechia | AA | 0 (0 to 0) | < 0.001 |
| Middle-aged and elderly age | Prevalence | Male | Czechia | AA | 0 (0 to 0) | < 0.001 |
| Middle-aged and elderly age | Prevalence | Both | Czechia | T1D | 1.69 (1.65 to 1.73) | < 0.001 |
| Middle-aged and elderly age | Prevalence | Female | Czechia | T1D | 1.65 (1.6 to 1.7) | < 0.001 |
| Middle-aged and elderly age | Prevalence | Male | Czechia | T1D | 1.72 (1.68 to 1.77) | < 0.001 |
| Middle-aged and elderly age | Prevalence | Both | Czechia | IBD | 1.55 (1.34 to 1.77) | < 0.001 |

| | | | | | | |
|---|---|---|---|---|---|---|
| Middle-aged and elderly age | Prevalence | Female | Czechia | IBD | 1.75<br>(1.55 to 1.96) | < 0.001 |
| Middle-aged and elderly age | Prevalence | Male | Czechia | IBD | 1.38<br>(1.2 to 1.55) | < 0.001 |
| Middle-aged and elderly age | Prevalence | Both | Czechia | MS | -0.26<br>(-0.31 to -0.21) | < 0.001 |
| Middle-aged and elderly age | Prevalence | Female | Czechia | MS | -0.1<br>(-0.16 to -0.03) | 0.003 |
| Middle-aged and elderly age | Prevalence | Male | Czechia | MS | -0.44<br>(-0.45 to -0.43) | < 0.001 |
| Middle-aged and elderly age | Prevalence | Both | Czechia | Psoriasis | -0.85<br>(-0.89 to -0.82) | < 0.001 |
| Middle-aged and elderly age | Prevalence | Female | Czechia | Psoriasis | -0.88<br>(-0.93 to -0.83) | < 0.001 |
| Middle-aged and elderly age | Prevalence | Male | Czechia | Psoriasis | -0.82<br>(-0.85 to -0.79) | < 0.001 |
| Middle-aged and elderly age | Prevalence | Both | Czechia | RHD | -2.32<br>(-2.55 to -2.09) | < 0.001 |
| Middle-aged and elderly age | Prevalence | Female | Czechia | RHD | -2.13<br>(-2.41 to -1.85) | < 0.001 |
| Middle-aged and elderly age | Prevalence | Male | Czechia | RHD | -2.53<br>(-2.74 to -2.31) | < 0.001 |
| Middle-aged and elderly age | Prevalence | Both | Czechia | RA | 0.72<br>(0.66 to 0.77) | < 0.001 |
| Middle-aged and elderly age | Prevalence | Female | Czechia | RA | 0.77<br>(0.71 to 0.83) | < 0.001 |
| Middle-aged and elderly age | Prevalence | Male | Czechia | RA | 0.78<br>(0.7 to 0.85) | < 0.001 |
| Middle-aged and elderly age | Prevalence | Both | Democratic People's Republic of Korea | AA | -0.08<br>(-0.09 to -0.07) | < 0.001 |
| Middle-aged and elderly age | Prevalence | Female | Democratic People's Republic of Korea | AA | 0<br>(0 to 0) | < 0.001 |
| Middle-aged and elderly age | Prevalence | Male | Democratic People's Republic of Korea | AA | 0 | 0.013 |

| | | | | | | |
|---|---|---|---|---|---|---|
| | | | | | (0 to 0) | |
| Middle-aged and elderly age | Prevalence | Both | Democratic People's Republic of Korea | T1D | 1.54<br>(1.5 to 1.58) | < 0.001 |
| Middle-aged and elderly age | Prevalence | Female | Democratic People's Republic of Korea | T1D | 1.5<br>(1.46 to 1.55) | < 0.001 |
| Middle-aged and elderly age | Prevalence | Male | Democratic People's Republic of Korea | T1D | 1.52<br>(1.47 to 1.57) | < 0.001 |
| Middle-aged and elderly age | Prevalence | Both | Democratic People's Republic of Korea | IBD | 0.08<br>(0.06 to 0.1) | < 0.001 |
| Middle-aged and elderly age | Prevalence | Female | Democratic People's Republic of Korea | IBD | 0.1<br>(0.07 to 0.13) | < 0.001 |
| Middle-aged and elderly age | Prevalence | Male | Democratic People's Republic of Korea | IBD | 0.03<br>(0 to 0.06) | 0.021 |
| Middle-aged and elderly age | Prevalence | Both | Democratic People's Republic of Korea | MS | 0.19<br>(0.09 to 0.29) | < 0.001 |
| Middle-aged and elderly age | Prevalence | Female | Democratic People's Republic of Korea | MS | 0.27<br>(0.21 to 0.33) | < 0.001 |
| Middle-aged and elderly age | Prevalence | Male | Democratic People's Republic of Korea | MS | 0.21<br>(0.16 to 0.25) | < 0.001 |
| Middle-aged and elderly age | Prevalence | Both | Democratic People's Republic of Korea | Psoriasis | -0.66<br>(-0.68 to -0.64) | < 0.001 |
| Middle-aged and elderly age | Prevalence | Female | Democratic People's Republic of Korea | Psoriasis | -0.72<br>(-0.74 to -0.7) | < 0.001 |
| Middle-aged and elderly age | Prevalence | Male | Democratic People's Republic of Korea | Psoriasis | -0.73<br>(-0.74 to -0.71) | < 0.001 |
| Middle-aged and elderly age | Prevalence | Both | Democratic People's Republic of Korea | RHD | 0.07<br>(0.06 to 0.08) | < 0.001 |
| Middle-aged and elderly age | Prevalence | Female | Democratic People's Republic of Korea | RHD | 0.13<br>(0.11 to 0.15) | < 0.001 |
| Middle-aged and elderly age | Prevalence | Male | Democratic People's Republic of Korea | RHD | 0.04<br>(0.03 to 0.05) | < 0.001 |
| Middle-aged and elderly age | Prevalence | Both | Democratic People's Republic of Korea | RA | -0.33<br>(-0.43 to -0.22) | < 0.001 |

| | | | | | | |
|---|---|---|---|---|---|---|
| Middle-aged and elderly age | Prevalence | Female | Democratic People's Republic of Korea | RA | -0.21 (-0.31 to -0.12) | < 0.001 |
| Middle-aged and elderly age | Prevalence | Male | Democratic People's Republic of Korea | RA | -0.13 (-0.25 to -0.01) | 0.038 |
| Middle-aged and elderly age | Prevalence | Both | Democratic Republic of the Congo | AA | -0.09 (-0.09 to -0.08) | < 0.001 |
| Middle-aged and elderly age | Prevalence | Female | Democratic Republic of the Congo | AA | 0 (0 to 0) | 0.027 |
| Middle-aged and elderly age | Prevalence | Male | Democratic Republic of the Congo | AA | 0 (0 to 0) | < 0.001 |
| Middle-aged and elderly age | Prevalence | Both | Democratic Republic of the Congo | T1D | 0.65 (0.64 to 0.67) | < 0.001 |
| Middle-aged and elderly age | Prevalence | Female | Democratic Republic of the Congo | T1D | 0.66 (0.64 to 0.68) | < 0.001 |
| Middle-aged and elderly age | Prevalence | Male | Democratic Republic of the Congo | T1D | 0.64 (0.63 to 0.66) | < 0.001 |
| Middle-aged and elderly age | Prevalence | Both | Democratic Republic of the Congo | IBD | -0.53 (-0.57 to -0.5) | < 0.001 |
| Middle-aged and elderly age | Prevalence | Female | Democratic Republic of the Congo | IBD | -0.77 (-0.87 to -0.67) | < 0.001 |
| Middle-aged and elderly age | Prevalence | Male | Democratic Republic of the Congo | IBD | -0.28 (-0.34 to -0.21) | < 0.001 |
| Middle-aged and elderly age | Prevalence | Both | Democratic Republic of the Congo | MS | 0.1 (0.07 to 0.13) | < 0.001 |
| Middle-aged and elderly age | Prevalence | Female | Democratic Republic of the Congo | MS | 0.15 (0.13 to 0.18) | < 0.001 |
| Middle-aged and elderly age | Prevalence | Male | Democratic Republic of the Congo | MS | 0.25 (0.21 to 0.28) | < 0.001 |
| Middle-aged and elderly age | Prevalence | Both | Democratic Republic of the Congo | Psoriasis | -0.65 (-0.72 to -0.58) | < 0.001 |
| Middle-aged and elderly age | Prevalence | Female | Democratic Republic of the Congo | Psoriasis | -0.65 (-0.72 to -0.57) | < 0.001 |
| Middle-aged and elderly age | Prevalence | Male | Democratic Republic of the Congo | Psoriasis | -0.64 | < 0.001 |

| | | | | | | |
|---|---|---|---|---|---|---|
| | | | | | (-0.71 to -0.57) | |
| Middle-aged and elderly age | Prevalence | Both | Democratic Republic of the Congo | RHD | -0.08<br>(-0.09 to -0.07) | < 0.001 |
| Middle-aged and elderly age | Prevalence | Female | Democratic Republic of the Congo | RHD | -0.02<br>(-0.04 to 0) | 0.014 |
| Middle-aged and elderly age | Prevalence | Male | Democratic Republic of the Congo | RHD | -0.11<br>(-0.14 to -0.09) | < 0.001 |
| Middle-aged and elderly age | Prevalence | Both | Democratic Republic of the Congo | RA | 0.38<br>(0.32 to 0.43) | < 0.001 |
| Middle-aged and elderly age | Prevalence | Female | Democratic Republic of the Congo | RA | 0.43<br>(0.38 to 0.48) | < 0.001 |
| Middle-aged and elderly age | Prevalence | Male | Democratic Republic of the Congo | RA | 0.42<br>(0.28 to 0.55) | < 0.001 |
| Middle-aged and elderly age | Prevalence | Both | Denmark | AA | -0.02<br>(-0.03 to -0.02) | < 0.001 |
| Middle-aged and elderly age | Prevalence | Female | Denmark | AA | 0<br>(0 to 0) | < 0.001 |
| Middle-aged and elderly age | Prevalence | Male | Denmark | AA | 0<br>(0 to 0) | < 0.001 |
| Middle-aged and elderly age | Prevalence | Both | Denmark | T1D | 1.76<br>(1.71 to 1.81) | < 0.001 |
| Middle-aged and elderly age | Prevalence | Female | Denmark | T1D | 1.79<br>(1.7 to 1.87) | < 0.001 |
| Middle-aged and elderly age | Prevalence | Male | Denmark | T1D | 1.73<br>(1.69 to 1.77) | < 0.001 |
| Middle-aged and elderly age | Prevalence | Both | Denmark | IBD | 0.02<br>(-0.07 to 0.12) | 0.624 |
| Middle-aged and elderly age | Prevalence | Female | Denmark | IBD | 0.02<br>(-0.08 to 0.13) | 0.655 |
| Middle-aged and elderly age | Prevalence | Male | Denmark | IBD | 0.02<br>(-0.06 to 0.11) | 0.618 |
| Middle-aged and elderly age | Prevalence | Both | Denmark | MS | 0.32<br>(0.27 to 0.36) | < 0.001 |

| | | | | | | |
|---|---|---|---|---|---|---|
| Middle-aged and elderly age | Prevalence | Female | Denmark | MS | 0.48<br>(0.44 to 0.52) | < 0.001 |
| Middle-aged and elderly age | Prevalence | Male | Denmark | MS | 0.08<br>(0.03 to 0.13) | 0.001 |
| Middle-aged and elderly age | Prevalence | Both | Denmark | Psoriasis | -0.35<br>(-0.35 to -0.34) | < 0.001 |
| Middle-aged and elderly age | Prevalence | Female | Denmark | Psoriasis | -0.34<br>(-0.35 to -0.34) | < 0.001 |
| Middle-aged and elderly age | Prevalence | Male | Denmark | Psoriasis | -0.35<br>(-0.36 to -0.34) | < 0.001 |
| Middle-aged and elderly age | Prevalence | Both | Denmark | RHD | -2.21<br>(-2.56 to -1.85) | < 0.001 |
| Middle-aged and elderly age | Prevalence | Female | Denmark | RHD | -1.98<br>(-2.28 to -1.68) | < 0.001 |
| Middle-aged and elderly age | Prevalence | Male | Denmark | RHD | -2.2<br>(-2.51 to -1.9) | < 0.001 |
| Middle-aged and elderly age | Prevalence | Both | Denmark | RA | 0.4<br>(0.36 to 0.44) | < 0.001 |
| Middle-aged and elderly age | Prevalence | Female | Denmark | RA | 0.45<br>(0.41 to 0.49) | < 0.001 |
| Middle-aged and elderly age | Prevalence | Male | Denmark | RA | 0.36<br>(0.31 to 0.41) | < 0.001 |
| Middle-aged and elderly age | Prevalence | Both | Djibouti | AA | -0.08<br>(-0.09 to -0.06) | < 0.001 |
| Middle-aged and elderly age | Prevalence | Female | Djibouti | AA | 0<br>(0 to 0) | 1.000 |
| Middle-aged and elderly age | Prevalence | Male | Djibouti | AA | 0<br>(0 to 0) | < 0.001 |
| Middle-aged and elderly age | Prevalence | Both | Djibouti | T1D | 0.58<br>(0.57 to 0.59) | < 0.001 |
| Middle-aged and elderly age | Prevalence | Female | Djibouti | T1D | 0.58<br>(0.56 to 0.6) | < 0.001 |
| Middle-aged and elderly age | Prevalence | Male | Djibouti | T1D | 0.57 | < 0.001 |

| | | | | | | |
|---|---|---|---|---|---|---|
| | | | | | (0.56 to 0.58) | |
| Middle-aged and elderly age | Prevalence | Both | Djibouti | IBD | 0.73 (0.63 to 0.83) | < 0.001 |
| Middle-aged and elderly age | Prevalence | Female | Djibouti | IBD | 0.47 (0.37 to 0.57) | < 0.001 |
| Middle-aged and elderly age | Prevalence | Male | Djibouti | IBD | 0.91 (0.82 to 1.01) | < 0.001 |
| Middle-aged and elderly age | Prevalence | Both | Djibouti | MS | 0.13 (0.1 to 0.15) | < 0.001 |
| Middle-aged and elderly age | Prevalence | Female | Djibouti | MS | 0.17 (0.14 to 0.2) | < 0.001 |
| Middle-aged and elderly age | Prevalence | Male | Djibouti | MS | 0.22 (0.19 to 0.26) | < 0.001 |
| Middle-aged and elderly age | Prevalence | Both | Djibouti | Psoriasis | -0.44 (-0.46 to -0.41) | < 0.001 |
| Middle-aged and elderly age | Prevalence | Female | Djibouti | Psoriasis | -0.44 (-0.46 to -0.42) | < 0.001 |
| Middle-aged and elderly age | Prevalence | Male | Djibouti | Psoriasis | -0.42 (-0.45 to -0.39) | < 0.001 |
| Middle-aged and elderly age | Prevalence | Both | Djibouti | RHD | 0.13 (0.11 to 0.15) | < 0.001 |
| Middle-aged and elderly age | Prevalence | Female | Djibouti | RHD | 0.19 (0.15 to 0.22) | < 0.001 |
| Middle-aged and elderly age | Prevalence | Male | Djibouti | RHD | 0.11 (0.09 to 0.13) | < 0.001 |
| Middle-aged and elderly age | Prevalence | Both | Djibouti | RA | 0.8 (0.7 to 0.89) | < 0.001 |
| Middle-aged and elderly age | Prevalence | Female | Djibouti | RA | 0.72 (0.63 to 0.81) | < 0.001 |
| Middle-aged and elderly age | Prevalence | Male | Djibouti | RA | 0.77 (0.68 to 0.86) | < 0.001 |
| Middle-aged and elderly age | Prevalence | Both | Dominica | AA | -0.3 (-0.32 to -0.28) | < 0.001 |

| | | | | | | |
|---|---|---|---|---|---|---|
| Middle-aged and elderly age | Prevalence | Female | Dominica | AA | 0<br>(0 to 0) | < 0.001 |
| Middle-aged and elderly age | Prevalence | Male | Dominica | AA | 0<br>(0 to 0) | < 0.001 |
| Middle-aged and elderly age | Prevalence | Both | Dominica | T1D | 1.36<br>(1.3 to 1.41) | < 0.001 |
| Middle-aged and elderly age | Prevalence | Female | Dominica | T1D | 1.17<br>(1.08 to 1.26) | < 0.001 |
| Middle-aged and elderly age | Prevalence | Male | Dominica | T1D | 1.31<br>(1.29 to 1.33) | < 0.001 |
| Middle-aged and elderly age | Prevalence | Both | Dominica | IBD | 0.02<br>(0 to 0.03) | 0.014 |
| Middle-aged and elderly age | Prevalence | Female | Dominica | IBD | 0.06<br>(0.02 to 0.09) | 0.001 |
| Middle-aged and elderly age | Prevalence | Male | Dominica | IBD | -0.01<br>(-0.02 to -0.01) | 0.001 |
| Middle-aged and elderly age | Prevalence | Both | Dominica | MS | 0.14<br>(0.09 to 0.18) | < 0.001 |
| Middle-aged and elderly age | Prevalence | Female | Dominica | MS | 0.51<br>(0.44 to 0.58) | < 0.001 |
| Middle-aged and elderly age | Prevalence | Male | Dominica | MS | 0.33<br>(0.32 to 0.35) | < 0.001 |
| Middle-aged and elderly age | Prevalence | Both | Dominica | Psoriasis | -0.48<br>(-0.5 to -0.45) | < 0.001 |
| Middle-aged and elderly age | Prevalence | Female | Dominica | Psoriasis | -0.46<br>(-0.47 to -0.44) | < 0.001 |
| Middle-aged and elderly age | Prevalence | Male | Dominica | Psoriasis | -0.46<br>(-0.49 to -0.42) | < 0.001 |
| Middle-aged and elderly age | Prevalence | Both | Dominica | RHD | -0.07<br>(-0.09 to -0.05) | < 0.001 |
| Middle-aged and elderly age | Prevalence | Female | Dominica | RHD | 0.07<br>(0.05 to 0.09) | < 0.001 |
| Middle-aged and elderly age | Prevalence | Male | Dominica | RHD | 0 | 0.922 |

| | | | | | | |
|---|---|---|---|---|---|---|
| | | | | | (-0.02 to 0.02) | |
| Middle-aged and elderly age | Prevalence | Both | Dominica | RA | 0.03<br>(0.01 to 0.06) | 0.006 |
| Middle-aged and elderly age | Prevalence | Female | Dominica | RA | 0.39<br>(0.36 to 0.42) | < 0.001 |
| Middle-aged and elderly age | Prevalence | Male | Dominica | RA | 0.49<br>(0.45 to 0.52) | < 0.001 |
| Middle-aged and elderly age | Prevalence | Both | Dominican Republic | AA | -0.01<br>(-0.02 to 0) | 0.005 |
| Middle-aged and elderly age | Prevalence | Female | Dominican Republic | AA | 0<br>(0 to 0) | < 0.001 |
| Middle-aged and elderly age | Prevalence | Male | Dominican Republic | AA | 0<br>(0 to 0) | < 0.001 |
| Middle-aged and elderly age | Prevalence | Both | Dominican Republic | T1D | 1.08<br>(1.07 to 1.08) | < 0.001 |
| Middle-aged and elderly age | Prevalence | Female | Dominican Republic | T1D | 1.01<br>(1.01 to 1.02) | < 0.001 |
| Middle-aged and elderly age | Prevalence | Male | Dominican Republic | T1D | 1.13<br>(1.12 to 1.14) | < 0.001 |
| Middle-aged and elderly age | Prevalence | Both | Dominican Republic | IBD | 0.03<br>(-0.03 to 0.09) | 0.346 |
| Middle-aged and elderly age | Prevalence | Female | Dominican Republic | IBD | 0.06<br>(-0.02 to 0.14) | 0.118 |
| Middle-aged and elderly age | Prevalence | Male | Dominican Republic | IBD | -0.03<br>(-0.11 to 0.05) | 0.485 |
| Middle-aged and elderly age | Prevalence | Both | Dominican Republic | MS | 0.6<br>(0.55 to 0.66) | < 0.001 |
| Middle-aged and elderly age | Prevalence | Female | Dominican Republic | MS | 0.64<br>(0.58 to 0.7) | < 0.001 |
| Middle-aged and elderly age | Prevalence | Male | Dominican Republic | MS | 0.55<br>(0.52 to 0.58) | < 0.001 |
| Middle-aged and elderly age | Prevalence | Both | Dominican Republic | Psoriasis | -0.55<br>(-0.59 to -0.51) | < 0.001 |

| | | | | | | |
|---|---|---|---|---|---|---|
| Middle-aged and elderly age | Prevalence | Female | Dominican Republic | Psoriasis | -0.54<br>(-0.57 to -0.51) | < 0.001 |
| Middle-aged and elderly age | Prevalence | Male | Dominican Republic | Psoriasis | -0.52<br>(-0.57 to -0.47) | < 0.001 |
| Middle-aged and elderly age | Prevalence | Both | Dominican Republic | RHD | 0.1<br>(0.09 to 0.12) | < 0.001 |
| Middle-aged and elderly age | Prevalence | Female | Dominican Republic | RHD | 0.13<br>(0.1 to 0.16) | < 0.001 |
| Middle-aged and elderly age | Prevalence | Male | Dominican Republic | RHD | 0.08<br>(0.05 to 0.11) | < 0.001 |
| Middle-aged and elderly age | Prevalence | Both | Dominican Republic | RA | 1.03<br>(1 to 1.07) | < 0.001 |
| Middle-aged and elderly age | Prevalence | Female | Dominican Republic | RA | 1.08<br>(1.01 to 1.15) | < 0.001 |
| Middle-aged and elderly age | Prevalence | Male | Dominican Republic | RA | 1.01<br>(0.98 to 1.03) | < 0.001 |
| Middle-aged and elderly age | Prevalence | Both | Ecuador | AA | 0.02<br>(0.02 to 0.02) | < 0.001 |
| Middle-aged and elderly age | Prevalence | Female | Ecuador | AA | 0<br>(0 to 0) | < 0.001 |
| Middle-aged and elderly age | Prevalence | Male | Ecuador | AA | 0<br>(0 to 0) | < 0.001 |
| Middle-aged and elderly age | Prevalence | Both | Ecuador | T1D | 0.85<br>(0.82 to 0.88) | < 0.001 |
| Middle-aged and elderly age | Prevalence | Female | Ecuador | T1D | 0.83<br>(0.8 to 0.86) | < 0.001 |
| Middle-aged and elderly age | Prevalence | Male | Ecuador | T1D | 0.85<br>(0.81 to 0.89) | < 0.001 |
| Middle-aged and elderly age | Prevalence | Both | Ecuador | IBD | 0.58<br>(0.4 to 0.77) | < 0.001 |
| Middle-aged and elderly age | Prevalence | Female | Ecuador | IBD | 0.79<br>(0.62 to 0.97) | < 0.001 |
| Middle-aged and elderly age | Prevalence | Male | Ecuador | IBD | 0.41 | < 0.001 |

| | | | | | | |
|---|---|---|---|---|---|---|
| | | | | | (0.2 to 0.62) | |
| Middle-aged and elderly age | Prevalence | Both | Ecuador | MS | 1.03 (1 to 1.07) | < 0.001 |
| Middle-aged and elderly age | Prevalence | Female | Ecuador | MS | 1.1 (1.07 to 1.14) | < 0.001 |
| Middle-aged and elderly age | Prevalence | Male | Ecuador | MS | 0.79 (0.75 to 0.83) | < 0.001 |
| Middle-aged and elderly age | Prevalence | Both | Ecuador | Psoriasis | -0.66 (-0.74 to -0.59) | < 0.001 |
| Middle-aged and elderly age | Prevalence | Female | Ecuador | Psoriasis | -0.66 (-0.74 to -0.59) | < 0.001 |
| Middle-aged and elderly age | Prevalence | Male | Ecuador | Psoriasis | -0.66 (-0.73 to -0.59) | < 0.001 |
| Middle-aged and elderly age | Prevalence | Both | Ecuador | RHD | 0.08 (0.07 to 0.08) | < 0.001 |
| Middle-aged and elderly age | Prevalence | Female | Ecuador | RHD | 0.1 (0.09 to 0.11) | < 0.001 |
| Middle-aged and elderly age | Prevalence | Male | Ecuador | RHD | 0.02 (0 to 0.03) | 0.008 |
| Middle-aged and elderly age | Prevalence | Both | Ecuador | RA | 1.56 (1.52 to 1.6) | < 0.001 |
| Middle-aged and elderly age | Prevalence | Female | Ecuador | RA | 1.55 (1.51 to 1.59) | < 0.001 |
| Middle-aged and elderly age | Prevalence | Male | Ecuador | RA | 1.44 (1.41 to 1.46) | < 0.001 |
| Middle-aged and elderly age | Prevalence | Both | Egypt | AA | -0.03 (-0.04 to -0.03) | < 0.001 |
| Middle-aged and elderly age | Prevalence | Female | Egypt | AA | 0 (0 to 0) | < 0.001 |
| Middle-aged and elderly age | Prevalence | Male | Egypt | AA | 0 (0 to 0) | < 0.001 |
| Middle-aged and elderly age | Prevalence | Both | Egypt | T1D | 1.59 (1.53 to 1.64) | < 0.001 |

| | | | | | | |
|---|---|---|---|---|---|---|
| Middle-aged and elderly age | Prevalence | Female | Egypt | T1D | 1.71 (1.58 to 1.84) | < 0.001 |
| Middle-aged and elderly age | Prevalence | Male | Egypt | T1D | 1.46 (1.31 to 1.61) | < 0.001 |
| Middle-aged and elderly age | Prevalence | Both | Egypt | IBD | 0.82 (0.77 to 0.88) | < 0.001 |
| Middle-aged and elderly age | Prevalence | Female | Egypt | IBD | 0.79 (0.73 to 0.85) | < 0.001 |
| Middle-aged and elderly age | Prevalence | Male | Egypt | IBD | 0.84 (0.77 to 0.91) | < 0.001 |
| Middle-aged and elderly age | Prevalence | Both | Egypt | MS | 0.72 (0.63 to 0.82) | < 0.001 |
| Middle-aged and elderly age | Prevalence | Female | Egypt | MS | 0.6 (0.42 to 0.79) | < 0.001 |
| Middle-aged and elderly age | Prevalence | Male | Egypt | MS | 1.09 (1.05 to 1.12) | < 0.001 |
| Middle-aged and elderly age | Prevalence | Both | Egypt | Psoriasis | -0.81 (-0.89 to -0.73) | < 0.001 |
| Middle-aged and elderly age | Prevalence | Female | Egypt | Psoriasis | -0.74 (-0.82 to -0.65) | < 0.001 |
| Middle-aged and elderly age | Prevalence | Male | Egypt | Psoriasis | -0.88 (-0.93 to -0.83) | < 0.001 |
| Middle-aged and elderly age | Prevalence | Both | Egypt | RHD | 0.16 (0.1 to 0.21) | < 0.001 |
| Middle-aged and elderly age | Prevalence | Female | Egypt | RHD | 0.2 (0.16 to 0.25) | < 0.001 |
| Middle-aged and elderly age | Prevalence | Male | Egypt | RHD | 0.2 (0.16 to 0.24) | < 0.001 |
| Middle-aged and elderly age | Prevalence | Both | Egypt | RA | 0.97 (0.89 to 1.05) | < 0.001 |
| Middle-aged and elderly age | Prevalence | Female | Egypt | RA | 1.05 (0.98 to 1.12) | < 0.001 |
| Middle-aged and elderly age | Prevalence | Male | Egypt | RA | 0.95 | < 0.001 |

| | | | | | | |
|---|---|---|---|---|---|---|
| | | | | | (0.86 to 1.04) | |
| Middle-aged and elderly age | Prevalence | Both | El Salvador | AA | 0.11<br>(0.1 to 0.11) | < 0.001 |
| Middle-aged and elderly age | Prevalence | Female | El Salvador | AA | 0<br>(0 to 0) | < 0.001 |
| Middle-aged and elderly age | Prevalence | Male | El Salvador | AA | 0<br>(0 to 0) | < 0.001 |
| Middle-aged and elderly age | Prevalence | Both | El Salvador | T1D | 0.76<br>(0.75 to 0.76) | < 0.001 |
| Middle-aged and elderly age | Prevalence | Female | El Salvador | T1D | 0.75<br>(0.74 to 0.77) | < 0.001 |
| Middle-aged and elderly age | Prevalence | Male | El Salvador | T1D | 0.75<br>(0.74 to 0.77) | < 0.001 |
| Middle-aged and elderly age | Prevalence | Both | El Salvador | IBD | 0.11<br>(0.09 to 0.12) | < 0.001 |
| Middle-aged and elderly age | Prevalence | Female | El Salvador | IBD | 0.18<br>(0.16 to 0.19) | < 0.001 |
| Middle-aged and elderly age | Prevalence | Male | El Salvador | IBD | 0.12<br>(0.1 to 0.15) | < 0.001 |
| Middle-aged and elderly age | Prevalence | Both | El Salvador | MS | 0.93<br>(0.91 to 0.96) | < 0.001 |
| Middle-aged and elderly age | Prevalence | Female | El Salvador | MS | 0.83<br>(0.79 to 0.86) | < 0.001 |
| Middle-aged and elderly age | Prevalence | Male | El Salvador | MS | 0.73<br>(0.7 to 0.76) | < 0.001 |
| Middle-aged and elderly age | Prevalence | Both | El Salvador | Psoriasis | -0.56<br>(-0.58 to -0.54) | < 0.001 |
| Middle-aged and elderly age | Prevalence | Female | El Salvador | Psoriasis | -0.57<br>(-0.59 to -0.55) | < 0.001 |
| Middle-aged and elderly age | Prevalence | Male | El Salvador | Psoriasis | -0.56<br>(-0.57 to -0.54) | < 0.001 |
| Middle-aged and elderly age | Prevalence | Both | El Salvador | RHD | 0.19<br>(0.16 to 0.21) | < 0.001 |

| | | | | | | |
|---|---|---|---|---|---|---|
| Middle-aged and elderly age | Prevalence | Female | El Salvador | RHD | 0.14<br>(0.12 to 0.16) | < 0.001 |
| Middle-aged and elderly age | Prevalence | Male | El Salvador | RHD | 0.1<br>(0.05 to 0.14) | < 0.001 |
| Middle-aged and elderly age | Prevalence | Both | El Salvador | RA | 1.23<br>(1.17 to 1.29) | < 0.001 |
| Middle-aged and elderly age | Prevalence | Female | El Salvador | RA | 1.14<br>(1.09 to 1.18) | < 0.001 |
| Middle-aged and elderly age | Prevalence | Male | El Salvador | RA | 1.02<br>(0.93 to 1.1) | < 0.001 |
| Middle-aged and elderly age | Prevalence | Both | Equatorial Guinea | AA | 0.08<br>(0.07 to 0.09) | < 0.001 |
| Middle-aged and elderly age | Prevalence | Female | Equatorial Guinea | AA | 0<br>(0 to 0) | 0.027 |
| Middle-aged and elderly age | Prevalence | Male | Equatorial Guinea | AA | 0<br>(0 to 0) | < 0.001 |
| Middle-aged and elderly age | Prevalence | Both | Equatorial Guinea | T1D | 0.73<br>(0.72 to 0.74) | < 0.001 |
| Middle-aged and elderly age | Prevalence | Female | Equatorial Guinea | T1D | 0.74<br>(0.73 to 0.75) | < 0.001 |
| Middle-aged and elderly age | Prevalence | Male | Equatorial Guinea | T1D | 0.74<br>(0.73 to 0.74) | < 0.001 |
| Middle-aged and elderly age | Prevalence | Both | Equatorial Guinea | IBD | 1.3<br>(1.08 to 1.53) | < 0.001 |
| Middle-aged and elderly age | Prevalence | Female | Equatorial Guinea | IBD | 1.08<br>(0.89 to 1.27) | < 0.001 |
| Middle-aged and elderly age | Prevalence | Male | Equatorial Guinea | IBD | 1.57<br>(1.44 to 1.71) | < 0.001 |
| Middle-aged and elderly age | Prevalence | Both | Equatorial Guinea | MS | 0.48<br>(0.47 to 0.5) | < 0.001 |
| Middle-aged and elderly age | Prevalence | Female | Equatorial Guinea | MS | 0.35<br>(0.29 to 0.41) | < 0.001 |
| Middle-aged and elderly age | Prevalence | Male | Equatorial Guinea | MS | 0.51 | < 0.001 |

| | | | | | | |
|---|---|---|---|---|---|---|
| | | | | | (0.49 to 0.53) | |
| Middle-aged and elderly age | Prevalence | Both | Equatorial Guinea | Psoriasis | -2.23 (-2.29 to -2.18) | < 0.001 |
| Middle-aged and elderly age | Prevalence | Female | Equatorial Guinea | Psoriasis | -2.25 (-2.31 to -2.19) | < 0.001 |
| Middle-aged and elderly age | Prevalence | Male | Equatorial Guinea | Psoriasis | -2.23 (-2.29 to -2.17) | < 0.001 |
| Middle-aged and elderly age | Prevalence | Both | Equatorial Guinea | RHD | 0.09 (0.08 to 0.1) | < 0.001 |
| Middle-aged and elderly age | Prevalence | Female | Equatorial Guinea | RHD | 0.12 (0.1 to 0.14) | < 0.001 |
| Middle-aged and elderly age | Prevalence | Male | Equatorial Guinea | RHD | 0.02 (0 to 0.03) | 0.013 |
| Middle-aged and elderly age | Prevalence | Both | Equatorial Guinea | RA | 1.56 (1.38 to 1.73) | < 0.001 |
| Middle-aged and elderly age | Prevalence | Female | Equatorial Guinea | RA | 1.63 (1.49 to 1.76) | < 0.001 |
| Middle-aged and elderly age | Prevalence | Male | Equatorial Guinea | RA | 1.39 (1.17 to 1.62) | < 0.001 |
| Middle-aged and elderly age | Prevalence | Both | Eritrea | AA | -0.03 (-0.03 to -0.02) | < 0.001 |
| Middle-aged and elderly age | Prevalence | Female | Eritrea | AA | 0 (0 to 0) | 1.000 |
| Middle-aged and elderly age | Prevalence | Male | Eritrea | AA | 0 (0 to 0) | < 0.001 |
| Middle-aged and elderly age | Prevalence | Both | Eritrea | T1D | 0.5 (0.49 to 0.51) | < 0.001 |
| Middle-aged and elderly age | Prevalence | Female | Eritrea | T1D | 0.49 (0.48 to 0.5) | < 0.001 |
| Middle-aged and elderly age | Prevalence | Male | Eritrea | T1D | 0.51 (0.51 to 0.51) | < 0.001 |
| Middle-aged and elderly age | Prevalence | Both | Eritrea | IBD | 0.17 (0.1 to 0.25) | < 0.001 |

| | | | | | | |
|---|---|---|---|---|---|---|
| Middle-aged and elderly age | Prevalence | Female | Eritrea | IBD | 0<br>(-0.05 to 0.06) | 0.933 |
| Middle-aged and elderly age | Prevalence | Male | Eritrea | IBD | 0.38<br>(0.35 to 0.4) | < 0.001 |
| Middle-aged and elderly age | Prevalence | Both | Eritrea | MS | 0.28<br>(0.24 to 0.32) | < 0.001 |
| Middle-aged and elderly age | Prevalence | Female | Eritrea | MS | 0.29<br>(0.25 to 0.32) | < 0.001 |
| Middle-aged and elderly age | Prevalence | Male | Eritrea | MS | 0.35<br>(0.3 to 0.4) | < 0.001 |
| Middle-aged and elderly age | Prevalence | Both | Eritrea | Psoriasis | -0.46<br>(-0.48 to -0.44) | < 0.001 |
| Middle-aged and elderly age | Prevalence | Female | Eritrea | Psoriasis | -0.47<br>(-0.52 to -0.42) | < 0.001 |
| Middle-aged and elderly age | Prevalence | Male | Eritrea | Psoriasis | -0.45<br>(-0.48 to -0.42) | < 0.001 |
| Middle-aged and elderly age | Prevalence | Both | Eritrea | RHD | 0.03<br>(-0.05 to 0.1) | 0.507 |
| Middle-aged and elderly age | Prevalence | Female | Eritrea | RHD | 0.06<br>(0 to 0.13) | 0.057 |
| Middle-aged and elderly age | Prevalence | Male | Eritrea | RHD | 0.01<br>(-0.07 to 0.1) | 0.789 |
| Middle-aged and elderly age | Prevalence | Both | Eritrea | RA | 0.92<br>(0.74 to 1.1) | < 0.001 |
| Middle-aged and elderly age | Prevalence | Female | Eritrea | RA | 0.89<br>(0.76 to 1.03) | < 0.001 |
| Middle-aged and elderly age | Prevalence | Male | Eritrea | RA | 0.91<br>(0.81 to 1.01) | < 0.001 |
| Middle-aged and elderly age | Prevalence | Both | Estonia | AA | -0.05<br>(-0.06 to -0.05) | < 0.001 |
| Middle-aged and elderly age | Prevalence | Female | Estonia | AA | 0<br>(0 to 0) | < 0.001 |
| Middle-aged and elderly age | Prevalence | Male | Estonia | AA | 0 | < 0.001 |

| | | | | | | |
|---|---|---|---|---|---|---|
| | | | | | (0 to 0) | |
| Middle-aged and elderly age | Prevalence | Both | Estonia | T1D | 1.36 (1.18 to 1.55) | < 0.001 |
| Middle-aged and elderly age | Prevalence | Female | Estonia | T1D | 1.24 (1.04 to 1.44) | < 0.001 |
| Middle-aged and elderly age | Prevalence | Male | Estonia | T1D | 1.43 (1.33 to 1.53) | < 0.001 |
| Middle-aged and elderly age | Prevalence | Both | Estonia | IBD | 1.46 (1.21 to 1.72) | < 0.001 |
| Middle-aged and elderly age | Prevalence | Female | Estonia | IBD | 1.46 (1.24 to 1.67) | < 0.001 |
| Middle-aged and elderly age | Prevalence | Male | Estonia | IBD | 1.47 (1.17 to 1.78) | < 0.001 |
| Middle-aged and elderly age | Prevalence | Both | Estonia | MS | -0.13 (-0.16 to -0.1) | < 0.001 |
| Middle-aged and elderly age | Prevalence | Female | Estonia | MS | -0.01 (-0.06 to 0.04) | 0.702 |
| Middle-aged and elderly age | Prevalence | Male | Estonia | MS | -0.24 (-0.27 to -0.21) | < 0.001 |
| Middle-aged and elderly age | Prevalence | Both | Estonia | Psoriasis | -0.83 (-0.85 to -0.81) | < 0.001 |
| Middle-aged and elderly age | Prevalence | Female | Estonia | Psoriasis | -0.82 (-0.84 to -0.81) | < 0.001 |
| Middle-aged and elderly age | Prevalence | Male | Estonia | Psoriasis | -0.83 (-0.85 to -0.8) | < 0.001 |
| Middle-aged and elderly age | Prevalence | Both | Estonia | RHD | -2.04 (-2.13 to -1.95) | < 0.001 |
| Middle-aged and elderly age | Prevalence | Female | Estonia | RHD | -1.88 (-1.99 to -1.78) | < 0.001 |
| Middle-aged and elderly age | Prevalence | Male | Estonia | RHD | -2.28 (-2.41 to -2.15) | < 0.001 |
| Middle-aged and elderly age | Prevalence | Both | Estonia | RA | 0.71 (0.58 to 0.83) | < 0.001 |

| | | | | | | |
|---|---|---|---|---|---|---|
| Middle-aged and elderly age | Prevalence | Female | Estonia | RA | 0.83 (0.72 to 0.95) | < 0.001 |
| Middle-aged and elderly age | Prevalence | Male | Estonia | RA | 0.68 (0.55 to 0.8) | < 0.001 |
| Middle-aged and elderly age | Prevalence | Both | Eswatini | AA | 0.11 (0.1 to 0.11) | < 0.001 |
| Middle-aged and elderly age | Prevalence | Female | Eswatini | AA | 0 (0 to 0) | 0.027 |
| Middle-aged and elderly age | Prevalence | Male | Eswatini | AA | 0 (0 to 0) | < 0.001 |
| Middle-aged and elderly age | Prevalence | Both | Eswatini | T1D | 0.44 (0.43 to 0.45) | < 0.001 |
| Middle-aged and elderly age | Prevalence | Female | Eswatini | T1D | 0.45 (0.44 to 0.47) | < 0.001 |
| Middle-aged and elderly age | Prevalence | Male | Eswatini | T1D | 0.44 (0.41 to 0.47) | < 0.001 |
| Middle-aged and elderly age | Prevalence | Both | Eswatini | IBD | 0.52 (0.45 to 0.58) | < 0.001 |
| Middle-aged and elderly age | Prevalence | Female | Eswatini | IBD | 0.46 (0.38 to 0.53) | < 0.001 |
| Middle-aged and elderly age | Prevalence | Male | Eswatini | IBD | 0.58 (0.55 to 0.61) | < 0.001 |
| Middle-aged and elderly age | Prevalence | Both | Eswatini | MS | 0.34 (0.26 to 0.42) | < 0.001 |
| Middle-aged and elderly age | Prevalence | Female | Eswatini | MS | 0.16 (0.11 to 0.21) | < 0.001 |
| Middle-aged and elderly age | Prevalence | Male | Eswatini | MS | 0.32 (0.28 to 0.35) | < 0.001 |
| Middle-aged and elderly age | Prevalence | Both | Eswatini | Psoriasis | -0.6 (-0.68 to -0.53) | < 0.001 |
| Middle-aged and elderly age | Prevalence | Female | Eswatini | Psoriasis | -0.64 (-0.68 to -0.6) | < 0.001 |
| Middle-aged and elderly age | Prevalence | Male | Eswatini | Psoriasis | -0.57 | < 0.001 |

| | | | | | | |
|---|---|---|---|---|---|---|
| | | | | | (-0.61 to -0.52) | |
| Middle-aged and elderly age | Prevalence | Both | Eswatini | RHD | 0.06<br>(0.04 to 0.08) | < 0.001 |
| Middle-aged and elderly age | Prevalence | Female | Eswatini | RHD | 0.04<br>(-0.01 to 0.08) | 0.091 |
| Middle-aged and elderly age | Prevalence | Male | Eswatini | RHD | 0.01<br>(0.01 to 0.02) | < 0.001 |
| Middle-aged and elderly age | Prevalence | Both | Eswatini | RA | 0.44<br>(0.34 to 0.54) | < 0.001 |
| Middle-aged and elderly age | Prevalence | Female | Eswatini | RA | 0.36<br>(0.25 to 0.47) | < 0.001 |
| Middle-aged and elderly age | Prevalence | Male | Eswatini | RA | 0.39<br>(0.3 to 0.48) | < 0.001 |
| Middle-aged and elderly age | Prevalence | Both | Ethiopia | AA | 0<br>(-0.01 to 0.02) | 0.411 |
| Middle-aged and elderly age | Prevalence | Female | Ethiopia | AA | 0<br>(0 to 0) | < 0.001 |
| Middle-aged and elderly age | Prevalence | Male | Ethiopia | AA | 0<br>(0 to 0) | < 0.001 |
| Middle-aged and elderly age | Prevalence | Both | Ethiopia | T1D | 0.38<br>(0.37 to 0.38) | < 0.001 |
| Middle-aged and elderly age | Prevalence | Female | Ethiopia | T1D | 0.39<br>(0.36 to 0.43) | < 0.001 |
| Middle-aged and elderly age | Prevalence | Male | Ethiopia | T1D | 0.36<br>(0.35 to 0.37) | < 0.001 |
| Middle-aged and elderly age | Prevalence | Both | Ethiopia | IBD | 0.33<br>(0.29 to 0.37) | < 0.001 |
| Middle-aged and elderly age | Prevalence | Female | Ethiopia | IBD | 0.18<br>(0.12 to 0.24) | < 0.001 |
| Middle-aged and elderly age | Prevalence | Male | Ethiopia | IBD | 0.5<br>(0.45 to 0.55) | < 0.001 |
| Middle-aged and elderly age | Prevalence | Both | Ethiopia | MS | 0.21<br>(0.16 to 0.26) | < 0.001 |

| | | | | | | |
|---|---|---|---|---|---|---|
| Middle-aged and elderly age | Prevalence | Female | Ethiopia | MS | 0.18<br>(0.14 to 0.21) | < 0.001 |
| Middle-aged and elderly age | Prevalence | Male | Ethiopia | MS | 0.25<br>(0.2 to 0.3) | < 0.001 |
| Middle-aged and elderly age | Prevalence | Both | Ethiopia | Psoriasis | -0.44<br>(-0.47 to -0.4) | < 0.001 |
| Middle-aged and elderly age | Prevalence | Female | Ethiopia | Psoriasis | -0.47<br>(-0.51 to -0.42) | < 0.001 |
| Middle-aged and elderly age | Prevalence | Male | Ethiopia | Psoriasis | -0.44<br>(-0.47 to -0.4) | < 0.001 |
| Middle-aged and elderly age | Prevalence | Both | Ethiopia | RHD | 0.23<br>(0.21 to 0.25) | < 0.001 |
| Middle-aged and elderly age | Prevalence | Female | Ethiopia | RHD | 0.22<br>(0.14 to 0.3) | < 0.001 |
| Middle-aged and elderly age | Prevalence | Male | Ethiopia | RHD | 0.25<br>(0.2 to 0.29) | < 0.001 |
| Middle-aged and elderly age | Prevalence | Both | Ethiopia | RA | 0.18<br>(0.1 to 0.27) | < 0.001 |
| Middle-aged and elderly age | Prevalence | Female | Ethiopia | RA | 0.21<br>(0.13 to 0.29) | < 0.001 |
| Middle-aged and elderly age | Prevalence | Male | Ethiopia | RA | 0.17<br>(0.09 to 0.25) | < 0.001 |
| Middle-aged and elderly age | Prevalence | Both | Fiji | AA | -0.01<br>(-0.03 to 0) | 0.087 |
| Middle-aged and elderly age | Prevalence | Female | Fiji | AA | 0<br>(0 to 0) | < 0.001 |
| Middle-aged and elderly age | Prevalence | Male | Fiji | AA | 0<br>(0 to 0) | < 0.001 |
| Middle-aged and elderly age | Prevalence | Both | Fiji | T1D | 1.02<br>(1.01 to 1.04) | < 0.001 |
| Middle-aged and elderly age | Prevalence | Female | Fiji | T1D | 0.98<br>(0.97 to 1) | < 0.001 |
| Middle-aged and elderly age | Prevalence | Male | Fiji | T1D | 1.04 | < 0.001 |

| | | | | | | |
|---|---|---|---|---|---|---|
| | | | | | (1 to 1.09) | |
| Middle-aged and elderly age | Prevalence | Both | Fiji | IBD | -0.25 (-0.35 to -0.15) | < 0.001 |
| Middle-aged and elderly age | Prevalence | Female | Fiji | IBD | -0.38 (-0.49 to -0.28) | < 0.001 |
| Middle-aged and elderly age | Prevalence | Male | Fiji | IBD | -0.15 (-0.26 to -0.03) | 0.011 |
| Middle-aged and elderly age | Prevalence | Both | Fiji | MS | 0.12 (0.09 to 0.14) | < 0.001 |
| Middle-aged and elderly age | Prevalence | Female | Fiji | MS | 0.15 (0.12 to 0.18) | < 0.001 |
| Middle-aged and elderly age | Prevalence | Male | Fiji | MS | 0.09 (0.05 to 0.14) | < 0.001 |
| Middle-aged and elderly age | Prevalence | Both | Fiji | Psoriasis | -0.52 (-0.53 to -0.5) | < 0.001 |
| Middle-aged and elderly age | Prevalence | Female | Fiji | Psoriasis | -0.51 (-0.54 to -0.49) | < 0.001 |
| Middle-aged and elderly age | Prevalence | Male | Fiji | Psoriasis | -0.54 (-0.58 to -0.5) | < 0.001 |
| Middle-aged and elderly age | Prevalence | Both | Fiji | RHD | 0.54 (0.43 to 0.65) | < 0.001 |
| Middle-aged and elderly age | Prevalence | Female | Fiji | RHD | 0.58 (0.45 to 0.71) | < 0.001 |
| Middle-aged and elderly age | Prevalence | Male | Fiji | RHD | 0.51 (0.41 to 0.62) | < 0.001 |
| Middle-aged and elderly age | Prevalence | Both | Fiji | RA | 0.33 (0.25 to 0.41) | < 0.001 |
| Middle-aged and elderly age | Prevalence | Female | Fiji | RA | 0.37 (0.28 to 0.46) | < 0.001 |
| Middle-aged and elderly age | Prevalence | Male | Fiji | RA | 0.31 (0.26 to 0.35) | < 0.001 |
| Middle-aged and elderly age | Prevalence | Both | Finland | AA | -0.03 (-0.03 to -0.03) | < 0.001 |

| | | | | | | |
|---|---|---|---|---|---|---|
| Middle-aged and elderly age | Prevalence | Female | Finland | AA | 0<br>(0 to 0) | < 0.001 |
| Middle-aged and elderly age | Prevalence | Male | Finland | AA | 0<br>(0 to 0) | < 0.001 |
| Middle-aged and elderly age | Prevalence | Both | Finland | T1D | -0.08<br>(-0.4 to 0.24) | 0.624 |
| Middle-aged and elderly age | Prevalence | Female | Finland | T1D | 0.03<br>(-0.31 to 0.37) | 0.854 |
| Middle-aged and elderly age | Prevalence | Male | Finland | T1D | 0.18<br>(-0.14 to 0.5) | 0.284 |
| Middle-aged and elderly age | Prevalence | Both | Finland | IBD | 2.51<br>(2.37 to 2.65) | < 0.001 |
| Middle-aged and elderly age | Prevalence | Female | Finland | IBD | 2.26<br>(1.98 to 2.53) | < 0.001 |
| Middle-aged and elderly age | Prevalence | Male | Finland | IBD | 2.72<br>(2.61 to 2.82) | < 0.001 |
| Middle-aged and elderly age | Prevalence | Both | Finland | MS | 0.59<br>(0.46 to 0.72) | < 0.001 |
| Middle-aged and elderly age | Prevalence | Female | Finland | MS | 0.86<br>(0.67 to 1.05) | < 0.001 |
| Middle-aged and elderly age | Prevalence | Male | Finland | MS | 0.17<br>(0.08 to 0.26) | < 0.001 |
| Middle-aged and elderly age | Prevalence | Both | Finland | Psoriasis | -0.37<br>(-0.38 to -0.36) | < 0.001 |
| Middle-aged and elderly age | Prevalence | Female | Finland | Psoriasis | -0.38<br>(-0.39 to -0.37) | < 0.001 |
| Middle-aged and elderly age | Prevalence | Male | Finland | Psoriasis | -0.39<br>(-0.4 to -0.37) | < 0.001 |
| Middle-aged and elderly age | Prevalence | Both | Finland | RHD | -1.59<br>(-2.24 to -0.94) | < 0.001 |
| Middle-aged and elderly age | Prevalence | Female | Finland | RHD | -1.41<br>(-1.97 to -0.86) | < 0.001 |
| Middle-aged and elderly age | Prevalence | Male | Finland | RHD | -2.01 | < 0.001 |

| | | | | | | |
|---|---|---|---|---|---|---|
| | | | | | (-2.61 to -1.4) | |
| Middle-aged and elderly age | Prevalence | Both | Finland | RA | 0.3<br>(0.17 to 0.44) | < 0.001 |
| Middle-aged and elderly age | Prevalence | Female | Finland | RA | 0.34<br>(0.24 to 0.44) | < 0.001 |
| Middle-aged and elderly age | Prevalence | Male | Finland | RA | 0.34<br>(0.04 to 0.63) | 0.024 |
| Middle-aged and elderly age | Prevalence | Both | France | AA | 0.01<br>(0 to 0.02) | 0.044 |
| Middle-aged and elderly age | Prevalence | Female | France | AA | 0<br>(0 to 0) | < 0.001 |
| Middle-aged and elderly age | Prevalence | Male | France | AA | 0<br>(0 to 0) | < 0.001 |
| Middle-aged and elderly age | Prevalence | Both | France | T1D | 3.1<br>(3.02 to 3.17) | < 0.001 |
| Middle-aged and elderly age | Prevalence | Female | France | T1D | 3.08<br>(2.99 to 3.18) | < 0.001 |
| Middle-aged and elderly age | Prevalence | Male | France | T1D | 3.13<br>(3.05 to 3.21) | < 0.001 |
| Middle-aged and elderly age | Prevalence | Both | France | IBD | 1.36<br>(0.84 to 1.89) | < 0.001 |
| Middle-aged and elderly age | Prevalence | Female | France | IBD | 1.56<br>(1.06 to 2.06) | < 0.001 |
| Middle-aged and elderly age | Prevalence | Male | France | IBD | 1.15<br>(0.61 to 1.7) | < 0.001 |
| Middle-aged and elderly age | Prevalence | Both | France | MS | 1.03<br>(0.99 to 1.08) | < 0.001 |
| Middle-aged and elderly age | Prevalence | Female | France | MS | 0.99<br>(0.94 to 1.03) | < 0.001 |
| Middle-aged and elderly age | Prevalence | Male | France | MS | 1.12<br>(1.08 to 1.16) | < 0.001 |
| Middle-aged and elderly age | Prevalence | Both | France | Psoriasis | -0.41<br>(-0.43 to -0.39) | < 0.001 |

| | | | | | | |
|---|---|---|---|---|---|---|
| Middle-aged and elderly age | Prevalence | Female | France | Psoriasis | -0.43<br>(-0.46 to -0.4) | < 0.001 |
| Middle-aged and elderly age | Prevalence | Male | France | Psoriasis | -0.39<br>(-0.45 to -0.34) | < 0.001 |
| Middle-aged and elderly age | Prevalence | Both | France | RHD | -0.83<br>(-0.92 to -0.74) | < 0.001 |
| Middle-aged and elderly age | Prevalence | Female | France | RHD | -0.97<br>(-1.13 to -0.8) | < 0.001 |
| Middle-aged and elderly age | Prevalence | Male | France | RHD | -0.64<br>(-0.71 to -0.57) | < 0.001 |
| Middle-aged and elderly age | Prevalence | Both | France | RA | 0.51<br>(0.46 to 0.57) | < 0.001 |
| Middle-aged and elderly age | Prevalence | Female | France | RA | 0.5<br>(0.45 to 0.56) | < 0.001 |
| Middle-aged and elderly age | Prevalence | Male | France | RA | 0.55<br>(0.47 to 0.63) | < 0.001 |
| Middle-aged and elderly age | Prevalence | Both | Gabon | AA | -0.09<br>(-0.1 to -0.07) | < 0.001 |
| Middle-aged and elderly age | Prevalence | Female | Gabon | AA | 0<br>(0 to 0) | 0.027 |
| Middle-aged and elderly age | Prevalence | Male | Gabon | AA | 0<br>(0 to 0) | < 0.001 |
| Middle-aged and elderly age | Prevalence | Both | Gabon | T1D | 0.81<br>(0.81 to 0.82) | < 0.001 |
| Middle-aged and elderly age | Prevalence | Female | Gabon | T1D | 0.81<br>(0.8 to 0.82) | < 0.001 |
| Middle-aged and elderly age | Prevalence | Male | Gabon | T1D | 0.8<br>(0.79 to 0.82) | < 0.001 |
| Middle-aged and elderly age | Prevalence | Both | Gabon | IBD | 0.5<br>(0.43 to 0.56) | < 0.001 |
| Middle-aged and elderly age | Prevalence | Female | Gabon | IBD | 0.43<br>(0.4 to 0.45) | < 0.001 |
| Middle-aged and elderly age | Prevalence | Male | Gabon | IBD | 0.53 | < 0.001 |

| | | | | | | |
|---|---|---|---|---|---|---|
| | | | | | (0.46 to 0.61) | |
| Middle-aged and elderly age | Prevalence | Both | Gabon | MS | 0.21 (0.19 to 0.23) | < 0.001 |
| Middle-aged and elderly age | Prevalence | Female | Gabon | MS | 0.24 (0.22 to 0.26) | < 0.001 |
| Middle-aged and elderly age | Prevalence | Male | Gabon | MS | 0.38 (0.34 to 0.42) | < 0.001 |
| Middle-aged and elderly age | Prevalence | Both | Gabon | Psoriasis | -1.18 (-1.2 to -1.16) | < 0.001 |
| Middle-aged and elderly age | Prevalence | Female | Gabon | Psoriasis | -1.17 (-1.2 to -1.14) | < 0.001 |
| Middle-aged and elderly age | Prevalence | Male | Gabon | Psoriasis | -1.17 (-1.2 to -1.14) | < 0.001 |
| Middle-aged and elderly age | Prevalence | Both | Gabon | RHD | 0 (-0.02 to 0.02) | 0.991 |
| Middle-aged and elderly age | Prevalence | Female | Gabon | RHD | 0.05 (0.05 to 0.06) | < 0.001 |
| Middle-aged and elderly age | Prevalence | Male | Gabon | RHD | -0.04 (-0.07 to 0) | 0.027 |
| Middle-aged and elderly age | Prevalence | Both | Gabon | RA | 0.77 (0.65 to 0.89) | < 0.001 |
| Middle-aged and elderly age | Prevalence | Female | Gabon | RA | 0.79 (0.66 to 0.92) | < 0.001 |
| Middle-aged and elderly age | Prevalence | Male | Gabon | RA | 0.83 (0.75 to 0.91) | < 0.001 |
| Middle-aged and elderly age | Prevalence | Both | Gambia | AA | 0.12 (0.1 to 0.13) | < 0.001 |
| Middle-aged and elderly age | Prevalence | Female | Gambia | AA | 0 (0 to 0) | 0.027 |
| Middle-aged and elderly age | Prevalence | Male | Gambia | AA | 0 (0 to 0) | < 0.001 |
| Middle-aged and elderly age | Prevalence | Both | Gambia | T1D | 0.54 (0.54 to 0.55) | < 0.001 |

| | | | | | | |
|---|---|---|---|---|---|---|
| Middle-aged and elderly age | Prevalence | Female | Gambia | T1D | 0.56 (0.54 to 0.57) | < 0.001 |
| Middle-aged and elderly age | Prevalence | Male | Gambia | T1D | 0.54 (0.53 to 0.56) | < 0.001 |
| Middle-aged and elderly age | Prevalence | Both | Gambia | IBD | -0.11 (-0.17 to -0.05) | < 0.001 |
| Middle-aged and elderly age | Prevalence | Female | Gambia | IBD | -0.3 (-0.37 to -0.23) | < 0.001 |
| Middle-aged and elderly age | Prevalence | Male | Gambia | IBD | -0.04 (-0.11 to 0.04) | 0.341 |
| Middle-aged and elderly age | Prevalence | Both | Gambia | MS | 0.47 (0.45 to 0.48) | < 0.001 |
| Middle-aged and elderly age | Prevalence | Female | Gambia | MS | 0.31 (0.29 to 0.32) | < 0.001 |
| Middle-aged and elderly age | Prevalence | Male | Gambia | MS | 0.35 (0.31 to 0.4) | < 0.001 |
| Middle-aged and elderly age | Prevalence | Both | Gambia | Psoriasis | -0.73 (-0.88 to -0.58) | < 0.001 |
| Middle-aged and elderly age | Prevalence | Female | Gambia | Psoriasis | -0.82 (-1 to -0.64) | < 0.001 |
| Middle-aged and elderly age | Prevalence | Male | Gambia | Psoriasis | -0.69 (-0.83 to -0.54) | < 0.001 |
| Middle-aged and elderly age | Prevalence | Both | Gambia | RHD | 0.16 (0.15 to 0.17) | < 0.001 |
| Middle-aged and elderly age | Prevalence | Female | Gambia | RHD | 0.17 (0.16 to 0.19) | < 0.001 |
| Middle-aged and elderly age | Prevalence | Male | Gambia | RHD | 0.1 (0.08 to 0.13) | < 0.001 |
| Middle-aged and elderly age | Prevalence | Both | Gambia | RA | 0.6 (0.57 to 0.63) | < 0.001 |
| Middle-aged and elderly age | Prevalence | Female | Gambia | RA | 0.53 (0.46 to 0.59) | < 0.001 |
| Middle-aged and elderly age | Prevalence | Male | Gambia | RA | 0.58 | < 0.001 |

| | | | | | | |
|---|---|---|---|---|---|---|
| | | | | | (0.55 to 0.6) | |
| Middle-aged and elderly age | Prevalence | Both | Georgia | AA | 0<br>(0 to 0.01) | 0.166 |
| Middle-aged and elderly age | Prevalence | Female | Georgia | AA | 0<br>(0 to 0) | < 0.001 |
| Middle-aged and elderly age | Prevalence | Male | Georgia | AA | 0<br>(0 to 0) | < 0.001 |
| Middle-aged and elderly age | Prevalence | Both | Georgia | T1D | 1.27<br>(1.25 to 1.29) | < 0.001 |
| Middle-aged and elderly age | Prevalence | Female | Georgia | T1D | 1.24<br>(1.21 to 1.27) | < 0.001 |
| Middle-aged and elderly age | Prevalence | Male | Georgia | T1D | 1.3<br>(1.27 to 1.32) | < 0.001 |
| Middle-aged and elderly age | Prevalence | Both | Georgia | IBD | 0.04<br>(-0.03 to 0.11) | 0.221 |
| Middle-aged and elderly age | Prevalence | Female | Georgia | IBD | -0.03<br>(-0.06 to -0.01) | 0.014 |
| Middle-aged and elderly age | Prevalence | Male | Georgia | IBD | 0.08<br>(-0.07 to 0.23) | 0.282 |
| Middle-aged and elderly age | Prevalence | Both | Georgia | MS | 0.93<br>(0.75 to 1.1) | < 0.001 |
| Middle-aged and elderly age | Prevalence | Female | Georgia | MS | 1.13<br>(0.99 to 1.28) | < 0.001 |
| Middle-aged and elderly age | Prevalence | Male | Georgia | MS | 0.66<br>(0.46 to 0.87) | < 0.001 |
| Middle-aged and elderly age | Prevalence | Both | Georgia | Psoriasis | -0.49<br>(-0.54 to -0.44) | < 0.001 |
| Middle-aged and elderly age | Prevalence | Female | Georgia | Psoriasis | -0.51<br>(-0.55 to -0.46) | < 0.001 |
| Middle-aged and elderly age | Prevalence | Male | Georgia | Psoriasis | -0.48<br>(-0.54 to -0.42) | < 0.001 |
| Middle-aged and elderly age | Prevalence | Both | Georgia | RHD | 0.07<br>(0.05 to 0.09) | < 0.001 |

| | | | | | | |
|---|---|---|---|---|---|---|
| Middle-aged and elderly age | Prevalence | Female | Georgia | RHD | 0.08 (0.04 to 0.12) | < 0.001 |
| Middle-aged and elderly age | Prevalence | Male | Georgia | RHD | 0.04 (0.01 to 0.06) | 0.002 |
| Middle-aged and elderly age | Prevalence | Both | Georgia | RA | 0.39 (0.29 to 0.5) | < 0.001 |
| Middle-aged and elderly age | Prevalence | Female | Georgia | RA | 0.36 (0.24 to 0.48) | < 0.001 |
| Middle-aged and elderly age | Prevalence | Male | Georgia | RA | 0.46 (0.38 to 0.54) | < 0.001 |
| Middle-aged and elderly age | Prevalence | Both | Germany | AA | 0 (-0.01 to 0) | 0.356 |
| Middle-aged and elderly age | Prevalence | Female | Germany | AA | 0 (0 to 0) | < 0.001 |
| Middle-aged and elderly age | Prevalence | Male | Germany | AA | 0 (0 to 0) | < 0.001 |
| Middle-aged and elderly age | Prevalence | Both | Germany | T1D | 2.58 (2.47 to 2.69) | < 0.001 |
| Middle-aged and elderly age | Prevalence | Female | Germany | T1D | 2.46 (2.33 to 2.59) | < 0.001 |
| Middle-aged and elderly age | Prevalence | Male | Germany | T1D | 2.71 (2.63 to 2.78) | < 0.001 |
| Middle-aged and elderly age | Prevalence | Both | Germany | IBD | 2.78 (2.54 to 3.01) | < 0.001 |
| Middle-aged and elderly age | Prevalence | Female | Germany | IBD | 2.91 (2.63 to 3.19) | < 0.001 |
| Middle-aged and elderly age | Prevalence | Male | Germany | IBD | 2.63 (2.44 to 2.81) | < 0.001 |
| Middle-aged and elderly age | Prevalence | Both | Germany | MS | 0.63 (0.61 to 0.65) | < 0.001 |
| Middle-aged and elderly age | Prevalence | Female | Germany | MS | 0.73 (0.71 to 0.75) | < 0.001 |
| Middle-aged and elderly age | Prevalence | Male | Germany | MS | 0.44 | < 0.001 |

| | | | | | | |
|---|---|---|---|---|---|---|
| | | | | | (0.42 to 0.46) | |
| Middle-aged and elderly age | Prevalence | Both | Germany | Psoriasis | -0.29<br>(-0.5 to -0.07) | 0.009 |
| Middle-aged and elderly age | Prevalence | Female | Germany | Psoriasis | -0.29<br>(-0.46 to -0.11) | 0.002 |
| Middle-aged and elderly age | Prevalence | Male | Germany | Psoriasis | -0.29<br>(-0.54 to -0.04) | 0.023 |
| Middle-aged and elderly age | Prevalence | Both | Germany | RHD | -1.82<br>(-2.14 to -1.5) | < 0.001 |
| Middle-aged and elderly age | Prevalence | Female | Germany | RHD | -1.99<br>(-2.31 to -1.67) | < 0.001 |
| Middle-aged and elderly age | Prevalence | Male | Germany | RHD | -1.59<br>(-1.88 to -1.31) | < 0.001 |
| Middle-aged and elderly age | Prevalence | Both | Germany | RA | 0.4<br>(0.36 to 0.43) | < 0.001 |
| Middle-aged and elderly age | Prevalence | Female | Germany | RA | 0.38<br>(0.32 to 0.43) | < 0.001 |
| Middle-aged and elderly age | Prevalence | Male | Germany | RA | 0.43<br>(0.38 to 0.48) | < 0.001 |
| Middle-aged and elderly age | Prevalence | Both | Ghana | AA | 0.09<br>(0.08 to 0.1) | < 0.001 |
| Middle-aged and elderly age | Prevalence | Female | Ghana | AA | 0<br>(0 to 0) | 0.027 |
| Middle-aged and elderly age | Prevalence | Male | Ghana | AA | 0<br>(0 to 0) | < 0.001 |
| Middle-aged and elderly age | Prevalence | Both | Ghana | T1D | 0.49<br>(0.48 to 0.51) | < 0.001 |
| Middle-aged and elderly age | Prevalence | Female | Ghana | T1D | 0.5<br>(0.49 to 0.52) | < 0.001 |
| Middle-aged and elderly age | Prevalence | Male | Ghana | T1D | 0.49<br>(0.48 to 0.51) | < 0.001 |
| Middle-aged and elderly age | Prevalence | Both | Ghana | IBD | 0.45<br>(0.36 to 0.54) | < 0.001 |

| | | | | | | |
|---|---|---|---|---|---|---|
| Middle-aged and elderly age | Prevalence | Female | Ghana | IBD | 0.28<br>(0.18 to 0.39) | < 0.001 |
| Middle-aged and elderly age | Prevalence | Male | Ghana | IBD | 0.64<br>(0.49 to 0.79) | < 0.001 |
| Middle-aged and elderly age | Prevalence | Both | Ghana | MS | 1.88<br>(1.8 to 1.96) | < 0.001 |
| Middle-aged and elderly age | Prevalence | Female | Ghana | MS | 1.78<br>(1.69 to 1.87) | < 0.001 |
| Middle-aged and elderly age | Prevalence | Male | Ghana | MS | 0.59<br>(0.49 to 0.69) | < 0.001 |
| Middle-aged and elderly age | Prevalence | Both | Ghana | Psoriasis | -0.7<br>(-0.9 to -0.5) | < 0.001 |
| Middle-aged and elderly age | Prevalence | Female | Ghana | Psoriasis | -0.81<br>(-1.07 to -0.56) | < 0.001 |
| Middle-aged and elderly age | Prevalence | Male | Ghana | Psoriasis | -0.58<br>(-0.66 to -0.51) | < 0.001 |
| Middle-aged and elderly age | Prevalence | Both | Ghana | RHD | 0.05<br>(0.03 to 0.08) | < 0.001 |
| Middle-aged and elderly age | Prevalence | Female | Ghana | RHD | 0.07<br>(0.05 to 0.09) | < 0.001 |
| Middle-aged and elderly age | Prevalence | Male | Ghana | RHD | -0.01<br>(-0.05 to 0.03) | 0.590 |
| Middle-aged and elderly age | Prevalence | Both | Ghana | RA | 0.83<br>(0.78 to 0.87) | < 0.001 |
| Middle-aged and elderly age | Prevalence | Female | Ghana | RA | 0.73<br>(0.68 to 0.78) | < 0.001 |
| Middle-aged and elderly age | Prevalence | Male | Ghana | RA | 0.88<br>(0.81 to 0.94) | < 0.001 |
| Middle-aged and elderly age | Prevalence | Both | Greece | AA | 0.02<br>(0.01 to 0.02) | < 0.001 |
| Middle-aged and elderly age | Prevalence | Female | Greece | AA | 0<br>(0 to 0) | < 0.001 |
| Middle-aged and elderly age | Prevalence | Male | Greece | AA | 0 | < 0.001 |

| | | | | | | |
|---|---|---|---|---|---|---|
| | | | | | (0 to 0) | |
| Middle-aged and elderly age | Prevalence | Both | Greece | T1D | 3.07 (2.9 to 3.24) | < 0.001 |
| Middle-aged and elderly age | Prevalence | Female | Greece | T1D | 3.09 (2.88 to 3.3) | < 0.001 |
| Middle-aged and elderly age | Prevalence | Male | Greece | T1D | 3.07 (2.92 to 3.21) | < 0.001 |
| Middle-aged and elderly age | Prevalence | Both | Greece | IBD | -1.19 (-1.28 to -1.1) | < 0.001 |
| Middle-aged and elderly age | Prevalence | Female | Greece | IBD | -0.99 (-1.06 to -0.91) | < 0.001 |
| Middle-aged and elderly age | Prevalence | Male | Greece | IBD | -1.39 (-1.5 to -1.27) | < 0.001 |
| Middle-aged and elderly age | Prevalence | Both | Greece | MS | 1.06 (1.04 to 1.09) | < 0.001 |
| Middle-aged and elderly age | Prevalence | Female | Greece | MS | 1.12 (1.08 to 1.15) | < 0.001 |
| Middle-aged and elderly age | Prevalence | Male | Greece | MS | 0.93 (0.89 to 0.97) | < 0.001 |
| Middle-aged and elderly age | Prevalence | Both | Greece | Psoriasis | -0.44 (-0.45 to -0.43) | < 0.001 |
| Middle-aged and elderly age | Prevalence | Female | Greece | Psoriasis | -0.44 (-0.45 to -0.43) | < 0.001 |
| Middle-aged and elderly age | Prevalence | Male | Greece | Psoriasis | -0.44 (-0.46 to -0.43) | < 0.001 |
| Middle-aged and elderly age | Prevalence | Both | Greece | RHD | -0.66 (-0.81 to -0.52) | < 0.001 |
| Middle-aged and elderly age | Prevalence | Female | Greece | RHD | -0.81 (-0.97 to -0.65) | < 0.001 |
| Middle-aged and elderly age | Prevalence | Male | Greece | RHD | -0.48 (-0.58 to -0.38) | < 0.001 |
| Middle-aged and elderly age | Prevalence | Both | Greece | RA | 0.42 (0.37 to 0.46) | < 0.001 |

| | | | | | | |
|---|---|---|---|---|---|---|
| Middle-aged and elderly age | Prevalence | Female | Greece | RA | 0.41 (0.37 to 0.45) | < 0.001 |
| Middle-aged and elderly age | Prevalence | Male | Greece | RA | 0.38 (0.31 to 0.46) | < 0.001 |
| Middle-aged and elderly age | Prevalence | Both | Greenland | AA | 0.09 (0.06 to 0.12) | < 0.001 |
| Middle-aged and elderly age | Prevalence | Female | Greenland | AA | 0 (0 to 0) | < 0.001 |
| Middle-aged and elderly age | Prevalence | Male | Greenland | AA | 0 (0 to 0) | < 0.001 |
| Middle-aged and elderly age | Prevalence | Both | Greenland | T1D | 0.45 (0.37 to 0.53) | < 0.001 |
| Middle-aged and elderly age | Prevalence | Female | Greenland | T1D | 0.38 (0.3 to 0.47) | < 0.001 |
| Middle-aged and elderly age | Prevalence | Male | Greenland | T1D | 0.51 (0.42 to 0.61) | < 0.001 |
| Middle-aged and elderly age | Prevalence | Both | Greenland | IBD | 0.27 (0.25 to 0.29) | < 0.001 |
| Middle-aged and elderly age | Prevalence | Female | Greenland | IBD | 0.21 (0.19 to 0.23) | < 0.001 |
| Middle-aged and elderly age | Prevalence | Male | Greenland | IBD | 0.31 (0.28 to 0.35) | < 0.001 |
| Middle-aged and elderly age | Prevalence | Both | Greenland | MS | 0.39 (0.29 to 0.5) | < 0.001 |
| Middle-aged and elderly age | Prevalence | Female | Greenland | MS | 0.27 (0.15 to 0.4) | < 0.001 |
| Middle-aged and elderly age | Prevalence | Male | Greenland | MS | 0.47 (0.41 to 0.54) | < 0.001 |
| Middle-aged and elderly age | Prevalence | Both | Greenland | Psoriasis | -0.45 (-0.47 to -0.44) | < 0.001 |
| Middle-aged and elderly age | Prevalence | Female | Greenland | Psoriasis | -0.45 (-0.47 to -0.43) | < 0.001 |
| Middle-aged and elderly age | Prevalence | Male | Greenland | Psoriasis | -0.45 | < 0.001 |

| | | | | | | |
|---|---|---|---|---|---|---|
| | | | | | (-0.47 to -0.44) | |
| Middle-aged and elderly age | Prevalence | Both | Greenland | RHD | -1.11<br>(-1.17 to -1.06) | < 0.001 |
| Middle-aged and elderly age | Prevalence | Female | Greenland | RHD | -1.42<br>(-1.47 to -1.38) | < 0.001 |
| Middle-aged and elderly age | Prevalence | Male | Greenland | RHD | -0.84<br>(-0.91 to -0.78) | < 0.001 |
| Middle-aged and elderly age | Prevalence | Both | Greenland | RA | 1.18<br>(1.13 to 1.23) | < 0.001 |
| Middle-aged and elderly age | Prevalence | Female | Greenland | RA | 1.05<br>(1.01 to 1.09) | < 0.001 |
| Middle-aged and elderly age | Prevalence | Male | Greenland | RA | 1.25<br>(1.19 to 1.3) | < 0.001 |
| Middle-aged and elderly age | Prevalence | Both | Grenada | AA | -0.13<br>(-0.14 to -0.12) | < 0.001 |
| Middle-aged and elderly age | Prevalence | Female | Grenada | AA | 0<br>(0 to 0) | < 0.001 |
| Middle-aged and elderly age | Prevalence | Male | Grenada | AA | 0<br>(0 to 0) | < 0.001 |
| Middle-aged and elderly age | Prevalence | Both | Grenada | T1D | 1.3<br>(1.28 to 1.32) | < 0.001 |
| Middle-aged and elderly age | Prevalence | Female | Grenada | T1D | 1.19<br>(1.17 to 1.21) | < 0.001 |
| Middle-aged and elderly age | Prevalence | Male | Grenada | T1D | 1.33<br>(1.32 to 1.35) | < 0.001 |
| Middle-aged and elderly age | Prevalence | Both | Grenada | IBD | 0.16<br>(0.08 to 0.23) | < 0.001 |
| Middle-aged and elderly age | Prevalence | Female | Grenada | IBD | 0.17<br>(0.11 to 0.23) | < 0.001 |
| Middle-aged and elderly age | Prevalence | Male | Grenada | IBD | 0.15<br>(0.07 to 0.23) | < 0.001 |
| Middle-aged and elderly age | Prevalence | Both | Grenada | MS | 0.57<br>(0.52 to 0.61) | < 0.001 |

| | | | | | | |
|---|---|---|---|---|---|---|
| Middle-aged and elderly age | Prevalence | Female | Grenada | MS | 0.63<br>(0.59 to 0.68) | < 0.001 |
| Middle-aged and elderly age | Prevalence | Male | Grenada | MS | 0.85<br>(0.75 to 0.95) | < 0.001 |
| Middle-aged and elderly age | Prevalence | Both | Grenada | Psoriasis | -0.64<br>(-0.66 to -0.61) | < 0.001 |
| Middle-aged and elderly age | Prevalence | Female | Grenada | Psoriasis | -0.62<br>(-0.65 to -0.59) | < 0.001 |
| Middle-aged and elderly age | Prevalence | Male | Grenada | Psoriasis | -0.63<br>(-0.67 to -0.59) | < 0.001 |
| Middle-aged and elderly age | Prevalence | Both | Grenada | RHD | 0.02<br>(0.01 to 0.02) | 0.002 |
| Middle-aged and elderly age | Prevalence | Female | Grenada | RHD | 0.08<br>(0.05 to 0.11) | < 0.001 |
| Middle-aged and elderly age | Prevalence | Male | Grenada | RHD | 0.07<br>(0.05 to 0.08) | < 0.001 |
| Middle-aged and elderly age | Prevalence | Both | Grenada | RA | 0.8<br>(0.75 to 0.86) | < 0.001 |
| Middle-aged and elderly age | Prevalence | Female | Grenada | RA | 0.96<br>(0.91 to 1) | < 0.001 |
| Middle-aged and elderly age | Prevalence | Male | Grenada | RA | 1.01<br>(0.94 to 1.08) | < 0.001 |
| Middle-aged and elderly age | Prevalence | Both | Guam | AA | -0.03<br>(-0.05 to 0) | 0.029 |
| Middle-aged and elderly age | Prevalence | Female | Guam | AA | 0<br>(0 to 0) | < 0.001 |
| Middle-aged and elderly age | Prevalence | Male | Guam | AA | 0<br>(0 to 0) | < 0.001 |
| Middle-aged and elderly age | Prevalence | Both | Guam | T1D | 0.73<br>(0.68 to 0.78) | < 0.001 |
| Middle-aged and elderly age | Prevalence | Female | Guam | T1D | 0.71<br>(0.67 to 0.75) | < 0.001 |
| Middle-aged and elderly age | Prevalence | Male | Guam | T1D | 0.75 | < 0.001 |

| | | | | | | |
|---|---|---|---|---|---|---|
| | | | | | (0.7 to 0.8) | |
| Middle-aged and elderly age | Prevalence | Both | Guam | IBD | -0.23<br>(-0.29 to -0.16) | < 0.001 |
| Middle-aged and elderly age | Prevalence | Female | Guam | IBD | -0.19<br>(-0.3 to -0.07) | 0.002 |
| Middle-aged and elderly age | Prevalence | Male | Guam | IBD | -0.28<br>(-0.32 to -0.25) | < 0.001 |
| Middle-aged and elderly age | Prevalence | Both | Guam | MS | 0.03<br>(0 to 0.05) | 0.038 |
| Middle-aged and elderly age | Prevalence | Female | Guam | MS | 0.03<br>(0 to 0.06) | 0.024 |
| Middle-aged and elderly age | Prevalence | Male | Guam | MS | 0.04<br>(0.01 to 0.07) | 0.005 |
| Middle-aged and elderly age | Prevalence | Both | Guam | Psoriasis | -0.44<br>(-0.5 to -0.38) | < 0.001 |
| Middle-aged and elderly age | Prevalence | Female | Guam | Psoriasis | -0.45<br>(-0.49 to -0.41) | < 0.001 |
| Middle-aged and elderly age | Prevalence | Male | Guam | Psoriasis | -0.46<br>(-0.5 to -0.42) | < 0.001 |
| Middle-aged and elderly age | Prevalence | Both | Guam | RHD | 0.15<br>(0.14 to 0.16) | < 0.001 |
| Middle-aged and elderly age | Prevalence | Female | Guam | RHD | 0.17<br>(0.15 to 0.19) | < 0.001 |
| Middle-aged and elderly age | Prevalence | Male | Guam | RHD | 0.16<br>(0.12 to 0.19) | < 0.001 |
| Middle-aged and elderly age | Prevalence | Both | Guam | RA | 0.2<br>(0.13 to 0.27) | < 0.001 |
| Middle-aged and elderly age | Prevalence | Female | Guam | RA | 0.24<br>(0.15 to 0.33) | < 0.001 |
| Middle-aged and elderly age | Prevalence | Male | Guam | RA | 0.19<br>(0.14 to 0.25) | < 0.001 |
| Middle-aged and elderly age | Prevalence | Both | Guatemala | AA | 0.11<br>(0.11 to 0.11) | < 0.001 |

| | | | | | | |
|---|---|---|---|---|---|---|
| Middle-aged and elderly age | Prevalence | Female | Guatemala | AA | 0<br>(0 to 0) | < 0.001 |
| Middle-aged and elderly age | Prevalence | Male | Guatemala | AA | 0<br>(0 to 0) | < 0.001 |
| Middle-aged and elderly age | Prevalence | Both | Guatemala | T1D | 0.67<br>(0.63 to 0.71) | < 0.001 |
| Middle-aged and elderly age | Prevalence | Female | Guatemala | T1D | 0.7<br>(0.67 to 0.72) | < 0.001 |
| Middle-aged and elderly age | Prevalence | Male | Guatemala | T1D | 0.64<br>(0.59 to 0.68) | < 0.001 |
| Middle-aged and elderly age | Prevalence | Both | Guatemala | IBD | 0.23<br>(0.21 to 0.25) | < 0.001 |
| Middle-aged and elderly age | Prevalence | Female | Guatemala | IBD | 0.33<br>(0.31 to 0.34) | < 0.001 |
| Middle-aged and elderly age | Prevalence | Male | Guatemala | IBD | 0.23<br>(0.22 to 0.25) | < 0.001 |
| Middle-aged and elderly age | Prevalence | Both | Guatemala | MS | 1.2<br>(1.18 to 1.23) | < 0.001 |
| Middle-aged and elderly age | Prevalence | Female | Guatemala | MS | 1.2<br>(1.17 to 1.23) | < 0.001 |
| Middle-aged and elderly age | Prevalence | Male | Guatemala | MS | 0.74<br>(0.73 to 0.76) | < 0.001 |
| Middle-aged and elderly age | Prevalence | Both | Guatemala | Psoriasis | -0.68<br>(-0.71 to -0.66) | < 0.001 |
| Middle-aged and elderly age | Prevalence | Female | Guatemala | Psoriasis | -0.7<br>(-0.72 to -0.68) | < 0.001 |
| Middle-aged and elderly age | Prevalence | Male | Guatemala | Psoriasis | -0.68<br>(-0.72 to -0.64) | < 0.001 |
| Middle-aged and elderly age | Prevalence | Both | Guatemala | RHD | 0.27<br>(0.25 to 0.28) | < 0.001 |
| Middle-aged and elderly age | Prevalence | Female | Guatemala | RHD | 0.21<br>(0.2 to 0.22) | < 0.001 |
| Middle-aged and elderly age | Prevalence | Male | Guatemala | RHD | 0.21 | < 0.001 |

| | | | | | | |
|---|---|---|---|---|---|---|
| | | | | | (0.2 to 0.23) | |
| Middle-aged and elderly age | Prevalence | Both | Guatemala | RA | 1.6<br>(1.54 to 1.67) | < 0.001 |
| Middle-aged and elderly age | Prevalence | Female | Guatemala | RA | 1.51<br>(1.46 to 1.57) | < 0.001 |
| Middle-aged and elderly age | Prevalence | Male | Guatemala | RA | 1.23<br>(1.18 to 1.27) | < 0.001 |
| Middle-aged and elderly age | Prevalence | Both | Guinea | AA | 0.01<br>(0 to 0.02) | 0.240 |
| Middle-aged and elderly age | Prevalence | Female | Guinea | AA | 0<br>(0 to 0) | 0.027 |
| Middle-aged and elderly age | Prevalence | Male | Guinea | AA | 0<br>(0 to 0) | < 0.001 |
| Middle-aged and elderly age | Prevalence | Both | Guinea | T1D | 0.19<br>(0.19 to 0.2) | < 0.001 |
| Middle-aged and elderly age | Prevalence | Female | Guinea | T1D | 0.2<br>(0.18 to 0.22) | < 0.001 |
| Middle-aged and elderly age | Prevalence | Male | Guinea | T1D | 0.19<br>(0.17 to 0.21) | < 0.001 |
| Middle-aged and elderly age | Prevalence | Both | Guinea | IBD | 0.37<br>(0.35 to 0.39) | < 0.001 |
| Middle-aged and elderly age | Prevalence | Female | Guinea | IBD | 0.18<br>(0.16 to 0.21) | < 0.001 |
| Middle-aged and elderly age | Prevalence | Male | Guinea | IBD | 0.55<br>(0.52 to 0.59) | < 0.001 |
| Middle-aged and elderly age | Prevalence | Both | Guinea | MS | 0.29<br>(0.27 to 0.31) | < 0.001 |
| Middle-aged and elderly age | Prevalence | Female | Guinea | MS | 0.23<br>(0.2 to 0.26) | < 0.001 |
| Middle-aged and elderly age | Prevalence | Male | Guinea | MS | 0.38<br>(0.35 to 0.41) | < 0.001 |
| Middle-aged and elderly age | Prevalence | Both | Guinea | Psoriasis | -0.55<br>(-0.7 to -0.39) | < 0.001 |

| | | | | | | |
|---|---|---|---|---|---|---|
| Middle-aged and elderly age | Prevalence | Female | Guinea | Psoriasis | -0.63<br>(-0.77 to -0.49) | < 0.001 |
| Middle-aged and elderly age | Prevalence | Male | Guinea | Psoriasis | -0.5<br>(-0.62 to -0.38) | < 0.001 |
| Middle-aged and elderly age | Prevalence | Both | Guinea | RHD | 0.28<br>(0.26 to 0.3) | < 0.001 |
| Middle-aged and elderly age | Prevalence | Female | Guinea | RHD | 0.31<br>(0.3 to 0.33) | < 0.001 |
| Middle-aged and elderly age | Prevalence | Male | Guinea | RHD | 0.24<br>(0.22 to 0.27) | < 0.001 |
| Middle-aged and elderly age | Prevalence | Both | Guinea | RA | 0.47<br>(0.43 to 0.51) | < 0.001 |
| Middle-aged and elderly age | Prevalence | Female | Guinea | RA | 0.43<br>(0.38 to 0.49) | < 0.001 |
| Middle-aged and elderly age | Prevalence | Male | Guinea | RA | 0.54<br>(0.51 to 0.57) | < 0.001 |
| Middle-aged and elderly age | Prevalence | Both | Guinea-Bissau | AA | -0.01<br>(-0.02 to -0.01) | < 0.001 |
| Middle-aged and elderly age | Prevalence | Female | Guinea-Bissau | AA | 0<br>(0 to 0) | 0.027 |
| Middle-aged and elderly age | Prevalence | Male | Guinea-Bissau | AA | 0<br>(0 to 0) | < 0.001 |
| Middle-aged and elderly age | Prevalence | Both | Guinea-Bissau | T1D | 0.34<br>(0.33 to 0.35) | < 0.001 |
| Middle-aged and elderly age | Prevalence | Female | Guinea-Bissau | T1D | 0.33<br>(0.31 to 0.35) | < 0.001 |
| Middle-aged and elderly age | Prevalence | Male | Guinea-Bissau | T1D | 0.35<br>(0.33 to 0.36) | < 0.001 |
| Middle-aged and elderly age | Prevalence | Both | Guinea-Bissau | IBD | 0.42<br>(0.35 to 0.48) | < 0.001 |
| Middle-aged and elderly age | Prevalence | Female | Guinea-Bissau | IBD | 0.29<br>(0.21 to 0.36) | < 0.001 |
| Middle-aged and elderly age | Prevalence | Male | Guinea-Bissau | IBD | 0.54 | < 0.001 |

| | | | | | | |
|---|---|---|---|---|---|---|
| | | | | | (0.47 to 0.61) | |
| Middle-aged and elderly age | Prevalence | Both | Guinea-Bissau | MS | 0.17<br>(0.12 to 0.21) | < 0.001 |
| Middle-aged and elderly age | Prevalence | Female | Guinea-Bissau | MS | 0.19<br>(0.15 to 0.23) | < 0.001 |
| Middle-aged and elderly age | Prevalence | Male | Guinea-Bissau | MS | 0.21<br>(0.16 to 0.25) | < 0.001 |
| Middle-aged and elderly age | Prevalence | Both | Guinea-Bissau | Psoriasis | -0.64<br>(-0.79 to -0.49) | < 0.001 |
| Middle-aged and elderly age | Prevalence | Female | Guinea-Bissau | Psoriasis | -0.71<br>(-0.87 to -0.56) | < 0.001 |
| Middle-aged and elderly age | Prevalence | Male | Guinea-Bissau | Psoriasis | -0.55<br>(-0.69 to -0.42) | < 0.001 |
| Middle-aged and elderly age | Prevalence | Both | Guinea-Bissau | RHD | 0.17<br>(0.16 to 0.18) | < 0.001 |
| Middle-aged and elderly age | Prevalence | Female | Guinea-Bissau | RHD | 0.2<br>(0.18 to 0.21) | < 0.001 |
| Middle-aged and elderly age | Prevalence | Male | Guinea-Bissau | RHD | 0.15<br>(0.12 to 0.18) | < 0.001 |
| Middle-aged and elderly age | Prevalence | Both | Guinea-Bissau | RA | 0.49<br>(0.45 to 0.53) | < 0.001 |
| Middle-aged and elderly age | Prevalence | Female | Guinea-Bissau | RA | 0.49<br>(0.45 to 0.53) | < 0.001 |
| Middle-aged and elderly age | Prevalence | Male | Guinea-Bissau | RA | 0.55<br>(0.49 to 0.61) | < 0.001 |
| Middle-aged and elderly age | Prevalence | Both | Guyana | AA | 0<br>(0 to 0.01) | 0.122 |
| Middle-aged and elderly age | Prevalence | Female | Guyana | AA | 0<br>(0 to 0) | < 0.001 |
| Middle-aged and elderly age | Prevalence | Male | Guyana | AA | 0<br>(0 to 0) | < 0.001 |
| Middle-aged and elderly age | Prevalence | Both | Guyana | T1D | 1.1<br>(1.09 to 1.11) | < 0.001 |

| | | | | | | |
|---|---|---|---|---|---|---|
| Middle-aged and elderly age | Prevalence | Female | Guyana | T1D | 1.04<br>(1.01 to 1.07) | < 0.001 |
| Middle-aged and elderly age | Prevalence | Male | Guyana | T1D | 1.17<br>(1.16 to 1.18) | < 0.001 |
| Middle-aged and elderly age | Prevalence | Both | Guyana | IBD | -0.01<br>(-0.06 to 0.03) | 0.498 |
| Middle-aged and elderly age | Prevalence | Female | Guyana | IBD | 0.05<br>(-0.02 to 0.11) | 0.165 |
| Middle-aged and elderly age | Prevalence | Male | Guyana | IBD | -0.05<br>(-0.07 to -0.03) | < 0.001 |
| Middle-aged and elderly age | Prevalence | Both | Guyana | MS | 0.58<br>(0.54 to 0.61) | < 0.001 |
| Middle-aged and elderly age | Prevalence | Female | Guyana | MS | 0.64<br>(0.59 to 0.69) | < 0.001 |
| Middle-aged and elderly age | Prevalence | Male | Guyana | MS | 0.44<br>(0.38 to 0.51) | < 0.001 |
| Middle-aged and elderly age | Prevalence | Both | Guyana | Psoriasis | -0.43<br>(-0.44 to -0.41) | < 0.001 |
| Middle-aged and elderly age | Prevalence | Female | Guyana | Psoriasis | -0.42<br>(-0.44 to -0.41) | < 0.001 |
| Middle-aged and elderly age | Prevalence | Male | Guyana | Psoriasis | -0.43<br>(-0.45 to -0.41) | < 0.001 |
| Middle-aged and elderly age | Prevalence | Both | Guyana | RHD | 0.05<br>(0.03 to 0.06) | < 0.001 |
| Middle-aged and elderly age | Prevalence | Female | Guyana | RHD | 0.09<br>(0.05 to 0.12) | < 0.001 |
| Middle-aged and elderly age | Prevalence | Male | Guyana | RHD | 0.01<br>(0 to 0.01) | 0.103 |
| Middle-aged and elderly age | Prevalence | Both | Guyana | RA | 0.87<br>(0.82 to 0.92) | < 0.001 |
| Middle-aged and elderly age | Prevalence | Female | Guyana | RA | 0.86<br>(0.81 to 0.9) | < 0.001 |
| Middle-aged and elderly age | Prevalence | Male | Guyana | RA | 0.87 | < 0.001 |

| | | | | | | |
|---|---|---|---|---|---|---|
| | | | | | (0.81 to 0.94) | |
| Middle-aged and elderly age | Prevalence | Both | Haiti | AA | 0.06<br>(0.05 to 0.06) | < 0.001 |
| Middle-aged and elderly age | Prevalence | Female | Haiti | AA | 0<br>(0 to 0) | < 0.001 |
| Middle-aged and elderly age | Prevalence | Male | Haiti | AA | 0<br>(0 to 0) | < 0.001 |
| Middle-aged and elderly age | Prevalence | Both | Haiti | T1D | 1.2<br>(1.19 to 1.21) | < 0.001 |
| Middle-aged and elderly age | Prevalence | Female | Haiti | T1D | 1.15<br>(1.12 to 1.17) | < 0.001 |
| Middle-aged and elderly age | Prevalence | Male | Haiti | T1D | 1.27<br>(1.26 to 1.28) | < 0.001 |
| Middle-aged and elderly age | Prevalence | Both | Haiti | IBD | -0.09<br>(-0.15 to -0.03) | 0.005 |
| Middle-aged and elderly age | Prevalence | Female | Haiti | IBD | -0.11<br>(-0.2 to -0.02) | 0.015 |
| Middle-aged and elderly age | Prevalence | Male | Haiti | IBD | -0.09<br>(-0.14 to -0.04) | 0.001 |
| Middle-aged and elderly age | Prevalence | Both | Haiti | MS | 0.76<br>(0.7 to 0.82) | < 0.001 |
| Middle-aged and elderly age | Prevalence | Female | Haiti | MS | 0.78<br>(0.7 to 0.85) | < 0.001 |
| Middle-aged and elderly age | Prevalence | Male | Haiti | MS | 0.52<br>(0.49 to 0.55) | < 0.001 |
| Middle-aged and elderly age | Prevalence | Both | Haiti | Psoriasis | -0.36<br>(-0.39 to -0.33) | < 0.001 |
| Middle-aged and elderly age | Prevalence | Female | Haiti | Psoriasis | -0.36<br>(-0.4 to -0.33) | < 0.001 |
| Middle-aged and elderly age | Prevalence | Male | Haiti | Psoriasis | -0.38<br>(-0.4 to -0.35) | < 0.001 |
| Middle-aged and elderly age | Prevalence | Both | Haiti | RHD | 0.11<br>(0.09 to 0.13) | < 0.001 |

| | | | | | | |
|---|---|---|---|---|---|---|
| Middle-aged and elderly age | Prevalence | Female | Haiti | RHD | 0.14 (0.11 to 0.16) | < 0.001 |
| Middle-aged and elderly age | Prevalence | Male | Haiti | RHD | 0.04 (0.01 to 0.07) | 0.003 |
| Middle-aged and elderly age | Prevalence | Both | Haiti | RA | 0.88 (0.83 to 0.93) | < 0.001 |
| Middle-aged and elderly age | Prevalence | Female | Haiti | RA | 0.77 (0.68 to 0.85) | < 0.001 |
| Middle-aged and elderly age | Prevalence | Male | Haiti | RA | 0.79 (0.76 to 0.82) | < 0.001 |
| Middle-aged and elderly age | Prevalence | Both | Honduras | AA | 0.04 (0.03 to 0.04) | < 0.001 |
| Middle-aged and elderly age | Prevalence | Female | Honduras | AA | 0 (0 to 0) | < 0.001 |
| Middle-aged and elderly age | Prevalence | Male | Honduras | AA | 0 (0 to 0) | < 0.001 |
| Middle-aged and elderly age | Prevalence | Both | Honduras | T1D | 0.6 (0.59 to 0.61) | < 0.001 |
| Middle-aged and elderly age | Prevalence | Female | Honduras | T1D | 0.63 (0.63 to 0.64) | < 0.001 |
| Middle-aged and elderly age | Prevalence | Male | Honduras | T1D | 0.57 (0.55 to 0.58) | < 0.001 |
| Middle-aged and elderly age | Prevalence | Both | Honduras | IBD | 0.11 (0.09 to 0.13) | < 0.001 |
| Middle-aged and elderly age | Prevalence | Female | Honduras | IBD | 0.16 (0.14 to 0.17) | < 0.001 |
| Middle-aged and elderly age | Prevalence | Male | Honduras | IBD | 0.11 (0.08 to 0.14) | < 0.001 |
| Middle-aged and elderly age | Prevalence | Both | Honduras | MS | 0.74 (0.72 to 0.76) | < 0.001 |
| Middle-aged and elderly age | Prevalence | Female | Honduras | MS | 0.79 (0.76 to 0.81) | < 0.001 |
| Middle-aged and elderly age | Prevalence | Male | Honduras | MS | 0.52 | < 0.001 |

| | | | | | | |
|---|---|---|---|---|---|---|
| | | | | | (0.51 to 0.54) | |
| Middle-aged and elderly age | Prevalence | Both | Honduras | Psoriasis | -0.5 (-0.52 to -0.48) | < 0.001 |
| Middle-aged and elderly age | Prevalence | Female | Honduras | Psoriasis | -0.51 (-0.53 to -0.49) | < 0.001 |
| Middle-aged and elderly age | Prevalence | Male | Honduras | Psoriasis | -0.49 (-0.51 to -0.47) | < 0.001 |
| Middle-aged and elderly age | Prevalence | Both | Honduras | RHD | 0.07 (0.05 to 0.09) | < 0.001 |
| Middle-aged and elderly age | Prevalence | Female | Honduras | RHD | 0.06 (0.03 to 0.09) | < 0.001 |
| Middle-aged and elderly age | Prevalence | Male | Honduras | RHD | 0.05 (0.02 to 0.08) | < 0.001 |
| Middle-aged and elderly age | Prevalence | Both | Honduras | RA | 1.11 (1.05 to 1.17) | < 0.001 |
| Middle-aged and elderly age | Prevalence | Female | Honduras | RA | 1.09 (1.03 to 1.14) | < 0.001 |
| Middle-aged and elderly age | Prevalence | Male | Honduras | RA | 0.84 (0.76 to 0.93) | < 0.001 |
| Middle-aged and elderly age | Prevalence | Both | Hungary | AA | -0.05 (-0.05 to -0.05) | < 0.001 |
| Middle-aged and elderly age | Prevalence | Female | Hungary | AA | 0 (0 to 0) | < 0.001 |
| Middle-aged and elderly age | Prevalence | Male | Hungary | AA | 0 (0 to 0) | < 0.001 |
| Middle-aged and elderly age | Prevalence | Both | Hungary | T1D | 1.97 (1.91 to 2.04) | < 0.001 |
| Middle-aged and elderly age | Prevalence | Female | Hungary | T1D | 1.94 (1.88 to 1.99) | < 0.001 |
| Middle-aged and elderly age | Prevalence | Male | Hungary | T1D | 1.99 (1.92 to 2.06) | < 0.001 |
| Middle-aged and elderly age | Prevalence | Both | Hungary | IBD | 1.71 (1.57 to 1.86) | < 0.001 |

| | | | | | | |
|---|---|---|---|---|---|---|
| Middle-aged and elderly age | Prevalence | Female | Hungary | IBD | 1.78<br>(1.62 to 1.95) | < 0.001 |
| Middle-aged and elderly age | Prevalence | Male | Hungary | IBD | 1.61<br>(1.47 to 1.75) | < 0.001 |
| Middle-aged and elderly age | Prevalence | Both | Hungary | MS | -1.1<br>(-1.31 to -0.9) | < 0.001 |
| Middle-aged and elderly age | Prevalence | Female | Hungary | MS | -1.3<br>(-1.63 to -0.96) | < 0.001 |
| Middle-aged and elderly age | Prevalence | Male | Hungary | MS | -0.65<br>(-0.92 to -0.38) | < 0.001 |
| Middle-aged and elderly age | Prevalence | Both | Hungary | Psoriasis | -0.84<br>(-0.88 to -0.81) | < 0.001 |
| Middle-aged and elderly age | Prevalence | Female | Hungary | Psoriasis | -0.87<br>(-0.92 to -0.81) | < 0.001 |
| Middle-aged and elderly age | Prevalence | Male | Hungary | Psoriasis | -0.81<br>(-0.84 to -0.78) | < 0.001 |
| Middle-aged and elderly age | Prevalence | Both | Hungary | RHD | -2.19<br>(-2.35 to -2.04) | < 0.001 |
| Middle-aged and elderly age | Prevalence | Female | Hungary | RHD | -2.16<br>(-2.35 to -1.96) | < 0.001 |
| Middle-aged and elderly age | Prevalence | Male | Hungary | RHD | -2.2<br>(-2.3 to -2.1) | < 0.001 |
| Middle-aged and elderly age | Prevalence | Both | Hungary | RA | 0.36<br>(0.3 to 0.42) | < 0.001 |
| Middle-aged and elderly age | Prevalence | Female | Hungary | RA | 0.39<br>(0.31 to 0.47) | < 0.001 |
| Middle-aged and elderly age | Prevalence | Male | Hungary | RA | 0.47<br>(0.39 to 0.56) | < 0.001 |
| Middle-aged and elderly age | Prevalence | Both | Iceland | AA | 0<br>(-0.03 to 0.03) | 0.982 |
| Middle-aged and elderly age | Prevalence | Female | Iceland | AA | 0<br>(0 to 0) | < 0.001 |
| Middle-aged and elderly age | Prevalence | Male | Iceland | AA | 0 | < 0.001 |

| | | | | | | |
|---|---|---|---|---|---|---|
| | | | | | (0 to 0) | |
| Middle-aged and elderly age | Prevalence | Both | Iceland | T1D | 1.41 (1.27 to 1.56) | < 0.001 |
| Middle-aged and elderly age | Prevalence | Female | Iceland | T1D | 1.28 (0.89 to 1.66) | < 0.001 |
| Middle-aged and elderly age | Prevalence | Male | Iceland | T1D | 1.37 (0.97 to 1.76) | < 0.001 |
| Middle-aged and elderly age | Prevalence | Both | Iceland | IBD | 1.37 (1.22 to 1.52) | < 0.001 |
| Middle-aged and elderly age | Prevalence | Female | Iceland | IBD | 1.4 (1.22 to 1.59) | < 0.001 |
| Middle-aged and elderly age | Prevalence | Male | Iceland | IBD | 1.29 (1.21 to 1.38) | < 0.001 |
| Middle-aged and elderly age | Prevalence | Both | Iceland | MS | 0.09 (0 to 0.19) | 0.062 |
| Middle-aged and elderly age | Prevalence | Female | Iceland | MS | 0.11 (0.03 to 0.2) | 0.008 |
| Middle-aged and elderly age | Prevalence | Male | Iceland | MS | -0.02 (-0.09 to 0.04) | 0.514 |
| Middle-aged and elderly age | Prevalence | Both | Iceland | Psoriasis | -0.43 (-0.45 to -0.41) | < 0.001 |
| Middle-aged and elderly age | Prevalence | Female | Iceland | Psoriasis | -0.43 (-0.45 to -0.41) | < 0.001 |
| Middle-aged and elderly age | Prevalence | Male | Iceland | Psoriasis | -0.43 (-0.44 to -0.42) | < 0.001 |
| Middle-aged and elderly age | Prevalence | Both | Iceland | RHD | -0.56 (-0.7 to -0.42) | < 0.001 |
| Middle-aged and elderly age | Prevalence | Female | Iceland | RHD | -1.07 (-1.55 to -0.59) | < 0.001 |
| Middle-aged and elderly age | Prevalence | Male | Iceland | RHD | 0.06 (-0.09 to 0.21) | 0.418 |
| Middle-aged and elderly age | Prevalence | Both | Iceland | RA | 0.3 (0.26 to 0.34) | < 0.001 |

| | | | | | | |
|---|---|---|---|---|---|---|
| Middle-aged and elderly age | Prevalence | Female | Iceland | RA | 0.27<br>(0.22 to 0.32) | < 0.001 |
| Middle-aged and elderly age | Prevalence | Male | Iceland | RA | 0.43<br>(0.39 to 0.46) | < 0.001 |
| Middle-aged and elderly age | Prevalence | Both | India | AA | 0.06<br>(0.06 to 0.07) | < 0.001 |
| Middle-aged and elderly age | Prevalence | Female | India | AA | 0<br>(0 to 0) | < 0.001 |
| Middle-aged and elderly age | Prevalence | Male | India | AA | 0<br>(0 to 0) | 0.013 |
| Middle-aged and elderly age | Prevalence | Both | India | T1D | 0.96<br>(0.69 to 1.22) | < 0.001 |
| Middle-aged and elderly age | Prevalence | Female | India | T1D | 0.97<br>(0.86 to 1.08) | < 0.001 |
| Middle-aged and elderly age | Prevalence | Male | India | T1D | 1.05<br>(0.87 to 1.22) | < 0.001 |
| Middle-aged and elderly age | Prevalence | Both | India | IBD | 0.07<br>(-0.11 to 0.24) | 0.455 |
| Middle-aged and elderly age | Prevalence | Female | India | IBD | 0.18<br>(0.1 to 0.26) | < 0.001 |
| Middle-aged and elderly age | Prevalence | Male | India | IBD | 0<br>(-0.31 to 0.31) | 0.997 |
| Middle-aged and elderly age | Prevalence | Both | India | MS | 0.54<br>(0.5 to 0.58) | < 0.001 |
| Middle-aged and elderly age | Prevalence | Female | India | MS | 0.48<br>(0.46 to 0.5) | < 0.001 |
| Middle-aged and elderly age | Prevalence | Male | India | MS | 0.54<br>(0.46 to 0.61) | < 0.001 |
| Middle-aged and elderly age | Prevalence | Both | India | Psoriasis | -0.13<br>(-0.17 to -0.09) | < 0.001 |
| Middle-aged and elderly age | Prevalence | Female | India | Psoriasis | -0.15<br>(-0.19 to -0.11) | < 0.001 |
| Middle-aged and elderly age | Prevalence | Male | India | Psoriasis | -0.13 | < 0.001 |

| | | | | | | |
|---|---|---|---|---|---|---|
| | | | | | (-0.16 to -0.09) | |
| Middle-aged and elderly age | Prevalence | Both | India | RHD | 0.19<br>(0.04 to 0.34) | 0.016 |
| Middle-aged and elderly age | Prevalence | Female | India | RHD | 0.11<br>(-0.11 to 0.33) | 0.323 |
| Middle-aged and elderly age | Prevalence | Male | India | RHD | 0.16<br>(-0.02 to 0.35) | 0.088 |
| Middle-aged and elderly age | Prevalence | Both | India | RA | 0.39<br>(0.37 to 0.41) | < 0.001 |
| Middle-aged and elderly age | Prevalence | Female | India | RA | 0.28<br>(0.25 to 0.3) | < 0.001 |
| Middle-aged and elderly age | Prevalence | Male | India | RA | 0.35<br>(0.31 to 0.39) | < 0.001 |
| Middle-aged and elderly age | Prevalence | Both | Indonesia | AA | -0.02<br>(-0.02 to -0.01) | < 0.001 |
| Middle-aged and elderly age | Prevalence | Female | Indonesia | AA | 0<br>(NA to NA) | . |
| Middle-aged and elderly age | Prevalence | Male | Indonesia | AA | 0<br>(0 to 0) | < 0.001 |
| Middle-aged and elderly age | Prevalence | Both | Indonesia | T1D | 1.09<br>(1.07 to 1.11) | < 0.001 |
| Middle-aged and elderly age | Prevalence | Female | Indonesia | T1D | 1.04<br>(1.03 to 1.06) | < 0.001 |
| Middle-aged and elderly age | Prevalence | Male | Indonesia | T1D | 1.14<br>(1.1 to 1.18) | < 0.001 |
| Middle-aged and elderly age | Prevalence | Both | Indonesia | IBD | 0.69<br>(0.61 to 0.76) | < 0.001 |
| Middle-aged and elderly age | Prevalence | Female | Indonesia | IBD | 0.73<br>(0.68 to 0.79) | < 0.001 |
| Middle-aged and elderly age | Prevalence | Male | Indonesia | IBD | 0.64<br>(0.56 to 0.71) | < 0.001 |
| Middle-aged and elderly age | Prevalence | Both | Indonesia | MS | 0.22<br>(0.21 to 0.23) | < 0.001 |

| | | | | | | |
|---|---|---|---|---|---|---|
| Middle-aged and elderly age | Prevalence | Female | Indonesia | MS | 0.21 (0.17 to 0.25) | < 0.001 |
| Middle-aged and elderly age | Prevalence | Male | Indonesia | MS | 0.27 (0.24 to 0.31) | < 0.001 |
| Middle-aged and elderly age | Prevalence | Both | Indonesia | Psoriasis | -0.35 (-0.5 to -0.2) | < 0.001 |
| Middle-aged and elderly age | Prevalence | Female | Indonesia | Psoriasis | -0.33 (-0.41 to -0.25) | < 0.001 |
| Middle-aged and elderly age | Prevalence | Male | Indonesia | Psoriasis | -0.4 (-0.47 to -0.34) | < 0.001 |
| Middle-aged and elderly age | Prevalence | Both | Indonesia | RHD | -0.02 (-0.08 to 0.04) | 0.591 |
| Middle-aged and elderly age | Prevalence | Female | Indonesia | RHD | 0.18 (0.11 to 0.26) | < 0.001 |
| Middle-aged and elderly age | Prevalence | Male | Indonesia | RHD | -0.18 (-0.29 to -0.08) | 0.001 |
| Middle-aged and elderly age | Prevalence | Both | Indonesia | RA | 0 (-0.02 to 0.03) | 0.779 |
| Middle-aged and elderly age | Prevalence | Female | Indonesia | RA | 0.01 (0 to 0.03) | 0.105 |
| Middle-aged and elderly age | Prevalence | Male | Indonesia | RA | 0.09 (0.07 to 0.12) | < 0.001 |
| Middle-aged and elderly age | Prevalence | Both | Iran (Islamic Republic of) | AA | 0.06 (0.05 to 0.06) | < 0.001 |
| Middle-aged and elderly age | Prevalence | Female | Iran (Islamic Republic of) | AA | 0 (0 to 0) | < 0.001 |
| Middle-aged and elderly age | Prevalence | Male | Iran (Islamic Republic of) | AA | 0 (0 to 0) | < 0.001 |
| Middle-aged and elderly age | Prevalence | Both | Iran (Islamic Republic of) | T1D | 2.25 (2.14 to 2.36) | < 0.001 |
| Middle-aged and elderly age | Prevalence | Female | Iran (Islamic Republic of) | T1D | 2.28 (2.19 to 2.37) | < 0.001 |
| Middle-aged and elderly age | Prevalence | Male | Iran (Islamic Republic of) | T1D | 2.2 | < 0.001 |

| | | | | | | |
|---|---|---|---|---|---|---|
| | | | | | (2.01 to 2.38) | |
| Middle-aged and elderly age | Prevalence | Both | Iran (Islamic Republic of) | IBD | 0.41 (0.33 to 0.49) | < 0.001 |
| Middle-aged and elderly age | Prevalence | Female | Iran (Islamic Republic of) | IBD | 0.39 (0.3 to 0.48) | < 0.001 |
| Middle-aged and elderly age | Prevalence | Male | Iran (Islamic Republic of) | IBD | 0.44 (0.37 to 0.51) | < 0.001 |
| Middle-aged and elderly age | Prevalence | Both | Iran (Islamic Republic of) | MS | 0.03 (-0.16 to 0.22) | 0.751 |
| Middle-aged and elderly age | Prevalence | Female | Iran (Islamic Republic of) | MS | 0.16 (-0.17 to 0.5) | 0.340 |
| Middle-aged and elderly age | Prevalence | Male | Iran (Islamic Republic of) | MS | -0.33 (-0.48 to -0.18) | < 0.001 |
| Middle-aged and elderly age | Prevalence | Both | Iran (Islamic Republic of) | Psoriasis | -1.11 (-1.15 to -1.07) | < 0.001 |
| Middle-aged and elderly age | Prevalence | Female | Iran (Islamic Republic of) | Psoriasis | -1.12 (-1.16 to -1.08) | < 0.001 |
| Middle-aged and elderly age | Prevalence | Male | Iran (Islamic Republic of) | Psoriasis | -1.13 (-1.16 to -1.1) | < 0.001 |
| Middle-aged and elderly age | Prevalence | Both | Iran (Islamic Republic of) | RHD | 0.13 (0.11 to 0.15) | < 0.001 |
| Middle-aged and elderly age | Prevalence | Female | Iran (Islamic Republic of) | RHD | 0.14 (0.13 to 0.15) | < 0.001 |
| Middle-aged and elderly age | Prevalence | Male | Iran (Islamic Republic of) | RHD | 0.08 (0.04 to 0.12) | < 0.001 |
| Middle-aged and elderly age | Prevalence | Both | Iran (Islamic Republic of) | RA | 0.42 (0.37 to 0.46) | < 0.001 |
| Middle-aged and elderly age | Prevalence | Female | Iran (Islamic Republic of) | RA | 0.35 (0.26 to 0.45) | < 0.001 |
| Middle-aged and elderly age | Prevalence | Male | Iran (Islamic Republic of) | RA | 0.22 (0.15 to 0.29) | < 0.001 |
| Middle-aged and elderly age | Prevalence | Both | Iraq | AA | 0 (-0.01 to 0) | 0.265 |

| | | | | | | |
|---|---|---|---|---|---|---|
| Middle-aged and elderly age | Prevalence | Female | Iraq | AA | 0<br>(0 to 0) | < 0.001 |
| Middle-aged and elderly age | Prevalence | Male | Iraq | AA | 0<br>(0 to 0) | 0.070 |
| Middle-aged and elderly age | Prevalence | Both | Iraq | T1D | 2.09<br>(2.05 to 2.14) | < 0.001 |
| Middle-aged and elderly age | Prevalence | Female | Iraq | T1D | 2.08<br>(2.05 to 2.12) | < 0.001 |
| Middle-aged and elderly age | Prevalence | Male | Iraq | T1D | 2.1<br>(2.04 to 2.16) | < 0.001 |
| Middle-aged and elderly age | Prevalence | Both | Iraq | IBD | 1.37<br>(1.23 to 1.51) | < 0.001 |
| Middle-aged and elderly age | Prevalence | Female | Iraq | IBD | 1.28<br>(1.15 to 1.41) | < 0.001 |
| Middle-aged and elderly age | Prevalence | Male | Iraq | IBD | 1.47<br>(1.29 to 1.65) | < 0.001 |
| Middle-aged and elderly age | Prevalence | Both | Iraq | MS | 0.64<br>(0.61 to 0.67) | < 0.001 |
| Middle-aged and elderly age | Prevalence | Female | Iraq | MS | 0.62<br>(0.57 to 0.66) | < 0.001 |
| Middle-aged and elderly age | Prevalence | Male | Iraq | MS | 0.7<br>(0.66 to 0.74) | < 0.001 |
| Middle-aged and elderly age | Prevalence | Both | Iraq | Psoriasis | -1.12<br>(-1.15 to -1.08) | < 0.001 |
| Middle-aged and elderly age | Prevalence | Female | Iraq | Psoriasis | -1.16<br>(-1.21 to -1.1) | < 0.001 |
| Middle-aged and elderly age | Prevalence | Male | Iraq | Psoriasis | -1.06<br>(-1.11 to -1.02) | < 0.001 |
| Middle-aged and elderly age | Prevalence | Both | Iraq | RHD | 0.04<br>(0.04 to 0.05) | < 0.001 |
| Middle-aged and elderly age | Prevalence | Female | Iraq | RHD | 0.06<br>(0.05 to 0.07) | < 0.001 |
| Middle-aged and elderly age | Prevalence | Male | Iraq | RHD | 0.03 | < 0.001 |

| | | | | | | |
|---|---|---|---|---|---|---|
| | | | | | (0.02 to 0.03) | |
| Middle-aged and elderly age | Prevalence | Both | Iraq | RA | 0.85<br>(0.78 to 0.93) | < 0.001 |
| Middle-aged and elderly age | Prevalence | Female | Iraq | RA | 0.9<br>(0.83 to 0.98) | < 0.001 |
| Middle-aged and elderly age | Prevalence | Male | Iraq | RA | 0.74<br>(0.66 to 0.82) | < 0.001 |
| Middle-aged and elderly age | Prevalence | Both | Ireland | AA | 0.03<br>(0.03 to 0.04) | < 0.001 |
| Middle-aged and elderly age | Prevalence | Female | Ireland | AA | 0<br>(0 to 0) | < 0.001 |
| Middle-aged and elderly age | Prevalence | Male | Ireland | AA | 0<br>(0 to 0) | < 0.001 |
| Middle-aged and elderly age | Prevalence | Both | Ireland | T1D | 2.51<br>(2.18 to 2.84) | < 0.001 |
| Middle-aged and elderly age | Prevalence | Female | Ireland | T1D | 2.33<br>(2.11 to 2.55) | < 0.001 |
| Middle-aged and elderly age | Prevalence | Male | Ireland | T1D | 2.66<br>(2.23 to 3.1) | < 0.001 |
| Middle-aged and elderly age | Prevalence | Both | Ireland | IBD | 0.11<br>(-0.03 to 0.25) | 0.129 |
| Middle-aged and elderly age | Prevalence | Female | Ireland | IBD | 0.37<br>(0.27 to 0.46) | < 0.001 |
| Middle-aged and elderly age | Prevalence | Male | Ireland | IBD | -0.13<br>(-0.3 to 0.05) | 0.160 |
| Middle-aged and elderly age | Prevalence | Both | Ireland | MS | 0.31<br>(0.27 to 0.35) | < 0.001 |
| Middle-aged and elderly age | Prevalence | Female | Ireland | MS | 0.32<br>(0.28 to 0.36) | < 0.001 |
| Middle-aged and elderly age | Prevalence | Male | Ireland | MS | 0.22<br>(0.19 to 0.25) | < 0.001 |
| Middle-aged and elderly age | Prevalence | Both | Ireland | Psoriasis | -0.54<br>(-0.55 to -0.54) | < 0.001 |

| | | | | | | |
|---|---|---|---|---|---|---|
| Middle-aged and elderly age | Prevalence | Female | Ireland | Psoriasis | -0.54 (-0.54 to -0.53) | < 0.001 |
| Middle-aged and elderly age | Prevalence | Male | Ireland | Psoriasis | -0.54 (-0.55 to -0.53) | < 0.001 |
| Middle-aged and elderly age | Prevalence | Both | Ireland | RHD | -1.37 (-1.49 to -1.25) | < 0.001 |
| Middle-aged and elderly age | Prevalence | Female | Ireland | RHD | -1.87 (-1.98 to -1.75) | < 0.001 |
| Middle-aged and elderly age | Prevalence | Male | Ireland | RHD | -0.63 (-0.75 to -0.5) | < 0.001 |
| Middle-aged and elderly age | Prevalence | Both | Ireland | RA | 0.51 (0.35 to 0.67) | < 0.001 |
| Middle-aged and elderly age | Prevalence | Female | Ireland | RA | 0.43 (0.27 to 0.59) | < 0.001 |
| Middle-aged and elderly age | Prevalence | Male | Ireland | RA | 0.59 (0.46 to 0.72) | < 0.001 |
| Middle-aged and elderly age | Prevalence | Both | Israel | AA | -0.04 (-0.05 to -0.04) | < 0.001 |
| Middle-aged and elderly age | Prevalence | Female | Israel | AA | 0 (0 to 0) | < 0.001 |
| Middle-aged and elderly age | Prevalence | Male | Israel | AA | 0 (0 to 0) | < 0.001 |
| Middle-aged and elderly age | Prevalence | Both | Israel | T1D | 0.72 (0.56 to 0.89) | < 0.001 |
| Middle-aged and elderly age | Prevalence | Female | Israel | T1D | 0.57 (0.44 to 0.7) | < 0.001 |
| Middle-aged and elderly age | Prevalence | Male | Israel | T1D | 0.99 (0.65 to 1.34) | < 0.001 |
| Middle-aged and elderly age | Prevalence | Both | Israel | IBD | -0.34 (-0.41 to -0.27) | < 0.001 |
| Middle-aged and elderly age | Prevalence | Female | Israel | IBD | -0.32 (-0.4 to -0.24) | < 0.001 |
| Middle-aged and elderly age | Prevalence | Male | Israel | IBD | -0.37 | < 0.001 |

| | | | | | | |
|---|---|---|---|---|---|---|
| | | | | | (-0.44 to -0.31) | |
| Middle-aged and elderly age | Prevalence | Both | Israel | MS | 0.42 (0.39 to 0.46) | < 0.001 |
| Middle-aged and elderly age | Prevalence | Female | Israel | MS | 0.47 (0.44 to 0.49) | < 0.001 |
| Middle-aged and elderly age | Prevalence | Male | Israel | MS | 0.42 (0.37 to 0.47) | < 0.001 |
| Middle-aged and elderly age | Prevalence | Both | Israel | Psoriasis | -0.36 (-0.37 to -0.36) | < 0.001 |
| Middle-aged and elderly age | Prevalence | Female | Israel | Psoriasis | -0.37 (-0.38 to -0.36) | < 0.001 |
| Middle-aged and elderly age | Prevalence | Male | Israel | Psoriasis | -0.36 (-0.37 to -0.36) | < 0.001 |
| Middle-aged and elderly age | Prevalence | Both | Israel | RHD | -1.22 (-1.34 to -1.11) | < 0.001 |
| Middle-aged and elderly age | Prevalence | Female | Israel | RHD | -1.69 (-1.9 to -1.48) | < 0.001 |
| Middle-aged and elderly age | Prevalence | Male | Israel | RHD | -0.47 (-0.54 to -0.41) | < 0.001 |
| Middle-aged and elderly age | Prevalence | Both | Israel | RA | 0.63 (0.58 to 0.68) | < 0.001 |
| Middle-aged and elderly age | Prevalence | Female | Israel | RA | 0.71 (0.65 to 0.76) | < 0.001 |
| Middle-aged and elderly age | Prevalence | Male | Israel | RA | 0.57 (0.55 to 0.6) | < 0.001 |
| Middle-aged and elderly age | Prevalence | Both | Italy | AA | -0.02 (-0.02 to -0.01) | < 0.001 |
| Middle-aged and elderly age | Prevalence | Female | Italy | AA | 0 (0 to 0) | < 0.001 |
| Middle-aged and elderly age | Prevalence | Male | Italy | AA | 0 (0 to 0) | < 0.001 |
| Middle-aged and elderly age | Prevalence | Both | Italy | T1D | 1.04 (0.79 to 1.28) | < 0.001 |

| | | | | | | |
|---|---|---|---|---|---|---|
| Middle-aged and elderly age | Prevalence | Female | Italy | T1D | 0.98<br>(0.7 to 1.26) | < 0.001 |
| Middle-aged and elderly age | Prevalence | Male | Italy | T1D | 1.09<br>(0.86 to 1.33) | < 0.001 |
| Middle-aged and elderly age | Prevalence | Both | Italy | IBD | -0.57<br>(-0.66 to -0.49) | < 0.001 |
| Middle-aged and elderly age | Prevalence | Female | Italy | IBD | -0.58<br>(-0.66 to -0.49) | < 0.001 |
| Middle-aged and elderly age | Prevalence | Male | Italy | IBD | -0.57<br>(-0.66 to -0.48) | < 0.001 |
| Middle-aged and elderly age | Prevalence | Both | Italy | MS | 0.94<br>(0.89 to 1) | < 0.001 |
| Middle-aged and elderly age | Prevalence | Female | Italy | MS | 1<br>(0.92 to 1.09) | < 0.001 |
| Middle-aged and elderly age | Prevalence | Male | Italy | MS | 0.87<br>(0.85 to 0.89) | < 0.001 |
| Middle-aged and elderly age | Prevalence | Both | Italy | Psoriasis | -0.26<br>(-0.34 to -0.19) | < 0.001 |
| Middle-aged and elderly age | Prevalence | Female | Italy | Psoriasis | -0.27<br>(-0.35 to -0.2) | < 0.001 |
| Middle-aged and elderly age | Prevalence | Male | Italy | Psoriasis | -0.25<br>(-0.33 to -0.18) | < 0.001 |
| Middle-aged and elderly age | Prevalence | Both | Italy | RHD | -1.87<br>(-2.05 to -1.69) | < 0.001 |
| Middle-aged and elderly age | Prevalence | Female | Italy | RHD | -2.23<br>(-2.46 to -1.99) | < 0.001 |
| Middle-aged and elderly age | Prevalence | Male | Italy | RHD | -1.36<br>(-1.48 to -1.25) | < 0.001 |
| Middle-aged and elderly age | Prevalence | Both | Italy | RA | -0.04<br>(-0.05 to -0.04) | < 0.001 |
| Middle-aged and elderly age | Prevalence | Female | Italy | RA | -0.03<br>(-0.04 to -0.03) | < 0.001 |
| Middle-aged and elderly age | Prevalence | Male | Italy | RA | 0 | 0.412 |

| | | | | | | |
|---|---|---|---|---|---|---|
| | | | | | (-0.01 to 0.01) | |
| Middle-aged and elderly age | Prevalence | Both | Jamaica | AA | -0.04<br>(-0.05 to -0.03) | < 0.001 |
| Middle-aged and elderly age | Prevalence | Female | Jamaica | AA | 0<br>(0 to 0) | < 0.001 |
| Middle-aged and elderly age | Prevalence | Male | Jamaica | AA | 0<br>(0 to 0) | < 0.001 |
| Middle-aged and elderly age | Prevalence | Both | Jamaica | T1D | 1.4<br>(1.38 to 1.41) | < 0.001 |
| Middle-aged and elderly age | Prevalence | Female | Jamaica | T1D | 1.3<br>(1.27 to 1.34) | < 0.001 |
| Middle-aged and elderly age | Prevalence | Male | Jamaica | T1D | 1.46<br>(1.45 to 1.46) | < 0.001 |
| Middle-aged and elderly age | Prevalence | Both | Jamaica | IBD | 0.17<br>(0.16 to 0.18) | < 0.001 |
| Middle-aged and elderly age | Prevalence | Female | Jamaica | IBD | 0.18<br>(0.15 to 0.21) | < 0.001 |
| Middle-aged and elderly age | Prevalence | Male | Jamaica | IBD | 0.17<br>(0.11 to 0.23) | < 0.001 |
| Middle-aged and elderly age | Prevalence | Both | Jamaica | MS | 0.44<br>(0.41 to 0.47) | < 0.001 |
| Middle-aged and elderly age | Prevalence | Female | Jamaica | MS | 0.53<br>(0.44 to 0.63) | < 0.001 |
| Middle-aged and elderly age | Prevalence | Male | Jamaica | MS | 0.42<br>(0.39 to 0.46) | < 0.001 |
| Middle-aged and elderly age | Prevalence | Both | Jamaica | Psoriasis | -0.46<br>(-0.5 to -0.43) | < 0.001 |
| Middle-aged and elderly age | Prevalence | Female | Jamaica | Psoriasis | -0.46<br>(-0.48 to -0.44) | < 0.001 |
| Middle-aged and elderly age | Prevalence | Male | Jamaica | Psoriasis | -0.47<br>(-0.51 to -0.42) | < 0.001 |
| Middle-aged and elderly age | Prevalence | Both | Jamaica | RHD | 0.17<br>(0.16 to 0.19) | < 0.001 |

| | | | | | | |
|---|---|---|---|---|---|---|
| Middle-aged and elderly age | Prevalence | Female | Jamaica | RHD | 0.22<br>(0.19 to 0.24) | < 0.001 |
| Middle-aged and elderly age | Prevalence | Male | Jamaica | RHD | 0.15<br>(0.09 to 0.2) | < 0.001 |
| Middle-aged and elderly age | Prevalence | Both | Jamaica | RA | 0.8<br>(0.73 to 0.86) | < 0.001 |
| Middle-aged and elderly age | Prevalence | Female | Jamaica | RA | 0.83<br>(0.77 to 0.9) | < 0.001 |
| Middle-aged and elderly age | Prevalence | Male | Jamaica | RA | 0.86<br>(0.8 to 0.92) | < 0.001 |
| Middle-aged and elderly age | Prevalence | Both | Japan | AA | -0.03<br>(-0.03 to -0.03) | < 0.001 |
| Middle-aged and elderly age | Prevalence | Female | Japan | AA | 0<br>(0 to 0) | < 0.001 |
| Middle-aged and elderly age | Prevalence | Male | Japan | AA | 0<br>(0 to 0) | < 0.001 |
| Middle-aged and elderly age | Prevalence | Both | Japan | T1D | 0.5<br>(0.47 to 0.54) | < 0.001 |
| Middle-aged and elderly age | Prevalence | Female | Japan | T1D | 0.51<br>(0.48 to 0.55) | < 0.001 |
| Middle-aged and elderly age | Prevalence | Male | Japan | T1D | 0.49<br>(0.45 to 0.53) | < 0.001 |
| Middle-aged and elderly age | Prevalence | Both | Japan | IBD | 0.97<br>(0.91 to 1.04) | < 0.001 |
| Middle-aged and elderly age | Prevalence | Female | Japan | IBD | 1.07<br>(1.01 to 1.13) | < 0.001 |
| Middle-aged and elderly age | Prevalence | Male | Japan | IBD | 0.89<br>(0.83 to 0.96) | < 0.001 |
| Middle-aged and elderly age | Prevalence | Both | Japan | MS | 0.54<br>(0.5 to 0.58) | < 0.001 |
| Middle-aged and elderly age | Prevalence | Female | Japan | MS | 0.76<br>(0.72 to 0.8) | < 0.001 |
| Middle-aged and elderly age | Prevalence | Male | Japan | MS | 0.15 | < 0.001 |

| | | | | | | |
|---|---|---|---|---|---|---|
| | | | | | (0.07 to 0.23) | |
| Middle-aged and elderly age | Prevalence | Both | Japan | Psoriasis | 0<br>(-0.03 to 0.02) | 0.749 |
| Middle-aged and elderly age | Prevalence | Female | Japan | Psoriasis | -0.08<br>(-0.13 to -0.03) | 0.001 |
| Middle-aged and elderly age | Prevalence | Male | Japan | Psoriasis | 0.07<br>(0.05 to 0.09) | < 0.001 |
| Middle-aged and elderly age | Prevalence | Both | Japan | RHD | -1.71<br>(-1.81 to -1.62) | < 0.001 |
| Middle-aged and elderly age | Prevalence | Female | Japan | RHD | -1.88<br>(-2.03 to -1.73) | < 0.001 |
| Middle-aged and elderly age | Prevalence | Male | Japan | RHD | -1.34<br>(-1.43 to -1.25) | < 0.001 |
| Middle-aged and elderly age | Prevalence | Both | Japan | RA | 0.1<br>(-0.32 to 0.52) | 0.641 |
| Middle-aged and elderly age | Prevalence | Female | Japan | RA | 0.14<br>(-0.4 to 0.68) | 0.612 |
| Middle-aged and elderly age | Prevalence | Male | Japan | RA | 0.1<br>(-0.04 to 0.25) | 0.174 |
| Middle-aged and elderly age | Prevalence | Both | Jordan | AA | -0.02<br>(-0.03 to 0) | 0.014 |
| Middle-aged and elderly age | Prevalence | Female | Jordan | AA | 0<br>(0 to 0) | < 0.001 |
| Middle-aged and elderly age | Prevalence | Male | Jordan | AA | 0<br>(0 to 0) | 0.015 |
| Middle-aged and elderly age | Prevalence | Both | Jordan | T1D | 2.57<br>(2.51 to 2.63) | < 0.001 |
| Middle-aged and elderly age | Prevalence | Female | Jordan | T1D | 2.55<br>(2.48 to 2.62) | < 0.001 |
| Middle-aged and elderly age | Prevalence | Male | Jordan | T1D | 2.61<br>(2.56 to 2.66) | < 0.001 |
| Middle-aged and elderly age | Prevalence | Both | Jordan | IBD | 2.05<br>(1.79 to 2.31) | < 0.001 |

| | | | | | | |
|---|---|---|---|---|---|---|
| Middle-aged and elderly age | Prevalence | Female | Jordan | IBD | 1.87<br>(1.42 to 2.34) | < 0.001 |
| Middle-aged and elderly age | Prevalence | Male | Jordan | IBD | 2.16<br>(2.05 to 2.27) | < 0.001 |
| Middle-aged and elderly age | Prevalence | Both | Jordan | MS | 0.07<br>(0.01 to 0.13) | 0.013 |
| Middle-aged and elderly age | Prevalence | Female | Jordan | MS | 0.1<br>(0.04 to 0.15) | < 0.001 |
| Middle-aged and elderly age | Prevalence | Male | Jordan | MS | 0.05<br>(0.03 to 0.07) | < 0.001 |
| Middle-aged and elderly age | Prevalence | Both | Jordan | Psoriasis | -0.87<br>(-0.88 to -0.86) | < 0.001 |
| Middle-aged and elderly age | Prevalence | Female | Jordan | Psoriasis | -0.88<br>(-0.89 to -0.87) | < 0.001 |
| Middle-aged and elderly age | Prevalence | Male | Jordan | Psoriasis | -0.86<br>(-0.88 to -0.84) | < 0.001 |
| Middle-aged and elderly age | Prevalence | Both | Jordan | RHD | -0.93<br>(-1.11 to -0.74) | < 0.001 |
| Middle-aged and elderly age | Prevalence | Female | Jordan | RHD | -1.46<br>(-1.52 to -1.39) | < 0.001 |
| Middle-aged and elderly age | Prevalence | Male | Jordan | RHD | -0.37<br>(-0.56 to -0.19) | < 0.001 |
| Middle-aged and elderly age | Prevalence | Both | Jordan | RA | 1.08<br>(1.01 to 1.16) | < 0.001 |
| Middle-aged and elderly age | Prevalence | Female | Jordan | RA | 1.14<br>(1.07 to 1.21) | < 0.001 |
| Middle-aged and elderly age | Prevalence | Male | Jordan | RA | 1.08<br>(0.97 to 1.19) | < 0.001 |
| Middle-aged and elderly age | Prevalence | Both | Kazakhstan | AA | 0.01<br>(0 to 0.01) | 0.001 |
| Middle-aged and elderly age | Prevalence | Female | Kazakhstan | AA | 0<br>(0 to 0) | < 0.001 |
| Middle-aged and elderly age | Prevalence | Male | Kazakhstan | AA | 0 | < 0.001 |

| | | | | | | |
|---|---|---|---|---|---|---|
| | | | | | (0 to 0) | |
| Middle-aged and elderly age | Prevalence | Both | Kazakhstan | T1D | 1.21<br>(1.2 to 1.22) | < 0.001 |
| Middle-aged and elderly age | Prevalence | Female | Kazakhstan | T1D | 1.19<br>(1.18 to 1.21) | < 0.001 |
| Middle-aged and elderly age | Prevalence | Male | Kazakhstan | T1D | 1.23<br>(1.22 to 1.24) | < 0.001 |
| Middle-aged and elderly age | Prevalence | Both | Kazakhstan | IBD | 0.08<br>(0.06 to 0.11) | < 0.001 |
| Middle-aged and elderly age | Prevalence | Female | Kazakhstan | IBD | 0.06<br>(0.02 to 0.1) | 0.001 |
| Middle-aged and elderly age | Prevalence | Male | Kazakhstan | IBD | 0.11<br>(0.08 to 0.14) | < 0.001 |
| Middle-aged and elderly age | Prevalence | Both | Kazakhstan | MS | 0.04<br>(-0.1 to 0.19) | 0.556 |
| Middle-aged and elderly age | Prevalence | Female | Kazakhstan | MS | 0.04<br>(-0.05 to 0.12) | 0.365 |
| Middle-aged and elderly age | Prevalence | Male | Kazakhstan | MS | 0.02<br>(-0.1 to 0.14) | 0.743 |
| Middle-aged and elderly age | Prevalence | Both | Kazakhstan | Psoriasis | -0.8<br>(-0.83 to -0.77) | < 0.001 |
| Middle-aged and elderly age | Prevalence | Female | Kazakhstan | Psoriasis | -0.8<br>(-0.84 to -0.77) | < 0.001 |
| Middle-aged and elderly age | Prevalence | Male | Kazakhstan | Psoriasis | -0.78<br>(-0.83 to -0.74) | < 0.001 |
| Middle-aged and elderly age | Prevalence | Both | Kazakhstan | RHD | -2.36<br>(-2.68 to -2.04) | < 0.001 |
| Middle-aged and elderly age | Prevalence | Female | Kazakhstan | RHD | -2.47<br>(-2.77 to -2.16) | < 0.001 |
| Middle-aged and elderly age | Prevalence | Male | Kazakhstan | RHD | -2.15<br>(-2.3 to -2) | < 0.001 |
| Middle-aged and elderly age | Prevalence | Both | Kazakhstan | RA | 1.01<br>(0.93 to 1.09) | < 0.001 |

| | | | | | | |
|---|---|---|---|---|---|---|
| Middle-aged and elderly age | Prevalence | Female | Kazakhstan | RA | 1.01 (0.94 to 1.08) | < 0.001 |
| Middle-aged and elderly age | Prevalence | Male | Kazakhstan | RA | 0.95 (0.89 to 1.02) | < 0.001 |
| Middle-aged and elderly age | Prevalence | Both | Kenya | AA | -0.02 (-0.04 to -0.01) | 0.008 |
| Middle-aged and elderly age | Prevalence | Female | Kenya | AA | 0 (0 to 0) | < 0.001 |
| Middle-aged and elderly age | Prevalence | Male | Kenya | AA | 0 (0 to 0) | < 0.001 |
| Middle-aged and elderly age | Prevalence | Both | Kenya | T1D | 0.43 (0.42 to 0.44) | < 0.001 |
| Middle-aged and elderly age | Prevalence | Female | Kenya | T1D | 0.45 (0.44 to 0.45) | < 0.001 |
| Middle-aged and elderly age | Prevalence | Male | Kenya | T1D | 0.41 (0.39 to 0.44) | < 0.001 |
| Middle-aged and elderly age | Prevalence | Both | Kenya | IBD | 0.63 (0.52 to 0.73) | < 0.001 |
| Middle-aged and elderly age | Prevalence | Female | Kenya | IBD | 0.55 (0.46 to 0.64) | < 0.001 |
| Middle-aged and elderly age | Prevalence | Male | Kenya | IBD | 0.69 (0.63 to 0.75) | < 0.001 |
| Middle-aged and elderly age | Prevalence | Both | Kenya | MS | 0.19 (0.18 to 0.21) | < 0.001 |
| Middle-aged and elderly age | Prevalence | Female | Kenya | MS | 0.16 (0.14 to 0.17) | < 0.001 |
| Middle-aged and elderly age | Prevalence | Male | Kenya | MS | 0.32 (0.3 to 0.33) | < 0.001 |
| Middle-aged and elderly age | Prevalence | Both | Kenya | Psoriasis | -0.4 (-0.42 to -0.38) | < 0.001 |
| Middle-aged and elderly age | Prevalence | Female | Kenya | Psoriasis | -0.43 (-0.44 to -0.42) | < 0.001 |
| Middle-aged and elderly age | Prevalence | Male | Kenya | Psoriasis | -0.37 | < 0.001 |

| | | | | | | |
|---|---|---|---|---|---|---|
| | | | | | (-0.39 to -0.34) | |
| Middle-aged and elderly age | Prevalence | Both | Kenya | RHD | 0.1<br>(0.07 to 0.14) | < 0.001 |
| Middle-aged and elderly age | Prevalence | Female | Kenya | RHD | 0.18<br>(0.16 to 0.19) | < 0.001 |
| Middle-aged and elderly age | Prevalence | Male | Kenya | RHD | 0.03<br>(-0.01 to 0.07) | 0.090 |
| Middle-aged and elderly age | Prevalence | Both | Kenya | RA | -0.05<br>(-0.09 to -0.01) | 0.011 |
| Middle-aged and elderly age | Prevalence | Female | Kenya | RA | -0.11<br>(-0.14 to -0.09) | < 0.001 |
| Middle-aged and elderly age | Prevalence | Male | Kenya | RA | -0.05<br>(-0.09 to -0.01) | 0.027 |
| Middle-aged and elderly age | Prevalence | Both | Kiribati | AA | 0.01<br>(-0.01 to 0.02) | 0.283 |
| Middle-aged and elderly age | Prevalence | Female | Kiribati | AA | 0<br>(0 to 0) | < 0.001 |
| Middle-aged and elderly age | Prevalence | Male | Kiribati | AA | 0<br>(0 to 0) | < 0.001 |
| Middle-aged and elderly age | Prevalence | Both | Kiribati | T1D | 0.89<br>(0.88 to 0.91) | < 0.001 |
| Middle-aged and elderly age | Prevalence | Female | Kiribati | T1D | 0.86<br>(0.84 to 0.88) | < 0.001 |
| Middle-aged and elderly age | Prevalence | Male | Kiribati | T1D | 0.91<br>(0.87 to 0.95) | < 0.001 |
| Middle-aged and elderly age | Prevalence | Both | Kiribati | IBD | -0.71<br>(-0.88 to -0.54) | < 0.001 |
| Middle-aged and elderly age | Prevalence | Female | Kiribati | IBD | -1.02<br>(-1.13 to -0.91) | < 0.001 |
| Middle-aged and elderly age | Prevalence | Male | Kiribati | IBD | -0.39<br>(-0.49 to -0.28) | < 0.001 |
| Middle-aged and elderly age | Prevalence | Both | Kiribati | MS | 0.15<br>(0.07 to 0.22) | < 0.001 |

| | | | | | | |
|---|---|---|---|---|---|---|
| Middle-aged and elderly age | Prevalence | Female | Kiribati | MS | 0.17<br>(0.1 to 0.23) | < 0.001 |
| Middle-aged and elderly age | Prevalence | Male | Kiribati | MS | 0.07<br>(-0.05 to 0.2) | 0.252 |
| Middle-aged and elderly age | Prevalence | Both | Kiribati | Psoriasis | -0.16<br>(-0.18 to -0.13) | < 0.001 |
| Middle-aged and elderly age | Prevalence | Female | Kiribati | Psoriasis | -0.12<br>(-0.17 to -0.08) | < 0.001 |
| Middle-aged and elderly age | Prevalence | Male | Kiribati | Psoriasis | -0.16<br>(-0.21 to -0.12) | < 0.001 |
| Middle-aged and elderly age | Prevalence | Both | Kiribati | RHD | 0.08<br>(0.08 to 0.09) | < 0.001 |
| Middle-aged and elderly age | Prevalence | Female | Kiribati | RHD | 0.09<br>(0.09 to 0.1) | < 0.001 |
| Middle-aged and elderly age | Prevalence | Male | Kiribati | RHD | 0.06<br>(0.02 to 0.1) | 0.006 |
| Middle-aged and elderly age | Prevalence | Both | Kiribati | RA | 0.25<br>(0.19 to 0.32) | < 0.001 |
| Middle-aged and elderly age | Prevalence | Female | Kiribati | RA | 0.26<br>(0.18 to 0.35) | < 0.001 |
| Middle-aged and elderly age | Prevalence | Male | Kiribati | RA | 0.19<br>(0.13 to 0.25) | < 0.001 |
| Middle-aged and elderly age | Prevalence | Both | Kuwait | AA | 0.19<br>(0.16 to 0.22) | < 0.001 |
| Middle-aged and elderly age | Prevalence | Female | Kuwait | AA | 0<br>(0 to 0) | < 0.001 |
| Middle-aged and elderly age | Prevalence | Male | Kuwait | AA | 0<br>(0 to 0) | 0.015 |
| Middle-aged and elderly age | Prevalence | Both | Kuwait | T1D | 1.48<br>(0.64 to 2.33) | 0.001 |
| Middle-aged and elderly age | Prevalence | Female | Kuwait | T1D | 1.47<br>(1.2 to 1.74) | < 0.001 |
| Middle-aged and elderly age | Prevalence | Male | Kuwait | T1D | 1.62 | < 0.001 |

| | | | | | | |
|---|---|---|---|---|---|---|
| | | | | | (0.83 to 2.42) | |
| Middle-aged and elderly age | Prevalence | Both | Kuwait | IBD | 0.38 (0.27 to 0.49) | < 0.001 |
| Middle-aged and elderly age | Prevalence | Female | Kuwait | IBD | 0.27 (0.16 to 0.38) | < 0.001 |
| Middle-aged and elderly age | Prevalence | Male | Kuwait | IBD | 0.31 (0.25 to 0.38) | < 0.001 |
| Middle-aged and elderly age | Prevalence | Both | Kuwait | MS | 1.61 (1.53 to 1.69) | < 0.001 |
| Middle-aged and elderly age | Prevalence | Female | Kuwait | MS | 1.42 (1.34 to 1.5) | < 0.001 |
| Middle-aged and elderly age | Prevalence | Male | Kuwait | MS | 1.54 (1.46 to 1.61) | < 0.001 |
| Middle-aged and elderly age | Prevalence | Both | Kuwait | Psoriasis | -0.67 (-0.75 to -0.58) | < 0.001 |
| Middle-aged and elderly age | Prevalence | Female | Kuwait | Psoriasis | -0.68 (-0.74 to -0.63) | < 0.001 |
| Middle-aged and elderly age | Prevalence | Male | Kuwait | Psoriasis | -0.68 (-0.78 to -0.58) | < 0.001 |
| Middle-aged and elderly age | Prevalence | Both | Kuwait | RHD | -1.28 (-1.78 to -0.77) | < 0.001 |
| Middle-aged and elderly age | Prevalence | Female | Kuwait | RHD | -2.06 (-2.61 to -1.52) | < 0.001 |
| Middle-aged and elderly age | Prevalence | Male | Kuwait | RHD | -0.77 (-0.87 to -0.66) | < 0.001 |
| Middle-aged and elderly age | Prevalence | Both | Kuwait | RA | 1.1 (0.92 to 1.29) | < 0.001 |
| Middle-aged and elderly age | Prevalence | Female | Kuwait | RA | 0.89 (0.79 to 0.99) | < 0.001 |
| Middle-aged and elderly age | Prevalence | Male | Kuwait | RA | 0.81 (0.72 to 0.9) | < 0.001 |
| Middle-aged and elderly age | Prevalence | Both | Kyrgyzstan | AA | 0.01 (-0.01 to 0.02) | 0.479 |

| | | | | | | |
|---|---|---|---|---|---|---|
| Middle-aged and elderly age | Prevalence | Female | Kyrgyzstan | AA | 0<br>(0 to 0) | < 0.001 |
| Middle-aged and elderly age | Prevalence | Male | Kyrgyzstan | AA | 0<br>(0 to 0) | < 0.001 |
| Middle-aged and elderly age | Prevalence | Both | Kyrgyzstan | T1D | 1.13<br>(1.12 to 1.15) | < 0.001 |
| Middle-aged and elderly age | Prevalence | Female | Kyrgyzstan | T1D | 1.11<br>(1.1 to 1.13) | < 0.001 |
| Middle-aged and elderly age | Prevalence | Male | Kyrgyzstan | T1D | 1.15<br>(1.13 to 1.17) | < 0.001 |
| Middle-aged and elderly age | Prevalence | Both | Kyrgyzstan | IBD | -0.01<br>(-0.05 to 0.03) | 0.510 |
| Middle-aged and elderly age | Prevalence | Female | Kyrgyzstan | IBD | -0.04<br>(-0.08 to 0) | 0.062 |
| Middle-aged and elderly age | Prevalence | Male | Kyrgyzstan | IBD | 0<br>(-0.06 to 0.06) | 0.987 |
| Middle-aged and elderly age | Prevalence | Both | Kyrgyzstan | MS | 0.14<br>(0 to 0.28) | 0.043 |
| Middle-aged and elderly age | Prevalence | Female | Kyrgyzstan | MS | 0.15<br>(0 to 0.31) | 0.055 |
| Middle-aged and elderly age | Prevalence | Male | Kyrgyzstan | MS | 0.13<br>(0.01 to 0.26) | 0.036 |
| Middle-aged and elderly age | Prevalence | Both | Kyrgyzstan | Psoriasis | -0.55<br>(-0.61 to -0.49) | < 0.001 |
| Middle-aged and elderly age | Prevalence | Female | Kyrgyzstan | Psoriasis | -0.59<br>(-0.63 to -0.54) | < 0.001 |
| Middle-aged and elderly age | Prevalence | Male | Kyrgyzstan | Psoriasis | -0.53<br>(-0.61 to -0.45) | < 0.001 |
| Middle-aged and elderly age | Prevalence | Both | Kyrgyzstan | RHD | 0<br>(-0.01 to 0.01) | 0.829 |
| Middle-aged and elderly age | Prevalence | Female | Kyrgyzstan | RHD | 0.02<br>(0.01 to 0.03) | 0.003 |
| Middle-aged and elderly age | Prevalence | Male | Kyrgyzstan | RHD | -0.04 | 0.049 |

| | | | | | | |
|---|---|---|---|---|---|---|
| | | | | | (-0.07 to 0) | |
| Middle-aged and elderly age | Prevalence | Both | Kyrgyzstan | RA | 0.93<br>(0.9 to 0.97) | < 0.001 |
| Middle-aged and elderly age | Prevalence | Female | Kyrgyzstan | RA | 0.94<br>(0.92 to 0.97) | < 0.001 |
| Middle-aged and elderly age | Prevalence | Male | Kyrgyzstan | RA | 0.85<br>(0.79 to 0.91) | < 0.001 |
| Middle-aged and elderly age | Prevalence | Both | Lao People's Democratic Republic | AA | -0.03<br>(-0.05 to -0.01) | 0.003 |
| Middle-aged and elderly age | Prevalence | Female | Lao People's Democratic Republic | AA | 0<br>(0 to 0) | < 0.001 |
| Middle-aged and elderly age | Prevalence | Male | Lao People's Democratic Republic | AA | 0<br>(0 to 0) | < 0.001 |
| Middle-aged and elderly age | Prevalence | Both | Lao People's Democratic Republic | T1D | 0.66<br>(0.65 to 0.67) | < 0.001 |
| Middle-aged and elderly age | Prevalence | Female | Lao People's Democratic Republic | T1D | 0.6<br>(0.58 to 0.63) | < 0.001 |
| Middle-aged and elderly age | Prevalence | Male | Lao People's Democratic Republic | T1D | 0.69<br>(0.63 to 0.74) | < 0.001 |
| Middle-aged and elderly age | Prevalence | Both | Lao People's Democratic Republic | IBD | 0.53<br>(0.5 to 0.57) | < 0.001 |
| Middle-aged and elderly age | Prevalence | Female | Lao People's Democratic Republic | IBD | 0.36<br>(0.28 to 0.45) | < 0.001 |
| Middle-aged and elderly age | Prevalence | Male | Lao People's Democratic Republic | IBD | 0.76<br>(0.71 to 0.8) | < 0.001 |
| Middle-aged and elderly age | Prevalence | Both | Lao People's Democratic Republic | MS | 0.34<br>(0.3 to 0.39) | < 0.001 |
| Middle-aged and elderly age | Prevalence | Female | Lao People's Democratic Republic | MS | 0.41<br>(0.32 to 0.51) | < 0.001 |
| Middle-aged and elderly age | Prevalence | Male | Lao People's Democratic Republic | MS | 0.32<br>(0.26 to 0.39) | < 0.001 |
| Middle-aged and elderly age | Prevalence | Both | Lao People's Democratic Republic | Psoriasis | -0.65<br>(-0.7 to -0.61) | < 0.001 |

| | | | | | | |
|---|---|---|---|---|---|---|
| Middle-aged and elderly age | Prevalence | Female | Lao People's Democratic Republic | Psoriasis | -0.65<br>(-0.67 to -0.63) | < 0.001 |
| Middle-aged and elderly age | Prevalence | Male | Lao People's Democratic Republic | Psoriasis | -0.67<br>(-0.73 to -0.62) | < 0.001 |
| Middle-aged and elderly age | Prevalence | Both | Lao People's Democratic Republic | RHD | 0.03<br>(0.02 to 0.04) | < 0.001 |
| Middle-aged and elderly age | Prevalence | Female | Lao People's Democratic Republic | RHD | 0.07<br>(0.04 to 0.1) | < 0.001 |
| Middle-aged and elderly age | Prevalence | Male | Lao People's Democratic Republic | RHD | 0.03<br>(0.01 to 0.04) | < 0.001 |
| Middle-aged and elderly age | Prevalence | Both | Lao People's Democratic Republic | RA | 0.62<br>(0.59 to 0.65) | < 0.001 |
| Middle-aged and elderly age | Prevalence | Female | Lao People's Democratic Republic | RA | 0.75<br>(0.72 to 0.77) | < 0.001 |
| Middle-aged and elderly age | Prevalence | Male | Lao People's Democratic Republic | RA | 0.55<br>(0.53 to 0.56) | < 0.001 |
| Middle-aged and elderly age | Prevalence | Both | Latvia | AA | -0.02<br>(-0.03 to -0.02) | < 0.001 |
| Middle-aged and elderly age | Prevalence | Female | Latvia | AA | 0<br>(0 to 0) | < 0.001 |
| Middle-aged and elderly age | Prevalence | Male | Latvia | AA | 0<br>(0 to 0) | < 0.001 |
| Middle-aged and elderly age | Prevalence | Both | Latvia | T1D | 2.08<br>(2.04 to 2.12) | < 0.001 |
| Middle-aged and elderly age | Prevalence | Female | Latvia | T1D | 2.05<br>(2.02 to 2.09) | < 0.001 |
| Middle-aged and elderly age | Prevalence | Male | Latvia | T1D | 2.05<br>(2.01 to 2.09) | < 0.001 |
| Middle-aged and elderly age | Prevalence | Both | Latvia | IBD | 0.48<br>(0.44 to 0.52) | < 0.001 |
| Middle-aged and elderly age | Prevalence | Female | Latvia | IBD | 0.51<br>(0.42 to 0.6) | < 0.001 |
| Middle-aged and elderly age | Prevalence | Male | Latvia | IBD | 0.47 | < 0.001 |

| | | | | | | |
|---|---|---|---|---|---|---|
| | | | | | (0.43 to 0.52) | |
| Middle-aged and elderly age | Prevalence | Both | Latvia | MS | 0.07<br>(0.02 to 0.13) | 0.007 |
| Middle-aged and elderly age | Prevalence | Female | Latvia | MS | 0.11<br>(0.06 to 0.16) | < 0.001 |
| Middle-aged and elderly age | Prevalence | Male | Latvia | MS | 0.07<br>(-0.05 to 0.2) | 0.222 |
| Middle-aged and elderly age | Prevalence | Both | Latvia | Psoriasis | -0.76<br>(-0.79 to -0.72) | < 0.001 |
| Middle-aged and elderly age | Prevalence | Female | Latvia | Psoriasis | -0.75<br>(-0.79 to -0.72) | < 0.001 |
| Middle-aged and elderly age | Prevalence | Male | Latvia | Psoriasis | -0.75<br>(-0.79 to -0.72) | < 0.001 |
| Middle-aged and elderly age | Prevalence | Both | Latvia | RHD | -2.65<br>(-2.86 to -2.44) | < 0.001 |
| Middle-aged and elderly age | Prevalence | Female | Latvia | RHD | -2.65<br>(-2.95 to -2.34) | < 0.001 |
| Middle-aged and elderly age | Prevalence | Male | Latvia | RHD | -2.69<br>(-2.85 to -2.52) | < 0.001 |
| Middle-aged and elderly age | Prevalence | Both | Latvia | RA | 0.61<br>(0.53 to 0.69) | < 0.001 |
| Middle-aged and elderly age | Prevalence | Female | Latvia | RA | 0.66<br>(0.58 to 0.75) | < 0.001 |
| Middle-aged and elderly age | Prevalence | Male | Latvia | RA | 0.69<br>(0.61 to 0.77) | < 0.001 |
| Middle-aged and elderly age | Prevalence | Both | Lebanon | AA | 0.1<br>(0.07 to 0.12) | < 0.001 |
| Middle-aged and elderly age | Prevalence | Female | Lebanon | AA | 0<br>(0 to 0) | < 0.001 |
| Middle-aged and elderly age | Prevalence | Male | Lebanon | AA | 0<br>(0 to 0) | 0.015 |
| Middle-aged and elderly age | Prevalence | Both | Lebanon | T1D | 2.05<br>(1.97 to 2.12) | < 0.001 |

| | | | | | | |
|---|---|---|---|---|---|---|
| Middle-aged and elderly age | Prevalence | Female | Lebanon | T1D | 2.08<br>(2.02 to 2.14) | < 0.001 |
| Middle-aged and elderly age | Prevalence | Male | Lebanon | T1D | 2.07<br>(1.99 to 2.16) | < 0.001 |
| Middle-aged and elderly age | Prevalence | Both | Lebanon | IBD | 1.31<br>(1.17 to 1.45) | < 0.001 |
| Middle-aged and elderly age | Prevalence | Female | Lebanon | IBD | 1.36<br>(1.23 to 1.49) | < 0.001 |
| Middle-aged and elderly age | Prevalence | Male | Lebanon | IBD | 1.31<br>(1.17 to 1.46) | < 0.001 |
| Middle-aged and elderly age | Prevalence | Both | Lebanon | MS | 1.14<br>(1.11 to 1.18) | < 0.001 |
| Middle-aged and elderly age | Prevalence | Female | Lebanon | MS | 0.92<br>(0.88 to 0.97) | < 0.001 |
| Middle-aged and elderly age | Prevalence | Male | Lebanon | MS | 1.27<br>(1.26 to 1.28) | < 0.001 |
| Middle-aged and elderly age | Prevalence | Both | Lebanon | Psoriasis | -1.01<br>(-1.02 to -1) | < 0.001 |
| Middle-aged and elderly age | Prevalence | Female | Lebanon | Psoriasis | -1.02<br>(-1.03 to -1.01) | < 0.001 |
| Middle-aged and elderly age | Prevalence | Male | Lebanon | Psoriasis | -1.01<br>(-1.02 to -1.01) | < 0.001 |
| Middle-aged and elderly age | Prevalence | Both | Lebanon | RHD | -1.29<br>(-1.32 to -1.26) | < 0.001 |
| Middle-aged and elderly age | Prevalence | Female | Lebanon | RHD | -1.12<br>(-1.18 to -1.05) | < 0.001 |
| Middle-aged and elderly age | Prevalence | Male | Lebanon | RHD | -1.34<br>(-1.43 to -1.25) | < 0.001 |
| Middle-aged and elderly age | Prevalence | Both | Lebanon | RA | 1.32<br>(1.27 to 1.37) | < 0.001 |
| Middle-aged and elderly age | Prevalence | Female | Lebanon | RA | 1.27<br>(1.21 to 1.33) | < 0.001 |
| Middle-aged and elderly age | Prevalence | Male | Lebanon | RA | 1.11 | < 0.001 |

| | | | | | | |
|---|---|---|---|---|---|---|
| | | | | | (1.06 to 1.15) | |
| Middle-aged and elderly age | Prevalence | Both | Lesotho | AA | 0.09<br>(0.08 to 0.1) | < 0.001 |
| Middle-aged and elderly age | Prevalence | Female | Lesotho | AA | 0<br>(0 to 0) | 0.027 |
| Middle-aged and elderly age | Prevalence | Male | Lesotho | AA | 0<br>(0 to 0) | < 0.001 |
| Middle-aged and elderly age | Prevalence | Both | Lesotho | T1D | 0.39<br>(0.36 to 0.41) | < 0.001 |
| Middle-aged and elderly age | Prevalence | Female | Lesotho | T1D | 0.42<br>(0.4 to 0.44) | < 0.001 |
| Middle-aged and elderly age | Prevalence | Male | Lesotho | T1D | 0.38<br>(0.36 to 0.39) | < 0.001 |
| Middle-aged and elderly age | Prevalence | Both | Lesotho | IBD | 0.5<br>(0.45 to 0.56) | < 0.001 |
| Middle-aged and elderly age | Prevalence | Female | Lesotho | IBD | 0.49<br>(0.41 to 0.56) | < 0.001 |
| Middle-aged and elderly age | Prevalence | Male | Lesotho | IBD | 0.52<br>(0.47 to 0.58) | < 0.001 |
| Middle-aged and elderly age | Prevalence | Both | Lesotho | MS | 0.24<br>(0.16 to 0.33) | < 0.001 |
| Middle-aged and elderly age | Prevalence | Female | Lesotho | MS | 0.12<br>(0.03 to 0.21) | 0.007 |
| Middle-aged and elderly age | Prevalence | Male | Lesotho | MS | 0.28<br>(0.24 to 0.32) | < 0.001 |
| Middle-aged and elderly age | Prevalence | Both | Lesotho | Psoriasis | -0.61<br>(-0.68 to -0.54) | < 0.001 |
| Middle-aged and elderly age | Prevalence | Female | Lesotho | Psoriasis | -0.66<br>(-0.73 to -0.58) | < 0.001 |
| Middle-aged and elderly age | Prevalence | Male | Lesotho | Psoriasis | -0.57<br>(-0.65 to -0.49) | < 0.001 |
| Middle-aged and elderly age | Prevalence | Both | Lesotho | RHD | 0.04<br>(0.03 to 0.05) | < 0.001 |

| | | | | | | |
|---|---|---|---|---|---|---|
| Middle-aged and elderly age | Prevalence | Female | Lesotho | RHD | 0.03<br>(0 to 0.05) | 0.020 |
| Middle-aged and elderly age | Prevalence | Male | Lesotho | RHD | 0.01<br>(-0.01 to 0.03) | 0.446 |
| Middle-aged and elderly age | Prevalence | Both | Lesotho | RA | 0.35<br>(0.25 to 0.45) | < 0.001 |
| Middle-aged and elderly age | Prevalence | Female | Lesotho | RA | 0.34<br>(0.26 to 0.43) | < 0.001 |
| Middle-aged and elderly age | Prevalence | Male | Lesotho | RA | 0.24<br>(0.14 to 0.34) | < 0.001 |
| Middle-aged and elderly age | Prevalence | Both | Liberia | AA | 0.01<br>(-0.01 to 0.03) | 0.448 |
| Middle-aged and elderly age | Prevalence | Female | Liberia | AA | 0<br>(0 to 0) | 0.027 |
| Middle-aged and elderly age | Prevalence | Male | Liberia | AA | 0<br>(0 to 0) | < 0.001 |
| Middle-aged and elderly age | Prevalence | Both | Liberia | T1D | 0.46<br>(0.44 to 0.48) | < 0.001 |
| Middle-aged and elderly age | Prevalence | Female | Liberia | T1D | 0.46<br>(0.44 to 0.49) | < 0.001 |
| Middle-aged and elderly age | Prevalence | Male | Liberia | T1D | 0.46<br>(0.44 to 0.48) | < 0.001 |
| Middle-aged and elderly age | Prevalence | Both | Liberia | IBD | 0.1<br>(0.01 to 0.19) | 0.025 |
| Middle-aged and elderly age | Prevalence | Female | Liberia | IBD | -0.04<br>(-0.13 to 0.04) | 0.348 |
| Middle-aged and elderly age | Prevalence | Male | Liberia | IBD | 0.3<br>(0.21 to 0.4) | < 0.001 |
| Middle-aged and elderly age | Prevalence | Both | Liberia | MS | 0.29<br>(0.23 to 0.34) | < 0.001 |
| Middle-aged and elderly age | Prevalence | Female | Liberia | MS | 0.26<br>(0.24 to 0.28) | < 0.001 |
| Middle-aged and elderly age | Prevalence | Male | Liberia | MS | 0.31 | < 0.001 |

| | | | | | | |
|---|---|---|---|---|---|---|
| | | | | | (0.25 to 0.38) | |
| Middle-aged and elderly age | Prevalence | Both | Liberia | Psoriasis | -0.5<br>(-0.62 to -0.38) | < 0.001 |
| Middle-aged and elderly age | Prevalence | Female | Liberia | Psoriasis | -0.57<br>(-0.69 to -0.44) | < 0.001 |
| Middle-aged and elderly age | Prevalence | Male | Liberia | Psoriasis | -0.48<br>(-0.6 to -0.36) | < 0.001 |
| Middle-aged and elderly age | Prevalence | Both | Liberia | RHD | 0.2<br>(0.18 to 0.22) | < 0.001 |
| Middle-aged and elderly age | Prevalence | Female | Liberia | RHD | 0.25<br>(0.23 to 0.26) | < 0.001 |
| Middle-aged and elderly age | Prevalence | Male | Liberia | RHD | 0.17<br>(0.14 to 0.19) | < 0.001 |
| Middle-aged and elderly age | Prevalence | Both | Liberia | RA | 0.72<br>(0.67 to 0.76) | < 0.001 |
| Middle-aged and elderly age | Prevalence | Female | Liberia | RA | 0.69<br>(0.66 to 0.72) | < 0.001 |
| Middle-aged and elderly age | Prevalence | Male | Liberia | RA | 0.76<br>(0.71 to 0.81) | < 0.001 |
| Middle-aged and elderly age | Prevalence | Both | Libya | AA | 0.11<br>(0.09 to 0.13) | < 0.001 |
| Middle-aged and elderly age | Prevalence | Female | Libya | AA | 0<br>(0 to 0) | < 0.001 |
| Middle-aged and elderly age | Prevalence | Male | Libya | AA | 0<br>(0 to 0) | 0.015 |
| Middle-aged and elderly age | Prevalence | Both | Libya | T1D | 2.96<br>(2.92 to 3) | < 0.001 |
| Middle-aged and elderly age | Prevalence | Female | Libya | T1D | 2.85<br>(2.82 to 2.89) | < 0.001 |
| Middle-aged and elderly age | Prevalence | Male | Libya | T1D | 3.07<br>(3 to 3.13) | < 0.001 |
| Middle-aged and elderly age | Prevalence | Both | Libya | IBD | 0.16<br>(-0.13 to 0.46) | 0.277 |

| | | | | | | |
|---|---|---|---|---|---|---|
| Middle-aged and elderly age | Prevalence | Female | Libya | IBD | 0.19<br>(-0.11 to 0.49) | 0.221 |
| Middle-aged and elderly age | Prevalence | Male | Libya | IBD | 0.2<br>(-0.06 to 0.47) | 0.133 |
| Middle-aged and elderly age | Prevalence | Both | Libya | MS | 0.96<br>(0.9 to 1.01) | < 0.001 |
| Middle-aged and elderly age | Prevalence | Female | Libya | MS | 0.79<br>(0.74 to 0.85) | < 0.001 |
| Middle-aged and elderly age | Prevalence | Male | Libya | MS | 1.02<br>(1.01 to 1.04) | < 0.001 |
| Middle-aged and elderly age | Prevalence | Both | Libya | Psoriasis | -0.99<br>(-1.03 to -0.96) | < 0.001 |
| Middle-aged and elderly age | Prevalence | Female | Libya | Psoriasis | -1.01<br>(-1.04 to -0.97) | < 0.001 |
| Middle-aged and elderly age | Prevalence | Male | Libya | Psoriasis | -1<br>(-1.05 to -0.95) | < 0.001 |
| Middle-aged and elderly age | Prevalence | Both | Libya | RHD | 0.35<br>(0.33 to 0.36) | < 0.001 |
| Middle-aged and elderly age | Prevalence | Female | Libya | RHD | 0.32<br>(0.31 to 0.33) | < 0.001 |
| Middle-aged and elderly age | Prevalence | Male | Libya | RHD | 0.3<br>(0.25 to 0.34) | < 0.001 |
| Middle-aged and elderly age | Prevalence | Both | Libya | RA | 0.9<br>(0.8 to 1) | < 0.001 |
| Middle-aged and elderly age | Prevalence | Female | Libya | RA | 0.77<br>(0.63 to 0.9) | < 0.001 |
| Middle-aged and elderly age | Prevalence | Male | Libya | RA | 0.66<br>(0.56 to 0.75) | < 0.001 |
| Middle-aged and elderly age | Prevalence | Both | Lithuania | AA | -0.03<br>(-0.03 to -0.03) | < 0.001 |
| Middle-aged and elderly age | Prevalence | Female | Lithuania | AA | 0<br>(0 to 0) | < 0.001 |
| Middle-aged and elderly age | Prevalence | Male | Lithuania | AA | 0 | < 0.001 |

| | | | | | | |
|---|---|---|---|---|---|---|
| | | | | | (0 to 0) | |
| Middle-aged and elderly age | Prevalence | Both | Lithuania | T1D | 1.95 (1.92 to 1.98) | < 0.001 |
| Middle-aged and elderly age | Prevalence | Female | Lithuania | T1D | 1.97 (1.93 to 2) | < 0.001 |
| Middle-aged and elderly age | Prevalence | Male | Lithuania | T1D | 1.93 (1.84 to 2.01) | < 0.001 |
| Middle-aged and elderly age | Prevalence | Both | Lithuania | IBD | 0.37 (0.29 to 0.45) | < 0.001 |
| Middle-aged and elderly age | Prevalence | Female | Lithuania | IBD | 0.52 (0.44 to 0.6) | < 0.001 |
| Middle-aged and elderly age | Prevalence | Male | Lithuania | IBD | 0.2 (0.12 to 0.28) | < 0.001 |
| Middle-aged and elderly age | Prevalence | Both | Lithuania | MS | 0.02 (-0.05 to 0.08) | 0.567 |
| Middle-aged and elderly age | Prevalence | Female | Lithuania | MS | 0.15 (0.09 to 0.22) | < 0.001 |
| Middle-aged and elderly age | Prevalence | Male | Lithuania | MS | -0.17 (-0.2 to -0.14) | < 0.001 |
| Middle-aged and elderly age | Prevalence | Both | Lithuania | Psoriasis | -0.86 (-0.87 to -0.85) | < 0.001 |
| Middle-aged and elderly age | Prevalence | Female | Lithuania | Psoriasis | -0.85 (-0.86 to -0.84) | < 0.001 |
| Middle-aged and elderly age | Prevalence | Male | Lithuania | Psoriasis | -0.86 (-0.88 to -0.85) | < 0.001 |
| Middle-aged and elderly age | Prevalence | Both | Lithuania | RHD | -2.75 (-2.96 to -2.53) | < 0.001 |
| Middle-aged and elderly age | Prevalence | Female | Lithuania | RHD | -2.91 (-3.05 to -2.77) | < 0.001 |
| Middle-aged and elderly age | Prevalence | Male | Lithuania | RHD | -2.55 (-2.68 to -2.43) | < 0.001 |
| Middle-aged and elderly age | Prevalence | Both | Lithuania | RA | 0.38 (0.21 to 0.56) | < 0.001 |

| | | | | | | |
|---|---|---|---|---|---|---|
| Middle-aged and elderly age | Prevalence | Female | Lithuania | RA | 0.44 (0.26 to 0.62) | < 0.001 |
| Middle-aged and elderly age | Prevalence | Male | Lithuania | RA | 0.5 (0.32 to 0.68) | < 0.001 |
| Middle-aged and elderly age | Prevalence | Both | Luxembourg | AA | -0.06 (-0.08 to -0.04) | < 0.001 |
| Middle-aged and elderly age | Prevalence | Female | Luxembourg | AA | 0 (0 to 0) | < 0.001 |
| Middle-aged and elderly age | Prevalence | Male | Luxembourg | AA | 0 (0 to 0) | < 0.001 |
| Middle-aged and elderly age | Prevalence | Both | Luxembourg | T1D | 2.13 (2.05 to 2.22) | < 0.001 |
| Middle-aged and elderly age | Prevalence | Female | Luxembourg | T1D | 2.09 (1.99 to 2.19) | < 0.001 |
| Middle-aged and elderly age | Prevalence | Male | Luxembourg | T1D | 2.15 (2.07 to 2.24) | < 0.001 |
| Middle-aged and elderly age | Prevalence | Both | Luxembourg | IBD | 0.44 (0.4 to 0.48) | < 0.001 |
| Middle-aged and elderly age | Prevalence | Female | Luxembourg | IBD | 0.49 (0.43 to 0.55) | < 0.001 |
| Middle-aged and elderly age | Prevalence | Male | Luxembourg | IBD | 0.39 (0.35 to 0.43) | < 0.001 |
| Middle-aged and elderly age | Prevalence | Both | Luxembourg | MS | 0.27 (0.19 to 0.35) | < 0.001 |
| Middle-aged and elderly age | Prevalence | Female | Luxembourg | MS | 0.44 (0.37 to 0.5) | < 0.001 |
| Middle-aged and elderly age | Prevalence | Male | Luxembourg | MS | 0.11 (0.08 to 0.13) | < 0.001 |
| Middle-aged and elderly age | Prevalence | Both | Luxembourg | Psoriasis | -0.28 (-0.29 to -0.26) | < 0.001 |
| Middle-aged and elderly age | Prevalence | Female | Luxembourg | Psoriasis | -0.29 (-0.29 to -0.28) | < 0.001 |
| Middle-aged and elderly age | Prevalence | Male | Luxembourg | Psoriasis | -0.28 | < 0.001 |

| | | | | | | |
|---|---|---|---|---|---|---|
| | | | | | (-0.29 to -0.28) | |
| Middle-aged and elderly age | Prevalence | Both | Luxembourg | RHD | -0.63<br>(-0.83 to -0.43) | < 0.001 |
| Middle-aged and elderly age | Prevalence | Female | Luxembourg | RHD | -0.75<br>(-0.95 to -0.54) | < 0.001 |
| Middle-aged and elderly age | Prevalence | Male | Luxembourg | RHD | -0.61<br>(-0.75 to -0.48) | < 0.001 |
| Middle-aged and elderly age | Prevalence | Both | Luxembourg | RA | 0.44<br>(0.37 to 0.51) | < 0.001 |
| Middle-aged and elderly age | Prevalence | Female | Luxembourg | RA | 0.51<br>(0.45 to 0.56) | < 0.001 |
| Middle-aged and elderly age | Prevalence | Male | Luxembourg | RA | 0.52<br>(0.47 to 0.57) | < 0.001 |
| Middle-aged and elderly age | Prevalence | Both | Madagascar | AA | -0.02<br>(-0.02 to -0.01) | < 0.001 |
| Middle-aged and elderly age | Prevalence | Female | Madagascar | AA | 0<br>(0 to 0) | 1.000 |
| Middle-aged and elderly age | Prevalence | Male | Madagascar | AA | 0<br>(0 to 0) | < 0.001 |
| Middle-aged and elderly age | Prevalence | Both | Madagascar | T1D | 0.42<br>(0.41 to 0.43) | < 0.001 |
| Middle-aged and elderly age | Prevalence | Female | Madagascar | T1D | 0.43<br>(0.41 to 0.44) | < 0.001 |
| Middle-aged and elderly age | Prevalence | Male | Madagascar | T1D | 0.41<br>(0.41 to 0.41) | < 0.001 |
| Middle-aged and elderly age | Prevalence | Both | Madagascar | IBD | 0.22<br>(0.11 to 0.33) | < 0.001 |
| Middle-aged and elderly age | Prevalence | Female | Madagascar | IBD | 0.06<br>(-0.04 to 0.17) | 0.230 |
| Middle-aged and elderly age | Prevalence | Male | Madagascar | IBD | 0.38<br>(0.26 to 0.49) | < 0.001 |
| Middle-aged and elderly age | Prevalence | Both | Madagascar | MS | 0.16<br>(0.11 to 0.21) | < 0.001 |

| | | | | | | |
|---|---|---|---|---|---|---|
| Middle-aged and elderly age | Prevalence | Female | Madagascar | MS | 0.17 (0.14 to 0.21) | < 0.001 |
| Middle-aged and elderly age | Prevalence | Male | Madagascar | MS | 0.19 (0.12 to 0.26) | < 0.001 |
| Middle-aged and elderly age | Prevalence | Both | Madagascar | Psoriasis | -0.25 (-0.27 to -0.22) | < 0.001 |
| Middle-aged and elderly age | Prevalence | Female | Madagascar | Psoriasis | -0.26 (-0.29 to -0.22) | < 0.001 |
| Middle-aged and elderly age | Prevalence | Male | Madagascar | Psoriasis | -0.23 (-0.27 to -0.19) | < 0.001 |
| Middle-aged and elderly age | Prevalence | Both | Madagascar | RHD | 0.15 (0.14 to 0.17) | < 0.001 |
| Middle-aged and elderly age | Prevalence | Female | Madagascar | RHD | 0.18 (0.14 to 0.22) | < 0.001 |
| Middle-aged and elderly age | Prevalence | Male | Madagascar | RHD | 0.12 (0.1 to 0.15) | < 0.001 |
| Middle-aged and elderly age | Prevalence | Both | Madagascar | RA | 0.47 (0.41 to 0.54) | < 0.001 |
| Middle-aged and elderly age | Prevalence | Female | Madagascar | RA | 0.46 (0.42 to 0.5) | < 0.001 |
| Middle-aged and elderly age | Prevalence | Male | Madagascar | RA | 0.46 (0.39 to 0.53) | < 0.001 |
| Middle-aged and elderly age | Prevalence | Both | Malawi | AA | 0.01 (0 to 0.01) | 0.207 |
| Middle-aged and elderly age | Prevalence | Female | Malawi | AA | 0 (0 to 0) | 1.000 |
| Middle-aged and elderly age | Prevalence | Male | Malawi | AA | 0 (0 to 0) | < 0.001 |
| Middle-aged and elderly age | Prevalence | Both | Malawi | T1D | 0.43 (0.42 to 0.44) | < 0.001 |
| Middle-aged and elderly age | Prevalence | Female | Malawi | T1D | 0.43 (0.42 to 0.45) | < 0.001 |
| Middle-aged and elderly age | Prevalence | Male | Malawi | T1D | 0.43 | < 0.001 |

| | | | | | | |
|---|---|---|---|---|---|---|
| | | | | | (0.42 to 0.45) | |
| Middle-aged and elderly age | Prevalence | Both | Malawi | IBD | 0.28 (0.19 to 0.37) | < 0.001 |
| Middle-aged and elderly age | Prevalence | Female | Malawi | IBD | 0.24 (0.11 to 0.36) | < 0.001 |
| Middle-aged and elderly age | Prevalence | Male | Malawi | IBD | 0.33 (0.18 to 0.49) | < 0.001 |
| Middle-aged and elderly age | Prevalence | Both | Malawi | MS | 0.22 (0.18 to 0.27) | < 0.001 |
| Middle-aged and elderly age | Prevalence | Female | Malawi | MS | 0.2 (0.17 to 0.22) | < 0.001 |
| Middle-aged and elderly age | Prevalence | Male | Malawi | MS | 0.28 (0.24 to 0.32) | < 0.001 |
| Middle-aged and elderly age | Prevalence | Both | Malawi | Psoriasis | -0.39 (-0.47 to -0.31) | < 0.001 |
| Middle-aged and elderly age | Prevalence | Female | Malawi | Psoriasis | -0.39 (-0.47 to -0.32) | < 0.001 |
| Middle-aged and elderly age | Prevalence | Male | Malawi | Psoriasis | -0.35 (-0.41 to -0.3) | < 0.001 |
| Middle-aged and elderly age | Prevalence | Both | Malawi | RHD | 0.12 (0.11 to 0.13) | < 0.001 |
| Middle-aged and elderly age | Prevalence | Female | Malawi | RHD | 0.14 (0.12 to 0.17) | < 0.001 |
| Middle-aged and elderly age | Prevalence | Male | Malawi | RHD | 0.08 (0.02 to 0.15) | 0.015 |
| Middle-aged and elderly age | Prevalence | Both | Malawi | RA | 0.62 (0.56 to 0.68) | < 0.001 |
| Middle-aged and elderly age | Prevalence | Female | Malawi | RA | 0.51 (0.44 to 0.58) | < 0.001 |
| Middle-aged and elderly age | Prevalence | Male | Malawi | RA | 0.67 (0.62 to 0.72) | < 0.001 |
| Middle-aged and elderly age | Prevalence | Both | Malaysia | AA | -0.01 (-0.02 to 0) | 0.002 |

| | | | | | | |
|---|---|---|---|---|---|---|
| Middle-aged and elderly age | Prevalence | Female | Malaysia | AA | 0<br>(0 to 0) | < 0.001 |
| Middle-aged and elderly age | Prevalence | Male | Malaysia | AA | 0<br>(0 to 0) | < 0.001 |
| Middle-aged and elderly age | Prevalence | Both | Malaysia | T1D | 0.96<br>(0.87 to 1.04) | < 0.001 |
| Middle-aged and elderly age | Prevalence | Female | Malaysia | T1D | 0.89<br>(0.8 to 0.99) | < 0.001 |
| Middle-aged and elderly age | Prevalence | Male | Malaysia | T1D | 1.01<br>(0.9 to 1.12) | < 0.001 |
| Middle-aged and elderly age | Prevalence | Both | Malaysia | IBD | 1.31<br>(1.26 to 1.36) | < 0.001 |
| Middle-aged and elderly age | Prevalence | Female | Malaysia | IBD | 1.2<br>(1.14 to 1.26) | < 0.001 |
| Middle-aged and elderly age | Prevalence | Male | Malaysia | IBD | 1.46<br>(1.41 to 1.5) | < 0.001 |
| Middle-aged and elderly age | Prevalence | Both | Malaysia | MS | 0.24<br>(0.2 to 0.29) | < 0.001 |
| Middle-aged and elderly age | Prevalence | Female | Malaysia | MS | 0.28<br>(0.15 to 0.4) | < 0.001 |
| Middle-aged and elderly age | Prevalence | Male | Malaysia | MS | 0.28<br>(0.23 to 0.33) | < 0.001 |
| Middle-aged and elderly age | Prevalence | Both | Malaysia | Psoriasis | -0.61<br>(-0.64 to -0.57) | < 0.001 |
| Middle-aged and elderly age | Prevalence | Female | Malaysia | Psoriasis | -0.61<br>(-0.65 to -0.57) | < 0.001 |
| Middle-aged and elderly age | Prevalence | Male | Malaysia | Psoriasis | -0.65<br>(-0.68 to -0.61) | < 0.001 |
| Middle-aged and elderly age | Prevalence | Both | Malaysia | RHD | 0.13<br>(0.08 to 0.18) | < 0.001 |
| Middle-aged and elderly age | Prevalence | Female | Malaysia | RHD | 0.15<br>(0.14 to 0.17) | < 0.001 |
| Middle-aged and elderly age | Prevalence | Male | Malaysia | RHD | 0.14 | < 0.001 |

| | | | | | | |
|---|---|---|---|---|---|---|
| | | | | | (0.1 to 0.18) | |
| Middle-aged and elderly age | Prevalence | Both | Malaysia | RA | 0.65 (0.61 to 0.69) | < 0.001 |
| Middle-aged and elderly age | Prevalence | Female | Malaysia | RA | 0.7 (0.66 to 0.73) | < 0.001 |
| Middle-aged and elderly age | Prevalence | Male | Malaysia | RA | 0.59 (0.51 to 0.67) | < 0.001 |
| Middle-aged and elderly age | Prevalence | Both | Maldives | AA | -0.01 (-0.03 to 0.01) | 0.317 |
| Middle-aged and elderly age | Prevalence | Female | Maldives | AA | 0 (0 to 0) | < 0.001 |
| Middle-aged and elderly age | Prevalence | Male | Maldives | AA | 0 (0 to 0) | < 0.001 |
| Middle-aged and elderly age | Prevalence | Both | Maldives | T1D | 0.9 (0.88 to 0.93) | < 0.001 |
| Middle-aged and elderly age | Prevalence | Female | Maldives | T1D | 0.85 (0.82 to 0.87) | < 0.001 |
| Middle-aged and elderly age | Prevalence | Male | Maldives | T1D | 0.94 (0.91 to 0.98) | < 0.001 |
| Middle-aged and elderly age | Prevalence | Both | Maldives | IBD | 0.5 (0.47 to 0.52) | < 0.001 |
| Middle-aged and elderly age | Prevalence | Female | Maldives | IBD | 0.27 (0.22 to 0.32) | < 0.001 |
| Middle-aged and elderly age | Prevalence | Male | Maldives | IBD | 0.68 (0.65 to 0.71) | < 0.001 |
| Middle-aged and elderly age | Prevalence | Both | Maldives | MS | 0.28 (0.2 to 0.36) | < 0.001 |
| Middle-aged and elderly age | Prevalence | Female | Maldives | MS | 0.27 (0.17 to 0.37) | < 0.001 |
| Middle-aged and elderly age | Prevalence | Male | Maldives | MS | 0.31 (0.21 to 0.41) | < 0.001 |
| Middle-aged and elderly age | Prevalence | Both | Maldives | Psoriasis | -0.85 (-0.86 to -0.83) | < 0.001 |

| | | | | | | |
|---|---|---|---|---|---|---|
| Middle-aged and elderly age | Prevalence | Female | Maldives | Psoriasis | -0.83 (-0.86 to -0.8) | < 0.001 |
| Middle-aged and elderly age | Prevalence | Male | Maldives | Psoriasis | -0.87 (-0.91 to -0.82) | < 0.001 |
| Middle-aged and elderly age | Prevalence | Both | Maldives | RHD | 0.07 (0.04 to 0.1) | < 0.001 |
| Middle-aged and elderly age | Prevalence | Female | Maldives | RHD | 0.1 (0.09 to 0.12) | < 0.001 |
| Middle-aged and elderly age | Prevalence | Male | Maldives | RHD | 0.06 (0.04 to 0.09) | < 0.001 |
| Middle-aged and elderly age | Prevalence | Both | Maldives | RA | 1.04 (0.77 to 1.31) | < 0.001 |
| Middle-aged and elderly age | Prevalence | Female | Maldives | RA | 1.05 (0.83 to 1.26) | < 0.001 |
| Middle-aged and elderly age | Prevalence | Male | Maldives | RA | 1.03 (0.98 to 1.07) | < 0.001 |
| Middle-aged and elderly age | Prevalence | Both | Mali | AA | -0.03 (-0.04 to -0.03) | < 0.001 |
| Middle-aged and elderly age | Prevalence | Female | Mali | AA | 0 (0 to 0) | 0.027 |
| Middle-aged and elderly age | Prevalence | Male | Mali | AA | 0 (0 to 0) | < 0.001 |
| Middle-aged and elderly age | Prevalence | Both | Mali | T1D | 0.15 (0.13 to 0.16) | < 0.001 |
| Middle-aged and elderly age | Prevalence | Female | Mali | T1D | 0.15 (0.13 to 0.17) | < 0.001 |
| Middle-aged and elderly age | Prevalence | Male | Mali | T1D | 0.14 (0.13 to 0.15) | < 0.001 |
| Middle-aged and elderly age | Prevalence | Both | Mali | IBD | 0.34 (0.24 to 0.44) | < 0.001 |
| Middle-aged and elderly age | Prevalence | Female | Mali | IBD | 0.31 (0.22 to 0.4) | < 0.001 |
| Middle-aged and elderly age | Prevalence | Male | Mali | IBD | 0.36 | < 0.001 |

| | | | | | | |
|---|---|---|---|---|---|---|
| | | | | | (0.24 to 0.49) | |
| Middle-aged and elderly age | Prevalence | Both | Mali | MS | 0.17<br>(0.12 to 0.22) | < 0.001 |
| Middle-aged and elderly age | Prevalence | Female | Mali | MS | 0.18<br>(0.15 to 0.21) | < 0.001 |
| Middle-aged and elderly age | Prevalence | Male | Mali | MS | 0.28<br>(0.23 to 0.33) | < 0.001 |
| Middle-aged and elderly age | Prevalence | Both | Mali | Psoriasis | -0.77<br>(-0.99 to -0.56) | < 0.001 |
| Middle-aged and elderly age | Prevalence | Female | Mali | Psoriasis | -0.77<br>(-0.92 to -0.62) | < 0.001 |
| Middle-aged and elderly age | Prevalence | Male | Mali | Psoriasis | -0.73<br>(-1.01 to -0.44) | < 0.001 |
| Middle-aged and elderly age | Prevalence | Both | Mali | RHD | 0.06<br>(0.04 to 0.09) | < 0.001 |
| Middle-aged and elderly age | Prevalence | Female | Mali | RHD | 0.09<br>(0.06 to 0.12) | < 0.001 |
| Middle-aged and elderly age | Prevalence | Male | Mali | RHD | 0.05<br>(0.02 to 0.08) | 0.001 |
| Middle-aged and elderly age | Prevalence | Both | Mali | RA | 0.65<br>(0.61 to 0.69) | < 0.001 |
| Middle-aged and elderly age | Prevalence | Female | Mali | RA | 0.64<br>(0.62 to 0.67) | < 0.001 |
| Middle-aged and elderly age | Prevalence | Male | Mali | RA | 0.69<br>(0.66 to 0.73) | < 0.001 |
| Middle-aged and elderly age | Prevalence | Both | Malta | AA | -0.11<br>(-0.12 to -0.09) | < 0.001 |
| Middle-aged and elderly age | Prevalence | Female | Malta | AA | 0<br>(0 to 0) | < 0.001 |
| Middle-aged and elderly age | Prevalence | Male | Malta | AA | 0<br>(0 to 0) | < 0.001 |
| Middle-aged and elderly age | Prevalence | Both | Malta | T1D | 1.65<br>(1.56 to 1.73) | < 0.001 |

| | | | | | | |
|---|---|---|---|---|---|---|
| Middle-aged and elderly age | Prevalence | Female | Malta | T1D | 1.62 (1.48 to 1.75) | < 0.001 |
| Middle-aged and elderly age | Prevalence | Male | Malta | T1D | 1.72 (1.68 to 1.77) | < 0.001 |
| Middle-aged and elderly age | Prevalence | Both | Malta | IBD | 0.16 (0.11 to 0.21) | < 0.001 |
| Middle-aged and elderly age | Prevalence | Female | Malta | IBD | 0.04 (-0.01 to 0.09) | 0.117 |
| Middle-aged and elderly age | Prevalence | Male | Malta | IBD | 0.36 (0.32 to 0.4) | < 0.001 |
| Middle-aged and elderly age | Prevalence | Both | Malta | MS | 0.98 (0.81 to 1.15) | < 0.001 |
| Middle-aged and elderly age | Prevalence | Female | Malta | MS | 1.07 (0.97 to 1.17) | < 0.001 |
| Middle-aged and elderly age | Prevalence | Male | Malta | MS | 0.98 (0.91 to 1.05) | < 0.001 |
| Middle-aged and elderly age | Prevalence | Both | Malta | Psoriasis | -0.52 (-0.53 to -0.51) | < 0.001 |
| Middle-aged and elderly age | Prevalence | Female | Malta | Psoriasis | -0.53 (-0.54 to -0.52) | < 0.001 |
| Middle-aged and elderly age | Prevalence | Male | Malta | Psoriasis | -0.53 (-0.54 to -0.53) | < 0.001 |
| Middle-aged and elderly age | Prevalence | Both | Malta | RHD | -1.06 (-1.23 to -0.89) | < 0.001 |
| Middle-aged and elderly age | Prevalence | Female | Malta | RHD | -1.07 (-1.13 to -1) | < 0.001 |
| Middle-aged and elderly age | Prevalence | Male | Malta | RHD | -0.86 (-1.23 to -0.49) | < 0.001 |
| Middle-aged and elderly age | Prevalence | Both | Malta | RA | 0.36 (0.31 to 0.4) | < 0.001 |
| Middle-aged and elderly age | Prevalence | Female | Malta | RA | 0.49 (0.39 to 0.59) | < 0.001 |
| Middle-aged and elderly age | Prevalence | Male | Malta | RA | 0.51 | < 0.001 |

| | | | | | | |
|---|---|---|---|---|---|---|
| | | | | | (0.48 to 0.54) | |
| Middle-aged and elderly age | Prevalence | Both | Marshall Islands | AA | 0.05<br>(0.03 to 0.07) | < 0.001 |
| Middle-aged and elderly age | Prevalence | Female | Marshall Islands | AA | 0<br>(0 to 0) | < 0.001 |
| Middle-aged and elderly age | Prevalence | Male | Marshall Islands | AA | 0<br>(0 to 0) | < 0.001 |
| Middle-aged and elderly age | Prevalence | Both | Marshall Islands | T1D | 0.88<br>(0.86 to 0.89) | < 0.001 |
| Middle-aged and elderly age | Prevalence | Female | Marshall Islands | T1D | 0.85<br>(0.85 to 0.86) | < 0.001 |
| Middle-aged and elderly age | Prevalence | Male | Marshall Islands | T1D | 0.91<br>(0.88 to 0.95) | < 0.001 |
| Middle-aged and elderly age | Prevalence | Both | Marshall Islands | IBD | -0.28<br>(-0.38 to -0.18) | < 0.001 |
| Middle-aged and elderly age | Prevalence | Female | Marshall Islands | IBD | -0.42<br>(-0.53 to -0.32) | < 0.001 |
| Middle-aged and elderly age | Prevalence | Male | Marshall Islands | IBD | -0.19<br>(-0.27 to -0.11) | < 0.001 |
| Middle-aged and elderly age | Prevalence | Both | Marshall Islands | MS | 0.17<br>(0.12 to 0.21) | < 0.001 |
| Middle-aged and elderly age | Prevalence | Female | Marshall Islands | MS | 0.12<br>(0.08 to 0.15) | < 0.001 |
| Middle-aged and elderly age | Prevalence | Male | Marshall Islands | MS | 0.13<br>(0.1 to 0.16) | < 0.001 |
| Middle-aged and elderly age | Prevalence | Both | Marshall Islands | Psoriasis | -0.63<br>(-0.66 to -0.6) | < 0.001 |
| Middle-aged and elderly age | Prevalence | Female | Marshall Islands | Psoriasis | -0.58<br>(-0.59 to -0.56) | < 0.001 |
| Middle-aged and elderly age | Prevalence | Male | Marshall Islands | Psoriasis | -0.61<br>(-0.64 to -0.58) | < 0.001 |
| Middle-aged and elderly age | Prevalence | Both | Marshall Islands | RHD | 0.08<br>(0.06 to 0.09) | < 0.001 |

| | | | | | | |
|---|---|---|---|---|---|---|
| Middle-aged and elderly age | Prevalence | Female | Marshall Islands | RHD | 0.09 (0.08 to 0.1) | < 0.001 |
| Middle-aged and elderly age | Prevalence | Male | Marshall Islands | RHD | 0.02 (-0.01 to 0.06) | 0.215 |
| Middle-aged and elderly age | Prevalence | Both | Marshall Islands | RA | 0.36 (0.33 to 0.39) | < 0.001 |
| Middle-aged and elderly age | Prevalence | Female | Marshall Islands | RA | 0.28 (0.26 to 0.3) | < 0.001 |
| Middle-aged and elderly age | Prevalence | Male | Marshall Islands | RA | 0.3 (0.21 to 0.4) | < 0.001 |
| Middle-aged and elderly age | Prevalence | Both | Mauritania | AA | 0.01 (0 to 0.02) | 0.008 |
| Middle-aged and elderly age | Prevalence | Female | Mauritania | AA | 0 (0 to 0) | 0.027 |
| Middle-aged and elderly age | Prevalence | Male | Mauritania | AA | 0 (0 to 0) | < 0.001 |
| Middle-aged and elderly age | Prevalence | Both | Mauritania | T1D | 0.5 (0.49 to 0.51) | < 0.001 |
| Middle-aged and elderly age | Prevalence | Female | Mauritania | T1D | 0.5 (0.5 to 0.5) | < 0.001 |
| Middle-aged and elderly age | Prevalence | Male | Mauritania | T1D | 0.49 (0.48 to 0.51) | < 0.001 |
| Middle-aged and elderly age | Prevalence | Both | Mauritania | IBD | 0.81 (0.69 to 0.93) | < 0.001 |
| Middle-aged and elderly age | Prevalence | Female | Mauritania | IBD | 0.64 (0.52 to 0.76) | < 0.001 |
| Middle-aged and elderly age | Prevalence | Male | Mauritania | IBD | 1 (0.87 to 1.12) | < 0.001 |
| Middle-aged and elderly age | Prevalence | Both | Mauritania | MS | 0.24 (0.21 to 0.27) | < 0.001 |
| Middle-aged and elderly age | Prevalence | Female | Mauritania | MS | 0.24 (0.22 to 0.25) | < 0.001 |
| Middle-aged and elderly age | Prevalence | Male | Mauritania | MS | 0.22 | < 0.001 |

| | | | | | | |
|---|---|---|---|---|---|---|
| | | | | | (0.2 to 0.24) | |
| Middle-aged and elderly age | Prevalence | Both | Mauritania | Psoriasis | -0.69 (-0.82 to -0.57) | < 0.001 |
| Middle-aged and elderly age | Prevalence | Female | Mauritania | Psoriasis | -0.73 (-0.81 to -0.64) | < 0.001 |
| Middle-aged and elderly age | Prevalence | Male | Mauritania | Psoriasis | -0.61 (-0.75 to -0.46) | < 0.001 |
| Middle-aged and elderly age | Prevalence | Both | Mauritania | RHD | 0.03 (0.02 to 0.04) | < 0.001 |
| Middle-aged and elderly age | Prevalence | Female | Mauritania | RHD | 0.06 (0.02 to 0.09) | 0.001 |
| Middle-aged and elderly age | Prevalence | Male | Mauritania | RHD | -0.01 (-0.03 to 0.01) | 0.433 |
| Middle-aged and elderly age | Prevalence | Both | Mauritania | RA | 0.84 (0.82 to 0.86) | < 0.001 |
| Middle-aged and elderly age | Prevalence | Female | Mauritania | RA | 0.81 (0.78 to 0.84) | < 0.001 |
| Middle-aged and elderly age | Prevalence | Male | Mauritania | RA | 0.86 (0.81 to 0.9) | < 0.001 |
| Middle-aged and elderly age | Prevalence | Both | Mauritius | AA | -0.01 (-0.02 to -0.01) | < 0.001 |
| Middle-aged and elderly age | Prevalence | Female | Mauritius | AA | 0 (0 to 0) | < 0.001 |
| Middle-aged and elderly age | Prevalence | Male | Mauritius | AA | 0 (0 to 0) | < 0.001 |
| Middle-aged and elderly age | Prevalence | Both | Mauritius | T1D | 1.3 (1.19 to 1.4) | < 0.001 |
| Middle-aged and elderly age | Prevalence | Female | Mauritius | T1D | 1.19 (1.1 to 1.29) | < 0.001 |
| Middle-aged and elderly age | Prevalence | Male | Mauritius | T1D | 1.4 (1.28 to 1.52) | < 0.001 |
| Middle-aged and elderly age | Prevalence | Both | Mauritius | IBD | 0.67 (0.65 to 0.7) | < 0.001 |

| | | | | | | |
|---|---|---|---|---|---|---|
| Middle-aged and elderly age | Prevalence | Female | Mauritius | IBD | 0.71 (0.68 to 0.74) | < 0.001 |
| Middle-aged and elderly age | Prevalence | Male | Mauritius | IBD | 0.63 (0.58 to 0.68) | < 0.001 |
| Middle-aged and elderly age | Prevalence | Both | Mauritius | MS | 0.18 (0.17 to 0.2) | < 0.001 |
| Middle-aged and elderly age | Prevalence | Female | Mauritius | MS | 0.2 (0.15 to 0.24) | < 0.001 |
| Middle-aged and elderly age | Prevalence | Male | Mauritius | MS | 0.22 (0.2 to 0.24) | < 0.001 |
| Middle-aged and elderly age | Prevalence | Both | Mauritius | Psoriasis | -0.54 (-0.56 to -0.52) | < 0.001 |
| Middle-aged and elderly age | Prevalence | Female | Mauritius | Psoriasis | -0.53 (-0.53 to -0.52) | < 0.001 |
| Middle-aged and elderly age | Prevalence | Male | Mauritius | Psoriasis | -0.56 (-0.6 to -0.52) | < 0.001 |
| Middle-aged and elderly age | Prevalence | Both | Mauritius | RHD | 0.07 (0.05 to 0.08) | < 0.001 |
| Middle-aged and elderly age | Prevalence | Female | Mauritius | RHD | 0.09 (0.06 to 0.11) | < 0.001 |
| Middle-aged and elderly age | Prevalence | Male | Mauritius | RHD | 0.05 (0.02 to 0.09) | 0.003 |
| Middle-aged and elderly age | Prevalence | Both | Mauritius | RA | 0.56 (0.51 to 0.61) | < 0.001 |
| Middle-aged and elderly age | Prevalence | Female | Mauritius | RA | 0.65 (0.59 to 0.72) | < 0.001 |
| Middle-aged and elderly age | Prevalence | Male | Mauritius | RA | 0.43 (0.42 to 0.45) | < 0.001 |
| Middle-aged and elderly age | Prevalence | Both | Mexico | AA | 0.03 (0.02 to 0.04) | < 0.001 |
| Middle-aged and elderly age | Prevalence | Female | Mexico | AA | 0 (0 to 0) | < 0.001 |
| Middle-aged and elderly age | Prevalence | Male | Mexico | AA | 0 | < 0.001 |

| | | | | | | |
|---|---|---|---|---|---|---|
| | | | | | (0 to 0) | |
| Middle-aged and elderly age | Prevalence | Both | Mexico | T1D | 0.82 (0.8 to 0.85) | < 0.001 |
| Middle-aged and elderly age | Prevalence | Female | Mexico | T1D | 0.77 (0.73 to 0.82) | < 0.001 |
| Middle-aged and elderly age | Prevalence | Male | Mexico | T1D | 0.86 (0.83 to 0.89) | < 0.001 |
| Middle-aged and elderly age | Prevalence | Both | Mexico | IBD | -0.88 (-0.99 to -0.76) | < 0.001 |
| Middle-aged and elderly age | Prevalence | Female | Mexico | IBD | -0.57 (-0.61 to -0.54) | < 0.001 |
| Middle-aged and elderly age | Prevalence | Male | Mexico | IBD | -1.13 (-1.26 to -1) | < 0.001 |
| Middle-aged and elderly age | Prevalence | Both | Mexico | MS | 1.23 (1.21 to 1.26) | < 0.001 |
| Middle-aged and elderly age | Prevalence | Female | Mexico | MS | 1.19 (1.16 to 1.22) | < 0.001 |
| Middle-aged and elderly age | Prevalence | Male | Mexico | MS | 1.24 (1.21 to 1.26) | < 0.001 |
| Middle-aged and elderly age | Prevalence | Both | Mexico | Psoriasis | -0.45 (-0.47 to -0.43) | < 0.001 |
| Middle-aged and elderly age | Prevalence | Female | Mexico | Psoriasis | -0.47 (-0.48 to -0.46) | < 0.001 |
| Middle-aged and elderly age | Prevalence | Male | Mexico | Psoriasis | -0.45 (-0.47 to -0.42) | < 0.001 |
| Middle-aged and elderly age | Prevalence | Both | Mexico | RHD | -0.7 (-0.72 to -0.67) | < 0.001 |
| Middle-aged and elderly age | Prevalence | Female | Mexico | RHD | -0.76 (-0.78 to -0.74) | < 0.001 |
| Middle-aged and elderly age | Prevalence | Male | Mexico | RHD | -0.65 (-0.69 to -0.61) | < 0.001 |
| Middle-aged and elderly age | Prevalence | Both | Mexico | RA | -0.52 (-0.68 to -0.36) | < 0.001 |

| | | | | | | |
|---|---|---|---|---|---|---|
| Middle-aged and elderly age | Prevalence | Female | Mexico | RA | -0.83 (-1.03 to -0.64) | < 0.001 |
| Middle-aged and elderly age | Prevalence | Male | Mexico | RA | 0.39 (0.34 to 0.45) | < 0.001 |
| Middle-aged and elderly age | Prevalence | Both | Micronesia (Federated States of) | AA | 0 (0 to 0.01) | 0.183 |
| Middle-aged and elderly age | Prevalence | Female | Micronesia (Federated States of) | AA | 0 (0 to 0) | < 0.001 |
| Middle-aged and elderly age | Prevalence | Male | Micronesia (Federated States of) | AA | 0 (0 to 0) | < 0.001 |
| Middle-aged and elderly age | Prevalence | Both | Micronesia (Federated States of) | T1D | 0.71 (0.69 to 0.74) | < 0.001 |
| Middle-aged and elderly age | Prevalence | Female | Micronesia (Federated States of) | T1D | 0.7 (0.66 to 0.73) | < 0.001 |
| Middle-aged and elderly age | Prevalence | Male | Micronesia (Federated States of) | T1D | 0.74 (0.71 to 0.77) | < 0.001 |
| Middle-aged and elderly age | Prevalence | Both | Micronesia (Federated States of) | IBD | -0.67 (-0.74 to -0.61) | < 0.001 |
| Middle-aged and elderly age | Prevalence | Female | Micronesia (Federated States of) | IBD | -1.1 (-1.17 to -1.02) | < 0.001 |
| Middle-aged and elderly age | Prevalence | Male | Micronesia (Federated States of) | IBD | -0.2 (-0.27 to -0.13) | < 0.001 |
| Middle-aged and elderly age | Prevalence | Both | Micronesia (Federated States of) | MS | 0.25 (0.19 to 0.3) | < 0.001 |
| Middle-aged and elderly age | Prevalence | Female | Micronesia (Federated States of) | MS | 0.23 (0.19 to 0.27) | < 0.001 |
| Middle-aged and elderly age | Prevalence | Male | Micronesia (Federated States of) | MS | 0.28 (0.25 to 0.32) | < 0.001 |
| Middle-aged and elderly age | Prevalence | Both | Micronesia (Federated States of) | Psoriasis | -0.51 (-0.52 to -0.49) | < 0.001 |
| Middle-aged and elderly age | Prevalence | Female | Micronesia (Federated States of) | Psoriasis | -0.49 (-0.5 to -0.48) | < 0.001 |
| Middle-aged and elderly age | Prevalence | Male | Micronesia (Federated States of) | Psoriasis | -0.52 | < 0.001 |

| | | | | | | |
|---|---|---|---|---|---|---|
| | | | | | (-0.55 to -0.49) | |
| Middle-aged and elderly age | Prevalence | Both | Micronesia (Federated States of) | RHD | 0.06<br>(0.05 to 0.08) | < 0.001 |
| Middle-aged and elderly age | Prevalence | Female | Micronesia (Federated States of) | RHD | 0.09<br>(0.06 to 0.12) | < 0.001 |
| Middle-aged and elderly age | Prevalence | Male | Micronesia (Federated States of) | RHD | 0.03<br>(0.01 to 0.05) | 0.001 |
| Middle-aged and elderly age | Prevalence | Both | Micronesia (Federated States of) | RA | 0.53<br>(0.43 to 0.63) | < 0.001 |
| Middle-aged and elderly age | Prevalence | Female | Micronesia (Federated States of) | RA | 0.55<br>(0.5 to 0.6) | < 0.001 |
| Middle-aged and elderly age | Prevalence | Male | Micronesia (Federated States of) | RA | 0.51<br>(0.41 to 0.62) | < 0.001 |
| Middle-aged and elderly age | Prevalence | Both | Monaco | AA | -0.06<br>(-0.1 to -0.03) | < 0.001 |
| Middle-aged and elderly age | Prevalence | Female | Monaco | AA | 0<br>(0 to 0) | < 0.001 |
| Middle-aged and elderly age | Prevalence | Male | Monaco | AA | 0<br>(0 to 0) | < 0.001 |
| Middle-aged and elderly age | Prevalence | Both | Monaco | T1D | 1.69<br>(1.63 to 1.76) | < 0.001 |
| Middle-aged and elderly age | Prevalence | Female | Monaco | T1D | 1.68<br>(1.59 to 1.77) | < 0.001 |
| Middle-aged and elderly age | Prevalence | Male | Monaco | T1D | 1.68<br>(1.62 to 1.74) | < 0.001 |
| Middle-aged and elderly age | Prevalence | Both | Monaco | IBD | 0.29<br>(0.25 to 0.33) | < 0.001 |
| Middle-aged and elderly age | Prevalence | Female | Monaco | IBD | 0.34<br>(0.3 to 0.38) | < 0.001 |
| Middle-aged and elderly age | Prevalence | Male | Monaco | IBD | 0.24<br>(0.22 to 0.26) | < 0.001 |
| Middle-aged and elderly age | Prevalence | Both | Monaco | MS | 0.62<br>(0.55 to 0.69) | < 0.001 |

| | | | | | | |
|---|---|---|---|---|---|---|
| Middle-aged and elderly age | Prevalence | Female | Monaco | MS | 0.84 (0.81 to 0.87) | < 0.001 |
| Middle-aged and elderly age | Prevalence | Male | Monaco | MS | 0.33 (0.27 to 0.38) | < 0.001 |
| Middle-aged and elderly age | Prevalence | Both | Monaco | Psoriasis | -0.27 (-0.28 to -0.27) | < 0.001 |
| Middle-aged and elderly age | Prevalence | Female | Monaco | Psoriasis | -0.28 (-0.29 to -0.27) | < 0.001 |
| Middle-aged and elderly age | Prevalence | Male | Monaco | Psoriasis | -0.28 (-0.28 to -0.27) | < 0.001 |
| Middle-aged and elderly age | Prevalence | Both | Monaco | RHD | -0.33 (-0.37 to -0.29) | < 0.001 |
| Middle-aged and elderly age | Prevalence | Female | Monaco | RHD | -0.36 (-0.39 to -0.32) | < 0.001 |
| Middle-aged and elderly age | Prevalence | Male | Monaco | RHD | -0.26 (-0.31 to -0.2) | < 0.001 |
| Middle-aged and elderly age | Prevalence | Both | Monaco | RA | 0.35 (0.27 to 0.42) | < 0.001 |
| Middle-aged and elderly age | Prevalence | Female | Monaco | RA | 0.4 (0.34 to 0.46) | < 0.001 |
| Middle-aged and elderly age | Prevalence | Male | Monaco | RA | 0.38 (0.3 to 0.46) | < 0.001 |
| Middle-aged and elderly age | Prevalence | Both | Mongolia | AA | 0.07 (0.06 to 0.08) | < 0.001 |
| Middle-aged and elderly age | Prevalence | Female | Mongolia | AA | 0 (0 to 0) | < 0.001 |
| Middle-aged and elderly age | Prevalence | Male | Mongolia | AA | 0 (0 to 0) | < 0.001 |
| Middle-aged and elderly age | Prevalence | Both | Mongolia | T1D | 1.28 (1.26 to 1.3) | < 0.001 |
| Middle-aged and elderly age | Prevalence | Female | Mongolia | T1D | 1.28 (1.25 to 1.31) | < 0.001 |
| Middle-aged and elderly age | Prevalence | Male | Mongolia | T1D | 1.32 | < 0.001 |

| | | | | | | |
|---|---|---|---|---|---|---|
| | | | | | (1.3 to 1.34) | |
| Middle-aged and elderly age | Prevalence | Both | Mongolia | IBD | 0.06<br>(-0.01 to 0.14) | 0.102 |
| Middle-aged and elderly age | Prevalence | Female | Mongolia | IBD | 0.07<br>(0.05 to 0.1) | < 0.001 |
| Middle-aged and elderly age | Prevalence | Male | Mongolia | IBD | 0.11<br>(0.05 to 0.16) | < 0.001 |
| Middle-aged and elderly age | Prevalence | Both | Mongolia | MS | 0.2<br>(0.05 to 0.34) | 0.007 |
| Middle-aged and elderly age | Prevalence | Female | Mongolia | MS | 0.21<br>(-0.02 to 0.45) | 0.069 |
| Middle-aged and elderly age | Prevalence | Male | Mongolia | MS | 0.17<br>(0.01 to 0.33) | 0.039 |
| Middle-aged and elderly age | Prevalence | Both | Mongolia | Psoriasis | -0.89<br>(-0.92 to -0.86) | < 0.001 |
| Middle-aged and elderly age | Prevalence | Female | Mongolia | Psoriasis | -0.92<br>(-0.94 to -0.9) | < 0.001 |
| Middle-aged and elderly age | Prevalence | Male | Mongolia | Psoriasis | -0.87<br>(-0.91 to -0.83) | < 0.001 |
| Middle-aged and elderly age | Prevalence | Both | Mongolia | RHD | 0.07<br>(0.05 to 0.1) | < 0.001 |
| Middle-aged and elderly age | Prevalence | Female | Mongolia | RHD | 0.07<br>(0.04 to 0.1) | < 0.001 |
| Middle-aged and elderly age | Prevalence | Male | Mongolia | RHD | 0.02<br>(0.01 to 0.03) | < 0.001 |
| Middle-aged and elderly age | Prevalence | Both | Mongolia | RA | 1.08<br>(1.02 to 1.14) | < 0.001 |
| Middle-aged and elderly age | Prevalence | Female | Mongolia | RA | 1.14<br>(1.06 to 1.22) | < 0.001 |
| Middle-aged and elderly age | Prevalence | Male | Mongolia | RA | 0.74<br>(0.61 to 0.87) | < 0.001 |
| Middle-aged and elderly age | Prevalence | Both | Montenegro | AA | -0.02<br>(-0.03 to -0.02) | < 0.001 |

| | | | | | | |
|---|---|---|---|---|---|---|
| Middle-aged and elderly age | Prevalence | Female | Montenegro | AA | 0<br>(0 to 0) | < 0.001 |
| Middle-aged and elderly age | Prevalence | Male | Montenegro | AA | 0<br>(0 to 0) | < 0.001 |
| Middle-aged and elderly age | Prevalence | Both | Montenegro | T1D | 1.55<br>(1.3 to 1.81) | < 0.001 |
| Middle-aged and elderly age | Prevalence | Female | Montenegro | T1D | 1.53<br>(1.25 to 1.81) | < 0.001 |
| Middle-aged and elderly age | Prevalence | Male | Montenegro | T1D | 1.55<br>(1.28 to 1.82) | < 0.001 |
| Middle-aged and elderly age | Prevalence | Both | Montenegro | IBD | -0.07<br>(-0.14 to 0) | 0.055 |
| Middle-aged and elderly age | Prevalence | Female | Montenegro | IBD | -0.03<br>(-0.15 to 0.09) | 0.603 |
| Middle-aged and elderly age | Prevalence | Male | Montenegro | IBD | -0.09<br>(-0.15 to -0.03) | 0.002 |
| Middle-aged and elderly age | Prevalence | Both | Montenegro | MS | 0.24<br>(0.16 to 0.33) | < 0.001 |
| Middle-aged and elderly age | Prevalence | Female | Montenegro | MS | 0.45<br>(0.38 to 0.53) | < 0.001 |
| Middle-aged and elderly age | Prevalence | Male | Montenegro | MS | 0.03<br>(-0.07 to 0.12) | 0.591 |
| Middle-aged and elderly age | Prevalence | Both | Montenegro | Psoriasis | -0.68<br>(-0.71 to -0.64) | < 0.001 |
| Middle-aged and elderly age | Prevalence | Female | Montenegro | Psoriasis | -0.69<br>(-0.74 to -0.64) | < 0.001 |
| Middle-aged and elderly age | Prevalence | Male | Montenegro | Psoriasis | -0.66<br>(-0.68 to -0.64) | < 0.001 |
| Middle-aged and elderly age | Prevalence | Both | Montenegro | RHD | -0.46<br>(-0.55 to -0.36) | < 0.001 |
| Middle-aged and elderly age | Prevalence | Female | Montenegro | RHD | -0.48<br>(-0.57 to -0.4) | < 0.001 |
| Middle-aged and elderly age | Prevalence | Male | Montenegro | RHD | -0.37 | < 0.001 |

| | | | | | | |
|---|---|---|---|---|---|---|
| | | | | | (-0.51 to -0.24) | |
| Middle-aged and elderly age | Prevalence | Both | Montenegro | RA | 0.57<br>(0.5 to 0.63) | < 0.001 |
| Middle-aged and elderly age | Prevalence | Female | Montenegro | RA | 0.58<br>(0.5 to 0.67) | < 0.001 |
| Middle-aged and elderly age | Prevalence | Male | Montenegro | RA | 0.55<br>(0.48 to 0.62) | < 0.001 |
| Middle-aged and elderly age | Prevalence | Both | Morocco | AA | -0.02<br>(-0.04 to -0.01) | < 0.001 |
| Middle-aged and elderly age | Prevalence | Female | Morocco | AA | 0<br>(0 to 0) | < 0.001 |
| Middle-aged and elderly age | Prevalence | Male | Morocco | AA | 0<br>(0 to 0) | 0.015 |
| Middle-aged and elderly age | Prevalence | Both | Morocco | T1D | 2.02<br>(2 to 2.04) | < 0.001 |
| Middle-aged and elderly age | Prevalence | Female | Morocco | T1D | 2.01<br>(1.94 to 2.08) | < 0.001 |
| Middle-aged and elderly age | Prevalence | Male | Morocco | T1D | 2.03<br>(2 to 2.07) | < 0.001 |
| Middle-aged and elderly age | Prevalence | Both | Morocco | IBD | 0.43<br>(0.36 to 0.51) | < 0.001 |
| Middle-aged and elderly age | Prevalence | Female | Morocco | IBD | 0.43<br>(0.36 to 0.49) | < 0.001 |
| Middle-aged and elderly age | Prevalence | Male | Morocco | IBD | 0.44<br>(0.35 to 0.53) | < 0.001 |
| Middle-aged and elderly age | Prevalence | Both | Morocco | MS | 0.84<br>(0.8 to 0.88) | < 0.001 |
| Middle-aged and elderly age | Prevalence | Female | Morocco | MS | 0.79<br>(0.74 to 0.84) | < 0.001 |
| Middle-aged and elderly age | Prevalence | Male | Morocco | MS | 0.95<br>(0.92 to 0.98) | < 0.001 |
| Middle-aged and elderly age | Prevalence | Both | Morocco | Psoriasis | -0.87<br>(-0.87 to -0.86) | < 0.001 |

| | | | | | | |
|---|---|---|---|---|---|---|
| Middle-aged and elderly age | Prevalence | Female | Morocco | Psoriasis | -0.88 (-0.89 to -0.88) | < 0.001 |
| Middle-aged and elderly age | Prevalence | Male | Morocco | Psoriasis | -0.84 (-0.85 to -0.83) | < 0.001 |
| Middle-aged and elderly age | Prevalence | Both | Morocco | RHD | 0.1 (0.08 to 0.12) | < 0.001 |
| Middle-aged and elderly age | Prevalence | Female | Morocco | RHD | 0.12 (0.11 to 0.13) | < 0.001 |
| Middle-aged and elderly age | Prevalence | Male | Morocco | RHD | 0.08 (0.07 to 0.09) | < 0.001 |
| Middle-aged and elderly age | Prevalence | Both | Morocco | RA | 0.99 (0.94 to 1.04) | < 0.001 |
| Middle-aged and elderly age | Prevalence | Female | Morocco | RA | 1.06 (1.02 to 1.09) | < 0.001 |
| Middle-aged and elderly age | Prevalence | Male | Morocco | RA | 0.94 (0.89 to 0.98) | < 0.001 |
| Middle-aged and elderly age | Prevalence | Both | Mozambique | AA | 0.03 (0.03 to 0.04) | < 0.001 |
| Middle-aged and elderly age | Prevalence | Female | Mozambique | AA | 0 (0 to 0) | 1.000 |
| Middle-aged and elderly age | Prevalence | Male | Mozambique | AA | 0 (0 to 0) | < 0.001 |
| Middle-aged and elderly age | Prevalence | Both | Mozambique | T1D | 0.26 (0.25 to 0.28) | < 0.001 |
| Middle-aged and elderly age | Prevalence | Female | Mozambique | T1D | 0.27 (0.26 to 0.29) | < 0.001 |
| Middle-aged and elderly age | Prevalence | Male | Mozambique | T1D | 0.26 (0.23 to 0.28) | < 0.001 |
| Middle-aged and elderly age | Prevalence | Both | Mozambique | IBD | 0.28 (0.24 to 0.32) | < 0.001 |
| Middle-aged and elderly age | Prevalence | Female | Mozambique | IBD | 0.23 (0.18 to 0.28) | < 0.001 |
| Middle-aged and elderly age | Prevalence | Male | Mozambique | IBD | 0.34 | < 0.001 |

| | | | | | | |
|---|---|---|---|---|---|---|
| | | | | | (0.29 to 0.39) | |
| Middle-aged and elderly age | Prevalence | Both | Mozambique | MS | 0.36 (0.32 to 0.4) | < 0.001 |
| Middle-aged and elderly age | Prevalence | Female | Mozambique | MS | 0.28 (0.22 to 0.33) | < 0.001 |
| Middle-aged and elderly age | Prevalence | Male | Mozambique | MS | 0.41 (0.38 to 0.45) | < 0.001 |
| Middle-aged and elderly age | Prevalence | Both | Mozambique | Psoriasis | -0.49 (-0.53 to -0.45) | < 0.001 |
| Middle-aged and elderly age | Prevalence | Female | Mozambique | Psoriasis | -0.52 (-0.63 to -0.42) | < 0.001 |
| Middle-aged and elderly age | Prevalence | Male | Mozambique | Psoriasis | -0.47 (-0.56 to -0.39) | < 0.001 |
| Middle-aged and elderly age | Prevalence | Both | Mozambique | RHD | 0.15 (0.13 to 0.16) | < 0.001 |
| Middle-aged and elderly age | Prevalence | Female | Mozambique | RHD | 0.18 (0.16 to 0.2) | < 0.001 |
| Middle-aged and elderly age | Prevalence | Male | Mozambique | RHD | 0.07 (0.05 to 0.09) | < 0.001 |
| Middle-aged and elderly age | Prevalence | Both | Mozambique | RA | 0.81 (0.77 to 0.86) | < 0.001 |
| Middle-aged and elderly age | Prevalence | Female | Mozambique | RA | 0.78 (0.71 to 0.84) | < 0.001 |
| Middle-aged and elderly age | Prevalence | Male | Mozambique | RA | 0.89 (0.84 to 0.94) | < 0.001 |
| Middle-aged and elderly age | Prevalence | Both | Myanmar | AA | 0.04 (0.03 to 0.04) | < 0.001 |
| Middle-aged and elderly age | Prevalence | Female | Myanmar | AA | 0 (0 to 0) | < 0.001 |
| Middle-aged and elderly age | Prevalence | Male | Myanmar | AA | 0 (0 to 0) | < 0.001 |
| Middle-aged and elderly age | Prevalence | Both | Myanmar | T1D | 0.8 (0.79 to 0.81) | < 0.001 |

| | | | | | | |
|---|---|---|---|---|---|---|
| Middle-aged and elderly age | Prevalence | Female | Myanmar | T1D | 0.74 (0.73 to 0.75) | < 0.001 |
| Middle-aged and elderly age | Prevalence | Male | Myanmar | T1D | 0.87 (0.85 to 0.9) | < 0.001 |
| Middle-aged and elderly age | Prevalence | Both | Myanmar | IBD | 0.4 (0.33 to 0.47) | < 0.001 |
| Middle-aged and elderly age | Prevalence | Female | Myanmar | IBD | 0.18 (0.1 to 0.25) | < 0.001 |
| Middle-aged and elderly age | Prevalence | Male | Myanmar | IBD | 0.63 (0.59 to 0.67) | < 0.001 |
| Middle-aged and elderly age | Prevalence | Both | Myanmar | MS | 0.33 (0.28 to 0.38) | < 0.001 |
| Middle-aged and elderly age | Prevalence | Female | Myanmar | MS | 0.24 (0.14 to 0.35) | < 0.001 |
| Middle-aged and elderly age | Prevalence | Male | Myanmar | MS | 0.39 (0.36 to 0.42) | < 0.001 |
| Middle-aged and elderly age | Prevalence | Both | Myanmar | Psoriasis | -0.75 (-0.77 to -0.73) | < 0.001 |
| Middle-aged and elderly age | Prevalence | Female | Myanmar | Psoriasis | -0.71 (-0.72 to -0.7) | < 0.001 |
| Middle-aged and elderly age | Prevalence | Male | Myanmar | Psoriasis | -0.73 (-0.77 to -0.69) | < 0.001 |
| Middle-aged and elderly age | Prevalence | Both | Myanmar | RHD | -0.03 (-0.11 to 0.04) | 0.382 |
| Middle-aged and elderly age | Prevalence | Female | Myanmar | RHD | -0.13 (-0.19 to -0.07) | < 0.001 |
| Middle-aged and elderly age | Prevalence | Male | Myanmar | RHD | 0.07 (-0.03 to 0.17) | 0.146 |
| Middle-aged and elderly age | Prevalence | Both | Myanmar | RA | 0.79 (0.75 to 0.83) | < 0.001 |
| Middle-aged and elderly age | Prevalence | Female | Myanmar | RA | 0.74 (0.7 to 0.78) | < 0.001 |
| Middle-aged and elderly age | Prevalence | Male | Myanmar | RA | 0.58 | < 0.001 |

| | | | | | | |
|---|---|---|---|---|---|---|
| | | | | | (0.56 to 0.61) | |
| Middle-aged and elderly age | Prevalence | Both | Namibia | AA | 0.06<br>(0.05 to 0.07) | < 0.001 |
| Middle-aged and elderly age | Prevalence | Female | Namibia | AA | 0<br>(0 to 0) | 0.027 |
| Middle-aged and elderly age | Prevalence | Male | Namibia | AA | 0<br>(0 to 0) | < 0.001 |
| Middle-aged and elderly age | Prevalence | Both | Namibia | T1D | 0.36<br>(0.36 to 0.37) | < 0.001 |
| Middle-aged and elderly age | Prevalence | Female | Namibia | T1D | 0.37<br>(0.37 to 0.38) | < 0.001 |
| Middle-aged and elderly age | Prevalence | Male | Namibia | T1D | 0.36<br>(0.35 to 0.37) | < 0.001 |
| Middle-aged and elderly age | Prevalence | Both | Namibia | IBD | 0.7<br>(0.65 to 0.75) | < 0.001 |
| Middle-aged and elderly age | Prevalence | Female | Namibia | IBD | 0.64<br>(0.57 to 0.71) | < 0.001 |
| Middle-aged and elderly age | Prevalence | Male | Namibia | IBD | 0.77<br>(0.72 to 0.82) | < 0.001 |
| Middle-aged and elderly age | Prevalence | Both | Namibia | MS | 0.31<br>(0.29 to 0.34) | < 0.001 |
| Middle-aged and elderly age | Prevalence | Female | Namibia | MS | 0.2<br>(0.18 to 0.22) | < 0.001 |
| Middle-aged and elderly age | Prevalence | Male | Namibia | MS | 0.36<br>(0.34 to 0.38) | < 0.001 |
| Middle-aged and elderly age | Prevalence | Both | Namibia | Psoriasis | -0.58<br>(-0.65 to -0.51) | < 0.001 |
| Middle-aged and elderly age | Prevalence | Female | Namibia | Psoriasis | -0.59<br>(-0.67 to -0.52) | < 0.001 |
| Middle-aged and elderly age | Prevalence | Male | Namibia | Psoriasis | -0.55<br>(-0.61 to -0.49) | < 0.001 |
| Middle-aged and elderly age | Prevalence | Both | Namibia | RHD | 0.01<br>(0 to 0.03) | 0.008 |

| | | | | | | |
|---|---|---|---|---|---|---|
| Middle-aged and elderly age | Prevalence | Female | Namibia | RHD | -0.01 (-0.03 to 0.01) | 0.412 |
| Middle-aged and elderly age | Prevalence | Male | Namibia | RHD | 0 (-0.02 to 0.03) | 0.741 |
| Middle-aged and elderly age | Prevalence | Both | Namibia | RA | 0.87 (0.76 to 0.98) | < 0.001 |
| Middle-aged and elderly age | Prevalence | Female | Namibia | RA | 0.77 (0.64 to 0.9) | < 0.001 |
| Middle-aged and elderly age | Prevalence | Male | Namibia | RA | 0.88 (0.81 to 0.94) | < 0.001 |
| Middle-aged and elderly age | Prevalence | Both | Nauru | AA | 0.2 (0.19 to 0.22) | < 0.001 |
| Middle-aged and elderly age | Prevalence | Female | Nauru | AA | 0 (0 to 0) | < 0.001 |
| Middle-aged and elderly age | Prevalence | Male | Nauru | AA | 0 (0 to 0) | < 0.001 |
| Middle-aged and elderly age | Prevalence | Both | Nauru | T1D | 0.64 (0.63 to 0.66) | < 0.001 |
| Middle-aged and elderly age | Prevalence | Female | Nauru | T1D | 0.66 (0.65 to 0.68) | < 0.001 |
| Middle-aged and elderly age | Prevalence | Male | Nauru | T1D | 0.72 (0.7 to 0.73) | < 0.001 |
| Middle-aged and elderly age | Prevalence | Both | Nauru | IBD | -0.2 (-0.63 to 0.23) | 0.358 |
| Middle-aged and elderly age | Prevalence | Female | Nauru | IBD | -0.19 (-0.58 to 0.21) | 0.352 |
| Middle-aged and elderly age | Prevalence | Male | Nauru | IBD | -0.18 (-0.48 to 0.11) | 0.228 |
| Middle-aged and elderly age | Prevalence | Both | Nauru | MS | 0.34 (0.27 to 0.41) | < 0.001 |
| Middle-aged and elderly age | Prevalence | Female | Nauru | MS | 0.19 (0.15 to 0.23) | < 0.001 |
| Middle-aged and elderly age | Prevalence | Male | Nauru | MS | 0.22 | < 0.001 |

| | | | | | | |
|---|---|---|---|---|---|---|
| | | | | | (0.16 to 0.29) | |
| Middle-aged and elderly age | Prevalence | Both | Nauru | Psoriasis | -0.53 (-0.6 to -0.46) | < 0.001 |
| Middle-aged and elderly age | Prevalence | Female | Nauru | Psoriasis | -0.39 (-0.46 to -0.33) | < 0.001 |
| Middle-aged and elderly age | Prevalence | Male | Nauru | Psoriasis | -0.36 (-0.46 to -0.26) | < 0.001 |
| Middle-aged and elderly age | Prevalence | Both | Nauru | RHD | -0.72 (-0.94 to -0.5) | < 0.001 |
| Middle-aged and elderly age | Prevalence | Female | Nauru | RHD | -1.03 (-1.28 to -0.77) | < 0.001 |
| Middle-aged and elderly age | Prevalence | Male | Nauru | RHD | -0.63 (-0.75 to -0.51) | < 0.001 |
| Middle-aged and elderly age | Prevalence | Both | Nauru | RA | 0.59 (0.51 to 0.66) | < 0.001 |
| Middle-aged and elderly age | Prevalence | Female | Nauru | RA | 0.39 (0.34 to 0.45) | < 0.001 |
| Middle-aged and elderly age | Prevalence | Male | Nauru | RA | 0.23 (0.17 to 0.29) | < 0.001 |
| Middle-aged and elderly age | Prevalence | Both | Nepal | AA | 0.08 (0.07 to 0.09) | < 0.001 |
| Middle-aged and elderly age | Prevalence | Female | Nepal | AA | 0 (0 to 0) | < 0.001 |
| Middle-aged and elderly age | Prevalence | Male | Nepal | AA | 0 (0 to 0) | 0.001 |
| Middle-aged and elderly age | Prevalence | Both | Nepal | T1D | 0.53 (0.51 to 0.54) | < 0.001 |
| Middle-aged and elderly age | Prevalence | Female | Nepal | T1D | 0.51 (0.49 to 0.52) | < 0.001 |
| Middle-aged and elderly age | Prevalence | Male | Nepal | T1D | 0.58 (0.57 to 0.6) | < 0.001 |
| Middle-aged and elderly age | Prevalence | Both | Nepal | IBD | 0.44 (0.41 to 0.46) | < 0.001 |

| | | | | | | |
|---|---|---|---|---|---|---|
| Middle-aged and elderly age | Prevalence | Female | Nepal | IBD | 0.44<br>(0.39 to 0.48) | < 0.001 |
| Middle-aged and elderly age | Prevalence | Male | Nepal | IBD | 0.5<br>(0.49 to 0.52) | < 0.001 |
| Middle-aged and elderly age | Prevalence | Both | Nepal | MS | 0.48<br>(0.44 to 0.53) | < 0.001 |
| Middle-aged and elderly age | Prevalence | Female | Nepal | MS | 0.4<br>(0.37 to 0.44) | < 0.001 |
| Middle-aged and elderly age | Prevalence | Male | Nepal | MS | 0.4<br>(0.32 to 0.49) | < 0.001 |
| Middle-aged and elderly age | Prevalence | Both | Nepal | Psoriasis | -1.07<br>(-1.14 to -0.99) | < 0.001 |
| Middle-aged and elderly age | Prevalence | Female | Nepal | Psoriasis | -1.08<br>(-1.14 to -1.01) | < 0.001 |
| Middle-aged and elderly age | Prevalence | Male | Nepal | Psoriasis | -1.07<br>(-1.15 to -0.99) | < 0.001 |
| Middle-aged and elderly age | Prevalence | Both | Nepal | RHD | 0.04<br>(-0.01 to 0.08) | 0.097 |
| Middle-aged and elderly age | Prevalence | Female | Nepal | RHD | 0.04<br>(0.01 to 0.07) | 0.017 |
| Middle-aged and elderly age | Prevalence | Male | Nepal | RHD | -0.04<br>(-0.06 to -0.02) | < 0.001 |
| Middle-aged and elderly age | Prevalence | Both | Nepal | RA | 1.38<br>(1.35 to 1.41) | < 0.001 |
| Middle-aged and elderly age | Prevalence | Female | Nepal | RA | 1.29<br>(1.26 to 1.32) | < 0.001 |
| Middle-aged and elderly age | Prevalence | Male | Nepal | RA | 1.26<br>(1.22 to 1.31) | < 0.001 |
| Middle-aged and elderly age | Prevalence | Both | Netherlands | AA | 0<br>(0 to 0) | 0.014 |
| Middle-aged and elderly age | Prevalence | Female | Netherlands | AA | 0<br>(0 to 0) | < 0.001 |
| Middle-aged and elderly age | Prevalence | Male | Netherlands | AA | -0.01 | < 0.001 |

| | | | | | | |
|---|---|---|---|---|---|---|
| | | | | | (-0.01 to -0.01) | |
| Middle-aged and elderly age | Prevalence | Both | Netherlands | T1D | 2.03<br>(1.85 to 2.21) | < 0.001 |
| Middle-aged and elderly age | Prevalence | Female | Netherlands | T1D | 1.99<br>(1.73 to 2.25) | < 0.001 |
| Middle-aged and elderly age | Prevalence | Male | Netherlands | T1D | 2.07<br>(1.89 to 2.26) | < 0.001 |
| Middle-aged and elderly age | Prevalence | Both | Netherlands | IBD | -1.64<br>(-1.8 to -1.49) | < 0.001 |
| Middle-aged and elderly age | Prevalence | Female | Netherlands | IBD | -1.42<br>(-1.56 to -1.29) | < 0.001 |
| Middle-aged and elderly age | Prevalence | Male | Netherlands | IBD | -1.81<br>(-2 to -1.62) | < 0.001 |
| Middle-aged and elderly age | Prevalence | Both | Netherlands | MS | 0.26<br>(0.21 to 0.31) | < 0.001 |
| Middle-aged and elderly age | Prevalence | Female | Netherlands | MS | 0.4<br>(0.36 to 0.43) | < 0.001 |
| Middle-aged and elderly age | Prevalence | Male | Netherlands | MS | -0.03<br>(-0.1 to 0.04) | 0.383 |
| Middle-aged and elderly age | Prevalence | Both | Netherlands | Psoriasis | -0.35<br>(-0.36 to -0.34) | < 0.001 |
| Middle-aged and elderly age | Prevalence | Female | Netherlands | Psoriasis | -0.34<br>(-0.36 to -0.33) | < 0.001 |
| Middle-aged and elderly age | Prevalence | Male | Netherlands | Psoriasis | -0.35<br>(-0.36 to -0.34) | < 0.001 |
| Middle-aged and elderly age | Prevalence | Both | Netherlands | RHD | -0.2<br>(-0.36 to -0.03) | 0.018 |
| Middle-aged and elderly age | Prevalence | Female | Netherlands | RHD | -0.28<br>(-0.45 to -0.12) | 0.001 |
| Middle-aged and elderly age | Prevalence | Male | Netherlands | RHD | -0.16<br>(-0.33 to 0) | 0.050 |
| Middle-aged and elderly age | Prevalence | Both | Netherlands | RA | -0.02<br>(-0.15 to 0.1) | 0.727 |

| | | | | | | |
|---|---|---|---|---|---|---|
| Middle-aged and elderly age | Prevalence | Female | Netherlands | RA | -0.05<br>(-0.14 to 0.04) | 0.308 |
| Middle-aged and elderly age | Prevalence | Male | Netherlands | RA | 0.15<br>(-0.12 to 0.41) | 0.273 |
| Middle-aged and elderly age | Prevalence | Both | New Zealand | AA | 0.09<br>(0.09 to 0.1) | < 0.001 |
| Middle-aged and elderly age | Prevalence | Female | New Zealand | AA | 0<br>(0 to 0) | 1.000 |
| Middle-aged and elderly age | Prevalence | Male | New Zealand | AA | 0<br>(0 to 0) | < 0.001 |
| Middle-aged and elderly age | Prevalence | Both | New Zealand | T1D | 0.47<br>(0.43 to 0.51) | < 0.001 |
| Middle-aged and elderly age | Prevalence | Female | New Zealand | T1D | 0.33<br>(0.26 to 0.4) | < 0.001 |
| Middle-aged and elderly age | Prevalence | Male | New Zealand | T1D | 0.61<br>(0.58 to 0.65) | < 0.001 |
| Middle-aged and elderly age | Prevalence | Both | New Zealand | IBD | -0.32<br>(-0.34 to -0.29) | < 0.001 |
| Middle-aged and elderly age | Prevalence | Female | New Zealand | IBD | -0.35<br>(-0.38 to -0.31) | < 0.001 |
| Middle-aged and elderly age | Prevalence | Male | New Zealand | IBD | -0.29<br>(-0.32 to -0.26) | < 0.001 |
| Middle-aged and elderly age | Prevalence | Both | New Zealand | MS | -0.22<br>(-0.25 to -0.18) | < 0.001 |
| Middle-aged and elderly age | Prevalence | Female | New Zealand | MS | -0.14<br>(-0.17 to -0.12) | < 0.001 |
| Middle-aged and elderly age | Prevalence | Male | New Zealand | MS | -0.69<br>(-0.75 to -0.63) | < 0.001 |
| Middle-aged and elderly age | Prevalence | Both | New Zealand | Psoriasis | -0.38<br>(-0.39 to -0.37) | < 0.001 |
| Middle-aged and elderly age | Prevalence | Female | New Zealand | Psoriasis | -0.38<br>(-0.39 to -0.37) | < 0.001 |
| Middle-aged and elderly age | Prevalence | Male | New Zealand | Psoriasis | -0.39 | < 0.001 |

| | | | | | | |
|---|---|---|---|---|---|---|
| | | | | | (-0.41 to -0.37) | |
| Middle-aged and elderly age | Prevalence | Both | New Zealand | RHD | -1.24 (-1.32 to -1.17) | < 0.001 |
| Middle-aged and elderly age | Prevalence | Female | New Zealand | RHD | -1.34 (-1.65 to -1.04) | < 0.001 |
| Middle-aged and elderly age | Prevalence | Male | New Zealand | RHD | -1.11 (-1.4 to -0.81) | < 0.001 |
| Middle-aged and elderly age | Prevalence | Both | New Zealand | RA | -0.07 (-0.15 to 0.01) | 0.076 |
| Middle-aged and elderly age | Prevalence | Female | New Zealand | RA | -0.26 (-0.35 to -0.17) | < 0.001 |
| Middle-aged and elderly age | Prevalence | Male | New Zealand | RA | 0.08 (-0.08 to 0.24) | 0.320 |
| Middle-aged and elderly age | Prevalence | Both | Nicaragua | AA | 0.05 (0.05 to 0.06) | < 0.001 |
| Middle-aged and elderly age | Prevalence | Female | Nicaragua | AA | 0 (0 to 0) | < 0.001 |
| Middle-aged and elderly age | Prevalence | Male | Nicaragua | AA | 0 (0 to 0) | < 0.001 |
| Middle-aged and elderly age | Prevalence | Both | Nicaragua | T1D | 0.7 (0.69 to 0.72) | < 0.001 |
| Middle-aged and elderly age | Prevalence | Female | Nicaragua | T1D | 0.73 (0.71 to 0.74) | < 0.001 |
| Middle-aged and elderly age | Prevalence | Male | Nicaragua | T1D | 0.69 (0.68 to 0.71) | < 0.001 |
| Middle-aged and elderly age | Prevalence | Both | Nicaragua | IBD | 0.35 (0.22 to 0.48) | < 0.001 |
| Middle-aged and elderly age | Prevalence | Female | Nicaragua | IBD | 0.37 (0.28 to 0.45) | < 0.001 |
| Middle-aged and elderly age | Prevalence | Male | Nicaragua | IBD | 0.4 (0.23 to 0.58) | < 0.001 |
| Middle-aged and elderly age | Prevalence | Both | Nicaragua | MS | 0.88 (0.86 to 0.9) | < 0.001 |

| | | | | | | |
|---|---|---|---|---|---|---|
| Middle-aged and elderly age | Prevalence | Female | Nicaragua | MS | 0.93 (0.9 to 0.95) | < 0.001 |
| Middle-aged and elderly age | Prevalence | Male | Nicaragua | MS | 0.53 (0.51 to 0.55) | < 0.001 |
| Middle-aged and elderly age | Prevalence | Both | Nicaragua | Psoriasis | -0.56 (-0.58 to -0.55) | < 0.001 |
| Middle-aged and elderly age | Prevalence | Female | Nicaragua | Psoriasis | -0.58 (-0.59 to -0.56) | < 0.001 |
| Middle-aged and elderly age | Prevalence | Male | Nicaragua | Psoriasis | -0.55 (-0.57 to -0.54) | < 0.001 |
| Middle-aged and elderly age | Prevalence | Both | Nicaragua | RHD | 0.14 (0.1 to 0.18) | < 0.001 |
| Middle-aged and elderly age | Prevalence | Female | Nicaragua | RHD | 0.12 (0.06 to 0.17) | < 0.001 |
| Middle-aged and elderly age | Prevalence | Male | Nicaragua | RHD | 0.13 (0.11 to 0.15) | < 0.001 |
| Middle-aged and elderly age | Prevalence | Both | Nicaragua | RA | 1.15 (1.09 to 1.21) | < 0.001 |
| Middle-aged and elderly age | Prevalence | Female | Nicaragua | RA | 1.1 (1.03 to 1.17) | < 0.001 |
| Middle-aged and elderly age | Prevalence | Male | Nicaragua | RA | 0.98 (0.92 to 1.04) | < 0.001 |
| Middle-aged and elderly age | Prevalence | Both | Niger | AA | 0.11 (0.08 to 0.14) | < 0.001 |
| Middle-aged and elderly age | Prevalence | Female | Niger | AA | 0 (0 to 0) | 0.027 |
| Middle-aged and elderly age | Prevalence | Male | Niger | AA | 0 (0 to 0) | < 0.001 |
| Middle-aged and elderly age | Prevalence | Both | Niger | T1D | 0.12 (0.11 to 0.13) | < 0.001 |
| Middle-aged and elderly age | Prevalence | Female | Niger | T1D | 0.13 (0.12 to 0.13) | < 0.001 |
| Middle-aged and elderly age | Prevalence | Male | Niger | T1D | 0.12 | < 0.001 |

| | | | | | | |
|---|---|---|---|---|---|---|
| | | | | | (0.12 to 0.13) | |
| Middle-aged and elderly age | Prevalence | Both | Niger | IBD | 0.28<br>(0.21 to 0.36) | < 0.001 |
| Middle-aged and elderly age | Prevalence | Female | Niger | IBD | 0.27<br>(0.13 to 0.4) | < 0.001 |
| Middle-aged and elderly age | Prevalence | Male | Niger | IBD | 0.27<br>(0.22 to 0.32) | < 0.001 |
| Middle-aged and elderly age | Prevalence | Both | Niger | MS | 0.31<br>(0.26 to 0.35) | < 0.001 |
| Middle-aged and elderly age | Prevalence | Female | Niger | MS | 0.14<br>(0.11 to 0.18) | < 0.001 |
| Middle-aged and elderly age | Prevalence | Male | Niger | MS | 0.22<br>(0.17 to 0.27) | < 0.001 |
| Middle-aged and elderly age | Prevalence | Both | Niger | Psoriasis | -0.33<br>(-0.44 to -0.22) | < 0.001 |
| Middle-aged and elderly age | Prevalence | Female | Niger | Psoriasis | -0.43<br>(-0.56 to -0.29) | < 0.001 |
| Middle-aged and elderly age | Prevalence | Male | Niger | Psoriasis | -0.29<br>(-0.48 to -0.09) | 0.003 |
| Middle-aged and elderly age | Prevalence | Both | Niger | RHD | 0.18<br>(0.13 to 0.22) | < 0.001 |
| Middle-aged and elderly age | Prevalence | Female | Niger | RHD | 0.19<br>(0.14 to 0.24) | < 0.001 |
| Middle-aged and elderly age | Prevalence | Male | Niger | RHD | 0.11<br>(0.09 to 0.12) | < 0.001 |
| Middle-aged and elderly age | Prevalence | Both | Niger | RA | 0.63<br>(0.59 to 0.67) | < 0.001 |
| Middle-aged and elderly age | Prevalence | Female | Niger | RA | 0.54<br>(0.5 to 0.59) | < 0.001 |
| Middle-aged and elderly age | Prevalence | Male | Niger | RA | 0.6<br>(0.55 to 0.65) | < 0.001 |
| Middle-aged and elderly age | Prevalence | Both | Nigeria | AA | 0.32<br>(0.29 to 0.35) | < 0.001 |

| | | | | | | |
|---|---|---|---|---|---|---|
| Middle-aged and elderly age | Prevalence | Female | Nigeria | AA | 0<br>(0 to 0) | < 0.001 |
| Middle-aged and elderly age | Prevalence | Male | Nigeria | AA | 0<br>(0 to 0) | < 0.001 |
| Middle-aged and elderly age | Prevalence | Both | Nigeria | T1D | 0.6<br>(0.58 to 0.61) | < 0.001 |
| Middle-aged and elderly age | Prevalence | Female | Nigeria | T1D | 0.64<br>(0.61 to 0.67) | < 0.001 |
| Middle-aged and elderly age | Prevalence | Male | Nigeria | T1D | 0.58<br>(0.56 to 0.6) | < 0.001 |
| Middle-aged and elderly age | Prevalence | Both | Nigeria | IBD | 0.65<br>(0.59 to 0.7) | < 0.001 |
| Middle-aged and elderly age | Prevalence | Female | Nigeria | IBD | 0.59<br>(0.47 to 0.71) | < 0.001 |
| Middle-aged and elderly age | Prevalence | Male | Nigeria | IBD | 0.64<br>(0.57 to 0.71) | < 0.001 |
| Middle-aged and elderly age | Prevalence | Both | Nigeria | MS | 0.65<br>(0.59 to 0.7) | < 0.001 |
| Middle-aged and elderly age | Prevalence | Female | Nigeria | MS | 0.18<br>(0.11 to 0.25) | < 0.001 |
| Middle-aged and elderly age | Prevalence | Male | Nigeria | MS | 0.41<br>(0.34 to 0.48) | < 0.001 |
| Middle-aged and elderly age | Prevalence | Both | Nigeria | Psoriasis | -1.25<br>(-1.29 to -1.22) | < 0.001 |
| Middle-aged and elderly age | Prevalence | Female | Nigeria | Psoriasis | -1.36<br>(-1.41 to -1.3) | < 0.001 |
| Middle-aged and elderly age | Prevalence | Male | Nigeria | Psoriasis | -1.17<br>(-1.2 to -1.15) | < 0.001 |
| Middle-aged and elderly age | Prevalence | Both | Nigeria | RHD | 0.33<br>(0.31 to 0.36) | < 0.001 |
| Middle-aged and elderly age | Prevalence | Female | Nigeria | RHD | 0.38<br>(0.36 to 0.41) | < 0.001 |
| Middle-aged and elderly age | Prevalence | Male | Nigeria | RHD | 0.17 | < 0.001 |

| | | | | | | |
|---|---|---|---|---|---|---|
| | | | | | (0.15 to 0.2) | |
| Middle-aged and elderly age | Prevalence | Both | Nigeria | RA | 1.01<br>(0.91 to 1.11) | < 0.001 |
| Middle-aged and elderly age | Prevalence | Female | Nigeria | RA | 0.36<br>(0.32 to 0.41) | < 0.001 |
| Middle-aged and elderly age | Prevalence | Male | Nigeria | RA | 1.36<br>(1.16 to 1.56) | < 0.001 |
| Middle-aged and elderly age | Prevalence | Both | Niue | AA | -0.09<br>(-0.11 to -0.07) | < 0.001 |
| Middle-aged and elderly age | Prevalence | Female | Niue | AA | 0<br>(0 to 0) | < 0.001 |
| Middle-aged and elderly age | Prevalence | Male | Niue | AA | 0<br>(0 to 0) | < 0.001 |
| Middle-aged and elderly age | Prevalence | Both | Niue | T1D | 1.03<br>(0.99 to 1.07) | < 0.001 |
| Middle-aged and elderly age | Prevalence | Female | Niue | T1D | 0.97<br>(0.95 to 0.98) | < 0.001 |
| Middle-aged and elderly age | Prevalence | Male | Niue | T1D | 1.04<br>(0.96 to 1.12) | < 0.001 |
| Middle-aged and elderly age | Prevalence | Both | Niue | IBD | -0.34<br>(-0.45 to -0.23) | < 0.001 |
| Middle-aged and elderly age | Prevalence | Female | Niue | IBD | -0.38<br>(-0.67 to -0.09) | 0.010 |
| Middle-aged and elderly age | Prevalence | Male | Niue | IBD | -0.26<br>(-0.32 to -0.21) | < 0.001 |
| Middle-aged and elderly age | Prevalence | Both | Niue | MS | 0.13<br>(0.09 to 0.16) | < 0.001 |
| Middle-aged and elderly age | Prevalence | Female | Niue | MS | 0.2<br>(0.15 to 0.24) | < 0.001 |
| Middle-aged and elderly age | Prevalence | Male | Niue | MS | 0.21<br>(0.17 to 0.24) | < 0.001 |
| Middle-aged and elderly age | Prevalence | Both | Niue | Psoriasis | -0.51<br>(-0.53 to -0.48) | < 0.001 |

| | | | | | | |
|---|---|---|---|---|---|---|
| Middle-aged and elderly age | Prevalence | Female | Niue | Psoriasis | -0.53 (-0.54 to -0.52) | < 0.001 |
| Middle-aged and elderly age | Prevalence | Male | Niue | Psoriasis | -0.56 (-0.58 to -0.54) | < 0.001 |
| Middle-aged and elderly age | Prevalence | Both | Niue | RHD | -0.63 (-0.67 to -0.59) | < 0.001 |
| Middle-aged and elderly age | Prevalence | Female | Niue | RHD | -0.6 (-0.66 to -0.55) | < 0.001 |
| Middle-aged and elderly age | Prevalence | Male | Niue | RHD | -0.59 (-0.62 to -0.56) | < 0.001 |
| Middle-aged and elderly age | Prevalence | Both | Niue | RA | 0.5 (0.44 to 0.57) | < 0.001 |
| Middle-aged and elderly age | Prevalence | Female | Niue | RA | 0.68 (0.56 to 0.79) | < 0.001 |
| Middle-aged and elderly age | Prevalence | Male | Niue | RA | 0.54 (0.48 to 0.6) | < 0.001 |
| Middle-aged and elderly age | Prevalence | Both | North Macedonia | AA | -0.04 (-0.05 to -0.04) | < 0.001 |
| Middle-aged and elderly age | Prevalence | Female | North Macedonia | AA | 0 (0 to 0) | < 0.001 |
| Middle-aged and elderly age | Prevalence | Male | North Macedonia | AA | 0 (0 to 0) | < 0.001 |
| Middle-aged and elderly age | Prevalence | Both | North Macedonia | T1D | 1.52 (1.48 to 1.57) | < 0.001 |
| Middle-aged and elderly age | Prevalence | Female | North Macedonia | T1D | 1.54 (1.49 to 1.58) | < 0.001 |
| Middle-aged and elderly age | Prevalence | Male | North Macedonia | T1D | 1.52 (1.48 to 1.56) | < 0.001 |
| Middle-aged and elderly age | Prevalence | Both | North Macedonia | IBD | 0.12 (0.05 to 0.18) | 0.001 |
| Middle-aged and elderly age | Prevalence | Female | North Macedonia | IBD | 0.1 (0.07 to 0.13) | < 0.001 |
| Middle-aged and elderly age | Prevalence | Male | North Macedonia | IBD | 0.12 | 0.018 |

| | | | | | | |
|---|---|---|---|---|---|---|
| | | | | | (0.02 to 0.22) | |
| Middle-aged and elderly age | Prevalence | Both | North Macedonia | MS | 0.85<br>(0.82 to 0.88) | < 0.001 |
| Middle-aged and elderly age | Prevalence | Female | North Macedonia | MS | 0.9<br>(0.88 to 0.92) | < 0.001 |
| Middle-aged and elderly age | Prevalence | Male | North Macedonia | MS | 0.86<br>(0.81 to 0.9) | < 0.001 |
| Middle-aged and elderly age | Prevalence | Both | North Macedonia | Psoriasis | -0.82<br>(-0.85 to -0.8) | < 0.001 |
| Middle-aged and elderly age | Prevalence | Female | North Macedonia | Psoriasis | -0.86<br>(-0.89 to -0.82) | < 0.001 |
| Middle-aged and elderly age | Prevalence | Male | North Macedonia | Psoriasis | -0.81<br>(-0.83 to -0.79) | < 0.001 |
| Middle-aged and elderly age | Prevalence | Both | North Macedonia | RHD | -1.75<br>(-1.82 to -1.67) | < 0.001 |
| Middle-aged and elderly age | Prevalence | Female | North Macedonia | RHD | -2.09<br>(-2.15 to -2.03) | < 0.001 |
| Middle-aged and elderly age | Prevalence | Male | North Macedonia | RHD | -1.23<br>(-1.33 to -1.13) | < 0.001 |
| Middle-aged and elderly age | Prevalence | Both | North Macedonia | RA | 0.82<br>(0.72 to 0.92) | < 0.001 |
| Middle-aged and elderly age | Prevalence | Female | North Macedonia | RA | 0.9<br>(0.8 to 0.99) | < 0.001 |
| Middle-aged and elderly age | Prevalence | Male | North Macedonia | RA | 0.78<br>(0.66 to 0.91) | < 0.001 |
| Middle-aged and elderly age | Prevalence | Both | Northern Mariana Islands | AA | 0.34<br>(0.04 to 0.65) | 0.025 |
| Middle-aged and elderly age | Prevalence | Female | Northern Mariana Islands | AA | 0<br>(0 to 0) | < 0.001 |
| Middle-aged and elderly age | Prevalence | Male | Northern Mariana Islands | AA | 0<br>(0 to 0) | < 0.001 |
| Middle-aged and elderly age | Prevalence | Both | Northern Mariana Islands | T1D | 0.55<br>(0.41 to 0.68) | < 0.001 |

| | | | | | | |
|---|---|---|---|---|---|---|
| Middle-aged and elderly age | Prevalence | Female | Northern Mariana Islands | T1D | 0.61<br>(0.49 to 0.73) | < 0.001 |
| Middle-aged and elderly age | Prevalence | Male | Northern Mariana Islands | T1D | 0.67<br>(0.57 to 0.78) | < 0.001 |
| Middle-aged and elderly age | Prevalence | Both | Northern Mariana Islands | IBD | -0.63<br>(-0.71 to -0.56) | < 0.001 |
| Middle-aged and elderly age | Prevalence | Female | Northern Mariana Islands | IBD | -0.62<br>(-0.73 to -0.52) | < 0.001 |
| Middle-aged and elderly age | Prevalence | Male | Northern Mariana Islands | IBD | -0.58<br>(-0.66 to -0.51) | < 0.001 |
| Middle-aged and elderly age | Prevalence | Both | Northern Mariana Islands | MS | 0.36<br>(0.24 to 0.49) | < 0.001 |
| Middle-aged and elderly age | Prevalence | Female | Northern Mariana Islands | MS | 0.08<br>(0.07 to 0.09) | < 0.001 |
| Middle-aged and elderly age | Prevalence | Male | Northern Mariana Islands | MS | 0.08<br>(0.05 to 0.12) | < 0.001 |
| Middle-aged and elderly age | Prevalence | Both | Northern Mariana Islands | Psoriasis | -0.35<br>(-0.44 to -0.26) | < 0.001 |
| Middle-aged and elderly age | Prevalence | Female | Northern Mariana Islands | Psoriasis | -0.15<br>(-0.18 to -0.12) | < 0.001 |
| Middle-aged and elderly age | Prevalence | Male | Northern Mariana Islands | Psoriasis | -0.17<br>(-0.19 to -0.15) | < 0.001 |
| Middle-aged and elderly age | Prevalence | Both | Northern Mariana Islands | RHD | 0.23<br>(0.14 to 0.32) | < 0.001 |
| Middle-aged and elderly age | Prevalence | Female | Northern Mariana Islands | RHD | 0.13<br>(0.09 to 0.16) | < 0.001 |
| Middle-aged and elderly age | Prevalence | Male | Northern Mariana Islands | RHD | 0.12<br>(0.07 to 0.17) | < 0.001 |
| Middle-aged and elderly age | Prevalence | Both | Northern Mariana Islands | RA | 0.71<br>(0.55 to 0.86) | < 0.001 |
| Middle-aged and elderly age | Prevalence | Female | Northern Mariana Islands | RA | 0.36<br>(0.31 to 0.4) | < 0.001 |
| Middle-aged and elderly age | Prevalence | Male | Northern Mariana Islands | RA | 0.31 | < 0.001 |

| | | | | | | |
|---|---|---|---|---|---|---|
| | | | | | (0.26 to 0.36) | |
| Middle-aged and elderly age | Prevalence | Both | Norway | AA | -0.05 (-0.05 to -0.04) | < 0.001 |
| Middle-aged and elderly age | Prevalence | Female | Norway | AA | 0 (0 to 0) | < 0.001 |
| Middle-aged and elderly age | Prevalence | Male | Norway | AA | 0 (0 to 0) | < 0.001 |
| Middle-aged and elderly age | Prevalence | Both | Norway | T1D | 0.74 (0.56 to 0.92) | < 0.001 |
| Middle-aged and elderly age | Prevalence | Female | Norway | T1D | 1.05 (0.89 to 1.21) | < 0.001 |
| Middle-aged and elderly age | Prevalence | Male | Norway | T1D | 0.44 (0.39 to 0.49) | < 0.001 |
| Middle-aged and elderly age | Prevalence | Both | Norway | IBD | 0.41 (0.39 to 0.44) | < 0.001 |
| Middle-aged and elderly age | Prevalence | Female | Norway | IBD | 0.16 (0.15 to 0.17) | < 0.001 |
| Middle-aged and elderly age | Prevalence | Male | Norway | IBD | 0.74 (0.68 to 0.8) | < 0.001 |
| Middle-aged and elderly age | Prevalence | Both | Norway | MS | 1.58 (1.43 to 1.73) | < 0.001 |
| Middle-aged and elderly age | Prevalence | Female | Norway | MS | 1.87 (1.63 to 2.11) | < 0.001 |
| Middle-aged and elderly age | Prevalence | Male | Norway | MS | 1.23 (1.07 to 1.4) | < 0.001 |
| Middle-aged and elderly age | Prevalence | Both | Norway | Psoriasis | -0.27 (-0.31 to -0.22) | < 0.001 |
| Middle-aged and elderly age | Prevalence | Female | Norway | Psoriasis | -0.26 (-0.29 to -0.23) | < 0.001 |
| Middle-aged and elderly age | Prevalence | Male | Norway | Psoriasis | -0.28 (-0.35 to -0.21) | < 0.001 |
| Middle-aged and elderly age | Prevalence | Both | Norway | RHD | -0.78 (-0.88 to -0.67) | < 0.001 |

| | | | | | | |
|---|---|---|---|---|---|---|
| Middle-aged and elderly age | Prevalence | Female | Norway | RHD | -0.87 (-0.98 to -0.76) | < 0.001 |
| Middle-aged and elderly age | Prevalence | Male | Norway | RHD | -0.68 (-0.8 to -0.57) | < 0.001 |
| Middle-aged and elderly age | Prevalence | Both | Norway | RA | 0.16 (-0.02 to 0.35) | 0.087 |
| Middle-aged and elderly age | Prevalence | Female | Norway | RA | 0.12 (-0.16 to 0.4) | 0.404 |
| Middle-aged and elderly age | Prevalence | Male | Norway | RA | 0.36 (0.13 to 0.59) | 0.002 |
| Middle-aged and elderly age | Prevalence | Both | Oman | AA | -0.15 (-0.26 to -0.03) | 0.013 |
| Middle-aged and elderly age | Prevalence | Female | Oman | AA | 0 (0 to 0) | < 0.001 |
| Middle-aged and elderly age | Prevalence | Male | Oman | AA | 0 (0 to 0) | 0.015 |
| Middle-aged and elderly age | Prevalence | Both | Oman | T1D | 2.41 (2.34 to 2.47) | < 0.001 |
| Middle-aged and elderly age | Prevalence | Female | Oman | T1D | 2.41 (2.34 to 2.48) | < 0.001 |
| Middle-aged and elderly age | Prevalence | Male | Oman | T1D | 2.38 (2.29 to 2.47) | < 0.001 |
| Middle-aged and elderly age | Prevalence | Both | Oman | IBD | 0.19 (0.09 to 0.28) | < 0.001 |
| Middle-aged and elderly age | Prevalence | Female | Oman | IBD | 0.14 (0.03 to 0.25) | 0.013 |
| Middle-aged and elderly age | Prevalence | Male | Oman | IBD | 0.18 (0.04 to 0.32) | 0.011 |
| Middle-aged and elderly age | Prevalence | Both | Oman | MS | 1 (0.9 to 1.1) | < 0.001 |
| Middle-aged and elderly age | Prevalence | Female | Oman | MS | 0.99 (0.93 to 1.05) | < 0.001 |
| Middle-aged and elderly age | Prevalence | Male | Oman | MS | 1.24 | < 0.001 |

| | | | | | | |
|---|---|---|---|---|---|---|
| | | | | | (1.21 to 1.28) | |
| Middle-aged and elderly age | Prevalence | Both | Oman | Psoriasis | -1.52<br>(-1.55 to -1.5) | < 0.001 |
| Middle-aged and elderly age | Prevalence | Female | Oman | Psoriasis | -1.51<br>(-1.53 to -1.49) | < 0.001 |
| Middle-aged and elderly age | Prevalence | Male | Oman | Psoriasis | -1.52<br>(-1.54 to -1.49) | < 0.001 |
| Middle-aged and elderly age | Prevalence | Both | Oman | RHD | -0.37<br>(-0.48 to -0.27) | < 0.001 |
| Middle-aged and elderly age | Prevalence | Female | Oman | RHD | -0.62<br>(-0.71 to -0.53) | < 0.001 |
| Middle-aged and elderly age | Prevalence | Male | Oman | RHD | -0.12<br>(-0.21 to -0.02) | 0.015 |
| Middle-aged and elderly age | Prevalence | Both | Oman | RA | 1.07<br>(0.77 to 1.36) | < 0.001 |
| Middle-aged and elderly age | Prevalence | Female | Oman | RA | 1.24<br>(1.23 to 1.26) | < 0.001 |
| Middle-aged and elderly age | Prevalence | Male | Oman | RA | 1.22<br>(1.16 to 1.28) | < 0.001 |
| Middle-aged and elderly age | Prevalence | Both | Pakistan | AA | 0<br>(-0.01 to 0.01) | 0.913 |
| Middle-aged and elderly age | Prevalence | Female | Pakistan | AA | 0<br>(0 to 0) | < 0.001 |
| Middle-aged and elderly age | Prevalence | Male | Pakistan | AA | 0<br>(0 to 0) | 0.013 |
| Middle-aged and elderly age | Prevalence | Both | Pakistan | T1D | 0.64<br>(0.63 to 0.65) | < 0.001 |
| Middle-aged and elderly age | Prevalence | Female | Pakistan | T1D | 0.6<br>(0.58 to 0.62) | < 0.001 |
| Middle-aged and elderly age | Prevalence | Male | Pakistan | T1D | 0.67<br>(0.65 to 0.7) | < 0.001 |
| Middle-aged and elderly age | Prevalence | Both | Pakistan | IBD | 0.69<br>(0.59 to 0.8) | < 0.001 |

| | | | | | | |
|---|---|---|---|---|---|---|
| Middle-aged and elderly age | Prevalence | Female | Pakistan | IBD | 0.68<br>(0.61 to 0.75) | < 0.001 |
| Middle-aged and elderly age | Prevalence | Male | Pakistan | IBD | 0.68<br>(0.6 to 0.76) | < 0.001 |
| Middle-aged and elderly age | Prevalence | Both | Pakistan | MS | 0.31<br>(0.27 to 0.35) | < 0.001 |
| Middle-aged and elderly age | Prevalence | Female | Pakistan | MS | 0.24<br>(0.18 to 0.31) | < 0.001 |
| Middle-aged and elderly age | Prevalence | Male | Pakistan | MS | 0.36<br>(0.25 to 0.48) | < 0.001 |
| Middle-aged and elderly age | Prevalence | Both | Pakistan | Psoriasis | -0.62<br>(-0.64 to -0.61) | < 0.001 |
| Middle-aged and elderly age | Prevalence | Female | Pakistan | Psoriasis | -0.63<br>(-0.64 to -0.61) | < 0.001 |
| Middle-aged and elderly age | Prevalence | Male | Pakistan | Psoriasis | -0.62<br>(-0.63 to -0.61) | < 0.001 |
| Middle-aged and elderly age | Prevalence | Both | Pakistan | RHD | 0.25<br>(0.23 to 0.26) | < 0.001 |
| Middle-aged and elderly age | Prevalence | Female | Pakistan | RHD | 0.29<br>(0.25 to 0.33) | < 0.001 |
| Middle-aged and elderly age | Prevalence | Male | Pakistan | RHD | 0.2<br>(0.14 to 0.26) | < 0.001 |
| Middle-aged and elderly age | Prevalence | Both | Pakistan | RA | 0.04<br>(-0.01 to 0.09) | 0.142 |
| Middle-aged and elderly age | Prevalence | Female | Pakistan | RA | 0.07<br>(0.02 to 0.13) | 0.009 |
| Middle-aged and elderly age | Prevalence | Male | Pakistan | RA | -0.03<br>(-0.12 to 0.05) | 0.460 |
| Middle-aged and elderly age | Prevalence | Both | Palau | AA | -0.05<br>(-0.08 to -0.01) | 0.009 |
| Middle-aged and elderly age | Prevalence | Female | Palau | AA | 0<br>(0 to 0) | < 0.001 |
| Middle-aged and elderly age | Prevalence | Male | Palau | AA | 0 | < 0.001 |

| | | | | | | |
|---|---|---|---|---|---|---|
| | | | | | (0 to 0) | |
| Middle-aged and elderly age | Prevalence | Both | Palau | T1D | 1<br>(0.95 to 1.04) | < 0.001 |
| Middle-aged and elderly age | Prevalence | Female | Palau | T1D | 0.93<br>(0.9 to 0.96) | < 0.001 |
| Middle-aged and elderly age | Prevalence | Male | Palau | T1D | 0.98<br>(0.96 to 1.01) | < 0.001 |
| Middle-aged and elderly age | Prevalence | Both | Palau | IBD | -0.4<br>(-0.44 to -0.35) | < 0.001 |
| Middle-aged and elderly age | Prevalence | Female | Palau | IBD | -0.48<br>(-0.57 to -0.38) | < 0.001 |
| Middle-aged and elderly age | Prevalence | Male | Palau | IBD | -0.32<br>(-0.39 to -0.25) | < 0.001 |
| Middle-aged and elderly age | Prevalence | Both | Palau | MS | 0.15<br>(0.11 to 0.19) | < 0.001 |
| Middle-aged and elderly age | Prevalence | Female | Palau | MS | 0.2<br>(0.18 to 0.22) | < 0.001 |
| Middle-aged and elderly age | Prevalence | Male | Palau | MS | 0.17<br>(0.11 to 0.23) | < 0.001 |
| Middle-aged and elderly age | Prevalence | Both | Palau | Psoriasis | -0.4<br>(-0.45 to -0.35) | < 0.001 |
| Middle-aged and elderly age | Prevalence | Female | Palau | Psoriasis | -0.42<br>(-0.43 to -0.4) | < 0.001 |
| Middle-aged and elderly age | Prevalence | Male | Palau | Psoriasis | -0.44<br>(-0.47 to -0.41) | < 0.001 |
| Middle-aged and elderly age | Prevalence | Both | Palau | RHD | -0.61<br>(-0.69 to -0.53) | < 0.001 |
| Middle-aged and elderly age | Prevalence | Female | Palau | RHD | -0.84<br>(-0.95 to -0.72) | < 0.001 |
| Middle-aged and elderly age | Prevalence | Male | Palau | RHD | -0.33<br>(-0.36 to -0.3) | < 0.001 |
| Middle-aged and elderly age | Prevalence | Both | Palau | RA | 0.4<br>(0.24 to 0.57) | < 0.001 |

| | | | | | | |
|---|---|---|---|---|---|---|
| Middle-aged and elderly age | Prevalence | Female | Palau | RA | 0.49 (0.46 to 0.52) | < 0.001 |
| Middle-aged and elderly age | Prevalence | Male | Palau | RA | 0.41 (0.33 to 0.48) | < 0.001 |
| Middle-aged and elderly age | Prevalence | Both | Palestine | AA | -0.19 (-0.21 to -0.16) | < 0.001 |
| Middle-aged and elderly age | Prevalence | Female | Palestine | AA | 0 (0 to 0) | < 0.001 |
| Middle-aged and elderly age | Prevalence | Male | Palestine | AA | 0 (0 to 0) | 0.015 |
| Middle-aged and elderly age | Prevalence | Both | Palestine | T1D | 2.03 (1.93 to 2.13) | < 0.001 |
| Middle-aged and elderly age | Prevalence | Female | Palestine | T1D | 1.97 (1.94 to 2.01) | < 0.001 |
| Middle-aged and elderly age | Prevalence | Male | Palestine | T1D | 2 (1.86 to 2.13) | < 0.001 |
| Middle-aged and elderly age | Prevalence | Both | Palestine | IBD | 0.46 (0.41 to 0.51) | < 0.001 |
| Middle-aged and elderly age | Prevalence | Female | Palestine | IBD | 0.54 (0.48 to 0.6) | < 0.001 |
| Middle-aged and elderly age | Prevalence | Male | Palestine | IBD | 0.35 (0.28 to 0.42) | < 0.001 |
| Middle-aged and elderly age | Prevalence | Both | Palestine | MS | 0.52 (0.5 to 0.54) | < 0.001 |
| Middle-aged and elderly age | Prevalence | Female | Palestine | MS | 0.61 (0.59 to 0.63) | < 0.001 |
| Middle-aged and elderly age | Prevalence | Male | Palestine | MS | 0.82 (0.8 to 0.83) | < 0.001 |
| Middle-aged and elderly age | Prevalence | Both | Palestine | Psoriasis | -1.3 (-1.39 to -1.2) | < 0.001 |
| Middle-aged and elderly age | Prevalence | Female | Palestine | Psoriasis | -1.29 (-1.38 to -1.21) | < 0.001 |
| Middle-aged and elderly age | Prevalence | Male | Palestine | Psoriasis | -1.28 | < 0.001 |

| | | | | | | |
|---|---|---|---|---|---|---|
| | | | | | (-1.35 to -1.21) | |
| Middle-aged and elderly age | Prevalence | Both | Palestine | RHD | 0.04 (0.03 to 0.05) | < 0.001 |
| Middle-aged and elderly age | Prevalence | Female | Palestine | RHD | 0.14 (0.13 to 0.14) | < 0.001 |
| Middle-aged and elderly age | Prevalence | Male | Palestine | RHD | 0.07 (0.05 to 0.09) | < 0.001 |
| Middle-aged and elderly age | Prevalence | Both | Palestine | RA | 0.53 (0.49 to 0.57) | < 0.001 |
| Middle-aged and elderly age | Prevalence | Female | Palestine | RA | 0.8 (0.78 to 0.81) | < 0.001 |
| Middle-aged and elderly age | Prevalence | Male | Palestine | RA | 0.74 (0.68 to 0.79) | < 0.001 |
| Middle-aged and elderly age | Prevalence | Both | Panama | AA | 0.03 (0.03 to 0.04) | < 0.001 |
| Middle-aged and elderly age | Prevalence | Female | Panama | AA | 0 (0 to 0) | < 0.001 |
| Middle-aged and elderly age | Prevalence | Male | Panama | AA | 0 (0 to 0) | < 0.001 |
| Middle-aged and elderly age | Prevalence | Both | Panama | T1D | 0.8 (0.79 to 0.81) | < 0.001 |
| Middle-aged and elderly age | Prevalence | Female | Panama | T1D | 0.8 (0.78 to 0.82) | < 0.001 |
| Middle-aged and elderly age | Prevalence | Male | Panama | T1D | 0.81 (0.8 to 0.81) | < 0.001 |
| Middle-aged and elderly age | Prevalence | Both | Panama | IBD | 0.11 (0.09 to 0.12) | < 0.001 |
| Middle-aged and elderly age | Prevalence | Female | Panama | IBD | 0.13 (0.11 to 0.16) | < 0.001 |
| Middle-aged and elderly age | Prevalence | Male | Panama | IBD | 0.11 (0.05 to 0.17) | 0.001 |
| Middle-aged and elderly age | Prevalence | Both | Panama | MS | 0.51 (0.46 to 0.57) | < 0.001 |

| | | | | | | |
|---|---|---|---|---|---|---|
| Middle-aged and elderly age | Prevalence | Female | Panama | MS | 0.45 (0.41 to 0.49) | < 0.001 |
| Middle-aged and elderly age | Prevalence | Male | Panama | MS | 0.52 (0.45 to 0.59) | < 0.001 |
| Middle-aged and elderly age | Prevalence | Both | Panama | Psoriasis | -0.4 (-0.46 to -0.34) | < 0.001 |
| Middle-aged and elderly age | Prevalence | Female | Panama | Psoriasis | -0.41 (-0.46 to -0.35) | < 0.001 |
| Middle-aged and elderly age | Prevalence | Male | Panama | Psoriasis | -0.39 (-0.42 to -0.35) | < 0.001 |
| Middle-aged and elderly age | Prevalence | Both | Panama | RHD | 0.08 (0.04 to 0.12) | < 0.001 |
| Middle-aged and elderly age | Prevalence | Female | Panama | RHD | 0.06 (0.03 to 0.09) | < 0.001 |
| Middle-aged and elderly age | Prevalence | Male | Panama | RHD | 0.06 (0 to 0.13) | 0.047 |
| Middle-aged and elderly age | Prevalence | Both | Panama | RA | 0.77 (0.68 to 0.87) | < 0.001 |
| Middle-aged and elderly age | Prevalence | Female | Panama | RA | 0.67 (0.54 to 0.8) | < 0.001 |
| Middle-aged and elderly age | Prevalence | Male | Panama | RA | 0.84 (0.7 to 0.97) | < 0.001 |
| Middle-aged and elderly age | Prevalence | Both | Papua New Guinea | AA | 0 (-0.03 to 0.02) | 0.776 |
| Middle-aged and elderly age | Prevalence | Female | Papua New Guinea | AA | 0 (0 to 0) | < 0.001 |
| Middle-aged and elderly age | Prevalence | Male | Papua New Guinea | AA | 0 (0 to 0) | < 0.001 |
| Middle-aged and elderly age | Prevalence | Both | Papua New Guinea | T1D | 0.7 (0.69 to 0.7) | < 0.001 |
| Middle-aged and elderly age | Prevalence | Female | Papua New Guinea | T1D | 0.66 (0.66 to 0.67) | < 0.001 |
| Middle-aged and elderly age | Prevalence | Male | Papua New Guinea | T1D | 0.72 | < 0.001 |

| | | | | | | |
|---|---|---|---|---|---|---|
| | | | | | (0.7 to 0.74) | |
| Middle-aged and elderly age | Prevalence | Both | Papua New Guinea | IBD | -0.52<br>(-0.59 to -0.44) | < 0.001 |
| Middle-aged and elderly age | Prevalence | Female | Papua New Guinea | IBD | -0.61<br>(-0.72 to -0.51) | < 0.001 |
| Middle-aged and elderly age | Prevalence | Male | Papua New Guinea | IBD | -0.47<br>(-0.54 to -0.39) | < 0.001 |
| Middle-aged and elderly age | Prevalence | Both | Papua New Guinea | MS | 0.13<br>(0.09 to 0.17) | < 0.001 |
| Middle-aged and elderly age | Prevalence | Female | Papua New Guinea | MS | 0.16<br>(0.12 to 0.19) | < 0.001 |
| Middle-aged and elderly age | Prevalence | Male | Papua New Guinea | MS | 0.12<br>(0.07 to 0.17) | < 0.001 |
| Middle-aged and elderly age | Prevalence | Both | Papua New Guinea | Psoriasis | -0.38<br>(-0.41 to -0.35) | < 0.001 |
| Middle-aged and elderly age | Prevalence | Female | Papua New Guinea | Psoriasis | -0.36<br>(-0.38 to -0.34) | < 0.001 |
| Middle-aged and elderly age | Prevalence | Male | Papua New Guinea | Psoriasis | -0.37<br>(-0.43 to -0.32) | < 0.001 |
| Middle-aged and elderly age | Prevalence | Both | Papua New Guinea | RHD | 0.17<br>(0.16 to 0.18) | < 0.001 |
| Middle-aged and elderly age | Prevalence | Female | Papua New Guinea | RHD | 0.2<br>(0.14 to 0.26) | < 0.001 |
| Middle-aged and elderly age | Prevalence | Male | Papua New Guinea | RHD | 0.14<br>(0.12 to 0.17) | < 0.001 |
| Middle-aged and elderly age | Prevalence | Both | Papua New Guinea | RA | 0.25<br>(0.21 to 0.3) | < 0.001 |
| Middle-aged and elderly age | Prevalence | Female | Papua New Guinea | RA | 0.27<br>(0.25 to 0.29) | < 0.001 |
| Middle-aged and elderly age | Prevalence | Male | Papua New Guinea | RA | 0.23<br>(0.19 to 0.26) | < 0.001 |
| Middle-aged and elderly age | Prevalence | Both | Paraguay | AA | 0<br>(0 to 0.01) | 0.816 |

| | | | | | | |
|---|---|---|---|---|---|---|
| Middle-aged and elderly age | Prevalence | Female | Paraguay | AA | 0<br>(0 to 0) | < 0.001 |
| Middle-aged and elderly age | Prevalence | Male | Paraguay | AA | 0<br>(0 to 0) | < 0.001 |
| Middle-aged and elderly age | Prevalence | Both | Paraguay | T1D | 1.27<br>(1.23 to 1.31) | < 0.001 |
| Middle-aged and elderly age | Prevalence | Female | Paraguay | T1D | 1.22<br>(1.19 to 1.24) | < 0.001 |
| Middle-aged and elderly age | Prevalence | Male | Paraguay | T1D | 1.48<br>(1.39 to 1.56) | < 0.001 |
| Middle-aged and elderly age | Prevalence | Both | Paraguay | IBD | -0.31<br>(-0.39 to -0.22) | < 0.001 |
| Middle-aged and elderly age | Prevalence | Female | Paraguay | IBD | -0.23<br>(-0.31 to -0.16) | < 0.001 |
| Middle-aged and elderly age | Prevalence | Male | Paraguay | IBD | -0.37<br>(-0.46 to -0.27) | < 0.001 |
| Middle-aged and elderly age | Prevalence | Both | Paraguay | MS | 0.33<br>(0.29 to 0.37) | < 0.001 |
| Middle-aged and elderly age | Prevalence | Female | Paraguay | MS | 0.34<br>(0.28 to 0.4) | < 0.001 |
| Middle-aged and elderly age | Prevalence | Male | Paraguay | MS | 0.32<br>(0.29 to 0.35) | < 0.001 |
| Middle-aged and elderly age | Prevalence | Both | Paraguay | Psoriasis | -0.14<br>(-0.17 to -0.12) | < 0.001 |
| Middle-aged and elderly age | Prevalence | Female | Paraguay | Psoriasis | -0.14<br>(-0.16 to -0.12) | < 0.001 |
| Middle-aged and elderly age | Prevalence | Male | Paraguay | Psoriasis | -0.14<br>(-0.17 to -0.12) | < 0.001 |
| Middle-aged and elderly age | Prevalence | Both | Paraguay | RHD | 0.04<br>(0.01 to 0.06) | 0.002 |
| Middle-aged and elderly age | Prevalence | Female | Paraguay | RHD | 0.06<br>(0.03 to 0.08) | < 0.001 |
| Middle-aged and elderly age | Prevalence | Male | Paraguay | RHD | 0.02 | 0.043 |

| | | | | | | |
|---|---|---|---|---|---|---|
| | | | | | (0 to 0.04) | |
| Middle-aged and elderly age | Prevalence | Both | Paraguay | RA | 1.35 (1.27 to 1.44) | < 0.001 |
| Middle-aged and elderly age | Prevalence | Female | Paraguay | RA | 1.34 (1.25 to 1.44) | < 0.001 |
| Middle-aged and elderly age | Prevalence | Male | Paraguay | RA | 1.3 (1.24 to 1.37) | < 0.001 |
| Middle-aged and elderly age | Prevalence | Both | Peru | AA | 0.03 (0.03 to 0.03) | < 0.001 |
| Middle-aged and elderly age | Prevalence | Female | Peru | AA | 0 (0 to 0) | < 0.001 |
| Middle-aged and elderly age | Prevalence | Male | Peru | AA | 0 (0 to 0) | < 0.001 |
| Middle-aged and elderly age | Prevalence | Both | Peru | T1D | 0.84 (0.83 to 0.85) | < 0.001 |
| Middle-aged and elderly age | Prevalence | Female | Peru | T1D | 0.83 (0.8 to 0.87) | < 0.001 |
| Middle-aged and elderly age | Prevalence | Male | Peru | T1D | 0.83 (0.81 to 0.85) | < 0.001 |
| Middle-aged and elderly age | Prevalence | Both | Peru | IBD | -0.09 (-0.12 to -0.06) | < 0.001 |
| Middle-aged and elderly age | Prevalence | Female | Peru | IBD | -0.02 (-0.07 to 0.03) | 0.404 |
| Middle-aged and elderly age | Prevalence | Male | Peru | IBD | -0.14 (-0.17 to -0.1) | < 0.001 |
| Middle-aged and elderly age | Prevalence | Both | Peru | MS | 1.15 (1.12 to 1.19) | < 0.001 |
| Middle-aged and elderly age | Prevalence | Female | Peru | MS | 1.31 (1.26 to 1.36) | < 0.001 |
| Middle-aged and elderly age | Prevalence | Male | Peru | MS | 0.64 (0.59 to 0.7) | < 0.001 |
| Middle-aged and elderly age | Prevalence | Both | Peru | Psoriasis | -0.61 (-0.68 to -0.53) | < 0.001 |

| | | | | | | |
|---|---|---|---|---|---|---|
| Middle-aged and elderly age | Prevalence | Female | Peru | Psoriasis | -0.61<br>(-0.69 to -0.53) | < 0.001 |
| Middle-aged and elderly age | Prevalence | Male | Peru | Psoriasis | -0.61<br>(-0.69 to -0.54) | < 0.001 |
| Middle-aged and elderly age | Prevalence | Both | Peru | RHD | 0.17<br>(0.16 to 0.19) | < 0.001 |
| Middle-aged and elderly age | Prevalence | Female | Peru | RHD | 0.19<br>(0.17 to 0.2) | < 0.001 |
| Middle-aged and elderly age | Prevalence | Male | Peru | RHD | 0.13<br>(0.11 to 0.15) | < 0.001 |
| Middle-aged and elderly age | Prevalence | Both | Peru | RA | 1.69<br>(1.62 to 1.76) | < 0.001 |
| Middle-aged and elderly age | Prevalence | Female | Peru | RA | 1.71<br>(1.63 to 1.79) | < 0.001 |
| Middle-aged and elderly age | Prevalence | Male | Peru | RA | 1.46<br>(1.33 to 1.59) | < 0.001 |
| Middle-aged and elderly age | Prevalence | Both | Philippines | AA | -0.01<br>(-0.01 to -0.01) | < 0.001 |
| Middle-aged and elderly age | Prevalence | Female | Philippines | AA | 0<br>(NA to NA) | . |
| Middle-aged and elderly age | Prevalence | Male | Philippines | AA | 0<br>(0 to 0) | < 0.001 |
| Middle-aged and elderly age | Prevalence | Both | Philippines | T1D | 0.73<br>(0.72 to 0.75) | < 0.001 |
| Middle-aged and elderly age | Prevalence | Female | Philippines | T1D | 0.67<br>(0.64 to 0.7) | < 0.001 |
| Middle-aged and elderly age | Prevalence | Male | Philippines | T1D | 0.78<br>(0.75 to 0.82) | < 0.001 |
| Middle-aged and elderly age | Prevalence | Both | Philippines | IBD | 0.28<br>(0.14 to 0.41) | < 0.001 |
| Middle-aged and elderly age | Prevalence | Female | Philippines | IBD | 0.03<br>(-0.02 to 0.08) | 0.226 |
| Middle-aged and elderly age | Prevalence | Male | Philippines | IBD | 0.49 | < 0.001 |

| | | | | | | |
|---|---|---|---|---|---|---|
| | | | | | (0.34 to 0.65) | |
| Middle-aged and elderly age | Prevalence | Both | Philippines | MS | 0.26<br>(0.24 to 0.29) | < 0.001 |
| Middle-aged and elderly age | Prevalence | Female | Philippines | MS | 0.25<br>(0.21 to 0.29) | < 0.001 |
| Middle-aged and elderly age | Prevalence | Male | Philippines | MS | 0.31<br>(0.27 to 0.35) | < 0.001 |
| Middle-aged and elderly age | Prevalence | Both | Philippines | Psoriasis | -0.3<br>(-0.35 to -0.26) | < 0.001 |
| Middle-aged and elderly age | Prevalence | Female | Philippines | Psoriasis | -0.29<br>(-0.32 to -0.26) | < 0.001 |
| Middle-aged and elderly age | Prevalence | Male | Philippines | Psoriasis | -0.31<br>(-0.37 to -0.24) | < 0.001 |
| Middle-aged and elderly age | Prevalence | Both | Philippines | RHD | 0.09<br>(0.08 to 0.1) | < 0.001 |
| Middle-aged and elderly age | Prevalence | Female | Philippines | RHD | 0.14<br>(0.12 to 0.16) | < 0.001 |
| Middle-aged and elderly age | Prevalence | Male | Philippines | RHD | 0.06<br>(0.04 to 0.08) | < 0.001 |
| Middle-aged and elderly age | Prevalence | Both | Philippines | RA | -0.03<br>(-0.14 to 0.08) | 0.631 |
| Middle-aged and elderly age | Prevalence | Female | Philippines | RA | 0.08<br>(0.02 to 0.13) | 0.005 |
| Middle-aged and elderly age | Prevalence | Male | Philippines | RA | -0.24<br>(-0.39 to -0.08) | 0.003 |
| Middle-aged and elderly age | Prevalence | Both | Poland | AA | -0.04<br>(-0.04 to -0.03) | < 0.001 |
| Middle-aged and elderly age | Prevalence | Female | Poland | AA | 0<br>(0 to 0) | < 0.001 |
| Middle-aged and elderly age | Prevalence | Male | Poland | AA | 0<br>(0 to 0) | < 0.001 |
| Middle-aged and elderly age | Prevalence | Both | Poland | T1D | 1.75<br>(1.64 to 1.85) | < 0.001 |

| | | | | | | |
|---|---|---|---|---|---|---|
| Middle-aged and elderly age | Prevalence | Female | Poland | T1D | 1.7<br>(1.63 to 1.77) | < 0.001 |
| Middle-aged and elderly age | Prevalence | Male | Poland | T1D | 1.78<br>(1.72 to 1.83) | < 0.001 |
| Middle-aged and elderly age | Prevalence | Both | Poland | IBD | -4<br>(-4.13 to -3.87) | < 0.001 |
| Middle-aged and elderly age | Prevalence | Female | Poland | IBD | -4.07<br>(-4.21 to -3.93) | < 0.001 |
| Middle-aged and elderly age | Prevalence | Male | Poland | IBD | -3.93<br>(-4.06 to -3.79) | < 0.001 |
| Middle-aged and elderly age | Prevalence | Both | Poland | MS | 0.02<br>(-0.01 to 0.05) | 0.233 |
| Middle-aged and elderly age | Prevalence | Female | Poland | MS | 0.26<br>(0.18 to 0.34) | < 0.001 |
| Middle-aged and elderly age | Prevalence | Male | Poland | MS | -0.29<br>(-0.33 to -0.26) | < 0.001 |
| Middle-aged and elderly age | Prevalence | Both | Poland | Psoriasis | -0.38<br>(-0.43 to -0.34) | < 0.001 |
| Middle-aged and elderly age | Prevalence | Female | Poland | Psoriasis | -0.34<br>(-0.39 to -0.3) | < 0.001 |
| Middle-aged and elderly age | Prevalence | Male | Poland | Psoriasis | -0.41<br>(-0.43 to -0.4) | < 0.001 |
| Middle-aged and elderly age | Prevalence | Both | Poland | RHD | -3.03<br>(-3.26 to -2.81) | < 0.001 |
| Middle-aged and elderly age | Prevalence | Female | Poland | RHD | -3.05<br>(-3.4 to -2.7) | < 0.001 |
| Middle-aged and elderly age | Prevalence | Male | Poland | RHD | -3.01<br>(-3.16 to -2.85) | < 0.001 |
| Middle-aged and elderly age | Prevalence | Both | Poland | RA | -0.84<br>(-1.02 to -0.66) | < 0.001 |
| Middle-aged and elderly age | Prevalence | Female | Poland | RA | -0.78<br>(-0.85 to -0.72) | < 0.001 |
| Middle-aged and elderly age | Prevalence | Male | Poland | RA | -0.55 | < 0.001 |

| | | | | | | |
|---|---|---|---|---|---|---|
| | | | | | (-0.74 to -0.35) | |
| Middle-aged and elderly age | Prevalence | Both | Portugal | AA | -0.01<br>(-0.01 to 0) | < 0.001 |
| Middle-aged and elderly age | Prevalence | Female | Portugal | AA | 0<br>(0 to 0) | < 0.001 |
| Middle-aged and elderly age | Prevalence | Male | Portugal | AA | 0<br>(0 to 0) | < 0.001 |
| Middle-aged and elderly age | Prevalence | Both | Portugal | T1D | 2.6<br>(2.48 to 2.72) | < 0.001 |
| Middle-aged and elderly age | Prevalence | Female | Portugal | T1D | 2.5<br>(2.37 to 2.64) | < 0.001 |
| Middle-aged and elderly age | Prevalence | Male | Portugal | T1D | 2.68<br>(2.46 to 2.89) | < 0.001 |
| Middle-aged and elderly age | Prevalence | Both | Portugal | IBD | 0.71<br>(0.58 to 0.84) | < 0.001 |
| Middle-aged and elderly age | Prevalence | Female | Portugal | IBD | 0.68<br>(0.54 to 0.82) | < 0.001 |
| Middle-aged and elderly age | Prevalence | Male | Portugal | IBD | 0.76<br>(0.66 to 0.86) | < 0.001 |
| Middle-aged and elderly age | Prevalence | Both | Portugal | MS | 0.06<br>(0.05 to 0.07) | < 0.001 |
| Middle-aged and elderly age | Prevalence | Female | Portugal | MS | 0.18<br>(0.17 to 0.2) | < 0.001 |
| Middle-aged and elderly age | Prevalence | Male | Portugal | MS | -0.14<br>(-0.18 to -0.1) | < 0.001 |
| Middle-aged and elderly age | Prevalence | Both | Portugal | Psoriasis | -0.53<br>(-0.54 to -0.52) | < 0.001 |
| Middle-aged and elderly age | Prevalence | Female | Portugal | Psoriasis | -0.53<br>(-0.54 to -0.52) | < 0.001 |
| Middle-aged and elderly age | Prevalence | Male | Portugal | Psoriasis | -0.53<br>(-0.54 to -0.52) | < 0.001 |
| Middle-aged and elderly age | Prevalence | Both | Portugal | RHD | -2.8<br>(-2.89 to -2.71) | < 0.001 |

| | | | | | | |
|---|---|---|---|---|---|---|
| Middle-aged and elderly age | Prevalence | Female | Portugal | RHD | -3.37<br>(-3.49 to -3.26) | < 0.001 |
| Middle-aged and elderly age | Prevalence | Male | Portugal | RHD | -1.93<br>(-2.03 to -1.84) | < 0.001 |
| Middle-aged and elderly age | Prevalence | Both | Portugal | RA | 0.82<br>(0.74 to 0.9) | < 0.001 |
| Middle-aged and elderly age | Prevalence | Female | Portugal | RA | 0.9<br>(0.76 to 1.04) | < 0.001 |
| Middle-aged and elderly age | Prevalence | Male | Portugal | RA | 0.67<br>(0.61 to 0.72) | < 0.001 |
| Middle-aged and elderly age | Prevalence | Both | Puerto Rico | AA | 0.01<br>(0 to 0.01) | 0.056 |
| Middle-aged and elderly age | Prevalence | Female | Puerto Rico | AA | 0<br>(0 to 0) | < 0.001 |
| Middle-aged and elderly age | Prevalence | Male | Puerto Rico | AA | 0<br>(0 to 0) | < 0.001 |
| Middle-aged and elderly age | Prevalence | Both | Puerto Rico | T1D | 0.58<br>(0.52 to 0.65) | < 0.001 |
| Middle-aged and elderly age | Prevalence | Female | Puerto Rico | T1D | 0.8<br>(0.74 to 0.85) | < 0.001 |
| Middle-aged and elderly age | Prevalence | Male | Puerto Rico | T1D | 0.4<br>(0.31 to 0.48) | < 0.001 |
| Middle-aged and elderly age | Prevalence | Both | Puerto Rico | IBD | 0.19<br>(0.17 to 0.22) | < 0.001 |
| Middle-aged and elderly age | Prevalence | Female | Puerto Rico | IBD | 0.21<br>(0.17 to 0.25) | < 0.001 |
| Middle-aged and elderly age | Prevalence | Male | Puerto Rico | IBD | 0.18<br>(0.16 to 0.2) | < 0.001 |
| Middle-aged and elderly age | Prevalence | Both | Puerto Rico | MS | 0.68<br>(0.64 to 0.71) | < 0.001 |
| Middle-aged and elderly age | Prevalence | Female | Puerto Rico | MS | 0.61<br>(0.56 to 0.66) | < 0.001 |
| Middle-aged and elderly age | Prevalence | Male | Puerto Rico | MS | 0.84 | < 0.001 |

| | | | | | | |
|---|---|---|---|---|---|---|
| | | | | | (0.8 to 0.88) | |
| Middle-aged and elderly age | Prevalence | Both | Puerto Rico | Psoriasis | -0.39<br>(-0.39 to -0.38) | < 0.001 |
| Middle-aged and elderly age | Prevalence | Female | Puerto Rico | Psoriasis | -0.39<br>(-0.4 to -0.38) | < 0.001 |
| Middle-aged and elderly age | Prevalence | Male | Puerto Rico | Psoriasis | -0.39<br>(-0.41 to -0.37) | < 0.001 |
| Middle-aged and elderly age | Prevalence | Both | Puerto Rico | RHD | -0.61<br>(-0.67 to -0.54) | < 0.001 |
| Middle-aged and elderly age | Prevalence | Female | Puerto Rico | RHD | -0.87<br>(-0.97 to -0.78) | < 0.001 |
| Middle-aged and elderly age | Prevalence | Male | Puerto Rico | RHD | -0.09<br>(-0.14 to -0.04) | 0.001 |
| Middle-aged and elderly age | Prevalence | Both | Puerto Rico | RA | 1.07<br>(1.01 to 1.13) | < 0.001 |
| Middle-aged and elderly age | Prevalence | Female | Puerto Rico | RA | 1.08<br>(1.03 to 1.13) | < 0.001 |
| Middle-aged and elderly age | Prevalence | Male | Puerto Rico | RA | 1<br>(0.95 to 1.05) | < 0.001 |
| Middle-aged and elderly age | Prevalence | Both | Qatar | AA | -0.13<br>(-0.17 to -0.09) | < 0.001 |
| Middle-aged and elderly age | Prevalence | Female | Qatar | AA | 0<br>(0 to 0) | < 0.001 |
| Middle-aged and elderly age | Prevalence | Male | Qatar | AA | 0<br>(0 to 0) | 0.015 |
| Middle-aged and elderly age | Prevalence | Both | Qatar | T1D | 2.47<br>(2.4 to 2.55) | < 0.001 |
| Middle-aged and elderly age | Prevalence | Female | Qatar | T1D | 2.44<br>(2.41 to 2.48) | < 0.001 |
| Middle-aged and elderly age | Prevalence | Male | Qatar | T1D | 2.46<br>(2.39 to 2.53) | < 0.001 |
| Middle-aged and elderly age | Prevalence | Both | Qatar | IBD | 0.54<br>(0.42 to 0.66) | < 0.001 |

| | | | | | | |
|---|---|---|---|---|---|---|
| Middle-aged and elderly age | Prevalence | Female | Qatar | IBD | 0.55<br>(0.41 to 0.68) | < 0.001 |
| Middle-aged and elderly age | Prevalence | Male | Qatar | IBD | 0.52<br>(0.39 to 0.65) | < 0.001 |
| Middle-aged and elderly age | Prevalence | Both | Qatar | MS | 1.57<br>(1.32 to 1.83) | < 0.001 |
| Middle-aged and elderly age | Prevalence | Female | Qatar | MS | 1.62<br>(1.55 to 1.7) | < 0.001 |
| Middle-aged and elderly age | Prevalence | Male | Qatar | MS | 1.86<br>(1.78 to 1.93) | < 0.001 |
| Middle-aged and elderly age | Prevalence | Both | Qatar | Psoriasis | -0.96<br>(-0.97 to -0.95) | < 0.001 |
| Middle-aged and elderly age | Prevalence | Female | Qatar | Psoriasis | -0.96<br>(-0.98 to -0.94) | < 0.001 |
| Middle-aged and elderly age | Prevalence | Male | Qatar | Psoriasis | -0.95<br>(-0.96 to -0.94) | < 0.001 |
| Middle-aged and elderly age | Prevalence | Both | Qatar | RHD | -1.74<br>(-1.89 to -1.58) | < 0.001 |
| Middle-aged and elderly age | Prevalence | Female | Qatar | RHD | -1.48<br>(-1.53 to -1.43) | < 0.001 |
| Middle-aged and elderly age | Prevalence | Male | Qatar | RHD | -1.76<br>(-1.91 to -1.61) | < 0.001 |
| Middle-aged and elderly age | Prevalence | Both | Qatar | RA | 1.08<br>(1 to 1.16) | < 0.001 |
| Middle-aged and elderly age | Prevalence | Female | Qatar | RA | 1.47<br>(1.4 to 1.53) | < 0.001 |
| Middle-aged and elderly age | Prevalence | Male | Qatar | RA | 1.1<br>(1.03 to 1.18) | < 0.001 |
| Middle-aged and elderly age | Prevalence | Both | Republic of Korea | AA | -0.09<br>(-0.1 to -0.08) | < 0.001 |
| Middle-aged and elderly age | Prevalence | Female | Republic of Korea | AA | 0<br>(0 to 0) | < 0.001 |
| Middle-aged and elderly age | Prevalence | Male | Republic of Korea | AA | 0 | 0.002 |

| | | | | | | |
|---|---|---|---|---|---|---|
| | | | | | (0 to 0) | |
| Middle-aged and elderly age | Prevalence | Both | Republic of Korea | T1D | 0.54<br>(0.46 to 0.61) | < 0.001 |
| Middle-aged and elderly age | Prevalence | Female | Republic of Korea | T1D | 0.53<br>(0.46 to 0.61) | < 0.001 |
| Middle-aged and elderly age | Prevalence | Male | Republic of Korea | T1D | 0.52<br>(0.44 to 0.6) | < 0.001 |
| Middle-aged and elderly age | Prevalence | Both | Republic of Korea | IBD | 1.52<br>(1.31 to 1.73) | < 0.001 |
| Middle-aged and elderly age | Prevalence | Female | Republic of Korea | IBD | 1.48<br>(1.3 to 1.67) | < 0.001 |
| Middle-aged and elderly age | Prevalence | Male | Republic of Korea | IBD | 1.54<br>(1.32 to 1.76) | < 0.001 |
| Middle-aged and elderly age | Prevalence | Both | Republic of Korea | MS | 0.14<br>(0.12 to 0.15) | < 0.001 |
| Middle-aged and elderly age | Prevalence | Female | Republic of Korea | MS | 0.2<br>(0.17 to 0.23) | < 0.001 |
| Middle-aged and elderly age | Prevalence | Male | Republic of Korea | MS | 0.13<br>(0.13 to 0.14) | < 0.001 |
| Middle-aged and elderly age | Prevalence | Both | Republic of Korea | Psoriasis | -0.47<br>(-0.49 to -0.44) | < 0.001 |
| Middle-aged and elderly age | Prevalence | Female | Republic of Korea | Psoriasis | -0.54<br>(-0.56 to -0.52) | < 0.001 |
| Middle-aged and elderly age | Prevalence | Male | Republic of Korea | Psoriasis | -0.41<br>(-0.43 to -0.39) | < 0.001 |
| Middle-aged and elderly age | Prevalence | Both | Republic of Korea | RHD | -0.95<br>(-1.04 to -0.87) | < 0.001 |
| Middle-aged and elderly age | Prevalence | Female | Republic of Korea | RHD | -1.21<br>(-1.35 to -1.07) | < 0.001 |
| Middle-aged and elderly age | Prevalence | Male | Republic of Korea | RHD | -0.36<br>(-0.42 to -0.3) | < 0.001 |
| Middle-aged and elderly age | Prevalence | Both | Republic of Korea | RA | 0.75<br>(0.67 to 0.84) | < 0.001 |

| | | | | | | |
|---|---|---|---|---|---|---|
| Middle-aged and elderly age | Prevalence | Female | Republic of Korea | RA | 0.88 (0.83 to 0.93) | < 0.001 |
| Middle-aged and elderly age | Prevalence | Male | Republic of Korea | RA | 0.71 (0.64 to 0.78) | < 0.001 |
| Middle-aged and elderly age | Prevalence | Both | Republic of Moldova | AA | -0.01 (-0.02 to 0) | 0.053 |
| Middle-aged and elderly age | Prevalence | Female | Republic of Moldova | AA | 0 (0 to 0) | < 0.001 |
| Middle-aged and elderly age | Prevalence | Male | Republic of Moldova | AA | 0 (0 to 0) | < 0.001 |
| Middle-aged and elderly age | Prevalence | Both | Republic of Moldova | T1D | 1.42 (1.4 to 1.44) | < 0.001 |
| Middle-aged and elderly age | Prevalence | Female | Republic of Moldova | T1D | 1.43 (1.4 to 1.46) | < 0.001 |
| Middle-aged and elderly age | Prevalence | Male | Republic of Moldova | T1D | 1.4 (1.35 to 1.46) | < 0.001 |
| Middle-aged and elderly age | Prevalence | Both | Republic of Moldova | IBD | -0.31 (-0.49 to -0.12) | 0.001 |
| Middle-aged and elderly age | Prevalence | Female | Republic of Moldova | IBD | -0.39 (-0.58 to -0.2) | < 0.001 |
| Middle-aged and elderly age | Prevalence | Male | Republic of Moldova | IBD | -0.29 (-0.48 to -0.09) | 0.004 |
| Middle-aged and elderly age | Prevalence | Both | Republic of Moldova | MS | 0.35 (0.2 to 0.5) | < 0.001 |
| Middle-aged and elderly age | Prevalence | Female | Republic of Moldova | MS | 0.58 (0.39 to 0.76) | < 0.001 |
| Middle-aged and elderly age | Prevalence | Male | Republic of Moldova | MS | 0.07 (0.03 to 0.1) | < 0.001 |
| Middle-aged and elderly age | Prevalence | Both | Republic of Moldova | Psoriasis | -0.66 (-0.69 to -0.64) | < 0.001 |
| Middle-aged and elderly age | Prevalence | Female | Republic of Moldova | Psoriasis | -0.66 (-0.68 to -0.64) | < 0.001 |
| Middle-aged and elderly age | Prevalence | Male | Republic of Moldova | Psoriasis | -0.67 | < 0.001 |

| | | | | | | |
|---|---|---|---|---|---|---|
| | | | | | (-0.7 to -0.65) | |
| Middle-aged and elderly age | Prevalence | Both | Republic of Moldova | RHD | -2.96 (-3.08 to -2.84) | < 0.001 |
| Middle-aged and elderly age | Prevalence | Female | Republic of Moldova | RHD | -3.02 (-3.16 to -2.88) | < 0.001 |
| Middle-aged and elderly age | Prevalence | Male | Republic of Moldova | RHD | -2.84 (-3.03 to -2.64) | < 0.001 |
| Middle-aged and elderly age | Prevalence | Both | Republic of Moldova | RA | 0.76 (0.7 to 0.83) | < 0.001 |
| Middle-aged and elderly age | Prevalence | Female | Republic of Moldova | RA | 0.8 (0.73 to 0.87) | < 0.001 |
| Middle-aged and elderly age | Prevalence | Male | Republic of Moldova | RA | 0.71 (0.65 to 0.77) | < 0.001 |
| Middle-aged and elderly age | Prevalence | Both | Romania | AA | -0.03 (-0.03 to -0.03) | < 0.001 |
| Middle-aged and elderly age | Prevalence | Female | Romania | AA | 0 (0 to 0) | < 0.001 |
| Middle-aged and elderly age | Prevalence | Male | Romania | AA | 0 (0 to 0) | < 0.001 |
| Middle-aged and elderly age | Prevalence | Both | Romania | T1D | 2.06 (2 to 2.13) | < 0.001 |
| Middle-aged and elderly age | Prevalence | Female | Romania | T1D | 1.92 (1.8 to 2.03) | < 0.001 |
| Middle-aged and elderly age | Prevalence | Male | Romania | T1D | 2.23 (2.17 to 2.29) | < 0.001 |
| Middle-aged and elderly age | Prevalence | Both | Romania | IBD | -0.28 (-0.49 to -0.07) | 0.010 |
| Middle-aged and elderly age | Prevalence | Female | Romania | IBD | -0.18 (-0.31 to -0.05) | 0.008 |
| Middle-aged and elderly age | Prevalence | Male | Romania | IBD | -0.39 (-0.6 to -0.18) | < 0.001 |
| Middle-aged and elderly age | Prevalence | Both | Romania | MS | 0.07 (-0.05 to 0.2) | 0.261 |

| | | | | | | |
|---|---|---|---|---|---|---|
| Middle-aged and elderly age | Prevalence | Female | Romania | MS | 0.13<br>(0 to 0.26) | 0.057 |
| Middle-aged and elderly age | Prevalence | Male | Romania | MS | -0.02<br>(-0.12 to 0.08) | 0.710 |
| Middle-aged and elderly age | Prevalence | Both | Romania | Psoriasis | -0.85<br>(-0.91 to -0.79) | < 0.001 |
| Middle-aged and elderly age | Prevalence | Female | Romania | Psoriasis | -0.88<br>(-0.98 to -0.78) | < 0.001 |
| Middle-aged and elderly age | Prevalence | Male | Romania | Psoriasis | -0.82<br>(-0.86 to -0.79) | < 0.001 |
| Middle-aged and elderly age | Prevalence | Both | Romania | RHD | -2.67<br>(-2.94 to -2.4) | < 0.001 |
| Middle-aged and elderly age | Prevalence | Female | Romania | RHD | -3.01<br>(-3.42 to -2.61) | < 0.001 |
| Middle-aged and elderly age | Prevalence | Male | Romania | RHD | -2.2<br>(-2.38 to -2.02) | < 0.001 |
| Middle-aged and elderly age | Prevalence | Both | Romania | RA | 0.87<br>(0.79 to 0.94) | < 0.001 |
| Middle-aged and elderly age | Prevalence | Female | Romania | RA | 0.95<br>(0.87 to 1.03) | < 0.001 |
| Middle-aged and elderly age | Prevalence | Male | Romania | RA | 0.8<br>(0.73 to 0.88) | < 0.001 |
| Middle-aged and elderly age | Prevalence | Both | Russian Federation | AA | 0<br>(-0.01 to 0) | 0.423 |
| Middle-aged and elderly age | Prevalence | Female | Russian Federation | AA | 0<br>(0 to 0) | < 0.001 |
| Middle-aged and elderly age | Prevalence | Male | Russian Federation | AA | 0<br>(0 to 0) | < 0.001 |
| Middle-aged and elderly age | Prevalence | Both | Russian Federation | T1D | 1.25<br>(1.08 to 1.43) | < 0.001 |
| Middle-aged and elderly age | Prevalence | Female | Russian Federation | T1D | 1.33<br>(1.2 to 1.47) | < 0.001 |
| Middle-aged and elderly age | Prevalence | Male | Russian Federation | T1D | 1.14 | < 0.001 |

| | | | | | | |
|---|---|---|---|---|---|---|
| | | | | | (0.91 to 1.37) | |
| Middle-aged and elderly age | Prevalence | Both | Russian Federation | IBD | -0.45 (-0.51 to -0.39) | < 0.001 |
| Middle-aged and elderly age | Prevalence | Female | Russian Federation | IBD | -0.5 (-0.54 to -0.47) | < 0.001 |
| Middle-aged and elderly age | Prevalence | Male | Russian Federation | IBD | -0.39 (-0.49 to -0.29) | < 0.001 |
| Middle-aged and elderly age | Prevalence | Both | Russian Federation | MS | -0.45 (-0.53 to -0.38) | < 0.001 |
| Middle-aged and elderly age | Prevalence | Female | Russian Federation | MS | -0.38 (-0.46 to -0.3) | < 0.001 |
| Middle-aged and elderly age | Prevalence | Male | Russian Federation | MS | -0.57 (-0.63 to -0.51) | < 0.001 |
| Middle-aged and elderly age | Prevalence | Both | Russian Federation | Psoriasis | -0.39 (-0.41 to -0.38) | < 0.001 |
| Middle-aged and elderly age | Prevalence | Female | Russian Federation | Psoriasis | -0.39 (-0.41 to -0.37) | < 0.001 |
| Middle-aged and elderly age | Prevalence | Male | Russian Federation | Psoriasis | -0.39 (-0.41 to -0.38) | < 0.001 |
| Middle-aged and elderly age | Prevalence | Both | Russian Federation | RHD | -2.55 (-2.65 to -2.46) | < 0.001 |
| Middle-aged and elderly age | Prevalence | Female | Russian Federation | RHD | -2.51 (-2.6 to -2.42) | < 0.001 |
| Middle-aged and elderly age | Prevalence | Male | Russian Federation | RHD | -2.62 (-2.75 to -2.49) | < 0.001 |
| Middle-aged and elderly age | Prevalence | Both | Russian Federation | RA | 0.09 (0.05 to 0.13) | < 0.001 |
| Middle-aged and elderly age | Prevalence | Female | Russian Federation | RA | 0.08 (0.05 to 0.11) | < 0.001 |
| Middle-aged and elderly age | Prevalence | Male | Russian Federation | RA | 0.12 (0.08 to 0.15) | < 0.001 |
| Middle-aged and elderly age | Prevalence | Both | Rwanda | AA | -0.02 (-0.03 to 0) | 0.008 |

| | | | | | | |
|---|---|---|---|---|---|---|
| Middle-aged and elderly age | Prevalence | Female | Rwanda | AA | 0 (0 to 0) | 1.000 |
| Middle-aged and elderly age | Prevalence | Male | Rwanda | AA | 0 (0 to 0) | < 0.001 |
| Middle-aged and elderly age | Prevalence | Both | Rwanda | T1D | 0.41 (0.37 to 0.44) | < 0.001 |
| Middle-aged and elderly age | Prevalence | Female | Rwanda | T1D | 0.41 (0.38 to 0.44) | < 0.001 |
| Middle-aged and elderly age | Prevalence | Male | Rwanda | T1D | 0.41 (0.39 to 0.43) | < 0.001 |
| Middle-aged and elderly age | Prevalence | Both | Rwanda | IBD | 0.4 (0.28 to 0.52) | < 0.001 |
| Middle-aged and elderly age | Prevalence | Female | Rwanda | IBD | 0.19 (0.06 to 0.32) | 0.003 |
| Middle-aged and elderly age | Prevalence | Male | Rwanda | IBD | 0.68 (0.56 to 0.8) | < 0.001 |
| Middle-aged and elderly age | Prevalence | Both | Rwanda | MS | 0.21 (0.18 to 0.24) | < 0.001 |
| Middle-aged and elderly age | Prevalence | Female | Rwanda | MS | 0.21 (0.18 to 0.25) | < 0.001 |
| Middle-aged and elderly age | Prevalence | Male | Rwanda | MS | 0.29 (0.26 to 0.32) | < 0.001 |
| Middle-aged and elderly age | Prevalence | Both | Rwanda | Psoriasis | -0.42 (-0.44 to -0.39) | < 0.001 |
| Middle-aged and elderly age | Prevalence | Female | Rwanda | Psoriasis | -0.44 (-0.48 to -0.39) | < 0.001 |
| Middle-aged and elderly age | Prevalence | Male | Rwanda | Psoriasis | -0.39 (-0.46 to -0.33) | < 0.001 |
| Middle-aged and elderly age | Prevalence | Both | Rwanda | RHD | 0.04 (0.03 to 0.06) | < 0.001 |
| Middle-aged and elderly age | Prevalence | Female | Rwanda | RHD | 0.09 (0.08 to 0.11) | < 0.001 |
| Middle-aged and elderly age | Prevalence | Male | Rwanda | RHD | -0.01 | 0.417 |

| | | | | | | |
|---|---|---|---|---|---|---|
| | | | | | (-0.04 to 0.02) | |
| Middle-aged and elderly age | Prevalence | Both | Rwanda | RA | 1.04 (0.83 to 1.24) | < 0.001 |
| Middle-aged and elderly age | Prevalence | Female | Rwanda | RA | 0.97 (0.83 to 1.12) | < 0.001 |
| Middle-aged and elderly age | Prevalence | Male | Rwanda | RA | 1.02 (0.86 to 1.18) | < 0.001 |
| Middle-aged and elderly age | Prevalence | Both | Saint Kitts and Nevis | AA | -0.13 (-0.14 to -0.11) | < 0.001 |
| Middle-aged and elderly age | Prevalence | Female | Saint Kitts and Nevis | AA | 0 (0 to 0) | < 0.001 |
| Middle-aged and elderly age | Prevalence | Male | Saint Kitts and Nevis | AA | 0 (0 to 0) | < 0.001 |
| Middle-aged and elderly age | Prevalence | Both | Saint Kitts and Nevis | T1D | 1.27 (1.26 to 1.28) | < 0.001 |
| Middle-aged and elderly age | Prevalence | Female | Saint Kitts and Nevis | T1D | 1.17 (1.15 to 1.18) | < 0.001 |
| Middle-aged and elderly age | Prevalence | Male | Saint Kitts and Nevis | T1D | 1.31 (1.3 to 1.33) | < 0.001 |
| Middle-aged and elderly age | Prevalence | Both | Saint Kitts and Nevis | IBD | 0.16 (0.13 to 0.19) | < 0.001 |
| Middle-aged and elderly age | Prevalence | Female | Saint Kitts and Nevis | IBD | 0.21 (0.17 to 0.25) | < 0.001 |
| Middle-aged and elderly age | Prevalence | Male | Saint Kitts and Nevis | IBD | 0.11 (0.07 to 0.15) | < 0.001 |
| Middle-aged and elderly age | Prevalence | Both | Saint Kitts and Nevis | MS | 0.37 (0.3 to 0.43) | < 0.001 |
| Middle-aged and elderly age | Prevalence | Female | Saint Kitts and Nevis | MS | 0.28 (0.19 to 0.37) | < 0.001 |
| Middle-aged and elderly age | Prevalence | Male | Saint Kitts and Nevis | MS | 0.73 (0.62 to 0.84) | < 0.001 |
| Middle-aged and elderly age | Prevalence | Both | Saint Kitts and Nevis | Psoriasis | -0.48 (-0.49 to -0.47) | < 0.001 |

| | | | | | | |
|---|---|---|---|---|---|---|
| Middle-aged and elderly age | Prevalence | Female | Saint Kitts and Nevis | Psoriasis | -0.46 (-0.47 to -0.46) | < 0.001 |
| Middle-aged and elderly age | Prevalence | Male | Saint Kitts and Nevis | Psoriasis | -0.47 (-0.48 to -0.46) | < 0.001 |
| Middle-aged and elderly age | Prevalence | Both | Saint Kitts and Nevis | RHD | -2.13 (-2.24 to -2.02) | < 0.001 |
| Middle-aged and elderly age | Prevalence | Female | Saint Kitts and Nevis | RHD | -2.32 (-2.42 to -2.22) | < 0.001 |
| Middle-aged and elderly age | Prevalence | Male | Saint Kitts and Nevis | RHD | -1.35 (-1.41 to -1.3) | < 0.001 |
| Middle-aged and elderly age | Prevalence | Both | Saint Kitts and Nevis | RA | 0.51 (0.39 to 0.63) | < 0.001 |
| Middle-aged and elderly age | Prevalence | Female | Saint Kitts and Nevis | RA | 0.72 (0.61 to 0.82) | < 0.001 |
| Middle-aged and elderly age | Prevalence | Male | Saint Kitts and Nevis | RA | 0.89 (0.87 to 0.91) | < 0.001 |
| Middle-aged and elderly age | Prevalence | Both | Saint Lucia | AA | -0.08 (-0.09 to -0.06) | < 0.001 |
| Middle-aged and elderly age | Prevalence | Female | Saint Lucia | AA | 0 (0 to 0) | < 0.001 |
| Middle-aged and elderly age | Prevalence | Male | Saint Lucia | AA | 0 (0 to 0) | < 0.001 |
| Middle-aged and elderly age | Prevalence | Both | Saint Lucia | T1D | 1.4 (1.38 to 1.41) | < 0.001 |
| Middle-aged and elderly age | Prevalence | Female | Saint Lucia | T1D | 1.3 (1.29 to 1.31) | < 0.001 |
| Middle-aged and elderly age | Prevalence | Male | Saint Lucia | T1D | 1.46 (1.44 to 1.49) | < 0.001 |
| Middle-aged and elderly age | Prevalence | Both | Saint Lucia | IBD | 0.34 (0.32 to 0.35) | < 0.001 |
| Middle-aged and elderly age | Prevalence | Female | Saint Lucia | IBD | 0.34 (0.32 to 0.36) | < 0.001 |
| Middle-aged and elderly age | Prevalence | Male | Saint Lucia | IBD | 0.31 | < 0.001 |

| | | | | | | |
|---|---|---|---|---|---|---|
| | | | | | (0.26 to 0.36) | |
| Middle-aged and elderly age | Prevalence | Both | Saint Lucia | MS | 0.48<br>(0.44 to 0.51) | < 0.001 |
| Middle-aged and elderly age | Prevalence | Female | Saint Lucia | MS | 0.57<br>(0.53 to 0.62) | < 0.001 |
| Middle-aged and elderly age | Prevalence | Male | Saint Lucia | MS | 0.52<br>(0.43 to 0.6) | < 0.001 |
| Middle-aged and elderly age | Prevalence | Both | Saint Lucia | Psoriasis | -0.49<br>(-0.51 to -0.47) | < 0.001 |
| Middle-aged and elderly age | Prevalence | Female | Saint Lucia | Psoriasis | -0.48<br>(-0.5 to -0.47) | < 0.001 |
| Middle-aged and elderly age | Prevalence | Male | Saint Lucia | Psoriasis | -0.49<br>(-0.52 to -0.47) | < 0.001 |
| Middle-aged and elderly age | Prevalence | Both | Saint Lucia | RHD | 0<br>(-0.01 to 0.01) | 0.991 |
| Middle-aged and elderly age | Prevalence | Female | Saint Lucia | RHD | 0.07<br>(0.05 to 0.08) | < 0.001 |
| Middle-aged and elderly age | Prevalence | Male | Saint Lucia | RHD | 0.01<br>(-0.01 to 0.03) | 0.267 |
| Middle-aged and elderly age | Prevalence | Both | Saint Lucia | RA | 0.78<br>(0.72 to 0.84) | < 0.001 |
| Middle-aged and elderly age | Prevalence | Female | Saint Lucia | RA | 0.89<br>(0.85 to 0.93) | < 0.001 |
| Middle-aged and elderly age | Prevalence | Male | Saint Lucia | RA | 1.02<br>(0.96 to 1.08) | < 0.001 |
| Middle-aged and elderly age | Prevalence | Both | Saint Vincent and the Grenadines | AA | -0.11<br>(-0.12 to -0.1) | < 0.001 |
| Middle-aged and elderly age | Prevalence | Female | Saint Vincent and the Grenadines | AA | 0<br>(0 to 0) | < 0.001 |
| Middle-aged and elderly age | Prevalence | Male | Saint Vincent and the Grenadines | AA | 0<br>(0 to 0) | < 0.001 |
| Middle-aged and elderly age | Prevalence | Both | Saint Vincent and the Grenadines | T1D | 1.39<br>(1.39 to 1.4) | < 0.001 |

| | | | | | | |
|---|---|---|---|---|---|---|
| Middle-aged and elderly age | Prevalence | Female | Saint Vincent and the Grenadines | T1D | 1.28 (1.27 to 1.29) | < 0.001 |
| Middle-aged and elderly age | Prevalence | Male | Saint Vincent and the Grenadines | T1D | 1.44 (1.43 to 1.44) | < 0.001 |
| Middle-aged and elderly age | Prevalence | Both | Saint Vincent and the Grenadines | IBD | 0.11 (0.07 to 0.15) | < 0.001 |
| Middle-aged and elderly age | Prevalence | Female | Saint Vincent and the Grenadines | IBD | 0.14 (0.06 to 0.22) | < 0.001 |
| Middle-aged and elderly age | Prevalence | Male | Saint Vincent and the Grenadines | IBD | 0.08 (0.02 to 0.15) | 0.014 |
| Middle-aged and elderly age | Prevalence | Both | Saint Vincent and the Grenadines | MS | 0.45 (0.42 to 0.49) | < 0.001 |
| Middle-aged and elderly age | Prevalence | Female | Saint Vincent and the Grenadines | MS | 0.71 (0.66 to 0.77) | < 0.001 |
| Middle-aged and elderly age | Prevalence | Male | Saint Vincent and the Grenadines | MS | 0.33 (0.31 to 0.35) | < 0.001 |
| Middle-aged and elderly age | Prevalence | Both | Saint Vincent and the Grenadines | Psoriasis | -0.5 (-0.51 to -0.48) | < 0.001 |
| Middle-aged and elderly age | Prevalence | Female | Saint Vincent and the Grenadines | Psoriasis | -0.49 (-0.5 to -0.47) | < 0.001 |
| Middle-aged and elderly age | Prevalence | Male | Saint Vincent and the Grenadines | Psoriasis | -0.49 (-0.5 to -0.48) | < 0.001 |
| Middle-aged and elderly age | Prevalence | Both | Saint Vincent and the Grenadines | RHD | 0.01 (0 to 0.02) | 0.116 |
| Middle-aged and elderly age | Prevalence | Female | Saint Vincent and the Grenadines | RHD | 0.08 (0.08 to 0.09) | < 0.001 |
| Middle-aged and elderly age | Prevalence | Male | Saint Vincent and the Grenadines | RHD | 0.01 (0.01 to 0.02) | < 0.001 |
| Middle-aged and elderly age | Prevalence | Both | Saint Vincent and the Grenadines | RA | 0.67 (0.62 to 0.72) | < 0.001 |
| Middle-aged and elderly age | Prevalence | Female | Saint Vincent and the Grenadines | RA | 0.78 (0.73 to 0.83) | < 0.001 |
| Middle-aged and elderly age | Prevalence | Male | Saint Vincent and the Grenadines | RA | 0.86 | < 0.001 |

| | | | | | | |
|---|---|---|---|---|---|---|
| | | | | | (0.81 to 0.91) | |
| Middle-aged and elderly age | Prevalence | Both | Samoa | AA | -0.04<br>(-0.05 to -0.03) | < 0.001 |
| Middle-aged and elderly age | Prevalence | Female | Samoa | AA | 0<br>(0 to 0) | < 0.001 |
| Middle-aged and elderly age | Prevalence | Male | Samoa | AA | 0<br>(0 to 0) | < 0.001 |
| Middle-aged and elderly age | Prevalence | Both | Samoa | T1D | 0.95<br>(0.93 to 0.96) | < 0.001 |
| Middle-aged and elderly age | Prevalence | Female | Samoa | T1D | 0.9<br>(0.87 to 0.93) | < 0.001 |
| Middle-aged and elderly age | Prevalence | Male | Samoa | T1D | 0.96<br>(0.94 to 0.98) | < 0.001 |
| Middle-aged and elderly age | Prevalence | Both | Samoa | IBD | -0.58<br>(-0.84 to -0.32) | < 0.001 |
| Middle-aged and elderly age | Prevalence | Female | Samoa | IBD | -0.6<br>(-0.87 to -0.33) | < 0.001 |
| Middle-aged and elderly age | Prevalence | Male | Samoa | IBD | -0.57<br>(-0.82 to -0.31) | < 0.001 |
| Middle-aged and elderly age | Prevalence | Both | Samoa | MS | 0.05<br>(-0.01 to 0.12) | 0.109 |
| Middle-aged and elderly age | Prevalence | Female | Samoa | MS | 0.18<br>(0.09 to 0.26) | < 0.001 |
| Middle-aged and elderly age | Prevalence | Male | Samoa | MS | 0.05<br>(0.01 to 0.09) | 0.018 |
| Middle-aged and elderly age | Prevalence | Both | Samoa | Psoriasis | -0.13<br>(-0.14 to -0.11) | < 0.001 |
| Middle-aged and elderly age | Prevalence | Female | Samoa | Psoriasis | -0.14<br>(-0.15 to -0.13) | < 0.001 |
| Middle-aged and elderly age | Prevalence | Male | Samoa | Psoriasis | -0.17<br>(-0.23 to -0.12) | < 0.001 |
| Middle-aged and elderly age | Prevalence | Both | Samoa | RHD | 0.1<br>(0.09 to 0.11) | < 0.001 |

| | | | | | | |
|---|---|---|---|---|---|---|
| Middle-aged and elderly age | Prevalence | Female | Samoa | RHD | 0.14 (0.13 to 0.15) | < 0.001 |
| Middle-aged and elderly age | Prevalence | Male | Samoa | RHD | 0.1 (0.08 to 0.13) | < 0.001 |
| Middle-aged and elderly age | Prevalence | Both | Samoa | RA | 0.29 (0.23 to 0.35) | < 0.001 |
| Middle-aged and elderly age | Prevalence | Female | Samoa | RA | 0.37 (0.27 to 0.46) | < 0.001 |
| Middle-aged and elderly age | Prevalence | Male | Samoa | RA | 0.3 (0.23 to 0.38) | < 0.001 |
| Middle-aged and elderly age | Prevalence | Both | San Marino | AA | 0.03 (0.02 to 0.04) | < 0.001 |
| Middle-aged and elderly age | Prevalence | Female | San Marino | AA | 0 (0 to 0) | < 0.001 |
| Middle-aged and elderly age | Prevalence | Male | San Marino | AA | 0 (0 to 0) | < 0.001 |
| Middle-aged and elderly age | Prevalence | Both | San Marino | T1D | 1.68 (1.65 to 1.71) | < 0.001 |
| Middle-aged and elderly age | Prevalence | Female | San Marino | T1D | 1.67 (1.64 to 1.7) | < 0.001 |
| Middle-aged and elderly age | Prevalence | Male | San Marino | T1D | 1.69 (1.65 to 1.73) | < 0.001 |
| Middle-aged and elderly age | Prevalence | Both | San Marino | IBD | 0.19 (0.13 to 0.24) | < 0.001 |
| Middle-aged and elderly age | Prevalence | Female | San Marino | IBD | 0.2 (0.16 to 0.23) | < 0.001 |
| Middle-aged and elderly age | Prevalence | Male | San Marino | IBD | 0.16 (0.11 to 0.2) | < 0.001 |
| Middle-aged and elderly age | Prevalence | Both | San Marino | MS | 0.37 (0.29 to 0.45) | < 0.001 |
| Middle-aged and elderly age | Prevalence | Female | San Marino | MS | 0.37 (0.31 to 0.44) | < 0.001 |
| Middle-aged and elderly age | Prevalence | Male | San Marino | MS | 0.29 | < 0.001 |

| | | | | | | |
|---|---|---|---|---|---|---|
| | | | | | (0.23 to 0.36) | |
| Middle-aged and elderly age | Prevalence | Both | San Marino | Psoriasis | -0.3<br>(-0.3 to -0.3) | < 0.001 |
| Middle-aged and elderly age | Prevalence | Female | San Marino | Psoriasis | -0.3<br>(-0.3 to -0.29) | < 0.001 |
| Middle-aged and elderly age | Prevalence | Male | San Marino | Psoriasis | -0.3<br>(-0.3 to -0.29) | < 0.001 |
| Middle-aged and elderly age | Prevalence | Both | San Marino | RHD | -0.56<br>(-0.63 to -0.49) | < 0.001 |
| Middle-aged and elderly age | Prevalence | Female | San Marino | RHD | -0.54<br>(-0.58 to -0.49) | < 0.001 |
| Middle-aged and elderly age | Prevalence | Male | San Marino | RHD | -0.59<br>(-0.68 to -0.5) | < 0.001 |
| Middle-aged and elderly age | Prevalence | Both | San Marino | RA | 0.42<br>(0.37 to 0.46) | < 0.001 |
| Middle-aged and elderly age | Prevalence | Female | San Marino | RA | 0.44<br>(0.29 to 0.58) | < 0.001 |
| Middle-aged and elderly age | Prevalence | Male | San Marino | RA | 0.37<br>(0.28 to 0.46) | < 0.001 |
| Middle-aged and elderly age | Prevalence | Both | Sao Tome and Principe | AA | -0.01<br>(-0.02 to 0.01) | 0.321 |
| Middle-aged and elderly age | Prevalence | Female | Sao Tome and Principe | AA | 0<br>(0 to 0) | 0.027 |
| Middle-aged and elderly age | Prevalence | Male | Sao Tome and Principe | AA | 0<br>(0 to 0) | < 0.001 |
| Middle-aged and elderly age | Prevalence | Both | Sao Tome and Principe | T1D | 0.65<br>(0.64 to 0.66) | < 0.001 |
| Middle-aged and elderly age | Prevalence | Female | Sao Tome and Principe | T1D | 0.65<br>(0.63 to 0.66) | < 0.001 |
| Middle-aged and elderly age | Prevalence | Male | Sao Tome and Principe | T1D | 0.64<br>(0.62 to 0.65) | < 0.001 |
| Middle-aged and elderly age | Prevalence | Both | Sao Tome and Principe | IBD | 0.41<br>(0.36 to 0.46) | < 0.001 |

| | | | | | | |
|---|---|---|---|---|---|---|
| Middle-aged and elderly age | Prevalence | Female | Sao Tome and Principe | IBD | 0.37 (0.28 to 0.46) | < 0.001 |
| Middle-aged and elderly age | Prevalence | Male | Sao Tome and Principe | IBD | 0.46 (0.38 to 0.53) | < 0.001 |
| Middle-aged and elderly age | Prevalence | Both | Sao Tome and Principe | MS | 0.34 (0.3 to 0.39) | < 0.001 |
| Middle-aged and elderly age | Prevalence | Female | Sao Tome and Principe | MS | 0.32 (0.3 to 0.35) | < 0.001 |
| Middle-aged and elderly age | Prevalence | Male | Sao Tome and Principe | MS | 0.43 (0.33 to 0.54) | < 0.001 |
| Middle-aged and elderly age | Prevalence | Both | Sao Tome and Principe | Psoriasis | -0.77 (-0.95 to -0.59) | < 0.001 |
| Middle-aged and elderly age | Prevalence | Female | Sao Tome and Principe | Psoriasis | -0.84 (-1.02 to -0.67) | < 0.001 |
| Middle-aged and elderly age | Prevalence | Male | Sao Tome and Principe | Psoriasis | -0.67 (-0.82 to -0.53) | < 0.001 |
| Middle-aged and elderly age | Prevalence | Both | Sao Tome and Principe | RHD | 0.27 (0.25 to 0.29) | < 0.001 |
| Middle-aged and elderly age | Prevalence | Female | Sao Tome and Principe | RHD | 0.32 (0.3 to 0.34) | < 0.001 |
| Middle-aged and elderly age | Prevalence | Male | Sao Tome and Principe | RHD | 0.23 (0.22 to 0.24) | < 0.001 |
| Middle-aged and elderly age | Prevalence | Both | Sao Tome and Principe | RA | 0.78 (0.74 to 0.81) | < 0.001 |
| Middle-aged and elderly age | Prevalence | Female | Sao Tome and Principe | RA | 0.76 (0.71 to 0.82) | < 0.001 |
| Middle-aged and elderly age | Prevalence | Male | Sao Tome and Principe | RA | 0.8 (0.75 to 0.84) | < 0.001 |
| Middle-aged and elderly age | Prevalence | Both | Saudi Arabia | AA | 0.05 (0.03 to 0.06) | < 0.001 |
| Middle-aged and elderly age | Prevalence | Female | Saudi Arabia | AA | 0 (0 to 0) | < 0.001 |
| Middle-aged and elderly age | Prevalence | Male | Saudi Arabia | AA | 0 | 0.015 |

| | | | | | | |
|---|---|---|---|---|---|---|
| | | | | | (0 to 0) | |
| Middle-aged and elderly age | Prevalence | Both | Saudi Arabia | T1D | 2.86<br>(2.56 to 3.17) | < 0.001 |
| Middle-aged and elderly age | Prevalence | Female | Saudi Arabia | T1D | 2.76<br>(2.59 to 2.93) | < 0.001 |
| Middle-aged and elderly age | Prevalence | Male | Saudi Arabia | T1D | 2.95<br>(2.54 to 3.36) | < 0.001 |
| Middle-aged and elderly age | Prevalence | Both | Saudi Arabia | IBD | -0.02<br>(-0.13 to 0.09) | 0.692 |
| Middle-aged and elderly age | Prevalence | Female | Saudi Arabia | IBD | 0.25<br>(0.07 to 0.43) | 0.006 |
| Middle-aged and elderly age | Prevalence | Male | Saudi Arabia | IBD | -0.17<br>(-0.34 to 0) | 0.045 |
| Middle-aged and elderly age | Prevalence | Both | Saudi Arabia | MS | 0.99<br>(0.94 to 1.05) | < 0.001 |
| Middle-aged and elderly age | Prevalence | Female | Saudi Arabia | MS | 0.89<br>(0.83 to 0.95) | < 0.001 |
| Middle-aged and elderly age | Prevalence | Male | Saudi Arabia | MS | 1.02<br>(1 to 1.04) | < 0.001 |
| Middle-aged and elderly age | Prevalence | Both | Saudi Arabia | Psoriasis | -1.78<br>(-1.8 to -1.76) | < 0.001 |
| Middle-aged and elderly age | Prevalence | Female | Saudi Arabia | Psoriasis | -1.78<br>(-1.81 to -1.75) | < 0.001 |
| Middle-aged and elderly age | Prevalence | Male | Saudi Arabia | Psoriasis | -1.79<br>(-1.81 to -1.77) | < 0.001 |
| Middle-aged and elderly age | Prevalence | Both | Saudi Arabia | RHD | -0.81<br>(-1.04 to -0.57) | < 0.001 |
| Middle-aged and elderly age | Prevalence | Female | Saudi Arabia | RHD | -0.94<br>(-1.25 to -0.62) | < 0.001 |
| Middle-aged and elderly age | Prevalence | Male | Saudi Arabia | RHD | -0.75<br>(-0.84 to -0.65) | < 0.001 |
| Middle-aged and elderly age | Prevalence | Both | Saudi Arabia | RA | 1.36<br>(1.28 to 1.44) | < 0.001 |

| | | | | | | |
|---|---|---|---|---|---|---|
| Middle-aged and elderly age | Prevalence | Female | Saudi Arabia | RA | 1.33 (1.25 to 1.42) | < 0.001 |
| Middle-aged and elderly age | Prevalence | Male | Saudi Arabia | RA | 1.26 (1.2 to 1.33) | < 0.001 |
| Middle-aged and elderly age | Prevalence | Both | Senegal | AA | 0.03 (0.02 to 0.05) | < 0.001 |
| Middle-aged and elderly age | Prevalence | Female | Senegal | AA | 0 (0 to 0) | 0.027 |
| Middle-aged and elderly age | Prevalence | Male | Senegal | AA | 0 (0 to 0) | < 0.001 |
| Middle-aged and elderly age | Prevalence | Both | Senegal | T1D | 0.32 (0.3 to 0.33) | < 0.001 |
| Middle-aged and elderly age | Prevalence | Female | Senegal | T1D | 0.32 (0.3 to 0.35) | < 0.001 |
| Middle-aged and elderly age | Prevalence | Male | Senegal | T1D | 0.31 (0.3 to 0.33) | < 0.001 |
| Middle-aged and elderly age | Prevalence | Both | Senegal | IBD | 0.17 (0.08 to 0.27) | < 0.001 |
| Middle-aged and elderly age | Prevalence | Female | Senegal | IBD | -0.04 (-0.2 to 0.12) | 0.607 |
| Middle-aged and elderly age | Prevalence | Male | Senegal | IBD | 0.38 (0.25 to 0.51) | < 0.001 |
| Middle-aged and elderly age | Prevalence | Both | Senegal | MS | 0.32 (0.3 to 0.34) | < 0.001 |
| Middle-aged and elderly age | Prevalence | Female | Senegal | MS | 0.29 (0.26 to 0.31) | < 0.001 |
| Middle-aged and elderly age | Prevalence | Male | Senegal | MS | 0.29 (0.24 to 0.35) | < 0.001 |
| Middle-aged and elderly age | Prevalence | Both | Senegal | Psoriasis | -0.58 (-0.74 to -0.43) | < 0.001 |
| Middle-aged and elderly age | Prevalence | Female | Senegal | Psoriasis | -0.64 (-0.75 to -0.53) | < 0.001 |
| Middle-aged and elderly age | Prevalence | Male | Senegal | Psoriasis | -0.5 | < 0.001 |

| | | | | | | |
|---|---|---|---|---|---|---|
| | | | | | (-0.64 to -0.34) | |
| Middle-aged and elderly age | Prevalence | Both | Senegal | RHD | 0.09<br>(0.07 to 0.11) | < 0.001 |
| Middle-aged and elderly age | Prevalence | Female | Senegal | RHD | 0.12<br>(0.06 to 0.18) | < 0.001 |
| Middle-aged and elderly age | Prevalence | Male | Senegal | RHD | 0.04<br>(0.02 to 0.07) | < 0.001 |
| Middle-aged and elderly age | Prevalence | Both | Senegal | RA | 0.56<br>(0.53 to 0.6) | < 0.001 |
| Middle-aged and elderly age | Prevalence | Female | Senegal | RA | 0.53<br>(0.47 to 0.59) | < 0.001 |
| Middle-aged and elderly age | Prevalence | Male | Senegal | RA | 0.58<br>(0.55 to 0.6) | < 0.001 |
| Middle-aged and elderly age | Prevalence | Both | Serbia | AA | -0.02<br>(-0.03 to -0.01) | 0.002 |
| Middle-aged and elderly age | Prevalence | Female | Serbia | AA | 0<br>(0 to 0) | < 0.001 |
| Middle-aged and elderly age | Prevalence | Male | Serbia | AA | 0<br>(0 to 0) | < 0.001 |
| Middle-aged and elderly age | Prevalence | Both | Serbia | T1D | 1.76<br>(1.72 to 1.8) | < 0.001 |
| Middle-aged and elderly age | Prevalence | Female | Serbia | T1D | 1.75<br>(1.72 to 1.79) | < 0.001 |
| Middle-aged and elderly age | Prevalence | Male | Serbia | T1D | 1.74<br>(1.67 to 1.8) | < 0.001 |
| Middle-aged and elderly age | Prevalence | Both | Serbia | IBD | 0.77<br>(0.68 to 0.87) | < 0.001 |
| Middle-aged and elderly age | Prevalence | Female | Serbia | IBD | 0.8<br>(0.7 to 0.89) | < 0.001 |
| Middle-aged and elderly age | Prevalence | Male | Serbia | IBD | 0.75<br>(0.67 to 0.83) | < 0.001 |
| Middle-aged and elderly age | Prevalence | Both | Serbia | MS | 0.84<br>(0.82 to 0.87) | < 0.001 |

| | | | | | | |
|---|---|---|---|---|---|---|
| Middle-aged and elderly age | Prevalence | Female | Serbia | MS | 0.82 (0.81 to 0.83) | < 0.001 |
| Middle-aged and elderly age | Prevalence | Male | Serbia | MS | 1 (0.91 to 1.09) | < 0.001 |
| Middle-aged and elderly age | Prevalence | Both | Serbia | Psoriasis | -0.86 (-0.88 to -0.83) | < 0.001 |
| Middle-aged and elderly age | Prevalence | Female | Serbia | Psoriasis | -0.88 (-0.93 to -0.84) | < 0.001 |
| Middle-aged and elderly age | Prevalence | Male | Serbia | Psoriasis | -0.84 (-0.85 to -0.82) | < 0.001 |
| Middle-aged and elderly age | Prevalence | Both | Serbia | RHD | -0.75 (-0.91 to -0.59) | < 0.001 |
| Middle-aged and elderly age | Prevalence | Female | Serbia | RHD | -0.86 (-0.92 to -0.8) | < 0.001 |
| Middle-aged and elderly age | Prevalence | Male | Serbia | RHD | -0.52 (-0.75 to -0.28) | < 0.001 |
| Middle-aged and elderly age | Prevalence | Both | Serbia | RA | 1.53 (1.2 to 1.87) | < 0.001 |
| Middle-aged and elderly age | Prevalence | Female | Serbia | RA | 1.56 (1.24 to 1.88) | < 0.001 |
| Middle-aged and elderly age | Prevalence | Male | Serbia | RA | 1.71 (1.49 to 1.93) | < 0.001 |
| Middle-aged and elderly age | Prevalence | Both | Seychelles | AA | -0.05 (-0.06 to -0.04) | < 0.001 |
| Middle-aged and elderly age | Prevalence | Female | Seychelles | AA | 0 (0 to 0) | < 0.001 |
| Middle-aged and elderly age | Prevalence | Male | Seychelles | AA | 0 (0 to 0) | < 0.001 |
| Middle-aged and elderly age | Prevalence | Both | Seychelles | T1D | 1.11 (1.1 to 1.12) | < 0.001 |
| Middle-aged and elderly age | Prevalence | Female | Seychelles | T1D | 1.04 (1.03 to 1.05) | < 0.001 |
| Middle-aged and elderly age | Prevalence | Male | Seychelles | T1D | 1.13 | < 0.001 |

| | | | | | | |
|---|---|---|---|---|---|---|
| | | | | | (1.06 to 1.2) | |
| Middle-aged and elderly age | Prevalence | Both | Seychelles | IBD | 0.56<br>(0.5 to 0.62) | < 0.001 |
| Middle-aged and elderly age | Prevalence | Female | Seychelles | IBD | 0.59<br>(0.57 to 0.61) | < 0.001 |
| Middle-aged and elderly age | Prevalence | Male | Seychelles | IBD | 0.55<br>(0.5 to 0.59) | < 0.001 |
| Middle-aged and elderly age | Prevalence | Both | Seychelles | MS | 0.28<br>(0.21 to 0.35) | < 0.001 |
| Middle-aged and elderly age | Prevalence | Female | Seychelles | MS | 0.49<br>(0.41 to 0.57) | < 0.001 |
| Middle-aged and elderly age | Prevalence | Male | Seychelles | MS | 0.19<br>(0.16 to 0.22) | < 0.001 |
| Middle-aged and elderly age | Prevalence | Both | Seychelles | Psoriasis | -0.4<br>(-0.42 to -0.38) | < 0.001 |
| Middle-aged and elderly age | Prevalence | Female | Seychelles | Psoriasis | -0.42<br>(-0.43 to -0.41) | < 0.001 |
| Middle-aged and elderly age | Prevalence | Male | Seychelles | Psoriasis | -0.46<br>(-0.48 to -0.43) | < 0.001 |
| Middle-aged and elderly age | Prevalence | Both | Seychelles | RHD | -0.12<br>(-0.2 to -0.05) | 0.001 |
| Middle-aged and elderly age | Prevalence | Female | Seychelles | RHD | -0.04<br>(-0.1 to 0.02) | 0.173 |
| Middle-aged and elderly age | Prevalence | Male | Seychelles | RHD | -0.07<br>(-0.16 to 0.01) | 0.089 |
| Middle-aged and elderly age | Prevalence | Both | Seychelles | RA | 0.5<br>(0.44 to 0.56) | < 0.001 |
| Middle-aged and elderly age | Prevalence | Female | Seychelles | RA | 0.63<br>(0.56 to 0.71) | < 0.001 |
| Middle-aged and elderly age | Prevalence | Male | Seychelles | RA | 0.47<br>(0.44 to 0.5) | < 0.001 |
| Middle-aged and elderly age | Prevalence | Both | Sierra Leone | AA | -0.03<br>(-0.05 to -0.02) | < 0.001 |

| | | | | | | |
|---|---|---|---|---|---|---|
| Middle-aged and elderly age | Prevalence | Female | Sierra Leone | AA | 0<br>(0 to 0) | 0.027 |
| Middle-aged and elderly age | Prevalence | Male | Sierra Leone | AA | 0<br>(0 to 0) | < 0.001 |
| Middle-aged and elderly age | Prevalence | Both | Sierra Leone | T1D | 0.3<br>(0.29 to 0.31) | < 0.001 |
| Middle-aged and elderly age | Prevalence | Female | Sierra Leone | T1D | 0.3<br>(0.29 to 0.32) | < 0.001 |
| Middle-aged and elderly age | Prevalence | Male | Sierra Leone | T1D | 0.29<br>(0.27 to 0.31) | < 0.001 |
| Middle-aged and elderly age | Prevalence | Both | Sierra Leone | IBD | 0.4<br>(0.34 to 0.46) | < 0.001 |
| Middle-aged and elderly age | Prevalence | Female | Sierra Leone | IBD | 0.18<br>(0.11 to 0.25) | < 0.001 |
| Middle-aged and elderly age | Prevalence | Male | Sierra Leone | IBD | 0.6<br>(0.53 to 0.67) | < 0.001 |
| Middle-aged and elderly age | Prevalence | Both | Sierra Leone | MS | 0.27<br>(0.25 to 0.29) | < 0.001 |
| Middle-aged and elderly age | Prevalence | Female | Sierra Leone | MS | 0.31<br>(0.29 to 0.33) | < 0.001 |
| Middle-aged and elderly age | Prevalence | Male | Sierra Leone | MS | 0.34<br>(0.28 to 0.41) | < 0.001 |
| Middle-aged and elderly age | Prevalence | Both | Sierra Leone | Psoriasis | -0.55<br>(-0.7 to -0.4) | < 0.001 |
| Middle-aged and elderly age | Prevalence | Female | Sierra Leone | Psoriasis | -0.61<br>(-0.72 to -0.5) | < 0.001 |
| Middle-aged and elderly age | Prevalence | Male | Sierra Leone | Psoriasis | -0.47<br>(-0.67 to -0.27) | < 0.001 |
| Middle-aged and elderly age | Prevalence | Both | Sierra Leone | RHD | 0.15<br>(0.15 to 0.16) | < 0.001 |
| Middle-aged and elderly age | Prevalence | Female | Sierra Leone | RHD | 0.2<br>(0.19 to 0.21) | < 0.001 |
| Middle-aged and elderly age | Prevalence | Male | Sierra Leone | RHD | 0.12 | < 0.001 |

| | | | | | | |
|---|---|---|---|---|---|---|
| | | | | | (0.1 to 0.14) | |
| Middle-aged and elderly age | Prevalence | Both | Sierra Leone | RA | 0.52<br>(0.48 to 0.56) | < 0.001 |
| Middle-aged and elderly age | Prevalence | Female | Sierra Leone | RA | 0.54<br>(0.51 to 0.56) | < 0.001 |
| Middle-aged and elderly age | Prevalence | Male | Sierra Leone | RA | 0.55<br>(0.5 to 0.61) | < 0.001 |
| Middle-aged and elderly age | Prevalence | Both | Singapore | AA | -0.18<br>(-0.22 to -0.15) | < 0.001 |
| Middle-aged and elderly age | Prevalence | Female | Singapore | AA | 0<br>(0 to 0) | < 0.001 |
| Middle-aged and elderly age | Prevalence | Male | Singapore | AA | 0<br>(0 to 0) | 0.002 |
| Middle-aged and elderly age | Prevalence | Both | Singapore | T1D | 0.38<br>(0.37 to 0.39) | < 0.001 |
| Middle-aged and elderly age | Prevalence | Female | Singapore | T1D | 0.36<br>(0.34 to 0.38) | < 0.001 |
| Middle-aged and elderly age | Prevalence | Male | Singapore | T1D | 0.37<br>(0.35 to 0.39) | < 0.001 |
| Middle-aged and elderly age | Prevalence | Both | Singapore | IBD | -0.55<br>(-0.64 to -0.45) | < 0.001 |
| Middle-aged and elderly age | Prevalence | Female | Singapore | IBD | -0.52<br>(-0.61 to -0.43) | < 0.001 |
| Middle-aged and elderly age | Prevalence | Male | Singapore | IBD | -0.61<br>(-0.71 to -0.51) | < 0.001 |
| Middle-aged and elderly age | Prevalence | Both | Singapore | MS | 0.01<br>(-0.09 to 0.1) | 0.853 |
| Middle-aged and elderly age | Prevalence | Female | Singapore | MS | 0.16<br>(0.09 to 0.23) | < 0.001 |
| Middle-aged and elderly age | Prevalence | Male | Singapore | MS | -0.04<br>(-0.12 to 0.04) | 0.364 |
| Middle-aged and elderly age | Prevalence | Both | Singapore | Psoriasis | -0.34<br>(-0.37 to -0.31) | < 0.001 |

| | | | | | | |
|---|---|---|---|---|---|---|
| Middle-aged and elderly age | Prevalence | Female | Singapore | Psoriasis | -0.42 (-0.47 to -0.37) | < 0.001 |
| Middle-aged and elderly age | Prevalence | Male | Singapore | Psoriasis | -0.28 (-0.3 to -0.27) | < 0.001 |
| Middle-aged and elderly age | Prevalence | Both | Singapore | RHD | -3.45 (-3.64 to -3.26) | < 0.001 |
| Middle-aged and elderly age | Prevalence | Female | Singapore | RHD | -3.6 (-3.77 to -3.43) | < 0.001 |
| Middle-aged and elderly age | Prevalence | Male | Singapore | RHD | -2.58 (-2.68 to -2.47) | < 0.001 |
| Middle-aged and elderly age | Prevalence | Both | Singapore | RA | 0.67 (0.63 to 0.71) | < 0.001 |
| Middle-aged and elderly age | Prevalence | Female | Singapore | RA | 0.81 (0.79 to 0.84) | < 0.001 |
| Middle-aged and elderly age | Prevalence | Male | Singapore | RA | 0.7 (0.64 to 0.77) | < 0.001 |
| Middle-aged and elderly age | Prevalence | Both | Slovakia | AA | -0.06 (-0.06 to -0.05) | < 0.001 |
| Middle-aged and elderly age | Prevalence | Female | Slovakia | AA | 0 (0 to 0) | < 0.001 |
| Middle-aged and elderly age | Prevalence | Male | Slovakia | AA | 0 (0 to 0) | < 0.001 |
| Middle-aged and elderly age | Prevalence | Both | Slovakia | T1D | 2.22 (2.14 to 2.29) | < 0.001 |
| Middle-aged and elderly age | Prevalence | Female | Slovakia | T1D | 2.19 (2.16 to 2.21) | < 0.001 |
| Middle-aged and elderly age | Prevalence | Male | Slovakia | T1D | 2.24 (2.08 to 2.4) | < 0.001 |
| Middle-aged and elderly age | Prevalence | Both | Slovakia | IBD | -0.3 (-0.32 to -0.28) | < 0.001 |
| Middle-aged and elderly age | Prevalence | Female | Slovakia | IBD | -0.31 (-0.33 to -0.29) | < 0.001 |
| Middle-aged and elderly age | Prevalence | Male | Slovakia | IBD | -0.32 | < 0.001 |

| | | | | | | |
|---|---|---|---|---|---|---|
| | | | | | (-0.36 to -0.29) | |
| Middle-aged and elderly age | Prevalence | Both | Slovakia | MS | 0.5<br>(0.43 to 0.57) | < 0.001 |
| Middle-aged and elderly age | Prevalence | Female | Slovakia | MS | 0.63<br>(0.58 to 0.68) | < 0.001 |
| Middle-aged and elderly age | Prevalence | Male | Slovakia | MS | 0.39<br>(0.32 to 0.46) | < 0.001 |
| Middle-aged and elderly age | Prevalence | Both | Slovakia | Psoriasis | -0.95<br>(-0.99 to -0.91) | < 0.001 |
| Middle-aged and elderly age | Prevalence | Female | Slovakia | Psoriasis | -0.97<br>(-1.03 to -0.91) | < 0.001 |
| Middle-aged and elderly age | Prevalence | Male | Slovakia | Psoriasis | -0.92<br>(-0.94 to -0.9) | < 0.001 |
| Middle-aged and elderly age | Prevalence | Both | Slovakia | RHD | 0.08<br>(-0.05 to 0.22) | 0.227 |
| Middle-aged and elderly age | Prevalence | Female | Slovakia | RHD | 0.12<br>(-0.05 to 0.28) | 0.167 |
| Middle-aged and elderly age | Prevalence | Male | Slovakia | RHD | 0.1<br>(0.05 to 0.16) | < 0.001 |
| Middle-aged and elderly age | Prevalence | Both | Slovakia | RA | 0.68<br>(0.58 to 0.78) | < 0.001 |
| Middle-aged and elderly age | Prevalence | Female | Slovakia | RA | 0.78<br>(0.68 to 0.88) | < 0.001 |
| Middle-aged and elderly age | Prevalence | Male | Slovakia | RA | 0.68<br>(0.57 to 0.79) | < 0.001 |
| Middle-aged and elderly age | Prevalence | Both | Slovenia | AA | -0.05<br>(-0.05 to -0.04) | < 0.001 |
| Middle-aged and elderly age | Prevalence | Female | Slovenia | AA | 0<br>(0 to 0) | < 0.001 |
| Middle-aged and elderly age | Prevalence | Male | Slovenia | AA | 0<br>(0 to 0) | < 0.001 |
| Middle-aged and elderly age | Prevalence | Both | Slovenia | T1D | 2.1<br>(2.05 to 2.16) | < 0.001 |

| | | | | | | |
|---|---|---|---|---|---|---|
| Middle-aged and elderly age | Prevalence | Female | Slovenia | T1D | 2.01 (1.98 to 2.05) | < 0.001 |
| Middle-aged and elderly age | Prevalence | Male | Slovenia | T1D | 2.18 (2.12 to 2.24) | < 0.001 |
| Middle-aged and elderly age | Prevalence | Both | Slovenia | IBD | 0.03 (0.02 to 0.04) | < 0.001 |
| Middle-aged and elderly age | Prevalence | Female | Slovenia | IBD | 0.13 (0.11 to 0.15) | < 0.001 |
| Middle-aged and elderly age | Prevalence | Male | Slovenia | IBD | -0.08 (-0.1 to -0.06) | < 0.001 |
| Middle-aged and elderly age | Prevalence | Both | Slovenia | MS | 0.28 (0.25 to 0.3) | < 0.001 |
| Middle-aged and elderly age | Prevalence | Female | Slovenia | MS | 0.29 (0.28 to 0.31) | < 0.001 |
| Middle-aged and elderly age | Prevalence | Male | Slovenia | MS | 0.27 (0.25 to 0.3) | < 0.001 |
| Middle-aged and elderly age | Prevalence | Both | Slovenia | Psoriasis | -0.77 (-0.79 to -0.75) | < 0.001 |
| Middle-aged and elderly age | Prevalence | Female | Slovenia | Psoriasis | -0.8 (-0.83 to -0.76) | < 0.001 |
| Middle-aged and elderly age | Prevalence | Male | Slovenia | Psoriasis | -0.75 (-0.76 to -0.73) | < 0.001 |
| Middle-aged and elderly age | Prevalence | Both | Slovenia | RHD | -1.02 (-1.69 to -0.34) | 0.003 |
| Middle-aged and elderly age | Prevalence | Female | Slovenia | RHD | -0.89 (-1.76 to -0.01) | 0.048 |
| Middle-aged and elderly age | Prevalence | Male | Slovenia | RHD | -1.15 (-1.57 to -0.73) | < 0.001 |
| Middle-aged and elderly age | Prevalence | Both | Slovenia | RA | 0.77 (0.74 to 0.8) | < 0.001 |
| Middle-aged and elderly age | Prevalence | Female | Slovenia | RA | 0.87 (0.85 to 0.89) | < 0.001 |
| Middle-aged and elderly age | Prevalence | Male | Slovenia | RA | 0.84 | < 0.001 |

| | | | | | | |
|---|---|---|---|---|---|---|
| | | | | | (0.81 to 0.86) | |
| Middle-aged and elderly age | Prevalence | Both | Solomon Islands | AA | 0.04 (0.04 to 0.05) | < 0.001 |
| Middle-aged and elderly age | Prevalence | Female | Solomon Islands | AA | 0 (0 to 0) | < 0.001 |
| Middle-aged and elderly age | Prevalence | Male | Solomon Islands | AA | 0 (0 to 0) | < 0.001 |
| Middle-aged and elderly age | Prevalence | Both | Solomon Islands | T1D | 0.8 (0.79 to 0.81) | < 0.001 |
| Middle-aged and elderly age | Prevalence | Female | Solomon Islands | T1D | 0.79 (0.77 to 0.81) | < 0.001 |
| Middle-aged and elderly age | Prevalence | Male | Solomon Islands | T1D | 0.83 (0.81 to 0.85) | < 0.001 |
| Middle-aged and elderly age | Prevalence | Both | Solomon Islands | IBD | -0.31 (-0.4 to -0.22) | < 0.001 |
| Middle-aged and elderly age | Prevalence | Female | Solomon Islands | IBD | -0.54 (-0.6 to -0.47) | < 0.001 |
| Middle-aged and elderly age | Prevalence | Male | Solomon Islands | IBD | -0.1 (-0.2 to 0) | 0.046 |
| Middle-aged and elderly age | Prevalence | Both | Solomon Islands | MS | 0.24 (0.18 to 0.29) | < 0.001 |
| Middle-aged and elderly age | Prevalence | Female | Solomon Islands | MS | 0.24 (0.21 to 0.26) | < 0.001 |
| Middle-aged and elderly age | Prevalence | Male | Solomon Islands | MS | 0.17 (0.08 to 0.27) | < 0.001 |
| Middle-aged and elderly age | Prevalence | Both | Solomon Islands | Psoriasis | -0.52 (-0.55 to -0.49) | < 0.001 |
| Middle-aged and elderly age | Prevalence | Female | Solomon Islands | Psoriasis | -0.47 (-0.5 to -0.44) | < 0.001 |
| Middle-aged and elderly age | Prevalence | Male | Solomon Islands | Psoriasis | -0.5 (-0.54 to -0.46) | < 0.001 |
| Middle-aged and elderly age | Prevalence | Both | Solomon Islands | RHD | 0.07 (0.05 to 0.09) | < 0.001 |

| | | | | | | |
|---|---|---|---|---|---|---|
| Middle-aged and elderly age | Prevalence | Female | Solomon Islands | RHD | 0.07 (0.05 to 0.09) | < 0.001 |
| Middle-aged and elderly age | Prevalence | Male | Solomon Islands | RHD | 0.04 (0.03 to 0.04) | < 0.001 |
| Middle-aged and elderly age | Prevalence | Both | Solomon Islands | RA | 0.49 (0.45 to 0.53) | < 0.001 |
| Middle-aged and elderly age | Prevalence | Female | Solomon Islands | RA | 0.43 (0.37 to 0.48) | < 0.001 |
| Middle-aged and elderly age | Prevalence | Male | Solomon Islands | RA | 0.45 (0.42 to 0.49) | < 0.001 |
| Middle-aged and elderly age | Prevalence | Both | Somalia | AA | 0.06 (0.05 to 0.06) | < 0.001 |
| Middle-aged and elderly age | Prevalence | Female | Somalia | AA | 0 (0 to 0) | 1.000 |
| Middle-aged and elderly age | Prevalence | Male | Somalia | AA | 0 (0 to 0) | < 0.001 |
| Middle-aged and elderly age | Prevalence | Both | Somalia | T1D | 0.17 (0.15 to 0.2) | < 0.001 |
| Middle-aged and elderly age | Prevalence | Female | Somalia | T1D | 0.19 (0.16 to 0.22) | < 0.001 |
| Middle-aged and elderly age | Prevalence | Male | Somalia | T1D | 0.17 (0.14 to 0.2) | < 0.001 |
| Middle-aged and elderly age | Prevalence | Both | Somalia | IBD | -0.06 (-0.09 to -0.04) | < 0.001 |
| Middle-aged and elderly age | Prevalence | Female | Somalia | IBD | -0.08 (-0.11 to -0.04) | < 0.001 |
| Middle-aged and elderly age | Prevalence | Male | Somalia | IBD | -0.06 (-0.08 to -0.03) | < 0.001 |
| Middle-aged and elderly age | Prevalence | Both | Somalia | MS | 0.19 (0.15 to 0.22) | < 0.001 |
| Middle-aged and elderly age | Prevalence | Female | Somalia | MS | 0.12 (0.08 to 0.16) | < 0.001 |
| Middle-aged and elderly age | Prevalence | Male | Somalia | MS | 0.15 | < 0.001 |

| | | | | | | |
|---|---|---|---|---|---|---|
| | | | | | (0.13 to 0.18) | |
| Middle-aged and elderly age | Prevalence | Both | Somalia | Psoriasis | 0.12<br>(0.1 to 0.14) | < 0.001 |
| Middle-aged and elderly age | Prevalence | Female | Somalia | Psoriasis | 0.09<br>(0.04 to 0.14) | < 0.001 |
| Middle-aged and elderly age | Prevalence | Male | Somalia | Psoriasis | 0.13<br>(0.08 to 0.17) | < 0.001 |
| Middle-aged and elderly age | Prevalence | Both | Somalia | RHD | 0.13<br>(0.1 to 0.15) | < 0.001 |
| Middle-aged and elderly age | Prevalence | Female | Somalia | RHD | 0.15<br>(0.1 to 0.2) | < 0.001 |
| Middle-aged and elderly age | Prevalence | Male | Somalia | RHD | 0.09<br>(0.05 to 0.14) | < 0.001 |
| Middle-aged and elderly age | Prevalence | Both | Somalia | RA | 0.16<br>(0.11 to 0.22) | < 0.001 |
| Middle-aged and elderly age | Prevalence | Female | Somalia | RA | 0.17<br>(0.1 to 0.24) | < 0.001 |
| Middle-aged and elderly age | Prevalence | Male | Somalia | RA | 0.26<br>(0.2 to 0.32) | < 0.001 |
| Middle-aged and elderly age | Prevalence | Both | South Africa | AA | 0<br>(-0.01 to 0.01) | 0.935 |
| Middle-aged and elderly age | Prevalence | Female | South Africa | AA | 0<br>(0 to 0) | < 0.001 |
| Middle-aged and elderly age | Prevalence | Male | South Africa | AA | 0<br>(0 to 0) | < 0.001 |
| Middle-aged and elderly age | Prevalence | Both | South Africa | T1D | 0.46<br>(0.46 to 0.47) | < 0.001 |
| Middle-aged and elderly age | Prevalence | Female | South Africa | T1D | 0.48<br>(0.47 to 0.48) | < 0.001 |
| Middle-aged and elderly age | Prevalence | Male | South Africa | T1D | 0.45<br>(0.41 to 0.48) | < 0.001 |
| Middle-aged and elderly age | Prevalence | Both | South Africa | IBD | 0.43<br>(0.36 to 0.5) | < 0.001 |

| | | | | | | |
|---|---|---|---|---|---|---|
| Middle-aged and elderly age | Prevalence | Female | South Africa | IBD | 0.37<br>(0.28 to 0.46) | < 0.001 |
| Middle-aged and elderly age | Prevalence | Male | South Africa | IBD | 0.48<br>(0.4 to 0.56) | < 0.001 |
| Middle-aged and elderly age | Prevalence | Both | South Africa | MS | 0.16<br>(0.05 to 0.28) | 0.005 |
| Middle-aged and elderly age | Prevalence | Female | South Africa | MS | 0.17<br>(0.06 to 0.28) | 0.002 |
| Middle-aged and elderly age | Prevalence | Male | South Africa | MS | 0.18<br>(0.13 to 0.22) | < 0.001 |
| Middle-aged and elderly age | Prevalence | Both | South Africa | Psoriasis | -0.42<br>(-0.51 to -0.34) | < 0.001 |
| Middle-aged and elderly age | Prevalence | Female | South Africa | Psoriasis | -0.46<br>(-0.52 to -0.39) | < 0.001 |
| Middle-aged and elderly age | Prevalence | Male | South Africa | Psoriasis | -0.37<br>(-0.47 to -0.27) | < 0.001 |
| Middle-aged and elderly age | Prevalence | Both | South Africa | RHD | 0.07<br>(0.05 to 0.09) | < 0.001 |
| Middle-aged and elderly age | Prevalence | Female | South Africa | RHD | 0.08<br>(0.05 to 0.12) | < 0.001 |
| Middle-aged and elderly age | Prevalence | Male | South Africa | RHD | 0.04<br>(0.02 to 0.06) | < 0.001 |
| Middle-aged and elderly age | Prevalence | Both | South Africa | RA | -0.16<br>(-0.27 to -0.05) | 0.004 |
| Middle-aged and elderly age | Prevalence | Female | South Africa | RA | -0.19<br>(-0.25 to -0.13) | < 0.001 |
| Middle-aged and elderly age | Prevalence | Male | South Africa | RA | -0.17<br>(-0.25 to -0.08) | < 0.001 |
| Middle-aged and elderly age | Prevalence | Both | South Sudan | AA | 0.14<br>(0.13 to 0.16) | < 0.001 |
| Middle-aged and elderly age | Prevalence | Female | South Sudan | AA | 0<br>(0 to 0) | 1.000 |
| Middle-aged and elderly age | Prevalence | Male | South Sudan | AA | 0 | < 0.001 |

| | | | | | | |
|---|---|---|---|---|---|---|
| | | | | | (0 to 0) | |
| Middle-aged and elderly age | Prevalence | Both | South Sudan | T1D | 0.34<br>(0.33 to 0.36) | < 0.001 |
| Middle-aged and elderly age | Prevalence | Female | South Sudan | T1D | 0.35<br>(0.34 to 0.37) | < 0.001 |
| Middle-aged and elderly age | Prevalence | Male | South Sudan | T1D | 0.35<br>(0.33 to 0.36) | < 0.001 |
| Middle-aged and elderly age | Prevalence | Both | South Sudan | IBD | 0.32<br>(0.29 to 0.35) | < 0.001 |
| Middle-aged and elderly age | Prevalence | Female | South Sudan | IBD | 0.23<br>(0.18 to 0.28) | < 0.001 |
| Middle-aged and elderly age | Prevalence | Male | South Sudan | IBD | 0.37<br>(0.34 to 0.4) | < 0.001 |
| Middle-aged and elderly age | Prevalence | Both | South Sudan | MS | 0.31<br>(0.29 to 0.34) | < 0.001 |
| Middle-aged and elderly age | Prevalence | Female | South Sudan | MS | 0.13<br>(0.1 to 0.17) | < 0.001 |
| Middle-aged and elderly age | Prevalence | Male | South Sudan | MS | 0.19<br>(0.15 to 0.23) | < 0.001 |
| Middle-aged and elderly age | Prevalence | Both | South Sudan | Psoriasis | -0.17<br>(-0.19 to -0.15) | < 0.001 |
| Middle-aged and elderly age | Prevalence | Female | South Sudan | Psoriasis | -0.21<br>(-0.24 to -0.18) | < 0.001 |
| Middle-aged and elderly age | Prevalence | Male | South Sudan | Psoriasis | -0.18<br>(-0.26 to -0.11) | < 0.001 |
| Middle-aged and elderly age | Prevalence | Both | South Sudan | RHD | 0.15<br>(0.11 to 0.19) | < 0.001 |
| Middle-aged and elderly age | Prevalence | Female | South Sudan | RHD | 0.14<br>(0.12 to 0.15) | < 0.001 |
| Middle-aged and elderly age | Prevalence | Male | South Sudan | RHD | 0.07<br>(0.02 to 0.13) | 0.013 |
| Middle-aged and elderly age | Prevalence | Both | South Sudan | RA | 0.16<br>(0.09 to 0.24) | < 0.001 |

| | | | | | | |
|---|---|---|---|---|---|---|
| Middle-aged and elderly age | Prevalence | Female | South Sudan | RA | 0.29 (0.25 to 0.33) | < 0.001 |
| Middle-aged and elderly age | Prevalence | Male | South Sudan | RA | 0.29 (0.2 to 0.37) | < 0.001 |
| Middle-aged and elderly age | Prevalence | Both | Spain | AA | -0.02 (-0.03 to -0.02) | < 0.001 |
| Middle-aged and elderly age | Prevalence | Female | Spain | AA | 0 (0 to 0) | < 0.001 |
| Middle-aged and elderly age | Prevalence | Male | Spain | AA | 0 (0 to 0) | < 0.001 |
| Middle-aged and elderly age | Prevalence | Both | Spain | T1D | 2.52 (2.19 to 2.85) | < 0.001 |
| Middle-aged and elderly age | Prevalence | Female | Spain | T1D | 2.31 (2 to 2.63) | < 0.001 |
| Middle-aged and elderly age | Prevalence | Male | Spain | T1D | 2.71 (2.4 to 3.03) | < 0.001 |
| Middle-aged and elderly age | Prevalence | Both | Spain | IBD | 1.37 (1.29 to 1.44) | < 0.001 |
| Middle-aged and elderly age | Prevalence | Female | Spain | IBD | 1.4 (1.33 to 1.47) | < 0.001 |
| Middle-aged and elderly age | Prevalence | Male | Spain | IBD | 1.33 (1.25 to 1.41) | < 0.001 |
| Middle-aged and elderly age | Prevalence | Both | Spain | MS | 1.41 (1.14 to 1.68) | < 0.001 |
| Middle-aged and elderly age | Prevalence | Female | Spain | MS | 1.41 (1.19 to 1.63) | < 0.001 |
| Middle-aged and elderly age | Prevalence | Male | Spain | MS | 1.42 (1.11 to 1.73) | < 0.001 |
| Middle-aged and elderly age | Prevalence | Both | Spain | Psoriasis | -0.44 (-0.46 to -0.43) | < 0.001 |
| Middle-aged and elderly age | Prevalence | Female | Spain | Psoriasis | -0.44 (-0.46 to -0.43) | < 0.001 |
| Middle-aged and elderly age | Prevalence | Male | Spain | Psoriasis | -0.45 | < 0.001 |

| | | | | | | |
|---|---|---|---|---|---|---|
| | | | | | (-0.47 to -0.43) | |
| Middle-aged and elderly age | Prevalence | Both | Spain | RHD | -2.98<br>(-3.09 to -2.87) | < 0.001 |
| Middle-aged and elderly age | Prevalence | Female | Spain | RHD | -3.42<br>(-3.56 to -3.29) | < 0.001 |
| Middle-aged and elderly age | Prevalence | Male | Spain | RHD | -2.18<br>(-2.34 to -2.02) | < 0.001 |
| Middle-aged and elderly age | Prevalence | Both | Spain | RA | 0.39<br>(0.36 to 0.41) | < 0.001 |
| Middle-aged and elderly age | Prevalence | Female | Spain | RA | 0.4<br>(0.37 to 0.44) | < 0.001 |
| Middle-aged and elderly age | Prevalence | Male | Spain | RA | 0.43<br>(0.42 to 0.45) | < 0.001 |
| Middle-aged and elderly age | Prevalence | Both | Sri Lanka | AA | -0.01<br>(-0.01 to 0) | < 0.001 |
| Middle-aged and elderly age | Prevalence | Female | Sri Lanka | AA | 0<br>(0 to 0) | < 0.001 |
| Middle-aged and elderly age | Prevalence | Male | Sri Lanka | AA | -0.11<br>(-0.11 to -0.11) | < 0.001 |
| Middle-aged and elderly age | Prevalence | Both | Sri Lanka | T1D | 0.99<br>(0.93 to 1.05) | < 0.001 |
| Middle-aged and elderly age | Prevalence | Female | Sri Lanka | T1D | 0.94<br>(0.87 to 1.02) | < 0.001 |
| Middle-aged and elderly age | Prevalence | Male | Sri Lanka | T1D | 1.07<br>(0.99 to 1.14) | < 0.001 |
| Middle-aged and elderly age | Prevalence | Both | Sri Lanka | IBD | 1.28<br>(1.23 to 1.34) | < 0.001 |
| Middle-aged and elderly age | Prevalence | Female | Sri Lanka | IBD | 1.39<br>(1.3 to 1.49) | < 0.001 |
| Middle-aged and elderly age | Prevalence | Male | Sri Lanka | IBD | 1.11<br>(1.07 to 1.15) | < 0.001 |
| Middle-aged and elderly age | Prevalence | Both | Sri Lanka | MS | 0.28<br>(0.24 to 0.32) | < 0.001 |

| | | | | | | |
|---|---|---|---|---|---|---|
| Middle-aged and elderly age | Prevalence | Female | Sri Lanka | MS | 0.24 (0.18 to 0.3) | < 0.001 |
| Middle-aged and elderly age | Prevalence | Male | Sri Lanka | MS | 0.22 (0.17 to 0.26) | < 0.001 |
| Middle-aged and elderly age | Prevalence | Both | Sri Lanka | Psoriasis | -0.78 (-0.81 to -0.76) | < 0.001 |
| Middle-aged and elderly age | Prevalence | Female | Sri Lanka | Psoriasis | -0.77 (-0.79 to -0.76) | < 0.001 |
| Middle-aged and elderly age | Prevalence | Male | Sri Lanka | Psoriasis | -0.75 (-0.78 to -0.72) | < 0.001 |
| Middle-aged and elderly age | Prevalence | Both | Sri Lanka | RHD | -1.01 (-1.05 to -0.96) | < 0.001 |
| Middle-aged and elderly age | Prevalence | Female | Sri Lanka | RHD | -1.23 (-1.31 to -1.16) | < 0.001 |
| Middle-aged and elderly age | Prevalence | Male | Sri Lanka | RHD | -0.78 (-0.85 to -0.7) | < 0.001 |
| Middle-aged and elderly age | Prevalence | Both | Sri Lanka | RA | 0.65 (0.63 to 0.68) | < 0.001 |
| Middle-aged and elderly age | Prevalence | Female | Sri Lanka | RA | 0.69 (0.68 to 0.7) | < 0.001 |
| Middle-aged and elderly age | Prevalence | Male | Sri Lanka | RA | 0.44 (0.4 to 0.48) | < 0.001 |
| Middle-aged and elderly age | Prevalence | Both | Sudan | AA | -0.04 (-0.05 to -0.03) | < 0.001 |
| Middle-aged and elderly age | Prevalence | Female | Sudan | AA | 0 (0 to 0) | < 0.001 |
| Middle-aged and elderly age | Prevalence | Male | Sudan | AA | 0 (0 to 0) | 0.015 |
| Middle-aged and elderly age | Prevalence | Both | Sudan | T1D | 2.37 (2.28 to 2.45) | < 0.001 |
| Middle-aged and elderly age | Prevalence | Female | Sudan | T1D | 2.28 (2.22 to 2.34) | < 0.001 |
| Middle-aged and elderly age | Prevalence | Male | Sudan | T1D | 2.4 | < 0.001 |

| | | | | | | |
|---|---|---|---|---|---|---|
| | | | | | (2.29 to 2.5) | |
| Middle-aged and elderly age | Prevalence | Both | Sudan | IBD | 0.73 (0.68 to 0.79) | < 0.001 |
| Middle-aged and elderly age | Prevalence | Female | Sudan | IBD | 0.69 (0.62 to 0.76) | < 0.001 |
| Middle-aged and elderly age | Prevalence | Male | Sudan | IBD | 0.77 (0.63 to 0.92) | < 0.001 |
| Middle-aged and elderly age | Prevalence | Both | Sudan | MS | 0.75 (0.73 to 0.77) | < 0.001 |
| Middle-aged and elderly age | Prevalence | Female | Sudan | MS | 0.69 (0.66 to 0.72) | < 0.001 |
| Middle-aged and elderly age | Prevalence | Male | Sudan | MS | 0.91 (0.9 to 0.92) | < 0.001 |
| Middle-aged and elderly age | Prevalence | Both | Sudan | Psoriasis | -1.17 (-1.18 to -1.16) | < 0.001 |
| Middle-aged and elderly age | Prevalence | Female | Sudan | Psoriasis | -1.18 (-1.19 to -1.17) | < 0.001 |
| Middle-aged and elderly age | Prevalence | Male | Sudan | Psoriasis | -1.15 (-1.16 to -1.15) | < 0.001 |
| Middle-aged and elderly age | Prevalence | Both | Sudan | RHD | 0.2 (0.18 to 0.23) | < 0.001 |
| Middle-aged and elderly age | Prevalence | Female | Sudan | RHD | 0.22 (0.21 to 0.24) | < 0.001 |
| Middle-aged and elderly age | Prevalence | Male | Sudan | RHD | 0.22 (0.15 to 0.29) | < 0.001 |
| Middle-aged and elderly age | Prevalence | Both | Sudan | RA | 0.97 (0.91 to 1.03) | < 0.001 |
| Middle-aged and elderly age | Prevalence | Female | Sudan | RA | 1.04 (1 to 1.08) | < 0.001 |
| Middle-aged and elderly age | Prevalence | Male | Sudan | RA | 0.99 (0.92 to 1.06) | < 0.001 |
| Middle-aged and elderly age | Prevalence | Both | Suriname | AA | -0.01 (-0.01 to 0) | 0.071 |

| | | | | | | |
|---|---|---|---|---|---|---|
| Middle-aged and elderly age | Prevalence | Female | Suriname | AA | 0<br>(0 to 0) | < 0.001 |
| Middle-aged and elderly age | Prevalence | Male | Suriname | AA | 0<br>(0 to 0) | < 0.001 |
| Middle-aged and elderly age | Prevalence | Both | Suriname | T1D | 1.31<br>(1.29 to 1.34) | < 0.001 |
| Middle-aged and elderly age | Prevalence | Female | Suriname | T1D | 1.23<br>(1.2 to 1.25) | < 0.001 |
| Middle-aged and elderly age | Prevalence | Male | Suriname | T1D | 1.39<br>(1.37 to 1.4) | < 0.001 |
| Middle-aged and elderly age | Prevalence | Both | Suriname | IBD | 0.05<br>(-0.07 to 0.18) | 0.400 |
| Middle-aged and elderly age | Prevalence | Female | Suriname | IBD | 0.13<br>(0.05 to 0.22) | 0.002 |
| Middle-aged and elderly age | Prevalence | Male | Suriname | IBD | -0.01<br>(-0.12 to 0.1) | 0.840 |
| Middle-aged and elderly age | Prevalence | Both | Suriname | MS | 0.59<br>(0.57 to 0.62) | < 0.001 |
| Middle-aged and elderly age | Prevalence | Female | Suriname | MS | 0.6<br>(0.58 to 0.63) | < 0.001 |
| Middle-aged and elderly age | Prevalence | Male | Suriname | MS | 0.59<br>(0.55 to 0.63) | < 0.001 |
| Middle-aged and elderly age | Prevalence | Both | Suriname | Psoriasis | -0.4<br>(-0.44 to -0.35) | < 0.001 |
| Middle-aged and elderly age | Prevalence | Female | Suriname | Psoriasis | -0.4<br>(-0.42 to -0.37) | < 0.001 |
| Middle-aged and elderly age | Prevalence | Male | Suriname | Psoriasis | -0.4<br>(-0.43 to -0.37) | < 0.001 |
| Middle-aged and elderly age | Prevalence | Both | Suriname | RHD | 0.05<br>(0.04 to 0.07) | < 0.001 |
| Middle-aged and elderly age | Prevalence | Female | Suriname | RHD | 0.07<br>(0.06 to 0.09) | < 0.001 |
| Middle-aged and elderly age | Prevalence | Male | Suriname | RHD | 0.03 | 0.535 |

| | | | | | | |
|---|---|---|---|---|---|---|
| | | | | | (-0.05 to 0.1) | |
| Middle-aged and elderly age | Prevalence | Both | Suriname | RA | 0.85 (0.75 to 0.96) | < 0.001 |
| Middle-aged and elderly age | Prevalence | Female | Suriname | RA | 0.83 (0.75 to 0.9) | < 0.001 |
| Middle-aged and elderly age | Prevalence | Male | Suriname | RA | 0.93 (0.85 to 1) | < 0.001 |
| Middle-aged and elderly age | Prevalence | Both | Sweden | AA | -0.02 (-0.03 to -0.02) | < 0.001 |
| Middle-aged and elderly age | Prevalence | Female | Sweden | AA | 0 (0 to 0) | < 0.001 |
| Middle-aged and elderly age | Prevalence | Male | Sweden | AA | 0 (0 to 0) | < 0.001 |
| Middle-aged and elderly age | Prevalence | Both | Sweden | T1D | 0.44 (0.28 to 0.6) | < 0.001 |
| Middle-aged and elderly age | Prevalence | Female | Sweden | T1D | 0.72 (0.57 to 0.88) | < 0.001 |
| Middle-aged and elderly age | Prevalence | Male | Sweden | T1D | 0.2 (0.03 to 0.38) | 0.022 |
| Middle-aged and elderly age | Prevalence | Both | Sweden | IBD | 0.3 (0.19 to 0.41) | < 0.001 |
| Middle-aged and elderly age | Prevalence | Female | Sweden | IBD | 0.4 (0.29 to 0.52) | < 0.001 |
| Middle-aged and elderly age | Prevalence | Male | Sweden | IBD | 0.2 (0.1 to 0.31) | < 0.001 |
| Middle-aged and elderly age | Prevalence | Both | Sweden | MS | 0.69 (0.64 to 0.73) | < 0.001 |
| Middle-aged and elderly age | Prevalence | Female | Sweden | MS | 0.79 (0.71 to 0.87) | < 0.001 |
| Middle-aged and elderly age | Prevalence | Male | Sweden | MS | 0.53 (0.5 to 0.57) | < 0.001 |
| Middle-aged and elderly age | Prevalence | Both | Sweden | Psoriasis | -0.16 (-0.17 to -0.15) | < 0.001 |

| | | | | | | |
|---|---|---|---|---|---|---|
| Middle-aged and elderly age | Prevalence | Female | Sweden | Psoriasis | -0.15<br>(-0.19 to -0.11) | < 0.001 |
| Middle-aged and elderly age | Prevalence | Male | Sweden | Psoriasis | -0.17<br>(-0.2 to -0.15) | < 0.001 |
| Middle-aged and elderly age | Prevalence | Both | Sweden | RHD | -0.97<br>(-1.29 to -0.64) | < 0.001 |
| Middle-aged and elderly age | Prevalence | Female | Sweden | RHD | -1.07<br>(-1.39 to -0.75) | < 0.001 |
| Middle-aged and elderly age | Prevalence | Male | Sweden | RHD | -0.83<br>(-1.2 to -0.46) | < 0.001 |
| Middle-aged and elderly age | Prevalence | Both | Sweden | RA | 0.3<br>(0.26 to 0.34) | < 0.001 |
| Middle-aged and elderly age | Prevalence | Female | Sweden | RA | 0.28<br>(0.25 to 0.32) | < 0.001 |
| Middle-aged and elderly age | Prevalence | Male | Sweden | RA | 0.42<br>(0.39 to 0.45) | < 0.001 |
| Middle-aged and elderly age | Prevalence | Both | Switzerland | AA | -0.04<br>(-0.04 to -0.03) | < 0.001 |
| Middle-aged and elderly age | Prevalence | Female | Switzerland | AA | 0<br>(0 to 0) | < 0.001 |
| Middle-aged and elderly age | Prevalence | Male | Switzerland | AA | 0<br>(0 to 0) | < 0.001 |
| Middle-aged and elderly age | Prevalence | Both | Switzerland | T1D | 1.79<br>(1.76 to 1.82) | < 0.001 |
| Middle-aged and elderly age | Prevalence | Female | Switzerland | T1D | 1.79<br>(1.75 to 1.82) | < 0.001 |
| Middle-aged and elderly age | Prevalence | Male | Switzerland | T1D | 1.76<br>(1.68 to 1.85) | < 0.001 |
| Middle-aged and elderly age | Prevalence | Both | Switzerland | IBD | 0.06<br>(0.04 to 0.08) | < 0.001 |
| Middle-aged and elderly age | Prevalence | Female | Switzerland | IBD | 0.03<br>(0 to 0.06) | 0.027 |
| Middle-aged and elderly age | Prevalence | Male | Switzerland | IBD | 0.09 | < 0.001 |

| | | | | | | |
|---|---|---|---|---|---|---|
| | | | | | (0.08 to 0.11) | |
| Middle-aged and elderly age | Prevalence | Both | Switzerland | MS | 0.18 (0.15 to 0.2) | < 0.001 |
| Middle-aged and elderly age | Prevalence | Female | Switzerland | MS | 0.3 (0.27 to 0.33) | < 0.001 |
| Middle-aged and elderly age | Prevalence | Male | Switzerland | MS | -0.01 (-0.04 to 0.03) | 0.657 |
| Middle-aged and elderly age | Prevalence | Both | Switzerland | Psoriasis | -0.25 (-0.25 to -0.24) | < 0.001 |
| Middle-aged and elderly age | Prevalence | Female | Switzerland | Psoriasis | -0.25 (-0.26 to -0.25) | < 0.001 |
| Middle-aged and elderly age | Prevalence | Male | Switzerland | Psoriasis | -0.25 (-0.26 to -0.25) | < 0.001 |
| Middle-aged and elderly age | Prevalence | Both | Switzerland | RHD | -1.12 (-1.25 to -0.98) | < 0.001 |
| Middle-aged and elderly age | Prevalence | Female | Switzerland | RHD | -0.98 (-1.23 to -0.74) | < 0.001 |
| Middle-aged and elderly age | Prevalence | Male | Switzerland | RHD | -1.17 (-1.46 to -0.87) | < 0.001 |
| Middle-aged and elderly age | Prevalence | Both | Switzerland | RA | 0.26 (0.18 to 0.35) | < 0.001 |
| Middle-aged and elderly age | Prevalence | Female | Switzerland | RA | 0.32 (0.24 to 0.4) | < 0.001 |
| Middle-aged and elderly age | Prevalence | Male | Switzerland | RA | 0.21 (0.12 to 0.3) | < 0.001 |
| Middle-aged and elderly age | Prevalence | Both | Syrian Arab Republic | AA | 0 (0 to 0) | 0.950 |
| Middle-aged and elderly age | Prevalence | Female | Syrian Arab Republic | AA | 0 (0 to 0) | < 0.001 |
| Middle-aged and elderly age | Prevalence | Male | Syrian Arab Republic | AA | 0 (0 to 0) | 0.015 |
| Middle-aged and elderly age | Prevalence | Both | Syrian Arab Republic | T1D | 2.46 (2.37 to 2.55) | < 0.001 |

| | | | | | | |
|---|---|---|---|---|---|---|
| Middle-aged and elderly age | Prevalence | Female | Syrian Arab Republic | T1D | 2.44 (2.35 to 2.53) | < 0.001 |
| Middle-aged and elderly age | Prevalence | Male | Syrian Arab Republic | T1D | 2.47 (2.38 to 2.56) | < 0.001 |
| Middle-aged and elderly age | Prevalence | Both | Syrian Arab Republic | IBD | 0.57 (0.45 to 0.7) | < 0.001 |
| Middle-aged and elderly age | Prevalence | Female | Syrian Arab Republic | IBD | 0.56 (0.45 to 0.68) | < 0.001 |
| Middle-aged and elderly age | Prevalence | Male | Syrian Arab Republic | IBD | 0.56 (0.44 to 0.68) | < 0.001 |
| Middle-aged and elderly age | Prevalence | Both | Syrian Arab Republic | MS | 0.7 (0.63 to 0.77) | < 0.001 |
| Middle-aged and elderly age | Prevalence | Female | Syrian Arab Republic | MS | 0.64 (0.54 to 0.74) | < 0.001 |
| Middle-aged and elderly age | Prevalence | Male | Syrian Arab Republic | MS | 0.84 (0.78 to 0.89) | < 0.001 |
| Middle-aged and elderly age | Prevalence | Both | Syrian Arab Republic | Psoriasis | -0.97 (-0.98 to -0.97) | < 0.001 |
| Middle-aged and elderly age | Prevalence | Female | Syrian Arab Republic | Psoriasis | -0.99 (-1 to -0.97) | < 0.001 |
| Middle-aged and elderly age | Prevalence | Male | Syrian Arab Republic | Psoriasis | -0.96 (-0.97 to -0.95) | < 0.001 |
| Middle-aged and elderly age | Prevalence | Both | Syrian Arab Republic | RHD | 0.1 (0.08 to 0.11) | < 0.001 |
| Middle-aged and elderly age | Prevalence | Female | Syrian Arab Republic | RHD | 0.12 (0.11 to 0.13) | < 0.001 |
| Middle-aged and elderly age | Prevalence | Male | Syrian Arab Republic | RHD | 0.07 (0.06 to 0.09) | < 0.001 |
| Middle-aged and elderly age | Prevalence | Both | Syrian Arab Republic | RA | 0.94 (0.84 to 1.05) | < 0.001 |
| Middle-aged and elderly age | Prevalence | Female | Syrian Arab Republic | RA | 0.99 (0.88 to 1.1) | < 0.001 |
| Middle-aged and elderly age | Prevalence | Male | Syrian Arab Republic | RA | 0.85 | < 0.001 |

| | | | | | | |
|---|---|---|---|---|---|---|
| | | | | | (0.71 to 0.98) | |
| Middle-aged and elderly age | Prevalence | Both | Taiwan (Province of China) | AA | 0.05<br>(0.04 to 0.05) | < 0.001 |
| Middle-aged and elderly age | Prevalence | Female | Taiwan (Province of China) | AA | 0<br>(0 to 0) | < 0.001 |
| Middle-aged and elderly age | Prevalence | Male | Taiwan (Province of China) | AA | 0<br>(0 to 0) | < 0.001 |
| Middle-aged and elderly age | Prevalence | Both | Taiwan (Province of China) | T1D | 2.43<br>(2.4 to 2.47) | < 0.001 |
| Middle-aged and elderly age | Prevalence | Female | Taiwan (Province of China) | T1D | 2.58<br>(2.52 to 2.65) | < 0.001 |
| Middle-aged and elderly age | Prevalence | Male | Taiwan (Province of China) | T1D | 2.3<br>(2.27 to 2.33) | < 0.001 |
| Middle-aged and elderly age | Prevalence | Both | Taiwan (Province of China) | IBD | 3.24<br>(2.47 to 4.02) | < 0.001 |
| Middle-aged and elderly age | Prevalence | Female | Taiwan (Province of China) | IBD | 3.33<br>(2.57 to 4.09) | < 0.001 |
| Middle-aged and elderly age | Prevalence | Male | Taiwan (Province of China) | IBD | 3.17<br>(2.39 to 3.96) | < 0.001 |
| Middle-aged and elderly age | Prevalence | Both | Taiwan (Province of China) | MS | 3.14<br>(3.03 to 3.26) | < 0.001 |
| Middle-aged and elderly age | Prevalence | Female | Taiwan (Province of China) | MS | 3.37<br>(3.28 to 3.46) | < 0.001 |
| Middle-aged and elderly age | Prevalence | Male | Taiwan (Province of China) | MS | 2.65<br>(2.5 to 2.81) | < 0.001 |
| Middle-aged and elderly age | Prevalence | Both | Taiwan (Province of China) | Psoriasis | -1.11<br>(-1.15 to -1.07) | < 0.001 |
| Middle-aged and elderly age | Prevalence | Female | Taiwan (Province of China) | Psoriasis | -0.98<br>(-1.03 to -0.93) | < 0.001 |
| Middle-aged and elderly age | Prevalence | Male | Taiwan (Province of China) | Psoriasis | -1.11<br>(-1.15 to -1.08) | < 0.001 |
| Middle-aged and elderly age | Prevalence | Both | Taiwan (Province of China) | RHD | -1.07<br>(-1.26 to -0.88) | < 0.001 |

| | | | | | | |
|---|---|---|---|---|---|---|
| Middle-aged and elderly age | Prevalence | Female | Taiwan (Province of China) | RHD | -1.44<br>(-1.57 to -1.3) | < 0.001 |
| Middle-aged and elderly age | Prevalence | Male | Taiwan (Province of China) | RHD | -0.53<br>(-0.78 to -0.28) | < 0.001 |
| Middle-aged and elderly age | Prevalence | Both | Taiwan (Province of China) | RA | 0.16<br>(0.1 to 0.22) | < 0.001 |
| Middle-aged and elderly age | Prevalence | Female | Taiwan (Province of China) | RA | 0.06<br>(0.05 to 0.08) | < 0.001 |
| Middle-aged and elderly age | Prevalence | Male | Taiwan (Province of China) | RA | 0.09<br>(0.06 to 0.12) | < 0.001 |
| Middle-aged and elderly age | Prevalence | Both | Tajikistan | AA | 0<br>(-0.01 to 0.01) | 0.908 |
| Middle-aged and elderly age | Prevalence | Female | Tajikistan | AA | 0<br>(0 to 0) | < 0.001 |
| Middle-aged and elderly age | Prevalence | Male | Tajikistan | AA | 0<br>(0 to 0) | < 0.001 |
| Middle-aged and elderly age | Prevalence | Both | Tajikistan | T1D | 1.03<br>(1.02 to 1.04) | < 0.001 |
| Middle-aged and elderly age | Prevalence | Female | Tajikistan | T1D | 1.01<br>(1 to 1.03) | < 0.001 |
| Middle-aged and elderly age | Prevalence | Male | Tajikistan | T1D | 1.05<br>(1.03 to 1.06) | < 0.001 |
| Middle-aged and elderly age | Prevalence | Both | Tajikistan | IBD | 0.1<br>(0.03 to 0.17) | 0.003 |
| Middle-aged and elderly age | Prevalence | Female | Tajikistan | IBD | 0.05<br>(-0.15 to 0.25) | 0.629 |
| Middle-aged and elderly age | Prevalence | Male | Tajikistan | IBD | 0.14<br>(0.06 to 0.22) | < 0.001 |
| Middle-aged and elderly age | Prevalence | Both | Tajikistan | MS | 0.3<br>(0.17 to 0.44) | < 0.001 |
| Middle-aged and elderly age | Prevalence | Female | Tajikistan | MS | 0.31<br>(0.18 to 0.44) | < 0.001 |
| Middle-aged and elderly age | Prevalence | Male | Tajikistan | MS | 0.27 | 0.007 |

| | | | | | | |
|---|---|---|---|---|---|---|
| | | | | | (0.07 to 0.46) | |
| Middle-aged and elderly age | Prevalence | Both | Tajikistan | Psoriasis | -0.66<br>(-0.77 to -0.55) | < 0.001 |
| Middle-aged and elderly age | Prevalence | Female | Tajikistan | Psoriasis | -0.67<br>(-0.79 to -0.56) | < 0.001 |
| Middle-aged and elderly age | Prevalence | Male | Tajikistan | Psoriasis | -0.64<br>(-0.74 to -0.54) | < 0.001 |
| Middle-aged and elderly age | Prevalence | Both | Tajikistan | RHD | 0.02<br>(0 to 0.04) | 0.096 |
| Middle-aged and elderly age | Prevalence | Female | Tajikistan | RHD | 0.03<br>(0.02 to 0.04) | < 0.001 |
| Middle-aged and elderly age | Prevalence | Male | Tajikistan | RHD | 0.01<br>(-0.02 to 0.03) | 0.569 |
| Middle-aged and elderly age | Prevalence | Both | Tajikistan | RA | 0.59<br>(0.56 to 0.62) | < 0.001 |
| Middle-aged and elderly age | Prevalence | Female | Tajikistan | RA | 0.61<br>(0.59 to 0.63) | < 0.001 |
| Middle-aged and elderly age | Prevalence | Male | Tajikistan | RA | 0.57<br>(0.53 to 0.6) | < 0.001 |
| Middle-aged and elderly age | Prevalence | Both | Thailand | AA | 0.01<br>(0 to 0.01) | 0.010 |
| Middle-aged and elderly age | Prevalence | Female | Thailand | AA | 0<br>(0 to 0) | < 0.001 |
| Middle-aged and elderly age | Prevalence | Male | Thailand | AA | 0<br>(0 to 0) | < 0.001 |
| Middle-aged and elderly age | Prevalence | Both | Thailand | T1D | 1.01<br>(0.99 to 1.03) | < 0.001 |
| Middle-aged and elderly age | Prevalence | Female | Thailand | T1D | 0.94<br>(0.93 to 0.95) | < 0.001 |
| Middle-aged and elderly age | Prevalence | Male | Thailand | T1D | 1.08<br>(1.04 to 1.12) | < 0.001 |
| Middle-aged and elderly age | Prevalence | Both | Thailand | IBD | 0.86<br>(0.81 to 0.9) | < 0.001 |

| | | | | | | |
|---|---|---|---|---|---|---|
| Middle-aged and elderly age | Prevalence | Female | Thailand | IBD | 0.82 (0.76 to 0.88) | < 0.001 |
| Middle-aged and elderly age | Prevalence | Male | Thailand | IBD | 0.89 (0.85 to 0.94) | < 0.001 |
| Middle-aged and elderly age | Prevalence | Both | Thailand | MS | 0.25 (0.23 to 0.28) | < 0.001 |
| Middle-aged and elderly age | Prevalence | Female | Thailand | MS | 0.26 (0.24 to 0.29) | < 0.001 |
| Middle-aged and elderly age | Prevalence | Male | Thailand | MS | 0.22 (0.2 to 0.25) | < 0.001 |
| Middle-aged and elderly age | Prevalence | Both | Thailand | Psoriasis | -0.51 (-0.53 to -0.5) | < 0.001 |
| Middle-aged and elderly age | Prevalence | Female | Thailand | Psoriasis | -0.5 (-0.52 to -0.48) | < 0.001 |
| Middle-aged and elderly age | Prevalence | Male | Thailand | Psoriasis | -0.53 (-0.58 to -0.49) | < 0.001 |
| Middle-aged and elderly age | Prevalence | Both | Thailand | RHD | 0 (-0.01 to 0.01) | 0.790 |
| Middle-aged and elderly age | Prevalence | Female | Thailand | RHD | 0.01 (0 to 0.02) | 0.065 |
| Middle-aged and elderly age | Prevalence | Male | Thailand | RHD | -0.02 (-0.03 to -0.01) | < 0.001 |
| Middle-aged and elderly age | Prevalence | Both | Thailand | RA | 0.57 (0.29 to 0.85) | < 0.001 |
| Middle-aged and elderly age | Prevalence | Female | Thailand | RA | 0.62 (0.46 to 0.79) | < 0.001 |
| Middle-aged and elderly age | Prevalence | Male | Thailand | RA | 0.39 (0.11 to 0.67) | 0.006 |
| Middle-aged and elderly age | Prevalence | Both | Timor-Leste | AA | -0.02 (-0.03 to -0.01) | < 0.001 |
| Middle-aged and elderly age | Prevalence | Female | Timor-Leste | AA | 0 (0 to 0) | < 0.001 |
| Middle-aged and elderly age | Prevalence | Male | Timor-Leste | AA | 0 | < 0.001 |

| | | | | | | |
|---|---|---|---|---|---|---|
| | | | | | (0 to 0) | |
| Middle-aged and elderly age | Prevalence | Both | Timor-Leste | T1D | 0.88<br>(0.87 to 0.89) | < 0.001 |
| Middle-aged and elderly age | Prevalence | Female | Timor-Leste | T1D | 0.82<br>(0.78 to 0.85) | < 0.001 |
| Middle-aged and elderly age | Prevalence | Male | Timor-Leste | T1D | 0.92<br>(0.91 to 0.94) | < 0.001 |
| Middle-aged and elderly age | Prevalence | Both | Timor-Leste | IBD | 0.49<br>(0.37 to 0.61) | < 0.001 |
| Middle-aged and elderly age | Prevalence | Female | Timor-Leste | IBD | 0.39<br>(0.35 to 0.43) | < 0.001 |
| Middle-aged and elderly age | Prevalence | Male | Timor-Leste | IBD | 0.64<br>(0.56 to 0.72) | < 0.001 |
| Middle-aged and elderly age | Prevalence | Both | Timor-Leste | MS | 0.32<br>(0.25 to 0.39) | < 0.001 |
| Middle-aged and elderly age | Prevalence | Female | Timor-Leste | MS | 0.38<br>(0.29 to 0.47) | < 0.001 |
| Middle-aged and elderly age | Prevalence | Male | Timor-Leste | MS | 0.33<br>(0.25 to 0.41) | < 0.001 |
| Middle-aged and elderly age | Prevalence | Both | Timor-Leste | Psoriasis | -0.73<br>(-0.79 to -0.67) | < 0.001 |
| Middle-aged and elderly age | Prevalence | Female | Timor-Leste | Psoriasis | -0.72<br>(-0.78 to -0.66) | < 0.001 |
| Middle-aged and elderly age | Prevalence | Male | Timor-Leste | Psoriasis | -0.75<br>(-0.82 to -0.67) | < 0.001 |
| Middle-aged and elderly age | Prevalence | Both | Timor-Leste | RHD | 0.14<br>(0.11 to 0.16) | < 0.001 |
| Middle-aged and elderly age | Prevalence | Female | Timor-Leste | RHD | 0.14<br>(0.13 to 0.16) | < 0.001 |
| Middle-aged and elderly age | Prevalence | Male | Timor-Leste | RHD | 0.16<br>(0.14 to 0.17) | < 0.001 |
| Middle-aged and elderly age | Prevalence | Both | Timor-Leste | RA | 0.57<br>(0.52 to 0.61) | < 0.001 |

| | | | | | | |
|---|---|---|---|---|---|---|
| Middle-aged and elderly age | Prevalence | Female | Timor-Leste | RA | 0.67 (0.63 to 0.72) | < 0.001 |
| Middle-aged and elderly age | Prevalence | Male | Timor-Leste | RA | 0.46 (0.43 to 0.49) | < 0.001 |
| Middle-aged and elderly age | Prevalence | Both | Togo | AA | -0.01 (-0.05 to 0.02) | 0.462 |
| Middle-aged and elderly age | Prevalence | Female | Togo | AA | 0 (0 to 0) | 0.027 |
| Middle-aged and elderly age | Prevalence | Male | Togo | AA | 0 (0 to 0) | < 0.001 |
| Middle-aged and elderly age | Prevalence | Both | Togo | T1D | 0.47 (0.46 to 0.48) | < 0.001 |
| Middle-aged and elderly age | Prevalence | Female | Togo | T1D | 0.47 (0.46 to 0.48) | < 0.001 |
| Middle-aged and elderly age | Prevalence | Male | Togo | T1D | 0.48 (0.46 to 0.49) | < 0.001 |
| Middle-aged and elderly age | Prevalence | Both | Togo | IBD | -0.26 (-0.38 to -0.14) | < 0.001 |
| Middle-aged and elderly age | Prevalence | Female | Togo | IBD | -0.39 (-0.52 to -0.26) | < 0.001 |
| Middle-aged and elderly age | Prevalence | Male | Togo | IBD | -0.1 (-0.22 to 0.02) | 0.089 |
| Middle-aged and elderly age | Prevalence | Both | Togo | MS | 0.18 (0.09 to 0.28) | < 0.001 |
| Middle-aged and elderly age | Prevalence | Female | Togo | MS | 0.16 (0.14 to 0.17) | < 0.001 |
| Middle-aged and elderly age | Prevalence | Male | Togo | MS | 0.35 (0.28 to 0.42) | < 0.001 |
| Middle-aged and elderly age | Prevalence | Both | Togo | Psoriasis | -0.58 (-0.71 to -0.45) | < 0.001 |
| Middle-aged and elderly age | Prevalence | Female | Togo | Psoriasis | -0.65 (-0.76 to -0.53) | < 0.001 |
| Middle-aged and elderly age | Prevalence | Male | Togo | Psoriasis | -0.49 | < 0.001 |

| | | | | | | |
|---|---|---|---|---|---|---|
| | | | | | (-0.64 to -0.33) | |
| Middle-aged and elderly age | Prevalence | Both | Togo | RHD | 0.13<br>(0.11 to 0.16) | < 0.001 |
| Middle-aged and elderly age | Prevalence | Female | Togo | RHD | 0.16<br>(0.15 to 0.17) | < 0.001 |
| Middle-aged and elderly age | Prevalence | Male | Togo | RHD | 0.09<br>(0.07 to 0.1) | < 0.001 |
| Middle-aged and elderly age | Prevalence | Both | Togo | RA | 0.47<br>(0.42 to 0.53) | < 0.001 |
| Middle-aged and elderly age | Prevalence | Female | Togo | RA | 0.44<br>(0.4 to 0.48) | < 0.001 |
| Middle-aged and elderly age | Prevalence | Male | Togo | RA | 0.56<br>(0.51 to 0.61) | < 0.001 |
| Middle-aged and elderly age | Prevalence | Both | Tokelau | AA | -0.11<br>(-0.25 to 0.04) | 0.152 |
| Middle-aged and elderly age | Prevalence | Female | Tokelau | AA | 0<br>(0 to 0) | < 0.001 |
| Middle-aged and elderly age | Prevalence | Male | Tokelau | AA | 0<br>(0 to 0) | < 0.001 |
| Middle-aged and elderly age | Prevalence | Both | Tokelau | T1D | 0.79<br>(0.68 to 0.9) | < 0.001 |
| Middle-aged and elderly age | Prevalence | Female | Tokelau | T1D | 0.75<br>(0.61 to 0.88) | < 0.001 |
| Middle-aged and elderly age | Prevalence | Male | Tokelau | T1D | 0.78<br>(0.69 to 0.86) | < 0.001 |
| Middle-aged and elderly age | Prevalence | Both | Tokelau | IBD | 0.18<br>(0.01 to 0.34) | 0.035 |
| Middle-aged and elderly age | Prevalence | Female | Tokelau | IBD | -0.21<br>(-0.45 to 0.03) | 0.080 |
| Middle-aged and elderly age | Prevalence | Male | Tokelau | IBD | 0.52<br>(0.37 to 0.67) | < 0.001 |
| Middle-aged and elderly age | Prevalence | Both | Tokelau | MS | 0.19<br>(0.06 to 0.32) | 0.004 |

| | | | | | | |
|---|---|---|---|---|---|---|
| Middle-aged and elderly age | Prevalence | Female | Tokelau | MS | 0.26 (0.23 to 0.29) | < 0.001 |
| Middle-aged and elderly age | Prevalence | Male | Tokelau | MS | 0.28 (0.24 to 0.32) | < 0.001 |
| Middle-aged and elderly age | Prevalence | Both | Tokelau | Psoriasis | -0.6 (-0.67 to -0.54) | < 0.001 |
| Middle-aged and elderly age | Prevalence | Female | Tokelau | Psoriasis | -0.65 (-0.66 to -0.64) | < 0.001 |
| Middle-aged and elderly age | Prevalence | Male | Tokelau | Psoriasis | -0.68 (-0.71 to -0.65) | < 0.001 |
| Middle-aged and elderly age | Prevalence | Both | Tokelau | RHD | -0.9 (-1 to -0.8) | < 0.001 |
| Middle-aged and elderly age | Prevalence | Female | Tokelau | RHD | -0.99 (-1.09 to -0.88) | < 0.001 |
| Middle-aged and elderly age | Prevalence | Male | Tokelau | RHD | -0.6 (-0.66 to -0.53) | < 0.001 |
| Middle-aged and elderly age | Prevalence | Both | Tokelau | RA | 0.5 (0.27 to 0.72) | < 0.001 |
| Middle-aged and elderly age | Prevalence | Female | Tokelau | RA | 0.71 (0.63 to 0.79) | < 0.001 |
| Middle-aged and elderly age | Prevalence | Male | Tokelau | RA | 0.58 (0.53 to 0.64) | < 0.001 |
| Middle-aged and elderly age | Prevalence | Both | Tonga | AA | -0.04 (-0.04 to -0.03) | < 0.001 |
| Middle-aged and elderly age | Prevalence | Female | Tonga | AA | 0 (0 to 0) | < 0.001 |
| Middle-aged and elderly age | Prevalence | Male | Tonga | AA | 0 (0 to 0) | < 0.001 |
| Middle-aged and elderly age | Prevalence | Both | Tonga | T1D | 0.92 (0.91 to 0.93) | < 0.001 |
| Middle-aged and elderly age | Prevalence | Female | Tonga | T1D | 0.89 (0.88 to 0.9) | < 0.001 |
| Middle-aged and elderly age | Prevalence | Male | Tonga | T1D | 0.93 | < 0.001 |

| | | | | | | |
|---|---|---|---|---|---|---|
| | | | | | (0.9 to 0.95) | |
| Middle-aged and elderly age | Prevalence | Both | Tonga | IBD | -0.53<br>(-0.6 to -0.45) | < 0.001 |
| Middle-aged and elderly age | Prevalence | Female | Tonga | IBD | -0.51<br>(-0.59 to -0.42) | < 0.001 |
| Middle-aged and elderly age | Prevalence | Male | Tonga | IBD | -0.5<br>(-0.63 to -0.38) | < 0.001 |
| Middle-aged and elderly age | Prevalence | Both | Tonga | MS | 0.08<br>(0.06 to 0.09) | < 0.001 |
| Middle-aged and elderly age | Prevalence | Female | Tonga | MS | 0.11<br>(0.09 to 0.12) | < 0.001 |
| Middle-aged and elderly age | Prevalence | Male | Tonga | MS | 0.08<br>(0.03 to 0.13) | 0.001 |
| Middle-aged and elderly age | Prevalence | Both | Tonga | Psoriasis | -0.45<br>(-0.48 to -0.43) | < 0.001 |
| Middle-aged and elderly age | Prevalence | Female | Tonga | Psoriasis | -0.44<br>(-0.48 to -0.41) | < 0.001 |
| Middle-aged and elderly age | Prevalence | Male | Tonga | Psoriasis | -0.48<br>(-0.5 to -0.45) | < 0.001 |
| Middle-aged and elderly age | Prevalence | Both | Tonga | RHD | 0.1<br>(-0.02 to 0.21) | 0.090 |
| Middle-aged and elderly age | Prevalence | Female | Tonga | RHD | 0.14<br>(0.04 to 0.24) | 0.008 |
| Middle-aged and elderly age | Prevalence | Male | Tonga | RHD | 0.05<br>(-0.01 to 0.12) | 0.123 |
| Middle-aged and elderly age | Prevalence | Both | Tonga | RA | 0.27<br>(0.21 to 0.33) | < 0.001 |
| Middle-aged and elderly age | Prevalence | Female | Tonga | RA | 0.31<br>(0.26 to 0.36) | < 0.001 |
| Middle-aged and elderly age | Prevalence | Male | Tonga | RA | 0.33<br>(0.25 to 0.41) | < 0.001 |
| Middle-aged and elderly age | Prevalence | Both | Trinidad and Tobago | AA | -0.02<br>(-0.03 to -0.02) | < 0.001 |

| | | | | | | |
|---|---|---|---|---|---|---|
| Middle-aged and elderly age | Prevalence | Female | Trinidad and Tobago | AA | 0<br>(0 to 0) | < 0.001 |
| Middle-aged and elderly age | Prevalence | Male | Trinidad and Tobago | AA | 0<br>(0 to 0) | < 0.001 |
| Middle-aged and elderly age | Prevalence | Both | Trinidad and Tobago | T1D | 1.28<br>(1.27 to 1.29) | < 0.001 |
| Middle-aged and elderly age | Prevalence | Female | Trinidad and Tobago | T1D | 1.21<br>(1.2 to 1.22) | < 0.001 |
| Middle-aged and elderly age | Prevalence | Male | Trinidad and Tobago | T1D | 1.33<br>(1.32 to 1.34) | < 0.001 |
| Middle-aged and elderly age | Prevalence | Both | Trinidad and Tobago | IBD | 0.1<br>(0.07 to 0.13) | < 0.001 |
| Middle-aged and elderly age | Prevalence | Female | Trinidad and Tobago | IBD | 0.15<br>(0.07 to 0.23) | < 0.001 |
| Middle-aged and elderly age | Prevalence | Male | Trinidad and Tobago | IBD | 0.05<br>(0.01 to 0.09) | 0.007 |
| Middle-aged and elderly age | Prevalence | Both | Trinidad and Tobago | MS | 0.59<br>(0.57 to 0.62) | < 0.001 |
| Middle-aged and elderly age | Prevalence | Female | Trinidad and Tobago | MS | 0.77<br>(0.71 to 0.84) | < 0.001 |
| Middle-aged and elderly age | Prevalence | Male | Trinidad and Tobago | MS | 0.32<br>(0.24 to 0.39) | < 0.001 |
| Middle-aged and elderly age | Prevalence | Both | Trinidad and Tobago | Psoriasis | -0.4<br>(-0.42 to -0.38) | < 0.001 |
| Middle-aged and elderly age | Prevalence | Female | Trinidad and Tobago | Psoriasis | -0.4<br>(-0.41 to -0.39) | < 0.001 |
| Middle-aged and elderly age | Prevalence | Male | Trinidad and Tobago | Psoriasis | -0.39<br>(-0.42 to -0.37) | < 0.001 |
| Middle-aged and elderly age | Prevalence | Both | Trinidad and Tobago | RHD | 0.07<br>(0.05 to 0.08) | < 0.001 |
| Middle-aged and elderly age | Prevalence | Female | Trinidad and Tobago | RHD | 0.1<br>(0.09 to 0.11) | < 0.001 |
| Middle-aged and elderly age | Prevalence | Male | Trinidad and Tobago | RHD | 0.05 | < 0.001 |

| | | | | | | |
|---|---|---|---|---|---|---|
| | | | | | (0.04 to 0.06) | |
| Middle-aged and elderly age | Prevalence | Both | Trinidad and Tobago | RA | 0.67<br>(0.62 to 0.72) | < 0.001 |
| Middle-aged and elderly age | Prevalence | Female | Trinidad and Tobago | RA | 0.67<br>(0.6 to 0.73) | < 0.001 |
| Middle-aged and elderly age | Prevalence | Male | Trinidad and Tobago | RA | 0.91<br>(0.87 to 0.96) | < 0.001 |
| Middle-aged and elderly age | Prevalence | Both | Tunisia | AA | 0.01<br>(0.01 to 0.02) | < 0.001 |
| Middle-aged and elderly age | Prevalence | Female | Tunisia | AA | 0<br>(0 to 0) | < 0.001 |
| Middle-aged and elderly age | Prevalence | Male | Tunisia | AA | 0<br>(0 to 0) | 0.015 |
| Middle-aged and elderly age | Prevalence | Both | Tunisia | T1D | 2.91<br>(2.84 to 2.97) | < 0.001 |
| Middle-aged and elderly age | Prevalence | Female | Tunisia | T1D | 2.92<br>(2.85 to 3) | < 0.001 |
| Middle-aged and elderly age | Prevalence | Male | Tunisia | T1D | 2.9<br>(2.83 to 2.97) | < 0.001 |
| Middle-aged and elderly age | Prevalence | Both | Tunisia | IBD | 0.54<br>(0.51 to 0.58) | < 0.001 |
| Middle-aged and elderly age | Prevalence | Female | Tunisia | IBD | 0.53<br>(0.5 to 0.56) | < 0.001 |
| Middle-aged and elderly age | Prevalence | Male | Tunisia | IBD | 0.57<br>(0.51 to 0.62) | < 0.001 |
| Middle-aged and elderly age | Prevalence | Both | Tunisia | MS | 0.88<br>(0.86 to 0.9) | < 0.001 |
| Middle-aged and elderly age | Prevalence | Female | Tunisia | MS | 0.81<br>(0.78 to 0.84) | < 0.001 |
| Middle-aged and elderly age | Prevalence | Male | Tunisia | MS | 0.97<br>(0.96 to 0.99) | < 0.001 |
| Middle-aged and elderly age | Prevalence | Both | Tunisia | Psoriasis | -0.91<br>(-0.94 to -0.89) | < 0.001 |

| | | | | | | |
|---|---|---|---|---|---|---|
| Middle-aged and elderly age | Prevalence | Female | Tunisia | Psoriasis | -0.93 (-0.94 to -0.91) | < 0.001 |
| Middle-aged and elderly age | Prevalence | Male | Tunisia | Psoriasis | -0.9 (-0.93 to -0.87) | < 0.001 |
| Middle-aged and elderly age | Prevalence | Both | Tunisia | RHD | -0.86 (-0.91 to -0.81) | < 0.001 |
| Middle-aged and elderly age | Prevalence | Female | Tunisia | RHD | -1.04 (-1.15 to -0.93) | < 0.001 |
| Middle-aged and elderly age | Prevalence | Male | Tunisia | RHD | -0.62 (-0.67 to -0.57) | < 0.001 |
| Middle-aged and elderly age | Prevalence | Both | Tunisia | RA | 1.08 (1.01 to 1.14) | < 0.001 |
| Middle-aged and elderly age | Prevalence | Female | Tunisia | RA | 1.09 (1 to 1.19) | < 0.001 |
| Middle-aged and elderly age | Prevalence | Male | Tunisia | RA | 0.91 (0.85 to 0.97) | < 0.001 |
| Middle-aged and elderly age | Prevalence | Both | Turkey | AA | -0.01 (-0.05 to 0.04) | 0.808 |
| Middle-aged and elderly age | Prevalence | Female | Turkey | AA | 0 (0 to 0) | < 0.001 |
| Middle-aged and elderly age | Prevalence | Male | Turkey | AA | -0.01 (-0.01 to -0.01) | < 0.001 |
| Middle-aged and elderly age | Prevalence | Both | Turkey | T1D | 2.3 (2.27 to 2.33) | < 0.001 |
| Middle-aged and elderly age | Prevalence | Female | Turkey | T1D | 2.28 (2.25 to 2.3) | < 0.001 |
| Middle-aged and elderly age | Prevalence | Male | Turkey | T1D | 2.31 (2.28 to 2.34) | < 0.001 |
| Middle-aged and elderly age | Prevalence | Both | Turkey | IBD | 2.32 (1.55 to 3.09) | < 0.001 |
| Middle-aged and elderly age | Prevalence | Female | Turkey | IBD | 2.16 (1.72 to 2.6) | < 0.001 |
| Middle-aged and elderly age | Prevalence | Male | Turkey | IBD | 2.61 | < 0.001 |

| | | | | | | |
|---|---|---|---|---|---|---|
| | | | | | (1.62 to 3.61) | |
| Middle-aged and elderly age | Prevalence | Both | Turkey | MS | 0.1 (0.06 to 0.14) | < 0.001 |
| Middle-aged and elderly age | Prevalence | Female | Turkey | MS | 0.07 (0.06 to 0.08) | < 0.001 |
| Middle-aged and elderly age | Prevalence | Male | Turkey | MS | 0.13 (0.12 to 0.15) | < 0.001 |
| Middle-aged and elderly age | Prevalence | Both | Turkey | Psoriasis | -1 (-1.03 to -0.98) | < 0.001 |
| Middle-aged and elderly age | Prevalence | Female | Turkey | Psoriasis | -1.04 (-1.05 to -1.03) | < 0.001 |
| Middle-aged and elderly age | Prevalence | Male | Turkey | Psoriasis | -0.95 (-0.96 to -0.93) | < 0.001 |
| Middle-aged and elderly age | Prevalence | Both | Turkey | RHD | -1.06 (-1.19 to -0.92) | < 0.001 |
| Middle-aged and elderly age | Prevalence | Female | Turkey | RHD | -0.9 (-1.01 to -0.8) | < 0.001 |
| Middle-aged and elderly age | Prevalence | Male | Turkey | RHD | -1.24 (-1.42 to -1.07) | < 0.001 |
| Middle-aged and elderly age | Prevalence | Both | Turkey | RA | 1.2 (1.1 to 1.3) | < 0.001 |
| Middle-aged and elderly age | Prevalence | Female | Turkey | RA | 1.26 (1.15 to 1.36) | < 0.001 |
| Middle-aged and elderly age | Prevalence | Male | Turkey | RA | 1.15 (1.06 to 1.25) | < 0.001 |
| Middle-aged and elderly age | Prevalence | Both | Turkmenistan | AA | 0.04 (0.03 to 0.05) | < 0.001 |
| Middle-aged and elderly age | Prevalence | Female | Turkmenistan | AA | 0 (0 to 0) | < 0.001 |
| Middle-aged and elderly age | Prevalence | Male | Turkmenistan | AA | 0 (0 to 0) | < 0.001 |
| Middle-aged and elderly age | Prevalence | Both | Turkmenistan | T1D | 1.03 (1.01 to 1.05) | < 0.001 |

| | | | | | | |
|---|---|---|---|---|---|---|
| Middle-aged and elderly age | Prevalence | Female | Turkmenistan | T1D | 1.04 (1.02 to 1.06) | < 0.001 |
| Middle-aged and elderly age | Prevalence | Male | Turkmenistan | T1D | 1.06 (1.03 to 1.08) | < 0.001 |
| Middle-aged and elderly age | Prevalence | Both | Turkmenistan | IBD | 0.49 (0.19 to 0.79) | 0.001 |
| Middle-aged and elderly age | Prevalence | Female | Turkmenistan | IBD | 0.48 (0.22 to 0.74) | < 0.001 |
| Middle-aged and elderly age | Prevalence | Male | Turkmenistan | IBD | 0.51 (0.17 to 0.85) | 0.003 |
| Middle-aged and elderly age | Prevalence | Both | Turkmenistan | MS | 0.1 (0.08 to 0.13) | < 0.001 |
| Middle-aged and elderly age | Prevalence | Female | Turkmenistan | MS | -0.06 (-0.09 to -0.04) | < 0.001 |
| Middle-aged and elderly age | Prevalence | Male | Turkmenistan | MS | 0.29 (0.22 to 0.37) | < 0.001 |
| Middle-aged and elderly age | Prevalence | Both | Turkmenistan | Psoriasis | -1.01 (-1.09 to -0.92) | < 0.001 |
| Middle-aged and elderly age | Prevalence | Female | Turkmenistan | Psoriasis | -1.01 (-1.12 to -0.9) | < 0.001 |
| Middle-aged and elderly age | Prevalence | Male | Turkmenistan | Psoriasis | -0.98 (-1.01 to -0.95) | < 0.001 |
| Middle-aged and elderly age | Prevalence | Both | Turkmenistan | RHD | 0.11 (0.09 to 0.13) | < 0.001 |
| Middle-aged and elderly age | Prevalence | Female | Turkmenistan | RHD | 0.12 (0.09 to 0.14) | < 0.001 |
| Middle-aged and elderly age | Prevalence | Male | Turkmenistan | RHD | 0.07 (0.02 to 0.11) | 0.002 |
| Middle-aged and elderly age | Prevalence | Both | Turkmenistan | RA | 0.97 (0.88 to 1.07) | < 0.001 |
| Middle-aged and elderly age | Prevalence | Female | Turkmenistan | RA | 0.96 (0.88 to 1.05) | < 0.001 |
| Middle-aged and elderly age | Prevalence | Male | Turkmenistan | RA | 0.88 | < 0.001 |

| | | | | | | |
|---|---|---|---|---|---|---|
| | | | | | (0.79 to 0.97) | |
| Middle-aged and elderly age | Prevalence | Both | Tuvalu | AA | -0.12 (-0.13 to -0.11) | < 0.001 |
| Middle-aged and elderly age | Prevalence | Female | Tuvalu | AA | 0 (0 to 0) | < 0.001 |
| Middle-aged and elderly age | Prevalence | Male | Tuvalu | AA | 0 (0 to 0) | < 0.001 |
| Middle-aged and elderly age | Prevalence | Both | Tuvalu | T1D | 0.84 (0.83 to 0.86) | < 0.001 |
| Middle-aged and elderly age | Prevalence | Female | Tuvalu | T1D | 0.78 (0.75 to 0.8) | < 0.001 |
| Middle-aged and elderly age | Prevalence | Male | Tuvalu | T1D | 0.84 (0.82 to 0.86) | < 0.001 |
| Middle-aged and elderly age | Prevalence | Both | Tuvalu | IBD | -0.48 (-0.59 to -0.38) | < 0.001 |
| Middle-aged and elderly age | Prevalence | Female | Tuvalu | IBD | -0.66 (-0.77 to -0.56) | < 0.001 |
| Middle-aged and elderly age | Prevalence | Male | Tuvalu | IBD | -0.36 (-0.46 to -0.27) | < 0.001 |
| Middle-aged and elderly age | Prevalence | Both | Tuvalu | MS | 0.11 (0.04 to 0.18) | 0.001 |
| Middle-aged and elderly age | Prevalence | Female | Tuvalu | MS | 0.22 (0.16 to 0.29) | < 0.001 |
| Middle-aged and elderly age | Prevalence | Male | Tuvalu | MS | 0.2 (0.09 to 0.3) | < 0.001 |
| Middle-aged and elderly age | Prevalence | Both | Tuvalu | Psoriasis | -0.5 (-0.53 to -0.47) | < 0.001 |
| Middle-aged and elderly age | Prevalence | Female | Tuvalu | Psoriasis | -0.55 (-0.57 to -0.54) | < 0.001 |
| Middle-aged and elderly age | Prevalence | Male | Tuvalu | Psoriasis | -0.57 (-0.6 to -0.55) | < 0.001 |
| Middle-aged and elderly age | Prevalence | Both | Tuvalu | RHD | -0.66 (-0.75 to -0.57) | < 0.001 |

| | | | | | | |
|---|---|---|---|---|---|---|
| Middle-aged and elderly age | Prevalence | Female | Tuvalu | RHD | -0.65<br>(-0.77 to -0.52) | < 0.001 |
| Middle-aged and elderly age | Prevalence | Male | Tuvalu | RHD | -0.52<br>(-0.58 to -0.45) | < 0.001 |
| Middle-aged and elderly age | Prevalence | Both | Tuvalu | RA | 0.39<br>(0.36 to 0.42) | < 0.001 |
| Middle-aged and elderly age | Prevalence | Female | Tuvalu | RA | 0.57<br>(0.53 to 0.6) | < 0.001 |
| Middle-aged and elderly age | Prevalence | Male | Tuvalu | RA | 0.46<br>(0.41 to 0.51) | < 0.001 |
| Middle-aged and elderly age | Prevalence | Both | Uganda | AA | 0.06<br>(0.05 to 0.06) | < 0.001 |
| Middle-aged and elderly age | Prevalence | Female | Uganda | AA | 0<br>(0 to 0) | 1.000 |
| Middle-aged and elderly age | Prevalence | Male | Uganda | AA | 0<br>(0 to 0) | < 0.001 |
| Middle-aged and elderly age | Prevalence | Both | Uganda | T1D | 0.5<br>(0.5 to 0.51) | < 0.001 |
| Middle-aged and elderly age | Prevalence | Female | Uganda | T1D | 0.51<br>(0.5 to 0.52) | < 0.001 |
| Middle-aged and elderly age | Prevalence | Male | Uganda | T1D | 0.51<br>(0.5 to 0.52) | < 0.001 |
| Middle-aged and elderly age | Prevalence | Both | Uganda | IBD | 0.6<br>(0.51 to 0.69) | < 0.001 |
| Middle-aged and elderly age | Prevalence | Female | Uganda | IBD | 0.39<br>(0.31 to 0.48) | < 0.001 |
| Middle-aged and elderly age | Prevalence | Male | Uganda | IBD | 0.8<br>(0.74 to 0.87) | < 0.001 |
| Middle-aged and elderly age | Prevalence | Both | Uganda | MS | 0.3<br>(0.27 to 0.33) | < 0.001 |
| Middle-aged and elderly age | Prevalence | Female | Uganda | MS | 0.23<br>(0.21 to 0.26) | < 0.001 |
| Middle-aged and elderly age | Prevalence | Male | Uganda | MS | 0.27 | < 0.001 |

| | | | | | | |
|---|---|---|---|---|---|---|
| | | | | | (0.24 to 0.3) | |
| Middle-aged and elderly age | Prevalence | Both | Uganda | Psoriasis | -0.6 (-0.65 to -0.56) | < 0.001 |
| Middle-aged and elderly age | Prevalence | Female | Uganda | Psoriasis | -0.61 (-0.65 to -0.56) | < 0.001 |
| Middle-aged and elderly age | Prevalence | Male | Uganda | Psoriasis | -0.61 (-0.66 to -0.56) | < 0.001 |
| Middle-aged and elderly age | Prevalence | Both | Uganda | RHD | 0.12 (0.05 to 0.2) | 0.002 |
| Middle-aged and elderly age | Prevalence | Female | Uganda | RHD | 0.17 (0.1 to 0.24) | < 0.001 |
| Middle-aged and elderly age | Prevalence | Male | Uganda | RHD | 0.05 (-0.04 to 0.14) | 0.249 |
| Middle-aged and elderly age | Prevalence | Both | Uganda | RA | 0.66 (0.59 to 0.74) | < 0.001 |
| Middle-aged and elderly age | Prevalence | Female | Uganda | RA | 0.69 (0.61 to 0.76) | < 0.001 |
| Middle-aged and elderly age | Prevalence | Male | Uganda | RA | 0.73 (0.64 to 0.82) | < 0.001 |
| Middle-aged and elderly age | Prevalence | Both | Ukraine | AA | 0.02 (0.01 to 0.03) | < 0.001 |
| Middle-aged and elderly age | Prevalence | Female | Ukraine | AA | 0 (0 to 0) | < 0.001 |
| Middle-aged and elderly age | Prevalence | Male | Ukraine | AA | 0 (0 to 0) | < 0.001 |
| Middle-aged and elderly age | Prevalence | Both | Ukraine | T1D | 1.34 (1.27 to 1.42) | < 0.001 |
| Middle-aged and elderly age | Prevalence | Female | Ukraine | T1D | 1.4 (1.34 to 1.46) | < 0.001 |
| Middle-aged and elderly age | Prevalence | Male | Ukraine | T1D | 1.25 (1.18 to 1.32) | < 0.001 |
| Middle-aged and elderly age | Prevalence | Both | Ukraine | IBD | -0.03 (-0.08 to 0.03) | 0.305 |

| | | | | | | |
|---|---|---|---|---|---|---|
| Middle-aged and elderly age | Prevalence | Female | Ukraine | IBD | 0<br>(-0.05 to 0.04) | 0.837 |
| Middle-aged and elderly age | Prevalence | Male | Ukraine | IBD | -0.06<br>(-0.12 to 0) | 0.067 |
| Middle-aged and elderly age | Prevalence | Both | Ukraine | MS | -0.84<br>(-0.95 to -0.72) | < 0.001 |
| Middle-aged and elderly age | Prevalence | Female | Ukraine | MS | -0.8<br>(-0.88 to -0.71) | < 0.001 |
| Middle-aged and elderly age | Prevalence | Male | Ukraine | MS | -0.91<br>(-1.05 to -0.76) | < 0.001 |
| Middle-aged and elderly age | Prevalence | Both | Ukraine | Psoriasis | -0.52<br>(-0.54 to -0.5) | < 0.001 |
| Middle-aged and elderly age | Prevalence | Female | Ukraine | Psoriasis | -0.53<br>(-0.55 to -0.51) | < 0.001 |
| Middle-aged and elderly age | Prevalence | Male | Ukraine | Psoriasis | -0.52<br>(-0.54 to -0.5) | < 0.001 |
| Middle-aged and elderly age | Prevalence | Both | Ukraine | RHD | -1.36<br>(-1.47 to -1.26) | < 0.001 |
| Middle-aged and elderly age | Prevalence | Female | Ukraine | RHD | -1.45<br>(-1.62 to -1.27) | < 0.001 |
| Middle-aged and elderly age | Prevalence | Male | Ukraine | RHD | -1.27<br>(-1.34 to -1.2) | < 0.001 |
| Middle-aged and elderly age | Prevalence | Both | Ukraine | RA | 0.24<br>(0.09 to 0.39) | 0.002 |
| Middle-aged and elderly age | Prevalence | Female | Ukraine | RA | 0.23<br>(0.07 to 0.39) | 0.006 |
| Middle-aged and elderly age | Prevalence | Male | Ukraine | RA | 0.12<br>(0.02 to 0.22) | 0.017 |
| Middle-aged and elderly age | Prevalence | Both | United Arab Emirates | AA | -0.09<br>(-0.12 to -0.06) | < 0.001 |
| Middle-aged and elderly age | Prevalence | Female | United Arab Emirates | AA | 0<br>(0 to 0) | < 0.001 |
| Middle-aged and elderly age | Prevalence | Male | United Arab Emirates | AA | 0 | 0.015 |

| | | | | | | |
|---|---|---|---|---|---|---|
| | | | | | (0 to 0) | |
| Middle-aged and elderly age | Prevalence | Both | United Arab Emirates | T1D | 3.3 (3.2 to 3.4) | < 0.001 |
| Middle-aged and elderly age | Prevalence | Female | United Arab Emirates | T1D | 3.26 (3.14 to 3.37) | < 0.001 |
| Middle-aged and elderly age | Prevalence | Male | United Arab Emirates | T1D | 3.29 (3.17 to 3.41) | < 0.001 |
| Middle-aged and elderly age | Prevalence | Both | United Arab Emirates | IBD | 0.49 (0.38 to 0.59) | < 0.001 |
| Middle-aged and elderly age | Prevalence | Female | United Arab Emirates | IBD | 0.47 (0.38 to 0.57) | < 0.001 |
| Middle-aged and elderly age | Prevalence | Male | United Arab Emirates | IBD | 0.5 (0.34 to 0.66) | < 0.001 |
| Middle-aged and elderly age | Prevalence | Both | United Arab Emirates | MS | 0.01 (-0.1 to 0.13) | 0.810 |
| Middle-aged and elderly age | Prevalence | Female | United Arab Emirates | MS | 0.32 (0.25 to 0.39) | < 0.001 |
| Middle-aged and elderly age | Prevalence | Male | United Arab Emirates | MS | -0.04 (-0.19 to 0.11) | 0.581 |
| Middle-aged and elderly age | Prevalence | Both | United Arab Emirates | Psoriasis | -1.02 (-1.03 to -1) | < 0.001 |
| Middle-aged and elderly age | Prevalence | Female | United Arab Emirates | Psoriasis | -1 (-1.03 to -0.98) | < 0.001 |
| Middle-aged and elderly age | Prevalence | Male | United Arab Emirates | Psoriasis | -1.01 (-1.03 to -1) | < 0.001 |
| Middle-aged and elderly age | Prevalence | Both | United Arab Emirates | RHD | -0.08 (-0.08 to -0.07) | < 0.001 |
| Middle-aged and elderly age | Prevalence | Female | United Arab Emirates | RHD | -0.01 (-0.03 to 0) | 0.014 |
| Middle-aged and elderly age | Prevalence | Male | United Arab Emirates | RHD | -0.06 (-0.07 to -0.06) | < 0.001 |
| Middle-aged and elderly age | Prevalence | Both | United Arab Emirates | RA | 0.78 (0.7 to 0.87) | < 0.001 |

| | | | | | | |
|---|---|---|---|---|---|---|
| Middle-aged and elderly age | Prevalence | Female | United Arab Emirates | RA | 0.95<br>(0.9 to 1) | < 0.001 |
| Middle-aged and elderly age | Prevalence | Male | United Arab Emirates | RA | 0.9<br>(0.83 to 0.97) | < 0.001 |
| Middle-aged and elderly age | Prevalence | Both | United Kingdom | AA | 0.01<br>(0.01 to 0.01) | < 0.001 |
| Middle-aged and elderly age | Prevalence | Female | United Kingdom | AA | 0<br>(0 to 0) | 0.024 |
| Middle-aged and elderly age | Prevalence | Male | United Kingdom | AA | 0<br>(0 to 0) | 0.694 |
| Middle-aged and elderly age | Prevalence | Both | United Kingdom | T1D | 1.46<br>(1.36 to 1.57) | < 0.001 |
| Middle-aged and elderly age | Prevalence | Female | United Kingdom | T1D | 1.43<br>(1.29 to 1.58) | < 0.001 |
| Middle-aged and elderly age | Prevalence | Male | United Kingdom | T1D | 1.54<br>(1.38 to 1.7) | < 0.001 |
| Middle-aged and elderly age | Prevalence | Both | United Kingdom | IBD | 0.05<br>(0.02 to 0.08) | 0.002 |
| Middle-aged and elderly age | Prevalence | Female | United Kingdom | IBD | 0.07<br>(0.03 to 0.12) | 0.001 |
| Middle-aged and elderly age | Prevalence | Male | United Kingdom | IBD | 0.04<br>(0.02 to 0.07) | 0.001 |
| Middle-aged and elderly age | Prevalence | Both | United Kingdom | MS | 1.18<br>(1.12 to 1.24) | < 0.001 |
| Middle-aged and elderly age | Prevalence | Female | United Kingdom | MS | 1.28<br>(1.21 to 1.35) | < 0.001 |
| Middle-aged and elderly age | Prevalence | Male | United Kingdom | MS | 1<br>(0.94 to 1.05) | < 0.001 |
| Middle-aged and elderly age | Prevalence | Both | United Kingdom | Psoriasis | -0.42<br>(-0.44 to -0.4) | < 0.001 |
| Middle-aged and elderly age | Prevalence | Female | United Kingdom | Psoriasis | -0.43<br>(-0.45 to -0.4) | < 0.001 |
| Middle-aged and elderly age | Prevalence | Male | United Kingdom | Psoriasis | -0.41 | < 0.001 |

| | | | | | | |
|---|---|---|---|---|---|---|
| | | | | | (-0.42 to -0.39) | |
| Middle-aged and elderly age | Prevalence | Both | United Kingdom | RHD | -2.2<br>(-2.31 to -2.08) | < 0.001 |
| Middle-aged and elderly age | Prevalence | Female | United Kingdom | RHD | -2.52<br>(-2.65 to -2.4) | < 0.001 |
| Middle-aged and elderly age | Prevalence | Male | United Kingdom | RHD | -1.7<br>(-1.8 to -1.6) | < 0.001 |
| Middle-aged and elderly age | Prevalence | Both | United Kingdom | RA | -0.28<br>(-0.37 to -0.19) | < 0.001 |
| Middle-aged and elderly age | Prevalence | Female | United Kingdom | RA | -0.36<br>(-0.47 to -0.25) | < 0.001 |
| Middle-aged and elderly age | Prevalence | Male | United Kingdom | RA | -0.07<br>(-0.12 to -0.02) | 0.003 |
| Middle-aged and elderly age | Prevalence | Both | United Republic of Tanzania | AA | 0.01<br>(0 to 0.02) | 0.125 |
| Middle-aged and elderly age | Prevalence | Female | United Republic of Tanzania | AA | 0.04<br>(0.03 to 0.05) | < 0.001 |
| Middle-aged and elderly age | Prevalence | Male | United Republic of Tanzania | AA | -0.03<br>(-0.03 to -0.03) | < 0.001 |
| Middle-aged and elderly age | Prevalence | Both | United Republic of Tanzania | T1D | 0.44<br>(0.44 to 0.45) | < 0.001 |
| Middle-aged and elderly age | Prevalence | Female | United Republic of Tanzania | T1D | 0.45<br>(0.44 to 0.47) | < 0.001 |
| Middle-aged and elderly age | Prevalence | Male | United Republic of Tanzania | T1D | 0.43<br>(0.42 to 0.44) | < 0.001 |
| Middle-aged and elderly age | Prevalence | Both | United Republic of Tanzania | IBD | 0.09<br>(-0.03 to 0.21) | 0.126 |
| Middle-aged and elderly age | Prevalence | Female | United Republic of Tanzania | IBD | -0.04<br>(-0.17 to 0.09) | 0.543 |
| Middle-aged and elderly age | Prevalence | Male | United Republic of Tanzania | IBD | 0.31<br>(0.22 to 0.4) | < 0.001 |
| Middle-aged and elderly age | Prevalence | Both | United Republic of Tanzania | MS | 0.2<br>(0.18 to 0.22) | < 0.001 |

| | | | | | | |
|---|---|---|---|---|---|---|
| Middle-aged and elderly age | Prevalence | Female | United Republic of Tanzania | MS | 0.19<br>(0.16 to 0.22) | < 0.001 |
| Middle-aged and elderly age | Prevalence | Male | United Republic of Tanzania | MS | 0.27<br>(0.23 to 0.31) | < 0.001 |
| Middle-aged and elderly age | Prevalence | Both | United Republic of Tanzania | Psoriasis | -0.44<br>(-0.47 to -0.4) | < 0.001 |
| Middle-aged and elderly age | Prevalence | Female | United Republic of Tanzania | Psoriasis | -0.43<br>(-0.45 to -0.42) | < 0.001 |
| Middle-aged and elderly age | Prevalence | Male | United Republic of Tanzania | Psoriasis | -0.43<br>(-0.48 to -0.38) | < 0.001 |
| Middle-aged and elderly age | Prevalence | Both | United Republic of Tanzania | RHD | 0.24<br>(0.22 to 0.27) | < 0.001 |
| Middle-aged and elderly age | Prevalence | Female | United Republic of Tanzania | RHD | 0.27<br>(0.24 to 0.3) | < 0.001 |
| Middle-aged and elderly age | Prevalence | Male | United Republic of Tanzania | RHD | 0.21<br>(0.15 to 0.27) | < 0.001 |
| Middle-aged and elderly age | Prevalence | Both | United Republic of Tanzania | RA | 0.65<br>(0.58 to 0.72) | < 0.001 |
| Middle-aged and elderly age | Prevalence | Female | United Republic of Tanzania | RA | 0.62<br>(0.57 to 0.67) | < 0.001 |
| Middle-aged and elderly age | Prevalence | Male | United Republic of Tanzania | RA | 0.63<br>(0.57 to 0.69) | < 0.001 |
| Middle-aged and elderly age | Prevalence | Both | United States of America | AA | -0.18<br>(-0.2 to -0.16) | < 0.001 |
| Middle-aged and elderly age | Prevalence | Female | United States of America | AA | -0.18<br>(-0.2 to -0.15) | < 0.001 |
| Middle-aged and elderly age | Prevalence | Male | United States of America | AA | 0.03<br>(0 to 0.06) | 0.031 |
| Middle-aged and elderly age | Prevalence | Both | United States of America | T1D | 0.46<br>(0.25 to 0.66) | < 0.001 |
| Middle-aged and elderly age | Prevalence | Female | United States of America | T1D | 0.34<br>(0.25 to 0.43) | < 0.001 |
| Middle-aged and elderly age | Prevalence | Male | United States of America | T1D | 0.41 | 0.063 |

| | | | | | | |
|---|---|---|---|---|---|---|
| | | | | | (-0.02 to 0.85) | |
| Middle-aged and elderly age | Prevalence | Both | United States of America | IBD | -2.74 (-2.85 to -2.63) | < 0.001 |
| Middle-aged and elderly age | Prevalence | Female | United States of America | IBD | -2.57 (-2.7 to -2.45) | < 0.001 |
| Middle-aged and elderly age | Prevalence | Male | United States of America | IBD | -2.93 (-3.04 to -2.81) | < 0.001 |
| Middle-aged and elderly age | Prevalence | Both | United States of America | MS | 0.11 (0.07 to 0.16) | < 0.001 |
| Middle-aged and elderly age | Prevalence | Female | United States of America | MS | 0.17 (0.12 to 0.21) | < 0.001 |
| Middle-aged and elderly age | Prevalence | Male | United States of America | MS | 0.06 (0.01 to 0.1) | 0.011 |
| Middle-aged and elderly age | Prevalence | Both | United States of America | Psoriasis | -0.69 (-0.74 to -0.64) | < 0.001 |
| Middle-aged and elderly age | Prevalence | Female | United States of America | Psoriasis | -0.67 (-0.73 to -0.61) | < 0.001 |
| Middle-aged and elderly age | Prevalence | Male | United States of America | Psoriasis | -0.71 (-0.73 to -0.69) | < 0.001 |
| Middle-aged and elderly age | Prevalence | Both | United States of America | RHD | -1.06 (-1.2 to -0.92) | < 0.001 |
| Middle-aged and elderly age | Prevalence | Female | United States of America | RHD | -1.29 (-1.39 to -1.2) | < 0.001 |
| Middle-aged and elderly age | Prevalence | Male | United States of America | RHD | -0.9 (-1.01 to -0.79) | < 0.001 |
| Middle-aged and elderly age | Prevalence | Both | United States of America | RA | 0.58 (0.55 to 0.62) | < 0.001 |
| Middle-aged and elderly age | Prevalence | Female | United States of America | RA | 0.64 (0.6 to 0.67) | < 0.001 |
| Middle-aged and elderly age | Prevalence | Male | United States of America | RA | 0.52 (0.48 to 0.56) | < 0.001 |
| Middle-aged and elderly age | Prevalence | Both | United States Virgin Islands | AA | 0.03 (0.02 to 0.03) | < 0.001 |

| | | | | | | |
|---|---|---|---|---|---|---|
| Middle-aged and elderly age | Prevalence | Female | United States Virgin Islands | AA | 0<br>(0 to 0) | < 0.001 |
| Middle-aged and elderly age | Prevalence | Male | United States Virgin Islands | AA | 0<br>(0 to 0) | < 0.001 |
| Middle-aged and elderly age | Prevalence | Both | United States Virgin Islands | T1D | 1.06<br>(0.95 to 1.17) | < 0.001 |
| Middle-aged and elderly age | Prevalence | Female | United States Virgin Islands | T1D | 1.18<br>(0.96 to 1.41) | < 0.001 |
| Middle-aged and elderly age | Prevalence | Male | United States Virgin Islands | T1D | 0.86<br>(0.69 to 1.03) | < 0.001 |
| Middle-aged and elderly age | Prevalence | Both | United States Virgin Islands | IBD | 0.28<br>(0.25 to 0.31) | < 0.001 |
| Middle-aged and elderly age | Prevalence | Female | United States Virgin Islands | IBD | 0.32<br>(0.29 to 0.36) | < 0.001 |
| Middle-aged and elderly age | Prevalence | Male | United States Virgin Islands | IBD | 0.24<br>(0.2 to 0.28) | < 0.001 |
| Middle-aged and elderly age | Prevalence | Both | United States Virgin Islands | MS | 0.64<br>(0.59 to 0.69) | < 0.001 |
| Middle-aged and elderly age | Prevalence | Female | United States Virgin Islands | MS | 0.64<br>(0.58 to 0.7) | < 0.001 |
| Middle-aged and elderly age | Prevalence | Male | United States Virgin Islands | MS | 0.45<br>(0.44 to 0.46) | < 0.001 |
| Middle-aged and elderly age | Prevalence | Both | United States Virgin Islands | Psoriasis | -0.52<br>(-0.53 to -0.5) | < 0.001 |
| Middle-aged and elderly age | Prevalence | Female | United States Virgin Islands | Psoriasis | -0.52<br>(-0.54 to -0.5) | < 0.001 |
| Middle-aged and elderly age | Prevalence | Male | United States Virgin Islands | Psoriasis | -0.52<br>(-0.54 to -0.5) | < 0.001 |
| Middle-aged and elderly age | Prevalence | Both | United States Virgin Islands | RHD | -0.31<br>(-0.4 to -0.22) | < 0.001 |
| Middle-aged and elderly age | Prevalence | Female | United States Virgin Islands | RHD | -0.71<br>(-0.86 to -0.57) | < 0.001 |
| Middle-aged and elderly age | Prevalence | Male | United States Virgin Islands | RHD | 0.31 | < 0.001 |

| | | | | | | |
|---|---|---|---|---|---|---|
| | | | | | (0.21 to 0.4) | |
| Middle-aged and elderly age | Prevalence | Both | United States Virgin Islands | RA | 0.92<br>(0.87 to 0.97) | < 0.001 |
| Middle-aged and elderly age | Prevalence | Female | United States Virgin Islands | RA | 0.86<br>(0.81 to 0.9) | < 0.001 |
| Middle-aged and elderly age | Prevalence | Male | United States Virgin Islands | RA | 0.94<br>(0.86 to 1.02) | < 0.001 |
| Middle-aged and elderly age | Prevalence | Both | Uruguay | AA | 0.01<br>(0.01 to 0.02) | < 0.001 |
| Middle-aged and elderly age | Prevalence | Female | Uruguay | AA | 0<br>(0 to 0) | 0.000 |
| Middle-aged and elderly age | Prevalence | Male | Uruguay | AA | 0<br>(0 to 0) | 0.002 |
| Middle-aged and elderly age | Prevalence | Both | Uruguay | T1D | 0.45<br>(0.42 to 0.48) | < 0.001 |
| Middle-aged and elderly age | Prevalence | Female | Uruguay | T1D | 0.34<br>(0.3 to 0.38) | < 0.001 |
| Middle-aged and elderly age | Prevalence | Male | Uruguay | T1D | 0.59<br>(0.53 to 0.64) | < 0.001 |
| Middle-aged and elderly age | Prevalence | Both | Uruguay | IBD | -0.07<br>(-0.1 to -0.04) | < 0.001 |
| Middle-aged and elderly age | Prevalence | Female | Uruguay | IBD | -0.04<br>(-0.12 to 0.04) | 0.345 |
| Middle-aged and elderly age | Prevalence | Male | Uruguay | IBD | -0.11<br>(-0.13 to -0.08) | < 0.001 |
| Middle-aged and elderly age | Prevalence | Both | Uruguay | MS | 0.16<br>(0.12 to 0.2) | < 0.001 |
| Middle-aged and elderly age | Prevalence | Female | Uruguay | MS | 0.12<br>(0.11 to 0.13) | < 0.001 |
| Middle-aged and elderly age | Prevalence | Male | Uruguay | MS | 0.19<br>(0.09 to 0.29) | < 0.001 |
| Middle-aged and elderly age | Prevalence | Both | Uruguay | Psoriasis | -0.43<br>(-0.45 to -0.42) | < 0.001 |

| | | | | | | |
|---|---|---|---|---|---|---|
| Middle-aged and elderly age | Prevalence | Female | Uruguay | Psoriasis | -0.45<br>(-0.46 to -0.44) | < 0.001 |
| Middle-aged and elderly age | Prevalence | Male | Uruguay | Psoriasis | -0.42<br>(-0.44 to -0.39) | < 0.001 |
| Middle-aged and elderly age | Prevalence | Both | Uruguay | RHD | 0.05<br>(-0.1 to 0.21) | 0.490 |
| Middle-aged and elderly age | Prevalence | Female | Uruguay | RHD | -0.04<br>(-0.23 to 0.16) | 0.703 |
| Middle-aged and elderly age | Prevalence | Male | Uruguay | RHD | 0.19<br>(0.08 to 0.3) | 0.001 |
| Middle-aged and elderly age | Prevalence | Both | Uruguay | RA | 0.94<br>(0.9 to 0.99) | < 0.001 |
| Middle-aged and elderly age | Prevalence | Female | Uruguay | RA | 0.93<br>(0.9 to 0.96) | < 0.001 |
| Middle-aged and elderly age | Prevalence | Male | Uruguay | RA | 0.93<br>(0.88 to 0.99) | < 0.001 |
| Middle-aged and elderly age | Prevalence | Both | Uzbekistan | AA | 0.03<br>(0.02 to 0.04) | < 0.001 |
| Middle-aged and elderly age | Prevalence | Female | Uzbekistan | AA | 0<br>(0 to 0) | < 0.001 |
| Middle-aged and elderly age | Prevalence | Male | Uzbekistan | AA | 0<br>(0 to 0) | < 0.001 |
| Middle-aged and elderly age | Prevalence | Both | Uzbekistan | T1D | 1.11<br>(1.09 to 1.12) | < 0.001 |
| Middle-aged and elderly age | Prevalence | Female | Uzbekistan | T1D | 1.1<br>(1.08 to 1.11) | < 0.001 |
| Middle-aged and elderly age | Prevalence | Male | Uzbekistan | T1D | 1.13<br>(1.11 to 1.14) | < 0.001 |
| Middle-aged and elderly age | Prevalence | Both | Uzbekistan | IBD | 0.13<br>(0.09 to 0.17) | < 0.001 |
| Middle-aged and elderly age | Prevalence | Female | Uzbekistan | IBD | 0.15<br>(0.12 to 0.18) | < 0.001 |
| Middle-aged and elderly age | Prevalence | Male | Uzbekistan | IBD | 0.12 | 0.001 |

| | | | | | | |
|---|---|---|---|---|---|---|
| | | | | | (0.05 to 0.19) | |
| Middle-aged and elderly age | Prevalence | Both | Uzbekistan | MS | -0.91 (-0.96 to -0.85) | < 0.001 |
| Middle-aged and elderly age | Prevalence | Female | Uzbekistan | MS | -0.79 (-0.82 to -0.75) | < 0.001 |
| Middle-aged and elderly age | Prevalence | Male | Uzbekistan | MS | -1 (-1.1 to -0.89) | < 0.001 |
| Middle-aged and elderly age | Prevalence | Both | Uzbekistan | Psoriasis | -0.93 (-0.95 to -0.9) | < 0.001 |
| Middle-aged and elderly age | Prevalence | Female | Uzbekistan | Psoriasis | -0.95 (-0.99 to -0.92) | < 0.001 |
| Middle-aged and elderly age | Prevalence | Male | Uzbekistan | Psoriasis | -0.93 (-0.95 to -0.92) | < 0.001 |
| Middle-aged and elderly age | Prevalence | Both | Uzbekistan | RHD | 0.3 (0.28 to 0.32) | < 0.001 |
| Middle-aged and elderly age | Prevalence | Female | Uzbekistan | RHD | 0.3 (0.28 to 0.31) | < 0.001 |
| Middle-aged and elderly age | Prevalence | Male | Uzbekistan | RHD | 0.28 (0.26 to 0.3) | < 0.001 |
| Middle-aged and elderly age | Prevalence | Both | Uzbekistan | RA | 0.65 (0.62 to 0.68) | < 0.001 |
| Middle-aged and elderly age | Prevalence | Female | Uzbekistan | RA | 0.64 (0.58 to 0.69) | < 0.001 |
| Middle-aged and elderly age | Prevalence | Male | Uzbekistan | RA | 0.58 (0.53 to 0.63) | < 0.001 |
| Middle-aged and elderly age | Prevalence | Both | Vanuatu | AA | 0.05 (0.05 to 0.06) | < 0.001 |
| Middle-aged and elderly age | Prevalence | Female | Vanuatu | AA | 0 (0 to 0) | < 0.001 |
| Middle-aged and elderly age | Prevalence | Male | Vanuatu | AA | 0 (0 to 0) | < 0.001 |
| Middle-aged and elderly age | Prevalence | Both | Vanuatu | T1D | 0.75 (0.74 to 0.76) | < 0.001 |

| | | | | | | |
|---|---|---|---|---|---|---|
| Middle-aged and elderly age | Prevalence | Female | Vanuatu | T1D | 0.73 (0.73 to 0.74) | < 0.001 |
| Middle-aged and elderly age | Prevalence | Male | Vanuatu | T1D | 0.78 (0.76 to 0.8) | < 0.001 |
| Middle-aged and elderly age | Prevalence | Both | Vanuatu | IBD | -0.6 (-0.68 to -0.52) | < 0.001 |
| Middle-aged and elderly age | Prevalence | Female | Vanuatu | IBD | -0.99 (-1.09 to -0.89) | < 0.001 |
| Middle-aged and elderly age | Prevalence | Male | Vanuatu | IBD | -0.27 (-0.35 to -0.18) | < 0.001 |
| Middle-aged and elderly age | Prevalence | Both | Vanuatu | MS | 0.15 (0.12 to 0.18) | < 0.001 |
| Middle-aged and elderly age | Prevalence | Female | Vanuatu | MS | 0.14 (0.12 to 0.15) | < 0.001 |
| Middle-aged and elderly age | Prevalence | Male | Vanuatu | MS | 0.07 (0 to 0.15) | 0.061 |
| Middle-aged and elderly age | Prevalence | Both | Vanuatu | Psoriasis | -0.48 (-0.5 to -0.46) | < 0.001 |
| Middle-aged and elderly age | Prevalence | Female | Vanuatu | Psoriasis | -0.42 (-0.44 to -0.41) | < 0.001 |
| Middle-aged and elderly age | Prevalence | Male | Vanuatu | Psoriasis | -0.46 (-0.5 to -0.42) | < 0.001 |
| Middle-aged and elderly age | Prevalence | Both | Vanuatu | RHD | 0.07 (0.06 to 0.09) | < 0.001 |
| Middle-aged and elderly age | Prevalence | Female | Vanuatu | RHD | 0.09 (0.06 to 0.12) | < 0.001 |
| Middle-aged and elderly age | Prevalence | Male | Vanuatu | RHD | 0.03 (0.01 to 0.04) | < 0.001 |
| Middle-aged and elderly age | Prevalence | Both | Vanuatu | RA | 0.29 (0.2 to 0.38) | < 0.001 |
| Middle-aged and elderly age | Prevalence | Female | Vanuatu | RA | 0.21 (0.12 to 0.3) | < 0.001 |
| Middle-aged and elderly age | Prevalence | Male | Vanuatu | RA | 0.26 | < 0.001 |

| | | | | | | |
|---|---|---|---|---|---|---|
| | | | | | (0.19 to 0.33) | |
| Middle-aged and elderly age | Prevalence | Both | Venezuela (Bolivarian Republic of) | AA | 0.01<br>(0 to 0.01) | 0.001 |
| Middle-aged and elderly age | Prevalence | Female | Venezuela (Bolivarian Republic of) | AA | 0<br>(0 to 0) | < 0.001 |
| Middle-aged and elderly age | Prevalence | Male | Venezuela (Bolivarian Republic of) | AA | 0<br>(0 to 0) | < 0.001 |
| Middle-aged and elderly age | Prevalence | Both | Venezuela (Bolivarian Republic of) | T1D | 0.7<br>(0.68 to 0.72) | < 0.001 |
| Middle-aged and elderly age | Prevalence | Female | Venezuela (Bolivarian Republic of) | T1D | 0.71<br>(0.7 to 0.73) | < 0.001 |
| Middle-aged and elderly age | Prevalence | Male | Venezuela (Bolivarian Republic of) | T1D | 0.68<br>(0.67 to 0.7) | < 0.001 |
| Middle-aged and elderly age | Prevalence | Both | Venezuela (Bolivarian Republic of) | IBD | 0.05<br>(0.01 to 0.08) | 0.009 |
| Middle-aged and elderly age | Prevalence | Female | Venezuela (Bolivarian Republic of) | IBD | 0.01<br>(-0.05 to 0.07) | 0.792 |
| Middle-aged and elderly age | Prevalence | Male | Venezuela (Bolivarian Republic of) | IBD | 0.09<br>(0.05 to 0.13) | < 0.001 |
| Middle-aged and elderly age | Prevalence | Both | Venezuela (Bolivarian Republic of) | MS | 0.78<br>(0.71 to 0.85) | < 0.001 |
| Middle-aged and elderly age | Prevalence | Female | Venezuela (Bolivarian Republic of) | MS | 0.87<br>(0.81 to 0.94) | < 0.001 |
| Middle-aged and elderly age | Prevalence | Male | Venezuela (Bolivarian Republic of) | MS | 0.53<br>(0.44 to 0.62) | < 0.001 |
| Middle-aged and elderly age | Prevalence | Both | Venezuela (Bolivarian Republic of) | Psoriasis | -0.29<br>(-0.36 to -0.22) | < 0.001 |
| Middle-aged and elderly age | Prevalence | Female | Venezuela (Bolivarian Republic of) | Psoriasis | -0.3<br>(-0.38 to -0.23) | < 0.001 |
| Middle-aged and elderly age | Prevalence | Male | Venezuela (Bolivarian Republic of) | Psoriasis | -0.28<br>(-0.34 to -0.21) | < 0.001 |
| Middle-aged and elderly age | Prevalence | Both | Venezuela (Bolivarian Republic of) | RHD | -1.17<br>(-1.38 to -0.95) | < 0.001 |

| | | | | | | |
|---|---|---|---|---|---|---|
| Middle-aged and elderly age | Prevalence | Female | Venezuela (Bolivarian Republic of) | RHD | -1.33 (-1.58 to -1.08) | < 0.001 |
| Middle-aged and elderly age | Prevalence | Male | Venezuela (Bolivarian Republic of) | RHD | -0.9 (-1.06 to -0.74) | < 0.001 |
| Middle-aged and elderly age | Prevalence | Both | Venezuela (Bolivarian Republic of) | RA | 0.57 (0.49 to 0.64) | < 0.001 |
| Middle-aged and elderly age | Prevalence | Female | Venezuela (Bolivarian Republic of) | RA | 0.52 (0.44 to 0.61) | < 0.001 |
| Middle-aged and elderly age | Prevalence | Male | Venezuela (Bolivarian Republic of) | RA | 0.64 (0.6 to 0.69) | < 0.001 |
| Middle-aged and elderly age | Prevalence | Both | Viet Nam | AA | -0.04 (-0.05 to -0.04) | < 0.001 |
| Middle-aged and elderly age | Prevalence | Female | Viet Nam | AA | 0 (0 to 0) | < 0.001 |
| Middle-aged and elderly age | Prevalence | Male | Viet Nam | AA | 0 (0 to 0) | < 0.001 |
| Middle-aged and elderly age | Prevalence | Both | Viet Nam | T1D | 0.83 (0.81 to 0.85) | < 0.001 |
| Middle-aged and elderly age | Prevalence | Female | Viet Nam | T1D | 0.77 (0.74 to 0.79) | < 0.001 |
| Middle-aged and elderly age | Prevalence | Male | Viet Nam | T1D | 0.89 (0.85 to 0.94) | < 0.001 |
| Middle-aged and elderly age | Prevalence | Both | Viet Nam | IBD | 2.62 (2.31 to 2.92) | < 0.001 |
| Middle-aged and elderly age | Prevalence | Female | Viet Nam | IBD | 2.77 (2.52 to 3.03) | < 0.001 |
| Middle-aged and elderly age | Prevalence | Male | Viet Nam | IBD | 2.45 (2.06 to 2.84) | < 0.001 |
| Middle-aged and elderly age | Prevalence | Both | Viet Nam | MS | 0.29 (0.25 to 0.32) | < 0.001 |
| Middle-aged and elderly age | Prevalence | Female | Viet Nam | MS | 0.29 (0.24 to 0.33) | < 0.001 |
| Middle-aged and elderly age | Prevalence | Male | Viet Nam | MS | 0.44 | < 0.001 |

| | | | | | | |
|---|---|---|---|---|---|---|
| | | | | | (0.38 to 0.49) | |
| Middle-aged and elderly age | Prevalence | Both | Viet Nam | Psoriasis | -0.71<br>(-0.73 to -0.69) | < 0.001 |
| Middle-aged and elderly age | Prevalence | Female | Viet Nam | Psoriasis | -0.72<br>(-0.73 to -0.71) | < 0.001 |
| Middle-aged and elderly age | Prevalence | Male | Viet Nam | Psoriasis | -0.74<br>(-0.79 to -0.69) | < 0.001 |
| Middle-aged and elderly age | Prevalence | Both | Viet Nam | RHD | 0.23<br>(0.06 to 0.39) | 0.006 |
| Middle-aged and elderly age | Prevalence | Female | Viet Nam | RHD | -0.31<br>(-0.51 to -0.11) | 0.002 |
| Middle-aged and elderly age | Prevalence | Male | Viet Nam | RHD | 1.02<br>(0.88 to 1.15) | < 0.001 |
| Middle-aged and elderly age | Prevalence | Both | Viet Nam | RA | 0.81<br>(0.8 to 0.82) | < 0.001 |
| Middle-aged and elderly age | Prevalence | Female | Viet Nam | RA | 0.97<br>(0.94 to 1) | < 0.001 |
| Middle-aged and elderly age | Prevalence | Male | Viet Nam | RA | 0.69<br>(0.64 to 0.74) | < 0.001 |
| Middle-aged and elderly age | Prevalence | Both | Yemen | AA | 0.07<br>(0.06 to 0.09) | < 0.001 |
| Middle-aged and elderly age | Prevalence | Female | Yemen | AA | 0<br>(0 to 0) | < 0.001 |
| Middle-aged and elderly age | Prevalence | Male | Yemen | AA | 0<br>(0 to 0) | 0.015 |
| Middle-aged and elderly age | Prevalence | Both | Yemen | T1D | 1.89<br>(1.86 to 1.92) | < 0.001 |
| Middle-aged and elderly age | Prevalence | Female | Yemen | T1D | 1.91<br>(1.85 to 1.97) | < 0.001 |
| Middle-aged and elderly age | Prevalence | Male | Yemen | T1D | 1.91<br>(1.88 to 1.94) | < 0.001 |
| Middle-aged and elderly age | Prevalence | Both | Yemen | IBD | 0.62<br>(0.53 to 0.72) | < 0.001 |

| | | | | | | |
|---|---|---|---|---|---|---|
| Middle-aged and elderly age | Prevalence | Female | Yemen | IBD | 0.59 (0.52 to 0.65) | < 0.001 |
| Middle-aged and elderly age | Prevalence | Male | Yemen | IBD | 0.65 (0.58 to 0.73) | < 0.001 |
| Middle-aged and elderly age | Prevalence | Both | Yemen | MS | 0.84 (0.8 to 0.89) | < 0.001 |
| Middle-aged and elderly age | Prevalence | Female | Yemen | MS | 0.68 (0.63 to 0.73) | < 0.001 |
| Middle-aged and elderly age | Prevalence | Male | Yemen | MS | 0.94 (0.92 to 0.96) | < 0.001 |
| Middle-aged and elderly age | Prevalence | Both | Yemen | Psoriasis | -1.07 (-1.09 to -1.05) | < 0.001 |
| Middle-aged and elderly age | Prevalence | Female | Yemen | Psoriasis | -1.09 (-1.11 to -1.07) | < 0.001 |
| Middle-aged and elderly age | Prevalence | Male | Yemen | Psoriasis | -1.05 (-1.08 to -1.03) | < 0.001 |
| Middle-aged and elderly age | Prevalence | Both | Yemen | RHD | 0.14 (0.04 to 0.25) | 0.008 |
| Middle-aged and elderly age | Prevalence | Female | Yemen | RHD | 0.18 (0.11 to 0.24) | < 0.001 |
| Middle-aged and elderly age | Prevalence | Male | Yemen | RHD | 0.09 (0.02 to 0.17) | 0.019 |
| Middle-aged and elderly age | Prevalence | Both | Yemen | RA | 0.77 (0.71 to 0.84) | < 0.001 |
| Middle-aged and elderly age | Prevalence | Female | Yemen | RA | 0.67 (0.63 to 0.71) | < 0.001 |
| Middle-aged and elderly age | Prevalence | Male | Yemen | RA | 0.58 (0.53 to 0.62) | < 0.001 |
| Middle-aged and elderly age | Prevalence | Both | Zambia | AA | -0.03 (-0.03 to -0.02) | < 0.001 |
| Middle-aged and elderly age | Prevalence | Female | Zambia | AA | 0 (0 to 0) | 1.000 |
| Middle-aged and elderly age | Prevalence | Male | Zambia | AA | 0 | < 0.001 |

| | | | | | | |
|---|---|---|---|---|---|---|
| | | | | | (0 to 0) | |
| Middle-aged and elderly age | Prevalence | Both | Zambia | T1D | 0.54 (0.53 to 0.54) | < 0.001 |
| Middle-aged and elderly age | Prevalence | Female | Zambia | T1D | 0.54 (0.53 to 0.54) | < 0.001 |
| Middle-aged and elderly age | Prevalence | Male | Zambia | T1D | 0.53 (0.52 to 0.55) | < 0.001 |
| Middle-aged and elderly age | Prevalence | Both | Zambia | IBD | 0.86 (0.61 to 1.12) | < 0.001 |
| Middle-aged and elderly age | Prevalence | Female | Zambia | IBD | 0.67 (0.53 to 0.8) | < 0.001 |
| Middle-aged and elderly age | Prevalence | Male | Zambia | IBD | 1.14 (1.01 to 1.27) | < 0.001 |
| Middle-aged and elderly age | Prevalence | Both | Zambia | MS | 0.2 (0.18 to 0.21) | < 0.001 |
| Middle-aged and elderly age | Prevalence | Female | Zambia | MS | 0.16 (0.13 to 0.18) | < 0.001 |
| Middle-aged and elderly age | Prevalence | Male | Zambia | MS | 0.34 (0.32 to 0.36) | < 0.001 |
| Middle-aged and elderly age | Prevalence | Both | Zambia | Psoriasis | -0.49 (-0.56 to -0.43) | < 0.001 |
| Middle-aged and elderly age | Prevalence | Female | Zambia | Psoriasis | -0.5 (-0.55 to -0.45) | < 0.001 |
| Middle-aged and elderly age | Prevalence | Male | Zambia | Psoriasis | -0.46 (-0.52 to -0.4) | < 0.001 |
| Middle-aged and elderly age | Prevalence | Both | Zambia | RHD | 0.12 (0.07 to 0.17) | < 0.001 |
| Middle-aged and elderly age | Prevalence | Female | Zambia | RHD | 0.13 (0.04 to 0.21) | 0.003 |
| Middle-aged and elderly age | Prevalence | Male | Zambia | RHD | 0.1 (0.09 to 0.11) | < 0.001 |
| Middle-aged and elderly age | Prevalence | Both | Zambia | RA | 0.89 (0.8 to 0.98) | < 0.001 |

| | | | | | | |
|---|---|---|---|---|---|---|
| Middle-aged and elderly age | Prevalence | Female | Zambia | RA | 0.75<br>(0.6 to 0.9) | < 0.001 |
| Middle-aged and elderly age | Prevalence | Male | Zambia | RA | 0.95<br>(0.87 to 1.04) | < 0.001 |
| Middle-aged and elderly age | Prevalence | Both | Zimbabwe | AA | 0.14<br>(0.12 to 0.16) | < 0.001 |
| Middle-aged and elderly age | Prevalence | Female | Zimbabwe | AA | 0<br>(0 to 0) | 0.027 |
| Middle-aged and elderly age | Prevalence | Male | Zimbabwe | AA | 0<br>(0 to 0) | < 0.001 |
| Middle-aged and elderly age | Prevalence | Both | Zimbabwe | T1D | 0.43<br>(0.41 to 0.45) | < 0.001 |
| Middle-aged and elderly age | Prevalence | Female | Zimbabwe | T1D | 0.44<br>(0.4 to 0.47) | < 0.001 |
| Middle-aged and elderly age | Prevalence | Male | Zimbabwe | T1D | 0.43<br>(0.4 to 0.45) | < 0.001 |
| Middle-aged and elderly age | Prevalence | Both | Zimbabwe | IBD | 0.05<br>(-0.06 to 0.17) | 0.364 |
| Middle-aged and elderly age | Prevalence | Female | Zimbabwe | IBD | -0.02<br>(-0.12 to 0.08) | 0.670 |
| Middle-aged and elderly age | Prevalence | Male | Zimbabwe | IBD | 0.16<br>(0.02 to 0.29) | 0.021 |
| Middle-aged and elderly age | Prevalence | Both | Zimbabwe | MS | 0.24<br>(0.18 to 0.31) | < 0.001 |
| Middle-aged and elderly age | Prevalence | Female | Zimbabwe | MS | 0.09<br>(0.02 to 0.15) | 0.009 |
| Middle-aged and elderly age | Prevalence | Male | Zimbabwe | MS | 0.09<br>(0.05 to 0.14) | < 0.001 |
| Middle-aged and elderly age | Prevalence | Both | Zimbabwe | Psoriasis | -0.17<br>(-0.21 to -0.13) | < 0.001 |
| Middle-aged and elderly age | Prevalence | Female | Zimbabwe | Psoriasis | -0.2<br>(-0.23 to -0.16) | < 0.001 |
| Middle-aged and elderly age | Prevalence | Male | Zimbabwe | Psoriasis | -0.17 | < 0.001 |

| | | | | | | |
|---|---|---|---|---|---|---|
| | | | | | (-0.22 to -0.13) | |
| Middle-aged and elderly age | Prevalence | Both | Zimbabwe | RHD | 0.07<br>(0.05 to 0.08) | < 0.001 |
| Middle-aged and elderly age | Prevalence | Female | Zimbabwe | RHD | 0.02<br>(0 to 0.04) | 0.035 |
| Middle-aged and elderly age | Prevalence | Male | Zimbabwe | RHD | 0.06<br>(0.03 to 0.08) | < 0.001 |
| Middle-aged and elderly age | Prevalence | Both | Zimbabwe | RA | 0.01<br>(-0.04 to 0.07) | 0.623 |
| Middle-aged and elderly age | Prevalence | Female | Zimbabwe | RA | -0.06<br>(-0.1 to -0.01) | 0.013 |
| Middle-aged and elderly age | Prevalence | Male | Zimbabwe | RA | 0.01<br>(-0.06 to 0.07) | 0.818 |

**Abbreviations: IBD, Inflammatory bowel disease; RHD, Rheumatic heart disease; RA, Rheumatoid arthritis; AA, Alopecia areata; MS, Multiple sclerosis; T1DM, Diabetes mellitus type 1.**

**Table S3 Age effects on ADs disorders prevalence of global in 0-69 years from 1990 to 2021.**

| Location | Age | Cause | Sex | RR |
|---|---|---|---|---|
| Global | 0 to 4 | T1D | Both | 0.16 (0.15, 0.17） |
| Global | 0 to 4 | T1D | Female | 0.16 (0.15, 0.17） |
| Global | 0 to 4 | T1D | Male | 0.16 (0.16, 0.17） |
| Global | 10 to 14 | T1D | Both | 2.12 (2.08, 2.16） |
| Global | 10 to 14 | T1D | Female | 2.11 (2.08, 2.15） |
| Global | 10 to 14 | T1D | Male | 2.13 (2.09, 2.18） |
| Global | 15 to 19 | T1D | Both | 3.04 (2.98, 3.10） |
| Global | 15 to 19 | T1D | Female | 2.97 (2.91, 3.02） |
| Global | 15 to 19 | T1D | Male | 3.11 (3.05, 3.17） |
| Global | 20 to 24 | T1D | Both | 3.83 (3.76, 3.91） |
| Global | 20 to 24 | T1D | Female | 3.67 (3.60, 3.74） |
| Global | 20 to 24 | T1D | Male | 3.99 (3.91, 4.08） |
| Global | 25 to 29 | T1D | Both | 4.71 (4.62, 4.80） |
| Global | 25 to 29 | T1D | Female | 4.45 (4.36, 4.53） |
| Global | 25 to 29 | T1D | Male | 4.98 (4.87, 5.08） |
| Global | 30 to 34 | T1D | Both | 5.48 (5.37, 5.59） |
| Global | 30 to 34 | T1D | Female | 5.12 (5.02, 5.22） |
| Global | 30 to 34 | T1D | Male | 5.84 (5.72, 5.97） |
| Global | 35 to 39 | T1D | Both | 6.23 (6.10, 6.36） |
| Global | 35 to 39 | T1D | Female | 5.78 (5.67, 5.90） |
| Global | 35 to 39 | T1D | Male | 6.69 (6.54, 6.84） |
| Global | 40 to 44 | T1D | Both | 7.02 (6.88, 7.17） |
| Global | 40 to 44 | T1D | Female | 6.48 (6.34, 6.61） |
| Global | 40 to 44 | T1D | Male | 7.58 (7.41, 7.75） |
| Global | 45 to 49 | T1D | Both | 7.84 (7.67, 8.01） |
| Global | 45 to 49 | T1D | Female | 7.20 (7.05, 7.36） |
| Global | 45 to 49 | T1D | Male | 8.48 (8.29, 8.68） |
| Global | 5 to 9 | T1D | Both | 1.00 (1.00, 1.00） |
| Global | 5 to 9 | T1D | Female | 1.00 (1.00, 1.00） |
| Global | 5 to 9 | T1D | Male | 1.00 (1.00, 1.00） |
| Global | 50 to 54 | T1D | Both | 8.68 (8.49, 8.87） |

| | | | | |
|---|---|---|---|---|
| Global | 50 to 54 | T1D | Female | 7.97 (7.79, 8.14） |
| Global | 50 to 54 | T1D | Male | 9.41 (9.19, 9.64） |
| Global | 55 to 59 | T1D | Both | 9.57 (9.35, 9.79） |
| Global | 55 to 59 | T1D | Female | 8.79 (8.59, 8.99） |
| Global | 55 to 59 | T1D | Male | 10.39 (10.14, 10.65） |
| Global | 60 to 64 | T1D | Both | 10.50 (10.25, 10.75） |
| Global | 60 to 64 | T1D | Female | 9.66 (9.43, 9.89） |
| Global | 60 to 64 | T1D | Male | 11.39 (11.11, 11.69） |
| Global | 65 to 69 | T1D | Both | 11.58 (11.30, 11.88） |
| Global | 65 to 69 | T1D | Female | 10.68 (10.42, 10.95） |
| Global | 65 to 69 | T1D | Male | 12.56 (12.23, 12.90） |
| African Region | 0 to 4 | T1D | Both | 0.15 (0.15, 0.15） |
| African Region | 0 to 4 | T1D | Female | 0.15 (0.15, 0.15） |
| African Region | 0 to 4 | T1D | Male | 0.15 (0.15, 0.15） |
| African Region | 10 to 14 | T1D | Both | 2.11 (2.10, 2.13） |
| African Region | 10 to 14 | T1D | Female | 2.12 (2.10, 2.13） |
| African Region | 10 to 14 | T1D | Male | 2.11 (2.09, 2.12） |
| African Region | 15 to 19 | T1D | Both | 3.11 (3.09, 3.13） |
| African Region | 15 to 19 | T1D | Female | 3.12 (3.09, 3.14） |
| African Region | 15 to 19 | T1D | Male | 3.10 (3.08, 3.12） |
| African Region | 20 to 24 | T1D | Both | 4.01 (3.98, 4.04） |
| African Region | 20 to 24 | T1D | Female | 4.03 (3.99, 4.06） |
| African Region | 20 to 24 | T1D | Male | 4.00 (3.98, 4.03） |
| African Region | 25 to 29 | T1D | Both | 4.97 (4.93, 5.00） |
| African Region | 25 to 29 | T1D | Female | 4.99 (4.95, 5.03） |
| African Region | 25 to 29 | T1D | Male | 4.95 (4.92, 4.99） |
| African Region | 30 to 34 | T1D | Both | 5.62 (5.58, 5.67） |
| African Region | 30 to 34 | T1D | Female | 5.65 (5.60, 5.70） |
| African Region | 30 to 34 | T1D | Male | 5.60 (5.56, 5.64） |
| African Region | 35 to 39 | T1D | Both | 6.13 (6.08, 6.19） |
| African Region | 35 to 39 | T1D | Female | 6.18 (6.12, 6.24） |
| African Region | 35 to 39 | T1D | Male | 6.10 (6.05, 6.15） |
| African Region | 40 to 44 | T1D | Both | 6.58 (6.53, 6.64） |

| | | | | |
|---|---|---|---|---|
| African Region | 40 to 44 | T1D | Female | 6.64 (6.58, 6.71） |
| African Region | 40 to 44 | T1D | Male | 6.53 (6.48, 6.59） |
| African Region | 45 to 49 | T1D | Both | 6.99 (6.93, 7.06） |
| African Region | 45 to 49 | T1D | Female | 7.07 (7.00, 7.14） |
| African Region | 45 to 49 | T1D | Male | 6.92 (6.86, 6.98） |
| African Region | 5 to 9 | T1D | Both | 1.00 (1.00, 1.00） |
| African Region | 5 to 9 | T1D | Female | 1.00 (1.00, 1.00） |
| African Region | 5 to 9 | T1D | Male | 1.00 (1.00, 1.00） |
| African Region | 50 to 54 | T1D | Both | 7.35 (7.28, 7.42） |
| African Region | 50 to 54 | T1D | Female | 7.46 (7.37, 7.54） |
| African Region | 50 to 54 | T1D | Male | 7.25 (7.19, 7.32） |
| African Region | 55 to 59 | T1D | Both | 7.66 (7.58, 7.75） |
| African Region | 55 to 59 | T1D | Female | 7.81 (7.72, 7.91） |
| African Region | 55 to 59 | T1D | Male | 7.53 (7.46, 7.61） |
| African Region | 60 to 64 | T1D | Both | 7.94 (7.85, 8.03） |
| African Region | 60 to 64 | T1D | Female | 8.14 (8.03, 8.24） |
| African Region | 60 to 64 | T1D | Male | 7.77 (7.69, 7.85） |
| African Region | 65 to 69 | T1D | Both | 8.18 (8.07, 8.28） |
| African Region | 65 to 69 | T1D | Female | 8.42 (8.30, 8.54） |
| African Region | 65 to 69 | T1D | Male | 7.96 (7.87, 8.06） |
| Eastern Mediterranean Region | 0 to 4 | T1D | Both | 0.14 (0.14, 0.14） |
| Eastern Mediterranean Region | 0 to 4 | T1D | Female | 0.14 (0.14, 0.15） |
| Eastern Mediterranean Region | 0 to 4 | T1D | Male | 0.14 (0.13, 0.14） |
| Eastern Mediterranean Region | 10 to 14 | T1D | Both | 2.31 (2.29, 2.32） |
| Eastern Mediterranean Region | 10 to 14 | T1D | Female | 2.35 (2.33, 2.37） |
| Eastern Mediterranean Region | 10 to 14 | T1D | Male | 2.27 (2.26, 2.29） |
| Eastern Mediterranean Region | 15 to 19 | T1D | Both | 3.50 (3.48, 3.52） |
| Eastern Mediterranean Region | 15 to 19 | T1D | Female | 3.54 (3.51, 3.57） |
| Eastern Mediterranean Region | 15 to 19 | T1D | Male | 3.47 (3.44, 3.49） |
| Eastern Mediterranean Region | 20 to 24 | T1D | Both | 4.58 (4.55, 4.60） |
| Eastern Mediterranean Region | 20 to 24 | T1D | Female | 4.54 (4.50, 4.57） |
| Eastern Mediterranean Region | 20 to 24 | T1D | Male | 4.61 (4.58, 4.65） |
| Eastern Mediterranean Region | 25 to 29 | T1D | Both | 5.81 (5.78, 5.85） |

| | | | | |
|---|---|---|---|---|
| Eastern Mediterranean Region | 25 to 29 | T1D | Female | 5.64 (5.60, 5.69） |
| Eastern Mediterranean Region | 25 to 29 | T1D | Male | 5.96 (5.92, 6.00） |
| Eastern Mediterranean Region | 30 to 34 | T1D | Both | 7.03 (6.98, 7.07） |
| Eastern Mediterranean Region | 30 to 34 | T1D | Female | 6.70 (6.65, 6.76） |
| Eastern Mediterranean Region | 30 to 34 | T1D | Male | 7.30 (7.25, 7.36） |
| Eastern Mediterranean Region | 35 to 39 | T1D | Both | 8.23 (8.17, 8.28） |
| Eastern Mediterranean Region | 35 to 39 | T1D | Female | 7.76 (7.69, 7.82） |
| Eastern Mediterranean Region | 35 to 39 | T1D | Male | 8.63 (8.56, 8.70） |
| Eastern Mediterranean Region | 40 to 44 | T1D | Both | 9.43 (9.36, 9.49） |
| Eastern Mediterranean Region | 40 to 44 | T1D | Female | 8.81 (8.73, 8.90） |
| Eastern Mediterranean Region | 40 to 44 | T1D | Male | 9.95 (9.87, 10.03） |
| Eastern Mediterranean Region | 45 to 49 | T1D | Both | 10.66 (10.58, 10.73） |
| Eastern Mediterranean Region | 45 to 49 | T1D | Female | 9.94 (9.85, 10.04） |
| Eastern Mediterranean Region | 45 to 49 | T1D | Male | 11.26 (11.17, 11.36） |
| Eastern Mediterranean Region | 5 to 9 | T1D | Both | 1.00 (1.00, 1.00） |
| Eastern Mediterranean Region | 5 to 9 | T1D | Female | 1.00 (1.00, 1.00） |
| Eastern Mediterranean Region | 5 to 9 | T1D | Male | 1.00 (1.00, 1.00） |
| Eastern Mediterranean Region | 50 to 54 | T1D | Both | 11.89 (11.80, 11.98） |
| Eastern Mediterranean Region | 50 to 54 | T1D | Female | 11.12 (11.01, 11.23） |
| Eastern Mediterranean Region | 50 to 54 | T1D | Male | 12.54 (12.42, 12.65） |
| Eastern Mediterranean Region | 55 to 59 | T1D | Both | 13.18 (13.07, 13.29） |
| Eastern Mediterranean Region | 55 to 59 | T1D | Female | 12.37 (12.24, 12.51） |
| Eastern Mediterranean Region | 55 to 59 | T1D | Male | 13.86 (13.72, 13.99） |
| Eastern Mediterranean Region | 60 to 64 | T1D | Both | 14.50 (14.38, 14.63） |
| Eastern Mediterranean Region | 60 to 64 | T1D | Female | 13.69 (13.53, 13.85） |
| Eastern Mediterranean Region | 60 to 64 | T1D | Male | 15.18 (15.02, 15.34） |
| Eastern Mediterranean Region | 65 to 69 | T1D | Both | 15.90 (15.75, 16.05） |
| Eastern Mediterranean Region | 65 to 69 | T1D | Female | 15.08 (14.89, 15.27） |
| Eastern Mediterranean Region | 65 to 69 | T1D | Male | 16.58 (16.39, 16.77） |
| European Region | 0 to 4 | T1D | Both | 0.18 (0.17, 0.19） |
| European Region | 0 to 4 | T1D | Female | 0.18 (0.17, 0.19） |
| European Region | 0 to 4 | T1D | Male | 0.18 (0.17, 0.19） |
| European Region | 10 to 14 | T1D | Both | 2.19 (2.14, 2.23） |

| | | | | |
|---|---|---|---|---|
| European Region | 10 to 14 | T1D | Female | 2.17 (2.13, 2.21） |
| European Region | 10 to 14 | T1D | Male | 2.20 (2.16, 2.25） |
| European Region | 15 to 19 | T1D | Both | 3.36 (3.30, 3.43） |
| European Region | 15 to 19 | T1D | Female | 3.27 (3.21, 3.34） |
| European Region | 15 to 19 | T1D | Male | 3.45 (3.38, 3.52） |
| European Region | 20 to 24 | T1D | Both | 4.49 (4.40, 4.58） |
| European Region | 20 to 24 | T1D | Female | 4.30 (4.22, 4.38） |
| European Region | 20 to 24 | T1D | Male | 4.69 (4.59, 4.79） |
| European Region | 25 to 29 | T1D | Both | 5.82 (5.70, 5.94） |
| European Region | 25 to 29 | T1D | Female | 5.49 (5.38, 5.60） |
| European Region | 25 to 29 | T1D | Male | 6.16 (6.03, 6.29） |
| European Region | 30 to 34 | T1D | Both | 7.13 (6.98, 7.27） |
| European Region | 30 to 34 | T1D | Female | 6.65 (6.52, 6.78） |
| European Region | 30 to 34 | T1D | Male | 7.62 (7.45, 7.79） |
| European Region | 35 to 39 | T1D | Both | 8.51 (8.33, 8.69） |
| European Region | 35 to 39 | T1D | Female | 7.87 (7.71, 8.02） |
| European Region | 35 to 39 | T1D | Male | 9.18 (8.98, 9.39） |
| European Region | 40 to 44 | T1D | Both | 10.01 (9.79, 10.22） |
| European Region | 40 to 44 | T1D | Female | 9.18 (8.99, 9.37） |
| European Region | 40 to 44 | T1D | Male | 10.87 (10.63, 11.12） |
| European Region | 45 to 49 | T1D | Both | 11.60 (11.35, 11.85） |
| European Region | 45 to 49 | T1D | Female | 10.58 (10.37, 10.80） |
| European Region | 45 to 49 | T1D | Male | 12.68 (12.39, 12.98） |
| European Region | 5 to 9 | T1D | Both | 1.00 (1.00, 1.00） |
| European Region | 5 to 9 | T1D | Female | 1.00 (1.00, 1.00） |
| European Region | 5 to 9 | T1D | Male | 1.00 (1.00, 1.00） |
| European Region | 50 to 54 | T1D | Both | 13.34 (13.05, 13.63） |
| European Region | 50 to 54 | T1D | Female | 12.14 (11.89, 12.40） |
| European Region | 50 to 54 | T1D | Male | 14.64 (14.30, 14.99） |
| European Region | 55 to 59 | T1D | Both | 15.28 (14.94, 15.62） |
| European Region | 55 to 59 | T1D | Female | 13.88 (13.58, 14.18） |
| European Region | 55 to 59 | T1D | Male | 16.83 (16.43, 17.24） |
| European Region | 60 to 64 | T1D | Both | 17.45 (17.06, 17.85） |

| | | | | |
|---|---|---|---|---|
| European Region | 60 to 64 | T1D | Female | 15.83 (15.49, 16.18） |
| European Region | 60 to 64 | T1D | Male | 19.30 (18.83, 19.78） |
| European Region | 65 to 69 | T1D | Both | 19.91 (19.45, 20.38） |
| European Region | 65 to 69 | T1D | Female | 18.04 (17.64, 18.44） |
| European Region | 65 to 69 | T1D | Male | 22.12 (21.57, 22.68） |
| Region of the Americas | 0 to 4 | T1D | Both | 0.21 (0.21, 0.22） |
| Region of the Americas | 0 to 4 | T1D | Female | 0.20 (0.19, 0.20） |
| Region of the Americas | 0 to 4 | T1D | Male | 0.23 (0.22, 0.23） |
| Region of the Americas | 10 to 14 | T1D | Both | 1.99 (1.96, 2.02） |
| Region of the Americas | 10 to 14 | T1D | Female | 2.04 (2.01, 2.07） |
| Region of the Americas | 10 to 14 | T1D | Male | 1.94 (1.91, 1.96） |
| Region of the Americas | 15 to 19 | T1D | Both | 2.76 (2.73, 2.80） |
| Region of the Americas | 15 to 19 | T1D | Female | 2.79 (2.75, 2.84） |
| Region of the Americas | 15 to 19 | T1D | Male | 2.73 (2.70, 2.77） |
| Region of the Americas | 20 to 24 | T1D | Both | 3.44 (3.39, 3.49） |
| Region of the Americas | 20 to 24 | T1D | Female | 3.40 (3.35, 3.46） |
| Region of the Americas | 20 to 24 | T1D | Male | 3.47 (3.43, 3.52） |
| Region of the Americas | 25 to 29 | T1D | Both | 4.26 (4.20, 4.32） |
| Region of the Americas | 25 to 29 | T1D | Female | 4.14 (4.07, 4.21） |
| Region of the Americas | 25 to 29 | T1D | Male | 4.38 (4.32, 4.44） |
| Region of the Americas | 30 to 34 | T1D | Both | 4.95 (4.88, 5.02） |
| Region of the Americas | 30 to 34 | T1D | Female | 4.77 (4.69, 4.85） |
| Region of the Americas | 30 to 34 | T1D | Male | 5.14 (5.06, 5.21） |
| Region of the Americas | 35 to 39 | T1D | Both | 5.58 (5.50, 5.67） |
| Region of the Americas | 35 to 39 | T1D | Female | 5.36 (5.26, 5.45） |
| Region of the Americas | 35 to 39 | T1D | Male | 5.82 (5.73, 5.91） |
| Region of the Americas | 40 to 44 | T1D | Both | 6.23 (6.14, 6.33） |
| Region of the Americas | 40 to 44 | T1D | Female | 5.97 (5.86, 6.08） |
| Region of the Americas | 40 to 44 | T1D | Male | 6.51 (6.42, 6.61） |
| Region of the Americas | 45 to 49 | T1D | Both | 6.89 (6.78, 7.00） |
| Region of the Americas | 45 to 49 | T1D | Female | 6.58 (6.46, 6.71） |
| Region of the Americas | 45 to 49 | T1D | Male | 7.21 (7.10, 7.32） |
| Region of the Americas | 5 to 9 | T1D | Both | 1.00 (1.00, 1.00） |

| | | | | |
|---|---|---|---|---|
| Region of the Americas | 5 to 9 | T1D | Female | 1.00 (1.00, 1.00） |
| Region of the Americas | 5 to 9 | T1D | Male | 1.00 (1.00, 1.00） |
| Region of the Americas | 50 to 54 | T1D | Both | 7.53 (7.41, 7.65） |
| Region of the Americas | 50 to 54 | T1D | Female | 7.19 (7.06, 7.33） |
| Region of the Americas | 50 to 54 | T1D | Male | 7.89 (7.77, 8.02） |
| Region of the Americas | 55 to 59 | T1D | Both | 8.17 (8.04, 8.31） |
| Region of the Americas | 55 to 59 | T1D | Female | 7.81 (7.66, 7.96） |
| Region of the Americas | 55 to 59 | T1D | Male | 8.57 (8.43, 8.71） |
| Region of the Americas | 60 to 64 | T1D | Both | 8.83 (8.68, 8.98） |
| Region of the Americas | 60 to 64 | T1D | Female | 8.44 (8.28, 8.62） |
| Region of the Americas | 60 to 64 | T1D | Male | 9.25 (9.10, 9.41） |
| Region of the Americas | 65 to 69 | T1D | Both | 9.51 (9.35, 9.68） |
| Region of the Americas | 65 to 69 | T1D | Female | 9.13 (8.94, 9.32） |
| Region of the Americas | 65 to 69 | T1D | Male | 9.95 (9.77, 10.12） |
| South-East Asia Region | 0 to 4 | T1D | Both | 0.14 (0.13, 0.15） |
| South-East Asia Region | 0 to 4 | T1D | Female | 0.14 (0.14, 0.15） |
| South-East Asia Region | 0 to 4 | T1D | Male | 0.14 (0.13, 0.16） |
| South-East Asia Region | 10 to 14 | T1D | Both | 2.17 (2.10, 2.23） |
| South-East Asia Region | 10 to 14 | T1D | Female | 2.10 (2.06, 2.14） |
| South-East Asia Region | 10 to 14 | T1D | Male | 2.23 (2.14, 2.31） |
| South-East Asia Region | 15 to 19 | T1D | Both | 3.00 (2.92, 3.09） |
| South-East Asia Region | 15 to 19 | T1D | Female | 2.83 (2.77, 2.88） |
| South-East Asia Region | 15 to 19 | T1D | Male | 3.17 (3.06, 3.30） |
| South-East Asia Region | 20 to 24 | T1D | Both | 3.62 (3.52, 3.73） |
| South-East Asia Region | 20 to 24 | T1D | Female | 3.34 (3.28, 3.41） |
| South-East Asia Region | 20 to 24 | T1D | Male | 3.90 (3.75, 4.05） |
| South-East Asia Region | 25 to 29 | T1D | Both | 4.29 (4.16, 4.42） |
| South-East Asia Region | 25 to 29 | T1D | Female | 3.90 (3.82, 3.98） |
| South-East Asia Region | 25 to 29 | T1D | Male | 4.69 (4.50, 4.87） |
| South-East Asia Region | 30 to 34 | T1D | Both | 4.84 (4.69, 4.99） |
| South-East Asia Region | 30 to 34 | T1D | Female | 4.36 (4.27, 4.45） |
| South-East Asia Region | 30 to 34 | T1D | Male | 5.33 (5.12, 5.55） |
| South-East Asia Region | 35 to 39 | T1D | Both | 5.34 (5.17, 5.51） |

| | | | | |
|---|---|---|---|---|
| South-East Asia Region | 35 to 39 | T1D | Female | 4.78 (4.68, 4.88） |
| South-East Asia Region | 35 to 39 | T1D | Male | 5.92 (5.67, 6.17） |
| South-East Asia Region | 40 to 44 | T1D | Both | 5.83 (5.64, 6.03） |
| South-East Asia Region | 40 to 44 | T1D | Female | 5.19 (5.07, 5.30） |
| South-East Asia Region | 40 to 44 | T1D | Male | 6.49 (6.21, 6.78） |
| South-East Asia Region | 45 to 49 | T1D | Both | 6.30 (6.09, 6.53） |
| South-East Asia Region | 45 to 49 | T1D | Female | 5.60 (5.47, 5.73） |
| South-East Asia Region | 45 to 49 | T1D | Male | 7.03 (6.72, 7.35） |
| South-East Asia Region | 5 to 9 | T1D | Both | 1.00 (1.00, 1.00） |
| South-East Asia Region | 5 to 9 | T1D | Female | 1.00 (1.00, 1.00） |
| South-East Asia Region | 5 to 9 | T1D | Male | 1.00 (1.00, 1.00） |
| South-East Asia Region | 50 to 54 | T1D | Both | 6.74 (6.50, 6.99） |
| South-East Asia Region | 50 to 54 | T1D | Female | 5.99 (5.85, 6.14） |
| South-East Asia Region | 50 to 54 | T1D | Male | 7.51 (7.17, 7.88） |
| South-East Asia Region | 55 to 59 | T1D | Both | 7.24 (6.97, 7.52） |
| South-East Asia Region | 55 to 59 | T1D | Female | 6.47 (6.31, 6.64） |
| South-East Asia Region | 55 to 59 | T1D | Male | 8.05 (7.66, 8.46） |
| South-East Asia Region | 60 to 64 | T1D | Both | 7.79 (7.48, 8.11） |
| South-East Asia Region | 60 to 64 | T1D | Female | 7.02 (6.83, 7.21） |
| South-East Asia Region | 60 to 64 | T1D | Male | 8.63 (8.19, 9.10） |
| South-East Asia Region | 65 to 69 | T1D | Both | 8.29 (7.94, 8.65） |
| South-East Asia Region | 65 to 69 | T1D | Female | 7.51 (7.29, 7.73） |
| South-East Asia Region | 65 to 69 | T1D | Male | 9.16 (8.66, 9.70） |
| Western Pacific Region | 0 to 4 | T1D | Both | 0.15 (0.13, 0.16） |
| Western Pacific Region | 0 to 4 | T1D | Female | 0.14 (0.13, 0.16） |
| Western Pacific Region | 0 to 4 | T1D | Male | 0.15 (0.13, 0.16） |
| Western Pacific Region | 10 to 14 | T1D | Both | 2.02 (1.95, 2.10） |
| Western Pacific Region | 10 to 14 | T1D | Female | 1.99 (1.92, 2.07） |
| Western Pacific Region | 10 to 14 | T1D | Male | 2.05 (1.97, 2.14） |
| Western Pacific Region | 15 to 19 | T1D | Both | 2.72 (2.62, 2.82） |
| Western Pacific Region | 15 to 19 | T1D | Female | 2.61 (2.52, 2.70） |
| Western Pacific Region | 15 to 19 | T1D | Male | 2.82 (2.70, 2.94） |
| Western Pacific Region | 20 to 24 | T1D | Both | 3.29 (3.16, 3.42） |

| | | | | |
|---|---|---|---|---|
| Western Pacific Region | 20 to 24 | T1D | Female | 3.07 (2.96, 3.18） |
| Western Pacific Region | 20 to 24 | T1D | Male | 3.50 (3.35, 3.65） |
| Western Pacific Region | 25 to 29 | T1D | Both | 3.86 (3.72, 4.01） |
| Western Pacific Region | 25 to 29 | T1D | Female | 3.52 (3.40, 3.66） |
| Western Pacific Region | 25 to 29 | T1D | Male | 4.19 (4.02, 4.38） |
| Western Pacific Region | 30 to 34 | T1D | Both | 4.38 (4.21, 4.56） |
| Western Pacific Region | 30 to 34 | T1D | Female | 3.92 (3.77, 4.06） |
| Western Pacific Region | 30 to 34 | T1D | Male | 4.83 (4.63, 5.05） |
| Western Pacific Region | 35 to 39 | T1D | Both | 4.91 (4.71, 5.11） |
| Western Pacific Region | 35 to 39 | T1D | Female | 4.31 (4.15, 4.48） |
| Western Pacific Region | 35 to 39 | T1D | Male | 5.50 (5.25, 5.75） |
| Western Pacific Region | 40 to 44 | T1D | Both | 5.50 (5.27, 5.73） |
| Western Pacific Region | 40 to 44 | T1D | Female | 4.75 (4.57, 4.94） |
| Western Pacific Region | 40 to 44 | T1D | Male | 6.23 (5.95, 6.53） |
| Western Pacific Region | 45 to 49 | T1D | Both | 6.09 (5.84, 6.36） |
| Western Pacific Region | 45 to 49 | T1D | Female | 5.21 (5.01, 5.43） |
| Western Pacific Region | 45 to 49 | T1D | Male | 6.98 (6.66, 7.31） |
| Western Pacific Region | 5 to 9 | T1D | Both | 1.00 (1.00, 1.00） |
| Western Pacific Region | 5 to 9 | T1D | Female | 1.00 (1.00, 1.00） |
| Western Pacific Region | 5 to 9 | T1D | Male | 1.00 (1.00, 1.00） |
| Western Pacific Region | 50 to 54 | T1D | Both | 6.70 (6.41, 7.00） |
| Western Pacific Region | 50 to 54 | T1D | Female | 5.68 (5.45, 5.92） |
| Western Pacific Region | 50 to 54 | T1D | Male | 7.74 (7.37, 8.12） |
| Western Pacific Region | 55 to 59 | T1D | Both | 7.34 (7.02, 7.68） |
| Western Pacific Region | 55 to 59 | T1D | Female | 6.18 (5.92, 6.45） |
| Western Pacific Region | 55 to 59 | T1D | Male | 8.54 (8.12, 8.97） |
| Western Pacific Region | 60 to 64 | T1D | Both | 8.01 (7.64, 8.38） |
| Western Pacific Region | 60 to 64 | T1D | Female | 6.71 (6.42, 7.02） |
| Western Pacific Region | 60 to 64 | T1D | Male | 9.36 (8.89, 9.85） |
| Western Pacific Region | 65 to 69 | T1D | Both | 8.78 (8.36, 9.21） |
| Western Pacific Region | 65 to 69 | T1D | Female | 7.34 (7.01, 7.69） |
| Western Pacific Region | 65 to 69 | T1D | Male | 10.32 (9.78, 10.88） |
| Afghanistan | 0 to 4 | T1D | Both | 0.13 (0.12, 0.13） |

| | | | | |
|---|---|---|---|---|
| Afghanistan | 0 to 4 | T1D | Female | 0.13 (0.12, 0.14） |
| Afghanistan | 0 to 4 | T1D | Male | 0.12 (0.11, 0.13） |
| Afghanistan | 10 to 14 | T1D | Both | 2.52 (2.47, 2.58） |
| Afghanistan | 10 to 14 | T1D | Female | 2.66 (2.58, 2.75） |
| Afghanistan | 10 to 14 | T1D | Male | 2.40 (2.33, 2.47） |
| Afghanistan | 15 to 19 | T1D | Both | 3.91 (3.82, 3.99） |
| Afghanistan | 15 to 19 | T1D | Female | 4.18 (4.04, 4.32） |
| Afghanistan | 15 to 19 | T1D | Male | 3.68 (3.57, 3.80） |
| Afghanistan | 20 to 24 | T1D | Both | 5.04 (4.92, 5.16） |
| Afghanistan | 20 to 24 | T1D | Female | 5.33 (5.15, 5.52） |
| Afghanistan | 20 to 24 | T1D | Male | 4.80 (4.65, 4.96） |
| Afghanistan | 25 to 29 | T1D | Both | 6.22 (6.06, 6.37） |
| Afghanistan | 25 to 29 | T1D | Female | 6.48 (6.24, 6.72） |
| Afghanistan | 25 to 29 | T1D | Male | 6.00 (5.79, 6.21） |
| Afghanistan | 30 to 34 | T1D | Both | 7.31 (7.12, 7.51） |
| Afghanistan | 30 to 34 | T1D | Female | 7.54 (7.25, 7.84） |
| Afghanistan | 30 to 34 | T1D | Male | 7.12 (6.87, 7.39） |
| Afghanistan | 35 to 39 | T1D | Both | 8.39 (8.16, 8.63） |
| Afghanistan | 35 to 39 | T1D | Female | 8.58 (8.23, 8.95） |
| Afghanistan | 35 to 39 | T1D | Male | 8.23 (7.92, 8.55） |
| Afghanistan | 40 to 44 | T1D | Both | 9.43 (9.15, 9.72） |
| Afghanistan | 40 to 44 | T1D | Female | 9.61 (9.20, 10.04） |
| Afghanistan | 40 to 44 | T1D | Male | 9.27 (8.90, 9.66） |
| Afghanistan | 45 to 49 | T1D | Both | 10.45 (10.12, 10.79） |
| Afghanistan | 45 to 49 | T1D | Female | 10.64 (10.15, 11.15） |
| Afghanistan | 45 to 49 | T1D | Male | 10.28 (9.84, 10.75） |
| Afghanistan | 5 to 9 | T1D | Both | 1.00 (1.00, 1.00） |
| Afghanistan | 5 to 9 | T1D | Female | 1.00 (1.00, 1.00） |
| Afghanistan | 5 to 9 | T1D | Male | 1.00 (1.00, 1.00） |
| Afghanistan | 50 to 54 | T1D | Both | 11.50 (11.09, 11.92） |
| Afghanistan | 50 to 54 | T1D | Female | 11.72 (11.13, 12.35） |
| Afghanistan | 50 to 54 | T1D | Male | 11.30 (10.75, 11.88） |
| Afghanistan | 55 to 59 | T1D | Both | 12.58 (12.08, 13.09） |

| | | | | |
|---|---|---|---|---|
| Afghanistan | 55 to 59 | T1D | Female | 12.85 (12.14, 13.61） |
| Afghanistan | 55 to 59 | T1D | Male | 12.32 (11.64, 13.03） |
| Afghanistan | 60 to 64 | T1D | Both | 13.62 (13.05, 14.23） |
| Afghanistan | 60 to 64 | T1D | Female | 13.97 (13.14, 14.86） |
| Afghanistan | 60 to 64 | T1D | Male | 13.29 (12.50, 14.13） |
| Afghanistan | 65 to 69 | T1D | Both | 14.64 (13.96, 15.34） |
| Afghanistan | 65 to 69 | T1D | Female | 15.10 (14.11, 16.15） |
| Afghanistan | 65 to 69 | T1D | Male | 14.20 (13.28, 15.17） |
| Albania | 0 to 4 | T1D | Both | 0.18 (0.15, 0.21） |
| Albania | 0 to 4 | T1D | Female | 0.18 (0.14, 0.22） |
| Albania | 0 to 4 | T1D | Male | 0.19 (0.15, 0.23） |
| Albania | 10 to 14 | T1D | Both | 2.20 (2.04, 2.37） |
| Albania | 10 to 14 | T1D | Female | 2.18 (1.96, 2.43） |
| Albania | 10 to 14 | T1D | Male | 2.20 (1.97, 2.44） |
| Albania | 15 to 19 | T1D | Both | 3.30 (3.06, 3.55） |
| Albania | 15 to 19 | T1D | Female | 3.23 (2.90, 3.58） |
| Albania | 15 to 19 | T1D | Male | 3.36 (3.03, 3.73） |
| Albania | 20 to 24 | T1D | Both | 4.21 (3.90, 4.54） |
| Albania | 20 to 24 | T1D | Female | 4.03 (3.62, 4.49） |
| Albania | 20 to 24 | T1D | Male | 4.38 (3.94, 4.86） |
| Albania | 25 to 29 | T1D | Both | 5.16 (4.78, 5.57） |
| Albania | 25 to 29 | T1D | Female | 4.83 (4.32, 5.40） |
| Albania | 25 to 29 | T1D | Male | 5.49 (4.92, 6.11） |
| Albania | 30 to 34 | T1D | Both | 5.98 (5.52, 6.48） |
| Albania | 30 to 34 | T1D | Female | 5.52 (4.93, 6.19） |
| Albania | 30 to 34 | T1D | Male | 6.46 (5.78, 7.22） |
| Albania | 35 to 39 | T1D | Both | 6.77 (6.24, 7.35） |
| Albania | 35 to 39 | T1D | Female | 6.18 (5.50, 6.95） |
| Albania | 35 to 39 | T1D | Male | 7.39 (6.59, 8.29） |
| Albania | 40 to 44 | T1D | Both | 7.58 (6.97, 8.23） |
| Albania | 40 to 44 | T1D | Female | 6.86 (6.08, 7.73） |
| Albania | 40 to 44 | T1D | Male | 8.33 (7.42, 9.36） |
| Albania | 45 to 49 | T1D | Both | 8.38 (7.70, 9.13） |

| | | | | |
|---|---|---|---|---|
| Albania | 45 to 49 | T1D | Female | 7.55 (6.68, 8.53） |
| Albania | 45 to 49 | T1D | Male | 9.26 (8.22, 10.43） |
| Albania | 5 to 9 | T1D | Both | 1.00 (1.00, 1.00） |
| Albania | 5 to 9 | T1D | Female | 1.00 (1.00, 1.00） |
| Albania | 5 to 9 | T1D | Male | 1.00 (1.00, 1.00） |
| Albania | 50 to 54 | T1D | Both | 9.18 (8.41, 10.02） |
| Albania | 50 to 54 | T1D | Female | 8.27 (7.29, 9.37） |
| Albania | 50 to 54 | T1D | Male | 10.16 (9.00, 11.47） |
| Albania | 55 to 59 | T1D | Both | 9.98 (9.12, 10.92） |
| Albania | 55 to 59 | T1D | Female | 9.00 (7.90, 10.24） |
| Albania | 55 to 59 | T1D | Male | 11.05 (9.75, 12.52） |
| Albania | 60 to 64 | T1D | Both | 10.80 (9.83, 11.85） |
| Albania | 60 to 64 | T1D | Female | 9.78 (8.54, 11.19） |
| Albania | 60 to 64 | T1D | Male | 11.93 (10.49, 13.58） |
| Albania | 65 to 69 | T1D | Both | 11.65 (10.57, 12.85） |
| Albania | 65 to 69 | T1D | Female | 10.60 (9.20, 12.22） |
| Albania | 65 to 69 | T1D | Male | 12.86 (11.23, 14.72） |
| Algeria | 0 to 4 | T1D | Both | 0.14 (0.13, 0.14） |
| Algeria | 0 to 4 | T1D | Female | 0.15 (0.14, 0.16） |
| Algeria | 0 to 4 | T1D | Male | 0.13 (0.12, 0.14） |
| Algeria | 10 to 14 | T1D | Both | 2.38 (2.32, 2.43） |
| Algeria | 10 to 14 | T1D | Female | 2.47 (2.39, 2.55） |
| Algeria | 10 to 14 | T1D | Male | 2.30 (2.23, 2.37） |
| Algeria | 15 to 19 | T1D | Both | 3.69 (3.61, 3.77） |
| Algeria | 15 to 19 | T1D | Female | 3.91 (3.78, 4.03） |
| Algeria | 15 to 19 | T1D | Male | 3.50 (3.40, 3.61） |
| Algeria | 20 to 24 | T1D | Both | 4.89 (4.78, 5.00） |
| Algeria | 20 to 24 | T1D | Female | 5.16 (4.99, 5.33） |
| Algeria | 20 to 24 | T1D | Male | 4.66 (4.52, 4.81） |
| Algeria | 25 to 29 | T1D | Both | 6.28 (6.13, 6.42） |
| Algeria | 25 to 29 | T1D | Female | 6.56 (6.35, 6.78） |
| Algeria | 25 to 29 | T1D | Male | 6.04 (5.85, 6.23） |
| Algeria | 30 to 34 | T1D | Both | 7.71 (7.53, 7.89） |

| | | | | |
|---|---|---|---|---|
| Algeria | 30 to 34 | T1D | Female | 8.00 (7.73, 8.28） |
| Algeria | 30 to 34 | T1D | Male | 7.47 (7.23, 7.71） |
| Algeria | 35 to 39 | T1D | Both | 9.26 (9.04, 9.49） |
| Algeria | 35 to 39 | T1D | Female | 9.55 (9.21, 9.89） |
| Algeria | 35 to 39 | T1D | Male | 9.03 (8.73, 9.34） |
| Algeria | 40 to 44 | T1D | Both | 10.99 (10.72, 11.27） |
| Algeria | 40 to 44 | T1D | Female | 11.27 (10.86, 11.70） |
| Algeria | 40 to 44 | T1D | Male | 10.77 (10.41, 11.16） |
| Algeria | 45 to 49 | T1D | Both | 12.92 (12.59, 13.27） |
| Algeria | 45 to 49 | T1D | Female | 13.20 (12.70, 13.72） |
| Algeria | 45 to 49 | T1D | Male | 12.72 (12.26, 13.19） |
| Algeria | 5 to 9 | T1D | Both | 1.00 (1.00, 1.00） |
| Algeria | 5 to 9 | T1D | Female | 1.00 (1.00, 1.00） |
| Algeria | 5 to 9 | T1D | Male | 1.00 (1.00, 1.00） |
| Algeria | 50 to 54 | T1D | Both | 15.07 (14.66, 15.50） |
| Algeria | 50 to 54 | T1D | Female | 15.37 (14.76, 16.00） |
| Algeria | 50 to 54 | T1D | Male | 14.85 (14.30, 15.43） |
| Algeria | 55 to 59 | T1D | Both | 17.48 (16.97, 18.00） |
| Algeria | 55 to 59 | T1D | Female | 17.80 (17.06, 18.58） |
| Algeria | 55 to 59 | T1D | Male | 17.22 (16.54, 17.93） |
| Algeria | 60 to 64 | T1D | Both | 20.18 (19.56, 20.82） |
| Algeria | 60 to 64 | T1D | Female | 20.59 (19.67, 21.54） |
| Algeria | 60 to 64 | T1D | Male | 19.86 (19.02, 20.74） |
| Algeria | 65 to 69 | T1D | Both | 23.29 (22.52, 24.08） |
| Algeria | 65 to 69 | T1D | Female | 23.80 (22.68, 24.98） |
| Algeria | 65 to 69 | T1D | Male | 22.87 (21.84, 23.96） |
| American Samoa | 0 to 4 | T1D | Both | 0.05 (0.01, 0.77） |
| American Samoa | 0 to 4 | T1D | Female | 0.09 (0.01, 1.62） |
| American Samoa | 0 to 4 | T1D | Male | 0.09 (0.01, 1.63） |
| American Samoa | 10 to 14 | T1D | Both | 1.75 (0.81, 3.77） |
| American Samoa | 10 to 14 | T1D | Female | 1.87 (0.64, 5.47） |
| American Samoa | 10 to 14 | T1D | Male | 1.88 (0.64, 5.50） |
| American Samoa | 15 to 19 | T1D | Both | 2.54 (1.18, 5.51） |

| | | | | |
|---|---|---|---|---|
| American Samoa | 15 to 19 | T1D | Female | 2.59 (0.87, 7.73） |
| American Samoa | 15 to 19 | T1D | Male | 2.68 (0.90, 8.00） |
| American Samoa | 20 to 24 | T1D | Both | 3.29 (1.47, 7.35） |
| American Samoa | 20 to 24 | T1D | Female | 3.29 (1.06, 10.23） |
| American Samoa | 20 to 24 | T1D | Male | 3.47 (1.12, 10.79） |
| American Samoa | 25 to 29 | T1D | Both | 3.84 (1.67, 8.86） |
| American Samoa | 25 to 29 | T1D | Female | 3.82 (1.17, 12.43） |
| American Samoa | 25 to 29 | T1D | Male | 4.06 (1.25, 13.19） |
| American Samoa | 30 to 34 | T1D | Both | 4.67 (1.98, 11.03） |
| American Samoa | 30 to 34 | T1D | Female | 4.08 (1.20, 13.93） |
| American Samoa | 30 to 34 | T1D | Male | 4.93 (1.46, 16.67） |
| American Samoa | 35 to 39 | T1D | Both | 4.98 (2.04, 12.14） |
| American Samoa | 35 to 39 | T1D | Female | 4.37 (1.22, 15.65） |
| American Samoa | 35 to 39 | T1D | Male | 5.80 (1.65, 20.37） |
| American Samoa | 40 to 44 | T1D | Both | 5.89 (2.36, 14.72） |
| American Samoa | 40 to 44 | T1D | Female | 4.64 (1.24, 17.34） |
| American Samoa | 40 to 44 | T1D | Male | 6.94 (1.94, 24.86） |
| American Samoa | 45 to 49 | T1D | Both | 6.31 (2.44, 16.29） |
| American Samoa | 45 to 49 | T1D | Female | 5.01 (1.27, 19.74） |
| American Samoa | 45 to 49 | T1D | Male | 7.31 (1.94, 27.52） |
| American Samoa | 5 to 9 | T1D | Both | 1.00 (1.00, 1.00） |
| American Samoa | 5 to 9 | T1D | Female | 1.00 (1.00, 1.00） |
| American Samoa | 5 to 9 | T1D | Male | 1.00 (1.00, 1.00） |
| American Samoa | 50 to 54 | T1D | Both | 7.00 (2.59, 18.89） |
| American Samoa | 50 to 54 | T1D | Female | 5.51 (1.32, 23.04） |
| American Samoa | 50 to 54 | T1D | Male | 8.04 (2.02, 32.06） |
| American Samoa | 55 to 59 | T1D | Both | 7.52 (2.64, 21.40） |
| American Samoa | 55 to 59 | T1D | Female | 6.08 (1.36, 27.26） |
| American Samoa | 55 to 59 | T1D | Male | 8.96 (2.09, 38.39） |
| American Samoa | 60 to 64 | T1D | Both | 8.37 (2.74, 25.59） |
| American Samoa | 60 to 64 | T1D | Female | 6.10 (1.18, 31.46） |
| American Samoa | 60 to 64 | T1D | Male | 9.45 (1.99, 44.84） |
| American Samoa | 65 to 69 | T1D | Both | 8.66 (2.53, 29.64） |

| | | | | |
|---|---|---|---|---|
| American Samoa | 65 to 69 | T1D | Female | 8.05 (1.44, 45.13） |
| American Samoa | 65 to 69 | T1D | Male | 11.36 (2.12, 60.88） |
| Andorra | 0 to 4 | T1D | Both | 0.19 (0.07, 0.48） |
| Andorra | 0 to 4 | T1D | Female | 0.33 (0.10, 1.14） |
| Andorra | 0 to 4 | T1D | Male | 0.33 (0.11, 0.99） |
| Andorra | 10 to 14 | T1D | Both | 2.01 (1.29, 3.15） |
| Andorra | 10 to 14 | T1D | Female | 2.13 (1.10, 4.14） |
| Andorra | 10 to 14 | T1D | Male | 2.13 (1.13, 4.01） |
| Andorra | 15 to 19 | T1D | Both | 3.09 (1.98, 4.84） |
| Andorra | 15 to 19 | T1D | Female | 3.18 (1.64, 6.19） |
| Andorra | 15 to 19 | T1D | Male | 3.27 (1.74, 6.13） |
| Andorra | 20 to 24 | T1D | Both | 4.12 (2.62, 6.48） |
| Andorra | 20 to 24 | T1D | Female | 4.22 (2.16, 8.24） |
| Andorra | 20 to 24 | T1D | Male | 4.45 (2.36, 8.37） |
| Andorra | 25 to 29 | T1D | Both | 5.38 (3.41, 8.47） |
| Andorra | 25 to 29 | T1D | Female | 5.37 (2.75, 10.49） |
| Andorra | 25 to 29 | T1D | Male | 5.84 (3.09, 11.04） |
| Andorra | 30 to 34 | T1D | Both | 6.57 (4.16, 10.39） |
| Andorra | 30 to 34 | T1D | Female | 6.57 (3.35, 12.92） |
| Andorra | 30 to 34 | T1D | Male | 7.09 (3.73, 13.46） |
| Andorra | 35 to 39 | T1D | Both | 7.89 (4.96, 12.54） |
| Andorra | 35 to 39 | T1D | Female | 7.85 (3.96, 15.56） |
| Andorra | 35 to 39 | T1D | Male | 8.56 (4.48, 16.37） |
| Andorra | 40 to 44 | T1D | Both | 9.31 (5.82, 14.87） |
| Andorra | 40 to 44 | T1D | Female | 9.12 (4.56, 18.24） |
| Andorra | 40 to 44 | T1D | Male | 10.23 (5.32, 19.68） |
| Andorra | 45 to 49 | T1D | Both | 10.87 (6.76, 17.48） |
| Andorra | 45 to 49 | T1D | Female | 10.56 (5.23, 21.32） |
| Andorra | 45 to 49 | T1D | Male | 11.90 (6.14, 23.06） |
| Andorra | 5 to 9 | T1D | Both | 1.00 (1.00, 1.00） |
| Andorra | 5 to 9 | T1D | Female | 1.00 (1.00, 1.00） |
| Andorra | 5 to 9 | T1D | Male | 1.00 (1.00, 1.00） |
| Andorra | 50 to 54 | T1D | Both | 12.63 (7.80, 20.45） |

| | | | | |
|---|---|---|---|---|
| Andorra | 50 to 54 | T1D | Female | 12.24 (6.00, 24.99） |
| Andorra | 50 to 54 | T1D | Male | 13.80 (7.06, 26.97） |
| Andorra | 55 to 59 | T1D | Both | 14.44 (8.84, 23.60） |
| Andorra | 55 to 59 | T1D | Female | 14.11 (6.81, 29.23） |
| Andorra | 55 to 59 | T1D | Male | 15.91 (8.05, 31.45） |
| Andorra | 60 to 64 | T1D | Both | 16.55 (10.01, 27.34） |
| Andorra | 60 to 64 | T1D | Female | 15.90 (7.54, 33.51） |
| Andorra | 60 to 64 | T1D | Male | 18.19 (9.08, 36.47） |
| Andorra | 65 to 69 | T1D | Both | 18.81 (11.24, 31.49） |
| Andorra | 65 to 69 | T1D | Female | 17.96 (8.35, 38.62） |
| Andorra | 65 to 69 | T1D | Male | 20.40 (10.01, 41.61） |
| Angola | 0 to 4 | T1D | Both | 0.15 (0.14, 0.16） |
| Angola | 0 to 4 | T1D | Female | 0.15 (0.14, 0.16） |
| Angola | 0 to 4 | T1D | Male | 0.15 (0.14, 0.16） |
| Angola | 10 to 14 | T1D | Both | 2.10 (2.04, 2.16） |
| Angola | 10 to 14 | T1D | Female | 2.10 (2.02, 2.19） |
| Angola | 10 to 14 | T1D | Male | 2.10 (2.02, 2.19） |
| Angola | 15 to 19 | T1D | Both | 3.07 (2.98, 3.16） |
| Angola | 15 to 19 | T1D | Female | 3.07 (2.95, 3.21） |
| Angola | 15 to 19 | T1D | Male | 3.07 (2.95, 3.20） |
| Angola | 20 to 24 | T1D | Both | 3.87 (3.75, 3.99） |
| Angola | 20 to 24 | T1D | Female | 3.88 (3.71, 4.05） |
| Angola | 20 to 24 | T1D | Male | 3.87 (3.70, 4.03） |
| Angola | 25 to 29 | T1D | Both | 4.65 (4.51, 4.80） |
| Angola | 25 to 29 | T1D | Female | 4.68 (4.48, 4.89） |
| Angola | 25 to 29 | T1D | Male | 4.64 (4.44, 4.85） |
| Angola | 30 to 34 | T1D | Both | 5.20 (5.03, 5.37） |
| Angola | 30 to 34 | T1D | Female | 5.25 (5.01, 5.50） |
| Angola | 30 to 34 | T1D | Male | 5.17 (4.93, 5.41） |
| Angola | 35 to 39 | T1D | Both | 5.59 (5.40, 5.78） |
| Angola | 35 to 39 | T1D | Female | 5.66 (5.39, 5.94） |
| Angola | 35 to 39 | T1D | Male | 5.54 (5.28, 5.82） |
| Angola | 40 to 44 | T1D | Both | 5.89 (5.68, 6.11） |

| | | | | |
|---|---|---|---|---|
| Angola | 40 to 44 | T1D | Female | 5.98 (5.68, 6.30） |
| Angola | 40 to 44 | T1D | Male | 5.83 (5.54, 6.13） |
| Angola | 45 to 49 | T1D | Both | 6.16 (5.93, 6.41） |
| Angola | 45 to 49 | T1D | Female | 6.28 (5.94, 6.63） |
| Angola | 45 to 49 | T1D | Male | 6.08 (5.76, 6.42） |
| Angola | 5 to 9 | T1D | Both | 1.00 (1.00, 1.00） |
| Angola | 5 to 9 | T1D | Female | 1.00 (1.00, 1.00） |
| Angola | 5 to 9 | T1D | Male | 1.00 (1.00, 1.00） |
| Angola | 50 to 54 | T1D | Both | 6.43 (6.17, 6.70） |
| Angola | 50 to 54 | T1D | Female | 6.57 (6.20, 6.96） |
| Angola | 50 to 54 | T1D | Male | 6.33 (5.97, 6.70） |
| Angola | 55 to 59 | T1D | Both | 6.67 (6.38, 6.97） |
| Angola | 55 to 59 | T1D | Female | 6.83 (6.42, 7.27） |
| Angola | 55 to 59 | T1D | Male | 6.54 (6.14, 6.96） |
| Angola | 60 to 64 | T1D | Both | 6.86 (6.53, 7.20） |
| Angola | 60 to 64 | T1D | Female | 7.06 (6.59, 7.56） |
| Angola | 60 to 64 | T1D | Male | 6.72 (6.27, 7.20） |
| Angola | 65 to 69 | T1D | Both | 7.01 (6.63, 7.40） |
| Angola | 65 to 69 | T1D | Female | 7.23 (6.69, 7.81） |
| Angola | 65 to 69 | T1D | Male | 6.84 (6.32, 7.39） |
| Antigua and Barbuda | 0 to 4 | T1D | Both | 0.17 (0.03, 0.97） |
| Antigua and Barbuda | 0 to 4 | T1D | Female | 0.07 (0.01, 1.15） |
| Antigua and Barbuda | 0 to 4 | T1D | Male | 0.07 (0.01, 1.28） |
| Antigua and Barbuda | 10 to 14 | T1D | Both | 2.25 (1.21, 4.16） |
| Antigua and Barbuda | 10 to 14 | T1D | Female | 2.19 (0.96, 5.00） |
| Antigua and Barbuda | 10 to 14 | T1D | Male | 1.96 (0.80, 4.78） |
| Antigua and Barbuda | 15 to 19 | T1D | Both | 3.31 (1.79, 6.12） |
| Antigua and Barbuda | 15 to 19 | T1D | Female | 2.98 (1.29, 6.90） |
| Antigua and Barbuda | 15 to 19 | T1D | Male | 3.13 (1.31, 7.46） |
| Antigua and Barbuda | 20 to 24 | T1D | Both | 4.42 (2.36, 8.27） |
| Antigua and Barbuda | 20 to 24 | T1D | Female | 3.75 (1.59, 8.85） |
| Antigua and Barbuda | 20 to 24 | T1D | Male | 4.12 (1.68, 10.07） |
| Antigua and Barbuda | 25 to 29 | T1D | Both | 5.29 (2.80, 10.02） |

| | | | | |
|---|---|---|---|---|
| Antigua and Barbuda | 25 to 29 | T1D | Female | 4.53 (1.88, 10.91） |
| Antigua and Barbuda | 25 to 29 | T1D | Male | 5.28 (2.12, 13.18） |
| Antigua and Barbuda | 30 to 34 | T1D | Both | 6.23 (3.25, 11.95） |
| Antigua and Barbuda | 30 to 34 | T1D | Female | 5.02 (2.05, 12.28） |
| Antigua and Barbuda | 30 to 34 | T1D | Male | 6.42 (2.53, 16.28） |
| Antigua and Barbuda | 35 to 39 | T1D | Both | 7.09 (3.64, 13.79） |
| Antigua and Barbuda | 35 to 39 | T1D | Female | 5.43 (2.17, 13.58） |
| Antigua and Barbuda | 35 to 39 | T1D | Male | 7.54 (2.91, 19.54） |
| Antigua and Barbuda | 40 to 44 | T1D | Both | 8.04 (4.07, 15.87） |
| Antigua and Barbuda | 40 to 44 | T1D | Female | 6.12 (2.40, 15.63） |
| Antigua and Barbuda | 40 to 44 | T1D | Male | 8.63 (3.27, 22.81） |
| Antigua and Barbuda | 45 to 49 | T1D | Both | 8.92 (4.45, 17.91） |
| Antigua and Barbuda | 45 to 49 | T1D | Female | 6.89 (2.63, 18.02） |
| Antigua and Barbuda | 45 to 49 | T1D | Male | 9.62 (3.55, 26.07） |
| Antigua and Barbuda | 5 to 9 | T1D | Both | 1.00 (1.00, 1.00） |
| Antigua and Barbuda | 5 to 9 | T1D | Female | 1.00 (1.00, 1.00） |
| Antigua and Barbuda | 5 to 9 | T1D | Male | 1.00 (1.00, 1.00） |
| Antigua and Barbuda | 50 to 54 | T1D | Both | 9.71 (4.74, 19.91） |
| Antigua and Barbuda | 50 to 54 | T1D | Female | 7.27 (2.69, 19.66） |
| Antigua and Barbuda | 50 to 54 | T1D | Male | 10.69 (3.82, 29.87） |
| Antigua and Barbuda | 55 to 59 | T1D | Both | 10.82 (5.13, 22.83） |
| Antigua and Barbuda | 55 to 59 | T1D | Female | 7.60 (2.68, 21.51） |
| Antigua and Barbuda | 55 to 59 | T1D | Male | 12.21 (4.20, 35.51） |
| Antigua and Barbuda | 60 to 64 | T1D | Both | 11.34 (5.20, 24.74） |
| Antigua and Barbuda | 60 to 64 | T1D | Female | 8.27 (2.78, 24.59） |
| Antigua and Barbuda | 60 to 64 | T1D | Male | 14.36 (4.75, 43.38） |
| Antigua and Barbuda | 65 to 69 | T1D | Both | 12.30 (5.42, 27.91） |
| Antigua and Barbuda | 65 to 69 | T1D | Female | 9.21 (2.92, 29.04） |
| Antigua and Barbuda | 65 to 69 | T1D | Male | 15.89 (4.98, 50.67） |
| Argentina | 0 to 4 | T1D | Both | 0.19 (0.19, 0.20） |
| Argentina | 0 to 4 | T1D | Female | 0.18 (0.17, 0.19） |
| Argentina | 0 to 4 | T1D | Male | 0.20 (0.19, 0.21） |
| Argentina | 10 to 14 | T1D | Both | 1.82 (1.79, 1.85） |

| | | | | |
|---|---|---|---|---|
| Argentina | 10 to 14 | T1D | Female | 1.81 (1.78, 1.85） |
| Argentina | 10 to 14 | T1D | Male | 1.83 (1.79, 1.87） |
| Argentina | 15 to 19 | T1D | Both | 2.23 (2.19, 2.26） |
| Argentina | 15 to 19 | T1D | Female | 2.20 (2.16, 2.25） |
| Argentina | 15 to 19 | T1D | Male | 2.25 (2.20, 2.29） |
| Argentina | 20 to 24 | T1D | Both | 2.45 (2.41, 2.50） |
| Argentina | 20 to 24 | T1D | Female | 2.42 (2.37, 2.48） |
| Argentina | 20 to 24 | T1D | Male | 2.49 (2.44, 2.54） |
| Argentina | 25 to 29 | T1D | Both | 2.69 (2.64, 2.74） |
| Argentina | 25 to 29 | T1D | Female | 2.65 (2.59, 2.71） |
| Argentina | 25 to 29 | T1D | Male | 2.73 (2.68, 2.79） |
| Argentina | 30 to 34 | T1D | Both | 2.91 (2.86, 2.97） |
| Argentina | 30 to 34 | T1D | Female | 2.86 (2.79, 2.93） |
| Argentina | 30 to 34 | T1D | Male | 2.97 (2.90, 3.03） |
| Argentina | 35 to 39 | T1D | Both | 3.14 (3.08, 3.20） |
| Argentina | 35 to 39 | T1D | Female | 3.08 (3.01, 3.16） |
| Argentina | 35 to 39 | T1D | Male | 3.20 (3.13, 3.28） |
| Argentina | 40 to 44 | T1D | Both | 3.38 (3.31, 3.45） |
| Argentina | 40 to 44 | T1D | Female | 3.31 (3.23, 3.40） |
| Argentina | 40 to 44 | T1D | Male | 3.45 (3.37, 3.54） |
| Argentina | 45 to 49 | T1D | Both | 3.63 (3.55, 3.71） |
| Argentina | 45 to 49 | T1D | Female | 3.56 (3.47, 3.66） |
| Argentina | 45 to 49 | T1D | Male | 3.70 (3.61, 3.80） |
| Argentina | 5 to 9 | T1D | Both | 1.00 (1.00, 1.00） |
| Argentina | 5 to 9 | T1D | Female | 1.00 (1.00, 1.00） |
| Argentina | 5 to 9 | T1D | Male | 1.00 (1.00, 1.00） |
| Argentina | 50 to 54 | T1D | Both | 3.88 (3.80, 3.98） |
| Argentina | 50 to 54 | T1D | Female | 3.82 (3.71, 3.93） |
| Argentina | 50 to 54 | T1D | Male | 3.97 (3.86, 4.07） |
| Argentina | 55 to 59 | T1D | Both | 4.15 (4.05, 4.25） |
| Argentina | 55 to 59 | T1D | Female | 4.08 (3.96, 4.20） |
| Argentina | 55 to 59 | T1D | Male | 4.23 (4.11, 4.35） |
| Argentina | 60 to 64 | T1D | Both | 4.42 (4.31, 4.53） |

| | | | | |
|---|---|---|---|---|
| Argentina | 60 to 64 | T1D | Female | 4.36 (4.23, 4.50） |
| Argentina | 60 to 64 | T1D | Male | 4.51 (4.37, 4.65） |
| Argentina | 65 to 69 | T1D | Both | 4.71 (4.59, 4.84） |
| Argentina | 65 to 69 | T1D | Female | 4.66 (4.51, 4.82） |
| Argentina | 65 to 69 | T1D | Male | 4.80 (4.65, 4.96） |
| Armenia | 0 to 4 | T1D | Both | 0.17 (0.14, 0.20） |
| Armenia | 0 to 4 | T1D | Female | 0.16 (0.12, 0.22） |
| Armenia | 0 to 4 | T1D | Male | 0.17 (0.13, 0.22） |
| Armenia | 10 to 14 | T1D | Both | 2.19 (2.02, 2.38） |
| Armenia | 10 to 14 | T1D | Female | 2.19 (1.95, 2.47） |
| Armenia | 10 to 14 | T1D | Male | 2.20 (1.96, 2.46） |
| Armenia | 15 to 19 | T1D | Both | 3.38 (3.11, 3.66） |
| Armenia | 15 to 19 | T1D | Female | 3.34 (2.97, 3.75） |
| Armenia | 15 to 19 | T1D | Male | 3.42 (3.06, 3.83） |
| Armenia | 20 to 24 | T1D | Both | 4.44 (4.09, 4.81） |
| Armenia | 20 to 24 | T1D | Female | 4.30 (3.83, 4.84） |
| Armenia | 20 to 24 | T1D | Male | 4.57 (4.09, 5.11） |
| Armenia | 25 to 29 | T1D | Both | 5.58 (5.14, 6.06） |
| Armenia | 25 to 29 | T1D | Female | 5.31 (4.72, 5.98） |
| Armenia | 25 to 29 | T1D | Male | 5.86 (5.24, 6.56） |
| Armenia | 30 to 34 | T1D | Both | 6.58 (6.06, 7.16） |
| Armenia | 30 to 34 | T1D | Female | 6.18 (5.48, 6.98） |
| Armenia | 30 to 34 | T1D | Male | 7.00 (6.24, 7.86） |
| Armenia | 35 to 39 | T1D | Both | 7.50 (6.88, 8.17） |
| Armenia | 35 to 39 | T1D | Female | 6.99 (6.17, 7.91） |
| Armenia | 35 to 39 | T1D | Male | 8.05 (7.15, 9.07） |
| Armenia | 40 to 44 | T1D | Both | 8.39 (7.69, 9.15） |
| Armenia | 40 to 44 | T1D | Female | 7.76 (6.84, 8.80） |
| Armenia | 40 to 44 | T1D | Male | 9.06 (8.03, 10.23） |
| Armenia | 45 to 49 | T1D | Both | 9.28 (8.49, 10.14） |
| Armenia | 45 to 49 | T1D | Female | 8.56 (7.53, 9.73） |
| Armenia | 45 to 49 | T1D | Male | 10.07 (8.90, 11.38） |
| Armenia | 5 to 9 | T1D | Both | 1.00 (1.00, 1.00） |

| | | | | |
|---|---|---|---|---|
| Armenia | 5 to 9 | T1D | Female | 1.00 (1.00, 1.00） |
| Armenia | 5 to 9 | T1D | Male | 1.00 (1.00, 1.00） |
| Armenia | 50 to 54 | T1D | Both | 10.19 (9.31, 11.15） |
| Armenia | 50 to 54 | T1D | Female | 9.41 (8.26, 10.72） |
| Armenia | 50 to 54 | T1D | Male | 11.09 (9.78, 12.56） |
| Armenia | 55 to 59 | T1D | Both | 11.08 (10.10, 12.16） |
| Armenia | 55 to 59 | T1D | Female | 10.26 (8.98, 11.73） |
| Armenia | 55 to 59 | T1D | Male | 12.06 (10.60, 13.72） |
| Armenia | 60 to 64 | T1D | Both | 11.95 (10.86, 13.15） |
| Armenia | 60 to 64 | T1D | Female | 11.13 (9.70, 12.78） |
| Armenia | 60 to 64 | T1D | Male | 12.99 (11.37, 14.84） |
| Armenia | 65 to 69 | T1D | Both | 12.87 (11.65, 14.22） |
| Armenia | 65 to 69 | T1D | Female | 12.07 (10.46, 13.93） |
| Armenia | 65 to 69 | T1D | Male | 13.94 (12.12, 16.03） |
| Australia | 0 to 4 | T1D | Both | 0.24 (0.23, 0.26） |
| Australia | 0 to 4 | T1D | Female | 0.24 (0.22, 0.25） |
| Australia | 0 to 4 | T1D | Male | 0.25 (0.23, 0.27） |
| Australia | 10 to 14 | T1D | Both | 2.22 (2.17, 2.28） |
| Australia | 10 to 14 | T1D | Female | 2.21 (2.14, 2.29） |
| Australia | 10 to 14 | T1D | Male | 2.24 (2.16, 2.31） |
| Australia | 15 to 19 | T1D | Both | 3.47 (3.39, 3.55） |
| Australia | 15 to 19 | T1D | Female | 3.34 (3.23, 3.45） |
| Australia | 15 to 19 | T1D | Male | 3.61 (3.49, 3.73） |
| Australia | 20 to 24 | T1D | Both | 4.49 (4.38, 4.60） |
| Australia | 20 to 24 | T1D | Female | 4.20 (4.06, 4.35） |
| Australia | 20 to 24 | T1D | Male | 4.79 (4.62, 4.96） |
| Australia | 25 to 29 | T1D | Both | 5.51 (5.38, 5.65） |
| Australia | 25 to 29 | T1D | Female | 5.04 (4.86, 5.21） |
| Australia | 25 to 29 | T1D | Male | 6.03 (5.82, 6.25） |
| Australia | 30 to 34 | T1D | Both | 6.53 (6.37, 6.70） |
| Australia | 30 to 34 | T1D | Female | 5.82 (5.62, 6.03） |
| Australia | 30 to 34 | T1D | Male | 7.33 (7.07, 7.60） |
| Australia | 35 to 39 | T1D | Both | 7.58 (7.39, 7.78） |

| | | | | |
|---|---|---|---|---|
| Australia | 35 to 39 | T1D | Female | 6.62 (6.38, 6.86） |
| Australia | 35 to 39 | T1D | Male | 8.68 (8.36, 9.00） |
| Australia | 40 to 44 | T1D | Both | 8.68 (8.45, 8.91） |
| Australia | 40 to 44 | T1D | Female | 7.47 (7.20, 7.74） |
| Australia | 40 to 44 | T1D | Male | 10.07 (9.70, 10.45） |
| Australia | 45 to 49 | T1D | Both | 9.82 (9.57, 10.09） |
| Australia | 45 to 49 | T1D | Female | 8.35 (8.05, 8.67） |
| Australia | 45 to 49 | T1D | Male | 11.53 (11.10, 11.98） |
| Australia | 5 to 9 | T1D | Both | 1.00 (1.00, 1.00） |
| Australia | 5 to 9 | T1D | Female | 1.00 (1.00, 1.00） |
| Australia | 5 to 9 | T1D | Male | 1.00 (1.00, 1.00） |
| Australia | 50 to 54 | T1D | Both | 11.01 (10.72, 11.32） |
| Australia | 50 to 54 | T1D | Female | 9.28 (8.94, 9.64） |
| Australia | 50 to 54 | T1D | Male | 13.04 (12.55, 13.56） |
| Australia | 55 to 59 | T1D | Both | 12.26 (11.93, 12.60） |
| Australia | 55 to 59 | T1D | Female | 10.27 (9.88, 10.67） |
| Australia | 55 to 59 | T1D | Male | 14.61 (14.05, 15.20） |
| Australia | 60 to 64 | T1D | Both | 13.57 (13.20, 13.96） |
| Australia | 60 to 64 | T1D | Female | 11.32 (10.88, 11.77） |
| Australia | 60 to 64 | T1D | Male | 16.25 (15.62, 16.92） |
| Australia | 65 to 69 | T1D | Both | 14.99 (14.56, 15.43） |
| Australia | 65 to 69 | T1D | Female | 12.47 (11.97, 12.98） |
| Australia | 65 to 69 | T1D | Male | 18.01 (17.28, 18.77） |
| Austria | 0 to 4 | T1D | Both | 0.19 (0.18, 0.21） |
| Austria | 0 to 4 | T1D | Female | 0.19 (0.17, 0.22） |
| Austria | 0 to 4 | T1D | Male | 0.20 (0.17, 0.22） |
| Austria | 10 to 14 | T1D | Both | 2.12 (2.03, 2.22） |
| Austria | 10 to 14 | T1D | Female | 2.10 (1.97, 2.24） |
| Austria | 10 to 14 | T1D | Male | 2.13 (2.00, 2.27） |
| Austria | 15 to 19 | T1D | Both | 3.33 (3.18, 3.48） |
| Austria | 15 to 19 | T1D | Female | 3.29 (3.09, 3.50） |
| Austria | 15 to 19 | T1D | Male | 3.35 (3.15, 3.57） |
| Austria | 20 to 24 | T1D | Both | 4.51 (4.32, 4.72） |

| | | | | |
|---|---|---|---|---|
| Austria | 20 to 24 | T1D | Female | 4.44 (4.17, 4.73） |
| Austria | 20 to 24 | T1D | Male | 4.57 (4.29, 4.87） |
| Austria | 25 to 29 | T1D | Both | 5.89 (5.63, 6.16） |
| Austria | 25 to 29 | T1D | Female | 5.75 (5.39, 6.12） |
| Austria | 25 to 29 | T1D | Male | 6.02 (5.65, 6.42） |
| Austria | 30 to 34 | T1D | Both | 7.45 (7.12, 7.80） |
| Austria | 30 to 34 | T1D | Female | 7.22 (6.77, 7.70） |
| Austria | 30 to 34 | T1D | Male | 7.67 (7.19, 8.19） |
| Austria | 35 to 39 | T1D | Both | 9.26 (8.84, 9.71） |
| Austria | 35 to 39 | T1D | Female | 8.94 (8.37, 9.56） |
| Austria | 35 to 39 | T1D | Male | 9.56 (8.95, 10.22） |
| Austria | 40 to 44 | T1D | Both | 11.38 (10.85, 11.93） |
| Austria | 40 to 44 | T1D | Female | 10.98 (10.26, 11.74） |
| Austria | 40 to 44 | T1D | Male | 11.76 (11.00, 12.58） |
| Austria | 45 to 49 | T1D | Both | 13.82 (13.18, 14.50） |
| Austria | 45 to 49 | T1D | Female | 13.34 (12.46, 14.27） |
| Austria | 45 to 49 | T1D | Male | 14.29 (13.35, 15.29） |
| Austria | 5 to 9 | T1D | Both | 1.00 (1.00, 1.00） |
| Austria | 5 to 9 | T1D | Female | 1.00 (1.00, 1.00） |
| Austria | 5 to 9 | T1D | Male | 1.00 (1.00, 1.00） |
| Austria | 50 to 54 | T1D | Both | 16.63 (15.84, 17.46） |
| Austria | 50 to 54 | T1D | Female | 16.07 (15.00, 17.22） |
| Austria | 50 to 54 | T1D | Male | 17.16 (16.02, 18.39） |
| Austria | 55 to 59 | T1D | Both | 19.85 (18.89, 20.87） |
| Austria | 55 to 59 | T1D | Female | 19.26 (17.95, 20.67） |
| Austria | 55 to 59 | T1D | Male | 20.42 (19.03, 21.91） |
| Austria | 60 to 64 | T1D | Both | 23.59 (22.42, 24.82） |
| Austria | 60 to 64 | T1D | Female | 23.00 (21.40, 24.71） |
| Austria | 60 to 64 | T1D | Male | 24.14 (22.47, 25.94） |
| Austria | 65 to 69 | T1D | Both | 27.95 (26.53, 29.44） |
| Austria | 65 to 69 | T1D | Female | 27.39 (25.46, 29.48） |
| Austria | 65 to 69 | T1D | Male | 28.43 (26.42, 30.60） |
| Azerbaijan | 0 to 4 | T1D | Both | 0.16 (0.15, 0.18） |

| | | | | |
|---|---|---|---|---|
| Azerbaijan | 0 to 4 | T1D | Female | 0.16 (0.14, 0.19） |
| Azerbaijan | 0 to 4 | T1D | Male | 0.16 (0.14, 0.19） |
| Azerbaijan | 10 to 14 | T1D | Both | 2.22 (2.12, 2.33） |
| Azerbaijan | 10 to 14 | T1D | Female | 2.23 (2.08, 2.38） |
| Azerbaijan | 10 to 14 | T1D | Male | 2.22 (2.08, 2.36） |
| Azerbaijan | 15 to 19 | T1D | Both | 3.42 (3.26, 3.58） |
| Azerbaijan | 15 to 19 | T1D | Female | 3.37 (3.16, 3.61） |
| Azerbaijan | 15 to 19 | T1D | Male | 3.45 (3.23, 3.68） |
| Azerbaijan | 20 to 24 | T1D | Both | 4.47 (4.26, 4.68） |
| Azerbaijan | 20 to 24 | T1D | Female | 4.33 (4.05, 4.64） |
| Azerbaijan | 20 to 24 | T1D | Male | 4.60 (4.31, 4.90） |
| Azerbaijan | 25 to 29 | T1D | Both | 5.59 (5.33, 5.86） |
| Azerbaijan | 25 to 29 | T1D | Female | 5.31 (4.96, 5.69） |
| Azerbaijan | 25 to 29 | T1D | Male | 5.87 (5.50, 6.26） |
| Azerbaijan | 30 to 34 | T1D | Both | 6.56 (6.25, 6.88） |
| Azerbaijan | 30 to 34 | T1D | Female | 6.14 (5.72, 6.59） |
| Azerbaijan | 30 to 34 | T1D | Male | 6.96 (6.51, 7.44） |
| Azerbaijan | 35 to 39 | T1D | Both | 7.42 (7.06, 7.80） |
| Azerbaijan | 35 to 39 | T1D | Female | 6.89 (6.41, 7.41） |
| Azerbaijan | 35 to 39 | T1D | Male | 7.94 (7.41, 8.51） |
| Azerbaijan | 40 to 44 | T1D | Both | 8.25 (7.84, 8.68） |
| Azerbaijan | 40 to 44 | T1D | Female | 7.61 (7.07, 8.19） |
| Azerbaijan | 40 to 44 | T1D | Male | 8.87 (8.27, 9.52） |
| Azerbaijan | 45 to 49 | T1D | Both | 9.07 (8.61, 9.55） |
| Azerbaijan | 45 to 49 | T1D | Female | 8.34 (7.73, 8.99） |
| Azerbaijan | 45 to 49 | T1D | Male | 9.78 (9.10, 10.51） |
| Azerbaijan | 5 to 9 | T1D | Both | 1.00 (1.00, 1.00） |
| Azerbaijan | 5 to 9 | T1D | Female | 1.00 (1.00, 1.00） |
| Azerbaijan | 5 to 9 | T1D | Male | 1.00 (1.00, 1.00） |
| Azerbaijan | 50 to 54 | T1D | Both | 9.88 (9.36, 10.43） |
| Azerbaijan | 50 to 54 | T1D | Female | 9.09 (8.41, 9.83） |
| Azerbaijan | 50 to 54 | T1D | Male | 10.66 (9.90, 11.49） |
| Azerbaijan | 55 to 59 | T1D | Both | 10.66 (10.07, 11.27） |

| | | | | |
|---|---|---|---|---|
| Azerbaijan | 55 to 59 | T1D | Female | 9.84 (9.07, 10.67） |
| Azerbaijan | 55 to 59 | T1D | Male | 11.50 (10.64, 12.43） |
| Azerbaijan | 60 to 64 | T1D | Both | 11.40 (10.75, 12.10） |
| Azerbaijan | 60 to 64 | T1D | Female | 10.58 (9.72, 11.52） |
| Azerbaijan | 60 to 64 | T1D | Male | 12.29 (11.32, 13.34） |
| Azerbaijan | 65 to 69 | T1D | Both | 12.17 (11.42, 12.97） |
| Azerbaijan | 65 to 69 | T1D | Female | 11.39 (10.40, 12.47） |
| Azerbaijan | 65 to 69 | T1D | Male | 13.08 (11.97, 14.29） |
| Bahamas | 0 to 4 | T1D | Both | 0.15 (0.08, 0.29） |
| Bahamas | 0 to 4 | T1D | Female | 0.16 (0.06, 0.40） |
| Bahamas | 0 to 4 | T1D | Male | 0.16 (0.06, 0.40） |
| Bahamas | 10 to 14 | T1D | Both | 2.16 (1.63, 2.85） |
| Bahamas | 10 to 14 | T1D | Female | 2.36 (1.58, 3.50） |
| Bahamas | 10 to 14 | T1D | Male | 2.09 (1.40, 3.14） |
| Bahamas | 15 to 19 | T1D | Both | 3.28 (2.48, 4.35） |
| Bahamas | 15 to 19 | T1D | Female | 3.56 (2.39, 5.32） |
| Bahamas | 15 to 19 | T1D | Male | 3.24 (2.15, 4.86） |
| Bahamas | 20 to 24 | T1D | Both | 4.27 (3.20, 5.68） |
| Bahamas | 20 to 24 | T1D | Female | 4.41 (2.93, 6.63） |
| Bahamas | 20 to 24 | T1D | Male | 4.37 (2.89, 6.59） |
| Bahamas | 25 to 29 | T1D | Both | 5.33 (3.98, 7.13） |
| Bahamas | 25 to 29 | T1D | Female | 5.32 (3.51, 8.07） |
| Bahamas | 25 to 29 | T1D | Male | 5.78 (3.80, 8.80） |
| Bahamas | 30 to 34 | T1D | Both | 6.28 (4.67, 8.46） |
| Bahamas | 30 to 34 | T1D | Female | 6.15 (4.03, 9.41） |
| Bahamas | 30 to 34 | T1D | Male | 7.07 (4.60, 10.84） |
| Bahamas | 35 to 39 | T1D | Both | 7.28 (5.36, 9.87） |
| Bahamas | 35 to 39 | T1D | Female | 6.96 (4.51, 10.75） |
| Bahamas | 35 to 39 | T1D | Male | 8.34 (5.38, 12.92） |
| Bahamas | 40 to 44 | T1D | Both | 8.32 (6.10, 11.36） |
| Bahamas | 40 to 44 | T1D | Female | 7.92 (5.08, 12.35） |
| Bahamas | 40 to 44 | T1D | Male | 9.67 (6.19, 15.11） |
| Bahamas | 45 to 49 | T1D | Both | 9.40 (6.83, 12.94） |

| | | | | |
|---|---|---|---|---|
| Bahamas | 45 to 49 | T1D | Female | 8.83 (5.60, 13.94） |
| Bahamas | 45 to 49 | T1D | Male | 11.11 (7.03, 17.56） |
| Bahamas | 5 to 9 | T1D | Both | 1.00 (1.00, 1.00） |
| Bahamas | 5 to 9 | T1D | Female | 1.00 (1.00, 1.00） |
| Bahamas | 5 to 9 | T1D | Male | 1.00 (1.00, 1.00） |
| Bahamas | 50 to 54 | T1D | Both | 10.52 (7.56, 14.63） |
| Bahamas | 50 to 54 | T1D | Female | 9.73 (6.07, 15.60） |
| Bahamas | 50 to 54 | T1D | Male | 12.40 (7.73, 19.89） |
| Bahamas | 55 to 59 | T1D | Both | 11.65 (8.26, 16.42） |
| Bahamas | 55 to 59 | T1D | Female | 10.75 (6.59, 17.54） |
| Bahamas | 55 to 59 | T1D | Male | 13.98 (8.56, 22.83） |
| Bahamas | 60 to 64 | T1D | Both | 12.83 (8.95, 18.39） |
| Bahamas | 60 to 64 | T1D | Female | 11.84 (7.08, 19.78） |
| Bahamas | 60 to 64 | T1D | Male | 15.54 (9.30, 25.98） |
| Bahamas | 65 to 69 | T1D | Both | 14.10 (9.61, 20.68） |
| Bahamas | 65 to 69 | T1D | Female | 13.13 (7.62, 22.62） |
| Bahamas | 65 to 69 | T1D | Male | 17.11 (9.91, 29.55） |
| Bahrain | 0 to 4 | T1D | Both | 0.16 (0.11, 0.22） |
| Bahrain | 0 to 4 | T1D | Female | 0.16 (0.10, 0.27） |
| Bahrain | 0 to 4 | T1D | Male | 0.15 (0.09, 0.24） |
| Bahrain | 10 to 14 | T1D | Both | 2.30 (1.97, 2.67） |
| Bahrain | 10 to 14 | T1D | Female | 2.41 (1.93, 3.02） |
| Bahrain | 10 to 14 | T1D | Male | 2.24 (1.82, 2.75） |
| Bahrain | 15 to 19 | T1D | Both | 3.59 (3.08, 4.18） |
| Bahrain | 15 to 19 | T1D | Female | 3.84 (3.06, 4.81） |
| Bahrain | 15 to 19 | T1D | Male | 3.47 (2.82, 4.27） |
| Bahrain | 20 to 24 | T1D | Both | 4.82 (4.13, 5.61） |
| Bahrain | 20 to 24 | T1D | Female | 5.07 (4.03, 6.38） |
| Bahrain | 20 to 24 | T1D | Male | 4.67 (3.80, 5.74） |
| Bahrain | 25 to 29 | T1D | Both | 6.23 (5.34, 7.26） |
| Bahrain | 25 to 29 | T1D | Female | 6.46 (5.11, 8.15） |
| Bahrain | 25 to 29 | T1D | Male | 6.07 (4.93, 7.47） |
| Bahrain | 30 to 34 | T1D | Both | 7.67 (6.56, 8.97） |

| | | | | |
|---|---|---|---|---|
| Bahrain | 30 to 34 | T1D | Female | 7.82 (6.16, 9.93） |
| Bahrain | 30 to 34 | T1D | Male | 7.50 (6.07, 9.25） |
| Bahrain | 35 to 39 | T1D | Both | 9.21 (7.84, 10.82） |
| Bahrain | 35 to 39 | T1D | Female | 9.29 (7.27, 11.88） |
| Bahrain | 35 to 39 | T1D | Male | 9.03 (7.28, 11.19） |
| Bahrain | 40 to 44 | T1D | Both | 10.86 (9.21, 12.81） |
| Bahrain | 40 to 44 | T1D | Female | 10.86 (8.43, 13.99） |
| Bahrain | 40 to 44 | T1D | Male | 10.65 (8.55, 13.28） |
| Bahrain | 45 to 49 | T1D | Both | 12.62 (10.65, 14.96） |
| Bahrain | 45 to 49 | T1D | Female | 12.58 (9.68, 16.35） |
| Bahrain | 45 to 49 | T1D | Male | 12.42 (9.91, 15.57） |
| Bahrain | 5 to 9 | T1D | Both | 1.00 (1.00, 1.00） |
| Bahrain | 5 to 9 | T1D | Female | 1.00 (1.00, 1.00） |
| Bahrain | 5 to 9 | T1D | Male | 1.00 (1.00, 1.00） |
| Bahrain | 50 to 54 | T1D | Both | 14.51 (12.17, 17.29） |
| Bahrain | 50 to 54 | T1D | Female | 14.42 (10.97, 18.96） |
| Bahrain | 50 to 54 | T1D | Male | 14.27 (11.31, 18.00） |
| Bahrain | 55 to 59 | T1D | Both | 16.57 (13.79, 19.92） |
| Bahrain | 55 to 59 | T1D | Female | 16.51 (12.36, 22.06） |
| Bahrain | 55 to 59 | T1D | Male | 16.33 (12.82, 20.81） |
| Bahrain | 60 to 64 | T1D | Both | 18.79 (15.42, 22.90） |
| Bahrain | 60 to 64 | T1D | Female | 18.82 (13.76, 25.73） |
| Bahrain | 60 to 64 | T1D | Male | 18.50 (14.28, 23.96） |
| Bahrain | 65 to 69 | T1D | Both | 21.19 (17.00, 26.41） |
| Bahrain | 65 to 69 | T1D | Female | 21.53 (15.24, 30.41） |
| Bahrain | 65 to 69 | T1D | Male | 20.98 (15.72, 28.01） |
| Bangladesh | 0 to 4 | T1D | Both | 0.16 (0.15, 0.16） |
| Bangladesh | 0 to 4 | T1D | Female | 0.15 (0.15, 0.16） |
| Bangladesh | 0 to 4 | T1D | Male | 0.16 (0.15, 0.16） |
| Bangladesh | 10 to 14 | T1D | Both | 2.16 (2.14, 2.18） |
| Bangladesh | 10 to 14 | T1D | Female | 2.10 (2.07, 2.13） |
| Bangladesh | 10 to 14 | T1D | Male | 2.23 (2.19, 2.26） |
| Bangladesh | 15 to 19 | T1D | Both | 3.05 (3.02, 3.08） |

| | | | | |
|---|---|---|---|---|
| Bangladesh | 15 to 19 | T1D | Female | 2.85 (2.80, 2.89） |
| Bangladesh | 15 to 19 | T1D | Male | 3.26 (3.21, 3.31） |
| Bangladesh | 20 to 24 | T1D | Both | 3.66 (3.62, 3.70） |
| Bangladesh | 20 to 24 | T1D | Female | 3.34 (3.29, 3.39） |
| Bangladesh | 20 to 24 | T1D | Male | 4.02 (3.96, 4.08） |
| Bangladesh | 25 to 29 | T1D | Both | 4.27 (4.22, 4.31） |
| Bangladesh | 25 to 29 | T1D | Female | 3.83 (3.77, 3.89） |
| Bangladesh | 25 to 29 | T1D | Male | 4.76 (4.69, 4.84） |
| Bangladesh | 30 to 34 | T1D | Both | 4.74 (4.69, 4.80） |
| Bangladesh | 30 to 34 | T1D | Female | 4.21 (4.14, 4.28） |
| Bangladesh | 30 to 34 | T1D | Male | 5.33 (5.25, 5.42） |
| Bangladesh | 35 to 39 | T1D | Both | 5.16 (5.10, 5.22） |
| Bangladesh | 35 to 39 | T1D | Female | 4.54 (4.47, 4.62） |
| Bangladesh | 35 to 39 | T1D | Male | 5.83 (5.73, 5.93） |
| Bangladesh | 40 to 44 | T1D | Both | 5.55 (5.48, 5.62） |
| Bangladesh | 40 to 44 | T1D | Female | 4.87 (4.78, 4.96） |
| Bangladesh | 40 to 44 | T1D | Male | 6.28 (6.17, 6.40） |
| Bangladesh | 45 to 49 | T1D | Both | 5.90 (5.82, 5.97） |
| Bangladesh | 45 to 49 | T1D | Female | 5.17 (5.07, 5.27） |
| Bangladesh | 45 to 49 | T1D | Male | 6.70 (6.58, 6.82） |
| Bangladesh | 5 to 9 | T1D | Both | 1.00 (1.00, 1.00） |
| Bangladesh | 5 to 9 | T1D | Female | 1.00 (1.00, 1.00） |
| Bangladesh | 5 to 9 | T1D | Male | 1.00 (1.00, 1.00） |
| Bangladesh | 50 to 54 | T1D | Both | 6.22 (6.14, 6.31） |
| Bangladesh | 50 to 54 | T1D | Female | 5.46 (5.35, 5.57） |
| Bangladesh | 50 to 54 | T1D | Male | 7.08 (6.94, 7.22） |
| Bangladesh | 55 to 59 | T1D | Both | 6.56 (6.46, 6.66） |
| Bangladesh | 55 to 59 | T1D | Female | 5.76 (5.64, 5.89） |
| Bangladesh | 55 to 59 | T1D | Male | 7.46 (7.31, 7.61） |
| Bangladesh | 60 to 64 | T1D | Both | 6.91 (6.80, 7.02） |
| Bangladesh | 60 to 64 | T1D | Female | 6.09 (5.95, 6.24） |
| Bangladesh | 60 to 64 | T1D | Male | 7.84 (7.67, 8.02） |
| Bangladesh | 65 to 69 | T1D | Both | 7.26 (7.14, 7.39） |

| | | | | |
|---|---|---|---|---|
| Bangladesh | 65 to 69 | T1D | Female | 6.43 (6.27, 6.60） |
| Bangladesh | 65 to 69 | T1D | Male | 8.22 (8.03, 8.42） |
| Barbados | 0 to 4 | T1D | Both | 0.14 (0.06, 0.35） |
| Barbados | 0 to 4 | T1D | Female | 0.03 (0.01, 0.47） |
| Barbados | 0 to 4 | T1D | Male | 0.23 (0.07, 0.77） |
| Barbados | 10 to 14 | T1D | Both | 2.36 (1.62, 3.45） |
| Barbados | 10 to 14 | T1D | Female | 2.73 (1.58, 4.72） |
| Barbados | 10 to 14 | T1D | Male | 2.18 (1.25, 3.81） |
| Barbados | 15 to 19 | T1D | Both | 3.35 (2.29, 4.90） |
| Barbados | 15 to 19 | T1D | Female | 3.93 (2.27, 6.80） |
| Barbados | 15 to 19 | T1D | Male | 3.43 (1.97, 5.98） |
| Barbados | 20 to 24 | T1D | Both | 4.12 (2.81, 6.06） |
| Barbados | 20 to 24 | T1D | Female | 4.56 (2.60, 7.98） |
| Barbados | 20 to 24 | T1D | Male | 4.42 (2.51, 7.79） |
| Barbados | 25 to 29 | T1D | Both | 4.90 (3.31, 7.23） |
| Barbados | 25 to 29 | T1D | Female | 5.22 (2.95, 9.21） |
| Barbados | 25 to 29 | T1D | Male | 5.46 (3.08, 9.68） |
| Barbados | 30 to 34 | T1D | Both | 5.61 (3.77, 8.35） |
| Barbados | 30 to 34 | T1D | Female | 5.71 (3.20, 10.17） |
| Barbados | 30 to 34 | T1D | Male | 6.53 (3.65, 11.69） |
| Barbados | 35 to 39 | T1D | Both | 6.32 (4.21, 9.47） |
| Barbados | 35 to 39 | T1D | Female | 6.22 (3.45, 11.20） |
| Barbados | 35 to 39 | T1D | Male | 7.47 (4.13, 13.52） |
| Barbados | 40 to 44 | T1D | Both | 6.99 (4.62, 10.56） |
| Barbados | 40 to 44 | T1D | Female | 6.73 (3.70, 12.26） |
| Barbados | 40 to 44 | T1D | Male | 8.30 (4.54, 15.18） |
| Barbados | 45 to 49 | T1D | Both | 7.67 (5.04, 11.68） |
| Barbados | 45 to 49 | T1D | Female | 7.31 (3.98, 13.45） |
| Barbados | 45 to 49 | T1D | Male | 9.56 (5.18, 17.65） |
| Barbados | 5 to 9 | T1D | Both | 1.00 (1.00, 1.00） |
| Barbados | 5 to 9 | T1D | Female | 1.00 (1.00, 1.00） |
| Barbados | 5 to 9 | T1D | Male | 1.00 (1.00, 1.00） |
| Barbados | 50 to 54 | T1D | Both | 8.41 (5.47, 12.92） |

| | | | | |
|---|---|---|---|---|
| Barbados | 50 to 54 | T1D | Female | 7.86 (4.22, 14.65） |
| Barbados | 50 to 54 | T1D | Male | 10.53 (5.63, 19.69） |
| Barbados | 55 to 59 | T1D | Both | 9.10 (5.85, 14.15） |
| Barbados | 55 to 59 | T1D | Female | 8.41 (4.44, 15.93） |
| Barbados | 55 to 59 | T1D | Male | 11.40 (5.99, 21.67） |
| Barbados | 60 to 64 | T1D | Both | 9.89 (6.28, 15.60） |
| Barbados | 60 to 64 | T1D | Female | 9.06 (4.68, 17.51） |
| Barbados | 60 to 64 | T1D | Male | 12.51 (6.45, 24.25） |
| Barbados | 65 to 69 | T1D | Both | 10.57 (6.58, 16.99） |
| Barbados | 65 to 69 | T1D | Female | 10.03 (5.06, 19.86） |
| Barbados | 65 to 69 | T1D | Male | 13.65 (6.85, 27.21） |
| Belarus | 0 to 4 | T1D | Both | 0.18 (0.16, 0.20） |
| Belarus | 0 to 4 | T1D | Female | 0.18 (0.15, 0.22） |
| Belarus | 0 to 4 | T1D | Male | 0.18 (0.15, 0.22） |
| Belarus | 10 to 14 | T1D | Both | 2.35 (2.22, 2.49） |
| Belarus | 10 to 14 | T1D | Female | 2.36 (2.17, 2.56） |
| Belarus | 10 to 14 | T1D | Male | 2.34 (2.16, 2.55） |
| Belarus | 15 to 19 | T1D | Both | 3.72 (3.51, 3.94） |
| Belarus | 15 to 19 | T1D | Female | 3.68 (3.39, 3.99） |
| Belarus | 15 to 19 | T1D | Male | 3.76 (3.47, 4.08） |
| Belarus | 20 to 24 | T1D | Both | 4.89 (4.62, 5.18） |
| Belarus | 20 to 24 | T1D | Female | 4.73 (4.36, 5.13） |
| Belarus | 20 to 24 | T1D | Male | 5.04 (4.65, 5.47） |
| Belarus | 25 to 29 | T1D | Both | 6.18 (5.83, 6.54） |
| Belarus | 25 to 29 | T1D | Female | 5.84 (5.39, 6.34） |
| Belarus | 25 to 29 | T1D | Male | 6.51 (6.01, 7.06） |
| Belarus | 30 to 34 | T1D | Both | 7.40 (6.98, 7.85） |
| Belarus | 30 to 34 | T1D | Female | 6.90 (6.36, 7.50） |
| Belarus | 30 to 34 | T1D | Male | 7.92 (7.30, 8.60） |
| Belarus | 35 to 39 | T1D | Both | 8.65 (8.15, 9.18） |
| Belarus | 35 to 39 | T1D | Female | 8.00 (7.35, 8.70） |
| Belarus | 35 to 39 | T1D | Male | 9.34 (8.59, 10.16） |
| Belarus | 40 to 44 | T1D | Both | 9.96 (9.37, 10.57） |

| | | | | |
|---|---|---|---|---|
| Belarus | 40 to 44 | T1D | Female | 9.16 (8.42, 9.98） |
| Belarus | 40 to 44 | T1D | Male | 10.81 (9.93, 11.77） |
| Belarus | 45 to 49 | T1D | Both | 11.33 (10.66, 12.04） |
| Belarus | 45 to 49 | T1D | Female | 10.42 (9.56, 11.36） |
| Belarus | 45 to 49 | T1D | Male | 12.32 (11.31, 13.43） |
| Belarus | 5 to 9 | T1D | Both | 1.00 (1.00, 1.00） |
| Belarus | 5 to 9 | T1D | Female | 1.00 (1.00, 1.00） |
| Belarus | 5 to 9 | T1D | Male | 1.00 (1.00, 1.00） |
| Belarus | 50 to 54 | T1D | Both | 12.77 (12.00, 13.58） |
| Belarus | 50 to 54 | T1D | Female | 11.78 (10.80, 12.86） |
| Belarus | 50 to 54 | T1D | Male | 13.87 (12.71, 15.14） |
| Belarus | 55 to 59 | T1D | Both | 14.30 (13.43, 15.22） |
| Belarus | 55 to 59 | T1D | Female | 13.29 (12.17, 14.52） |
| Belarus | 55 to 59 | T1D | Male | 15.48 (14.16, 16.91） |
| Belarus | 60 to 64 | T1D | Both | 15.94 (14.96, 16.99） |
| Belarus | 60 to 64 | T1D | Female | 14.94 (13.66, 16.34） |
| Belarus | 60 to 64 | T1D | Male | 17.14 (15.65, 18.77） |
| Belarus | 65 to 69 | T1D | Both | 17.70 (16.58, 18.90） |
| Belarus | 65 to 69 | T1D | Female | 16.75 (15.28, 18.36） |
| Belarus | 65 to 69 | T1D | Male | 18.88 (17.19, 20.74） |
| Belgium | 0 to 4 | T1D | Both | 0.18 (0.17, 0.19） |
| Belgium | 0 to 4 | T1D | Female | 0.17 (0.15, 0.19） |
| Belgium | 0 to 4 | T1D | Male | 0.19 (0.17, 0.21） |
| Belgium | 10 to 14 | T1D | Both | 2.15 (2.07, 2.23） |
| Belgium | 10 to 14 | T1D | Female | 2.13 (2.02, 2.24） |
| Belgium | 10 to 14 | T1D | Male | 2.17 (2.06, 2.29） |
| Belgium | 15 to 19 | T1D | Both | 3.26 (3.15, 3.39） |
| Belgium | 15 to 19 | T1D | Female | 3.16 (3.00, 3.33） |
| Belgium | 15 to 19 | T1D | Male | 3.36 (3.19, 3.54） |
| Belgium | 20 to 24 | T1D | Both | 4.28 (4.13, 4.45） |
| Belgium | 20 to 24 | T1D | Female | 4.07 (3.86, 4.30） |
| Belgium | 20 to 24 | T1D | Male | 4.50 (4.27, 4.74） |
| Belgium | 25 to 29 | T1D | Both | 5.43 (5.23, 5.64） |

| | | | | |
|---|---|---|---|---|
| Belgium | 25 to 29 | T1D | Female | 5.06 (4.79, 5.34） |
| Belgium | 25 to 29 | T1D | Male | 5.80 (5.50, 6.12） |
| Belgium | 30 to 34 | T1D | Both | 6.56 (6.31, 6.82） |
| Belgium | 30 to 34 | T1D | Female | 6.01 (5.69, 6.35） |
| Belgium | 30 to 34 | T1D | Male | 7.11 (6.74, 7.51） |
| Belgium | 35 to 39 | T1D | Both | 7.77 (7.47, 8.08） |
| Belgium | 35 to 39 | T1D | Female | 7.02 (6.64, 7.43） |
| Belgium | 35 to 39 | T1D | Male | 8.53 (8.07, 9.01） |
| Belgium | 40 to 44 | T1D | Both | 9.10 (8.74, 9.47） |
| Belgium | 40 to 44 | T1D | Female | 8.13 (7.68, 8.61） |
| Belgium | 40 to 44 | T1D | Male | 10.08 (9.54, 10.66） |
| Belgium | 45 to 49 | T1D | Both | 10.53 (10.11, 10.97） |
| Belgium | 45 to 49 | T1D | Female | 9.32 (8.80, 9.88） |
| Belgium | 45 to 49 | T1D | Male | 11.77 (11.13, 12.45） |
| Belgium | 5 to 9 | T1D | Both | 1.00 (1.00, 1.00） |
| Belgium | 5 to 9 | T1D | Female | 1.00 (1.00, 1.00） |
| Belgium | 5 to 9 | T1D | Male | 1.00 (1.00, 1.00） |
| Belgium | 50 to 54 | T1D | Both | 12.09 (11.60, 12.59） |
| Belgium | 50 to 54 | T1D | Female | 10.62 (10.01, 11.27） |
| Belgium | 50 to 54 | T1D | Male | 13.59 (12.84, 14.39） |
| Belgium | 55 to 59 | T1D | Both | 13.75 (13.19, 14.34） |
| Belgium | 55 to 59 | T1D | Female | 12.03 (11.32, 12.77） |
| Belgium | 55 to 59 | T1D | Male | 15.56 (14.68, 16.49） |
| Belgium | 60 to 64 | T1D | Both | 15.56 (14.91, 16.24） |
| Belgium | 60 to 64 | T1D | Female | 13.56 (12.75, 14.42） |
| Belgium | 60 to 64 | T1D | Male | 17.70 (16.68, 18.79） |
| Belgium | 65 to 69 | T1D | Both | 17.55 (16.80, 18.33） |
| Belgium | 65 to 69 | T1D | Female | 15.27 (14.35, 16.26） |
| Belgium | 65 to 69 | T1D | Male | 20.07 (18.88, 21.32） |
| Belize | 0 to 4 | T1D | Both | 0.16 (0.09, 0.30） |
| Belize | 0 to 4 | T1D | Female | 0.14 (0.06, 0.36） |
| Belize | 0 to 4 | T1D | Male | 0.14 (0.06, 0.36） |
| Belize | 10 to 14 | T1D | Both | 2.26 (1.71, 2.97） |

| | | | | |
|---|---|---|---|---|
| Belize | 10 to 14 | T1D | Female | 2.41 (1.64, 3.53） |
| Belize | 10 to 14 | T1D | Male | 2.04 (1.38, 3.02） |
| Belize | 15 to 19 | T1D | Both | 3.37 (2.55, 4.45） |
| Belize | 15 to 19 | T1D | Female | 3.51 (2.38, 5.18） |
| Belize | 15 to 19 | T1D | Male | 3.07 (2.07, 4.58） |
| Belize | 20 to 24 | T1D | Both | 4.27 (3.21, 5.70） |
| Belize | 20 to 24 | T1D | Female | 4.22 (2.82, 6.30） |
| Belize | 20 to 24 | T1D | Male | 4.12 (2.74, 6.19） |
| Belize | 25 to 29 | T1D | Both | 5.18 (3.85, 6.98） |
| Belize | 25 to 29 | T1D | Female | 4.89 (3.23, 7.42） |
| Belize | 25 to 29 | T1D | Male | 5.21 (3.42, 7.95） |
| Belize | 30 to 34 | T1D | Both | 5.94 (4.36, 8.10） |
| Belize | 30 to 34 | T1D | Female | 5.48 (3.55, 8.45） |
| Belize | 30 to 34 | T1D | Male | 6.20 (4.00, 9.61） |
| Belize | 35 to 39 | T1D | Both | 6.72 (4.87, 9.27） |
| Belize | 35 to 39 | T1D | Female | 6.03 (3.84, 9.49） |
| Belize | 35 to 39 | T1D | Male | 7.03 (4.45, 11.10） |
| Belize | 40 to 44 | T1D | Both | 7.40 (5.29, 10.36） |
| Belize | 40 to 44 | T1D | Female | 6.57 (4.09, 10.56） |
| Belize | 40 to 44 | T1D | Male | 8.08 (5.02, 12.99） |
| Belize | 45 to 49 | T1D | Both | 8.14 (5.72, 11.59） |
| Belize | 45 to 49 | T1D | Female | 7.25 (4.40, 11.94） |
| Belize | 45 to 49 | T1D | Male | 8.83 (5.37, 14.51） |
| Belize | 5 to 9 | T1D | Both | 1.00 (1.00, 1.00） |
| Belize | 5 to 9 | T1D | Female | 1.00 (1.00, 1.00） |
| Belize | 5 to 9 | T1D | Male | 1.00 (1.00, 1.00） |
| Belize | 50 to 54 | T1D | Both | 8.94 (6.16, 12.97） |
| Belize | 50 to 54 | T1D | Female | 7.84 (4.61, 13.33） |
| Belize | 50 to 54 | T1D | Male | 9.60 (5.69, 16.20） |
| Belize | 55 to 59 | T1D | Both | 9.66 (6.49, 14.37） |
| Belize | 55 to 59 | T1D | Female | 8.40 (4.75, 14.87） |
| Belize | 55 to 59 | T1D | Male | 10.46 (6.01, 18.23） |
| Belize | 60 to 64 | T1D | Both | 10.40 (6.77, 15.95） |

| | | | | |
|---|---|---|---|---|
| Belize | 60 to 64 | T1D | Female | 9.18 (4.95, 17.03） |
| Belize | 60 to 64 | T1D | Male | 11.16 (6.15, 20.25） |
| Belize | 65 to 69 | T1D | Both | 11.06 (6.92, 17.66） |
| Belize | 65 to 69 | T1D | Female | 9.66 (4.88, 19.11） |
| Belize | 65 to 69 | T1D | Male | 12.33 (6.48, 23.44） |
| Benin | 0 to 4 | T1D | Both | 0.15 (0.14, 0.17） |
| Benin | 0 to 4 | T1D | Female | 0.15 (0.13, 0.17） |
| Benin | 0 to 4 | T1D | Male | 0.15 (0.13, 0.17） |
| Benin | 10 to 14 | T1D | Both | 2.10 (2.01, 2.19） |
| Benin | 10 to 14 | T1D | Female | 2.09 (1.96, 2.23） |
| Benin | 10 to 14 | T1D | Male | 2.10 (1.98, 2.23） |
| Benin | 15 to 19 | T1D | Both | 3.04 (2.90, 3.17） |
| Benin | 15 to 19 | T1D | Female | 3.04 (2.85, 3.24） |
| Benin | 15 to 19 | T1D | Male | 3.04 (2.86, 3.24） |
| Benin | 20 to 24 | T1D | Both | 3.84 (3.67, 4.02） |
| Benin | 20 to 24 | T1D | Female | 3.86 (3.61, 4.13） |
| Benin | 20 to 24 | T1D | Male | 3.84 (3.60, 4.10） |
| Benin | 25 to 29 | T1D | Both | 4.67 (4.45, 4.90） |
| Benin | 25 to 29 | T1D | Female | 4.70 (4.39, 5.03） |
| Benin | 25 to 29 | T1D | Male | 4.66 (4.35, 4.98） |
| Benin | 30 to 34 | T1D | Both | 5.18 (4.93, 5.45） |
| Benin | 30 to 34 | T1D | Female | 5.23 (4.87, 5.62） |
| Benin | 30 to 34 | T1D | Male | 5.16 (4.81, 5.54） |
| Benin | 35 to 39 | T1D | Both | 5.55 (5.26, 5.86） |
| Benin | 35 to 39 | T1D | Female | 5.60 (5.20, 6.05） |
| Benin | 35 to 39 | T1D | Male | 5.51 (5.11, 5.94） |
| Benin | 40 to 44 | T1D | Both | 5.85 (5.53, 6.19） |
| Benin | 40 to 44 | T1D | Female | 5.91 (5.45, 6.40） |
| Benin | 40 to 44 | T1D | Male | 5.78 (5.34, 6.26） |
| Benin | 45 to 49 | T1D | Both | 6.08 (5.73, 6.46） |
| Benin | 45 to 49 | T1D | Female | 6.16 (5.66, 6.71） |
| Benin | 45 to 49 | T1D | Male | 6.01 (5.52, 6.54） |
| Benin | 5 to 9 | T1D | Both | 1.00 (1.00, 1.00） |

| | | | | |
|---|---|---|---|---|
| Benin | 5 to 9 | T1D | Female | 1.00 (1.00, 1.00） |
| Benin | 5 to 9 | T1D | Male | 1.00 (1.00, 1.00） |
| Benin | 50 to 54 | T1D | Both | 6.27 (5.87, 6.69） |
| Benin | 50 to 54 | T1D | Female | 6.36 (5.80, 6.97） |
| Benin | 50 to 54 | T1D | Male | 6.17 (5.63, 6.77） |
| Benin | 55 to 59 | T1D | Both | 6.40 (5.96, 6.86） |
| Benin | 55 to 59 | T1D | Female | 6.50 (5.88, 7.17） |
| Benin | 55 to 59 | T1D | Male | 6.29 (5.69, 6.94） |
| Benin | 60 to 64 | T1D | Both | 6.47 (6.00, 6.99） |
| Benin | 60 to 64 | T1D | Female | 6.60 (5.92, 7.35） |
| Benin | 60 to 64 | T1D | Male | 6.36 (5.70, 7.09） |
| Benin | 65 to 69 | T1D | Both | 6.52 (5.99, 7.10） |
| Benin | 65 to 69 | T1D | Female | 6.65 (5.91, 7.50） |
| Benin | 65 to 69 | T1D | Male | 6.39 (5.66, 7.23） |
| Bermuda | 0 to 4 | T1D | Both | 0.05 (0.01, 0.79） |
| Bermuda | 0 to 4 | T1D | Female | 0.09 (0.01, 1.68） |
| Bermuda | 0 to 4 | T1D | Male | 0.09 (0.01, 1.63） |
| Bermuda | 10 to 14 | T1D | Both | 2.01 (0.95, 4.25） |
| Bermuda | 10 to 14 | T1D | Female | 2.17 (0.76, 6.16） |
| Bermuda | 10 to 14 | T1D | Male | 1.88 (0.65, 5.49） |
| Bermuda | 15 to 19 | T1D | Both | 2.83 (1.34, 5.99） |
| Bermuda | 15 to 19 | T1D | Female | 3.15 (1.12, 8.88） |
| Bermuda | 15 to 19 | T1D | Male | 2.53 (0.85, 7.47） |
| Bermuda | 20 to 24 | T1D | Both | 3.70 (1.74, 7.89） |
| Bermuda | 20 to 24 | T1D | Female | 3.49 (1.21, 10.10） |
| Bermuda | 20 to 24 | T1D | Male | 3.95 (1.35, 11.54） |
| Bermuda | 25 to 29 | T1D | Both | 4.79 (2.25, 10.23） |
| Bermuda | 25 to 29 | T1D | Female | 4.36 (1.50, 12.67） |
| Bermuda | 25 to 29 | T1D | Male | 4.88 (1.65, 14.40） |
| Bermuda | 30 to 34 | T1D | Both | 5.72 (2.66, 12.31） |
| Bermuda | 30 to 34 | T1D | Female | 5.32 (1.82, 15.60） |
| Bermuda | 30 to 34 | T1D | Male | 6.65 (2.24, 19.73） |
| Bermuda | 35 to 39 | T1D | Both | 6.89 (3.16, 15.00） |

| | | | | |
|---|---|---|---|---|
| Bermuda | 35 to 39 | T1D | Female | 5.83 (1.94, 17.45） |
| Bermuda | 35 to 39 | T1D | Male | 7.59 (2.51, 22.98） |
| Bermuda | 40 to 44 | T1D | Both | 7.97 (3.63, 17.52） |
| Bermuda | 40 to 44 | T1D | Female | 7.15 (2.36, 21.66） |
| Bermuda | 40 to 44 | T1D | Male | 9.08 (2.96, 27.82） |
| Bermuda | 45 to 49 | T1D | Both | 9.33 (4.21, 20.70） |
| Bermuda | 45 to 49 | T1D | Female | 8.02 (2.61, 24.63） |
| Bermuda | 45 to 49 | T1D | Male | 10.89 (3.51, 33.84） |
| Bermuda | 5 to 9 | T1D | Both | 1.00 (1.00, 1.00） |
| Bermuda | 5 to 9 | T1D | Female | 1.00 (1.00, 1.00） |
| Bermuda | 5 to 9 | T1D | Male | 1.00 (1.00, 1.00） |
| Bermuda | 50 to 54 | T1D | Both | 10.56 (4.71, 23.68） |
| Bermuda | 50 to 54 | T1D | Female | 9.47 (3.04, 29.50） |
| Bermuda | 50 to 54 | T1D | Male | 12.85 (4.07, 40.57） |
| Bermuda | 55 to 59 | T1D | Both | 12.25 (5.39, 27.84） |
| Bermuda | 55 to 59 | T1D | Female | 10.13 (3.18, 32.29） |
| Bermuda | 55 to 59 | T1D | Male | 14.75 (4.58, 47.45） |
| Bermuda | 60 to 64 | T1D | Both | 13.95 (6.04, 32.25） |
| Bermuda | 60 to 64 | T1D | Female | 11.61 (3.56, 37.86） |
| Bermuda | 60 to 64 | T1D | Male | 16.88 (5.12, 55.69） |
| Bermuda | 65 to 69 | T1D | Both | 15.92 (6.74, 37.58） |
| Bermuda | 65 to 69 | T1D | Female | 13.79 (4.11, 46.23） |
| Bermuda | 65 to 69 | T1D | Male | 19.07 (5.60, 64.95） |
| Bhutan | 0 to 4 | T1D | Both | 0.15 (0.10, 0.21） |
| Bhutan | 0 to 4 | T1D | Female | 0.15 (0.09, 0.25） |
| Bhutan | 0 to 4 | T1D | Male | 0.14 (0.08, 0.23） |
| Bhutan | 10 to 14 | T1D | Both | 2.17 (1.85, 2.54） |
| Bhutan | 10 to 14 | T1D | Female | 2.10 (1.68, 2.61） |
| Bhutan | 10 to 14 | T1D | Male | 2.21 (1.76, 2.78） |
| Bhutan | 15 to 19 | T1D | Both | 3.03 (2.59, 3.55） |
| Bhutan | 15 to 19 | T1D | Female | 2.81 (2.26, 3.51） |
| Bhutan | 15 to 19 | T1D | Male | 3.24 (2.59, 4.05） |
| Bhutan | 20 to 24 | T1D | Both | 3.63 (3.10, 4.26） |

| | | | | |
|---|---|---|---|---|
| Bhutan | 20 to 24 | T1D | Female | 3.27 (2.61, 4.09） |
| Bhutan | 20 to 24 | T1D | Male | 3.94 (3.14, 4.94） |
| Bhutan | 25 to 29 | T1D | Both | 4.20 (3.57, 4.95） |
| Bhutan | 25 to 29 | T1D | Female | 3.71 (2.94, 4.68） |
| Bhutan | 25 to 29 | T1D | Male | 4.61 (3.66, 5.81） |
| Bhutan | 30 to 34 | T1D | Both | 4.62 (3.90, 5.48） |
| Bhutan | 30 to 34 | T1D | Female | 4.05 (3.17, 5.16） |
| Bhutan | 30 to 34 | T1D | Male | 5.11 (4.03, 6.49） |
| Bhutan | 35 to 39 | T1D | Both | 4.97 (4.16, 5.93） |
| Bhutan | 35 to 39 | T1D | Female | 4.33 (3.36, 5.59） |
| Bhutan | 35 to 39 | T1D | Male | 5.56 (4.33, 7.12） |
| Bhutan | 40 to 44 | T1D | Both | 5.29 (4.40, 6.38） |
| Bhutan | 40 to 44 | T1D | Female | 4.57 (3.49, 5.98） |
| Bhutan | 40 to 44 | T1D | Male | 5.93 (4.57, 7.68） |
| Bhutan | 45 to 49 | T1D | Both | 5.58 (4.59, 6.78） |
| Bhutan | 45 to 49 | T1D | Female | 4.81 (3.63, 6.39） |
| Bhutan | 45 to 49 | T1D | Male | 6.27 (4.78, 8.22） |
| Bhutan | 5 to 9 | T1D | Both | 1.00 (1.00, 1.00） |
| Bhutan | 5 to 9 | T1D | Female | 1.00 (1.00, 1.00） |
| Bhutan | 5 to 9 | T1D | Male | 1.00 (1.00, 1.00） |
| Bhutan | 50 to 54 | T1D | Both | 5.84 (4.75, 7.17） |
| Bhutan | 50 to 54 | T1D | Female | 5.06 (3.75, 6.82） |
| Bhutan | 50 to 54 | T1D | Male | 6.56 (4.93, 8.71） |
| Bhutan | 55 to 59 | T1D | Both | 6.08 (4.89, 7.56） |
| Bhutan | 55 to 59 | T1D | Female | 5.25 (3.82, 7.21） |
| Bhutan | 55 to 59 | T1D | Male | 6.84 (5.06, 9.24） |
| Bhutan | 60 to 64 | T1D | Both | 6.36 (5.04, 8.03） |
| Bhutan | 60 to 64 | T1D | Female | 5.48 (3.90, 7.70） |
| Bhutan | 60 to 64 | T1D | Male | 7.14 (5.18, 9.83） |
| Bhutan | 65 to 69 | T1D | Both | 6.59 (5.12, 8.47） |
| Bhutan | 65 to 69 | T1D | Female | 5.75 (3.98, 8.31） |
| Bhutan | 65 to 69 | T1D | Male | 7.51 (5.31, 10.61） |
| Bolivia (Plurinational State of) | 0 to 4 | T1D | Both | 0.15 (0.13, 0.16） |

| | | | | |
|---|---|---|---|---|
| Bolivia (Plurinational State of) | 0 to 4 | T1D | Female | 0.14 (0.12, 0.16） |
| Bolivia (Plurinational State of) | 0 to 4 | T1D | Male | 0.16 (0.13, 0.18） |
| Bolivia (Plurinational State of) | 10 to 14 | T1D | Both | 2.08 (1.98, 2.18） |
| Bolivia (Plurinational State of) | 10 to 14 | T1D | Female | 2.14 (2.01, 2.28） |
| Bolivia (Plurinational State of) | 10 to 14 | T1D | Male | 2.01 (1.87, 2.15） |
| Bolivia (Plurinational State of) | 15 to 19 | T1D | Both | 3.01 (2.87, 3.15） |
| Bolivia (Plurinational State of) | 15 to 19 | T1D | Female | 3.08 (2.89, 3.29） |
| Bolivia (Plurinational State of) | 15 to 19 | T1D | Male | 2.90 (2.70, 3.12） |
| Bolivia (Plurinational State of) | 20 to 24 | T1D | Both | 3.75 (3.57, 3.93） |
| Bolivia (Plurinational State of) | 20 to 24 | T1D | Female | 3.77 (3.53, 4.03） |
| Bolivia (Plurinational State of) | 20 to 24 | T1D | Male | 3.70 (3.44, 3.99） |
| Bolivia (Plurinational State of) | 25 to 29 | T1D | Both | 4.46 (4.24, 4.70） |
| Bolivia (Plurinational State of) | 25 to 29 | T1D | Female | 4.39 (4.10, 4.70） |
| Bolivia (Plurinational State of) | 25 to 29 | T1D | Male | 4.54 (4.21, 4.90） |
| Bolivia (Plurinational State of) | 30 to 34 | T1D | Both | 5.05 (4.79, 5.32） |
| Bolivia (Plurinational State of) | 30 to 34 | T1D | Female | 4.89 (4.56, 5.25） |
| Bolivia (Plurinational State of) | 30 to 34 | T1D | Male | 5.23 (4.84, 5.66） |
| Bolivia (Plurinational State of) | 35 to 39 | T1D | Both | 5.57 (5.27, 5.88） |
| Bolivia (Plurinational State of) | 35 to 39 | T1D | Female | 5.35 (4.97, 5.76） |
| Bolivia (Plurinational State of) | 35 to 39 | T1D | Male | 5.85 (5.39, 6.34） |
| Bolivia (Plurinational State of) | 40 to 44 | T1D | Both | 6.07 (5.73, 6.43） |
| Bolivia (Plurinational State of) | 40 to 44 | T1D | Female | 5.79 (5.36, 6.25） |
| Bolivia (Plurinational State of) | 40 to 44 | T1D | Male | 6.42 (5.90, 7.00） |
| Bolivia (Plurinational State of) | 45 to 49 | T1D | Both | 6.55 (6.17, 6.96） |
| Bolivia (Plurinational State of) | 45 to 49 | T1D | Female | 6.23 (5.75, 6.76） |
| Bolivia (Plurinational State of) | 45 to 49 | T1D | Male | 6.97 (6.38, 7.63） |
| Bolivia (Plurinational State of) | 5 to 9 | T1D | Both | 1.00 (1.00, 1.00） |
| Bolivia (Plurinational State of) | 5 to 9 | T1D | Female | 1.00 (1.00, 1.00） |
| Bolivia (Plurinational State of) | 5 to 9 | T1D | Male | 1.00 (1.00, 1.00） |
| Bolivia (Plurinational State of) | 50 to 54 | T1D | Both | 7.04 (6.61, 7.49） |
| Bolivia (Plurinational State of) | 50 to 54 | T1D | Female | 6.68 (6.13, 7.27） |
| Bolivia (Plurinational State of) | 50 to 54 | T1D | Male | 7.49 (6.82, 8.23） |
| Bolivia (Plurinational State of) | 55 to 59 | T1D | Both | 7.50 (7.02, 8.01） |

| | | | | |
|---|---|---|---|---|
| Bolivia (Plurinational State of) | 55 to 59 | T1D | Female | 7.12 (6.51, 7.79） |
| Bolivia (Plurinational State of) | 55 to 59 | T1D | Male | 7.98 (7.23, 8.80） |
| Bolivia (Plurinational State of) | 60 to 64 | T1D | Both | 7.95 (7.41, 8.53） |
| Bolivia (Plurinational State of) | 60 to 64 | T1D | Female | 7.57 (6.89, 8.33） |
| Bolivia (Plurinational State of) | 60 to 64 | T1D | Male | 8.42 (7.58, 9.35） |
| Bolivia (Plurinational State of) | 65 to 69 | T1D | Both | 8.42 (7.81, 9.08） |
| Bolivia (Plurinational State of) | 65 to 69 | T1D | Female | 8.05 (7.27, 8.91） |
| Bolivia (Plurinational State of) | 65 to 69 | T1D | Male | 8.86 (7.92, 9.92） |
| Bosnia and Herzegovina | 0 to 4 | T1D | Both | 0.18 (0.15, 0.22） |
| Bosnia and Herzegovina | 0 to 4 | T1D | Female | 0.17 (0.13, 0.22） |
| Bosnia and Herzegovina | 0 to 4 | T1D | Male | 0.20 (0.16, 0.25） |
| Bosnia and Herzegovina | 10 to 14 | T1D | Both | 2.16 (2.00, 2.33） |
| Bosnia and Herzegovina | 10 to 14 | T1D | Female | 2.20 (1.97, 2.46） |
| Bosnia and Herzegovina | 10 to 14 | T1D | Male | 2.12 (1.90, 2.36） |
| Bosnia and Herzegovina | 15 to 19 | T1D | Both | 3.23 (2.99, 3.48） |
| Bosnia and Herzegovina | 15 to 19 | T1D | Female | 3.20 (2.87, 3.57） |
| Bosnia and Herzegovina | 15 to 19 | T1D | Male | 3.26 (2.93, 3.62） |
| Bosnia and Herzegovina | 20 to 24 | T1D | Both | 4.18 (3.87, 4.51） |
| Bosnia and Herzegovina | 20 to 24 | T1D | Female | 4.00 (3.59, 4.47） |
| Bosnia and Herzegovina | 20 to 24 | T1D | Male | 4.34 (3.90, 4.82） |
| Bosnia and Herzegovina | 25 to 29 | T1D | Both | 5.25 (4.86, 5.67） |
| Bosnia and Herzegovina | 25 to 29 | T1D | Female | 4.89 (4.37, 5.47） |
| Bosnia and Herzegovina | 25 to 29 | T1D | Male | 5.58 (5.01, 6.22） |
| Bosnia and Herzegovina | 30 to 34 | T1D | Both | 6.35 (5.86, 6.87） |
| Bosnia and Herzegovina | 30 to 34 | T1D | Female | 5.79 (5.16, 6.49） |
| Bosnia and Herzegovina | 30 to 34 | T1D | Male | 6.89 (6.17, 7.69） |
| Bosnia and Herzegovina | 35 to 39 | T1D | Both | 7.53 (6.95, 8.17） |
| Bosnia and Herzegovina | 35 to 39 | T1D | Female | 6.74 (6.00, 7.58） |
| Bosnia and Herzegovina | 35 to 39 | T1D | Male | 8.29 (7.41, 9.28） |
| Bosnia and Herzegovina | 40 to 44 | T1D | Both | 8.83 (8.13, 9.58） |
| Bosnia and Herzegovina | 40 to 44 | T1D | Female | 7.80 (6.92, 8.79） |
| Bosnia and Herzegovina | 40 to 44 | T1D | Male | 9.83 (8.77, 11.01） |
| Bosnia and Herzegovina | 45 to 49 | T1D | Both | 10.25 (9.42, 11.14） |

| | | | | |
|---|---|---|---|---|
| Bosnia and Herzegovina | 45 to 49 | T1D | Female | 8.98 (7.95, 10.14） |
| Bosnia and Herzegovina | 45 to 49 | T1D | Male | 11.49 (10.23, 12.89） |
| Bosnia and Herzegovina | 5 to 9 | T1D | Both | 1.00 (1.00, 1.00） |
| Bosnia and Herzegovina | 5 to 9 | T1D | Female | 1.00 (1.00, 1.00） |
| Bosnia and Herzegovina | 5 to 9 | T1D | Male | 1.00 (1.00, 1.00） |
| Bosnia and Herzegovina | 50 to 54 | T1D | Both | 11.77 (10.81, 12.82） |
| Bosnia and Herzegovina | 50 to 54 | T1D | Female | 10.27 (9.08, 11.62） |
| Bosnia and Herzegovina | 50 to 54 | T1D | Male | 13.27 (11.80, 14.93） |
| Bosnia and Herzegovina | 55 to 59 | T1D | Both | 13.42 (12.30, 14.63） |
| Bosnia and Herzegovina | 55 to 59 | T1D | Female | 11.72 (10.33, 13.29） |
| Bosnia and Herzegovina | 55 to 59 | T1D | Male | 15.19 (13.48, 17.12） |
| Bosnia and Herzegovina | 60 to 64 | T1D | Both | 15.20 (13.91, 16.61） |
| Bosnia and Herzegovina | 60 to 64 | T1D | Female | 13.33 (11.72, 15.17） |
| Bosnia and Herzegovina | 60 to 64 | T1D | Male | 17.27 (15.28, 19.52） |
| Bosnia and Herzegovina | 65 to 69 | T1D | Both | 17.15 (15.65, 18.79） |
| Bosnia and Herzegovina | 65 to 69 | T1D | Female | 15.15 (13.27, 17.30） |
| Bosnia and Herzegovina | 65 to 69 | T1D | Male | 19.53 (17.21, 22.16） |
| Botswana | 0 to 4 | T1D | Both | 0.17 (0.13, 0.20） |
| Botswana | 0 to 4 | T1D | Female | 0.17 (0.12, 0.22） |
| Botswana | 0 to 4 | T1D | Male | 0.17 (0.13, 0.22） |
| Botswana | 10 to 14 | T1D | Both | 2.05 (1.87, 2.26） |
| Botswana | 10 to 14 | T1D | Female | 2.06 (1.80, 2.36） |
| Botswana | 10 to 14 | T1D | Male | 2.05 (1.80, 2.34） |
| Botswana | 15 to 19 | T1D | Both | 2.99 (2.72, 3.29） |
| Botswana | 15 to 19 | T1D | Female | 3.00 (2.61, 3.43） |
| Botswana | 15 to 19 | T1D | Male | 2.99 (2.61, 3.41） |
| Botswana | 20 to 24 | T1D | Both | 3.79 (3.44, 4.18） |
| Botswana | 20 to 24 | T1D | Female | 3.80 (3.31, 4.37） |
| Botswana | 20 to 24 | T1D | Male | 3.80 (3.32, 4.35） |
| Botswana | 25 to 29 | T1D | Both | 4.63 (4.20, 5.11） |
| Botswana | 25 to 29 | T1D | Female | 4.66 (4.05, 5.36） |
| Botswana | 25 to 29 | T1D | Male | 4.63 (4.03, 5.32） |
| Botswana | 30 to 34 | T1D | Both | 5.27 (4.76, 5.83） |

| | | | | |
|---|---|---|---|---|
| Botswana | 30 to 34 | T1D | Female | 5.29 (4.58, 6.12） |
| Botswana | 30 to 34 | T1D | Male | 5.25 (4.55, 6.06） |
| Botswana | 35 to 39 | T1D | Both | 5.76 (5.18, 6.40） |
| Botswana | 35 to 39 | T1D | Female | 5.80 (4.98, 6.75） |
| Botswana | 35 to 39 | T1D | Male | 5.74 (4.94, 6.66） |
| Botswana | 40 to 44 | T1D | Both | 6.16 (5.51, 6.89） |
| Botswana | 40 to 44 | T1D | Female | 6.21 (5.31, 7.28） |
| Botswana | 40 to 44 | T1D | Male | 6.13 (5.25, 7.17） |
| Botswana | 45 to 49 | T1D | Both | 6.56 (5.84, 7.37） |
| Botswana | 45 to 49 | T1D | Female | 6.62 (5.62, 7.81） |
| Botswana | 45 to 49 | T1D | Male | 6.52 (5.52, 7.69） |
| Botswana | 5 to 9 | T1D | Both | 1.00 (1.00, 1.00） |
| Botswana | 5 to 9 | T1D | Female | 1.00 (1.00, 1.00） |
| Botswana | 5 to 9 | T1D | Male | 1.00 (1.00, 1.00） |
| Botswana | 50 to 54 | T1D | Both | 6.96 (6.15, 7.87） |
| Botswana | 50 to 54 | T1D | Female | 7.04 (5.92, 8.37） |
| Botswana | 50 to 54 | T1D | Male | 6.90 (5.79, 8.22） |
| Botswana | 55 to 59 | T1D | Both | 7.30 (6.41, 8.32） |
| Botswana | 55 to 59 | T1D | Female | 7.41 (6.17, 8.90） |
| Botswana | 55 to 59 | T1D | Male | 7.23 (6.00, 8.71） |
| Botswana | 60 to 64 | T1D | Both | 7.65 (6.65, 8.79） |
| Botswana | 60 to 64 | T1D | Female | 7.76 (6.38, 9.44） |
| Botswana | 60 to 64 | T1D | Male | 7.54 (6.17, 9.22） |
| Botswana | 65 to 69 | T1D | Both | 7.93 (6.80, 9.23） |
| Botswana | 65 to 69 | T1D | Female | 8.02 (6.49, 9.92） |
| Botswana | 65 to 69 | T1D | Male | 7.82 (6.27, 9.75） |
| Brazil | 0 to 4 | T1D | Both | 0.14 (0.14, 0.15） |
| Brazil | 0 to 4 | T1D | Female | 0.13 (0.13, 0.14） |
| Brazil | 0 to 4 | T1D | Male | 0.16 (0.16, 0.17） |
| Brazil | 10 to 14 | T1D | Both | 2.23 (2.21, 2.25） |
| Brazil | 10 to 14 | T1D | Female | 2.29 (2.27, 2.32） |
| Brazil | 10 to 14 | T1D | Male | 2.11 (2.08, 2.14） |
| Brazil | 15 to 19 | T1D | Both | 3.28 (3.25, 3.31） |

| | | | | |
|---|---|---|---|---|
| Brazil | 15 to 19 | T1D | Female | 3.34 (3.31, 3.38） |
| Brazil | 15 to 19 | T1D | Male | 3.11 (3.06, 3.16） |
| Brazil | 20 to 24 | T1D | Both | 4.11 (4.07, 4.15） |
| Brazil | 20 to 24 | T1D | Female | 4.15 (4.10, 4.19） |
| Brazil | 20 to 24 | T1D | Male | 3.94 (3.88, 4.00） |
| Brazil | 25 to 29 | T1D | Both | 4.95 (4.90, 4.99） |
| Brazil | 25 to 29 | T1D | Female | 4.97 (4.91, 5.02） |
| Brazil | 25 to 29 | T1D | Male | 4.73 (4.66, 4.81） |
| Brazil | 30 to 34 | T1D | Both | 5.63 (5.58, 5.69） |
| Brazil | 30 to 34 | T1D | Female | 5.71 (5.64, 5.77） |
| Brazil | 30 to 34 | T1D | Male | 5.29 (5.21, 5.38） |
| Brazil | 35 to 39 | T1D | Both | 6.29 (6.23, 6.35） |
| Brazil | 35 to 39 | T1D | Female | 6.47 (6.39, 6.55） |
| Brazil | 35 to 39 | T1D | Male | 5.77 (5.67, 5.87） |
| Brazil | 40 to 44 | T1D | Both | 6.96 (6.89, 7.03） |
| Brazil | 40 to 44 | T1D | Female | 7.30 (7.21, 7.39） |
| Brazil | 40 to 44 | T1D | Male | 6.21 (6.10, 6.31） |
| Brazil | 45 to 49 | T1D | Both | 7.63 (7.56, 7.71） |
| Brazil | 45 to 49 | T1D | Female | 8.18 (8.07, 8.29） |
| Brazil | 45 to 49 | T1D | Male | 6.59 (6.47, 6.70） |
| Brazil | 5 to 9 | T1D | Both | 1.00 (1.00, 1.00） |
| Brazil | 5 to 9 | T1D | Female | 1.00 (1.00, 1.00） |
| Brazil | 5 to 9 | T1D | Male | 1.00 (1.00, 1.00） |
| Brazil | 50 to 54 | T1D | Both | 8.32 (8.23, 8.40） |
| Brazil | 50 to 54 | T1D | Female | 9.12 (9.00, 9.24） |
| Brazil | 50 to 54 | T1D | Male | 6.93 (6.80, 7.05） |
| Brazil | 55 to 59 | T1D | Both | 9.02 (8.92, 9.12） |
| Brazil | 55 to 59 | T1D | Female | 10.14 (10.01, 10.28） |
| Brazil | 55 to 59 | T1D | Male | 7.22 (7.09, 7.36） |
| Brazil | 60 to 64 | T1D | Both | 9.74 (9.63, 9.85） |
| Brazil | 60 to 64 | T1D | Female | 11.25 (11.09, 11.42） |
| Brazil | 60 to 64 | T1D | Male | 7.48 (7.34, 7.63） |
| Brazil | 65 to 69 | T1D | Both | 10.49 (10.36, 10.62） |

| | | | | |
|---|---|---|---|---|
| Brazil | 65 to 69 | T1D | Female | 12.46 (12.27, 12.65） |
| Brazil | 65 to 69 | T1D | Male | 7.70 (7.55, 7.86） |
| Brunei Darussalam | 0 to 4 | T1D | Both | 0.15 (0.11, 0.20） |
| Brunei Darussalam | 0 to 4 | T1D | Female | 0.15 (0.10, 0.23） |
| Brunei Darussalam | 0 to 4 | T1D | Male | 0.15 (0.10, 0.23） |
| Brunei Darussalam | 10 to 14 | T1D | Both | 1.83 (1.61, 2.08） |
| Brunei Darussalam | 10 to 14 | T1D | Female | 1.80 (1.50, 2.17） |
| Brunei Darussalam | 10 to 14 | T1D | Male | 1.83 (1.54, 2.19） |
| Brunei Darussalam | 15 to 19 | T1D | Both | 2.06 (1.81, 2.35） |
| Brunei Darussalam | 15 to 19 | T1D | Female | 2.03 (1.68, 2.45） |
| Brunei Darussalam | 15 to 19 | T1D | Male | 2.07 (1.73, 2.47） |
| Brunei Darussalam | 20 to 24 | T1D | Both | 2.11 (1.84, 2.41） |
| Brunei Darussalam | 20 to 24 | T1D | Female | 2.07 (1.70, 2.52） |
| Brunei Darussalam | 20 to 24 | T1D | Male | 2.11 (1.76, 2.54） |
| Brunei Darussalam | 25 to 29 | T1D | Both | 2.15 (1.87, 2.47） |
| Brunei Darussalam | 25 to 29 | T1D | Female | 2.12 (1.73, 2.59） |
| Brunei Darussalam | 25 to 29 | T1D | Male | 2.16 (1.79, 2.62） |
| Brunei Darussalam | 30 to 34 | T1D | Both | 2.18 (1.89, 2.52） |
| Brunei Darussalam | 30 to 34 | T1D | Female | 2.14 (1.73, 2.65） |
| Brunei Darussalam | 30 to 34 | T1D | Male | 2.20 (1.81, 2.68） |
| Brunei Darussalam | 35 to 39 | T1D | Both | 2.21 (1.90, 2.57） |
| Brunei Darussalam | 35 to 39 | T1D | Female | 2.16 (1.73, 2.70） |
| Brunei Darussalam | 35 to 39 | T1D | Male | 2.24 (1.82, 2.75） |
| Brunei Darussalam | 40 to 44 | T1D | Both | 2.25 (1.91, 2.63） |
| Brunei Darussalam | 40 to 44 | T1D | Female | 2.19 (1.73, 2.77） |
| Brunei Darussalam | 40 to 44 | T1D | Male | 2.27 (1.83, 2.82） |
| Brunei Darussalam | 45 to 49 | T1D | Both | 2.27 (1.92, 2.69） |
| Brunei Darussalam | 45 to 49 | T1D | Female | 2.22 (1.73, 2.85） |
| Brunei Darussalam | 45 to 49 | T1D | Male | 2.28 (1.81, 2.88） |
| Brunei Darussalam | 5 to 9 | T1D | Both | 1.00 (1.00, 1.00） |
| Brunei Darussalam | 5 to 9 | T1D | Female | 1.00 (1.00, 1.00） |
| Brunei Darussalam | 5 to 9 | T1D | Male | 1.00 (1.00, 1.00） |
| Brunei Darussalam | 50 to 54 | T1D | Both | 2.28 (1.90, 2.74） |

| | | | | |
|---|---|---|---|---|
| Brunei Darussalam | 50 to 54 | T1D | Female | 2.23 (1.71, 2.92） |
| Brunei Darussalam | 50 to 54 | T1D | Male | 2.32 (1.81, 2.97） |
| Brunei Darussalam | 55 to 59 | T1D | Both | 2.31 (1.89, 2.82） |
| Brunei Darussalam | 55 to 59 | T1D | Female | 2.26 (1.68, 3.03） |
| Brunei Darussalam | 55 to 59 | T1D | Male | 2.31 (1.76, 3.04） |
| Brunei Darussalam | 60 to 64 | T1D | Both | 2.32 (1.85, 2.91） |
| Brunei Darussalam | 60 to 64 | T1D | Female | 2.27 (1.63, 3.17） |
| Brunei Darussalam | 60 to 64 | T1D | Male | 2.34 (1.71, 3.20） |
| Brunei Darussalam | 65 to 69 | T1D | Both | 2.31 (1.76, 3.02） |
| Brunei Darussalam | 65 to 69 | T1D | Female | 2.26 (1.53, 3.35） |
| Brunei Darussalam | 65 to 69 | T1D | Male | 2.35 (1.62, 3.41） |
| Bulgaria | 0 to 4 | T1D | Both | 0.19 (0.17, 0.22） |
| Bulgaria | 0 to 4 | T1D | Female | 0.18 (0.15, 0.22） |
| Bulgaria | 0 to 4 | T1D | Male | 0.20 (0.17, 0.24） |
| Bulgaria | 10 to 14 | T1D | Both | 2.20 (2.06, 2.34） |
| Bulgaria | 10 to 14 | T1D | Female | 2.20 (2.01, 2.40） |
| Bulgaria | 10 to 14 | T1D | Male | 2.19 (2.01, 2.39） |
| Bulgaria | 15 to 19 | T1D | Both | 3.33 (3.14, 3.54） |
| Bulgaria | 15 to 19 | T1D | Female | 3.25 (2.98, 3.55） |
| Bulgaria | 15 to 19 | T1D | Male | 3.41 (3.13, 3.72） |
| Bulgaria | 20 to 24 | T1D | Both | 4.32 (4.07, 4.59） |
| Bulgaria | 20 to 24 | T1D | Female | 4.10 (3.76, 4.48） |
| Bulgaria | 20 to 24 | T1D | Male | 4.53 (4.16, 4.94） |
| Bulgaria | 25 to 29 | T1D | Both | 5.41 (5.09, 5.76） |
| Bulgaria | 25 to 29 | T1D | Female | 5.01 (4.59, 5.46） |
| Bulgaria | 25 to 29 | T1D | Male | 5.81 (5.34, 6.34） |
| Bulgaria | 30 to 34 | T1D | Both | 6.47 (6.08, 6.88） |
| Bulgaria | 30 to 34 | T1D | Female | 5.86 (5.36, 6.40） |
| Bulgaria | 30 to 34 | T1D | Male | 7.08 (6.49, 7.72） |
| Bulgaria | 35 to 39 | T1D | Both | 7.58 (7.11, 8.07） |
| Bulgaria | 35 to 39 | T1D | Female | 6.75 (6.17, 7.39） |
| Bulgaria | 35 to 39 | T1D | Male | 8.40 (7.69, 9.18） |
| Bulgaria | 40 to 44 | T1D | Both | 8.78 (8.24, 9.36） |

| | | | | |
|---|---|---|---|---|
| Bulgaria | 40 to 44 | T1D | Female | 7.72 (7.04, 8.46） |
| Bulgaria | 40 to 44 | T1D | Male | 9.84 (9.00, 10.76） |
| Bulgaria | 45 to 49 | T1D | Both | 10.07 (9.44, 10.75） |
| Bulgaria | 45 to 49 | T1D | Female | 8.77 (7.99, 9.62） |
| Bulgaria | 45 to 49 | T1D | Male | 11.38 (10.40, 12.46） |
| Bulgaria | 5 to 9 | T1D | Both | 1.00 (1.00, 1.00） |
| Bulgaria | 5 to 9 | T1D | Female | 1.00 (1.00, 1.00） |
| Bulgaria | 5 to 9 | T1D | Male | 1.00 (1.00, 1.00） |
| Bulgaria | 50 to 54 | T1D | Both | 11.45 (10.72, 12.22） |
| Bulgaria | 50 to 54 | T1D | Female | 9.91 (9.02, 10.89） |
| Bulgaria | 50 to 54 | T1D | Male | 13.02 (11.88, 14.27） |
| Bulgaria | 55 to 59 | T1D | Both | 12.90 (12.07, 13.78） |
| Bulgaria | 55 to 59 | T1D | Female | 11.16 (10.14, 12.27） |
| Bulgaria | 55 to 59 | T1D | Male | 14.73 (13.43, 16.17） |
| Bulgaria | 60 to 64 | T1D | Both | 14.45 (13.51, 15.46） |
| Bulgaria | 60 to 64 | T1D | Female | 12.53 (11.38, 13.80） |
| Bulgaria | 60 to 64 | T1D | Male | 16.58 (15.09, 18.21） |
| Bulgaria | 65 to 69 | T1D | Both | 16.14 (15.07, 17.29） |
| Bulgaria | 65 to 69 | T1D | Female | 14.06 (12.75, 15.51） |
| Bulgaria | 65 to 69 | T1D | Male | 18.58 (16.89, 20.45） |
| Burkina Faso | 0 to 4 | T1D | Both | 0.15 (0.14, 0.16） |
| Burkina Faso | 0 to 4 | T1D | Female | 0.15 (0.14, 0.17） |
| Burkina Faso | 0 to 4 | T1D | Male | 0.15 (0.14, 0.17） |
| Burkina Faso | 10 to 14 | T1D | Both | 2.10 (2.03, 2.17） |
| Burkina Faso | 10 to 14 | T1D | Female | 2.10 (2.01, 2.20） |
| Burkina Faso | 10 to 14 | T1D | Male | 2.09 (2.00, 2.19） |
| Burkina Faso | 15 to 19 | T1D | Both | 3.02 (2.93, 3.13） |
| Burkina Faso | 15 to 19 | T1D | Female | 3.04 (2.90, 3.19） |
| Burkina Faso | 15 to 19 | T1D | Male | 3.02 (2.88, 3.16） |
| Burkina Faso | 20 to 24 | T1D | Both | 3.81 (3.68, 3.94） |
| Burkina Faso | 20 to 24 | T1D | Female | 3.84 (3.65, 4.03） |
| Burkina Faso | 20 to 24 | T1D | Male | 3.80 (3.62, 3.98） |
| Burkina Faso | 25 to 29 | T1D | Both | 4.59 (4.43, 4.76） |

| | | | | |
|---|---|---|---|---|
| Burkina Faso | 25 to 29 | T1D | Female | 4.63 (4.40, 4.87） |
| Burkina Faso | 25 to 29 | T1D | Male | 4.57 (4.35, 4.81） |
| Burkina Faso | 30 to 34 | T1D | Both | 5.06 (4.88, 5.26） |
| Burkina Faso | 30 to 34 | T1D | Female | 5.12 (4.85, 5.40） |
| Burkina Faso | 30 to 34 | T1D | Male | 5.03 (4.77, 5.31） |
| Burkina Faso | 35 to 39 | T1D | Both | 5.38 (5.17, 5.60） |
| Burkina Faso | 35 to 39 | T1D | Female | 5.44 (5.15, 5.76） |
| Burkina Faso | 35 to 39 | T1D | Male | 5.33 (5.04, 5.64） |
| Burkina Faso | 40 to 44 | T1D | Both | 5.62 (5.39, 5.86） |
| Burkina Faso | 40 to 44 | T1D | Female | 5.69 (5.37, 6.04） |
| Burkina Faso | 40 to 44 | T1D | Male | 5.56 (5.24, 5.90） |
| Burkina Faso | 45 to 49 | T1D | Both | 5.79 (5.54, 6.06） |
| Burkina Faso | 45 to 49 | T1D | Female | 5.88 (5.53, 6.26） |
| Burkina Faso | 45 to 49 | T1D | Male | 5.72 (5.37, 6.10） |
| Burkina Faso | 5 to 9 | T1D | Both | 1.00 (1.00, 1.00） |
| Burkina Faso | 5 to 9 | T1D | Female | 1.00 (1.00, 1.00） |
| Burkina Faso | 5 to 9 | T1D | Male | 1.00 (1.00, 1.00） |
| Burkina Faso | 50 to 54 | T1D | Both | 5.92 (5.64, 6.20） |
| Burkina Faso | 50 to 54 | T1D | Female | 6.02 (5.63, 6.43） |
| Burkina Faso | 50 to 54 | T1D | Male | 5.83 (5.44, 6.24） |
| Burkina Faso | 55 to 59 | T1D | Both | 5.99 (5.69, 6.30） |
| Burkina Faso | 55 to 59 | T1D | Female | 6.10 (5.68, 6.55） |
| Burkina Faso | 55 to 59 | T1D | Male | 5.88 (5.46, 6.33） |
| Burkina Faso | 60 to 64 | T1D | Both | 6.01 (5.69, 6.36） |
| Burkina Faso | 60 to 64 | T1D | Female | 6.14 (5.68, 6.63） |
| Burkina Faso | 60 to 64 | T1D | Male | 5.89 (5.44, 6.38） |
| Burkina Faso | 65 to 69 | T1D | Both | 6.00 (5.65, 6.38） |
| Burkina Faso | 65 to 69 | T1D | Female | 6.15 (5.64, 6.70） |
| Burkina Faso | 65 to 69 | T1D | Male | 5.87 (5.37, 6.41） |
| Burundi | 0 to 4 | T1D | Both | 0.15 (0.14, 0.17） |
| Burundi | 0 to 4 | T1D | Female | 0.15 (0.13, 0.17） |
| Burundi | 0 to 4 | T1D | Male | 0.15 (0.13, 0.17） |
| Burundi | 10 to 14 | T1D | Both | 2.05 (1.96, 2.14） |

| | | | | |
|---|---|---|---|---|
| Burundi | 10 to 14 | T1D | Female | 2.05 (1.93, 2.18） |
| Burundi | 10 to 14 | T1D | Male | 2.05 (1.93, 2.18） |
| Burundi | 15 to 19 | T1D | Both | 2.99 (2.86, 3.12） |
| Burundi | 15 to 19 | T1D | Female | 2.99 (2.81, 3.19） |
| Burundi | 15 to 19 | T1D | Male | 2.98 (2.80, 3.18） |
| Burundi | 20 to 24 | T1D | Both | 3.84 (3.67, 4.02） |
| Burundi | 20 to 24 | T1D | Female | 3.85 (3.61, 4.11） |
| Burundi | 20 to 24 | T1D | Male | 3.83 (3.59, 4.09） |
| Burundi | 25 to 29 | T1D | Both | 4.68 (4.46, 4.91） |
| Burundi | 25 to 29 | T1D | Female | 4.70 (4.39, 5.03） |
| Burundi | 25 to 29 | T1D | Male | 4.66 (4.36, 4.99） |
| Burundi | 30 to 34 | T1D | Both | 5.19 (4.94, 5.46） |
| Burundi | 30 to 34 | T1D | Female | 5.22 (4.86, 5.61） |
| Burundi | 30 to 34 | T1D | Male | 5.16 (4.81, 5.54） |
| Burundi | 35 to 39 | T1D | Both | 5.54 (5.26, 5.84） |
| Burundi | 35 to 39 | T1D | Female | 5.58 (5.18, 6.02） |
| Burundi | 35 to 39 | T1D | Male | 5.50 (5.10, 5.92） |
| Burundi | 40 to 44 | T1D | Both | 5.81 (5.50, 6.14） |
| Burundi | 40 to 44 | T1D | Female | 5.87 (5.42, 6.35） |
| Burundi | 40 to 44 | T1D | Male | 5.75 (5.32, 6.21） |
| Burundi | 45 to 49 | T1D | Both | 6.01 (5.67, 6.37） |
| Burundi | 45 to 49 | T1D | Female | 6.08 (5.59, 6.61） |
| Burundi | 45 to 49 | T1D | Male | 5.93 (5.46, 6.43） |
| Burundi | 5 to 9 | T1D | Both | 1.00 (1.00, 1.00） |
| Burundi | 5 to 9 | T1D | Female | 1.00 (1.00, 1.00） |
| Burundi | 5 to 9 | T1D | Male | 1.00 (1.00, 1.00） |
| Burundi | 50 to 54 | T1D | Both | 6.15 (5.78, 6.54） |
| Burundi | 50 to 54 | T1D | Female | 6.23 (5.70, 6.81） |
| Burundi | 50 to 54 | T1D | Male | 6.04 (5.54, 6.60） |
| Burundi | 55 to 59 | T1D | Both | 6.25 (5.84, 6.68） |
| Burundi | 55 to 59 | T1D | Female | 6.34 (5.75, 6.97） |
| Burundi | 55 to 59 | T1D | Male | 6.13 (5.58, 6.73） |
| Burundi | 60 to 64 | T1D | Both | 6.31 (5.86, 6.79） |

| | | | | |
|---|---|---|---|---|
| Burundi | 60 to 64 | T1D | Female | 6.41 (5.77, 7.13） |
| Burundi | 60 to 64 | T1D | Male | 6.17 (5.56, 6.84） |
| Burundi | 65 to 69 | T1D | Both | 6.34 (5.83, 6.89） |
| Burundi | 65 to 69 | T1D | Female | 6.46 (5.74, 7.28） |
| Burundi | 65 to 69 | T1D | Male | 6.19 (5.50, 6.96） |
| Cabo Verde | 0 to 4 | T1D | Both | 0.16 (0.11, 0.25） |
| Cabo Verde | 0 to 4 | T1D | Female | 0.14 (0.07, 0.27） |
| Cabo Verde | 0 to 4 | T1D | Male | 0.19 (0.11, 0.33） |
| Cabo Verde | 10 to 14 | T1D | Both | 2.07 (1.71, 2.51） |
| Cabo Verde | 10 to 14 | T1D | Female | 2.09 (1.58, 2.76） |
| Cabo Verde | 10 to 14 | T1D | Male | 2.08 (1.59, 2.72） |
| Cabo Verde | 15 to 19 | T1D | Both | 3.01 (2.49, 3.65） |
| Cabo Verde | 15 to 19 | T1D | Female | 3.05 (2.31, 4.02） |
| Cabo Verde | 15 to 19 | T1D | Male | 3.02 (2.31, 3.94） |
| Cabo Verde | 20 to 24 | T1D | Both | 3.86 (3.17, 4.69） |
| Cabo Verde | 20 to 24 | T1D | Female | 3.91 (2.95, 5.19） |
| Cabo Verde | 20 to 24 | T1D | Male | 3.84 (2.92, 5.05） |
| Cabo Verde | 25 to 29 | T1D | Both | 4.73 (3.87, 5.78） |
| Cabo Verde | 25 to 29 | T1D | Female | 4.80 (3.59, 6.42） |
| Cabo Verde | 25 to 29 | T1D | Male | 4.73 (3.58, 6.26） |
| Cabo Verde | 30 to 34 | T1D | Both | 5.34 (4.34, 6.57） |
| Cabo Verde | 30 to 34 | T1D | Female | 5.43 (4.02, 7.33） |
| Cabo Verde | 30 to 34 | T1D | Male | 5.32 (3.99, 7.11） |
| Cabo Verde | 35 to 39 | T1D | Both | 5.81 (4.67, 7.22） |
| Cabo Verde | 35 to 39 | T1D | Female | 5.91 (4.32, 8.10） |
| Cabo Verde | 35 to 39 | T1D | Male | 5.79 (4.27, 7.85） |
| Cabo Verde | 40 to 44 | T1D | Both | 6.23 (4.96, 7.82） |
| Cabo Verde | 40 to 44 | T1D | Female | 6.37 (4.60, 8.84） |
| Cabo Verde | 40 to 44 | T1D | Male | 6.21 (4.51, 8.54） |
| Cabo Verde | 45 to 49 | T1D | Both | 6.63 (5.23, 8.41） |
| Cabo Verde | 45 to 49 | T1D | Female | 6.79 (4.84, 9.54） |
| Cabo Verde | 45 to 49 | T1D | Male | 6.58 (4.71, 9.20） |
| Cabo Verde | 5 to 9 | T1D | Both | 1.00 (1.00, 1.00） |

| | | | | |
|---|---|---|---|---|
| Cabo Verde | 5 to 9 | T1D | Female | 1.00 (1.00, 1.00） |
| Cabo Verde | 5 to 9 | T1D | Male | 1.00 (1.00, 1.00） |
| Cabo Verde | 50 to 54 | T1D | Both | 6.97 (5.42, 8.96） |
| Cabo Verde | 50 to 54 | T1D | Female | 7.14 (5.00, 10.21） |
| Cabo Verde | 50 to 54 | T1D | Male | 6.83 (4.78, 9.78） |
| Cabo Verde | 55 to 59 | T1D | Both | 7.30 (5.57, 9.55） |
| Cabo Verde | 55 to 59 | T1D | Female | 7.46 (5.11, 10.89） |
| Cabo Verde | 55 to 59 | T1D | Male | 7.23 (4.91, 10.65） |
| Cabo Verde | 60 to 64 | T1D | Both | 7.59 (5.70, 10.11） |
| Cabo Verde | 60 to 64 | T1D | Female | 7.83 (5.26, 11.65） |
| Cabo Verde | 60 to 64 | T1D | Male | 7.38 (4.85, 11.23） |
| Cabo Verde | 65 to 69 | T1D | Both | 7.81 (5.72, 10.64） |
| Cabo Verde | 65 to 69 | T1D | Female | 8.11 (5.29, 12.41） |
| Cabo Verde | 65 to 69 | T1D | Male | 7.77 (4.92, 12.29） |
| Cambodia | 0 to 4 | T1D | Both | 0.14 (0.12, 0.16） |
| Cambodia | 0 to 4 | T1D | Female | 0.14 (0.12, 0.16） |
| Cambodia | 0 to 4 | T1D | Male | 0.14 (0.11, 0.16） |
| Cambodia | 10 to 14 | T1D | Both | 2.14 (2.03, 2.26） |
| Cambodia | 10 to 14 | T1D | Female | 2.12 (1.97, 2.29） |
| Cambodia | 10 to 14 | T1D | Male | 2.17 (2.01, 2.35） |
| Cambodia | 15 to 19 | T1D | Both | 2.99 (2.83, 3.16） |
| Cambodia | 15 to 19 | T1D | Female | 2.90 (2.69, 3.13） |
| Cambodia | 15 to 19 | T1D | Male | 3.10 (2.87, 3.35） |
| Cambodia | 20 to 24 | T1D | Both | 3.63 (3.43, 3.84） |
| Cambodia | 20 to 24 | T1D | Female | 3.43 (3.17, 3.71） |
| Cambodia | 20 to 24 | T1D | Male | 3.85 (3.56, 4.18） |
| Cambodia | 25 to 29 | T1D | Both | 4.21 (3.98, 4.47） |
| Cambodia | 25 to 29 | T1D | Female | 3.89 (3.59, 4.22） |
| Cambodia | 25 to 29 | T1D | Male | 4.58 (4.22, 4.97） |
| Cambodia | 30 to 34 | T1D | Both | 4.61 (4.34, 4.90） |
| Cambodia | 30 to 34 | T1D | Female | 4.20 (3.86, 4.57） |
| Cambodia | 30 to 34 | T1D | Male | 5.08 (4.66, 5.54） |
| Cambodia | 35 to 39 | T1D | Both | 4.93 (4.63, 5.25） |

| | | | | |
|---|---|---|---|---|
| Cambodia | 35 to 39 | T1D | Female | 4.45 (4.07, 4.86） |
| Cambodia | 35 to 39 | T1D | Male | 5.48 (5.01, 6.01） |
| Cambodia | 40 to 44 | T1D | Both | 5.20 (4.86, 5.56） |
| Cambodia | 40 to 44 | T1D | Female | 4.66 (4.25, 5.12） |
| Cambodia | 40 to 44 | T1D | Male | 5.81 (5.28, 6.40） |
| Cambodia | 45 to 49 | T1D | Both | 5.43 (5.06, 5.82） |
| Cambodia | 45 to 49 | T1D | Female | 4.86 (4.41, 5.36） |
| Cambodia | 45 to 49 | T1D | Male | 6.10 (5.52, 6.74） |
| Cambodia | 5 to 9 | T1D | Both | 1.00 (1.00, 1.00） |
| Cambodia | 5 to 9 | T1D | Female | 1.00 (1.00, 1.00） |
| Cambodia | 5 to 9 | T1D | Male | 1.00 (1.00, 1.00） |
| Cambodia | 50 to 54 | T1D | Both | 5.62 (5.22, 6.05） |
| Cambodia | 50 to 54 | T1D | Female | 5.03 (4.54, 5.57） |
| Cambodia | 50 to 54 | T1D | Male | 6.32 (5.68, 7.03） |
| Cambodia | 55 to 59 | T1D | Both | 5.81 (5.37, 6.28） |
| Cambodia | 55 to 59 | T1D | Female | 5.20 (4.66, 5.79） |
| Cambodia | 55 to 59 | T1D | Male | 6.52 (5.81, 7.31） |
| Cambodia | 60 to 64 | T1D | Both | 5.97 (5.48, 6.50） |
| Cambodia | 60 to 64 | T1D | Female | 5.36 (4.77, 6.03） |
| Cambodia | 60 to 64 | T1D | Male | 6.70 (5.91, 7.60） |
| Cambodia | 65 to 69 | T1D | Both | 6.12 (5.57, 6.72） |
| Cambodia | 65 to 69 | T1D | Female | 5.51 (4.85, 6.26） |
| Cambodia | 65 to 69 | T1D | Male | 6.84 (5.94, 7.86） |
| Cameroon | 0 to 4 | T1D | Both | 0.16 (0.15, 0.17） |
| Cameroon | 0 to 4 | T1D | Female | 0.16 (0.15, 0.18） |
| Cameroon | 0 to 4 | T1D | Male | 0.16 (0.15, 0.18） |
| Cameroon | 10 to 14 | T1D | Both | 2.06 (2.00, 2.12） |
| Cameroon | 10 to 14 | T1D | Female | 2.06 (1.98, 2.15） |
| Cameroon | 10 to 14 | T1D | Male | 2.06 (1.98, 2.14） |
| Cameroon | 15 to 19 | T1D | Both | 3.02 (2.93, 3.11） |
| Cameroon | 15 to 19 | T1D | Female | 3.03 (2.91, 3.17） |
| Cameroon | 15 to 19 | T1D | Male | 3.01 (2.89, 3.14） |
| Cameroon | 20 to 24 | T1D | Both | 3.87 (3.75, 3.99） |

| | | | | |
|---|---|---|---|---|
| Cameroon | 20 to 24 | T1D | Female | 3.89 (3.73, 4.07） |
| Cameroon | 20 to 24 | T1D | Male | 3.85 (3.69, 4.02） |
| Cameroon | 25 to 29 | T1D | Both | 4.75 (4.60, 4.90） |
| Cameroon | 25 to 29 | T1D | Female | 4.79 (4.58, 5.01） |
| Cameroon | 25 to 29 | T1D | Male | 4.73 (4.52, 4.94） |
| Cameroon | 30 to 34 | T1D | Both | 5.34 (5.17, 5.52） |
| Cameroon | 30 to 34 | T1D | Female | 5.39 (5.14, 5.65） |
| Cameroon | 30 to 34 | T1D | Male | 5.30 (5.06, 5.55） |
| Cameroon | 35 to 39 | T1D | Both | 5.79 (5.59, 6.00） |
| Cameroon | 35 to 39 | T1D | Female | 5.85 (5.56, 6.15） |
| Cameroon | 35 to 39 | T1D | Male | 5.74 (5.47, 6.03） |
| Cameroon | 40 to 44 | T1D | Both | 6.18 (5.96, 6.41） |
| Cameroon | 40 to 44 | T1D | Female | 6.25 (5.93, 6.59） |
| Cameroon | 40 to 44 | T1D | Male | 6.11 (5.80, 6.44） |
| Cameroon | 45 to 49 | T1D | Both | 6.51 (6.26, 6.77） |
| Cameroon | 45 to 49 | T1D | Female | 6.60 (6.24, 6.98） |
| Cameroon | 45 to 49 | T1D | Male | 6.43 (6.09, 6.79） |
| Cameroon | 5 to 9 | T1D | Both | 1.00 (1.00, 1.00） |
| Cameroon | 5 to 9 | T1D | Female | 1.00 (1.00, 1.00） |
| Cameroon | 5 to 9 | T1D | Male | 1.00 (1.00, 1.00） |
| Cameroon | 50 to 54 | T1D | Both | 6.80 (6.52, 7.08） |
| Cameroon | 50 to 54 | T1D | Female | 6.90 (6.50, 7.32） |
| Cameroon | 50 to 54 | T1D | Male | 6.69 (6.31, 7.09） |
| Cameroon | 55 to 59 | T1D | Both | 7.03 (6.72, 7.35） |
| Cameroon | 55 to 59 | T1D | Female | 7.15 (6.71, 7.62） |
| Cameroon | 55 to 59 | T1D | Male | 6.91 (6.49, 7.35） |
| Cameroon | 60 to 64 | T1D | Both | 7.22 (6.88, 7.58） |
| Cameroon | 60 to 64 | T1D | Female | 7.36 (6.87, 7.88） |
| Cameroon | 60 to 64 | T1D | Male | 7.08 (6.62, 7.58） |
| Cameroon | 65 to 69 | T1D | Both | 7.37 (6.99, 7.77） |
| Cameroon | 65 to 69 | T1D | Female | 7.53 (6.99, 8.12） |
| Cameroon | 65 to 69 | T1D | Male | 7.21 (6.70, 7.77） |
| Canada | 0 to 4 | T1D | Both | 0.25 (0.23, 0.27） |

| | | | | |
|---|---|---|---|---|
| Canada | 0 to 4 | T1D | Female | 0.24 (0.22, 0.26） |
| Canada | 0 to 4 | T1D | Male | 0.25 (0.24, 0.27） |
| Canada | 10 to 14 | T1D | Both | 1.86 (1.79, 1.94） |
| Canada | 10 to 14 | T1D | Female | 1.85 (1.78, 1.93） |
| Canada | 10 to 14 | T1D | Male | 1.88 (1.80, 1.95） |
| Canada | 15 to 19 | T1D | Both | 2.69 (2.58, 2.79） |
| Canada | 15 to 19 | T1D | Female | 2.66 (2.56, 2.77） |
| Canada | 15 to 19 | T1D | Male | 2.71 (2.60, 2.82） |
| Canada | 20 to 24 | T1D | Both | 3.39 (3.27, 3.53） |
| Canada | 20 to 24 | T1D | Female | 3.35 (3.22, 3.49） |
| Canada | 20 to 24 | T1D | Male | 3.43 (3.29, 3.57） |
| Canada | 25 to 29 | T1D | Both | 4.02 (3.86, 4.18） |
| Canada | 25 to 29 | T1D | Female | 3.92 (3.77, 4.08） |
| Canada | 25 to 29 | T1D | Male | 4.10 (3.93, 4.27） |
| Canada | 30 to 34 | T1D | Both | 4.65 (4.47, 4.84） |
| Canada | 30 to 34 | T1D | Female | 4.50 (4.32, 4.69） |
| Canada | 30 to 34 | T1D | Male | 4.79 (4.59, 4.99） |
| Canada | 35 to 39 | T1D | Both | 5.29 (5.08, 5.51） |
| Canada | 35 to 39 | T1D | Female | 5.08 (4.87, 5.30） |
| Canada | 35 to 39 | T1D | Male | 5.49 (5.26, 5.73） |
| Canada | 40 to 44 | T1D | Both | 5.94 (5.69, 6.19） |
| Canada | 40 to 44 | T1D | Female | 5.66 (5.42, 5.91） |
| Canada | 40 to 44 | T1D | Male | 6.21 (5.95, 6.49） |
| Canada | 45 to 49 | T1D | Both | 6.60 (6.33, 6.89） |
| Canada | 45 to 49 | T1D | Female | 6.25 (5.99, 6.53） |
| Canada | 45 to 49 | T1D | Male | 6.96 (6.67, 7.27） |
| Canada | 5 to 9 | T1D | Both | 1.00 (1.00, 1.00） |
| Canada | 5 to 9 | T1D | Female | 1.00 (1.00, 1.00） |
| Canada | 5 to 9 | T1D | Male | 1.00 (1.00, 1.00） |
| Canada | 50 to 54 | T1D | Both | 7.30 (6.99, 7.62） |
| Canada | 50 to 54 | T1D | Female | 6.87 (6.57, 7.18） |
| Canada | 50 to 54 | T1D | Male | 7.75 (7.42, 8.10） |
| Canada | 55 to 59 | T1D | Both | 8.02 (7.68, 8.38） |

| | | | | |
|---|---|---|---|---|
| Canada | 55 to 59 | T1D | Female | 7.51 (7.18, 7.85） |
| Canada | 55 to 59 | T1D | Male | 8.57 (8.19, 8.96） |
| Canada | 60 to 64 | T1D | Both | 8.78 (8.40, 9.19） |
| Canada | 60 to 64 | T1D | Female | 8.18 (7.81, 8.56） |
| Canada | 60 to 64 | T1D | Male | 9.42 (9.00, 9.87） |
| Canada | 65 to 69 | T1D | Both | 9.61 (9.18, 10.06） |
| Canada | 65 to 69 | T1D | Female | 8.90 (8.49, 9.33） |
| Canada | 65 to 69 | T1D | Male | 10.34 (9.86, 10.84） |
| Central African Republic | 0 to 4 | T1D | Both | 0.14 (0.13, 0.16） |
| Central African Republic | 0 to 4 | T1D | Female | 0.14 (0.12, 0.17） |
| Central African Republic | 0 to 4 | T1D | Male | 0.14 (0.12, 0.17） |
| Central African Republic | 10 to 14 | T1D | Both | 2.16 (2.03, 2.30） |
| Central African Republic | 10 to 14 | T1D | Female | 2.15 (1.97, 2.35） |
| Central African Republic | 10 to 14 | T1D | Male | 2.16 (1.99, 2.36） |
| Central African Republic | 15 to 19 | T1D | Both | 3.16 (2.97, 3.37） |
| Central African Republic | 15 to 19 | T1D | Female | 3.16 (2.89, 3.45） |
| Central African Republic | 15 to 19 | T1D | Male | 3.17 (2.90, 3.45） |
| Central African Republic | 20 to 24 | T1D | Both | 3.97 (3.72, 4.23） |
| Central African Republic | 20 to 24 | T1D | Female | 3.96 (3.61, 4.34） |
| Central African Republic | 20 to 24 | T1D | Male | 3.96 (3.62, 4.34） |
| Central African Republic | 25 to 29 | T1D | Both | 4.74 (4.43, 5.07） |
| Central African Republic | 25 to 29 | T1D | Female | 4.75 (4.32, 5.23） |
| Central African Republic | 25 to 29 | T1D | Male | 4.72 (4.30, 5.19） |
| Central African Republic | 30 to 34 | T1D | Both | 5.24 (4.89, 5.62） |
| Central African Republic | 30 to 34 | T1D | Female | 5.27 (4.77, 5.82） |
| Central African Republic | 30 to 34 | T1D | Male | 5.21 (4.72, 5.75） |
| Central African Republic | 35 to 39 | T1D | Both | 5.58 (5.18, 6.00） |
| Central African Republic | 35 to 39 | T1D | Female | 5.62 (5.06, 6.24） |
| Central African Republic | 35 to 39 | T1D | Male | 5.53 (4.99, 6.12） |
| Central African Republic | 40 to 44 | T1D | Both | 5.81 (5.38, 6.27） |
| Central African Republic | 40 to 44 | T1D | Female | 5.87 (5.25, 6.55） |
| Central African Republic | 40 to 44 | T1D | Male | 5.74 (5.15, 6.39） |
| Central African Republic | 45 to 49 | T1D | Both | 5.99 (5.52, 6.50） |

| | | | | |
|---|---|---|---|---|
| Central African Republic | 45 to 49 | T1D | Female | 6.06 (5.39, 6.81） |
| Central African Republic | 45 to 49 | T1D | Male | 5.89 (5.26, 6.61） |
| Central African Republic | 5 to 9 | T1D | Both | 1.00 (1.00, 1.00） |
| Central African Republic | 5 to 9 | T1D | Female | 1.00 (1.00, 1.00） |
| Central African Republic | 5 to 9 | T1D | Male | 1.00 (1.00, 1.00） |
| Central African Republic | 50 to 54 | T1D | Both | 6.13 (5.62, 6.69） |
| Central African Republic | 50 to 54 | T1D | Female | 6.23 (5.50, 7.05） |
| Central African Republic | 50 to 54 | T1D | Male | 6.01 (5.32, 6.80） |
| Central African Republic | 55 to 59 | T1D | Both | 6.22 (5.66, 6.84） |
| Central African Republic | 55 to 59 | T1D | Female | 6.34 (5.55, 7.25） |
| Central African Republic | 55 to 59 | T1D | Male | 6.10 (5.34, 6.97） |
| Central African Republic | 60 to 64 | T1D | Both | 6.26 (5.64, 6.94） |
| Central African Republic | 60 to 64 | T1D | Female | 6.40 (5.53, 7.41） |
| Central African Republic | 60 to 64 | T1D | Male | 6.11 (5.27, 7.09） |
| Central African Republic | 65 to 69 | T1D | Both | 6.22 (5.53, 7.00） |
| Central African Republic | 65 to 69 | T1D | Female | 6.38 (5.42, 7.52） |
| Central African Republic | 65 to 69 | T1D | Male | 6.05 (5.09, 7.18） |
| Chad | 0 to 4 | T1D | Both | 0.15 (0.14, 0.16） |
| Chad | 0 to 4 | T1D | Female | 0.15 (0.13, 0.16） |
| Chad | 0 to 4 | T1D | Male | 0.15 (0.13, 0.16） |
| Chad | 10 to 14 | T1D | Both | 2.13 (2.05, 2.21） |
| Chad | 10 to 14 | T1D | Female | 2.13 (2.01, 2.25） |
| Chad | 10 to 14 | T1D | Male | 2.13 (2.02, 2.25） |
| Chad | 15 to 19 | T1D | Both | 3.08 (2.96, 3.21） |
| Chad | 15 to 19 | T1D | Female | 3.09 (2.92, 3.27） |
| Chad | 15 to 19 | T1D | Male | 3.08 (2.92, 3.26） |
| Chad | 20 to 24 | T1D | Both | 3.89 (3.73, 4.06） |
| Chad | 20 to 24 | T1D | Female | 3.91 (3.68, 4.15） |
| Chad | 20 to 24 | T1D | Male | 3.89 (3.67, 4.13） |
| Chad | 25 to 29 | T1D | Both | 4.69 (4.49, 4.90） |
| Chad | 25 to 29 | T1D | Female | 4.72 (4.44, 5.02） |
| Chad | 25 to 29 | T1D | Male | 4.68 (4.40, 4.98） |
| Chad | 30 to 34 | T1D | Both | 5.17 (4.93, 5.41） |

| | | | | |
|---|---|---|---|---|
| Chad | 30 to 34 | T1D | Female | 5.21 (4.88, 5.56） |
| Chad | 30 to 34 | T1D | Male | 5.15 (4.82, 5.49） |
| Chad | 35 to 39 | T1D | Both | 5.49 (5.23, 5.76） |
| Chad | 35 to 39 | T1D | Female | 5.54 (5.17, 5.93） |
| Chad | 35 to 39 | T1D | Male | 5.45 (5.09, 5.84） |
| Chad | 40 to 44 | T1D | Both | 5.73 (5.44, 6.04） |
| Chad | 40 to 44 | T1D | Female | 5.79 (5.38, 6.23） |
| Chad | 40 to 44 | T1D | Male | 5.68 (5.28, 6.11） |
| Chad | 45 to 49 | T1D | Both | 5.91 (5.60, 6.25） |
| Chad | 45 to 49 | T1D | Female | 5.98 (5.53, 6.47） |
| Chad | 45 to 49 | T1D | Male | 5.85 (5.41, 6.32） |
| Chad | 5 to 9 | T1D | Both | 1.00 (1.00, 1.00） |
| Chad | 5 to 9 | T1D | Female | 1.00 (1.00, 1.00） |
| Chad | 5 to 9 | T1D | Male | 1.00 (1.00, 1.00） |
| Chad | 50 to 54 | T1D | Both | 6.03 (5.69, 6.40） |
| Chad | 50 to 54 | T1D | Female | 6.11 (5.62, 6.65） |
| Chad | 50 to 54 | T1D | Male | 5.95 (5.48, 6.47） |
| Chad | 55 to 59 | T1D | Both | 6.10 (5.72, 6.50） |
| Chad | 55 to 59 | T1D | Female | 6.19 (5.64, 6.78） |
| Chad | 55 to 59 | T1D | Male | 6.00 (5.48, 6.57） |
| Chad | 60 to 64 | T1D | Both | 6.11 (5.70, 6.56） |
| Chad | 60 to 64 | T1D | Female | 6.22 (5.62, 6.88） |
| Chad | 60 to 64 | T1D | Male | 5.99 (5.43, 6.61） |
| Chad | 65 to 69 | T1D | Both | 6.07 (5.62, 6.57） |
| Chad | 65 to 69 | T1D | Female | 6.18 (5.52, 6.92） |
| Chad | 65 to 69 | T1D | Male | 5.93 (5.32, 6.61） |
| Chile | 0 to 4 | T1D | Both | 0.19 (0.18, 0.20） |
| Chile | 0 to 4 | T1D | Female | 0.18 (0.17, 0.20） |
| Chile | 0 to 4 | T1D | Male | 0.20 (0.19, 0.22） |
| Chile | 10 to 14 | T1D | Both | 1.85 (1.80, 1.91） |
| Chile | 10 to 14 | T1D | Female | 1.84 (1.78, 1.91） |
| Chile | 10 to 14 | T1D | Male | 1.86 (1.80, 1.93） |
| Chile | 15 to 19 | T1D | Both | 2.36 (2.29, 2.43） |

| | | | | |
|---|---|---|---|---|
| Chile | 15 to 19 | T1D | Female | 2.33 (2.25, 2.41） |
| Chile | 15 to 19 | T1D | Male | 2.38 (2.30, 2.46） |
| Chile | 20 to 24 | T1D | Both | 2.69 (2.61, 2.77） |
| Chile | 20 to 24 | T1D | Female | 2.66 (2.56, 2.75） |
| Chile | 20 to 24 | T1D | Male | 2.72 (2.63, 2.82） |
| Chile | 25 to 29 | T1D | Both | 3.03 (2.94, 3.13） |
| Chile | 25 to 29 | T1D | Female | 2.99 (2.88, 3.10） |
| Chile | 25 to 29 | T1D | Male | 3.08 (2.97, 3.19） |
| Chile | 30 to 34 | T1D | Both | 3.37 (3.26, 3.47） |
| Chile | 30 to 34 | T1D | Female | 3.31 (3.18, 3.44） |
| Chile | 30 to 34 | T1D | Male | 3.43 (3.30, 3.56） |
| Chile | 35 to 39 | T1D | Both | 3.71 (3.59, 3.84） |
| Chile | 35 to 39 | T1D | Female | 3.64 (3.49, 3.79） |
| Chile | 35 to 39 | T1D | Male | 3.79 (3.64, 3.94） |
| Chile | 40 to 44 | T1D | Both | 4.08 (3.94, 4.22） |
| Chile | 40 to 44 | T1D | Female | 3.98 (3.82, 4.15） |
| Chile | 40 to 44 | T1D | Male | 4.17 (4.01, 4.35） |
| Chile | 45 to 49 | T1D | Both | 4.46 (4.30, 4.62） |
| Chile | 45 to 49 | T1D | Female | 4.34 (4.16, 4.54） |
| Chile | 45 to 49 | T1D | Male | 4.58 (4.39, 4.77） |
| Chile | 5 to 9 | T1D | Both | 1.00 (1.00, 1.00） |
| Chile | 5 to 9 | T1D | Female | 1.00 (1.00, 1.00） |
| Chile | 5 to 9 | T1D | Male | 1.00 (1.00, 1.00） |
| Chile | 50 to 54 | T1D | Both | 4.85 (4.68, 5.04） |
| Chile | 50 to 54 | T1D | Female | 4.72 (4.51, 4.94） |
| Chile | 50 to 54 | T1D | Male | 5.00 (4.78, 5.22） |
| Chile | 55 to 59 | T1D | Both | 5.27 (5.07, 5.48） |
| Chile | 55 to 59 | T1D | Female | 5.12 (4.88, 5.37） |
| Chile | 55 to 59 | T1D | Male | 5.44 (5.19, 5.69） |
| Chile | 60 to 64 | T1D | Both | 5.72 (5.49, 5.96） |
| Chile | 60 to 64 | T1D | Female | 5.55 (5.27, 5.83） |
| Chile | 60 to 64 | T1D | Male | 5.91 (5.63, 6.21） |
| Chile | 65 to 69 | T1D | Both | 6.20 (5.93, 6.48） |

| | | | | |
|---|---|---|---|---|
| Chile | 65 to 69 | T1D | Female | 6.01 (5.69, 6.34） |
| Chile | 65 to 69 | T1D | Male | 6.43 (6.10, 6.78） |
| China | 0 to 4 | T1D | Both | 0.13 (0.11, 0.16） |
| China | 0 to 4 | T1D | Female | 0.13 (0.11, 0.15） |
| China | 0 to 4 | T1D | Male | 0.13 (0.11, 0.16） |
| China | 10 to 14 | T1D | Both | 2.17 (2.03, 2.31） |
| China | 10 to 14 | T1D | Female | 2.11 (1.99, 2.24） |
| China | 10 to 14 | T1D | Male | 2.21 (2.06, 2.38） |
| China | 15 to 19 | T1D | Both | 3.11 (2.92, 3.31） |
| China | 15 to 19 | T1D | Female | 2.93 (2.76, 3.10） |
| China | 15 to 19 | T1D | Male | 3.28 (3.06, 3.52） |
| China | 20 to 24 | T1D | Both | 3.96 (3.72, 4.22） |
| China | 20 to 24 | T1D | Female | 3.58 (3.38, 3.80） |
| China | 20 to 24 | T1D | Male | 4.33 (4.04, 4.65） |
| China | 25 to 29 | T1D | Both | 4.90 (4.60, 5.21） |
| China | 25 to 29 | T1D | Female | 4.25 (4.01, 4.51） |
| China | 25 to 29 | T1D | Male | 5.54 (5.16, 5.95） |
| China | 30 to 34 | T1D | Both | 5.76 (5.40, 6.14） |
| China | 30 to 34 | T1D | Female | 4.85 (4.57, 5.15） |
| China | 30 to 34 | T1D | Male | 6.68 (6.22, 7.18） |
| China | 35 to 39 | T1D | Both | 6.68 (6.26, 7.14） |
| China | 35 to 39 | T1D | Female | 5.49 (5.16, 5.83） |
| China | 35 to 39 | T1D | Male | 7.90 (7.34, 8.49） |
| China | 40 to 44 | T1D | Both | 7.68 (7.19, 8.21） |
| China | 40 to 44 | T1D | Female | 6.18 (5.81, 6.58） |
| China | 40 to 44 | T1D | Male | 9.22 (8.56, 9.93） |
| China | 45 to 49 | T1D | Both | 8.75 (8.18, 9.36） |
| China | 45 to 49 | T1D | Female | 6.94 (6.51, 7.40） |
| China | 45 to 49 | T1D | Male | 10.62 (9.85, 11.45） |
| China | 5 to 9 | T1D | Both | 1.00 (1.00, 1.00） |
| China | 5 to 9 | T1D | Female | 1.00 (1.00, 1.00） |
| China | 5 to 9 | T1D | Male | 1.00 (1.00, 1.00） |
| China | 50 to 54 | T1D | Both | 9.92 (9.25, 10.63） |

| | | | | |
|---|---|---|---|---|
| China | 50 to 54 | T1D | Female | 7.77 (7.28, 8.30） |
| China | 50 to 54 | T1D | Male | 12.16 (11.26, 13.14） |
| China | 55 to 59 | T1D | Both | 11.21 (10.43, 12.04） |
| China | 55 to 59 | T1D | Female | 8.70 (8.13, 9.31） |
| China | 55 to 59 | T1D | Male | 13.88 (12.81, 15.03） |
| China | 60 to 64 | T1D | Both | 12.58 (11.68, 13.55） |
| China | 60 to 64 | T1D | Female | 9.71 (9.05, 10.42） |
| China | 60 to 64 | T1D | Male | 15.67 (14.44, 17.01） |
| China | 65 to 69 | T1D | Both | 14.15 (13.10, 15.30） |
| China | 65 to 69 | T1D | Female | 10.91 (10.13, 11.75） |
| China | 65 to 69 | T1D | Male | 17.75 (16.29, 19.34） |
| Colombia | 0 to 4 | T1D | Both | 0.14 (0.13, 0.15） |
| Colombia | 0 to 4 | T1D | Female | 0.12 (0.11, 0.13） |
| Colombia | 0 to 4 | T1D | Male | 0.16 (0.15, 0.17） |
| Colombia | 10 to 14 | T1D | Both | 2.16 (2.10, 2.21） |
| Colombia | 10 to 14 | T1D | Female | 2.33 (2.25, 2.41） |
| Colombia | 10 to 14 | T1D | Male | 1.98 (1.91, 2.05） |
| Colombia | 15 to 19 | T1D | Both | 2.99 (2.91, 3.06） |
| Colombia | 15 to 19 | T1D | Female | 3.14 (3.03, 3.25） |
| Colombia | 15 to 19 | T1D | Male | 2.83 (2.73, 2.93） |
| Colombia | 20 to 24 | T1D | Both | 3.60 (3.51, 3.70） |
| Colombia | 20 to 24 | T1D | Female | 3.63 (3.51, 3.77） |
| Colombia | 20 to 24 | T1D | Male | 3.56 (3.43, 3.70） |
| Colombia | 25 to 29 | T1D | Both | 4.22 (4.11, 4.33） |
| Colombia | 25 to 29 | T1D | Female | 4.10 (3.96, 4.26） |
| Colombia | 25 to 29 | T1D | Male | 4.34 (4.18, 4.51） |
| Colombia | 30 to 34 | T1D | Both | 4.76 (4.63, 4.89） |
| Colombia | 30 to 34 | T1D | Female | 4.51 (4.34, 4.68） |
| Colombia | 30 to 34 | T1D | Male | 5.04 (4.84, 5.24） |
| Colombia | 35 to 39 | T1D | Both | 5.27 (5.12, 5.42） |
| Colombia | 35 to 39 | T1D | Female | 4.90 (4.71, 5.10） |
| Colombia | 35 to 39 | T1D | Male | 5.69 (5.47, 5.93） |
| Colombia | 40 to 44 | T1D | Both | 5.78 (5.62, 5.95） |

| | | | | |
|---|---|---|---|---|
| Colombia | 40 to 44 | T1D | Female | 5.30 (5.09, 5.52） |
| Colombia | 40 to 44 | T1D | Male | 6.34 (6.08, 6.61） |
| Colombia | 45 to 49 | T1D | Both | 6.29 (6.11, 6.48） |
| Colombia | 45 to 49 | T1D | Female | 5.70 (5.47, 5.95） |
| Colombia | 45 to 49 | T1D | Male | 6.97 (6.68, 7.28） |
| Colombia | 5 to 9 | T1D | Both | 1.00 (1.00, 1.00） |
| Colombia | 5 to 9 | T1D | Female | 1.00 (1.00, 1.00） |
| Colombia | 5 to 9 | T1D | Male | 1.00 (1.00, 1.00） |
| Colombia | 50 to 54 | T1D | Both | 6.80 (6.59, 7.01） |
| Colombia | 50 to 54 | T1D | Female | 6.11 (5.85, 6.39） |
| Colombia | 50 to 54 | T1D | Male | 7.60 (7.27, 7.95） |
| Colombia | 55 to 59 | T1D | Both | 7.28 (7.05, 7.53） |
| Colombia | 55 to 59 | T1D | Female | 6.53 (6.24, 6.84） |
| Colombia | 55 to 59 | T1D | Male | 8.19 (7.82, 8.58） |
| Colombia | 60 to 64 | T1D | Both | 7.77 (7.50, 8.04） |
| Colombia | 60 to 64 | T1D | Female | 6.96 (6.62, 7.30） |
| Colombia | 60 to 64 | T1D | Male | 8.77 (8.34, 9.21） |
| Colombia | 65 to 69 | T1D | Both | 8.27 (7.97, 8.58） |
| Colombia | 65 to 69 | T1D | Female | 7.42 (7.04, 7.81） |
| Colombia | 65 to 69 | T1D | Male | 9.36 (8.88, 9.86） |
| Comoros | 0 to 4 | T1D | Both | 0.16 (0.11, 0.23） |
| Comoros | 0 to 4 | T1D | Female | 0.16 (0.10, 0.27） |
| Comoros | 0 to 4 | T1D | Male | 0.16 (0.10, 0.26） |
| Comoros | 10 to 14 | T1D | Both | 2.05 (1.74, 2.42） |
| Comoros | 10 to 14 | T1D | Female | 2.04 (1.61, 2.59） |
| Comoros | 10 to 14 | T1D | Male | 2.06 (1.64, 2.59） |
| Comoros | 15 to 19 | T1D | Both | 3.03 (2.57, 3.57） |
| Comoros | 15 to 19 | T1D | Female | 3.01 (2.38, 3.82） |
| Comoros | 15 to 19 | T1D | Male | 3.05 (2.42, 3.84） |
| Comoros | 20 to 24 | T1D | Both | 3.95 (3.34, 4.68） |
| Comoros | 20 to 24 | T1D | Female | 3.94 (3.09, 5.02） |
| Comoros | 20 to 24 | T1D | Male | 3.98 (3.15, 5.04） |
| Comoros | 25 to 29 | T1D | Both | 4.90 (4.12, 5.82） |

| | | | | |
|---|---|---|---|---|
| Comoros | 25 to 29 | T1D | Female | 4.90 (3.83, 6.28） |
| Comoros | 25 to 29 | T1D | Male | 4.90 (3.85, 6.24） |
| Comoros | 30 to 34 | T1D | Both | 5.54 (4.63, 6.63） |
| Comoros | 30 to 34 | T1D | Female | 5.55 (4.29, 7.17） |
| Comoros | 30 to 34 | T1D | Male | 5.55 (4.32, 7.13） |
| Comoros | 35 to 39 | T1D | Both | 6.06 (5.03, 7.30） |
| Comoros | 35 to 39 | T1D | Female | 6.08 (4.65, 7.94） |
| Comoros | 35 to 39 | T1D | Male | 6.02 (4.64, 7.82） |
| Comoros | 40 to 44 | T1D | Both | 6.49 (5.35, 7.89） |
| Comoros | 40 to 44 | T1D | Female | 6.53 (4.95, 8.63） |
| Comoros | 40 to 44 | T1D | Male | 6.48 (4.94, 8.51） |
| Comoros | 45 to 49 | T1D | Both | 6.92 (5.64, 8.49） |
| Comoros | 45 to 49 | T1D | Female | 7.00 (5.23, 9.37） |
| Comoros | 45 to 49 | T1D | Male | 6.86 (5.15, 9.14） |
| Comoros | 5 to 9 | T1D | Both | 1.00 (1.00, 1.00） |
| Comoros | 5 to 9 | T1D | Female | 1.00 (1.00, 1.00） |
| Comoros | 5 to 9 | T1D | Male | 1.00 (1.00, 1.00） |
| Comoros | 50 to 54 | T1D | Both | 7.26 (5.85, 9.01） |
| Comoros | 50 to 54 | T1D | Female | 7.34 (5.41, 9.98） |
| Comoros | 50 to 54 | T1D | Male | 7.20 (5.32, 9.76） |
| Comoros | 55 to 59 | T1D | Both | 7.62 (6.06, 9.57） |
| Comoros | 55 to 59 | T1D | Female | 7.67 (5.55, 10.60） |
| Comoros | 55 to 59 | T1D | Male | 7.46 (5.39, 10.31） |
| Comoros | 60 to 64 | T1D | Both | 7.90 (6.19, 10.09） |
| Comoros | 60 to 64 | T1D | Female | 8.03 (5.69, 11.32） |
| Comoros | 60 to 64 | T1D | Male | 7.76 (5.48, 10.99） |
| Comoros | 65 to 69 | T1D | Both | 8.24 (6.32, 10.73） |
| Comoros | 65 to 69 | T1D | Female | 8.34 (5.76, 12.09） |
| Comoros | 65 to 69 | T1D | Male | 8.05 (5.51, 11.75） |
| Congo | 0 to 4 | T1D | Both | 0.16 (0.14, 0.18） |
| Congo | 0 to 4 | T1D | Female | 0.16 (0.13, 0.20） |
| Congo | 0 to 4 | T1D | Male | 0.16 (0.13, 0.19） |
| Congo | 10 to 14 | T1D | Both | 2.09 (1.95, 2.23） |

| | | | | |
|---|---|---|---|---|
| Congo | 10 to 14 | T1D | Female | 2.08 (1.90, 2.29） |
| Congo | 10 to 14 | T1D | Male | 2.09 (1.91, 2.29） |
| Congo | 15 to 19 | T1D | Both | 3.09 (2.90, 3.31） |
| Congo | 15 to 19 | T1D | Female | 3.10 (2.82, 3.40） |
| Congo | 15 to 19 | T1D | Male | 3.10 (2.82, 3.40） |
| Congo | 20 to 24 | T1D | Both | 3.96 (3.70, 4.24） |
| Congo | 20 to 24 | T1D | Female | 3.97 (3.60, 4.37） |
| Congo | 20 to 24 | T1D | Male | 3.96 (3.61, 4.36） |
| Congo | 25 to 29 | T1D | Both | 4.87 (4.55, 5.22） |
| Congo | 25 to 29 | T1D | Female | 4.89 (4.43, 5.40） |
| Congo | 25 to 29 | T1D | Male | 4.86 (4.41, 5.36） |
| Congo | 30 to 34 | T1D | Both | 5.56 (5.18, 5.98） |
| Congo | 30 to 34 | T1D | Female | 5.60 (5.06, 6.20） |
| Congo | 30 to 34 | T1D | Male | 5.53 (5.00, 6.11） |
| Congo | 35 to 39 | T1D | Both | 6.11 (5.68, 6.59） |
| Congo | 35 to 39 | T1D | Female | 6.17 (5.55, 6.86） |
| Congo | 35 to 39 | T1D | Male | 6.06 (5.46, 6.73） |
| Congo | 40 to 44 | T1D | Both | 6.59 (6.10, 7.13） |
| Congo | 40 to 44 | T1D | Female | 6.68 (5.98, 7.46） |
| Congo | 40 to 44 | T1D | Male | 6.51 (5.84, 7.26） |
| Congo | 45 to 49 | T1D | Both | 7.06 (6.51, 7.67） |
| Congo | 45 to 49 | T1D | Female | 7.18 (6.39, 8.08） |
| Congo | 45 to 49 | T1D | Male | 6.95 (6.20, 7.79） |
| Congo | 5 to 9 | T1D | Both | 1.00 (1.00, 1.00） |
| Congo | 5 to 9 | T1D | Female | 1.00 (1.00, 1.00） |
| Congo | 5 to 9 | T1D | Male | 1.00 (1.00, 1.00） |
| Congo | 50 to 54 | T1D | Both | 7.54 (6.91, 8.23） |
| Congo | 50 to 54 | T1D | Female | 7.68 (6.78, 8.70） |
| Congo | 50 to 54 | T1D | Male | 7.40 (6.55, 8.36） |
| Congo | 55 to 59 | T1D | Both | 8.01 (7.30, 8.79） |
| Congo | 55 to 59 | T1D | Female | 8.19 (7.18, 9.35） |
| Congo | 55 to 59 | T1D | Male | 7.83 (6.87, 8.93） |
| Congo | 60 to 64 | T1D | Both | 8.44 (7.63, 9.33） |

| | | | | |
|---|---|---|---|---|
| Congo | 60 to 64 | T1D | Female | 8.65 (7.51, 9.97） |
| Congo | 60 to 64 | T1D | Male | 8.22 (7.13, 9.49） |
| Congo | 65 to 69 | T1D | Both | 8.83 (7.90, 9.86） |
| Congo | 65 to 69 | T1D | Female | 9.07 (7.77, 10.59） |
| Congo | 65 to 69 | T1D | Male | 8.57 (7.31, 10.05） |
| Cook Islands | 0 to 4 | T1D | Both | 0.17 (0.01, 4.28） |
| Cook Islands | 0 to 4 | T1D | Female | 0.90 (0.02, 39.05） |
| Cook Islands | 0 to 4 | T1D | Male | 0.91 (0.02, 39.20） |
| Cook Islands | 10 to 14 | T1D | Both | 1.75 (0.36, 8.63） |
| Cook Islands | 10 to 14 | T1D | Female | 1.14 (0.03, 44.94） |
| Cook Islands | 10 to 14 | T1D | Male | 1.13 (0.03, 44.84） |
| Cook Islands | 15 to 19 | T1D | Both | 1.86 (0.38, 9.05） |
| Cook Islands | 15 to 19 | T1D | Female | 12.78 (0.69, 235.66） |
| Cook Islands | 15 to 19 | T1D | Male | 13.40 (0.73, 246.88） |
| Cook Islands | 20 to 24 | T1D | Both | 2.02 (0.41, 9.90） |
| Cook Islands | 20 to 24 | T1D | Female | 14.26 (0.74, 276.25） |
| Cook Islands | 20 to 24 | T1D | Male | 16.57 (0.86, 320.78） |
| Cook Islands | 25 to 29 | T1D | Both | 2.57 (0.52, 12.76） |
| Cook Islands | 25 to 29 | T1D | Female | 16.04 (0.79, 323.75） |
| Cook Islands | 25 to 29 | T1D | Male | 19.58 (0.97, 394.90） |
| Cook Islands | 30 to 34 | T1D | Both | 3.63 (0.70, 18.83） |
| Cook Islands | 30 to 34 | T1D | Female | 17.85 (0.85, 374.87） |
| Cook Islands | 30 to 34 | T1D | Male | 22.56 (1.08, 473.31） |
| Cook Islands | 35 to 39 | T1D | Both | 4.50 (0.83, 24.37） |
| Cook Islands | 35 to 39 | T1D | Female | 19.08 (0.87, 415.99） |
| Cook Islands | 35 to 39 | T1D | Male | 24.47 (1.12, 532.65） |
| Cook Islands | 40 to 44 | T1D | Both | 4.68 (0.84, 26.11） |
| Cook Islands | 40 to 44 | T1D | Female | 19.75 (0.88, 444.78） |
| Cook Islands | 40 to 44 | T1D | Male | 25.32 (1.13, 568.77） |
| Cook Islands | 45 to 49 | T1D | Both | 4.93 (0.84, 28.77） |
| Cook Islands | 45 to 49 | T1D | Female | 19.55 (0.83, 459.15） |
| Cook Islands | 45 to 49 | T1D | Male | 26.34 (1.13, 613.78） |
| Cook Islands | 5 to 9 | T1D | Both | 1.00 (1.00, 1.00） |

| | | | | |
|---|---|---|---|---|
| Cook Islands | 5 to 9 | T1D | Female | 1.00 (1.00, 1.00） |
| Cook Islands | 5 to 9 | T1D | Male | 1.00 (1.00, 1.00） |
| Cook Islands | 50 to 54 | T1D | Both | 5.89 (0.96, 36.13） |
| Cook Islands | 50 to 54 | T1D | Female | 18.95 (0.76, 470.30） |
| Cook Islands | 50 to 54 | T1D | Male | 26.56 (1.10, 641.72） |
| Cook Islands | 55 to 59 | T1D | Both | 6.34 (0.97, 41.31） |
| Cook Islands | 55 to 59 | T1D | Female | 21.72 (0.82, 574.03） |
| Cook Islands | 55 to 59 | T1D | Male | 27.95 (1.11, 703.34） |
| Cook Islands | 60 to 64 | T1D | Both | 7.22 (1.03, 50.42） |
| Cook Islands | 60 to 64 | T1D | Female | 30.79 (1.09, 870.03） |
| Cook Islands | 60 to 64 | T1D | Male | 31.00 (1.17, 823.18） |
| Cook Islands | 65 to 69 | T1D | Both | 7.10 (0.92, 54.80） |
| Cook Islands | 65 to 69 | T1D | Female | 26.97 (0.81, 896.60） |
| Cook Islands | 65 to 69 | T1D | Male | 36.75 (1.28, 1054.23） |
| Costa Rica | 0 to 4 | T1D | Both | 0.13 (0.11, 0.16） |
| Costa Rica | 0 to 4 | T1D | Female | 0.12 (0.09, 0.16） |
| Costa Rica | 0 to 4 | T1D | Male | 0.15 (0.11, 0.19） |
| Costa Rica | 10 to 14 | T1D | Both | 2.18 (2.01, 2.37） |
| Costa Rica | 10 to 14 | T1D | Female | 2.32 (2.08, 2.60） |
| Costa Rica | 10 to 14 | T1D | Male | 2.01 (1.78, 2.27） |
| Costa Rica | 15 to 19 | T1D | Both | 2.98 (2.74, 3.24） |
| Costa Rica | 15 to 19 | T1D | Female | 3.14 (2.80, 3.52） |
| Costa Rica | 15 to 19 | T1D | Male | 2.79 (2.46, 3.15） |
| Costa Rica | 20 to 24 | T1D | Both | 3.55 (3.26, 3.86） |
| Costa Rica | 20 to 24 | T1D | Female | 3.63 (3.23, 4.08） |
| Costa Rica | 20 to 24 | T1D | Male | 3.42 (3.02, 3.88） |
| Costa Rica | 25 to 29 | T1D | Both | 4.10 (3.76, 4.47） |
| Costa Rica | 25 to 29 | T1D | Female | 4.08 (3.62, 4.59） |
| Costa Rica | 25 to 29 | T1D | Male | 4.09 (3.60, 4.65） |
| Costa Rica | 30 to 34 | T1D | Both | 4.56 (4.17, 4.99） |
| Costa Rica | 30 to 34 | T1D | Female | 4.44 (3.93, 5.03） |
| Costa Rica | 30 to 34 | T1D | Male | 4.65 (4.08, 5.31） |
| Costa Rica | 35 to 39 | T1D | Both | 4.97 (4.53, 5.46） |

| | | | | |
|---|---|---|---|---|
| Costa Rica | 35 to 39 | T1D | Female | 4.77 (4.20, 5.42） |
| Costa Rica | 35 to 39 | T1D | Male | 5.16 (4.50, 5.91） |
| Costa Rica | 40 to 44 | T1D | Both | 5.36 (4.86, 5.90） |
| Costa Rica | 40 to 44 | T1D | Female | 5.09 (4.46, 5.81） |
| Costa Rica | 40 to 44 | T1D | Male | 5.63 (4.89, 6.48） |
| Costa Rica | 45 to 49 | T1D | Both | 5.73 (5.18, 6.33） |
| Costa Rica | 45 to 49 | T1D | Female | 5.39 (4.70, 6.19） |
| Costa Rica | 45 to 49 | T1D | Male | 6.06 (5.24, 7.02） |
| Costa Rica | 5 to 9 | T1D | Both | 1.00 (1.00, 1.00） |
| Costa Rica | 5 to 9 | T1D | Female | 1.00 (1.00, 1.00） |
| Costa Rica | 5 to 9 | T1D | Male | 1.00 (1.00, 1.00） |
| Costa Rica | 50 to 54 | T1D | Both | 6.07 (5.47, 6.74） |
| Costa Rica | 50 to 54 | T1D | Female | 5.69 (4.93, 6.56） |
| Costa Rica | 50 to 54 | T1D | Male | 6.47 (5.56, 7.53） |
| Costa Rica | 55 to 59 | T1D | Both | 6.40 (5.74, 7.14） |
| Costa Rica | 55 to 59 | T1D | Female | 5.98 (5.15, 6.95） |
| Costa Rica | 55 to 59 | T1D | Male | 6.84 (5.83, 8.02） |
| Costa Rica | 60 to 64 | T1D | Both | 6.69 (5.96, 7.52） |
| Costa Rica | 60 to 64 | T1D | Female | 6.24 (5.32, 7.32） |
| Costa Rica | 60 to 64 | T1D | Male | 7.18 (6.06, 8.50） |
| Costa Rica | 65 to 69 | T1D | Both | 6.99 (6.17, 7.92） |
| Costa Rica | 65 to 69 | T1D | Female | 6.51 (5.49, 7.74） |
| Costa Rica | 65 to 69 | T1D | Male | 7.51 (6.26, 9.00） |
| Coted'Ivoire | 0 to 4 | T1D | Both | 0.15 (0.14, 0.16） |
| Coted'Ivoire | 0 to 4 | T1D | Female | 0.15 (0.14, 0.17） |
| Coted'Ivoire | 0 to 4 | T1D | Male | 0.15 (0.14, 0.17） |
| Coted'Ivoire | 10 to 14 | T1D | Both | 2.09 (2.03, 2.15） |
| Coted'Ivoire | 10 to 14 | T1D | Female | 2.10 (2.01, 2.19） |
| Coted'Ivoire | 10 to 14 | T1D | Male | 2.09 (2.00, 2.17） |
| Coted'Ivoire | 15 to 19 | T1D | Both | 3.05 (2.96, 3.14） |
| Coted'Ivoire | 15 to 19 | T1D | Female | 3.06 (2.93, 3.19） |
| Coted'Ivoire | 15 to 19 | T1D | Male | 3.04 (2.91, 3.16） |
| Coted'Ivoire | 20 to 24 | T1D | Both | 3.87 (3.75, 3.99） |

| | | | | |
|---|---|---|---|---|
| Coted'Ivoire | 20 to 24 | T1D | Female | 3.89 (3.72, 4.06） |
| Coted'Ivoire | 20 to 24 | T1D | Male | 3.85 (3.69, 4.02） |
| Coted'Ivoire | 25 to 29 | T1D | Both | 4.71 (4.56, 4.86） |
| Coted'Ivoire | 25 to 29 | T1D | Female | 4.74 (4.53, 4.96） |
| Coted'Ivoire | 25 to 29 | T1D | Male | 4.68 (4.48, 4.89） |
| Coted'Ivoire | 30 to 34 | T1D | Both | 5.24 (5.07, 5.41） |
| Coted'Ivoire | 30 to 34 | T1D | Female | 5.28 (5.04, 5.54） |
| Coted'Ivoire | 30 to 34 | T1D | Male | 5.20 (4.97, 5.44） |
| Coted'Ivoire | 35 to 39 | T1D | Both | 5.62 (5.43, 5.82） |
| Coted'Ivoire | 35 to 39 | T1D | Female | 5.68 (5.40, 5.97） |
| Coted'Ivoire | 35 to 39 | T1D | Male | 5.57 (5.31, 5.84） |
| Coted'Ivoire | 40 to 44 | T1D | Both | 5.94 (5.73, 6.16） |
| Coted'Ivoire | 40 to 44 | T1D | Female | 6.00 (5.69, 6.32） |
| Coted'Ivoire | 40 to 44 | T1D | Male | 5.87 (5.58, 6.17） |
| Coted'Ivoire | 45 to 49 | T1D | Both | 6.19 (5.96, 6.43） |
| Coted'Ivoire | 45 to 49 | T1D | Female | 6.26 (5.92, 6.62） |
| Coted'Ivoire | 45 to 49 | T1D | Male | 6.11 (5.79, 6.44） |
| Coted'Ivoire | 5 to 9 | T1D | Both | 1.00 (1.00, 1.00） |
| Coted'Ivoire | 5 to 9 | T1D | Female | 1.00 (1.00, 1.00） |
| Coted'Ivoire | 5 to 9 | T1D | Male | 1.00 (1.00, 1.00） |
| Coted'Ivoire | 50 to 54 | T1D | Both | 6.39 (6.13, 6.66） |
| Coted'Ivoire | 50 to 54 | T1D | Female | 6.48 (6.10, 6.88） |
| Coted'Ivoire | 50 to 54 | T1D | Male | 6.29 (5.94, 6.65） |
| Coted'Ivoire | 55 to 59 | T1D | Both | 6.53 (6.25, 6.83） |
| Coted'Ivoire | 55 to 59 | T1D | Female | 6.64 (6.22, 7.09） |
| Coted'Ivoire | 55 to 59 | T1D | Male | 6.42 (6.04, 6.82） |
| Coted'Ivoire | 60 to 64 | T1D | Both | 6.63 (6.31, 6.96） |
| Coted'Ivoire | 60 to 64 | T1D | Female | 6.75 (6.29, 7.25） |
| Coted'Ivoire | 60 to 64 | T1D | Male | 6.50 (6.08, 6.95） |
| Coted'Ivoire | 65 to 69 | T1D | Both | 6.68 (6.32, 7.06） |
| Coted'Ivoire | 65 to 69 | T1D | Female | 6.83 (6.30, 7.41） |
| Coted'Ivoire | 65 to 69 | T1D | Male | 6.54 (6.06, 7.05） |
| Croatia | 0 to 4 | T1D | Both | 0.21 (0.18, 0.24） |

| | | | | |
|---|---|---|---|---|
| Croatia | 0 to 4 | T1D | Female | 0.20 (0.17, 0.25） |
| Croatia | 0 to 4 | T1D | Male | 0.21 (0.17, 0.26） |
| Croatia | 10 to 14 | T1D | Both | 2.17 (2.01, 2.34） |
| Croatia | 10 to 14 | T1D | Female | 2.13 (1.92, 2.36） |
| Croatia | 10 to 14 | T1D | Male | 2.22 (1.99, 2.47） |
| Croatia | 15 to 19 | T1D | Both | 3.64 (3.39, 3.92） |
| Croatia | 15 to 19 | T1D | Female | 3.54 (3.20, 3.92） |
| Croatia | 15 to 19 | T1D | Male | 3.76 (3.38, 4.17） |
| Croatia | 20 to 24 | T1D | Both | 5.10 (4.74, 5.48） |
| Croatia | 20 to 24 | T1D | Female | 4.91 (4.44, 5.44） |
| Croatia | 20 to 24 | T1D | Male | 5.30 (4.77, 5.89） |
| Croatia | 25 to 29 | T1D | Both | 6.72 (6.24, 7.23） |
| Croatia | 25 to 29 | T1D | Female | 6.38 (5.75, 7.07） |
| Croatia | 25 to 29 | T1D | Male | 7.09 (6.38, 7.88） |
| Croatia | 30 to 34 | T1D | Both | 8.59 (7.97, 9.25） |
| Croatia | 30 to 34 | T1D | Female | 8.06 (7.26, 8.95） |
| Croatia | 30 to 34 | T1D | Male | 9.17 (8.23, 10.20） |
| Croatia | 35 to 39 | T1D | Both | 10.76 (9.97, 11.61） |
| Croatia | 35 to 39 | T1D | Female | 10.01 (9.00, 11.14） |
| Croatia | 35 to 39 | T1D | Male | 11.57 (10.38, 12.91） |
| Croatia | 40 to 44 | T1D | Both | 13.30 (12.31, 14.36） |
| Croatia | 40 to 44 | T1D | Female | 12.31 (11.05, 13.71） |
| Croatia | 40 to 44 | T1D | Male | 14.39 (12.89, 16.07） |
| Croatia | 45 to 49 | T1D | Both | 16.27 (15.05, 17.59） |
| Croatia | 45 to 49 | T1D | Female | 15.01 (13.46, 16.75） |
| Croatia | 45 to 49 | T1D | Male | 17.65 (15.78, 19.73） |
| Croatia | 5 to 9 | T1D | Both | 1.00 (1.00, 1.00） |
| Croatia | 5 to 9 | T1D | Female | 1.00 (1.00, 1.00） |
| Croatia | 5 to 9 | T1D | Male | 1.00 (1.00, 1.00） |
| Croatia | 50 to 54 | T1D | Both | 19.71 (18.21, 21.33） |
| Croatia | 50 to 54 | T1D | Female | 18.18 (16.28, 20.31） |
| Croatia | 50 to 54 | T1D | Male | 21.41 (19.12, 23.97） |
| Croatia | 55 to 59 | T1D | Both | 23.74 (21.91, 25.72） |

| | | | | |
|---|---|---|---|---|
| Croatia | 55 to 59 | T1D | Female | 21.93 (19.60, 24.54） |
| Croatia | 55 to 59 | T1D | Male | 25.76 (22.97, 28.89） |
| Croatia | 60 to 64 | T1D | Both | 28.41 (26.19, 30.82） |
| Croatia | 60 to 64 | T1D | Female | 26.34 (23.51, 29.52） |
| Croatia | 60 to 64 | T1D | Male | 30.76 (27.38, 34.56） |
| Croatia | 65 to 69 | T1D | Both | 33.93 (31.23, 36.86） |
| Croatia | 65 to 69 | T1D | Female | 31.60 (28.15, 35.47） |
| Croatia | 65 to 69 | T1D | Male | 36.60 (32.50, 41.21） |
| Cuba | 0 to 4 | T1D | Both | 0.13 (0.12, 0.16） |
| Cuba | 0 to 4 | T1D | Female | 0.13 (0.10, 0.16） |
| Cuba | 0 to 4 | T1D | Male | 0.14 (0.12, 0.18） |
| Cuba | 10 to 14 | T1D | Both | 2.31 (2.17, 2.45） |
| Cuba | 10 to 14 | T1D | Female | 2.46 (2.26, 2.67） |
| Cuba | 10 to 14 | T1D | Male | 2.15 (1.97, 2.34） |
| Cuba | 15 to 19 | T1D | Both | 3.34 (3.15, 3.55） |
| Cuba | 15 to 19 | T1D | Female | 3.51 (3.24, 3.82） |
| Cuba | 15 to 19 | T1D | Male | 3.17 (2.91, 3.45） |
| Cuba | 20 to 24 | T1D | Both | 4.14 (3.90, 4.40） |
| Cuba | 20 to 24 | T1D | Female | 4.20 (3.87, 4.57） |
| Cuba | 20 to 24 | T1D | Male | 4.08 (3.74, 4.44） |
| Cuba | 25 to 29 | T1D | Both | 4.96 (4.67, 5.26） |
| Cuba | 25 to 29 | T1D | Female | 4.86 (4.46, 5.29） |
| Cuba | 25 to 29 | T1D | Male | 5.05 (4.64, 5.50） |
| Cuba | 30 to 34 | T1D | Both | 5.70 (5.36, 6.06） |
| Cuba | 30 to 34 | T1D | Female | 5.45 (5.00, 5.94） |
| Cuba | 30 to 34 | T1D | Male | 5.96 (5.46, 6.50） |
| Cuba | 35 to 39 | T1D | Both | 6.47 (6.08, 6.88） |
| Cuba | 35 to 39 | T1D | Female | 6.06 (5.55, 6.61） |
| Cuba | 35 to 39 | T1D | Male | 6.89 (6.31, 7.53） |
| Cuba | 40 to 44 | T1D | Both | 7.28 (6.83, 7.75） |
| Cuba | 40 to 44 | T1D | Female | 6.71 (6.14, 7.33） |
| Cuba | 40 to 44 | T1D | Male | 7.88 (7.20, 8.62） |
| Cuba | 45 to 49 | T1D | Both | 8.12 (7.61, 8.66） |

| | | | | |
|---|---|---|---|---|
| Cuba | 45 to 49 | T1D | Female | 7.39 (6.75, 8.09） |
| Cuba | 45 to 49 | T1D | Male | 8.90 (8.12, 9.75） |
| Cuba | 5 to 9 | T1D | Both | 1.00 (1.00, 1.00） |
| Cuba | 5 to 9 | T1D | Female | 1.00 (1.00, 1.00） |
| Cuba | 5 to 9 | T1D | Male | 1.00 (1.00, 1.00） |
| Cuba | 50 to 54 | T1D | Both | 8.99 (8.42, 9.60） |
| Cuba | 50 to 54 | T1D | Female | 8.11 (7.39, 8.89） |
| Cuba | 50 to 54 | T1D | Male | 9.96 (9.07, 10.94） |
| Cuba | 55 to 59 | T1D | Both | 9.92 (9.27, 10.61） |
| Cuba | 55 to 59 | T1D | Female | 8.87 (8.07, 9.76） |
| Cuba | 55 to 59 | T1D | Male | 11.08 (10.06, 12.20） |
| Cuba | 60 to 64 | T1D | Both | 10.91 (10.17, 11.70） |
| Cuba | 60 to 64 | T1D | Female | 9.71 (8.80, 10.71） |
| Cuba | 60 to 64 | T1D | Male | 12.26 (11.10, 13.54） |
| Cuba | 65 to 69 | T1D | Both | 12.00 (11.16, 12.90） |
| Cuba | 65 to 69 | T1D | Female | 10.63 (9.60, 11.76） |
| Cuba | 65 to 69 | T1D | Male | 13.54 (12.22, 15.01） |
| Cyprus | 0 to 4 | T1D | Both | 0.19 (0.15, 0.24） |
| Cyprus | 0 to 4 | T1D | Female | 0.19 (0.14, 0.27） |
| Cyprus | 0 to 4 | T1D | Male | 0.19 (0.13, 0.26） |
| Cyprus | 10 to 14 | T1D | Both | 2.11 (1.88, 2.37） |
| Cyprus | 10 to 14 | T1D | Female | 2.08 (1.77, 2.44） |
| Cyprus | 10 to 14 | T1D | Male | 2.14 (1.82, 2.52） |
| Cyprus | 15 to 19 | T1D | Both | 3.26 (2.91, 3.65） |
| Cyprus | 15 to 19 | T1D | Female | 3.21 (2.73, 3.76） |
| Cyprus | 15 to 19 | T1D | Male | 3.31 (2.81, 3.88） |
| Cyprus | 20 to 24 | T1D | Both | 4.37 (3.90, 4.90） |
| Cyprus | 20 to 24 | T1D | Female | 4.29 (3.65, 5.03） |
| Cyprus | 20 to 24 | T1D | Male | 4.44 (3.78, 5.22） |
| Cyprus | 25 to 29 | T1D | Both | 5.69 (5.07, 6.39） |
| Cyprus | 25 to 29 | T1D | Female | 5.51 (4.68, 6.48） |
| Cyprus | 25 to 29 | T1D | Male | 5.84 (4.96, 6.88） |
| Cyprus | 30 to 34 | T1D | Both | 7.19 (6.39, 8.10） |

| | | | | |
|---|---|---|---|---|
| Cyprus | 30 to 34 | T1D | Female | 6.88 (5.83, 8.12） |
| Cyprus | 30 to 34 | T1D | Male | 7.47 (6.32, 8.83） |
| Cyprus | 35 to 39 | T1D | Both | 8.94 (7.92, 10.09） |
| Cyprus | 35 to 39 | T1D | Female | 8.45 (7.13, 10.02） |
| Cyprus | 35 to 39 | T1D | Male | 9.38 (7.90, 11.15） |
| Cyprus | 40 to 44 | T1D | Both | 11.00 (9.71, 12.45） |
| Cyprus | 40 to 44 | T1D | Female | 10.30 (8.65, 12.26） |
| Cyprus | 40 to 44 | T1D | Male | 11.69 (9.79, 13.95） |
| Cyprus | 45 to 49 | T1D | Both | 13.41 (11.81, 15.23） |
| Cyprus | 45 to 49 | T1D | Female | 12.43 (10.40, 14.85） |
| Cyprus | 45 to 49 | T1D | Male | 14.42 (12.03, 17.28） |
| Cyprus | 5 to 9 | T1D | Both | 1.00 (1.00, 1.00） |
| Cyprus | 5 to 9 | T1D | Female | 1.00 (1.00, 1.00） |
| Cyprus | 5 to 9 | T1D | Male | 1.00 (1.00, 1.00） |
| Cyprus | 50 to 54 | T1D | Both | 16.25 (14.26, 18.50） |
| Cyprus | 50 to 54 | T1D | Female | 14.89 (12.40, 17.88） |
| Cyprus | 50 to 54 | T1D | Male | 17.63 (14.65, 21.22） |
| Cyprus | 55 to 59 | T1D | Both | 19.54 (17.09, 22.33） |
| Cyprus | 55 to 59 | T1D | Female | 17.75 (14.71, 21.41） |
| Cyprus | 55 to 59 | T1D | Male | 21.46 (17.74, 25.96） |
| Cyprus | 60 to 64 | T1D | Both | 23.38 (20.38, 26.82） |
| Cyprus | 60 to 64 | T1D | Female | 21.03 (17.34, 25.49） |
| Cyprus | 60 to 64 | T1D | Male | 25.90 (21.30, 31.49） |
| Cyprus | 65 to 69 | T1D | Both | 27.86 (24.19, 32.09） |
| Cyprus | 65 to 69 | T1D | Female | 24.87 (20.40, 30.31） |
| Cyprus | 65 to 69 | T1D | Male | 31.14 (25.45, 38.10） |
| Czechia | 0 to 4 | T1D | Both | 0.22 (0.20, 0.24） |
| Czechia | 0 to 4 | T1D | Female | 0.21 (0.19, 0.25） |
| Czechia | 0 to 4 | T1D | Male | 0.22 (0.19, 0.25） |
| Czechia | 10 to 14 | T1D | Both | 2.15 (2.05, 2.26） |
| Czechia | 10 to 14 | T1D | Female | 2.13 (1.99, 2.28） |
| Czechia | 10 to 14 | T1D | Male | 2.18 (2.03, 2.34） |
| Czechia | 15 to 19 | T1D | Both | 3.23 (3.08, 3.39） |

| | | | | |
|---|---|---|---|---|
| Czechia | 15 to 19 | T1D | Female | 3.11 (2.90, 3.33） |
| Czechia | 15 to 19 | T1D | Male | 3.36 (3.14, 3.61） |
| Czechia | 20 to 24 | T1D | Both | 4.18 (3.98, 4.39） |
| Czechia | 20 to 24 | T1D | Female | 3.91 (3.65, 4.18） |
| Czechia | 20 to 24 | T1D | Male | 4.47 (4.17, 4.80） |
| Czechia | 25 to 29 | T1D | Both | 5.26 (5.01, 5.52） |
| Czechia | 25 to 29 | T1D | Female | 4.78 (4.46, 5.12） |
| Czechia | 25 to 29 | T1D | Male | 5.78 (5.39, 6.20） |
| Czechia | 30 to 34 | T1D | Both | 6.31 (6.00, 6.63） |
| Czechia | 30 to 34 | T1D | Female | 5.60 (5.23, 6.01） |
| Czechia | 30 to 34 | T1D | Male | 7.07 (6.59, 7.59） |
| Czechia | 35 to 39 | T1D | Both | 7.42 (7.06, 7.80） |
| Czechia | 35 to 39 | T1D | Female | 6.48 (6.04, 6.96） |
| Czechia | 35 to 39 | T1D | Male | 8.45 (7.87, 9.08） |
| Czechia | 40 to 44 | T1D | Both | 8.64 (8.21, 9.09） |
| Czechia | 40 to 44 | T1D | Female | 7.45 (6.93, 8.00） |
| Czechia | 40 to 44 | T1D | Male | 9.95 (9.26, 10.70） |
| Czechia | 45 to 49 | T1D | Both | 9.95 (9.45, 10.48） |
| Czechia | 45 to 49 | T1D | Female | 8.50 (7.90, 9.14） |
| Czechia | 45 to 49 | T1D | Male | 11.56 (10.74, 12.44） |
| Czechia | 5 to 9 | T1D | Both | 1.00 (1.00, 1.00） |
| Czechia | 5 to 9 | T1D | Female | 1.00 (1.00, 1.00） |
| Czechia | 5 to 9 | T1D | Male | 1.00 (1.00, 1.00） |
| Czechia | 50 to 54 | T1D | Both | 11.37 (10.79, 11.98） |
| Czechia | 50 to 54 | T1D | Female | 9.66 (8.98, 10.40） |
| Czechia | 50 to 54 | T1D | Male | 13.28 (12.32, 14.30） |
| Czechia | 55 to 59 | T1D | Both | 12.92 (12.25, 13.62） |
| Czechia | 55 to 59 | T1D | Female | 10.96 (10.17, 11.81） |
| Czechia | 55 to 59 | T1D | Male | 15.13 (14.03, 16.32） |
| Czechia | 60 to 64 | T1D | Both | 14.62 (13.86, 15.43） |
| Czechia | 60 to 64 | T1D | Female | 12.42 (11.51, 13.39） |
| Czechia | 60 to 64 | T1D | Male | 17.16 (15.89, 18.52） |
| Czechia | 65 to 69 | T1D | Both | 16.51 (15.64, 17.44） |

| | | | | |
|---|---|---|---|---|
| Czechia | 65 to 69 | T1D | Female | 14.06 (13.02, 15.18） |
| Czechia | 65 to 69 | T1D | Male | 19.37 (17.92, 20.95） |
| Democratic People's Republic of Korea | 0 to 4 | T1D | Both | 0.16 (0.14, 0.17） |
| Democratic People's Republic of Korea | 0 to 4 | T1D | Female | 0.15 (0.13, 0.17） |
| Democratic People's Republic of Korea | 0 to 4 | T1D | Male | 0.16 (0.14, 0.18） |
| Democratic People's Republic of Korea | 10 to 14 | T1D | Both | 1.99 (1.91, 2.08） |
| Democratic People's Republic of Korea | 10 to 14 | T1D | Female | 1.98 (1.86, 2.10） |
| Democratic People's Republic of Korea | 10 to 14 | T1D | Male | 2.00 (1.88, 2.13） |
| Democratic People's Republic of Korea | 15 to 19 | T1D | Both | 2.90 (2.78, 3.03） |
| Democratic People's Republic of Korea | 15 to 19 | T1D | Female | 2.79 (2.63, 2.96） |
| Democratic People's Republic of Korea | 15 to 19 | T1D | Male | 3.02 (2.84, 3.20） |
| Democratic People's Republic of Korea | 20 to 24 | T1D | Both | 3.73 (3.57, 3.89） |
| Democratic People's Republic of Korea | 20 to 24 | T1D | Female | 3.44 (3.24, 3.66） |
| Democratic People's Republic of Korea | 20 to 24 | T1D | Male | 4.02 (3.78, 4.28） |
| Democratic People's Republic of Korea | 25 to 29 | T1D | Both | 4.60 (4.40, 4.81） |
| Democratic People's Republic of Korea | 25 to 29 | T1D | Female | 4.09 (3.84, 4.36） |
| Democratic People's Republic of Korea | 25 to 29 | T1D | Male | 5.13 (4.82, 5.47） |
| Democratic People's Republic of Korea | 30 to 34 | T1D | Both | 5.42 (5.17, 5.67） |
| Democratic People's Republic of Korea | 30 to 34 | T1D | Female | 4.69 (4.39, 5.00） |
| Democratic People's Republic of Korea | 30 to 34 | T1D | Male | 6.15 (5.76, 6.56） |
| Democratic People's Republic of Korea | 35 to 39 | T1D | Both | 6.23 (5.95, 6.53） |
| Democratic People's Republic of Korea | 35 to 39 | T1D | Female | 5.30 (4.96, 5.66） |
| Democratic People's Republic of Korea | 35 to 39 | T1D | Male | 7.15 (6.70, 7.64） |
| Democratic People's Republic of Korea | 40 to 44 | T1D | Both | 7.07 (6.74, 7.41） |
| Democratic People's Republic of Korea | 40 to 44 | T1D | Female | 5.94 (5.55, 6.36） |
| Democratic People's Republic of Korea | 40 to 44 | T1D | Male | 8.18 (7.65, 8.74） |
| Democratic People's Republic of Korea | 45 to 49 | T1D | Both | 7.92 (7.54, 8.32） |
| Democratic People's Republic of Korea | 45 to 49 | T1D | Female | 6.63 (6.18, 7.11） |
| Democratic People's Republic of Korea | 45 to 49 | T1D | Male | 9.21 (8.59, 9.86） |
| Democratic People's Republic of Korea | 5 to 9 | T1D | Both | 1.00 (1.00, 1.00） |
| Democratic People's Republic of Korea | 5 to 9 | T1D | Female | 1.00 (1.00, 1.00） |
| Democratic People's Republic of Korea | 5 to 9 | T1D | Male | 1.00 (1.00, 1.00） |
| Democratic People's Republic of Korea | 50 to 54 | T1D | Both | 8.79 (8.35, 9.25） |

| | | | | |
|---|---|---|---|---|
| Democratic People's Republic of Korea | 50 to 54 | T1D | Female | 7.36 (6.84, 7.91） |
| Democratic People's Republic of Korea | 50 to 54 | T1D | Male | 10.24 (9.53, 10.99） |
| Democratic People's Republic of Korea | 55 to 59 | T1D | Both | 9.68 (9.18, 10.20） |
| Democratic People's Republic of Korea | 55 to 59 | T1D | Female | 8.14 (7.55, 8.78） |
| Democratic People's Republic of Korea | 55 to 59 | T1D | Male | 11.28 (10.48, 12.15） |
| Democratic People's Republic of Korea | 60 to 64 | T1D | Both | 10.61 (10.05, 11.20） |
| Democratic People's Republic of Korea | 60 to 64 | T1D | Female | 8.99 (8.32, 9.71） |
| Democratic People's Republic of Korea | 60 to 64 | T1D | Male | 12.35 (11.44, 13.34） |
| Democratic People's Republic of Korea | 65 to 69 | T1D | Both | 11.54 (10.90, 12.22） |
| Democratic People's Republic of Korea | 65 to 69 | T1D | Female | 9.91 (9.15, 10.74） |
| Democratic People's Republic of Korea | 65 to 69 | T1D | Male | 13.46 (12.42, 14.59） |
| Democratic Republic of the Congo | 0 to 4 | T1D | Both | 0.16 (0.15, 0.16） |
| Democratic Republic of the Congo | 0 to 4 | T1D | Female | 0.16 (0.15, 0.16） |
| Democratic Republic of the Congo | 0 to 4 | T1D | Male | 0.15 (0.15, 0.16） |
| Democratic Republic of the Congo | 10 to 14 | T1D | Both | 2.09 (2.06, 2.13） |
| Democratic Republic of the Congo | 10 to 14 | T1D | Female | 2.10 (2.05, 2.14） |
| Democratic Republic of the Congo | 10 to 14 | T1D | Male | 2.09 (2.05, 2.14） |
| Democratic Republic of the Congo | 15 to 19 | T1D | Both | 3.08 (3.04, 3.13） |
| Democratic Republic of the Congo | 15 to 19 | T1D | Female | 3.09 (3.02, 3.16） |
| Democratic Republic of the Congo | 15 to 19 | T1D | Male | 3.08 (3.01, 3.15） |
| Democratic Republic of the Congo | 20 to 24 | T1D | Both | 3.92 (3.86, 3.99） |
| Democratic Republic of the Congo | 20 to 24 | T1D | Female | 3.94 (3.85, 4.03） |
| Democratic Republic of the Congo | 20 to 24 | T1D | Male | 3.91 (3.82, 4.00） |
| Democratic Republic of the Congo | 25 to 29 | T1D | Both | 4.78 (4.70, 4.86） |
| Democratic Republic of the Congo | 25 to 29 | T1D | Female | 4.81 (4.69, 4.92） |
| Democratic Republic of the Congo | 25 to 29 | T1D | Male | 4.75 (4.64, 4.87） |
| Democratic Republic of the Congo | 30 to 34 | T1D | Both | 5.40 (5.31, 5.50） |
| Democratic Republic of the Congo | 30 to 34 | T1D | Female | 5.45 (5.31, 5.59） |
| Democratic Republic of the Congo | 30 to 34 | T1D | Male | 5.36 (5.23, 5.49） |
| Democratic Republic of the Congo | 35 to 39 | T1D | Both | 5.87 (5.77, 5.98） |
| Democratic Republic of the Congo | 35 to 39 | T1D | Female | 5.93 (5.78, 6.09） |
| Democratic Republic of the Congo | 35 to 39 | T1D | Male | 5.82 (5.67, 5.97） |
| Democratic Republic of the Congo | 40 to 44 | T1D | Both | 6.26 (6.13, 6.38） |

| | | | | |
|---|---|---|---|---|
| Democratic Republic of the Congo | 40 to 44 | T1D | Female | 6.33 (6.16, 6.51） |
| Democratic Republic of the Congo | 40 to 44 | T1D | Male | 6.18 (6.01, 6.35） |
| Democratic Republic of the Congo | 45 to 49 | T1D | Both | 6.61 (6.48, 6.75） |
| Democratic Republic of the Congo | 45 to 49 | T1D | Female | 6.71 (6.51, 6.91） |
| Democratic Republic of the Congo | 45 to 49 | T1D | Male | 6.52 (6.33, 6.71） |
| Democratic Republic of the Congo | 5 to 9 | T1D | Both | 1.00 (1.00, 1.00） |
| Democratic Republic of the Congo | 5 to 9 | T1D | Female | 1.00 (1.00, 1.00） |
| Democratic Republic of the Congo | 5 to 9 | T1D | Male | 1.00 (1.00, 1.00） |
| Democratic Republic of the Congo | 50 to 54 | T1D | Both | 6.96 (6.80, 7.11） |
| Democratic Republic of the Congo | 50 to 54 | T1D | Female | 7.07 (6.86, 7.30） |
| Democratic Republic of the Congo | 50 to 54 | T1D | Male | 6.84 (6.63, 7.06） |
| Democratic Republic of the Congo | 55 to 59 | T1D | Both | 7.26 (7.09, 7.44） |
| Democratic Republic of the Congo | 55 to 59 | T1D | Female | 7.41 (7.16, 7.66） |
| Democratic Republic of the Congo | 55 to 59 | T1D | Male | 7.13 (6.89, 7.37） |
| Democratic Republic of the Congo | 60 to 64 | T1D | Both | 7.53 (7.34, 7.73） |
| Democratic Republic of the Congo | 60 to 64 | T1D | Female | 7.69 (7.42, 7.98） |
| Democratic Republic of the Congo | 60 to 64 | T1D | Male | 7.37 (7.10, 7.65） |
| Democratic Republic of the Congo | 65 to 69 | T1D | Both | 7.73 (7.51, 7.96） |
| Democratic Republic of the Congo | 65 to 69 | T1D | Female | 7.92 (7.61, 8.25） |
| Democratic Republic of the Congo | 65 to 69 | T1D | Male | 7.55 (7.24, 7.87） |
| Denmark | 0 to 4 | T1D | Both | 0.21 (0.19, 0.23） |
| Denmark | 0 to 4 | T1D | Female | 0.19 (0.16, 0.22） |
| Denmark | 0 to 4 | T1D | Male | 0.22 (0.19, 0.25） |
| Denmark | 10 to 14 | T1D | Both | 2.10 (2.00, 2.21） |
| Denmark | 10 to 14 | T1D | Female | 2.09 (1.95, 2.24） |
| Denmark | 10 to 14 | T1D | Male | 2.11 (1.97, 2.26） |
| Denmark | 15 to 19 | T1D | Both | 3.21 (3.06, 3.38） |
| Denmark | 15 to 19 | T1D | Female | 3.11 (2.90, 3.33） |
| Denmark | 15 to 19 | T1D | Male | 3.32 (3.10, 3.55） |
| Denmark | 20 to 24 | T1D | Both | 4.24 (4.04, 4.46） |
| Denmark | 20 to 24 | T1D | Female | 4.00 (3.73, 4.30） |
| Denmark | 20 to 24 | T1D | Male | 4.48 (4.18, 4.80） |
| Denmark | 25 to 29 | T1D | Both | 5.39 (5.12, 5.67） |

| | | | | |
|---|---|---|---|---|
| Denmark | 25 to 29 | T1D | Female | 4.97 (4.62, 5.35） |
| Denmark | 25 to 29 | T1D | Male | 5.80 (5.40, 6.22） |
| Denmark | 30 to 34 | T1D | Both | 6.52 (6.19, 6.86） |
| Denmark | 30 to 34 | T1D | Female | 5.92 (5.49, 6.37） |
| Denmark | 30 to 34 | T1D | Male | 7.11 (6.62, 7.63） |
| Denmark | 35 to 39 | T1D | Both | 7.70 (7.31, 8.12） |
| Denmark | 35 to 39 | T1D | Female | 6.91 (6.41, 7.46） |
| Denmark | 35 to 39 | T1D | Male | 8.48 (7.89, 9.12） |
| Denmark | 40 to 44 | T1D | Both | 8.98 (8.52, 9.47） |
| Denmark | 40 to 44 | T1D | Female | 8.00 (7.41, 8.64） |
| Denmark | 40 to 44 | T1D | Male | 9.96 (9.26, 10.72） |
| Denmark | 45 to 49 | T1D | Both | 10.36 (9.82, 10.93） |
| Denmark | 45 to 49 | T1D | Female | 9.18 (8.49, 9.92） |
| Denmark | 45 to 49 | T1D | Male | 11.54 (10.72, 12.44） |
| Denmark | 5 to 9 | T1D | Both | 1.00 (1.00, 1.00） |
| Denmark | 5 to 9 | T1D | Female | 1.00 (1.00, 1.00） |
| Denmark | 5 to 9 | T1D | Male | 1.00 (1.00, 1.00） |
| Denmark | 50 to 54 | T1D | Both | 11.83 (11.20, 12.49） |
| Denmark | 50 to 54 | T1D | Female | 10.45 (9.65, 11.31） |
| Denmark | 50 to 54 | T1D | Male | 13.22 (12.26, 14.26） |
| Denmark | 55 to 59 | T1D | Both | 13.40 (12.68, 14.17） |
| Denmark | 55 to 59 | T1D | Female | 11.83 (10.92, 12.83） |
| Denmark | 55 to 59 | T1D | Male | 15.00 (13.90, 16.20） |
| Denmark | 60 to 64 | T1D | Both | 15.09 (14.26, 15.96） |
| Denmark | 60 to 64 | T1D | Female | 13.35 (12.30, 14.49） |
| Denmark | 60 to 64 | T1D | Male | 16.91 (15.64, 18.28） |
| Denmark | 65 to 69 | T1D | Both | 16.92 (15.98, 17.93） |
| Denmark | 65 to 69 | T1D | Female | 15.02 (13.82, 16.33） |
| Denmark | 65 to 69 | T1D | Male | 18.98 (17.53, 20.54） |
| Djibouti | 0 to 4 | T1D | Both | 0.15 (0.11, 0.20） |
| Djibouti | 0 to 4 | T1D | Female | 0.15 (0.09, 0.24） |
| Djibouti | 0 to 4 | T1D | Male | 0.15 (0.10, 0.24） |
| Djibouti | 10 to 14 | T1D | Both | 2.06 (1.76, 2.40） |

| | | | | |
|---|---|---|---|---|
| Djibouti | 10 to 14 | T1D | Female | 2.07 (1.64, 2.61） |
| Djibouti | 10 to 14 | T1D | Male | 2.04 (1.65, 2.51） |
| Djibouti | 15 to 19 | T1D | Both | 3.01 (2.58, 3.52） |
| Djibouti | 15 to 19 | T1D | Female | 3.02 (2.39, 3.82） |
| Djibouti | 15 to 19 | T1D | Male | 2.99 (2.43, 3.69） |
| Djibouti | 20 to 24 | T1D | Both | 3.89 (3.32, 4.56） |
| Djibouti | 20 to 24 | T1D | Female | 3.92 (3.09, 4.96） |
| Djibouti | 20 to 24 | T1D | Male | 3.88 (3.13, 4.80） |
| Djibouti | 25 to 29 | T1D | Both | 4.79 (4.07, 5.63） |
| Djibouti | 25 to 29 | T1D | Female | 4.80 (3.77, 6.12） |
| Djibouti | 25 to 29 | T1D | Male | 4.78 (3.84, 5.95） |
| Djibouti | 30 to 34 | T1D | Both | 5.36 (4.53, 6.33） |
| Djibouti | 30 to 34 | T1D | Female | 5.40 (4.21, 6.93） |
| Djibouti | 30 to 34 | T1D | Male | 5.36 (4.27, 6.71） |
| Djibouti | 35 to 39 | T1D | Both | 5.80 (4.87, 6.90） |
| Djibouti | 35 to 39 | T1D | Female | 5.87 (4.53, 7.61） |
| Djibouti | 35 to 39 | T1D | Male | 5.79 (4.58, 7.32） |
| Djibouti | 40 to 44 | T1D | Both | 6.18 (5.16, 7.41） |
| Djibouti | 40 to 44 | T1D | Female | 6.24 (4.76, 8.19） |
| Djibouti | 40 to 44 | T1D | Male | 6.13 (4.80, 7.82） |
| Djibouti | 45 to 49 | T1D | Both | 6.49 (5.37, 7.85） |
| Djibouti | 45 to 49 | T1D | Female | 6.58 (4.95, 8.75） |
| Djibouti | 45 to 49 | T1D | Male | 6.44 (4.99, 8.32） |
| Djibouti | 5 to 9 | T1D | Both | 1.00 (1.00, 1.00） |
| Djibouti | 5 to 9 | T1D | Female | 1.00 (1.00, 1.00） |
| Djibouti | 5 to 9 | T1D | Male | 1.00 (1.00, 1.00） |
| Djibouti | 50 to 54 | T1D | Both | 6.76 (5.53, 8.26） |
| Djibouti | 50 to 54 | T1D | Female | 6.88 (5.08, 9.31） |
| Djibouti | 50 to 54 | T1D | Male | 6.70 (5.11, 8.77） |
| Djibouti | 55 to 59 | T1D | Both | 7.01 (5.65, 8.69） |
| Djibouti | 55 to 59 | T1D | Female | 7.07 (5.11, 9.78） |
| Djibouti | 55 to 59 | T1D | Male | 6.96 (5.22, 9.28） |
| Djibouti | 60 to 64 | T1D | Both | 7.21 (5.71, 9.11） |

| | | | | |
|---|---|---|---|---|
| Djibouti | 60 to 64 | T1D | Female | 7.33 (5.16, 10.42） |
| Djibouti | 60 to 64 | T1D | Male | 7.17 (5.25, 9.81） |
| Djibouti | 65 to 69 | T1D | Both | 7.48 (5.76, 9.70） |
| Djibouti | 65 to 69 | T1D | Female | 7.62 (5.16, 11.26） |
| Djibouti | 65 to 69 | T1D | Male | 7.22 (5.08, 10.27） |
| Dominica | 0 to 4 | T1D | Both | 0.27 (0.08, 0.92） |
| Dominica | 0 to 4 | T1D | Female | 0.06 (0.01, 1.06） |
| Dominica | 0 to 4 | T1D | Male | 0.06 (0.01, 1.08） |
| Dominica | 10 to 14 | T1D | Both | 2.17 (1.19, 3.96） |
| Dominica | 10 to 14 | T1D | Female | 1.83 (0.83, 4.07） |
| Dominica | 10 to 14 | T1D | Male | 1.72 (0.77, 3.85） |
| Dominica | 15 to 19 | T1D | Both | 3.36 (1.85, 6.11） |
| Dominica | 15 to 19 | T1D | Female | 2.99 (1.39, 6.45） |
| Dominica | 15 to 19 | T1D | Male | 2.53 (1.15, 5.55） |
| Dominica | 20 to 24 | T1D | Both | 4.33 (2.34, 7.99） |
| Dominica | 20 to 24 | T1D | Female | 3.61 (1.63, 8.01） |
| Dominica | 20 to 24 | T1D | Male | 3.65 (1.64, 8.10） |
| Dominica | 25 to 29 | T1D | Both | 5.45 (2.92, 10.18） |
| Dominica | 25 to 29 | T1D | Female | 4.26 (1.87, 9.72） |
| Dominica | 25 to 29 | T1D | Male | 4.55 (2.02, 10.30） |
| Dominica | 30 to 34 | T1D | Both | 6.44 (3.40, 12.20） |
| Dominica | 30 to 34 | T1D | Female | 5.01 (2.16, 11.65） |
| Dominica | 30 to 34 | T1D | Male | 5.74 (2.51, 13.17） |
| Dominica | 35 to 39 | T1D | Both | 7.43 (3.86, 14.31） |
| Dominica | 35 to 39 | T1D | Female | 5.56 (2.31, 13.37） |
| Dominica | 35 to 39 | T1D | Male | 6.64 (2.82, 15.65） |
| Dominica | 40 to 44 | T1D | Both | 8.35 (4.27, 16.31） |
| Dominica | 40 to 44 | T1D | Female | 6.34 (2.58, 15.59） |
| Dominica | 40 to 44 | T1D | Male | 7.26 (3.02, 17.42） |
| Dominica | 45 to 49 | T1D | Both | 9.67 (4.88, 19.17） |
| Dominica | 45 to 49 | T1D | Female | 6.82 (2.70, 17.26） |
| Dominica | 45 to 49 | T1D | Male | 8.73 (3.57, 21.32） |
| Dominica | 5 to 9 | T1D | Both | 1.00 (1.00, 1.00） |

| | | | | |
|---|---|---|---|---|
| Dominica | 5 to 9 | T1D | Female | 1.00 (1.00, 1.00） |
| Dominica | 5 to 9 | T1D | Male | 1.00 (1.00, 1.00） |
| Dominica | 50 to 54 | T1D | Both | 10.53 (5.20, 21.34） |
| Dominica | 50 to 54 | T1D | Female | 7.51 (2.86, 19.72） |
| Dominica | 50 to 54 | T1D | Male | 9.61 (3.82, 24.14） |
| Dominica | 55 to 59 | T1D | Both | 11.63 (5.61, 24.13） |
| Dominica | 55 to 59 | T1D | Female | 8.30 (3.04, 22.68） |
| Dominica | 55 to 59 | T1D | Male | 10.60 (4.09, 27.49） |
| Dominica | 60 to 64 | T1D | Both | 12.87 (6.05, 27.34） |
| Dominica | 60 to 64 | T1D | Female | 8.57 (3.01, 24.42） |
| Dominica | 60 to 64 | T1D | Male | 11.86 (4.41, 31.88） |
| Dominica | 65 to 69 | T1D | Both | 13.31 (6.04, 29.34） |
| Dominica | 65 to 69 | T1D | Female | 9.69 (3.25, 28.91） |
| Dominica | 65 to 69 | T1D | Male | 12.54 (4.44, 35.40） |
| Dominican Republic | 0 to 4 | T1D | Both | 0.14 (0.13, 0.16） |
| Dominican Republic | 0 to 4 | T1D | Female | 0.13 (0.11, 0.15） |
| Dominican Republic | 0 to 4 | T1D | Male | 0.16 (0.13, 0.18） |
| Dominican Republic | 10 to 14 | T1D | Both | 2.26 (2.15, 2.38） |
| Dominican Republic | 10 to 14 | T1D | Female | 2.41 (2.25, 2.60） |
| Dominican Republic | 10 to 14 | T1D | Male | 2.10 (1.94, 2.26） |
| Dominican Republic | 15 to 19 | T1D | Both | 3.35 (3.18, 3.54） |
| Dominican Republic | 15 to 19 | T1D | Female | 3.51 (3.27, 3.78） |
| Dominican Republic | 15 to 19 | T1D | Male | 3.17 (2.94, 3.42） |
| Dominican Republic | 20 to 24 | T1D | Both | 4.23 (4.01, 4.47） |
| Dominican Republic | 20 to 24 | T1D | Female | 4.24 (3.94, 4.57） |
| Dominican Republic | 20 to 24 | T1D | Male | 4.21 (3.90, 4.54） |
| Dominican Republic | 25 to 29 | T1D | Both | 5.13 (4.85, 5.42） |
| Dominican Republic | 25 to 29 | T1D | Female | 4.92 (4.56, 5.31） |
| Dominican Republic | 25 to 29 | T1D | Male | 5.35 (4.94, 5.79） |
| Dominican Republic | 30 to 34 | T1D | Both | 5.89 (5.56, 6.23） |
| Dominican Republic | 30 to 34 | T1D | Female | 5.48 (5.06, 5.93） |
| Dominican Republic | 30 to 34 | T1D | Male | 6.32 (5.83, 6.85） |
| Dominican Republic | 35 to 39 | T1D | Both | 6.61 (6.23, 7.00） |

| | | | | |
|---|---|---|---|---|
| Dominican Republic | 35 to 39 | T1D | Female | 6.02 (5.55, 6.54） |
| Dominican Republic | 35 to 39 | T1D | Male | 7.24 (6.66, 7.87） |
| Dominican Republic | 40 to 44 | T1D | Both | 7.33 (6.90, 7.79） |
| Dominican Republic | 40 to 44 | T1D | Female | 6.57 (6.04, 7.16） |
| Dominican Republic | 40 to 44 | T1D | Male | 8.15 (7.48, 8.89） |
| Dominican Republic | 45 to 49 | T1D | Both | 8.04 (7.55, 8.57） |
| Dominican Republic | 45 to 49 | T1D | Female | 7.12 (6.52, 7.78） |
| Dominican Republic | 45 to 49 | T1D | Male | 9.04 (8.26, 9.88） |
| Dominican Republic | 5 to 9 | T1D | Both | 1.00 (1.00, 1.00） |
| Dominican Republic | 5 to 9 | T1D | Female | 1.00 (1.00, 1.00） |
| Dominican Republic | 5 to 9 | T1D | Male | 1.00 (1.00, 1.00） |
| Dominican Republic | 50 to 54 | T1D | Both | 8.73 (8.18, 9.33） |
| Dominican Republic | 50 to 54 | T1D | Female | 7.67 (6.99, 8.42） |
| Dominican Republic | 50 to 54 | T1D | Male | 9.87 (8.99, 10.84） |
| Dominican Republic | 55 to 59 | T1D | Both | 9.41 (8.78, 10.08） |
| Dominican Republic | 55 to 59 | T1D | Female | 8.23 (7.47, 9.08） |
| Dominican Republic | 55 to 59 | T1D | Male | 10.68 (9.69, 11.78） |
| Dominican Republic | 60 to 64 | T1D | Both | 10.09 (9.38, 10.85） |
| Dominican Republic | 60 to 64 | T1D | Female | 8.80 (7.94, 9.77） |
| Dominican Republic | 60 to 64 | T1D | Male | 11.46 (10.34, 12.71） |
| Dominican Republic | 65 to 69 | T1D | Both | 10.77 (9.96, 11.65） |
| Dominican Republic | 65 to 69 | T1D | Female | 9.41 (8.42, 10.52） |
| Dominican Republic | 65 to 69 | T1D | Male | 12.25 (10.97, 13.68） |
| Ecuador | 0 to 4 | T1D | Both | 0.15 (0.14, 0.17） |
| Ecuador | 0 to 4 | T1D | Female | 0.15 (0.13, 0.17） |
| Ecuador | 0 to 4 | T1D | Male | 0.16 (0.14, 0.18） |
| Ecuador | 10 to 14 | T1D | Both | 2.06 (1.98, 2.15） |
| Ecuador | 10 to 14 | T1D | Female | 2.12 (2.01, 2.24） |
| Ecuador | 10 to 14 | T1D | Male | 1.99 (1.87, 2.12） |
| Ecuador | 15 to 19 | T1D | Both | 2.99 (2.87, 3.11） |
| Ecuador | 15 to 19 | T1D | Female | 3.06 (2.90, 3.24） |
| Ecuador | 15 to 19 | T1D | Male | 2.88 (2.71, 3.06） |
| Ecuador | 20 to 24 | T1D | Both | 3.73 (3.58, 3.89） |

| | | | | |
|---|---|---|---|---|
| Ecuador | 20 to 24 | T1D | Female | 3.76 (3.55, 3.97） |
| Ecuador | 20 to 24 | T1D | Male | 3.68 (3.46, 3.92） |
| Ecuador | 25 to 29 | T1D | Both | 4.46 (4.28, 4.66） |
| Ecuador | 25 to 29 | T1D | Female | 4.40 (4.15, 4.66） |
| Ecuador | 25 to 29 | T1D | Male | 4.52 (4.24, 4.82） |
| Ecuador | 30 to 34 | T1D | Both | 5.07 (4.85, 5.30） |
| Ecuador | 30 to 34 | T1D | Female | 4.92 (4.64, 5.23） |
| Ecuador | 30 to 34 | T1D | Male | 5.23 (4.89, 5.59） |
| Ecuador | 35 to 39 | T1D | Both | 5.62 (5.37, 5.89） |
| Ecuador | 35 to 39 | T1D | Female | 5.41 (5.08, 5.75） |
| Ecuador | 35 to 39 | T1D | Male | 5.87 (5.48, 6.29） |
| Ecuador | 40 to 44 | T1D | Both | 6.15 (5.87, 6.45） |
| Ecuador | 40 to 44 | T1D | Female | 5.88 (5.51, 6.27） |
| Ecuador | 40 to 44 | T1D | Male | 6.48 (6.03, 6.96） |
| Ecuador | 45 to 49 | T1D | Both | 6.68 (6.36, 7.02） |
| Ecuador | 45 to 49 | T1D | Female | 6.35 (5.94, 6.79） |
| Ecuador | 45 to 49 | T1D | Male | 7.07 (6.57, 7.61） |
| Ecuador | 5 to 9 | T1D | Both | 1.00 (1.00, 1.00） |
| Ecuador | 5 to 9 | T1D | Female | 1.00 (1.00, 1.00） |
| Ecuador | 5 to 9 | T1D | Male | 1.00 (1.00, 1.00） |
| Ecuador | 50 to 54 | T1D | Both | 7.20 (6.83, 7.58） |
| Ecuador | 50 to 54 | T1D | Female | 6.83 (6.37, 7.33） |
| Ecuador | 50 to 54 | T1D | Male | 7.63 (7.07, 8.25） |
| Ecuador | 55 to 59 | T1D | Both | 7.70 (7.30, 8.13） |
| Ecuador | 55 to 59 | T1D | Female | 7.31 (6.80, 7.87） |
| Ecuador | 55 to 59 | T1D | Male | 8.16 (7.53, 8.85） |
| Ecuador | 60 to 64 | T1D | Both | 8.21 (7.75, 8.69） |
| Ecuador | 60 to 64 | T1D | Female | 7.81 (7.23, 8.43） |
| Ecuador | 60 to 64 | T1D | Male | 8.68 (7.97, 9.45） |
| Ecuador | 65 to 69 | T1D | Both | 8.74 (8.22, 9.29） |
| Ecuador | 65 to 69 | T1D | Female | 8.33 (7.68, 9.05） |
| Ecuador | 65 to 69 | T1D | Male | 9.19 (8.39, 10.07） |
| Egypt | 0 to 4 | T1D | Both | 0.15 (0.14, 0.15） |

| | | | | |
|---|---|---|---|---|
| Egypt | 0 to 4 | T1D | Female | 0.16 (0.15, 0.16） |
| Egypt | 0 to 4 | T1D | Male | 0.14 (0.14, 0.15） |
| Egypt | 10 to 14 | T1D | Both | 2.31 (2.28, 2.34） |
| Egypt | 10 to 14 | T1D | Female | 2.42 (2.37, 2.47） |
| Egypt | 10 to 14 | T1D | Male | 2.22 (2.18, 2.26） |
| Egypt | 15 to 19 | T1D | Both | 3.59 (3.54, 3.64） |
| Egypt | 15 to 19 | T1D | Female | 3.81 (3.73, 3.89） |
| Egypt | 15 to 19 | T1D | Male | 3.41 (3.34, 3.47） |
| Egypt | 20 to 24 | T1D | Both | 4.75 (4.68, 4.82） |
| Egypt | 20 to 24 | T1D | Female | 5.01 (4.90, 5.11） |
| Egypt | 20 to 24 | T1D | Male | 4.55 (4.47, 4.65） |
| Egypt | 25 to 29 | T1D | Both | 6.04 (5.95, 6.13） |
| Egypt | 25 to 29 | T1D | Female | 6.28 (6.15, 6.42） |
| Egypt | 25 to 29 | T1D | Male | 5.85 (5.73, 5.97） |
| Egypt | 30 to 34 | T1D | Both | 7.26 (7.15, 7.38） |
| Egypt | 30 to 34 | T1D | Female | 7.49 (7.32, 7.67） |
| Egypt | 30 to 34 | T1D | Male | 7.09 (6.94, 7.24） |
| Egypt | 35 to 39 | T1D | Both | 8.46 (8.32, 8.60） |
| Egypt | 35 to 39 | T1D | Female | 8.68 (8.48, 8.89） |
| Egypt | 35 to 39 | T1D | Male | 8.29 (8.11, 8.48） |
| Egypt | 40 to 44 | T1D | Both | 9.67 (9.50, 9.83） |
| Egypt | 40 to 44 | T1D | Female | 9.88 (9.64, 10.13） |
| Egypt | 40 to 44 | T1D | Male | 9.49 (9.28, 9.71） |
| Egypt | 45 to 49 | T1D | Both | 10.88 (10.69, 11.07） |
| Egypt | 45 to 49 | T1D | Female | 11.12 (10.84, 11.41） |
| Egypt | 45 to 49 | T1D | Male | 10.68 (10.43, 10.94） |
| Egypt | 5 to 9 | T1D | Both | 1.00 (1.00, 1.00） |
| Egypt | 5 to 9 | T1D | Female | 1.00 (1.00, 1.00） |
| Egypt | 5 to 9 | T1D | Male | 1.00 (1.00, 1.00） |
| Egypt | 50 to 54 | T1D | Both | 12.11 (11.89, 12.33） |
| Egypt | 50 to 54 | T1D | Female | 12.40 (12.07, 12.74） |
| Egypt | 50 to 54 | T1D | Male | 11.86 (11.57, 12.16） |
| Egypt | 55 to 59 | T1D | Both | 13.36 (13.11, 13.62） |

| | | | | |
|---|---|---|---|---|
| Egypt | 55 to 59 | T1D | Female | 13.73 (13.34, 14.12） |
| Egypt | 55 to 59 | T1D | Male | 13.03 (12.70, 13.37） |
| Egypt | 60 to 64 | T1D | Both | 14.65 (14.35, 14.95） |
| Egypt | 60 to 64 | T1D | Female | 15.10 (14.65, 15.56） |
| Egypt | 60 to 64 | T1D | Male | 14.20 (13.82, 14.59） |
| Egypt | 65 to 69 | T1D | Both | 15.97 (15.62, 16.33） |
| Egypt | 65 to 69 | T1D | Female | 16.56 (16.03, 17.11） |
| Egypt | 65 to 69 | T1D | Male | 15.39 (14.94, 15.85） |
| El Salvador | 0 to 4 | T1D | Both | 0.13 (0.11, 0.15） |
| El Salvador | 0 to 4 | T1D | Female | 0.12 (0.09, 0.15） |
| El Salvador | 0 to 4 | T1D | Male | 0.14 (0.11, 0.17） |
| El Salvador | 10 to 14 | T1D | Both | 2.24 (2.10, 2.39） |
| El Salvador | 10 to 14 | T1D | Female | 2.41 (2.21, 2.63） |
| El Salvador | 10 to 14 | T1D | Male | 2.04 (1.86, 2.25） |
| El Salvador | 15 to 19 | T1D | Both | 3.04 (2.85, 3.24） |
| El Salvador | 15 to 19 | T1D | Female | 3.22 (2.95, 3.51） |
| El Salvador | 15 to 19 | T1D | Male | 2.82 (2.57, 3.10） |
| El Salvador | 20 to 24 | T1D | Both | 3.58 (3.35, 3.83） |
| El Salvador | 20 to 24 | T1D | Female | 3.66 (3.35, 4.01） |
| El Salvador | 20 to 24 | T1D | Male | 3.43 (3.11, 3.79） |
| El Salvador | 25 to 29 | T1D | Both | 4.09 (3.82, 4.38） |
| El Salvador | 25 to 29 | T1D | Female | 4.06 (3.70, 4.45） |
| El Salvador | 25 to 29 | T1D | Male | 4.06 (3.67, 4.50） |
| El Salvador | 30 to 34 | T1D | Both | 4.51 (4.19, 4.84） |
| El Salvador | 30 to 34 | T1D | Female | 4.38 (3.98, 4.83） |
| El Salvador | 30 to 34 | T1D | Male | 4.59 (4.12, 5.11） |
| El Salvador | 35 to 39 | T1D | Both | 4.87 (4.52, 5.25） |
| El Salvador | 35 to 39 | T1D | Female | 4.67 (4.22, 5.17） |
| El Salvador | 35 to 39 | T1D | Male | 5.06 (4.52, 5.66） |
| El Salvador | 40 to 44 | T1D | Both | 5.22 (4.83, 5.65） |
| El Salvador | 40 to 44 | T1D | Female | 4.95 (4.45, 5.50） |
| El Salvador | 40 to 44 | T1D | Male | 5.51 (4.90, 6.19） |
| El Salvador | 45 to 49 | T1D | Both | 5.56 (5.12, 6.04） |

| | | | | |
|---|---|---|---|---|
| El Salvador | 45 to 49 | T1D | Female | 5.22 (4.67, 5.84） |
| El Salvador | 45 to 49 | T1D | Male | 5.91 (5.23, 6.69） |
| El Salvador | 5 to 9 | T1D | Both | 1.00 (1.00, 1.00） |
| El Salvador | 5 to 9 | T1D | Female | 1.00 (1.00, 1.00） |
| El Salvador | 5 to 9 | T1D | Male | 1.00 (1.00, 1.00） |
| El Salvador | 50 to 54 | T1D | Both | 5.88 (5.39, 6.41） |
| El Salvador | 50 to 54 | T1D | Female | 5.49 (4.89, 6.18） |
| El Salvador | 50 to 54 | T1D | Male | 6.30 (5.53, 7.17） |
| El Salvador | 55 to 59 | T1D | Both | 6.18 (5.64, 6.78） |
| El Salvador | 55 to 59 | T1D | Female | 5.76 (5.08, 6.51） |
| El Salvador | 55 to 59 | T1D | Male | 6.66 (5.80, 7.64） |
| El Salvador | 60 to 64 | T1D | Both | 6.47 (5.87, 7.14） |
| El Salvador | 60 to 64 | T1D | Female | 6.01 (5.26, 6.86） |
| El Salvador | 60 to 64 | T1D | Male | 6.98 (6.03, 8.08） |
| El Salvador | 65 to 69 | T1D | Both | 6.77 (6.09, 7.52） |
| El Salvador | 65 to 69 | T1D | Female | 6.29 (5.46, 7.25） |
| El Salvador | 65 to 69 | T1D | Male | 7.30 (6.23, 8.55） |
| Equatorial Guinea | 0 to 4 | T1D | Both | 0.16 (0.13, 0.21） |
| Equatorial Guinea | 0 to 4 | T1D | Female | 0.16 (0.11, 0.24） |
| Equatorial Guinea | 0 to 4 | T1D | Male | 0.16 (0.12, 0.24） |
| Equatorial Guinea | 10 to 14 | T1D | Both | 2.06 (1.81, 2.33） |
| Equatorial Guinea | 10 to 14 | T1D | Female | 2.05 (1.70, 2.48） |
| Equatorial Guinea | 10 to 14 | T1D | Male | 2.07 (1.75, 2.44） |
| Equatorial Guinea | 15 to 19 | T1D | Both | 3.02 (2.65, 3.44） |
| Equatorial Guinea | 15 to 19 | T1D | Female | 3.01 (2.48, 3.65） |
| Equatorial Guinea | 15 to 19 | T1D | Male | 3.03 (2.55, 3.61） |
| Equatorial Guinea | 20 to 24 | T1D | Both | 3.82 (3.34, 4.38） |
| Equatorial Guinea | 20 to 24 | T1D | Female | 3.82 (3.12, 4.67） |
| Equatorial Guinea | 20 to 24 | T1D | Male | 3.84 (3.20, 4.61） |
| Equatorial Guinea | 25 to 29 | T1D | Both | 4.64 (4.03, 5.34） |
| Equatorial Guinea | 25 to 29 | T1D | Female | 4.63 (3.76, 5.70） |
| Equatorial Guinea | 25 to 29 | T1D | Male | 4.65 (3.83, 5.64） |
| Equatorial Guinea | 30 to 34 | T1D | Both | 5.25 (4.52, 6.09） |

| | | | | |
|---|---|---|---|---|
| Equatorial Guinea | 30 to 34 | T1D | Female | 5.25 (4.22, 6.53） |
| Equatorial Guinea | 30 to 34 | T1D | Male | 5.25 (4.28, 6.45） |
| Equatorial Guinea | 35 to 39 | T1D | Both | 5.71 (4.87, 6.69） |
| Equatorial Guinea | 35 to 39 | T1D | Female | 5.76 (4.57, 7.24） |
| Equatorial Guinea | 35 to 39 | T1D | Male | 5.69 (4.56, 7.10） |
| Equatorial Guinea | 40 to 44 | T1D | Both | 6.09 (5.15, 7.21） |
| Equatorial Guinea | 40 to 44 | T1D | Female | 6.13 (4.82, 7.81） |
| Equatorial Guinea | 40 to 44 | T1D | Male | 6.04 (4.77, 7.66） |
| Equatorial Guinea | 45 to 49 | T1D | Both | 6.47 (5.41, 7.75） |
| Equatorial Guinea | 45 to 49 | T1D | Female | 6.51 (5.05, 8.41） |
| Equatorial Guinea | 45 to 49 | T1D | Male | 6.40 (4.95, 8.27） |
| Equatorial Guinea | 5 to 9 | T1D | Both | 1.00 (1.00, 1.00） |
| Equatorial Guinea | 5 to 9 | T1D | Female | 1.00 (1.00, 1.00） |
| Equatorial Guinea | 5 to 9 | T1D | Male | 1.00 (1.00, 1.00） |
| Equatorial Guinea | 50 to 54 | T1D | Both | 6.86 (5.66, 8.32） |
| Equatorial Guinea | 50 to 54 | T1D | Female | 6.91 (5.27, 9.06） |
| Equatorial Guinea | 50 to 54 | T1D | Male | 6.82 (5.17, 8.99） |
| Equatorial Guinea | 55 to 59 | T1D | Both | 7.24 (5.88, 8.90） |
| Equatorial Guinea | 55 to 59 | T1D | Female | 7.34 (5.50, 9.81） |
| Equatorial Guinea | 55 to 59 | T1D | Male | 7.16 (5.30, 9.67） |
| Equatorial Guinea | 60 to 64 | T1D | Both | 7.61 (6.08, 9.52） |
| Equatorial Guinea | 60 to 64 | T1D | Female | 7.73 (5.66, 10.55） |
| Equatorial Guinea | 60 to 64 | T1D | Male | 7.52 (5.42, 10.43） |
| Equatorial Guinea | 65 to 69 | T1D | Both | 8.04 (6.27, 10.30） |
| Equatorial Guinea | 65 to 69 | T1D | Female | 8.16 (5.80, 11.48） |
| Equatorial Guinea | 65 to 69 | T1D | Male | 7.79 (5.40, 11.24） |
| Eritrea | 0 to 4 | T1D | Both | 0.15 (0.14, 0.17） |
| Eritrea | 0 to 4 | T1D | Female | 0.15 (0.13, 0.18） |
| Eritrea | 0 to 4 | T1D | Male | 0.15 (0.13, 0.18） |
| Eritrea | 10 to 14 | T1D | Both | 2.06 (1.94, 2.17） |
| Eritrea | 10 to 14 | T1D | Female | 2.06 (1.90, 2.23） |
| Eritrea | 10 to 14 | T1D | Male | 2.06 (1.90, 2.22） |
| Eritrea | 15 to 19 | T1D | Both | 3.01 (2.85, 3.19） |

| | | | | |
|---|---|---|---|---|
| Eritrea | 15 to 19 | T1D | Female | 3.01 (2.77, 3.27） |
| Eritrea | 15 to 19 | T1D | Male | 3.02 (2.79, 3.26） |
| Eritrea | 20 to 24 | T1D | Both | 3.90 (3.68, 4.14） |
| Eritrea | 20 to 24 | T1D | Female | 3.90 (3.58, 4.24） |
| Eritrea | 20 to 24 | T1D | Male | 3.90 (3.60, 4.23） |
| Eritrea | 25 to 29 | T1D | Both | 4.79 (4.51, 5.09） |
| Eritrea | 25 to 29 | T1D | Female | 4.79 (4.39, 5.23） |
| Eritrea | 25 to 29 | T1D | Male | 4.78 (4.40, 5.20） |
| Eritrea | 30 to 34 | T1D | Both | 5.34 (5.01, 5.69） |
| Eritrea | 30 to 34 | T1D | Female | 5.36 (4.89, 5.87） |
| Eritrea | 30 to 34 | T1D | Male | 5.32 (4.88, 5.81） |
| Eritrea | 35 to 39 | T1D | Both | 5.73 (5.36, 6.13） |
| Eritrea | 35 to 39 | T1D | Female | 5.76 (5.23, 6.34） |
| Eritrea | 35 to 39 | T1D | Male | 5.70 (5.20, 6.25） |
| Eritrea | 40 to 44 | T1D | Both | 6.05 (5.64, 6.49） |
| Eritrea | 40 to 44 | T1D | Female | 6.10 (5.51, 6.75） |
| Eritrea | 40 to 44 | T1D | Male | 6.01 (5.45, 6.62） |
| Eritrea | 45 to 49 | T1D | Both | 6.31 (5.86, 6.79） |
| Eritrea | 45 to 49 | T1D | Female | 6.38 (5.73, 7.09） |
| Eritrea | 45 to 49 | T1D | Male | 6.25 (5.64, 6.93） |
| Eritrea | 5 to 9 | T1D | Both | 1.00 (1.00, 1.00） |
| Eritrea | 5 to 9 | T1D | Female | 1.00 (1.00, 1.00） |
| Eritrea | 5 to 9 | T1D | Male | 1.00 (1.00, 1.00） |
| Eritrea | 50 to 54 | T1D | Both | 6.50 (6.01, 7.04） |
| Eritrea | 50 to 54 | T1D | Female | 6.60 (5.89, 7.39） |
| Eritrea | 50 to 54 | T1D | Male | 6.44 (5.76, 7.20） |
| Eritrea | 55 to 59 | T1D | Both | 6.67 (6.12, 7.27） |
| Eritrea | 55 to 59 | T1D | Female | 6.78 (6.01, 7.66） |
| Eritrea | 55 to 59 | T1D | Male | 6.59 (5.83, 7.45） |
| Eritrea | 60 to 64 | T1D | Both | 6.80 (6.18, 7.47） |
| Eritrea | 60 to 64 | T1D | Female | 6.93 (6.07, 7.90） |
| Eritrea | 60 to 64 | T1D | Male | 6.69 (5.83, 7.68） |
| Eritrea | 65 to 69 | T1D | Both | 6.89 (6.19, 7.68） |

| | | | | |
|---|---|---|---|---|
| Eritrea | 65 to 69 | T1D | Female | 7.05 (6.08, 8.18） |
| Eritrea | 65 to 69 | T1D | Male | 6.75 (5.75, 7.94） |
| Estonia | 0 to 4 | T1D | Both | 0.22 (0.17, 0.29） |
| Estonia | 0 to 4 | T1D | Female | 0.22 (0.15, 0.33） |
| Estonia | 0 to 4 | T1D | Male | 0.22 (0.15, 0.33） |
| Estonia | 10 to 14 | T1D | Both | 2.09 (1.83, 2.38） |
| Estonia | 10 to 14 | T1D | Female | 2.10 (1.74, 2.54） |
| Estonia | 10 to 14 | T1D | Male | 2.07 (1.72, 2.50） |
| Estonia | 15 to 19 | T1D | Both | 3.10 (2.72, 3.54） |
| Estonia | 15 to 19 | T1D | Female | 3.01 (2.50, 3.63） |
| Estonia | 15 to 19 | T1D | Male | 3.18 (2.65, 3.83） |
| Estonia | 20 to 24 | T1D | Both | 3.89 (3.41, 4.43） |
| Estonia | 20 to 24 | T1D | Female | 3.64 (3.03, 4.38） |
| Estonia | 20 to 24 | T1D | Male | 4.12 (3.43, 4.95） |
| Estonia | 25 to 29 | T1D | Both | 4.71 (4.13, 5.37） |
| Estonia | 25 to 29 | T1D | Female | 4.31 (3.58, 5.20） |
| Estonia | 25 to 29 | T1D | Male | 5.11 (4.25, 6.14） |
| Estonia | 30 to 34 | T1D | Both | 5.50 (4.82, 6.28） |
| Estonia | 30 to 34 | T1D | Female | 4.95 (4.10, 5.99） |
| Estonia | 30 to 34 | T1D | Male | 6.05 (5.02, 7.29） |
| Estonia | 35 to 39 | T1D | Both | 6.30 (5.50, 7.22） |
| Estonia | 35 to 39 | T1D | Female | 5.62 (4.63, 6.81） |
| Estonia | 35 to 39 | T1D | Male | 6.98 (5.77, 8.44） |
| Estonia | 40 to 44 | T1D | Both | 7.14 (6.23, 8.19） |
| Estonia | 40 to 44 | T1D | Female | 6.35 (5.22, 7.72） |
| Estonia | 40 to 44 | T1D | Male | 7.95 (6.56, 9.64） |
| Estonia | 45 to 49 | T1D | Both | 8.05 (7.00, 9.25） |
| Estonia | 45 to 49 | T1D | Female | 7.18 (5.89, 8.75） |
| Estonia | 45 to 49 | T1D | Male | 8.94 (7.36, 10.87） |
| Estonia | 5 to 9 | T1D | Both | 1.00 (1.00, 1.00） |
| Estonia | 5 to 9 | T1D | Female | 1.00 (1.00, 1.00） |
| Estonia | 5 to 9 | T1D | Male | 1.00 (1.00, 1.00） |
| Estonia | 50 to 54 | T1D | Both | 8.99 (7.81, 10.35） |

| | | | | |
|---|---|---|---|---|
| Estonia | 50 to 54 | T1D | Female | 8.09 (6.61, 9.88） |
| Estonia | 50 to 54 | T1D | Male | 9.97 (8.18, 12.15） |
| Estonia | 55 to 59 | T1D | Both | 10.02 (8.69, 11.56） |
| Estonia | 55 to 59 | T1D | Female | 9.11 (7.43, 11.16） |
| Estonia | 55 to 59 | T1D | Male | 11.06 (9.05, 13.52） |
| Estonia | 60 to 64 | T1D | Both | 11.15 (9.64, 12.88） |
| Estonia | 60 to 64 | T1D | Female | 10.30 (8.38, 12.66） |
| Estonia | 60 to 64 | T1D | Male | 12.18 (9.93, 14.93） |
| Estonia | 65 to 69 | T1D | Both | 12.40 (10.69, 14.37） |
| Estonia | 65 to 69 | T1D | Female | 11.65 (9.44, 14.36） |
| Estonia | 65 to 69 | T1D | Male | 13.39 (10.86, 16.51） |
| Eswatini | 0 to 4 | T1D | Both | 0.16 (0.13, 0.21） |
| Eswatini | 0 to 4 | T1D | Female | 0.17 (0.12, 0.24） |
| Eswatini | 0 to 4 | T1D | Male | 0.16 (0.11, 0.22） |
| Eswatini | 10 to 14 | T1D | Both | 2.07 (1.84, 2.33） |
| Eswatini | 10 to 14 | T1D | Female | 2.07 (1.75, 2.45） |
| Eswatini | 10 to 14 | T1D | Male | 2.07 (1.75, 2.44） |
| Eswatini | 15 to 19 | T1D | Both | 3.01 (2.68, 3.39） |
| Eswatini | 15 to 19 | T1D | Female | 3.02 (2.55, 3.57） |
| Eswatini | 15 to 19 | T1D | Male | 3.02 (2.56, 3.57） |
| Eswatini | 20 to 24 | T1D | Both | 3.81 (3.37, 4.30） |
| Eswatini | 20 to 24 | T1D | Female | 3.82 (3.22, 4.54） |
| Eswatini | 20 to 24 | T1D | Male | 3.83 (3.22, 4.54） |
| Eswatini | 25 to 29 | T1D | Both | 4.64 (4.09, 5.26） |
| Eswatini | 25 to 29 | T1D | Female | 4.66 (3.90, 5.57） |
| Eswatini | 25 to 29 | T1D | Male | 4.65 (3.90, 5.56） |
| Eswatini | 30 to 34 | T1D | Both | 5.23 (4.59, 5.97） |
| Eswatini | 30 to 34 | T1D | Female | 5.28 (4.39, 6.35） |
| Eswatini | 30 to 34 | T1D | Male | 5.24 (4.35, 6.31） |
| Eswatini | 35 to 39 | T1D | Both | 5.67 (4.94, 6.51） |
| Eswatini | 35 to 39 | T1D | Female | 5.72 (4.71, 6.95） |
| Eswatini | 35 to 39 | T1D | Male | 5.67 (4.66, 6.90） |
| Eswatini | 40 to 44 | T1D | Both | 6.04 (5.22, 6.99） |

| | | | | |
|---|---|---|---|---|
| Eswatini | 40 to 44 | T1D | Female | 6.08 (4.95, 7.46） |
| Eswatini | 40 to 44 | T1D | Male | 6.02 (4.89, 7.40） |
| Eswatini | 45 to 49 | T1D | Both | 6.35 (5.44, 7.42） |
| Eswatini | 45 to 49 | T1D | Female | 6.43 (5.17, 7.98） |
| Eswatini | 45 to 49 | T1D | Male | 6.34 (5.08, 7.91） |
| Eswatini | 5 to 9 | T1D | Both | 1.00 (1.00, 1.00） |
| Eswatini | 5 to 9 | T1D | Female | 1.00 (1.00, 1.00） |
| Eswatini | 5 to 9 | T1D | Male | 1.00 (1.00, 1.00） |
| Eswatini | 50 to 54 | T1D | Both | 6.65 (5.64, 7.85） |
| Eswatini | 50 to 54 | T1D | Female | 6.76 (5.37, 8.51） |
| Eswatini | 50 to 54 | T1D | Male | 6.59 (5.20, 8.35） |
| Eswatini | 55 to 59 | T1D | Both | 6.90 (5.79, 8.24） |
| Eswatini | 55 to 59 | T1D | Female | 7.05 (5.51, 9.00） |
| Eswatini | 55 to 59 | T1D | Male | 6.80 (5.27, 8.77） |
| Eswatini | 60 to 64 | T1D | Both | 7.12 (5.89, 8.61） |
| Eswatini | 60 to 64 | T1D | Female | 7.24 (5.57, 9.41） |
| Eswatini | 60 to 64 | T1D | Male | 7.05 (5.35, 9.30） |
| Eswatini | 65 to 69 | T1D | Both | 7.28 (5.91, 8.97） |
| Eswatini | 65 to 69 | T1D | Female | 7.45 (5.61, 9.90） |
| Eswatini | 65 to 69 | T1D | Male | 7.17 (5.26, 9.76） |
| Ethiopia | 0 to 4 | T1D | Both | 0.13 (0.13, 0.14） |
| Ethiopia | 0 to 4 | T1D | Female | 0.13 (0.13, 0.14） |
| Ethiopia | 0 to 4 | T1D | Male | 0.13 (0.13, 0.14） |
| Ethiopia | 10 to 14 | T1D | Both | 2.16 (2.14, 2.19） |
| Ethiopia | 10 to 14 | T1D | Female | 2.16 (2.12, 2.21） |
| Ethiopia | 10 to 14 | T1D | Male | 2.16 (2.12, 2.21） |
| Ethiopia | 15 to 19 | T1D | Both | 3.18 (3.14, 3.23） |
| Ethiopia | 15 to 19 | T1D | Female | 3.18 (3.12, 3.25） |
| Ethiopia | 15 to 19 | T1D | Male | 3.18 (3.12, 3.25） |
| Ethiopia | 20 to 24 | T1D | Both | 4.12 (4.06, 4.18） |
| Ethiopia | 20 to 24 | T1D | Female | 4.12 (4.04, 4.21） |
| Ethiopia | 20 to 24 | T1D | Male | 4.12 (4.04, 4.20） |
| Ethiopia | 25 to 29 | T1D | Both | 5.08 (5.00, 5.15） |

| | | | | |
|---|---|---|---|---|
| Ethiopia | 25 to 29 | T1D | Female | 5.09 (4.98, 5.20） |
| Ethiopia | 25 to 29 | T1D | Male | 5.08 (4.97, 5.18） |
| Ethiopia | 30 to 34 | T1D | Both | 5.63 (5.54, 5.72） |
| Ethiopia | 30 to 34 | T1D | Female | 5.65 (5.53, 5.78） |
| Ethiopia | 30 to 34 | T1D | Male | 5.63 (5.50, 5.75） |
| Ethiopia | 35 to 39 | T1D | Both | 6.00 (5.90, 6.10） |
| Ethiopia | 35 to 39 | T1D | Female | 6.03 (5.89, 6.18） |
| Ethiopia | 35 to 39 | T1D | Male | 5.98 (5.84, 6.12） |
| Ethiopia | 40 to 44 | T1D | Both | 6.28 (6.18, 6.39） |
| Ethiopia | 40 to 44 | T1D | Female | 6.33 (6.17, 6.49） |
| Ethiopia | 40 to 44 | T1D | Male | 6.24 (6.09, 6.39） |
| Ethiopia | 45 to 49 | T1D | Both | 6.49 (6.37, 6.62） |
| Ethiopia | 45 to 49 | T1D | Female | 6.55 (6.38, 6.73） |
| Ethiopia | 45 to 49 | T1D | Male | 6.43 (6.26, 6.60） |
| Ethiopia | 5 to 9 | T1D | Both | 1.00 (1.00, 1.00） |
| Ethiopia | 5 to 9 | T1D | Female | 1.00 (1.00, 1.00） |
| Ethiopia | 5 to 9 | T1D | Male | 1.00 (1.00, 1.00） |
| Ethiopia | 50 to 54 | T1D | Both | 6.64 (6.51, 6.77） |
| Ethiopia | 50 to 54 | T1D | Female | 6.72 (6.53, 6.92） |
| Ethiopia | 50 to 54 | T1D | Male | 6.55 (6.37, 6.73） |
| Ethiopia | 55 to 59 | T1D | Both | 6.74 (6.59, 6.88） |
| Ethiopia | 55 to 59 | T1D | Female | 6.84 (6.64, 7.06） |
| Ethiopia | 55 to 59 | T1D | Male | 6.62 (6.43, 6.82） |
| Ethiopia | 60 to 64 | T1D | Both | 6.79 (6.63, 6.95） |
| Ethiopia | 60 to 64 | T1D | Female | 6.92 (6.69, 7.16） |
| Ethiopia | 60 to 64 | T1D | Male | 6.65 (6.44, 6.87） |
| Ethiopia | 65 to 69 | T1D | Both | 6.78 (6.61, 6.96） |
| Ethiopia | 65 to 69 | T1D | Female | 6.95 (6.69, 7.22） |
| Ethiopia | 65 to 69 | T1D | Male | 6.62 (6.38, 6.86） |
| Fiji | 0 to 4 | T1D | Both | 0.17 (0.11, 0.28） |
| Fiji | 0 to 4 | T1D | Female | 0.18 (0.09, 0.34） |
| Fiji | 0 to 4 | T1D | Male | 0.17 (0.09, 0.33） |
| Fiji | 10 to 14 | T1D | Both | 2.01 (1.60, 2.51） |

| | | | | |
|---|---|---|---|---|
| Fiji | 10 to 14 | T1D | Female | 1.99 (1.44, 2.73） |
| Fiji | 10 to 14 | T1D | Male | 1.97 (1.44, 2.71） |
| Fiji | 15 to 19 | T1D | Both | 2.90 (2.32, 3.64） |
| Fiji | 15 to 19 | T1D | Female | 2.81 (2.04, 3.86） |
| Fiji | 15 to 19 | T1D | Male | 2.94 (2.15, 4.03） |
| Fiji | 20 to 24 | T1D | Both | 3.76 (2.99, 4.72） |
| Fiji | 20 to 24 | T1D | Female | 3.54 (2.56, 4.89） |
| Fiji | 20 to 24 | T1D | Male | 3.93 (2.86, 5.40） |
| Fiji | 25 to 29 | T1D | Both | 4.65 (3.69, 5.87） |
| Fiji | 25 to 29 | T1D | Female | 4.21 (3.02, 5.86） |
| Fiji | 25 to 29 | T1D | Male | 4.98 (3.60, 6.88） |
| Fiji | 30 to 34 | T1D | Both | 5.39 (4.24, 6.84） |
| Fiji | 30 to 34 | T1D | Female | 4.80 (3.41, 6.75） |
| Fiji | 30 to 34 | T1D | Male | 5.84 (4.19, 8.15） |
| Fiji | 35 to 39 | T1D | Both | 6.04 (4.72, 7.73） |
| Fiji | 35 to 39 | T1D | Female | 5.35 (3.76, 7.61） |
| Fiji | 35 to 39 | T1D | Male | 6.62 (4.70, 9.33） |
| Fiji | 40 to 44 | T1D | Both | 6.70 (5.20, 8.64） |
| Fiji | 40 to 44 | T1D | Female | 5.87 (4.08, 8.45） |
| Fiji | 40 to 44 | T1D | Male | 7.38 (5.18, 10.50） |
| Fiji | 45 to 49 | T1D | Both | 7.36 (5.67, 9.56） |
| Fiji | 45 to 49 | T1D | Female | 6.39 (4.39, 9.32） |
| Fiji | 45 to 49 | T1D | Male | 8.17 (5.69, 11.75） |
| Fiji | 5 to 9 | T1D | Both | 1.00 (1.00, 1.00） |
| Fiji | 5 to 9 | T1D | Female | 1.00 (1.00, 1.00） |
| Fiji | 5 to 9 | T1D | Male | 1.00 (1.00, 1.00） |
| Fiji | 50 to 54 | T1D | Both | 8.05 (6.14, 10.56） |
| Fiji | 50 to 54 | T1D | Female | 7.01 (4.74, 10.35） |
| Fiji | 50 to 54 | T1D | Male | 8.93 (6.13, 12.99） |
| Fiji | 55 to 59 | T1D | Both | 8.70 (6.55, 11.54） |
| Fiji | 55 to 59 | T1D | Female | 7.60 (5.05, 11.43） |
| Fiji | 55 to 59 | T1D | Male | 9.55 (6.45, 14.12） |
| Fiji | 60 to 64 | T1D | Both | 9.31 (6.90, 12.57） |

| | | | | |
|---|---|---|---|---|
| Fiji | 60 to 64 | T1D | Female | 8.27 (5.37, 12.74） |
| Fiji | 60 to 64 | T1D | Male | 10.27 (6.78, 15.57） |
| Fiji | 65 to 69 | T1D | Both | 9.91 (7.16, 13.72） |
| Fiji | 65 to 69 | T1D | Female | 8.75 (5.48, 13.96） |
| Fiji | 65 to 69 | T1D | Male | 10.92 (6.94, 17.16） |
| Finland | 0 to 4 | T1D | Both | 0.27 (0.25, 0.29） |
| Finland | 0 to 4 | T1D | Female | 0.27 (0.23, 0.31） |
| Finland | 0 to 4 | T1D | Male | 0.27 (0.25, 0.29） |
| Finland | 10 to 14 | T1D | Both | 1.75 (1.66, 1.83） |
| Finland | 10 to 14 | T1D | Female | 1.71 (1.58, 1.86） |
| Finland | 10 to 14 | T1D | Male | 1.78 (1.70, 1.86） |
| Finland | 15 to 19 | T1D | Both | 2.46 (2.34, 2.58） |
| Finland | 15 to 19 | T1D | Female | 2.33 (2.15, 2.53） |
| Finland | 15 to 19 | T1D | Male | 2.58 (2.46, 2.70） |
| Finland | 20 to 24 | T1D | Both | 3.04 (2.89, 3.19） |
| Finland | 20 to 24 | T1D | Female | 2.76 (2.54, 3.00） |
| Finland | 20 to 24 | T1D | Male | 3.31 (3.16, 3.47） |
| Finland | 25 to 29 | T1D | Both | 3.51 (3.34, 3.68） |
| Finland | 25 to 29 | T1D | Female | 3.04 (2.79, 3.30） |
| Finland | 25 to 29 | T1D | Male | 3.99 (3.80, 4.18） |
| Finland | 30 to 34 | T1D | Both | 3.94 (3.74, 4.14） |
| Finland | 30 to 34 | T1D | Female | 3.26 (2.99, 3.55） |
| Finland | 30 to 34 | T1D | Male | 4.66 (4.43, 4.89） |
| Finland | 35 to 39 | T1D | Both | 4.34 (4.13, 4.57） |
| Finland | 35 to 39 | T1D | Female | 3.44 (3.15, 3.75） |
| Finland | 35 to 39 | T1D | Male | 5.33 (5.07, 5.60） |
| Finland | 40 to 44 | T1D | Both | 4.73 (4.49, 4.98） |
| Finland | 40 to 44 | T1D | Female | 3.59 (3.28, 3.92） |
| Finland | 40 to 44 | T1D | Male | 6.01 (5.72, 6.32） |
| Finland | 45 to 49 | T1D | Both | 5.10 (4.84, 5.38） |
| Finland | 45 to 49 | T1D | Female | 3.72 (3.40, 4.07） |
| Finland | 45 to 49 | T1D | Male | 6.72 (6.38, 7.07） |
| Finland | 5 to 9 | T1D | Both | 1.00 (1.00, 1.00） |

| | | | | |
|---|---|---|---|---|
| Finland | 5 to 9 | T1D | Female | 1.00 (1.00, 1.00） |
| Finland | 5 to 9 | T1D | Male | 1.00 (1.00, 1.00） |
| Finland | 50 to 54 | T1D | Both | 5.46 (5.18, 5.76） |
| Finland | 50 to 54 | T1D | Female | 3.84 (3.51, 4.21） |
| Finland | 50 to 54 | T1D | Male | 7.43 (7.06, 7.83） |
| Finland | 55 to 59 | T1D | Both | 5.82 (5.51, 6.14） |
| Finland | 55 to 59 | T1D | Female | 3.94 (3.59, 4.32） |
| Finland | 55 to 59 | T1D | Male | 8.18 (7.76, 8.62） |
| Finland | 60 to 64 | T1D | Both | 6.14 (5.81, 6.49） |
| Finland | 60 to 64 | T1D | Female | 4.02 (3.66, 4.42） |
| Finland | 60 to 64 | T1D | Male | 8.93 (8.46, 9.42） |
| Finland | 65 to 69 | T1D | Both | 6.49 (6.13, 6.87） |
| Finland | 65 to 69 | T1D | Female | 4.13 (3.76, 4.55） |
| Finland | 65 to 69 | T1D | Male | 9.72 (9.20, 10.26） |
| France | 0 to 4 | T1D | Both | 0.18 (0.17, 0.18） |
| France | 0 to 4 | T1D | Female | 0.17 (0.16, 0.18） |
| France | 0 to 4 | T1D | Male | 0.18 (0.17, 0.19） |
| France | 10 to 14 | T1D | Both | 2.23 (2.19, 2.28） |
| France | 10 to 14 | T1D | Female | 2.15 (2.09, 2.21） |
| France | 10 to 14 | T1D | Male | 2.31 (2.26, 2.36） |
| France | 15 to 19 | T1D | Both | 3.61 (3.54, 3.69） |
| France | 15 to 19 | T1D | Female | 3.38 (3.28, 3.47） |
| France | 15 to 19 | T1D | Male | 3.87 (3.78, 3.96） |
| France | 20 to 24 | T1D | Both | 5.09 (4.98, 5.19） |
| France | 20 to 24 | T1D | Female | 4.63 (4.50, 4.77） |
| France | 20 to 24 | T1D | Male | 5.58 (5.45, 5.71） |
| France | 25 to 29 | T1D | Both | 6.91 (6.77, 7.06） |
| France | 25 to 29 | T1D | Female | 6.17 (5.99, 6.35） |
| France | 25 to 29 | T1D | Male | 7.74 (7.56, 7.92） |
| France | 30 to 34 | T1D | Both | 9.04 (8.84, 9.24） |
| France | 30 to 34 | T1D | Female | 7.94 (7.70, 8.18） |
| France | 30 to 34 | T1D | Male | 10.26 (10.02, 10.51） |
| France | 35 to 39 | T1D | Both | 11.59 (11.34, 11.85） |

| | | | | |
|---|---|---|---|---|
| France | 35 to 39 | T1D | Female | 10.06 (9.75, 10.37） |
| France | 35 to 39 | T1D | Male | 13.32 (12.99, 13.65） |
| France | 40 to 44 | T1D | Both | 14.68 (14.35, 15.02） |
| France | 40 to 44 | T1D | Female | 12.62 (12.22, 13.03） |
| France | 40 to 44 | T1D | Male | 17.01 (16.59, 17.44） |
| France | 45 to 49 | T1D | Both | 18.37 (17.95, 18.81） |
| France | 45 to 49 | T1D | Female | 15.70 (15.20, 16.22） |
| France | 45 to 49 | T1D | Male | 21.41 (20.88, 21.96） |
| France | 5 to 9 | T1D | Both | 1.00 (1.00, 1.00） |
| France | 5 to 9 | T1D | Female | 1.00 (1.00, 1.00） |
| France | 5 to 9 | T1D | Male | 1.00 (1.00, 1.00） |
| France | 50 to 54 | T1D | Both | 22.76 (22.22, 23.30） |
| France | 50 to 54 | T1D | Female | 19.38 (18.75, 20.03） |
| France | 50 to 54 | T1D | Male | 26.62 (25.94, 27.31） |
| France | 55 to 59 | T1D | Both | 27.95 (27.28, 28.63） |
| France | 55 to 59 | T1D | Female | 23.79 (22.99, 24.61） |
| France | 55 to 59 | T1D | Male | 32.77 (31.92, 33.64） |
| France | 60 to 64 | T1D | Both | 34.09 (33.26, 34.95） |
| France | 60 to 64 | T1D | Female | 29.03 (28.04, 30.06） |
| France | 60 to 64 | T1D | Male | 40.04 (38.98, 41.13） |
| France | 65 to 69 | T1D | Both | 41.36 (40.33, 42.42） |
| France | 65 to 69 | T1D | Female | 35.28 (34.04, 36.55） |
| France | 65 to 69 | T1D | Male | 48.69 (47.37, 50.05） |
| Gabon | 0 to 4 | T1D | Both | 0.16 (0.13, 0.20） |
| Gabon | 0 to 4 | T1D | Female | 0.16 (0.12, 0.22） |
| Gabon | 0 to 4 | T1D | Male | 0.16 (0.12, 0.22） |
| Gabon | 10 to 14 | T1D | Both | 2.08 (1.87, 2.32） |
| Gabon | 10 to 14 | T1D | Female | 2.08 (1.79, 2.43） |
| Gabon | 10 to 14 | T1D | Male | 2.07 (1.78, 2.41） |
| Gabon | 15 to 19 | T1D | Both | 3.07 (2.76, 3.43） |
| Gabon | 15 to 19 | T1D | Female | 3.07 (2.64, 3.59） |
| Gabon | 15 to 19 | T1D | Male | 3.07 (2.64, 3.58） |
| Gabon | 20 to 24 | T1D | Both | 3.94 (3.53, 4.41） |

| | | | | |
|---|---|---|---|---|
| Gabon | 20 to 24 | T1D | Female | 3.95 (3.38, 4.63） |
| Gabon | 20 to 24 | T1D | Male | 3.93 (3.36, 4.60） |
| Gabon | 25 to 29 | T1D | Both | 4.85 (4.32, 5.43） |
| Gabon | 25 to 29 | T1D | Female | 4.87 (4.14, 5.73） |
| Gabon | 25 to 29 | T1D | Male | 4.82 (4.10, 5.67） |
| Gabon | 30 to 34 | T1D | Both | 5.54 (4.92, 6.24） |
| Gabon | 30 to 34 | T1D | Female | 5.58 (4.72, 6.60） |
| Gabon | 30 to 34 | T1D | Male | 5.51 (4.66, 6.52） |
| Gabon | 35 to 39 | T1D | Both | 6.12 (5.40, 6.92） |
| Gabon | 35 to 39 | T1D | Female | 6.17 (5.18, 7.35） |
| Gabon | 35 to 39 | T1D | Male | 6.06 (5.09, 7.21） |
| Gabon | 40 to 44 | T1D | Both | 6.62 (5.82, 7.53） |
| Gabon | 40 to 44 | T1D | Female | 6.67 (5.56, 8.01） |
| Gabon | 40 to 44 | T1D | Male | 6.55 (5.47, 7.85） |
| Gabon | 45 to 49 | T1D | Both | 7.12 (6.22, 8.14） |
| Gabon | 45 to 49 | T1D | Female | 7.21 (5.95, 8.73） |
| Gabon | 45 to 49 | T1D | Male | 7.03 (5.82, 8.48） |
| Gabon | 5 to 9 | T1D | Both | 1.00 (1.00, 1.00） |
| Gabon | 5 to 9 | T1D | Female | 1.00 (1.00, 1.00） |
| Gabon | 5 to 9 | T1D | Male | 1.00 (1.00, 1.00） |
| Gabon | 50 to 54 | T1D | Both | 7.61 (6.61, 8.76） |
| Gabon | 50 to 54 | T1D | Female | 7.74 (6.32, 9.47） |
| Gabon | 50 to 54 | T1D | Male | 7.52 (6.18, 9.16） |
| Gabon | 55 to 59 | T1D | Both | 8.11 (6.99, 9.42） |
| Gabon | 55 to 59 | T1D | Female | 8.25 (6.65, 10.22） |
| Gabon | 55 to 59 | T1D | Male | 7.96 (6.46, 9.81） |
| Gabon | 60 to 64 | T1D | Both | 8.58 (7.31, 10.07） |
| Gabon | 60 to 64 | T1D | Female | 8.78 (6.99, 11.04） |
| Gabon | 60 to 64 | T1D | Male | 8.38 (6.70, 10.49） |
| Gabon | 65 to 69 | T1D | Both | 9.01 (7.57, 10.73） |
| Gabon | 65 to 69 | T1D | Female | 9.24 (7.22, 11.81） |
| Gabon | 65 to 69 | T1D | Male | 8.89 (6.93, 11.39） |
| Gambia | 0 to 4 | T1D | Both | 0.15 (0.13, 0.19） |

| | | | | |
|---|---|---|---|---|
| Gambia | 0 to 4 | T1D | Female | 0.16 (0.12, 0.21） |
| Gambia | 0 to 4 | T1D | Male | 0.15 (0.12, 0.21） |
| Gambia | 10 to 14 | T1D | Both | 2.09 (1.89, 2.32） |
| Gambia | 10 to 14 | T1D | Female | 2.09 (1.81, 2.42） |
| Gambia | 10 to 14 | T1D | Male | 2.10 (1.82, 2.42） |
| Gambia | 15 to 19 | T1D | Both | 3.07 (2.77, 3.40） |
| Gambia | 15 to 19 | T1D | Female | 3.08 (2.65, 3.56） |
| Gambia | 15 to 19 | T1D | Male | 3.07 (2.65, 3.54） |
| Gambia | 20 to 24 | T1D | Both | 3.93 (3.53, 4.37） |
| Gambia | 20 to 24 | T1D | Female | 3.94 (3.39, 4.58） |
| Gambia | 20 to 24 | T1D | Male | 3.92 (3.38, 4.55） |
| Gambia | 25 to 29 | T1D | Both | 4.82 (4.32, 5.38） |
| Gambia | 25 to 29 | T1D | Female | 4.85 (4.15, 5.67） |
| Gambia | 25 to 29 | T1D | Male | 4.81 (4.12, 5.61） |
| Gambia | 30 to 34 | T1D | Both | 5.42 (4.84, 6.08） |
| Gambia | 30 to 34 | T1D | Female | 5.46 (4.64, 6.43） |
| Gambia | 30 to 34 | T1D | Male | 5.39 (4.59, 6.33） |
| Gambia | 35 to 39 | T1D | Both | 5.89 (5.22, 6.65） |
| Gambia | 35 to 39 | T1D | Female | 5.94 (5.01, 7.06） |
| Gambia | 35 to 39 | T1D | Male | 5.85 (4.93, 6.93） |
| Gambia | 40 to 44 | T1D | Both | 6.31 (5.55, 7.16） |
| Gambia | 40 to 44 | T1D | Female | 6.37 (5.31, 7.63） |
| Gambia | 40 to 44 | T1D | Male | 6.23 (5.21, 7.45） |
| Gambia | 45 to 49 | T1D | Both | 6.66 (5.81, 7.62） |
| Gambia | 45 to 49 | T1D | Female | 6.73 (5.55, 8.16） |
| Gambia | 45 to 49 | T1D | Male | 6.58 (5.44, 7.95） |
| Gambia | 5 to 9 | T1D | Both | 1.00 (1.00, 1.00） |
| Gambia | 5 to 9 | T1D | Female | 1.00 (1.00, 1.00） |
| Gambia | 5 to 9 | T1D | Male | 1.00 (1.00, 1.00） |
| Gambia | 50 to 54 | T1D | Both | 6.98 (6.03, 8.07） |
| Gambia | 50 to 54 | T1D | Female | 7.06 (5.73, 8.70） |
| Gambia | 50 to 54 | T1D | Male | 6.88 (5.62, 8.43） |
| Gambia | 55 to 59 | T1D | Both | 7.23 (6.18, 8.47） |

| | | | | |
|---|---|---|---|---|
| Gambia | 55 to 59 | T1D | Female | 7.33 (5.84, 9.21） |
| Gambia | 55 to 59 | T1D | Male | 7.13 (5.74, 8.87） |
| Gambia | 60 to 64 | T1D | Both | 7.45 (6.28, 8.82） |
| Gambia | 60 to 64 | T1D | Female | 7.59 (5.94, 9.71） |
| Gambia | 60 to 64 | T1D | Male | 7.33 (5.80, 9.27） |
| Gambia | 65 to 69 | T1D | Both | 7.62 (6.33, 9.18） |
| Gambia | 65 to 69 | T1D | Female | 7.72 (5.90, 10.11） |
| Gambia | 65 to 69 | T1D | Male | 7.50 (5.80, 9.70） |
| Georgia | 0 to 4 | T1D | Both | 0.18 (0.15, 0.21） |
| Georgia | 0 to 4 | T1D | Female | 0.17 (0.14, 0.22） |
| Georgia | 0 to 4 | T1D | Male | 0.18 (0.14, 0.22） |
| Georgia | 10 to 14 | T1D | Both | 2.19 (2.03, 2.35） |
| Georgia | 10 to 14 | T1D | Female | 2.18 (1.96, 2.42） |
| Georgia | 10 to 14 | T1D | Male | 2.20 (1.99, 2.43） |
| Georgia | 15 to 19 | T1D | Both | 3.40 (3.17, 3.66） |
| Georgia | 15 to 19 | T1D | Female | 3.35 (3.02, 3.72） |
| Georgia | 15 to 19 | T1D | Male | 3.46 (3.14, 3.83） |
| Georgia | 20 to 24 | T1D | Both | 4.52 (4.21, 4.86） |
| Georgia | 20 to 24 | T1D | Female | 4.37 (3.94, 4.85） |
| Georgia | 20 to 24 | T1D | Male | 4.69 (4.24, 5.18） |
| Georgia | 25 to 29 | T1D | Both | 5.76 (5.36, 6.20） |
| Georgia | 25 to 29 | T1D | Female | 5.46 (4.92, 6.06） |
| Georgia | 25 to 29 | T1D | Male | 6.09 (5.51, 6.73） |
| Georgia | 30 to 34 | T1D | Both | 6.88 (6.40, 7.41） |
| Georgia | 30 to 34 | T1D | Female | 6.43 (5.78, 7.15） |
| Georgia | 30 to 34 | T1D | Male | 7.38 (6.66, 8.17） |
| Georgia | 35 to 39 | T1D | Both | 7.95 (7.37, 8.57） |
| Georgia | 35 to 39 | T1D | Female | 7.35 (6.59, 8.19） |
| Georgia | 35 to 39 | T1D | Male | 8.60 (7.75, 9.55） |
| Georgia | 40 to 44 | T1D | Both | 9.00 (8.34, 9.72） |
| Georgia | 40 to 44 | T1D | Female | 8.26 (7.40, 9.22） |
| Georgia | 40 to 44 | T1D | Male | 9.82 (8.83, 10.91） |
| Georgia | 45 to 49 | T1D | Both | 10.09 (9.34, 10.91） |

| | | | | |
|---|---|---|---|---|
| Georgia | 45 to 49 | T1D | Female | 9.22 (8.24, 10.31） |
| Georgia | 45 to 49 | T1D | Male | 11.06 (9.93, 12.31） |
| Georgia | 5 to 9 | T1D | Both | 1.00 (1.00, 1.00） |
| Georgia | 5 to 9 | T1D | Female | 1.00 (1.00, 1.00） |
| Georgia | 5 to 9 | T1D | Male | 1.00 (1.00, 1.00） |
| Georgia | 50 to 54 | T1D | Both | 11.21 (10.36, 12.13） |
| Georgia | 50 to 54 | T1D | Female | 10.23 (9.13, 11.46） |
| Georgia | 50 to 54 | T1D | Male | 12.32 (11.04, 13.74） |
| Georgia | 55 to 59 | T1D | Both | 12.32 (11.37, 13.35） |
| Georgia | 55 to 59 | T1D | Female | 11.27 (10.04, 12.66） |
| Georgia | 55 to 59 | T1D | Male | 13.55 (12.12, 15.15） |
| Georgia | 60 to 64 | T1D | Both | 13.42 (12.36, 14.57） |
| Georgia | 60 to 64 | T1D | Female | 12.35 (10.98, 13.89） |
| Georgia | 60 to 64 | T1D | Male | 14.77 (13.18, 16.56） |
| Georgia | 65 to 69 | T1D | Both | 14.62 (13.44, 15.90） |
| Georgia | 65 to 69 | T1D | Female | 13.54 (12.00, 15.27） |
| Georgia | 65 to 69 | T1D | Male | 16.05 (14.27, 18.06） |
| Germany | 0 to 4 | T1D | Both | 0.18 (0.16, 0.19） |
| Germany | 0 to 4 | T1D | Female | 0.17 (0.16, 0.18） |
| Germany | 0 to 4 | T1D | Male | 0.18 (0.16, 0.19） |
| Germany | 10 to 14 | T1D | Both | 2.27 (2.20, 2.34） |
| Germany | 10 to 14 | T1D | Female | 2.24 (2.19, 2.29） |
| Germany | 10 to 14 | T1D | Male | 2.30 (2.21, 2.39） |
| Germany | 15 to 19 | T1D | Both | 3.42 (3.32, 3.53） |
| Germany | 15 to 19 | T1D | Female | 3.28 (3.21, 3.36） |
| Germany | 15 to 19 | T1D | Male | 3.57 (3.44, 3.72） |
| Germany | 20 to 24 | T1D | Both | 4.50 (4.37, 4.64） |
| Germany | 20 to 24 | T1D | Female | 4.20 (4.10, 4.30） |
| Germany | 20 to 24 | T1D | Male | 4.83 (4.64, 5.02） |
| Germany | 25 to 29 | T1D | Both | 5.76 (5.58, 5.94） |
| Germany | 25 to 29 | T1D | Female | 5.24 (5.12, 5.36） |
| Germany | 25 to 29 | T1D | Male | 6.33 (6.08, 6.59） |
| Germany | 30 to 34 | T1D | Both | 7.11 (6.89, 7.34） |

| | | | | |
|---|---|---|---|---|
| Germany | 30 to 34 | T1D | Female | 6.34 (6.19, 6.49） |
| Germany | 30 to 34 | T1D | Male | 7.96 (7.64, 8.29） |
| Germany | 35 to 39 | T1D | Both | 8.67 (8.40, 8.96） |
| Germany | 35 to 39 | T1D | Female | 7.61 (7.43, 7.79） |
| Germany | 35 to 39 | T1D | Male | 9.87 (9.47, 10.29） |
| Germany | 40 to 44 | T1D | Both | 10.50 (10.16, 10.85） |
| Germany | 40 to 44 | T1D | Female | 9.07 (8.85, 9.29） |
| Germany | 40 to 44 | T1D | Male | 12.12 (11.62, 12.63） |
| Germany | 45 to 49 | T1D | Both | 12.57 (12.17, 13.00） |
| Germany | 45 to 49 | T1D | Female | 10.72 (10.46, 10.99） |
| Germany | 45 to 49 | T1D | Male | 14.69 (14.08, 15.32） |
| Germany | 5 to 9 | T1D | Both | 1.00 (1.00, 1.00） |
| Germany | 5 to 9 | T1D | Female | 1.00 (1.00, 1.00） |
| Germany | 5 to 9 | T1D | Male | 1.00 (1.00, 1.00） |
| Germany | 50 to 54 | T1D | Both | 14.93 (14.44, 15.44） |
| Germany | 50 to 54 | T1D | Female | 12.60 (12.29, 12.92） |
| Germany | 50 to 54 | T1D | Male | 17.62 (16.88, 18.39） |
| Germany | 55 to 59 | T1D | Both | 17.62 (17.03, 18.23） |
| Germany | 55 to 59 | T1D | Female | 14.74 (14.37, 15.12） |
| Germany | 55 to 59 | T1D | Male | 20.98 (20.08, 21.91） |
| Germany | 60 to 64 | T1D | Both | 20.73 (20.03, 21.47） |
| Germany | 60 to 64 | T1D | Female | 17.21 (16.77, 17.66） |
| Germany | 60 to 64 | T1D | Male | 24.90 (23.82, 26.03） |
| Germany | 65 to 69 | T1D | Both | 24.38 (23.54, 25.26） |
| Germany | 65 to 69 | T1D | Female | 20.08 (19.56, 20.62） |
| Germany | 65 to 69 | T1D | Male | 29.52 (28.22, 30.88） |
| Ghana | 0 to 4 | T1D | Both | 0.17 (0.16, 0.18） |
| Ghana | 0 to 4 | T1D | Female | 0.17 (0.16, 0.19） |
| Ghana | 0 to 4 | T1D | Male | 0.17 (0.16, 0.19） |
| Ghana | 10 to 14 | T1D | Both | 2.04 (1.98, 2.09） |
| Ghana | 10 to 14 | T1D | Female | 2.04 (1.96, 2.12） |
| Ghana | 10 to 14 | T1D | Male | 2.04 (1.96, 2.12） |
| Ghana | 15 to 19 | T1D | Both | 3.00 (2.92, 3.09） |

| | | | | |
|---|---|---|---|---|
| Ghana | 15 to 19 | T1D | Female | 3.01 (2.89, 3.12） |
| Ghana | 15 to 19 | T1D | Male | 3.00 (2.89, 3.12） |
| Ghana | 20 to 24 | T1D | Both | 3.87 (3.76, 3.98） |
| Ghana | 20 to 24 | T1D | Female | 3.89 (3.73, 4.04） |
| Ghana | 20 to 24 | T1D | Male | 3.86 (3.72, 4.02） |
| Ghana | 25 to 29 | T1D | Both | 4.79 (4.66, 4.93） |
| Ghana | 25 to 29 | T1D | Female | 4.83 (4.64, 5.03） |
| Ghana | 25 to 29 | T1D | Male | 4.78 (4.59, 4.97） |
| Ghana | 30 to 34 | T1D | Both | 5.44 (5.28, 5.60） |
| Ghana | 30 to 34 | T1D | Female | 5.49 (5.26, 5.72） |
| Ghana | 30 to 34 | T1D | Male | 5.41 (5.19, 5.63） |
| Ghana | 35 to 39 | T1D | Both | 5.95 (5.77, 6.14） |
| Ghana | 35 to 39 | T1D | Female | 6.02 (5.76, 6.29） |
| Ghana | 35 to 39 | T1D | Male | 5.91 (5.66, 6.17） |
| Ghana | 40 to 44 | T1D | Both | 6.42 (6.22, 6.63） |
| Ghana | 40 to 44 | T1D | Female | 6.49 (6.20, 6.80） |
| Ghana | 40 to 44 | T1D | Male | 6.36 (6.08, 6.65） |
| Ghana | 45 to 49 | T1D | Both | 6.82 (6.60, 7.06） |
| Ghana | 45 to 49 | T1D | Female | 6.91 (6.59, 7.25） |
| Ghana | 45 to 49 | T1D | Male | 6.75 (6.44, 7.07） |
| Ghana | 5 to 9 | T1D | Both | 1.00 (1.00, 1.00） |
| Ghana | 5 to 9 | T1D | Female | 1.00 (1.00, 1.00） |
| Ghana | 5 to 9 | T1D | Male | 1.00 (1.00, 1.00） |
| Ghana | 50 to 54 | T1D | Both | 7.18 (6.93, 7.44） |
| Ghana | 50 to 54 | T1D | Female | 7.29 (6.93, 7.67） |
| Ghana | 50 to 54 | T1D | Male | 7.09 (6.74, 7.45） |
| Ghana | 55 to 59 | T1D | Both | 7.48 (7.20, 7.77） |
| Ghana | 55 to 59 | T1D | Female | 7.62 (7.22, 8.04） |
| Ghana | 55 to 59 | T1D | Male | 7.38 (6.99, 7.78） |
| Ghana | 60 to 64 | T1D | Both | 7.75 (7.44, 8.07） |
| Ghana | 60 to 64 | T1D | Female | 7.91 (7.47, 8.37） |
| Ghana | 60 to 64 | T1D | Male | 7.62 (7.20, 8.07） |
| Ghana | 65 to 69 | T1D | Both | 7.98 (7.63, 8.34） |

| | | | | |
|---|---|---|---|---|
| Ghana | 65 to 69 | T1D | Female | 8.16 (7.67, 8.69） |
| Ghana | 65 to 69 | T1D | Male | 7.84 (7.36, 8.35） |
| Greece | 0 to 4 | T1D | Both | 0.17 (0.15, 0.18） |
| Greece | 0 to 4 | T1D | Female | 0.16 (0.14, 0.18） |
| Greece | 0 to 4 | T1D | Male | 0.18 (0.16, 0.20） |
| Greece | 10 to 14 | T1D | Both | 2.19 (2.11, 2.28） |
| Greece | 10 to 14 | T1D | Female | 2.17 (2.05, 2.29） |
| Greece | 10 to 14 | T1D | Male | 2.22 (2.10, 2.35） |
| Greece | 15 to 19 | T1D | Both | 3.36 (3.23, 3.49） |
| Greece | 15 to 19 | T1D | Female | 3.22 (3.05, 3.40） |
| Greece | 15 to 19 | T1D | Male | 3.49 (3.31, 3.69） |
| Greece | 20 to 24 | T1D | Both | 4.52 (4.35, 4.70） |
| Greece | 20 to 24 | T1D | Female | 4.23 (4.00, 4.47） |
| Greece | 20 to 24 | T1D | Male | 4.83 (4.57, 5.10） |
| Greece | 25 to 29 | T1D | Both | 5.98 (5.75, 6.22） |
| Greece | 25 to 29 | T1D | Female | 5.44 (5.14, 5.75） |
| Greece | 25 to 29 | T1D | Male | 6.54 (6.19, 6.92） |
| Greece | 30 to 34 | T1D | Both | 7.64 (7.34, 7.96） |
| Greece | 30 to 34 | T1D | Female | 6.83 (6.45, 7.23） |
| Greece | 30 to 34 | T1D | Male | 8.51 (8.04, 9.00） |
| Greece | 35 to 39 | T1D | Both | 9.63 (9.24, 10.03） |
| Greece | 35 to 39 | T1D | Female | 8.48 (8.00, 8.99） |
| Greece | 35 to 39 | T1D | Male | 10.86 (10.25, 11.50） |
| Greece | 40 to 44 | T1D | Both | 12.03 (11.54, 12.54） |
| Greece | 40 to 44 | T1D | Female | 10.49 (9.89, 11.13） |
| Greece | 40 to 44 | T1D | Male | 13.70 (12.92, 14.52） |
| Greece | 45 to 49 | T1D | Both | 14.90 (14.29, 15.55） |
| Greece | 45 to 49 | T1D | Female | 12.90 (12.14, 13.71） |
| Greece | 45 to 49 | T1D | Male | 17.10 (16.12, 18.15） |
| Greece | 5 to 9 | T1D | Both | 1.00 (1.00, 1.00） |
| Greece | 5 to 9 | T1D | Female | 1.00 (1.00, 1.00） |
| Greece | 5 to 9 | T1D | Male | 1.00 (1.00, 1.00） |
| Greece | 50 to 54 | T1D | Both | 18.33 (17.56, 19.14） |

| | | | | |
|---|---|---|---|---|
| Greece | 50 to 54 | T1D | Female | 15.78 (14.84, 16.79） |
| Greece | 50 to 54 | T1D | Male | 21.17 (19.92, 22.49） |
| Greece | 55 to 59 | T1D | Both | 22.43 (21.46, 23.44） |
| Greece | 55 to 59 | T1D | Female | 19.25 (18.07, 20.50） |
| Greece | 55 to 59 | T1D | Male | 26.01 (24.46, 27.67） |
| Greece | 60 to 64 | T1D | Both | 27.29 (26.09, 28.54） |
| Greece | 60 to 64 | T1D | Female | 23.39 (21.93, 24.94） |
| Greece | 60 to 64 | T1D | Male | 31.74 (29.81, 33.80） |
| Greece | 65 to 69 | T1D | Both | 33.19 (31.70, 34.74） |
| Greece | 65 to 69 | T1D | Female | 28.47 (26.66, 30.39） |
| Greece | 65 to 69 | T1D | Male | 38.67 (36.27, 41.23） |
| Greenland | 0 to 4 | T1D | Both | 0.23 (0.12, 0.46） |
| Greenland | 0 to 4 | T1D | Female | 0.23 (0.09, 0.62） |
| Greenland | 0 to 4 | T1D | Male | 0.22 (0.08, 0.56） |
| Greenland | 10 to 14 | T1D | Both | 1.91 (1.33, 2.73） |
| Greenland | 10 to 14 | T1D | Female | 1.80 (1.08, 3.00） |
| Greenland | 10 to 14 | T1D | Male | 1.82 (1.11, 2.99） |
| Greenland | 15 to 19 | T1D | Both | 2.57 (1.79, 3.69） |
| Greenland | 15 to 19 | T1D | Female | 2.42 (1.45, 4.06） |
| Greenland | 15 to 19 | T1D | Male | 2.52 (1.53, 4.15） |
| Greenland | 20 to 24 | T1D | Both | 3.21 (2.24, 4.62） |
| Greenland | 20 to 24 | T1D | Female | 3.12 (1.86, 5.22） |
| Greenland | 20 to 24 | T1D | Male | 3.18 (1.93, 5.23） |
| Greenland | 25 to 29 | T1D | Both | 3.81 (2.64, 5.51） |
| Greenland | 25 to 29 | T1D | Female | 3.61 (2.13, 6.11） |
| Greenland | 25 to 29 | T1D | Male | 3.78 (2.28, 6.26） |
| Greenland | 30 to 34 | T1D | Both | 4.22 (2.90, 6.15） |
| Greenland | 30 to 34 | T1D | Female | 4.05 (2.36, 6.94） |
| Greenland | 30 to 34 | T1D | Male | 4.28 (2.56, 7.16） |
| Greenland | 35 to 39 | T1D | Both | 4.60 (3.13, 6.76） |
| Greenland | 35 to 39 | T1D | Female | 4.40 (2.53, 7.65） |
| Greenland | 35 to 39 | T1D | Male | 4.65 (2.75, 7.87） |
| Greenland | 40 to 44 | T1D | Both | 4.90 (3.31, 7.24） |

| | | | | |
|---|---|---|---|---|
| Greenland | 40 to 44 | T1D | Female | 4.69 (2.67, 8.25） |
| Greenland | 40 to 44 | T1D | Male | 5.00 (2.93, 8.53） |
| Greenland | 45 to 49 | T1D | Both | 5.20 (3.50, 7.73） |
| Greenland | 45 to 49 | T1D | Female | 4.85 (2.73, 8.61） |
| Greenland | 45 to 49 | T1D | Male | 5.35 (3.12, 9.19） |
| Greenland | 5 to 9 | T1D | Both | 1.00 (1.00, 1.00） |
| Greenland | 5 to 9 | T1D | Female | 1.00 (1.00, 1.00） |
| Greenland | 5 to 9 | T1D | Male | 1.00 (1.00, 1.00） |
| Greenland | 50 to 54 | T1D | Both | 5.45 (3.63, 8.17） |
| Greenland | 50 to 54 | T1D | Female | 5.12 (2.84, 9.23） |
| Greenland | 50 to 54 | T1D | Male | 5.54 (3.19, 9.62） |
| Greenland | 55 to 59 | T1D | Both | 5.67 (3.73, 8.63） |
| Greenland | 55 to 59 | T1D | Female | 5.22 (2.83, 9.65） |
| Greenland | 55 to 59 | T1D | Male | 5.77 (3.26, 10.19） |
| Greenland | 60 to 64 | T1D | Both | 5.91 (3.81, 9.16） |
| Greenland | 60 to 64 | T1D | Female | 5.47 (2.87, 10.42） |
| Greenland | 60 to 64 | T1D | Male | 5.92 (3.27, 10.72） |
| Greenland | 65 to 69 | T1D | Both | 6.11 (3.83, 9.76） |
| Greenland | 65 to 69 | T1D | Female | 5.54 (2.77, 11.05） |
| Greenland | 65 to 69 | T1D | Male | 6.10 (3.23, 11.51） |
| Grenada | 0 to 4 | T1D | Both | 0.25 (0.10, 0.66） |
| Grenada | 0 to 4 | T1D | Female | 0.05 (0.01, 0.85） |
| Grenada | 0 to 4 | T1D | Male | 0.05 (0.01, 0.85） |
| Grenada | 10 to 14 | T1D | Both | 2.27 (1.36, 3.80） |
| Grenada | 10 to 14 | T1D | Female | 2.48 (1.20, 5.11） |
| Grenada | 10 to 14 | T1D | Male | 2.09 (1.00, 4.38） |
| Grenada | 15 to 19 | T1D | Both | 3.39 (2.04, 5.64） |
| Grenada | 15 to 19 | T1D | Female | 3.66 (1.78, 7.53） |
| Grenada | 15 to 19 | T1D | Male | 3.08 (1.48, 6.40） |
| Grenada | 20 to 24 | T1D | Both | 4.33 (2.60, 7.23） |
| Grenada | 20 to 24 | T1D | Female | 4.63 (2.23, 9.61） |
| Grenada | 20 to 24 | T1D | Male | 4.12 (1.98, 8.58） |
| Grenada | 25 to 29 | T1D | Both | 5.28 (3.13, 8.92） |

| | | | | |
|---|---|---|---|---|
| Grenada | 25 to 29 | T1D | Female | 5.40 (2.55, 11.43） |
| Grenada | 25 to 29 | T1D | Male | 5.27 (2.49, 11.14） |
| Grenada | 30 to 34 | T1D | Both | 6.11 (3.53, 10.55） |
| Grenada | 30 to 34 | T1D | Female | 6.15 (2.81, 13.46） |
| Grenada | 30 to 34 | T1D | Male | 6.19 (2.85, 13.47） |
| Grenada | 35 to 39 | T1D | Both | 7.15 (4.06, 12.58） |
| Grenada | 35 to 39 | T1D | Female | 7.22 (3.21, 16.25） |
| Grenada | 35 to 39 | T1D | Male | 7.33 (3.29, 16.36） |
| Grenada | 40 to 44 | T1D | Both | 8.09 (4.54, 14.43） |
| Grenada | 40 to 44 | T1D | Female | 8.19 (3.57, 18.78） |
| Grenada | 40 to 44 | T1D | Male | 8.26 (3.64, 18.76） |
| Grenada | 45 to 49 | T1D | Both | 8.97 (4.95, 16.24） |
| Grenada | 45 to 49 | T1D | Female | 9.05 (3.85, 21.25） |
| Grenada | 45 to 49 | T1D | Male | 9.65 (4.18, 22.29） |
| Grenada | 5 to 9 | T1D | Both | 1.00 (1.00, 1.00） |
| Grenada | 5 to 9 | T1D | Female | 1.00 (1.00, 1.00） |
| Grenada | 5 to 9 | T1D | Male | 1.00 (1.00, 1.00） |
| Grenada | 50 to 54 | T1D | Both | 9.71 (5.28, 17.88） |
| Grenada | 50 to 54 | T1D | Female | 10.15 (4.22, 24.42） |
| Grenada | 50 to 54 | T1D | Male | 10.27 (4.35, 24.28） |
| Grenada | 55 to 59 | T1D | Both | 10.90 (5.78, 20.54） |
| Grenada | 55 to 59 | T1D | Female | 10.94 (4.38, 27.31） |
| Grenada | 55 to 59 | T1D | Male | 11.41 (4.68, 27.82） |
| Grenada | 60 to 64 | T1D | Both | 11.99 (6.15, 23.38） |
| Grenada | 60 to 64 | T1D | Female | 12.30 (4.71, 32.11） |
| Grenada | 60 to 64 | T1D | Male | 12.12 (4.75, 30.94） |
| Grenada | 65 to 69 | T1D | Both | 12.84 (6.29, 26.22） |
| Grenada | 65 to 69 | T1D | Female | 11.41 (4.07, 32.02） |
| Grenada | 65 to 69 | T1D | Male | 13.37 (4.95, 36.16） |
| Guam | 0 to 4 | T1D | Both | 0.23 (0.09, 0.61） |
| Guam | 0 to 4 | T1D | Female | 0.04 (0.01, 0.75） |
| Guam | 0 to 4 | T1D | Male | 0.04 (0.01, 0.75） |
| Guam | 10 to 14 | T1D | Both | 2.02 (1.18, 3.45） |

| | | | | |
|---|---|---|---|---|
| Guam | 10 to 14 | T1D | Female | 1.95 (0.92, 4.12） |
| Guam | 10 to 14 | T1D | Male | 1.97 (0.93, 4.17） |
| Guam | 15 to 19 | T1D | Both | 2.89 (1.69, 4.95） |
| Guam | 15 to 19 | T1D | Female | 2.69 (1.26, 5.72） |
| Guam | 15 to 19 | T1D | Male | 2.87 (1.36, 6.05） |
| Guam | 20 to 24 | T1D | Both | 3.75 (2.17, 6.47） |
| Guam | 20 to 24 | T1D | Female | 3.35 (1.55, 7.23） |
| Guam | 20 to 24 | T1D | Male | 3.84 (1.81, 8.13） |
| Guam | 25 to 29 | T1D | Both | 4.63 (2.65, 8.09） |
| Guam | 25 to 29 | T1D | Female | 4.12 (1.88, 9.03） |
| Guam | 25 to 29 | T1D | Male | 4.90 (2.28, 10.56） |
| Guam | 30 to 34 | T1D | Both | 5.30 (2.99, 9.41） |
| Guam | 30 to 34 | T1D | Female | 4.77 (2.13, 10.70） |
| Guam | 30 to 34 | T1D | Male | 5.47 (2.49, 12.03） |
| Guam | 35 to 39 | T1D | Both | 5.98 (3.33, 10.74） |
| Guam | 35 to 39 | T1D | Female | 4.97 (2.16, 11.41） |
| Guam | 35 to 39 | T1D | Male | 6.39 (2.87, 14.27） |
| Guam | 40 to 44 | T1D | Both | 6.56 (3.61, 11.92） |
| Guam | 40 to 44 | T1D | Female | 5.67 (2.42, 13.26） |
| Guam | 40 to 44 | T1D | Male | 7.13 (3.15, 16.13） |
| Guam | 45 to 49 | T1D | Both | 7.37 (4.01, 13.55） |
| Guam | 45 to 49 | T1D | Female | 6.41 (2.69, 15.27） |
| Guam | 45 to 49 | T1D | Male | 7.96 (3.47, 18.30） |
| Guam | 5 to 9 | T1D | Both | 1.00 (1.00, 1.00） |
| Guam | 5 to 9 | T1D | Female | 1.00 (1.00, 1.00） |
| Guam | 5 to 9 | T1D | Male | 1.00 (1.00, 1.00） |
| Guam | 50 to 54 | T1D | Both | 7.94 (4.25, 14.85） |
| Guam | 50 to 54 | T1D | Female | 6.91 (2.83, 16.91） |
| Guam | 50 to 54 | T1D | Male | 8.55 (3.63, 20.12） |
| Guam | 55 to 59 | T1D | Both | 8.51 (4.45, 16.25） |
| Guam | 55 to 59 | T1D | Female | 7.36 (2.91, 18.61） |
| Guam | 55 to 59 | T1D | Male | 9.05 (3.73, 21.94） |
| Guam | 60 to 64 | T1D | Both | 9.19 (4.69, 18.03） |

| | | | | |
|---|---|---|---|---|
| Guam | 60 to 64 | T1D | Female | 8.16 (3.11, 21.39） |
| Guam | 60 to 64 | T1D | Male | 9.96 (3.96, 25.05） |
| Guam | 65 to 69 | T1D | Both | 9.68 (4.74, 19.76） |
| Guam | 65 to 69 | T1D | Female | 8.45 (3.04, 23.50） |
| Guam | 65 to 69 | T1D | Male | 11.15 (4.22, 29.47） |
| Guatemala | 0 to 4 | T1D | Both | 0.13 (0.11, 0.14） |
| Guatemala | 0 to 4 | T1D | Female | 0.11 (0.10, 0.13） |
| Guatemala | 0 to 4 | T1D | Male | 0.14 (0.12, 0.16） |
| Guatemala | 10 to 14 | T1D | Both | 2.26 (2.17, 2.36） |
| Guatemala | 10 to 14 | T1D | Female | 2.44 (2.31, 2.59） |
| Guatemala | 10 to 14 | T1D | Male | 2.06 (1.94, 2.19） |
| Guatemala | 15 to 19 | T1D | Both | 3.05 (2.92, 3.18） |
| Guatemala | 15 to 19 | T1D | Female | 3.24 (3.06, 3.43） |
| Guatemala | 15 to 19 | T1D | Male | 2.82 (2.65, 3.00） |
| Guatemala | 20 to 24 | T1D | Both | 3.55 (3.40, 3.71） |
| Guatemala | 20 to 24 | T1D | Female | 3.66 (3.44, 3.88） |
| Guatemala | 20 to 24 | T1D | Male | 3.40 (3.19, 3.63） |
| Guatemala | 25 to 29 | T1D | Both | 4.02 (3.84, 4.21） |
| Guatemala | 25 to 29 | T1D | Female | 4.01 (3.77, 4.27） |
| Guatemala | 25 to 29 | T1D | Male | 3.98 (3.72, 4.27） |
| Guatemala | 30 to 34 | T1D | Both | 4.38 (4.17, 4.60） |
| Guatemala | 30 to 34 | T1D | Female | 4.28 (4.01, 4.58） |
| Guatemala | 30 to 34 | T1D | Male | 4.46 (4.14, 4.79） |
| Guatemala | 35 to 39 | T1D | Both | 4.69 (4.45, 4.94） |
| Guatemala | 35 to 39 | T1D | Female | 4.52 (4.22, 4.85） |
| Guatemala | 35 to 39 | T1D | Male | 4.85 (4.50, 5.24） |
| Guatemala | 40 to 44 | T1D | Both | 4.97 (4.70, 5.25） |
| Guatemala | 40 to 44 | T1D | Female | 4.74 (4.40, 5.11） |
| Guatemala | 40 to 44 | T1D | Male | 5.20 (4.79, 5.65） |
| Guatemala | 45 to 49 | T1D | Both | 5.22 (4.92, 5.54） |
| Guatemala | 45 to 49 | T1D | Female | 4.95 (4.57, 5.35） |
| Guatemala | 45 to 49 | T1D | Male | 5.51 (5.05, 6.01） |
| Guatemala | 5 to 9 | T1D | Both | 1.00 (1.00, 1.00） |

| | | | | |
|---|---|---|---|---|
| Guatemala | 5 to 9 | T1D | Female | 1.00 (1.00, 1.00） |
| Guatemala | 5 to 9 | T1D | Male | 1.00 (1.00, 1.00） |
| Guatemala | 50 to 54 | T1D | Both | 5.45 (5.12, 5.80） |
| Guatemala | 50 to 54 | T1D | Female | 5.15 (4.73, 5.60） |
| Guatemala | 50 to 54 | T1D | Male | 5.79 (5.28, 6.35） |
| Guatemala | 55 to 59 | T1D | Both | 5.66 (5.29, 6.05） |
| Guatemala | 55 to 59 | T1D | Female | 5.33 (4.86, 5.83） |
| Guatemala | 55 to 59 | T1D | Male | 6.02 (5.46, 6.65） |
| Guatemala | 60 to 64 | T1D | Both | 5.84 (5.44, 6.28） |
| Guatemala | 60 to 64 | T1D | Female | 5.50 (4.99, 6.07） |
| Guatemala | 60 to 64 | T1D | Male | 6.22 (5.60, 6.92） |
| Guatemala | 65 to 69 | T1D | Both | 6.02 (5.57, 6.51） |
| Guatemala | 65 to 69 | T1D | Female | 5.67 (5.09, 6.31） |
| Guatemala | 65 to 69 | T1D | Male | 6.41 (5.72, 7.20） |
| Guinea | 0 to 4 | T1D | Both | 0.15 (0.14, 0.17） |
| Guinea | 0 to 4 | T1D | Female | 0.15 (0.13, 0.17） |
| Guinea | 0 to 4 | T1D | Male | 0.15 (0.13, 0.17） |
| Guinea | 10 to 14 | T1D | Both | 2.11 (2.02, 2.20） |
| Guinea | 10 to 14 | T1D | Female | 2.11 (1.98, 2.24） |
| Guinea | 10 to 14 | T1D | Male | 2.11 (1.99, 2.23） |
| Guinea | 15 to 19 | T1D | Both | 3.05 (2.92, 3.18） |
| Guinea | 15 to 19 | T1D | Female | 3.06 (2.88, 3.25） |
| Guinea | 15 to 19 | T1D | Male | 3.05 (2.87, 3.24） |
| Guinea | 20 to 24 | T1D | Both | 3.85 (3.68, 4.02） |
| Guinea | 20 to 24 | T1D | Female | 3.87 (3.63, 4.12） |
| Guinea | 20 to 24 | T1D | Male | 3.84 (3.61, 4.09） |
| Guinea | 25 to 29 | T1D | Both | 4.64 (4.44, 4.86） |
| Guinea | 25 to 29 | T1D | Female | 4.68 (4.39, 4.99） |
| Guinea | 25 to 29 | T1D | Male | 4.63 (4.34, 4.94） |
| Guinea | 30 to 34 | T1D | Both | 5.13 (4.89, 5.38） |
| Guinea | 30 to 34 | T1D | Female | 5.18 (4.84, 5.54） |
| Guinea | 30 to 34 | T1D | Male | 5.10 (4.76, 5.46） |
| Guinea | 35 to 39 | T1D | Both | 5.45 (5.18, 5.73） |

| | | | | |
|---|---|---|---|---|
| Guinea | 35 to 39 | T1D | Female | 5.52 (5.14, 5.93） |
| Guinea | 35 to 39 | T1D | Male | 5.41 (5.03, 5.81） |
| Guinea | 40 to 44 | T1D | Both | 5.70 (5.40, 6.01） |
| Guinea | 40 to 44 | T1D | Female | 5.78 (5.36, 6.23） |
| Guinea | 40 to 44 | T1D | Male | 5.64 (5.23, 6.08） |
| Guinea | 45 to 49 | T1D | Both | 5.89 (5.56, 6.23） |
| Guinea | 45 to 49 | T1D | Female | 5.98 (5.52, 6.47） |
| Guinea | 45 to 49 | T1D | Male | 5.81 (5.36, 6.29） |
| Guinea | 5 to 9 | T1D | Both | 1.00 (1.00, 1.00） |
| Guinea | 5 to 9 | T1D | Female | 1.00 (1.00, 1.00） |
| Guinea | 5 to 9 | T1D | Male | 1.00 (1.00, 1.00） |
| Guinea | 50 to 54 | T1D | Both | 6.02 (5.67, 6.39） |
| Guinea | 50 to 54 | T1D | Female | 6.11 (5.62, 6.66） |
| Guinea | 50 to 54 | T1D | Male | 5.92 (5.44, 6.44） |
| Guinea | 55 to 59 | T1D | Both | 6.09 (5.71, 6.49） |
| Guinea | 55 to 59 | T1D | Female | 6.20 (5.66, 6.79） |
| Guinea | 55 to 59 | T1D | Male | 5.98 (5.46, 6.54） |
| Guinea | 60 to 64 | T1D | Both | 6.11 (5.70, 6.54） |
| Guinea | 60 to 64 | T1D | Female | 6.24 (5.66, 6.88） |
| Guinea | 60 to 64 | T1D | Male | 5.98 (5.43, 6.58） |
| Guinea | 65 to 69 | T1D | Both | 6.08 (5.64, 6.56） |
| Guinea | 65 to 69 | T1D | Female | 6.22 (5.59, 6.93） |
| Guinea | 65 to 69 | T1D | Male | 5.94 (5.35, 6.61） |
| Guinea-Bissau | 0 to 4 | T1D | Both | 0.15 (0.12, 0.19） |
| Guinea-Bissau | 0 to 4 | T1D | Female | 0.15 (0.11, 0.21） |
| Guinea-Bissau | 0 to 4 | T1D | Male | 0.15 (0.11, 0.21） |
| Guinea-Bissau | 10 to 14 | T1D | Both | 2.10 (1.89, 2.34） |
| Guinea-Bissau | 10 to 14 | T1D | Female | 2.12 (1.82, 2.47） |
| Guinea-Bissau | 10 to 14 | T1D | Male | 2.09 (1.81, 2.42） |
| Guinea-Bissau | 15 to 19 | T1D | Both | 3.06 (2.75, 3.41） |
| Guinea-Bissau | 15 to 19 | T1D | Female | 3.09 (2.65, 3.60） |
| Guinea-Bissau | 15 to 19 | T1D | Male | 3.05 (2.63, 3.53） |
| Guinea-Bissau | 20 to 24 | T1D | Both | 3.88 (3.48, 4.32） |

| | | | | |
|---|---|---|---|---|
| Guinea-Bissau | 20 to 24 | T1D | Female | 3.93 (3.36, 4.59） |
| Guinea-Bissau | 20 to 24 | T1D | Male | 3.85 (3.30, 4.48） |
| Guinea-Bissau | 25 to 29 | T1D | Both | 4.71 (4.21, 5.27） |
| Guinea-Bissau | 25 to 29 | T1D | Female | 4.77 (4.06, 5.60） |
| Guinea-Bissau | 25 to 29 | T1D | Male | 4.66 (3.98, 5.46） |
| Guinea-Bissau | 30 to 34 | T1D | Both | 5.21 (4.63, 5.87） |
| Guinea-Bissau | 30 to 34 | T1D | Female | 5.29 (4.47, 6.26） |
| Guinea-Bissau | 30 to 34 | T1D | Male | 5.16 (4.37, 6.09） |
| Guinea-Bissau | 35 to 39 | T1D | Both | 5.57 (4.92, 6.32） |
| Guinea-Bissau | 35 to 39 | T1D | Female | 5.65 (4.73, 6.75） |
| Guinea-Bissau | 35 to 39 | T1D | Male | 5.49 (4.60, 6.55） |
| Guinea-Bissau | 40 to 44 | T1D | Both | 5.85 (5.13, 6.68） |
| Guinea-Bissau | 40 to 44 | T1D | Female | 5.94 (4.93, 7.17） |
| Guinea-Bissau | 40 to 44 | T1D | Male | 5.76 (4.77, 6.94） |
| Guinea-Bissau | 45 to 49 | T1D | Both | 6.07 (5.27, 6.99） |
| Guinea-Bissau | 45 to 49 | T1D | Female | 6.15 (5.04, 7.51） |
| Guinea-Bissau | 45 to 49 | T1D | Male | 5.97 (4.88, 7.29） |
| Guinea-Bissau | 5 to 9 | T1D | Both | 1.00 (1.00, 1.00） |
| Guinea-Bissau | 5 to 9 | T1D | Female | 1.00 (1.00, 1.00） |
| Guinea-Bissau | 5 to 9 | T1D | Male | 1.00 (1.00, 1.00） |
| Guinea-Bissau | 50 to 54 | T1D | Both | 6.23 (5.36, 7.25） |
| Guinea-Bissau | 50 to 54 | T1D | Female | 6.32 (5.11, 7.83） |
| Guinea-Bissau | 50 to 54 | T1D | Male | 6.11 (4.93, 7.58） |
| Guinea-Bissau | 55 to 59 | T1D | Both | 6.33 (5.38, 7.46） |
| Guinea-Bissau | 55 to 59 | T1D | Female | 6.45 (5.12, 8.12） |
| Guinea-Bissau | 55 to 59 | T1D | Male | 6.22 (4.92, 7.85） |
| Guinea-Bissau | 60 to 64 | T1D | Both | 6.36 (5.32, 7.61） |
| Guinea-Bissau | 60 to 64 | T1D | Female | 6.50 (5.05, 8.35） |
| Guinea-Bissau | 60 to 64 | T1D | Male | 6.24 (4.83, 8.06） |
| Guinea-Bissau | 65 to 69 | T1D | Both | 6.40 (5.23, 7.83） |
| Guinea-Bissau | 65 to 69 | T1D | Female | 6.57 (4.96, 8.70） |
| Guinea-Bissau | 65 to 69 | T1D | Male | 6.24 (4.68, 8.34） |
| Guyana | 0 to 4 | T1D | Both | 0.15 (0.10, 0.22） |

| | | | | |
|---|---|---|---|---|
| Guyana | 0 to 4 | T1D | Female | 0.14 (0.08, 0.24） |
| Guyana | 0 to 4 | T1D | Male | 0.16 (0.10, 0.28） |
| Guyana | 10 to 14 | T1D | Both | 2.24 (1.88, 2.66） |
| Guyana | 10 to 14 | T1D | Female | 2.37 (1.87, 3.01） |
| Guyana | 10 to 14 | T1D | Male | 2.09 (1.63, 2.68） |
| Guyana | 15 to 19 | T1D | Both | 3.34 (2.81, 3.96） |
| Guyana | 15 to 19 | T1D | Female | 3.46 (2.72, 4.39） |
| Guyana | 15 to 19 | T1D | Male | 3.20 (2.50, 4.10） |
| Guyana | 20 to 24 | T1D | Both | 4.25 (3.56, 5.06） |
| Guyana | 20 to 24 | T1D | Female | 4.22 (3.30, 5.39） |
| Guyana | 20 to 24 | T1D | Male | 4.26 (3.31, 5.49） |
| Guyana | 25 to 29 | T1D | Both | 5.16 (4.31, 6.18） |
| Guyana | 25 to 29 | T1D | Female | 4.92 (3.82, 6.34） |
| Guyana | 25 to 29 | T1D | Male | 5.45 (4.20, 7.06） |
| Guyana | 30 to 34 | T1D | Both | 5.97 (4.95, 7.19） |
| Guyana | 30 to 34 | T1D | Female | 5.53 (4.26, 7.17） |
| Guyana | 30 to 34 | T1D | Male | 6.48 (4.96, 8.46） |
| Guyana | 35 to 39 | T1D | Both | 6.72 (5.55, 8.14） |
| Guyana | 35 to 39 | T1D | Female | 6.09 (4.65, 7.97） |
| Guyana | 35 to 39 | T1D | Male | 7.49 (5.70, 9.85） |
| Guyana | 40 to 44 | T1D | Both | 7.50 (6.16, 9.14） |
| Guyana | 40 to 44 | T1D | Female | 6.70 (5.07, 8.84） |
| Guyana | 40 to 44 | T1D | Male | 8.47 (6.40, 11.23） |
| Guyana | 45 to 49 | T1D | Both | 8.24 (6.71, 10.11） |
| Guyana | 45 to 49 | T1D | Female | 7.23 (5.41, 9.65） |
| Guyana | 45 to 49 | T1D | Male | 9.42 (7.05, 12.61） |
| Guyana | 5 to 9 | T1D | Both | 1.00 (1.00, 1.00） |
| Guyana | 5 to 9 | T1D | Female | 1.00 (1.00, 1.00） |
| Guyana | 5 to 9 | T1D | Male | 1.00 (1.00, 1.00） |
| Guyana | 50 to 54 | T1D | Both | 8.97 (7.24, 11.11） |
| Guyana | 50 to 54 | T1D | Female | 7.89 (5.83, 10.67） |
| Guyana | 50 to 54 | T1D | Male | 10.35 (7.64, 14.00） |
| Guyana | 55 to 59 | T1D | Both | 9.71 (7.75, 12.16） |

| | | | | |
|---|---|---|---|---|
| Guyana | 55 to 59 | T1D | Female | 8.42 (6.12, 11.59） |
| Guyana | 55 to 59 | T1D | Male | 11.13 (8.09, 15.31） |
| Guyana | 60 to 64 | T1D | Both | 10.36 (8.14, 13.19） |
| Guyana | 60 to 64 | T1D | Female | 9.07 (6.44, 12.76） |
| Guyana | 60 to 64 | T1D | Male | 11.99 (8.53, 16.85） |
| Guyana | 65 to 69 | T1D | Both | 11.05 (8.49, 14.38） |
| Guyana | 65 to 69 | T1D | Female | 9.63 (6.63, 14.00） |
| Guyana | 65 to 69 | T1D | Male | 12.87 (8.88, 18.65） |
| Haiti | 0 to 4 | T1D | Both | 0.14 (0.13, 0.16） |
| Haiti | 0 to 4 | T1D | Female | 0.13 (0.11, 0.15） |
| Haiti | 0 to 4 | T1D | Male | 0.15 (0.13, 0.18） |
| Haiti | 10 to 14 | T1D | Both | 2.29 (2.18, 2.40） |
| Haiti | 10 to 14 | T1D | Female | 2.44 (2.29, 2.61） |
| Haiti | 10 to 14 | T1D | Male | 2.12 (1.98, 2.28） |
| Haiti | 15 to 19 | T1D | Both | 3.40 (3.24, 3.57） |
| Haiti | 15 to 19 | T1D | Female | 3.55 (3.32, 3.80） |
| Haiti | 15 to 19 | T1D | Male | 3.23 (3.01, 3.47） |
| Haiti | 20 to 24 | T1D | Both | 4.29 (4.08, 4.51） |
| Haiti | 20 to 24 | T1D | Female | 4.27 (3.99, 4.58） |
| Haiti | 20 to 24 | T1D | Male | 4.29 (3.99, 4.62） |
| Haiti | 25 to 29 | T1D | Both | 5.18 (4.92, 5.45） |
| Haiti | 25 to 29 | T1D | Female | 4.94 (4.60, 5.30） |
| Haiti | 25 to 29 | T1D | Male | 5.46 (5.06, 5.88） |
| Haiti | 30 to 34 | T1D | Both | 5.91 (5.60, 6.24） |
| Haiti | 30 to 34 | T1D | Female | 5.48 (5.09, 5.90） |
| Haiti | 30 to 34 | T1D | Male | 6.44 (5.96, 6.97） |
| Haiti | 35 to 39 | T1D | Both | 6.60 (6.24, 6.98） |
| Haiti | 35 to 39 | T1D | Female | 5.98 (5.54, 6.47） |
| Haiti | 35 to 39 | T1D | Male | 7.37 (6.79, 7.99） |
| Haiti | 40 to 44 | T1D | Both | 7.29 (6.87, 7.73） |
| Haiti | 40 to 44 | T1D | Female | 6.49 (5.99, 7.04） |
| Haiti | 40 to 44 | T1D | Male | 8.26 (7.59, 9.00） |
| Haiti | 45 to 49 | T1D | Both | 7.95 (7.47, 8.45） |

| | | | | |
|---|---|---|---|---|
| Haiti | 45 to 49 | T1D | Female | 6.99 (6.42, 7.62） |
| Haiti | 45 to 49 | T1D | Male | 9.11 (8.33, 9.96） |
| Haiti | 5 to 9 | T1D | Both | 1.00 (1.00, 1.00） |
| Haiti | 5 to 9 | T1D | Female | 1.00 (1.00, 1.00） |
| Haiti | 5 to 9 | T1D | Male | 1.00 (1.00, 1.00） |
| Haiti | 50 to 54 | T1D | Both | 8.56 (8.02, 9.14） |
| Haiti | 50 to 54 | T1D | Female | 7.48 (6.83, 8.19） |
| Haiti | 50 to 54 | T1D | Male | 9.89 (9.01, 10.86） |
| Haiti | 55 to 59 | T1D | Both | 9.15 (8.53, 9.80） |
| Haiti | 55 to 59 | T1D | Female | 7.96 (7.23, 8.77） |
| Haiti | 55 to 59 | T1D | Male | 10.61 (9.61, 11.72） |
| Haiti | 60 to 64 | T1D | Both | 9.71 (9.01, 10.46） |
| Haiti | 60 to 64 | T1D | Female | 8.44 (7.61, 9.37） |
| Haiti | 60 to 64 | T1D | Male | 11.30 (10.16, 12.56） |
| Haiti | 65 to 69 | T1D | Both | 10.24 (9.44, 11.10） |
| Haiti | 65 to 69 | T1D | Female | 8.92 (7.96, 10.01） |
| Haiti | 65 to 69 | T1D | Male | 11.92 (10.62, 13.37） |
| High SDI | 0 to 4 | T1D | Both | 0.22 (0.21, 0.22） |
| High SDI | 0 to 4 | T1D | Female | 0.21 (0.20, 0.22） |
| High SDI | 0 to 4 | T1D | Male | 0.22 (0.22, 0.23） |
| High SDI | 10 to 14 | T1D | Both | 1.95 (1.92, 1.98） |
| High SDI | 10 to 14 | T1D | Female | 1.95 (1.92, 1.98） |
| High SDI | 10 to 14 | T1D | Male | 1.96 (1.93, 1.99） |
| High SDI | 15 to 19 | T1D | Both | 2.65 (2.61, 2.69） |
| High SDI | 15 to 19 | T1D | Female | 2.60 (2.56, 2.64） |
| High SDI | 15 to 19 | T1D | Male | 2.70 (2.66, 2.74） |
| High SDI | 20 to 24 | T1D | Both | 3.22 (3.17, 3.27） |
| High SDI | 20 to 24 | T1D | Female | 3.11 (3.06, 3.16） |
| High SDI | 20 to 24 | T1D | Male | 3.33 (3.27, 3.38） |
| High SDI | 25 to 29 | T1D | Both | 3.90 (3.84, 3.97） |
| High SDI | 25 to 29 | T1D | Female | 3.70 (3.64, 3.77） |
| High SDI | 25 to 29 | T1D | Male | 4.09 (4.02, 4.16） |
| High SDI | 30 to 34 | T1D | Both | 4.52 (4.45, 4.60） |

| | | | | |
|---|---|---|---|---|
| High SDI | 30 to 34 | T1D | Female | 4.24 (4.17, 4.31） |
| High SDI | 30 to 34 | T1D | Male | 4.79 (4.71, 4.88） |
| High SDI | 35 to 39 | T1D | Both | 5.14 (5.06, 5.23） |
| High SDI | 35 to 39 | T1D | Female | 4.77 (4.69, 4.85） |
| High SDI | 35 to 39 | T1D | Male | 5.50 (5.41, 5.60） |
| High SDI | 40 to 44 | T1D | Both | 5.79 (5.70, 5.90） |
| High SDI | 40 to 44 | T1D | Female | 5.33 (5.24, 5.42） |
| High SDI | 40 to 44 | T1D | Male | 6.25 (6.14, 6.36） |
| High SDI | 45 to 49 | T1D | Both | 6.47 (6.36, 6.59） |
| High SDI | 45 to 49 | T1D | Female | 5.91 (5.81, 6.02） |
| High SDI | 45 to 49 | T1D | Male | 7.02 (6.90, 7.15） |
| High SDI | 5 to 9 | T1D | Both | 1.00 (1.00, 1.00） |
| High SDI | 5 to 9 | T1D | Female | 1.00 (1.00, 1.00） |
| High SDI | 5 to 9 | T1D | Male | 1.00 (1.00, 1.00） |
| High SDI | 50 to 54 | T1D | Both | 7.16 (7.03, 7.29） |
| High SDI | 50 to 54 | T1D | Female | 6.50 (6.39, 6.62） |
| High SDI | 50 to 54 | T1D | Male | 7.81 (7.67, 7.95） |
| High SDI | 55 to 59 | T1D | Both | 7.85 (7.71, 7.99） |
| High SDI | 55 to 59 | T1D | Female | 7.10 (6.97, 7.23） |
| High SDI | 55 to 59 | T1D | Male | 8.61 (8.45, 8.77） |
| High SDI | 60 to 64 | T1D | Both | 8.58 (8.42, 8.74） |
| High SDI | 60 to 64 | T1D | Female | 7.74 (7.59, 7.88） |
| High SDI | 60 to 64 | T1D | Male | 9.44 (9.26, 9.62） |
| High SDI | 65 to 69 | T1D | Both | 9.35 (9.17, 9.53） |
| High SDI | 65 to 69 | T1D | Female | 8.42 (8.26, 8.58） |
| High SDI | 65 to 69 | T1D | Male | 10.32 (10.12, 10.52） |
| High-middle SDI | 0 to 4 | T1D | Both | 0.17 (0.16, 0.18） |
| High-middle SDI | 0 to 4 | T1D | Female | 0.16 (0.15, 0.18） |
| High-middle SDI | 0 to 4 | T1D | Male | 0.17 (0.16, 0.18） |
| High-middle SDI | 10 to 14 | T1D | Both | 2.12 (2.05, 2.18） |
| High-middle SDI | 10 to 14 | T1D | Female | 2.11 (2.04, 2.17） |
| High-middle SDI | 10 to 14 | T1D | Male | 2.13 (2.06, 2.19） |
| High-middle SDI | 15 to 19 | T1D | Both | 3.04 (2.95, 3.13） |

| | | | | |
|---|---|---|---|---|
| High-middle SDI | 15 to 19 | T1D | Female | 2.97 (2.88, 3.06） |
| High-middle SDI | 15 to 19 | T1D | Male | 3.12 (3.02, 3.21） |
| High-middle SDI | 20 to 24 | T1D | Both | 3.85 (3.74, 3.97） |
| High-middle SDI | 20 to 24 | T1D | Female | 3.70 (3.59, 3.81） |
| High-middle SDI | 20 to 24 | T1D | Male | 4.00 (3.88, 4.13） |
| High-middle SDI | 25 to 29 | T1D | Both | 4.79 (4.65, 4.93） |
| High-middle SDI | 25 to 29 | T1D | Female | 4.56 (4.42, 4.70） |
| High-middle SDI | 25 to 29 | T1D | Male | 5.02 (4.87, 5.18） |
| High-middle SDI | 30 to 34 | T1D | Both | 5.67 (5.50, 5.85） |
| High-middle SDI | 30 to 34 | T1D | Female | 5.37 (5.20, 5.53） |
| High-middle SDI | 30 to 34 | T1D | Male | 5.97 (5.79, 6.16） |
| High-middle SDI | 35 to 39 | T1D | Both | 6.58 (6.38, 6.79） |
| High-middle SDI | 35 to 39 | T1D | Female | 6.20 (6.01, 6.40） |
| High-middle SDI | 35 to 39 | T1D | Male | 6.97 (6.75, 7.20） |
| High-middle SDI | 40 to 44 | T1D | Both | 7.55 (7.31, 7.79） |
| High-middle SDI | 40 to 44 | T1D | Female | 7.08 (6.86, 7.31） |
| High-middle SDI | 40 to 44 | T1D | Male | 8.03 (7.77, 8.29） |
| High-middle SDI | 45 to 49 | T1D | Both | 8.57 (8.29, 8.85） |
| High-middle SDI | 45 to 49 | T1D | Female | 8.03 (7.77, 8.30） |
| High-middle SDI | 45 to 49 | T1D | Male | 9.11 (8.82, 9.42） |
| High-middle SDI | 5 to 9 | T1D | Both | 1.00 (1.00, 1.00） |
| High-middle SDI | 5 to 9 | T1D | Female | 1.00 (1.00, 1.00） |
| High-middle SDI | 5 to 9 | T1D | Male | 1.00 (1.00, 1.00） |
| High-middle SDI | 50 to 54 | T1D | Both | 9.65 (9.33, 9.97） |
| High-middle SDI | 50 to 54 | T1D | Female | 9.08 (8.78, 9.38） |
| High-middle SDI | 50 to 54 | T1D | Male | 10.24 (9.90, 10.59） |
| High-middle SDI | 55 to 59 | T1D | Both | 10.85 (10.49, 11.23） |
| High-middle SDI | 55 to 59 | T1D | Female | 10.27 (9.92, 10.62） |
| High-middle SDI | 55 to 59 | T1D | Male | 11.46 (11.07, 11.87） |
| High-middle SDI | 60 to 64 | T1D | Both | 12.15 (11.74, 12.58） |
| High-middle SDI | 60 to 64 | T1D | Female | 11.57 (11.17, 11.98） |
| High-middle SDI | 60 to 64 | T1D | Male | 12.76 (12.32, 13.23） |
| High-middle SDI | 65 to 69 | T1D | Both | 13.63 (13.15, 14.13） |

| | | | | |
|---|---|---|---|---|
| High-middle SDI | 65 to 69 | T1D | Female | 13.05 (12.59, 13.53） |
| High-middle SDI | 65 to 69 | T1D | Male | 14.25 (13.73, 14.79） |
| Honduras | 0 to 4 | T1D | Both | 0.13 (0.11, 0.14） |
| Honduras | 0 to 4 | T1D | Female | 0.12 (0.10, 0.14） |
| Honduras | 0 to 4 | T1D | Male | 0.14 (0.12, 0.17） |
| Honduras | 10 to 14 | T1D | Both | 2.26 (2.14, 2.39） |
| Honduras | 10 to 14 | T1D | Female | 2.44 (2.27, 2.63） |
| Honduras | 10 to 14 | T1D | Male | 2.06 (1.90, 2.23） |
| Honduras | 15 to 19 | T1D | Both | 3.06 (2.90, 3.24） |
| Honduras | 15 to 19 | T1D | Female | 3.25 (3.01, 3.51） |
| Honduras | 15 to 19 | T1D | Male | 2.83 (2.60, 3.07） |
| Honduras | 20 to 24 | T1D | Both | 3.58 (3.37, 3.79） |
| Honduras | 20 to 24 | T1D | Female | 3.68 (3.40, 3.98） |
| Honduras | 20 to 24 | T1D | Male | 3.41 (3.13, 3.72） |
| Honduras | 25 to 29 | T1D | Both | 4.06 (3.82, 4.31） |
| Honduras | 25 to 29 | T1D | Female | 4.05 (3.73, 4.40） |
| Honduras | 25 to 29 | T1D | Male | 4.01 (3.66, 4.39） |
| Honduras | 30 to 34 | T1D | Both | 4.43 (4.15, 4.73） |
| Honduras | 30 to 34 | T1D | Female | 4.34 (3.97, 4.73） |
| Honduras | 30 to 34 | T1D | Male | 4.48 (4.08, 4.93） |
| Honduras | 35 to 39 | T1D | Both | 4.75 (4.44, 5.09） |
| Honduras | 35 to 39 | T1D | Female | 4.59 (4.18, 5.03） |
| Honduras | 35 to 39 | T1D | Male | 4.90 (4.43, 5.42） |
| Honduras | 40 to 44 | T1D | Both | 5.03 (4.68, 5.41） |
| Honduras | 40 to 44 | T1D | Female | 4.81 (4.36, 5.31） |
| Honduras | 40 to 44 | T1D | Male | 5.25 (4.72, 5.84） |
| Honduras | 45 to 49 | T1D | Both | 5.30 (4.90, 5.72） |
| Honduras | 45 to 49 | T1D | Female | 5.02 (4.52, 5.57） |
| Honduras | 45 to 49 | T1D | Male | 5.56 (4.97, 6.23） |
| Honduras | 5 to 9 | T1D | Both | 1.00 (1.00, 1.00） |
| Honduras | 5 to 9 | T1D | Female | 1.00 (1.00, 1.00） |
| Honduras | 5 to 9 | T1D | Male | 1.00 (1.00, 1.00） |
| Honduras | 50 to 54 | T1D | Both | 5.53 (5.09, 6.00） |

| | | | | |
|---|---|---|---|---|
| Honduras | 50 to 54 | T1D | Female | 5.22 (4.66, 5.84） |
| Honduras | 50 to 54 | T1D | Male | 5.83 (5.17, 6.58） |
| Honduras | 55 to 59 | T1D | Both | 5.73 (5.25, 6.26） |
| Honduras | 55 to 59 | T1D | Female | 5.41 (4.79, 6.10） |
| Honduras | 55 to 59 | T1D | Male | 6.07 (5.33, 6.90） |
| Honduras | 60 to 64 | T1D | Both | 5.91 (5.37, 6.50） |
| Honduras | 60 to 64 | T1D | Female | 5.56 (4.88, 6.34） |
| Honduras | 60 to 64 | T1D | Male | 6.24 (5.42, 7.17） |
| Honduras | 65 to 69 | T1D | Both | 6.07 (5.47, 6.75） |
| Honduras | 65 to 69 | T1D | Female | 5.71 (4.95, 6.60） |
| Honduras | 65 to 69 | T1D | Male | 6.40 (5.49, 7.46） |
| Hungary | 0 to 4 | T1D | Both | 0.20 (0.18, 0.23） |
| Hungary | 0 to 4 | T1D | Female | 0.20 (0.17, 0.23） |
| Hungary | 0 to 4 | T1D | Male | 0.21 (0.18, 0.24） |
| Hungary | 10 to 14 | T1D | Both | 2.19 (2.08, 2.31） |
| Hungary | 10 to 14 | T1D | Female | 2.17 (2.02, 2.33） |
| Hungary | 10 to 14 | T1D | Male | 2.22 (2.07, 2.38） |
| Hungary | 15 to 19 | T1D | Both | 3.38 (3.21, 3.55） |
| Hungary | 15 to 19 | T1D | Female | 3.25 (3.03, 3.49） |
| Hungary | 15 to 19 | T1D | Male | 3.50 (3.27, 3.76） |
| Hungary | 20 to 24 | T1D | Both | 4.44 (4.23, 4.67） |
| Hungary | 20 to 24 | T1D | Female | 4.19 (3.90, 4.50） |
| Hungary | 20 to 24 | T1D | Male | 4.71 (4.38, 5.05） |
| Hungary | 25 to 29 | T1D | Both | 5.68 (5.40, 5.98） |
| Hungary | 25 to 29 | T1D | Female | 5.23 (4.87, 5.63） |
| Hungary | 25 to 29 | T1D | Male | 6.14 (5.71, 6.59） |
| Hungary | 30 to 34 | T1D | Both | 6.94 (6.59, 7.30） |
| Hungary | 30 to 34 | T1D | Female | 6.27 (5.83, 6.75） |
| Hungary | 30 to 34 | T1D | Male | 7.62 (7.09, 8.19） |
| Hungary | 35 to 39 | T1D | Both | 8.30 (7.88, 8.74） |
| Hungary | 35 to 39 | T1D | Female | 7.39 (6.86, 7.96） |
| Hungary | 35 to 39 | T1D | Male | 9.24 (8.58, 9.94） |
| Hungary | 40 to 44 | T1D | Both | 9.83 (9.32, 10.37） |

| | | | | |
|---|---|---|---|---|
| Hungary | 40 to 44 | T1D | Female | 8.66 (8.03, 9.34） |
| Hungary | 40 to 44 | T1D | Male | 11.06 (10.27, 11.92） |
| Hungary | 45 to 49 | T1D | Both | 11.54 (10.93, 12.18） |
| Hungary | 45 to 49 | T1D | Female | 10.10 (9.35, 10.90） |
| Hungary | 45 to 49 | T1D | Male | 13.08 (12.13, 14.10） |
| Hungary | 5 to 9 | T1D | Both | 1.00 (1.00, 1.00） |
| Hungary | 5 to 9 | T1D | Female | 1.00 (1.00, 1.00） |
| Hungary | 5 to 9 | T1D | Male | 1.00 (1.00, 1.00） |
| Hungary | 50 to 54 | T1D | Both | 13.42 (12.71, 14.17） |
| Hungary | 50 to 54 | T1D | Female | 11.72 (10.84, 12.67） |
| Hungary | 50 to 54 | T1D | Male | 15.28 (14.15, 16.50） |
| Hungary | 55 to 59 | T1D | Both | 15.51 (14.68, 16.39） |
| Hungary | 55 to 59 | T1D | Female | 13.55 (12.53, 14.66） |
| Hungary | 55 to 59 | T1D | Male | 17.72 (16.40, 19.15） |
| Hungary | 60 to 64 | T1D | Both | 17.83 (16.86, 18.85） |
| Hungary | 60 to 64 | T1D | Female | 15.63 (14.44, 16.93） |
| Hungary | 60 to 64 | T1D | Male | 20.42 (18.87, 22.09） |
| Hungary | 65 to 69 | T1D | Both | 20.44 (19.31, 21.65） |
| Hungary | 65 to 69 | T1D | Female | 18.04 (16.63, 19.56） |
| Hungary | 65 to 69 | T1D | Male | 23.44 (21.63, 25.41） |
| Iceland | 0 to 4 | T1D | Both | 0.17 (0.11, 0.25） |
| Iceland | 0 to 4 | T1D | Female | 0.15 (0.08, 0.28） |
| Iceland | 0 to 4 | T1D | Male | 0.16 (0.09, 0.29） |
| Iceland | 10 to 14 | T1D | Both | 2.19 (1.80, 2.67） |
| Iceland | 10 to 14 | T1D | Female | 2.17 (1.64, 2.87） |
| Iceland | 10 to 14 | T1D | Male | 2.17 (1.64, 2.86） |
| Iceland | 15 to 19 | T1D | Both | 3.20 (2.62, 3.89） |
| Iceland | 15 to 19 | T1D | Female | 3.10 (2.34, 4.11） |
| Iceland | 15 to 19 | T1D | Male | 3.27 (2.48, 4.32） |
| Iceland | 20 to 24 | T1D | Both | 4.04 (3.31, 4.94） |
| Iceland | 20 to 24 | T1D | Female | 3.80 (2.86, 5.07） |
| Iceland | 20 to 24 | T1D | Male | 4.22 (3.19, 5.58） |
| Iceland | 25 to 29 | T1D | Both | 4.91 (4.00, 6.02） |

| | | | | |
|---|---|---|---|---|
| Iceland | 25 to 29 | T1D | Female | 4.51 (3.36, 6.04） |
| Iceland | 25 to 29 | T1D | Male | 5.24 (3.94, 6.97） |
| Iceland | 30 to 34 | T1D | Both | 5.68 (4.60, 7.00） |
| Iceland | 30 to 34 | T1D | Female | 5.12 (3.79, 6.93） |
| Iceland | 30 to 34 | T1D | Male | 6.16 (4.60, 8.26） |
| Iceland | 35 to 39 | T1D | Both | 6.44 (5.19, 7.99） |
| Iceland | 35 to 39 | T1D | Female | 5.69 (4.17, 7.76） |
| Iceland | 35 to 39 | T1D | Male | 7.02 (5.20, 9.48） |
| Iceland | 40 to 44 | T1D | Both | 7.18 (5.76, 8.95） |
| Iceland | 40 to 44 | T1D | Female | 6.34 (4.62, 8.71） |
| Iceland | 40 to 44 | T1D | Male | 7.91 (5.83, 10.74） |
| Iceland | 45 to 49 | T1D | Both | 7.93 (6.33, 9.93） |
| Iceland | 45 to 49 | T1D | Female | 6.95 (5.02, 9.61） |
| Iceland | 45 to 49 | T1D | Male | 8.79 (6.43, 12.01） |
| Iceland | 5 to 9 | T1D | Both | 1.00 (1.00, 1.00） |
| Iceland | 5 to 9 | T1D | Female | 1.00 (1.00, 1.00） |
| Iceland | 5 to 9 | T1D | Male | 1.00 (1.00, 1.00） |
| Iceland | 50 to 54 | T1D | Both | 8.66 (6.88, 10.90） |
| Iceland | 50 to 54 | T1D | Female | 7.55 (5.42, 10.54） |
| Iceland | 50 to 54 | T1D | Male | 9.67 (7.03, 13.30） |
| Iceland | 55 to 59 | T1D | Both | 9.42 (7.43, 11.94） |
| Iceland | 55 to 59 | T1D | Female | 8.22 (5.83, 11.58） |
| Iceland | 55 to 59 | T1D | Male | 10.57 (7.62, 14.66） |
| Iceland | 60 to 64 | T1D | Both | 10.21 (7.99, 13.03） |
| Iceland | 60 to 64 | T1D | Female | 8.79 (6.16, 12.54） |
| Iceland | 60 to 64 | T1D | Male | 11.36 (8.11, 15.91） |
| Iceland | 65 to 69 | T1D | Both | 10.99 (8.52, 14.17） |
| Iceland | 65 to 69 | T1D | Female | 9.50 (6.57, 13.75） |
| Iceland | 65 to 69 | T1D | Male | 12.30 (8.67, 17.45） |
| India | 0 to 4 | T1D | Both | 0.14 (0.13, 0.15） |
| India | 0 to 4 | T1D | Female | 0.14 (0.13, 0.15） |
| India | 0 to 4 | T1D | Male | 0.14 (0.13, 0.16） |
| India | 10 to 14 | T1D | Both | 2.15 (2.08, 2.23） |

| | | | | |
|---|---|---|---|---|
| India | 10 to 14 | T1D | Female | 2.09 (2.05, 2.14） |
| India | 10 to 14 | T1D | Male | 2.21 (2.11, 2.32） |
| India | 15 to 19 | T1D | Both | 2.96 (2.86, 3.06） |
| India | 15 to 19 | T1D | Female | 2.79 (2.73, 2.86） |
| India | 15 to 19 | T1D | Male | 3.12 (2.98, 3.27） |
| India | 20 to 24 | T1D | Both | 3.54 (3.42, 3.67） |
| India | 20 to 24 | T1D | Female | 3.27 (3.20, 3.35） |
| India | 20 to 24 | T1D | Male | 3.81 (3.63, 3.99） |
| India | 25 to 29 | T1D | Both | 4.18 (4.03, 4.34） |
| India | 25 to 29 | T1D | Female | 3.80 (3.71, 3.90） |
| India | 25 to 29 | T1D | Male | 4.56 (4.35, 4.79） |
| India | 30 to 34 | T1D | Both | 4.71 (4.53, 4.89） |
| India | 30 to 34 | T1D | Female | 4.24 (4.13, 4.35） |
| India | 30 to 34 | T1D | Male | 5.18 (4.93, 5.45） |
| India | 35 to 39 | T1D | Both | 5.20 (5.00, 5.41） |
| India | 35 to 39 | T1D | Female | 4.65 (4.53, 4.77） |
| India | 35 to 39 | T1D | Male | 5.76 (5.46, 6.06） |
| India | 40 to 44 | T1D | Both | 5.70 (5.47, 5.94） |
| India | 40 to 44 | T1D | Female | 5.08 (4.94, 5.22） |
| India | 40 to 44 | T1D | Male | 6.33 (6.00, 6.68） |
| India | 45 to 49 | T1D | Both | 6.19 (5.93, 6.46） |
| India | 45 to 49 | T1D | Female | 5.50 (5.35, 5.66） |
| India | 45 to 49 | T1D | Male | 6.88 (6.51, 7.28） |
| India | 5 to 9 | T1D | Both | 1.00 (1.00, 1.00） |
| India | 5 to 9 | T1D | Female | 1.00 (1.00, 1.00） |
| India | 5 to 9 | T1D | Male | 1.00 (1.00, 1.00） |
| India | 50 to 54 | T1D | Both | 6.67 (6.37, 6.97） |
| India | 50 to 54 | T1D | Female | 5.93 (5.75, 6.11） |
| India | 50 to 54 | T1D | Male | 7.42 (7.00, 7.87） |
| India | 55 to 59 | T1D | Both | 7.14 (6.81, 7.49） |
| India | 55 to 59 | T1D | Female | 6.37 (6.17, 6.58） |
| India | 55 to 59 | T1D | Male | 7.95 (7.48, 8.46） |
| India | 60 to 64 | T1D | Both | 7.60 (7.23, 7.99） |

| | | | | |
|---|---|---|---|---|
| India | 60 to 64 | T1D | Female | 6.83 (6.60, 7.06） |
| India | 60 to 64 | T1D | Male | 8.45 (7.92, 9.02） |
| India | 65 to 69 | T1D | Both | 8.05 (7.63, 8.49） |
| India | 65 to 69 | T1D | Female | 7.27 (7.01, 7.54） |
| India | 65 to 69 | T1D | Male | 8.91 (8.31, 9.55） |
| Indonesia | 0 to 4 | T1D | Both | 0.13 (0.13, 0.14） |
| Indonesia | 0 to 4 | T1D | Female | 0.13 (0.13, 0.14） |
| Indonesia | 0 to 4 | T1D | Male | 0.13 (0.12, 0.14） |
| Indonesia | 10 to 14 | T1D | Both | 2.25 (2.22, 2.28） |
| Indonesia | 10 to 14 | T1D | Female | 2.21 (2.17, 2.26） |
| Indonesia | 10 to 14 | T1D | Male | 2.29 (2.25, 2.34） |
| Indonesia | 15 to 19 | T1D | Both | 3.32 (3.27, 3.37） |
| Indonesia | 15 to 19 | T1D | Female | 3.19 (3.12, 3.26） |
| Indonesia | 15 to 19 | T1D | Male | 3.45 (3.38, 3.52） |
| Indonesia | 20 to 24 | T1D | Both | 4.26 (4.20, 4.33） |
| Indonesia | 20 to 24 | T1D | Female | 4.01 (3.93, 4.09） |
| Indonesia | 20 to 24 | T1D | Male | 4.53 (4.43, 4.62） |
| Indonesia | 25 to 29 | T1D | Both | 5.26 (5.18, 5.34） |
| Indonesia | 25 to 29 | T1D | Female | 4.85 (4.74, 4.95） |
| Indonesia | 25 to 29 | T1D | Male | 5.70 (5.58, 5.82） |
| Indonesia | 30 to 34 | T1D | Both | 6.04 (5.95, 6.13） |
| Indonesia | 30 to 34 | T1D | Female | 5.49 (5.37, 5.61） |
| Indonesia | 30 to 34 | T1D | Male | 6.62 (6.48, 6.77） |
| Indonesia | 35 to 39 | T1D | Both | 6.73 (6.62, 6.84） |
| Indonesia | 35 to 39 | T1D | Female | 6.06 (5.93, 6.20） |
| Indonesia | 35 to 39 | T1D | Male | 7.44 (7.27, 7.61） |
| Indonesia | 40 to 44 | T1D | Both | 7.41 (7.29, 7.53） |
| Indonesia | 40 to 44 | T1D | Female | 6.63 (6.48, 6.78） |
| Indonesia | 40 to 44 | T1D | Male | 8.23 (8.04, 8.42） |
| Indonesia | 45 to 49 | T1D | Both | 8.07 (7.93, 8.20） |
| Indonesia | 45 to 49 | T1D | Female | 7.20 (7.03, 7.37） |
| Indonesia | 45 to 49 | T1D | Male | 8.98 (8.77, 9.20） |
| Indonesia | 5 to 9 | T1D | Both | 1.00 (1.00, 1.00） |

| | | | | |
|---|---|---|---|---|
| Indonesia | 5 to 9 | T1D | Female | 1.00 (1.00, 1.00） |
| Indonesia | 5 to 9 | T1D | Male | 1.00 (1.00, 1.00） |
| Indonesia | 50 to 54 | T1D | Both | 8.71 (8.56, 8.86） |
| Indonesia | 50 to 54 | T1D | Female | 7.76 (7.57, 7.96） |
| Indonesia | 50 to 54 | T1D | Male | 9.71 (9.47, 9.95） |
| Indonesia | 55 to 59 | T1D | Both | 9.35 (9.18, 9.52） |
| Indonesia | 55 to 59 | T1D | Female | 8.35 (8.13, 8.57） |
| Indonesia | 55 to 59 | T1D | Male | 10.41 (10.15, 10.68） |
| Indonesia | 60 to 64 | T1D | Both | 9.99 (9.80, 10.19） |
| Indonesia | 60 to 64 | T1D | Female | 8.95 (8.70, 9.20） |
| Indonesia | 60 to 64 | T1D | Male | 11.10 (10.80, 11.41） |
| Indonesia | 65 to 69 | T1D | Both | 10.63 (10.41, 10.85） |
| Indonesia | 65 to 69 | T1D | Female | 9.57 (9.29, 9.86） |
| Indonesia | 65 to 69 | T1D | Male | 11.77 (11.42, 12.12） |
| Iran (Islamic Republic of) | 0 to 4 | T1D | Both | 0.13 (0.12, 0.13） |
| Iran (Islamic Republic of) | 0 to 4 | T1D | Female | 0.13 (0.12, 0.14） |
| Iran (Islamic Republic of) | 0 to 4 | T1D | Male | 0.12 (0.12, 0.13） |
| Iran (Islamic Republic of) | 10 to 14 | T1D | Both | 2.39 (2.36, 2.43） |
| Iran (Islamic Republic of) | 10 to 14 | T1D | Female | 2.42 (2.37, 2.48） |
| Iran (Islamic Republic of) | 10 to 14 | T1D | Male | 2.36 (2.32, 2.42） |
| Iran (Islamic Republic of) | 15 to 19 | T1D | Both | 3.79 (3.73, 3.85） |
| Iran (Islamic Republic of) | 15 to 19 | T1D | Female | 3.87 (3.78, 3.96） |
| Iran (Islamic Republic of) | 15 to 19 | T1D | Male | 3.72 (3.64, 3.79） |
| Iran (Islamic Republic of) | 20 to 24 | T1D | Both | 5.18 (5.10, 5.26） |
| Iran (Islamic Republic of) | 20 to 24 | T1D | Female | 5.27 (5.15, 5.39） |
| Iran (Islamic Republic of) | 20 to 24 | T1D | Male | 5.11 (5.00, 5.21） |
| Iran (Islamic Republic of) | 25 to 29 | T1D | Both | 6.85 (6.74, 6.95） |
| Iran (Islamic Republic of) | 25 to 29 | T1D | Female | 6.90 (6.74, 7.05） |
| Iran (Islamic Republic of) | 25 to 29 | T1D | Male | 6.80 (6.66, 6.95） |
| Iran (Islamic Republic of) | 30 to 34 | T1D | Both | 8.49 (8.35, 8.62） |
| Iran (Islamic Republic of) | 30 to 34 | T1D | Female | 8.50 (8.30, 8.70） |
| Iran (Islamic Republic of) | 30 to 34 | T1D | Male | 8.48 (8.30, 8.67） |
| Iran (Islamic Republic of) | 35 to 39 | T1D | Both | 10.21 (10.05, 10.38） |

| | | | | |
|---|---|---|---|---|
| Iran (Islamic Republic of) | 35 to 39 | T1D | Female | 10.19 (9.95, 10.44） |
| Iran (Islamic Republic of) | 35 to 39 | T1D | Male | 10.23 (10.01, 10.47） |
| Iran (Islamic Republic of) | 40 to 44 | T1D | Both | 12.07 (11.87, 12.28） |
| Iran (Islamic Republic of) | 40 to 44 | T1D | Female | 12.03 (11.73, 12.33） |
| Iran (Islamic Republic of) | 40 to 44 | T1D | Male | 12.12 (11.83, 12.41） |
| Iran (Islamic Republic of) | 45 to 49 | T1D | Both | 14.09 (13.85, 14.35） |
| Iran (Islamic Republic of) | 45 to 49 | T1D | Female | 14.05 (13.69, 14.41） |
| Iran (Islamic Republic of) | 45 to 49 | T1D | Male | 14.14 (13.80, 14.49） |
| Iran (Islamic Republic of) | 5 to 9 | T1D | Both | 1.00 (1.00, 1.00） |
| Iran (Islamic Republic of) | 5 to 9 | T1D | Female | 1.00 (1.00, 1.00） |
| Iran (Islamic Republic of) | 5 to 9 | T1D | Male | 1.00 (1.00, 1.00） |
| Iran (Islamic Republic of) | 50 to 54 | T1D | Both | 16.27 (15.97, 16.58） |
| Iran (Islamic Republic of) | 50 to 54 | T1D | Female | 16.26 (15.83, 16.71） |
| Iran (Islamic Republic of) | 50 to 54 | T1D | Male | 16.29 (15.88, 16.71） |
| Iran (Islamic Republic of) | 55 to 59 | T1D | Both | 18.64 (18.28, 19.01） |
| Iran (Islamic Republic of) | 55 to 59 | T1D | Female | 18.72 (18.20, 19.26） |
| Iran (Islamic Republic of) | 55 to 59 | T1D | Male | 18.59 (18.09, 19.10） |
| Iran (Islamic Republic of) | 60 to 64 | T1D | Both | 21.22 (20.79, 21.67） |
| Iran (Islamic Republic of) | 60 to 64 | T1D | Female | 21.45 (20.82, 22.10） |
| Iran (Islamic Republic of) | 60 to 64 | T1D | Male | 21.05 (20.46, 21.66） |
| Iran (Islamic Republic of) | 65 to 69 | T1D | Both | 24.02 (23.49, 24.56） |
| Iran (Islamic Republic of) | 65 to 69 | T1D | Female | 24.47 (23.70, 25.26） |
| Iran (Islamic Republic of) | 65 to 69 | T1D | Male | 23.66 (22.94, 24.39） |
| Iraq | 0 to 4 | T1D | Both | 0.15 (0.14, 0.15） |
| Iraq | 0 to 4 | T1D | Female | 0.15 (0.14, 0.16） |
| Iraq | 0 to 4 | T1D | Male | 0.14 (0.13, 0.15） |
| Iraq | 10 to 14 | T1D | Both | 2.34 (2.30, 2.39） |
| Iraq | 10 to 14 | T1D | Female | 2.45 (2.38, 2.53） |
| Iraq | 10 to 14 | T1D | Male | 2.26 (2.19, 2.32） |
| Iraq | 15 to 19 | T1D | Both | 3.66 (3.59, 3.74） |
| Iraq | 15 to 19 | T1D | Female | 3.88 (3.76, 4.00） |
| Iraq | 15 to 19 | T1D | Male | 3.49 (3.39, 3.59） |
| Iraq | 20 to 24 | T1D | Both | 4.87 (4.77, 4.97） |

| | | | | |
|---|---|---|---|---|
| Iraq | 20 to 24 | T1D | Female | 5.11 (4.95, 5.27） |
| Iraq | 20 to 24 | T1D | Male | 4.67 (4.54, 4.81） |
| Iraq | 25 to 29 | T1D | Both | 6.23 (6.09, 6.37） |
| Iraq | 25 to 29 | T1D | Female | 6.45 (6.24, 6.67） |
| Iraq | 25 to 29 | T1D | Male | 6.04 (5.86, 6.22） |
| Iraq | 30 to 34 | T1D | Both | 7.57 (7.40, 7.75） |
| Iraq | 30 to 34 | T1D | Female | 7.77 (7.50, 8.04） |
| Iraq | 30 to 34 | T1D | Male | 7.41 (7.18, 7.65） |
| Iraq | 35 to 39 | T1D | Both | 8.96 (8.74, 9.18） |
| Iraq | 35 to 39 | T1D | Female | 9.13 (8.80, 9.46） |
| Iraq | 35 to 39 | T1D | Male | 8.82 (8.53, 9.12） |
| Iraq | 40 to 44 | T1D | Both | 10.44 (10.18, 10.71） |
| Iraq | 40 to 44 | T1D | Female | 10.58 (10.19, 10.99） |
| Iraq | 40 to 44 | T1D | Male | 10.32 (9.97, 10.69） |
| Iraq | 45 to 49 | T1D | Both | 12.02 (11.70, 12.35） |
| Iraq | 45 to 49 | T1D | Female | 12.16 (11.68, 12.66） |
| Iraq | 45 to 49 | T1D | Male | 11.91 (11.48, 12.36） |
| Iraq | 5 to 9 | T1D | Both | 1.00 (1.00, 1.00） |
| Iraq | 5 to 9 | T1D | Female | 1.00 (1.00, 1.00） |
| Iraq | 5 to 9 | T1D | Male | 1.00 (1.00, 1.00） |
| Iraq | 50 to 54 | T1D | Both | 13.71 (13.32, 14.12） |
| Iraq | 50 to 54 | T1D | Female | 13.88 (13.30, 14.49） |
| Iraq | 50 to 54 | T1D | Male | 13.59 (13.06, 14.13） |
| Iraq | 55 to 59 | T1D | Both | 15.53 (15.05, 16.02） |
| Iraq | 55 to 59 | T1D | Female | 15.76 (15.05, 16.50） |
| Iraq | 55 to 59 | T1D | Male | 15.37 (14.73, 16.03） |
| Iraq | 60 to 64 | T1D | Both | 17.49 (16.91, 18.08） |
| Iraq | 60 to 64 | T1D | Female | 17.82 (16.96, 18.72） |
| Iraq | 60 to 64 | T1D | Male | 17.27 (16.50, 18.08） |
| Iraq | 65 to 69 | T1D | Both | 19.60 (18.90, 20.34） |
| Iraq | 65 to 69 | T1D | Female | 20.09 (19.04, 21.20） |
| Iraq | 65 to 69 | T1D | Male | 19.31 (18.36, 20.31） |
| Ireland | 0 to 4 | T1D | Both | 0.21 (0.19, 0.23） |

| | | | | |
|---|---|---|---|---|
| Ireland | 0 to 4 | T1D | Female | 0.20 (0.17, 0.23） |
| Ireland | 0 to 4 | T1D | Male | 0.21 (0.19, 0.24） |
| Ireland | 10 to 14 | T1D | Both | 2.22 (2.11, 2.33） |
| Ireland | 10 to 14 | T1D | Female | 2.19 (2.04, 2.35） |
| Ireland | 10 to 14 | T1D | Male | 2.24 (2.09, 2.40） |
| Ireland | 15 to 19 | T1D | Both | 3.58 (3.41, 3.76） |
| Ireland | 15 to 19 | T1D | Female | 3.39 (3.15, 3.64） |
| Ireland | 15 to 19 | T1D | Male | 3.76 (3.51, 4.03） |
| Ireland | 20 to 24 | T1D | Both | 4.91 (4.67, 5.16） |
| Ireland | 20 to 24 | T1D | Female | 4.47 (4.15, 4.81） |
| Ireland | 20 to 24 | T1D | Male | 5.34 (4.97, 5.73） |
| Ireland | 25 to 29 | T1D | Both | 6.43 (6.11, 6.77） |
| Ireland | 25 to 29 | T1D | Female | 5.70 (5.29, 6.13） |
| Ireland | 25 to 29 | T1D | Male | 7.17 (6.68, 7.70） |
| Ireland | 30 to 34 | T1D | Both | 8.12 (7.70, 8.55） |
| Ireland | 30 to 34 | T1D | Female | 6.99 (6.49, 7.54） |
| Ireland | 30 to 34 | T1D | Male | 9.27 (8.62, 9.97） |
| Ireland | 35 to 39 | T1D | Both | 10.01 (9.49, 10.56） |
| Ireland | 35 to 39 | T1D | Female | 8.42 (7.79, 9.10） |
| Ireland | 35 to 39 | T1D | Male | 11.66 (10.83, 12.56） |
| Ireland | 40 to 44 | T1D | Both | 12.17 (11.52, 12.85） |
| Ireland | 40 to 44 | T1D | Female | 10.01 (9.25, 10.84） |
| Ireland | 40 to 44 | T1D | Male | 14.40 (13.35, 15.53） |
| Ireland | 45 to 49 | T1D | Both | 14.59 (13.79, 15.43） |
| Ireland | 45 to 49 | T1D | Female | 11.80 (10.87, 12.80） |
| Ireland | 45 to 49 | T1D | Male | 17.51 (16.21, 18.92） |
| Ireland | 5 to 9 | T1D | Both | 1.00 (1.00, 1.00） |
| Ireland | 5 to 9 | T1D | Female | 1.00 (1.00, 1.00） |
| Ireland | 5 to 9 | T1D | Male | 1.00 (1.00, 1.00） |
| Ireland | 50 to 54 | T1D | Both | 17.33 (16.37, 18.36） |
| Ireland | 50 to 54 | T1D | Female | 13.80 (12.70, 15.00） |
| Ireland | 50 to 54 | T1D | Male | 21.05 (19.45, 22.79） |
| Ireland | 55 to 59 | T1D | Both | 20.42 (19.26, 21.66） |

| | | | | |
|---|---|---|---|---|
| Ireland | 55 to 59 | T1D | Female | 16.06 (14.74, 17.50） |
| Ireland | 55 to 59 | T1D | Male | 25.07 (23.12, 27.18） |
| Ireland | 60 to 64 | T1D | Both | 23.90 (22.50, 25.38） |
| Ireland | 60 to 64 | T1D | Female | 18.60 (17.03, 20.31） |
| Ireland | 60 to 64 | T1D | Male | 29.59 (27.23, 32.15） |
| Ireland | 65 to 69 | T1D | Both | 27.80 (26.13, 29.58） |
| Ireland | 65 to 69 | T1D | Female | 21.48 (19.62, 23.52） |
| Ireland | 65 to 69 | T1D | Male | 34.73 (31.89, 37.83） |
| Israel | 0 to 4 | T1D | Both | 0.23 (0.19, 0.28） |
| Israel | 0 to 4 | T1D | Female | 0.22 (0.18, 0.27） |
| Israel | 0 to 4 | T1D | Male | 0.24 (0.20, 0.29） |
| Israel | 10 to 14 | T1D | Both | 2.18 (1.97, 2.42） |
| Israel | 10 to 14 | T1D | Female | 2.15 (1.93, 2.40） |
| Israel | 10 to 14 | T1D | Male | 2.21 (2.01, 2.44） |
| Israel | 15 to 19 | T1D | Both | 2.91 (2.63, 3.22） |
| Israel | 15 to 19 | T1D | Female | 2.82 (2.53, 3.14） |
| Israel | 15 to 19 | T1D | Male | 3.00 (2.73, 3.31） |
| Israel | 20 to 24 | T1D | Both | 3.41 (3.08, 3.78） |
| Israel | 20 to 24 | T1D | Female | 3.34 (2.99, 3.72） |
| Israel | 20 to 24 | T1D | Male | 3.49 (3.17, 3.85） |
| Israel | 25 to 29 | T1D | Both | 3.78 (3.41, 4.20） |
| Israel | 25 to 29 | T1D | Female | 3.65 (3.27, 4.07） |
| Israel | 25 to 29 | T1D | Male | 3.92 (3.55, 4.32） |
| Israel | 30 to 34 | T1D | Both | 4.14 (3.73, 4.61） |
| Israel | 30 to 34 | T1D | Female | 3.95 (3.53, 4.42） |
| Israel | 30 to 34 | T1D | Male | 4.34 (3.93, 4.80） |
| Israel | 35 to 39 | T1D | Both | 4.51 (4.04, 5.03） |
| Israel | 35 to 39 | T1D | Female | 4.23 (3.77, 4.75） |
| Israel | 35 to 39 | T1D | Male | 4.79 (4.32, 5.31） |
| Israel | 40 to 44 | T1D | Both | 4.86 (4.34, 5.43） |
| Israel | 40 to 44 | T1D | Female | 4.47 (3.97, 5.04） |
| Israel | 40 to 44 | T1D | Male | 5.25 (4.72, 5.84） |
| Israel | 45 to 49 | T1D | Both | 5.21 (4.64, 5.85） |

| | | | | |
|---|---|---|---|---|
| Israel | 45 to 49 | T1D | Female | 4.72 (4.18, 5.33） |
| Israel | 45 to 49 | T1D | Male | 5.72 (5.13, 6.38） |
| Israel | 5 to 9 | T1D | Both | 1.00 (1.00, 1.00） |
| Israel | 5 to 9 | T1D | Female | 1.00 (1.00, 1.00） |
| Israel | 5 to 9 | T1D | Male | 1.00 (1.00, 1.00） |
| Israel | 50 to 54 | T1D | Both | 5.55 (4.93, 6.25） |
| Israel | 50 to 54 | T1D | Female | 4.94 (4.36, 5.61） |
| Israel | 50 to 54 | T1D | Male | 6.19 (5.54, 6.93） |
| Israel | 55 to 59 | T1D | Both | 5.86 (5.19, 6.62） |
| Israel | 55 to 59 | T1D | Female | 5.14 (4.51, 5.85） |
| Israel | 55 to 59 | T1D | Male | 6.66 (5.93, 7.48） |
| Israel | 60 to 64 | T1D | Both | 6.17 (5.44, 7.00） |
| Israel | 60 to 64 | T1D | Female | 5.33 (4.66, 6.09） |
| Israel | 60 to 64 | T1D | Male | 7.12 (6.32, 8.03） |
| Israel | 65 to 69 | T1D | Both | 6.52 (5.72, 7.44） |
| Israel | 65 to 69 | T1D | Female | 5.55 (4.83, 6.38） |
| Israel | 65 to 69 | T1D | Male | 7.63 (6.73, 8.65） |
| Italy | 0 to 4 | T1D | Both | 0.19 (0.17, 0.21） |
| Italy | 0 to 4 | T1D | Female | 0.19 (0.17, 0.21） |
| Italy | 0 to 4 | T1D | Male | 0.19 (0.17, 0.21） |
| Italy | 10 to 14 | T1D | Both | 2.03 (1.93, 2.14） |
| Italy | 10 to 14 | T1D | Female | 2.03 (1.93, 2.12） |
| Italy | 10 to 14 | T1D | Male | 2.04 (1.93, 2.15） |
| Italy | 15 to 19 | T1D | Both | 3.04 (2.89, 3.20） |
| Italy | 15 to 19 | T1D | Female | 3.03 (2.89, 3.18） |
| Italy | 15 to 19 | T1D | Male | 3.06 (2.90, 3.22） |
| Italy | 20 to 24 | T1D | Both | 4.03 (3.83, 4.24） |
| Italy | 20 to 24 | T1D | Female | 4.00 (3.81, 4.19） |
| Italy | 20 to 24 | T1D | Male | 4.06 (3.85, 4.28） |
| Italy | 25 to 29 | T1D | Both | 5.19 (4.93, 5.46） |
| Italy | 25 to 29 | T1D | Female | 5.10 (4.87, 5.35） |
| Italy | 25 to 29 | T1D | Male | 5.26 (4.99, 5.55） |
| Italy | 30 to 34 | T1D | Both | 6.25 (5.94, 6.58） |

| | | | | |
|---|---|---|---|---|
| Italy | 30 to 34 | T1D | Female | 6.12 (5.83, 6.42） |
| Italy | 30 to 34 | T1D | Male | 6.39 (6.06, 6.74） |
| Italy | 35 to 39 | T1D | Both | 7.36 (6.99, 7.75） |
| Italy | 35 to 39 | T1D | Female | 7.16 (6.82, 7.51） |
| Italy | 35 to 39 | T1D | Male | 7.56 (7.16, 7.99） |
| Italy | 40 to 44 | T1D | Both | 8.56 (8.13, 9.03） |
| Italy | 40 to 44 | T1D | Female | 8.29 (7.89, 8.70） |
| Italy | 40 to 44 | T1D | Male | 8.84 (8.37, 9.34） |
| Italy | 45 to 49 | T1D | Both | 9.84 (9.34, 10.38） |
| Italy | 45 to 49 | T1D | Female | 9.49 (9.03, 9.98） |
| Italy | 45 to 49 | T1D | Male | 10.19 (9.64, 10.78） |
| Italy | 5 to 9 | T1D | Both | 1.00 (1.00, 1.00） |
| Italy | 5 to 9 | T1D | Female | 1.00 (1.00, 1.00） |
| Italy | 5 to 9 | T1D | Male | 1.00 (1.00, 1.00） |
| Italy | 50 to 54 | T1D | Both | 11.21 (10.62, 11.83） |
| Italy | 50 to 54 | T1D | Female | 10.79 (10.26, 11.35） |
| Italy | 50 to 54 | T1D | Male | 11.63 (11.00, 12.31） |
| Italy | 55 to 59 | T1D | Both | 12.70 (12.03, 13.42） |
| Italy | 55 to 59 | T1D | Female | 12.22 (11.61, 12.86） |
| Italy | 55 to 59 | T1D | Male | 13.20 (12.46, 13.97） |
| Italy | 60 to 64 | T1D | Both | 14.34 (13.58, 15.16） |
| Italy | 60 to 64 | T1D | Female | 13.80 (13.11, 14.53） |
| Italy | 60 to 64 | T1D | Male | 14.90 (14.06, 15.79） |
| Italy | 65 to 69 | T1D | Both | 16.13 (15.25, 17.05） |
| Italy | 65 to 69 | T1D | Female | 15.53 (14.74, 16.36） |
| Italy | 65 to 69 | T1D | Male | 16.72 (15.76, 17.73） |
| Jamaica | 0 to 4 | T1D | Both | 0.16 (0.13, 0.20） |
| Jamaica | 0 to 4 | T1D | Female | 0.15 (0.11, 0.20） |
| Jamaica | 0 to 4 | T1D | Male | 0.18 (0.13, 0.24） |
| Jamaica | 10 to 14 | T1D | Both | 2.20 (1.99, 2.42） |
| Jamaica | 10 to 14 | T1D | Female | 2.33 (2.03, 2.66） |
| Jamaica | 10 to 14 | T1D | Male | 2.06 (1.80, 2.37） |
| Jamaica | 15 to 19 | T1D | Both | 3.31 (3.01, 3.65） |

| | | | | |
|---|---|---|---|---|
| Jamaica | 15 to 19 | T1D | Female | 3.44 (3.01, 3.93） |
| Jamaica | 15 to 19 | T1D | Male | 3.18 (2.78, 3.65） |
| Jamaica | 20 to 24 | T1D | Both | 4.29 (3.89, 4.73） |
| Jamaica | 20 to 24 | T1D | Female | 4.26 (3.72, 4.88） |
| Jamaica | 20 to 24 | T1D | Male | 4.31 (3.75, 4.95） |
| Jamaica | 25 to 29 | T1D | Both | 5.33 (4.83, 5.89） |
| Jamaica | 25 to 29 | T1D | Female | 5.09 (4.42, 5.85） |
| Jamaica | 25 to 29 | T1D | Male | 5.61 (4.87, 6.46） |
| Jamaica | 30 to 34 | T1D | Both | 6.27 (5.66, 6.95） |
| Jamaica | 30 to 34 | T1D | Female | 5.80 (5.02, 6.70） |
| Jamaica | 30 to 34 | T1D | Male | 6.81 (5.88, 7.89） |
| Jamaica | 35 to 39 | T1D | Both | 7.22 (6.50, 8.03） |
| Jamaica | 35 to 39 | T1D | Female | 6.54 (5.63, 7.58） |
| Jamaica | 35 to 39 | T1D | Male | 8.02 (6.89, 9.32） |
| Jamaica | 40 to 44 | T1D | Both | 8.24 (7.39, 9.19） |
| Jamaica | 40 to 44 | T1D | Female | 7.32 (6.28, 8.53） |
| Jamaica | 40 to 44 | T1D | Male | 9.30 (7.97, 10.85） |
| Jamaica | 45 to 49 | T1D | Both | 9.29 (8.31, 10.40） |
| Jamaica | 45 to 49 | T1D | Female | 8.13 (6.94, 9.53） |
| Jamaica | 45 to 49 | T1D | Male | 10.61 (9.05, 12.44） |
| Jamaica | 5 to 9 | T1D | Both | 1.00 (1.00, 1.00） |
| Jamaica | 5 to 9 | T1D | Female | 1.00 (1.00, 1.00） |
| Jamaica | 5 to 9 | T1D | Male | 1.00 (1.00, 1.00） |
| Jamaica | 50 to 54 | T1D | Both | 10.36 (9.22, 11.64） |
| Jamaica | 50 to 54 | T1D | Female | 8.96 (7.60, 10.56） |
| Jamaica | 50 to 54 | T1D | Male | 11.92 (10.11, 14.05） |
| Jamaica | 55 to 59 | T1D | Both | 11.46 (10.15, 12.95） |
| Jamaica | 55 to 59 | T1D | Female | 9.89 (8.32, 11.75） |
| Jamaica | 55 to 59 | T1D | Male | 13.27 (11.18, 15.75） |
| Jamaica | 60 to 64 | T1D | Both | 12.64 (11.13, 14.36） |
| Jamaica | 60 to 64 | T1D | Female | 10.87 (9.07, 13.03） |
| Jamaica | 60 to 64 | T1D | Male | 14.65 (12.25, 17.53） |
| Jamaica | 65 to 69 | T1D | Both | 13.90 (12.15, 15.90） |

| | | | | |
|---|---|---|---|---|
| Jamaica | 65 to 69 | T1D | Female | 11.89 (9.81, 14.41） |
| Jamaica | 65 to 69 | T1D | Male | 16.17 (13.39, 19.53） |
| Japan | 0 to 4 | T1D | Both | 0.15 (0.14, 0.15） |
| Japan | 0 to 4 | T1D | Female | 0.14 (0.14, 0.15） |
| Japan | 0 to 4 | T1D | Male | 0.15 (0.15, 0.16） |
| Japan | 10 to 14 | T1D | Both | 1.85 (1.83, 1.87） |
| Japan | 10 to 14 | T1D | Female | 1.85 (1.82, 1.88） |
| Japan | 10 to 14 | T1D | Male | 1.85 (1.83, 1.88） |
| Japan | 15 to 19 | T1D | Both | 2.14 (2.12, 2.16） |
| Japan | 15 to 19 | T1D | Female | 2.13 (2.10, 2.16） |
| Japan | 15 to 19 | T1D | Male | 2.15 (2.12, 2.17） |
| Japan | 20 to 24 | T1D | Both | 2.24 (2.22, 2.27） |
| Japan | 20 to 24 | T1D | Female | 2.23 (2.20, 2.26） |
| Japan | 20 to 24 | T1D | Male | 2.26 (2.23, 2.29） |
| Japan | 25 to 29 | T1D | Both | 2.34 (2.32, 2.37） |
| Japan | 25 to 29 | T1D | Female | 2.32 (2.29, 2.36） |
| Japan | 25 to 29 | T1D | Male | 2.37 (2.33, 2.40） |
| Japan | 30 to 34 | T1D | Both | 2.43 (2.40, 2.46） |
| Japan | 30 to 34 | T1D | Female | 2.40 (2.37, 2.44） |
| Japan | 30 to 34 | T1D | Male | 2.46 (2.42, 2.49） |
| Japan | 35 to 39 | T1D | Both | 2.51 (2.48, 2.54） |
| Japan | 35 to 39 | T1D | Female | 2.48 (2.44, 2.52） |
| Japan | 35 to 39 | T1D | Male | 2.55 (2.51, 2.58） |
| Japan | 40 to 44 | T1D | Both | 2.60 (2.57, 2.63） |
| Japan | 40 to 44 | T1D | Female | 2.56 (2.52, 2.60） |
| Japan | 40 to 44 | T1D | Male | 2.63 (2.60, 2.67） |
| Japan | 45 to 49 | T1D | Both | 2.68 (2.65, 2.72） |
| Japan | 45 to 49 | T1D | Female | 2.64 (2.60, 2.68） |
| Japan | 45 to 49 | T1D | Male | 2.72 (2.68, 2.76） |
| Japan | 5 to 9 | T1D | Both | 1.00 (1.00, 1.00） |
| Japan | 5 to 9 | T1D | Female | 1.00 (1.00, 1.00） |
| Japan | 5 to 9 | T1D | Male | 1.00 (1.00, 1.00） |
| Japan | 50 to 54 | T1D | Both | 2.76 (2.73, 2.80） |

| | | | | |
|---|---|---|---|---|
| Japan | 50 to 54 | T1D | Female | 2.72 (2.68, 2.77） |
| Japan | 50 to 54 | T1D | Male | 2.81 (2.76, 2.85） |
| Japan | 55 to 59 | T1D | Both | 2.85 (2.81, 2.88） |
| Japan | 55 to 59 | T1D | Female | 2.81 (2.76, 2.85） |
| Japan | 55 to 59 | T1D | Male | 2.89 (2.84, 2.94） |
| Japan | 60 to 64 | T1D | Both | 2.93 (2.89, 2.97） |
| Japan | 60 to 64 | T1D | Female | 2.89 (2.84, 2.94） |
| Japan | 60 to 64 | T1D | Male | 2.97 (2.92, 3.02） |
| Japan | 65 to 69 | T1D | Both | 3.01 (2.97, 3.05） |
| Japan | 65 to 69 | T1D | Female | 2.98 (2.92, 3.03） |
| Japan | 65 to 69 | T1D | Male | 3.05 (3.00, 3.10） |
| Jordan | 0 to 4 | T1D | Both | 0.12 (0.11, 0.14） |
| Jordan | 0 to 4 | T1D | Female | 0.13 (0.11, 0.15） |
| Jordan | 0 to 4 | T1D | Male | 0.12 (0.10, 0.14） |
| Jordan | 10 to 14 | T1D | Both | 2.49 (2.38, 2.62） |
| Jordan | 10 to 14 | T1D | Female | 2.59 (2.42, 2.78） |
| Jordan | 10 to 14 | T1D | Male | 2.41 (2.26, 2.57） |
| Jordan | 15 to 19 | T1D | Both | 3.70 (3.52, 3.88） |
| Jordan | 15 to 19 | T1D | Female | 3.93 (3.66, 4.23） |
| Jordan | 15 to 19 | T1D | Male | 3.50 (3.27, 3.74） |
| Jordan | 20 to 24 | T1D | Both | 4.67 (4.43, 4.91） |
| Jordan | 20 to 24 | T1D | Female | 4.98 (4.62, 5.37） |
| Jordan | 20 to 24 | T1D | Male | 4.39 (4.10, 4.71） |
| Jordan | 25 to 29 | T1D | Both | 5.74 (5.44, 6.05） |
| Jordan | 25 to 29 | T1D | Female | 6.13 (5.67, 6.62） |
| Jordan | 25 to 29 | T1D | Male | 5.40 (5.03, 5.80） |
| Jordan | 30 to 34 | T1D | Both | 6.86 (6.50, 7.25） |
| Jordan | 30 to 34 | T1D | Female | 7.33 (6.76, 7.95） |
| Jordan | 30 to 34 | T1D | Male | 6.46 (5.99, 6.95） |
| Jordan | 35 to 39 | T1D | Both | 8.10 (7.65, 8.58） |
| Jordan | 35 to 39 | T1D | Female | 8.66 (7.95, 9.42） |
| Jordan | 35 to 39 | T1D | Male | 7.63 (7.06, 8.25） |
| Jordan | 40 to 44 | T1D | Both | 9.50 (8.94, 10.09） |

| | | | | |
|---|---|---|---|---|
| Jordan | 40 to 44 | T1D | Female | 10.15 (9.28, 11.09） |
| Jordan | 40 to 44 | T1D | Male | 8.94 (8.23, 9.70） |
| Jordan | 45 to 49 | T1D | Both | 11.04 (10.36, 11.78） |
| Jordan | 45 to 49 | T1D | Female | 11.79 (10.73, 12.97） |
| Jordan | 45 to 49 | T1D | Male | 10.38 (9.52, 11.33） |
| Jordan | 5 to 9 | T1D | Both | 1.00 (1.00, 1.00） |
| Jordan | 5 to 9 | T1D | Female | 1.00 (1.00, 1.00） |
| Jordan | 5 to 9 | T1D | Male | 1.00 (1.00, 1.00） |
| Jordan | 50 to 54 | T1D | Both | 12.76 (11.91, 13.67） |
| Jordan | 50 to 54 | T1D | Female | 13.66 (12.34, 15.11） |
| Jordan | 50 to 54 | T1D | Male | 11.97 (10.90, 13.14） |
| Jordan | 55 to 59 | T1D | Both | 14.71 (13.65, 15.84） |
| Jordan | 55 to 59 | T1D | Female | 15.77 (14.14, 17.58） |
| Jordan | 55 to 59 | T1D | Male | 13.77 (12.44, 15.24） |
| Jordan | 60 to 64 | T1D | Both | 16.91 (15.60, 18.33） |
| Jordan | 60 to 64 | T1D | Female | 18.15 (16.13, 20.43） |
| Jordan | 60 to 64 | T1D | Male | 15.81 (14.15, 17.66） |
| Jordan | 65 to 69 | T1D | Both | 19.38 (17.75, 21.17） |
| Jordan | 65 to 69 | T1D | Female | 20.87 (18.36, 23.73） |
| Jordan | 65 to 69 | T1D | Male | 18.09 (16.02, 20.42） |
| Kazakhstan | 0 to 4 | T1D | Both | 0.17 (0.16, 0.18） |
| Kazakhstan | 0 to 4 | T1D | Female | 0.17 (0.15, 0.19） |
| Kazakhstan | 0 to 4 | T1D | Male | 0.17 (0.15, 0.19） |
| Kazakhstan | 10 to 14 | T1D | Both | 2.20 (2.13, 2.28） |
| Kazakhstan | 10 to 14 | T1D | Female | 2.20 (2.09, 2.31） |
| Kazakhstan | 10 to 14 | T1D | Male | 2.21 (2.10, 2.32） |
| Kazakhstan | 15 to 19 | T1D | Both | 3.41 (3.29, 3.53） |
| Kazakhstan | 15 to 19 | T1D | Female | 3.36 (3.19, 3.53） |
| Kazakhstan | 15 to 19 | T1D | Male | 3.46 (3.30, 3.64） |
| Kazakhstan | 20 to 24 | T1D | Both | 4.50 (4.35, 4.66） |
| Kazakhstan | 20 to 24 | T1D | Female | 4.35 (4.14, 4.58） |
| Kazakhstan | 20 to 24 | T1D | Male | 4.66 (4.44, 4.89） |
| Kazakhstan | 25 to 29 | T1D | Both | 5.70 (5.50, 5.90） |

| | | | | |
|---|---|---|---|---|
| Kazakhstan | 25 to 29 | T1D | Female | 5.40 (5.13, 5.68） |
| Kazakhstan | 25 to 29 | T1D | Male | 6.01 (5.72, 6.31） |
| Kazakhstan | 30 to 34 | T1D | Both | 6.76 (6.52, 7.01） |
| Kazakhstan | 30 to 34 | T1D | Female | 6.31 (6.00, 6.65） |
| Kazakhstan | 30 to 34 | T1D | Male | 7.22 (6.87, 7.59） |
| Kazakhstan | 35 to 39 | T1D | Both | 7.75 (7.47, 8.04） |
| Kazakhstan | 35 to 39 | T1D | Female | 7.18 (6.80, 7.57） |
| Kazakhstan | 35 to 39 | T1D | Male | 8.36 (7.95, 8.81） |
| Kazakhstan | 40 to 44 | T1D | Both | 8.73 (8.40, 9.06） |
| Kazakhstan | 40 to 44 | T1D | Female | 8.03 (7.61, 8.48） |
| Kazakhstan | 40 to 44 | T1D | Male | 9.48 (9.00, 9.99） |
| Kazakhstan | 45 to 49 | T1D | Both | 9.73 (9.36, 10.11） |
| Kazakhstan | 45 to 49 | T1D | Female | 8.93 (8.45, 9.44） |
| Kazakhstan | 45 to 49 | T1D | Male | 10.62 (10.06, 11.20） |
| Kazakhstan | 5 to 9 | T1D | Both | 1.00 (1.00, 1.00） |
| Kazakhstan | 5 to 9 | T1D | Female | 1.00 (1.00, 1.00） |
| Kazakhstan | 5 to 9 | T1D | Male | 1.00 (1.00, 1.00） |
| Kazakhstan | 50 to 54 | T1D | Both | 10.76 (10.35, 11.20） |
| Kazakhstan | 50 to 54 | T1D | Female | 9.89 (9.35, 10.47） |
| Kazakhstan | 50 to 54 | T1D | Male | 11.78 (11.15, 12.44） |
| Kazakhstan | 55 to 59 | T1D | Both | 11.79 (11.32, 12.28） |
| Kazakhstan | 55 to 59 | T1D | Female | 10.88 (10.26, 11.54） |
| Kazakhstan | 55 to 59 | T1D | Male | 12.91 (12.19, 13.66） |
| Kazakhstan | 60 to 64 | T1D | Both | 12.80 (12.27, 13.36） |
| Kazakhstan | 60 to 64 | T1D | Female | 11.90 (11.20, 12.64） |
| Kazakhstan | 60 to 64 | T1D | Male | 14.01 (13.20, 14.87） |
| Kazakhstan | 65 to 69 | T1D | Both | 13.86 (13.26, 14.50） |
| Kazakhstan | 65 to 69 | T1D | Female | 13.01 (12.21, 13.86） |
| Kazakhstan | 65 to 69 | T1D | Male | 15.14 (14.21, 16.14） |
| Kenya | 0 to 4 | T1D | Both | 0.14 (0.14, 0.15） |
| Kenya | 0 to 4 | T1D | Female | 0.14 (0.13, 0.15） |
| Kenya | 0 to 4 | T1D | Male | 0.14 (0.13, 0.15） |
| Kenya | 10 to 14 | T1D | Both | 2.12 (2.07, 2.16） |

| | | | | |
|---|---|---|---|---|
| Kenya | 10 to 14 | T1D | Female | 2.12 (2.06, 2.18） |
| Kenya | 10 to 14 | T1D | Male | 2.12 (2.06, 2.18） |
| Kenya | 15 to 19 | T1D | Both | 3.12 (3.06, 3.18） |
| Kenya | 15 to 19 | T1D | Female | 3.12 (3.03, 3.21） |
| Kenya | 15 to 19 | T1D | Male | 3.12 (3.03, 3.21） |
| Kenya | 20 to 24 | T1D | Both | 4.08 (4.00, 4.17） |
| Kenya | 20 to 24 | T1D | Female | 4.09 (3.97, 4.22） |
| Kenya | 20 to 24 | T1D | Male | 4.08 (3.96, 4.20） |
| Kenya | 25 to 29 | T1D | Both | 5.13 (5.02, 5.24） |
| Kenya | 25 to 29 | T1D | Female | 5.14 (4.99, 5.30） |
| Kenya | 25 to 29 | T1D | Male | 5.12 (4.97, 5.28） |
| Kenya | 30 to 34 | T1D | Both | 5.81 (5.68, 5.94） |
| Kenya | 30 to 34 | T1D | Female | 5.83 (5.64, 6.02） |
| Kenya | 30 to 34 | T1D | Male | 5.80 (5.62, 5.98） |
| Kenya | 35 to 39 | T1D | Both | 6.32 (6.17, 6.47） |
| Kenya | 35 to 39 | T1D | Female | 6.34 (6.13, 6.56） |
| Kenya | 35 to 39 | T1D | Male | 6.29 (6.09, 6.51） |
| Kenya | 40 to 44 | T1D | Both | 6.74 (6.58, 6.91） |
| Kenya | 40 to 44 | T1D | Female | 6.78 (6.55, 7.03） |
| Kenya | 40 to 44 | T1D | Male | 6.70 (6.47, 6.94） |
| Kenya | 45 to 49 | T1D | Both | 7.09 (6.91, 7.28） |
| Kenya | 45 to 49 | T1D | Female | 7.16 (6.89, 7.43） |
| Kenya | 45 to 49 | T1D | Male | 7.03 (6.78, 7.30） |
| Kenya | 5 to 9 | T1D | Both | 1.00 (1.00, 1.00） |
| Kenya | 5 to 9 | T1D | Female | 1.00 (1.00, 1.00） |
| Kenya | 5 to 9 | T1D | Male | 1.00 (1.00, 1.00） |
| Kenya | 50 to 54 | T1D | Both | 7.38 (7.17, 7.59） |
| Kenya | 50 to 54 | T1D | Female | 7.47 (7.17, 7.78） |
| Kenya | 50 to 54 | T1D | Male | 7.29 (7.01, 7.58） |
| Kenya | 55 to 59 | T1D | Both | 7.61 (7.38, 7.84） |
| Kenya | 55 to 59 | T1D | Female | 7.73 (7.40, 8.07） |
| Kenya | 55 to 59 | T1D | Male | 7.49 (7.18, 7.81） |
| Kenya | 60 to 64 | T1D | Both | 7.79 (7.53, 8.05） |

| | | | | |
|---|---|---|---|---|
| Kenya | 60 to 64 | T1D | Female | 7.95 (7.58, 8.33） |
| Kenya | 60 to 64 | T1D | Male | 7.63 (7.29, 7.99） |
| Kenya | 65 to 69 | T1D | Both | 7.92 (7.63, 8.21） |
| Kenya | 65 to 69 | T1D | Female | 8.12 (7.71, 8.55） |
| Kenya | 65 to 69 | T1D | Male | 7.73 (7.34, 8.14） |
| Kiribati | 0 to 4 | T1D | Both | 0.26 (0.08, 0.87） |
| Kiribati | 0 to 4 | T1D | Female | 0.06 (0.01, 0.96） |
| Kiribati | 0 to 4 | T1D | Male | 0.06 (0.01, 0.95） |
| Kiribati | 10 to 14 | T1D | Both | 2.17 (1.17, 4.03） |
| Kiribati | 10 to 14 | T1D | Female | 1.77 (0.75, 4.17） |
| Kiribati | 10 to 14 | T1D | Male | 1.78 (0.76, 4.21） |
| Kiribati | 15 to 19 | T1D | Both | 3.06 (1.64, 5.71） |
| Kiribati | 15 to 19 | T1D | Female | 2.51 (1.05, 6.01） |
| Kiribati | 15 to 19 | T1D | Male | 2.60 (1.09, 6.24） |
| Kiribati | 20 to 24 | T1D | Both | 3.89 (2.05, 7.40） |
| Kiribati | 20 to 24 | T1D | Female | 3.29 (1.34, 8.12） |
| Kiribati | 20 to 24 | T1D | Male | 3.62 (1.47, 8.92） |
| Kiribati | 25 to 29 | T1D | Both | 4.65 (2.40, 9.02） |
| Kiribati | 25 to 29 | T1D | Female | 3.81 (1.49, 9.77） |
| Kiribati | 25 to 29 | T1D | Male | 4.46 (1.75, 11.36） |
| Kiribati | 30 to 34 | T1D | Both | 5.31 (2.67, 10.57） |
| Kiribati | 30 to 34 | T1D | Female | 4.40 (1.66, 11.67） |
| Kiribati | 30 to 34 | T1D | Male | 5.54 (2.11, 14.57） |
| Kiribati | 35 to 39 | T1D | Both | 5.73 (2.80, 11.72） |
| Kiribati | 35 to 39 | T1D | Female | 4.82 (1.75, 13.27） |
| Kiribati | 35 to 39 | T1D | Male | 5.92 (2.16, 16.19） |
| Kiribati | 40 to 44 | T1D | Both | 6.34 (3.02, 13.31） |
| Kiribati | 40 to 44 | T1D | Female | 5.32 (1.86, 15.26） |
| Kiribati | 40 to 44 | T1D | Male | 6.23 (2.19, 17.75） |
| Kiribati | 45 to 49 | T1D | Both | 6.97 (3.21, 15.10） |
| Kiribati | 45 to 49 | T1D | Female | 5.85 (1.95, 17.55） |
| Kiribati | 45 to 49 | T1D | Male | 6.83 (2.30, 20.23） |
| Kiribati | 5 to 9 | T1D | Both | 1.00 (1.00, 1.00） |

| | | | | |
|---|---|---|---|---|
| Kiribati | 5 to 9 | T1D | Female | 1.00 (1.00, 1.00） |
| Kiribati | 5 to 9 | T1D | Male | 1.00 (1.00, 1.00） |
| Kiribati | 50 to 54 | T1D | Both | 7.34 (3.25, 16.60） |
| Kiribati | 50 to 54 | T1D | Female | 5.93 (1.85, 19.03） |
| Kiribati | 50 to 54 | T1D | Male | 7.50 (2.41, 23.35） |
| Kiribati | 55 to 59 | T1D | Both | 8.03 (3.39, 18.98） |
| Kiribati | 55 to 59 | T1D | Female | 6.55 (1.92, 22.34） |
| Kiribati | 55 to 59 | T1D | Male | 7.53 (2.23, 25.43） |
| Kiribati | 60 to 64 | T1D | Both | 8.26 (3.26, 20.93） |
| Kiribati | 60 to 64 | T1D | Female | 6.52 (1.71, 24.87） |
| Kiribati | 60 to 64 | T1D | Male | 7.63 (2.03, 28.70） |
| Kiribati | 65 to 69 | T1D | Both | 9.38 (3.35, 26.25） |
| Kiribati | 65 to 69 | T1D | Female | 7.70 (1.83, 32.44） |
| Kiribati | 65 to 69 | T1D | Male | 8.99 (2.02, 39.92） |
| Kuwait | 0 to 4 | T1D | Both | 0.17 (0.15, 0.20） |
| Kuwait | 0 to 4 | T1D | Female | 0.21 (0.17, 0.26） |
| Kuwait | 0 to 4 | T1D | Male | 0.16 (0.13, 0.19） |
| Kuwait | 10 to 14 | T1D | Both | 2.36 (2.19, 2.54） |
| Kuwait | 10 to 14 | T1D | Female | 2.28 (2.03, 2.57） |
| Kuwait | 10 to 14 | T1D | Male | 2.39 (2.18, 2.63） |
| Kuwait | 15 to 19 | T1D | Both | 3.98 (3.69, 4.29） |
| Kuwait | 15 to 19 | T1D | Female | 3.57 (3.17, 4.03） |
| Kuwait | 15 to 19 | T1D | Male | 4.22 (3.82, 4.65） |
| Kuwait | 20 to 24 | T1D | Both | 5.56 (5.16, 6.00） |
| Kuwait | 20 to 24 | T1D | Female | 4.51 (4.00, 5.08） |
| Kuwait | 20 to 24 | T1D | Male | 6.28 (5.70, 6.93） |
| Kuwait | 25 to 29 | T1D | Both | 7.35 (6.81, 7.92） |
| Kuwait | 25 to 29 | T1D | Female | 5.41 (4.80, 6.09） |
| Kuwait | 25 to 29 | T1D | Male | 8.70 (7.89, 9.60） |
| Kuwait | 30 to 34 | T1D | Both | 9.31 (8.63, 10.05） |
| Kuwait | 30 to 34 | T1D | Female | 6.19 (5.49, 6.99） |
| Kuwait | 30 to 34 | T1D | Male | 11.55 (10.46, 12.76） |
| Kuwait | 35 to 39 | T1D | Both | 11.38 (10.53, 12.31） |

| | | | | |
|---|---|---|---|---|
| Kuwait | 35 to 39 | T1D | Female | 6.93 (6.12, 7.85） |
| Kuwait | 35 to 39 | T1D | Male | 14.83 (13.40, 16.42） |
| Kuwait | 40 to 44 | T1D | Both | 13.73 (12.67, 14.87） |
| Kuwait | 40 to 44 | T1D | Female | 7.69 (6.77, 8.74） |
| Kuwait | 40 to 44 | T1D | Male | 18.65 (16.82, 20.68） |
| Kuwait | 45 to 49 | T1D | Both | 16.40 (15.11, 17.80） |
| Kuwait | 45 to 49 | T1D | Female | 8.48 (7.44, 9.67） |
| Kuwait | 45 to 49 | T1D | Male | 23.17 (20.85, 25.75） |
| Kuwait | 5 to 9 | T1D | Both | 1.00 (1.00, 1.00） |
| Kuwait | 5 to 9 | T1D | Female | 1.00 (1.00, 1.00） |
| Kuwait | 5 to 9 | T1D | Male | 1.00 (1.00, 1.00） |
| Kuwait | 50 to 54 | T1D | Both | 19.41 (17.83, 21.12） |
| Kuwait | 50 to 54 | T1D | Female | 9.32 (8.12, 10.69） |
| Kuwait | 50 to 54 | T1D | Male | 28.28 (25.37, 31.53） |
| Kuwait | 55 to 59 | T1D | Both | 22.61 (20.68, 24.71） |
| Kuwait | 55 to 59 | T1D | Female | 10.20 (8.82, 11.81） |
| Kuwait | 55 to 59 | T1D | Male | 34.20 (30.53, 38.32） |
| Kuwait | 60 to 64 | T1D | Both | 26.48 (24.09, 29.11） |
| Kuwait | 60 to 64 | T1D | Female | 11.18 (9.55, 13.09） |
| Kuwait | 60 to 64 | T1D | Male | 41.34 (36.67, 46.60） |
| Kuwait | 65 to 69 | T1D | Both | 30.87 (27.87, 34.20） |
| Kuwait | 65 to 69 | T1D | Female | 12.27 (10.32, 14.59） |
| Kuwait | 65 to 69 | T1D | Male | 49.50 (43.54, 56.28） |
| Kyrgyzstan | 0 to 4 | T1D | Both | 0.17 (0.15, 0.19） |
| Kyrgyzstan | 0 to 4 | T1D | Female | 0.17 (0.14, 0.20） |
| Kyrgyzstan | 0 to 4 | T1D | Male | 0.17 (0.15, 0.20） |
| Kyrgyzstan | 10 to 14 | T1D | Both | 2.20 (2.09, 2.33） |
| Kyrgyzstan | 10 to 14 | T1D | Female | 2.21 (2.04, 2.39） |
| Kyrgyzstan | 10 to 14 | T1D | Male | 2.20 (2.04, 2.38） |
| Kyrgyzstan | 15 to 19 | T1D | Both | 3.42 (3.24, 3.61） |
| Kyrgyzstan | 15 to 19 | T1D | Female | 3.38 (3.12, 3.66） |
| Kyrgyzstan | 15 to 19 | T1D | Male | 3.46 (3.21, 3.74） |
| Kyrgyzstan | 20 to 24 | T1D | Both | 4.52 (4.27, 4.77） |

| | | | | |
|---|---|---|---|---|
| Kyrgyzstan | 20 to 24 | T1D | Female | 4.38 (4.05, 4.75） |
| Kyrgyzstan | 20 to 24 | T1D | Male | 4.65 (4.31, 5.02） |
| Kyrgyzstan | 25 to 29 | T1D | Both | 5.71 (5.39, 6.04） |
| Kyrgyzstan | 25 to 29 | T1D | Female | 5.43 (5.00, 5.89） |
| Kyrgyzstan | 25 to 29 | T1D | Male | 5.98 (5.53, 6.47） |
| Kyrgyzstan | 30 to 34 | T1D | Both | 6.75 (6.37, 7.16） |
| Kyrgyzstan | 30 to 34 | T1D | Female | 6.33 (5.82, 6.89） |
| Kyrgyzstan | 30 to 34 | T1D | Male | 7.17 (6.61, 7.77） |
| Kyrgyzstan | 35 to 39 | T1D | Both | 7.71 (7.26, 8.19） |
| Kyrgyzstan | 35 to 39 | T1D | Female | 7.17 (6.57, 7.83） |
| Kyrgyzstan | 35 to 39 | T1D | Male | 8.26 (7.60, 8.99） |
| Kyrgyzstan | 40 to 44 | T1D | Both | 8.64 (8.12, 9.20） |
| Kyrgyzstan | 40 to 44 | T1D | Female | 7.99 (7.31, 8.74） |
| Kyrgyzstan | 40 to 44 | T1D | Male | 9.32 (8.55, 10.16） |
| Kyrgyzstan | 45 to 49 | T1D | Both | 9.59 (8.99, 10.23） |
| Kyrgyzstan | 45 to 49 | T1D | Female | 8.85 (8.07, 9.71） |
| Kyrgyzstan | 45 to 49 | T1D | Male | 10.38 (9.49, 11.35） |
| Kyrgyzstan | 5 to 9 | T1D | Both | 1.00 (1.00, 1.00） |
| Kyrgyzstan | 5 to 9 | T1D | Female | 1.00 (1.00, 1.00） |
| Kyrgyzstan | 5 to 9 | T1D | Male | 1.00 (1.00, 1.00） |
| Kyrgyzstan | 50 to 54 | T1D | Both | 10.54 (9.86, 11.27） |
| Kyrgyzstan | 50 to 54 | T1D | Female | 9.74 (8.84, 10.72） |
| Kyrgyzstan | 50 to 54 | T1D | Male | 11.44 (10.42, 12.55） |
| Kyrgyzstan | 55 to 59 | T1D | Both | 11.46 (10.68, 12.30） |
| Kyrgyzstan | 55 to 59 | T1D | Female | 10.64 (9.61, 11.77） |
| Kyrgyzstan | 55 to 59 | T1D | Male | 12.45 (11.28, 13.73） |
| Kyrgyzstan | 60 to 64 | T1D | Both | 12.36 (11.47, 13.32） |
| Kyrgyzstan | 60 to 64 | T1D | Female | 11.54 (10.37, 12.84） |
| Kyrgyzstan | 60 to 64 | T1D | Male | 13.42 (12.08, 14.90） |
| Kyrgyzstan | 65 to 69 | T1D | Both | 13.33 (12.29, 14.45） |
| Kyrgyzstan | 65 to 69 | T1D | Female | 12.54 (11.18, 14.07） |
| Kyrgyzstan | 65 to 69 | T1D | Male | 14.41 (12.86, 16.16） |
| Lao People's Democratic Republic | 0 to 4 | T1D | Both | 0.14 (0.11, 0.16） |

| | | | | |
|---|---|---|---|---|
| Lao People's Democratic Republic | 0 to 4 | T1D | Female | 0.14 (0.10, 0.18） |
| Lao People's Democratic Republic | 0 to 4 | T1D | Male | 0.14 (0.10, 0.18） |
| Lao People's Democratic Republic | 10 to 14 | T1D | Both | 2.17 (2.00, 2.36） |
| Lao People's Democratic Republic | 10 to 14 | T1D | Female | 2.14 (1.91, 2.40） |
| Lao People's Democratic Republic | 10 to 14 | T1D | Male | 2.20 (1.96, 2.48） |
| Lao People's Democratic Republic | 15 to 19 | T1D | Both | 3.03 (2.79, 3.30） |
| Lao People's Democratic Republic | 15 to 19 | T1D | Female | 2.92 (2.60, 3.28） |
| Lao People's Democratic Republic | 15 to 19 | T1D | Male | 3.15 (2.80, 3.55） |
| Lao People's Democratic Republic | 20 to 24 | T1D | Both | 3.68 (3.38, 4.00） |
| Lao People's Democratic Republic | 20 to 24 | T1D | Female | 3.44 (3.06, 3.88） |
| Lao People's Democratic Republic | 20 to 24 | T1D | Male | 3.92 (3.47, 4.43） |
| Lao People's Democratic Republic | 25 to 29 | T1D | Both | 4.27 (3.91, 4.66） |
| Lao People's Democratic Republic | 25 to 29 | T1D | Female | 3.91 (3.45, 4.42） |
| Lao People's Democratic Republic | 25 to 29 | T1D | Male | 4.65 (4.10, 5.27） |
| Lao People's Democratic Republic | 30 to 34 | T1D | Both | 4.67 (4.26, 5.12） |
| Lao People's Democratic Republic | 30 to 34 | T1D | Female | 4.21 (3.70, 4.80） |
| Lao People's Democratic Republic | 30 to 34 | T1D | Male | 5.16 (4.52, 5.89） |
| Lao People's Democratic Republic | 35 to 39 | T1D | Both | 4.99 (4.53, 5.50） |
| Lao People's Democratic Republic | 35 to 39 | T1D | Female | 4.46 (3.89, 5.11） |
| Lao People's Democratic Republic | 35 to 39 | T1D | Male | 5.56 (4.84, 6.39） |
| Lao People's Democratic Republic | 40 to 44 | T1D | Both | 5.28 (4.76, 5.85） |
| Lao People's Democratic Republic | 40 to 44 | T1D | Female | 4.69 (4.06, 5.41） |
| Lao People's Democratic Republic | 40 to 44 | T1D | Male | 5.90 (5.10, 6.83） |
| Lao People's Democratic Republic | 45 to 49 | T1D | Both | 5.51 (4.95, 6.14） |
| Lao People's Democratic Republic | 45 to 49 | T1D | Female | 4.90 (4.21, 5.70） |
| Lao People's Democratic Republic | 45 to 49 | T1D | Male | 6.19 (5.31, 7.22） |
| Lao People's Democratic Republic | 5 to 9 | T1D | Both | 1.00 (1.00, 1.00） |
| Lao People's Democratic Republic | 5 to 9 | T1D | Female | 1.00 (1.00, 1.00） |
| Lao People's Democratic Republic | 5 to 9 | T1D | Male | 1.00 (1.00, 1.00） |
| Lao People's Democratic Republic | 50 to 54 | T1D | Both | 5.72 (5.10, 6.41） |
| Lao People's Democratic Republic | 50 to 54 | T1D | Female | 5.06 (4.31, 5.95） |
| Lao People's Democratic Republic | 50 to 54 | T1D | Male | 6.43 (5.47, 7.57） |
| Lao People's Democratic Republic | 55 to 59 | T1D | Both | 5.92 (5.23, 6.69） |

| | | | | |
|---|---|---|---|---|
| Lao People's Democratic Republic | 55 to 59 | T1D | Female | 5.23 (4.40, 6.22） |
| Lao People's Democratic Republic | 55 to 59 | T1D | Male | 6.65 (5.59, 7.92） |
| Lao People's Democratic Republic | 60 to 64 | T1D | Both | 6.09 (5.33, 6.96） |
| Lao People's Democratic Republic | 60 to 64 | T1D | Female | 5.40 (4.46, 6.52） |
| Lao People's Democratic Republic | 60 to 64 | T1D | Male | 6.80 (5.63, 8.23） |
| Lao People's Democratic Republic | 65 to 69 | T1D | Both | 6.25 (5.39, 7.26） |
| Lao People's Democratic Republic | 65 to 69 | T1D | Female | 5.58 (4.52, 6.89） |
| Lao People's Democratic Republic | 65 to 69 | T1D | Male | 6.92 (5.60, 8.55） |
| Latvia | 0 to 4 | T1D | Both | 0.18 (0.14, 0.24） |
| Latvia | 0 to 4 | T1D | Female | 0.17 (0.11, 0.25） |
| Latvia | 0 to 4 | T1D | Male | 0.19 (0.13, 0.28） |
| Latvia | 10 to 14 | T1D | Both | 2.33 (2.06, 2.63） |
| Latvia | 10 to 14 | T1D | Female | 2.38 (2.00, 2.85） |
| Latvia | 10 to 14 | T1D | Male | 2.29 (1.93, 2.72） |
| Latvia | 15 to 19 | T1D | Both | 3.62 (3.21, 4.08） |
| Latvia | 15 to 19 | T1D | Female | 3.60 (3.03, 4.28） |
| Latvia | 15 to 19 | T1D | Male | 3.66 (3.10, 4.33） |
| Latvia | 20 to 24 | T1D | Both | 4.73 (4.20, 5.33） |
| Latvia | 20 to 24 | T1D | Female | 4.58 (3.85, 5.44） |
| Latvia | 20 to 24 | T1D | Male | 4.91 (4.16, 5.80） |
| Latvia | 25 to 29 | T1D | Both | 5.99 (5.31, 6.76） |
| Latvia | 25 to 29 | T1D | Female | 5.63 (4.73, 6.71） |
| Latvia | 25 to 29 | T1D | Male | 6.37 (5.39, 7.53） |
| Latvia | 30 to 34 | T1D | Both | 7.26 (6.42, 8.21） |
| Latvia | 30 to 34 | T1D | Female | 6.68 (5.59, 7.98） |
| Latvia | 30 to 34 | T1D | Male | 7.88 (6.65, 9.34） |
| Latvia | 35 to 39 | T1D | Both | 8.64 (7.63, 9.80） |
| Latvia | 35 to 39 | T1D | Female | 7.83 (6.53, 9.39） |
| Latvia | 35 to 39 | T1D | Male | 9.52 (8.01, 11.32） |
| Latvia | 40 to 44 | T1D | Both | 10.19 (8.98, 11.57） |
| Latvia | 40 to 44 | T1D | Female | 9.13 (7.60, 10.97） |
| Latvia | 40 to 44 | T1D | Male | 11.35 (9.52, 13.52） |
| Latvia | 45 to 49 | T1D | Both | 11.88 (10.45, 13.50） |

| | | | | |
|---|---|---|---|---|
| Latvia | 45 to 49 | T1D | Female | 10.57 (8.78, 12.73） |
| Latvia | 45 to 49 | T1D | Male | 13.35 (11.18, 15.94） |
| Latvia | 5 to 9 | T1D | Both | 1.00 (1.00, 1.00） |
| Latvia | 5 to 9 | T1D | Female | 1.00 (1.00, 1.00） |
| Latvia | 5 to 9 | T1D | Male | 1.00 (1.00, 1.00） |
| Latvia | 50 to 54 | T1D | Both | 13.70 (12.03, 15.60） |
| Latvia | 50 to 54 | T1D | Female | 12.18 (10.09, 14.70） |
| Latvia | 50 to 54 | T1D | Male | 15.50 (12.95, 18.56） |
| Latvia | 55 to 59 | T1D | Both | 15.70 (13.76, 17.91） |
| Latvia | 55 to 59 | T1D | Female | 13.99 (11.56, 16.93） |
| Latvia | 55 to 59 | T1D | Male | 17.86 (14.88, 21.44） |
| Latvia | 60 to 64 | T1D | Both | 17.91 (15.67, 20.48） |
| Latvia | 60 to 64 | T1D | Female | 16.10 (13.27, 19.53） |
| Latvia | 60 to 64 | T1D | Male | 20.46 (16.99, 24.65） |
| Latvia | 65 to 69 | T1D | Both | 20.40 (17.80, 23.38） |
| Latvia | 65 to 69 | T1D | Female | 18.51 (15.21, 22.53） |
| Latvia | 65 to 69 | T1D | Male | 23.37 (19.31, 28.28） |
| Lebanon | 0 to 4 | T1D | Both | 0.15 (0.13, 0.17） |
| Lebanon | 0 to 4 | T1D | Female | 0.16 (0.13, 0.19） |
| Lebanon | 0 to 4 | T1D | Male | 0.14 (0.12, 0.17） |
| Lebanon | 10 to 14 | T1D | Both | 2.30 (2.15, 2.45） |
| Lebanon | 10 to 14 | T1D | Female | 2.39 (2.17, 2.64） |
| Lebanon | 10 to 14 | T1D | Male | 2.22 (2.04, 2.43） |
| Lebanon | 15 to 19 | T1D | Both | 3.60 (3.37, 3.85） |
| Lebanon | 15 to 19 | T1D | Female | 3.80 (3.45, 4.20） |
| Lebanon | 15 to 19 | T1D | Male | 3.44 (3.15, 3.76） |
| Lebanon | 20 to 24 | T1D | Both | 4.81 (4.50, 5.14） |
| Lebanon | 20 to 24 | T1D | Female | 5.03 (4.55, 5.55） |
| Lebanon | 20 to 24 | T1D | Male | 4.63 (4.24, 5.07） |
| Lebanon | 25 to 29 | T1D | Both | 6.20 (5.80, 6.63） |
| Lebanon | 25 to 29 | T1D | Female | 6.40 (5.79, 7.07） |
| Lebanon | 25 to 29 | T1D | Male | 6.05 (5.52, 6.62） |
| Lebanon | 30 to 34 | T1D | Both | 7.58 (7.08, 8.13） |

| | | | | |
|---|---|---|---|---|
| Lebanon | 30 to 34 | T1D | Female | 7.76 (7.00, 8.60） |
| Lebanon | 30 to 34 | T1D | Male | 7.49 (6.82, 8.22） |
| Lebanon | 35 to 39 | T1D | Both | 9.02 (8.40, 9.69） |
| Lebanon | 35 to 39 | T1D | Female | 9.18 (8.26, 10.21） |
| Lebanon | 35 to 39 | T1D | Male | 8.98 (8.15, 9.91） |
| Lebanon | 40 to 44 | T1D | Both | 10.56 (9.81, 11.37） |
| Lebanon | 40 to 44 | T1D | Female | 10.71 (9.60, 11.94） |
| Lebanon | 40 to 44 | T1D | Male | 10.59 (9.57, 11.71） |
| Lebanon | 45 to 49 | T1D | Both | 12.25 (11.35, 13.22） |
| Lebanon | 45 to 49 | T1D | Female | 12.38 (11.07, 13.85） |
| Lebanon | 45 to 49 | T1D | Male | 12.31 (11.09, 13.66） |
| Lebanon | 5 to 9 | T1D | Both | 1.00 (1.00, 1.00） |
| Lebanon | 5 to 9 | T1D | Female | 1.00 (1.00, 1.00） |
| Lebanon | 5 to 9 | T1D | Male | 1.00 (1.00, 1.00） |
| Lebanon | 50 to 54 | T1D | Both | 14.04 (12.98, 15.20） |
| Lebanon | 50 to 54 | T1D | Female | 14.18 (12.63, 15.92） |
| Lebanon | 50 to 54 | T1D | Male | 14.13 (12.68, 15.74） |
| Lebanon | 55 to 59 | T1D | Both | 16.03 (14.77, 17.40） |
| Lebanon | 55 to 59 | T1D | Female | 16.19 (14.36, 18.26） |
| Lebanon | 55 to 59 | T1D | Male | 16.09 (14.37, 18.01） |
| Lebanon | 60 to 64 | T1D | Both | 18.16 (16.69, 19.77） |
| Lebanon | 60 to 64 | T1D | Female | 18.42 (16.27, 20.85） |
| Lebanon | 60 to 64 | T1D | Male | 18.20 (16.19, 20.45） |
| Lebanon | 65 to 69 | T1D | Both | 20.55 (18.82, 22.44） |
| Lebanon | 65 to 69 | T1D | Female | 20.88 (18.36, 23.74） |
| Lebanon | 65 to 69 | T1D | Male | 20.53 (18.19, 23.18） |
| Lesotho | 0 to 4 | T1D | Both | 0.16 (0.13, 0.20） |
| Lesotho | 0 to 4 | T1D | Female | 0.16 (0.12, 0.22） |
| Lesotho | 0 to 4 | T1D | Male | 0.16 (0.12, 0.21） |
| Lesotho | 10 to 14 | T1D | Both | 2.07 (1.89, 2.26） |
| Lesotho | 10 to 14 | T1D | Female | 2.07 (1.82, 2.35） |
| Lesotho | 10 to 14 | T1D | Male | 2.07 (1.82, 2.34） |
| Lesotho | 15 to 19 | T1D | Both | 3.01 (2.75, 3.29） |

| | | | | |
|---|---|---|---|---|
| Lesotho | 15 to 19 | T1D | Female | 3.00 (2.64, 3.42） |
| Lesotho | 15 to 19 | T1D | Male | 3.03 (2.67, 3.43） |
| Lesotho | 20 to 24 | T1D | Both | 3.82 (3.48, 4.18） |
| Lesotho | 20 to 24 | T1D | Female | 3.81 (3.34, 4.34） |
| Lesotho | 20 to 24 | T1D | Male | 3.83 (3.38, 4.35） |
| Lesotho | 25 to 29 | T1D | Both | 4.65 (4.24, 5.10） |
| Lesotho | 25 to 29 | T1D | Female | 4.65 (4.07, 5.31） |
| Lesotho | 25 to 29 | T1D | Male | 4.67 (4.10, 5.31） |
| Lesotho | 30 to 34 | T1D | Both | 5.25 (4.77, 5.78） |
| Lesotho | 30 to 34 | T1D | Female | 5.25 (4.57, 6.03） |
| Lesotho | 30 to 34 | T1D | Male | 5.25 (4.59, 6.01） |
| Lesotho | 35 to 39 | T1D | Both | 5.69 (5.14, 6.29） |
| Lesotho | 35 to 39 | T1D | Female | 5.70 (4.93, 6.59） |
| Lesotho | 35 to 39 | T1D | Male | 5.68 (4.93, 6.54） |
| Lesotho | 40 to 44 | T1D | Both | 6.02 (5.42, 6.70） |
| Lesotho | 40 to 44 | T1D | Female | 6.05 (5.20, 7.04） |
| Lesotho | 40 to 44 | T1D | Male | 6.01 (5.18, 6.97） |
| Lesotho | 45 to 49 | T1D | Both | 6.32 (5.66, 7.06） |
| Lesotho | 45 to 49 | T1D | Female | 6.37 (5.44, 7.46） |
| Lesotho | 45 to 49 | T1D | Male | 6.29 (5.38, 7.36） |
| Lesotho | 5 to 9 | T1D | Both | 1.00 (1.00, 1.00） |
| Lesotho | 5 to 9 | T1D | Female | 1.00 (1.00, 1.00） |
| Lesotho | 5 to 9 | T1D | Male | 1.00 (1.00, 1.00） |
| Lesotho | 50 to 54 | T1D | Both | 6.58 (5.86, 7.39） |
| Lesotho | 50 to 54 | T1D | Female | 6.65 (5.64, 7.83） |
| Lesotho | 50 to 54 | T1D | Male | 6.54 (5.55, 7.71） |
| Lesotho | 55 to 59 | T1D | Both | 6.81 (6.02, 7.69） |
| Lesotho | 55 to 59 | T1D | Female | 6.89 (5.80, 8.19） |
| Lesotho | 55 to 59 | T1D | Male | 6.75 (5.67, 8.03） |
| Lesotho | 60 to 64 | T1D | Both | 6.96 (6.11, 7.93） |
| Lesotho | 60 to 64 | T1D | Female | 7.08 (5.89, 8.50） |
| Lesotho | 60 to 64 | T1D | Male | 6.90 (5.72, 8.32） |
| Lesotho | 65 to 69 | T1D | Both | 7.03 (6.10, 8.09） |

| | | | | |
|---|---|---|---|---|
| Lesotho | 65 to 69 | T1D | Female | 7.19 (5.92, 8.74） |
| Lesotho | 65 to 69 | T1D | Male | 6.95 (5.66, 8.54） |
| Liberia | 0 to 4 | T1D | Both | 0.16 (0.14, 0.18） |
| Liberia | 0 to 4 | T1D | Female | 0.16 (0.13, 0.20） |
| Liberia | 0 to 4 | T1D | Male | 0.16 (0.13, 0.19） |
| Liberia | 10 to 14 | T1D | Both | 2.07 (1.93, 2.22） |
| Liberia | 10 to 14 | T1D | Female | 2.07 (1.88, 2.29） |
| Liberia | 10 to 14 | T1D | Male | 2.07 (1.88, 2.28） |
| Liberia | 15 to 19 | T1D | Both | 3.03 (2.82, 3.25） |
| Liberia | 15 to 19 | T1D | Female | 3.03 (2.74, 3.36） |
| Liberia | 15 to 19 | T1D | Male | 3.03 (2.74, 3.34） |
| Liberia | 20 to 24 | T1D | Both | 3.86 (3.59, 4.16） |
| Liberia | 20 to 24 | T1D | Female | 3.88 (3.49, 4.30） |
| Liberia | 20 to 24 | T1D | Male | 3.86 (3.48, 4.28） |
| Liberia | 25 to 29 | T1D | Both | 4.73 (4.38, 5.10） |
| Liberia | 25 to 29 | T1D | Female | 4.75 (4.26, 5.29） |
| Liberia | 25 to 29 | T1D | Male | 4.72 (4.24, 5.25） |
| Liberia | 30 to 34 | T1D | Both | 5.31 (4.90, 5.74） |
| Liberia | 30 to 34 | T1D | Female | 5.33 (4.76, 5.97） |
| Liberia | 30 to 34 | T1D | Male | 5.29 (4.73, 5.91） |
| Liberia | 35 to 39 | T1D | Both | 5.75 (5.29, 6.25） |
| Liberia | 35 to 39 | T1D | Female | 5.78 (5.14, 6.51） |
| Liberia | 35 to 39 | T1D | Male | 5.71 (5.08, 6.41） |
| Liberia | 40 to 44 | T1D | Both | 6.12 (5.61, 6.68） |
| Liberia | 40 to 44 | T1D | Female | 6.16 (5.44, 6.97） |
| Liberia | 40 to 44 | T1D | Male | 6.06 (5.37, 6.85） |
| Liberia | 45 to 49 | T1D | Both | 6.44 (5.87, 7.07） |
| Liberia | 45 to 49 | T1D | Female | 6.50 (5.69, 7.42） |
| Liberia | 45 to 49 | T1D | Male | 6.37 (5.59, 7.25） |
| Liberia | 5 to 9 | T1D | Both | 1.00 (1.00, 1.00） |
| Liberia | 5 to 9 | T1D | Female | 1.00 (1.00, 1.00） |
| Liberia | 5 to 9 | T1D | Male | 1.00 (1.00, 1.00） |
| Liberia | 50 to 54 | T1D | Both | 6.72 (6.08, 7.42） |

| | | | | |
|---|---|---|---|---|
| Liberia | 50 to 54 | T1D | Female | 6.79 (5.89, 7.83） |
| Liberia | 50 to 54 | T1D | Male | 6.61 (5.75, 7.59） |
| Liberia | 55 to 59 | T1D | Both | 6.94 (6.24, 7.72） |
| Liberia | 55 to 59 | T1D | Female | 7.03 (6.03, 8.20） |
| Liberia | 55 to 59 | T1D | Male | 6.83 (5.88, 7.92） |
| Liberia | 60 to 64 | T1D | Both | 7.11 (6.34, 7.98） |
| Liberia | 60 to 64 | T1D | Female | 7.23 (6.13, 8.54） |
| Liberia | 60 to 64 | T1D | Male | 6.98 (5.94, 8.20） |
| Liberia | 65 to 69 | T1D | Both | 7.25 (6.39, 8.23） |
| Liberia | 65 to 69 | T1D | Female | 7.40 (6.17, 8.88） |
| Liberia | 65 to 69 | T1D | Male | 7.10 (5.94, 8.47） |
| Libya | 0 to 4 | T1D | Both | 0.17 (0.15, 0.19） |
| Libya | 0 to 4 | T1D | Female | 0.18 (0.16, 0.21） |
| Libya | 0 to 4 | T1D | Male | 0.15 (0.13, 0.18） |
| Libya | 10 to 14 | T1D | Both | 2.36 (2.24, 2.48） |
| Libya | 10 to 14 | T1D | Female | 2.44 (2.27, 2.63） |
| Libya | 10 to 14 | T1D | Male | 2.27 (2.12, 2.44） |
| Libya | 15 to 19 | T1D | Both | 3.84 (3.65, 4.04） |
| Libya | 15 to 19 | T1D | Female | 4.04 (3.75, 4.35） |
| Libya | 15 to 19 | T1D | Male | 3.67 (3.42, 3.93） |
| Libya | 20 to 24 | T1D | Both | 5.28 (5.02, 5.56） |
| Libya | 20 to 24 | T1D | Female | 5.45 (5.06, 5.87） |
| Libya | 20 to 24 | T1D | Male | 5.12 (4.77, 5.50） |
| Libya | 25 to 29 | T1D | Both | 7.03 (6.67, 7.40） |
| Libya | 25 to 29 | T1D | Female | 7.11 (6.59, 7.67） |
| Libya | 25 to 29 | T1D | Male | 6.93 (6.45, 7.45） |
| Libya | 30 to 34 | T1D | Both | 8.93 (8.46, 9.42） |
| Libya | 30 to 34 | T1D | Female | 8.88 (8.21, 9.59） |
| Libya | 30 to 34 | T1D | Male | 8.96 (8.32, 9.64） |
| Libya | 35 to 39 | T1D | Both | 11.05 (10.45, 11.68） |
| Libya | 35 to 39 | T1D | Female | 10.81 (9.98, 11.72） |
| Libya | 35 to 39 | T1D | Male | 11.23 (10.40, 12.12） |
| Libya | 40 to 44 | T1D | Both | 13.48 (12.73, 14.28） |

| | | | | |
|---|---|---|---|---|
| Libya | 40 to 44 | T1D | Female | 13.04 (12.00, 14.17） |
| Libya | 40 to 44 | T1D | Male | 13.85 (12.79, 15.00） |
| Libya | 45 to 49 | T1D | Both | 16.30 (15.35, 17.31） |
| Libya | 45 to 49 | T1D | Female | 15.64 (14.33, 17.06） |
| Libya | 45 to 49 | T1D | Male | 16.87 (15.52, 18.33） |
| Libya | 5 to 9 | T1D | Both | 1.00 (1.00, 1.00） |
| Libya | 5 to 9 | T1D | Female | 1.00 (1.00, 1.00） |
| Libya | 5 to 9 | T1D | Male | 1.00 (1.00, 1.00） |
| Libya | 50 to 54 | T1D | Both | 19.52 (18.32, 20.80） |
| Libya | 50 to 54 | T1D | Female | 18.64 (17.00, 20.44） |
| Libya | 50 to 54 | T1D | Male | 20.32 (18.62, 22.18） |
| Libya | 55 to 59 | T1D | Both | 23.26 (21.75, 24.87） |
| Libya | 55 to 59 | T1D | Female | 22.15 (20.09, 24.43） |
| Libya | 55 to 59 | T1D | Male | 24.28 (22.13, 26.63） |
| Libya | 60 to 64 | T1D | Both | 27.66 (25.75, 29.71） |
| Libya | 60 to 64 | T1D | Female | 26.29 (23.69, 29.18） |
| Libya | 60 to 64 | T1D | Male | 28.95 (26.23, 31.94） |
| Libya | 65 to 69 | T1D | Both | 32.89 (30.47, 35.52） |
| Libya | 65 to 69 | T1D | Female | 31.31 (27.99, 35.02） |
| Libya | 65 to 69 | T1D | Male | 34.36 (30.93, 38.18） |
| Lithuania | 0 to 4 | T1D | Both | 0.19 (0.15, 0.23） |
| Lithuania | 0 to 4 | T1D | Female | 0.17 (0.13, 0.24） |
| Lithuania | 0 to 4 | T1D | Male | 0.20 (0.15, 0.26） |
| Lithuania | 10 to 14 | T1D | Both | 2.38 (2.15, 2.63） |
| Lithuania | 10 to 14 | T1D | Female | 2.41 (2.09, 2.78） |
| Lithuania | 10 to 14 | T1D | Male | 2.35 (2.05, 2.71） |
| Lithuania | 15 to 19 | T1D | Both | 3.74 (3.39, 4.12） |
| Lithuania | 15 to 19 | T1D | Female | 3.66 (3.19, 4.20） |
| Lithuania | 15 to 19 | T1D | Male | 3.81 (3.33, 4.37） |
| Lithuania | 20 to 24 | T1D | Both | 4.93 (4.47, 5.44） |
| Lithuania | 20 to 24 | T1D | Female | 4.67 (4.06, 5.36） |
| Lithuania | 20 to 24 | T1D | Male | 5.19 (4.53, 5.95） |
| Lithuania | 25 to 29 | T1D | Both | 6.26 (5.67, 6.91） |

| | | | | |
|---|---|---|---|---|
| Lithuania | 25 to 29 | T1D | Female | 5.72 (4.97, 6.59） |
| Lithuania | 25 to 29 | T1D | Male | 6.78 (5.91, 7.78） |
| Lithuania | 30 to 34 | T1D | Both | 7.67 (6.94, 8.47） |
| Lithuania | 30 to 34 | T1D | Female | 6.79 (5.88, 7.84） |
| Lithuania | 30 to 34 | T1D | Male | 8.57 (7.45, 9.85） |
| Lithuania | 35 to 39 | T1D | Both | 9.14 (8.25, 10.12） |
| Lithuania | 35 to 39 | T1D | Female | 7.92 (6.84, 9.16） |
| Lithuania | 35 to 39 | T1D | Male | 10.41 (9.03, 12.00） |
| Lithuania | 40 to 44 | T1D | Both | 10.65 (9.61, 11.81） |
| Lithuania | 40 to 44 | T1D | Female | 9.14 (7.88, 10.59） |
| Lithuania | 40 to 44 | T1D | Male | 12.29 (10.65, 14.19） |
| Lithuania | 45 to 49 | T1D | Both | 12.25 (11.03, 13.59） |
| Lithuania | 45 to 49 | T1D | Female | 10.45 (8.99, 12.13） |
| Lithuania | 45 to 49 | T1D | Male | 14.23 (12.30, 16.46） |
| Lithuania | 5 to 9 | T1D | Both | 1.00 (1.00, 1.00） |
| Lithuania | 5 to 9 | T1D | Female | 1.00 (1.00, 1.00） |
| Lithuania | 5 to 9 | T1D | Male | 1.00 (1.00, 1.00） |
| Lithuania | 50 to 54 | T1D | Both | 13.93 (12.53, 15.48） |
| Lithuania | 50 to 54 | T1D | Female | 11.88 (10.21, 13.83） |
| Lithuania | 50 to 54 | T1D | Male | 16.27 (14.03, 18.86） |
| Lithuania | 55 to 59 | T1D | Both | 15.75 (14.15, 17.54） |
| Lithuania | 55 to 59 | T1D | Female | 13.49 (11.56, 15.74） |
| Lithuania | 55 to 59 | T1D | Male | 18.47 (15.89, 21.46） |
| Lithuania | 60 to 64 | T1D | Both | 17.78 (15.93, 19.84） |
| Lithuania | 60 to 64 | T1D | Female | 15.34 (13.11, 17.94） |
| Lithuania | 60 to 64 | T1D | Male | 20.91 (17.94, 24.39） |
| Lithuania | 65 to 69 | T1D | Both | 20.01 (17.89, 22.38） |
| Lithuania | 65 to 69 | T1D | Female | 17.41 (14.85, 20.42） |
| Lithuania | 65 to 69 | T1D | Male | 23.60 (20.16, 27.63） |
| Low SDI | 0 to 4 | T1D | Both | 0.14 (0.14, 0.15） |
| Low SDI | 0 to 4 | T1D | Female | 0.14 (0.14, 0.15） |
| Low SDI | 0 to 4 | T1D | Male | 0.14 (0.14, 0.15） |
| Low SDI | 10 to 14 | T1D | Both | 2.16 (2.13, 2.18） |

| | | | | |
|---|---|---|---|---|
| Low SDI | 10 to 14 | T1D | Female | 2.14 (2.13, 2.16） |
| Low SDI | 10 to 14 | T1D | Male | 2.17 (2.14, 2.19） |
| Low SDI | 15 to 19 | T1D | Both | 3.10 (3.07, 3.13） |
| Low SDI | 15 to 19 | T1D | Female | 3.05 (3.03, 3.08） |
| Low SDI | 15 to 19 | T1D | Male | 3.15 (3.11, 3.19） |
| Low SDI | 20 to 24 | T1D | Both | 3.88 (3.84, 3.92） |
| Low SDI | 20 to 24 | T1D | Female | 3.79 (3.76, 3.83） |
| Low SDI | 20 to 24 | T1D | Male | 3.98 (3.92, 4.03） |
| Low SDI | 25 to 29 | T1D | Both | 4.68 (4.63, 4.73） |
| Low SDI | 25 to 29 | T1D | Female | 4.55 (4.51, 4.59） |
| Low SDI | 25 to 29 | T1D | Male | 4.82 (4.76, 4.89） |
| Low SDI | 30 to 34 | T1D | Both | 5.24 (5.18, 5.30） |
| Low SDI | 30 to 34 | T1D | Female | 5.07 (5.03, 5.12） |
| Low SDI | 30 to 34 | T1D | Male | 5.41 (5.34, 5.49） |
| Low SDI | 35 to 39 | T1D | Both | 5.68 (5.61, 5.75） |
| Low SDI | 35 to 39 | T1D | Female | 5.49 (5.43, 5.54） |
| Low SDI | 35 to 39 | T1D | Male | 5.88 (5.79, 5.96） |
| Low SDI | 40 to 44 | T1D | Both | 6.07 (5.99, 6.14） |
| Low SDI | 40 to 44 | T1D | Female | 5.85 (5.79, 5.91） |
| Low SDI | 40 to 44 | T1D | Male | 6.28 (6.18, 6.37） |
| Low SDI | 45 to 49 | T1D | Both | 6.40 (6.32, 6.49） |
| Low SDI | 45 to 49 | T1D | Female | 6.18 (6.11, 6.24） |
| Low SDI | 45 to 49 | T1D | Male | 6.62 (6.51, 6.73） |
| Low SDI | 5 to 9 | T1D | Both | 1.00 (1.00, 1.00） |
| Low SDI | 5 to 9 | T1D | Female | 1.00 (1.00, 1.00） |
| Low SDI | 5 to 9 | T1D | Male | 1.00 (1.00, 1.00） |
| Low SDI | 50 to 54 | T1D | Both | 6.69 (6.59, 6.79） |
| Low SDI | 50 to 54 | T1D | Female | 6.47 (6.40, 6.54） |
| Low SDI | 50 to 54 | T1D | Male | 6.90 (6.78, 7.02） |
| Low SDI | 55 to 59 | T1D | Both | 6.94 (6.84, 7.05） |
| Low SDI | 55 to 59 | T1D | Female | 6.74 (6.66, 6.82） |
| Low SDI | 55 to 59 | T1D | Male | 7.15 (7.02, 7.29） |
| Low SDI | 60 to 64 | T1D | Both | 7.17 (7.05, 7.29） |

| | | | | |
|---|---|---|---|---|
| Low SDI | 60 to 64 | T1D | Female | 6.98 (6.89, 7.07） |
| Low SDI | 60 to 64 | T1D | Male | 7.37 (7.22, 7.52） |
| Low SDI | 65 to 69 | T1D | Both | 7.37 (7.23, 7.50） |
| Low SDI | 65 to 69 | T1D | Female | 7.20 (7.09, 7.30） |
| Low SDI | 65 to 69 | T1D | Male | 7.54 (7.37, 7.71） |
| Low-middle SDI | 0 to 4 | T1D | Both | 0.14 (0.14, 0.15） |
| Low-middle SDI | 0 to 4 | T1D | Female | 0.14 (0.14, 0.15） |
| Low-middle SDI | 0 to 4 | T1D | Male | 0.14 (0.14, 0.15） |
| Low-middle SDI | 10 to 14 | T1D | Both | 2.18 (2.14, 2.22） |
| Low-middle SDI | 10 to 14 | T1D | Female | 2.15 (2.13, 2.18） |
| Low-middle SDI | 10 to 14 | T1D | Male | 2.20 (2.15, 2.26） |
| Low-middle SDI | 15 to 19 | T1D | Both | 3.07 (3.02, 3.13） |
| Low-middle SDI | 15 to 19 | T1D | Female | 2.97 (2.93, 3.00） |
| Low-middle SDI | 15 to 19 | T1D | Male | 3.18 (3.10, 3.26） |
| Low-middle SDI | 20 to 24 | T1D | Both | 3.76 (3.69, 3.83） |
| Low-middle SDI | 20 to 24 | T1D | Female | 3.57 (3.52, 3.61） |
| Low-middle SDI | 20 to 24 | T1D | Male | 3.96 (3.87, 4.06） |
| Low-middle SDI | 25 to 29 | T1D | Both | 4.47 (4.39, 4.56） |
| Low-middle SDI | 25 to 29 | T1D | Female | 4.18 (4.13, 4.23） |
| Low-middle SDI | 25 to 29 | T1D | Male | 4.78 (4.66, 4.90） |
| Low-middle SDI | 30 to 34 | T1D | Both | 5.04 (4.95, 5.14） |
| Low-middle SDI | 30 to 34 | T1D | Female | 4.67 (4.61, 4.73） |
| Low-middle SDI | 30 to 34 | T1D | Male | 5.43 (5.29, 5.57） |
| Low-middle SDI | 35 to 39 | T1D | Both | 5.56 (5.45, 5.68） |
| Low-middle SDI | 35 to 39 | T1D | Female | 5.13 (5.06, 5.20） |
| Low-middle SDI | 35 to 39 | T1D | Male | 6.02 (5.86, 6.19） |
| Low-middle SDI | 40 to 44 | T1D | Both | 6.08 (5.95, 6.21） |
| Low-middle SDI | 40 to 44 | T1D | Female | 5.58 (5.50, 5.66） |
| Low-middle SDI | 40 to 44 | T1D | Male | 6.59 (6.41, 6.78） |
| Low-middle SDI | 45 to 49 | T1D | Both | 6.56 (6.41, 6.70） |
| Low-middle SDI | 45 to 49 | T1D | Female | 6.01 (5.92, 6.10） |
| Low-middle SDI | 45 to 49 | T1D | Male | 7.12 (6.91, 7.33） |
| Low-middle SDI | 5 to 9 | T1D | Both | 1.00 (1.00, 1.00） |

| | | | | |
|---|---|---|---|---|
| Low-middle SDI | 5 to 9 | T1D | Female | 1.00 (1.00, 1.00） |
| Low-middle SDI | 5 to 9 | T1D | Male | 1.00 (1.00, 1.00） |
| Low-middle SDI | 50 to 54 | T1D | Both | 7.00 (6.84, 7.17） |
| Low-middle SDI | 50 to 54 | T1D | Female | 6.44 (6.34, 6.54） |
| Low-middle SDI | 50 to 54 | T1D | Male | 7.59 (7.36, 7.83） |
| Low-middle SDI | 55 to 59 | T1D | Both | 7.47 (7.29, 7.66） |
| Low-middle SDI | 55 to 59 | T1D | Female | 6.91 (6.80, 7.03） |
| Low-middle SDI | 55 to 59 | T1D | Male | 8.08 (7.82, 8.35） |
| Low-middle SDI | 60 to 64 | T1D | Both | 7.96 (7.76, 8.17） |
| Low-middle SDI | 60 to 64 | T1D | Female | 7.41 (7.28, 7.54） |
| Low-middle SDI | 60 to 64 | T1D | Male | 8.57 (8.28, 8.88） |
| Low-middle SDI | 65 to 69 | T1D | Both | 8.42 (8.19, 8.67） |
| Low-middle SDI | 65 to 69 | T1D | Female | 7.89 (7.74, 8.04） |
| Low-middle SDI | 65 to 69 | T1D | Male | 9.04 (8.70, 9.38） |
| Luxembourg | 0 to 4 | T1D | Both | 0.19 (0.14, 0.27） |
| Luxembourg | 0 to 4 | T1D | Female | 0.17 (0.10, 0.28） |
| Luxembourg | 0 to 4 | T1D | Male | 0.20 (0.13, 0.32） |
| Luxembourg | 10 to 14 | T1D | Both | 2.15 (1.81, 2.55） |
| Luxembourg | 10 to 14 | T1D | Female | 2.13 (1.66, 2.72） |
| Luxembourg | 10 to 14 | T1D | Male | 2.10 (1.66, 2.66） |
| Luxembourg | 15 to 19 | T1D | Both | 3.26 (2.75, 3.87） |
| Luxembourg | 15 to 19 | T1D | Female | 3.12 (2.44, 3.99） |
| Luxembourg | 15 to 19 | T1D | Male | 3.29 (2.60, 4.17） |
| Luxembourg | 20 to 24 | T1D | Both | 4.29 (3.61, 5.11） |
| Luxembourg | 20 to 24 | T1D | Female | 3.99 (3.11, 5.13） |
| Luxembourg | 20 to 24 | T1D | Male | 4.45 (3.51, 5.65） |
| Luxembourg | 25 to 29 | T1D | Both | 5.44 (4.57, 6.49） |
| Luxembourg | 25 to 29 | T1D | Female | 4.92 (3.82, 6.34） |
| Luxembourg | 25 to 29 | T1D | Male | 5.80 (4.56, 7.38） |
| Luxembourg | 30 to 34 | T1D | Both | 6.61 (5.53, 7.90） |
| Luxembourg | 30 to 34 | T1D | Female | 5.81 (4.49, 7.52） |
| Luxembourg | 30 to 34 | T1D | Male | 7.16 (5.60, 9.14） |
| Luxembourg | 35 to 39 | T1D | Both | 7.85 (6.55, 9.42） |

| | | | | |
|---|---|---|---|---|
| Luxembourg | 35 to 39 | T1D | Female | 6.79 (5.21, 8.83） |
| Luxembourg | 35 to 39 | T1D | Male | 8.63 (6.73, 11.07） |
| Luxembourg | 40 to 44 | T1D | Both | 9.25 (7.69, 11.13） |
| Luxembourg | 40 to 44 | T1D | Female | 7.87 (6.02, 10.29） |
| Luxembourg | 40 to 44 | T1D | Male | 10.26 (7.97, 13.21） |
| Luxembourg | 45 to 49 | T1D | Both | 10.79 (8.94, 13.01） |
| Luxembourg | 45 to 49 | T1D | Female | 9.06 (6.89, 11.91） |
| Luxembourg | 45 to 49 | T1D | Male | 12.05 (9.32, 15.58） |
| Luxembourg | 5 to 9 | T1D | Both | 1.00 (1.00, 1.00） |
| Luxembourg | 5 to 9 | T1D | Female | 1.00 (1.00, 1.00） |
| Luxembourg | 5 to 9 | T1D | Male | 1.00 (1.00, 1.00） |
| Luxembourg | 50 to 54 | T1D | Both | 12.45 (10.28, 15.08） |
| Luxembourg | 50 to 54 | T1D | Female | 10.39 (7.86, 13.74） |
| Luxembourg | 50 to 54 | T1D | Male | 14.01 (10.79, 18.19） |
| Luxembourg | 55 to 59 | T1D | Both | 14.28 (11.74, 17.36） |
| Luxembourg | 55 to 59 | T1D | Female | 11.82 (8.88, 15.74） |
| Luxembourg | 55 to 59 | T1D | Male | 16.17 (12.39, 21.11） |
| Luxembourg | 60 to 64 | T1D | Both | 16.24 (13.29, 19.84） |
| Luxembourg | 60 to 64 | T1D | Female | 13.44 (10.03, 18.02） |
| Luxembourg | 60 to 64 | T1D | Male | 18.52 (14.10, 24.32） |
| Luxembourg | 65 to 69 | T1D | Both | 18.45 (15.03, 22.65） |
| Luxembourg | 65 to 69 | T1D | Female | 15.21 (11.26, 20.55） |
| Luxembourg | 65 to 69 | T1D | Male | 21.21 (16.05, 28.04） |
| Madagascar | 0 to 4 | T1D | Both | 0.16 (0.15, 0.17） |
| Madagascar | 0 to 4 | T1D | Female | 0.16 (0.14, 0.17） |
| Madagascar | 0 to 4 | T1D | Male | 0.16 (0.14, 0.17） |
| Madagascar | 10 to 14 | T1D | Both | 2.04 (1.98, 2.10） |
| Madagascar | 10 to 14 | T1D | Female | 2.04 (1.96, 2.13） |
| Madagascar | 10 to 14 | T1D | Male | 2.04 (1.96, 2.13） |
| Madagascar | 15 to 19 | T1D | Both | 3.00 (2.91, 3.09） |
| Madagascar | 15 to 19 | T1D | Female | 3.00 (2.88, 3.13） |
| Madagascar | 15 to 19 | T1D | Male | 3.00 (2.88, 3.12） |
| Madagascar | 20 to 24 | T1D | Both | 3.89 (3.78, 4.01） |

| | | | | |
|---|---|---|---|---|
| Madagascar | 20 to 24 | T1D | Female | 3.90 (3.74, 4.07） |
| Madagascar | 20 to 24 | T1D | Male | 3.89 (3.73, 4.05） |
| Madagascar | 25 to 29 | T1D | Both | 4.80 (4.65, 4.95） |
| Madagascar | 25 to 29 | T1D | Female | 4.82 (4.61, 5.04） |
| Madagascar | 25 to 29 | T1D | Male | 4.78 (4.58, 4.99） |
| Madagascar | 30 to 34 | T1D | Both | 5.38 (5.21, 5.56） |
| Madagascar | 30 to 34 | T1D | Female | 5.42 (5.18, 5.68） |
| Madagascar | 30 to 34 | T1D | Male | 5.35 (5.11, 5.60） |
| Madagascar | 35 to 39 | T1D | Both | 5.82 (5.63, 6.02） |
| Madagascar | 35 to 39 | T1D | Female | 5.88 (5.60, 6.17） |
| Madagascar | 35 to 39 | T1D | Male | 5.77 (5.50, 6.05） |
| Madagascar | 40 to 44 | T1D | Both | 6.18 (5.97, 6.41） |
| Madagascar | 40 to 44 | T1D | Female | 6.26 (5.95, 6.59） |
| Madagascar | 40 to 44 | T1D | Male | 6.11 (5.81, 6.42） |
| Madagascar | 45 to 49 | T1D | Both | 6.48 (6.24, 6.73） |
| Madagascar | 45 to 49 | T1D | Female | 6.59 (6.24, 6.95） |
| Madagascar | 45 to 49 | T1D | Male | 6.38 (6.06, 6.73） |
| Madagascar | 5 to 9 | T1D | Both | 1.00 (1.00, 1.00） |
| Madagascar | 5 to 9 | T1D | Female | 1.00 (1.00, 1.00） |
| Madagascar | 5 to 9 | T1D | Male | 1.00 (1.00, 1.00） |
| Madagascar | 50 to 54 | T1D | Both | 6.72 (6.45, 6.99） |
| Madagascar | 50 to 54 | T1D | Female | 6.85 (6.46, 7.25） |
| Madagascar | 50 to 54 | T1D | Male | 6.60 (6.24, 6.98） |
| Madagascar | 55 to 59 | T1D | Both | 6.91 (6.62, 7.21） |
| Madagascar | 55 to 59 | T1D | Female | 7.07 (6.64, 7.52） |
| Madagascar | 55 to 59 | T1D | Male | 6.77 (6.37, 7.19） |
| Madagascar | 60 to 64 | T1D | Both | 7.06 (6.74, 7.41） |
| Madagascar | 60 to 64 | T1D | Female | 7.24 (6.77, 7.75） |
| Madagascar | 60 to 64 | T1D | Male | 6.90 (6.45, 7.37） |
| Madagascar | 65 to 69 | T1D | Both | 7.18 (6.81, 7.58） |
| Madagascar | 65 to 69 | T1D | Female | 7.39 (6.85, 7.97） |
| Madagascar | 65 to 69 | T1D | Male | 6.99 (6.48, 7.54） |
| Malawi | 0 to 4 | T1D | Both | 0.15 (0.14, 0.16） |

| | | | | |
|---|---|---|---|---|
| Malawi | 0 to 4 | T1D | Female | 0.15 (0.14, 0.17） |
| Malawi | 0 to 4 | T1D | Male | 0.15 (0.14, 0.17） |
| Malawi | 10 to 14 | T1D | Both | 2.05 (1.98, 2.12） |
| Malawi | 10 to 14 | T1D | Female | 2.05 (1.96, 2.16） |
| Malawi | 10 to 14 | T1D | Male | 2.05 (1.95, 2.15） |
| Malawi | 15 to 19 | T1D | Both | 3.00 (2.90, 3.11） |
| Malawi | 15 to 19 | T1D | Female | 3.01 (2.86, 3.16） |
| Malawi | 15 to 19 | T1D | Male | 2.99 (2.85, 3.14） |
| Malawi | 20 to 24 | T1D | Both | 3.87 (3.74, 4.02） |
| Malawi | 20 to 24 | T1D | Female | 3.89 (3.70, 4.10） |
| Malawi | 20 to 24 | T1D | Male | 3.87 (3.67, 4.07） |
| Malawi | 25 to 29 | T1D | Both | 4.76 (4.59, 4.94） |
| Malawi | 25 to 29 | T1D | Female | 4.78 (4.54, 5.04） |
| Malawi | 25 to 29 | T1D | Male | 4.74 (4.50, 5.00） |
| Malawi | 30 to 34 | T1D | Both | 5.32 (5.12, 5.53） |
| Malawi | 30 to 34 | T1D | Female | 5.36 (5.07, 5.66） |
| Malawi | 30 to 34 | T1D | Male | 5.29 (5.01, 5.59） |
| Malawi | 35 to 39 | T1D | Both | 5.74 (5.51, 5.98） |
| Malawi | 35 to 39 | T1D | Female | 5.79 (5.46, 6.13） |
| Malawi | 35 to 39 | T1D | Male | 5.69 (5.38, 6.03） |
| Malawi | 40 to 44 | T1D | Both | 6.08 (5.82, 6.35） |
| Malawi | 40 to 44 | T1D | Female | 6.15 (5.78, 6.54） |
| Malawi | 40 to 44 | T1D | Male | 6.02 (5.66, 6.40） |
| Malawi | 45 to 49 | T1D | Both | 6.36 (6.07, 6.66） |
| Malawi | 45 to 49 | T1D | Female | 6.44 (6.03, 6.88） |
| Malawi | 45 to 49 | T1D | Male | 6.28 (5.89, 6.71） |
| Malawi | 5 to 9 | T1D | Both | 1.00 (1.00, 1.00） |
| Malawi | 5 to 9 | T1D | Female | 1.00 (1.00, 1.00） |
| Malawi | 5 to 9 | T1D | Male | 1.00 (1.00, 1.00） |
| Malawi | 50 to 54 | T1D | Both | 6.58 (6.26, 6.92） |
| Malawi | 50 to 54 | T1D | Female | 6.69 (6.23, 7.17） |
| Malawi | 50 to 54 | T1D | Male | 6.49 (6.05, 6.96） |
| Malawi | 55 to 59 | T1D | Both | 6.77 (6.42, 7.14） |

| | | | | |
|---|---|---|---|---|
| Malawi | 55 to 59 | T1D | Female | 6.89 (6.40, 7.43） |
| Malawi | 55 to 59 | T1D | Male | 6.66 (6.18, 7.18） |
| Malawi | 60 to 64 | T1D | Both | 6.92 (6.54, 7.33） |
| Malawi | 60 to 64 | T1D | Female | 7.07 (6.52, 7.66） |
| Malawi | 60 to 64 | T1D | Male | 6.80 (6.27, 7.37） |
| Malawi | 65 to 69 | T1D | Both | 7.04 (6.61, 7.49） |
| Malawi | 65 to 69 | T1D | Female | 7.20 (6.60, 7.87） |
| Malawi | 65 to 69 | T1D | Male | 6.90 (6.30, 7.55） |
| Malaysia | 0 to 4 | T1D | Both | 0.15 (0.14, 0.17） |
| Malaysia | 0 to 4 | T1D | Female | 0.15 (0.13, 0.17） |
| Malaysia | 0 to 4 | T1D | Male | 0.15 (0.13, 0.17） |
| Malaysia | 10 to 14 | T1D | Both | 2.05 (1.97, 2.14） |
| Malaysia | 10 to 14 | T1D | Female | 2.03 (1.91, 2.15） |
| Malaysia | 10 to 14 | T1D | Male | 2.08 (1.96, 2.21） |
| Malaysia | 15 to 19 | T1D | Both | 2.93 (2.81, 3.06） |
| Malaysia | 15 to 19 | T1D | Female | 2.84 (2.67, 3.01） |
| Malaysia | 15 to 19 | T1D | Male | 3.02 (2.85, 3.21） |
| Malaysia | 20 to 24 | T1D | Both | 3.69 (3.54, 3.85） |
| Malaysia | 20 to 24 | T1D | Female | 3.49 (3.29, 3.71） |
| Malaysia | 20 to 24 | T1D | Male | 3.89 (3.66, 4.13） |
| Malaysia | 25 to 29 | T1D | Both | 4.48 (4.29, 4.68） |
| Malaysia | 25 to 29 | T1D | Female | 4.15 (3.89, 4.41） |
| Malaysia | 25 to 29 | T1D | Male | 4.81 (4.52, 5.12） |
| Malaysia | 30 to 34 | T1D | Both | 5.11 (4.88, 5.35） |
| Malaysia | 30 to 34 | T1D | Female | 4.66 (4.36, 4.97） |
| Malaysia | 30 to 34 | T1D | Male | 5.56 (5.22, 5.93） |
| Malaysia | 35 to 39 | T1D | Both | 5.68 (5.42, 5.96） |
| Malaysia | 35 to 39 | T1D | Female | 5.12 (4.79, 5.48） |
| Malaysia | 35 to 39 | T1D | Male | 6.24 (5.84, 6.67） |
| Malaysia | 40 to 44 | T1D | Both | 6.25 (5.96, 6.57） |
| Malaysia | 40 to 44 | T1D | Female | 5.59 (5.22, 6.00） |
| Malaysia | 40 to 44 | T1D | Male | 6.92 (6.46, 7.40） |
| Malaysia | 45 to 49 | T1D | Both | 6.81 (6.48, 7.17） |

| | | | | |
|---|---|---|---|---|
| Malaysia | 45 to 49 | T1D | Female | 6.06 (5.64, 6.52） |
| Malaysia | 45 to 49 | T1D | Male | 7.57 (7.05, 8.12） |
| Malaysia | 5 to 9 | T1D | Both | 1.00 (1.00, 1.00） |
| Malaysia | 5 to 9 | T1D | Female | 1.00 (1.00, 1.00） |
| Malaysia | 5 to 9 | T1D | Male | 1.00 (1.00, 1.00） |
| Malaysia | 50 to 54 | T1D | Both | 7.37 (6.99, 7.77） |
| Malaysia | 50 to 54 | T1D | Female | 6.55 (6.07, 7.06） |
| Malaysia | 50 to 54 | T1D | Male | 8.20 (7.62, 8.82） |
| Malaysia | 55 to 59 | T1D | Both | 7.93 (7.50, 8.38） |
| Malaysia | 55 to 59 | T1D | Female | 7.05 (6.52, 7.63） |
| Malaysia | 55 to 59 | T1D | Male | 8.83 (8.17, 9.53） |
| Malaysia | 60 to 64 | T1D | Both | 8.51 (8.03, 9.02） |
| Malaysia | 60 to 64 | T1D | Female | 7.58 (6.98, 8.24） |
| Malaysia | 60 to 64 | T1D | Male | 9.47 (8.73, 10.27） |
| Malaysia | 65 to 69 | T1D | Both | 9.12 (8.57, 9.71） |
| Malaysia | 65 to 69 | T1D | Female | 8.15 (7.46, 8.92） |
| Malaysia | 65 to 69 | T1D | Male | 10.12 (9.28, 11.05） |
| Maldives | 0 to 4 | T1D | Both | 0.12 (0.05, 0.30） |
| Maldives | 0 to 4 | T1D | Female | 0.24 (0.09, 0.62） |
| Maldives | 0 to 4 | T1D | Male | 0.25 (0.10, 0.67） |
| Maldives | 10 to 14 | T1D | Both | 2.09 (1.43, 3.04） |
| Maldives | 10 to 14 | T1D | Female | 2.08 (1.22, 3.54） |
| Maldives | 10 to 14 | T1D | Male | 2.15 (1.25, 3.69） |
| Maldives | 15 to 19 | T1D | Both | 2.95 (2.03, 4.29） |
| Maldives | 15 to 19 | T1D | Female | 2.91 (1.71, 4.95） |
| Maldives | 15 to 19 | T1D | Male | 3.15 (1.85, 5.36） |
| Maldives | 20 to 24 | T1D | Both | 3.61 (2.48, 5.27） |
| Maldives | 20 to 24 | T1D | Female | 3.54 (2.07, 6.05） |
| Maldives | 20 to 24 | T1D | Male | 3.85 (2.26, 6.54） |
| Maldives | 25 to 29 | T1D | Both | 4.26 (2.90, 6.25） |
| Maldives | 25 to 29 | T1D | Female | 4.04 (2.33, 7.02） |
| Maldives | 25 to 29 | T1D | Male | 4.63 (2.71, 7.92） |
| Maldives | 30 to 34 | T1D | Both | 4.74 (3.19, 7.06） |

| | | | | |
|---|---|---|---|---|
| Maldives | 30 to 34 | T1D | Female | 4.47 (2.52, 7.92） |
| Maldives | 30 to 34 | T1D | Male | 5.28 (3.03, 9.19） |
| Maldives | 35 to 39 | T1D | Both | 5.20 (3.42, 7.90） |
| Maldives | 35 to 39 | T1D | Female | 4.72 (2.58, 8.64） |
| Maldives | 35 to 39 | T1D | Male | 5.67 (3.16, 10.18） |
| Maldives | 40 to 44 | T1D | Both | 5.69 (3.66, 8.83） |
| Maldives | 40 to 44 | T1D | Female | 5.30 (2.81, 10.01） |
| Maldives | 40 to 44 | T1D | Male | 6.15 (3.33, 11.36） |
| Maldives | 45 to 49 | T1D | Both | 5.98 (3.76, 9.51） |
| Maldives | 45 to 49 | T1D | Female | 5.54 (2.83, 10.84） |
| Maldives | 45 to 49 | T1D | Male | 6.72 (3.53, 12.79） |
| Maldives | 5 to 9 | T1D | Both | 1.00 (1.00, 1.00） |
| Maldives | 5 to 9 | T1D | Female | 1.00 (1.00, 1.00） |
| Maldives | 5 to 9 | T1D | Male | 1.00 (1.00, 1.00） |
| Maldives | 50 to 54 | T1D | Both | 6.41 (3.91, 10.51） |
| Maldives | 50 to 54 | T1D | Female | 5.69 (2.77, 11.70） |
| Maldives | 50 to 54 | T1D | Male | 6.96 (3.51, 13.83） |
| Maldives | 55 to 59 | T1D | Both | 6.73 (3.95, 11.48） |
| Maldives | 55 to 59 | T1D | Female | 6.09 (2.81, 13.20） |
| Maldives | 55 to 59 | T1D | Male | 7.22 (3.45, 15.14） |
| Maldives | 60 to 64 | T1D | Both | 7.12 (3.99, 12.68） |
| Maldives | 60 to 64 | T1D | Female | 6.44 (2.78, 14.92） |
| Maldives | 60 to 64 | T1D | Male | 8.06 (3.64, 17.88） |
| Maldives | 65 to 69 | T1D | Both | 7.68 (4.08, 14.46） |
| Maldives | 65 to 69 | T1D | Female | 7.04 (2.78, 17.81） |
| Maldives | 65 to 69 | T1D | Male | 7.88 (3.27, 18.96） |
| Mali | 0 to 4 | T1D | Both | 0.15 (0.14, 0.16） |
| Mali | 0 to 4 | T1D | Female | 0.15 (0.14, 0.17） |
| Mali | 0 to 4 | T1D | Male | 0.15 (0.14, 0.17） |
| Mali | 10 to 14 | T1D | Both | 2.10 (2.03, 2.17） |
| Mali | 10 to 14 | T1D | Female | 2.10 (2.00, 2.20） |
| Mali | 10 to 14 | T1D | Male | 2.10 (2.00, 2.20） |
| Mali | 15 to 19 | T1D | Both | 3.03 (2.92, 3.14） |

| | | | | |
|---|---|---|---|---|
| Mali | 15 to 19 | T1D | Female | 3.04 (2.89, 3.19） |
| Mali | 15 to 19 | T1D | Male | 3.03 (2.88, 3.18） |
| Mali | 20 to 24 | T1D | Both | 3.81 (3.67, 3.95） |
| Mali | 20 to 24 | T1D | Female | 3.83 (3.64, 4.04） |
| Mali | 20 to 24 | T1D | Male | 3.80 (3.61, 3.99） |
| Mali | 25 to 29 | T1D | Both | 4.59 (4.42, 4.76） |
| Mali | 25 to 29 | T1D | Female | 4.63 (4.38, 4.88） |
| Mali | 25 to 29 | T1D | Male | 4.57 (4.33, 4.82） |
| Mali | 30 to 34 | T1D | Both | 5.05 (4.86, 5.26） |
| Mali | 30 to 34 | T1D | Female | 5.10 (4.82, 5.40） |
| Mali | 30 to 34 | T1D | Male | 5.02 (4.75, 5.31） |
| Mali | 35 to 39 | T1D | Both | 5.37 (5.15, 5.60） |
| Mali | 35 to 39 | T1D | Female | 5.42 (5.11, 5.76） |
| Mali | 35 to 39 | T1D | Male | 5.32 (5.02, 5.65） |
| Mali | 40 to 44 | T1D | Both | 5.60 (5.36, 5.85） |
| Mali | 40 to 44 | T1D | Female | 5.66 (5.32, 6.03） |
| Mali | 40 to 44 | T1D | Male | 5.55 (5.21, 5.90） |
| Mali | 45 to 49 | T1D | Both | 5.78 (5.51, 6.05） |
| Mali | 45 to 49 | T1D | Female | 5.84 (5.47, 6.25） |
| Mali | 45 to 49 | T1D | Male | 5.71 (5.34, 6.10） |
| Mali | 5 to 9 | T1D | Both | 1.00 (1.00, 1.00） |
| Mali | 5 to 9 | T1D | Female | 1.00 (1.00, 1.00） |
| Mali | 5 to 9 | T1D | Male | 1.00 (1.00, 1.00） |
| Mali | 50 to 54 | T1D | Both | 5.89 (5.61, 6.19） |
| Mali | 50 to 54 | T1D | Female | 5.97 (5.56, 6.41） |
| Mali | 50 to 54 | T1D | Male | 5.82 (5.42, 6.24） |
| Mali | 55 to 59 | T1D | Both | 5.96 (5.65, 6.28） |
| Mali | 55 to 59 | T1D | Female | 6.05 (5.60, 6.52） |
| Mali | 55 to 59 | T1D | Male | 5.87 (5.45, 6.32） |
| Mali | 60 to 64 | T1D | Both | 5.98 (5.65, 6.33） |
| Mali | 60 to 64 | T1D | Female | 6.08 (5.60, 6.60） |
| Mali | 60 to 64 | T1D | Male | 5.88 (5.42, 6.37） |
| Mali | 65 to 69 | T1D | Both | 5.97 (5.60, 6.36） |

| | | | | |
|---|---|---|---|---|
| Mali | 65 to 69 | T1D | Female | 6.08 (5.55, 6.65） |
| Mali | 65 to 69 | T1D | Male | 5.86 (5.36, 6.40） |
| Malta | 0 to 4 | T1D | Both | 0.21 (0.15, 0.29） |
| Malta | 0 to 4 | T1D | Female | 0.20 (0.13, 0.32） |
| Malta | 0 to 4 | T1D | Male | 0.21 (0.13, 0.33） |
| Malta | 10 to 14 | T1D | Both | 1.89 (1.61, 2.22） |
| Malta | 10 to 14 | T1D | Female | 1.91 (1.53, 2.39） |
| Malta | 10 to 14 | T1D | Male | 1.90 (1.51, 2.38） |
| Malta | 15 to 19 | T1D | Both | 2.87 (2.45, 3.36） |
| Malta | 15 to 19 | T1D | Female | 2.90 (2.32, 3.62） |
| Malta | 15 to 19 | T1D | Male | 2.87 (2.29, 3.60） |
| Malta | 20 to 24 | T1D | Both | 3.92 (3.33, 4.60） |
| Malta | 20 to 24 | T1D | Female | 3.97 (3.17, 4.98） |
| Malta | 20 to 24 | T1D | Male | 3.88 (3.08, 4.88） |
| Malta | 25 to 29 | T1D | Both | 5.03 (4.27, 5.92） |
| Malta | 25 to 29 | T1D | Female | 5.11 (4.06, 6.44） |
| Malta | 25 to 29 | T1D | Male | 4.99 (3.95, 6.30） |
| Malta | 30 to 34 | T1D | Both | 6.16 (5.21, 7.28） |
| Malta | 30 to 34 | T1D | Female | 6.30 (4.98, 7.97） |
| Malta | 30 to 34 | T1D | Male | 6.07 (4.78, 7.70） |
| Malta | 35 to 39 | T1D | Both | 7.38 (6.22, 8.75） |
| Malta | 35 to 39 | T1D | Female | 7.59 (5.97, 9.66） |
| Malta | 35 to 39 | T1D | Male | 7.22 (5.66, 9.21） |
| Malta | 40 to 44 | T1D | Both | 8.68 (7.30, 10.32） |
| Malta | 40 to 44 | T1D | Female | 9.00 (7.05, 11.51） |
| Malta | 40 to 44 | T1D | Male | 8.43 (6.59, 10.79） |
| Malta | 45 to 49 | T1D | Both | 10.08 (8.45, 12.03） |
| Malta | 45 to 49 | T1D | Female | 10.56 (8.23, 13.55） |
| Malta | 45 to 49 | T1D | Male | 9.68 (7.53, 12.44） |
| Malta | 5 to 9 | T1D | Both | 1.00 (1.00, 1.00） |
| Malta | 5 to 9 | T1D | Female | 1.00 (1.00, 1.00） |
| Malta | 5 to 9 | T1D | Male | 1.00 (1.00, 1.00） |
| Malta | 50 to 54 | T1D | Both | 11.59 (9.69, 13.86） |

| | | | | |
|---|---|---|---|---|
| Malta | 50 to 54 | T1D | Female | 12.29 (9.55, 15.83） |
| Malta | 50 to 54 | T1D | Male | 10.97 (8.50, 14.15） |
| Malta | 55 to 59 | T1D | Both | 13.18 (11.00, 15.81） |
| Malta | 55 to 59 | T1D | Female | 14.17 (10.97, 18.31） |
| Malta | 55 to 59 | T1D | Male | 12.34 (9.53, 15.97） |
| Malta | 60 to 64 | T1D | Both | 14.93 (12.41, 17.96） |
| Malta | 60 to 64 | T1D | Female | 16.22 (12.50, 21.05） |
| Malta | 60 to 64 | T1D | Male | 13.81 (10.61, 17.96） |
| Malta | 65 to 69 | T1D | Both | 16.83 (13.93, 20.33） |
| Malta | 65 to 69 | T1D | Female | 18.49 (14.17, 24.12） |
| Malta | 65 to 69 | T1D | Male | 15.40 (11.76, 20.16） |
| Marshall Islands | 0 to 4 | T1D | Both | 0.05 (0.01, 0.82） |
| Marshall Islands | 0 to 4 | T1D | Female | 0.10 (0.01, 1.73） |
| Marshall Islands | 0 to 4 | T1D | Male | 0.10 (0.01, 1.77） |
| Marshall Islands | 10 to 14 | T1D | Both | 1.66 (0.76, 3.61） |
| Marshall Islands | 10 to 14 | T1D | Female | 2.21 (0.78, 6.29） |
| Marshall Islands | 10 to 14 | T1D | Male | 1.79 (0.60, 5.33） |
| Marshall Islands | 15 to 19 | T1D | Both | 2.51 (1.15, 5.46） |
| Marshall Islands | 15 to 19 | T1D | Female | 2.56 (0.86, 7.61） |
| Marshall Islands | 15 to 19 | T1D | Male | 2.55 (0.86, 7.57） |
| Marshall Islands | 20 to 24 | T1D | Both | 3.17 (1.42, 7.09） |
| Marshall Islands | 20 to 24 | T1D | Female | 2.81 (0.90, 8.72） |
| Marshall Islands | 20 to 24 | T1D | Male | 2.97 (0.96, 9.19） |
| Marshall Islands | 25 to 29 | T1D | Both | 3.92 (1.72, 8.95） |
| Marshall Islands | 25 to 29 | T1D | Female | 3.30 (1.02, 10.72） |
| Marshall Islands | 25 to 29 | T1D | Male | 4.13 (1.34, 12.75） |
| Marshall Islands | 30 to 34 | T1D | Both | 4.38 (1.85, 10.37） |
| Marshall Islands | 30 to 34 | T1D | Female | 3.67 (1.07, 12.64） |
| Marshall Islands | 30 to 34 | T1D | Male | 4.27 (1.32, 13.89） |
| Marshall Islands | 35 to 39 | T1D | Both | 4.76 (1.92, 11.82） |
| Marshall Islands | 35 to 39 | T1D | Female | 4.01 (1.08, 14.85） |
| Marshall Islands | 35 to 39 | T1D | Male | 4.50 (1.30, 15.54） |
| Marshall Islands | 40 to 44 | T1D | Both | 5.11 (1.96, 13.28） |

| | | | | |
|---|---|---|---|---|
| Marshall Islands | 40 to 44 | T1D | Female | 4.94 (1.26, 19.39） |
| Marshall Islands | 40 to 44 | T1D | Male | 4.76 (1.29, 17.55） |
| Marshall Islands | 45 to 49 | T1D | Both | 5.84 (2.14, 15.94） |
| Marshall Islands | 45 to 49 | T1D | Female | 5.72 (1.34, 24.45） |
| Marshall Islands | 45 to 49 | T1D | Male | 5.16 (1.32, 20.22） |
| Marshall Islands | 5 to 9 | T1D | Both | 1.00 (1.00, 1.00） |
| Marshall Islands | 5 to 9 | T1D | Female | 1.00 (1.00, 1.00） |
| Marshall Islands | 5 to 9 | T1D | Male | 1.00 (1.00, 1.00） |
| Marshall Islands | 50 to 54 | T1D | Both | 5.85 (2.02, 16.99） |
| Marshall Islands | 50 to 54 | T1D | Female | 6.50 (1.37, 30.74） |
| Marshall Islands | 50 to 54 | T1D | Male | 5.87 (1.39, 24.71） |
| Marshall Islands | 55 to 59 | T1D | Both | 5.87 (1.86, 18.53） |
| Marshall Islands | 55 to 59 | T1D | Female | 6.85 (1.26, 37.37） |
| Marshall Islands | 55 to 59 | T1D | Male | 6.75 (1.45, 31.40） |
| Marshall Islands | 60 to 64 | T1D | Both | 6.49 (1.86, 22.69） |
| Marshall Islands | 60 to 64 | T1D | Female | 8.08 (1.29, 50.44） |
| Marshall Islands | 60 to 64 | T1D | Male | 7.16 (1.23, 41.80） |
| Marshall Islands | 65 to 69 | T1D | Both | 7.29 (1.74, 30.57） |
| Marshall Islands | 65 to 69 | T1D | Female | 8.49 (0.81, 88.64） |
| Marshall Islands | 65 to 69 | T1D | Male | 8.59 (1.20, 61.55） |
| Mauritania | 0 to 4 | T1D | Both | 0.17 (0.14, 0.19） |
| Mauritania | 0 to 4 | T1D | Female | 0.17 (0.14, 0.21） |
| Mauritania | 0 to 4 | T1D | Male | 0.17 (0.14, 0.21） |
| Mauritania | 10 to 14 | T1D | Both | 2.05 (1.91, 2.20） |
| Mauritania | 10 to 14 | T1D | Female | 2.05 (1.85, 2.28） |
| Mauritania | 10 to 14 | T1D | Male | 2.05 (1.86, 2.27） |
| Mauritania | 15 to 19 | T1D | Both | 3.01 (2.80, 3.24） |
| Mauritania | 15 to 19 | T1D | Female | 3.02 (2.72, 3.35） |
| Mauritania | 15 to 19 | T1D | Male | 3.02 (2.73, 3.34） |
| Mauritania | 20 to 24 | T1D | Both | 3.86 (3.58, 4.16） |
| Mauritania | 20 to 24 | T1D | Female | 3.89 (3.49, 4.33） |
| Mauritania | 20 to 24 | T1D | Male | 3.86 (3.48, 4.29） |
| Mauritania | 25 to 29 | T1D | Both | 4.76 (4.40, 5.14） |

| | | | | |
|---|---|---|---|---|
| Mauritania | 25 to 29 | T1D | Female | 4.80 (4.29, 5.37） |
| Mauritania | 25 to 29 | T1D | Male | 4.75 (4.26, 5.29） |
| Mauritania | 30 to 34 | T1D | Both | 5.36 (4.94, 5.81） |
| Mauritania | 30 to 34 | T1D | Female | 5.41 (4.82, 6.08） |
| Mauritania | 30 to 34 | T1D | Male | 5.35 (4.77, 6.00） |
| Mauritania | 35 to 39 | T1D | Both | 5.84 (5.36, 6.36） |
| Mauritania | 35 to 39 | T1D | Female | 5.91 (5.23, 6.67） |
| Mauritania | 35 to 39 | T1D | Male | 5.81 (5.16, 6.55） |
| Mauritania | 40 to 44 | T1D | Both | 6.26 (5.73, 6.84） |
| Mauritania | 40 to 44 | T1D | Female | 6.34 (5.58, 7.20） |
| Mauritania | 40 to 44 | T1D | Male | 6.22 (5.49, 7.05） |
| Mauritania | 45 to 49 | T1D | Both | 6.63 (6.04, 7.28） |
| Mauritania | 45 to 49 | T1D | Female | 6.73 (5.89, 7.69） |
| Mauritania | 45 to 49 | T1D | Male | 6.58 (5.77, 7.50） |
| Mauritania | 5 to 9 | T1D | Both | 1.00 (1.00, 1.00） |
| Mauritania | 5 to 9 | T1D | Female | 1.00 (1.00, 1.00） |
| Mauritania | 5 to 9 | T1D | Male | 1.00 (1.00, 1.00） |
| Mauritania | 50 to 54 | T1D | Both | 6.96 (6.30, 7.69） |
| Mauritania | 50 to 54 | T1D | Female | 7.08 (6.15, 8.16） |
| Mauritania | 50 to 54 | T1D | Male | 6.89 (5.99, 7.92） |
| Mauritania | 55 to 59 | T1D | Both | 7.25 (6.52, 8.05） |
| Mauritania | 55 to 59 | T1D | Female | 7.40 (6.36, 8.60） |
| Mauritania | 55 to 59 | T1D | Male | 7.15 (6.16, 8.29） |
| Mauritania | 60 to 64 | T1D | Both | 7.49 (6.68, 8.39） |
| Mauritania | 60 to 64 | T1D | Female | 7.65 (6.50, 9.00） |
| Mauritania | 60 to 64 | T1D | Male | 7.37 (6.28, 8.64） |
| Mauritania | 65 to 69 | T1D | Both | 7.72 (6.82, 8.74） |
| Mauritania | 65 to 69 | T1D | Female | 7.88 (6.60, 9.42） |
| Mauritania | 65 to 69 | T1D | Male | 7.57 (6.37, 9.01） |
| Mauritius | 0 to 4 | T1D | Both | 0.14 (0.08, 0.24） |
| Mauritius | 0 to 4 | T1D | Female | 0.16 (0.08, 0.32） |
| Mauritius | 0 to 4 | T1D | Male | 0.17 (0.08, 0.34） |
| Mauritius | 10 to 14 | T1D | Both | 2.12 (1.70, 2.64） |

| | | | | |
|---|---|---|---|---|
| Mauritius | 10 to 14 | T1D | Female | 2.01 (1.48, 2.73） |
| Mauritius | 10 to 14 | T1D | Male | 2.10 (1.53, 2.88） |
| Mauritius | 15 to 19 | T1D | Both | 2.99 (2.40, 3.72） |
| Mauritius | 15 to 19 | T1D | Female | 2.78 (2.06, 3.76） |
| Mauritius | 15 to 19 | T1D | Male | 3.03 (2.22, 4.14） |
| Mauritius | 20 to 24 | T1D | Both | 3.70 (2.97, 4.62） |
| Mauritius | 20 to 24 | T1D | Female | 3.34 (2.46, 4.53） |
| Mauritius | 20 to 24 | T1D | Male | 3.87 (2.82, 5.30） |
| Mauritius | 25 to 29 | T1D | Both | 4.46 (3.56, 5.58） |
| Mauritius | 25 to 29 | T1D | Female | 3.95 (2.89, 5.40） |
| Mauritius | 25 to 29 | T1D | Male | 4.72 (3.43, 6.50） |
| Mauritius | 30 to 34 | T1D | Both | 5.10 (4.06, 6.42） |
| Mauritius | 30 to 34 | T1D | Female | 4.45 (3.23, 6.12） |
| Mauritius | 30 to 34 | T1D | Male | 5.50 (3.97, 7.61） |
| Mauritius | 35 to 39 | T1D | Both | 5.72 (4.52, 7.23） |
| Mauritius | 35 to 39 | T1D | Female | 4.92 (3.55, 6.82） |
| Mauritius | 35 to 39 | T1D | Male | 6.24 (4.47, 8.71） |
| Mauritius | 40 to 44 | T1D | Both | 6.36 (5.00, 8.08） |
| Mauritius | 40 to 44 | T1D | Female | 5.44 (3.89, 7.60） |
| Mauritius | 40 to 44 | T1D | Male | 6.97 (4.96, 9.79） |
| Mauritius | 45 to 49 | T1D | Both | 7.04 (5.51, 8.99） |
| Mauritius | 45 to 49 | T1D | Female | 5.95 (4.23, 8.38） |
| Mauritius | 45 to 49 | T1D | Male | 7.78 (5.50, 11.00） |
| Mauritius | 5 to 9 | T1D | Both | 1.00 (1.00, 1.00） |
| Mauritius | 5 to 9 | T1D | Female | 1.00 (1.00, 1.00） |
| Mauritius | 5 to 9 | T1D | Male | 1.00 (1.00, 1.00） |
| Mauritius | 50 to 54 | T1D | Both | 7.70 (5.99, 9.90） |
| Mauritius | 50 to 54 | T1D | Female | 6.48 (4.56, 9.21） |
| Mauritius | 50 to 54 | T1D | Male | 8.55 (5.99, 12.19） |
| Mauritius | 55 to 59 | T1D | Both | 8.40 (6.48, 10.88） |
| Mauritius | 55 to 59 | T1D | Female | 7.11 (4.94, 10.21） |
| Mauritius | 55 to 59 | T1D | Male | 9.42 (6.53, 13.61） |
| Mauritius | 60 to 64 | T1D | Both | 9.16 (6.99, 11.99） |

| | | | | |
|---|---|---|---|---|
| Mauritius | 60 to 64 | T1D | Female | 7.68 (5.26, 11.20） |
| Mauritius | 60 to 64 | T1D | Male | 10.26 (6.99, 15.05） |
| Mauritius | 65 to 69 | T1D | Both | 10.01 (7.52, 13.31） |
| Mauritius | 65 to 69 | T1D | Female | 8.28 (5.56, 12.34） |
| Mauritius | 65 to 69 | T1D | Male | 11.17 (7.43, 16.79） |
| Mexico | 0 to 4 | T1D | Both | 0.12 (0.11, 0.12） |
| Mexico | 0 to 4 | T1D | Female | 0.11 (0.11, 0.12） |
| Mexico | 0 to 4 | T1D | Male | 0.12 (0.12, 0.13） |
| Mexico | 10 to 14 | T1D | Both | 2.34 (2.30, 2.39） |
| Mexico | 10 to 14 | T1D | Female | 2.43 (2.38, 2.48） |
| Mexico | 10 to 14 | T1D | Male | 2.24 (2.18, 2.29） |
| Mexico | 15 to 19 | T1D | Both | 3.25 (3.19, 3.31） |
| Mexico | 15 to 19 | T1D | Female | 3.35 (3.27, 3.42） |
| Mexico | 15 to 19 | T1D | Male | 3.12 (3.04, 3.19） |
| Mexico | 20 to 24 | T1D | Both | 3.86 (3.79, 3.94） |
| Mexico | 20 to 24 | T1D | Female | 3.91 (3.82, 4.00） |
| Mexico | 20 to 24 | T1D | Male | 3.77 (3.68, 3.86） |
| Mexico | 25 to 29 | T1D | Both | 4.44 (4.36, 4.53） |
| Mexico | 25 to 29 | T1D | Female | 4.42 (4.32, 4.52） |
| Mexico | 25 to 29 | T1D | Male | 4.43 (4.32, 4.54） |
| Mexico | 30 to 34 | T1D | Both | 4.89 (4.80, 4.99） |
| Mexico | 30 to 34 | T1D | Female | 4.81 (4.70, 4.93） |
| Mexico | 30 to 34 | T1D | Male | 4.95 (4.82, 5.09） |
| Mexico | 35 to 39 | T1D | Both | 5.30 (5.19, 5.41） |
| Mexico | 35 to 39 | T1D | Female | 5.17 (5.04, 5.30） |
| Mexico | 35 to 39 | T1D | Male | 5.42 (5.28, 5.57） |
| Mexico | 40 to 44 | T1D | Both | 5.70 (5.57, 5.82） |
| Mexico | 40 to 44 | T1D | Female | 5.52 (5.38, 5.66） |
| Mexico | 40 to 44 | T1D | Male | 5.87 (5.71, 6.04） |
| Mexico | 45 to 49 | T1D | Both | 6.07 (5.94, 6.21） |
| Mexico | 45 to 49 | T1D | Female | 5.86 (5.70, 6.01） |
| Mexico | 45 to 49 | T1D | Male | 6.30 (6.12, 6.49） |
| Mexico | 5 to 9 | T1D | Both | 1.00 (1.00, 1.00） |

| | | | | |
|---|---|---|---|---|
| Mexico | 5 to 9 | T1D | Female | 1.00 (1.00, 1.00） |
| Mexico | 5 to 9 | T1D | Male | 1.00 (1.00, 1.00） |
| Mexico | 50 to 54 | T1D | Both | 6.44 (6.28, 6.59） |
| Mexico | 50 to 54 | T1D | Female | 6.18 (6.01, 6.36） |
| Mexico | 50 to 54 | T1D | Male | 6.70 (6.49, 6.91） |
| Mexico | 55 to 59 | T1D | Both | 6.78 (6.61, 6.95） |
| Mexico | 55 to 59 | T1D | Female | 6.49 (6.31, 6.69） |
| Mexico | 55 to 59 | T1D | Male | 7.06 (6.83, 7.30） |
| Mexico | 60 to 64 | T1D | Both | 7.10 (6.91, 7.29） |
| Mexico | 60 to 64 | T1D | Female | 6.79 (6.58, 7.01） |
| Mexico | 60 to 64 | T1D | Male | 7.40 (7.14, 7.67） |
| Mexico | 65 to 69 | T1D | Both | 7.42 (7.21, 7.64） |
| Mexico | 65 to 69 | T1D | Female | 7.09 (6.86, 7.34） |
| Mexico | 65 to 69 | T1D | Male | 7.73 (7.43, 8.03） |
| Micronesia (Federated States of) | 0 to 4 | T1D | Both | 0.27 (0.10, 0.71） |
| Micronesia (Federated States of) | 0 to 4 | T1D | Female | 0.06 (0.01, 0.95） |
| Micronesia (Federated States of) | 0 to 4 | T1D | Male | 0.06 (0.01, 0.96） |
| Micronesia (Federated States of) | 10 to 14 | T1D | Both | 1.85 (1.07, 3.18） |
| Micronesia (Federated States of) | 10 to 14 | T1D | Female | 1.77 (0.82, 3.80） |
| Micronesia (Federated States of) | 10 to 14 | T1D | Male | 1.78 (0.83, 3.83） |
| Micronesia (Federated States of) | 15 to 19 | T1D | Both | 2.68 (1.56, 4.62） |
| Micronesia (Federated States of) | 15 to 19 | T1D | Female | 2.45 (1.13, 5.32） |
| Micronesia (Federated States of) | 15 to 19 | T1D | Male | 2.61 (1.21, 5.64） |
| Micronesia (Federated States of) | 20 to 24 | T1D | Both | 3.37 (1.91, 5.93） |
| Micronesia (Federated States of) | 20 to 24 | T1D | Female | 3.10 (1.38, 6.95） |
| Micronesia (Federated States of) | 20 to 24 | T1D | Male | 3.42 (1.54, 7.62） |
| Micronesia (Federated States of) | 25 to 29 | T1D | Both | 4.05 (2.25, 7.28） |
| Micronesia (Federated States of) | 25 to 29 | T1D | Female | 3.85 (1.66, 8.90） |
| Micronesia (Federated States of) | 25 to 29 | T1D | Male | 4.51 (1.99, 10.25） |
| Micronesia (Federated States of) | 30 to 34 | T1D | Both | 4.67 (2.53, 8.61） |
| Micronesia (Federated States of) | 30 to 34 | T1D | Female | 4.44 (1.85, 10.63） |
| Micronesia (Federated States of) | 30 to 34 | T1D | Male | 5.06 (2.15, 11.92） |
| Micronesia (Federated States of) | 35 to 39 | T1D | Both | 5.19 (2.74, 9.84） |

| | | | | |
|---|---|---|---|---|
| Micronesia (Federated States of) | 35 to 39 | T1D | Female | 4.55 (1.79, 11.53） |
| Micronesia (Federated States of) | 35 to 39 | T1D | Male | 5.73 (2.34, 14.06） |
| Micronesia (Federated States of) | 40 to 44 | T1D | Both | 5.65 (2.91, 10.97） |
| Micronesia (Federated States of) | 40 to 44 | T1D | Female | 5.27 (2.02, 13.70） |
| Micronesia (Federated States of) | 40 to 44 | T1D | Male | 6.05 (2.39, 15.29） |
| Micronesia (Federated States of) | 45 to 49 | T1D | Both | 5.86 (2.93, 11.73） |
| Micronesia (Federated States of) | 45 to 49 | T1D | Female | 5.71 (2.09, 15.60） |
| Micronesia (Federated States of) | 45 to 49 | T1D | Male | 6.28 (2.39, 16.51） |
| Micronesia (Federated States of) | 5 to 9 | T1D | Both | 1.00 (1.00, 1.00） |
| Micronesia (Federated States of) | 5 to 9 | T1D | Female | 1.00 (1.00, 1.00） |
| Micronesia (Federated States of) | 5 to 9 | T1D | Male | 1.00 (1.00, 1.00） |
| Micronesia (Federated States of) | 50 to 54 | T1D | Both | 6.71 (3.24, 13.88） |
| Micronesia (Federated States of) | 50 to 54 | T1D | Female | 6.42 (2.22, 18.60） |
| Micronesia (Federated States of) | 50 to 54 | T1D | Male | 6.98 (2.54, 19.21） |
| Micronesia (Federated States of) | 55 to 59 | T1D | Both | 7.04 (3.27, 15.16） |
| Micronesia (Federated States of) | 55 to 59 | T1D | Female | 6.89 (2.24, 21.15） |
| Micronesia (Federated States of) | 55 to 59 | T1D | Male | 7.36 (2.53, 21.39） |
| Micronesia (Federated States of) | 60 to 64 | T1D | Both | 6.92 (3.00, 15.95） |
| Micronesia (Federated States of) | 60 to 64 | T1D | Female | 6.17 (1.76, 21.59） |
| Micronesia (Federated States of) | 60 to 64 | T1D | Male | 7.60 (2.38, 24.20） |
| Micronesia (Federated States of) | 65 to 69 | T1D | Both | 7.50 (2.89, 19.46） |
| Micronesia (Federated States of) | 65 to 69 | T1D | Female | 7.44 (1.78, 31.11） |
| Micronesia (Federated States of) | 65 to 69 | T1D | Male | 8.17 (2.05, 32.63） |
| Middle SDI | 0 to 4 | T1D | Both | 0.14 (0.14, 0.15） |
| Middle SDI | 0 to 4 | T1D | Female | 0.14 (0.14, 0.15） |
| Middle SDI | 0 to 4 | T1D | Male | 0.14 (0.14, 0.15） |
| Middle SDI | 10 to 14 | T1D | Both | 2.21 (2.18, 2.24） |
| Middle SDI | 10 to 14 | T1D | Female | 2.22 (2.19, 2.24） |
| Middle SDI | 10 to 14 | T1D | Male | 2.21 (2.17, 2.25） |
| Middle SDI | 15 to 19 | T1D | Both | 3.26 (3.22, 3.31） |
| Middle SDI | 15 to 19 | T1D | Female | 3.22 (3.18, 3.25） |
| Middle SDI | 15 to 19 | T1D | Male | 3.31 (3.25, 3.37） |
| Middle SDI | 20 to 24 | T1D | Both | 4.21 (4.15, 4.27） |

| | | | | |
|---|---|---|---|---|
| Middle SDI | 20 to 24 | T1D | Female | 4.06 (4.01, 4.11） |
| Middle SDI | 20 to 24 | T1D | Male | 4.36 (4.28, 4.44） |
| Middle SDI | 25 to 29 | T1D | Both | 5.21 (5.13, 5.29） |
| Middle SDI | 25 to 29 | T1D | Female | 4.93 (4.87, 4.99） |
| Middle SDI | 25 to 29 | T1D | Male | 5.50 (5.39, 5.60） |
| Middle SDI | 30 to 34 | T1D | Both | 6.08 (5.99, 6.17） |
| Middle SDI | 30 to 34 | T1D | Female | 5.67 (5.60, 5.74） |
| Middle SDI | 30 to 34 | T1D | Male | 6.49 (6.37, 6.62） |
| Middle SDI | 35 to 39 | T1D | Both | 6.93 (6.83, 7.04） |
| Middle SDI | 35 to 39 | T1D | Female | 6.40 (6.32, 6.48） |
| Middle SDI | 35 to 39 | T1D | Male | 7.48 (7.33, 7.63） |
| Middle SDI | 40 to 44 | T1D | Both | 7.82 (7.70, 7.95） |
| Middle SDI | 40 to 44 | T1D | Female | 7.15 (7.06, 7.25） |
| Middle SDI | 40 to 44 | T1D | Male | 8.51 (8.34, 8.69） |
| Middle SDI | 45 to 49 | T1D | Both | 8.70 (8.56, 8.85） |
| Middle SDI | 45 to 49 | T1D | Female | 7.92 (7.81, 8.03） |
| Middle SDI | 45 to 49 | T1D | Male | 9.52 (9.31, 9.72） |
| Middle SDI | 5 to 9 | T1D | Both | 1.00 (1.00, 1.00） |
| Middle SDI | 5 to 9 | T1D | Female | 1.00 (1.00, 1.00） |
| Middle SDI | 5 to 9 | T1D | Male | 1.00 (1.00, 1.00） |
| Middle SDI | 50 to 54 | T1D | Both | 9.57 (9.41, 9.73） |
| Middle SDI | 50 to 54 | T1D | Female | 8.70 (8.58, 8.82） |
| Middle SDI | 50 to 54 | T1D | Male | 10.49 (10.26, 10.72） |
| Middle SDI | 55 to 59 | T1D | Both | 10.52 (10.34, 10.71） |
| Middle SDI | 55 to 59 | T1D | Female | 9.57 (9.43, 9.72） |
| Middle SDI | 55 to 59 | T1D | Male | 11.53 (11.26, 11.80） |
| Middle SDI | 60 to 64 | T1D | Both | 11.51 (11.30, 11.72） |
| Middle SDI | 60 to 64 | T1D | Female | 10.52 (10.36, 10.69） |
| Middle SDI | 60 to 64 | T1D | Male | 12.58 (12.28, 12.89） |
| Middle SDI | 65 to 69 | T1D | Both | 12.51 (12.26, 12.76） |
| Middle SDI | 65 to 69 | T1D | Female | 11.47 (11.28, 11.66） |
| Middle SDI | 65 to 69 | T1D | Male | 13.67 (13.32, 14.03） |
| Monaco | 0 to 4 | T1D | Both | 0.29 (0.07, 1.22） |

| | | | | |
|---|---|---|---|---|
| Monaco | 0 to 4 | T1D | Female | 0.11 (0.01, 1.91） |
| Monaco | 0 to 4 | T1D | Male | 0.10 (0.01, 1.87） |
| Monaco | 10 to 14 | T1D | Both | 2.13 (1.03, 4.42） |
| Monaco | 10 to 14 | T1D | Female | 2.18 (0.78, 6.11） |
| Monaco | 10 to 14 | T1D | Male | 2.17 (0.78, 6.08） |
| Monaco | 15 to 19 | T1D | Both | 3.40 (1.62, 7.11） |
| Monaco | 15 to 19 | T1D | Female | 3.09 (1.11, 8.59） |
| Monaco | 15 to 19 | T1D | Male | 3.41 (1.24, 9.34） |
| Monaco | 20 to 24 | T1D | Both | 4.65 (2.20, 9.81） |
| Monaco | 20 to 24 | T1D | Female | 4.24 (1.52, 11.84） |
| Monaco | 20 to 24 | T1D | Male | 4.58 (1.65, 12.72） |
| Monaco | 25 to 29 | T1D | Both | 5.86 (2.75, 12.49） |
| Monaco | 25 to 29 | T1D | Female | 5.19 (1.84, 14.64） |
| Monaco | 25 to 29 | T1D | Male | 6.33 (2.27, 17.67） |
| Monaco | 30 to 34 | T1D | Both | 7.10 (3.29, 15.30） |
| Monaco | 30 to 34 | T1D | Female | 6.33 (2.22, 18.09） |
| Monaco | 30 to 34 | T1D | Male | 7.53 (2.65, 21.42） |
| Monaco | 35 to 39 | T1D | Both | 8.32 (3.83, 18.08） |
| Monaco | 35 to 39 | T1D | Female | 7.37 (2.54, 21.38） |
| Monaco | 35 to 39 | T1D | Male | 9.26 (3.22, 26.67） |
| Monaco | 40 to 44 | T1D | Both | 9.90 (4.53, 21.64） |
| Monaco | 40 to 44 | T1D | Female | 8.68 (2.97, 25.42） |
| Monaco | 40 to 44 | T1D | Male | 10.72 (3.70, 31.10） |
| Monaco | 45 to 49 | T1D | Both | 11.23 (5.10, 24.75） |
| Monaco | 45 to 49 | T1D | Female | 9.70 (3.27, 28.77） |
| Monaco | 45 to 49 | T1D | Male | 12.34 (4.21, 36.17） |
| Monaco | 5 to 9 | T1D | Both | 1.00 (1.00, 1.00） |
| Monaco | 5 to 9 | T1D | Female | 1.00 (1.00, 1.00） |
| Monaco | 5 to 9 | T1D | Male | 1.00 (1.00, 1.00） |
| Monaco | 50 to 54 | T1D | Both | 12.95 (5.83, 28.76） |
| Monaco | 50 to 54 | T1D | Female | 11.34 (3.78, 34.02） |
| Monaco | 50 to 54 | T1D | Male | 14.45 (4.88, 42.76） |
| Monaco | 55 to 59 | T1D | Both | 14.66 (6.55, 32.83） |

| | | | | |
|---|---|---|---|---|
| Monaco | 55 to 59 | T1D | Female | 12.40 (4.08, 37.67） |
| Monaco | 55 to 59 | T1D | Male | 16.08 (5.37, 48.17） |
| Monaco | 60 to 64 | T1D | Both | 16.46 (7.30, 37.14） |
| Monaco | 60 to 64 | T1D | Female | 13.99 (4.55, 42.97） |
| Monaco | 60 to 64 | T1D | Male | 17.97 (5.94, 54.38） |
| Monaco | 65 to 69 | T1D | Both | 18.38 (8.08, 41.81） |
| Monaco | 65 to 69 | T1D | Female | 15.38 (4.95, 47.82） |
| Monaco | 65 to 69 | T1D | Male | 20.28 (6.61, 62.15） |
| Mongolia | 0 to 4 | T1D | Both | 0.16 (0.13, 0.19） |
| Mongolia | 0 to 4 | T1D | Female | 0.16 (0.12, 0.20） |
| Mongolia | 0 to 4 | T1D | Male | 0.16 (0.12, 0.20） |
| Mongolia | 10 to 14 | T1D | Both | 2.25 (2.08, 2.44） |
| Mongolia | 10 to 14 | T1D | Female | 2.25 (2.00, 2.52） |
| Mongolia | 10 to 14 | T1D | Male | 2.25 (2.01, 2.52） |
| Mongolia | 15 to 19 | T1D | Both | 3.48 (3.21, 3.77） |
| Mongolia | 15 to 19 | T1D | Female | 3.42 (3.05, 3.83） |
| Mongolia | 15 to 19 | T1D | Male | 3.54 (3.16, 3.95） |
| Mongolia | 20 to 24 | T1D | Both | 4.57 (4.22, 4.95） |
| Mongolia | 20 to 24 | T1D | Female | 4.40 (3.92, 4.93） |
| Mongolia | 20 to 24 | T1D | Male | 4.73 (4.23, 5.29） |
| Mongolia | 25 to 29 | T1D | Both | 5.74 (5.30, 6.23） |
| Mongolia | 25 to 29 | T1D | Female | 5.41 (4.82, 6.08） |
| Mongolia | 25 to 29 | T1D | Male | 6.07 (5.42, 6.79） |
| Mongolia | 30 to 34 | T1D | Both | 6.77 (6.23, 7.36） |
| Mongolia | 30 to 34 | T1D | Female | 6.29 (5.58, 7.09） |
| Mongolia | 30 to 34 | T1D | Male | 7.25 (6.46, 8.15） |
| Mongolia | 35 to 39 | T1D | Both | 7.72 (7.08, 8.41） |
| Mongolia | 35 to 39 | T1D | Female | 7.10 (6.28, 8.04） |
| Mongolia | 35 to 39 | T1D | Male | 8.34 (7.40, 9.41） |
| Mongolia | 40 to 44 | T1D | Both | 8.65 (7.91, 9.45） |
| Mongolia | 40 to 44 | T1D | Female | 7.90 (6.96, 8.98） |
| Mongolia | 40 to 44 | T1D | Male | 9.40 (8.31, 10.64） |
| Mongolia | 45 to 49 | T1D | Both | 9.59 (8.75, 10.52） |

| | | | | |
|---|---|---|---|---|
| Mongolia | 45 to 49 | T1D | Female | 8.75 (7.67, 9.99） |
| Mongolia | 45 to 49 | T1D | Male | 10.48 (9.22, 11.91） |
| Mongolia | 5 to 9 | T1D | Both | 1.00 (1.00, 1.00） |
| Mongolia | 5 to 9 | T1D | Female | 1.00 (1.00, 1.00） |
| Mongolia | 5 to 9 | T1D | Male | 1.00 (1.00, 1.00） |
| Mongolia | 50 to 54 | T1D | Both | 10.57 (9.60, 11.64） |
| Mongolia | 50 to 54 | T1D | Female | 9.65 (8.40, 11.08） |
| Mongolia | 50 to 54 | T1D | Male | 11.58 (10.12, 13.24） |
| Mongolia | 55 to 59 | T1D | Both | 11.53 (10.41, 12.77） |
| Mongolia | 55 to 59 | T1D | Female | 10.58 (9.13, 12.25） |
| Mongolia | 55 to 59 | T1D | Male | 12.64 (10.96, 14.58） |
| Mongolia | 60 to 64 | T1D | Both | 12.49 (11.18, 13.95） |
| Mongolia | 60 to 64 | T1D | Female | 11.52 (9.84, 13.50） |
| Mongolia | 60 to 64 | T1D | Male | 13.67 (11.71, 15.95） |
| Mongolia | 65 to 69 | T1D | Both | 13.47 (11.92, 15.22） |
| Mongolia | 65 to 69 | T1D | Female | 12.54 (10.54, 14.91） |
| Mongolia | 65 to 69 | T1D | Male | 14.74 (12.41, 17.51） |
| Montenegro | 0 to 4 | T1D | Both | 0.21 (0.15, 0.30） |
| Montenegro | 0 to 4 | T1D | Female | 0.22 (0.14, 0.35） |
| Montenegro | 0 to 4 | T1D | Male | 0.24 (0.15, 0.39） |
| Montenegro | 10 to 14 | T1D | Both | 2.17 (1.82, 2.58） |
| Montenegro | 10 to 14 | T1D | Female | 2.11 (1.65, 2.69） |
| Montenegro | 10 to 14 | T1D | Male | 2.28 (1.76, 2.94） |
| Montenegro | 15 to 19 | T1D | Both | 3.39 (2.85, 4.04） |
| Montenegro | 15 to 19 | T1D | Female | 3.13 (2.46, 3.98） |
| Montenegro | 15 to 19 | T1D | Male | 3.77 (2.93, 4.84） |
| Montenegro | 20 to 24 | T1D | Both | 4.45 (3.74, 5.31） |
| Montenegro | 20 to 24 | T1D | Female | 3.96 (3.10, 5.05） |
| Montenegro | 20 to 24 | T1D | Male | 5.09 (3.95, 6.55） |
| Montenegro | 25 to 29 | T1D | Both | 5.61 (4.70, 6.70） |
| Montenegro | 25 to 29 | T1D | Female | 4.88 (3.80, 6.27） |
| Montenegro | 25 to 29 | T1D | Male | 6.53 (5.05, 8.43） |
| Montenegro | 30 to 34 | T1D | Both | 6.75 (5.63, 8.09） |

| | | | | |
|---|---|---|---|---|
| Montenegro | 30 to 34 | T1D | Female | 5.80 (4.50, 7.49） |
| Montenegro | 30 to 34 | T1D | Male | 7.97 (6.14, 10.35） |
| Montenegro | 35 to 39 | T1D | Both | 7.97 (6.62, 9.59） |
| Montenegro | 35 to 39 | T1D | Female | 6.75 (5.20, 8.77） |
| Montenegro | 35 to 39 | T1D | Male | 9.54 (7.31, 12.44） |
| Montenegro | 40 to 44 | T1D | Both | 9.28 (7.69, 11.21） |
| Montenegro | 40 to 44 | T1D | Female | 7.78 (5.96, 10.14） |
| Montenegro | 40 to 44 | T1D | Male | 11.21 (8.56, 14.68） |
| Montenegro | 45 to 49 | T1D | Both | 10.69 (8.83, 12.95） |
| Montenegro | 45 to 49 | T1D | Female | 8.92 (6.80, 11.69） |
| Montenegro | 45 to 49 | T1D | Male | 12.99 (9.89, 17.07） |
| Montenegro | 5 to 9 | T1D | Both | 1.00 (1.00, 1.00） |
| Montenegro | 5 to 9 | T1D | Female | 1.00 (1.00, 1.00） |
| Montenegro | 5 to 9 | T1D | Male | 1.00 (1.00, 1.00） |
| Montenegro | 50 to 54 | T1D | Both | 12.20 (10.04, 14.82） |
| Montenegro | 50 to 54 | T1D | Female | 10.17 (7.72, 13.40） |
| Montenegro | 50 to 54 | T1D | Male | 14.85 (11.26, 19.60） |
| Montenegro | 55 to 59 | T1D | Both | 13.82 (11.34, 16.85） |
| Montenegro | 55 to 59 | T1D | Female | 11.55 (8.72, 15.30） |
| Montenegro | 55 to 59 | T1D | Male | 16.81 (12.67, 22.30） |
| Montenegro | 60 to 64 | T1D | Both | 15.56 (12.70, 19.05） |
| Montenegro | 60 to 64 | T1D | Female | 13.03 (9.77, 17.37） |
| Montenegro | 60 to 64 | T1D | Male | 18.90 (14.16, 25.23） |
| Montenegro | 65 to 69 | T1D | Both | 17.40 (14.13, 21.43） |
| Montenegro | 65 to 69 | T1D | Female | 14.69 (10.94, 19.73） |
| Montenegro | 65 to 69 | T1D | Male | 21.13 (15.70, 28.44） |
| Morocco | 0 to 4 | T1D | Both | 0.14 (0.13, 0.15） |
| Morocco | 0 to 4 | T1D | Female | 0.15 (0.14, 0.16） |
| Morocco | 0 to 4 | T1D | Male | 0.14 (0.13, 0.15） |
| Morocco | 10 to 14 | T1D | Both | 2.35 (2.30, 2.41） |
| Morocco | 10 to 14 | T1D | Female | 2.46 (2.38, 2.55） |
| Morocco | 10 to 14 | T1D | Male | 2.26 (2.20, 2.33） |
| Morocco | 15 to 19 | T1D | Both | 3.65 (3.57, 3.73） |

| | | | | |
|---|---|---|---|---|
| Morocco | 15 to 19 | T1D | Female | 3.87 (3.75, 4.00） |
| Morocco | 15 to 19 | T1D | Male | 3.46 (3.36, 3.57） |
| Morocco | 20 to 24 | T1D | Both | 4.80 (4.69, 4.91） |
| Morocco | 20 to 24 | T1D | Female | 5.05 (4.88, 5.22） |
| Morocco | 20 to 24 | T1D | Male | 4.60 (4.46, 4.75） |
| Morocco | 25 to 29 | T1D | Both | 6.08 (5.94, 6.22） |
| Morocco | 25 to 29 | T1D | Female | 6.31 (6.10, 6.53） |
| Morocco | 25 to 29 | T1D | Male | 5.90 (5.71, 6.09） |
| Morocco | 30 to 34 | T1D | Both | 7.32 (7.14, 7.50） |
| Morocco | 30 to 34 | T1D | Female | 7.53 (7.26, 7.80） |
| Morocco | 30 to 34 | T1D | Male | 7.17 (6.94, 7.41） |
| Morocco | 35 to 39 | T1D | Both | 8.58 (8.37, 8.80） |
| Morocco | 35 to 39 | T1D | Female | 8.75 (8.44, 9.08） |
| Morocco | 35 to 39 | T1D | Male | 8.47 (8.18, 8.76） |
| Morocco | 40 to 44 | T1D | Both | 9.90 (9.64, 10.16） |
| Morocco | 40 to 44 | T1D | Female | 10.04 (9.67, 10.43） |
| Morocco | 40 to 44 | T1D | Male | 9.81 (9.47, 10.17） |
| Morocco | 45 to 49 | T1D | Both | 11.29 (10.99, 11.59） |
| Morocco | 45 to 49 | T1D | Female | 11.41 (10.97, 11.87） |
| Morocco | 45 to 49 | T1D | Male | 11.21 (10.80, 11.63） |
| Morocco | 5 to 9 | T1D | Both | 1.00 (1.00, 1.00） |
| Morocco | 5 to 9 | T1D | Female | 1.00 (1.00, 1.00） |
| Morocco | 5 to 9 | T1D | Male | 1.00 (1.00, 1.00） |
| Morocco | 50 to 54 | T1D | Both | 12.74 (12.39, 13.10） |
| Morocco | 50 to 54 | T1D | Female | 12.87 (12.35, 13.41） |
| Morocco | 50 to 54 | T1D | Male | 12.65 (12.17, 13.14） |
| Morocco | 55 to 59 | T1D | Both | 14.26 (13.85, 14.68） |
| Morocco | 55 to 59 | T1D | Female | 14.43 (13.83, 15.06） |
| Morocco | 55 to 59 | T1D | Male | 14.14 (13.59, 14.72） |
| Morocco | 60 to 64 | T1D | Both | 15.87 (15.39, 16.37） |
| Morocco | 60 to 64 | T1D | Female | 16.11 (15.40, 16.85） |
| Morocco | 60 to 64 | T1D | Male | 15.70 (15.04, 16.38） |
| Morocco | 65 to 69 | T1D | Both | 17.59 (17.01, 18.18） |

| | | | | |
|---|---|---|---|---|
| Morocco | 65 to 69 | T1D | Female | 17.91 (17.06, 18.80） |
| Morocco | 65 to 69 | T1D | Male | 17.32 (16.54, 18.14） |
| Mozambique | 0 to 4 | T1D | Both | 0.15 (0.14, 0.16） |
| Mozambique | 0 to 4 | T1D | Female | 0.15 (0.14, 0.16） |
| Mozambique | 0 to 4 | T1D | Male | 0.15 (0.14, 0.16） |
| Mozambique | 10 to 14 | T1D | Both | 2.07 (2.01, 2.12） |
| Mozambique | 10 to 14 | T1D | Female | 2.06 (1.98, 2.15） |
| Mozambique | 10 to 14 | T1D | Male | 2.07 (1.99, 2.15） |
| Mozambique | 15 to 19 | T1D | Both | 3.01 (2.92, 3.09） |
| Mozambique | 15 to 19 | T1D | Female | 3.00 (2.89, 3.13） |
| Mozambique | 15 to 19 | T1D | Male | 3.01 (2.90, 3.14） |
| Mozambique | 20 to 24 | T1D | Both | 3.86 (3.74, 3.97） |
| Mozambique | 20 to 24 | T1D | Female | 3.86 (3.71, 4.03） |
| Mozambique | 20 to 24 | T1D | Male | 3.86 (3.71, 4.03） |
| Mozambique | 25 to 29 | T1D | Both | 4.69 (4.55, 4.83） |
| Mozambique | 25 to 29 | T1D | Female | 4.70 (4.50, 4.91） |
| Mozambique | 25 to 29 | T1D | Male | 4.69 (4.49, 4.90） |
| Mozambique | 30 to 34 | T1D | Both | 5.19 (5.03, 5.36） |
| Mozambique | 30 to 34 | T1D | Female | 5.21 (4.98, 5.45） |
| Mozambique | 30 to 34 | T1D | Male | 5.18 (4.95, 5.42） |
| Mozambique | 35 to 39 | T1D | Both | 5.53 (5.35, 5.72） |
| Mozambique | 35 to 39 | T1D | Female | 5.57 (5.31, 5.83） |
| Mozambique | 35 to 39 | T1D | Male | 5.51 (5.25, 5.77） |
| Mozambique | 40 to 44 | T1D | Both | 5.80 (5.60, 6.01） |
| Mozambique | 40 to 44 | T1D | Female | 5.85 (5.56, 6.14） |
| Mozambique | 40 to 44 | T1D | Male | 5.75 (5.47, 6.05） |
| Mozambique | 45 to 49 | T1D | Both | 6.00 (5.78, 6.23） |
| Mozambique | 45 to 49 | T1D | Female | 6.07 (5.75, 6.40） |
| Mozambique | 45 to 49 | T1D | Male | 5.94 (5.63, 6.26） |
| Mozambique | 5 to 9 | T1D | Both | 1.00 (1.00, 1.00） |
| Mozambique | 5 to 9 | T1D | Female | 1.00 (1.00, 1.00） |
| Mozambique | 5 to 9 | T1D | Male | 1.00 (1.00, 1.00） |
| Mozambique | 50 to 54 | T1D | Both | 6.15 (5.91, 6.40） |

| | | | | |
|---|---|---|---|---|
| Mozambique | 50 to 54 | T1D | Female | 6.23 (5.89, 6.59） |
| Mozambique | 50 to 54 | T1D | Male | 6.07 (5.73, 6.42） |
| Mozambique | 55 to 59 | T1D | Both | 6.26 (5.99, 6.53） |
| Mozambique | 55 to 59 | T1D | Female | 6.36 (5.98, 6.75） |
| Mozambique | 55 to 59 | T1D | Male | 6.16 (5.79, 6.55） |
| Mozambique | 60 to 64 | T1D | Both | 6.33 (6.04, 6.64） |
| Mozambique | 60 to 64 | T1D | Female | 6.45 (6.04, 6.88） |
| Mozambique | 60 to 64 | T1D | Male | 6.22 (5.82, 6.65） |
| Mozambique | 65 to 69 | T1D | Both | 6.37 (6.05, 6.71） |
| Mozambique | 65 to 69 | T1D | Female | 6.51 (6.05, 7.00） |
| Mozambique | 65 to 69 | T1D | Male | 6.24 (5.79, 6.73） |
| Myanmar | 0 to 4 | T1D | Both | 0.14 (0.13, 0.15） |
| Myanmar | 0 to 4 | T1D | Female | 0.14 (0.13, 0.16） |
| Myanmar | 0 to 4 | T1D | Male | 0.14 (0.13, 0.15） |
| Myanmar | 10 to 14 | T1D | Both | 2.11 (2.05, 2.18） |
| Myanmar | 10 to 14 | T1D | Female | 2.09 (2.01, 2.18） |
| Myanmar | 10 to 14 | T1D | Male | 2.14 (2.05, 2.23） |
| Myanmar | 15 to 19 | T1D | Both | 2.97 (2.88, 3.07） |
| Myanmar | 15 to 19 | T1D | Female | 2.88 (2.76, 3.01） |
| Myanmar | 15 to 19 | T1D | Male | 3.07 (2.94, 3.21） |
| Myanmar | 20 to 24 | T1D | Both | 3.66 (3.55, 3.78） |
| Myanmar | 20 to 24 | T1D | Female | 3.47 (3.32, 3.62） |
| Myanmar | 20 to 24 | T1D | Male | 3.88 (3.71, 4.05） |
| Myanmar | 25 to 29 | T1D | Both | 4.33 (4.19, 4.47） |
| Myanmar | 25 to 29 | T1D | Female | 4.01 (3.83, 4.19） |
| Myanmar | 25 to 29 | T1D | Male | 4.69 (4.48, 4.91） |
| Myanmar | 30 to 34 | T1D | Both | 4.82 (4.66, 4.98） |
| Myanmar | 30 to 34 | T1D | Female | 4.40 (4.20, 4.61） |
| Myanmar | 30 to 34 | T1D | Male | 5.29 (5.05, 5.55） |
| Myanmar | 35 to 39 | T1D | Both | 5.23 (5.06, 5.41） |
| Myanmar | 35 to 39 | T1D | Female | 4.74 (4.52, 4.97） |
| Myanmar | 35 to 39 | T1D | Male | 5.80 (5.52, 6.09） |
| Myanmar | 40 to 44 | T1D | Both | 5.61 (5.42, 5.81） |

| | | | | |
|---|---|---|---|---|
| Myanmar | 40 to 44 | T1D | Female | 5.06 (4.81, 5.31） |
| Myanmar | 40 to 44 | T1D | Male | 6.26 (5.96, 6.59） |
| Myanmar | 45 to 49 | T1D | Both | 5.96 (5.75, 6.19） |
| Myanmar | 45 to 49 | T1D | Female | 5.36 (5.09, 5.64） |
| Myanmar | 45 to 49 | T1D | Male | 6.68 (6.34, 7.04） |
| Myanmar | 5 to 9 | T1D | Both | 1.00 (1.00, 1.00） |
| Myanmar | 5 to 9 | T1D | Female | 1.00 (1.00, 1.00） |
| Myanmar | 5 to 9 | T1D | Male | 1.00 (1.00, 1.00） |
| Myanmar | 50 to 54 | T1D | Both | 6.29 (6.05, 6.54） |
| Myanmar | 50 to 54 | T1D | Female | 5.65 (5.36, 5.96） |
| Myanmar | 50 to 54 | T1D | Male | 7.06 (6.68, 7.46） |
| Myanmar | 55 to 59 | T1D | Both | 6.61 (6.34, 6.88） |
| Myanmar | 55 to 59 | T1D | Female | 5.95 (5.62, 6.29） |
| Myanmar | 55 to 59 | T1D | Male | 7.42 (7.00, 7.86） |
| Myanmar | 60 to 64 | T1D | Both | 6.91 (6.62, 7.22） |
| Myanmar | 60 to 64 | T1D | Female | 6.24 (5.88, 6.63） |
| Myanmar | 60 to 64 | T1D | Male | 7.75 (7.29, 8.25） |
| Myanmar | 65 to 69 | T1D | Both | 7.21 (6.88, 7.56） |
| Myanmar | 65 to 69 | T1D | Female | 6.54 (6.12, 6.98） |
| Myanmar | 65 to 69 | T1D | Male | 8.06 (7.54, 8.63） |
| Namibia | 0 to 4 | T1D | Both | 0.17 (0.14, 0.20） |
| Namibia | 0 to 4 | T1D | Female | 0.17 (0.13, 0.22） |
| Namibia | 0 to 4 | T1D | Male | 0.17 (0.13, 0.22） |
| Namibia | 10 to 14 | T1D | Both | 2.04 (1.87, 2.23） |
| Namibia | 10 to 14 | T1D | Female | 2.04 (1.80, 2.31） |
| Namibia | 10 to 14 | T1D | Male | 2.04 (1.80, 2.31） |
| Namibia | 15 to 19 | T1D | Both | 2.97 (2.72, 3.24） |
| Namibia | 15 to 19 | T1D | Female | 2.97 (2.62, 3.37） |
| Namibia | 15 to 19 | T1D | Male | 2.97 (2.62, 3.36） |
| Namibia | 20 to 24 | T1D | Both | 3.76 (3.44, 4.12） |
| Namibia | 20 to 24 | T1D | Female | 3.77 (3.31, 4.29） |
| Namibia | 20 to 24 | T1D | Male | 3.76 (3.31, 4.27） |
| Namibia | 25 to 29 | T1D | Both | 4.59 (4.18, 5.03） |

| | | | | |
|---|---|---|---|---|
| Namibia | 25 to 29 | T1D | Female | 4.60 (4.03, 5.26） |
| Namibia | 25 to 29 | T1D | Male | 4.58 (4.02, 5.22） |
| Namibia | 30 to 34 | T1D | Both | 5.18 (4.70, 5.71） |
| Namibia | 30 to 34 | T1D | Female | 5.21 (4.54, 5.98） |
| Namibia | 30 to 34 | T1D | Male | 5.16 (4.51, 5.92） |
| Namibia | 35 to 39 | T1D | Both | 5.63 (5.09, 6.23） |
| Namibia | 35 to 39 | T1D | Female | 5.66 (4.90, 6.54） |
| Namibia | 35 to 39 | T1D | Male | 5.59 (4.85, 6.45） |
| Namibia | 40 to 44 | T1D | Both | 5.99 (5.39, 6.65） |
| Namibia | 40 to 44 | T1D | Female | 6.04 (5.20, 7.01） |
| Namibia | 40 to 44 | T1D | Male | 5.94 (5.12, 6.90） |
| Namibia | 45 to 49 | T1D | Both | 6.32 (5.65, 7.06） |
| Namibia | 45 to 49 | T1D | Female | 6.37 (5.45, 7.46） |
| Namibia | 45 to 49 | T1D | Male | 6.27 (5.35, 7.34） |
| Namibia | 5 to 9 | T1D | Both | 1.00 (1.00, 1.00） |
| Namibia | 5 to 9 | T1D | Female | 1.00 (1.00, 1.00） |
| Namibia | 5 to 9 | T1D | Male | 1.00 (1.00, 1.00） |
| Namibia | 50 to 54 | T1D | Both | 6.64 (5.90, 7.47） |
| Namibia | 50 to 54 | T1D | Female | 6.70 (5.68, 7.91） |
| Namibia | 50 to 54 | T1D | Male | 6.56 (5.55, 7.76） |
| Namibia | 55 to 59 | T1D | Both | 6.93 (6.11, 7.85） |
| Namibia | 55 to 59 | T1D | Female | 7.03 (5.90, 8.38） |
| Namibia | 55 to 59 | T1D | Male | 6.81 (5.70, 8.15） |
| Namibia | 60 to 64 | T1D | Both | 7.17 (6.27, 8.20） |
| Namibia | 60 to 64 | T1D | Female | 7.28 (6.04, 8.78） |
| Namibia | 60 to 64 | T1D | Male | 7.04 (5.81, 8.53） |
| Namibia | 65 to 69 | T1D | Both | 7.37 (6.38, 8.52） |
| Namibia | 65 to 69 | T1D | Female | 7.53 (6.16, 9.20） |
| Namibia | 65 to 69 | T1D | Male | 7.21 (5.84, 8.89） |
| Nauru | 0 to 4 | T1D | Both | 0.81 (0.02, 35.06） |
| Nauru | 0 to 4 | T1D | Female | 0.80 (0.02, 35.59） |
| Nauru | 0 to 4 | T1D | Male | 0.87 (0.02, 38.38） |
| Nauru | 10 to 14 | T1D | Both | 11.47 (0.71, 185.03） |

| | | | | |
|---|---|---|---|---|
| Nauru | 10 to 14 | T1D | Female | 1.16 (0.03, 47.38） |
| Nauru | 10 to 14 | T1D | Male | 1.33 (0.03, 54.34） |
| Nauru | 15 to 19 | T1D | Both | 12.58 (0.75, 211.08） |
| Nauru | 15 to 19 | T1D | Female | 1.28 (0.03, 61.40） |
| Nauru | 15 to 19 | T1D | Male | 1.77 (0.04, 83.21） |
| Nauru | 20 to 24 | T1D | Both | 13.75 (0.79, 238.51） |
| Nauru | 20 to 24 | T1D | Female | 1.40 (0.02, 81.29） |
| Nauru | 20 to 24 | T1D | Male | 2.37 (0.04, 130.31） |
| Nauru | 25 to 29 | T1D | Both | 14.91 (0.83, 267.60） |
| Nauru | 25 to 29 | T1D | Female | 1.52 (0.02, 110.37） |
| Nauru | 25 to 29 | T1D | Male | 6.68 (0.17, 261.76） |
| Nauru | 30 to 34 | T1D | Both | 16.23 (0.87, 302.36） |
| Nauru | 30 to 34 | T1D | Female | 1.67 (0.02, 155.81） |
| Nauru | 30 to 34 | T1D | Male | 9.74 (0.21, 452.46） |
| Nauru | 35 to 39 | T1D | Both | 18.71 (0.97, 362.28） |
| Nauru | 35 to 39 | T1D | Female | 1.87 (0.02, 225.92） |
| Nauru | 35 to 39 | T1D | Male | 6.63 (0.07, 615.28） |
| Nauru | 40 to 44 | T1D | Both | 22.98 (1.14, 461.92） |
| Nauru | 40 to 44 | T1D | Female | 2.24 (0.01, 336.98） |
| Nauru | 40 to 44 | T1D | Male | 8.51 (0.08, 961.04） |
| Nauru | 45 to 49 | T1D | Both | 28.80 (1.37, 604.23） |
| Nauru | 45 to 49 | T1D | Female | 2.63 (0.01, 489.66） |
| Nauru | 45 to 49 | T1D | Male | 10.61 (0.08, 1478.60） |
| Nauru | 5 to 9 | T1D | Both | 1.00 (1.00, 1.00） |
| Nauru | 5 to 9 | T1D | Female | 1.00 (1.00, 1.00） |
| Nauru | 5 to 9 | T1D | Male | 1.00 (1.00, 1.00） |
| Nauru | 50 to 54 | T1D | Both | 23.63 (0.95, 588.12） |
| Nauru | 50 to 54 | T1D | Female | 3.38 (0.01, 775.95） |
| Nauru | 50 to 54 | T1D | Male | 14.50 (0.08, 2502.78） |
| Nauru | 55 to 59 | T1D | Both | 5.75 (0.10, 330.84） |
| Nauru | 55 to 59 | T1D | Female | 3.93 (0.01, 1104.59） |
| Nauru | 55 to 59 | T1D | Male | 19.77 (0.09, 4227.05） |
| Nauru | 60 to 64 | T1D | Both | 10.47 (0.15, 709.73） |

| | | | | |
|---|---|---|---|---|
| Nauru | 60 to 64 | T1D | Female | 6.47 (0.02, 2237.93） |
| Nauru | 60 to 64 | T1D | Male | 35.66 (0.13, 9427.04） |
| Nauru | 65 to 69 | T1D | Both | 27.08 (0.30, 2428.97） |
| Nauru | 65 to 69 | T1D | Female | 17.13 (0.04, 7610.49） |
| Nauru | 65 to 69 | T1D | Male | 90.44 (0.27, 30661.65） |
| Nepal | 0 to 4 | T1D | Both | 0.15 (0.14, 0.16） |
| Nepal | 0 to 4 | T1D | Female | 0.15 (0.14, 0.16） |
| Nepal | 0 to 4 | T1D | Male | 0.15 (0.14, 0.16） |
| Nepal | 10 to 14 | T1D | Both | 2.17 (2.12, 2.23） |
| Nepal | 10 to 14 | T1D | Female | 2.11 (2.04, 2.18） |
| Nepal | 10 to 14 | T1D | Male | 2.24 (2.16, 2.32） |
| Nepal | 15 to 19 | T1D | Both | 3.04 (2.97, 3.12） |
| Nepal | 15 to 19 | T1D | Female | 2.84 (2.75, 2.94） |
| Nepal | 15 to 19 | T1D | Male | 3.26 (3.14, 3.37） |
| Nepal | 20 to 24 | T1D | Both | 3.62 (3.52, 3.71） |
| Nepal | 20 to 24 | T1D | Female | 3.30 (3.19, 3.42） |
| Nepal | 20 to 24 | T1D | Male | 3.98 (3.83, 4.13） |
| Nepal | 25 to 29 | T1D | Both | 4.15 (4.04, 4.26） |
| Nepal | 25 to 29 | T1D | Female | 3.74 (3.60, 3.88） |
| Nepal | 25 to 29 | T1D | Male | 4.65 (4.48, 4.83） |
| Nepal | 30 to 34 | T1D | Both | 4.55 (4.42, 4.67） |
| Nepal | 30 to 34 | T1D | Female | 4.06 (3.91, 4.22） |
| Nepal | 30 to 34 | T1D | Male | 5.14 (4.94, 5.35） |
| Nepal | 35 to 39 | T1D | Both | 4.88 (4.74, 5.02） |
| Nepal | 35 to 39 | T1D | Female | 4.33 (4.16, 4.51） |
| Nepal | 35 to 39 | T1D | Male | 5.55 (5.32, 5.78） |
| Nepal | 40 to 44 | T1D | Both | 5.17 (5.02, 5.33） |
| Nepal | 40 to 44 | T1D | Female | 4.58 (4.39, 4.78） |
| Nepal | 40 to 44 | T1D | Male | 5.90 (5.65, 6.16） |
| Nepal | 45 to 49 | T1D | Both | 5.43 (5.26, 5.61） |
| Nepal | 45 to 49 | T1D | Female | 4.80 (4.59, 5.02） |
| Nepal | 45 to 49 | T1D | Male | 6.20 (5.93, 6.49） |
| Nepal | 5 to 9 | T1D | Both | 1.00 (1.00, 1.00） |

| | | | | |
|---|---|---|---|---|
| Nepal | 5 to 9 | T1D | Female | 1.00 (1.00, 1.00） |
| Nepal | 5 to 9 | T1D | Male | 1.00 (1.00, 1.00） |
| Nepal | 50 to 54 | T1D | Both | 5.66 (5.47, 5.85） |
| Nepal | 50 to 54 | T1D | Female | 5.00 (4.76, 5.24） |
| Nepal | 50 to 54 | T1D | Male | 6.46 (6.16, 6.77） |
| Nepal | 55 to 59 | T1D | Both | 5.87 (5.67, 6.08） |
| Nepal | 55 to 59 | T1D | Female | 5.20 (4.94, 5.46） |
| Nepal | 55 to 59 | T1D | Male | 6.70 (6.37, 7.04） |
| Nepal | 60 to 64 | T1D | Both | 6.08 (5.86, 6.31） |
| Nepal | 60 to 64 | T1D | Female | 5.41 (5.13, 5.70） |
| Nepal | 60 to 64 | T1D | Male | 6.93 (6.57, 7.30） |
| Nepal | 65 to 69 | T1D | Both | 6.28 (6.03, 6.54） |
| Nepal | 65 to 69 | T1D | Female | 5.61 (5.30, 5.95） |
| Nepal | 65 to 69 | T1D | Male | 7.14 (6.74, 7.56） |
| Netherlands | 0 to 4 | T1D | Both | 0.20 (0.19, 0.21） |
| Netherlands | 0 to 4 | T1D | Female | 0.20 (0.18, 0.21） |
| Netherlands | 0 to 4 | T1D | Male | 0.21 (0.19, 0.23） |
| Netherlands | 10 to 14 | T1D | Both | 2.16 (2.10, 2.23） |
| Netherlands | 10 to 14 | T1D | Female | 2.15 (2.06, 2.24） |
| Netherlands | 10 to 14 | T1D | Male | 2.17 (2.09, 2.27） |
| Netherlands | 15 to 19 | T1D | Both | 3.33 (3.24, 3.43） |
| Netherlands | 15 to 19 | T1D | Female | 3.23 (3.10, 3.37） |
| Netherlands | 15 to 19 | T1D | Male | 3.44 (3.30, 3.58） |
| Netherlands | 20 to 24 | T1D | Both | 4.39 (4.26, 4.52） |
| Netherlands | 20 to 24 | T1D | Female | 4.14 (3.97, 4.32） |
| Netherlands | 20 to 24 | T1D | Male | 4.64 (4.45, 4.83） |
| Netherlands | 25 to 29 | T1D | Both | 5.57 (5.41, 5.74） |
| Netherlands | 25 to 29 | T1D | Female | 5.13 (4.92, 5.35） |
| Netherlands | 25 to 29 | T1D | Male | 6.02 (5.78, 6.28） |
| Netherlands | 30 to 34 | T1D | Both | 6.73 (6.53, 6.94） |
| Netherlands | 30 to 34 | T1D | Female | 6.09 (5.84, 6.36） |
| Netherlands | 30 to 34 | T1D | Male | 7.41 (7.10, 7.73） |
| Netherlands | 35 to 39 | T1D | Both | 7.98 (7.74, 8.23） |

| | | | | |
|---|---|---|---|---|
| Netherlands | 35 to 39 | T1D | Female | 7.12 (6.82, 7.44） |
| Netherlands | 35 to 39 | T1D | Male | 8.90 (8.52, 9.29） |
| Netherlands | 40 to 44 | T1D | Both | 9.36 (9.07, 9.66） |
| Netherlands | 40 to 44 | T1D | Female | 8.26 (7.90, 8.64） |
| Netherlands | 40 to 44 | T1D | Male | 10.53 (10.08, 11.01） |
| Netherlands | 45 to 49 | T1D | Both | 10.86 (10.52, 11.20） |
| Netherlands | 45 to 49 | T1D | Female | 9.51 (9.09, 9.94） |
| Netherlands | 45 to 49 | T1D | Male | 12.31 (11.77, 12.87） |
| Netherlands | 5 to 9 | T1D | Both | 1.00 (1.00, 1.00） |
| Netherlands | 5 to 9 | T1D | Female | 1.00 (1.00, 1.00） |
| Netherlands | 5 to 9 | T1D | Male | 1.00 (1.00, 1.00） |
| Netherlands | 50 to 54 | T1D | Both | 12.48 (12.08, 12.88） |
| Netherlands | 50 to 54 | T1D | Female | 10.86 (10.38, 11.37） |
| Netherlands | 50 to 54 | T1D | Male | 14.22 (13.60, 14.88） |
| Netherlands | 55 to 59 | T1D | Both | 14.23 (13.77, 14.70） |
| Netherlands | 55 to 59 | T1D | Female | 12.34 (11.77, 12.93） |
| Netherlands | 55 to 59 | T1D | Male | 16.28 (15.55, 17.04） |
| Netherlands | 60 to 64 | T1D | Both | 16.13 (15.60, 16.67） |
| Netherlands | 60 to 64 | T1D | Female | 13.96 (13.31, 14.64） |
| Netherlands | 60 to 64 | T1D | Male | 18.51 (17.66, 19.39） |
| Netherlands | 65 to 69 | T1D | Both | 18.22 (17.61, 18.85） |
| Netherlands | 65 to 69 | T1D | Female | 15.76 (15.02, 16.55） |
| Netherlands | 65 to 69 | T1D | Male | 20.96 (19.98, 21.98） |
| New Zealand | 0 to 4 | T1D | Both | 0.24 (0.22, 0.26） |
| New Zealand | 0 to 4 | T1D | Female | 0.24 (0.21, 0.27） |
| New Zealand | 0 to 4 | T1D | Male | 0.23 (0.20, 0.27） |
| New Zealand | 10 to 14 | T1D | Both | 1.96 (1.87, 2.06） |
| New Zealand | 10 to 14 | T1D | Female | 1.94 (1.81, 2.08） |
| New Zealand | 10 to 14 | T1D | Male | 1.99 (1.85, 2.14） |
| New Zealand | 15 to 19 | T1D | Both | 2.88 (2.74, 3.02） |
| New Zealand | 15 to 19 | T1D | Female | 2.80 (2.62, 3.00） |
| New Zealand | 15 to 19 | T1D | Male | 2.95 (2.75, 3.16） |
| New Zealand | 20 to 24 | T1D | Both | 3.68 (3.51, 3.87） |

| | | | | |
|---|---|---|---|---|
| New Zealand | 20 to 24 | T1D | Female | 3.54 (3.30, 3.79） |
| New Zealand | 20 to 24 | T1D | Male | 3.83 (3.57, 4.12） |
| New Zealand | 25 to 29 | T1D | Both | 4.57 (4.34, 4.81） |
| New Zealand | 25 to 29 | T1D | Female | 4.32 (4.02, 4.63） |
| New Zealand | 25 to 29 | T1D | Male | 4.83 (4.49, 5.20） |
| New Zealand | 30 to 34 | T1D | Both | 5.27 (5.01, 5.55） |
| New Zealand | 30 to 34 | T1D | Female | 4.92 (4.58, 5.28） |
| New Zealand | 30 to 34 | T1D | Male | 5.65 (5.24, 6.08） |
| New Zealand | 35 to 39 | T1D | Both | 5.92 (5.62, 6.24） |
| New Zealand | 35 to 39 | T1D | Female | 5.47 (5.09, 5.88） |
| New Zealand | 35 to 39 | T1D | Male | 6.41 (5.95, 6.92） |
| New Zealand | 40 to 44 | T1D | Both | 6.56 (6.23, 6.92） |
| New Zealand | 40 to 44 | T1D | Female | 6.01 (5.58, 6.47） |
| New Zealand | 40 to 44 | T1D | Male | 7.18 (6.65, 7.76） |
| New Zealand | 45 to 49 | T1D | Both | 7.17 (6.79, 7.57） |
| New Zealand | 45 to 49 | T1D | Female | 6.51 (6.04, 7.02） |
| New Zealand | 45 to 49 | T1D | Male | 7.92 (7.32, 8.56） |
| New Zealand | 5 to 9 | T1D | Both | 1.00 (1.00, 1.00） |
| New Zealand | 5 to 9 | T1D | Female | 1.00 (1.00, 1.00） |
| New Zealand | 5 to 9 | T1D | Male | 1.00 (1.00, 1.00） |
| New Zealand | 50 to 54 | T1D | Both | 7.73 (7.32, 8.17） |
| New Zealand | 50 to 54 | T1D | Female | 6.97 (6.46, 7.53） |
| New Zealand | 50 to 54 | T1D | Male | 8.60 (7.94, 9.31） |
| New Zealand | 55 to 59 | T1D | Both | 8.26 (7.81, 8.74） |
| New Zealand | 55 to 59 | T1D | Female | 7.41 (6.86, 8.01） |
| New Zealand | 55 to 59 | T1D | Male | 9.24 (8.52, 10.02） |
| New Zealand | 60 to 64 | T1D | Both | 8.77 (8.28, 9.29） |
| New Zealand | 60 to 64 | T1D | Female | 7.83 (7.23, 8.48） |
| New Zealand | 60 to 64 | T1D | Male | 9.86 (9.07, 10.71） |
| New Zealand | 65 to 69 | T1D | Both | 9.29 (8.76, 9.85） |
| New Zealand | 65 to 69 | T1D | Female | 8.26 (7.61, 8.97） |
| New Zealand | 65 to 69 | T1D | Male | 10.48 (9.62, 11.41） |
| Nicaragua | 0 to 4 | T1D | Both | 0.13 (0.11, 0.15） |

| | | | | |
|---|---|---|---|---|
| Nicaragua | 0 to 4 | T1D | Female | 0.12 (0.09, 0.15） |
| Nicaragua | 0 to 4 | T1D | Male | 0.14 (0.11, 0.17） |
| Nicaragua | 10 to 14 | T1D | Both | 2.25 (2.11, 2.40） |
| Nicaragua | 10 to 14 | T1D | Female | 2.42 (2.22, 2.64） |
| Nicaragua | 10 to 14 | T1D | Male | 2.06 (1.87, 2.27） |
| Nicaragua | 15 to 19 | T1D | Both | 3.04 (2.85, 3.25） |
| Nicaragua | 15 to 19 | T1D | Female | 3.21 (2.94, 3.52） |
| Nicaragua | 15 to 19 | T1D | Male | 2.83 (2.57, 3.12） |
| Nicaragua | 20 to 24 | T1D | Both | 3.55 (3.32, 3.80） |
| Nicaragua | 20 to 24 | T1D | Female | 3.64 (3.32, 4.00） |
| Nicaragua | 20 to 24 | T1D | Male | 3.43 (3.10, 3.79） |
| Nicaragua | 25 to 29 | T1D | Both | 4.03 (3.76, 4.33） |
| Nicaragua | 25 to 29 | T1D | Female | 4.02 (3.65, 4.42） |
| Nicaragua | 25 to 29 | T1D | Male | 4.04 (3.64, 4.48） |
| Nicaragua | 30 to 34 | T1D | Both | 4.43 (4.11, 4.77） |
| Nicaragua | 30 to 34 | T1D | Female | 4.32 (3.90, 4.78） |
| Nicaragua | 30 to 34 | T1D | Male | 4.54 (4.07, 5.07） |
| Nicaragua | 35 to 39 | T1D | Both | 4.77 (4.41, 5.16） |
| Nicaragua | 35 to 39 | T1D | Female | 4.59 (4.12, 5.10） |
| Nicaragua | 35 to 39 | T1D | Male | 4.98 (4.43, 5.59） |
| Nicaragua | 40 to 44 | T1D | Both | 5.09 (4.69, 5.53） |
| Nicaragua | 40 to 44 | T1D | Female | 4.84 (4.33, 5.41） |
| Nicaragua | 40 to 44 | T1D | Male | 5.38 (4.76, 6.08） |
| Nicaragua | 45 to 49 | T1D | Both | 5.39 (4.94, 5.89） |
| Nicaragua | 45 to 49 | T1D | Female | 5.09 (4.51, 5.73） |
| Nicaragua | 45 to 49 | T1D | Male | 5.76 (5.06, 6.55） |
| Nicaragua | 5 to 9 | T1D | Both | 1.00 (1.00, 1.00） |
| Nicaragua | 5 to 9 | T1D | Female | 1.00 (1.00, 1.00） |
| Nicaragua | 5 to 9 | T1D | Male | 1.00 (1.00, 1.00） |
| Nicaragua | 50 to 54 | T1D | Both | 5.68 (5.18, 6.24） |
| Nicaragua | 50 to 54 | T1D | Female | 5.32 (4.69, 6.05） |
| Nicaragua | 50 to 54 | T1D | Male | 6.09 (5.30, 6.99） |
| Nicaragua | 55 to 59 | T1D | Both | 5.96 (5.39, 6.59） |

| | | | | |
|---|---|---|---|---|
| Nicaragua | 55 to 59 | T1D | Female | 5.55 (4.84, 6.37） |
| Nicaragua | 55 to 59 | T1D | Male | 6.41 (5.52, 7.43） |
| Nicaragua | 60 to 64 | T1D | Both | 6.21 (5.56, 6.92） |
| Nicaragua | 60 to 64 | T1D | Female | 5.78 (4.98, 6.71） |
| Nicaragua | 60 to 64 | T1D | Male | 6.71 (5.71, 7.88） |
| Nicaragua | 65 to 69 | T1D | Both | 6.46 (5.72, 7.29） |
| Nicaragua | 65 to 69 | T1D | Female | 6.01 (5.10, 7.10） |
| Nicaragua | 65 to 69 | T1D | Male | 6.98 (5.84, 8.34） |
| Niger | 0 to 4 | T1D | Both | 0.15 (0.14, 0.16） |
| Niger | 0 to 4 | T1D | Female | 0.15 (0.13, 0.16） |
| Niger | 0 to 4 | T1D | Male | 0.15 (0.13, 0.16） |
| Niger | 10 to 14 | T1D | Both | 2.12 (2.05, 2.19） |
| Niger | 10 to 14 | T1D | Female | 2.12 (2.02, 2.23） |
| Niger | 10 to 14 | T1D | Male | 2.11 (2.02, 2.21） |
| Niger | 15 to 19 | T1D | Both | 3.05 (2.95, 3.16） |
| Niger | 15 to 19 | T1D | Female | 3.07 (2.93, 3.23） |
| Niger | 15 to 19 | T1D | Male | 3.04 (2.90, 3.19） |
| Niger | 20 to 24 | T1D | Both | 3.84 (3.70, 3.98） |
| Niger | 20 to 24 | T1D | Female | 3.87 (3.68, 4.08） |
| Niger | 20 to 24 | T1D | Male | 3.82 (3.63, 4.01） |
| Niger | 25 to 29 | T1D | Both | 4.61 (4.44, 4.78） |
| Niger | 25 to 29 | T1D | Female | 4.66 (4.41, 4.91） |
| Niger | 25 to 29 | T1D | Male | 4.57 (4.34, 4.82） |
| Niger | 30 to 34 | T1D | Both | 5.05 (4.86, 5.26） |
| Niger | 30 to 34 | T1D | Female | 5.12 (4.83, 5.41） |
| Niger | 30 to 34 | T1D | Male | 5.00 (4.73, 5.28） |
| Niger | 35 to 39 | T1D | Both | 5.34 (5.12, 5.57） |
| Niger | 35 to 39 | T1D | Female | 5.42 (5.10, 5.75） |
| Niger | 35 to 39 | T1D | Male | 5.27 (4.97, 5.59） |
| Niger | 40 to 44 | T1D | Both | 5.55 (5.31, 5.80） |
| Niger | 40 to 44 | T1D | Female | 5.64 (5.29, 6.00） |
| Niger | 40 to 44 | T1D | Male | 5.47 (5.14, 5.82） |
| Niger | 45 to 49 | T1D | Both | 5.70 (5.43, 5.97） |

| | | | | |
|---|---|---|---|---|
| Niger | 45 to 49 | T1D | Female | 5.80 (5.42, 6.21） |
| Niger | 45 to 49 | T1D | Male | 5.61 (5.25, 5.99） |
| Niger | 5 to 9 | T1D | Both | 1.00 (1.00, 1.00） |
| Niger | 5 to 9 | T1D | Female | 1.00 (1.00, 1.00） |
| Niger | 5 to 9 | T1D | Male | 1.00 (1.00, 1.00） |
| Niger | 50 to 54 | T1D | Both | 5.79 (5.51, 6.09） |
| Niger | 50 to 54 | T1D | Female | 5.91 (5.50, 6.35） |
| Niger | 50 to 54 | T1D | Male | 5.69 (5.30, 6.10） |
| Niger | 55 to 59 | T1D | Both | 5.84 (5.53, 6.16） |
| Niger | 55 to 59 | T1D | Female | 5.97 (5.52, 6.45） |
| Niger | 55 to 59 | T1D | Male | 5.72 (5.30, 6.17） |
| Niger | 60 to 64 | T1D | Both | 5.84 (5.50, 6.20） |
| Niger | 60 to 64 | T1D | Female | 5.99 (5.50, 6.52） |
| Niger | 60 to 64 | T1D | Male | 5.71 (5.25, 6.21） |
| Niger | 65 to 69 | T1D | Both | 5.81 (5.43, 6.21） |
| Niger | 65 to 69 | T1D | Female | 5.96 (5.42, 6.56） |
| Niger | 65 to 69 | T1D | Male | 5.66 (5.15, 6.22） |
| Nigeria | 0 to 4 | T1D | Both | 0.14 (0.14, 0.15） |
| Nigeria | 0 to 4 | T1D | Female | 0.14 (0.14, 0.15） |
| Nigeria | 0 to 4 | T1D | Male | 0.14 (0.14, 0.15） |
| Nigeria | 10 to 14 | T1D | Both | 2.14 (2.12, 2.16） |
| Nigeria | 10 to 14 | T1D | Female | 2.15 (2.12, 2.18） |
| Nigeria | 10 to 14 | T1D | Male | 2.14 (2.11, 2.17） |
| Nigeria | 15 to 19 | T1D | Both | 3.17 (3.13, 3.20） |
| Nigeria | 15 to 19 | T1D | Female | 3.18 (3.13, 3.22） |
| Nigeria | 15 to 19 | T1D | Male | 3.16 (3.12, 3.21） |
| Nigeria | 20 to 24 | T1D | Both | 4.14 (4.10, 4.18） |
| Nigeria | 20 to 24 | T1D | Female | 4.16 (4.09, 4.22） |
| Nigeria | 20 to 24 | T1D | Male | 4.13 (4.07, 4.20） |
| Nigeria | 25 to 29 | T1D | Both | 5.22 (5.16, 5.28） |
| Nigeria | 25 to 29 | T1D | Female | 5.25 (5.17, 5.33） |
| Nigeria | 25 to 29 | T1D | Male | 5.21 (5.12, 5.29） |
| Nigeria | 30 to 34 | T1D | Both | 5.97 (5.90, 6.04） |

| | | | | |
|---|---|---|---|---|
| Nigeria | 30 to 34 | T1D | Female | 6.01 (5.91, 6.11） |
| Nigeria | 30 to 34 | T1D | Male | 5.95 (5.85, 6.04） |
| Nigeria | 35 to 39 | T1D | Both | 6.59 (6.51, 6.67） |
| Nigeria | 35 to 39 | T1D | Female | 6.64 (6.53, 6.76） |
| Nigeria | 35 to 39 | T1D | Male | 6.55 (6.43, 6.66） |
| Nigeria | 40 to 44 | T1D | Both | 7.16 (7.06, 7.25） |
| Nigeria | 40 to 44 | T1D | Female | 7.23 (7.10, 7.36） |
| Nigeria | 40 to 44 | T1D | Male | 7.09 (6.97, 7.22） |
| Nigeria | 45 to 49 | T1D | Both | 7.66 (7.56, 7.77） |
| Nigeria | 45 to 49 | T1D | Female | 7.77 (7.62, 7.92） |
| Nigeria | 45 to 49 | T1D | Male | 7.58 (7.44, 7.72） |
| Nigeria | 5 to 9 | T1D | Both | 1.00 (1.00, 1.00） |
| Nigeria | 5 to 9 | T1D | Female | 1.00 (1.00, 1.00） |
| Nigeria | 5 to 9 | T1D | Male | 1.00 (1.00, 1.00） |
| Nigeria | 50 to 54 | T1D | Both | 8.11 (8.00, 8.22） |
| Nigeria | 50 to 54 | T1D | Female | 8.27 (8.10, 8.43） |
| Nigeria | 50 to 54 | T1D | Male | 7.98 (7.83, 8.14） |
| Nigeria | 55 to 59 | T1D | Both | 8.51 (8.38, 8.64） |
| Nigeria | 55 to 59 | T1D | Female | 8.74 (8.55, 8.93） |
| Nigeria | 55 to 59 | T1D | Male | 8.33 (8.16, 8.51） |
| Nigeria | 60 to 64 | T1D | Both | 8.86 (8.72, 9.01） |
| Nigeria | 60 to 64 | T1D | Female | 9.19 (8.98, 9.41） |
| Nigeria | 60 to 64 | T1D | Male | 8.61 (8.42, 8.81） |
| Nigeria | 65 to 69 | T1D | Both | 9.20 (9.04, 9.37） |
| Nigeria | 65 to 69 | T1D | Female | 9.62 (9.37, 9.87） |
| Nigeria | 65 to 69 | T1D | Male | 8.89 (8.67, 9.12） |
| Niue | 0 to 4 | T1D | Both | 0.89 (0.02, 40.01） |
| Niue | 0 to 4 | T1D | Female | 0.92 (0.02, 41.23） |
| Niue | 0 to 4 | T1D | Male | 0.87 (0.02, 39.14） |
| Niue | 10 to 14 | T1D | Both | 1.13 (0.03, 46.37） |
| Niue | 10 to 14 | T1D | Female | 1.19 (0.03, 48.77） |
| Niue | 10 to 14 | T1D | Male | 1.08 (0.03, 44.39） |
| Niue | 15 to 19 | T1D | Both | 1.42 (0.03, 68.12） |

| | | | | |
|---|---|---|---|---|
| Niue | 15 to 19 | T1D | Female | 1.50 (0.03, 72.02） |
| Niue | 15 to 19 | T1D | Male | 1.36 (0.03, 65.23） |
| Niue | 20 to 24 | T1D | Both | 1.87 (0.03, 108.39） |
| Niue | 20 to 24 | T1D | Female | 1.94 (0.03, 112.93） |
| Niue | 20 to 24 | T1D | Male | 1.81 (0.03, 104.91） |
| Niue | 25 to 29 | T1D | Both | 2.24 (0.03, 162.33） |
| Niue | 25 to 29 | T1D | Female | 2.34 (0.03, 169.56） |
| Niue | 25 to 29 | T1D | Male | 2.16 (0.03, 156.82） |
| Niue | 30 to 34 | T1D | Both | 2.57 (0.03, 240.14） |
| Niue | 30 to 34 | T1D | Female | 2.65 (0.03, 247.72） |
| Niue | 30 to 34 | T1D | Male | 2.51 (0.03, 234.64） |
| Niue | 35 to 39 | T1D | Both | 2.80 (0.02, 339.47） |
| Niue | 35 to 39 | T1D | Female | 2.87 (0.02, 347.64） |
| Niue | 35 to 39 | T1D | Male | 2.75 (0.02, 333.18） |
| Niue | 40 to 44 | T1D | Both | 2.89 (0.02, 435.13） |
| Niue | 40 to 44 | T1D | Female | 2.97 (0.02, 446.11） |
| Niue | 40 to 44 | T1D | Male | 2.84 (0.02, 427.39） |
| Niue | 45 to 49 | T1D | Both | 3.05 (0.02, 567.89） |
| Niue | 45 to 49 | T1D | Female | 3.11 (0.02, 578.63） |
| Niue | 45 to 49 | T1D | Male | 3.03 (0.02, 563.63） |
| Niue | 5 to 9 | T1D | Both | 1.00 (1.00, 1.00） |
| Niue | 5 to 9 | T1D | Female | 1.00 (1.00, 1.00） |
| Niue | 5 to 9 | T1D | Male | 1.00 (1.00, 1.00） |
| Niue | 50 to 54 | T1D | Both | 3.26 (0.01, 748.44） |
| Niue | 50 to 54 | T1D | Female | 3.31 (0.01, 760.01） |
| Niue | 50 to 54 | T1D | Male | 3.24 (0.01, 743.15） |
| Niue | 55 to 59 | T1D | Both | 3.66 (0.01, 1030.28） |
| Niue | 55 to 59 | T1D | Female | 3.76 (0.01, 1057.43） |
| Niue | 55 to 59 | T1D | Male | 3.63 (0.01, 1021.30） |
| Niue | 60 to 64 | T1D | Both | 4.20 (0.01, 1453.11） |
| Niue | 60 to 64 | T1D | Female | 4.22 (0.01, 1459.52） |
| Niue | 60 to 64 | T1D | Male | 4.23 (0.01, 1464.64） |
| Niue | 65 to 69 | T1D | Both | 5.30 (0.01, 2356.03） |

| | | | | |
|---|---|---|---|---|
| Niue | 65 to 69 | T1D | Female | 5.08 (0.01, 2259.28） |
| Niue | 65 to 69 | T1D | Male | 5.67 (0.01, 2519.11） |
| North Macedonia | 0 to 4 | T1D | Both | 0.19 (0.15, 0.24） |
| North Macedonia | 0 to 4 | T1D | Female | 0.18 (0.13, 0.25） |
| North Macedonia | 0 to 4 | T1D | Male | 0.19 (0.14, 0.26） |
| North Macedonia | 10 to 14 | T1D | Both | 2.20 (1.98, 2.44） |
| North Macedonia | 10 to 14 | T1D | Female | 2.19 (1.89, 2.53） |
| North Macedonia | 10 to 14 | T1D | Male | 2.20 (1.90, 2.54） |
| North Macedonia | 15 to 19 | T1D | Both | 3.34 (3.02, 3.70） |
| North Macedonia | 15 to 19 | T1D | Female | 3.26 (2.82, 3.76） |
| North Macedonia | 15 to 19 | T1D | Male | 3.40 (2.94, 3.93） |
| North Macedonia | 20 to 24 | T1D | Both | 4.32 (3.90, 4.79） |
| North Macedonia | 20 to 24 | T1D | Female | 4.12 (3.57, 4.77） |
| North Macedonia | 20 to 24 | T1D | Male | 4.49 (3.89, 5.19） |
| North Macedonia | 25 to 29 | T1D | Both | 5.39 (4.86, 5.98） |
| North Macedonia | 25 to 29 | T1D | Female | 5.01 (4.32, 5.81） |
| North Macedonia | 25 to 29 | T1D | Male | 5.72 (4.94, 6.61） |
| North Macedonia | 30 to 34 | T1D | Both | 6.36 (5.72, 7.07） |
| North Macedonia | 30 to 34 | T1D | Female | 5.81 (5.00, 6.75） |
| North Macedonia | 30 to 34 | T1D | Male | 6.85 (5.91, 7.95） |
| North Macedonia | 35 to 39 | T1D | Both | 7.33 (6.58, 8.17） |
| North Macedonia | 35 to 39 | T1D | Female | 6.61 (5.67, 7.72） |
| North Macedonia | 35 to 39 | T1D | Male | 7.99 (6.87, 9.30） |
| North Macedonia | 40 to 44 | T1D | Both | 8.35 (7.48, 9.33） |
| North Macedonia | 40 to 44 | T1D | Female | 7.47 (6.38, 8.74） |
| North Macedonia | 40 to 44 | T1D | Male | 9.17 (7.87, 10.70） |
| North Macedonia | 45 to 49 | T1D | Both | 9.42 (8.43, 10.54） |
| North Macedonia | 45 to 49 | T1D | Female | 8.37 (7.13, 9.82） |
| North Macedonia | 45 to 49 | T1D | Male | 10.39 (8.89, 12.15） |
| North Macedonia | 5 to 9 | T1D | Both | 1.00 (1.00, 1.00） |
| North Macedonia | 5 to 9 | T1D | Female | 1.00 (1.00, 1.00） |
| North Macedonia | 5 to 9 | T1D | Male | 1.00 (1.00, 1.00） |
| North Macedonia | 50 to 54 | T1D | Both | 10.53 (9.40, 11.81） |

| | | | | |
|---|---|---|---|---|
| North Macedonia | 50 to 54 | T1D | Female | 9.34 (7.93, 10.99） |
| North Macedonia | 50 to 54 | T1D | Male | 11.64 (9.92, 13.65） |
| North Macedonia | 55 to 59 | T1D | Both | 11.69 (10.40, 13.13） |
| North Macedonia | 55 to 59 | T1D | Female | 10.38 (8.79, 12.27） |
| North Macedonia | 55 to 59 | T1D | Male | 12.93 (10.99, 15.21） |
| North Macedonia | 60 to 64 | T1D | Both | 12.92 (11.46, 14.55） |
| North Macedonia | 60 to 64 | T1D | Female | 11.51 (9.70, 13.65） |
| North Macedonia | 60 to 64 | T1D | Male | 14.26 (12.07, 16.85） |
| North Macedonia | 65 to 69 | T1D | Both | 14.26 (12.60, 16.12） |
| North Macedonia | 65 to 69 | T1D | Female | 12.75 (10.70, 15.20） |
| North Macedonia | 65 to 69 | T1D | Male | 15.69 (13.21, 18.64） |
| Northern Mariana Islands | 0 to 4 | T1D | Both | 0.06 (0.01, 1.11） |
| Northern Mariana Islands | 0 to 4 | T1D | Female | 0.10 (0.01, 2.08） |
| Northern Mariana Islands | 0 to 4 | T1D | Male | 0.11 (0.01, 2.22） |
| Northern Mariana Islands | 10 to 14 | T1D | Both | 1.97 (0.74, 5.25） |
| Northern Mariana Islands | 10 to 14 | T1D | Female | 1.21 (0.37, 4.01） |
| Northern Mariana Islands | 10 to 14 | T1D | Male | 1.33 (0.37, 4.84） |
| Northern Mariana Islands | 15 to 19 | T1D | Both | 3.02 (1.13, 8.06） |
| Northern Mariana Islands | 15 to 19 | T1D | Female | 1.68 (0.51, 5.51） |
| Northern Mariana Islands | 15 to 19 | T1D | Male | 2.11 (0.59, 7.59） |
| Northern Mariana Islands | 20 to 24 | T1D | Both | 4.20 (1.52, 11.60） |
| Northern Mariana Islands | 20 to 24 | T1D | Female | 1.96 (0.59, 6.54） |
| Northern Mariana Islands | 20 to 24 | T1D | Male | 2.68 (0.69, 10.37） |
| Northern Mariana Islands | 25 to 29 | T1D | Both | 5.16 (1.84, 14.49） |
| Northern Mariana Islands | 25 to 29 | T1D | Female | 2.34 (0.69, 7.92） |
| Northern Mariana Islands | 25 to 29 | T1D | Male | 3.28 (0.81, 13.22） |
| Northern Mariana Islands | 30 to 34 | T1D | Both | 6.06 (2.10, 17.48） |
| Northern Mariana Islands | 30 to 34 | T1D | Female | 2.58 (0.72, 9.28） |
| Northern Mariana Islands | 30 to 34 | T1D | Male | 3.85 (0.93, 15.87） |
| Northern Mariana Islands | 35 to 39 | T1D | Both | 6.89 (2.35, 20.16） |
| Northern Mariana Islands | 35 to 39 | T1D | Female | 3.07 (0.84, 11.25） |
| Northern Mariana Islands | 35 to 39 | T1D | Male | 4.48 (1.08, 18.59） |
| Northern Mariana Islands | 40 to 44 | T1D | Both | 7.85 (2.64, 23.30） |

| | | | | |
|---|---|---|---|---|
| Northern Mariana Islands | 40 to 44 | T1D | Female | 3.56 (0.94, 13.42） |
| Northern Mariana Islands | 40 to 44 | T1D | Male | 4.94 (1.16, 21.09） |
| Northern Mariana Islands | 45 to 49 | T1D | Both | 8.36 (2.76, 25.34） |
| Northern Mariana Islands | 45 to 49 | T1D | Female | 3.66 (0.93, 14.52） |
| Northern Mariana Islands | 45 to 49 | T1D | Male | 5.41 (1.24, 23.57） |
| Northern Mariana Islands | 5 to 9 | T1D | Both | 1.00 (1.00, 1.00） |
| Northern Mariana Islands | 5 to 9 | T1D | Female | 1.00 (1.00, 1.00） |
| Northern Mariana Islands | 5 to 9 | T1D | Male | 1.00 (1.00, 1.00） |
| Northern Mariana Islands | 50 to 54 | T1D | Both | 9.09 (2.91, 28.37） |
| Northern Mariana Islands | 50 to 54 | T1D | Female | 4.40 (1.08, 17.97） |
| Northern Mariana Islands | 50 to 54 | T1D | Male | 6.28 (1.38, 28.56） |
| Northern Mariana Islands | 55 to 59 | T1D | Both | 10.41 (3.20, 33.84） |
| Northern Mariana Islands | 55 to 59 | T1D | Female | 4.59 (1.01, 20.78） |
| Northern Mariana Islands | 55 to 59 | T1D | Male | 6.71 (1.40, 32.13） |
| Northern Mariana Islands | 60 to 64 | T1D | Both | 11.31 (3.23, 39.61） |
| Northern Mariana Islands | 60 to 64 | T1D | Female | 5.03 (0.98, 25.78） |
| Northern Mariana Islands | 60 to 64 | T1D | Male | 6.98 (1.30, 37.61） |
| Northern Mariana Islands | 65 to 69 | T1D | Both | 11.97 (3.00, 47.81） |
| Northern Mariana Islands | 65 to 69 | T1D | Female | 5.64 (0.91, 35.08） |
| Northern Mariana Islands | 65 to 69 | T1D | Male | 8.88 (1.47, 53.80） |
| Norway | 0 to 4 | T1D | Both | 0.20 (0.19, 0.22） |
| Norway | 0 to 4 | T1D | Female | 0.20 (0.18, 0.23） |
| Norway | 0 to 4 | T1D | Male | 0.20 (0.18, 0.23） |
| Norway | 10 to 14 | T1D | Both | 2.03 (1.94, 2.12） |
| Norway | 10 to 14 | T1D | Female | 2.03 (1.91, 2.15） |
| Norway | 10 to 14 | T1D | Male | 2.03 (1.91, 2.17） |
| Norway | 15 to 19 | T1D | Both | 3.08 (2.95, 3.22） |
| Norway | 15 to 19 | T1D | Female | 3.09 (2.91, 3.29） |
| Norway | 15 to 19 | T1D | Male | 3.07 (2.89, 3.27） |
| Norway | 20 to 24 | T1D | Both | 4.06 (3.88, 4.24） |
| Norway | 20 to 24 | T1D | Female | 4.12 (3.87, 4.38） |
| Norway | 20 to 24 | T1D | Male | 4.01 (3.76, 4.27） |
| Norway | 25 to 29 | T1D | Both | 5.12 (4.90, 5.36） |

| | | | | |
|---|---|---|---|---|
| Norway | 25 to 29 | T1D | Female | 5.26 (4.95, 5.60） |
| Norway | 25 to 29 | T1D | Male | 4.99 (4.69, 5.32） |
| Norway | 30 to 34 | T1D | Both | 6.12 (5.85, 6.40） |
| Norway | 30 to 34 | T1D | Female | 6.40 (6.01, 6.82） |
| Norway | 30 to 34 | T1D | Male | 5.87 (5.50, 6.26） |
| Norway | 35 to 39 | T1D | Both | 7.13 (6.81, 7.47） |
| Norway | 35 to 39 | T1D | Female | 7.62 (7.14, 8.12） |
| Norway | 35 to 39 | T1D | Male | 6.70 (6.27, 7.16） |
| Norway | 40 to 44 | T1D | Both | 8.18 (7.81, 8.58） |
| Norway | 40 to 44 | T1D | Female | 8.95 (8.38, 9.55） |
| Norway | 40 to 44 | T1D | Male | 7.52 (7.03, 8.04） |
| Norway | 45 to 49 | T1D | Both | 9.25 (8.82, 9.70） |
| Norway | 45 to 49 | T1D | Female | 10.36 (9.69, 11.07） |
| Norway | 45 to 49 | T1D | Male | 8.30 (7.75, 8.88） |
| Norway | 5 to 9 | T1D | Both | 1.00 (1.00, 1.00） |
| Norway | 5 to 9 | T1D | Female | 1.00 (1.00, 1.00） |
| Norway | 5 to 9 | T1D | Male | 1.00 (1.00, 1.00） |
| Norway | 50 to 54 | T1D | Both | 10.32 (9.84, 10.83） |
| Norway | 50 to 54 | T1D | Female | 11.87 (11.10, 12.71） |
| Norway | 50 to 54 | T1D | Male | 9.03 (8.42, 9.67） |
| Norway | 55 to 59 | T1D | Both | 11.42 (10.87, 11.99） |
| Norway | 55 to 59 | T1D | Female | 13.48 (12.58, 14.44） |
| Norway | 55 to 59 | T1D | Male | 9.73 (9.07, 10.44） |
| Norway | 60 to 64 | T1D | Both | 12.54 (11.92, 13.18） |
| Norway | 60 to 64 | T1D | Female | 15.17 (14.14, 16.27） |
| Norway | 60 to 64 | T1D | Male | 10.41 (9.69, 11.18） |
| Norway | 65 to 69 | T1D | Both | 13.69 (13.01, 14.41） |
| Norway | 65 to 69 | T1D | Female | 16.96 (15.78, 18.22） |
| Norway | 65 to 69 | T1D | Male | 11.06 (10.28, 11.90） |
| Oman | 0 to 4 | T1D | Both | 0.15 (0.13, 0.18） |
| Oman | 0 to 4 | T1D | Female | 0.16 (0.12, 0.20） |
| Oman | 0 to 4 | T1D | Male | 0.14 (0.11, 0.18） |
| Oman | 10 to 14 | T1D | Both | 2.31 (2.14, 2.49） |

| | | | | |
|---|---|---|---|---|
| Oman | 10 to 14 | T1D | Female | 2.41 (2.15, 2.70） |
| Oman | 10 to 14 | T1D | Male | 2.23 (2.00, 2.47） |
| Oman | 15 to 19 | T1D | Both | 3.63 (3.36, 3.93） |
| Oman | 15 to 19 | T1D | Female | 3.85 (3.43, 4.32） |
| Oman | 15 to 19 | T1D | Male | 3.45 (3.10, 3.83） |
| Oman | 20 to 24 | T1D | Both | 4.91 (4.55, 5.30） |
| Oman | 20 to 24 | T1D | Female | 5.13 (4.57, 5.76） |
| Oman | 20 to 24 | T1D | Male | 4.69 (4.23, 5.20） |
| Oman | 25 to 29 | T1D | Both | 6.43 (5.96, 6.94） |
| Oman | 25 to 29 | T1D | Female | 6.57 (5.85, 7.39） |
| Oman | 25 to 29 | T1D | Male | 6.16 (5.56, 6.82） |
| Oman | 30 to 34 | T1D | Both | 8.00 (7.39, 8.66） |
| Oman | 30 to 34 | T1D | Female | 8.04 (7.12, 9.08） |
| Oman | 30 to 34 | T1D | Male | 7.69 (6.92, 8.55） |
| Oman | 35 to 39 | T1D | Both | 9.68 (8.91, 10.51） |
| Oman | 35 to 39 | T1D | Female | 9.62 (8.46, 10.93） |
| Oman | 35 to 39 | T1D | Male | 9.33 (8.36, 10.41） |
| Oman | 40 to 44 | T1D | Both | 11.52 (10.57, 12.56） |
| Oman | 40 to 44 | T1D | Female | 11.38 (9.94, 13.03） |
| Oman | 40 to 44 | T1D | Male | 11.12 (9.93, 12.46） |
| Oman | 45 to 49 | T1D | Both | 13.54 (12.37, 14.81） |
| Oman | 45 to 49 | T1D | Female | 13.29 (11.51, 15.35） |
| Oman | 45 to 49 | T1D | Male | 13.08 (11.63, 14.71） |
| Oman | 5 to 9 | T1D | Both | 1.00 (1.00, 1.00） |
| Oman | 5 to 9 | T1D | Female | 1.00 (1.00, 1.00） |
| Oman | 5 to 9 | T1D | Male | 1.00 (1.00, 1.00） |
| Oman | 50 to 54 | T1D | Both | 15.70 (14.29, 17.26） |
| Oman | 50 to 54 | T1D | Female | 15.45 (13.26, 18.00） |
| Oman | 50 to 54 | T1D | Male | 15.23 (13.47, 17.23） |
| Oman | 55 to 59 | T1D | Both | 18.09 (16.34, 20.01） |
| Oman | 55 to 59 | T1D | Female | 17.86 (15.16, 21.04） |
| Oman | 55 to 59 | T1D | Male | 17.60 (15.44, 20.06） |
| Oman | 60 to 64 | T1D | Both | 20.63 (18.47, 23.05） |

| | | | | |
|---|---|---|---|---|
| Oman | 60 to 64 | T1D | Female | 20.58 (17.24, 24.56） |
| Oman | 60 to 64 | T1D | Male | 20.13 (17.43, 23.25） |
| Oman | 65 to 69 | T1D | Both | 23.57 (20.81, 26.70） |
| Oman | 65 to 69 | T1D | Female | 23.82 (19.59, 28.96） |
| Oman | 65 to 69 | T1D | Male | 23.02 (19.53, 27.12） |
| Pakistan | 0 to 4 | T1D | Both | 0.14 (0.14, 0.14） |
| Pakistan | 0 to 4 | T1D | Female | 0.14 (0.13, 0.14） |
| Pakistan | 0 to 4 | T1D | Male | 0.14 (0.13, 0.14） |
| Pakistan | 10 to 14 | T1D | Both | 2.18 (2.16, 2.20） |
| Pakistan | 10 to 14 | T1D | Female | 2.13 (2.11, 2.16） |
| Pakistan | 10 to 14 | T1D | Male | 2.24 (2.21, 2.27） |
| Pakistan | 15 to 19 | T1D | Both | 3.13 (3.10, 3.16） |
| Pakistan | 15 to 19 | T1D | Female | 2.96 (2.93, 3.00） |
| Pakistan | 15 to 19 | T1D | Male | 3.30 (3.26, 3.35） |
| Pakistan | 20 to 24 | T1D | Both | 3.87 (3.84, 3.91） |
| Pakistan | 20 to 24 | T1D | Female | 3.57 (3.53, 3.62） |
| Pakistan | 20 to 24 | T1D | Male | 4.18 (4.13, 4.24） |
| Pakistan | 25 to 29 | T1D | Both | 4.63 (4.58, 4.67） |
| Pakistan | 25 to 29 | T1D | Female | 4.19 (4.13, 4.25） |
| Pakistan | 25 to 29 | T1D | Male | 5.08 (5.01, 5.15） |
| Pakistan | 30 to 34 | T1D | Both | 5.17 (5.12, 5.22） |
| Pakistan | 30 to 34 | T1D | Female | 4.64 (4.57, 4.71） |
| Pakistan | 30 to 34 | T1D | Male | 5.72 (5.64, 5.81） |
| Pakistan | 35 to 39 | T1D | Both | 5.62 (5.56, 5.68） |
| Pakistan | 35 to 39 | T1D | Female | 5.02 (4.94, 5.09） |
| Pakistan | 35 to 39 | T1D | Male | 6.25 (6.16, 6.35） |
| Pakistan | 40 to 44 | T1D | Both | 6.03 (5.96, 6.10） |
| Pakistan | 40 to 44 | T1D | Female | 5.37 (5.28, 5.46） |
| Pakistan | 40 to 44 | T1D | Male | 6.73 (6.63, 6.84） |
| Pakistan | 45 to 49 | T1D | Both | 6.39 (6.32, 6.47） |
| Pakistan | 45 to 49 | T1D | Female | 5.68 (5.58, 5.78） |
| Pakistan | 45 to 49 | T1D | Male | 7.15 (7.03, 7.27） |
| Pakistan | 5 to 9 | T1D | Both | 1.00 (1.00, 1.00） |

| | | | | |
|---|---|---|---|---|
| Pakistan | 5 to 9 | T1D | Female | 1.00 (1.00, 1.00） |
| Pakistan | 5 to 9 | T1D | Male | 1.00 (1.00, 1.00） |
| Pakistan | 50 to 54 | T1D | Both | 6.72 (6.64, 6.81） |
| Pakistan | 50 to 54 | T1D | Female | 5.97 (5.86, 6.08） |
| Pakistan | 50 to 54 | T1D | Male | 7.51 (7.38, 7.64） |
| Pakistan | 55 to 59 | T1D | Both | 7.03 (6.93, 7.13） |
| Pakistan | 55 to 59 | T1D | Female | 6.25 (6.12, 6.38） |
| Pakistan | 55 to 59 | T1D | Male | 7.83 (7.68, 7.98） |
| Pakistan | 60 to 64 | T1D | Both | 7.31 (7.20, 7.42） |
| Pakistan | 60 to 64 | T1D | Female | 6.52 (6.38, 6.67） |
| Pakistan | 60 to 64 | T1D | Male | 8.11 (7.95, 8.28） |
| Pakistan | 65 to 69 | T1D | Both | 7.54 (7.41, 7.66） |
| Pakistan | 65 to 69 | T1D | Female | 6.77 (6.60, 6.93） |
| Pakistan | 65 to 69 | T1D | Male | 8.34 (8.16, 8.53） |
| Palau | 0 to 4 | T1D | Both | 0.88 (0.02, 37.76） |
| Palau | 0 to 4 | T1D | Female | 0.91 (0.02, 39.61） |
| Palau | 0 to 4 | T1D | Male | 0.91 (0.02, 39.48） |
| Palau | 10 to 14 | T1D | Both | 10.49 (0.65, 169.09） |
| Palau | 10 to 14 | T1D | Female | 1.01 (0.03, 40.49） |
| Palau | 10 to 14 | T1D | Male | 1.03 (0.03, 40.90） |
| Palau | 15 to 19 | T1D | Both | 11.18 (0.67, 187.03） |
| Palau | 15 to 19 | T1D | Female | 3.27 (0.11, 97.59） |
| Palau | 15 to 19 | T1D | Male | 8.23 (0.38, 176.27） |
| Palau | 20 to 24 | T1D | Both | 11.48 (0.67, 198.20） |
| Palau | 20 to 24 | T1D | Female | 8.65 (0.34, 221.51） |
| Palau | 20 to 24 | T1D | Male | 9.11 (0.43, 194.55） |
| Palau | 25 to 29 | T1D | Both | 19.34 (1.13, 330.16） |
| Palau | 25 to 29 | T1D | Female | 9.52 (0.37, 247.73） |
| Palau | 25 to 29 | T1D | Male | 8.93 (0.40, 199.77） |
| Palau | 30 to 34 | T1D | Both | 22.47 (1.29, 390.08） |
| Palau | 30 to 34 | T1D | Female | 9.97 (0.36, 274.83） |
| Palau | 30 to 34 | T1D | Male | 8.71 (0.37, 203.34） |
| Palau | 35 to 39 | T1D | Both | 23.62 (1.33, 420.81） |

| | | | | |
|---|---|---|---|---|
| Palau | 35 to 39 | T1D | Female | 10.38 (0.36, 298.16） |
| Palau | 35 to 39 | T1D | Male | 9.03 (0.37, 218.94） |
| Palau | 40 to 44 | T1D | Both | 23.72 (1.30, 432.14） |
| Palau | 40 to 44 | T1D | Female | 10.84 (0.37, 320.49） |
| Palau | 40 to 44 | T1D | Male | 9.30 (0.37, 231.31） |
| Palau | 45 to 49 | T1D | Both | 30.52 (1.65, 563.92） |
| Palau | 45 to 49 | T1D | Female | 11.56 (0.38, 353.69） |
| Palau | 45 to 49 | T1D | Male | 11.91 (0.47, 300.02） |
| Palau | 5 to 9 | T1D | Both | 1.00 (1.00, 1.00） |
| Palau | 5 to 9 | T1D | Female | 1.00 (1.00, 1.00） |
| Palau | 5 to 9 | T1D | Male | 1.00 (1.00, 1.00） |
| Palau | 50 to 54 | T1D | Both | 31.69 (1.66, 604.69） |
| Palau | 50 to 54 | T1D | Female | 12.48 (0.39, 402.15） |
| Palau | 50 to 54 | T1D | Male | 11.79 (0.44, 315.61） |
| Palau | 55 to 59 | T1D | Both | 35.07 (1.79, 688.84） |
| Palau | 55 to 59 | T1D | Female | 13.24 (0.38, 459.28） |
| Palau | 55 to 59 | T1D | Male | 13.26 (0.47, 373.07） |
| Palau | 60 to 64 | T1D | Both | 33.38 (1.61, 691.34） |
| Palau | 60 to 64 | T1D | Female | 15.09 (0.39, 587.66） |
| Palau | 60 to 64 | T1D | Male | 15.07 (0.50, 456.57） |
| Palau | 65 to 69 | T1D | Both | 42.74 (1.94, 943.51） |
| Palau | 65 to 69 | T1D | Female | 18.24 (0.37, 890.01） |
| Palau | 65 to 69 | T1D | Male | 16.08 (0.39, 661.02） |
| Palestine | 0 to 4 | T1D | Both | 0.15 (0.13, 0.17） |
| Palestine | 0 to 4 | T1D | Female | 0.16 (0.13, 0.19） |
| Palestine | 0 to 4 | T1D | Male | 0.14 (0.12, 0.17） |
| Palestine | 10 to 14 | T1D | Both | 2.31 (2.18, 2.45） |
| Palestine | 10 to 14 | T1D | Female | 2.42 (2.22, 2.63） |
| Palestine | 10 to 14 | T1D | Male | 2.23 (2.06, 2.41） |
| Palestine | 15 to 19 | T1D | Both | 3.63 (3.42, 3.85） |
| Palestine | 15 to 19 | T1D | Female | 3.85 (3.52, 4.20） |
| Palestine | 15 to 19 | T1D | Male | 3.46 (3.19, 3.75） |
| Palestine | 20 to 24 | T1D | Both | 4.84 (4.55, 5.15） |

| | | | | |
|---|---|---|---|---|
| Palestine | 20 to 24 | T1D | Female | 5.08 (4.64, 5.56） |
| Palestine | 20 to 24 | T1D | Male | 4.65 (4.28, 5.06） |
| Palestine | 25 to 29 | T1D | Both | 6.21 (5.83, 6.63） |
| Palestine | 25 to 29 | T1D | Female | 6.43 (5.85, 7.07） |
| Palestine | 25 to 29 | T1D | Male | 6.04 (5.54, 6.59） |
| Palestine | 30 to 34 | T1D | Both | 7.57 (7.07, 8.10） |
| Palestine | 30 to 34 | T1D | Female | 7.76 (7.02, 8.58） |
| Palestine | 30 to 34 | T1D | Male | 7.43 (6.77, 8.15） |
| Palestine | 35 to 39 | T1D | Both | 8.97 (8.35, 9.63） |
| Palestine | 35 to 39 | T1D | Female | 9.12 (8.21, 10.13） |
| Palestine | 35 to 39 | T1D | Male | 8.86 (8.04, 9.77） |
| Palestine | 40 to 44 | T1D | Both | 10.45 (9.69, 11.26） |
| Palestine | 40 to 44 | T1D | Female | 10.56 (9.45, 11.80） |
| Palestine | 40 to 44 | T1D | Male | 10.36 (9.36, 11.48） |
| Palestine | 45 to 49 | T1D | Both | 12.01 (11.09, 13.00） |
| Palestine | 45 to 49 | T1D | Female | 12.11 (10.77, 13.62） |
| Palestine | 45 to 49 | T1D | Male | 11.93 (10.71, 13.30） |
| Palestine | 5 to 9 | T1D | Both | 1.00 (1.00, 1.00） |
| Palestine | 5 to 9 | T1D | Female | 1.00 (1.00, 1.00） |
| Palestine | 5 to 9 | T1D | Male | 1.00 (1.00, 1.00） |
| Palestine | 50 to 54 | T1D | Both | 13.65 (12.54, 14.86） |
| Palestine | 50 to 54 | T1D | Female | 13.76 (12.14, 15.59） |
| Palestine | 50 to 54 | T1D | Male | 13.56 (12.08, 15.23） |
| Palestine | 55 to 59 | T1D | Both | 15.39 (14.04, 16.87） |
| Palestine | 55 to 59 | T1D | Female | 15.51 (13.56, 17.75） |
| Palestine | 55 to 59 | T1D | Male | 15.29 (13.48, 17.33） |
| Palestine | 60 to 64 | T1D | Both | 17.22 (15.58, 19.03） |
| Palestine | 60 to 64 | T1D | Female | 17.41 (15.06, 20.13） |
| Palestine | 60 to 64 | T1D | Male | 17.05 (14.86, 19.57） |
| Palestine | 65 to 69 | T1D | Both | 19.23 (17.24, 21.45） |
| Palestine | 65 to 69 | T1D | Female | 19.56 (16.71, 22.89） |
| Palestine | 65 to 69 | T1D | Male | 18.94 (16.26, 22.05） |
| Panama | 0 to 4 | T1D | Both | 0.14 (0.11, 0.17） |

| | | | | |
|---|---|---|---|---|
| Panama | 0 to 4 | T1D | Female | 0.12 (0.09, 0.17） |
| Panama | 0 to 4 | T1D | Male | 0.15 (0.11, 0.20） |
| Panama | 10 to 14 | T1D | Both | 2.18 (1.99, 2.39） |
| Panama | 10 to 14 | T1D | Female | 2.32 (2.05, 2.63） |
| Panama | 10 to 14 | T1D | Male | 2.02 (1.77, 2.32） |
| Panama | 15 to 19 | T1D | Both | 3.01 (2.74, 3.30） |
| Panama | 15 to 19 | T1D | Female | 3.15 (2.78, 3.58） |
| Panama | 15 to 19 | T1D | Male | 2.83 (2.47, 3.25） |
| Panama | 20 to 24 | T1D | Both | 3.61 (3.28, 3.97） |
| Panama | 20 to 24 | T1D | Female | 3.68 (3.23, 4.19） |
| Panama | 20 to 24 | T1D | Male | 3.51 (3.05, 4.03） |
| Panama | 25 to 29 | T1D | Both | 4.20 (3.81, 4.63） |
| Panama | 25 to 29 | T1D | Female | 4.16 (3.63, 4.76） |
| Panama | 25 to 29 | T1D | Male | 4.23 (3.67, 4.89） |
| Panama | 30 to 34 | T1D | Both | 4.70 (4.25, 5.20） |
| Panama | 30 to 34 | T1D | Female | 4.56 (3.97, 5.24） |
| Panama | 30 to 34 | T1D | Male | 4.86 (4.19, 5.63） |
| Panama | 35 to 39 | T1D | Both | 5.16 (4.65, 5.74） |
| Panama | 35 to 39 | T1D | Female | 4.93 (4.27, 5.69） |
| Panama | 35 to 39 | T1D | Male | 5.43 (4.66, 6.32） |
| Panama | 40 to 44 | T1D | Both | 5.61 (5.04, 6.25） |
| Panama | 40 to 44 | T1D | Female | 5.29 (4.56, 6.14） |
| Panama | 40 to 44 | T1D | Male | 5.97 (5.10, 6.99） |
| Panama | 45 to 49 | T1D | Both | 6.04 (5.40, 6.76） |
| Panama | 45 to 49 | T1D | Female | 5.64 (4.83, 6.58） |
| Panama | 45 to 49 | T1D | Male | 6.48 (5.50, 7.63） |
| Panama | 5 to 9 | T1D | Both | 1.00 (1.00, 1.00） |
| Panama | 5 to 9 | T1D | Female | 1.00 (1.00, 1.00） |
| Panama | 5 to 9 | T1D | Male | 1.00 (1.00, 1.00） |
| Panama | 50 to 54 | T1D | Both | 6.46 (5.74, 7.26） |
| Panama | 50 to 54 | T1D | Female | 6.00 (5.10, 7.05） |
| Panama | 50 to 54 | T1D | Male | 6.98 (5.89, 8.27） |
| Panama | 55 to 59 | T1D | Both | 6.86 (6.07, 7.76） |

| | | | | |
|---|---|---|---|---|
| Panama | 55 to 59 | T1D | Female | 6.33 (5.34, 7.51） |
| Panama | 55 to 59 | T1D | Male | 7.44 (6.23, 8.89） |
| Panama | 60 to 64 | T1D | Both | 7.23 (6.35, 8.23） |
| Panama | 60 to 64 | T1D | Female | 6.69 (5.58, 8.01） |
| Panama | 60 to 64 | T1D | Male | 7.87 (6.53, 9.49） |
| Panama | 65 to 69 | T1D | Both | 7.62 (6.64, 8.75） |
| Panama | 65 to 69 | T1D | Female | 7.01 (5.78, 8.50） |
| Panama | 65 to 69 | T1D | Male | 8.32 (6.82, 10.15） |
| Papua New Guinea | 0 to 4 | T1D | Both | 0.15 (0.12, 0.17） |
| Papua New Guinea | 0 to 4 | T1D | Female | 0.14 (0.11, 0.18） |
| Papua New Guinea | 0 to 4 | T1D | Male | 0.15 (0.12, 0.19） |
| Papua New Guinea | 10 to 14 | T1D | Both | 2.05 (1.90, 2.22） |
| Papua New Guinea | 10 to 14 | T1D | Female | 2.04 (1.82, 2.27） |
| Papua New Guinea | 10 to 14 | T1D | Male | 2.07 (1.86, 2.31） |
| Papua New Guinea | 15 to 19 | T1D | Both | 2.94 (2.72, 3.18） |
| Papua New Guinea | 15 to 19 | T1D | Female | 2.84 (2.54, 3.18） |
| Papua New Guinea | 15 to 19 | T1D | Male | 3.03 (2.71, 3.38） |
| Papua New Guinea | 20 to 24 | T1D | Both | 3.70 (3.41, 4.01） |
| Papua New Guinea | 20 to 24 | T1D | Female | 3.46 (3.08, 3.88） |
| Papua New Guinea | 20 to 24 | T1D | Male | 3.94 (3.52, 4.41） |
| Papua New Guinea | 25 to 29 | T1D | Both | 4.43 (4.08, 4.82） |
| Papua New Guinea | 25 to 29 | T1D | Female | 4.02 (3.57, 4.53） |
| Papua New Guinea | 25 to 29 | T1D | Male | 4.86 (4.33, 5.47） |
| Papua New Guinea | 30 to 34 | T1D | Both | 4.97 (4.56, 5.42） |
| Papua New Guinea | 30 to 34 | T1D | Female | 4.42 (3.91, 5.01） |
| Papua New Guinea | 30 to 34 | T1D | Male | 5.55 (4.92, 6.27） |
| Papua New Guinea | 35 to 39 | T1D | Both | 5.42 (4.95, 5.94） |
| Papua New Guinea | 35 to 39 | T1D | Female | 4.77 (4.19, 5.44） |
| Papua New Guinea | 35 to 39 | T1D | Male | 6.13 (5.40, 6.96） |
| Papua New Guinea | 40 to 44 | T1D | Both | 5.85 (5.31, 6.43） |
| Papua New Guinea | 40 to 44 | T1D | Female | 5.10 (4.45, 5.85） |
| Papua New Guinea | 40 to 44 | T1D | Male | 6.63 (5.81, 7.56） |
| Papua New Guinea | 45 to 49 | T1D | Both | 6.23 (5.64, 6.89） |

| | | | | |
|---|---|---|---|---|
| Papua New Guinea | 45 to 49 | T1D | Female | 5.41 (4.68, 6.26） |
| Papua New Guinea | 45 to 49 | T1D | Male | 7.07 (6.15, 8.13） |
| Papua New Guinea | 5 to 9 | T1D | Both | 1.00 (1.00, 1.00） |
| Papua New Guinea | 5 to 9 | T1D | Female | 1.00 (1.00, 1.00） |
| Papua New Guinea | 5 to 9 | T1D | Male | 1.00 (1.00, 1.00） |
| Papua New Guinea | 50 to 54 | T1D | Both | 6.59 (5.93, 7.34） |
| Papua New Guinea | 50 to 54 | T1D | Female | 5.71 (4.88, 6.67） |
| Papua New Guinea | 50 to 54 | T1D | Male | 7.47 (6.45, 8.65） |
| Papua New Guinea | 55 to 59 | T1D | Both | 6.91 (6.16, 7.75） |
| Papua New Guinea | 55 to 59 | T1D | Female | 6.00 (5.06, 7.10） |
| Papua New Guinea | 55 to 59 | T1D | Male | 7.82 (6.68, 9.15） |
| Papua New Guinea | 60 to 64 | T1D | Both | 7.20 (6.35, 8.16） |
| Papua New Guinea | 60 to 64 | T1D | Female | 6.24 (5.18, 7.52） |
| Papua New Guinea | 60 to 64 | T1D | Male | 8.13 (6.86, 9.63） |
| Papua New Guinea | 65 to 69 | T1D | Both | 7.45 (6.48, 8.58） |
| Papua New Guinea | 65 to 69 | T1D | Female | 6.52 (5.28, 8.05） |
| Papua New Guinea | 65 to 69 | T1D | Male | 8.37 (6.92, 10.12） |
| Paraguay | 0 to 4 | T1D | Both | 0.15 (0.13, 0.17） |
| Paraguay | 0 to 4 | T1D | Female | 0.16 (0.14, 0.18） |
| Paraguay | 0 to 4 | T1D | Male | 0.12 (0.10, 0.16） |
| Paraguay | 10 to 14 | T1D | Both | 2.29 (2.17, 2.42） |
| Paraguay | 10 to 14 | T1D | Female | 2.29 (2.15, 2.43） |
| Paraguay | 10 to 14 | T1D | Male | 2.30 (2.07, 2.57） |
| Paraguay | 15 to 19 | T1D | Both | 3.36 (3.19, 3.54） |
| Paraguay | 15 to 19 | T1D | Female | 3.38 (3.18, 3.59） |
| Paraguay | 15 to 19 | T1D | Male | 3.27 (2.93, 3.65） |
| Paraguay | 20 to 24 | T1D | Both | 4.18 (3.96, 4.42） |
| Paraguay | 20 to 24 | T1D | Female | 4.22 (3.97, 4.49） |
| Paraguay | 20 to 24 | T1D | Male | 4.02 (3.60, 4.50） |
| Paraguay | 25 to 29 | T1D | Both | 5.00 (4.73, 5.29） |
| Paraguay | 25 to 29 | T1D | Female | 5.07 (4.76, 5.41） |
| Paraguay | 25 to 29 | T1D | Male | 4.77 (4.25, 5.36） |
| Paraguay | 30 to 34 | T1D | Both | 5.72 (5.40, 6.07） |

| | | | | |
|---|---|---|---|---|
| Paraguay | 30 to 34 | T1D | Female | 5.83 (5.46, 6.23） |
| Paraguay | 30 to 34 | T1D | Male | 5.41 (4.80, 6.11） |
| Paraguay | 35 to 39 | T1D | Both | 6.43 (6.05, 6.84） |
| Paraguay | 35 to 39 | T1D | Female | 6.59 (6.14, 7.06） |
| Paraguay | 35 to 39 | T1D | Male | 6.05 (5.33, 6.86） |
| Paraguay | 40 to 44 | T1D | Both | 7.17 (6.72, 7.64） |
| Paraguay | 40 to 44 | T1D | Female | 7.37 (6.85, 7.92） |
| Paraguay | 40 to 44 | T1D | Male | 6.68 (5.85, 7.62） |
| Paraguay | 45 to 49 | T1D | Both | 7.92 (7.41, 8.46） |
| Paraguay | 45 to 49 | T1D | Female | 8.16 (7.57, 8.80） |
| Paraguay | 45 to 49 | T1D | Male | 7.32 (6.37, 8.41） |
| Paraguay | 5 to 9 | T1D | Both | 1.00 (1.00, 1.00） |
| Paraguay | 5 to 9 | T1D | Female | 1.00 (1.00, 1.00） |
| Paraguay | 5 to 9 | T1D | Male | 1.00 (1.00, 1.00） |
| Paraguay | 50 to 54 | T1D | Both | 8.70 (8.12, 9.33） |
| Paraguay | 50 to 54 | T1D | Female | 8.97 (8.29, 9.71） |
| Paraguay | 50 to 54 | T1D | Male | 7.94 (6.87, 9.19） |
| Paraguay | 55 to 59 | T1D | Both | 9.53 (8.85, 10.25） |
| Paraguay | 55 to 59 | T1D | Female | 9.82 (9.04, 10.67） |
| Paraguay | 55 to 59 | T1D | Male | 8.57 (7.34, 10.01） |
| Paraguay | 60 to 64 | T1D | Both | 10.45 (9.67, 11.30） |
| Paraguay | 60 to 64 | T1D | Female | 10.71 (9.81, 11.70） |
| Paraguay | 60 to 64 | T1D | Male | 9.18 (7.78, 10.84） |
| Paraguay | 65 to 69 | T1D | Both | 11.47 (10.55, 12.47） |
| Paraguay | 65 to 69 | T1D | Female | 11.65 (10.60, 12.81） |
| Paraguay | 65 to 69 | T1D | Male | 9.84 (8.20, 11.80） |
| Peru | 0 to 4 | T1D | Both | 0.15 (0.14, 0.16） |
| Peru | 0 to 4 | T1D | Female | 0.15 (0.14, 0.16） |
| Peru | 0 to 4 | T1D | Male | 0.16 (0.15, 0.18） |
| Peru | 10 to 14 | T1D | Both | 2.05 (1.99, 2.11） |
| Peru | 10 to 14 | T1D | Female | 2.11 (2.03, 2.20） |
| Peru | 10 to 14 | T1D | Male | 1.98 (1.90, 2.07） |
| Peru | 15 to 19 | T1D | Both | 2.96 (2.88, 3.05） |

| | | | | |
|---|---|---|---|---|
| Peru | 15 to 19 | T1D | Female | 3.05 (2.94, 3.17） |
| Peru | 15 to 19 | T1D | Male | 2.87 (2.75, 2.99） |
| Peru | 20 to 24 | T1D | Both | 3.71 (3.60, 3.82） |
| Peru | 20 to 24 | T1D | Female | 3.75 (3.60, 3.90） |
| Peru | 20 to 24 | T1D | Male | 3.66 (3.50, 3.82） |
| Peru | 25 to 29 | T1D | Both | 4.44 (4.31, 4.58） |
| Peru | 25 to 29 | T1D | Female | 4.39 (4.22, 4.58） |
| Peru | 25 to 29 | T1D | Male | 4.50 (4.30, 4.70） |
| Peru | 30 to 34 | T1D | Both | 5.05 (4.89, 5.21） |
| Peru | 30 to 34 | T1D | Female | 4.92 (4.72, 5.13） |
| Peru | 30 to 34 | T1D | Male | 5.19 (4.96, 5.44） |
| Peru | 35 to 39 | T1D | Both | 5.60 (5.42, 5.78） |
| Peru | 35 to 39 | T1D | Female | 5.41 (5.18, 5.65） |
| Peru | 35 to 39 | T1D | Male | 5.83 (5.55, 6.11） |
| Peru | 40 to 44 | T1D | Both | 6.13 (5.93, 6.34） |
| Peru | 40 to 44 | T1D | Female | 5.89 (5.63, 6.16） |
| Peru | 40 to 44 | T1D | Male | 6.43 (6.12, 6.76） |
| Peru | 45 to 49 | T1D | Both | 6.65 (6.43, 6.89） |
| Peru | 45 to 49 | T1D | Female | 6.37 (6.08, 6.67） |
| Peru | 45 to 49 | T1D | Male | 7.01 (6.66, 7.38） |
| Peru | 5 to 9 | T1D | Both | 1.00 (1.00, 1.00） |
| Peru | 5 to 9 | T1D | Female | 1.00 (1.00, 1.00） |
| Peru | 5 to 9 | T1D | Male | 1.00 (1.00, 1.00） |
| Peru | 50 to 54 | T1D | Both | 7.17 (6.92, 7.44） |
| Peru | 50 to 54 | T1D | Female | 6.86 (6.53, 7.20） |
| Peru | 50 to 54 | T1D | Male | 7.57 (7.17, 7.99） |
| Peru | 55 to 59 | T1D | Both | 7.68 (7.40, 7.98） |
| Peru | 55 to 59 | T1D | Female | 7.35 (6.98, 7.73） |
| Peru | 55 to 59 | T1D | Male | 8.09 (7.65, 8.56） |
| Peru | 60 to 64 | T1D | Both | 8.19 (7.87, 8.52） |
| Peru | 60 to 64 | T1D | Female | 7.85 (7.44, 8.28） |
| Peru | 60 to 64 | T1D | Male | 8.60 (8.10, 9.12） |
| Peru | 65 to 69 | T1D | Both | 8.72 (8.35, 9.09） |

| | | | | |
|---|---|---|---|---|
| Peru | 65 to 69 | T1D | Female | 8.38 (7.92, 8.88） |
| Peru | 65 to 69 | T1D | Male | 9.11 (8.55, 9.70） |
| Philippines | 0 to 4 | T1D | Both | 0.13 (0.13, 0.14） |
| Philippines | 0 to 4 | T1D | Female | 0.14 (0.13, 0.15） |
| Philippines | 0 to 4 | T1D | Male | 0.13 (0.12, 0.14） |
| Philippines | 10 to 14 | T1D | Both | 2.20 (2.15, 2.24） |
| Philippines | 10 to 14 | T1D | Female | 2.16 (2.10, 2.23） |
| Philippines | 10 to 14 | T1D | Male | 2.23 (2.16, 2.30） |
| Philippines | 15 to 19 | T1D | Both | 3.21 (3.14, 3.28） |
| Philippines | 15 to 19 | T1D | Female | 3.11 (3.01, 3.20） |
| Philippines | 15 to 19 | T1D | Male | 3.32 (3.22, 3.42） |
| Philippines | 20 to 24 | T1D | Both | 4.12 (4.03, 4.21） |
| Philippines | 20 to 24 | T1D | Female | 3.90 (3.78, 4.03） |
| Philippines | 20 to 24 | T1D | Male | 4.35 (4.21, 4.48） |
| Philippines | 25 to 29 | T1D | Both | 5.09 (4.97, 5.21） |
| Philippines | 25 to 29 | T1D | Female | 4.73 (4.58, 4.88） |
| Philippines | 25 to 29 | T1D | Male | 5.47 (5.29, 5.65） |
| Philippines | 30 to 34 | T1D | Both | 5.83 (5.69, 5.97） |
| Philippines | 30 to 34 | T1D | Female | 5.34 (5.16, 5.52） |
| Philippines | 30 to 34 | T1D | Male | 6.33 (6.12, 6.54） |
| Philippines | 35 to 39 | T1D | Both | 6.46 (6.31, 6.63） |
| Philippines | 35 to 39 | T1D | Female | 5.87 (5.67, 6.08） |
| Philippines | 35 to 39 | T1D | Male | 7.08 (6.84, 7.33） |
| Philippines | 40 to 44 | T1D | Both | 7.07 (6.89, 7.25） |
| Philippines | 40 to 44 | T1D | Female | 6.37 (6.15, 6.61） |
| Philippines | 40 to 44 | T1D | Male | 7.78 (7.51, 8.07） |
| Philippines | 45 to 49 | T1D | Both | 7.64 (7.44, 7.84） |
| Philippines | 45 to 49 | T1D | Female | 6.86 (6.61, 7.13） |
| Philippines | 45 to 49 | T1D | Male | 8.44 (8.13, 8.76） |
| Philippines | 5 to 9 | T1D | Both | 1.00 (1.00, 1.00） |
| Philippines | 5 to 9 | T1D | Female | 1.00 (1.00, 1.00） |
| Philippines | 5 to 9 | T1D | Male | 1.00 (1.00, 1.00） |
| Philippines | 50 to 54 | T1D | Both | 8.17 (7.94, 8.39） |

| | | | | |
|---|---|---|---|---|
| Philippines | 50 to 54 | T1D | Female | 7.34 (7.05, 7.63） |
| Philippines | 50 to 54 | T1D | Male | 9.04 (8.69, 9.39） |
| Philippines | 55 to 59 | T1D | Both | 8.67 (8.42, 8.92） |
| Philippines | 55 to 59 | T1D | Female | 7.80 (7.48, 8.13） |
| Philippines | 55 to 59 | T1D | Male | 9.59 (9.20, 9.99） |
| Philippines | 60 to 64 | T1D | Both | 9.14 (8.87, 9.43） |
| Philippines | 60 to 64 | T1D | Female | 8.26 (7.90, 8.63） |
| Philippines | 60 to 64 | T1D | Male | 10.10 (9.67, 10.55） |
| Philippines | 65 to 69 | T1D | Both | 9.60 (9.29, 9.93） |
| Philippines | 65 to 69 | T1D | Female | 8.72 (8.31, 9.14） |
| Philippines | 65 to 69 | T1D | Male | 10.59 (10.09, 11.10） |
| Poland | 0 to 4 | T1D | Both | 0.16 (0.15, 0.17） |
| Poland | 0 to 4 | T1D | Female | 0.16 (0.15, 0.18） |
| Poland | 0 to 4 | T1D | Male | 0.16 (0.15, 0.17） |
| Poland | 10 to 14 | T1D | Both | 2.24 (2.18, 2.30） |
| Poland | 10 to 14 | T1D | Female | 2.20 (2.12, 2.28） |
| Poland | 10 to 14 | T1D | Male | 2.28 (2.20, 2.37） |
| Poland | 15 to 19 | T1D | Both | 3.42 (3.34, 3.51） |
| Poland | 15 to 19 | T1D | Female | 3.29 (3.18, 3.41） |
| Poland | 15 to 19 | T1D | Male | 3.56 (3.43, 3.69） |
| Poland | 20 to 24 | T1D | Both | 4.52 (4.40, 4.63） |
| Poland | 20 to 24 | T1D | Female | 4.25 (4.10, 4.40） |
| Poland | 20 to 24 | T1D | Male | 4.80 (4.63, 4.97） |
| Poland | 25 to 29 | T1D | Both | 5.77 (5.63, 5.92） |
| Poland | 25 to 29 | T1D | Female | 5.30 (5.11, 5.49） |
| Poland | 25 to 29 | T1D | Male | 6.28 (6.05, 6.51） |
| Poland | 30 to 34 | T1D | Both | 6.95 (6.78, 7.13） |
| Poland | 30 to 34 | T1D | Female | 6.26 (6.03, 6.49） |
| Poland | 30 to 34 | T1D | Male | 7.70 (7.42, 7.99） |
| Poland | 35 to 39 | T1D | Both | 8.16 (7.95, 8.38） |
| Poland | 35 to 39 | T1D | Female | 7.22 (6.96, 7.49） |
| Poland | 35 to 39 | T1D | Male | 9.19 (8.85, 9.54） |
| Poland | 40 to 44 | T1D | Both | 9.46 (9.20, 9.71） |

| | | | | |
|---|---|---|---|---|
| Poland | 40 to 44 | T1D | Female | 8.24 (7.94, 8.56） |
| Poland | 40 to 44 | T1D | Male | 10.79 (10.39, 11.21） |
| Poland | 45 to 49 | T1D | Both | 10.82 (10.52, 11.12） |
| Poland | 45 to 49 | T1D | Female | 9.32 (8.96, 9.68） |
| Poland | 45 to 49 | T1D | Male | 12.49 (12.01, 12.98） |
| Poland | 5 to 9 | T1D | Both | 1.00 (1.00, 1.00） |
| Poland | 5 to 9 | T1D | Female | 1.00 (1.00, 1.00） |
| Poland | 5 to 9 | T1D | Male | 1.00 (1.00, 1.00） |
| Poland | 50 to 54 | T1D | Both | 12.24 (11.90, 12.58） |
| Poland | 50 to 54 | T1D | Female | 10.45 (10.05, 10.86） |
| Poland | 50 to 54 | T1D | Male | 14.26 (13.71, 14.84） |
| Poland | 55 to 59 | T1D | Both | 13.72 (13.34, 14.11） |
| Poland | 55 to 59 | T1D | Female | 11.64 (11.18, 12.11） |
| Poland | 55 to 59 | T1D | Male | 16.12 (15.49, 16.78） |
| Poland | 60 to 64 | T1D | Both | 15.28 (14.84, 15.73） |
| Poland | 60 to 64 | T1D | Female | 12.93 (12.41, 13.46） |
| Poland | 60 to 64 | T1D | Male | 18.06 (17.33, 18.82） |
| Poland | 65 to 69 | T1D | Both | 16.96 (16.46, 17.47） |
| Poland | 65 to 69 | T1D | Female | 14.32 (13.74, 14.93） |
| Poland | 65 to 69 | T1D | Male | 20.14 (19.30, 21.01） |
| Portugal | 0 to 4 | T1D | Both | 0.19 (0.18, 0.21） |
| Portugal | 0 to 4 | T1D | Female | 0.18 (0.17, 0.21） |
| Portugal | 0 to 4 | T1D | Male | 0.20 (0.18, 0.22） |
| Portugal | 10 to 14 | T1D | Both | 2.14 (2.06, 2.22） |
| Portugal | 10 to 14 | T1D | Female | 2.11 (2.00, 2.22） |
| Portugal | 10 to 14 | T1D | Male | 2.17 (2.06, 2.29） |
| Portugal | 15 to 19 | T1D | Both | 3.27 (3.15, 3.40） |
| Portugal | 15 to 19 | T1D | Female | 3.13 (2.97, 3.30） |
| Portugal | 15 to 19 | T1D | Male | 3.41 (3.24, 3.60） |
| Portugal | 20 to 24 | T1D | Both | 4.36 (4.20, 4.52） |
| Portugal | 20 to 24 | T1D | Female | 4.06 (3.85, 4.28） |
| Portugal | 20 to 24 | T1D | Male | 4.66 (4.43, 4.91） |
| Portugal | 25 to 29 | T1D | Both | 5.63 (5.42, 5.84） |

| | | | | |
|---|---|---|---|---|
| Portugal | 25 to 29 | T1D | Female | 5.11 (4.85, 5.39） |
| Portugal | 25 to 29 | T1D | Male | 6.17 (5.85, 6.51） |
| Portugal | 30 to 34 | T1D | Both | 6.95 (6.69, 7.22） |
| Portugal | 30 to 34 | T1D | Female | 6.19 (5.86, 6.53） |
| Portugal | 30 to 34 | T1D | Male | 7.77 (7.37, 8.20） |
| Portugal | 35 to 39 | T1D | Both | 8.43 (8.11, 8.76） |
| Portugal | 35 to 39 | T1D | Female | 7.38 (6.99, 7.80） |
| Portugal | 35 to 39 | T1D | Male | 9.58 (9.07, 10.11） |
| Portugal | 40 to 44 | T1D | Both | 10.13 (9.74, 10.54） |
| Portugal | 40 to 44 | T1D | Female | 8.76 (8.29, 9.26） |
| Portugal | 40 to 44 | T1D | Male | 11.67 (11.04, 12.33） |
| Portugal | 45 to 49 | T1D | Both | 12.08 (11.61, 12.57） |
| Portugal | 45 to 49 | T1D | Female | 10.33 (9.76, 10.93） |
| Portugal | 45 to 49 | T1D | Male | 14.07 (13.30, 14.88） |
| Portugal | 5 to 9 | T1D | Both | 1.00 (1.00, 1.00） |
| Portugal | 5 to 9 | T1D | Female | 1.00 (1.00, 1.00） |
| Portugal | 5 to 9 | T1D | Male | 1.00 (1.00, 1.00） |
| Portugal | 50 to 54 | T1D | Both | 14.29 (13.72, 14.88） |
| Portugal | 50 to 54 | T1D | Female | 12.11 (11.44, 12.83） |
| Portugal | 50 to 54 | T1D | Male | 16.80 (15.87, 17.79） |
| Portugal | 55 to 59 | T1D | Both | 16.80 (16.12, 17.51） |
| Portugal | 55 to 59 | T1D | Female | 14.15 (13.34, 15.00） |
| Portugal | 55 to 59 | T1D | Male | 19.90 (18.78, 21.09） |
| Portugal | 60 to 64 | T1D | Both | 19.67 (18.86, 20.51） |
| Portugal | 60 to 64 | T1D | Female | 16.48 (15.52, 17.49） |
| Portugal | 60 to 64 | T1D | Male | 23.45 (22.11, 24.88） |
| Portugal | 65 to 69 | T1D | Both | 22.98 (22.02, 23.98） |
| Portugal | 65 to 69 | T1D | Female | 19.19 (18.06, 20.39） |
| Portugal | 65 to 69 | T1D | Male | 27.55 (25.94, 29.26） |
| Puerto Rico | 0 to 4 | T1D | Both | 0.21 (0.18, 0.25） |
| Puerto Rico | 0 to 4 | T1D | Female | 0.19 (0.15, 0.24） |
| Puerto Rico | 0 to 4 | T1D | Male | 0.24 (0.19, 0.30） |
| Puerto Rico | 10 to 14 | T1D | Both | 2.04 (1.87, 2.22） |

| | | | | |
|---|---|---|---|---|
| Puerto Rico | 10 to 14 | T1D | Female | 2.13 (1.89, 2.39） |
| Puerto Rico | 10 to 14 | T1D | Male | 1.95 (1.73, 2.20） |
| Puerto Rico | 15 to 19 | T1D | Both | 3.12 (2.87, 3.39） |
| Puerto Rico | 15 to 19 | T1D | Female | 3.17 (2.82, 3.56） |
| Puerto Rico | 15 to 19 | T1D | Male | 3.08 (2.74, 3.46） |
| Puerto Rico | 20 to 24 | T1D | Both | 4.05 (3.72, 4.40） |
| Puerto Rico | 20 to 24 | T1D | Female | 3.95 (3.51, 4.44） |
| Puerto Rico | 20 to 24 | T1D | Male | 4.15 (3.69, 4.67） |
| Puerto Rico | 25 to 29 | T1D | Both | 4.93 (4.53, 5.36） |
| Puerto Rico | 25 to 29 | T1D | Female | 4.70 (4.17, 5.30） |
| Puerto Rico | 25 to 29 | T1D | Male | 5.18 (4.59, 5.83） |
| Puerto Rico | 30 to 34 | T1D | Both | 5.66 (5.20, 6.17） |
| Puerto Rico | 30 to 34 | T1D | Female | 5.34 (4.72, 6.03） |
| Puerto Rico | 30 to 34 | T1D | Male | 6.03 (5.34, 6.81） |
| Puerto Rico | 35 to 39 | T1D | Both | 6.34 (5.81, 6.92） |
| Puerto Rico | 35 to 39 | T1D | Female | 5.96 (5.27, 6.75） |
| Puerto Rico | 35 to 39 | T1D | Male | 6.77 (5.99, 7.66） |
| Puerto Rico | 40 to 44 | T1D | Both | 7.01 (6.42, 7.66） |
| Puerto Rico | 40 to 44 | T1D | Female | 6.62 (5.84, 7.51） |
| Puerto Rico | 40 to 44 | T1D | Male | 7.47 (6.59, 8.46） |
| Puerto Rico | 45 to 49 | T1D | Both | 7.67 (7.01, 8.39） |
| Puerto Rico | 45 to 49 | T1D | Female | 7.29 (6.42, 8.28） |
| Puerto Rico | 45 to 49 | T1D | Male | 8.12 (7.16, 9.22） |
| Puerto Rico | 5 to 9 | T1D | Both | 1.00 (1.00, 1.00） |
| Puerto Rico | 5 to 9 | T1D | Female | 1.00 (1.00, 1.00） |
| Puerto Rico | 5 to 9 | T1D | Male | 1.00 (1.00, 1.00） |
| Puerto Rico | 50 to 54 | T1D | Both | 8.30 (7.58, 9.10） |
| Puerto Rico | 50 to 54 | T1D | Female | 7.97 (7.00, 9.08） |
| Puerto Rico | 50 to 54 | T1D | Male | 8.72 (7.67, 9.92） |
| Puerto Rico | 55 to 59 | T1D | Both | 8.94 (8.15, 9.81） |
| Puerto Rico | 55 to 59 | T1D | Female | 8.69 (7.62, 9.91） |
| Puerto Rico | 55 to 59 | T1D | Male | 9.29 (8.15, 10.58） |
| Puerto Rico | 60 to 64 | T1D | Both | 9.59 (8.73, 10.54） |

| | | | | |
|---|---|---|---|---|
| Puerto Rico | 60 to 64 | T1D | Female | 9.47 (8.28, 10.83） |
| Puerto Rico | 60 to 64 | T1D | Male | 9.84 (8.62, 11.24） |
| Puerto Rico | 65 to 69 | T1D | Both | 10.29 (9.34, 11.33） |
| Puerto Rico | 65 to 69 | T1D | Female | 10.33 (9.00, 11.85） |
| Puerto Rico | 65 to 69 | T1D | Male | 10.42 (9.10, 11.93） |
| Qatar | 0 to 4 | T1D | Both | 0.16 (0.12, 0.22） |
| Qatar | 0 to 4 | T1D | Female | 0.17 (0.11, 0.27） |
| Qatar | 0 to 4 | T1D | Male | 0.16 (0.10, 0.24） |
| Qatar | 10 to 14 | T1D | Both | 2.27 (1.95, 2.65） |
| Qatar | 10 to 14 | T1D | Female | 2.38 (1.89, 2.99） |
| Qatar | 10 to 14 | T1D | Male | 2.17 (1.76, 2.67） |
| Qatar | 15 to 19 | T1D | Both | 3.60 (3.07, 4.22） |
| Qatar | 15 to 19 | T1D | Female | 3.80 (2.99, 4.83） |
| Qatar | 15 to 19 | T1D | Male | 3.39 (2.74, 4.19） |
| Qatar | 20 to 24 | T1D | Both | 4.94 (4.23, 5.76） |
| Qatar | 20 to 24 | T1D | Female | 5.09 (3.99, 6.48） |
| Qatar | 20 to 24 | T1D | Male | 4.59 (3.75, 5.63） |
| Qatar | 25 to 29 | T1D | Both | 6.50 (5.56, 7.59） |
| Qatar | 25 to 29 | T1D | Female | 6.53 (5.12, 8.31） |
| Qatar | 25 to 29 | T1D | Male | 6.06 (4.93, 7.44） |
| Qatar | 30 to 34 | T1D | Both | 8.09 (6.90, 9.49） |
| Qatar | 30 to 34 | T1D | Female | 8.01 (6.23, 10.29） |
| Qatar | 30 to 34 | T1D | Male | 7.56 (6.13, 9.32） |
| Qatar | 35 to 39 | T1D | Both | 9.80 (8.32, 11.55） |
| Qatar | 35 to 39 | T1D | Female | 9.57 (7.37, 12.43） |
| Qatar | 35 to 39 | T1D | Male | 9.15 (7.38, 11.35） |
| Qatar | 40 to 44 | T1D | Both | 11.66 (9.86, 13.80） |
| Qatar | 40 to 44 | T1D | Female | 11.34 (8.64, 14.89） |
| Qatar | 40 to 44 | T1D | Male | 10.91 (8.76, 13.58） |
| Qatar | 45 to 49 | T1D | Both | 13.71 (11.54, 16.29） |
| Qatar | 45 to 49 | T1D | Female | 13.18 (9.92, 17.52） |
| Qatar | 45 to 49 | T1D | Male | 12.79 (10.23, 15.99） |
| Qatar | 5 to 9 | T1D | Both | 1.00 (1.00, 1.00） |

| | | | | |
|---|---|---|---|---|
| Qatar | 5 to 9 | T1D | Female | 1.00 (1.00, 1.00） |
| Qatar | 5 to 9 | T1D | Male | 1.00 (1.00, 1.00） |
| Qatar | 50 to 54 | T1D | Both | 15.90 (13.31, 19.00） |
| Qatar | 50 to 54 | T1D | Female | 15.22 (11.28, 20.54） |
| Qatar | 50 to 54 | T1D | Male | 14.86 (11.82, 18.69） |
| Qatar | 55 to 59 | T1D | Both | 18.33 (15.22, 22.08） |
| Qatar | 55 to 59 | T1D | Female | 17.61 (12.77, 24.28） |
| Qatar | 55 to 59 | T1D | Male | 17.10 (13.49, 21.67） |
| Qatar | 60 to 64 | T1D | Both | 20.94 (17.11, 25.63） |
| Qatar | 60 to 64 | T1D | Female | 20.51 (14.38, 29.26） |
| Qatar | 60 to 64 | T1D | Male | 19.60 (15.20, 25.26） |
| Qatar | 65 to 69 | T1D | Both | 23.90 (18.99, 30.09） |
| Qatar | 65 to 69 | T1D | Female | 23.01 (15.28, 34.66） |
| Qatar | 65 to 69 | T1D | Male | 22.13 (16.61, 29.47） |
| Republic of Korea | 0 to 4 | T1D | Both | 0.16 (0.16, 0.17） |
| Republic of Korea | 0 to 4 | T1D | Female | 0.16 (0.15, 0.17） |
| Republic of Korea | 0 to 4 | T1D | Male | 0.17 (0.16, 0.17） |
| Republic of Korea | 10 to 14 | T1D | Both | 1.80 (1.77, 1.82） |
| Republic of Korea | 10 to 14 | T1D | Female | 1.78 (1.75, 1.82） |
| Republic of Korea | 10 to 14 | T1D | Male | 1.81 (1.78, 1.84） |
| Republic of Korea | 15 to 19 | T1D | Both | 2.02 (2.00, 2.05） |
| Republic of Korea | 15 to 19 | T1D | Female | 2.01 (1.97, 2.04） |
| Republic of Korea | 15 to 19 | T1D | Male | 2.04 (2.00, 2.08） |
| Republic of Korea | 20 to 24 | T1D | Both | 2.09 (2.06, 2.12） |
| Republic of Korea | 20 to 24 | T1D | Female | 2.07 (2.03, 2.11） |
| Republic of Korea | 20 to 24 | T1D | Male | 2.10 (2.06, 2.14） |
| Republic of Korea | 25 to 29 | T1D | Both | 2.15 (2.12, 2.18） |
| Republic of Korea | 25 to 29 | T1D | Female | 2.13 (2.09, 2.18） |
| Republic of Korea | 25 to 29 | T1D | Male | 2.17 (2.13, 2.21） |
| Republic of Korea | 30 to 34 | T1D | Both | 2.22 (2.19, 2.25） |
| Republic of Korea | 30 to 34 | T1D | Female | 2.20 (2.15, 2.24） |
| Republic of Korea | 30 to 34 | T1D | Male | 2.24 (2.20, 2.28） |
| Republic of Korea | 35 to 39 | T1D | Both | 2.29 (2.25, 2.32） |

| | | | | |
|---|---|---|---|---|
| Republic of Korea | 35 to 39 | T1D | Female | 2.26 (2.22, 2.31） |
| Republic of Korea | 35 to 39 | T1D | Male | 2.31 (2.26, 2.35） |
| Republic of Korea | 40 to 44 | T1D | Both | 2.36 (2.33, 2.40） |
| Republic of Korea | 40 to 44 | T1D | Female | 2.34 (2.29, 2.39） |
| Republic of Korea | 40 to 44 | T1D | Male | 2.38 (2.33, 2.43） |
| Republic of Korea | 45 to 49 | T1D | Both | 2.44 (2.40, 2.47） |
| Republic of Korea | 45 to 49 | T1D | Female | 2.41 (2.36, 2.47） |
| Republic of Korea | 45 to 49 | T1D | Male | 2.46 (2.41, 2.51） |
| Republic of Korea | 5 to 9 | T1D | Both | 1.00 (1.00, 1.00） |
| Republic of Korea | 5 to 9 | T1D | Female | 1.00 (1.00, 1.00） |
| Republic of Korea | 5 to 9 | T1D | Male | 1.00 (1.00, 1.00） |
| Republic of Korea | 50 to 54 | T1D | Both | 2.52 (2.48, 2.56） |
| Republic of Korea | 50 to 54 | T1D | Female | 2.50 (2.44, 2.56） |
| Republic of Korea | 50 to 54 | T1D | Male | 2.54 (2.48, 2.59） |
| Republic of Korea | 55 to 59 | T1D | Both | 2.60 (2.56, 2.65） |
| Republic of Korea | 55 to 59 | T1D | Female | 2.59 (2.52, 2.65） |
| Republic of Korea | 55 to 59 | T1D | Male | 2.62 (2.56, 2.68） |
| Republic of Korea | 60 to 64 | T1D | Both | 2.69 (2.64, 2.74） |
| Republic of Korea | 60 to 64 | T1D | Female | 2.68 (2.61, 2.75） |
| Republic of Korea | 60 to 64 | T1D | Male | 2.70 (2.64, 2.77） |
| Republic of Korea | 65 to 69 | T1D | Both | 2.78 (2.73, 2.83） |
| Republic of Korea | 65 to 69 | T1D | Female | 2.78 (2.71, 2.86） |
| Republic of Korea | 65 to 69 | T1D | Male | 2.79 (2.72, 2.87） |
| Republic of Moldova | 0 to 4 | T1D | Both | 0.19 (0.15, 0.22） |
| Republic of Moldova | 0 to 4 | T1D | Female | 0.18 (0.14, 0.23） |
| Republic of Moldova | 0 to 4 | T1D | Male | 0.19 (0.15, 0.25） |
| Republic of Moldova | 10 to 14 | T1D | Both | 2.34 (2.15, 2.54） |
| Republic of Moldova | 10 to 14 | T1D | Female | 2.35 (2.08, 2.65） |
| Republic of Moldova | 10 to 14 | T1D | Male | 2.31 (2.06, 2.60） |
| Republic of Moldova | 15 to 19 | T1D | Both | 3.67 (3.38, 3.99） |
| Republic of Moldova | 15 to 19 | T1D | Female | 3.67 (3.26, 4.13） |
| Republic of Moldova | 15 to 19 | T1D | Male | 3.67 (3.27, 4.12） |
| Republic of Moldova | 20 to 24 | T1D | Both | 4.81 (4.43, 5.22） |

| | | | | |
|---|---|---|---|---|
| Republic of Moldova | 20 to 24 | T1D | Female | 4.71 (4.19, 5.30） |
| Republic of Moldova | 20 to 24 | T1D | Male | 4.88 (4.35, 5.48） |
| Republic of Moldova | 25 to 29 | T1D | Both | 6.05 (5.57, 6.57） |
| Republic of Moldova | 25 to 29 | T1D | Female | 5.80 (5.15, 6.53） |
| Republic of Moldova | 25 to 29 | T1D | Male | 6.27 (5.58, 7.04） |
| Republic of Moldova | 30 to 34 | T1D | Both | 7.21 (6.63, 7.85） |
| Republic of Moldova | 30 to 34 | T1D | Female | 6.81 (6.03, 7.68） |
| Republic of Moldova | 30 to 34 | T1D | Male | 7.58 (6.74, 8.53） |
| Republic of Moldova | 35 to 39 | T1D | Both | 8.37 (7.68, 9.13） |
| Republic of Moldova | 35 to 39 | T1D | Female | 7.82 (6.91, 8.85） |
| Republic of Moldova | 35 to 39 | T1D | Male | 8.89 (7.89, 10.03） |
| Republic of Moldova | 40 to 44 | T1D | Both | 9.57 (8.77, 10.44） |
| Republic of Moldova | 40 to 44 | T1D | Female | 8.89 (7.84, 10.07） |
| Republic of Moldova | 40 to 44 | T1D | Male | 10.23 (9.06, 11.56） |
| Republic of Moldova | 45 to 49 | T1D | Both | 10.80 (9.88, 11.80） |
| Republic of Moldova | 45 to 49 | T1D | Female | 10.02 (8.82, 11.37） |
| Republic of Moldova | 45 to 49 | T1D | Male | 11.58 (10.23, 13.10） |
| Republic of Moldova | 5 to 9 | T1D | Both | 1.00 (1.00, 1.00） |
| Republic of Moldova | 5 to 9 | T1D | Female | 1.00 (1.00, 1.00） |
| Republic of Moldova | 5 to 9 | T1D | Male | 1.00 (1.00, 1.00） |
| Republic of Moldova | 50 to 54 | T1D | Both | 12.04 (11.00, 13.18） |
| Republic of Moldova | 50 to 54 | T1D | Female | 11.20 (9.84, 12.74） |
| Republic of Moldova | 50 to 54 | T1D | Male | 12.93 (11.40, 14.66） |
| Republic of Moldova | 55 to 59 | T1D | Both | 13.34 (12.17, 14.62） |
| Republic of Moldova | 55 to 59 | T1D | Female | 12.49 (10.95, 14.23） |
| Republic of Moldova | 55 to 59 | T1D | Male | 14.31 (12.59, 16.27） |
| Republic of Moldova | 60 to 64 | T1D | Both | 14.70 (13.38, 16.14） |
| Republic of Moldova | 60 to 64 | T1D | Female | 13.88 (12.14, 15.87） |
| Republic of Moldova | 60 to 64 | T1D | Male | 15.68 (13.74, 17.89） |
| Republic of Moldova | 65 to 69 | T1D | Both | 16.13 (14.64, 17.78） |
| Republic of Moldova | 65 to 69 | T1D | Female | 15.39 (13.41, 17.66） |
| Republic of Moldova | 65 to 69 | T1D | Male | 17.09 (14.90, 19.60） |
| Romania | 0 to 4 | T1D | Both | 0.17 (0.16, 0.19） |

| | | | | |
|---|---|---|---|---|
| Romania | 0 to 4 | T1D | Female | 0.18 (0.16, 0.20） |
| Romania | 0 to 4 | T1D | Male | 0.17 (0.15, 0.19） |
| Romania | 10 to 14 | T1D | Both | 2.21 (2.11, 2.32） |
| Romania | 10 to 14 | T1D | Female | 2.18 (2.08, 2.29） |
| Romania | 10 to 14 | T1D | Male | 2.24 (2.12, 2.37） |
| Romania | 15 to 19 | T1D | Both | 3.21 (3.06, 3.36） |
| Romania | 15 to 19 | T1D | Female | 3.15 (3.00, 3.31） |
| Romania | 15 to 19 | T1D | Male | 3.27 (3.09, 3.46） |
| Romania | 20 to 24 | T1D | Both | 4.09 (3.90, 4.29） |
| Romania | 20 to 24 | T1D | Female | 3.99 (3.80, 4.19） |
| Romania | 20 to 24 | T1D | Male | 4.19 (3.95, 4.43） |
| Romania | 25 to 29 | T1D | Both | 5.14 (4.90, 5.39） |
| Romania | 25 to 29 | T1D | Female | 5.00 (4.76, 5.25） |
| Romania | 25 to 29 | T1D | Male | 5.29 (4.99, 5.61） |
| Romania | 30 to 34 | T1D | Both | 6.24 (5.95, 6.55） |
| Romania | 30 to 34 | T1D | Female | 6.03 (5.73, 6.34） |
| Romania | 30 to 34 | T1D | Male | 6.46 (6.09, 6.86） |
| Romania | 35 to 39 | T1D | Both | 7.47 (7.11, 7.85） |
| Romania | 35 to 39 | T1D | Female | 7.16 (6.81, 7.54） |
| Romania | 35 to 39 | T1D | Male | 7.79 (7.34, 8.28） |
| Romania | 40 to 44 | T1D | Both | 8.86 (8.42, 9.32） |
| Romania | 40 to 44 | T1D | Female | 8.43 (8.01, 8.88） |
| Romania | 40 to 44 | T1D | Male | 9.30 (8.74, 9.88） |
| Romania | 45 to 49 | T1D | Both | 10.45 (9.93, 11.00） |
| Romania | 45 to 49 | T1D | Female | 9.89 (9.38, 10.43） |
| Romania | 45 to 49 | T1D | Male | 11.03 (10.36, 11.74） |
| Romania | 5 to 9 | T1D | Both | 1.00 (1.00, 1.00） |
| Romania | 5 to 9 | T1D | Female | 1.00 (1.00, 1.00） |
| Romania | 5 to 9 | T1D | Male | 1.00 (1.00, 1.00） |
| Romania | 50 to 54 | T1D | Both | 12.25 (11.63, 12.91） |
| Romania | 50 to 54 | T1D | Female | 11.54 (10.94, 12.19） |
| Romania | 50 to 54 | T1D | Male | 13.00 (12.19, 13.85） |
| Romania | 55 to 59 | T1D | Both | 14.25 (13.51, 15.03） |

| | | | | |
|---|---|---|---|---|
| Romania | 55 to 59 | T1D | Female | 13.39 (12.67, 14.14） |
| Romania | 55 to 59 | T1D | Male | 15.18 (14.22, 16.19） |
| Romania | 60 to 64 | T1D | Both | 16.41 (15.55, 17.33） |
| Romania | 60 to 64 | T1D | Female | 15.37 (14.54, 16.25） |
| Romania | 60 to 64 | T1D | Male | 17.54 (16.42, 18.75） |
| Romania | 65 to 69 | T1D | Both | 18.85 (17.83, 19.94） |
| Romania | 65 to 69 | T1D | Female | 17.60 (16.62, 18.64） |
| Romania | 65 to 69 | T1D | Male | 20.22 (18.88, 21.65） |
| Russian Federation | 0 to 4 | T1D | Both | 0.18 (0.17, 0.20） |
| Russian Federation | 0 to 4 | T1D | Female | 0.19 (0.18, 0.20） |
| Russian Federation | 0 to 4 | T1D | Male | 0.18 (0.16, 0.20） |
| Russian Federation | 10 to 14 | T1D | Both | 2.24 (2.16, 2.31） |
| Russian Federation | 10 to 14 | T1D | Female | 2.24 (2.17, 2.30） |
| Russian Federation | 10 to 14 | T1D | Male | 2.24 (2.14, 2.35） |
| Russian Federation | 15 to 19 | T1D | Both | 3.48 (3.37, 3.59） |
| Russian Federation | 15 to 19 | T1D | Female | 3.40 (3.31, 3.50） |
| Russian Federation | 15 to 19 | T1D | Male | 3.55 (3.39, 3.72） |
| Russian Federation | 20 to 24 | T1D | Both | 4.64 (4.49, 4.79） |
| Russian Federation | 20 to 24 | T1D | Female | 4.47 (4.35, 4.60） |
| Russian Federation | 20 to 24 | T1D | Male | 4.81 (4.60, 5.03） |
| Russian Federation | 25 to 29 | T1D | Both | 5.95 (5.76, 6.14） |
| Russian Federation | 25 to 29 | T1D | Female | 5.72 (5.56, 5.88） |
| Russian Federation | 25 to 29 | T1D | Male | 6.18 (5.90, 6.46） |
| Russian Federation | 30 to 34 | T1D | Both | 7.10 (6.87, 7.34） |
| Russian Federation | 30 to 34 | T1D | Female | 6.86 (6.67, 7.06） |
| Russian Federation | 30 to 34 | T1D | Male | 7.35 (7.02, 7.70） |
| Russian Federation | 35 to 39 | T1D | Both | 8.26 (7.98, 8.54） |
| Russian Federation | 35 to 39 | T1D | Female | 8.04 (7.81, 8.27） |
| Russian Federation | 35 to 39 | T1D | Male | 8.49 (8.10, 8.89） |
| Russian Federation | 40 to 44 | T1D | Both | 9.46 (9.15, 9.79） |
| Russian Federation | 40 to 44 | T1D | Female | 9.31 (9.04, 9.59） |
| Russian Federation | 40 to 44 | T1D | Male | 9.64 (9.19, 10.10） |
| Russian Federation | 45 to 49 | T1D | Both | 10.69 (10.33, 11.06） |

| | | | | |
|---|---|---|---|---|
| Russian Federation | 45 to 49 | T1D | Female | 10.65 (10.33, 10.97） |
| Russian Federation | 45 to 49 | T1D | Male | 10.77 (10.27, 11.30） |
| Russian Federation | 5 to 9 | T1D | Both | 1.00 (1.00, 1.00） |
| Russian Federation | 5 to 9 | T1D | Female | 1.00 (1.00, 1.00） |
| Russian Federation | 5 to 9 | T1D | Male | 1.00 (1.00, 1.00） |
| Russian Federation | 50 to 54 | T1D | Both | 11.91 (11.50, 12.33） |
| Russian Federation | 50 to 54 | T1D | Female | 12.04 (11.68, 12.41） |
| Russian Federation | 50 to 54 | T1D | Male | 11.82 (11.26, 12.41） |
| Russian Federation | 55 to 59 | T1D | Both | 13.15 (12.70, 13.62） |
| Russian Federation | 55 to 59 | T1D | Female | 13.52 (13.12, 13.94） |
| Russian Federation | 55 to 59 | T1D | Male | 12.86 (12.24, 13.51） |
| Russian Federation | 60 to 64 | T1D | Both | 14.49 (13.98, 15.02） |
| Russian Federation | 60 to 64 | T1D | Female | 15.17 (14.71, 15.65） |
| Russian Federation | 60 to 64 | T1D | Male | 13.90 (13.21, 14.62） |
| Russian Federation | 65 to 69 | T1D | Both | 15.83 (15.26, 16.42） |
| Russian Federation | 65 to 69 | T1D | Female | 16.93 (16.40, 17.47） |
| Russian Federation | 65 to 69 | T1D | Male | 14.86 (14.11, 15.65） |
| Rwanda | 0 to 4 | T1D | Both | 0.16 (0.15, 0.17） |
| Rwanda | 0 to 4 | T1D | Female | 0.16 (0.14, 0.18） |
| Rwanda | 0 to 4 | T1D | Male | 0.16 (0.14, 0.18） |
| Rwanda | 10 to 14 | T1D | Both | 2.03 (1.95, 2.11） |
| Rwanda | 10 to 14 | T1D | Female | 2.03 (1.91, 2.15） |
| Rwanda | 10 to 14 | T1D | Male | 2.03 (1.91, 2.15） |
| Rwanda | 15 to 19 | T1D | Both | 2.95 (2.83, 3.08） |
| Rwanda | 15 to 19 | T1D | Female | 2.96 (2.79, 3.14） |
| Rwanda | 15 to 19 | T1D | Male | 2.95 (2.78, 3.12） |
| Rwanda | 20 to 24 | T1D | Both | 3.79 (3.64, 3.96） |
| Rwanda | 20 to 24 | T1D | Female | 3.81 (3.59, 4.04） |
| Rwanda | 20 to 24 | T1D | Male | 3.78 (3.57, 4.02） |
| Rwanda | 25 to 29 | T1D | Both | 4.63 (4.43, 4.84） |
| Rwanda | 25 to 29 | T1D | Female | 4.65 (4.37, 4.95） |
| Rwanda | 25 to 29 | T1D | Male | 4.61 (4.34, 4.90） |
| Rwanda | 30 to 34 | T1D | Both | 5.14 (4.91, 5.38） |

| | | | | |
|---|---|---|---|---|
| Rwanda | 30 to 34 | T1D | Female | 5.17 (4.85, 5.52） |
| Rwanda | 30 to 34 | T1D | Male | 5.11 (4.79, 5.45） |
| Rwanda | 35 to 39 | T1D | Both | 5.50 (5.24, 5.77） |
| Rwanda | 35 to 39 | T1D | Female | 5.55 (5.18, 5.94） |
| Rwanda | 35 to 39 | T1D | Male | 5.45 (5.09, 5.83） |
| Rwanda | 40 to 44 | T1D | Both | 5.77 (5.49, 6.07） |
| Rwanda | 40 to 44 | T1D | Female | 5.85 (5.45, 6.28） |
| Rwanda | 40 to 44 | T1D | Male | 5.71 (5.32, 6.14） |
| Rwanda | 45 to 49 | T1D | Both | 5.99 (5.68, 6.32） |
| Rwanda | 45 to 49 | T1D | Female | 6.08 (5.64, 6.56） |
| Rwanda | 45 to 49 | T1D | Male | 5.91 (5.48, 6.38） |
| Rwanda | 5 to 9 | T1D | Both | 1.00 (1.00, 1.00） |
| Rwanda | 5 to 9 | T1D | Female | 1.00 (1.00, 1.00） |
| Rwanda | 5 to 9 | T1D | Male | 1.00 (1.00, 1.00） |
| Rwanda | 50 to 54 | T1D | Both | 6.16 (5.82, 6.52） |
| Rwanda | 50 to 54 | T1D | Female | 6.27 (5.79, 6.79） |
| Rwanda | 50 to 54 | T1D | Male | 6.07 (5.59, 6.58） |
| Rwanda | 55 to 59 | T1D | Both | 6.30 (5.93, 6.69） |
| Rwanda | 55 to 59 | T1D | Female | 6.42 (5.90, 6.99） |
| Rwanda | 55 to 59 | T1D | Male | 6.19 (5.67, 6.75） |
| Rwanda | 60 to 64 | T1D | Both | 6.41 (6.00, 6.85） |
| Rwanda | 60 to 64 | T1D | Female | 6.55 (5.98, 7.18） |
| Rwanda | 60 to 64 | T1D | Male | 6.29 (5.71, 6.93） |
| Rwanda | 65 to 69 | T1D | Both | 6.49 (6.03, 6.99） |
| Rwanda | 65 to 69 | T1D | Female | 6.66 (6.01, 7.37） |
| Rwanda | 65 to 69 | T1D | Male | 6.35 (5.69, 7.09） |
| Saint Kitts and Nevis | 0 to 4 | T1D | Both | 0.05 (0.01, 0.88） |
| Saint Kitts and Nevis | 0 to 4 | T1D | Female | 0.11 (0.01, 1.91） |
| Saint Kitts and Nevis | 0 to 4 | T1D | Male | 0.10 (0.01, 1.85） |
| Saint Kitts and Nevis | 10 to 14 | T1D | Both | 2.03 (0.97, 4.25） |
| Saint Kitts and Nevis | 10 to 14 | T1D | Female | 1.99 (0.70, 5.66） |
| Saint Kitts and Nevis | 10 to 14 | T1D | Male | 2.03 (0.71, 5.76） |
| Saint Kitts and Nevis | 15 to 19 | T1D | Both | 3.12 (1.51, 6.48） |

| | | | | |
|---|---|---|---|---|
| Saint Kitts and Nevis | 15 to 19 | T1D | Female | 3.12 (1.11, 8.73） |
| Saint Kitts and Nevis | 15 to 19 | T1D | Male | 2.91 (1.03, 8.21） |
| Saint Kitts and Nevis | 20 to 24 | T1D | Both | 3.92 (1.87, 8.23） |
| Saint Kitts and Nevis | 20 to 24 | T1D | Female | 3.92 (1.38, 11.09） |
| Saint Kitts and Nevis | 20 to 24 | T1D | Male | 3.89 (1.36, 11.13） |
| Saint Kitts and Nevis | 25 to 29 | T1D | Both | 4.85 (2.28, 10.30） |
| Saint Kitts and Nevis | 25 to 29 | T1D | Female | 4.41 (1.52, 12.78） |
| Saint Kitts and Nevis | 25 to 29 | T1D | Male | 4.96 (1.70, 14.43） |
| Saint Kitts and Nevis | 30 to 34 | T1D | Both | 5.79 (2.68, 12.49） |
| Saint Kitts and Nevis | 30 to 34 | T1D | Female | 4.80 (1.62, 14.26） |
| Saint Kitts and Nevis | 30 to 34 | T1D | Male | 5.96 (2.01, 17.67） |
| Saint Kitts and Nevis | 35 to 39 | T1D | Both | 6.43 (2.92, 14.19） |
| Saint Kitts and Nevis | 35 to 39 | T1D | Female | 5.48 (1.80, 16.73） |
| Saint Kitts and Nevis | 35 to 39 | T1D | Male | 7.11 (2.33, 21.71） |
| Saint Kitts and Nevis | 40 to 44 | T1D | Both | 7.44 (3.31, 16.68） |
| Saint Kitts and Nevis | 40 to 44 | T1D | Female | 6.04 (1.93, 18.92） |
| Saint Kitts and Nevis | 40 to 44 | T1D | Male | 7.85 (2.51, 24.56） |
| Saint Kitts and Nevis | 45 to 49 | T1D | Both | 8.18 (3.57, 18.76） |
| Saint Kitts and Nevis | 45 to 49 | T1D | Female | 6.16 (1.90, 19.96） |
| Saint Kitts and Nevis | 45 to 49 | T1D | Male | 8.93 (2.79, 28.65） |
| Saint Kitts and Nevis | 5 to 9 | T1D | Both | 1.00 (1.00, 1.00） |
| Saint Kitts and Nevis | 5 to 9 | T1D | Female | 1.00 (1.00, 1.00） |
| Saint Kitts and Nevis | 5 to 9 | T1D | Male | 1.00 (1.00, 1.00） |
| Saint Kitts and Nevis | 50 to 54 | T1D | Both | 9.00 (3.83, 21.18） |
| Saint Kitts and Nevis | 50 to 54 | T1D | Female | 6.92 (2.06, 23.24） |
| Saint Kitts and Nevis | 50 to 54 | T1D | Male | 10.71 (3.23, 35.51） |
| Saint Kitts and Nevis | 55 to 59 | T1D | Both | 9.63 (3.95, 23.49） |
| Saint Kitts and Nevis | 55 to 59 | T1D | Female | 7.34 (2.07, 26.02） |
| Saint Kitts and Nevis | 55 to 59 | T1D | Male | 11.30 (3.24, 39.40） |
| Saint Kitts and Nevis | 60 to 64 | T1D | Both | 10.73 (4.21, 27.32） |
| Saint Kitts and Nevis | 60 to 64 | T1D | Female | 7.89 (2.08, 29.91） |
| Saint Kitts and Nevis | 60 to 64 | T1D | Male | 11.49 (3.06, 43.09） |
| Saint Kitts and Nevis | 65 to 69 | T1D | Both | 12.19 (4.51, 32.95） |

| | | | | |
|---|---|---|---|---|
| Saint Kitts and Nevis | 65 to 69 | T1D | Female | 9.24 (2.20, 38.88） |
| Saint Kitts and Nevis | 65 to 69 | T1D | Male | 12.46 (2.98, 52.18） |
| Saint Lucia | 0 to 4 | T1D | Both | 0.16 (0.06, 0.41） |
| Saint Lucia | 0 to 4 | T1D | Female | 0.03 (0.01, 0.52） |
| Saint Lucia | 0 to 4 | T1D | Male | 0.31 (0.10, 0.91） |
| Saint Lucia | 10 to 14 | T1D | Both | 2.25 (1.50, 3.38） |
| Saint Lucia | 10 to 14 | T1D | Female | 2.44 (1.38, 4.32） |
| Saint Lucia | 10 to 14 | T1D | Male | 2.15 (1.18, 3.91） |
| Saint Lucia | 15 to 19 | T1D | Both | 3.39 (2.26, 5.06） |
| Saint Lucia | 15 to 19 | T1D | Female | 3.56 (2.01, 6.28） |
| Saint Lucia | 15 to 19 | T1D | Male | 3.38 (1.87, 6.11） |
| Saint Lucia | 20 to 24 | T1D | Both | 4.33 (2.88, 6.53） |
| Saint Lucia | 20 to 24 | T1D | Female | 4.39 (2.45, 7.84） |
| Saint Lucia | 20 to 24 | T1D | Male | 4.48 (2.46, 8.16） |
| Saint Lucia | 25 to 29 | T1D | Both | 5.30 (3.48, 8.05） |
| Saint Lucia | 25 to 29 | T1D | Female | 5.19 (2.86, 9.42） |
| Saint Lucia | 25 to 29 | T1D | Male | 5.68 (3.08, 10.48） |
| Saint Lucia | 30 to 34 | T1D | Both | 6.16 (4.01, 9.47） |
| Saint Lucia | 30 to 34 | T1D | Female | 5.80 (3.14, 10.69） |
| Saint Lucia | 30 to 34 | T1D | Male | 6.91 (3.69, 12.93） |
| Saint Lucia | 35 to 39 | T1D | Both | 7.06 (4.54, 10.97） |
| Saint Lucia | 35 to 39 | T1D | Female | 6.56 (3.50, 12.30） |
| Saint Lucia | 35 to 39 | T1D | Male | 8.07 (4.25, 15.33） |
| Saint Lucia | 40 to 44 | T1D | Both | 8.08 (5.14, 12.69） |
| Saint Lucia | 40 to 44 | T1D | Female | 7.46 (3.92, 14.19） |
| Saint Lucia | 40 to 44 | T1D | Male | 9.32 (4.84, 17.96） |
| Saint Lucia | 45 to 49 | T1D | Both | 9.02 (5.67, 14.36） |
| Saint Lucia | 45 to 49 | T1D | Female | 8.06 (4.15, 15.64） |
| Saint Lucia | 45 to 49 | T1D | Male | 10.52 (5.37, 20.62） |
| Saint Lucia | 5 to 9 | T1D | Both | 1.00 (1.00, 1.00） |
| Saint Lucia | 5 to 9 | T1D | Female | 1.00 (1.00, 1.00） |
| Saint Lucia | 5 to 9 | T1D | Male | 1.00 (1.00, 1.00） |
| Saint Lucia | 50 to 54 | T1D | Both | 9.96 (6.15, 16.13） |

| | | | | |
|---|---|---|---|---|
| Saint Lucia | 50 to 54 | T1D | Female | 8.74 (4.38, 17.42） |
| Saint Lucia | 50 to 54 | T1D | Male | 11.95 (5.97, 23.94） |
| Saint Lucia | 55 to 59 | T1D | Both | 10.83 (6.54, 17.93） |
| Saint Lucia | 55 to 59 | T1D | Female | 9.88 (4.81, 20.27） |
| Saint Lucia | 55 to 59 | T1D | Male | 13.06 (6.32, 27.01） |
| Saint Lucia | 60 to 64 | T1D | Both | 11.80 (6.95, 20.04） |
| Saint Lucia | 60 to 64 | T1D | Female | 10.22 (4.80, 21.75） |
| Saint Lucia | 60 to 64 | T1D | Male | 14.40 (6.75, 30.75） |
| Saint Lucia | 65 to 69 | T1D | Both | 12.98 (7.43, 22.67） |
| Saint Lucia | 65 to 69 | T1D | Female | 10.99 (4.96, 24.38） |
| Saint Lucia | 65 to 69 | T1D | Male | 16.40 (7.40, 36.30） |
| Saint Vincent and the Grenadines | 0 to 4 | T1D | Both | 0.21 (0.08, 0.56） |
| Saint Vincent and the Grenadines | 0 to 4 | T1D | Female | 0.05 (0.01, 0.78） |
| Saint Vincent and the Grenadines | 0 to 4 | T1D | Male | 0.05 (0.01, 0.84） |
| Saint Vincent and the Grenadines | 10 to 14 | T1D | Both | 2.18 (1.36, 3.49） |
| Saint Vincent and the Grenadines | 10 to 14 | T1D | Female | 2.50 (1.26, 4.97） |
| Saint Vincent and the Grenadines | 10 to 14 | T1D | Male | 2.51 (1.22, 5.17） |
| Saint Vincent and the Grenadines | 15 to 19 | T1D | Both | 3.26 (2.04, 5.21） |
| Saint Vincent and the Grenadines | 15 to 19 | T1D | Female | 3.63 (1.83, 7.22） |
| Saint Vincent and the Grenadines | 15 to 19 | T1D | Male | 3.50 (1.69, 7.23） |
| Saint Vincent and the Grenadines | 20 to 24 | T1D | Both | 4.18 (2.59, 6.73） |
| Saint Vincent and the Grenadines | 20 to 24 | T1D | Female | 4.45 (2.21, 8.97） |
| Saint Vincent and the Grenadines | 20 to 24 | T1D | Male | 4.84 (2.32, 10.10） |
| Saint Vincent and the Grenadines | 25 to 29 | T1D | Both | 5.15 (3.16, 8.39） |
| Saint Vincent and the Grenadines | 25 to 29 | T1D | Female | 5.30 (2.60, 10.84） |
| Saint Vincent and the Grenadines | 25 to 29 | T1D | Male | 6.37 (3.01, 13.45） |
| Saint Vincent and the Grenadines | 30 to 34 | T1D | Both | 5.99 (3.63, 9.89） |
| Saint Vincent and the Grenadines | 30 to 34 | T1D | Female | 5.89 (2.83, 12.26） |
| Saint Vincent and the Grenadines | 30 to 34 | T1D | Male | 7.53 (3.50, 16.19） |
| Saint Vincent and the Grenadines | 35 to 39 | T1D | Both | 6.90 (4.12, 11.55） |
| Saint Vincent and the Grenadines | 35 to 39 | T1D | Female | 6.58 (3.09, 14.03） |
| Saint Vincent and the Grenadines | 35 to 39 | T1D | Male | 8.73 (3.99, 19.10） |
| Saint Vincent and the Grenadines | 40 to 44 | T1D | Both | 7.67 (4.52, 13.02） |

| | | | | |
|---|---|---|---|---|
| Saint Vincent and the Grenadines | 40 to 44 | T1D | Female | 7.44 (3.42, 16.18） |
| Saint Vincent and the Grenadines | 40 to 44 | T1D | Male | 9.73 (4.38, 21.63） |
| Saint Vincent and the Grenadines | 45 to 49 | T1D | Both | 8.58 (4.97, 14.83） |
| Saint Vincent and the Grenadines | 45 to 49 | T1D | Female | 8.15 (3.65, 18.21） |
| Saint Vincent and the Grenadines | 45 to 49 | T1D | Male | 11.48 (5.06, 26.02） |
| Saint Vincent and the Grenadines | 5 to 9 | T1D | Both | 1.00 (1.00, 1.00） |
| Saint Vincent and the Grenadines | 5 to 9 | T1D | Female | 1.00 (1.00, 1.00） |
| Saint Vincent and the Grenadines | 5 to 9 | T1D | Male | 1.00 (1.00, 1.00） |
| Saint Vincent and the Grenadines | 50 to 54 | T1D | Both | 9.40 (5.33, 16.58） |
| Saint Vincent and the Grenadines | 50 to 54 | T1D | Female | 8.75 (3.79, 20.21） |
| Saint Vincent and the Grenadines | 50 to 54 | T1D | Male | 12.12 (5.20, 28.22） |
| Saint Vincent and the Grenadines | 55 to 59 | T1D | Both | 10.42 (5.77, 18.83） |
| Saint Vincent and the Grenadines | 55 to 59 | T1D | Female | 9.57 (3.99, 22.92） |
| Saint Vincent and the Grenadines | 55 to 59 | T1D | Male | 13.15 (5.47, 31.62） |
| Saint Vincent and the Grenadines | 60 to 64 | T1D | Both | 11.12 (5.95, 20.76） |
| Saint Vincent and the Grenadines | 60 to 64 | T1D | Female | 10.81 (4.32, 27.02） |
| Saint Vincent and the Grenadines | 60 to 64 | T1D | Male | 14.26 (5.69, 35.74） |
| Saint Vincent and the Grenadines | 65 to 69 | T1D | Both | 12.24 (6.31, 23.77） |
| Saint Vincent and the Grenadines | 65 to 69 | T1D | Female | 11.75 (4.45, 31.02） |
| Saint Vincent and the Grenadines | 65 to 69 | T1D | Male | 16.66 (6.36, 43.64） |
| Samoa | 0 to 4 | T1D | Both | 0.21 (0.08, 0.52） |
| Samoa | 0 to 4 | T1D | Female | 0.04 (0.01, 0.66） |
| Samoa | 0 to 4 | T1D | Male | 0.04 (0.01, 0.68） |
| Samoa | 10 to 14 | T1D | Both | 2.00 (1.30, 3.08） |
| Samoa | 10 to 14 | T1D | Female | 1.96 (1.06, 3.62） |
| Samoa | 10 to 14 | T1D | Male | 2.02 (1.10, 3.72） |
| Samoa | 15 to 19 | T1D | Both | 2.92 (1.89, 4.52） |
| Samoa | 15 to 19 | T1D | Female | 2.77 (1.49, 5.16） |
| Samoa | 15 to 19 | T1D | Male | 3.02 (1.64, 5.57） |
| Samoa | 20 to 24 | T1D | Both | 3.74 (2.37, 5.89） |
| Samoa | 20 to 24 | T1D | Female | 3.55 (1.86, 6.75） |
| Samoa | 20 to 24 | T1D | Male | 4.05 (2.15, 7.62） |
| Samoa | 25 to 29 | T1D | Both | 4.57 (2.86, 7.31） |

| | | | | |
|---|---|---|---|---|
| Samoa | 25 to 29 | T1D | Female | 4.24 (2.18, 8.24） |
| Samoa | 25 to 29 | T1D | Male | 5.05 (2.64, 9.66） |
| Samoa | 30 to 34 | T1D | Both | 5.38 (3.31, 8.74） |
| Samoa | 30 to 34 | T1D | Female | 4.66 (2.33, 9.32） |
| Samoa | 30 to 34 | T1D | Male | 6.05 (3.10, 11.81） |
| Samoa | 35 to 39 | T1D | Both | 6.04 (3.64, 10.02） |
| Samoa | 35 to 39 | T1D | Female | 5.26 (2.54, 10.88） |
| Samoa | 35 to 39 | T1D | Male | 6.69 (3.33, 13.43） |
| Samoa | 40 to 44 | T1D | Both | 6.73 (3.98, 11.37） |
| Samoa | 40 to 44 | T1D | Female | 5.55 (2.60, 11.88） |
| Samoa | 40 to 44 | T1D | Male | 7.34 (3.57, 15.10） |
| Samoa | 45 to 49 | T1D | Both | 7.43 (4.30, 12.83） |
| Samoa | 45 to 49 | T1D | Female | 6.04 (2.72, 13.40） |
| Samoa | 45 to 49 | T1D | Male | 8.33 (3.95, 17.57） |
| Samoa | 5 to 9 | T1D | Both | 1.00 (1.00, 1.00） |
| Samoa | 5 to 9 | T1D | Female | 1.00 (1.00, 1.00） |
| Samoa | 5 to 9 | T1D | Male | 1.00 (1.00, 1.00） |
| Samoa | 50 to 54 | T1D | Both | 8.25 (4.64, 14.64） |
| Samoa | 50 to 54 | T1D | Female | 6.84 (2.97, 15.79） |
| Samoa | 50 to 54 | T1D | Male | 8.89 (4.05, 19.52） |
| Samoa | 55 to 59 | T1D | Both | 8.87 (4.83, 16.28） |
| Samoa | 55 to 59 | T1D | Female | 7.28 (3.01, 17.63） |
| Samoa | 55 to 59 | T1D | Male | 9.59 (4.17, 22.05） |
| Samoa | 60 to 64 | T1D | Both | 9.38 (4.88, 18.01） |
| Samoa | 60 to 64 | T1D | Female | 7.72 (2.99, 19.92） |
| Samoa | 60 to 64 | T1D | Male | 10.79 (4.43, 26.27） |
| Samoa | 65 to 69 | T1D | Both | 10.21 (5.01, 20.84） |
| Samoa | 65 to 69 | T1D | Female | 7.91 (2.78, 22.50） |
| Samoa | 65 to 69 | T1D | Male | 10.45 (3.81, 28.61） |
| San Marino | 0 to 4 | T1D | Both | 0.31 (0.09, 1.07） |
| San Marino | 0 to 4 | T1D | Female | 0.10 (0.01, 1.82） |
| San Marino | 0 to 4 | T1D | Male | 0.08 (0.01, 1.33） |
| San Marino | 10 to 14 | T1D | Both | 2.06 (1.06, 4.00） |

| | | | | |
|---|---|---|---|---|
| San Marino | 10 to 14 | T1D | Female | 2.41 (0.88, 6.60） |
| San Marino | 10 to 14 | T1D | Male | 2.05 (0.79, 5.31） |
| San Marino | 15 to 19 | T1D | Both | 3.15 (1.62, 6.11） |
| San Marino | 15 to 19 | T1D | Female | 3.55 (1.31, 9.62） |
| San Marino | 15 to 19 | T1D | Male | 3.25 (1.24, 8.55） |
| San Marino | 20 to 24 | T1D | Both | 4.12 (2.10, 8.09） |
| San Marino | 20 to 24 | T1D | Female | 4.73 (1.74, 12.86） |
| San Marino | 20 to 24 | T1D | Male | 4.37 (1.65, 11.53） |
| San Marino | 25 to 29 | T1D | Both | 5.20 (2.63, 10.27） |
| San Marino | 25 to 29 | T1D | Female | 5.69 (2.07, 15.66） |
| San Marino | 25 to 29 | T1D | Male | 5.75 (2.15, 15.35） |
| San Marino | 30 to 34 | T1D | Both | 6.31 (3.16, 12.60） |
| San Marino | 30 to 34 | T1D | Female | 7.27 (2.61, 20.25） |
| San Marino | 30 to 34 | T1D | Male | 6.72 (2.47, 18.26） |
| San Marino | 35 to 39 | T1D | Both | 7.35 (3.64, 14.86） |
| San Marino | 35 to 39 | T1D | Female | 8.33 (2.93, 23.66） |
| San Marino | 35 to 39 | T1D | Male | 8.18 (2.97, 22.54） |
| San Marino | 40 to 44 | T1D | Both | 8.60 (4.21, 17.55） |
| San Marino | 40 to 44 | T1D | Female | 9.81 (3.40, 28.26） |
| San Marino | 40 to 44 | T1D | Male | 9.12 (3.27, 25.48） |
| San Marino | 45 to 49 | T1D | Both | 9.91 (4.80, 20.46） |
| San Marino | 45 to 49 | T1D | Female | 11.43 (3.90, 33.51） |
| San Marino | 45 to 49 | T1D | Male | 10.82 (3.82, 30.62） |
| San Marino | 5 to 9 | T1D | Both | 1.00 (1.00, 1.00） |
| San Marino | 5 to 9 | T1D | Female | 1.00 (1.00, 1.00） |
| San Marino | 5 to 9 | T1D | Male | 1.00 (1.00, 1.00） |
| San Marino | 50 to 54 | T1D | Both | 11.31 (5.40, 23.68） |
| San Marino | 50 to 54 | T1D | Female | 12.86 (4.29, 38.55） |
| San Marino | 50 to 54 | T1D | Male | 12.12 (4.21, 34.93） |
| San Marino | 55 to 59 | T1D | Both | 12.63 (5.94, 26.85） |
| San Marino | 55 to 59 | T1D | Female | 14.50 (4.73, 44.47） |
| San Marino | 55 to 59 | T1D | Male | 13.62 (4.64, 39.96） |
| San Marino | 60 to 64 | T1D | Both | 14.26 (6.62, 30.73） |

| | | | | |
|---|---|---|---|---|
| San Marino | 60 to 64 | T1D | Female | 15.45 (4.93, 48.49） |
| San Marino | 60 to 64 | T1D | Male | 15.34 (5.14, 45.79） |
| San Marino | 65 to 69 | T1D | Both | 15.83 (7.23, 34.63） |
| San Marino | 65 to 69 | T1D | Female | 17.45 (5.46, 55.78） |
| San Marino | 65 to 69 | T1D | Male | 18.09 (5.94, 55.05） |
| Sao Tome and Principe | 0 to 4 | T1D | Both | 0.16 (0.08, 0.31） |
| Sao Tome and Principe | 0 to 4 | T1D | Female | 0.16 (0.06, 0.42） |
| Sao Tome and Principe | 0 to 4 | T1D | Male | 0.16 (0.06, 0.40） |
| Sao Tome and Principe | 10 to 14 | T1D | Both | 2.06 (1.51, 2.81） |
| Sao Tome and Principe | 10 to 14 | T1D | Female | 2.03 (1.31, 3.16） |
| Sao Tome and Principe | 10 to 14 | T1D | Male | 2.08 (1.35, 3.21） |
| Sao Tome and Principe | 15 to 19 | T1D | Both | 3.00 (2.19, 4.10） |
| Sao Tome and Principe | 15 to 19 | T1D | Female | 3.01 (1.92, 4.71） |
| Sao Tome and Principe | 15 to 19 | T1D | Male | 3.05 (1.96, 4.73） |
| Sao Tome and Principe | 20 to 24 | T1D | Both | 3.88 (2.81, 5.36） |
| Sao Tome and Principe | 20 to 24 | T1D | Female | 3.85 (2.42, 6.10） |
| Sao Tome and Principe | 20 to 24 | T1D | Male | 3.87 (2.46, 6.08） |
| Sao Tome and Principe | 25 to 29 | T1D | Both | 4.82 (3.46, 6.72） |
| Sao Tome and Principe | 25 to 29 | T1D | Female | 4.80 (2.99, 7.71） |
| Sao Tome and Principe | 25 to 29 | T1D | Male | 4.86 (3.05, 7.73） |
| Sao Tome and Principe | 30 to 34 | T1D | Both | 5.50 (3.90, 7.77） |
| Sao Tome and Principe | 30 to 34 | T1D | Female | 5.39 (3.29, 8.83） |
| Sao Tome and Principe | 30 to 34 | T1D | Male | 5.43 (3.35, 8.82） |
| Sao Tome and Principe | 35 to 39 | T1D | Both | 6.00 (4.17, 8.63） |
| Sao Tome and Principe | 35 to 39 | T1D | Female | 6.06 (3.61, 10.18） |
| Sao Tome and Principe | 35 to 39 | T1D | Male | 5.94 (3.57, 9.89） |
| Sao Tome and Principe | 40 to 44 | T1D | Both | 6.48 (4.43, 9.50） |
| Sao Tome and Principe | 40 to 44 | T1D | Female | 6.39 (3.71, 11.02） |
| Sao Tome and Principe | 40 to 44 | T1D | Male | 6.36 (3.72, 10.86） |
| Sao Tome and Principe | 45 to 49 | T1D | Both | 6.97 (4.66, 10.43） |
| Sao Tome and Principe | 45 to 49 | T1D | Female | 6.89 (3.88, 12.23） |
| Sao Tome and Principe | 45 to 49 | T1D | Male | 6.93 (3.94, 12.19） |
| Sao Tome and Principe | 5 to 9 | T1D | Both | 1.00 (1.00, 1.00） |

| | | | | |
|---|---|---|---|---|
| Sao Tome and Principe | 5 to 9 | T1D | Female | 1.00 (1.00, 1.00） |
| Sao Tome and Principe | 5 to 9 | T1D | Male | 1.00 (1.00, 1.00） |
| Sao Tome and Principe | 50 to 54 | T1D | Both | 7.31 (4.76, 11.24） |
| Sao Tome and Principe | 50 to 54 | T1D | Female | 7.17 (3.89, 13.21） |
| Sao Tome and Principe | 50 to 54 | T1D | Male | 7.29 (3.98, 13.33） |
| Sao Tome and Principe | 55 to 59 | T1D | Both | 7.64 (4.82, 12.09） |
| Sao Tome and Principe | 55 to 59 | T1D | Female | 7.54 (3.92, 14.48） |
| Sao Tome and Principe | 55 to 59 | T1D | Male | 7.36 (3.84, 14.10） |
| Sao Tome and Principe | 60 to 64 | T1D | Both | 7.90 (4.82, 12.96） |
| Sao Tome and Principe | 60 to 64 | T1D | Female | 7.64 (3.79, 15.43） |
| Sao Tome and Principe | 60 to 64 | T1D | Male | 7.74 (3.84, 15.59） |
| Sao Tome and Principe | 65 to 69 | T1D | Both | 8.12 (4.71, 13.98） |
| Sao Tome and Principe | 65 to 69 | T1D | Female | 8.70 (4.07, 18.57） |
| Sao Tome and Principe | 65 to 69 | T1D | Male | 8.73 (4.09, 18.67） |
| Saudi Arabia | 0 to 4 | T1D | Both | 0.17 (0.15, 0.20） |
| Saudi Arabia | 0 to 4 | T1D | Female | 0.19 (0.16, 0.22） |
| Saudi Arabia | 0 to 4 | T1D | Male | 0.16 (0.14, 0.18） |
| Saudi Arabia | 10 to 14 | T1D | Both | 2.30 (2.14, 2.47） |
| Saudi Arabia | 10 to 14 | T1D | Female | 2.33 (2.15, 2.52） |
| Saudi Arabia | 10 to 14 | T1D | Male | 2.27 (2.14, 2.42） |
| Saudi Arabia | 15 to 19 | T1D | Both | 3.40 (3.17, 3.65） |
| Saudi Arabia | 15 to 19 | T1D | Female | 3.40 (3.14, 3.69） |
| Saudi Arabia | 15 to 19 | T1D | Male | 3.41 (3.21, 3.63） |
| Saudi Arabia | 20 to 24 | T1D | Both | 4.25 (3.96, 4.57） |
| Saudi Arabia | 20 to 24 | T1D | Female | 4.15 (3.82, 4.51） |
| Saudi Arabia | 20 to 24 | T1D | Male | 4.38 (4.11, 4.66） |
| Saudi Arabia | 25 to 29 | T1D | Both | 5.29 (4.91, 5.69） |
| Saudi Arabia | 25 to 29 | T1D | Female | 5.07 (4.65, 5.52） |
| Saudi Arabia | 25 to 29 | T1D | Male | 5.54 (5.19, 5.91） |
| Saudi Arabia | 30 to 34 | T1D | Both | 6.46 (5.99, 6.97） |
| Saudi Arabia | 30 to 34 | T1D | Female | 6.09 (5.57, 6.66） |
| Saudi Arabia | 30 to 34 | T1D | Male | 6.85 (6.41, 7.32） |
| Saudi Arabia | 35 to 39 | T1D | Both | 7.81 (7.22, 8.45） |

| | | | | |
|---|---|---|---|---|
| Saudi Arabia | 35 to 39 | T1D | Female | 7.27 (6.62, 7.98） |
| Saudi Arabia | 35 to 39 | T1D | Male | 8.36 (7.80, 8.95） |
| Saudi Arabia | 40 to 44 | T1D | Both | 9.40 (8.66, 10.20） |
| Saudi Arabia | 40 to 44 | T1D | Female | 8.65 (7.84, 9.54） |
| Saudi Arabia | 40 to 44 | T1D | Male | 10.12 (9.42, 10.86） |
| Saudi Arabia | 45 to 49 | T1D | Both | 11.23 (10.31, 12.25） |
| Saudi Arabia | 45 to 49 | T1D | Female | 10.27 (9.25, 11.39） |
| Saudi Arabia | 45 to 49 | T1D | Male | 12.14 (11.28, 13.08） |
| Saudi Arabia | 5 to 9 | T1D | Both | 1.00 (1.00, 1.00） |
| Saudi Arabia | 5 to 9 | T1D | Female | 1.00 (1.00, 1.00） |
| Saudi Arabia | 5 to 9 | T1D | Male | 1.00 (1.00, 1.00） |
| Saudi Arabia | 50 to 54 | T1D | Both | 13.35 (12.18, 14.63） |
| Saudi Arabia | 50 to 54 | T1D | Female | 12.17 (10.89, 13.60） |
| Saudi Arabia | 50 to 54 | T1D | Male | 14.46 (13.37, 15.63） |
| Saudi Arabia | 55 to 59 | T1D | Both | 15.80 (14.33, 17.43） |
| Saudi Arabia | 55 to 59 | T1D | Female | 14.39 (12.77, 16.22） |
| Saudi Arabia | 55 to 59 | T1D | Male | 17.12 (15.76, 18.60） |
| Saudi Arabia | 60 to 64 | T1D | Both | 18.62 (16.74, 20.70） |
| Saudi Arabia | 60 to 64 | T1D | Female | 16.95 (14.87, 19.31） |
| Saudi Arabia | 60 to 64 | T1D | Male | 20.16 (18.43, 22.05） |
| Saudi Arabia | 65 to 69 | T1D | Both | 21.68 (19.27, 24.38） |
| Saudi Arabia | 65 to 69 | T1D | Female | 19.71 (17.04, 22.80） |
| Saudi Arabia | 65 to 69 | T1D | Male | 23.48 (21.28, 25.92） |
| Senegal | 0 to 4 | T1D | Both | 0.16 (0.14, 0.17） |
| Senegal | 0 to 4 | T1D | Female | 0.16 (0.14, 0.18） |
| Senegal | 0 to 4 | T1D | Male | 0.16 (0.14, 0.17） |
| Senegal | 10 to 14 | T1D | Both | 2.08 (2.01, 2.17） |
| Senegal | 10 to 14 | T1D | Female | 2.09 (1.98, 2.21） |
| Senegal | 10 to 14 | T1D | Male | 2.08 (1.98, 2.20） |
| Senegal | 15 to 19 | T1D | Both | 3.03 (2.92, 3.15） |
| Senegal | 15 to 19 | T1D | Female | 3.04 (2.88, 3.22） |
| Senegal | 15 to 19 | T1D | Male | 3.02 (2.86, 3.19） |
| Senegal | 20 to 24 | T1D | Both | 3.85 (3.70, 4.00） |

| | | | | |
|---|---|---|---|---|
| Senegal | 20 to 24 | T1D | Female | 3.87 (3.66, 4.10） |
| Senegal | 20 to 24 | T1D | Male | 3.83 (3.63, 4.05） |
| Senegal | 25 to 29 | T1D | Both | 4.68 (4.50, 4.88） |
| Senegal | 25 to 29 | T1D | Female | 4.72 (4.46, 5.01） |
| Senegal | 25 to 29 | T1D | Male | 4.66 (4.40, 4.93） |
| Senegal | 30 to 34 | T1D | Both | 5.22 (5.00, 5.45） |
| Senegal | 30 to 34 | T1D | Female | 5.27 (4.96, 5.60） |
| Senegal | 30 to 34 | T1D | Male | 5.18 (4.88, 5.50） |
| Senegal | 35 to 39 | T1D | Both | 5.61 (5.37, 5.87） |
| Senegal | 35 to 39 | T1D | Female | 5.68 (5.32, 6.05） |
| Senegal | 35 to 39 | T1D | Male | 5.56 (5.22, 5.92） |
| Senegal | 40 to 44 | T1D | Both | 5.93 (5.66, 6.22） |
| Senegal | 40 to 44 | T1D | Female | 6.01 (5.62, 6.43） |
| Senegal | 40 to 44 | T1D | Male | 5.87 (5.49, 6.27） |
| Senegal | 45 to 49 | T1D | Both | 6.20 (5.90, 6.52） |
| Senegal | 45 to 49 | T1D | Female | 6.29 (5.86, 6.75） |
| Senegal | 45 to 49 | T1D | Male | 6.12 (5.70, 6.56） |
| Senegal | 5 to 9 | T1D | Both | 1.00 (1.00, 1.00） |
| Senegal | 5 to 9 | T1D | Female | 1.00 (1.00, 1.00） |
| Senegal | 5 to 9 | T1D | Male | 1.00 (1.00, 1.00） |
| Senegal | 50 to 54 | T1D | Both | 6.41 (6.08, 6.76） |
| Senegal | 50 to 54 | T1D | Female | 6.52 (6.05, 7.03） |
| Senegal | 50 to 54 | T1D | Male | 6.32 (5.86, 6.81） |
| Senegal | 55 to 59 | T1D | Both | 6.57 (6.21, 6.96） |
| Senegal | 55 to 59 | T1D | Female | 6.69 (6.18, 7.25） |
| Senegal | 55 to 59 | T1D | Male | 6.45 (5.96, 6.99） |
| Senegal | 60 to 64 | T1D | Both | 6.68 (6.28, 7.10） |
| Senegal | 60 to 64 | T1D | Female | 6.82 (6.25, 7.44） |
| Senegal | 60 to 64 | T1D | Male | 6.55 (6.01, 7.14） |
| Senegal | 65 to 69 | T1D | Both | 6.75 (6.31, 7.23） |
| Senegal | 65 to 69 | T1D | Female | 6.92 (6.29, 7.61） |
| Senegal | 65 to 69 | T1D | Male | 6.61 (6.01, 7.27） |
| Serbia | 0 to 4 | T1D | Both | 0.21 (0.19, 0.23） |

| | | | | |
|---|---|---|---|---|
| Serbia | 0 to 4 | T1D | Female | 0.20 (0.17, 0.23） |
| Serbia | 0 to 4 | T1D | Male | 0.22 (0.19, 0.25） |
| Serbia | 10 to 14 | T1D | Both | 2.23 (2.12, 2.34） |
| Serbia | 10 to 14 | T1D | Female | 2.25 (2.10, 2.42） |
| Serbia | 10 to 14 | T1D | Male | 2.20 (2.05, 2.36） |
| Serbia | 15 to 19 | T1D | Both | 3.48 (3.32, 3.66） |
| Serbia | 15 to 19 | T1D | Female | 3.42 (3.19, 3.66） |
| Serbia | 15 to 19 | T1D | Male | 3.55 (3.31, 3.80） |
| Serbia | 20 to 24 | T1D | Both | 4.55 (4.34, 4.78） |
| Serbia | 20 to 24 | T1D | Female | 4.32 (4.03, 4.63） |
| Serbia | 20 to 24 | T1D | Male | 4.78 (4.46, 5.13） |
| Serbia | 25 to 29 | T1D | Both | 5.71 (5.43, 6.00） |
| Serbia | 25 to 29 | T1D | Female | 5.28 (4.92, 5.66） |
| Serbia | 25 to 29 | T1D | Male | 6.14 (5.72, 6.58） |
| Serbia | 30 to 34 | T1D | Both | 6.83 (6.49, 7.18） |
| Serbia | 30 to 34 | T1D | Female | 6.20 (5.77, 6.66） |
| Serbia | 30 to 34 | T1D | Male | 7.46 (6.95, 8.02） |
| Serbia | 35 to 39 | T1D | Both | 8.00 (7.60, 8.43） |
| Serbia | 35 to 39 | T1D | Female | 7.17 (6.66, 7.71） |
| Serbia | 35 to 39 | T1D | Male | 8.85 (8.23, 9.52） |
| Serbia | 40 to 44 | T1D | Both | 9.28 (8.81, 9.78） |
| Serbia | 40 to 44 | T1D | Female | 8.23 (7.64, 8.87） |
| Serbia | 40 to 44 | T1D | Male | 10.34 (9.61, 11.13） |
| Serbia | 45 to 49 | T1D | Both | 10.64 (10.09, 11.22） |
| Serbia | 45 to 49 | T1D | Female | 9.40 (8.71, 10.14） |
| Serbia | 45 to 49 | T1D | Male | 11.90 (11.05, 12.82） |
| Serbia | 5 to 9 | T1D | Both | 1.00 (1.00, 1.00） |
| Serbia | 5 to 9 | T1D | Female | 1.00 (1.00, 1.00） |
| Serbia | 5 to 9 | T1D | Male | 1.00 (1.00, 1.00） |
| Serbia | 50 to 54 | T1D | Both | 12.08 (11.44, 12.74） |
| Serbia | 50 to 54 | T1D | Female | 10.68 (9.89, 11.54） |
| Serbia | 50 to 54 | T1D | Male | 13.53 (12.55, 14.59） |
| Serbia | 55 to 59 | T1D | Both | 13.63 (12.91, 14.40） |

| | | | | |
|---|---|---|---|---|
| Serbia | 55 to 59 | T1D | Female | 12.11 (11.20, 13.10） |
| Serbia | 55 to 59 | T1D | Male | 15.28 (14.15, 16.49） |
| Serbia | 60 to 64 | T1D | Both | 15.31 (14.49, 16.19） |
| Serbia | 60 to 64 | T1D | Female | 13.70 (12.66, 14.83） |
| Serbia | 60 to 64 | T1D | Male | 17.13 (15.85, 18.51） |
| Serbia | 65 to 69 | T1D | Both | 17.13 (16.19, 18.13） |
| Serbia | 65 to 69 | T1D | Female | 15.49 (14.29, 16.80） |
| Serbia | 65 to 69 | T1D | Male | 19.13 (17.68, 20.71） |
| Seychelles | 0 to 4 | T1D | Both | 0.05 (0.01, 0.80） |
| Seychelles | 0 to 4 | T1D | Female | 0.10 (0.01, 1.71） |
| Seychelles | 0 to 4 | T1D | Male | 0.09 (0.01, 1.68） |
| Seychelles | 10 to 14 | T1D | Both | 1.70 (0.80, 3.65） |
| Seychelles | 10 to 14 | T1D | Female | 2.03 (0.71, 5.77） |
| Seychelles | 10 to 14 | T1D | Male | 2.05 (0.72, 5.84） |
| Seychelles | 15 to 19 | T1D | Both | 2.62 (1.24, 5.53） |
| Seychelles | 15 to 19 | T1D | Female | 2.09 (0.71, 6.17） |
| Seychelles | 15 to 19 | T1D | Male | 2.63 (0.93, 7.49） |
| Seychelles | 20 to 24 | T1D | Both | 3.34 (1.57, 7.10） |
| Seychelles | 20 to 24 | T1D | Female | 3.22 (1.10, 9.37） |
| Seychelles | 20 to 24 | T1D | Male | 3.60 (1.26, 10.28） |
| Seychelles | 25 to 29 | T1D | Both | 4.05 (1.88, 8.72） |
| Seychelles | 25 to 29 | T1D | Female | 3.30 (1.10, 9.96） |
| Seychelles | 25 to 29 | T1D | Male | 4.45 (1.54, 12.83） |
| Seychelles | 30 to 34 | T1D | Both | 4.65 (2.12, 10.20） |
| Seychelles | 30 to 34 | T1D | Female | 4.19 (1.37, 12.77） |
| Seychelles | 30 to 34 | T1D | Male | 5.15 (1.75, 15.19） |
| Seychelles | 35 to 39 | T1D | Both | 5.27 (2.35, 11.80） |
| Seychelles | 35 to 39 | T1D | Female | 4.40 (1.39, 13.90） |
| Seychelles | 35 to 39 | T1D | Male | 5.74 (1.89, 17.47） |
| Seychelles | 40 to 44 | T1D | Both | 5.52 (2.41, 12.64） |
| Seychelles | 40 to 44 | T1D | Female | 4.36 (1.33, 14.27） |
| Seychelles | 40 to 44 | T1D | Male | 6.49 (2.08, 20.24） |
| Seychelles | 45 to 49 | T1D | Both | 6.25 (2.67, 14.65） |

| | | | | |
|---|---|---|---|---|
| Seychelles | 45 to 49 | T1D | Female | 4.99 (1.47, 16.91） |
| Seychelles | 45 to 49 | T1D | Male | 6.93 (2.15, 22.38） |
| Seychelles | 5 to 9 | T1D | Both | 1.00 (1.00, 1.00） |
| Seychelles | 5 to 9 | T1D | Female | 1.00 (1.00, 1.00） |
| Seychelles | 5 to 9 | T1D | Male | 1.00 (1.00, 1.00） |
| Seychelles | 50 to 54 | T1D | Both | 6.90 (2.86, 16.66） |
| Seychelles | 50 to 54 | T1D | Female | 5.87 (1.66, 20.82） |
| Seychelles | 50 to 54 | T1D | Male | 7.11 (2.10, 24.09） |
| Seychelles | 55 to 59 | T1D | Both | 7.12 (2.82, 17.95） |
| Seychelles | 55 to 59 | T1D | Female | 6.81 (1.81, 25.60） |
| Seychelles | 55 to 59 | T1D | Male | 7.39 (2.05, 26.59） |
| Seychelles | 60 to 64 | T1D | Both | 7.57 (2.85, 20.11） |
| Seychelles | 60 to 64 | T1D | Female | 7.24 (1.77, 29.65） |
| Seychelles | 60 to 64 | T1D | Male | 7.92 (2.04, 30.78） |
| Seychelles | 65 to 69 | T1D | Both | 8.27 (2.93, 23.40） |
| Seychelles | 65 to 69 | T1D | Female | 7.77 (1.71, 35.23） |
| Seychelles | 65 to 69 | T1D | Male | 9.11 (2.12, 39.14） |
| Sierra Leone | 0 to 4 | T1D | Both | 0.15 (0.14, 0.17） |
| Sierra Leone | 0 to 4 | T1D | Female | 0.15 (0.13, 0.18） |
| Sierra Leone | 0 to 4 | T1D | Male | 0.15 (0.13, 0.18） |
| Sierra Leone | 10 to 14 | T1D | Both | 2.09 (1.98, 2.21） |
| Sierra Leone | 10 to 14 | T1D | Female | 2.09 (1.93, 2.26） |
| Sierra Leone | 10 to 14 | T1D | Male | 2.09 (1.94, 2.26） |
| Sierra Leone | 15 to 19 | T1D | Both | 3.04 (2.87, 3.21） |
| Sierra Leone | 15 to 19 | T1D | Female | 3.04 (2.81, 3.30） |
| Sierra Leone | 15 to 19 | T1D | Male | 3.04 (2.81, 3.29） |
| Sierra Leone | 20 to 24 | T1D | Both | 3.85 (3.63, 4.08） |
| Sierra Leone | 20 to 24 | T1D | Female | 3.86 (3.56, 4.19） |
| Sierra Leone | 20 to 24 | T1D | Male | 3.85 (3.55, 4.17） |
| Sierra Leone | 25 to 29 | T1D | Both | 4.67 (4.40, 4.96） |
| Sierra Leone | 25 to 29 | T1D | Female | 4.70 (4.32, 5.12） |
| Sierra Leone | 25 to 29 | T1D | Male | 4.66 (4.29, 5.08） |
| Sierra Leone | 30 to 34 | T1D | Both | 5.19 (4.88, 5.53） |

| | | | | |
|---|---|---|---|---|
| Sierra Leone | 30 to 34 | T1D | Female | 5.23 (4.79, 5.71） |
| Sierra Leone | 30 to 34 | T1D | Male | 5.17 (4.73, 5.65） |
| Sierra Leone | 35 to 39 | T1D | Both | 5.56 (5.20, 5.94） |
| Sierra Leone | 35 to 39 | T1D | Female | 5.60 (5.10, 6.14） |
| Sierra Leone | 35 to 39 | T1D | Male | 5.53 (5.04, 6.06） |
| Sierra Leone | 40 to 44 | T1D | Both | 5.85 (5.46, 6.27） |
| Sierra Leone | 40 to 44 | T1D | Female | 5.89 (5.34, 6.50） |
| Sierra Leone | 40 to 44 | T1D | Male | 5.80 (5.26, 6.39） |
| Sierra Leone | 45 to 49 | T1D | Both | 6.09 (5.66, 6.55） |
| Sierra Leone | 45 to 49 | T1D | Female | 6.14 (5.54, 6.81） |
| Sierra Leone | 45 to 49 | T1D | Male | 6.03 (5.44, 6.68） |
| Sierra Leone | 5 to 9 | T1D | Both | 1.00 (1.00, 1.00） |
| Sierra Leone | 5 to 9 | T1D | Female | 1.00 (1.00, 1.00） |
| Sierra Leone | 5 to 9 | T1D | Male | 1.00 (1.00, 1.00） |
| Sierra Leone | 50 to 54 | T1D | Both | 6.26 (5.79, 6.77） |
| Sierra Leone | 50 to 54 | T1D | Female | 6.33 (5.66, 7.08） |
| Sierra Leone | 50 to 54 | T1D | Male | 6.17 (5.53, 6.89） |
| Sierra Leone | 55 to 59 | T1D | Both | 6.37 (5.86, 6.93） |
| Sierra Leone | 55 to 59 | T1D | Female | 6.46 (5.74, 7.28） |
| Sierra Leone | 55 to 59 | T1D | Male | 6.28 (5.58, 7.06） |
| Sierra Leone | 60 to 64 | T1D | Both | 6.44 (5.88, 7.05） |
| Sierra Leone | 60 to 64 | T1D | Female | 6.54 (5.76, 7.44） |
| Sierra Leone | 60 to 64 | T1D | Male | 6.33 (5.57, 7.19） |
| Sierra Leone | 65 to 69 | T1D | Both | 6.47 (5.86, 7.14） |
| Sierra Leone | 65 to 69 | T1D | Female | 6.58 (5.72, 7.58） |
| Sierra Leone | 65 to 69 | T1D | Male | 6.35 (5.52, 7.30） |
| Singapore | 0 to 4 | T1D | Both | 0.15 (0.14, 0.17） |
| Singapore | 0 to 4 | T1D | Female | 0.14 (0.12, 0.17） |
| Singapore | 0 to 4 | T1D | Male | 0.15 (0.13, 0.18） |
| Singapore | 10 to 14 | T1D | Both | 1.82 (1.73, 1.91） |
| Singapore | 10 to 14 | T1D | Female | 1.81 (1.68, 1.94） |
| Singapore | 10 to 14 | T1D | Male | 1.82 (1.71, 1.95） |
| Singapore | 15 to 19 | T1D | Both | 2.06 (1.96, 2.16） |

| | | | | |
|---|---|---|---|---|
| Singapore | 15 to 19 | T1D | Female | 2.05 (1.91, 2.20） |
| Singapore | 15 to 19 | T1D | Male | 2.07 (1.94, 2.21） |
| Singapore | 20 to 24 | T1D | Both | 2.12 (2.02, 2.23） |
| Singapore | 20 to 24 | T1D | Female | 2.11 (1.97, 2.26） |
| Singapore | 20 to 24 | T1D | Male | 2.14 (2.00, 2.28） |
| Singapore | 25 to 29 | T1D | Both | 2.19 (2.08, 2.30） |
| Singapore | 25 to 29 | T1D | Female | 2.17 (2.02, 2.33） |
| Singapore | 25 to 29 | T1D | Male | 2.21 (2.06, 2.36） |
| Singapore | 30 to 34 | T1D | Both | 2.25 (2.14, 2.37） |
| Singapore | 30 to 34 | T1D | Female | 2.23 (2.07, 2.40） |
| Singapore | 30 to 34 | T1D | Male | 2.27 (2.12, 2.43） |
| Singapore | 35 to 39 | T1D | Both | 2.31 (2.19, 2.43） |
| Singapore | 35 to 39 | T1D | Female | 2.28 (2.11, 2.46） |
| Singapore | 35 to 39 | T1D | Male | 2.33 (2.17, 2.50） |
| Singapore | 40 to 44 | T1D | Both | 2.36 (2.24, 2.49） |
| Singapore | 40 to 44 | T1D | Female | 2.33 (2.16, 2.52） |
| Singapore | 40 to 44 | T1D | Male | 2.38 (2.22, 2.56） |
| Singapore | 45 to 49 | T1D | Both | 2.42 (2.29, 2.56） |
| Singapore | 45 to 49 | T1D | Female | 2.38 (2.20, 2.59） |
| Singapore | 45 to 49 | T1D | Male | 2.44 (2.26, 2.63） |
| Singapore | 5 to 9 | T1D | Both | 1.00 (1.00, 1.00） |
| Singapore | 5 to 9 | T1D | Female | 1.00 (1.00, 1.00） |
| Singapore | 5 to 9 | T1D | Male | 1.00 (1.00, 1.00） |
| Singapore | 50 to 54 | T1D | Both | 2.47 (2.34, 2.62） |
| Singapore | 50 to 54 | T1D | Female | 2.44 (2.24, 2.65） |
| Singapore | 50 to 54 | T1D | Male | 2.49 (2.31, 2.69） |
| Singapore | 55 to 59 | T1D | Both | 2.53 (2.38, 2.68） |
| Singapore | 55 to 59 | T1D | Female | 2.50 (2.29, 2.72） |
| Singapore | 55 to 59 | T1D | Male | 2.55 (2.35, 2.76） |
| Singapore | 60 to 64 | T1D | Both | 2.58 (2.43, 2.75） |
| Singapore | 60 to 64 | T1D | Female | 2.55 (2.33, 2.80） |
| Singapore | 60 to 64 | T1D | Male | 2.60 (2.39, 2.83） |
| Singapore | 65 to 69 | T1D | Both | 2.64 (2.47, 2.82） |

| | | | | |
|---|---|---|---|---|
| Singapore | 65 to 69 | T1D | Female | 2.61 (2.36, 2.87） |
| Singapore | 65 to 69 | T1D | Male | 2.65 (2.42, 2.91） |
| Slovakia | 0 to 4 | T1D | Both | 0.19 (0.16, 0.22） |
| Slovakia | 0 to 4 | T1D | Female | 0.18 (0.15, 0.22） |
| Slovakia | 0 to 4 | T1D | Male | 0.20 (0.16, 0.24） |
| Slovakia | 10 to 14 | T1D | Both | 2.21 (2.06, 2.37） |
| Slovakia | 10 to 14 | T1D | Female | 2.22 (2.01, 2.44） |
| Slovakia | 10 to 14 | T1D | Male | 2.21 (2.01, 2.44） |
| Slovakia | 15 to 19 | T1D | Both | 3.40 (3.18, 3.64） |
| Slovakia | 15 to 19 | T1D | Female | 3.31 (3.01, 3.64） |
| Slovakia | 15 to 19 | T1D | Male | 3.49 (3.17, 3.84） |
| Slovakia | 20 to 24 | T1D | Both | 4.45 (4.16, 4.77） |
| Slovakia | 20 to 24 | T1D | Female | 4.21 (3.83, 4.63） |
| Slovakia | 20 to 24 | T1D | Male | 4.71 (4.28, 5.18） |
| Slovakia | 25 to 29 | T1D | Both | 5.65 (5.28, 6.05） |
| Slovakia | 25 to 29 | T1D | Female | 5.19 (4.71, 5.72） |
| Slovakia | 25 to 29 | T1D | Male | 6.12 (5.56, 6.74） |
| Slovakia | 30 to 34 | T1D | Both | 6.84 (6.38, 7.33） |
| Slovakia | 30 to 34 | T1D | Female | 6.14 (5.57, 6.78） |
| Slovakia | 30 to 34 | T1D | Male | 7.56 (6.85, 8.33） |
| Slovakia | 35 to 39 | T1D | Both | 8.12 (7.57, 8.72） |
| Slovakia | 35 to 39 | T1D | Female | 7.17 (6.49, 7.93） |
| Slovakia | 35 to 39 | T1D | Male | 9.10 (8.24, 10.06） |
| Slovakia | 40 to 44 | T1D | Both | 9.55 (8.88, 10.26） |
| Slovakia | 40 to 44 | T1D | Female | 8.34 (7.52, 9.24） |
| Slovakia | 40 to 44 | T1D | Male | 10.81 (9.77, 11.96） |
| Slovakia | 45 to 49 | T1D | Both | 11.12 (10.33, 11.96） |
| Slovakia | 45 to 49 | T1D | Female | 9.63 (8.68, 10.70） |
| Slovakia | 45 to 49 | T1D | Male | 12.69 (11.45, 14.06） |
| Slovakia | 5 to 9 | T1D | Both | 1.00 (1.00, 1.00） |
| Slovakia | 5 to 9 | T1D | Female | 1.00 (1.00, 1.00） |
| Slovakia | 5 to 9 | T1D | Male | 1.00 (1.00, 1.00） |
| Slovakia | 50 to 54 | T1D | Both | 12.83 (11.91, 13.82） |

| | | | | |
|---|---|---|---|---|
| Slovakia | 50 to 54 | T1D | Female | 11.08 (9.96, 12.32） |
| Slovakia | 50 to 54 | T1D | Male | 14.72 (13.25, 16.34） |
| Slovakia | 55 to 59 | T1D | Both | 14.69 (13.62, 15.85） |
| Slovakia | 55 to 59 | T1D | Female | 12.69 (11.38, 14.14） |
| Slovakia | 55 to 59 | T1D | Male | 16.92 (15.21, 18.82） |
| Slovakia | 60 to 64 | T1D | Both | 16.74 (15.49, 18.10） |
| Slovakia | 60 to 64 | T1D | Female | 14.50 (12.98, 16.19） |
| Slovakia | 60 to 64 | T1D | Male | 19.32 (17.33, 21.55） |
| Slovakia | 65 to 69 | T1D | Both | 19.04 (17.58, 20.63） |
| Slovakia | 65 to 69 | T1D | Female | 16.58 (14.80, 18.58） |
| Slovakia | 65 to 69 | T1D | Male | 22.03 (19.68, 24.66） |
| Slovenia | 0 to 4 | T1D | Both | 0.20 (0.15, 0.25） |
| Slovenia | 0 to 4 | T1D | Female | 0.19 (0.13, 0.27） |
| Slovenia | 0 to 4 | T1D | Male | 0.20 (0.14, 0.28） |
| Slovenia | 10 to 14 | T1D | Both | 2.18 (1.93, 2.46） |
| Slovenia | 10 to 14 | T1D | Female | 2.20 (1.85, 2.60） |
| Slovenia | 10 to 14 | T1D | Male | 2.17 (1.83, 2.58） |
| Slovenia | 15 to 19 | T1D | Both | 3.37 (2.99, 3.79） |
| Slovenia | 15 to 19 | T1D | Female | 3.31 (2.79, 3.91） |
| Slovenia | 15 to 19 | T1D | Male | 3.44 (2.91, 4.07） |
| Slovenia | 20 to 24 | T1D | Both | 4.42 (3.93, 4.98） |
| Slovenia | 20 to 24 | T1D | Female | 4.23 (3.58, 5.01） |
| Slovenia | 20 to 24 | T1D | Male | 4.64 (3.92, 5.49） |
| Slovenia | 25 to 29 | T1D | Both | 5.64 (5.00, 6.35） |
| Slovenia | 25 to 29 | T1D | Female | 5.25 (4.43, 6.22） |
| Slovenia | 25 to 29 | T1D | Male | 6.04 (5.11, 7.15） |
| Slovenia | 30 to 34 | T1D | Both | 6.87 (6.09, 7.75） |
| Slovenia | 30 to 34 | T1D | Female | 6.26 (5.27, 7.44） |
| Slovenia | 30 to 34 | T1D | Male | 7.53 (6.35, 8.93） |
| Slovenia | 35 to 39 | T1D | Both | 8.22 (7.27, 9.30） |
| Slovenia | 35 to 39 | T1D | Female | 7.35 (6.17, 8.76） |
| Slovenia | 35 to 39 | T1D | Male | 9.15 (7.69, 10.88） |
| Slovenia | 40 to 44 | T1D | Both | 9.75 (8.61, 11.04） |

| | | | | |
|---|---|---|---|---|
| Slovenia | 40 to 44 | T1D | Female | 8.59 (7.19, 10.26） |
| Slovenia | 40 to 44 | T1D | Male | 10.98 (9.22, 13.09） |
| Slovenia | 45 to 49 | T1D | Both | 11.45 (10.10, 12.99） |
| Slovenia | 45 to 49 | T1D | Female | 9.98 (8.33, 11.94） |
| Slovenia | 45 to 49 | T1D | Male | 13.02 (10.90, 15.54） |
| Slovenia | 5 to 9 | T1D | Both | 1.00 (1.00, 1.00） |
| Slovenia | 5 to 9 | T1D | Female | 1.00 (1.00, 1.00） |
| Slovenia | 5 to 9 | T1D | Male | 1.00 (1.00, 1.00） |
| Slovenia | 50 to 54 | T1D | Both | 13.33 (11.73, 15.15） |
| Slovenia | 50 to 54 | T1D | Female | 11.54 (9.62, 13.85） |
| Slovenia | 50 to 54 | T1D | Male | 15.26 (12.75, 18.26） |
| Slovenia | 55 to 59 | T1D | Both | 15.42 (13.54, 17.55） |
| Slovenia | 55 to 59 | T1D | Female | 13.30 (11.05, 16.01） |
| Slovenia | 55 to 59 | T1D | Male | 17.73 (14.78, 21.28） |
| Slovenia | 60 to 64 | T1D | Both | 17.76 (15.57, 20.27） |
| Slovenia | 60 to 64 | T1D | Female | 15.30 (12.67, 18.48） |
| Slovenia | 60 to 64 | T1D | Male | 20.49 (17.02, 24.66） |
| Slovenia | 65 to 69 | T1D | Both | 20.43 (17.85, 23.37） |
| Slovenia | 65 to 69 | T1D | Female | 17.62 (14.54, 21.35） |
| Slovenia | 65 to 69 | T1D | Male | 23.67 (19.58, 28.62） |
| Solomon Islands | 0 to 4 | T1D | Both | 0.14 (0.08, 0.25） |
| Solomon Islands | 0 to 4 | T1D | Female | 0.15 (0.07, 0.32） |
| Solomon Islands | 0 to 4 | T1D | Male | 0.15 (0.07, 0.32） |
| Solomon Islands | 10 to 14 | T1D | Both | 2.04 (1.56, 2.67） |
| Solomon Islands | 10 to 14 | T1D | Female | 2.05 (1.39, 3.01） |
| Solomon Islands | 10 to 14 | T1D | Male | 2.13 (1.45, 3.12） |
| Solomon Islands | 15 to 19 | T1D | Both | 2.90 (2.20, 3.81） |
| Solomon Islands | 15 to 19 | T1D | Female | 2.88 (1.95, 4.26） |
| Solomon Islands | 15 to 19 | T1D | Male | 3.12 (2.11, 4.63） |
| Solomon Islands | 20 to 24 | T1D | Both | 3.68 (2.77, 4.88） |
| Solomon Islands | 20 to 24 | T1D | Female | 3.47 (2.31, 5.20） |
| Solomon Islands | 20 to 24 | T1D | Male | 4.08 (2.72, 6.11） |
| Solomon Islands | 25 to 29 | T1D | Both | 4.42 (3.30, 5.93） |

| | | | | |
|---|---|---|---|---|
| Solomon Islands | 25 to 29 | T1D | Female | 4.14 (2.72, 6.28） |
| Solomon Islands | 25 to 29 | T1D | Male | 5.09 (3.35, 7.72） |
| Solomon Islands | 30 to 34 | T1D | Both | 5.03 (3.70, 6.82） |
| Solomon Islands | 30 to 34 | T1D | Female | 4.56 (2.94, 7.07） |
| Solomon Islands | 30 to 34 | T1D | Male | 5.79 (3.76, 8.93） |
| Solomon Islands | 35 to 39 | T1D | Both | 5.51 (3.99, 7.59） |
| Solomon Islands | 35 to 39 | T1D | Female | 4.92 (3.10, 7.81） |
| Solomon Islands | 35 to 39 | T1D | Male | 6.44 (4.09, 10.12） |
| Solomon Islands | 40 to 44 | T1D | Both | 6.02 (4.29, 8.44） |
| Solomon Islands | 40 to 44 | T1D | Female | 5.36 (3.29, 8.75） |
| Solomon Islands | 40 to 44 | T1D | Male | 7.10 (4.41, 11.41） |
| Solomon Islands | 45 to 49 | T1D | Both | 6.43 (4.48, 9.22） |
| Solomon Islands | 45 to 49 | T1D | Female | 5.84 (3.47, 9.84） |
| Solomon Islands | 45 to 49 | T1D | Male | 7.58 (4.59, 12.54） |
| Solomon Islands | 5 to 9 | T1D | Both | 1.00 (1.00, 1.00） |
| Solomon Islands | 5 to 9 | T1D | Female | 1.00 (1.00, 1.00） |
| Solomon Islands | 5 to 9 | T1D | Male | 1.00 (1.00, 1.00） |
| Solomon Islands | 50 to 54 | T1D | Both | 6.91 (4.70, 10.17） |
| Solomon Islands | 50 to 54 | T1D | Female | 6.34 (3.62, 11.10） |
| Solomon Islands | 50 to 54 | T1D | Male | 8.17 (4.79, 13.94） |
| Solomon Islands | 55 to 59 | T1D | Both | 7.40 (4.89, 11.19） |
| Solomon Islands | 55 to 59 | T1D | Female | 6.55 (3.56, 12.05） |
| Solomon Islands | 55 to 59 | T1D | Male | 8.43 (4.75, 14.96） |
| Solomon Islands | 60 to 64 | T1D | Both | 7.66 (4.88, 12.00） |
| Solomon Islands | 60 to 64 | T1D | Female | 7.00 (3.62, 13.53） |
| Solomon Islands | 60 to 64 | T1D | Male | 8.55 (4.60, 15.88） |
| Solomon Islands | 65 to 69 | T1D | Both | 8.29 (5.06, 13.59） |
| Solomon Islands | 65 to 69 | T1D | Female | 7.18 (3.44, 14.98） |
| Solomon Islands | 65 to 69 | T1D | Male | 9.21 (4.67, 18.14） |
| Somalia | 0 to 4 | T1D | Both | 0.15 (0.14, 0.16） |
| Somalia | 0 to 4 | T1D | Female | 0.15 (0.13, 0.16） |
| Somalia | 0 to 4 | T1D | Male | 0.15 (0.13, 0.16） |
| Somalia | 10 to 14 | T1D | Both | 2.07 (2.00, 2.15） |

| | | | | |
|---|---|---|---|---|
| Somalia | 10 to 14 | T1D | Female | 2.07 (1.96, 2.17） |
| Somalia | 10 to 14 | T1D | Male | 2.08 (1.98, 2.18） |
| Somalia | 15 to 19 | T1D | Both | 3.03 (2.92, 3.14） |
| Somalia | 15 to 19 | T1D | Female | 3.02 (2.87, 3.18） |
| Somalia | 15 to 19 | T1D | Male | 3.03 (2.89, 3.18） |
| Somalia | 20 to 24 | T1D | Both | 3.89 (3.75, 4.04） |
| Somalia | 20 to 24 | T1D | Female | 3.89 (3.68, 4.11） |
| Somalia | 20 to 24 | T1D | Male | 3.90 (3.71, 4.10） |
| Somalia | 25 to 29 | T1D | Both | 4.74 (4.56, 4.93） |
| Somalia | 25 to 29 | T1D | Female | 4.75 (4.49, 5.02） |
| Somalia | 25 to 29 | T1D | Male | 4.74 (4.50, 5.00） |
| Somalia | 30 to 34 | T1D | Both | 5.24 (5.04, 5.46） |
| Somalia | 30 to 34 | T1D | Female | 5.26 (4.96, 5.58） |
| Somalia | 30 to 34 | T1D | Male | 5.24 (4.96, 5.53） |
| Somalia | 35 to 39 | T1D | Both | 5.59 (5.35, 5.83） |
| Somalia | 35 to 39 | T1D | Female | 5.62 (5.28, 5.97） |
| Somalia | 35 to 39 | T1D | Male | 5.57 (5.25, 5.90） |
| Somalia | 40 to 44 | T1D | Both | 5.84 (5.58, 6.11） |
| Somalia | 40 to 44 | T1D | Female | 5.88 (5.51, 6.28） |
| Somalia | 40 to 44 | T1D | Male | 5.80 (5.46, 6.18） |
| Somalia | 45 to 49 | T1D | Both | 6.01 (5.72, 6.30） |
| Somalia | 45 to 49 | T1D | Female | 6.07 (5.66, 6.51） |
| Somalia | 45 to 49 | T1D | Male | 5.96 (5.57, 6.37） |
| Somalia | 5 to 9 | T1D | Both | 1.00 (1.00, 1.00） |
| Somalia | 5 to 9 | T1D | Female | 1.00 (1.00, 1.00） |
| Somalia | 5 to 9 | T1D | Male | 1.00 (1.00, 1.00） |
| Somalia | 50 to 54 | T1D | Both | 6.11 (5.80, 6.43） |
| Somalia | 50 to 54 | T1D | Female | 6.19 (5.75, 6.67） |
| Somalia | 50 to 54 | T1D | Male | 6.04 (5.62, 6.49） |
| Somalia | 55 to 59 | T1D | Both | 6.16 (5.83, 6.51） |
| Somalia | 55 to 59 | T1D | Female | 6.27 (5.80, 6.78） |
| Somalia | 55 to 59 | T1D | Male | 6.08 (5.63, 6.57） |
| Somalia | 60 to 64 | T1D | Both | 6.18 (5.82, 6.56） |

| | | | | |
|---|---|---|---|---|
| Somalia | 60 to 64 | T1D | Female | 6.30 (5.79, 6.86） |
| Somalia | 60 to 64 | T1D | Male | 6.08 (5.58, 6.62） |
| Somalia | 65 to 69 | T1D | Both | 6.13 (5.72, 6.56） |
| Somalia | 65 to 69 | T1D | Female | 6.28 (5.71, 6.90） |
| Somalia | 65 to 69 | T1D | Male | 6.00 (5.43, 6.63） |
| South Africa | 0 to 4 | T1D | Both | 0.15 (0.15, 0.16） |
| South Africa | 0 to 4 | T1D | Female | 0.15 (0.14, 0.16） |
| South Africa | 0 to 4 | T1D | Male | 0.15 (0.14, 0.16） |
| South Africa | 10 to 14 | T1D | Both | 2.09 (2.05, 2.13） |
| South Africa | 10 to 14 | T1D | Female | 2.10 (2.04, 2.15） |
| South Africa | 10 to 14 | T1D | Male | 2.09 (2.03, 2.14） |
| South Africa | 15 to 19 | T1D | Both | 3.06 (3.00, 3.12） |
| South Africa | 15 to 19 | T1D | Female | 3.07 (2.98, 3.15） |
| South Africa | 15 to 19 | T1D | Male | 3.05 (2.97, 3.13） |
| South Africa | 20 to 24 | T1D | Both | 3.95 (3.87, 4.02） |
| South Africa | 20 to 24 | T1D | Female | 3.96 (3.86, 4.07） |
| South Africa | 20 to 24 | T1D | Male | 3.93 (3.83, 4.04） |
| South Africa | 25 to 29 | T1D | Both | 4.93 (4.84, 5.03） |
| South Africa | 25 to 29 | T1D | Female | 4.96 (4.82, 5.10） |
| South Africa | 25 to 29 | T1D | Male | 4.90 (4.77, 5.04） |
| South Africa | 30 to 34 | T1D | Both | 5.63 (5.51, 5.74） |
| South Africa | 30 to 34 | T1D | Female | 5.66 (5.50, 5.83） |
| South Africa | 30 to 34 | T1D | Male | 5.58 (5.43, 5.74） |
| South Africa | 35 to 39 | T1D | Both | 6.16 (6.03, 6.29） |
| South Africa | 35 to 39 | T1D | Female | 6.22 (6.03, 6.40） |
| South Africa | 35 to 39 | T1D | Male | 6.10 (5.93, 6.28） |
| South Africa | 40 to 44 | T1D | Both | 6.62 (6.48, 6.77） |
| South Africa | 40 to 44 | T1D | Female | 6.70 (6.49, 6.90） |
| South Africa | 40 to 44 | T1D | Male | 6.55 (6.36, 6.75） |
| South Africa | 45 to 49 | T1D | Both | 7.05 (6.89, 7.20） |
| South Africa | 45 to 49 | T1D | Female | 7.14 (6.92, 7.37） |
| South Africa | 45 to 49 | T1D | Male | 6.96 (6.75, 7.18） |
| South Africa | 5 to 9 | T1D | Both | 1.00 (1.00, 1.00） |

| | | | | |
|---|---|---|---|---|
| South Africa | 5 to 9 | T1D | Female | 1.00 (1.00, 1.00） |
| South Africa | 5 to 9 | T1D | Male | 1.00 (1.00, 1.00） |
| South Africa | 50 to 54 | T1D | Both | 7.44 (7.27, 7.61） |
| South Africa | 50 to 54 | T1D | Female | 7.56 (7.32, 7.81） |
| South Africa | 50 to 54 | T1D | Male | 7.33 (7.10, 7.57） |
| South Africa | 55 to 59 | T1D | Both | 7.80 (7.61, 7.98） |
| South Africa | 55 to 59 | T1D | Female | 7.94 (7.68, 8.21） |
| South Africa | 55 to 59 | T1D | Male | 7.66 (7.41, 7.93） |
| South Africa | 60 to 64 | T1D | Both | 8.10 (7.90, 8.30） |
| South Africa | 60 to 64 | T1D | Female | 8.27 (7.98, 8.57） |
| South Africa | 60 to 64 | T1D | Male | 7.95 (7.67, 8.24） |
| South Africa | 65 to 69 | T1D | Both | 8.35 (8.12, 8.57） |
| South Africa | 65 to 69 | T1D | Female | 8.55 (8.23, 8.87） |
| South Africa | 65 to 69 | T1D | Male | 8.17 (7.86, 8.49） |
| South Sudan | 0 to 4 | T1D | Both | 0.14 (0.13, 0.16） |
| South Sudan | 0 to 4 | T1D | Female | 0.14 (0.13, 0.17） |
| South Sudan | 0 to 4 | T1D | Male | 0.14 (0.13, 0.16） |
| South Sudan | 10 to 14 | T1D | Both | 2.09 (2.00, 2.19） |
| South Sudan | 10 to 14 | T1D | Female | 2.09 (1.96, 2.23） |
| South Sudan | 10 to 14 | T1D | Male | 2.10 (1.98, 2.23） |
| South Sudan | 15 to 19 | T1D | Both | 3.05 (2.91, 3.19） |
| South Sudan | 15 to 19 | T1D | Female | 3.05 (2.85, 3.25） |
| South Sudan | 15 to 19 | T1D | Male | 3.06 (2.87, 3.25） |
| South Sudan | 20 to 24 | T1D | Both | 3.92 (3.74, 4.11） |
| South Sudan | 20 to 24 | T1D | Female | 3.93 (3.67, 4.21） |
| South Sudan | 20 to 24 | T1D | Male | 3.93 (3.68, 4.19） |
| South Sudan | 25 to 29 | T1D | Both | 4.78 (4.55, 5.02） |
| South Sudan | 25 to 29 | T1D | Female | 4.80 (4.47, 5.15） |
| South Sudan | 25 to 29 | T1D | Male | 4.78 (4.47, 5.11） |
| South Sudan | 30 to 34 | T1D | Both | 5.29 (5.03, 5.57） |
| South Sudan | 30 to 34 | T1D | Female | 5.34 (4.96, 5.74） |
| South Sudan | 30 to 34 | T1D | Male | 5.29 (4.93, 5.67） |
| South Sudan | 35 to 39 | T1D | Both | 5.65 (5.36, 5.96） |

| | | | | |
|---|---|---|---|---|
| South Sudan | 35 to 39 | T1D | Female | 5.71 (5.29, 6.17） |
| South Sudan | 35 to 39 | T1D | Male | 5.63 (5.24, 6.06） |
| South Sudan | 40 to 44 | T1D | Both | 5.93 (5.61, 6.27） |
| South Sudan | 40 to 44 | T1D | Female | 6.01 (5.54, 6.52） |
| South Sudan | 40 to 44 | T1D | Male | 5.90 (5.46, 6.37） |
| South Sudan | 45 to 49 | T1D | Both | 6.15 (5.79, 6.52） |
| South Sudan | 45 to 49 | T1D | Female | 6.24 (5.73, 6.81） |
| South Sudan | 45 to 49 | T1D | Male | 6.09 (5.62, 6.61） |
| South Sudan | 5 to 9 | T1D | Both | 1.00 (1.00, 1.00） |
| South Sudan | 5 to 9 | T1D | Female | 1.00 (1.00, 1.00） |
| South Sudan | 5 to 9 | T1D | Male | 1.00 (1.00, 1.00） |
| South Sudan | 50 to 54 | T1D | Both | 6.30 (5.91, 6.72） |
| South Sudan | 50 to 54 | T1D | Female | 6.42 (5.85, 7.05） |
| South Sudan | 50 to 54 | T1D | Male | 6.24 (5.72, 6.81） |
| South Sudan | 55 to 59 | T1D | Both | 6.41 (5.98, 6.87） |
| South Sudan | 55 to 59 | T1D | Female | 6.55 (5.91, 7.27） |
| South Sudan | 55 to 59 | T1D | Male | 6.33 (5.75, 6.96） |
| South Sudan | 60 to 64 | T1D | Both | 6.47 (5.99, 7.00） |
| South Sudan | 60 to 64 | T1D | Female | 6.63 (5.91, 7.44） |
| South Sudan | 60 to 64 | T1D | Male | 6.37 (5.73, 7.07） |
| South Sudan | 65 to 69 | T1D | Both | 6.49 (5.94, 7.08） |
| South Sudan | 65 to 69 | T1D | Female | 6.68 (5.85, 7.62） |
| South Sudan | 65 to 69 | T1D | Male | 6.36 (5.65, 7.16） |
| Spain | 0 to 4 | T1D | Both | 0.21 (0.19, 0.24） |
| Spain | 0 to 4 | T1D | Female | 0.21 (0.18, 0.24） |
| Spain | 0 to 4 | T1D | Male | 0.22 (0.20, 0.24） |
| Spain | 10 to 14 | T1D | Both | 2.06 (1.95, 2.18） |
| Spain | 10 to 14 | T1D | Female | 2.00 (1.88, 2.14） |
| Spain | 10 to 14 | T1D | Male | 2.12 (2.03, 2.22） |
| Spain | 15 to 19 | T1D | Both | 3.04 (2.88, 3.21） |
| Spain | 15 to 19 | T1D | Female | 2.87 (2.69, 3.06） |
| Spain | 15 to 19 | T1D | Male | 3.22 (3.08, 3.37） |
| Spain | 20 to 24 | T1D | Both | 3.93 (3.72, 4.15） |

| | | | | |
|---|---|---|---|---|
| Spain | 20 to 24 | T1D | Female | 3.66 (3.43, 3.91） |
| Spain | 20 to 24 | T1D | Male | 4.22 (4.04, 4.42） |
| Spain | 25 to 29 | T1D | Both | 4.92 (4.65, 5.20） |
| Spain | 25 to 29 | T1D | Female | 4.52 (4.23, 4.84） |
| Spain | 25 to 29 | T1D | Male | 5.36 (5.12, 5.61） |
| Spain | 30 to 34 | T1D | Both | 5.98 (5.65, 6.32） |
| Spain | 30 to 34 | T1D | Female | 5.43 (5.07, 5.80） |
| Spain | 30 to 34 | T1D | Male | 6.60 (6.30, 6.91） |
| Spain | 35 to 39 | T1D | Both | 7.14 (6.75, 7.56） |
| Spain | 35 to 39 | T1D | Female | 6.40 (5.98, 6.85） |
| Spain | 35 to 39 | T1D | Male | 7.99 (7.63, 8.37） |
| Spain | 40 to 44 | T1D | Both | 8.46 (7.98, 8.96） |
| Spain | 40 to 44 | T1D | Female | 7.48 (6.98, 8.02） |
| Spain | 40 to 44 | T1D | Male | 9.58 (9.14, 10.04） |
| Spain | 45 to 49 | T1D | Both | 9.94 (9.37, 10.54） |
| Spain | 45 to 49 | T1D | Female | 8.70 (8.11, 9.34） |
| Spain | 45 to 49 | T1D | Male | 11.37 (10.84, 11.93） |
| Spain | 5 to 9 | T1D | Both | 1.00 (1.00, 1.00） |
| Spain | 5 to 9 | T1D | Female | 1.00 (1.00, 1.00） |
| Spain | 5 to 9 | T1D | Male | 1.00 (1.00, 1.00） |
| Spain | 50 to 54 | T1D | Both | 11.59 (10.92, 12.31） |
| Spain | 50 to 54 | T1D | Female | 10.06 (9.36, 10.81） |
| Spain | 50 to 54 | T1D | Male | 13.40 (12.76, 14.06） |
| Spain | 55 to 59 | T1D | Both | 13.45 (12.65, 14.29） |
| Spain | 55 to 59 | T1D | Female | 11.57 (10.75, 12.44） |
| Spain | 55 to 59 | T1D | Male | 15.68 (14.92, 16.47） |
| Spain | 60 to 64 | T1D | Both | 15.52 (14.59, 16.52） |
| Spain | 60 to 64 | T1D | Female | 13.24 (12.29, 14.27） |
| Spain | 60 to 64 | T1D | Male | 18.25 (17.35, 19.19） |
| Spain | 65 to 69 | T1D | Both | 17.84 (16.75, 19.01） |
| Spain | 65 to 69 | T1D | Female | 15.11 (14.01, 16.30） |
| Spain | 65 to 69 | T1D | Male | 21.14 (20.08, 22.26） |
| Sri Lanka | 0 to 4 | T1D | Both | 0.15 (0.13, 0.17） |

| | | | | |
|---|---|---|---|---|
| Sri Lanka | 0 to 4 | T1D | Female | 0.15 (0.13, 0.18） |
| Sri Lanka | 0 to 4 | T1D | Male | 0.15 (0.13, 0.18） |
| Sri Lanka | 10 to 14 | T1D | Both | 2.06 (1.95, 2.16） |
| Sri Lanka | 10 to 14 | T1D | Female | 2.04 (1.89, 2.19） |
| Sri Lanka | 10 to 14 | T1D | Male | 2.08 (1.93, 2.24） |
| Sri Lanka | 15 to 19 | T1D | Both | 2.93 (2.78, 3.09） |
| Sri Lanka | 15 to 19 | T1D | Female | 2.85 (2.65, 3.06） |
| Sri Lanka | 15 to 19 | T1D | Male | 3.02 (2.81, 3.26） |
| Sri Lanka | 20 to 24 | T1D | Both | 3.68 (3.50, 3.88） |
| Sri Lanka | 20 to 24 | T1D | Female | 3.50 (3.26, 3.77） |
| Sri Lanka | 20 to 24 | T1D | Male | 3.89 (3.61, 4.19） |
| Sri Lanka | 25 to 29 | T1D | Both | 4.46 (4.23, 4.71） |
| Sri Lanka | 25 to 29 | T1D | Female | 4.16 (3.86, 4.48） |
| Sri Lanka | 25 to 29 | T1D | Male | 4.80 (4.45, 5.18） |
| Sri Lanka | 30 to 34 | T1D | Both | 5.09 (4.82, 5.38） |
| Sri Lanka | 30 to 34 | T1D | Female | 4.68 (4.34, 5.05） |
| Sri Lanka | 30 to 34 | T1D | Male | 5.56 (5.14, 6.01） |
| Sri Lanka | 35 to 39 | T1D | Both | 5.67 (5.36, 5.99） |
| Sri Lanka | 35 to 39 | T1D | Female | 5.16 (4.77, 5.58） |
| Sri Lanka | 35 to 39 | T1D | Male | 6.24 (5.76, 6.76） |
| Sri Lanka | 40 to 44 | T1D | Both | 6.24 (5.89, 6.61） |
| Sri Lanka | 40 to 44 | T1D | Female | 5.65 (5.21, 6.12） |
| Sri Lanka | 40 to 44 | T1D | Male | 6.92 (6.38, 7.51） |
| Sri Lanka | 45 to 49 | T1D | Both | 6.81 (6.42, 7.22） |
| Sri Lanka | 45 to 49 | T1D | Female | 6.14 (5.65, 6.67） |
| Sri Lanka | 45 to 49 | T1D | Male | 7.58 (6.97, 8.24） |
| Sri Lanka | 5 to 9 | T1D | Both | 1.00 (1.00, 1.00） |
| Sri Lanka | 5 to 9 | T1D | Female | 1.00 (1.00, 1.00） |
| Sri Lanka | 5 to 9 | T1D | Male | 1.00 (1.00, 1.00） |
| Sri Lanka | 50 to 54 | T1D | Both | 7.37 (6.94, 7.83） |
| Sri Lanka | 50 to 54 | T1D | Female | 6.64 (6.10, 7.23） |
| Sri Lanka | 50 to 54 | T1D | Male | 8.23 (7.55, 8.97） |
| Sri Lanka | 55 to 59 | T1D | Both | 7.95 (7.47, 8.47） |

| | | | | |
|---|---|---|---|---|
| Sri Lanka | 55 to 59 | T1D | Female | 7.17 (6.57, 7.83） |
| Sri Lanka | 55 to 59 | T1D | Male | 8.88 (8.13, 9.71） |
| Sri Lanka | 60 to 64 | T1D | Both | 8.56 (8.02, 9.13） |
| Sri Lanka | 60 to 64 | T1D | Female | 7.73 (7.06, 8.47） |
| Sri Lanka | 60 to 64 | T1D | Male | 9.56 (8.71, 10.48） |
| Sri Lanka | 65 to 69 | T1D | Both | 9.19 (8.58, 9.85） |
| Sri Lanka | 65 to 69 | T1D | Female | 8.34 (7.57, 9.18） |
| Sri Lanka | 65 to 69 | T1D | Male | 10.25 (9.29, 11.30） |
| Sudan | 0 to 4 | T1D | Both | 0.12 (0.12, 0.13） |
| Sudan | 0 to 4 | T1D | Female | 0.12 (0.11, 0.13） |
| Sudan | 0 to 4 | T1D | Male | 0.13 (0.12, 0.14） |
| Sudan | 10 to 14 | T1D | Both | 2.47 (2.42, 2.52） |
| Sudan | 10 to 14 | T1D | Female | 2.71 (2.63, 2.80） |
| Sudan | 10 to 14 | T1D | Male | 2.29 (2.23, 2.35） |
| Sudan | 15 to 19 | T1D | Both | 3.78 (3.70, 3.86） |
| Sudan | 15 to 19 | T1D | Female | 4.14 (4.01, 4.27） |
| Sudan | 15 to 19 | T1D | Male | 3.51 (3.41, 3.61） |
| Sudan | 20 to 24 | T1D | Both | 4.89 (4.78, 4.99） |
| Sudan | 20 to 24 | T1D | Female | 5.21 (5.04, 5.38） |
| Sudan | 20 to 24 | T1D | Male | 4.67 (4.53, 4.81） |
| Sudan | 25 to 29 | T1D | Both | 6.10 (5.96, 6.24） |
| Sudan | 25 to 29 | T1D | Female | 6.32 (6.11, 6.53） |
| Sudan | 25 to 29 | T1D | Male | 6.01 (5.82, 6.19） |
| Sudan | 30 to 34 | T1D | Both | 7.30 (7.13, 7.48） |
| Sudan | 30 to 34 | T1D | Female | 7.39 (7.14, 7.66） |
| Sudan | 30 to 34 | T1D | Male | 7.36 (7.13, 7.60） |
| Sudan | 35 to 39 | T1D | Both | 8.59 (8.38, 8.81） |
| Sudan | 35 to 39 | T1D | Female | 8.52 (8.21, 8.84） |
| Sudan | 35 to 39 | T1D | Male | 8.80 (8.51, 9.11） |
| Sudan | 40 to 44 | T1D | Both | 10.01 (9.75, 10.28） |
| Sudan | 40 to 44 | T1D | Female | 9.73 (9.36, 10.13） |
| Sudan | 40 to 44 | T1D | Male | 10.36 (9.99, 10.73） |
| Sudan | 45 to 49 | T1D | Both | 11.55 (11.23, 11.88） |

| | | | | |
|---|---|---|---|---|
| Sudan | 45 to 49 | T1D | Female | 11.04 (10.58, 11.52） |
| Sudan | 45 to 49 | T1D | Male | 12.02 (11.57, 12.48） |
| Sudan | 5 to 9 | T1D | Both | 1.00 (1.00, 1.00） |
| Sudan | 5 to 9 | T1D | Female | 1.00 (1.00, 1.00） |
| Sudan | 5 to 9 | T1D | Male | 1.00 (1.00, 1.00） |
| Sudan | 50 to 54 | T1D | Both | 13.21 (12.82, 13.62） |
| Sudan | 50 to 54 | T1D | Female | 12.45 (11.89, 13.04） |
| Sudan | 50 to 54 | T1D | Male | 13.78 (13.23, 14.35） |
| Sudan | 55 to 59 | T1D | Both | 14.99 (14.51, 15.49） |
| Sudan | 55 to 59 | T1D | Female | 13.98 (13.30, 14.70） |
| Sudan | 55 to 59 | T1D | Male | 15.65 (14.99, 16.35） |
| Sudan | 60 to 64 | T1D | Both | 16.93 (16.34, 17.54） |
| Sudan | 60 to 64 | T1D | Female | 15.64 (14.81, 16.52） |
| Sudan | 60 to 64 | T1D | Male | 17.67 (16.86, 18.52） |
| Sudan | 65 to 69 | T1D | Both | 19.04 (18.30, 19.80） |
| Sudan | 65 to 69 | T1D | Female | 17.47 (16.43, 18.58） |
| Sudan | 65 to 69 | T1D | Male | 19.82 (18.83, 20.87） |
| Suriname | 0 to 4 | T1D | Both | 0.14 (0.08, 0.24） |
| Suriname | 0 to 4 | T1D | Female | 0.13 (0.05, 0.29） |
| Suriname | 0 to 4 | T1D | Male | 0.17 (0.08, 0.37） |
| Suriname | 10 to 14 | T1D | Both | 2.26 (1.79, 2.86） |
| Suriname | 10 to 14 | T1D | Female | 2.40 (1.74, 3.31） |
| Suriname | 10 to 14 | T1D | Male | 2.10 (1.51, 2.93） |
| Suriname | 15 to 19 | T1D | Both | 3.36 (2.66, 4.25） |
| Suriname | 15 to 19 | T1D | Female | 3.49 (2.52, 4.82） |
| Suriname | 15 to 19 | T1D | Male | 3.23 (2.31, 4.52） |
| Suriname | 20 to 24 | T1D | Both | 4.29 (3.37, 5.45） |
| Suriname | 20 to 24 | T1D | Female | 4.23 (3.04, 5.90） |
| Suriname | 20 to 24 | T1D | Male | 4.32 (3.07, 6.08） |
| Suriname | 25 to 29 | T1D | Both | 5.25 (4.11, 6.70） |
| Suriname | 25 to 29 | T1D | Female | 4.95 (3.53, 6.95） |
| Suriname | 25 to 29 | T1D | Male | 5.49 (3.88, 7.78） |
| Suriname | 30 to 34 | T1D | Both | 6.09 (4.74, 7.83） |

| | | | | |
|---|---|---|---|---|
| Suriname | 30 to 34 | T1D | Female | 5.58 (3.93, 7.91） |
| Suriname | 30 to 34 | T1D | Male | 6.57 (4.60, 9.39） |
| Suriname | 35 to 39 | T1D | Both | 6.84 (5.28, 8.86） |
| Suriname | 35 to 39 | T1D | Female | 6.19 (4.31, 8.89） |
| Suriname | 35 to 39 | T1D | Male | 7.62 (5.28, 11.00） |
| Suriname | 40 to 44 | T1D | Both | 7.69 (5.90, 10.03） |
| Suriname | 40 to 44 | T1D | Female | 6.80 (4.69, 9.87） |
| Suriname | 40 to 44 | T1D | Male | 8.59 (5.89, 12.51） |
| Suriname | 45 to 49 | T1D | Both | 8.50 (6.47, 11.17） |
| Suriname | 45 to 49 | T1D | Female | 7.45 (5.08, 10.93） |
| Suriname | 45 to 49 | T1D | Male | 9.65 (6.56, 14.21） |
| Suriname | 5 to 9 | T1D | Both | 1.00 (1.00, 1.00） |
| Suriname | 5 to 9 | T1D | Female | 1.00 (1.00, 1.00） |
| Suriname | 5 to 9 | T1D | Male | 1.00 (1.00, 1.00） |
| Suriname | 50 to 54 | T1D | Both | 9.30 (7.01, 12.35） |
| Suriname | 50 to 54 | T1D | Female | 8.14 (5.47, 12.11） |
| Suriname | 50 to 54 | T1D | Male | 10.70 (7.17, 15.96） |
| Suriname | 55 to 59 | T1D | Both | 10.11 (7.52, 13.59） |
| Suriname | 55 to 59 | T1D | Female | 8.76 (5.77, 13.28） |
| Suriname | 55 to 59 | T1D | Male | 11.69 (7.69, 17.77） |
| Suriname | 60 to 64 | T1D | Both | 10.88 (7.97, 14.86） |
| Suriname | 60 to 64 | T1D | Female | 9.53 (6.15, 14.77） |
| Suriname | 60 to 64 | T1D | Male | 12.77 (8.22, 19.83） |
| Suriname | 65 to 69 | T1D | Both | 11.73 (8.43, 16.32） |
| Suriname | 65 to 69 | T1D | Female | 10.22 (6.42, 16.26） |
| Suriname | 65 to 69 | T1D | Male | 13.73 (8.61, 21.91） |
| Sweden | 0 to 4 | T1D | Both | 0.21 (0.20, 0.23） |
| Sweden | 0 to 4 | T1D | Female | 0.20 (0.18, 0.22） |
| Sweden | 0 to 4 | T1D | Male | 0.22 (0.20, 0.24） |
| Sweden | 10 to 14 | T1D | Both | 1.89 (1.83, 1.95） |
| Sweden | 10 to 14 | T1D | Female | 1.88 (1.79, 1.97） |
| Sweden | 10 to 14 | T1D | Male | 1.89 (1.81, 1.98） |
| Sweden | 15 to 19 | T1D | Both | 2.66 (2.57, 2.74） |

| | | | | |
|---|---|---|---|---|
| Sweden | 15 to 19 | T1D | Female | 2.58 (2.47, 2.71） |
| Sweden | 15 to 19 | T1D | Male | 2.72 (2.60, 2.84） |
| Sweden | 20 to 24 | T1D | Both | 3.29 (3.18, 3.39） |
| Sweden | 20 to 24 | T1D | Female | 3.09 (2.95, 3.23） |
| Sweden | 20 to 24 | T1D | Male | 3.47 (3.32, 3.62） |
| Sweden | 25 to 29 | T1D | Both | 3.96 (3.83, 4.09） |
| Sweden | 25 to 29 | T1D | Female | 3.61 (3.44, 3.78） |
| Sweden | 25 to 29 | T1D | Male | 4.28 (4.10, 4.48） |
| Sweden | 30 to 34 | T1D | Both | 4.48 (4.33, 4.63） |
| Sweden | 30 to 34 | T1D | Female | 4.03 (3.84, 4.23） |
| Sweden | 30 to 34 | T1D | Male | 4.89 (4.67, 5.12） |
| Sweden | 35 to 39 | T1D | Both | 4.94 (4.77, 5.11） |
| Sweden | 35 to 39 | T1D | Female | 4.44 (4.23, 4.67） |
| Sweden | 35 to 39 | T1D | Male | 5.39 (5.15, 5.65） |
| Sweden | 40 to 44 | T1D | Both | 5.38 (5.20, 5.56） |
| Sweden | 40 to 44 | T1D | Female | 4.87 (4.63, 5.12） |
| Sweden | 40 to 44 | T1D | Male | 5.84 (5.57, 6.12） |
| Sweden | 45 to 49 | T1D | Both | 5.78 (5.58, 5.98） |
| Sweden | 45 to 49 | T1D | Female | 5.30 (5.04, 5.57） |
| Sweden | 45 to 49 | T1D | Male | 6.23 (5.94, 6.53） |
| Sweden | 5 to 9 | T1D | Both | 1.00 (1.00, 1.00） |
| Sweden | 5 to 9 | T1D | Female | 1.00 (1.00, 1.00） |
| Sweden | 5 to 9 | T1D | Male | 1.00 (1.00, 1.00） |
| Sweden | 50 to 54 | T1D | Both | 6.14 (5.93, 6.36） |
| Sweden | 50 to 54 | T1D | Female | 5.72 (5.43, 6.02） |
| Sweden | 50 to 54 | T1D | Male | 6.55 (6.24, 6.87） |
| Sweden | 55 to 59 | T1D | Both | 6.47 (6.25, 6.71） |
| Sweden | 55 to 59 | T1D | Female | 6.13 (5.82, 6.46） |
| Sweden | 55 to 59 | T1D | Male | 6.83 (6.50, 7.17） |
| Sweden | 60 to 64 | T1D | Both | 6.78 (6.54, 7.03） |
| Sweden | 60 to 64 | T1D | Female | 6.54 (6.20, 6.90） |
| Sweden | 60 to 64 | T1D | Male | 7.06 (6.72, 7.42） |
| Sweden | 65 to 69 | T1D | Both | 7.06 (6.80, 7.32） |

| | | | | |
|---|---|---|---|---|
| Sweden | 65 to 69 | T1D | Female | 6.96 (6.60, 7.35） |
| Sweden | 65 to 69 | T1D | Male | 7.26 (6.91, 7.64） |
| Switzerland | 0 to 4 | T1D | Both | 0.22 (0.20, 0.24） |
| Switzerland | 0 to 4 | T1D | Female | 0.21 (0.19, 0.24） |
| Switzerland | 0 to 4 | T1D | Male | 0.23 (0.20, 0.25） |
| Switzerland | 10 to 14 | T1D | Both | 2.09 (2.00, 2.18） |
| Switzerland | 10 to 14 | T1D | Female | 2.07 (1.94, 2.20） |
| Switzerland | 10 to 14 | T1D | Male | 2.10 (1.98, 2.23） |
| Switzerland | 15 to 19 | T1D | Both | 3.23 (3.09, 3.37） |
| Switzerland | 15 to 19 | T1D | Female | 3.15 (2.96, 3.35） |
| Switzerland | 15 to 19 | T1D | Male | 3.30 (3.11, 3.50） |
| Switzerland | 20 to 24 | T1D | Both | 4.33 (4.15, 4.52） |
| Switzerland | 20 to 24 | T1D | Female | 4.17 (3.92, 4.44） |
| Switzerland | 20 to 24 | T1D | Male | 4.49 (4.22, 4.77） |
| Switzerland | 25 to 29 | T1D | Both | 5.60 (5.36, 5.84） |
| Switzerland | 25 to 29 | T1D | Female | 5.30 (4.98, 5.65） |
| Switzerland | 25 to 29 | T1D | Male | 5.88 (5.53, 6.25） |
| Switzerland | 30 to 34 | T1D | Both | 6.82 (6.53, 7.13） |
| Switzerland | 30 to 34 | T1D | Female | 6.40 (6.01, 6.82） |
| Switzerland | 30 to 34 | T1D | Male | 7.23 (6.80, 7.69） |
| Switzerland | 35 to 39 | T1D | Both | 8.12 (7.76, 8.49） |
| Switzerland | 35 to 39 | T1D | Female | 7.56 (7.09, 8.07） |
| Switzerland | 35 to 39 | T1D | Male | 8.65 (8.13, 9.21） |
| Switzerland | 40 to 44 | T1D | Both | 9.52 (9.10, 9.96） |
| Switzerland | 40 to 44 | T1D | Female | 8.83 (8.27, 9.43） |
| Switzerland | 40 to 44 | T1D | Male | 10.20 (9.57, 10.87） |
| Switzerland | 45 to 49 | T1D | Both | 11.03 (10.53, 11.55） |
| Switzerland | 45 to 49 | T1D | Female | 10.20 (9.54, 10.90） |
| Switzerland | 45 to 49 | T1D | Male | 11.85 (11.11, 12.63） |
| Switzerland | 5 to 9 | T1D | Both | 1.00 (1.00, 1.00） |
| Switzerland | 5 to 9 | T1D | Female | 1.00 (1.00, 1.00） |
| Switzerland | 5 to 9 | T1D | Male | 1.00 (1.00, 1.00） |
| Switzerland | 50 to 54 | T1D | Both | 12.64 (12.07, 13.25） |

| | | | | |
|---|---|---|---|---|
| Switzerland | 50 to 54 | T1D | Female | 11.68 (10.92, 12.49） |
| Switzerland | 50 to 54 | T1D | Male | 13.60 (12.74, 14.51） |
| Switzerland | 55 to 59 | T1D | Both | 14.38 (13.72, 15.08） |
| Switzerland | 55 to 59 | T1D | Female | 13.29 (12.41, 14.23） |
| Switzerland | 55 to 59 | T1D | Male | 15.47 (14.48, 16.52） |
| Switzerland | 60 to 64 | T1D | Both | 16.25 (15.48, 17.05） |
| Switzerland | 60 to 64 | T1D | Female | 15.06 (14.05, 16.14） |
| Switzerland | 60 to 64 | T1D | Male | 17.47 (16.34, 18.68） |
| Switzerland | 65 to 69 | T1D | Both | 18.29 (17.42, 19.21） |
| Switzerland | 65 to 69 | T1D | Female | 17.02 (15.86, 18.27） |
| Switzerland | 65 to 69 | T1D | Male | 19.66 (18.36, 21.04） |
| Syrian Arab Republic | 0 to 4 | T1D | Both | 0.15 (0.14, 0.16） |
| Syrian Arab Republic | 0 to 4 | T1D | Female | 0.16 (0.15, 0.17） |
| Syrian Arab Republic | 0 to 4 | T1D | Male | 0.14 (0.13, 0.16） |
| Syrian Arab Republic | 10 to 14 | T1D | Both | 2.33 (2.27, 2.39） |
| Syrian Arab Republic | 10 to 14 | T1D | Female | 2.43 (2.34, 2.53） |
| Syrian Arab Republic | 10 to 14 | T1D | Male | 2.24 (2.16, 2.32） |
| Syrian Arab Republic | 15 to 19 | T1D | Both | 3.67 (3.57, 3.77） |
| Syrian Arab Republic | 15 to 19 | T1D | Female | 3.89 (3.73, 4.05） |
| Syrian Arab Republic | 15 to 19 | T1D | Male | 3.49 (3.37, 3.62） |
| Syrian Arab Republic | 20 to 24 | T1D | Both | 4.92 (4.78, 5.06） |
| Syrian Arab Republic | 20 to 24 | T1D | Female | 5.17 (4.95, 5.39） |
| Syrian Arab Republic | 20 to 24 | T1D | Male | 4.73 (4.55, 4.92） |
| Syrian Arab Republic | 25 to 29 | T1D | Both | 6.34 (6.15, 6.53） |
| Syrian Arab Republic | 25 to 29 | T1D | Female | 6.58 (6.30, 6.88） |
| Syrian Arab Republic | 25 to 29 | T1D | Male | 6.18 (5.93, 6.44） |
| Syrian Arab Republic | 30 to 34 | T1D | Both | 7.80 (7.56, 8.05） |
| Syrian Arab Republic | 30 to 34 | T1D | Female | 8.03 (7.67, 8.40） |
| Syrian Arab Republic | 30 to 34 | T1D | Male | 7.69 (7.37, 8.02） |
| Syrian Arab Republic | 35 to 39 | T1D | Both | 9.39 (9.09, 9.69） |
| Syrian Arab Republic | 35 to 39 | T1D | Female | 9.58 (9.14, 10.04） |
| Syrian Arab Republic | 35 to 39 | T1D | Male | 9.32 (8.92, 9.74） |
| Syrian Arab Republic | 40 to 44 | T1D | Both | 11.12 (10.75, 11.50） |

| | | | | |
|---|---|---|---|---|
| Syrian Arab Republic | 40 to 44 | T1D | Female | 11.28 (10.74, 11.85） |
| Syrian Arab Republic | 40 to 44 | T1D | Male | 11.09 (10.59, 11.61） |
| Syrian Arab Republic | 45 to 49 | T1D | Both | 13.03 (12.58, 13.49） |
| Syrian Arab Republic | 45 to 49 | T1D | Female | 13.16 (12.50, 13.86） |
| Syrian Arab Republic | 45 to 49 | T1D | Male | 13.03 (12.42, 13.67） |
| Syrian Arab Republic | 5 to 9 | T1D | Both | 1.00 (1.00, 1.00） |
| Syrian Arab Republic | 5 to 9 | T1D | Female | 1.00 (1.00, 1.00） |
| Syrian Arab Republic | 5 to 9 | T1D | Male | 1.00 (1.00, 1.00） |
| Syrian Arab Republic | 50 to 54 | T1D | Both | 15.13 (14.58, 15.70） |
| Syrian Arab Republic | 50 to 54 | T1D | Female | 15.27 (14.46, 16.12） |
| Syrian Arab Republic | 50 to 54 | T1D | Male | 15.14 (14.39, 15.93） |
| Syrian Arab Republic | 55 to 59 | T1D | Both | 17.46 (16.79, 18.16） |
| Syrian Arab Republic | 55 to 59 | T1D | Female | 17.62 (16.63, 18.68） |
| Syrian Arab Republic | 55 to 59 | T1D | Male | 17.45 (16.54, 18.42） |
| Syrian Arab Republic | 60 to 64 | T1D | Both | 20.06 (19.23, 20.93） |
| Syrian Arab Republic | 60 to 64 | T1D | Female | 20.30 (19.07, 21.61） |
| Syrian Arab Republic | 60 to 64 | T1D | Male | 20.01 (18.89, 21.19） |
| Syrian Arab Republic | 65 to 69 | T1D | Both | 23.00 (21.96, 24.09） |
| Syrian Arab Republic | 65 to 69 | T1D | Female | 23.37 (21.84, 25.01） |
| Syrian Arab Republic | 65 to 69 | T1D | Male | 22.87 (21.47, 24.35） |
| Taiwan (Province of China) | 0 to 4 | T1D | Both | 0.18 (0.16, 0.20） |
| Taiwan (Province of China) | 0 to 4 | T1D | Female | 0.18 (0.15, 0.21） |
| Taiwan (Province of China) | 0 to 4 | T1D | Male | 0.18 (0.16, 0.21） |
| Taiwan (Province of China) | 10 to 14 | T1D | Both | 2.04 (1.94, 2.14） |
| Taiwan (Province of China) | 10 to 14 | T1D | Female | 2.04 (1.91, 2.19） |
| Taiwan (Province of China) | 10 to 14 | T1D | Male | 2.03 (1.90, 2.18） |
| Taiwan (Province of China) | 15 to 19 | T1D | Both | 3.11 (2.97, 3.26） |
| Taiwan (Province of China) | 15 to 19 | T1D | Female | 3.09 (2.89, 3.30） |
| Taiwan (Province of China) | 15 to 19 | T1D | Male | 3.13 (2.93, 3.35） |
| Taiwan (Province of China) | 20 to 24 | T1D | Both | 4.22 (4.02, 4.42） |
| Taiwan (Province of China) | 20 to 24 | T1D | Female | 4.11 (3.85, 4.40） |
| Taiwan (Province of China) | 20 to 24 | T1D | Male | 4.32 (4.04, 4.62） |
| Taiwan (Province of China) | 25 to 29 | T1D | Both | 5.51 (5.26, 5.78） |

| | | | | |
|---|---|---|---|---|
| Taiwan (Province of China) | 25 to 29 | T1D | Female | 5.28 (4.94, 5.65） |
| Taiwan (Province of China) | 25 to 29 | T1D | Male | 5.74 (5.37, 6.14） |
| Taiwan (Province of China) | 30 to 34 | T1D | Both | 6.86 (6.53, 7.19） |
| Taiwan (Province of China) | 30 to 34 | T1D | Female | 6.51 (6.08, 6.97） |
| Taiwan (Province of China) | 30 to 34 | T1D | Male | 7.21 (6.73, 7.72） |
| Taiwan (Province of China) | 35 to 39 | T1D | Both | 8.36 (7.96, 8.78） |
| Taiwan (Province of China) | 35 to 39 | T1D | Female | 7.92 (7.39, 8.48） |
| Taiwan (Province of China) | 35 to 39 | T1D | Male | 8.82 (8.23, 9.46） |
| Taiwan (Province of China) | 40 to 44 | T1D | Both | 10.09 (9.60, 10.61） |
| Taiwan (Province of China) | 40 to 44 | T1D | Female | 9.56 (8.91, 10.26） |
| Taiwan (Province of China) | 40 to 44 | T1D | Male | 10.64 (9.92, 11.42） |
| Taiwan (Province of China) | 45 to 49 | T1D | Both | 12.07 (11.47, 12.69） |
| Taiwan (Province of China) | 45 to 49 | T1D | Female | 11.50 (10.70, 12.36） |
| Taiwan (Province of China) | 45 to 49 | T1D | Male | 12.67 (11.79, 13.61） |
| Taiwan (Province of China) | 5 to 9 | T1D | Both | 1.00 (1.00, 1.00） |
| Taiwan (Province of China) | 5 to 9 | T1D | Female | 1.00 (1.00, 1.00） |
| Taiwan (Province of China) | 5 to 9 | T1D | Male | 1.00 (1.00, 1.00） |
| Taiwan (Province of China) | 50 to 54 | T1D | Both | 14.31 (13.59, 15.07） |
| Taiwan (Province of China) | 50 to 54 | T1D | Female | 13.78 (12.80, 14.82） |
| Taiwan (Province of China) | 50 to 54 | T1D | Male | 14.92 (13.87, 16.05） |
| Taiwan (Province of China) | 55 to 59 | T1D | Both | 16.93 (16.05, 17.84） |
| Taiwan (Province of China) | 55 to 59 | T1D | Female | 16.50 (15.30, 17.78） |
| Taiwan (Province of China) | 55 to 59 | T1D | Male | 17.46 (16.20, 18.81） |
| Taiwan (Province of China) | 60 to 64 | T1D | Both | 19.98 (18.92, 21.10） |
| Taiwan (Province of China) | 60 to 64 | T1D | Female | 19.77 (18.30, 21.35） |
| Taiwan (Province of China) | 60 to 64 | T1D | Male | 20.35 (18.85, 21.98） |
| Taiwan (Province of China) | 65 to 69 | T1D | Both | 23.60 (22.30, 24.97） |
| Taiwan (Province of China) | 65 to 69 | T1D | Female | 23.75 (21.92, 25.73） |
| Taiwan (Province of China) | 65 to 69 | T1D | Male | 23.68 (21.86, 25.64） |
| Tajikistan | 0 to 4 | T1D | Both | 0.16 (0.14, 0.18） |
| Tajikistan | 0 to 4 | T1D | Female | 0.16 (0.14, 0.18） |
| Tajikistan | 0 to 4 | T1D | Male | 0.16 (0.14, 0.18） |
| Tajikistan | 10 to 14 | T1D | Both | 2.23 (2.13, 2.33） |

| | | | | |
|---|---|---|---|---|
| Tajikistan | 10 to 14 | T1D | Female | 2.23 (2.09, 2.38） |
| Tajikistan | 10 to 14 | T1D | Male | 2.23 (2.09, 2.37） |
| Tajikistan | 15 to 19 | T1D | Both | 3.44 (3.29, 3.60） |
| Tajikistan | 15 to 19 | T1D | Female | 3.40 (3.18, 3.62） |
| Tajikistan | 15 to 19 | T1D | Male | 3.48 (3.27, 3.70） |
| Tajikistan | 20 to 24 | T1D | Both | 4.51 (4.30, 4.72） |
| Tajikistan | 20 to 24 | T1D | Female | 4.36 (4.09, 4.66） |
| Tajikistan | 20 to 24 | T1D | Male | 4.64 (4.36, 4.95） |
| Tajikistan | 25 to 29 | T1D | Both | 5.65 (5.39, 5.92） |
| Tajikistan | 25 to 29 | T1D | Female | 5.35 (5.00, 5.73） |
| Tajikistan | 25 to 29 | T1D | Male | 5.94 (5.56, 6.34） |
| Tajikistan | 30 to 34 | T1D | Both | 6.62 (6.30, 6.96） |
| Tajikistan | 30 to 34 | T1D | Female | 6.18 (5.76, 6.63） |
| Tajikistan | 30 to 34 | T1D | Male | 7.05 (6.58, 7.54） |
| Tajikistan | 35 to 39 | T1D | Both | 7.49 (7.12, 7.89） |
| Tajikistan | 35 to 39 | T1D | Female | 6.92 (6.43, 7.46） |
| Tajikistan | 35 to 39 | T1D | Male | 8.05 (7.49, 8.64） |
| Tajikistan | 40 to 44 | T1D | Both | 8.32 (7.88, 8.77） |
| Tajikistan | 40 to 44 | T1D | Female | 7.63 (7.06, 8.24） |
| Tajikistan | 40 to 44 | T1D | Male | 8.99 (8.35, 9.68） |
| Tajikistan | 45 to 49 | T1D | Both | 9.13 (8.63, 9.65） |
| Tajikistan | 45 to 49 | T1D | Female | 8.35 (7.70, 9.05） |
| Tajikistan | 45 to 49 | T1D | Male | 9.89 (9.16, 10.69） |
| Tajikistan | 5 to 9 | T1D | Both | 1.00 (1.00, 1.00） |
| Tajikistan | 5 to 9 | T1D | Female | 1.00 (1.00, 1.00） |
| Tajikistan | 5 to 9 | T1D | Male | 1.00 (1.00, 1.00） |
| Tajikistan | 50 to 54 | T1D | Both | 9.93 (9.36, 10.53） |
| Tajikistan | 50 to 54 | T1D | Female | 9.08 (8.34, 9.88） |
| Tajikistan | 50 to 54 | T1D | Male | 10.78 (9.94, 11.69） |
| Tajikistan | 55 to 59 | T1D | Both | 10.67 (10.02, 11.36） |
| Tajikistan | 55 to 59 | T1D | Female | 9.79 (8.93, 10.72） |
| Tajikistan | 55 to 59 | T1D | Male | 11.56 (10.60, 12.61） |
| Tajikistan | 60 to 64 | T1D | Both | 11.37 (10.62, 12.18） |

| | | | | |
|---|---|---|---|---|
| Tajikistan | 60 to 64 | T1D | Female | 10.48 (9.49, 11.57） |
| Tajikistan | 60 to 64 | T1D | Male | 12.30 (11.19, 13.51） |
| Tajikistan | 65 to 69 | T1D | Both | 12.10 (11.22, 13.06） |
| Tajikistan | 65 to 69 | T1D | Female | 11.24 (10.06, 12.55） |
| Tajikistan | 65 to 69 | T1D | Male | 13.02 (11.73, 14.46） |
| Thailand | 0 to 4 | T1D | Both | 0.15 (0.14, 0.16） |
| Thailand | 0 to 4 | T1D | Female | 0.15 (0.13, 0.16） |
| Thailand | 0 to 4 | T1D | Male | 0.15 (0.13, 0.16） |
| Thailand | 10 to 14 | T1D | Both | 2.08 (2.02, 2.15） |
| Thailand | 10 to 14 | T1D | Female | 2.06 (1.97, 2.15） |
| Thailand | 10 to 14 | T1D | Male | 2.10 (2.01, 2.20） |
| Thailand | 15 to 19 | T1D | Both | 2.96 (2.87, 3.05） |
| Thailand | 15 to 19 | T1D | Female | 2.87 (2.75, 3.00） |
| Thailand | 15 to 19 | T1D | Male | 3.04 (2.91, 3.18） |
| Thailand | 20 to 24 | T1D | Both | 3.70 (3.59, 3.82） |
| Thailand | 20 to 24 | T1D | Female | 3.52 (3.36, 3.67） |
| Thailand | 20 to 24 | T1D | Male | 3.90 (3.73, 4.07） |
| Thailand | 25 to 29 | T1D | Both | 4.47 (4.33, 4.61） |
| Thailand | 25 to 29 | T1D | Female | 4.15 (3.97, 4.34） |
| Thailand | 25 to 29 | T1D | Male | 4.80 (4.59, 5.02） |
| Thailand | 30 to 34 | T1D | Both | 5.07 (4.91, 5.24） |
| Thailand | 30 to 34 | T1D | Female | 4.65 (4.44, 4.86） |
| Thailand | 30 to 34 | T1D | Male | 5.54 (5.29, 5.80） |
| Thailand | 35 to 39 | T1D | Both | 5.64 (5.46, 5.82） |
| Thailand | 35 to 39 | T1D | Female | 5.11 (4.88, 5.35） |
| Thailand | 35 to 39 | T1D | Male | 6.21 (5.93, 6.50） |
| Thailand | 40 to 44 | T1D | Both | 6.19 (5.99, 6.40） |
| Thailand | 40 to 44 | T1D | Female | 5.58 (5.32, 5.84） |
| Thailand | 40 to 44 | T1D | Male | 6.87 (6.55, 7.20） |
| Thailand | 45 to 49 | T1D | Both | 6.75 (6.52, 6.98） |
| Thailand | 45 to 49 | T1D | Female | 6.05 (5.77, 6.35） |
| Thailand | 45 to 49 | T1D | Male | 7.51 (7.16, 7.88） |
| Thailand | 5 to 9 | T1D | Both | 1.00 (1.00, 1.00） |

| | | | | |
|---|---|---|---|---|
| Thailand | 5 to 9 | T1D | Female | 1.00 (1.00, 1.00） |
| Thailand | 5 to 9 | T1D | Male | 1.00 (1.00, 1.00） |
| Thailand | 50 to 54 | T1D | Both | 7.30 (7.05, 7.56） |
| Thailand | 50 to 54 | T1D | Female | 6.54 (6.23, 6.87） |
| Thailand | 50 to 54 | T1D | Male | 8.15 (7.75, 8.56） |
| Thailand | 55 to 59 | T1D | Both | 7.87 (7.60, 8.16） |
| Thailand | 55 to 59 | T1D | Female | 7.06 (6.71, 7.42） |
| Thailand | 55 to 59 | T1D | Male | 8.79 (8.35, 9.25） |
| Thailand | 60 to 64 | T1D | Both | 8.48 (8.16, 8.80） |
| Thailand | 60 to 64 | T1D | Female | 7.61 (7.22, 8.02） |
| Thailand | 60 to 64 | T1D | Male | 9.45 (8.97, 9.97） |
| Thailand | 65 to 69 | T1D | Both | 9.11 (8.76, 9.48） |
| Thailand | 65 to 69 | T1D | Female | 8.21 (7.76, 8.68） |
| Thailand | 65 to 69 | T1D | Male | 10.15 (9.60, 10.73） |
| Timor-Leste | 0 to 4 | T1D | Both | 0.14 (0.09, 0.21） |
| Timor-Leste | 0 to 4 | T1D | Female | 0.14 (0.08, 0.25） |
| Timor-Leste | 0 to 4 | T1D | Male | 0.14 (0.08, 0.26） |
| Timor-Leste | 10 to 14 | T1D | Both | 2.11 (1.75, 2.55） |
| Timor-Leste | 10 to 14 | T1D | Female | 2.11 (1.62, 2.76） |
| Timor-Leste | 10 to 14 | T1D | Male | 2.14 (1.64, 2.80） |
| Timor-Leste | 15 to 19 | T1D | Both | 3.01 (2.49, 3.65） |
| Timor-Leste | 15 to 19 | T1D | Female | 2.90 (2.21, 3.80） |
| Timor-Leste | 15 to 19 | T1D | Male | 3.10 (2.36, 4.07） |
| Timor-Leste | 20 to 24 | T1D | Both | 3.73 (3.05, 4.55） |
| Timor-Leste | 20 to 24 | T1D | Female | 3.52 (2.65, 4.66） |
| Timor-Leste | 20 to 24 | T1D | Male | 3.94 (2.96, 5.23） |
| Timor-Leste | 25 to 29 | T1D | Both | 4.42 (3.59, 5.44） |
| Timor-Leste | 25 to 29 | T1D | Female | 4.04 (3.01, 5.42） |
| Timor-Leste | 25 to 29 | T1D | Male | 4.81 (3.58, 6.46） |
| Timor-Leste | 30 to 34 | T1D | Both | 4.94 (3.97, 6.16） |
| Timor-Leste | 30 to 34 | T1D | Female | 4.48 (3.28, 6.12） |
| Timor-Leste | 30 to 34 | T1D | Male | 5.45 (4.00, 7.43） |
| Timor-Leste | 35 to 39 | T1D | Both | 5.39 (4.28, 6.79） |

| | | | | |
|---|---|---|---|---|
| Timor-Leste | 35 to 39 | T1D | Female | 4.82 (3.47, 6.69） |
| Timor-Leste | 35 to 39 | T1D | Male | 5.99 (4.33, 8.29） |
| Timor-Leste | 40 to 44 | T1D | Both | 5.79 (4.56, 7.36） |
| Timor-Leste | 40 to 44 | T1D | Female | 5.11 (3.63, 7.20） |
| Timor-Leste | 40 to 44 | T1D | Male | 6.48 (4.63, 9.08） |
| Timor-Leste | 45 to 49 | T1D | Both | 6.21 (4.83, 8.00） |
| Timor-Leste | 45 to 49 | T1D | Female | 5.46 (3.80, 7.84） |
| Timor-Leste | 45 to 49 | T1D | Male | 6.95 (4.88, 9.90） |
| Timor-Leste | 5 to 9 | T1D | Both | 1.00 (1.00, 1.00） |
| Timor-Leste | 5 to 9 | T1D | Female | 1.00 (1.00, 1.00） |
| Timor-Leste | 5 to 9 | T1D | Male | 1.00 (1.00, 1.00） |
| Timor-Leste | 50 to 54 | T1D | Both | 6.55 (4.99, 8.59） |
| Timor-Leste | 50 to 54 | T1D | Female | 5.78 (3.91, 8.54） |
| Timor-Leste | 50 to 54 | T1D | Male | 7.38 (5.06, 10.76） |
| Timor-Leste | 55 to 59 | T1D | Both | 6.97 (5.23, 9.29） |
| Timor-Leste | 55 to 59 | T1D | Female | 6.04 (3.99, 9.15） |
| Timor-Leste | 55 to 59 | T1D | Male | 7.65 (5.13, 11.42） |
| Timor-Leste | 60 to 64 | T1D | Both | 7.31 (5.42, 9.86） |
| Timor-Leste | 60 to 64 | T1D | Female | 6.36 (4.15, 9.75） |
| Timor-Leste | 60 to 64 | T1D | Male | 8.11 (5.34, 12.31） |
| Timor-Leste | 65 to 69 | T1D | Both | 7.64 (5.52, 10.58） |
| Timor-Leste | 65 to 69 | T1D | Female | 6.70 (4.21, 10.66） |
| Timor-Leste | 65 to 69 | T1D | Male | 8.39 (5.32, 13.23） |
| Togo | 0 to 4 | T1D | Both | 0.16 (0.14, 0.18） |
| Togo | 0 to 4 | T1D | Female | 0.16 (0.14, 0.19） |
| Togo | 0 to 4 | T1D | Male | 0.16 (0.14, 0.19） |
| Togo | 10 to 14 | T1D | Both | 2.07 (1.97, 2.19） |
| Togo | 10 to 14 | T1D | Female | 2.07 (1.92, 2.24） |
| Togo | 10 to 14 | T1D | Male | 2.08 (1.93, 2.23） |
| Togo | 15 to 19 | T1D | Both | 3.04 (2.88, 3.20） |
| Togo | 15 to 19 | T1D | Female | 3.04 (2.81, 3.28） |
| Togo | 15 to 19 | T1D | Male | 3.04 (2.82, 3.27） |
| Togo | 20 to 24 | T1D | Both | 3.88 (3.67, 4.10） |

| | | | | |
|---|---|---|---|---|
| Togo | 20 to 24 | T1D | Female | 3.89 (3.60, 4.21） |
| Togo | 20 to 24 | T1D | Male | 3.89 (3.60, 4.20） |
| Togo | 25 to 29 | T1D | Both | 4.76 (4.50, 5.04） |
| Togo | 25 to 29 | T1D | Female | 4.79 (4.42, 5.19） |
| Togo | 25 to 29 | T1D | Male | 4.76 (4.40, 5.16） |
| Togo | 30 to 34 | T1D | Both | 5.36 (5.05, 5.68） |
| Togo | 30 to 34 | T1D | Female | 5.39 (4.95, 5.86） |
| Togo | 30 to 34 | T1D | Male | 5.35 (4.92, 5.81） |
| Togo | 35 to 39 | T1D | Both | 5.81 (5.46, 6.18） |
| Togo | 35 to 39 | T1D | Female | 5.85 (5.35, 6.39） |
| Togo | 35 to 39 | T1D | Male | 5.79 (5.31, 6.32） |
| Togo | 40 to 44 | T1D | Both | 6.19 (5.80, 6.61） |
| Togo | 40 to 44 | T1D | Female | 6.24 (5.69, 6.85） |
| Togo | 40 to 44 | T1D | Male | 6.16 (5.63, 6.75） |
| Togo | 45 to 49 | T1D | Both | 6.53 (6.10, 6.99） |
| Togo | 45 to 49 | T1D | Female | 6.59 (5.98, 7.27） |
| Togo | 45 to 49 | T1D | Male | 6.49 (5.89, 7.15） |
| Togo | 5 to 9 | T1D | Both | 1.00 (1.00, 1.00） |
| Togo | 5 to 9 | T1D | Female | 1.00 (1.00, 1.00） |
| Togo | 5 to 9 | T1D | Male | 1.00 (1.00, 1.00） |
| Togo | 50 to 54 | T1D | Both | 6.81 (6.33, 7.33） |
| Togo | 50 to 54 | T1D | Female | 6.88 (6.20, 7.63） |
| Togo | 50 to 54 | T1D | Male | 6.76 (6.09, 7.50） |
| Togo | 55 to 59 | T1D | Both | 7.04 (6.51, 7.63） |
| Togo | 55 to 59 | T1D | Female | 7.13 (6.38, 7.97） |
| Togo | 55 to 59 | T1D | Male | 6.97 (6.23, 7.81） |
| Togo | 60 to 64 | T1D | Both | 7.23 (6.63, 7.88） |
| Togo | 60 to 64 | T1D | Female | 7.33 (6.50, 8.26） |
| Togo | 60 to 64 | T1D | Male | 7.15 (6.31, 8.10） |
| Togo | 65 to 69 | T1D | Both | 7.37 (6.70, 8.12） |
| Togo | 65 to 69 | T1D | Female | 7.49 (6.56, 8.54） |
| Togo | 65 to 69 | T1D | Male | 7.27 (6.31, 8.36） |
| Tokelau | 0 to 4 | T1D | Both | 0.76 (0.02, 33.80） |

| | | | | |
|---|---|---|---|---|
| Tokelau | 0 to 4 | T1D | Female | 0.72 (0.02, 32.28） |
| Tokelau | 0 to 4 | T1D | Male | 0.79 (0.02, 35.14） |
| Tokelau | 10 to 14 | T1D | Both | 1.22 (0.03, 49.82） |
| Tokelau | 10 to 14 | T1D | Female | 1.23 (0.03, 50.36） |
| Tokelau | 10 to 14 | T1D | Male | 1.20 (0.03, 49.17） |
| Tokelau | 15 to 19 | T1D | Both | 1.51 (0.03, 72.47） |
| Tokelau | 15 to 19 | T1D | Female | 1.54 (0.03, 73.96） |
| Tokelau | 15 to 19 | T1D | Male | 1.47 (0.03, 70.66） |
| Tokelau | 20 to 24 | T1D | Both | 1.92 (0.03, 111.65） |
| Tokelau | 20 to 24 | T1D | Female | 1.94 (0.03, 112.77） |
| Tokelau | 20 to 24 | T1D | Male | 1.90 (0.03, 110.56） |
| Tokelau | 25 to 29 | T1D | Both | 2.31 (0.03, 167.85） |
| Tokelau | 25 to 29 | T1D | Female | 2.34 (0.03, 169.62） |
| Tokelau | 25 to 29 | T1D | Male | 2.30 (0.03, 166.66） |
| Tokelau | 30 to 34 | T1D | Both | 2.72 (0.03, 254.25） |
| Tokelau | 30 to 34 | T1D | Female | 2.71 (0.03, 253.81） |
| Tokelau | 30 to 34 | T1D | Male | 2.72 (0.03, 254.71） |
| Tokelau | 35 to 39 | T1D | Both | 3.05 (0.03, 369.96） |
| Tokelau | 35 to 39 | T1D | Female | 3.03 (0.03, 367.40） |
| Tokelau | 35 to 39 | T1D | Male | 3.05 (0.03, 368.97） |
| Tokelau | 40 to 44 | T1D | Both | 3.28 (0.02, 493.81） |
| Tokelau | 40 to 44 | T1D | Female | 3.29 (0.02, 494.44） |
| Tokelau | 40 to 44 | T1D | Male | 3.29 (0.02, 494.65） |
| Tokelau | 45 to 49 | T1D | Both | 3.60 (0.02, 669.90） |
| Tokelau | 45 to 49 | T1D | Female | 3.61 (0.02, 672.65） |
| Tokelau | 45 to 49 | T1D | Male | 3.55 (0.02, 660.68） |
| Tokelau | 5 to 9 | T1D | Both | 1.00 (1.00, 1.00） |
| Tokelau | 5 to 9 | T1D | Female | 1.00 (1.00, 1.00） |
| Tokelau | 5 to 9 | T1D | Male | 1.00 (1.00, 1.00） |
| Tokelau | 50 to 54 | T1D | Both | 3.94 (0.02, 903.91） |
| Tokelau | 50 to 54 | T1D | Female | 4.02 (0.02, 922.57） |
| Tokelau | 50 to 54 | T1D | Male | 3.88 (0.02, 890.21） |
| Tokelau | 55 to 59 | T1D | Both | 4.37 (0.02, 1229.10） |

| | | | | |
|---|---|---|---|---|
| Tokelau | 55 to 59 | T1D | Female | 4.46 (0.02, 1255.80） |
| Tokelau | 55 to 59 | T1D | Male | 4.22 (0.02, 1187.52） |
| Tokelau | 60 to 64 | T1D | Both | 5.02 (0.01, 1736.41） |
| Tokelau | 60 to 64 | T1D | Female | 5.33 (0.02, 1842.45） |
| Tokelau | 60 to 64 | T1D | Male | 4.66 (0.01, 1611.00） |
| Tokelau | 65 to 69 | T1D | Both | 6.44 (0.01, 2863.31） |
| Tokelau | 65 to 69 | T1D | Female | 7.42 (0.02, 3295.00） |
| Tokelau | 65 to 69 | T1D | Male | 5.54 (0.01, 2460.91） |
| Tonga | 0 to 4 | T1D | Both | 0.27 (0.10, 0.72） |
| Tonga | 0 to 4 | T1D | Female | 0.05 (0.01, 0.81） |
| Tonga | 0 to 4 | T1D | Male | 0.05 (0.01, 0.82） |
| Tonga | 10 to 14 | T1D | Both | 1.90 (1.07, 3.39） |
| Tonga | 10 to 14 | T1D | Female | 1.61 (0.74, 3.51） |
| Tonga | 10 to 14 | T1D | Male | 1.63 (0.75, 3.54） |
| Tonga | 15 to 19 | T1D | Both | 2.84 (1.59, 5.08） |
| Tonga | 15 to 19 | T1D | Female | 2.44 (1.12, 5.30） |
| Tonga | 15 to 19 | T1D | Male | 2.51 (1.15, 5.46） |
| Tonga | 20 to 24 | T1D | Both | 3.59 (1.96, 6.58） |
| Tonga | 20 to 24 | T1D | Female | 2.88 (1.28, 6.45） |
| Tonga | 20 to 24 | T1D | Male | 3.18 (1.42, 7.12） |
| Tonga | 25 to 29 | T1D | Both | 4.48 (2.40, 8.38） |
| Tonga | 25 to 29 | T1D | Female | 3.38 (1.46, 7.85） |
| Tonga | 25 to 29 | T1D | Male | 3.85 (1.67, 8.89） |
| Tonga | 30 to 34 | T1D | Both | 5.14 (2.68, 9.86） |
| Tonga | 30 to 34 | T1D | Female | 3.89 (1.62, 9.37） |
| Tonga | 30 to 34 | T1D | Male | 4.60 (1.92, 11.00） |
| Tonga | 35 to 39 | T1D | Both | 5.98 (3.04, 11.76） |
| Tonga | 35 to 39 | T1D | Female | 4.48 (1.79, 11.23） |
| Tonga | 35 to 39 | T1D | Male | 5.20 (2.09, 12.92） |
| Tonga | 40 to 44 | T1D | Both | 6.53 (3.23, 13.19） |
| Tonga | 40 to 44 | T1D | Female | 4.90 (1.87, 12.84） |
| Tonga | 40 to 44 | T1D | Male | 5.92 (2.29, 15.29） |
| Tonga | 45 to 49 | T1D | Both | 7.09 (3.40, 14.78） |

| | | | | |
|---|---|---|---|---|
| Tonga | 45 to 49 | T1D | Female | 5.43 (1.97, 15.01） |
| Tonga | 45 to 49 | T1D | Male | 6.50 (2.41, 17.55） |
| Tonga | 5 to 9 | T1D | Both | 1.00 (1.00, 1.00） |
| Tonga | 5 to 9 | T1D | Female | 1.00 (1.00, 1.00） |
| Tonga | 5 to 9 | T1D | Male | 1.00 (1.00, 1.00） |
| Tonga | 50 to 54 | T1D | Both | 7.57 (3.51, 16.34） |
| Tonga | 50 to 54 | T1D | Female | 6.24 (2.15, 18.11） |
| Tonga | 50 to 54 | T1D | Male | 6.99 (2.45, 19.90） |
| Tonga | 55 to 59 | T1D | Both | 8.52 (3.80, 19.10） |
| Tonga | 55 to 59 | T1D | Female | 6.55 (2.11, 20.29） |
| Tonga | 55 to 59 | T1D | Male | 7.70 (2.55, 23.30） |
| Tonga | 60 to 64 | T1D | Both | 9.35 (3.99, 21.93） |
| Tonga | 60 to 64 | T1D | Female | 7.94 (2.40, 26.33） |
| Tonga | 60 to 64 | T1D | Male | 8.13 (2.49, 26.55） |
| Tonga | 65 to 69 | T1D | Both | 9.89 (3.98, 24.61） |
| Tonga | 65 to 69 | T1D | Female | 8.77 (2.49, 30.88） |
| Tonga | 65 to 69 | T1D | Male | 10.14 (2.94, 35.03） |
| Trinidad and Tobago | 0 to 4 | T1D | Both | 0.16 (0.11, 0.23） |
| Trinidad and Tobago | 0 to 4 | T1D | Female | 0.14 (0.08, 0.23） |
| Trinidad and Tobago | 0 to 4 | T1D | Male | 0.18 (0.11, 0.29） |
| Trinidad and Tobago | 10 to 14 | T1D | Both | 2.17 (1.87, 2.53） |
| Trinidad and Tobago | 10 to 14 | T1D | Female | 2.31 (1.87, 2.85） |
| Trinidad and Tobago | 10 to 14 | T1D | Male | 2.06 (1.66, 2.56） |
| Trinidad and Tobago | 15 to 19 | T1D | Both | 3.26 (2.81, 3.79） |
| Trinidad and Tobago | 15 to 19 | T1D | Female | 3.41 (2.77, 4.20） |
| Trinidad and Tobago | 15 to 19 | T1D | Male | 3.16 (2.56, 3.92） |
| Trinidad and Tobago | 20 to 24 | T1D | Both | 4.19 (3.61, 4.87） |
| Trinidad and Tobago | 20 to 24 | T1D | Female | 4.20 (3.40, 5.18） |
| Trinidad and Tobago | 20 to 24 | T1D | Male | 4.24 (3.43, 5.26） |
| Trinidad and Tobago | 25 to 29 | T1D | Both | 5.18 (4.45, 6.02） |
| Trinidad and Tobago | 25 to 29 | T1D | Female | 4.98 (4.02, 6.16） |
| Trinidad and Tobago | 25 to 29 | T1D | Male | 5.48 (4.41, 6.80） |
| Trinidad and Tobago | 30 to 34 | T1D | Both | 6.05 (5.19, 7.06） |

| | | | | |
|---|---|---|---|---|
| Trinidad and Tobago | 30 to 34 | T1D | Female | 5.64 (4.54, 7.02） |
| Trinidad and Tobago | 30 to 34 | T1D | Male | 6.58 (5.28, 8.20） |
| Trinidad and Tobago | 35 to 39 | T1D | Both | 6.91 (5.90, 8.10） |
| Trinidad and Tobago | 35 to 39 | T1D | Female | 6.30 (5.03, 7.88） |
| Trinidad and Tobago | 35 to 39 | T1D | Male | 7.68 (6.12, 9.62） |
| Trinidad and Tobago | 40 to 44 | T1D | Both | 7.81 (6.64, 9.18） |
| Trinidad and Tobago | 40 to 44 | T1D | Female | 6.98 (5.55, 8.78） |
| Trinidad and Tobago | 40 to 44 | T1D | Male | 8.77 (6.96, 11.04） |
| Trinidad and Tobago | 45 to 49 | T1D | Both | 8.70 (7.37, 10.26） |
| Trinidad and Tobago | 45 to 49 | T1D | Female | 7.68 (6.07, 9.71） |
| Trinidad and Tobago | 45 to 49 | T1D | Male | 9.88 (7.81, 12.49） |
| Trinidad and Tobago | 5 to 9 | T1D | Both | 1.00 (1.00, 1.00） |
| Trinidad and Tobago | 5 to 9 | T1D | Female | 1.00 (1.00, 1.00） |
| Trinidad and Tobago | 5 to 9 | T1D | Male | 1.00 (1.00, 1.00） |
| Trinidad and Tobago | 50 to 54 | T1D | Both | 9.57 (8.08, 11.34） |
| Trinidad and Tobago | 50 to 54 | T1D | Female | 8.37 (6.58, 10.65） |
| Trinidad and Tobago | 50 to 54 | T1D | Male | 10.94 (8.61, 13.91） |
| Trinidad and Tobago | 55 to 59 | T1D | Both | 10.46 (8.78, 12.46） |
| Trinidad and Tobago | 55 to 59 | T1D | Female | 9.12 (7.11, 11.69） |
| Trinidad and Tobago | 55 to 59 | T1D | Male | 12.03 (9.40, 15.40） |
| Trinidad and Tobago | 60 to 64 | T1D | Both | 11.36 (9.48, 13.62） |
| Trinidad and Tobago | 60 to 64 | T1D | Female | 9.88 (7.63, 12.79） |
| Trinidad and Tobago | 60 to 64 | T1D | Male | 13.05 (10.11, 16.85） |
| Trinidad and Tobago | 65 to 69 | T1D | Both | 12.30 (10.18, 14.87） |
| Trinidad and Tobago | 65 to 69 | T1D | Female | 10.70 (8.16, 14.02） |
| Trinidad and Tobago | 65 to 69 | T1D | Male | 14.15 (10.83, 18.48） |
| Tunisia | 0 to 4 | T1D | Both | 0.14 (0.13, 0.16） |
| Tunisia | 0 to 4 | T1D | Female | 0.14 (0.12, 0.16） |
| Tunisia | 0 to 4 | T1D | Male | 0.14 (0.13, 0.16） |
| Tunisia | 10 to 14 | T1D | Both | 2.37 (2.27, 2.47） |
| Tunisia | 10 to 14 | T1D | Female | 2.53 (2.37, 2.69） |
| Tunisia | 10 to 14 | T1D | Male | 2.24 (2.12, 2.37） |
| Tunisia | 15 to 19 | T1D | Both | 3.74 (3.59, 3.90） |

| | | | | |
|---|---|---|---|---|
| Tunisia | 15 to 19 | T1D | Female | 3.99 (3.75, 4.25） |
| Tunisia | 15 to 19 | T1D | Male | 3.55 (3.36, 3.75） |
| Tunisia | 20 to 24 | T1D | Both | 5.04 (4.83, 5.26） |
| Tunisia | 20 to 24 | T1D | Female | 5.27 (4.95, 5.62） |
| Tunisia | 20 to 24 | T1D | Male | 4.88 (4.61, 5.17） |
| Tunisia | 25 to 29 | T1D | Both | 6.58 (6.30, 6.87） |
| Tunisia | 25 to 29 | T1D | Female | 6.74 (6.32, 7.19） |
| Tunisia | 25 to 29 | T1D | Male | 6.51 (6.14, 6.89） |
| Tunisia | 30 to 34 | T1D | Both | 8.23 (7.88, 8.61） |
| Tunisia | 30 to 34 | T1D | Female | 8.30 (7.77, 8.86） |
| Tunisia | 30 to 34 | T1D | Male | 8.28 (7.80, 8.78） |
| Tunisia | 35 to 39 | T1D | Both | 10.09 (9.64, 10.56） |
| Tunisia | 35 to 39 | T1D | Female | 10.04 (9.38, 10.74） |
| Tunisia | 35 to 39 | T1D | Male | 10.26 (9.65, 10.91） |
| Tunisia | 40 to 44 | T1D | Both | 12.23 (11.67, 12.82） |
| Tunisia | 40 to 44 | T1D | Female | 12.05 (11.23, 12.92） |
| Tunisia | 40 to 44 | T1D | Male | 12.52 (11.76, 13.34） |
| Tunisia | 45 to 49 | T1D | Both | 14.68 (13.99, 15.41） |
| Tunisia | 45 to 49 | T1D | Female | 14.37 (13.36, 15.44） |
| Tunisia | 45 to 49 | T1D | Male | 15.11 (14.16, 16.12） |
| Tunisia | 5 to 9 | T1D | Both | 1.00 (1.00, 1.00） |
| Tunisia | 5 to 9 | T1D | Female | 1.00 (1.00, 1.00） |
| Tunisia | 5 to 9 | T1D | Male | 1.00 (1.00, 1.00） |
| Tunisia | 50 to 54 | T1D | Both | 17.47 (16.62, 18.37） |
| Tunisia | 50 to 54 | T1D | Female | 17.03 (15.81, 18.36） |
| Tunisia | 50 to 54 | T1D | Male | 18.04 (16.87, 19.29） |
| Tunisia | 55 to 59 | T1D | Both | 20.65 (19.61, 21.76） |
| Tunisia | 55 to 59 | T1D | Female | 20.12 (18.61, 21.75） |
| Tunisia | 55 to 59 | T1D | Male | 21.37 (19.93, 22.92） |
| Tunisia | 60 to 64 | T1D | Both | 24.36 (23.07, 25.73） |
| Tunisia | 60 to 64 | T1D | Female | 23.73 (21.87, 25.76） |
| Tunisia | 60 to 64 | T1D | Male | 25.18 (23.40, 27.10） |
| Tunisia | 65 to 69 | T1D | Both | 28.70 (27.10, 30.40） |

| | | | | |
|---|---|---|---|---|
| Tunisia | 65 to 69 | T1D | Female | 27.99 (25.67, 30.51） |
| Tunisia | 65 to 69 | T1D | Male | 29.60 (27.40, 31.98） |
| Turkey | 0 to 4 | T1D | Both | 0.15 (0.14, 0.15） |
| Turkey | 0 to 4 | T1D | Female | 0.16 (0.15, 0.16） |
| Turkey | 0 to 4 | T1D | Male | 0.14 (0.14, 0.15） |
| Turkey | 10 to 14 | T1D | Both | 2.32 (2.29, 2.36） |
| Turkey | 10 to 14 | T1D | Female | 2.43 (2.38, 2.49） |
| Turkey | 10 to 14 | T1D | Male | 2.23 (2.19, 2.28） |
| Turkey | 15 to 19 | T1D | Both | 3.63 (3.58, 3.69） |
| Turkey | 15 to 19 | T1D | Female | 3.86 (3.77, 3.95） |
| Turkey | 15 to 19 | T1D | Male | 3.45 (3.38, 3.53） |
| Turkey | 20 to 24 | T1D | Both | 4.85 (4.77, 4.92） |
| Turkey | 20 to 24 | T1D | Female | 5.09 (4.98, 5.21） |
| Turkey | 20 to 24 | T1D | Male | 4.65 (4.55, 4.75） |
| Turkey | 25 to 29 | T1D | Both | 6.24 (6.14, 6.34） |
| Turkey | 25 to 29 | T1D | Female | 6.47 (6.32, 6.63） |
| Turkey | 25 to 29 | T1D | Male | 6.06 (5.93, 6.19） |
| Turkey | 30 to 34 | T1D | Both | 7.65 (7.53, 7.78） |
| Turkey | 30 to 34 | T1D | Female | 7.86 (7.67, 8.05） |
| Turkey | 30 to 34 | T1D | Male | 7.49 (7.32, 7.65） |
| Turkey | 35 to 39 | T1D | Both | 9.14 (8.99, 9.29） |
| Turkey | 35 to 39 | T1D | Female | 9.32 (9.09, 9.56） |
| Turkey | 35 to 39 | T1D | Male | 9.00 (8.79, 9.21） |
| Turkey | 40 to 44 | T1D | Both | 10.75 (10.57, 10.94） |
| Turkey | 40 to 44 | T1D | Female | 10.91 (10.63, 11.19） |
| Turkey | 40 to 44 | T1D | Male | 10.64 (10.39, 10.89） |
| Turkey | 45 to 49 | T1D | Both | 12.50 (12.28, 12.73） |
| Turkey | 45 to 49 | T1D | Female | 12.66 (12.33, 13.00） |
| Turkey | 45 to 49 | T1D | Male | 12.40 (12.10, 12.70） |
| Turkey | 5 to 9 | T1D | Both | 1.00 (1.00, 1.00） |
| Turkey | 5 to 9 | T1D | Female | 1.00 (1.00, 1.00） |
| Turkey | 5 to 9 | T1D | Male | 1.00 (1.00, 1.00） |
| Turkey | 50 to 54 | T1D | Both | 14.41 (14.15, 14.68） |

| | | | | |
|---|---|---|---|---|
| Turkey | 50 to 54 | T1D | Female | 14.59 (14.20, 15.00） |
| Turkey | 50 to 54 | T1D | Male | 14.31 (13.95, 14.67） |
| Turkey | 55 to 59 | T1D | Both | 16.52 (16.20, 16.84） |
| Turkey | 55 to 59 | T1D | Female | 16.76 (16.29, 17.24） |
| Turkey | 55 to 59 | T1D | Male | 16.40 (15.97, 16.83） |
| Turkey | 60 to 64 | T1D | Both | 18.83 (18.46, 19.21） |
| Turkey | 60 to 64 | T1D | Female | 19.18 (18.62, 19.75） |
| Turkey | 60 to 64 | T1D | Male | 18.68 (18.18, 19.20） |
| Turkey | 65 to 69 | T1D | Both | 21.42 (20.97, 21.88） |
| Turkey | 65 to 69 | T1D | Female | 21.91 (21.24, 22.60） |
| Turkey | 65 to 69 | T1D | Male | 21.23 (20.62, 21.86） |
| Turkmenistan | 0 to 4 | T1D | Both | 0.16 (0.14, 0.18） |
| Turkmenistan | 0 to 4 | T1D | Female | 0.16 (0.13, 0.19） |
| Turkmenistan | 0 to 4 | T1D | Male | 0.16 (0.13, 0.19） |
| Turkmenistan | 10 to 14 | T1D | Both | 2.22 (2.09, 2.36） |
| Turkmenistan | 10 to 14 | T1D | Female | 2.23 (2.04, 2.43） |
| Turkmenistan | 10 to 14 | T1D | Male | 2.22 (2.04, 2.41） |
| Turkmenistan | 15 to 19 | T1D | Both | 3.42 (3.22, 3.63） |
| Turkmenistan | 15 to 19 | T1D | Female | 3.37 (3.09, 3.68） |
| Turkmenistan | 15 to 19 | T1D | Male | 3.45 (3.17, 3.75） |
| Turkmenistan | 20 to 24 | T1D | Both | 4.48 (4.21, 4.76） |
| Turkmenistan | 20 to 24 | T1D | Female | 4.33 (3.96, 4.73） |
| Turkmenistan | 20 to 24 | T1D | Male | 4.60 (4.22, 5.00） |
| Turkmenistan | 25 to 29 | T1D | Both | 5.61 (5.27, 5.98） |
| Turkmenistan | 25 to 29 | T1D | Female | 5.30 (4.84, 5.80） |
| Turkmenistan | 25 to 29 | T1D | Male | 5.87 (5.38, 6.40） |
| Turkmenistan | 30 to 34 | T1D | Both | 6.58 (6.17, 7.02） |
| Turkmenistan | 30 to 34 | T1D | Female | 6.12 (5.57, 6.72） |
| Turkmenistan | 30 to 34 | T1D | Male | 6.96 (6.37, 7.61） |
| Turkmenistan | 35 to 39 | T1D | Both | 7.44 (6.96, 7.95） |
| Turkmenistan | 35 to 39 | T1D | Female | 6.86 (6.22, 7.55） |
| Turkmenistan | 35 to 39 | T1D | Male | 7.94 (7.24, 8.71） |
| Turkmenistan | 40 to 44 | T1D | Both | 8.25 (7.70, 8.84） |

| | | | | |
|---|---|---|---|---|
| Turkmenistan | 40 to 44 | T1D | Female | 7.56 (6.84, 8.36） |
| Turkmenistan | 40 to 44 | T1D | Male | 8.86 (8.06, 9.74） |
| Turkmenistan | 45 to 49 | T1D | Both | 9.06 (8.43, 9.72） |
| Turkmenistan | 45 to 49 | T1D | Female | 8.29 (7.48, 9.19） |
| Turkmenistan | 45 to 49 | T1D | Male | 9.76 (8.85, 10.77） |
| Turkmenistan | 5 to 9 | T1D | Both | 1.00 (1.00, 1.00） |
| Turkmenistan | 5 to 9 | T1D | Female | 1.00 (1.00, 1.00） |
| Turkmenistan | 5 to 9 | T1D | Male | 1.00 (1.00, 1.00） |
| Turkmenistan | 50 to 54 | T1D | Both | 9.87 (9.16, 10.63） |
| Turkmenistan | 50 to 54 | T1D | Female | 9.04 (8.12, 10.06） |
| Turkmenistan | 50 to 54 | T1D | Male | 10.64 (9.60, 11.79） |
| Turkmenistan | 55 to 59 | T1D | Both | 10.63 (9.83, 11.49） |
| Turkmenistan | 55 to 59 | T1D | Female | 9.78 (8.73, 10.95） |
| Turkmenistan | 55 to 59 | T1D | Male | 11.46 (10.28, 12.77） |
| Turkmenistan | 60 to 64 | T1D | Both | 11.35 (10.44, 12.34） |
| Turkmenistan | 60 to 64 | T1D | Female | 10.49 (9.30, 11.84） |
| Turkmenistan | 60 to 64 | T1D | Male | 12.22 (10.88, 13.73） |
| Turkmenistan | 65 to 69 | T1D | Both | 12.10 (11.05, 13.26） |
| Turkmenistan | 65 to 69 | T1D | Female | 11.29 (9.91, 12.87） |
| Turkmenistan | 65 to 69 | T1D | Male | 13.02 (11.46, 14.80） |
| Tuvalu | 0 to 4 | T1D | Both | 0.96 (0.02, 41.68） |
| Tuvalu | 0 to 4 | T1D | Female | 0.99 (0.02, 44.38） |
| Tuvalu | 0 to 4 | T1D | Male | 1.07 (0.02, 47.42） |
| Tuvalu | 10 to 14 | T1D | Both | 7.89 (0.43, 144.17） |
| Tuvalu | 10 to 14 | T1D | Female | 1.09 (0.03, 44.50） |
| Tuvalu | 10 to 14 | T1D | Male | 1.18 (0.03, 47.69） |
| Tuvalu | 15 to 19 | T1D | Both | 12.21 (0.71, 210.20） |
| Tuvalu | 15 to 19 | T1D | Female | 1.16 (0.02, 55.94） |
| Tuvalu | 15 to 19 | T1D | Male | 1.64 (0.04, 76.17） |
| Tuvalu | 20 to 24 | T1D | Both | 14.78 (0.81, 269.18） |
| Tuvalu | 20 to 24 | T1D | Female | 1.23 (0.02, 71.48） |
| Tuvalu | 20 to 24 | T1D | Male | 4.18 (0.12, 147.52） |
| Tuvalu | 25 to 29 | T1D | Both | 16.04 (0.83, 308.95） |

| | | | | |
|---|---|---|---|---|
| Tuvalu | 25 to 29 | T1D | Female | 1.28 (0.02, 93.15） |
| Tuvalu | 25 to 29 | T1D | Male | 6.41 (0.17, 242.00） |
| Tuvalu | 30 to 34 | T1D | Both | 16.85 (0.84, 339.46） |
| Tuvalu | 30 to 34 | T1D | Female | 1.35 (0.01, 126.08） |
| Tuvalu | 30 to 34 | T1D | Male | 9.21 (0.21, 410.27） |
| Tuvalu | 35 to 39 | T1D | Both | 17.54 (0.83, 369.14） |
| Tuvalu | 35 to 39 | T1D | Female | 1.42 (0.01, 171.97） |
| Tuvalu | 35 to 39 | T1D | Male | 12.99 (0.22, 770.25） |
| Tuvalu | 40 to 44 | T1D | Both | 18.02 (0.82, 393.64） |
| Tuvalu | 40 to 44 | T1D | Female | 1.50 (0.01, 225.31） |
| Tuvalu | 40 to 44 | T1D | Male | 8.92 (0.09, 902.66） |
| Tuvalu | 45 to 49 | T1D | Both | 19.46 (0.86, 438.58） |
| Tuvalu | 45 to 49 | T1D | Female | 1.60 (0.01, 298.45） |
| Tuvalu | 45 to 49 | T1D | Male | 9.86 (0.08, 1227.69） |
| Tuvalu | 5 to 9 | T1D | Both | 1.00 (1.00, 1.00） |
| Tuvalu | 5 to 9 | T1D | Female | 1.00 (1.00, 1.00） |
| Tuvalu | 5 to 9 | T1D | Male | 1.00 (1.00, 1.00） |
| Tuvalu | 50 to 54 | T1D | Both | 20.54 (0.88, 481.20） |
| Tuvalu | 50 to 54 | T1D | Female | 1.72 (0.01, 394.39） |
| Tuvalu | 50 to 54 | T1D | Male | 10.59 (0.07, 1632.33） |
| Tuvalu | 55 to 59 | T1D | Both | 22.63 (0.91, 561.68） |
| Tuvalu | 55 to 59 | T1D | Female | 1.90 (0.01, 533.57） |
| Tuvalu | 55 to 59 | T1D | Male | 11.71 (0.06, 2226.81） |
| Tuvalu | 60 to 64 | T1D | Both | 22.55 (0.77, 662.20） |
| Tuvalu | 60 to 64 | T1D | Female | 2.18 (0.01, 754.20） |
| Tuvalu | 60 to 64 | T1D | Male | 13.64 (0.06, 3206.20） |
| Tuvalu | 65 to 69 | T1D | Both | 24.55 (0.65, 921.62） |
| Tuvalu | 65 to 69 | T1D | Female | 2.56 (0.01, 1137.82） |
| Tuvalu | 65 to 69 | T1D | Male | 16.97 (0.06, 5120.49） |
| Uganda | 0 to 4 | T1D | Both | 0.15 (0.15, 0.16） |
| Uganda | 0 to 4 | T1D | Female | 0.15 (0.14, 0.16） |
| Uganda | 0 to 4 | T1D | Male | 0.15 (0.14, 0.16） |
| Uganda | 10 to 14 | T1D | Both | 2.05 (2.00, 2.10） |

| | | | | |
|---|---|---|---|---|
| Uganda | 10 to 14 | T1D | Female | 2.05 (1.98, 2.11） |
| Uganda | 10 to 14 | T1D | Male | 2.05 (1.99, 2.12） |
| Uganda | 15 to 19 | T1D | Both | 3.00 (2.93, 3.07） |
| Uganda | 15 to 19 | T1D | Female | 3.00 (2.90, 3.10） |
| Uganda | 15 to 19 | T1D | Male | 3.01 (2.91, 3.11） |
| Uganda | 20 to 24 | T1D | Both | 3.89 (3.79, 3.98） |
| Uganda | 20 to 24 | T1D | Female | 3.89 (3.76, 4.03） |
| Uganda | 20 to 24 | T1D | Male | 3.89 (3.76, 4.02） |
| Uganda | 25 to 29 | T1D | Both | 4.78 (4.66, 4.90） |
| Uganda | 25 to 29 | T1D | Female | 4.80 (4.64, 4.98） |
| Uganda | 25 to 29 | T1D | Male | 4.77 (4.61, 4.94） |
| Uganda | 30 to 34 | T1D | Both | 5.37 (5.23, 5.51） |
| Uganda | 30 to 34 | T1D | Female | 5.40 (5.20, 5.61） |
| Uganda | 30 to 34 | T1D | Male | 5.34 (5.15, 5.55） |
| Uganda | 35 to 39 | T1D | Both | 5.80 (5.64, 5.97） |
| Uganda | 35 to 39 | T1D | Female | 5.85 (5.62, 6.09） |
| Uganda | 35 to 39 | T1D | Male | 5.77 (5.54, 6.00） |
| Uganda | 40 to 44 | T1D | Both | 6.18 (6.00, 6.36） |
| Uganda | 40 to 44 | T1D | Female | 6.24 (5.98, 6.51） |
| Uganda | 40 to 44 | T1D | Male | 6.12 (5.87, 6.39） |
| Uganda | 45 to 49 | T1D | Both | 6.49 (6.29, 6.71） |
| Uganda | 45 to 49 | T1D | Female | 6.58 (6.29, 6.88） |
| Uganda | 45 to 49 | T1D | Male | 6.42 (6.14, 6.72） |
| Uganda | 5 to 9 | T1D | Both | 1.00 (1.00, 1.00） |
| Uganda | 5 to 9 | T1D | Female | 1.00 (1.00, 1.00） |
| Uganda | 5 to 9 | T1D | Male | 1.00 (1.00, 1.00） |
| Uganda | 50 to 54 | T1D | Both | 6.77 (6.54, 7.00） |
| Uganda | 50 to 54 | T1D | Female | 6.87 (6.55, 7.22） |
| Uganda | 50 to 54 | T1D | Male | 6.68 (6.36, 7.01） |
| Uganda | 55 to 59 | T1D | Both | 7.00 (6.75, 7.27） |
| Uganda | 55 to 59 | T1D | Female | 7.13 (6.77, 7.52） |
| Uganda | 55 to 59 | T1D | Male | 6.89 (6.54, 7.27） |
| Uganda | 60 to 64 | T1D | Both | 7.21 (6.92, 7.52） |

| | | | | |
|---|---|---|---|---|
| Uganda | 60 to 64 | T1D | Female | 7.36 (6.95, 7.80） |
| Uganda | 60 to 64 | T1D | Male | 7.09 (6.68, 7.51） |
| Uganda | 65 to 69 | T1D | Both | 7.39 (7.06, 7.74） |
| Uganda | 65 to 69 | T1D | Female | 7.57 (7.10, 8.06） |
| Uganda | 65 to 69 | T1D | Male | 7.24 (6.78, 7.74） |
| Ukraine | 0 to 4 | T1D | Both | 0.15 (0.15, 0.16） |
| Ukraine | 0 to 4 | T1D | Female | 0.15 (0.14, 0.17） |
| Ukraine | 0 to 4 | T1D | Male | 0.15 (0.14, 0.17） |
| Ukraine | 10 to 14 | T1D | Both | 2.29 (2.23, 2.35） |
| Ukraine | 10 to 14 | T1D | Female | 2.30 (2.21, 2.39） |
| Ukraine | 10 to 14 | T1D | Male | 2.29 (2.20, 2.37） |
| Ukraine | 15 to 19 | T1D | Both | 3.53 (3.44, 3.62） |
| Ukraine | 15 to 19 | T1D | Female | 3.53 (3.40, 3.67） |
| Ukraine | 15 to 19 | T1D | Male | 3.53 (3.40, 3.66） |
| Ukraine | 20 to 24 | T1D | Both | 4.66 (4.54, 4.78） |
| Ukraine | 20 to 24 | T1D | Female | 4.62 (4.45, 4.79） |
| Ukraine | 20 to 24 | T1D | Male | 4.70 (4.53, 4.87） |
| Ukraine | 25 to 29 | T1D | Both | 5.92 (5.76, 6.07） |
| Ukraine | 25 to 29 | T1D | Female | 5.80 (5.59, 6.02） |
| Ukraine | 25 to 29 | T1D | Male | 6.04 (5.82, 6.27） |
| Ukraine | 30 to 34 | T1D | Both | 7.01 (6.82, 7.20） |
| Ukraine | 30 to 34 | T1D | Female | 6.83 (6.58, 7.09） |
| Ukraine | 30 to 34 | T1D | Male | 7.19 (6.93, 7.47） |
| Ukraine | 35 to 39 | T1D | Both | 8.06 (7.85, 8.29） |
| Ukraine | 35 to 39 | T1D | Female | 7.84 (7.55, 8.15） |
| Ukraine | 35 to 39 | T1D | Male | 8.30 (7.99, 8.62） |
| Ukraine | 40 to 44 | T1D | Both | 9.15 (8.90, 9.40） |
| Ukraine | 40 to 44 | T1D | Female | 8.92 (8.58, 9.27） |
| Ukraine | 40 to 44 | T1D | Male | 9.41 (9.05, 9.78） |
| Ukraine | 45 to 49 | T1D | Both | 10.25 (9.97, 10.54） |
| Ukraine | 45 to 49 | T1D | Female | 10.04 (9.66, 10.44） |
| Ukraine | 45 to 49 | T1D | Male | 10.51 (10.11, 10.93） |
| Ukraine | 5 to 9 | T1D | Both | 1.00 (1.00, 1.00） |

| | | | | |
|---|---|---|---|---|
| Ukraine | 5 to 9 | T1D | Female | 1.00 (1.00, 1.00） |
| Ukraine | 5 to 9 | T1D | Male | 1.00 (1.00, 1.00） |
| Ukraine | 50 to 54 | T1D | Both | 11.37 (11.06, 11.69） |
| Ukraine | 50 to 54 | T1D | Female | 11.22 (10.78, 11.67） |
| Ukraine | 50 to 54 | T1D | Male | 11.59 (11.14, 12.06） |
| Ukraine | 55 to 59 | T1D | Both | 12.52 (12.17, 12.88） |
| Ukraine | 55 to 59 | T1D | Female | 12.48 (11.98, 12.99） |
| Ukraine | 55 to 59 | T1D | Male | 12.66 (12.16, 13.18） |
| Ukraine | 60 to 64 | T1D | Both | 13.72 (13.33, 14.12） |
| Ukraine | 60 to 64 | T1D | Female | 13.83 (13.28, 14.40） |
| Ukraine | 60 to 64 | T1D | Male | 13.71 (13.16, 14.29） |
| Ukraine | 65 to 69 | T1D | Both | 14.98 (14.55, 15.43） |
| Ukraine | 65 to 69 | T1D | Female | 15.30 (14.68, 15.95） |
| Ukraine | 65 to 69 | T1D | Male | 14.76 (14.15, 15.40） |
| United Arab Emirates | 0 to 4 | T1D | Both | 0.17 (0.14, 0.19） |
| United Arab Emirates | 0 to 4 | T1D | Female | 0.17 (0.14, 0.22） |
| United Arab Emirates | 0 to 4 | T1D | Male | 0.16 (0.13, 0.19） |
| United Arab Emirates | 10 to 14 | T1D | Both | 2.29 (2.13, 2.46） |
| United Arab Emirates | 10 to 14 | T1D | Female | 2.37 (2.13, 2.65） |
| United Arab Emirates | 10 to 14 | T1D | Male | 2.21 (2.00, 2.44） |
| United Arab Emirates | 15 to 19 | T1D | Both | 3.67 (3.41, 3.95） |
| United Arab Emirates | 15 to 19 | T1D | Female | 3.85 (3.45, 4.30） |
| United Arab Emirates | 15 to 19 | T1D | Male | 3.51 (3.18, 3.88） |
| United Arab Emirates | 20 to 24 | T1D | Both | 5.10 (4.74, 5.48） |
| United Arab Emirates | 20 to 24 | T1D | Female | 5.27 (4.71, 5.89） |
| United Arab Emirates | 20 to 24 | T1D | Male | 4.87 (4.42, 5.37） |
| United Arab Emirates | 25 to 29 | T1D | Both | 6.80 (6.32, 7.31） |
| United Arab Emirates | 25 to 29 | T1D | Female | 6.90 (6.17, 7.72） |
| United Arab Emirates | 25 to 29 | T1D | Male | 6.49 (5.89, 7.15） |
| United Arab Emirates | 30 to 34 | T1D | Both | 8.68 (8.06, 9.35） |
| United Arab Emirates | 30 to 34 | T1D | Female | 8.65 (7.71, 9.70） |
| United Arab Emirates | 30 to 34 | T1D | Male | 8.31 (7.53, 9.17） |
| United Arab Emirates | 35 to 39 | T1D | Both | 10.85 (10.05, 11.71） |

| | | | | |
|---|---|---|---|---|
| United Arab Emirates | 35 to 39 | T1D | Female | 10.72 (9.51, 12.08） |
| United Arab Emirates | 35 to 39 | T1D | Male | 10.38 (9.39, 11.48） |
| United Arab Emirates | 40 to 44 | T1D | Both | 13.39 (12.38, 14.48） |
| United Arab Emirates | 40 to 44 | T1D | Female | 13.06 (11.52, 14.80） |
| United Arab Emirates | 40 to 44 | T1D | Male | 12.81 (11.56, 14.20） |
| United Arab Emirates | 45 to 49 | T1D | Both | 16.27 (15.01, 17.63） |
| United Arab Emirates | 45 to 49 | T1D | Female | 15.77 (13.83, 17.99） |
| United Arab Emirates | 45 to 49 | T1D | Male | 15.58 (14.02, 17.31） |
| United Arab Emirates | 5 to 9 | T1D | Both | 1.00 (1.00, 1.00） |
| United Arab Emirates | 5 to 9 | T1D | Female | 1.00 (1.00, 1.00） |
| United Arab Emirates | 5 to 9 | T1D | Male | 1.00 (1.00, 1.00） |
| United Arab Emirates | 50 to 54 | T1D | Both | 19.56 (17.99, 21.27） |
| United Arab Emirates | 50 to 54 | T1D | Female | 18.94 (16.47, 21.78） |
| United Arab Emirates | 50 to 54 | T1D | Male | 18.77 (16.84, 20.92） |
| United Arab Emirates | 55 to 59 | T1D | Both | 23.23 (21.26, 25.39） |
| United Arab Emirates | 55 to 59 | T1D | Female | 22.61 (19.42, 26.32） |
| United Arab Emirates | 55 to 59 | T1D | Male | 22.31 (19.91, 24.99） |
| United Arab Emirates | 60 to 64 | T1D | Both | 27.52 (24.98, 30.32） |
| United Arab Emirates | 60 to 64 | T1D | Female | 26.93 (22.71, 31.93） |
| United Arab Emirates | 60 to 64 | T1D | Male | 26.48 (23.44, 29.92） |
| United Arab Emirates | 65 to 69 | T1D | Both | 32.52 (28.99, 36.48） |
| United Arab Emirates | 65 to 69 | T1D | Female | 32.12 (26.20, 39.39） |
| United Arab Emirates | 65 to 69 | T1D | Male | 31.52 (27.32, 36.36） |
| United Kingdom | 0 to 4 | T1D | Both | 0.18 (0.16, 0.19） |
| United Kingdom | 0 to 4 | T1D | Female | 0.18 (0.17, 0.18） |
| United Kingdom | 0 to 4 | T1D | Male | 0.18 (0.16, 0.19） |
| United Kingdom | 10 to 14 | T1D | Both | 2.08 (2.02, 2.14） |
| United Kingdom | 10 to 14 | T1D | Female | 2.04 (2.00, 2.09） |
| United Kingdom | 10 to 14 | T1D | Male | 2.11 (2.02, 2.21） |
| United Kingdom | 15 to 19 | T1D | Both | 3.07 (2.98, 3.17） |
| United Kingdom | 15 to 19 | T1D | Female | 2.96 (2.90, 3.02） |
| United Kingdom | 15 to 19 | T1D | Male | 3.19 (3.05, 3.33） |
| United Kingdom | 20 to 24 | T1D | Both | 4.03 (3.91, 4.15） |

| | | | | |
|---|---|---|---|---|
| United Kingdom | 20 to 24 | T1D | Female | 3.79 (3.71, 3.87） |
| United Kingdom | 20 to 24 | T1D | Male | 4.26 (4.08, 4.45） |
| United Kingdom | 25 to 29 | T1D | Both | 5.13 (4.97, 5.29） |
| United Kingdom | 25 to 29 | T1D | Female | 4.73 (4.63, 4.83） |
| United Kingdom | 25 to 29 | T1D | Male | 5.54 (5.30, 5.80） |
| United Kingdom | 30 to 34 | T1D | Both | 6.07 (5.88, 6.26） |
| United Kingdom | 30 to 34 | T1D | Female | 5.51 (5.39, 5.63） |
| United Kingdom | 30 to 34 | T1D | Male | 6.66 (6.37, 6.97） |
| United Kingdom | 35 to 39 | T1D | Both | 6.99 (6.77, 7.21） |
| United Kingdom | 35 to 39 | T1D | Female | 6.27 (6.13, 6.41） |
| United Kingdom | 35 to 39 | T1D | Male | 7.76 (7.41, 8.12） |
| United Kingdom | 40 to 44 | T1D | Both | 7.95 (7.70, 8.21） |
| United Kingdom | 40 to 44 | T1D | Female | 7.07 (6.91, 7.23） |
| United Kingdom | 40 to 44 | T1D | Male | 8.91 (8.50, 9.33） |
| United Kingdom | 45 to 49 | T1D | Both | 8.94 (8.65, 9.23） |
| United Kingdom | 45 to 49 | T1D | Female | 7.89 (7.71, 8.07） |
| United Kingdom | 45 to 49 | T1D | Male | 10.08 (9.62, 10.57） |
| United Kingdom | 5 to 9 | T1D | Both | 1.00 (1.00, 1.00） |
| United Kingdom | 5 to 9 | T1D | Female | 1.00 (1.00, 1.00） |
| United Kingdom | 5 to 9 | T1D | Male | 1.00 (1.00, 1.00） |
| United Kingdom | 50 to 54 | T1D | Both | 9.93 (9.61, 10.27） |
| United Kingdom | 50 to 54 | T1D | Female | 8.73 (8.53, 8.94） |
| United Kingdom | 50 to 54 | T1D | Male | 11.25 (10.72, 11.80） |
| United Kingdom | 55 to 59 | T1D | Both | 10.96 (10.60, 11.34） |
| United Kingdom | 55 to 59 | T1D | Female | 9.60 (9.38, 9.83） |
| United Kingdom | 55 to 59 | T1D | Male | 12.45 (11.86, 13.08） |
| United Kingdom | 60 to 64 | T1D | Both | 12.02 (11.61, 12.44） |
| United Kingdom | 60 to 64 | T1D | Female | 10.52 (10.27, 10.78） |
| United Kingdom | 60 to 64 | T1D | Male | 13.68 (13.01, 14.37） |
| United Kingdom | 65 to 69 | T1D | Both | 13.10 (12.65, 13.57） |
| United Kingdom | 65 to 69 | T1D | Female | 11.47 (11.19, 11.76） |
| United Kingdom | 65 to 69 | T1D | Male | 14.92 (14.18, 15.69） |
| United Republic of Tanzania | 0 to 4 | T1D | Both | 0.16 (0.15, 0.16） |

| | | | | |
|---|---|---|---|---|
| United Republic of Tanzania | 0 to 4 | T1D | Female | 0.16 (0.15, 0.17） |
| United Republic of Tanzania | 0 to 4 | T1D | Male | 0.16 (0.15, 0.16） |
| United Republic of Tanzania | 10 to 14 | T1D | Both | 2.03 (2.00, 2.08） |
| United Republic of Tanzania | 10 to 14 | T1D | Female | 2.03 (1.97, 2.09） |
| United Republic of Tanzania | 10 to 14 | T1D | Male | 2.04 (1.98, 2.10） |
| United Republic of Tanzania | 15 to 19 | T1D | Both | 2.99 (2.93, 3.05） |
| United Republic of Tanzania | 15 to 19 | T1D | Female | 2.99 (2.90, 3.07） |
| United Republic of Tanzania | 15 to 19 | T1D | Male | 2.99 (2.91, 3.08） |
| United Republic of Tanzania | 20 to 24 | T1D | Both | 3.87 (3.79, 3.95） |
| United Republic of Tanzania | 20 to 24 | T1D | Female | 3.88 (3.77, 4.00） |
| United Republic of Tanzania | 20 to 24 | T1D | Male | 3.88 (3.77, 3.99） |
| United Republic of Tanzania | 25 to 29 | T1D | Both | 4.77 (4.67, 4.88） |
| United Republic of Tanzania | 25 to 29 | T1D | Female | 4.79 (4.64, 4.93） |
| United Republic of Tanzania | 25 to 29 | T1D | Male | 4.78 (4.64, 4.93） |
| United Republic of Tanzania | 30 to 34 | T1D | Both | 5.37 (5.25, 5.49） |
| United Republic of Tanzania | 30 to 34 | T1D | Female | 5.39 (5.22, 5.56） |
| United Republic of Tanzania | 30 to 34 | T1D | Male | 5.36 (5.20, 5.54） |
| United Republic of Tanzania | 35 to 39 | T1D | Both | 5.82 (5.69, 5.96） |
| United Republic of Tanzania | 35 to 39 | T1D | Female | 5.85 (5.66, 6.05） |
| United Republic of Tanzania | 35 to 39 | T1D | Male | 5.80 (5.61, 6.00） |
| United Republic of Tanzania | 40 to 44 | T1D | Both | 6.21 (6.06, 6.36） |
| United Republic of Tanzania | 40 to 44 | T1D | Female | 6.25 (6.04, 6.48） |
| United Republic of Tanzania | 40 to 44 | T1D | Male | 6.17 (5.96, 6.39） |
| United Republic of Tanzania | 45 to 49 | T1D | Both | 6.53 (6.37, 6.71） |
| United Republic of Tanzania | 45 to 49 | T1D | Female | 6.60 (6.36, 6.85） |
| United Republic of Tanzania | 45 to 49 | T1D | Male | 6.48 (6.25, 6.73） |
| United Republic of Tanzania | 5 to 9 | T1D | Both | 1.00 (1.00, 1.00） |
| United Republic of Tanzania | 5 to 9 | T1D | Female | 1.00 (1.00, 1.00） |
| United Republic of Tanzania | 5 to 9 | T1D | Male | 1.00 (1.00, 1.00） |
| United Republic of Tanzania | 50 to 54 | T1D | Both | 6.81 (6.62, 7.00） |
| United Republic of Tanzania | 50 to 54 | T1D | Female | 6.88 (6.62, 7.16） |
| United Republic of Tanzania | 50 to 54 | T1D | Male | 6.73 (6.47, 7.00） |
| United Republic of Tanzania | 55 to 59 | T1D | Both | 7.04 (6.84, 7.26） |

| | | | | |
|---|---|---|---|---|
| United Republic of Tanzania | 55 to 59 | T1D | Female | 7.14 (6.84, 7.45） |
| United Republic of Tanzania | 55 to 59 | T1D | Male | 6.95 (6.66, 7.25） |
| United Republic of Tanzania | 60 to 64 | T1D | Both | 7.25 (7.02, 7.49） |
| United Republic of Tanzania | 60 to 64 | T1D | Female | 7.37 (7.04, 7.71） |
| United Republic of Tanzania | 60 to 64 | T1D | Male | 7.13 (6.82, 7.47） |
| United Republic of Tanzania | 65 to 69 | T1D | Both | 7.43 (7.17, 7.69） |
| United Republic of Tanzania | 65 to 69 | T1D | Female | 7.57 (7.20, 7.96） |
| United Republic of Tanzania | 65 to 69 | T1D | Male | 7.28 (6.93, 7.66） |
| United States of America | 0 to 4 | T1D | Both | 0.27 (0.25, 0.28） |
| United States of America | 0 to 4 | T1D | Female | 0.26 (0.24, 0.27） |
| United States of America | 0 to 4 | T1D | Male | 0.28 (0.27, 0.29） |
| United States of America | 10 to 14 | T1D | Both | 1.85 (1.79, 1.91） |
| United States of America | 10 to 14 | T1D | Female | 1.85 (1.78, 1.91） |
| United States of America | 10 to 14 | T1D | Male | 1.85 (1.79, 1.90） |
| United States of America | 15 to 19 | T1D | Both | 2.46 (2.38, 2.54） |
| United States of America | 15 to 19 | T1D | Female | 2.39 (2.31, 2.48） |
| United States of America | 15 to 19 | T1D | Male | 2.52 (2.45, 2.59） |
| United States of America | 20 to 24 | T1D | Both | 3.04 (2.95, 3.14） |
| United States of America | 20 to 24 | T1D | Female | 2.88 (2.78, 2.99） |
| United States of America | 20 to 24 | T1D | Male | 3.19 (3.10, 3.28） |
| United States of America | 25 to 29 | T1D | Both | 3.84 (3.72, 3.97） |
| United States of America | 25 to 29 | T1D | Female | 3.57 (3.45, 3.71） |
| United States of America | 25 to 29 | T1D | Male | 4.10 (3.98, 4.22） |
| United States of America | 30 to 34 | T1D | Both | 4.46 (4.32, 4.61） |
| United States of America | 30 to 34 | T1D | Female | 4.09 (3.94, 4.24） |
| United States of America | 30 to 34 | T1D | Male | 4.83 (4.69, 4.98） |
| United States of America | 35 to 39 | T1D | Both | 5.01 (4.85, 5.18） |
| United States of America | 35 to 39 | T1D | Female | 4.54 (4.37, 4.71） |
| United States of America | 35 to 39 | T1D | Male | 5.49 (5.33, 5.66） |
| United States of America | 40 to 44 | T1D | Both | 5.57 (5.38, 5.76） |
| United States of America | 40 to 44 | T1D | Female | 4.99 (4.80, 5.19） |
| United States of America | 40 to 44 | T1D | Male | 6.15 (5.97, 6.34） |
| United States of America | 45 to 49 | T1D | Both | 6.11 (5.90, 6.32） |

| | | | | |
|---|---|---|---|---|
| United States of America | 45 to 49 | T1D | Female | 5.44 (5.23, 5.65） |
| United States of America | 45 to 49 | T1D | Male | 6.79 (6.59, 7.01） |
| United States of America | 5 to 9 | T1D | Both | 1.00 (1.00, 1.00） |
| United States of America | 5 to 9 | T1D | Female | 1.00 (1.00, 1.00） |
| United States of America | 5 to 9 | T1D | Male | 1.00 (1.00, 1.00） |
| United States of America | 50 to 54 | T1D | Both | 6.63 (6.40, 6.86） |
| United States of America | 50 to 54 | T1D | Female | 5.87 (5.64, 6.11） |
| United States of America | 50 to 54 | T1D | Male | 7.41 (7.19, 7.65） |
| United States of America | 55 to 59 | T1D | Both | 7.14 (6.89, 7.40） |
| United States of America | 55 to 59 | T1D | Female | 6.30 (6.04, 6.56） |
| United States of America | 55 to 59 | T1D | Male | 8.02 (7.77, 8.28） |
| United States of America | 60 to 64 | T1D | Both | 7.64 (7.36, 7.92） |
| United States of America | 60 to 64 | T1D | Female | 6.72 (6.45, 7.01） |
| United States of America | 60 to 64 | T1D | Male | 8.62 (8.34, 8.91） |
| United States of America | 65 to 69 | T1D | Both | 8.16 (7.85, 8.47） |
| United States of America | 65 to 69 | T1D | Female | 7.17 (6.87, 7.49） |
| United States of America | 65 to 69 | T1D | Male | 9.23 (8.92, 9.55） |
| United States Virgin Islands | 0 to 4 | T1D | Both | 0.21 (0.08, 0.54） |
| United States Virgin Islands | 0 to 4 | T1D | Female | 0.05 (0.01, 0.77） |
| United States Virgin Islands | 0 to 4 | T1D | Male | 0.43 (0.14, 1.35） |
| United States Virgin Islands | 10 to 14 | T1D | Both | 2.13 (1.31, 3.46） |
| United States Virgin Islands | 10 to 14 | T1D | Female | 2.58 (1.25, 5.31） |
| United States Virgin Islands | 10 to 14 | T1D | Male | 2.30 (1.11, 4.78） |
| United States Virgin Islands | 15 to 19 | T1D | Both | 3.47 (2.15, 5.60） |
| United States Virgin Islands | 15 to 19 | T1D | Female | 4.39 (2.14, 9.00） |
| United States Virgin Islands | 15 to 19 | T1D | Male | 3.61 (1.75, 7.47） |
| United States Virgin Islands | 20 to 24 | T1D | Both | 4.56 (2.80, 7.45） |
| United States Virgin Islands | 20 to 24 | T1D | Female | 5.59 (2.68, 11.65） |
| United States Virgin Islands | 20 to 24 | T1D | Male | 5.00 (2.37, 10.56） |
| United States Virgin Islands | 25 to 29 | T1D | Both | 5.68 (3.45, 9.35） |
| United States Virgin Islands | 25 to 29 | T1D | Female | 6.71 (3.18, 14.16） |
| United States Virgin Islands | 25 to 29 | T1D | Male | 6.50 (3.05, 13.85） |
| United States Virgin Islands | 30 to 34 | T1D | Both | 6.71 (4.05, 11.13） |

| | | | | |
|---|---|---|---|---|
| United States Virgin Islands | 30 to 34 | T1D | Female | 8.03 (3.77, 17.11） |
| United States Virgin Islands | 30 to 34 | T1D | Male | 7.83 (3.65, 16.80） |
| United States Virgin Islands | 35 to 39 | T1D | Both | 7.80 (4.66, 13.05） |
| United States Virgin Islands | 35 to 39 | T1D | Female | 9.25 (4.28, 19.96） |
| United States Virgin Islands | 35 to 39 | T1D | Male | 9.02 (4.15, 19.60） |
| United States Virgin Islands | 40 to 44 | T1D | Both | 8.98 (5.33, 15.14） |
| United States Virgin Islands | 40 to 44 | T1D | Female | 10.31 (4.72, 22.52） |
| United States Virgin Islands | 40 to 44 | T1D | Male | 10.27 (4.69, 22.52） |
| United States Virgin Islands | 45 to 49 | T1D | Both | 10.13 (5.97, 17.20） |
| United States Virgin Islands | 45 to 49 | T1D | Female | 11.92 (5.41, 26.30） |
| United States Virgin Islands | 45 to 49 | T1D | Male | 11.53 (5.21, 25.51） |
| United States Virgin Islands | 5 to 9 | T1D | Both | 1.00 (1.00, 1.00） |
| United States Virgin Islands | 5 to 9 | T1D | Female | 1.00 (1.00, 1.00） |
| United States Virgin Islands | 5 to 9 | T1D | Male | 1.00 (1.00, 1.00） |
| United States Virgin Islands | 50 to 54 | T1D | Both | 11.26 (6.58, 19.26） |
| United States Virgin Islands | 50 to 54 | T1D | Female | 13.75 (6.17, 30.65） |
| United States Virgin Islands | 50 to 54 | T1D | Male | 12.77 (5.72, 28.50） |
| United States Virgin Islands | 55 to 59 | T1D | Both | 12.64 (7.33, 21.80） |
| United States Virgin Islands | 55 to 59 | T1D | Female | 15.13 (6.71, 34.12） |
| United States Virgin Islands | 55 to 59 | T1D | Male | 14.09 (6.25, 31.79） |
| United States Virgin Islands | 60 to 64 | T1D | Both | 13.79 (7.91, 24.05） |
| United States Virgin Islands | 60 to 64 | T1D | Female | 16.91 (7.38, 38.77） |
| United States Virgin Islands | 60 to 64 | T1D | Male | 15.54 (6.79, 35.55） |
| United States Virgin Islands | 65 to 69 | T1D | Both | 15.12 (8.56, 26.71） |
| United States Virgin Islands | 65 to 69 | T1D | Female | 19.50 (8.37, 45.46） |
| United States Virgin Islands | 65 to 69 | T1D | Male | 16.93 (7.28, 39.37） |
| Uruguay | 0 to 4 | T1D | Both | 0.33 (0.31, 0.36） |
| Uruguay | 0 to 4 | T1D | Female | 0.33 (0.30, 0.37） |
| Uruguay | 0 to 4 | T1D | Male | 0.33 (0.30, 0.37） |
| Uruguay | 10 to 14 | T1D | Both | 1.70 (1.62, 1.78） |
| Uruguay | 10 to 14 | T1D | Female | 1.70 (1.59, 1.82） |
| Uruguay | 10 to 14 | T1D | Male | 1.70 (1.59, 1.81） |
| Uruguay | 15 to 19 | T1D | Both | 2.19 (2.09, 2.30） |

| | | | | |
|---|---|---|---|---|
| Uruguay | 15 to 19 | T1D | Female | 2.20 (2.06, 2.35） |
| Uruguay | 15 to 19 | T1D | Male | 2.18 (2.05, 2.33） |
| Uruguay | 20 to 24 | T1D | Both | 2.53 (2.41, 2.65） |
| Uruguay | 20 to 24 | T1D | Female | 2.55 (2.38, 2.73） |
| Uruguay | 20 to 24 | T1D | Male | 2.50 (2.34, 2.68） |
| Uruguay | 25 to 29 | T1D | Both | 2.89 (2.75, 3.03） |
| Uruguay | 25 to 29 | T1D | Female | 2.92 (2.72, 3.13） |
| Uruguay | 25 to 29 | T1D | Male | 2.85 (2.66, 3.06） |
| Uruguay | 30 to 34 | T1D | Both | 3.21 (3.05, 3.37） |
| Uruguay | 30 to 34 | T1D | Female | 3.25 (3.02, 3.49） |
| Uruguay | 30 to 34 | T1D | Male | 3.17 (2.95, 3.40） |
| Uruguay | 35 to 39 | T1D | Both | 3.51 (3.34, 3.70） |
| Uruguay | 35 to 39 | T1D | Female | 3.56 (3.30, 3.83） |
| Uruguay | 35 to 39 | T1D | Male | 3.47 (3.23, 3.74） |
| Uruguay | 40 to 44 | T1D | Both | 3.82 (3.62, 4.03） |
| Uruguay | 40 to 44 | T1D | Female | 3.86 (3.58, 4.17） |
| Uruguay | 40 to 44 | T1D | Male | 3.78 (3.51, 4.08） |
| Uruguay | 45 to 49 | T1D | Both | 4.12 (3.90, 4.36） |
| Uruguay | 45 to 49 | T1D | Female | 4.16 (3.85, 4.50） |
| Uruguay | 45 to 49 | T1D | Male | 4.08 (3.78, 4.41） |
| Uruguay | 5 to 9 | T1D | Both | 1.00 (1.00, 1.00） |
| Uruguay | 5 to 9 | T1D | Female | 1.00 (1.00, 1.00） |
| Uruguay | 5 to 9 | T1D | Male | 1.00 (1.00, 1.00） |
| Uruguay | 50 to 54 | T1D | Both | 4.41 (4.17, 4.67） |
| Uruguay | 50 to 54 | T1D | Female | 4.45 (4.10, 4.82） |
| Uruguay | 50 to 54 | T1D | Male | 4.38 (4.04, 4.74） |
| Uruguay | 55 to 59 | T1D | Both | 4.70 (4.43, 4.98） |
| Uruguay | 55 to 59 | T1D | Female | 4.73 (4.36, 5.13） |
| Uruguay | 55 to 59 | T1D | Male | 4.66 (4.29, 5.06） |
| Uruguay | 60 to 64 | T1D | Both | 4.99 (4.70, 5.29） |
| Uruguay | 60 to 64 | T1D | Female | 5.01 (4.61, 5.46） |
| Uruguay | 60 to 64 | T1D | Male | 4.95 (4.54, 5.39） |
| Uruguay | 65 to 69 | T1D | Both | 5.28 (4.96, 5.62） |

| | | | | |
|---|---|---|---|---|
| Uruguay | 65 to 69 | T1D | Female | 5.30 (4.86, 5.78） |
| Uruguay | 65 to 69 | T1D | Male | 5.23 (4.79, 5.72） |
| Uzbekistan | 0 to 4 | T1D | Both | 0.16 (0.16, 0.17） |
| Uzbekistan | 0 to 4 | T1D | Female | 0.16 (0.15, 0.17） |
| Uzbekistan | 0 to 4 | T1D | Male | 0.17 (0.15, 0.18） |
| Uzbekistan | 10 to 14 | T1D | Both | 2.22 (2.16, 2.27） |
| Uzbekistan | 10 to 14 | T1D | Female | 2.22 (2.14, 2.30） |
| Uzbekistan | 10 to 14 | T1D | Male | 2.21 (2.14, 2.29） |
| Uzbekistan | 15 to 19 | T1D | Both | 3.42 (3.34, 3.51） |
| Uzbekistan | 15 to 19 | T1D | Female | 3.38 (3.26, 3.49） |
| Uzbekistan | 15 to 19 | T1D | Male | 3.47 (3.35, 3.58） |
| Uzbekistan | 20 to 24 | T1D | Both | 4.50 (4.39, 4.61） |
| Uzbekistan | 20 to 24 | T1D | Female | 4.35 (4.20, 4.51） |
| Uzbekistan | 20 to 24 | T1D | Male | 4.64 (4.48, 4.80） |
| Uzbekistan | 25 to 29 | T1D | Both | 5.67 (5.53, 5.81） |
| Uzbekistan | 25 to 29 | T1D | Female | 5.37 (5.18, 5.57） |
| Uzbekistan | 25 to 29 | T1D | Male | 5.96 (5.75, 6.17） |
| Uzbekistan | 30 to 34 | T1D | Both | 6.69 (6.52, 6.86） |
| Uzbekistan | 30 to 34 | T1D | Female | 6.25 (6.02, 6.48） |
| Uzbekistan | 30 to 34 | T1D | Male | 7.12 (6.87, 7.37） |
| Uzbekistan | 35 to 39 | T1D | Both | 7.62 (7.42, 7.82） |
| Uzbekistan | 35 to 39 | T1D | Female | 7.05 (6.79, 7.33） |
| Uzbekistan | 35 to 39 | T1D | Male | 8.18 (7.88, 8.49） |
| Uzbekistan | 40 to 44 | T1D | Both | 8.51 (8.28, 8.75） |
| Uzbekistan | 40 to 44 | T1D | Female | 7.83 (7.53, 8.15） |
| Uzbekistan | 40 to 44 | T1D | Male | 9.20 (8.86, 9.56） |
| Uzbekistan | 45 to 49 | T1D | Both | 9.42 (9.15, 9.69） |
| Uzbekistan | 45 to 49 | T1D | Female | 8.64 (8.29, 9.00） |
| Uzbekistan | 45 to 49 | T1D | Male | 10.22 (9.82, 10.63） |
| Uzbekistan | 5 to 9 | T1D | Both | 1.00 (1.00, 1.00） |
| Uzbekistan | 5 to 9 | T1D | Female | 1.00 (1.00, 1.00） |
| Uzbekistan | 5 to 9 | T1D | Male | 1.00 (1.00, 1.00） |
| Uzbekistan | 50 to 54 | T1D | Both | 10.33 (10.03, 10.64） |

| | | | | |
|---|---|---|---|---|
| Uzbekistan | 50 to 54 | T1D | Female | 9.49 (9.09, 9.90） |
| Uzbekistan | 50 to 54 | T1D | Male | 11.23 (10.77, 11.70） |
| Uzbekistan | 55 to 59 | T1D | Both | 11.21 (10.87, 11.57） |
| Uzbekistan | 55 to 59 | T1D | Female | 10.34 (9.88, 10.82） |
| Uzbekistan | 55 to 59 | T1D | Male | 12.19 (11.67, 12.74） |
| Uzbekistan | 60 to 64 | T1D | Both | 12.07 (11.67, 12.48） |
| Uzbekistan | 60 to 64 | T1D | Female | 11.19 (10.66, 11.74） |
| Uzbekistan | 60 to 64 | T1D | Male | 13.10 (12.49, 13.73） |
| Uzbekistan | 65 to 69 | T1D | Both | 12.96 (12.49, 13.45） |
| Uzbekistan | 65 to 69 | T1D | Female | 12.12 (11.49, 12.78） |
| Uzbekistan | 65 to 69 | T1D | Male | 14.02 (13.31, 14.77） |
| Vanuatu | 0 to 4 | T1D | Both | 0.13 (0.05, 0.33） |
| Vanuatu | 0 to 4 | T1D | Female | 0.23 (0.08, 0.67） |
| Vanuatu | 0 to 4 | T1D | Male | 0.24 (0.08, 0.68） |
| Vanuatu | 10 to 14 | T1D | Both | 2.01 (1.34, 3.01） |
| Vanuatu | 10 to 14 | T1D | Female | 1.97 (1.11, 3.49） |
| Vanuatu | 10 to 14 | T1D | Male | 1.99 (1.13, 3.52） |
| Vanuatu | 15 to 19 | T1D | Both | 2.88 (1.90, 4.36） |
| Vanuatu | 15 to 19 | T1D | Female | 2.74 (1.54, 4.90） |
| Vanuatu | 15 to 19 | T1D | Male | 2.91 (1.63, 5.19） |
| Vanuatu | 20 to 24 | T1D | Both | 3.66 (2.39, 5.59） |
| Vanuatu | 20 to 24 | T1D | Female | 3.36 (1.85, 6.10） |
| Vanuatu | 20 to 24 | T1D | Male | 3.76 (2.07, 6.84） |
| Vanuatu | 25 to 29 | T1D | Both | 4.46 (2.87, 6.93） |
| Vanuatu | 25 to 29 | T1D | Female | 3.92 (2.11, 7.28） |
| Vanuatu | 25 to 29 | T1D | Male | 4.75 (2.57, 8.79） |
| Vanuatu | 30 to 34 | T1D | Both | 4.99 (3.15, 7.90） |
| Vanuatu | 30 to 34 | T1D | Female | 4.49 (2.35, 8.57） |
| Vanuatu | 30 to 34 | T1D | Male | 5.55 (2.93, 10.53） |
| Vanuatu | 35 to 39 | T1D | Both | 5.55 (3.43, 8.99） |
| Vanuatu | 35 to 39 | T1D | Female | 4.73 (2.38, 9.40） |
| Vanuatu | 35 to 39 | T1D | Male | 6.32 (3.23, 12.38） |
| Vanuatu | 40 to 44 | T1D | Both | 6.01 (3.63, 9.97） |

| | | | | |
|---|---|---|---|---|
| Vanuatu | 40 to 44 | T1D | Female | 5.10 (2.48, 10.50） |
| Vanuatu | 40 to 44 | T1D | Male | 6.77 (3.35, 13.66） |
| Vanuatu | 45 to 49 | T1D | Both | 6.39 (3.75, 10.89） |
| Vanuatu | 45 to 49 | T1D | Female | 5.45 (2.54, 11.70） |
| Vanuatu | 45 to 49 | T1D | Male | 7.54 (3.61, 15.77） |
| Vanuatu | 5 to 9 | T1D | Both | 1.00 (1.00, 1.00） |
| Vanuatu | 5 to 9 | T1D | Female | 1.00 (1.00, 1.00） |
| Vanuatu | 5 to 9 | T1D | Male | 1.00 (1.00, 1.00） |
| Vanuatu | 50 to 54 | T1D | Both | 6.87 (3.90, 12.08） |
| Vanuatu | 50 to 54 | T1D | Female | 5.84 (2.59, 13.17） |
| Vanuatu | 50 to 54 | T1D | Male | 7.54 (3.45, 16.50） |
| Vanuatu | 55 to 59 | T1D | Both | 7.52 (4.13, 13.70） |
| Vanuatu | 55 to 59 | T1D | Female | 6.37 (2.67, 15.20） |
| Vanuatu | 55 to 59 | T1D | Male | 8.08 (3.53, 18.51） |
| Vanuatu | 60 to 64 | T1D | Both | 7.84 (4.13, 14.89） |
| Vanuatu | 60 to 64 | T1D | Female | 6.61 (2.59, 16.86） |
| Vanuatu | 60 to 64 | T1D | Male | 8.36 (3.46, 20.23） |
| Vanuatu | 65 to 69 | T1D | Both | 7.54 (3.67, 15.49） |
| Vanuatu | 65 to 69 | T1D | Female | 6.36 (2.21, 18.33） |
| Vanuatu | 65 to 69 | T1D | Male | 9.02 (3.44, 23.67） |
| Venezuela (Bolivarian Republic of) | 0 to 4 | T1D | Both | 0.13 (0.12, 0.14） |
| Venezuela (Bolivarian Republic of) | 0 to 4 | T1D | Female | 0.12 (0.11, 0.13） |
| Venezuela (Bolivarian Republic of) | 0 to 4 | T1D | Male | 0.14 (0.13, 0.16） |
| Venezuela (Bolivarian Republic of) | 10 to 14 | T1D | Both | 2.20 (2.13, 2.28） |
| Venezuela (Bolivarian Republic of) | 10 to 14 | T1D | Female | 2.36 (2.26, 2.47） |
| Venezuela (Bolivarian Republic of) | 10 to 14 | T1D | Male | 2.03 (1.93, 2.13） |
| Venezuela (Bolivarian Republic of) | 15 to 19 | T1D | Both | 3.00 (2.91, 3.11） |
| Venezuela (Bolivarian Republic of) | 15 to 19 | T1D | Female | 3.17 (3.03, 3.32） |
| Venezuela (Bolivarian Republic of) | 15 to 19 | T1D | Male | 2.81 (2.67, 2.95） |
| Venezuela (Bolivarian Republic of) | 20 to 24 | T1D | Both | 3.55 (3.43, 3.68） |
| Venezuela (Bolivarian Republic of) | 20 to 24 | T1D | Female | 3.64 (3.48, 3.81） |
| Venezuela (Bolivarian Republic of) | 20 to 24 | T1D | Male | 3.44 (3.27, 3.61） |
| Venezuela (Bolivarian Republic of) | 25 to 29 | T1D | Both | 4.09 (3.95, 4.23） |

| | | | | |
|---|---|---|---|---|
| Venezuela (Bolivarian Republic of) | 25 to 29 | T1D | Female | 4.06 (3.88, 4.26） |
| Venezuela (Bolivarian Republic of) | 25 to 29 | T1D | Male | 4.09 (3.88, 4.30） |
| Venezuela (Bolivarian Republic of) | 30 to 34 | T1D | Both | 4.52 (4.36, 4.69） |
| Venezuela (Bolivarian Republic of) | 30 to 34 | T1D | Female | 4.40 (4.19, 4.63） |
| Venezuela (Bolivarian Republic of) | 30 to 34 | T1D | Male | 4.64 (4.40, 4.89） |
| Venezuela (Bolivarian Republic of) | 35 to 39 | T1D | Both | 4.91 (4.73, 5.09） |
| Venezuela (Bolivarian Republic of) | 35 to 39 | T1D | Female | 4.71 (4.47, 4.95） |
| Venezuela (Bolivarian Republic of) | 35 to 39 | T1D | Male | 5.12 (4.85, 5.41） |
| Venezuela (Bolivarian Republic of) | 40 to 44 | T1D | Both | 5.27 (5.07, 5.48） |
| Venezuela (Bolivarian Republic of) | 40 to 44 | T1D | Female | 5.00 (4.74, 5.27） |
| Venezuela (Bolivarian Republic of) | 40 to 44 | T1D | Male | 5.57 (5.27, 5.90） |
| Venezuela (Bolivarian Republic of) | 45 to 49 | T1D | Both | 5.61 (5.39, 5.84） |
| Venezuela (Bolivarian Republic of) | 45 to 49 | T1D | Female | 5.28 (4.99, 5.58） |
| Venezuela (Bolivarian Republic of) | 45 to 49 | T1D | Male | 5.99 (5.65, 6.35） |
| Venezuela (Bolivarian Republic of) | 5 to 9 | T1D | Both | 1.00 (1.00, 1.00） |
| Venezuela (Bolivarian Republic of) | 5 to 9 | T1D | Female | 1.00 (1.00, 1.00） |
| Venezuela (Bolivarian Republic of) | 5 to 9 | T1D | Male | 1.00 (1.00, 1.00） |
| Venezuela (Bolivarian Republic of) | 50 to 54 | T1D | Both | 5.94 (5.69, 6.19） |
| Venezuela (Bolivarian Republic of) | 50 to 54 | T1D | Female | 5.55 (5.24, 5.88） |
| Venezuela (Bolivarian Republic of) | 50 to 54 | T1D | Male | 6.37 (5.99, 6.77） |
| Venezuela (Bolivarian Republic of) | 55 to 59 | T1D | Both | 6.24 (5.97, 6.52） |
| Venezuela (Bolivarian Republic of) | 55 to 59 | T1D | Female | 5.81 (5.46, 6.17） |
| Venezuela (Bolivarian Republic of) | 55 to 59 | T1D | Male | 6.72 (6.30, 7.16） |
| Venezuela (Bolivarian Republic of) | 60 to 64 | T1D | Both | 6.52 (6.22, 6.83） |
| Venezuela (Bolivarian Republic of) | 60 to 64 | T1D | Female | 6.06 (5.68, 6.46） |
| Venezuela (Bolivarian Republic of) | 60 to 64 | T1D | Male | 7.03 (6.56, 7.53） |
| Venezuela (Bolivarian Republic of) | 65 to 69 | T1D | Both | 6.79 (6.45, 7.15） |
| Venezuela (Bolivarian Republic of) | 65 to 69 | T1D | Female | 6.32 (5.89, 6.78） |
| Venezuela (Bolivarian Republic of) | 65 to 69 | T1D | Male | 7.33 (6.80, 7.89） |
| Viet Nam | 0 to 4 | T1D | Both | 0.15 (0.14, 0.15） |
| Viet Nam | 0 to 4 | T1D | Female | 0.15 (0.13, 0.16） |
| Viet Nam | 0 to 4 | T1D | Male | 0.14 (0.13, 0.16） |
| Viet Nam | 10 to 14 | T1D | Both | 2.09 (2.04, 2.14） |

| | | | | |
|---|---|---|---|---|
| Viet Nam | 10 to 14 | T1D | Female | 2.06 (1.99, 2.13） |
| Viet Nam | 10 to 14 | T1D | Male | 2.11 (2.04, 2.19） |
| Viet Nam | 15 to 19 | T1D | Both | 2.94 (2.87, 3.01） |
| Viet Nam | 15 to 19 | T1D | Female | 2.85 (2.76, 2.95） |
| Viet Nam | 15 to 19 | T1D | Male | 3.03 (2.93, 3.14） |
| Viet Nam | 20 to 24 | T1D | Both | 3.64 (3.55, 3.73） |
| Viet Nam | 20 to 24 | T1D | Female | 3.45 (3.34, 3.57） |
| Viet Nam | 20 to 24 | T1D | Male | 3.83 (3.70, 3.97） |
| Viet Nam | 25 to 29 | T1D | Both | 4.34 (4.23, 4.44） |
| Viet Nam | 25 to 29 | T1D | Female | 4.03 (3.89, 4.17） |
| Viet Nam | 25 to 29 | T1D | Male | 4.66 (4.50, 4.83） |
| Viet Nam | 30 to 34 | T1D | Both | 4.87 (4.74, 4.99） |
| Viet Nam | 30 to 34 | T1D | Female | 4.46 (4.30, 4.62） |
| Viet Nam | 30 to 34 | T1D | Male | 5.30 (5.11, 5.49） |
| Viet Nam | 35 to 39 | T1D | Both | 5.33 (5.19, 5.47） |
| Viet Nam | 35 to 39 | T1D | Female | 4.84 (4.66, 5.02） |
| Viet Nam | 35 to 39 | T1D | Male | 5.86 (5.64, 6.08） |
| Viet Nam | 40 to 44 | T1D | Both | 5.78 (5.62, 5.93） |
| Viet Nam | 40 to 44 | T1D | Female | 5.21 (5.01, 5.41） |
| Viet Nam | 40 to 44 | T1D | Male | 6.38 (6.14, 6.63） |
| Viet Nam | 45 to 49 | T1D | Both | 6.20 (6.03, 6.37） |
| Viet Nam | 45 to 49 | T1D | Female | 5.57 (5.35, 5.79） |
| Viet Nam | 45 to 49 | T1D | Male | 6.88 (6.61, 7.16） |
| Viet Nam | 5 to 9 | T1D | Both | 1.00 (1.00, 1.00） |
| Viet Nam | 5 to 9 | T1D | Female | 1.00 (1.00, 1.00） |
| Viet Nam | 5 to 9 | T1D | Male | 1.00 (1.00, 1.00） |
| Viet Nam | 50 to 54 | T1D | Both | 6.60 (6.41, 6.80） |
| Viet Nam | 50 to 54 | T1D | Female | 5.92 (5.68, 6.17） |
| Viet Nam | 50 to 54 | T1D | Male | 7.34 (7.04, 7.65） |
| Viet Nam | 55 to 59 | T1D | Both | 7.00 (6.79, 7.22） |
| Viet Nam | 55 to 59 | T1D | Female | 6.29 (6.02, 6.57） |
| Viet Nam | 55 to 59 | T1D | Male | 7.80 (7.46, 8.15） |
| Viet Nam | 60 to 64 | T1D | Both | 7.41 (7.17, 7.66） |

| | | | | |
|---|---|---|---|---|
| Viet Nam | 60 to 64 | T1D | Female | 6.67 (6.36, 6.98） |
| Viet Nam | 60 to 64 | T1D | Male | 8.25 (7.87, 8.65） |
| Viet Nam | 65 to 69 | T1D | Both | 7.82 (7.55, 8.11） |
| Viet Nam | 65 to 69 | T1D | Female | 7.06 (6.71, 7.42） |
| Viet Nam | 65 to 69 | T1D | Male | 8.70 (8.26, 9.16） |
| Yemen | 0 to 4 | T1D | Both | 0.13 (0.12, 0.14） |
| Yemen | 0 to 4 | T1D | Female | 0.13 (0.13, 0.15） |
| Yemen | 0 to 4 | T1D | Male | 0.13 (0.12, 0.14） |
| Yemen | 10 to 14 | T1D | Both | 2.42 (2.37, 2.48） |
| Yemen | 10 to 14 | T1D | Female | 2.55 (2.47, 2.64） |
| Yemen | 10 to 14 | T1D | Male | 2.32 (2.25, 2.39） |
| Yemen | 15 to 19 | T1D | Both | 3.73 (3.64, 3.82） |
| Yemen | 15 to 19 | T1D | Female | 3.99 (3.85, 4.13） |
| Yemen | 15 to 19 | T1D | Male | 3.53 (3.41, 3.64） |
| Yemen | 20 to 24 | T1D | Both | 4.85 (4.73, 4.97） |
| Yemen | 20 to 24 | T1D | Female | 5.13 (4.94, 5.32） |
| Yemen | 20 to 24 | T1D | Male | 4.63 (4.47, 4.79） |
| Yemen | 25 to 29 | T1D | Both | 6.05 (5.89, 6.21） |
| Yemen | 25 to 29 | T1D | Female | 6.32 (6.08, 6.56） |
| Yemen | 25 to 29 | T1D | Male | 5.85 (5.65, 6.06） |
| Yemen | 30 to 34 | T1D | Both | 7.20 (7.00, 7.40） |
| Yemen | 30 to 34 | T1D | Female | 7.45 (7.16, 7.76） |
| Yemen | 30 to 34 | T1D | Male | 7.02 (6.77, 7.29） |
| Yemen | 35 to 39 | T1D | Both | 8.35 (8.11, 8.59） |
| Yemen | 35 to 39 | T1D | Female | 8.58 (8.22, 8.96） |
| Yemen | 35 to 39 | T1D | Male | 8.18 (7.87, 8.51） |
| Yemen | 40 to 44 | T1D | Both | 9.51 (9.22, 9.81） |
| Yemen | 40 to 44 | T1D | Female | 9.74 (9.30, 10.19） |
| Yemen | 40 to 44 | T1D | Male | 9.36 (8.97, 9.76） |
| Yemen | 45 to 49 | T1D | Both | 10.70 (10.34, 11.06） |
| Yemen | 45 to 49 | T1D | Female | 10.94 (10.41, 11.49） |
| Yemen | 45 to 49 | T1D | Male | 10.53 (10.06, 11.03） |
| Yemen | 5 to 9 | T1D | Both | 1.00 (1.00, 1.00） |

| | | | | |
|---|---|---|---|---|
| Yemen | 5 to 9 | T1D | Female | 1.00 (1.00, 1.00） |
| Yemen | 5 to 9 | T1D | Male | 1.00 (1.00, 1.00） |
| Yemen | 50 to 54 | T1D | Both | 11.90 (11.48, 12.34） |
| Yemen | 50 to 54 | T1D | Female | 12.19 (11.56, 12.85） |
| Yemen | 50 to 54 | T1D | Male | 11.71 (11.15, 12.31） |
| Yemen | 55 to 59 | T1D | Both | 13.15 (12.65, 13.67） |
| Yemen | 55 to 59 | T1D | Female | 13.50 (12.75, 14.28） |
| Yemen | 55 to 59 | T1D | Male | 12.91 (12.24, 13.61） |
| Yemen | 60 to 64 | T1D | Both | 14.43 (13.83, 15.05） |
| Yemen | 60 to 64 | T1D | Female | 14.86 (13.97, 15.81） |
| Yemen | 60 to 64 | T1D | Male | 14.11 (13.32, 14.95） |
| Yemen | 65 to 69 | T1D | Both | 15.74 (15.02, 16.49） |
| Yemen | 65 to 69 | T1D | Female | 16.31 (15.22, 17.47） |
| Yemen | 65 to 69 | T1D | Male | 15.33 (14.38, 16.33） |
| Zambia | 0 to 4 | T1D | Both | 0.16 (0.15, 0.17） |
| Zambia | 0 to 4 | T1D | Female | 0.16 (0.14, 0.18） |
| Zambia | 0 to 4 | T1D | Male | 0.16 (0.14, 0.17） |
| Zambia | 10 to 14 | T1D | Both | 2.03 (1.96, 2.11） |
| Zambia | 10 to 14 | T1D | Female | 2.03 (1.93, 2.14） |
| Zambia | 10 to 14 | T1D | Male | 2.04 (1.94, 2.14） |
| Zambia | 15 to 19 | T1D | Both | 2.98 (2.88, 3.09） |
| Zambia | 15 to 19 | T1D | Female | 2.98 (2.84, 3.14） |
| Zambia | 15 to 19 | T1D | Male | 2.99 (2.85, 3.15） |
| Zambia | 20 to 24 | T1D | Both | 3.87 (3.73, 4.02） |
| Zambia | 20 to 24 | T1D | Female | 3.88 (3.68, 4.09） |
| Zambia | 20 to 24 | T1D | Male | 3.88 (3.69, 4.09） |
| Zambia | 25 to 29 | T1D | Both | 4.79 (4.61, 4.97） |
| Zambia | 25 to 29 | T1D | Female | 4.79 (4.54, 5.06） |
| Zambia | 25 to 29 | T1D | Male | 4.79 (4.54, 5.05） |
| Zambia | 30 to 34 | T1D | Both | 5.39 (5.18, 5.60） |
| Zambia | 30 to 34 | T1D | Female | 5.40 (5.10, 5.71） |
| Zambia | 30 to 34 | T1D | Male | 5.37 (5.08, 5.68） |
| Zambia | 35 to 39 | T1D | Both | 5.84 (5.60, 6.09） |

| | | | | |
|---|---|---|---|---|
| Zambia | 35 to 39 | T1D | Female | 5.86 (5.52, 6.23） |
| Zambia | 35 to 39 | T1D | Male | 5.81 (5.48, 6.16） |
| Zambia | 40 to 44 | T1D | Both | 6.23 (5.96, 6.51） |
| Zambia | 40 to 44 | T1D | Female | 6.27 (5.88, 6.68） |
| Zambia | 40 to 44 | T1D | Male | 6.18 (5.81, 6.58） |
| Zambia | 45 to 49 | T1D | Both | 6.56 (6.26, 6.88） |
| Zambia | 45 to 49 | T1D | Female | 6.61 (6.18, 7.07） |
| Zambia | 45 to 49 | T1D | Male | 6.50 (6.08, 6.94） |
| Zambia | 5 to 9 | T1D | Both | 1.00 (1.00, 1.00） |
| Zambia | 5 to 9 | T1D | Female | 1.00 (1.00, 1.00） |
| Zambia | 5 to 9 | T1D | Male | 1.00 (1.00, 1.00） |
| Zambia | 50 to 54 | T1D | Both | 6.84 (6.50, 7.20） |
| Zambia | 50 to 54 | T1D | Female | 6.92 (6.43, 7.44） |
| Zambia | 50 to 54 | T1D | Male | 6.76 (6.30, 7.27） |
| Zambia | 55 to 59 | T1D | Both | 7.10 (6.72, 7.50） |
| Zambia | 55 to 59 | T1D | Female | 7.19 (6.64, 7.77） |
| Zambia | 55 to 59 | T1D | Male | 7.00 (6.48, 7.57） |
| Zambia | 60 to 64 | T1D | Both | 7.32 (6.89, 7.78） |
| Zambia | 60 to 64 | T1D | Female | 7.43 (6.82, 8.09） |
| Zambia | 60 to 64 | T1D | Male | 7.21 (6.62, 7.85） |
| Zambia | 65 to 69 | T1D | Both | 7.51 (7.03, 8.03） |
| Zambia | 65 to 69 | T1D | Female | 7.65 (6.96, 8.41） |
| Zambia | 65 to 69 | T1D | Male | 7.38 (6.70, 8.11） |
| Zimbabwe | 0 to 4 | T1D | Both | 0.16 (0.15, 0.18） |
| Zimbabwe | 0 to 4 | T1D | Female | 0.16 (0.15, 0.18） |
| Zimbabwe | 0 to 4 | T1D | Male | 0.16 (0.15, 0.18） |
| Zimbabwe | 10 to 14 | T1D | Both | 2.07 (2.00, 2.14） |
| Zimbabwe | 10 to 14 | T1D | Female | 2.07 (1.98, 2.18） |
| Zimbabwe | 10 to 14 | T1D | Male | 2.07 (1.97, 2.17） |
| Zimbabwe | 15 to 19 | T1D | Both | 3.04 (2.94, 3.14） |
| Zimbabwe | 15 to 19 | T1D | Female | 3.04 (2.89, 3.19） |
| Zimbabwe | 15 to 19 | T1D | Male | 3.04 (2.90, 3.19） |
| Zimbabwe | 20 to 24 | T1D | Both | 3.88 (3.74, 4.01） |

| | | | | |
|---|---|---|---|---|
| Zimbabwe | 20 to 24 | T1D | Female | 3.88 (3.69, 4.08） |
| Zimbabwe | 20 to 24 | T1D | Male | 3.89 (3.70, 4.08） |
| Zimbabwe | 25 to 29 | T1D | Both | 4.76 (4.59, 4.93） |
| Zimbabwe | 25 to 29 | T1D | Female | 4.77 (4.53, 5.02） |
| Zimbabwe | 25 to 29 | T1D | Male | 4.76 (4.53, 5.01） |
| Zimbabwe | 30 to 34 | T1D | Both | 5.41 (5.22, 5.62） |
| Zimbabwe | 30 to 34 | T1D | Female | 5.44 (5.15, 5.73） |
| Zimbabwe | 30 to 34 | T1D | Male | 5.41 (5.13, 5.70） |
| Zimbabwe | 35 to 39 | T1D | Both | 5.92 (5.69, 6.16） |
| Zimbabwe | 35 to 39 | T1D | Female | 5.95 (5.63, 6.29） |
| Zimbabwe | 35 to 39 | T1D | Male | 5.91 (5.59, 6.24） |
| Zimbabwe | 40 to 44 | T1D | Both | 6.33 (6.07, 6.60） |
| Zimbabwe | 40 to 44 | T1D | Female | 6.38 (6.01, 6.76） |
| Zimbabwe | 40 to 44 | T1D | Male | 6.31 (5.95, 6.69） |
| Zimbabwe | 45 to 49 | T1D | Both | 6.71 (6.42, 7.02） |
| Zimbabwe | 45 to 49 | T1D | Female | 6.77 (6.36, 7.20） |
| Zimbabwe | 45 to 49 | T1D | Male | 6.68 (6.27, 7.12） |
| Zimbabwe | 5 to 9 | T1D | Both | 1.00 (1.00, 1.00） |
| Zimbabwe | 5 to 9 | T1D | Female | 1.00 (1.00, 1.00） |
| Zimbabwe | 5 to 9 | T1D | Male | 1.00 (1.00, 1.00） |
| Zimbabwe | 50 to 54 | T1D | Both | 7.07 (6.74, 7.41） |
| Zimbabwe | 50 to 54 | T1D | Female | 7.14 (6.68, 7.63） |
| Zimbabwe | 50 to 54 | T1D | Male | 7.02 (6.56, 7.52） |
| Zimbabwe | 55 to 59 | T1D | Both | 7.37 (7.01, 7.75） |
| Zimbabwe | 55 to 59 | T1D | Female | 7.47 (6.96, 8.01） |
| Zimbabwe | 55 to 59 | T1D | Male | 7.31 (6.80, 7.86） |
| Zimbabwe | 60 to 64 | T1D | Both | 7.61 (7.21, 8.04） |
| Zimbabwe | 60 to 64 | T1D | Female | 7.73 (7.17, 8.33） |
| Zimbabwe | 60 to 64 | T1D | Male | 7.53 (6.96, 8.14） |
| Zimbabwe | 65 to 69 | T1D | Both | 7.77 (7.31, 8.24） |
| Zimbabwe | 65 to 69 | T1D | Female | 7.92 (7.29, 8.61） |
| Zimbabwe | 65 to 69 | T1D | Male | 7.65 (7.02, 8.35） |
| Global | 0 to 4 | AA | Both | 0.73 (0.73, 0.73) |

| | | | | |
|---|---|---|---|---|
| Global | 0 to 4 | AA | Female | 0.74 (0.74, 0.74) |
| Global | 0 to 4 | AA | Male | 0.72 (0.72, 0.72) |
| Global | 10 to 14 | AA | Both | 1.28 (1.28, 1.29) |
| Global | 10 to 14 | AA | Female | 1.28 (1.28, 1.29) |
| Global | 10 to 14 | AA | Male | 1.29 (1.28, 1.29) |
| Global | 15 to 19 | AA | Both | 2.24 (2.23, 2.24) |
| Global | 15 to 19 | AA | Female | 2.25 (2.25, 2.26) |
| Global | 15 to 19 | AA | Male | 2.19 (2.18, 2.20) |
| Global | 20 to 24 | AA | Both | 3.55 (3.55, 3.56) |
| Global | 20 to 24 | AA | Female | 3.51 (3.51, 3.52) |
| Global | 20 to 24 | AA | Male | 3.56 (3.54, 3.57) |
| Global | 25 to 29 | AA | Both | 4.93 (4.93, 4.94) |
| Global | 25 to 29 | AA | Female | 4.87 (4.86, 4.88) |
| Global | 25 to 29 | AA | Male | 4.94 (4.92, 4.96) |
| Global | 30 to 34 | AA | Both | 5.26 (5.25, 5.27) |
| Global | 30 to 34 | AA | Female | 5.29 (5.28, 5.30) |
| Global | 30 to 34 | AA | Male | 5.13 (5.11, 5.15) |
| Global | 35 to 39 | AA | Both | 4.92 (4.92, 4.93) |
| Global | 35 to 39 | AA | Female | 5.03 (5.02, 5.04) |
| Global | 35 to 39 | AA | Male | 4.66 (4.65, 4.68) |
| Global | 40 to 44 | AA | Both | 4.21 (4.20, 4.21) |
| Global | 40 to 44 | AA | Female | 4.35 (4.34, 4.36) |
| Global | 40 to 44 | AA | Male | 3.90 (3.89, 3.92) |
| Global | 45 to 49 | AA | Both | 3.64 (3.63, 3.65) |
| Global | 45 to 49 | AA | Female | 3.80 (3.79, 3.81) |
| Global | 45 to 49 | AA | Male | 3.29 (3.28, 3.30) |
| Global | 5 to 9 | AA | Both | 1.00 (1.00, 1.00) |
| Global | 5 to 9 | AA | Female | 1.00 (1.00, 1.00) |
| Global | 5 to 9 | AA | Male | 1.00 (1.00, 1.00) |
| Global | 50 to 54 | AA | Both | 3.28 (3.27, 3.28) |
| Global | 50 to 54 | AA | Female | 3.48 (3.47, 3.49) |
| Global | 50 to 54 | AA | Male | 2.84 (2.83, 2.86) |
| Global | 55 to 59 | AA | Both | 3.16 (3.15, 3.16) |

| | | | | |
|---|---|---|---|---|
| Global | 55 to 59 | AA | Female | 3.40 (3.39, 3.41) |
| Global | 55 to 59 | AA | Male | 2.61 (2.60, 2.63) |
| Global | 60 to 64 | AA | Both | 3.31 (3.30, 3.32) |
| Global | 60 to 64 | AA | Female | 3.60 (3.59, 3.61) |
| Global | 60 to 64 | AA | Male | 2.62 (2.60, 2.63) |
| Global | 65 to 69 | AA | Both | 3.11 (3.11, 3.12) |
| Global | 65 to 69 | AA | Female | 3.38 (3.37, 3.39) |
| Global | 65 to 69 | AA | Male | 2.38 (2.37, 2.40) |
| Global | 0 to 4 | IBD | Both | 0.04 (0.02, 0.09) |
| Global | 0 to 4 | IBD | Female | 0.04 (0.02, 0.10) |
| Global | 0 to 4 | IBD | Male | 0.04 (0.01, 0.10) |
| Global | 10 to 14 | IBD | Both | 5.20 (4.31, 6.26) |
| Global | 10 to 14 | IBD | Female | 5.15 (4.25, 6.25) |
| Global | 10 to 14 | IBD | Male | 5.24 (4.29, 6.39) |
| Global | 15 to 19 | IBD | Both | 19.01 (15.87, 22.77) |
| Global | 15 to 19 | IBD | Female | 20.74 (17.21, 24.99) |
| Global | 15 to 19 | IBD | Male | 17.45 (14.38, 21.17) |
| Global | 20 to 24 | IBD | Both | 46.14 (38.56, 55.21) |
| Global | 20 to 24 | IBD | Female | 50.01 (41.52, 60.22) |
| Global | 20 to 24 | IBD | Male | 42.59 (35.14, 51.62) |
| Global | 25 to 29 | IBD | Both | 80.85 (67.56, 96.75) |
| Global | 25 to 29 | IBD | Female | 86.19 (71.56, 103.81) |
| Global | 25 to 29 | IBD | Male | 75.95 (62.66, 92.07) |
| Global | 30 to 34 | IBD | Both | 115.91 (96.84, 138.73) |
| Global | 30 to 34 | IBD | Female | 122.97 (102.08, 148.14) |
| Global | 30 to 34 | IBD | Male | 109.48 (90.30, 132.74) |
| Global | 35 to 39 | IBD | Both | 146.96 (122.75, 175.94) |
| Global | 35 to 39 | IBD | Female | 156.33 (129.75, 188.37) |
| Global | 35 to 39 | IBD | Male | 138.43 (114.14, 167.89) |
| Global | 40 to 44 | IBD | Both | 161.40 (134.79, 193.27) |
| Global | 40 to 44 | IBD | Female | 162.33 (134.69, 195.63) |
| Global | 40 to 44 | IBD | Male | 161.26 (132.94, 195.63) |
| Global | 45 to 49 | IBD | Both | 175.71 (146.70, 210.45) |

| | | | | |
|---|---|---|---|---|
| Global | 45 to 49 | IBD | Female | 172.77 (143.33, 208.27) |
| Global | 45 to 49 | IBD | Male | 179.49 (147.93, 217.79) |
| Global | 5 to 9 | IBD | Both | 1.00 (1.00, 1.00) |
| Global | 5 to 9 | IBD | Female | 1.00 (1.00, 1.00) |
| Global | 5 to 9 | IBD | Male | 1.00 (1.00, 1.00) |
| Global | 50 to 54 | IBD | Both | 186.13 (155.36, 222.99) |
| Global | 50 to 54 | IBD | Female | 179.94 (149.24, 216.96) |
| Global | 50 to 54 | IBD | Male | 193.33 (159.29, 234.65) |
| Global | 55 to 59 | IBD | Both | 191.50 (159.80, 229.49) |
| Global | 55 to 59 | IBD | Female | 184.25 (152.77, 222.21) |
| Global | 55 to 59 | IBD | Male | 199.96 (164.70, 242.78) |
| Global | 60 to 64 | IBD | Both | 188.95 (157.62, 226.50) |
| Global | 60 to 64 | IBD | Female | 181.31 (150.28, 218.75) |
| Global | 60 to 64 | IBD | Male | 197.97 (162.99, 240.45) |
| Global | 65 to 69 | IBD | Both | 178.80 (149.07, 214.45) |
| Global | 65 to 69 | IBD | Female | 171.91 (142.42, 207.51) |
| Global | 65 to 69 | IBD | Male | 187.17 (154.00, 227.47) |
| Global | 10 to 14 | MS | Both | 8.88 (8.52, 9.25) |
| Global | 10 to 14 | MS | Female | 9.30 (8.80, 9.83) |
| Global | 10 to 14 | MS | Male | 8.32 (7.81, 8.86) |
| Global | 15 to 19 | MS | Both | 34.64 (33.26, 36.07) |
| Global | 15 to 19 | MS | Female | 38.21 (36.21, 40.33) |
| Global | 15 to 19 | MS | Male | 29.75 (27.96, 31.65) |
| Global | 20 to 24 | MS | Both | 92.05 (88.39, 95.85) |
| Global | 20 to 24 | MS | Female | 105.08 (99.58, 110.89) |
| Global | 20 to 24 | MS | Male | 73.40 (69.00, 78.08) |
| Global | 25 to 29 | MS | Both | 189.72 (182.18, 197.56) |
| Global | 25 to 29 | MS | Female | 220.40 (208.87, 232.57) |
| Global | 25 to 29 | MS | Male | 145.60 (136.89, 154.88) |
| Global | 30 to 34 | MS | Both | 300.84 (288.89, 313.28) |
| Global | 30 to 34 | MS | Female | 350.77 (332.41, 370.15) |
| Global | 30 to 34 | MS | Male | 229.91 (216.13, 244.56) |
| Global | 35 to 39 | MS | Both | 404.88 (388.79, 421.64) |

| | | | | |
|---|---|---|---|---|
| Global | 35 to 39 | MS | Female | 469.92 (445.31, 495.90) |
| Global | 35 to 39 | MS | Male | 313.11 (294.34, 333.07) |
| Global | 40 to 44 | MS | Both | 490.19 (470.69, 510.48) |
| Global | 40 to 44 | MS | Female | 563.63 (534.10, 594.79) |
| Global | 40 to 44 | MS | Male | 386.40 (363.22, 411.05) |
| Global | 45 to 49 | MS | Both | 554.51 (532.45, 577.49) |
| Global | 45 to 49 | MS | Female | 629.86 (596.85, 664.70) |
| Global | 45 to 49 | MS | Male | 445.52 (418.78, 473.96) |
| Global | 5 to 9 | MS | Both | 1.00 (1.00, 1.00) |
| Global | 5 to 9 | MS | Female | 1.00 (1.00, 1.00) |
| Global | 5 to 9 | MS | Male | 1.00 (1.00, 1.00) |
| Global | 50 to 54 | MS | Both | 596.58 (572.83, 621.31) |
| Global | 50 to 54 | MS | Female | 668.75 (633.68, 705.76) |
| Global | 50 to 54 | MS | Male | 487.17 (457.91, 518.29) |
| Global | 55 to 59 | MS | Both | 609.19 (584.92, 634.46) |
| Global | 55 to 59 | MS | Female | 678.91 (643.29, 716.51) |
| Global | 55 to 59 | MS | Male | 496.78 (466.92, 528.54) |
| Global | 60 to 64 | MS | Both | 596.43 (572.65, 621.19) |
| Global | 60 to 64 | MS | Female | 665.52 (630.58, 702.40) |
| Global | 60 to 64 | MS | Male | 477.99 (449.23, 508.59) |
| Global | 65 to 69 | MS | Both | 583.73 (560.43, 608.00) |
| Global | 65 to 69 | MS | Female | 652.63 (618.33, 688.83) |
| Global | 65 to 69 | MS | Male | 456.45 (428.95, 485.73) |
| Global | 0 to 4 | Psoriasis | Both | 0.42 (0.41, 0.42) |
| Global | 0 to 4 | Psoriasis | Female | 0.41 (0.41, 0.42) |
| Global | 0 to 4 | Psoriasis | Male | 0.43 (0.42, 0.43) |
| Global | 10 to 14 | Psoriasis | Both | 1.29 (1.29, 1.30) |
| Global | 10 to 14 | Psoriasis | Female | 1.34 (1.33, 1.35) |
| Global | 10 to 14 | Psoriasis | Male | 1.25 (1.24, 1.26) |
| Global | 15 to 19 | Psoriasis | Both | 1.48 (1.47, 1.49) |
| Global | 15 to 19 | Psoriasis | Female | 1.56 (1.55, 1.57) |
| Global | 15 to 19 | Psoriasis | Male | 1.40 (1.39, 1.41) |
| Global | 20 to 24 | Psoriasis | Both | 1.65 (1.64, 1.66) |

| | | | | |
|---|---|---|---|---|
| Global | 20 to 24 | Psoriasis | Female | 1.70 (1.68, 1.71) |
| Global | 20 to 24 | Psoriasis | Male | 1.58 (1.57, 1.60) |
| Global | 25 to 29 | Psoriasis | Both | 1.89 (1.88, 1.91) |
| Global | 25 to 29 | Psoriasis | Female | 1.87 (1.85, 1.89) |
| Global | 25 to 29 | Psoriasis | Male | 1.92 (1.90, 1.93) |
| Global | 30 to 34 | Psoriasis | Both | 2.19 (2.18, 2.21) |
| Global | 30 to 34 | Psoriasis | Female | 2.08 (2.06, 2.10) |
| Global | 30 to 34 | Psoriasis | Male | 2.31 (2.30, 2.33) |
| Global | 35 to 39 | Psoriasis | Both | 2.49 (2.47, 2.51) |
| Global | 35 to 39 | Psoriasis | Female | 2.29 (2.26, 2.31) |
| Global | 35 to 39 | Psoriasis | Male | 2.71 (2.69, 2.73) |
| Global | 40 to 44 | Psoriasis | Both | 2.79 (2.77, 2.81) |
| Global | 40 to 44 | Psoriasis | Female | 2.48 (2.46, 2.51) |
| Global | 40 to 44 | Psoriasis | Male | 3.12 (3.10, 3.15) |
| Global | 45 to 49 | Psoriasis | Both | 3.03 (3.01, 3.06) |
| Global | 45 to 49 | Psoriasis | Female | 2.65 (2.63, 2.68) |
| Global | 45 to 49 | Psoriasis | Male | 3.45 (3.43, 3.48) |
| Global | 5 to 9 | Psoriasis | Both | 1.00 (1.00, 1.00) |
| Global | 5 to 9 | Psoriasis | Female | 1.00 (1.00, 1.00) |
| Global | 5 to 9 | Psoriasis | Male | 1.00 (1.00, 1.00) |
| Global | 50 to 54 | Psoriasis | Both | 3.29 (3.26, 3.31) |
| Global | 50 to 54 | Psoriasis | Female | 2.88 (2.85, 2.91) |
| Global | 50 to 54 | Psoriasis | Male | 3.75 (3.72, 3.77) |
| Global | 55 to 59 | Psoriasis | Both | 3.51 (3.48, 3.54) |
| Global | 55 to 59 | Psoriasis | Female | 3.07 (3.04, 3.11) |
| Global | 55 to 59 | Psoriasis | Male | 4.01 (3.98, 4.05) |
| Global | 60 to 64 | Psoriasis | Both | 3.62 (3.58, 3.65) |
| Global | 60 to 64 | Psoriasis | Female | 3.19 (3.16, 3.23) |
| Global | 60 to 64 | Psoriasis | Male | 4.10 (4.07, 4.14) |
| Global | 65 to 69 | Psoriasis | Both | 3.67 (3.63, 3.70) |
| Global | 65 to 69 | Psoriasis | Female | 3.33 (3.29, 3.37) |
| Global | 65 to 69 | Psoriasis | Male | 4.06 (4.03, 4.09) |
| Global | 0 to 4 | RHD | Both | 0.16 (0.16, 0.17) |

| | | | | |
|---|---|---|---|---|
| Global | 0 to 4 | RHD | Female | 0.16 (0.15, 0.17) |
| Global | 0 to 4 | RHD | Male | 0.16 (0.16, 0.17) |
| Global | 10 to 14 | RHD | Both | 2.21 (2.16, 2.26) |
| Global | 10 to 14 | RHD | Female | 2.24 (2.18, 2.29) |
| Global | 10 to 14 | RHD | Male | 2.18 (2.13, 2.23) |
| Global | 15 to 19 | RHD | Both | 3.46 (3.38, 3.55) |
| Global | 15 to 19 | RHD | Female | 3.57 (3.48, 3.66) |
| Global | 15 to 19 | RHD | Male | 3.34 (3.26, 3.43) |
| Global | 20 to 24 | RHD | Both | 4.29 (4.19, 4.40) |
| Global | 20 to 24 | RHD | Female | 4.52 (4.40, 4.63) |
| Global | 20 to 24 | RHD | Male | 4.04 (3.94, 4.14) |
| Global | 25 to 29 | RHD | Both | 4.66 (4.54, 4.78) |
| Global | 25 to 29 | RHD | Female | 4.96 (4.83, 5.09) |
| Global | 25 to 29 | RHD | Male | 4.33 (4.22, 4.44) |
| Global | 30 to 34 | RHD | Both | 4.71 (4.58, 4.83) |
| Global | 30 to 34 | RHD | Female | 5.03 (4.89, 5.16) |
| Global | 30 to 34 | RHD | Male | 4.35 (4.23, 4.46) |
| Global | 35 to 39 | RHD | Both | 4.52 (4.40, 4.65) |
| Global | 35 to 39 | RHD | Female | 4.82 (4.69, 4.96) |
| Global | 35 to 39 | RHD | Male | 4.19 (4.07, 4.30) |
| Global | 40 to 44 | RHD | Both | 4.17 (4.05, 4.29) |
| Global | 40 to 44 | RHD | Female | 4.43 (4.30, 4.56) |
| Global | 40 to 44 | RHD | Male | 3.88 (3.77, 3.99) |
| Global | 45 to 49 | RHD | Both | 3.67 (3.56, 3.79) |
| Global | 45 to 49 | RHD | Female | 3.92 (3.80, 4.04) |
| Global | 45 to 49 | RHD | Male | 3.40 (3.29, 3.50) |
| Global | 5 to 9 | RHD | Both | 1.00 (1.00, 1.00) |
| Global | 5 to 9 | RHD | Female | 1.00 (1.00, 1.00) |
| Global | 5 to 9 | RHD | Male | 1.00 (1.00, 1.00) |
| Global | 50 to 54 | RHD | Both | 3.12 (3.02, 3.22) |
| Global | 50 to 54 | RHD | Female | 3.37 (3.26, 3.49) |
| Global | 50 to 54 | RHD | Male | 2.83 (2.74, 2.93) |
| Global | 55 to 59 | RHD | Both | 2.74 (2.65, 2.84) |

| | | | | |
|---|---|---|---|---|
| Global | 55 to 59 | RHD | Female | 3.01 (2.90, 3.12) |
| Global | 55 to 59 | RHD | Male | 2.43 (2.34, 2.52) |
| Global | 60 to 64 | RHD | Both | 2.53 (2.43, 2.63) |
| Global | 60 to 64 | RHD | Female | 2.81 (2.70, 2.92) |
| Global | 60 to 64 | RHD | Male | 2.19 (2.10, 2.28) |
| Global | 65 to 69 | RHD | Both | 2.41 (2.31, 2.52) |
| Global | 65 to 69 | RHD | Female | 2.68 (2.57, 2.80) |
| Global | 65 to 69 | RHD | Male | 2.06 (1.97, 2.16) |
| Global | 10 to 14 | RA | Both | 6.96 (6.73, 7.20) |
| Global | 10 to 14 | RA | Female | 7.11 (6.85, 7.39) |
| Global | 10 to 14 | RA | Male | 6.70 (6.46, 6.95) |
| Global | 15 to 19 | RA | Both | 18.92 (18.30, 19.56) |
| Global | 15 to 19 | RA | Female | 19.92 (19.20, 20.67) |
| Global | 15 to 19 | RA | Male | 16.99 (16.39, 17.61) |
| Global | 20 to 24 | RA | Both | 36.38 (35.19, 37.61) |
| Global | 20 to 24 | RA | Female | 38.98 (37.57, 40.44) |
| Global | 20 to 24 | RA | Male | 30.95 (29.85, 32.08) |
| Global | 25 to 29 | RA | Both | 57.36 (55.48, 59.29) |
| Global | 25 to 29 | RA | Female | 62.38 (60.12, 64.72) |
| Global | 25 to 29 | RA | Male | 46.87 (45.21, 48.58) |
| Global | 30 to 34 | RA | Both | 82.41 (79.71, 85.20) |
| Global | 30 to 34 | RA | Female | 90.74 (87.44, 94.16) |
| Global | 30 to 34 | RA | Male | 65.57 (63.25, 67.98) |
| Global | 35 to 39 | RA | Both | 115.68 (111.89, 119.60) |
| Global | 35 to 39 | RA | Female | 127.94 (123.29, 132.77) |
| Global | 35 to 39 | RA | Male | 91.32 (88.08, 94.68) |
| Global | 40 to 44 | RA | Both | 159.17 (153.95, 164.57) |
| Global | 40 to 44 | RA | Female | 175.58 (169.20, 182.21) |
| Global | 40 to 44 | RA | Male | 126.56 (122.06, 131.22) |
| Global | 45 to 49 | RA | Both | 216.36 (209.26, 223.71) |
| Global | 45 to 49 | RA | Female | 235.82 (227.23, 244.73) |
| Global | 45 to 49 | RA | Male | 176.04 (169.77, 182.53) |
| Global | 5 to 9 | RA | Both | 1.00 (1.00, 1.00) |

| | | | | |
|---|---|---|---|---|
| Global | 5 to 9 | RA | Female | 1.00 (1.00, 1.00) |
| Global | 5 to 9 | RA | Male | 1.00 (1.00, 1.00) |
| Global | 50 to 54 | RA | Both | 287.80 (278.34, 297.59) |
| Global | 50 to 54 | RA | Female | 308.81 (297.55, 320.50) |
| Global | 50 to 54 | RA | Male | 239.96 (231.40, 248.83) |
| Global | 55 to 59 | RA | Both | 371.45 (359.21, 384.10) |
| Global | 55 to 59 | RA | Female | 392.44 (378.11, 407.31) |
| Global | 55 to 59 | RA | Male | 315.29 (304.03, 326.96) |
| Global | 60 to 64 | RA | Both | 467.59 (452.17, 483.53) |
| Global | 60 to 64 | RA | Female | 486.51 (468.73, 504.96) |
| Global | 60 to 64 | RA | Male | 402.50 (388.11, 417.43) |
| Global | 65 to 69 | RA | Both | 561.34 (542.81, 580.50) |
| Global | 65 to 69 | RA | Female | 572.49 (551.55, 594.23) |
| Global | 65 to 69 | RA | Male | 492.88 (475.23, 511.18) |
| African Region | 0 to 4 | AA | Both | 0.71 (0.71, 0.71) |
| African Region | 0 to 4 | AA | Female | 0.72 (0.71, 0.72) |
| African Region | 0 to 4 | AA | Male | 0.70 (0.69, 0.70) |
| African Region | 10 to 14 | AA | Both | 1.29 (1.28, 1.29) |
| African Region | 10 to 14 | AA | Female | 1.27 (1.26, 1.27) |
| African Region | 10 to 14 | AA | Male | 1.32 (1.32, 1.33) |
| African Region | 15 to 19 | AA | Both | 2.24 (2.23, 2.25) |
| African Region | 15 to 19 | AA | Female | 2.22 (2.21, 2.23) |
| African Region | 15 to 19 | AA | Male | 2.26 (2.24, 2.28) |
| African Region | 20 to 24 | AA | Both | 3.51 (3.49, 3.52) |
| African Region | 20 to 24 | AA | Female | 3.49 (3.47, 3.51) |
| African Region | 20 to 24 | AA | Male | 3.41 (3.39, 3.44) |
| African Region | 25 to 29 | AA | Both | 4.88 (4.86, 4.90) |
| African Region | 25 to 29 | AA | Female | 4.84 (4.82, 4.87) |
| African Region | 25 to 29 | AA | Male | 4.76 (4.73, 4.79) |
| African Region | 30 to 34 | AA | Both | 5.18 (5.16, 5.21) |
| African Region | 30 to 34 | AA | Female | 5.18 (5.15, 5.20) |
| African Region | 30 to 34 | AA | Male | 5.05 (5.01, 5.08) |
| African Region | 35 to 39 | AA | Both | 4.84 (4.81, 4.86) |

| | | | | |
|---|---|---|---|---|
| African Region | 35 to 39 | AA | Female | 4.84 (4.81, 4.87) |
| African Region | 35 to 39 | AA | Male | 4.73 (4.70, 4.77) |
| African Region | 40 to 44 | AA | Both | 4.11 (4.09, 4.13) |
| African Region | 40 to 44 | AA | Female | 4.12 (4.09, 4.14) |
| African Region | 40 to 44 | AA | Male | 4.05 (4.01, 4.08) |
| African Region | 45 to 49 | AA | Both | 3.53 (3.51, 3.55) |
| African Region | 45 to 49 | AA | Female | 3.54 (3.51, 3.56) |
| African Region | 45 to 49 | AA | Male | 3.46 (3.43, 3.50) |
| African Region | 5 to 9 | AA | Both | 1.00 (1.00, 1.00) |
| African Region | 5 to 9 | AA | Female | 1.00 (1.00, 1.00) |
| African Region | 5 to 9 | AA | Male | 1.00 (1.00, 1.00) |
| African Region | 50 to 54 | AA | Both | 3.16 (3.14, 3.18) |
| African Region | 50 to 54 | AA | Female | 3.17 (3.15, 3.20) |
| African Region | 50 to 54 | AA | Male | 3.07 (3.03, 3.10) |
| African Region | 55 to 59 | AA | Both | 3.01 (2.99, 3.03) |
| African Region | 55 to 59 | AA | Female | 2.99 (2.97, 3.02) |
| African Region | 55 to 59 | AA | Male | 2.94 (2.91, 2.97) |
| African Region | 60 to 64 | AA | Both | 3.14 (3.12, 3.16) |
| African Region | 60 to 64 | AA | Female | 3.11 (3.08, 3.14) |
| African Region | 60 to 64 | AA | Male | 3.04 (3.01, 3.08) |
| African Region | 65 to 69 | AA | Both | 2.93 (2.91, 2.96) |
| African Region | 65 to 69 | AA | Female | 2.87 (2.84, 2.90) |
| African Region | 65 to 69 | AA | Male | 2.85 (2.81, 2.90) |
| African Region | 0 to 4 | IBD | Both | 0.06 (0.04, 0.07) |
| African Region | 0 to 4 | IBD | Female | 0.06 (0.04, 0.08) |
| African Region | 0 to 4 | IBD | Male | 0.06 (0.04, 0.08) |
| African Region | 10 to 14 | IBD | Both | 3.63 (3.40, 3.88) |
| African Region | 10 to 14 | IBD | Female | 3.62 (3.29, 3.98) |
| African Region | 10 to 14 | IBD | Male | 3.64 (3.32, 4.00) |
| African Region | 15 to 19 | IBD | Both | 10.27 (9.62, 10.95) |
| African Region | 15 to 19 | IBD | Female | 11.53 (10.52, 12.64) |
| African Region | 15 to 19 | IBD | Male | 8.98 (8.19, 9.84) |
| African Region | 20 to 24 | IBD | Both | 20.80 (19.50, 22.19) |

| | | | | |
|---|---|---|---|---|
| African Region | 20 to 24 | IBD | Female | 23.25 (21.21, 25.48) |
| African Region | 20 to 24 | IBD | Male | 18.19 (16.60, 19.94) |
| African Region | 25 to 29 | IBD | Both | 35.22 (33.01, 37.59) |
| African Region | 25 to 29 | IBD | Female | 39.17 (35.73, 42.94) |
| African Region | 25 to 29 | IBD | Male | 30.98 (28.25, 33.96) |
| African Region | 30 to 34 | IBD | Both | 52.62 (49.29, 56.17) |
| African Region | 30 to 34 | IBD | Female | 58.46 (53.30, 64.12) |
| African Region | 30 to 34 | IBD | Male | 46.45 (42.34, 50.95) |
| African Region | 35 to 39 | IBD | Both | 72.35 (67.75, 77.27) |
| African Region | 35 to 39 | IBD | Female | 80.45 (73.30, 88.29) |
| African Region | 35 to 39 | IBD | Male | 63.96 (58.26, 70.21) |
| African Region | 40 to 44 | IBD | Both | 94.13 (88.10, 100.56) |
| African Region | 40 to 44 | IBD | Female | 104.96 (95.60, 115.24) |
| African Region | 40 to 44 | IBD | Male | 83.09 (75.65, 91.26) |
| African Region | 45 to 49 | IBD | Both | 117.61 (110.04, 125.71) |
| African Region | 45 to 49 | IBD | Female | 131.30 (119.53, 144.24) |
| African Region | 45 to 49 | IBD | Male | 103.69 (94.34, 113.96) |
| African Region | 5 to 9 | IBD | Both | 1.00 (1.00, 1.00) |
| African Region | 5 to 9 | IBD | Female | 1.00 (1.00, 1.00) |
| African Region | 5 to 9 | IBD | Male | 1.00 (1.00, 1.00) |
| African Region | 50 to 54 | IBD | Both | 127.94 (119.64, 136.82) |
| African Region | 50 to 54 | IBD | Female | 131.55 (119.65, 144.63) |
| African Region | 50 to 54 | IBD | Male | 123.70 (112.47, 136.05) |
| African Region | 55 to 59 | IBD | Both | 137.12 (128.15, 146.73) |
| African Region | 55 to 59 | IBD | Female | 135.06 (122.74, 148.62) |
| African Region | 55 to 59 | IBD | Male | 138.43 (125.75, 152.39) |
| African Region | 60 to 64 | IBD | Both | 142.73 (133.28, 152.85) |
| African Region | 60 to 64 | IBD | Female | 140.40 (127.45, 154.66) |
| African Region | 60 to 64 | IBD | Male | 144.23 (130.87, 158.95) |
| African Region | 65 to 69 | IBD | Both | 139.83 (130.41, 149.93) |
| African Region | 65 to 69 | IBD | Female | 136.75 (123.92, 150.90) |
| African Region | 65 to 69 | IBD | Male | 142.22 (128.82, 157.01) |
| African Region | 10 to 14 | MS | Both | 7.93 (6.98, 9.01) |

| | | | | |
|---|---|---|---|---|
| African Region | 10 to 14 | MS | Female | 8.21 (6.96, 9.69) |
| African Region | 10 to 14 | MS | Male | 7.52 (6.16, 9.18) |
| African Region | 15 to 19 | MS | Both | 26.82 (23.65, 30.40) |
| African Region | 15 to 19 | MS | Female | 28.79 (24.46, 33.90) |
| African Region | 15 to 19 | MS | Male | 23.70 (19.47, 28.85) |
| African Region | 20 to 24 | MS | Both | 63.71 (56.21, 72.21) |
| African Region | 20 to 24 | MS | Female | 70.09 (59.56, 82.49) |
| African Region | 20 to 24 | MS | Male | 52.69 (43.30, 64.11) |
| African Region | 25 to 29 | MS | Both | 123.03 (108.53, 139.46) |
| African Region | 25 to 29 | MS | Female | 137.75 (117.03, 162.12) |
| African Region | 25 to 29 | MS | Male | 97.60 (80.20, 118.79) |
| African Region | 30 to 34 | MS | Both | 190.64 (168.14, 216.16) |
| African Region | 30 to 34 | MS | Female | 216.37 (183.78, 254.73) |
| African Region | 30 to 34 | MS | Male | 148.65 (122.08, 181.00) |
| African Region | 35 to 39 | MS | Both | 256.28 (225.97, 290.66) |
| African Region | 35 to 39 | MS | Female | 293.11 (248.89, 345.18) |
| African Region | 35 to 39 | MS | Male | 198.90 (163.27, 242.32) |
| African Region | 40 to 44 | MS | Both | 312.16 (275.17, 354.12) |
| African Region | 40 to 44 | MS | Female | 358.28 (304.14, 422.06) |
| African Region | 40 to 44 | MS | Male | 242.94 (199.31, 296.11) |
| African Region | 45 to 49 | MS | Both | 354.99 (312.83, 402.82) |
| African Region | 45 to 49 | MS | Female | 406.94 (345.33, 479.54) |
| African Region | 45 to 49 | MS | Male | 276.71 (226.89, 337.46) |
| African Region | 5 to 9 | MS | Both | 1.00 (1.00, 1.00) |
| African Region | 5 to 9 | MS | Female | 1.00 (1.00, 1.00) |
| African Region | 5 to 9 | MS | Male | 1.00 (1.00, 1.00) |
| African Region | 50 to 54 | MS | Both | 380.44 (335.14, 431.87) |
| African Region | 50 to 54 | MS | Female | 433.96 (368.11, 511.59) |
| African Region | 50 to 54 | MS | Male | 296.15 (242.66, 361.43) |
| African Region | 55 to 59 | MS | Both | 383.65 (337.81, 435.71) |
| African Region | 55 to 59 | MS | Female | 435.90 (369.55, 514.15) |
| African Region | 55 to 59 | MS | Male | 296.66 (242.85, 362.39) |
| African Region | 60 to 64 | MS | Both | 370.17 (325.72, 420.68) |

| | | | | |
|---|---|---|---|---|
| African Region | 60 to 64 | MS | Female | 419.57 (355.44, 495.26) |
| African Region | 60 to 64 | MS | Male | 282.27 (230.76, 345.28) |
| African Region | 65 to 69 | MS | Both | 353.18 (310.44, 401.79) |
| African Region | 65 to 69 | MS | Female | 399.12 (337.73, 471.66) |
| African Region | 65 to 69 | MS | Male | 264.70 (215.91, 324.50) |
| African Region | 0 to 4 | Psoriasis | Both | 0.43 (0.43, 0.43) |
| African Region | 0 to 4 | Psoriasis | Female | 0.43 (0.43, 0.43) |
| African Region | 0 to 4 | Psoriasis | Male | 0.43 (0.43, 0.43) |
| African Region | 10 to 14 | Psoriasis | Both | 1.24 (1.23, 1.24) |
| African Region | 10 to 14 | Psoriasis | Female | 1.24 (1.23, 1.24) |
| African Region | 10 to 14 | Psoriasis | Male | 1.24 (1.23, 1.25) |
| African Region | 15 to 19 | Psoriasis | Both | 1.37 (1.37, 1.38) |
| African Region | 15 to 19 | Psoriasis | Female | 1.36 (1.36, 1.37) |
| African Region | 15 to 19 | Psoriasis | Male | 1.38 (1.37, 1.39) |
| African Region | 20 to 24 | Psoriasis | Both | 1.50 (1.50, 1.51) |
| African Region | 20 to 24 | Psoriasis | Female | 1.48 (1.48, 1.49) |
| African Region | 20 to 24 | Psoriasis | Male | 1.52 (1.51, 1.53) |
| African Region | 25 to 29 | Psoriasis | Both | 1.67 (1.66, 1.67) |
| African Region | 25 to 29 | Psoriasis | Female | 1.64 (1.63, 1.64) |
| African Region | 25 to 29 | Psoriasis | Male | 1.70 (1.69, 1.71) |
| African Region | 30 to 34 | Psoriasis | Both | 1.84 (1.84, 1.85) |
| African Region | 30 to 34 | Psoriasis | Female | 1.81 (1.80, 1.82) |
| African Region | 30 to 34 | Psoriasis | Male | 1.88 (1.87, 1.89) |
| African Region | 35 to 39 | Psoriasis | Both | 1.97 (1.97, 1.98) |
| African Region | 35 to 39 | Psoriasis | Female | 1.93 (1.92, 1.95) |
| African Region | 35 to 39 | Psoriasis | Male | 2.02 (2.01, 2.03) |
| African Region | 40 to 44 | Psoriasis | Both | 2.09 (2.08, 2.10) |
| African Region | 40 to 44 | Psoriasis | Female | 2.04 (2.03, 2.06) |
| African Region | 40 to 44 | Psoriasis | Male | 2.13 (2.12, 2.14) |
| African Region | 45 to 49 | Psoriasis | Both | 2.20 (2.19, 2.21) |
| African Region | 45 to 49 | Psoriasis | Female | 2.16 (2.14, 2.17) |
| African Region | 45 to 49 | Psoriasis | Male | 2.25 (2.24, 2.27) |
| African Region | 5 to 9 | Psoriasis | Both | 1.00 (1.00, 1.00) |

| | | | | |
|---|---|---|---|---|
| African Region | 5 to 9 | Psoriasis | Female | 1.00 (1.00, 1.00) |
| African Region | 5 to 9 | Psoriasis | Male | 1.00 (1.00, 1.00) |
| African Region | 50 to 54 | Psoriasis | Both | 2.35 (2.34, 2.36) |
| African Region | 50 to 54 | Psoriasis | Female | 2.30 (2.29, 2.32) |
| African Region | 50 to 54 | Psoriasis | Male | 2.40 (2.39, 2.42) |
| African Region | 55 to 59 | Psoriasis | Both | 2.50 (2.48, 2.51) |
| African Region | 55 to 59 | Psoriasis | Female | 2.44 (2.42, 2.46) |
| African Region | 55 to 59 | Psoriasis | Male | 2.55 (2.53, 2.57) |
| African Region | 60 to 64 | Psoriasis | Both | 2.51 (2.50, 2.53) |
| African Region | 60 to 64 | Psoriasis | Female | 2.45 (2.43, 2.47) |
| African Region | 60 to 64 | Psoriasis | Male | 2.58 (2.55, 2.60) |
| African Region | 65 to 69 | Psoriasis | Both | 2.41 (2.40, 2.43) |
| African Region | 65 to 69 | Psoriasis | Female | 2.35 (2.32, 2.37) |
| African Region | 65 to 69 | Psoriasis | Male | 2.48 (2.45, 2.50) |
| African Region | 0 to 4 | RHD | Both | 0.15 (0.15, 0.16) |
| African Region | 0 to 4 | RHD | Female | 0.15 (0.15, 0.15) |
| African Region | 0 to 4 | RHD | Male | 0.16 (0.15, 0.16) |
| African Region | 10 to 14 | RHD | Both | 2.34 (2.33, 2.35) |
| African Region | 10 to 14 | RHD | Female | 2.39 (2.38, 2.40) |
| African Region | 10 to 14 | RHD | Male | 2.30 (2.29, 2.30) |
| African Region | 15 to 19 | RHD | Both | 3.64 (3.63, 3.66) |
| African Region | 15 to 19 | RHD | Female | 3.80 (3.78, 3.81) |
| African Region | 15 to 19 | RHD | Male | 3.49 (3.48, 3.51) |
| African Region | 20 to 24 | RHD | Both | 4.14 (4.12, 4.16) |
| African Region | 20 to 24 | RHD | Female | 4.34 (4.32, 4.36) |
| African Region | 20 to 24 | RHD | Male | 3.93 (3.91, 3.94) |
| African Region | 25 to 29 | RHD | Both | 4.24 (4.23, 4.26) |
| African Region | 25 to 29 | RHD | Female | 4.46 (4.45, 4.48) |
| African Region | 25 to 29 | RHD | Male | 4.01 (3.99, 4.03) |
| African Region | 30 to 34 | RHD | Both | 4.15 (4.13, 4.17) |
| African Region | 30 to 34 | RHD | Female | 4.38 (4.36, 4.40) |
| African Region | 30 to 34 | RHD | Male | 3.91 (3.90, 3.93) |
| African Region | 35 to 39 | RHD | Both | 3.90 (3.89, 3.92) |

| | | | | |
|---|---|---|---|---|
| African Region | 35 to 39 | RHD | Female | 4.12 (4.11, 4.14) |
| African Region | 35 to 39 | RHD | Male | 3.68 (3.66, 3.70) |
| African Region | 40 to 44 | RHD | Both | 3.52 (3.51, 3.54) |
| African Region | 40 to 44 | RHD | Female | 3.73 (3.71, 3.75) |
| African Region | 40 to 44 | RHD | Male | 3.32 (3.30, 3.34) |
| African Region | 45 to 49 | RHD | Both | 3.01 (2.99, 3.02) |
| African Region | 45 to 49 | RHD | Female | 3.19 (3.18, 3.21) |
| African Region | 45 to 49 | RHD | Male | 2.82 (2.80, 2.84) |
| African Region | 5 to 9 | RHD | Both | 1.00 (1.00, 1.00) |
| African Region | 5 to 9 | RHD | Female | 1.00 (1.00, 1.00) |
| African Region | 5 to 9 | RHD | Male | 1.00 (1.00, 1.00) |
| African Region | 50 to 54 | RHD | Both | 2.41 (2.40, 2.43) |
| African Region | 50 to 54 | RHD | Female | 2.58 (2.57, 2.60) |
| African Region | 50 to 54 | RHD | Male | 2.24 (2.23, 2.26) |
| African Region | 55 to 59 | RHD | Both | 1.92 (1.90, 1.93) |
| African Region | 55 to 59 | RHD | Female | 2.07 (2.05, 2.08) |
| African Region | 55 to 59 | RHD | Male | 1.76 (1.74, 1.78) |
| African Region | 60 to 64 | RHD | Both | 1.53 (1.51, 1.54) |
| African Region | 60 to 64 | RHD | Female | 1.66 (1.65, 1.68) |
| African Region | 60 to 64 | RHD | Male | 1.39 (1.37, 1.40) |
| African Region | 65 to 69 | RHD | Both | 1.22 (1.20, 1.23) |
| African Region | 65 to 69 | RHD | Female | 1.33 (1.32, 1.35) |
| African Region | 65 to 69 | RHD | Male | 1.09 (1.07, 1.11) |
| African Region | 10 to 14 | RA | Both | 7.29 (6.95, 7.65) |
| African Region | 10 to 14 | RA | Female | 7.41 (7.09, 7.74) |
| African Region | 10 to 14 | RA | Male | 7.06 (6.64, 7.50) |
| African Region | 15 to 19 | RA | Both | 21.75 (20.74, 22.81) |
| African Region | 15 to 19 | RA | Female | 22.47 (21.51, 23.47) |
| African Region | 15 to 19 | RA | Male | 19.98 (18.82, 21.22) |
| African Region | 20 to 24 | RA | Both | 45.46 (43.35, 47.67) |
| African Region | 20 to 24 | RA | Female | 46.83 (44.84, 48.90) |
| African Region | 20 to 24 | RA | Male | 40.93 (38.55, 43.47) |
| African Region | 25 to 29 | RA | Both | 80.28 (76.56, 84.20) |

| | | | | |
|---|---|---|---|---|
| African Region | 25 to 29 | RA | Female | 82.06 (78.58, 85.71) |
| African Region | 25 to 29 | RA | Male | 73.28 (69.00, 77.82) |
| African Region | 30 to 34 | RA | Both | 126.63 (120.74, 132.81) |
| African Region | 30 to 34 | RA | Female | 127.94 (122.49, 133.64) |
| African Region | 30 to 34 | RA | Male | 120.49 (113.45, 127.98) |
| African Region | 35 to 39 | RA | Both | 187.16 (178.43, 196.32) |
| African Region | 35 to 39 | RA | Female | 182.01 (174.24, 190.14) |
| African Region | 35 to 39 | RA | Male | 195.62 (184.16, 207.79) |
| African Region | 40 to 44 | RA | Both | 271.39 (258.71, 284.69) |
| African Region | 40 to 44 | RA | Female | 245.74 (235.22, 256.73) |
| African Region | 40 to 44 | RA | Male | 325.16 (306.09, 345.42) |
| African Region | 45 to 49 | RA | Both | 416.31 (396.83, 436.74) |
| African Region | 45 to 49 | RA | Female | 329.17 (315.06, 343.91) |
| African Region | 45 to 49 | RA | Male | 601.87 (566.54, 639.41) |
| African Region | 5 to 9 | RA | Both | 1.00 (1.00, 1.00) |
| African Region | 5 to 9 | RA | Female | 1.00 (1.00, 1.00) |
| African Region | 5 to 9 | RA | Male | 1.00 (1.00, 1.00) |
| African Region | 50 to 54 | RA | Both | 607.82 (579.35, 637.70) |
| African Region | 50 to 54 | RA | Female | 427.99 (409.60, 447.20) |
| African Region | 50 to 54 | RA | Male | 993.81 (935.41, 1055.86) |
| African Region | 55 to 59 | RA | Both | 777.86 (741.35, 816.16) |
| African Region | 55 to 59 | RA | Female | 533.52 (510.56, 557.52) |
| African Region | 55 to 59 | RA | Male | 1304.60 (1227.83, 1386.17) |
| African Region | 60 to 64 | RA | Both | 917.14 (874.03, 962.39) |
| African Region | 60 to 64 | RA | Female | 642.75 (615.03, 671.73) |
| African Region | 60 to 64 | RA | Male | 1507.72 (1418.89, 1602.12) |
| African Region | 65 to 69 | RA | Both | 1002.73 (955.49, 1052.31) |
| African Region | 65 to 69 | RA | Female | 717.31 (686.29, 749.74) |
| African Region | 65 to 69 | RA | Male | 1614.32 (1519.07, 1715.55) |
| Eastern Mediterranean Region | 0 to 4 | AA | Both | 0.71 (0.71, 0.72) |
| Eastern Mediterranean Region | 0 to 4 | AA | Female | 0.73 (0.72, 0.73) |
| Eastern Mediterranean Region | 0 to 4 | AA | Male | 0.68 (0.68, 0.69) |
| Eastern Mediterranean Region | 10 to 14 | AA | Both | 1.28 (1.28, 1.29) |

| | | | | |
|---|---|---|---|---|
| Eastern Mediterranean Region | 10 to 14 | AA | Female | 1.27 (1.26, 1.28) |
| Eastern Mediterranean Region | 10 to 14 | AA | Male | 1.31 (1.30, 1.32) |
| Eastern Mediterranean Region | 15 to 19 | AA | Both | 2.16 (2.15, 2.17) |
| Eastern Mediterranean Region | 15 to 19 | AA | Female | 2.20 (2.18, 2.21) |
| Eastern Mediterranean Region | 15 to 19 | AA | Male | 2.10 (2.08, 2.12) |
| Eastern Mediterranean Region | 20 to 24 | AA | Both | 3.39 (3.38, 3.41) |
| Eastern Mediterranean Region | 20 to 24 | AA | Female | 3.46 (3.43, 3.48) |
| Eastern Mediterranean Region | 20 to 24 | AA | Male | 3.28 (3.25, 3.31) |
| Eastern Mediterranean Region | 25 to 29 | AA | Both | 4.72 (4.70, 4.74) |
| Eastern Mediterranean Region | 25 to 29 | AA | Female | 4.80 (4.77, 4.83) |
| Eastern Mediterranean Region | 25 to 29 | AA | Male | 4.60 (4.56, 4.64) |
| Eastern Mediterranean Region | 30 to 34 | AA | Both | 4.99 (4.96, 5.02) |
| Eastern Mediterranean Region | 30 to 34 | AA | Female | 5.11 (5.08, 5.15) |
| Eastern Mediterranean Region | 30 to 34 | AA | Male | 4.86 (4.82, 4.90) |
| Eastern Mediterranean Region | 35 to 39 | AA | Both | 4.66 (4.64, 4.69) |
| Eastern Mediterranean Region | 35 to 39 | AA | Female | 4.78 (4.75, 4.82) |
| Eastern Mediterranean Region | 35 to 39 | AA | Male | 4.55 (4.51, 4.59) |
| Eastern Mediterranean Region | 40 to 44 | AA | Both | 3.96 (3.94, 3.99) |
| Eastern Mediterranean Region | 40 to 44 | AA | Female | 4.06 (4.03, 4.09) |
| Eastern Mediterranean Region | 40 to 44 | AA | Male | 3.88 (3.84, 3.92) |
| Eastern Mediterranean Region | 45 to 49 | AA | Both | 3.42 (3.40, 3.44) |
| Eastern Mediterranean Region | 45 to 49 | AA | Female | 3.51 (3.48, 3.54) |
| Eastern Mediterranean Region | 45 to 49 | AA | Male | 3.34 (3.30, 3.38) |
| Eastern Mediterranean Region | 5 to 9 | AA | Both | 1.00 (1.00, 1.00) |
| Eastern Mediterranean Region | 5 to 9 | AA | Female | 1.00 (1.00, 1.00) |
| Eastern Mediterranean Region | 5 to 9 | AA | Male | 1.00 (1.00, 1.00) |
| Eastern Mediterranean Region | 50 to 54 | AA | Both | 3.05 (3.03, 3.07) |
| Eastern Mediterranean Region | 50 to 54 | AA | Female | 3.13 (3.11, 3.16) |
| Eastern Mediterranean Region | 50 to 54 | AA | Male | 2.97 (2.94, 3.01) |
| Eastern Mediterranean Region | 55 to 59 | AA | Both | 2.88 (2.86, 2.90) |
| Eastern Mediterranean Region | 55 to 59 | AA | Female | 2.96 (2.93, 2.99) |
| Eastern Mediterranean Region | 55 to 59 | AA | Male | 2.82 (2.78, 2.86) |
| Eastern Mediterranean Region | 60 to 64 | AA | Both | 2.99 (2.96, 3.02) |

| | | | | |
|---|---|---|---|---|
| Eastern Mediterranean Region | 60 to 64 | AA | Female | 3.08 (3.04, 3.11) |
| Eastern Mediterranean Region | 60 to 64 | AA | Male | 2.92 (2.88, 2.97) |
| Eastern Mediterranean Region | 65 to 69 | AA | Both | 2.81 (2.78, 2.84) |
| Eastern Mediterranean Region | 65 to 69 | AA | Female | 2.88 (2.84, 2.92) |
| Eastern Mediterranean Region | 65 to 69 | AA | Male | 2.76 (2.71, 2.81) |
| Eastern Mediterranean Region | 0 to 4 | IBD | Both | 0.05 (0.03, 0.07) |
| Eastern Mediterranean Region | 0 to 4 | IBD | Female | 0.05 (0.03, 0.08) |
| Eastern Mediterranean Region | 0 to 4 | IBD | Male | 0.05 (0.03, 0.07) |
| Eastern Mediterranean Region | 10 to 14 | IBD | Both | 4.30 (3.94, 4.69) |
| Eastern Mediterranean Region | 10 to 14 | IBD | Female | 4.26 (3.82, 4.75) |
| Eastern Mediterranean Region | 10 to 14 | IBD | Male | 4.34 (3.98, 4.72) |
| Eastern Mediterranean Region | 15 to 19 | IBD | Both | 14.55 (13.38, 15.82) |
| Eastern Mediterranean Region | 15 to 19 | IBD | Female | 16.37 (14.76, 18.17) |
| Eastern Mediterranean Region | 15 to 19 | IBD | Male | 12.90 (11.87, 14.01) |
| Eastern Mediterranean Region | 20 to 24 | IBD | Both | 33.59 (30.90, 36.51) |
| Eastern Mediterranean Region | 20 to 24 | IBD | Female | 37.34 (33.66, 41.42) |
| Eastern Mediterranean Region | 20 to 24 | IBD | Male | 30.19 (27.80, 32.77) |
| Eastern Mediterranean Region | 25 to 29 | IBD | Both | 62.40 (57.40, 67.83) |
| Eastern Mediterranean Region | 25 to 29 | IBD | Female | 68.69 (61.92, 76.20) |
| Eastern Mediterranean Region | 25 to 29 | IBD | Male | 56.77 (52.29, 61.64) |
| Eastern Mediterranean Region | 30 to 34 | IBD | Both | 98.03 (90.16, 106.59) |
| Eastern Mediterranean Region | 30 to 34 | IBD | Female | 107.12 (96.53, 118.86) |
| Eastern Mediterranean Region | 30 to 34 | IBD | Male | 90.11 (82.97, 97.86) |
| Eastern Mediterranean Region | 35 to 39 | IBD | Both | 136.05 (125.09, 147.97) |
| Eastern Mediterranean Region | 35 to 39 | IBD | Female | 147.34 (132.74, 163.54) |
| Eastern Mediterranean Region | 35 to 39 | IBD | Male | 126.36 (116.32, 137.27) |
| Eastern Mediterranean Region | 40 to 44 | IBD | Both | 173.88 (159.84, 189.16) |
| Eastern Mediterranean Region | 40 to 44 | IBD | Female | 188.08 (169.40, 208.82) |
| Eastern Mediterranean Region | 40 to 44 | IBD | Male | 161.70 (148.82, 175.71) |
| Eastern Mediterranean Region | 45 to 49 | IBD | Both | 198.20 (182.14, 215.68) |
| Eastern Mediterranean Region | 45 to 49 | IBD | Female | 204.84 (184.43, 227.51) |
| Eastern Mediterranean Region | 45 to 49 | IBD | Male | 193.15 (177.71, 209.94) |
| Eastern Mediterranean Region | 5 to 9 | IBD | Both | 1.00 (1.00, 1.00) |

| | | | | |
|---|---|---|---|---|
| Eastern Mediterranean Region | 5 to 9 | IBD | Female | 1.00 (1.00, 1.00) |
| Eastern Mediterranean Region | 5 to 9 | IBD | Male | 1.00 (1.00, 1.00) |
| Eastern Mediterranean Region | 50 to 54 | IBD | Both | 207.99 (191.07, 226.41) |
| Eastern Mediterranean Region | 50 to 54 | IBD | Female | 198.49 (178.63, 220.56) |
| Eastern Mediterranean Region | 50 to 54 | IBD | Male | 218.23 (200.71, 237.27) |
| Eastern Mediterranean Region | 55 to 59 | IBD | Both | 220.89 (202.83, 240.55) |
| Eastern Mediterranean Region | 55 to 59 | IBD | Female | 210.75 (189.57, 234.29) |
| Eastern Mediterranean Region | 55 to 59 | IBD | Male | 232.01 (213.30, 252.37) |
| Eastern Mediterranean Region | 60 to 64 | IBD | Both | 220.51 (202.37, 240.27) |
| Eastern Mediterranean Region | 60 to 64 | IBD | Female | 209.71 (188.50, 233.30) |
| Eastern Mediterranean Region | 60 to 64 | IBD | Male | 232.37 (213.51, 252.90) |
| Eastern Mediterranean Region | 65 to 69 | IBD | Both | 207.22 (189.99, 226.01) |
| Eastern Mediterranean Region | 65 to 69 | IBD | Female | 194.57 (174.67, 216.73) |
| Eastern Mediterranean Region | 65 to 69 | IBD | Male | 220.79 (202.68, 240.51) |
| Eastern Mediterranean Region | 10 to 14 | MS | Both | 9.14 (8.19, 10.20) |
| Eastern Mediterranean Region | 10 to 14 | MS | Female | 9.66 (8.39, 11.12) |
| Eastern Mediterranean Region | 10 to 14 | MS | Male | 8.57 (7.18, 10.22) |
| Eastern Mediterranean Region | 15 to 19 | MS | Both | 36.57 (32.84, 40.73) |
| Eastern Mediterranean Region | 15 to 19 | MS | Female | 40.17 (34.98, 46.13) |
| Eastern Mediterranean Region | 15 to 19 | MS | Male | 31.76 (26.71, 37.77) |
| Eastern Mediterranean Region | 20 to 24 | MS | Both | 97.01 (87.13, 108.00) |
| Eastern Mediterranean Region | 20 to 24 | MS | Female | 108.12 (94.19, 124.12) |
| Eastern Mediterranean Region | 20 to 24 | MS | Male | 81.47 (68.54, 96.83) |
| Eastern Mediterranean Region | 25 to 29 | MS | Both | 196.14 (176.17, 218.37) |
| Eastern Mediterranean Region | 25 to 29 | MS | Female | 218.22 (190.09, 250.52) |
| Eastern Mediterranean Region | 25 to 29 | MS | Male | 166.95 (140.46, 198.42) |
| Eastern Mediterranean Region | 30 to 34 | MS | Both | 306.26 (275.05, 341.01) |
| Eastern Mediterranean Region | 30 to 34 | MS | Female | 337.67 (294.10, 387.70) |
| Eastern Mediterranean Region | 30 to 34 | MS | Male | 269.30 (226.54, 320.13) |
| Eastern Mediterranean Region | 35 to 39 | MS | Both | 408.42 (366.75, 454.82) |
| Eastern Mediterranean Region | 35 to 39 | MS | Female | 443.79 (386.46, 509.62) |
| Eastern Mediterranean Region | 35 to 39 | MS | Male | 371.81 (312.70, 442.08) |
| Eastern Mediterranean Region | 40 to 44 | MS | Both | 491.34 (441.17, 547.22) |

| | | | | |
|---|---|---|---|---|
| Eastern Mediterranean Region | 40 to 44 | MS | Female | 525.50 (457.56, 603.54) |
| Eastern Mediterranean Region | 40 to 44 | MS | Male | 460.20 (386.96, 547.29) |
| Eastern Mediterranean Region | 45 to 49 | MS | Both | 557.19 (500.23, 620.65) |
| Eastern Mediterranean Region | 45 to 49 | MS | Female | 591.96 (515.34, 679.97) |
| Eastern Mediterranean Region | 45 to 49 | MS | Male | 528.83 (444.57, 629.06) |
| Eastern Mediterranean Region | 5 to 9 | MS | Both | 1.00 (1.00, 1.00) |
| Eastern Mediterranean Region | 5 to 9 | MS | Female | 1.00 (1.00, 1.00) |
| Eastern Mediterranean Region | 5 to 9 | MS | Male | 1.00 (1.00, 1.00) |
| Eastern Mediterranean Region | 50 to 54 | MS | Both | 610.26 (547.78, 679.86) |
| Eastern Mediterranean Region | 50 to 54 | MS | Female | 649.17 (565.03, 745.83) |
| Eastern Mediterranean Region | 50 to 54 | MS | Male | 579.19 (486.77, 689.16) |
| Eastern Mediterranean Region | 55 to 59 | MS | Both | 641.58 (575.78, 714.90) |
| Eastern Mediterranean Region | 55 to 59 | MS | Female | 687.74 (598.46, 790.33) |
| Eastern Mediterranean Region | 55 to 59 | MS | Male | 601.91 (505.68, 716.45) |
| Eastern Mediterranean Region | 60 to 64 | MS | Both | 655.37 (588.00, 730.46) |
| Eastern Mediterranean Region | 60 to 64 | MS | Female | 709.90 (617.55, 816.06) |
| Eastern Mediterranean Region | 60 to 64 | MS | Male | 602.92 (506.29, 717.99) |
| Eastern Mediterranean Region | 65 to 69 | MS | Both | 667.57 (598.72, 744.35) |
| Eastern Mediterranean Region | 65 to 69 | MS | Female | 728.35 (633.31, 837.65) |
| Eastern Mediterranean Region | 65 to 69 | MS | Male | 603.19 (506.16, 718.81) |
| Eastern Mediterranean Region | 0 to 4 | Psoriasis | Both | 0.45 (0.44, 0.45) |
| Eastern Mediterranean Region | 0 to 4 | Psoriasis | Female | 0.44 (0.44, 0.45) |
| Eastern Mediterranean Region | 0 to 4 | Psoriasis | Male | 0.45 (0.44, 0.45) |
| Eastern Mediterranean Region | 10 to 14 | Psoriasis | Both | 1.18 (1.18, 1.19) |
| Eastern Mediterranean Region | 10 to 14 | Psoriasis | Female | 1.20 (1.19, 1.21) |
| Eastern Mediterranean Region | 10 to 14 | Psoriasis | Male | 1.17 (1.16, 1.18) |
| Eastern Mediterranean Region | 15 to 19 | Psoriasis | Both | 1.30 (1.29, 1.31) |
| Eastern Mediterranean Region | 15 to 19 | Psoriasis | Female | 1.33 (1.33, 1.34) |
| Eastern Mediterranean Region | 15 to 19 | Psoriasis | Male | 1.26 (1.25, 1.27) |
| Eastern Mediterranean Region | 20 to 24 | Psoriasis | Both | 1.46 (1.45, 1.47) |
| Eastern Mediterranean Region | 20 to 24 | Psoriasis | Female | 1.49 (1.48, 1.50) |
| Eastern Mediterranean Region | 20 to 24 | Psoriasis | Male | 1.43 (1.42, 1.43) |
| Eastern Mediterranean Region | 25 to 29 | Psoriasis | Both | 1.68 (1.67, 1.69) |

| | | | | |
|---|---|---|---|---|
| Eastern Mediterranean Region | 25 to 29 | Psoriasis | Female | 1.65 (1.64, 1.66) |
| Eastern Mediterranean Region | 25 to 29 | Psoriasis | Male | 1.71 (1.70, 1.72) |
| Eastern Mediterranean Region | 30 to 34 | Psoriasis | Both | 1.89 (1.88, 1.90) |
| Eastern Mediterranean Region | 30 to 34 | Psoriasis | Female | 1.81 (1.80, 1.82) |
| Eastern Mediterranean Region | 30 to 34 | Psoriasis | Male | 1.98 (1.97, 1.99) |
| Eastern Mediterranean Region | 35 to 39 | Psoriasis | Both | 2.01 (2.00, 2.03) |
| Eastern Mediterranean Region | 35 to 39 | Psoriasis | Female | 1.91 (1.90, 1.93) |
| Eastern Mediterranean Region | 35 to 39 | Psoriasis | Male | 2.12 (2.11, 2.14) |
| Eastern Mediterranean Region | 40 to 44 | Psoriasis | Both | 2.08 (2.07, 2.10) |
| Eastern Mediterranean Region | 40 to 44 | Psoriasis | Female | 1.98 (1.97, 2.00) |
| Eastern Mediterranean Region | 40 to 44 | Psoriasis | Male | 2.19 (2.18, 2.21) |
| Eastern Mediterranean Region | 45 to 49 | Psoriasis | Both | 2.15 (2.14, 2.17) |
| Eastern Mediterranean Region | 45 to 49 | Psoriasis | Female | 2.06 (2.04, 2.07) |
| Eastern Mediterranean Region | 45 to 49 | Psoriasis | Male | 2.26 (2.24, 2.27) |
| Eastern Mediterranean Region | 5 to 9 | Psoriasis | Both | 1.00 (1.00, 1.00) |
| Eastern Mediterranean Region | 5 to 9 | Psoriasis | Female | 1.00 (1.00, 1.00) |
| Eastern Mediterranean Region | 5 to 9 | Psoriasis | Male | 1.00 (1.00, 1.00) |
| Eastern Mediterranean Region | 50 to 54 | Psoriasis | Both | 2.26 (2.24, 2.27) |
| Eastern Mediterranean Region | 50 to 54 | Psoriasis | Female | 2.16 (2.14, 2.18) |
| Eastern Mediterranean Region | 50 to 54 | Psoriasis | Male | 2.36 (2.35, 2.38) |
| Eastern Mediterranean Region | 55 to 59 | Psoriasis | Both | 2.35 (2.34, 2.37) |
| Eastern Mediterranean Region | 55 to 59 | Psoriasis | Female | 2.25 (2.23, 2.27) |
| Eastern Mediterranean Region | 55 to 59 | Psoriasis | Male | 2.47 (2.45, 2.49) |
| Eastern Mediterranean Region | 60 to 64 | Psoriasis | Both | 2.34 (2.32, 2.36) |
| Eastern Mediterranean Region | 60 to 64 | Psoriasis | Female | 2.22 (2.20, 2.24) |
| Eastern Mediterranean Region | 60 to 64 | Psoriasis | Male | 2.47 (2.45, 2.50) |
| Eastern Mediterranean Region | 65 to 69 | Psoriasis | Both | 2.23 (2.21, 2.25) |
| Eastern Mediterranean Region | 65 to 69 | Psoriasis | Female | 2.09 (2.07, 2.11) |
| Eastern Mediterranean Region | 65 to 69 | Psoriasis | Male | 2.38 (2.36, 2.40) |
| Eastern Mediterranean Region | 0 to 4 | RHD | Both | 0.16 (0.16, 0.17) |
| Eastern Mediterranean Region | 0 to 4 | RHD | Female | 0.16 (0.16, 0.17) |
| Eastern Mediterranean Region | 0 to 4 | RHD | Male | 0.16 (0.16, 0.17) |
| Eastern Mediterranean Region | 10 to 14 | RHD | Both | 2.16 (2.12, 2.20) |

| | | | | |
|---|---|---|---|---|
| Eastern Mediterranean Region | 10 to 14 | RHD | Female | 2.17 (2.13, 2.21) |
| Eastern Mediterranean Region | 10 to 14 | RHD | Male | 2.15 (2.11, 2.19) |
| Eastern Mediterranean Region | 15 to 19 | RHD | Both | 3.35 (3.28, 3.41) |
| Eastern Mediterranean Region | 15 to 19 | RHD | Female | 3.42 (3.36, 3.49) |
| Eastern Mediterranean Region | 15 to 19 | RHD | Male | 3.27 (3.21, 3.33) |
| Eastern Mediterranean Region | 20 to 24 | RHD | Both | 4.21 (4.13, 4.29) |
| Eastern Mediterranean Region | 20 to 24 | RHD | Female | 4.42 (4.33, 4.51) |
| Eastern Mediterranean Region | 20 to 24 | RHD | Male | 3.99 (3.91, 4.07) |
| Eastern Mediterranean Region | 25 to 29 | RHD | Both | 4.63 (4.54, 4.73) |
| Eastern Mediterranean Region | 25 to 29 | RHD | Female | 4.97 (4.87, 5.07) |
| Eastern Mediterranean Region | 25 to 29 | RHD | Male | 4.29 (4.20, 4.38) |
| Eastern Mediterranean Region | 30 to 34 | RHD | Both | 4.73 (4.63, 4.83) |
| Eastern Mediterranean Region | 30 to 34 | RHD | Female | 5.14 (5.03, 5.25) |
| Eastern Mediterranean Region | 30 to 34 | RHD | Male | 4.33 (4.23, 4.42) |
| Eastern Mediterranean Region | 35 to 39 | RHD | Both | 4.61 (4.51, 4.71) |
| Eastern Mediterranean Region | 35 to 39 | RHD | Female | 5.05 (4.93, 5.16) |
| Eastern Mediterranean Region | 35 to 39 | RHD | Male | 4.19 (4.10, 4.28) |
| Eastern Mediterranean Region | 40 to 44 | RHD | Both | 4.32 (4.22, 4.43) |
| Eastern Mediterranean Region | 40 to 44 | RHD | Female | 4.75 (4.63, 4.86) |
| Eastern Mediterranean Region | 40 to 44 | RHD | Male | 3.92 (3.83, 4.01) |
| Eastern Mediterranean Region | 45 to 49 | RHD | Both | 3.81 (3.71, 3.91) |
| Eastern Mediterranean Region | 45 to 49 | RHD | Female | 4.20 (4.09, 4.31) |
| Eastern Mediterranean Region | 45 to 49 | RHD | Male | 3.44 (3.35, 3.53) |
| Eastern Mediterranean Region | 5 to 9 | RHD | Both | 1.00 (1.00, 1.00) |
| Eastern Mediterranean Region | 5 to 9 | RHD | Female | 1.00 (1.00, 1.00) |
| Eastern Mediterranean Region | 5 to 9 | RHD | Male | 1.00 (1.00, 1.00) |
| Eastern Mediterranean Region | 50 to 54 | RHD | Both | 3.14 (3.05, 3.23) |
| Eastern Mediterranean Region | 50 to 54 | RHD | Female | 3.48 (3.38, 3.58) |
| Eastern Mediterranean Region | 50 to 54 | RHD | Male | 2.81 (2.73, 2.90) |
| Eastern Mediterranean Region | 55 to 59 | RHD | Both | 2.54 (2.45, 2.62) |
| Eastern Mediterranean Region | 55 to 59 | RHD | Female | 2.84 (2.75, 2.93) |
| Eastern Mediterranean Region | 55 to 59 | RHD | Male | 2.25 (2.18, 2.33) |
| Eastern Mediterranean Region | 60 to 64 | RHD | Both | 2.04 (1.96, 2.12) |

| | | | | |
|---|---|---|---|---|
| Eastern Mediterranean Region | 60 to 64 | RHD | Female | 2.30 (2.21, 2.39) |
| Eastern Mediterranean Region | 60 to 64 | RHD | Male | 1.80 (1.73, 1.87) |
| Eastern Mediterranean Region | 65 to 69 | RHD | Both | 1.64 (1.56, 1.72) |
| Eastern Mediterranean Region | 65 to 69 | RHD | Female | 1.86 (1.77, 1.94) |
| Eastern Mediterranean Region | 65 to 69 | RHD | Male | 1.43 (1.36, 1.50) |
| Eastern Mediterranean Region | 10 to 14 | RA | Both | 7.81 (7.56, 8.07) |
| Eastern Mediterranean Region | 10 to 14 | RA | Female | 7.97 (7.63, 8.31) |
| Eastern Mediterranean Region | 10 to 14 | RA | Male | 7.59 (7.21, 7.99) |
| Eastern Mediterranean Region | 15 to 19 | RA | Both | 25.03 (24.23, 25.85) |
| Eastern Mediterranean Region | 15 to 19 | RA | Female | 26.20 (25.13, 27.33) |
| Eastern Mediterranean Region | 15 to 19 | RA | Male | 23.32 (22.18, 24.53) |
| Eastern Mediterranean Region | 20 to 24 | RA | Both | 51.67 (50.03, 53.36) |
| Eastern Mediterranean Region | 20 to 24 | RA | Female | 55.30 (53.03, 57.66) |
| Eastern Mediterranean Region | 20 to 24 | RA | Male | 46.28 (44.00, 48.67) |
| Eastern Mediterranean Region | 25 to 29 | RA | Both | 80.55 (77.99, 83.19) |
| Eastern Mediterranean Region | 25 to 29 | RA | Female | 88.37 (84.74, 92.15) |
| Eastern Mediterranean Region | 25 to 29 | RA | Male | 69.49 (66.07, 73.10) |
| Eastern Mediterranean Region | 30 to 34 | RA | Both | 110.97 (107.44, 114.62) |
| Eastern Mediterranean Region | 30 to 34 | RA | Female | 124.42 (119.30, 129.77) |
| Eastern Mediterranean Region | 30 to 34 | RA | Male | 93.10 (88.50, 97.94) |
| Eastern Mediterranean Region | 35 to 39 | RA | Both | 146.04 (141.37, 150.85) |
| Eastern Mediterranean Region | 35 to 39 | RA | Female | 165.05 (158.24, 172.16) |
| Eastern Mediterranean Region | 35 to 39 | RA | Male | 121.60 (115.57, 127.94) |
| Eastern Mediterranean Region | 40 to 44 | RA | Both | 187.20 (181.21, 193.39) |
| Eastern Mediterranean Region | 40 to 44 | RA | Female | 210.69 (201.97, 219.78) |
| Eastern Mediterranean Region | 40 to 44 | RA | Male | 157.19 (149.38, 165.41) |
| Eastern Mediterranean Region | 45 to 49 | RA | Both | 234.20 (226.69, 241.97) |
| Eastern Mediterranean Region | 45 to 49 | RA | Female | 259.85 (249.07, 271.10) |
| Eastern Mediterranean Region | 45 to 49 | RA | Male | 202.08 (192.01, 212.69) |
| Eastern Mediterranean Region | 5 to 9 | RA | Both | 1.00 (1.00, 1.00) |
| Eastern Mediterranean Region | 5 to 9 | RA | Female | 1.00 (1.00, 1.00) |
| Eastern Mediterranean Region | 5 to 9 | RA | Male | 1.00 (1.00, 1.00) |
| Eastern Mediterranean Region | 50 to 54 | RA | Both | 286.89 (277.65, 296.43) |

| | | | | |
|---|---|---|---|---|
| Eastern Mediterranean Region | 50 to 54 | RA | Female | 313.94 (300.88, 327.57) |
| Eastern Mediterranean Region | 50 to 54 | RA | Male | 253.96 (241.27, 267.33) |
| Eastern Mediterranean Region | 55 to 59 | RA | Both | 347.15 (335.95, 358.73) |
| Eastern Mediterranean Region | 55 to 59 | RA | Female | 382.52 (366.56, 399.17) |
| Eastern Mediterranean Region | 55 to 59 | RA | Male | 304.28 (289.02, 320.35) |
| Eastern Mediterranean Region | 60 to 64 | RA | Both | 418.05 (404.52, 432.04) |
| Eastern Mediterranean Region | 60 to 64 | RA | Female | 472.44 (452.68, 493.06) |
| Eastern Mediterranean Region | 60 to 64 | RA | Male | 350.33 (332.70, 368.90) |
| Eastern Mediterranean Region | 65 to 69 | RA | Both | 497.26 (481.11, 513.96) |
| Eastern Mediterranean Region | 65 to 69 | RA | Female | 583.37 (558.90, 608.92) |
| Eastern Mediterranean Region | 65 to 69 | RA | Male | 386.53 (366.98, 407.11) |
| European Region | 0 to 4 | AA | Both | 0.73 (0.72, 0.73) |
| European Region | 0 to 4 | AA | Female | 0.74 (0.74, 0.75) |
| European Region | 0 to 4 | AA | Male | 0.71 (0.71, 0.72) |
| European Region | 10 to 14 | AA | Both | 1.27 (1.26, 1.27) |
| European Region | 10 to 14 | AA | Female | 1.30 (1.29, 1.31) |
| European Region | 10 to 14 | AA | Male | 1.23 (1.22, 1.24) |
| European Region | 15 to 19 | AA | Both | 2.21 (2.20, 2.22) |
| European Region | 15 to 19 | AA | Female | 2.26 (2.24, 2.27) |
| European Region | 15 to 19 | AA | Male | 2.14 (2.12, 2.16) |
| European Region | 20 to 24 | AA | Both | 3.54 (3.52, 3.55) |
| European Region | 20 to 24 | AA | Female | 3.44 (3.41, 3.46) |
| European Region | 20 to 24 | AA | Male | 3.66 (3.63, 3.69) |
| European Region | 25 to 29 | AA | Both | 4.93 (4.91, 4.95) |
| European Region | 25 to 29 | AA | Female | 4.76 (4.73, 4.79) |
| European Region | 25 to 29 | AA | Male | 5.14 (5.10, 5.18) |
| European Region | 30 to 34 | AA | Both | 5.23 (5.21, 5.26) |
| European Region | 30 to 34 | AA | Female | 5.29 (5.26, 5.33) |
| European Region | 30 to 34 | AA | Male | 5.12 (5.07, 5.16) |
| European Region | 35 to 39 | AA | Both | 4.89 (4.86, 4.91) |
| European Region | 35 to 39 | AA | Female | 5.17 (5.14, 5.20) |
| European Region | 35 to 39 | AA | Male | 4.47 (4.43, 4.50) |
| European Region | 40 to 44 | AA | Both | 4.19 (4.17, 4.22) |

| | | | | |
|---|---|---|---|---|
| European Region | 40 to 44 | AA | Female | 4.57 (4.54, 4.60) |
| European Region | 40 to 44 | AA | Male | 3.63 (3.60, 3.66) |
| European Region | 45 to 49 | AA | Both | 3.61 (3.59, 3.63) |
| European Region | 45 to 49 | AA | Female | 4.04 (4.01, 4.06) |
| European Region | 45 to 49 | AA | Male | 2.97 (2.94, 2.99) |
| European Region | 5 to 9 | AA | Both | 1.00 (1.00, 1.00) |
| European Region | 5 to 9 | AA | Female | 1.00 (1.00, 1.00) |
| European Region | 5 to 9 | AA | Male | 1.00 (1.00, 1.00) |
| European Region | 50 to 54 | AA | Both | 3.25 (3.23, 3.27) |
| European Region | 50 to 54 | AA | Female | 3.74 (3.71, 3.76) |
| European Region | 50 to 54 | AA | Male | 2.48 (2.46, 2.50) |
| European Region | 55 to 59 | AA | Both | 3.15 (3.13, 3.17) |
| European Region | 55 to 59 | AA | Female | 3.74 (3.72, 3.77) |
| European Region | 55 to 59 | AA | Male | 2.16 (2.14, 2.19) |
| European Region | 60 to 64 | AA | Both | 3.31 (3.29, 3.33) |
| European Region | 60 to 64 | AA | Female | 3.99 (3.96, 4.02) |
| European Region | 60 to 64 | AA | Male | 2.09 (2.07, 2.12) |
| European Region | 65 to 69 | AA | Both | 3.12 (3.10, 3.14) |
| European Region | 65 to 69 | AA | Female | 3.77 (3.74, 3.80) |
| European Region | 65 to 69 | AA | Male | 1.86 (1.84, 1.88) |
| European Region | 0 to 4 | IBD | Both | 0.03 (0.01, 0.08) |
| European Region | 0 to 4 | IBD | Female | 0.03 (0.01, 0.08) |
| European Region | 0 to 4 | IBD | Male | 0.03 (0.01, 0.08) |
| European Region | 10 to 14 | IBD | Both | 5.82 (4.98, 6.81) |
| European Region | 10 to 14 | IBD | Female | 5.78 (4.93, 6.79) |
| European Region | 10 to 14 | IBD | Male | 5.87 (4.97, 6.92) |
| European Region | 15 to 19 | IBD | Both | 21.96 (18.87, 25.56) |
| European Region | 15 to 19 | IBD | Female | 23.60 (20.21, 27.57) |
| European Region | 15 to 19 | IBD | Male | 20.40 (17.37, 23.95) |
| European Region | 20 to 24 | IBD | Both | 54.66 (47.00, 63.58) |
| European Region | 20 to 24 | IBD | Female | 60.46 (51.80, 70.56) |
| European Region | 20 to 24 | IBD | Male | 49.05 (41.81, 57.55) |
| European Region | 25 to 29 | IBD | Both | 93.83 (80.68, 109.13) |

| | | | | |
|---|---|---|---|---|
| European Region | 25 to 29 | IBD | Female | 102.56 (87.88, 119.70) |
| European Region | 25 to 29 | IBD | Male | 85.32 (72.73, 100.09) |
| European Region | 30 to 34 | IBD | Both | 130.30 (112.02, 151.56) |
| European Region | 30 to 34 | IBD | Female | 141.55 (121.28, 165.22) |
| European Region | 30 to 34 | IBD | Male | 119.31 (101.69, 139.98) |
| European Region | 35 to 39 | IBD | Both | 161.39 (138.73, 187.75) |
| European Region | 35 to 39 | IBD | Female | 173.93 (149.00, 203.04) |
| European Region | 35 to 39 | IBD | Male | 149.06 (127.03, 174.92) |
| European Region | 40 to 44 | IBD | Both | 170.87 (146.86, 198.80) |
| European Region | 40 to 44 | IBD | Female | 170.80 (146.29, 199.41) |
| European Region | 40 to 44 | IBD | Male | 171.55 (146.17, 201.33) |
| European Region | 45 to 49 | IBD | Both | 182.48 (156.82, 212.33) |
| European Region | 45 to 49 | IBD | Female | 177.94 (152.39, 207.76) |
| European Region | 45 to 49 | IBD | Male | 187.91 (160.09, 220.56) |
| European Region | 5 to 9 | IBD | Both | 1.00 (1.00, 1.00) |
| European Region | 5 to 9 | IBD | Female | 1.00 (1.00, 1.00) |
| European Region | 5 to 9 | IBD | Male | 1.00 (1.00, 1.00) |
| European Region | 50 to 54 | IBD | Both | 192.00 (164.98, 223.44) |
| European Region | 50 to 54 | IBD | Female | 184.69 (158.15, 215.67) |
| European Region | 50 to 54 | IBD | Male | 200.50 (170.80, 235.37) |
| European Region | 55 to 59 | IBD | Both | 195.99 (168.39, 228.11) |
| European Region | 55 to 59 | IBD | Female | 186.48 (159.67, 217.80) |
| European Region | 55 to 59 | IBD | Male | 207.19 (176.46, 243.26) |
| European Region | 60 to 64 | IBD | Both | 190.61 (163.74, 221.89) |
| European Region | 60 to 64 | IBD | Female | 180.28 (154.33, 210.59) |
| European Region | 60 to 64 | IBD | Male | 203.20 (173.04, 238.63) |
| European Region | 65 to 69 | IBD | Both | 175.00 (150.29, 203.78) |
| European Region | 65 to 69 | IBD | Female | 165.11 (141.31, 192.92) |
| European Region | 65 to 69 | IBD | Male | 187.68 (159.77, 220.47) |
| European Region | 10 to 14 | MS | Both | 9.97 (9.19, 10.81) |
| European Region | 10 to 14 | MS | Female | 10.84 (9.70, 12.12) |
| European Region | 10 to 14 | MS | Male | 9.06 (8.04, 10.20) |
| European Region | 15 to 19 | MS | Both | 41.92 (38.71, 45.40) |

| | | | | |
|---|---|---|---|---|
| European Region | 15 to 19 | MS | Female | 49.01 (43.92, 54.68) |
| European Region | 15 to 19 | MS | Male | 34.28 (30.51, 38.51) |
| European Region | 20 to 24 | MS | Both | 111.22 (102.73, 120.42) |
| European Region | 20 to 24 | MS | Female | 135.54 (121.52, 151.19) |
| European Region | 20 to 24 | MS | Male | 84.38 (75.13, 94.77) |
| European Region | 25 to 29 | MS | Both | 225.85 (208.62, 244.51) |
| European Region | 25 to 29 | MS | Female | 280.09 (251.12, 312.42) |
| European Region | 25 to 29 | MS | Male | 165.26 (147.16, 185.60) |
| European Region | 30 to 34 | MS | Both | 355.49 (328.36, 384.86) |
| European Region | 30 to 34 | MS | Female | 440.56 (394.97, 491.41) |
| European Region | 30 to 34 | MS | Male | 259.87 (231.39, 291.85) |
| European Region | 35 to 39 | MS | Both | 477.87 (441.38, 517.37) |
| European Region | 35 to 39 | MS | Female | 586.80 (526.06, 654.56) |
| European Region | 35 to 39 | MS | Male | 354.00 (315.18, 397.59) |
| European Region | 40 to 44 | MS | Both | 577.65 (533.54, 625.41) |
| European Region | 40 to 44 | MS | Female | 701.43 (628.81, 782.44) |
| European Region | 40 to 44 | MS | Male | 434.71 (387.03, 488.25) |
| European Region | 45 to 49 | MS | Both | 650.98 (601.26, 704.82) |
| European Region | 45 to 49 | MS | Female | 778.74 (698.10, 868.70) |
| European Region | 45 to 49 | MS | Male | 499.65 (444.84, 561.22) |
| European Region | 5 to 9 | MS | Both | 1.00 (1.00, 1.00) |
| European Region | 5 to 9 | MS | Female | 1.00 (1.00, 1.00) |
| European Region | 5 to 9 | MS | Male | 1.00 (1.00, 1.00) |
| European Region | 50 to 54 | MS | Both | 695.70 (642.54, 753.25) |
| European Region | 50 to 54 | MS | Female | 818.85 (734.03, 913.46) |
| European Region | 50 to 54 | MS | Male | 544.20 (484.48, 611.29) |
| European Region | 55 to 59 | MS | Both | 708.10 (653.97, 766.70) |
| European Region | 55 to 59 | MS | Female | 826.33 (740.72, 921.84) |
| European Region | 55 to 59 | MS | Male | 555.31 (494.34, 623.80) |
| European Region | 60 to 64 | MS | Both | 697.23 (643.92, 754.96) |
| European Region | 60 to 64 | MS | Female | 812.67 (728.44, 906.63) |
| European Region | 60 to 64 | MS | Male | 539.97 (480.65, 606.61) |
| European Region | 65 to 69 | MS | Both | 680.96 (628.86, 737.38) |

| | | | | |
|---|---|---|---|---|
| European Region | 65 to 69 | MS | Female | 793.48 (711.21, 885.27) |
| European Region | 65 to 69 | MS | Male | 517.40 (460.51, 581.31) |
| European Region | 0 to 4 | Psoriasis | Both | 0.40 (0.39, 0.40) |
| European Region | 0 to 4 | Psoriasis | Female | 0.39 (0.39, 0.39) |
| European Region | 0 to 4 | Psoriasis | Male | 0.40 (0.40, 0.41) |
| European Region | 10 to 14 | Psoriasis | Both | 1.49 (1.48, 1.49) |
| European Region | 10 to 14 | Psoriasis | Female | 1.56 (1.55, 1.57) |
| European Region | 10 to 14 | Psoriasis | Male | 1.40 (1.40, 1.41) |
| European Region | 15 to 19 | Psoriasis | Both | 1.86 (1.85, 1.87) |
| European Region | 15 to 19 | Psoriasis | Female | 2.02 (2.01, 2.03) |
| European Region | 15 to 19 | Psoriasis | Male | 1.68 (1.67, 1.69) |
| European Region | 20 to 24 | Psoriasis | Both | 2.12 (2.11, 2.13) |
| European Region | 20 to 24 | Psoriasis | Female | 2.24 (2.23, 2.26) |
| European Region | 20 to 24 | Psoriasis | Male | 1.99 (1.98, 2.00) |
| European Region | 25 to 29 | Psoriasis | Both | 2.49 (2.48, 2.51) |
| European Region | 25 to 29 | Psoriasis | Female | 2.44 (2.43, 2.45) |
| European Region | 25 to 29 | Psoriasis | Male | 2.55 (2.53, 2.56) |
| European Region | 30 to 34 | Psoriasis | Both | 2.93 (2.91, 2.94) |
| European Region | 30 to 34 | Psoriasis | Female | 2.71 (2.70, 2.73) |
| European Region | 30 to 34 | Psoriasis | Male | 3.17 (3.15, 3.19) |
| European Region | 35 to 39 | Psoriasis | Both | 3.30 (3.28, 3.32) |
| European Region | 35 to 39 | Psoriasis | Female | 2.95 (2.93, 2.97) |
| European Region | 35 to 39 | Psoriasis | Male | 3.68 (3.65, 3.70) |
| European Region | 40 to 44 | Psoriasis | Both | 3.62 (3.60, 3.64) |
| European Region | 40 to 44 | Psoriasis | Female | 3.16 (3.15, 3.18) |
| European Region | 40 to 44 | Psoriasis | Male | 4.12 (4.09, 4.14) |
| European Region | 45 to 49 | Psoriasis | Both | 3.95 (3.93, 3.98) |
| European Region | 45 to 49 | Psoriasis | Female | 3.48 (3.46, 3.50) |
| European Region | 45 to 49 | Psoriasis | Male | 4.49 (4.46, 4.52) |
| European Region | 5 to 9 | Psoriasis | Both | 1.00 (1.00, 1.00) |
| European Region | 5 to 9 | Psoriasis | Female | 1.00 (1.00, 1.00) |
| European Region | 5 to 9 | Psoriasis | Male | 1.00 (1.00, 1.00) |
| European Region | 50 to 54 | Psoriasis | Both | 4.39 (4.37, 4.42) |

| | | | | |
|---|---|---|---|---|
| European Region | 50 to 54 | Psoriasis | Female | 3.96 (3.93, 3.98) |
| European Region | 50 to 54 | Psoriasis | Male | 4.89 (4.85, 4.92) |
| European Region | 55 to 59 | Psoriasis | Both | 4.81 (4.78, 4.84) |
| European Region | 55 to 59 | Psoriasis | Female | 4.36 (4.34, 4.39) |
| European Region | 55 to 59 | Psoriasis | Male | 5.33 (5.29, 5.36) |
| European Region | 60 to 64 | Psoriasis | Both | 5.07 (5.04, 5.10) |
| European Region | 60 to 64 | Psoriasis | Female | 4.58 (4.55, 4.60) |
| European Region | 60 to 64 | Psoriasis | Male | 5.65 (5.62, 5.69) |
| European Region | 65 to 69 | Psoriasis | Both | 5.14 (5.11, 5.17) |
| European Region | 65 to 69 | Psoriasis | Female | 4.60 (4.58, 4.63) |
| European Region | 65 to 69 | Psoriasis | Male | 5.81 (5.77, 5.85) |
| European Region | 0 to 4 | RHD | Both | 0.20 (0.17, 0.23) |
| European Region | 0 to 4 | RHD | Female | 0.20 (0.17, 0.24) |
| European Region | 0 to 4 | RHD | Male | 0.20 (0.17, 0.23) |
| European Region | 10 to 14 | RHD | Both | 1.95 (1.80, 2.11) |
| European Region | 10 to 14 | RHD | Female | 1.95 (1.78, 2.14) |
| European Region | 10 to 14 | RHD | Male | 1.94 (1.82, 2.07) |
| European Region | 15 to 19 | RHD | Both | 2.71 (2.51, 2.93) |
| European Region | 15 to 19 | RHD | Female | 2.71 (2.48, 2.97) |
| European Region | 15 to 19 | RHD | Male | 2.70 (2.53, 2.88) |
| European Region | 20 to 24 | RHD | Both | 3.03 (2.80, 3.28) |
| European Region | 20 to 24 | RHD | Female | 3.04 (2.77, 3.33) |
| European Region | 20 to 24 | RHD | Male | 3.02 (2.83, 3.23) |
| European Region | 25 to 29 | RHD | Both | 3.12 (2.88, 3.39) |
| European Region | 25 to 29 | RHD | Female | 3.13 (2.85, 3.44) |
| European Region | 25 to 29 | RHD | Male | 3.11 (2.91, 3.33) |
| European Region | 30 to 34 | RHD | Both | 3.10 (2.85, 3.37) |
| European Region | 30 to 34 | RHD | Female | 3.11 (2.82, 3.43) |
| European Region | 30 to 34 | RHD | Male | 3.09 (2.88, 3.31) |
| European Region | 35 to 39 | RHD | Both | 3.17 (2.91, 3.46) |
| European Region | 35 to 39 | RHD | Female | 3.16 (2.86, 3.50) |
| European Region | 35 to 39 | RHD | Male | 3.17 (2.95, 3.41) |
| European Region | 40 to 44 | RHD | Both | 3.40 (3.11, 3.72) |

| | | | | |
|---|---|---|---|---|
| European Region | 40 to 44 | RHD | Female | 3.38 (3.05, 3.74) |
| European Region | 40 to 44 | RHD | Male | 3.41 (3.17, 3.68) |
| European Region | 45 to 49 | RHD | Both | 4.09 (3.74, 4.48) |
| European Region | 45 to 49 | RHD | Female | 4.22 (3.80, 4.68) |
| European Region | 45 to 49 | RHD | Male | 3.92 (3.64, 4.24) |
| European Region | 5 to 9 | RHD | Both | 1.00 (1.00, 1.00) |
| European Region | 5 to 9 | RHD | Female | 1.00 (1.00, 1.00) |
| European Region | 5 to 9 | RHD | Male | 1.00 (1.00, 1.00) |
| European Region | 50 to 54 | RHD | Both | 5.22 (4.76, 5.72) |
| European Region | 50 to 54 | RHD | Female | 5.64 (5.07, 6.26) |
| European Region | 50 to 54 | RHD | Male | 4.69 (4.34, 5.06) |
| European Region | 55 to 59 | RHD | Both | 6.43 (5.87, 7.05) |
| European Region | 55 to 59 | RHD | Female | 7.12 (6.41, 7.92) |
| European Region | 55 to 59 | RHD | Male | 5.51 (5.09, 5.96) |
| European Region | 60 to 64 | RHD | Both | 7.48 (6.82, 8.21) |
| European Region | 60 to 64 | RHD | Female | 8.34 (7.50, 9.28) |
| European Region | 60 to 64 | RHD | Male | 6.25 (5.77, 6.77) |
| European Region | 65 to 69 | RHD | Both | 8.12 (7.38, 8.92) |
| European Region | 65 to 69 | RHD | Female | 9.00 (8.08, 10.02) |
| European Region | 65 to 69 | RHD | Male | 6.72 (6.19, 7.29) |
| European Region | 10 to 14 | RA | Both | 6.42 (5.87, 7.01) |
| European Region | 10 to 14 | RA | Female | 6.49 (5.89, 7.16) |
| European Region | 10 to 14 | RA | Male | 6.24 (5.80, 6.71) |
| European Region | 15 to 19 | RA | Both | 15.37 (14.09, 16.76) |
| European Region | 15 to 19 | RA | Female | 15.90 (14.45, 17.49) |
| European Region | 15 to 19 | RA | Male | 14.16 (13.18, 15.20) |
| European Region | 20 to 24 | RA | Both | 27.58 (25.29, 30.08) |
| European Region | 20 to 24 | RA | Female | 28.98 (26.35, 31.87) |
| European Region | 20 to 24 | RA | Male | 24.16 (22.50, 25.94) |
| European Region | 25 to 29 | RA | Both | 42.69 (39.14, 46.55) |
| European Region | 25 to 29 | RA | Female | 45.34 (41.22, 49.86) |
| European Region | 25 to 29 | RA | Male | 35.96 (33.50, 38.61) |
| European Region | 30 to 34 | RA | Both | 60.87 (55.81, 66.39) |

| | | | | |
|---|---|---|---|---|
| European Region | 30 to 34 | RA | Female | 65.07 (59.16, 71.57) |
| European Region | 30 to 34 | RA | Male | 50.07 (46.63, 53.76) |
| European Region | 35 to 39 | RA | Both | 84.97 (77.90, 92.69) |
| European Region | 35 to 39 | RA | Female | 90.77 (82.51, 99.85) |
| European Region | 35 to 39 | RA | Male | 69.42 (64.65, 74.55) |
| European Region | 40 to 44 | RA | Both | 115.87 (106.21, 126.40) |
| European Region | 40 to 44 | RA | Female | 123.20 (111.98, 135.53) |
| European Region | 40 to 44 | RA | Male | 94.86 (88.32, 101.87) |
| European Region | 45 to 49 | RA | Both | 154.40 (141.53, 168.44) |
| European Region | 45 to 49 | RA | Female | 162.77 (147.94, 179.08) |
| European Region | 45 to 49 | RA | Male | 127.35 (118.57, 136.78) |
| European Region | 5 to 9 | RA | Both | 1.00 (1.00, 1.00) |
| European Region | 5 to 9 | RA | Female | 1.00 (1.00, 1.00) |
| European Region | 5 to 9 | RA | Male | 1.00 (1.00, 1.00) |
| European Region | 50 to 54 | RA | Both | 200.72 (183.98, 219.00) |
| European Region | 50 to 54 | RA | Female | 208.85 (189.81, 229.79) |
| European Region | 50 to 54 | RA | Male | 167.89 (156.30, 180.33) |
| European Region | 55 to 59 | RA | Both | 248.91 (228.13, 271.59) |
| European Region | 55 to 59 | RA | Female | 253.41 (230.29, 278.84) |
| European Region | 55 to 59 | RA | Male | 214.53 (199.71, 230.45) |
| European Region | 60 to 64 | RA | Both | 296.98 (272.16, 324.06) |
| European Region | 60 to 64 | RA | Female | 294.25 (267.39, 323.81) |
| European Region | 60 to 64 | RA | Male | 266.25 (247.84, 286.02) |
| European Region | 65 to 69 | RA | Both | 335.13 (307.10, 365.72) |
| European Region | 65 to 69 | RA | Female | 322.89 (293.39, 355.36) |
| European Region | 65 to 69 | RA | Male | 310.44 (288.95, 333.52) |
| Region of the Americas | 0 to 4 | AA | Both | 0.73 (0.73, 0.74) |
| Region of the Americas | 0 to 4 | AA | Female | 0.73 (0.72, 0.74) |
| Region of the Americas | 0 to 4 | AA | Male | 0.73 (0.72, 0.74) |
| Region of the Americas | 10 to 14 | AA | Both | 1.27 (1.26, 1.28) |
| Region of the Americas | 10 to 14 | AA | Female | 1.29 (1.28, 1.31) |
| Region of the Americas | 10 to 14 | AA | Male | 1.23 (1.21, 1.24) |
| Region of the Americas | 15 to 19 | AA | Both | 2.23 (2.21, 2.25) |

| | | | | |
|---|---|---|---|---|
| Region of the Americas | 15 to 19 | AA | Female | 2.28 (2.26, 2.30) |
| Region of the Americas | 15 to 19 | AA | Male | 2.14 (2.12, 2.17) |
| Region of the Americas | 20 to 24 | AA | Both | 3.52 (3.49, 3.55) |
| Region of the Americas | 20 to 24 | AA | Female | 3.41 (3.39, 3.44) |
| Region of the Americas | 20 to 24 | AA | Male | 3.67 (3.62, 3.71) |
| Region of the Americas | 25 to 29 | AA | Both | 4.94 (4.90, 4.98) |
| Region of the Americas | 25 to 29 | AA | Female | 4.80 (4.76, 4.84) |
| Region of the Americas | 25 to 29 | AA | Male | 5.10 (5.04, 5.16) |
| Region of the Americas | 30 to 34 | AA | Both | 5.28 (5.24, 5.32) |
| Region of the Americas | 30 to 34 | AA | Female | 5.35 (5.30, 5.39) |
| Region of the Americas | 30 to 34 | AA | Male | 5.04 (4.97, 5.10) |
| Region of the Americas | 35 to 39 | AA | Both | 4.99 (4.95, 5.03) |
| Region of the Americas | 35 to 39 | AA | Female | 5.26 (5.22, 5.31) |
| Region of the Americas | 35 to 39 | AA | Male | 4.36 (4.30, 4.42) |
| Region of the Americas | 40 to 44 | AA | Both | 4.29 (4.26, 4.33) |
| Region of the Americas | 40 to 44 | AA | Female | 4.68 (4.63, 4.72) |
| Region of the Americas | 40 to 44 | AA | Male | 3.47 (3.43, 3.52) |
| Region of the Americas | 45 to 49 | AA | Both | 3.71 (3.68, 3.74) |
| Region of the Americas | 45 to 49 | AA | Female | 4.13 (4.10, 4.17) |
| Region of the Americas | 45 to 49 | AA | Male | 2.81 (2.77, 2.86) |
| Region of the Americas | 5 to 9 | AA | Both | 1.00 (1.00, 1.00) |
| Region of the Americas | 5 to 9 | AA | Female | 1.00 (1.00, 1.00) |
| Region of the Americas | 5 to 9 | AA | Male | 1.00 (1.00, 1.00) |
| Region of the Americas | 50 to 54 | AA | Both | 3.39 (3.36, 3.42) |
| Region of the Americas | 50 to 54 | AA | Female | 3.89 (3.85, 3.92) |
| Region of the Americas | 50 to 54 | AA | Male | 2.32 (2.28, 2.36) |
| Region of the Americas | 55 to 59 | AA | Both | 3.33 (3.29, 3.36) |
| Region of the Americas | 55 to 59 | AA | Female | 3.94 (3.90, 3.98) |
| Region of the Americas | 55 to 59 | AA | Male | 1.98 (1.95, 2.02) |
| Region of the Americas | 60 to 64 | AA | Both | 3.50 (3.47, 3.54) |
| Region of the Americas | 60 to 64 | AA | Female | 4.20 (4.16, 4.24) |
| Region of the Americas | 60 to 64 | AA | Male | 1.90 (1.87, 1.94) |
| Region of the Americas | 65 to 69 | AA | Both | 3.26 (3.23, 3.30) |

| | | | | |
|---|---|---|---|---|
| Region of the Americas | 65 to 69 | AA | Female | 3.93 (3.89, 3.97) |
| Region of the Americas | 65 to 69 | AA | Male | 1.64 (1.61, 1.68) |
| Region of the Americas | 0 to 4 | IBD | Both | 0.03 (0.01, 0.21) |
| Region of the Americas | 0 to 4 | IBD | Female | 0.03 (0.01, 0.22) |
| Region of the Americas | 0 to 4 | IBD | Male | 0.03 (0.01, 0.21) |
| Region of the Americas | 10 to 14 | IBD | Both | 5.53 (3.89, 7.87) |
| Region of the Americas | 10 to 14 | IBD | Female | 5.60 (3.90, 8.03) |
| Region of the Americas | 10 to 14 | IBD | Male | 5.48 (3.85, 7.80) |
| Region of the Americas | 15 to 19 | IBD | Both | 19.59 (13.91, 27.57) |
| Region of the Americas | 15 to 19 | IBD | Female | 22.38 (15.76, 31.78) |
| Region of the Americas | 15 to 19 | IBD | Male | 17.04 (12.08, 24.04) |
| Region of the Americas | 20 to 24 | IBD | Both | 43.40 (30.87, 61.02) |
| Region of the Americas | 20 to 24 | IBD | Female | 50.61 (35.68, 71.80) |
| Region of the Americas | 20 to 24 | IBD | Male | 36.71 (26.06, 51.72) |
| Region of the Americas | 25 to 29 | IBD | Both | 66.70 (47.42, 93.83) |
| Region of the Americas | 25 to 29 | IBD | Female | 78.27 (55.15, 111.09) |
| Region of the Americas | 25 to 29 | IBD | Male | 55.82 (39.60, 78.68) |
| Region of the Americas | 30 to 34 | IBD | Both | 83.16 (59.08, 117.05) |
| Region of the Americas | 30 to 34 | IBD | Female | 98.79 (69.58, 140.28) |
| Region of the Americas | 30 to 34 | IBD | Male | 68.35 (48.45, 96.41) |
| Region of the Americas | 35 to 39 | IBD | Both | 90.24 (64.08, 127.09) |
| Region of the Americas | 35 to 39 | IBD | Female | 106.97 (75.30, 151.96) |
| Region of the Americas | 35 to 39 | IBD | Male | 74.35 (52.67, 104.95) |
| Region of the Americas | 40 to 44 | IBD | Both | 84.50 (59.97, 119.07) |
| Region of the Americas | 40 to 44 | IBD | Female | 94.36 (66.39, 134.12) |
| Region of the Americas | 40 to 44 | IBD | Male | 75.15 (53.21, 106.13) |
| Region of the Americas | 45 to 49 | IBD | Both | 82.34 (58.41, 116.07) |
| Region of the Americas | 45 to 49 | IBD | Female | 90.72 (63.80, 128.99) |
| Region of the Americas | 45 to 49 | IBD | Male | 74.34 (52.61, 105.05) |
| Region of the Americas | 5 to 9 | IBD | Both | 1.00 (1.00, 1.00) |
| Region of the Americas | 5 to 9 | IBD | Female | 1.00 (1.00, 1.00) |
| Region of the Americas | 5 to 9 | IBD | Male | 1.00 (1.00, 1.00) |
| Region of the Americas | 50 to 54 | IBD | Both | 81.07 (57.48, 114.34) |

| | | | | |
|---|---|---|---|---|
| Region of the Americas | 50 to 54 | IBD | Female | 88.45 (62.17, 125.82) |
| Region of the Americas | 50 to 54 | IBD | Male | 73.96 (52.31, 104.57) |
| Region of the Americas | 55 to 59 | IBD | Both | 80.04 (56.71, 112.95) |
| Region of the Americas | 55 to 59 | IBD | Female | 86.41 (60.71, 122.99) |
| Region of the Americas | 55 to 59 | IBD | Male | 73.83 (52.19, 104.46) |
| Region of the Americas | 60 to 64 | IBD | Both | 78.64 (55.68, 111.07) |
| Region of the Americas | 60 to 64 | IBD | Female | 84.42 (59.27, 120.24) |
| Region of the Americas | 60 to 64 | IBD | Male | 72.98 (51.54, 103.34) |
| Region of the Americas | 65 to 69 | IBD | Both | 75.08 (53.10, 106.16) |
| Region of the Americas | 65 to 69 | IBD | Female | 80.82 (56.68, 115.24) |
| Region of the Americas | 65 to 69 | IBD | Male | 69.43 (48.97, 98.45) |
| Region of the Americas | 10 to 14 | MS | Both | 9.51 (8.36, 10.82) |
| Region of the Americas | 10 to 14 | MS | Female | 10.14 (8.78, 11.69) |
| Region of the Americas | 10 to 14 | MS | Male | 8.71 (7.49, 10.13) |
| Region of the Americas | 15 to 19 | MS | Both | 42.49 (37.46, 48.20) |
| Region of the Americas | 15 to 19 | MS | Female | 48.79 (42.41, 56.14) |
| Region of the Americas | 15 to 19 | MS | Male | 34.01 (29.34, 39.43) |
| Region of the Americas | 20 to 24 | MS | Both | 132.47 (116.83, 150.20) |
| Region of the Americas | 20 to 24 | MS | Female | 160.05 (139.16, 184.08) |
| Region of the Americas | 20 to 24 | MS | Male | 94.01 (81.14, 108.93) |
| Region of the Americas | 25 to 29 | MS | Both | 305.20 (269.17, 346.04) |
| Region of the Americas | 25 to 29 | MS | Female | 375.18 (326.23, 431.48) |
| Region of the Americas | 25 to 29 | MS | Male | 205.06 (176.99, 237.57) |
| Region of the Americas | 30 to 34 | MS | Both | 504.60 (445.03, 572.15) |
| Region of the Americas | 30 to 34 | MS | Female | 620.43 (539.46, 713.55) |
| Region of the Americas | 30 to 34 | MS | Male | 336.62 (290.53, 390.01) |
| Region of the Americas | 35 to 39 | MS | Both | 680.62 (600.24, 771.76) |
| Region of the Americas | 35 to 39 | MS | Female | 829.91 (721.57, 954.51) |
| Region of the Americas | 35 to 39 | MS | Male | 461.97 (398.70, 535.28) |
| Region of the Americas | 40 to 44 | MS | Both | 811.77 (715.88, 920.49) |
| Region of the Americas | 40 to 44 | MS | Female | 975.27 (847.94, 1121.72) |
| Region of the Americas | 40 to 44 | MS | Male | 570.61 (492.44, 661.19) |
| Region of the Americas | 45 to 49 | MS | Both | 904.86 (797.96, 1026.08) |

| | | | | |
|---|---|---|---|---|
| Region of the Americas | 45 to 49 | MS | Female | 1069.61 (929.94, 1230.26) |
| Region of the Americas | 45 to 49 | MS | Male | 657.44 (567.35, 761.84) |
| Region of the Americas | 5 to 9 | MS | Both | 1.00 (1.00, 1.00) |
| Region of the Americas | 5 to 9 | MS | Female | 1.00 (1.00, 1.00) |
| Region of the Americas | 5 to 9 | MS | Male | 1.00 (1.00, 1.00) |
| Region of the Americas | 50 to 54 | MS | Both | 957.61 (844.45, 1085.94) |
| Region of the Americas | 50 to 54 | MS | Female | 1114.87 (969.26, 1282.36) |
| Region of the Americas | 50 to 54 | MS | Male | 715.45 (617.37, 829.11) |
| Region of the Americas | 55 to 59 | MS | Both | 973.95 (858.82, 1104.51) |
| Region of the Americas | 55 to 59 | MS | Female | 1124.82 (977.87, 1293.86) |
| Region of the Americas | 55 to 59 | MS | Male | 732.54 (632.07, 848.97) |
| Region of the Americas | 60 to 64 | MS | Both | 955.64 (842.63, 1083.82) |
| Region of the Americas | 60 to 64 | MS | Female | 1103.24 (959.06, 1269.10) |
| Region of the Americas | 60 to 64 | MS | Male | 709.70 (612.30, 822.58) |
| Region of the Americas | 65 to 69 | MS | Both | 915.18 (806.88, 1038.01) |
| Region of the Americas | 65 to 69 | MS | Female | 1057.33 (919.08, 1216.37) |
| Region of the Americas | 65 to 69 | MS | Male | 664.86 (573.54, 770.73) |
| Region of the Americas | 0 to 4 | Psoriasis | Both | 0.40 (0.39, 0.42) |
| Region of the Americas | 0 to 4 | Psoriasis | Female | 0.40 (0.38, 0.41) |
| Region of the Americas | 0 to 4 | Psoriasis | Male | 0.41 (0.39, 0.43) |
| Region of the Americas | 10 to 14 | Psoriasis | Both | 1.20 (1.16, 1.23) |
| Region of the Americas | 10 to 14 | Psoriasis | Female | 1.25 (1.21, 1.29) |
| Region of the Americas | 10 to 14 | Psoriasis | Male | 1.14 (1.11, 1.18) |
| Region of the Americas | 15 to 19 | Psoriasis | Both | 1.26 (1.22, 1.29) |
| Region of the Americas | 15 to 19 | Psoriasis | Female | 1.35 (1.31, 1.39) |
| Region of the Americas | 15 to 19 | Psoriasis | Male | 1.15 (1.12, 1.19) |
| Region of the Americas | 20 to 24 | Psoriasis | Both | 1.34 (1.30, 1.38) |
| Region of the Americas | 20 to 24 | Psoriasis | Female | 1.43 (1.39, 1.48) |
| Region of the Americas | 20 to 24 | Psoriasis | Male | 1.24 (1.21, 1.28) |
| Region of the Americas | 25 to 29 | Psoriasis | Both | 1.61 (1.56, 1.66) |
| Region of the Americas | 25 to 29 | Psoriasis | Female | 1.63 (1.58, 1.69) |
| Region of the Americas | 25 to 29 | Psoriasis | Male | 1.57 (1.53, 1.62) |
| Region of the Americas | 30 to 34 | Psoriasis | Both | 1.87 (1.81, 1.93) |

| | | | | |
|---|---|---|---|---|
| Region of the Americas | 30 to 34 | Psoriasis | Female | 1.83 (1.77, 1.90) |
| Region of the Americas | 30 to 34 | Psoriasis | Male | 1.91 (1.85, 1.97) |
| Region of the Americas | 35 to 39 | Psoriasis | Both | 2.05 (1.98, 2.12) |
| Region of the Americas | 35 to 39 | Psoriasis | Female | 1.96 (1.89, 2.03) |
| Region of the Americas | 35 to 39 | Psoriasis | Male | 2.14 (2.08, 2.21) |
| Region of the Americas | 40 to 44 | Psoriasis | Both | 2.20 (2.13, 2.28) |
| Region of the Americas | 40 to 44 | Psoriasis | Female | 2.07 (2.00, 2.15) |
| Region of the Americas | 40 to 44 | Psoriasis | Male | 2.34 (2.26, 2.41) |
| Region of the Americas | 45 to 49 | Psoriasis | Both | 2.35 (2.26, 2.43) |
| Region of the Americas | 45 to 49 | Psoriasis | Female | 2.22 (2.14, 2.30) |
| Region of the Americas | 45 to 49 | Psoriasis | Male | 2.48 (2.40, 2.57) |
| Region of the Americas | 5 to 9 | Psoriasis | Both | 1.00 (1.00, 1.00) |
| Region of the Americas | 5 to 9 | Psoriasis | Female | 1.00 (1.00, 1.00) |
| Region of the Americas | 5 to 9 | Psoriasis | Male | 1.00 (1.00, 1.00) |
| Region of the Americas | 50 to 54 | Psoriasis | Both | 2.56 (2.47, 2.65) |
| Region of the Americas | 50 to 54 | Psoriasis | Female | 2.47 (2.38, 2.56) |
| Region of the Americas | 50 to 54 | Psoriasis | Male | 2.66 (2.57, 2.75) |
| Region of the Americas | 55 to 59 | Psoriasis | Both | 2.75 (2.65, 2.86) |
| Region of the Americas | 55 to 59 | Psoriasis | Female | 2.66 (2.56, 2.77) |
| Region of the Americas | 55 to 59 | Psoriasis | Male | 2.85 (2.75, 2.96) |
| Region of the Americas | 60 to 64 | Psoriasis | Both | 2.91 (2.80, 3.02) |
| Region of the Americas | 60 to 64 | Psoriasis | Female | 2.81 (2.70, 2.93) |
| Region of the Americas | 60 to 64 | Psoriasis | Male | 3.02 (2.91, 3.13) |
| Region of the Americas | 65 to 69 | Psoriasis | Both | 3.05 (2.93, 3.17) |
| Region of the Americas | 65 to 69 | Psoriasis | Female | 2.96 (2.84, 3.09) |
| Region of the Americas | 65 to 69 | Psoriasis | Male | 3.13 (3.01, 3.26) |
| Region of the Americas | 0 to 4 | RHD | Both | 0.17 (0.17, 0.17) |
| Region of the Americas | 0 to 4 | RHD | Female | 0.17 (0.17, 0.17) |
| Region of the Americas | 0 to 4 | RHD | Male | 0.17 (0.17, 0.18) |
| Region of the Americas | 10 to 14 | RHD | Both | 2.16 (2.14, 2.18) |
| Region of the Americas | 10 to 14 | RHD | Female | 2.20 (2.18, 2.22) |
| Region of the Americas | 10 to 14 | RHD | Male | 2.12 (2.10, 2.15) |
| Region of the Americas | 15 to 19 | RHD | Both | 3.17 (3.14, 3.20) |

| | | | | |
|---|---|---|---|---|
| Region of the Americas | 15 to 19 | RHD | Female | 3.27 (3.24, 3.30) |
| Region of the Americas | 15 to 19 | RHD | Male | 3.06 (3.02, 3.09) |
| Region of the Americas | 20 to 24 | RHD | Both | 3.66 (3.62, 3.69) |
| Region of the Americas | 20 to 24 | RHD | Female | 3.81 (3.77, 3.84) |
| Region of the Americas | 20 to 24 | RHD | Male | 3.48 (3.44, 3.52) |
| Region of the Americas | 25 to 29 | RHD | Both | 3.82 (3.78, 3.86) |
| Region of the Americas | 25 to 29 | RHD | Female | 4.01 (3.97, 4.05) |
| Region of the Americas | 25 to 29 | RHD | Male | 3.58 (3.54, 3.62) |
| Region of the Americas | 30 to 34 | RHD | Both | 3.81 (3.77, 3.85) |
| Region of the Americas | 30 to 34 | RHD | Female | 4.03 (3.99, 4.07) |
| Region of the Americas | 30 to 34 | RHD | Male | 3.52 (3.48, 3.57) |
| Region of the Americas | 35 to 39 | RHD | Both | 3.69 (3.65, 3.73) |
| Region of the Americas | 35 to 39 | RHD | Female | 3.94 (3.90, 3.98) |
| Region of the Americas | 35 to 39 | RHD | Male | 3.38 (3.33, 3.42) |
| Region of the Americas | 40 to 44 | RHD | Both | 3.46 (3.42, 3.50) |
| Region of the Americas | 40 to 44 | RHD | Female | 3.72 (3.68, 3.76) |
| Region of the Americas | 40 to 44 | RHD | Male | 3.14 (3.10, 3.18) |
| Region of the Americas | 45 to 49 | RHD | Both | 3.11 (3.07, 3.15) |
| Region of the Americas | 45 to 49 | RHD | Female | 3.36 (3.32, 3.40) |
| Region of the Americas | 45 to 49 | RHD | Male | 2.79 (2.75, 2.84) |
| Region of the Americas | 5 to 9 | RHD | Both | 1.00 (1.00, 1.00) |
| Region of the Americas | 5 to 9 | RHD | Female | 1.00 (1.00, 1.00) |
| Region of the Americas | 5 to 9 | RHD | Male | 1.00 (1.00, 1.00) |
| Region of the Americas | 50 to 54 | RHD | Both | 2.73 (2.69, 2.76) |
| Region of the Americas | 50 to 54 | RHD | Female | 2.96 (2.92, 2.99) |
| Region of the Americas | 50 to 54 | RHD | Male | 2.43 (2.39, 2.47) |
| Region of the Americas | 55 to 59 | RHD | Both | 2.50 (2.46, 2.53) |
| Region of the Americas | 55 to 59 | RHD | Female | 2.69 (2.65, 2.72) |
| Region of the Americas | 55 to 59 | RHD | Male | 2.24 (2.20, 2.28) |
| Region of the Americas | 60 to 64 | RHD | Both | 2.45 (2.42, 2.49) |
| Region of the Americas | 60 to 64 | RHD | Female | 2.59 (2.56, 2.63) |
| Region of the Americas | 60 to 64 | RHD | Male | 2.25 (2.21, 2.29) |
| Region of the Americas | 65 to 69 | RHD | Both | 2.65 (2.61, 2.70) |

| | | | | |
|---|---|---|---|---|
| Region of the Americas | 65 to 69 | RHD | Female | 2.73 (2.69, 2.78) |
| Region of the Americas | 65 to 69 | RHD | Male | 2.51 (2.46, 2.56) |
| Region of the Americas | 10 to 14 | RA | Both | 6.55 (6.37, 6.75) |
| Region of the Americas | 10 to 14 | RA | Female | 6.59 (6.37, 6.82) |
| Region of the Americas | 10 to 14 | RA | Male | 6.47 (6.27, 6.67) |
| Region of the Americas | 15 to 19 | RA | Both | 16.21 (15.75, 16.68) |
| Region of the Americas | 15 to 19 | RA | Female | 16.42 (15.87, 16.98) |
| Region of the Americas | 15 to 19 | RA | Male | 15.57 (15.10, 16.05) |
| Region of the Americas | 20 to 24 | RA | Both | 29.38 (28.55, 30.23) |
| Region of the Americas | 20 to 24 | RA | Female | 29.90 (28.91, 30.94) |
| Region of the Americas | 20 to 24 | RA | Male | 27.43 (26.61, 28.27) |
| Region of the Americas | 25 to 29 | RA | Both | 45.16 (43.88, 46.47) |
| Region of the Americas | 25 to 29 | RA | Female | 46.32 (44.77, 47.92) |
| Region of the Americas | 25 to 29 | RA | Male | 40.66 (39.45, 41.92) |
| Region of the Americas | 30 to 34 | RA | Both | 64.13 (62.32, 65.99) |
| Region of the Americas | 30 to 34 | RA | Female | 66.30 (64.08, 68.60) |
| Region of the Americas | 30 to 34 | RA | Male | 55.92 (54.24, 57.65) |
| Region of the Americas | 35 to 39 | RA | Both | 89.11 (86.59, 91.71) |
| Region of the Americas | 35 to 39 | RA | Female | 92.37 (89.27, 95.58) |
| Region of the Americas | 35 to 39 | RA | Male | 76.66 (74.36, 79.04) |
| Region of the Americas | 40 to 44 | RA | Both | 120.97 (117.54, 124.50) |
| Region of the Americas | 40 to 44 | RA | Female | 125.13 (120.93, 129.48) |
| Region of the Americas | 40 to 44 | RA | Male | 104.44 (101.29, 107.68) |
| Region of the Americas | 45 to 49 | RA | Both | 160.36 (155.81, 165.05) |
| Region of the Americas | 45 to 49 | RA | Female | 164.64 (159.10, 170.38) |
| Region of the Americas | 45 to 49 | RA | Male | 140.49 (136.25, 144.86) |
| Region of the Americas | 5 to 9 | RA | Both | 1.00 (1.00, 1.00) |
| Region of the Americas | 5 to 9 | RA | Female | 1.00 (1.00, 1.00) |
| Region of the Americas | 5 to 9 | RA | Male | 1.00 (1.00, 1.00) |
| Region of the Americas | 50 to 54 | RA | Both | 206.51 (200.63, 212.55) |
| Region of the Americas | 50 to 54 | RA | Female | 209.41 (202.35, 216.72) |
| Region of the Americas | 50 to 54 | RA | Male | 185.76 (180.14, 191.55) |
| Region of the Americas | 55 to 59 | RA | Both | 256.13 (248.83, 263.64) |

| | | | | |
|---|---|---|---|---|
| Region of the Americas | 55 to 59 | RA | Female | 254.33 (245.74, 263.22) |
| Region of the Americas | 55 to 59 | RA | Male | 240.76 (233.47, 248.29) |
| Region of the Americas | 60 to 64 | RA | Both | 308.29 (299.49, 317.35) |
| Region of the Americas | 60 to 64 | RA | Female | 298.85 (288.74, 309.31) |
| Region of the Americas | 60 to 64 | RA | Male | 304.31 (295.07, 313.84) |
| Region of the Americas | 65 to 69 | RA | Both | 359.52 (349.23, 370.10) |
| Region of the Americas | 65 to 69 | RA | Female | 340.84 (329.28, 352.79) |
| Region of the Americas | 65 to 69 | RA | Male | 367.94 (356.75, 379.49) |
| South-East Asia Region | 0 to 4 | AA | Both | 0.74 (0.74, 0.75) |
| South-East Asia Region | 0 to 4 | AA | Female | 0.74 (0.74, 0.75) |
| South-East Asia Region | 0 to 4 | AA | Male | 0.74 (0.74, 0.75) |
| South-East Asia Region | 10 to 14 | AA | Both | 1.29 (1.28, 1.29) |
| South-East Asia Region | 10 to 14 | AA | Female | 1.28 (1.27, 1.28) |
| South-East Asia Region | 10 to 14 | AA | Male | 1.30 (1.30, 1.31) |
| South-East Asia Region | 15 to 19 | AA | Both | 2.23 (2.22, 2.23) |
| South-East Asia Region | 15 to 19 | AA | Female | 2.22 (2.21, 2.22) |
| South-East Asia Region | 15 to 19 | AA | Male | 2.24 (2.23, 2.25) |
| South-East Asia Region | 20 to 24 | AA | Both | 3.55 (3.54, 3.56) |
| South-East Asia Region | 20 to 24 | AA | Female | 3.50 (3.49, 3.52) |
| South-East Asia Region | 20 to 24 | AA | Male | 3.56 (3.54, 3.57) |
| South-East Asia Region | 25 to 29 | AA | Both | 4.91 (4.90, 4.92) |
| South-East Asia Region | 25 to 29 | AA | Female | 4.83 (4.81, 4.85) |
| South-East Asia Region | 25 to 29 | AA | Male | 4.92 (4.90, 4.94) |
| South-East Asia Region | 30 to 34 | AA | Both | 5.24 (5.22, 5.25) |
| South-East Asia Region | 30 to 34 | AA | Female | 5.19 (5.16, 5.21) |
| South-East Asia Region | 30 to 34 | AA | Male | 5.20 (5.17, 5.23) |
| South-East Asia Region | 35 to 39 | AA | Both | 4.88 (4.87, 4.90) |
| South-East Asia Region | 35 to 39 | AA | Female | 4.83 (4.81, 4.85) |
| South-East Asia Region | 35 to 39 | AA | Male | 4.89 (4.87, 4.92) |
| South-East Asia Region | 40 to 44 | AA | Both | 4.14 (4.13, 4.15) |
| South-East Asia Region | 40 to 44 | AA | Female | 4.10 (4.08, 4.12) |
| South-East Asia Region | 40 to 44 | AA | Male | 4.16 (4.14, 4.18) |
| South-East Asia Region | 45 to 49 | AA | Both | 3.58 (3.57, 3.60) |

| | | | | |
|---|---|---|---|---|
| South-East Asia Region | 45 to 49 | AA | Female | 3.55 (3.53, 3.57) |
| South-East Asia Region | 45 to 49 | AA | Male | 3.60 (3.57, 3.62) |
| South-East Asia Region | 5 to 9 | AA | Both | 1.00 (1.00, 1.00) |
| South-East Asia Region | 5 to 9 | AA | Female | 1.00 (1.00, 1.00) |
| South-East Asia Region | 5 to 9 | AA | Male | 1.00 (1.00, 1.00) |
| South-East Asia Region | 50 to 54 | AA | Both | 3.20 (3.18, 3.21) |
| South-East Asia Region | 50 to 54 | AA | Female | 3.16 (3.15, 3.18) |
| South-East Asia Region | 50 to 54 | AA | Male | 3.18 (3.16, 3.21) |
| South-East Asia Region | 55 to 59 | AA | Both | 3.06 (3.04, 3.07) |
| South-East Asia Region | 55 to 59 | AA | Female | 2.99 (2.97, 3.00) |
| South-East Asia Region | 55 to 59 | AA | Male | 3.06 (3.04, 3.08) |
| South-East Asia Region | 60 to 64 | AA | Both | 3.17 (3.15, 3.18) |
| South-East Asia Region | 60 to 64 | AA | Female | 3.09 (3.07, 3.11) |
| South-East Asia Region | 60 to 64 | AA | Male | 3.12 (3.10, 3.14) |
| South-East Asia Region | 65 to 69 | AA | Both | 2.98 (2.96, 2.99) |
| South-East Asia Region | 65 to 69 | AA | Female | 2.88 (2.86, 2.90) |
| South-East Asia Region | 65 to 69 | AA | Male | 2.93 (2.90, 2.95) |
| South-East Asia Region | 0 to 4 | IBD | Both | 0.05 (0.04, 0.08) |
| South-East Asia Region | 0 to 4 | IBD | Female | 0.05 (0.04, 0.08) |
| South-East Asia Region | 0 to 4 | IBD | Male | 0.05 (0.04, 0.08) |
| South-East Asia Region | 10 to 14 | IBD | Both | 3.88 (3.54, 4.25) |
| South-East Asia Region | 10 to 14 | IBD | Female | 3.76 (3.41, 4.15) |
| South-East Asia Region | 10 to 14 | IBD | Male | 3.98 (3.60, 4.41) |
| South-East Asia Region | 15 to 19 | IBD | Both | 11.79 (10.80, 12.88) |
| South-East Asia Region | 15 to 19 | IBD | Female | 12.92 (11.78, 14.18) |
| South-East Asia Region | 15 to 19 | IBD | Male | 10.85 (9.83, 11.96) |
| South-East Asia Region | 20 to 24 | IBD | Both | 25.75 (23.58, 28.11) |
| South-East Asia Region | 20 to 24 | IBD | Female | 27.73 (25.28, 30.42) |
| South-East Asia Region | 20 to 24 | IBD | Male | 24.07 (21.83, 26.53) |
| South-East Asia Region | 25 to 29 | IBD | Both | 47.49 (43.49, 51.85) |
| South-East Asia Region | 25 to 29 | IBD | Female | 50.70 (46.22, 55.63) |
| South-East Asia Region | 25 to 29 | IBD | Male | 44.76 (40.60, 49.34) |
| South-East Asia Region | 30 to 34 | IBD | Both | 76.97 (70.48, 84.06) |

| | | | | |
|---|---|---|---|---|
| South-East Asia Region | 30 to 34 | IBD | Female | 82.26 (74.97, 90.27) |
| South-East Asia Region | 30 to 34 | IBD | Male | 72.46 (65.71, 79.89) |
| South-East Asia Region | 35 to 39 | IBD | Both | 111.47 (102.05, 121.76) |
| South-East Asia Region | 35 to 39 | IBD | Female | 120.16 (109.49, 131.88) |
| South-East Asia Region | 35 to 39 | IBD | Male | 104.06 (94.35, 114.77) |
| South-East Asia Region | 40 to 44 | IBD | Both | 145.84 (133.49, 159.34) |
| South-East Asia Region | 40 to 44 | IBD | Female | 158.82 (144.68, 174.34) |
| South-East Asia Region | 40 to 44 | IBD | Male | 134.84 (122.23, 148.74) |
| South-East Asia Region | 45 to 49 | IBD | Both | 170.26 (155.80, 186.06) |
| South-East Asia Region | 45 to 49 | IBD | Female | 181.02 (164.86, 198.76) |
| South-East Asia Region | 45 to 49 | IBD | Male | 161.04 (145.95, 177.70) |
| South-East Asia Region | 5 to 9 | IBD | Both | 1.00 (1.00, 1.00) |
| South-East Asia Region | 5 to 9 | IBD | Female | 1.00 (1.00, 1.00) |
| South-East Asia Region | 5 to 9 | IBD | Male | 1.00 (1.00, 1.00) |
| South-East Asia Region | 50 to 54 | IBD | Both | 174.23 (159.39, 190.45) |
| South-East Asia Region | 50 to 54 | IBD | Female | 170.05 (154.82, 186.78) |
| South-East Asia Region | 50 to 54 | IBD | Male | 177.32 (160.66, 195.72) |
| South-East Asia Region | 55 to 59 | IBD | Both | 180.95 (165.49, 197.86) |
| South-East Asia Region | 55 to 59 | IBD | Female | 181.16 (164.89, 199.05) |
| South-East Asia Region | 55 to 59 | IBD | Male | 180.91 (163.85, 199.76) |
| South-East Asia Region | 60 to 64 | IBD | Both | 175.90 (160.81, 192.41) |
| South-East Asia Region | 60 to 64 | IBD | Female | 180.21 (163.96, 198.08) |
| South-East Asia Region | 60 to 64 | IBD | Male | 172.91 (156.53, 191.00) |
| South-East Asia Region | 65 to 69 | IBD | Both | 153.71 (140.42, 168.26) |
| South-East Asia Region | 65 to 69 | IBD | Female | 157.58 (143.26, 173.32) |
| South-East Asia Region | 65 to 69 | IBD | Male | 151.37 (136.91, 167.35) |
| South-East Asia Region | 10 to 14 | MS | Both | 7.95 (7.25, 8.71) |
| South-East Asia Region | 10 to 14 | MS | Female | 8.10 (7.18, 9.14) |
| South-East Asia Region | 10 to 14 | MS | Male | 7.72 (6.70, 8.88) |
| South-East Asia Region | 15 to 19 | MS | Both | 26.13 (23.87, 28.59) |
| South-East Asia Region | 15 to 19 | MS | Female | 27.30 (24.25, 30.74) |
| South-East Asia Region | 15 to 19 | MS | Male | 24.32 (21.18, 27.93) |
| South-East Asia Region | 20 to 24 | MS | Both | 58.40 (53.39, 63.89) |

| | | | | |
|---|---|---|---|---|
| South-East Asia Region | 20 to 24 | MS | Female | 61.83 (54.94, 69.59) |
| South-East Asia Region | 20 to 24 | MS | Male | 52.51 (45.75, 60.28) |
| South-East Asia Region | 25 to 29 | MS | Both | 105.98 (96.87, 115.94) |
| South-East Asia Region | 25 to 29 | MS | Female | 112.44 (99.90, 126.55) |
| South-East Asia Region | 25 to 29 | MS | Male | 94.37 (82.21, 108.33) |
| South-East Asia Region | 30 to 34 | MS | Both | 157.43 (143.88, 172.25) |
| South-East Asia Region | 30 to 34 | MS | Female | 166.23 (147.67, 187.12) |
| South-East Asia Region | 30 to 34 | MS | Male | 141.76 (123.46, 162.77) |
| South-East Asia Region | 35 to 39 | MS | Both | 205.90 (188.16, 225.32) |
| South-East Asia Region | 35 to 39 | MS | Female | 215.77 (191.64, 242.93) |
| South-East Asia Region | 35 to 39 | MS | Male | 188.64 (164.25, 216.65) |
| South-East Asia Region | 40 to 44 | MS | Both | 245.39 (224.21, 268.57) |
| South-East Asia Region | 40 to 44 | MS | Female | 255.34 (226.75, 287.53) |
| South-East Asia Region | 40 to 44 | MS | Male | 228.47 (198.89, 262.45) |
| South-East Asia Region | 45 to 49 | MS | Both | 272.71 (249.13, 298.52) |
| South-East Asia Region | 45 to 49 | MS | Female | 282.61 (250.92, 318.31) |
| South-East Asia Region | 45 to 49 | MS | Male | 255.13 (222.04, 293.14) |
| South-East Asia Region | 5 to 9 | MS | Both | 1.00 (1.00, 1.00) |
| South-East Asia Region | 5 to 9 | MS | Female | 1.00 (1.00, 1.00) |
| South-East Asia Region | 5 to 9 | MS | Male | 1.00 (1.00, 1.00) |
| South-East Asia Region | 50 to 54 | MS | Both | 284.89 (260.20, 311.91) |
| South-East Asia Region | 50 to 54 | MS | Female | 295.02 (261.87, 332.36) |
| South-East Asia Region | 50 to 54 | MS | Male | 264.77 (230.35, 304.32) |
| South-East Asia Region | 55 to 59 | MS | Both | 281.19 (256.76, 307.93) |
| South-East Asia Region | 55 to 59 | MS | Female | 292.70 (259.74, 329.85) |
| South-East Asia Region | 55 to 59 | MS | Male | 255.54 (222.23, 293.84) |
| South-East Asia Region | 60 to 64 | MS | Both | 264.62 (241.57, 289.88) |
| South-East Asia Region | 60 to 64 | MS | Female | 278.14 (246.73, 313.55) |
| South-East Asia Region | 60 to 64 | MS | Male | 232.66 (202.21, 267.68) |
| South-East Asia Region | 65 to 69 | MS | Both | 241.36 (220.21, 264.54) |
| South-East Asia Region | 65 to 69 | MS | Female | 256.77 (227.63, 289.64) |
| South-East Asia Region | 65 to 69 | MS | Male | 204.87 (177.88, 235.95) |
| South-East Asia Region | 0 to 4 | Psoriasis | Both | 0.40 (0.40, 0.40) |

| | | | | |
|---|---|---|---|---|
| South-East Asia Region | 0 to 4 | Psoriasis | Female | 0.40 (0.40, 0.40) |
| South-East Asia Region | 0 to 4 | Psoriasis | Male | 0.40 (0.40, 0.41) |
| South-East Asia Region | 10 to 14 | Psoriasis | Both | 1.32 (1.32, 1.33) |
| South-East Asia Region | 10 to 14 | Psoriasis | Female | 1.33 (1.32, 1.33) |
| South-East Asia Region | 10 to 14 | Psoriasis | Male | 1.32 (1.31, 1.33) |
| South-East Asia Region | 15 to 19 | Psoriasis | Both | 1.47 (1.46, 1.47) |
| South-East Asia Region | 15 to 19 | Psoriasis | Female | 1.47 (1.47, 1.48) |
| South-East Asia Region | 15 to 19 | Psoriasis | Male | 1.46 (1.45, 1.47) |
| South-East Asia Region | 20 to 24 | Psoriasis | Both | 1.60 (1.59, 1.61) |
| South-East Asia Region | 20 to 24 | Psoriasis | Female | 1.60 (1.59, 1.61) |
| South-East Asia Region | 20 to 24 | Psoriasis | Male | 1.60 (1.59, 1.61) |
| South-East Asia Region | 25 to 29 | Psoriasis | Both | 1.83 (1.82, 1.84) |
| South-East Asia Region | 25 to 29 | Psoriasis | Female | 1.82 (1.81, 1.83) |
| South-East Asia Region | 25 to 29 | Psoriasis | Male | 1.83 (1.82, 1.85) |
| South-East Asia Region | 30 to 34 | Psoriasis | Both | 2.11 (2.09, 2.12) |
| South-East Asia Region | 30 to 34 | Psoriasis | Female | 2.08 (2.07, 2.09) |
| South-East Asia Region | 30 to 34 | Psoriasis | Male | 2.13 (2.12, 2.14) |
| South-East Asia Region | 35 to 39 | Psoriasis | Both | 2.37 (2.36, 2.39) |
| South-East Asia Region | 35 to 39 | Psoriasis | Female | 2.33 (2.31, 2.34) |
| South-East Asia Region | 35 to 39 | Psoriasis | Male | 2.42 (2.41, 2.44) |
| South-East Asia Region | 40 to 44 | Psoriasis | Both | 2.62 (2.60, 2.64) |
| South-East Asia Region | 40 to 44 | Psoriasis | Female | 2.54 (2.53, 2.55) |
| South-East Asia Region | 40 to 44 | Psoriasis | Male | 2.70 (2.68, 2.72) |
| South-East Asia Region | 45 to 49 | Psoriasis | Both | 2.82 (2.80, 2.84) |
| South-East Asia Region | 45 to 49 | Psoriasis | Female | 2.71 (2.70, 2.73) |
| South-East Asia Region | 45 to 49 | Psoriasis | Male | 2.93 (2.91, 2.95) |
| South-East Asia Region | 5 to 9 | Psoriasis | Both | 1.00 (1.00, 1.00) |
| South-East Asia Region | 5 to 9 | Psoriasis | Female | 1.00 (1.00, 1.00) |
| South-East Asia Region | 5 to 9 | Psoriasis | Male | 1.00 (1.00, 1.00) |
| South-East Asia Region | 50 to 54 | Psoriasis | Both | 3.00 (2.98, 3.02) |
| South-East Asia Region | 50 to 54 | Psoriasis | Female | 2.86 (2.85, 2.88) |
| South-East Asia Region | 50 to 54 | Psoriasis | Male | 3.14 (3.11, 3.16) |
| South-East Asia Region | 55 to 59 | Psoriasis | Both | 3.16 (3.14, 3.18) |

| | | | | |
|---|---|---|---|---|
| South-East Asia Region | 55 to 59 | Psoriasis | Female | 3.02 (3.00, 3.04) |
| South-East Asia Region | 55 to 59 | Psoriasis | Male | 3.30 (3.28, 3.33) |
| South-East Asia Region | 60 to 64 | Psoriasis | Both | 3.25 (3.23, 3.28) |
| South-East Asia Region | 60 to 64 | Psoriasis | Female | 3.14 (3.11, 3.16) |
| South-East Asia Region | 60 to 64 | Psoriasis | Male | 3.37 (3.34, 3.40) |
| South-East Asia Region | 65 to 69 | Psoriasis | Both | 3.20 (3.18, 3.23) |
| South-East Asia Region | 65 to 69 | Psoriasis | Female | 3.15 (3.12, 3.17) |
| South-East Asia Region | 65 to 69 | Psoriasis | Male | 3.26 (3.23, 3.29) |
| South-East Asia Region | 0 to 4 | RHD | Both | 0.16 (0.13, 0.19) |
| South-East Asia Region | 0 to 4 | RHD | Female | 0.16 (0.13, 0.20) |
| South-East Asia Region | 0 to 4 | RHD | Male | 0.16 (0.13, 0.19) |
| South-East Asia Region | 10 to 14 | RHD | Both | 2.34 (2.14, 2.55) |
| South-East Asia Region | 10 to 14 | RHD | Female | 2.35 (2.15, 2.56) |
| South-East Asia Region | 10 to 14 | RHD | Male | 2.32 (2.13, 2.54) |
| South-East Asia Region | 15 to 19 | RHD | Both | 4.35 (3.99, 4.75) |
| South-East Asia Region | 15 to 19 | RHD | Female | 4.46 (4.09, 4.87) |
| South-East Asia Region | 15 to 19 | RHD | Male | 4.23 (3.87, 4.61) |
| South-East Asia Region | 20 to 24 | RHD | Both | 6.73 (6.16, 7.34) |
| South-East Asia Region | 20 to 24 | RHD | Female | 7.06 (6.47, 7.71) |
| South-East Asia Region | 20 to 24 | RHD | Male | 6.30 (5.77, 6.88) |
| South-East Asia Region | 25 to 29 | RHD | Both | 8.16 (7.46, 8.92) |
| South-East Asia Region | 25 to 29 | RHD | Female | 8.63 (7.89, 9.44) |
| South-East Asia Region | 25 to 29 | RHD | Male | 7.54 (6.89, 8.25) |
| South-East Asia Region | 30 to 34 | RHD | Both | 8.56 (7.81, 9.38) |
| South-East Asia Region | 30 to 34 | RHD | Female | 9.05 (8.26, 9.92) |
| South-East Asia Region | 30 to 34 | RHD | Male | 7.93 (7.23, 8.69) |
| South-East Asia Region | 35 to 39 | RHD | Both | 8.21 (7.47, 9.02) |
| South-East Asia Region | 35 to 39 | RHD | Female | 8.62 (7.85, 9.48) |
| South-East Asia Region | 35 to 39 | RHD | Male | 7.69 (6.98, 8.46) |
| South-East Asia Region | 40 to 44 | RHD | Both | 7.33 (6.65, 8.09) |
| South-East Asia Region | 40 to 44 | RHD | Female | 7.62 (6.91, 8.41) |
| South-East Asia Region | 40 to 44 | RHD | Male | 6.99 (6.33, 7.72) |
| South-East Asia Region | 45 to 49 | RHD | Both | 6.22 (5.61, 6.90) |

| | | | | |
|---|---|---|---|---|
| South-East Asia Region | 45 to 49 | RHD | Female | 6.44 (5.81, 7.14) |
| South-East Asia Region | 45 to 49 | RHD | Male | 5.97 (5.37, 6.63) |
| South-East Asia Region | 5 to 9 | RHD | Both | 1.00 (1.00, 1.00) |
| South-East Asia Region | 5 to 9 | RHD | Female | 1.00 (1.00, 1.00) |
| South-East Asia Region | 5 to 9 | RHD | Male | 1.00 (1.00, 1.00) |
| South-East Asia Region | 50 to 54 | RHD | Both | 5.02 (4.49, 5.61) |
| South-East Asia Region | 50 to 54 | RHD | Female | 5.21 (4.66, 5.82) |
| South-East Asia Region | 50 to 54 | RHD | Male | 4.77 (4.26, 5.35) |
| South-East Asia Region | 55 to 59 | RHD | Both | 4.07 (3.60, 4.60) |
| South-East Asia Region | 55 to 59 | RHD | Female | 4.22 (3.75, 4.76) |
| South-East Asia Region | 55 to 59 | RHD | Male | 3.83 (3.37, 4.34) |
| South-East Asia Region | 60 to 64 | RHD | Both | 3.34 (2.92, 3.83) |
| South-East Asia Region | 60 to 64 | RHD | Female | 3.45 (3.02, 3.94) |
| South-East Asia Region | 60 to 64 | RHD | Male | 3.14 (2.72, 3.61) |
| South-East Asia Region | 65 to 69 | RHD | Both | 2.75 (2.35, 3.22) |
| South-East Asia Region | 65 to 69 | RHD | Female | 2.81 (2.41, 3.28) |
| South-East Asia Region | 65 to 69 | RHD | Male | 2.60 (2.20, 3.07) |
| South-East Asia Region | 10 to 14 | RA | Both | 7.57 (7.21, 7.96) |
| South-East Asia Region | 10 to 14 | RA | Female | 8.03 (7.74, 8.33) |
| South-East Asia Region | 10 to 14 | RA | Male | 7.01 (6.67, 7.37) |
| South-East Asia Region | 15 to 19 | RA | Both | 23.40 (22.29, 24.57) |
| South-East Asia Region | 15 to 19 | RA | Female | 26.48 (25.54, 27.45) |
| South-East Asia Region | 15 to 19 | RA | Male | 19.40 (18.48, 20.37) |
| South-East Asia Region | 20 to 24 | RA | Both | 48.39 (46.10, 50.79) |
| South-East Asia Region | 20 to 24 | RA | Female | 56.24 (54.25, 58.30) |
| South-East Asia Region | 20 to 24 | RA | Male | 37.43 (35.66, 39.30) |
| South-East Asia Region | 25 to 29 | RA | Both | 77.71 (74.03, 81.57) |
| South-East Asia Region | 25 to 29 | RA | Female | 91.72 (88.48, 95.09) |
| South-East Asia Region | 25 to 29 | RA | Male | 57.63 (54.89, 60.50) |
| South-East Asia Region | 30 to 34 | RA | Both | 111.11 (105.84, 116.64) |
| South-East Asia Region | 30 to 34 | RA | Female | 133.06 (128.35, 137.95) |
| South-East Asia Region | 30 to 34 | RA | Male | 80.10 (76.28, 84.10) |
| South-East Asia Region | 35 to 39 | RA | Both | 153.96 (146.65, 161.64) |

| | | | | |
|---|---|---|---|---|
| South-East Asia Region | 35 to 39 | RA | Female | 186.50 (179.88, 193.36) |
| South-East Asia Region | 35 to 39 | RA | Male | 109.03 (103.82, 114.49) |
| South-East Asia Region | 40 to 44 | RA | Both | 208.78 (198.84, 219.21) |
| South-East Asia Region | 40 to 44 | RA | Female | 254.84 (245.79, 264.23) |
| South-East Asia Region | 40 to 44 | RA | Male | 146.79 (139.77, 154.16) |
| South-East Asia Region | 45 to 49 | RA | Both | 281.97 (268.53, 296.08) |
| South-East Asia Region | 45 to 49 | RA | Female | 343.74 (331.51, 356.42) |
| South-East Asia Region | 45 to 49 | RA | Male | 198.20 (188.70, 208.18) |
| South-East Asia Region | 5 to 9 | RA | Both | 1.00 (1.00, 1.00) |
| South-East Asia Region | 5 to 9 | RA | Female | 1.00 (1.00, 1.00) |
| South-East Asia Region | 5 to 9 | RA | Male | 1.00 (1.00, 1.00) |
| South-East Asia Region | 50 to 54 | RA | Both | 381.47 (363.26, 400.60) |
| South-East Asia Region | 50 to 54 | RA | Female | 460.98 (444.56, 478.00) |
| South-East Asia Region | 50 to 54 | RA | Male | 268.82 (255.91, 282.38) |
| South-East Asia Region | 55 to 59 | RA | Both | 534.39 (508.85, 561.22) |
| South-East Asia Region | 55 to 59 | RA | Female | 637.38 (614.66, 660.95) |
| South-East Asia Region | 55 to 59 | RA | Male | 375.97 (357.88, 394.97) |
| South-East Asia Region | 60 to 64 | RA | Both | 760.32 (723.95, 798.52) |
| South-East Asia Region | 60 to 64 | RA | Female | 896.32 (864.33, 929.48) |
| South-East Asia Region | 60 to 64 | RA | Male | 531.17 (505.58, 558.06) |
| South-East Asia Region | 65 to 69 | RA | Both | 1034.83 (985.28, 1086.88) |
| South-East Asia Region | 65 to 69 | RA | Female | 1203.84 (1160.85, 1248.42) |
| South-East Asia Region | 65 to 69 | RA | Male | 728.27 (693.13, 765.19) |
| Western Pacific Region | 0 to 4 | AA | Both | 0.75 (0.74, 0.75) |
| Western Pacific Region | 0 to 4 | AA | Female | 0.76 (0.76, 0.76) |
| Western Pacific Region | 0 to 4 | AA | Male | 0.73 (0.72, 0.73) |
| Western Pacific Region | 10 to 14 | AA | Both | 1.29 (1.29, 1.29) |
| Western Pacific Region | 10 to 14 | AA | Female | 1.28 (1.28, 1.29) |
| Western Pacific Region | 10 to 14 | AA | Male | 1.30 (1.29, 1.31) |
| Western Pacific Region | 15 to 19 | AA | Both | 2.27 (2.26, 2.27) |
| Western Pacific Region | 15 to 19 | AA | Female | 2.30 (2.29, 2.31) |
| Western Pacific Region | 15 to 19 | AA | Male | 2.18 (2.17, 2.19) |
| Western Pacific Region | 20 to 24 | AA | Both | 3.62 (3.61, 3.63) |

| | | | | |
|---|---|---|---|---|
| Western Pacific Region | 20 to 24 | AA | Female | 3.62 (3.60, 3.63) |
| Western Pacific Region | 20 to 24 | AA | Male | 3.54 (3.52, 3.56) |
| Western Pacific Region | 25 to 29 | AA | Both | 4.99 (4.98, 5.01) |
| Western Pacific Region | 25 to 29 | AA | Female | 4.99 (4.97, 5.01) |
| Western Pacific Region | 25 to 29 | AA | Male | 4.88 (4.85, 4.90) |
| Western Pacific Region | 30 to 34 | AA | Both | 5.34 (5.33, 5.36) |
| Western Pacific Region | 30 to 34 | AA | Female | 5.39 (5.37, 5.41) |
| Western Pacific Region | 30 to 34 | AA | Male | 5.15 (5.13, 5.18) |
| Western Pacific Region | 35 to 39 | AA | Both | 4.98 (4.97, 5.00) |
| Western Pacific Region | 35 to 39 | AA | Female | 5.09 (5.07, 5.11) |
| Western Pacific Region | 35 to 39 | AA | Male | 4.69 (4.67, 4.72) |
| Western Pacific Region | 40 to 44 | AA | Both | 4.25 (4.24, 4.27) |
| Western Pacific Region | 40 to 44 | AA | Female | 4.35 (4.34, 4.37) |
| Western Pacific Region | 40 to 44 | AA | Male | 3.98 (3.96, 4.00) |
| Western Pacific Region | 45 to 49 | AA | Both | 3.67 (3.66, 3.69) |
| Western Pacific Region | 45 to 49 | AA | Female | 3.78 (3.77, 3.80) |
| Western Pacific Region | 45 to 49 | AA | Male | 3.38 (3.36, 3.40) |
| Western Pacific Region | 5 to 9 | AA | Both | 1.00 (1.00, 1.00) |
| Western Pacific Region | 5 to 9 | AA | Female | 1.00 (1.00, 1.00) |
| Western Pacific Region | 5 to 9 | AA | Male | 1.00 (1.00, 1.00) |
| Western Pacific Region | 50 to 54 | AA | Both | 3.28 (3.27, 3.30) |
| Western Pacific Region | 50 to 54 | AA | Female | 3.41 (3.40, 3.43) |
| Western Pacific Region | 50 to 54 | AA | Male | 2.95 (2.93, 2.97) |
| Western Pacific Region | 55 to 59 | AA | Both | 3.13 (3.12, 3.14) |
| Western Pacific Region | 55 to 59 | AA | Female | 3.28 (3.26, 3.29) |
| Western Pacific Region | 55 to 59 | AA | Male | 2.75 (2.74, 2.77) |
| Western Pacific Region | 60 to 64 | AA | Both | 3.28 (3.27, 3.30) |
| Western Pacific Region | 60 to 64 | AA | Female | 3.47 (3.46, 3.49) |
| Western Pacific Region | 60 to 64 | AA | Male | 2.78 (2.76, 2.80) |
| Western Pacific Region | 65 to 69 | AA | Both | 3.08 (3.07, 3.09) |
| Western Pacific Region | 65 to 69 | AA | Female | 3.25 (3.24, 3.27) |
| Western Pacific Region | 65 to 69 | AA | Male | 2.54 (2.52, 2.56) |
| Western Pacific Region | 0 to 4 | IBD | Both | 0.04 (0.01, 0.22) |

| | | | | |
|---|---|---|---|---|
| Western Pacific Region | 0 to 4 | IBD | Female | 0.04 (0.01, 0.19) |
| Western Pacific Region | 0 to 4 | IBD | Male | 0.04 (0.01, 0.27) |
| Western Pacific Region | 10 to 14 | IBD | Both | 5.36 (3.71, 7.74) |
| Western Pacific Region | 10 to 14 | IBD | Female | 5.15 (3.71, 7.15) |
| Western Pacific Region | 10 to 14 | IBD | Male | 5.52 (3.67, 8.32) |
| Western Pacific Region | 15 to 19 | IBD | Both | 21.87 (15.33, 31.20) |
| Western Pacific Region | 15 to 19 | IBD | Female | 22.80 (16.62, 31.27) |
| Western Pacific Region | 15 to 19 | IBD | Male | 21.16 (14.23, 31.45) |
| Western Pacific Region | 20 to 24 | IBD | Both | 60.39 (42.40, 86.01) |
| Western Pacific Region | 20 to 24 | IBD | Female | 59.15 (43.18, 81.02) |
| Western Pacific Region | 20 to 24 | IBD | Male | 61.66 (41.56, 91.46) |
| Western Pacific Region | 25 to 29 | IBD | Both | 121.60 (85.38, 173.19) |
| Western Pacific Region | 25 to 29 | IBD | Female | 116.09 (84.76, 159.01) |
| Western Pacific Region | 25 to 29 | IBD | Male | 126.74 (85.44, 188.02) |
| Western Pacific Region | 30 to 34 | IBD | Both | 194.99 (136.86, 277.81) |
| Western Pacific Region | 30 to 34 | IBD | Female | 185.14 (135.13, 253.66) |
| Western Pacific Region | 30 to 34 | IBD | Male | 204.03 (137.48, 302.79) |
| Western Pacific Region | 35 to 39 | IBD | Both | 277.54 (194.73, 395.56) |
| Western Pacific Region | 35 to 39 | IBD | Female | 275.87 (201.29, 378.07) |
| Western Pacific Region | 35 to 39 | IBD | Male | 279.81 (188.46, 415.44) |
| Western Pacific Region | 40 to 44 | IBD | Both | 337.36 (236.62, 480.99) |
| Western Pacific Region | 40 to 44 | IBD | Female | 322.65 (235.36, 442.31) |
| Western Pacific Region | 40 to 44 | IBD | Male | 351.18 (236.44, 521.60) |
| Western Pacific Region | 45 to 49 | IBD | Both | 394.77 (276.78, 563.04) |
| Western Pacific Region | 45 to 49 | IBD | Female | 373.95 (272.69, 512.82) |
| Western Pacific Region | 45 to 49 | IBD | Male | 414.35 (278.85, 615.67) |
| Western Pacific Region | 5 to 9 | IBD | Both | 1.00 (1.00, 1.00) |
| Western Pacific Region | 5 to 9 | IBD | Female | 1.00 (1.00, 1.00) |
| Western Pacific Region | 5 to 9 | IBD | Male | 1.00 (1.00, 1.00) |
| Western Pacific Region | 50 to 54 | IBD | Both | 442.34 (310.01, 631.16) |
| Western Pacific Region | 50 to 54 | IBD | Female | 425.01 (309.82, 583.04) |
| Western Pacific Region | 50 to 54 | IBD | Male | 459.75 (309.26, 683.47) |
| Western Pacific Region | 55 to 59 | IBD | Both | 469.71 (329.03, 670.52) |

| | | | | |
|---|---|---|---|---|
| Western Pacific Region | 55 to 59 | IBD | Female | 457.77 (333.56, 628.24) |
| Western Pacific Region | 55 to 59 | IBD | Male | 483.51 (325.07, 719.18) |
| Western Pacific Region | 60 to 64 | IBD | Both | 476.64 (333.70, 680.79) |
| Western Pacific Region | 60 to 64 | IBD | Female | 466.13 (339.49, 640.03) |
| Western Pacific Region | 60 to 64 | IBD | Male | 490.39 (329.49, 729.86) |
| Western Pacific Region | 65 to 69 | IBD | Both | 460.62 (322.24, 658.44) |
| Western Pacific Region | 65 to 69 | IBD | Female | 452.82 (329.57, 622.17) |
| Western Pacific Region | 65 to 69 | IBD | Male | 474.08 (318.25, 706.22) |
| Western Pacific Region | 10 to 14 | MS | Both | 7.53 (6.61, 8.56) |
| Western Pacific Region | 10 to 14 | MS | Female | 7.61 (6.40, 9.05) |
| Western Pacific Region | 10 to 14 | MS | Male | 7.35 (6.07, 8.91) |
| Western Pacific Region | 15 to 19 | MS | Both | 22.88 (20.17, 25.96) |
| Western Pacific Region | 15 to 19 | MS | Female | 23.72 (20.02, 28.10) |
| Western Pacific Region | 15 to 19 | MS | Male | 21.45 (17.78, 25.88) |
| Western Pacific Region | 20 to 24 | MS | Both | 47.96 (42.30, 54.38) |
| Western Pacific Region | 20 to 24 | MS | Female | 50.93 (43.02, 60.31) |
| Western Pacific Region | 20 to 24 | MS | Male | 43.10 (35.75, 51.96) |
| Western Pacific Region | 25 to 29 | MS | Both | 83.88 (73.99, 95.10) |
| Western Pacific Region | 25 to 29 | MS | Female | 91.03 (76.88, 107.78) |
| Western Pacific Region | 25 to 29 | MS | Male | 72.87 (60.45, 87.84) |
| Western Pacific Region | 30 to 34 | MS | Both | 123.83 (109.21, 140.40) |
| Western Pacific Region | 30 to 34 | MS | Female | 135.02 (114.02, 159.88) |
| Western Pacific Region | 30 to 34 | MS | Male | 106.91 (88.68, 128.90) |
| Western Pacific Region | 35 to 39 | MS | Both | 162.29 (143.12, 184.04) |
| Western Pacific Region | 35 to 39 | MS | Female | 175.40 (148.11, 207.73) |
| Western Pacific Region | 35 to 39 | MS | Male | 142.16 (117.88, 171.43) |
| Western Pacific Region | 40 to 44 | MS | Both | 194.61 (171.61, 220.71) |
| Western Pacific Region | 40 to 44 | MS | Female | 207.86 (175.49, 246.20) |
| Western Pacific Region | 40 to 44 | MS | Male | 173.26 (143.65, 208.96) |
| Western Pacific Region | 45 to 49 | MS | Both | 216.38 (190.78, 245.42) |
| Western Pacific Region | 45 to 49 | MS | Female | 229.35 (193.61, 271.70) |
| Western Pacific Region | 45 to 49 | MS | Male | 194.21 (160.99, 234.28) |
| Western Pacific Region | 5 to 9 | MS | Both | 1.00 (1.00, 1.00) |

| | | | | |
|---|---|---|---|---|
| Western Pacific Region | 5 to 9 | MS | Female | 1.00 (1.00, 1.00) |
| Western Pacific Region | 5 to 9 | MS | Male | 1.00 (1.00, 1.00) |
| Western Pacific Region | 50 to 54 | MS | Both | 227.41 (200.47, 257.96) |
| Western Pacific Region | 50 to 54 | MS | Female | 240.61 (203.07, 285.08) |
| Western Pacific Region | 50 to 54 | MS | Male | 203.76 (168.87, 245.87) |
| Western Pacific Region | 55 to 59 | MS | Both | 227.45 (200.47, 258.06) |
| Western Pacific Region | 55 to 59 | MS | Female | 242.36 (204.50, 287.22) |
| Western Pacific Region | 55 to 59 | MS | Male | 200.54 (166.14, 242.06) |
| Western Pacific Region | 60 to 64 | MS | Both | 221.69 (195.34, 251.58) |
| Western Pacific Region | 60 to 64 | MS | Female | 238.90 (201.53, 283.20) |
| Western Pacific Region | 60 to 64 | MS | Male | 190.54 (157.79, 230.09) |
| Western Pacific Region | 65 to 69 | MS | Both | 223.23 (196.64, 253.41) |
| Western Pacific Region | 65 to 69 | MS | Female | 241.83 (203.93, 286.78) |
| Western Pacific Region | 65 to 69 | MS | Male | 187.31 (155.02, 226.32) |
| Western Pacific Region | 0 to 4 | Psoriasis | Both | 0.47 (0.47, 0.48) |
| Western Pacific Region | 0 to 4 | Psoriasis | Female | 0.44 (0.43, 0.44) |
| Western Pacific Region | 0 to 4 | Psoriasis | Male | 0.51 (0.50, 0.52) |
| Western Pacific Region | 10 to 14 | Psoriasis | Both | 1.28 (1.27, 1.28) |
| Western Pacific Region | 10 to 14 | Psoriasis | Female | 1.36 (1.35, 1.37) |
| Western Pacific Region | 10 to 14 | Psoriasis | Male | 1.18 (1.16, 1.19) |
| Western Pacific Region | 15 to 19 | Psoriasis | Both | 1.51 (1.51, 1.52) |
| Western Pacific Region | 15 to 19 | Psoriasis | Female | 1.63 (1.62, 1.64) |
| Western Pacific Region | 15 to 19 | Psoriasis | Male | 1.37 (1.36, 1.38) |
| Western Pacific Region | 20 to 24 | Psoriasis | Both | 1.68 (1.67, 1.69) |
| Western Pacific Region | 20 to 24 | Psoriasis | Female | 1.74 (1.73, 1.75) |
| Western Pacific Region | 20 to 24 | Psoriasis | Male | 1.59 (1.57, 1.60) |
| Western Pacific Region | 25 to 29 | Psoriasis | Both | 1.82 (1.81, 1.83) |
| Western Pacific Region | 25 to 29 | Psoriasis | Female | 1.81 (1.80, 1.82) |
| Western Pacific Region | 25 to 29 | Psoriasis | Male | 1.82 (1.81, 1.84) |
| Western Pacific Region | 30 to 34 | Psoriasis | Both | 2.08 (2.06, 2.09) |
| Western Pacific Region | 30 to 34 | Psoriasis | Female | 1.94 (1.93, 1.95) |
| Western Pacific Region | 30 to 34 | Psoriasis | Male | 2.22 (2.20, 2.25) |
| Western Pacific Region | 35 to 39 | Psoriasis | Both | 2.50 (2.48, 2.51) |

| | | | | |
|---|---|---|---|---|
| Western Pacific Region | 35 to 39 | Psoriasis | Female | 2.18 (2.16, 2.19) |
| Western Pacific Region | 35 to 39 | Psoriasis | Male | 2.87 (2.84, 2.90) |
| Western Pacific Region | 40 to 44 | Psoriasis | Both | 2.99 (2.97, 3.01) |
| Western Pacific Region | 40 to 44 | Psoriasis | Female | 2.42 (2.41, 2.43) |
| Western Pacific Region | 40 to 44 | Psoriasis | Male | 3.66 (3.62, 3.69) |
| Western Pacific Region | 45 to 49 | Psoriasis | Both | 3.24 (3.22, 3.26) |
| Western Pacific Region | 45 to 49 | Psoriasis | Female | 2.44 (2.43, 2.46) |
| Western Pacific Region | 45 to 49 | Psoriasis | Male | 4.18 (4.14, 4.22) |
| Western Pacific Region | 5 to 9 | Psoriasis | Both | 1.00 (1.00, 1.00) |
| Western Pacific Region | 5 to 9 | Psoriasis | Female | 1.00 (1.00, 1.00) |
| Western Pacific Region | 5 to 9 | Psoriasis | Male | 1.00 (1.00, 1.00) |
| Western Pacific Region | 50 to 54 | Psoriasis | Both | 3.33 (3.31, 3.35) |
| Western Pacific Region | 50 to 54 | Psoriasis | Female | 2.37 (2.36, 2.39) |
| Western Pacific Region | 50 to 54 | Psoriasis | Male | 4.47 (4.42, 4.52) |
| Western Pacific Region | 55 to 59 | Psoriasis | Both | 3.39 (3.36, 3.41) |
| Western Pacific Region | 55 to 59 | Psoriasis | Female | 2.32 (2.30, 2.33) |
| Western Pacific Region | 55 to 59 | Psoriasis | Male | 4.67 (4.62, 4.72) |
| Western Pacific Region | 60 to 64 | Psoriasis | Both | 3.32 (3.30, 3.34) |
| Western Pacific Region | 60 to 64 | Psoriasis | Female | 2.37 (2.35, 2.38) |
| Western Pacific Region | 60 to 64 | Psoriasis | Male | 4.48 (4.44, 4.53) |
| Western Pacific Region | 65 to 69 | Psoriasis | Both | 3.26 (3.23, 3.28) |
| Western Pacific Region | 65 to 69 | Psoriasis | Female | 2.63 (2.61, 2.65) |
| Western Pacific Region | 65 to 69 | Psoriasis | Male | 4.04 (3.99, 4.08) |
| Western Pacific Region | 0 to 4 | RHD | Both | 0.18 (0.16, 0.20) |
| Western Pacific Region | 0 to 4 | RHD | Female | 0.18 (0.16, 0.20) |
| Western Pacific Region | 0 to 4 | RHD | Male | 0.18 (0.16, 0.20) |
| Western Pacific Region | 10 to 14 | RHD | Both | 1.94 (1.85, 2.03) |
| Western Pacific Region | 10 to 14 | RHD | Female | 1.94 (1.85, 2.04) |
| Western Pacific Region | 10 to 14 | RHD | Male | 1.94 (1.85, 2.02) |
| Western Pacific Region | 15 to 19 | RHD | Both | 2.63 (2.52, 2.75) |
| Western Pacific Region | 15 to 19 | RHD | Female | 2.65 (2.53, 2.78) |
| Western Pacific Region | 15 to 19 | RHD | Male | 2.61 (2.50, 2.72) |
| Western Pacific Region | 20 to 24 | RHD | Both | 2.89 (2.76, 3.02) |

| | | | | |
|---|---|---|---|---|
| Western Pacific Region | 20 to 24 | RHD | Female | 2.94 (2.80, 3.08) |
| Western Pacific Region | 20 to 24 | RHD | Male | 2.82 (2.70, 2.94) |
| Western Pacific Region | 25 to 29 | RHD | Both | 3.03 (2.89, 3.17) |
| Western Pacific Region | 25 to 29 | RHD | Female | 3.12 (2.97, 3.28) |
| Western Pacific Region | 25 to 29 | RHD | Male | 2.92 (2.79, 3.05) |
| Western Pacific Region | 30 to 34 | RHD | Both | 3.07 (2.93, 3.22) |
| Western Pacific Region | 30 to 34 | RHD | Female | 3.20 (3.04, 3.36) |
| Western Pacific Region | 30 to 34 | RHD | Male | 2.94 (2.81, 3.07) |
| Western Pacific Region | 35 to 39 | RHD | Both | 3.04 (2.90, 3.20) |
| Western Pacific Region | 35 to 39 | RHD | Female | 3.19 (3.03, 3.36) |
| Western Pacific Region | 35 to 39 | RHD | Male | 2.88 (2.75, 3.02) |
| Western Pacific Region | 40 to 44 | RHD | Both | 2.95 (2.81, 3.10) |
| Western Pacific Region | 40 to 44 | RHD | Female | 3.13 (2.97, 3.29) |
| Western Pacific Region | 40 to 44 | RHD | Male | 2.76 (2.63, 2.90) |
| Western Pacific Region | 45 to 49 | RHD | Both | 2.71 (2.58, 2.86) |
| Western Pacific Region | 45 to 49 | RHD | Female | 2.93 (2.77, 3.09) |
| Western Pacific Region | 45 to 49 | RHD | Male | 2.49 (2.37, 2.61) |
| Western Pacific Region | 5 to 9 | RHD | Both | 1.00 (1.00, 1.00) |
| Western Pacific Region | 5 to 9 | RHD | Female | 1.00 (1.00, 1.00) |
| Western Pacific Region | 5 to 9 | RHD | Male | 1.00 (1.00, 1.00) |
| Western Pacific Region | 50 to 54 | RHD | Both | 2.38 (2.25, 2.51) |
| Western Pacific Region | 50 to 54 | RHD | Female | 2.63 (2.49, 2.79) |
| Western Pacific Region | 50 to 54 | RHD | Male | 2.11 (2.00, 2.22) |
| Western Pacific Region | 55 to 59 | RHD | Both | 2.14 (2.02, 2.27) |
| Western Pacific Region | 55 to 59 | RHD | Female | 2.44 (2.30, 2.59) |
| Western Pacific Region | 55 to 59 | RHD | Male | 1.82 (1.72, 1.92) |
| Western Pacific Region | 60 to 64 | RHD | Both | 2.01 (1.89, 2.14) |
| Western Pacific Region | 60 to 64 | RHD | Female | 2.37 (2.22, 2.52) |
| Western Pacific Region | 60 to 64 | RHD | Male | 1.64 (1.54, 1.75) |
| Western Pacific Region | 65 to 69 | RHD | Both | 1.95 (1.82, 2.09) |
| Western Pacific Region | 65 to 69 | RHD | Female | 2.32 (2.17, 2.49) |
| Western Pacific Region | 65 to 69 | RHD | Male | 1.54 (1.44, 1.65) |
| Western Pacific Region | 10 to 14 | RA | Both | 6.75 (6.54, 6.97) |

| | | | | |
|---|---|---|---|---|
| Western Pacific Region | 10 to 14 | RA | Female | 7.05 (6.78, 7.33) |
| Western Pacific Region | 10 to 14 | RA | Male | 6.34 (6.16, 6.52) |
| Western Pacific Region | 15 to 19 | RA | Both | 17.50 (16.96, 18.05) |
| Western Pacific Region | 15 to 19 | RA | Female | 19.58 (18.84, 20.34) |
| Western Pacific Region | 15 to 19 | RA | Male | 14.50 (14.10, 14.91) |
| Western Pacific Region | 20 to 24 | RA | Both | 32.80 (31.80, 33.84) |
| Western Pacific Region | 20 to 24 | RA | Female | 38.25 (36.81, 39.74) |
| Western Pacific Region | 20 to 24 | RA | Male | 24.65 (23.97, 25.35) |
| Western Pacific Region | 25 to 29 | RA | Both | 51.21 (49.64, 52.82) |
| Western Pacific Region | 25 to 29 | RA | Female | 61.19 (58.90, 63.57) |
| Western Pacific Region | 25 to 29 | RA | Male | 36.39 (35.39, 37.42) |
| Western Pacific Region | 30 to 34 | RA | Both | 73.60 (71.35, 75.93) |
| Western Pacific Region | 30 to 34 | RA | Female | 89.26 (85.91, 92.74) |
| Western Pacific Region | 30 to 34 | RA | Male | 50.61 (49.22, 52.04) |
| Western Pacific Region | 35 to 39 | RA | Both | 104.91 (101.69, 108.24) |
| Western Pacific Region | 35 to 39 | RA | Female | 128.01 (123.20, 133.00) |
| Western Pacific Region | 35 to 39 | RA | Male | 71.05 (69.09, 73.06) |
| Western Pacific Region | 40 to 44 | RA | Both | 146.93 (142.41, 151.58) |
| Western Pacific Region | 40 to 44 | RA | Female | 179.16 (172.43, 186.15) |
| Western Pacific Region | 40 to 44 | RA | Male | 99.07 (96.34, 101.88) |
| Western Pacific Region | 45 to 49 | RA | Both | 202.33 (196.11, 208.75) |
| Western Pacific Region | 45 to 49 | RA | Female | 245.56 (236.34, 255.14) |
| Western Pacific Region | 45 to 49 | RA | Male | 136.80 (133.02, 140.69) |
| Western Pacific Region | 5 to 9 | RA | Both | 1.00 (1.00, 1.00) |
| Western Pacific Region | 5 to 9 | RA | Female | 1.00 (1.00, 1.00) |
| Western Pacific Region | 5 to 9 | RA | Male | 1.00 (1.00, 1.00) |
| Western Pacific Region | 50 to 54 | RA | Both | 271.35 (263.00, 279.97) |
| Western Pacific Region | 50 to 54 | RA | Female | 326.85 (314.56, 339.61) |
| Western Pacific Region | 50 to 54 | RA | Male | 184.69 (179.59, 189.95) |
| Western Pacific Region | 55 to 59 | RA | Both | 353.34 (342.45, 364.57) |
| Western Pacific Region | 55 to 59 | RA | Female | 421.25 (405.40, 437.72) |
| Western Pacific Region | 55 to 59 | RA | Male | 243.49 (236.74, 250.42) |
| Western Pacific Region | 60 to 64 | RA | Both | 444.60 (430.89, 458.74) |

| | | | | |
|---|---|---|---|---|
| Western Pacific Region | 60 to 64 | RA | Female | 522.18 (502.52, 542.60) |
| Western Pacific Region | 60 to 64 | RA | Male | 312.47 (303.80, 321.38) |
| Western Pacific Region | 65 to 69 | RA | Both | 524.03 (507.85, 540.72) |
| Western Pacific Region | 65 to 69 | RA | Female | 597.87 (575.34, 621.27) |
| Western Pacific Region | 65 to 69 | RA | Male | 383.20 (372.56, 394.15) |
| Afghanistan | 0 to 4 | AA | Both | 0.70 (0.68, 0.71) |
| Afghanistan | 0 to 4 | AA | Female | 0.72 (0.70, 0.75) |
| Afghanistan | 0 to 4 | AA | Male | 0.66 (0.63, 0.68) |
| Afghanistan | 10 to 14 | AA | Both | 1.28 (1.25, 1.31) |
| Afghanistan | 10 to 14 | AA | Female | 1.26 (1.22, 1.30) |
| Afghanistan | 10 to 14 | AA | Male | 1.31 (1.26, 1.36) |
| Afghanistan | 15 to 19 | AA | Both | 2.14 (2.09, 2.18) |
| Afghanistan | 15 to 19 | AA | Female | 2.20 (2.14, 2.26) |
| Afghanistan | 15 to 19 | AA | Male | 2.04 (1.97, 2.12) |
| Afghanistan | 20 to 24 | AA | Both | 3.34 (3.26, 3.42) |
| Afghanistan | 20 to 24 | AA | Female | 3.44 (3.34, 3.54) |
| Afghanistan | 20 to 24 | AA | Male | 3.20 (3.08, 3.32) |
| Afghanistan | 25 to 29 | AA | Both | 4.67 (4.56, 4.79) |
| Afghanistan | 25 to 29 | AA | Female | 4.81 (4.66, 4.95) |
| Afghanistan | 25 to 29 | AA | Male | 4.50 (4.32, 4.68) |
| Afghanistan | 30 to 34 | AA | Both | 4.94 (4.81, 5.07) |
| Afghanistan | 30 to 34 | AA | Female | 5.08 (4.92, 5.24) |
| Afghanistan | 30 to 34 | AA | Male | 4.75 (4.56, 4.96) |
| Afghanistan | 35 to 39 | AA | Both | 4.63 (4.51, 4.76) |
| Afghanistan | 35 to 39 | AA | Female | 4.77 (4.61, 4.94) |
| Afghanistan | 35 to 39 | AA | Male | 4.43 (4.23, 4.63) |
| Afghanistan | 40 to 44 | AA | Both | 3.94 (3.82, 4.05) |
| Afghanistan | 40 to 44 | AA | Female | 4.04 (3.90, 4.20) |
| Afghanistan | 40 to 44 | AA | Male | 3.79 (3.61, 3.98) |
| Afghanistan | 45 to 49 | AA | Both | 3.40 (3.29, 3.51) |
| Afghanistan | 45 to 49 | AA | Female | 3.50 (3.36, 3.65) |
| Afghanistan | 45 to 49 | AA | Male | 3.26 (3.08, 3.44) |
| Afghanistan | 5 to 9 | AA | Both | 1.00 (1.00, 1.00) |

| | | | | |
|---|---|---|---|---|
| Afghanistan | 5 to 9 | AA | Female | 1.00 (1.00, 1.00) |
| Afghanistan | 5 to 9 | AA | Male | 1.00 (1.00, 1.00) |
| Afghanistan | 50 to 54 | AA | Both | 3.03 (2.92, 3.15) |
| Afghanistan | 50 to 54 | AA | Female | 3.13 (2.99, 3.28) |
| Afghanistan | 50 to 54 | AA | Male | 2.91 (2.73, 3.11) |
| Afghanistan | 55 to 59 | AA | Both | 2.86 (2.74, 2.98) |
| Afghanistan | 55 to 59 | AA | Female | 2.95 (2.80, 3.11) |
| Afghanistan | 55 to 59 | AA | Male | 2.75 (2.55, 2.97) |
| Afghanistan | 60 to 64 | AA | Both | 2.97 (2.83, 3.12) |
| Afghanistan | 60 to 64 | AA | Female | 3.07 (2.90, 3.25) |
| Afghanistan | 60 to 64 | AA | Male | 2.86 (2.63, 3.11) |
| Afghanistan | 65 to 69 | AA | Both | 2.79 (2.64, 2.94) |
| Afghanistan | 65 to 69 | AA | Female | 2.87 (2.69, 3.07) |
| Afghanistan | 65 to 69 | AA | Male | 2.69 (2.45, 2.96) |
| Afghanistan | 0 to 4 | IBD | Both | 0.06 (0.03, 0.16) |
| Afghanistan | 0 to 4 | IBD | Female | 0.05 (0.01, 0.30) |
| Afghanistan | 0 to 4 | IBD | Male | 0.05 (0.01, 0.27) |
| Afghanistan | 10 to 14 | IBD | Both | 4.22 (3.24, 5.51) |
| Afghanistan | 10 to 14 | IBD | Female | 4.11 (2.82, 6.00) |
| Afghanistan | 10 to 14 | IBD | Male | 4.28 (2.97, 6.16) |
| Afghanistan | 15 to 19 | IBD | Both | 13.98 (10.79, 18.10) |
| Afghanistan | 15 to 19 | IBD | Female | 15.40 (10.68, 22.19) |
| Afghanistan | 15 to 19 | IBD | Male | 12.46 (8.71, 17.83) |
| Afghanistan | 20 to 24 | IBD | Both | 31.38 (24.22, 40.65) |
| Afghanistan | 20 to 24 | IBD | Female | 34.24 (23.73, 49.39) |
| Afghanistan | 20 to 24 | IBD | Male | 28.28 (19.76, 40.46) |
| Afghanistan | 25 to 29 | IBD | Both | 56.43 (43.50, 73.22) |
| Afghanistan | 25 to 29 | IBD | Female | 60.98 (42.18, 88.16) |
| Afghanistan | 25 to 29 | IBD | Male | 51.27 (35.76, 73.52) |
| Afghanistan | 30 to 34 | IBD | Both | 86.12 (66.28, 111.89) |
| Afghanistan | 30 to 34 | IBD | Female | 92.34 (63.75, 133.74) |
| Afghanistan | 30 to 34 | IBD | Male | 78.92 (54.92, 113.41) |
| Afghanistan | 35 to 39 | IBD | Both | 116.71 (89.71, 151.84) |

| | | | | |
|---|---|---|---|---|
| Afghanistan | 35 to 39 | IBD | Female | 124.83 (86.04, 181.11) |
| Afghanistan | 35 to 39 | IBD | Male | 107.02 (74.32, 154.10) |
| Afghanistan | 40 to 44 | IBD | Both | 144.30 (110.72, 188.05) |
| Afghanistan | 40 to 44 | IBD | Female | 154.30 (106.13, 224.34) |
| Afghanistan | 40 to 44 | IBD | Male | 132.12 (91.50, 190.79) |
| Afghanistan | 45 to 49 | IBD | Both | 167.17 (127.96, 218.39) |
| Afghanistan | 45 to 49 | IBD | Female | 179.71 (123.24, 262.04) |
| Afghanistan | 45 to 49 | IBD | Male | 152.45 (105.12, 221.08) |
| Afghanistan | 5 to 9 | IBD | Both | 1.00 (1.00, 1.00) |
| Afghanistan | 5 to 9 | IBD | Female | 1.00 (1.00, 1.00) |
| Afghanistan | 5 to 9 | IBD | Male | 1.00 (1.00, 1.00) |
| Afghanistan | 50 to 54 | IBD | Both | 160.03 (122.06, 209.81) |
| Afghanistan | 50 to 54 | IBD | Female | 153.66 (104.86, 225.18) |
| Afghanistan | 50 to 54 | IBD | Male | 166.32 (114.08, 242.48) |
| Afghanistan | 55 to 59 | IBD | Both | 162.46 (123.51, 213.69) |
| Afghanistan | 55 to 59 | IBD | Female | 154.12 (104.73, 226.81) |
| Afghanistan | 55 to 59 | IBD | Male | 172.50 (117.71, 252.79) |
| Afghanistan | 60 to 64 | IBD | Both | 157.93 (119.66, 208.42) |
| Afghanistan | 60 to 64 | IBD | Female | 148.96 (100.73, 220.29) |
| Afghanistan | 60 to 64 | IBD | Male | 169.22 (114.94, 249.11) |
| Afghanistan | 65 to 69 | IBD | Both | 144.91 (109.18, 192.32) |
| Afghanistan | 65 to 69 | IBD | Female | 135.33 (90.72, 201.88) |
| Afghanistan | 65 to 69 | IBD | Male | 156.08 (105.24, 231.49) |
| Afghanistan | 10 to 14 | MS | Both | 10.18 (6.82, 15.22) |
| Afghanistan | 10 to 14 | MS | Female | 10.77 (6.46, 17.94) |
| Afghanistan | 10 to 14 | MS | Male | 9.21 (4.81, 17.66) |
| Afghanistan | 15 to 19 | MS | Both | 44.80 (30.16, 66.54) |
| Afghanistan | 15 to 19 | MS | Female | 49.89 (30.19, 82.45) |
| Afghanistan | 15 to 19 | MS | Male | 36.96 (19.44, 70.29) |
| Afghanistan | 20 to 24 | MS | Both | 127.47 (85.86, 189.26) |
| Afghanistan | 20 to 24 | MS | Female | 146.52 (88.70, 242.03) |
| Afghanistan | 20 to 24 | MS | Male | 98.98 (52.11, 188.01) |
| Afghanistan | 25 to 29 | MS | Both | 263.22 (177.17, 391.06) |

| | | | | |
|---|---|---|---|---|
| Afghanistan | 25 to 29 | MS | Female | 302.43 (182.98, 499.84) |
| Afghanistan | 25 to 29 | MS | Male | 204.46 (107.53, 388.74) |
| Afghanistan | 30 to 34 | MS | Both | 401.80 (270.30, 597.29) |
| Afghanistan | 30 to 34 | MS | Female | 452.32 (273.47, 748.12) |
| Afghanistan | 30 to 34 | MS | Male | 326.57 (171.55, 621.68) |
| Afghanistan | 35 to 39 | MS | Both | 517.12 (347.67, 769.15) |
| Afghanistan | 35 to 39 | MS | Female | 566.64 (342.36, 937.84) |
| Afghanistan | 35 to 39 | MS | Male | 443.51 (232.73, 845.21) |
| Afghanistan | 40 to 44 | MS | Both | 597.96 (401.73, 890.06) |
| Afghanistan | 40 to 44 | MS | Female | 641.20 (387.09, 1062.10) |
| Afghanistan | 40 to 44 | MS | Male | 536.86 (281.29, 1024.65) |
| Afghanistan | 45 to 49 | MS | Both | 651.91 (437.51, 971.39) |
| Afghanistan | 45 to 49 | MS | Female | 690.85 (416.59, 1145.67) |
| Afghanistan | 45 to 49 | MS | Male | 601.71 (314.54, 1151.05) |
| Afghanistan | 5 to 9 | MS | Both | 1.00 (1.00, 1.00) |
| Afghanistan | 5 to 9 | MS | Female | 1.00 (1.00, 1.00) |
| Afghanistan | 5 to 9 | MS | Male | 1.00 (1.00, 1.00) |
| Afghanistan | 50 to 54 | MS | Both | 681.07 (456.48, 1016.15) |
| Afghanistan | 50 to 54 | MS | Female | 718.73 (432.78, 1193.60) |
| Afghanistan | 50 to 54 | MS | Male | 639.67 (333.44, 1227.15) |
| Afghanistan | 55 to 59 | MS | Both | 690.11 (461.92, 1031.02) |
| Afghanistan | 55 to 59 | MS | Female | 729.04 (438.34, 1212.52) |
| Afghanistan | 55 to 59 | MS | Male | 651.93 (338.86, 1254.25) |
| Afghanistan | 60 to 64 | MS | Both | 685.68 (458.35, 1025.77) |
| Afghanistan | 60 to 64 | MS | Female | 726.32 (436.03, 1209.87) |
| Afghanistan | 60 to 64 | MS | Male | 641.95 (332.83, 1238.17) |
| Afghanistan | 65 to 69 | MS | Both | 678.78 (452.83, 1017.46) |
| Afghanistan | 65 to 69 | MS | Female | 722.46 (432.70, 1206.25) |
| Afghanistan | 65 to 69 | MS | Male | 628.97 (324.92, 1217.56) |
| Afghanistan | 0 to 4 | Psoriasis | Both | 0.45 (0.44, 0.46) |
| Afghanistan | 0 to 4 | Psoriasis | Female | 0.45 (0.44, 0.46) |
| Afghanistan | 0 to 4 | Psoriasis | Male | 0.45 (0.44, 0.46) |
| Afghanistan | 10 to 14 | Psoriasis | Both | 1.17 (1.15, 1.18) |

| | | | | |
|---|---|---|---|---|
| Afghanistan | 10 to 14 | Psoriasis | Female | 1.19 (1.17, 1.20) |
| Afghanistan | 10 to 14 | Psoriasis | Male | 1.14 (1.12, 1.16) |
| Afghanistan | 15 to 19 | Psoriasis | Both | 1.33 (1.31, 1.34) |
| Afghanistan | 15 to 19 | Psoriasis | Female | 1.37 (1.35, 1.40) |
| Afghanistan | 15 to 19 | Psoriasis | Male | 1.27 (1.25, 1.30) |
| Afghanistan | 20 to 24 | Psoriasis | Both | 1.56 (1.54, 1.58) |
| Afghanistan | 20 to 24 | Psoriasis | Female | 1.60 (1.57, 1.63) |
| Afghanistan | 20 to 24 | Psoriasis | Male | 1.52 (1.49, 1.55) |
| Afghanistan | 25 to 29 | Psoriasis | Both | 1.86 (1.83, 1.88) |
| Afghanistan | 25 to 29 | Psoriasis | Female | 1.80 (1.77, 1.84) |
| Afghanistan | 25 to 29 | Psoriasis | Male | 1.92 (1.88, 1.96) |
| Afghanistan | 30 to 34 | Psoriasis | Both | 2.12 (2.09, 2.15) |
| Afghanistan | 30 to 34 | Psoriasis | Female | 1.98 (1.94, 2.02) |
| Afghanistan | 30 to 34 | Psoriasis | Male | 2.29 (2.24, 2.34) |
| Afghanistan | 35 to 39 | Psoriasis | Both | 2.29 (2.25, 2.32) |
| Afghanistan | 35 to 39 | Psoriasis | Female | 2.11 (2.06, 2.15) |
| Afghanistan | 35 to 39 | Psoriasis | Male | 2.49 (2.44, 2.55) |
| Afghanistan | 40 to 44 | Psoriasis | Both | 2.40 (2.36, 2.43) |
| Afghanistan | 40 to 44 | Psoriasis | Female | 2.21 (2.16, 2.26) |
| Afghanistan | 40 to 44 | Psoriasis | Male | 2.61 (2.55, 2.67) |
| Afghanistan | 45 to 49 | Psoriasis | Both | 2.50 (2.45, 2.54) |
| Afghanistan | 45 to 49 | Psoriasis | Female | 2.31 (2.25, 2.37) |
| Afghanistan | 45 to 49 | Psoriasis | Male | 2.71 (2.64, 2.78) |
| Afghanistan | 5 to 9 | Psoriasis | Both | 1.00 (1.00, 1.00) |
| Afghanistan | 5 to 9 | Psoriasis | Female | 1.00 (1.00, 1.00) |
| Afghanistan | 5 to 9 | Psoriasis | Male | 1.00 (1.00, 1.00) |
| Afghanistan | 50 to 54 | Psoriasis | Both | 2.65 (2.60, 2.71) |
| Afghanistan | 50 to 54 | Psoriasis | Female | 2.45 (2.39, 2.52) |
| Afghanistan | 50 to 54 | Psoriasis | Male | 2.88 (2.80, 2.97) |
| Afghanistan | 55 to 59 | Psoriasis | Both | 2.82 (2.76, 2.88) |
| Afghanistan | 55 to 59 | Psoriasis | Female | 2.59 (2.52, 2.67) |
| Afghanistan | 55 to 59 | Psoriasis | Male | 3.09 (2.99, 3.19) |
| Afghanistan | 60 to 64 | Psoriasis | Both | 2.85 (2.79, 2.92) |

| | | | | |
|---|---|---|---|---|
| Afghanistan | 60 to 64 | Psoriasis | Female | 2.59 (2.51, 2.67) |
| Afghanistan | 60 to 64 | Psoriasis | Male | 3.17 (3.06, 3.28) |
| Afghanistan | 65 to 69 | Psoriasis | Both | 2.75 (2.68, 2.82) |
| Afghanistan | 65 to 69 | Psoriasis | Female | 2.46 (2.38, 2.55) |
| Afghanistan | 65 to 69 | Psoriasis | Male | 3.10 (2.99, 3.21) |
| Afghanistan | 0 to 4 | RHD | Both | 0.17 (0.17, 0.18) |
| Afghanistan | 0 to 4 | RHD | Female | 0.17 (0.17, 0.18) |
| Afghanistan | 0 to 4 | RHD | Male | 0.17 (0.17, 0.18) |
| Afghanistan | 10 to 14 | RHD | Both | 1.94 (1.92, 1.96) |
| Afghanistan | 10 to 14 | RHD | Female | 1.94 (1.90, 1.97) |
| Afghanistan | 10 to 14 | RHD | Male | 1.94 (1.91, 1.98) |
| Afghanistan | 15 to 19 | RHD | Both | 2.83 (2.79, 2.87) |
| Afghanistan | 15 to 19 | RHD | Female | 2.84 (2.79, 2.89) |
| Afghanistan | 15 to 19 | RHD | Male | 2.82 (2.77, 2.87) |
| Afghanistan | 20 to 24 | RHD | Both | 3.56 (3.51, 3.61) |
| Afghanistan | 20 to 24 | RHD | Female | 3.61 (3.54, 3.68) |
| Afghanistan | 20 to 24 | RHD | Male | 3.52 (3.45, 3.58) |
| Afghanistan | 25 to 29 | RHD | Both | 3.98 (3.92, 4.04) |
| Afghanistan | 25 to 29 | RHD | Female | 4.06 (3.98, 4.14) |
| Afghanistan | 25 to 29 | RHD | Male | 3.91 (3.83, 3.99) |
| Afghanistan | 30 to 34 | RHD | Both | 4.12 (4.06, 4.19) |
| Afghanistan | 30 to 34 | RHD | Female | 4.21 (4.12, 4.30) |
| Afghanistan | 30 to 34 | RHD | Male | 4.04 (3.96, 4.13) |
| Afghanistan | 35 to 39 | RHD | Both | 4.07 (4.00, 4.13) |
| Afghanistan | 35 to 39 | RHD | Female | 4.14 (4.05, 4.24) |
| Afghanistan | 35 to 39 | RHD | Male | 3.99 (3.90, 4.09) |
| Afghanistan | 40 to 44 | RHD | Both | 3.82 (3.76, 3.89) |
| Afghanistan | 40 to 44 | RHD | Female | 3.89 (3.79, 3.98) |
| Afghanistan | 40 to 44 | RHD | Male | 3.76 (3.67, 3.86) |
| Afghanistan | 45 to 49 | RHD | Both | 3.32 (3.26, 3.39) |
| Afghanistan | 45 to 49 | RHD | Female | 3.39 (3.30, 3.48) |
| Afghanistan | 45 to 49 | RHD | Male | 3.26 (3.17, 3.35) |
| Afghanistan | 5 to 9 | RHD | Both | 1.00 (1.00, 1.00) |

| | | | | |
|---|---|---|---|---|
| Afghanistan | 5 to 9 | RHD | Female | 1.00 (1.00, 1.00) |
| Afghanistan | 5 to 9 | RHD | Male | 1.00 (1.00, 1.00) |
| Afghanistan | 50 to 54 | RHD | Both | 2.64 (2.58, 2.71) |
| Afghanistan | 50 to 54 | RHD | Female | 2.72 (2.63, 2.80) |
| Afghanistan | 50 to 54 | RHD | Male | 2.57 (2.48, 2.66) |
| Afghanistan | 55 to 59 | RHD | Both | 2.06 (2.00, 2.12) |
| Afghanistan | 55 to 59 | RHD | Female | 2.13 (2.05, 2.21) |
| Afghanistan | 55 to 59 | RHD | Male | 1.98 (1.90, 2.07) |
| Afghanistan | 60 to 64 | RHD | Both | 1.60 (1.55, 1.66) |
| Afghanistan | 60 to 64 | RHD | Female | 1.67 (1.59, 1.75) |
| Afghanistan | 60 to 64 | RHD | Male | 1.53 (1.45, 1.61) |
| Afghanistan | 65 to 69 | RHD | Both | 1.25 (1.19, 1.30) |
| Afghanistan | 65 to 69 | RHD | Female | 1.30 (1.23, 1.38) |
| Afghanistan | 65 to 69 | RHD | Male | 1.18 (1.11, 1.26) |
| Afghanistan | 10 to 14 | RA | Both | 7.21 (6.13, 8.49) |
| Afghanistan | 10 to 14 | RA | Female | 7.57 (6.21, 9.22) |
| Afghanistan | 10 to 14 | RA | Male | 6.87 (5.10, 9.26) |
| Afghanistan | 15 to 19 | RA | Both | 21.16 (18.01, 24.86) |
| Afghanistan | 15 to 19 | RA | Female | 23.23 (19.11, 28.23) |
| Afghanistan | 15 to 19 | RA | Male | 18.13 (13.48, 24.39) |
| Afghanistan | 20 to 24 | RA | Both | 42.27 (35.95, 49.69) |
| Afghanistan | 20 to 24 | RA | Female | 47.45 (39.02, 57.70) |
| Afghanistan | 20 to 24 | RA | Male | 34.16 (25.37, 46.01) |
| Afghanistan | 25 to 29 | RA | Both | 67.04 (56.97, 78.88) |
| Afghanistan | 25 to 29 | RA | Female | 76.10 (62.52, 92.63) |
| Afghanistan | 25 to 29 | RA | Male | 52.40 (38.83, 70.72) |
| Afghanistan | 30 to 34 | RA | Both | 94.96 (80.63, 111.82) |
| Afghanistan | 30 to 34 | RA | Female | 108.42 (88.99, 132.08) |
| Afghanistan | 30 to 34 | RA | Male | 72.92 (53.93, 98.59) |
| Afghanistan | 35 to 39 | RA | Both | 126.93 (107.71, 149.58) |
| Afghanistan | 35 to 39 | RA | Female | 144.28 (118.35, 175.90) |
| Afghanistan | 35 to 39 | RA | Male | 98.33 (72.62, 133.16) |
| Afghanistan | 40 to 44 | RA | Both | 162.16 (137.50, 191.24) |

| | | | | |
|---|---|---|---|---|
| Afghanistan | 40 to 44 | RA | Female | 182.72 (149.75, 222.95) |
| Afghanistan | 40 to 44 | RA | Male | 129.32 (95.31, 175.47) |
| Afghanistan | 45 to 49 | RA | Both | 198.10 (167.77, 233.90) |
| Afghanistan | 45 to 49 | RA | Female | 220.79 (180.73, 269.73) |
| Afghanistan | 45 to 49 | RA | Male | 165.03 (121.24, 224.64) |
| Afghanistan | 5 to 9 | RA | Both | 1.00 (1.00, 1.00) |
| Afghanistan | 5 to 9 | RA | Female | 1.00 (1.00, 1.00) |
| Afghanistan | 5 to 9 | RA | Male | 1.00 (1.00, 1.00) |
| Afghanistan | 50 to 54 | RA | Both | 232.58 (196.70, 275.01) |
| Afghanistan | 50 to 54 | RA | Female | 256.21 (209.41, 313.46) |
| Afghanistan | 50 to 54 | RA | Male | 204.92 (149.97, 280.02) |
| Afghanistan | 55 to 59 | RA | Both | 260.48 (220.03, 308.38) |
| Afghanistan | 55 to 59 | RA | Female | 283.42 (231.36, 347.20) |
| Afghanistan | 55 to 59 | RA | Male | 243.63 (177.74, 333.94) |
| Afghanistan | 60 to 64 | RA | Both | 280.46 (236.66, 332.38) |
| Afghanistan | 60 to 64 | RA | Female | 299.79 (244.42, 367.71) |
| Afghanistan | 60 to 64 | RA | Male | 278.65 (202.92, 382.64) |
| Afghanistan | 65 to 69 | RA | Both | 283.11 (238.49, 336.09) |
| Afghanistan | 65 to 69 | RA | Female | 295.85 (240.71, 363.62) |
| Afghanistan | 65 to 69 | RA | Male | 297.67 (216.19, 409.86) |
| Albania | 0 to 4 | AA | Both | 0.73 (0.67, 0.80) |
| Albania | 0 to 4 | AA | Female | 0.73 (0.65, 0.82) |
| Albania | 0 to 4 | AA | Male | 0.74 (0.63, 0.86) |
| Albania | 10 to 14 | AA | Both | 1.28 (1.19, 1.38) |
| Albania | 10 to 14 | AA | Female | 1.27 (1.15, 1.39) |
| Albania | 10 to 14 | AA | Male | 1.29 (1.14, 1.47) |
| Albania | 15 to 19 | AA | Both | 2.23 (2.07, 2.39) |
| Albania | 15 to 19 | AA | Female | 2.20 (2.01, 2.40) |
| Albania | 15 to 19 | AA | Male | 2.23 (1.98, 2.52) |
| Albania | 20 to 24 | AA | Both | 3.49 (3.25, 3.75) |
| Albania | 20 to 24 | AA | Female | 3.46 (3.17, 3.78) |
| Albania | 20 to 24 | AA | Male | 3.52 (3.13, 3.96) |
| Albania | 25 to 29 | AA | Both | 4.84 (4.51, 5.20) |

| | | | | |
|---|---|---|---|---|
| Albania | 25 to 29 | AA | Female | 4.79 (4.38, 5.24) |
| Albania | 25 to 29 | AA | Male | 4.89 (4.34, 5.52) |
| Albania | 30 to 34 | AA | Both | 5.21 (4.83, 5.61) |
| Albania | 30 to 34 | AA | Female | 5.14 (4.69, 5.64) |
| Albania | 30 to 34 | AA | Male | 5.18 (4.57, 5.87) |
| Albania | 35 to 39 | AA | Both | 4.88 (4.52, 5.28) |
| Albania | 35 to 39 | AA | Female | 4.80 (4.36, 5.28) |
| Albania | 35 to 39 | AA | Male | 4.88 (4.29, 5.56) |
| Albania | 40 to 44 | AA | Both | 4.14 (3.82, 4.48) |
| Albania | 40 to 44 | AA | Female | 4.06 (3.67, 4.48) |
| Albania | 40 to 44 | AA | Male | 4.15 (3.63, 4.75) |
| Albania | 45 to 49 | AA | Both | 3.57 (3.29, 3.89) |
| Albania | 45 to 49 | AA | Female | 3.51 (3.16, 3.89) |
| Albania | 45 to 49 | AA | Male | 3.56 (3.09, 4.10) |
| Albania | 5 to 9 | AA | Both | 1.00 (1.00, 1.00) |
| Albania | 5 to 9 | AA | Female | 1.00 (1.00, 1.00) |
| Albania | 5 to 9 | AA | Male | 1.00 (1.00, 1.00) |
| Albania | 50 to 54 | AA | Both | 3.20 (2.93, 3.49) |
| Albania | 50 to 54 | AA | Female | 3.12 (2.80, 3.48) |
| Albania | 50 to 54 | AA | Male | 3.18 (2.75, 3.69) |
| Albania | 55 to 59 | AA | Both | 3.05 (2.79, 3.35) |
| Albania | 55 to 59 | AA | Female | 2.97 (2.65, 3.33) |
| Albania | 55 to 59 | AA | Male | 3.02 (2.59, 3.52) |
| Albania | 60 to 64 | AA | Both | 3.19 (2.90, 3.52) |
| Albania | 60 to 64 | AA | Female | 3.09 (2.74, 3.48) |
| Albania | 60 to 64 | AA | Male | 3.14 (2.67, 3.70) |
| Albania | 65 to 69 | AA | Both | 3.00 (2.70, 3.33) |
| Albania | 65 to 69 | AA | Female | 2.88 (2.52, 3.29) |
| Albania | 65 to 69 | AA | Male | 2.94 (2.45, 3.51) |
| Albania | 0 to 4 | IBD | Both | 0.02 (0.01, 0.41) |
| Albania | 0 to 4 | IBD | Female | 0.05 (0.01, 0.87) |
| Albania | 0 to 4 | IBD | Male | 0.05 (0.01, 0.86) |
| Albania | 10 to 14 | IBD | Both | 5.76 (3.72, 8.92) |

| | | | | |
|---|---|---|---|---|
| Albania | 10 to 14 | IBD | Female | 5.51 (2.93, 10.36) |
| Albania | 10 to 14 | IBD | Male | 6.48 (3.46, 12.13) |
| Albania | 15 to 19 | IBD | Both | 22.35 (14.67, 34.06) |
| Albania | 15 to 19 | IBD | Female | 23.85 (13.04, 43.63) |
| Albania | 15 to 19 | IBD | Male | 22.25 (12.15, 40.73) |
| Albania | 20 to 24 | IBD | Both | 49.18 (32.33, 74.83) |
| Albania | 20 to 24 | IBD | Female | 51.72 (28.33, 94.45) |
| Albania | 20 to 24 | IBD | Male | 49.74 (27.24, 90.82) |
| Albania | 25 to 29 | IBD | Both | 78.55 (51.60, 119.57) |
| Albania | 25 to 29 | IBD | Female | 81.30 (44.50, 148.51) |
| Albania | 25 to 29 | IBD | Male | 80.89 (44.29, 147.74) |
| Albania | 30 to 34 | IBD | Both | 102.08 (67.01, 155.50) |
| Albania | 30 to 34 | IBD | Female | 104.49 (57.14, 191.08) |
| Albania | 30 to 34 | IBD | Male | 106.09 (58.01, 194.02) |
| Albania | 35 to 39 | IBD | Both | 122.60 (80.42, 186.89) |
| Albania | 35 to 39 | IBD | Female | 126.35 (69.04, 231.24) |
| Albania | 35 to 39 | IBD | Male | 126.69 (69.19, 231.95) |
| Albania | 40 to 44 | IBD | Both | 131.50 (86.22, 200.57) |
| Albania | 40 to 44 | IBD | Female | 129.28 (70.57, 236.83) |
| Albania | 40 to 44 | IBD | Male | 143.24 (78.19, 262.40) |
| Albania | 45 to 49 | IBD | Both | 135.45 (88.75, 206.72) |
| Albania | 45 to 49 | IBD | Female | 123.58 (67.39, 226.63) |
| Albania | 45 to 49 | IBD | Male | 157.94 (86.15, 289.54) |
| Albania | 5 to 9 | IBD | Both | 1.00 (1.00, 1.00) |
| Albania | 5 to 9 | IBD | Female | 1.00 (1.00, 1.00) |
| Albania | 5 to 9 | IBD | Male | 1.00 (1.00, 1.00) |
| Albania | 50 to 54 | IBD | Both | 146.17 (95.71, 223.23) |
| Albania | 50 to 54 | IBD | Female | 132.17 (72.01, 242.60) |
| Albania | 50 to 54 | IBD | Male | 171.52 (93.48, 314.72) |
| Albania | 55 to 59 | IBD | Both | 154.48 (101.06, 236.12) |
| Albania | 55 to 59 | IBD | Female | 139.70 (76.02, 256.75) |
| Albania | 55 to 59 | IBD | Male | 181.72 (98.93, 333.81) |
| Albania | 60 to 64 | IBD | Both | 159.08 (103.95, 243.43) |

| | | | | |
|---|---|---|---|---|
| Albania | 60 to 64 | IBD | Female | 144.92 (78.72, 266.80) |
| Albania | 60 to 64 | IBD | Male | 186.60 (101.43, 343.30) |
| Albania | 65 to 69 | IBD | Both | 156.14 (101.85, 239.36) |
| Albania | 65 to 69 | IBD | Female | 142.49 (77.19, 263.02) |
| Albania | 65 to 69 | IBD | Male | 182.76 (99.11, 337.02) |
| Albania | 10 to 14 | MS | Both | 38.96 (2.35, 645.17) |
| Albania | 10 to 14 | MS | Female | 22.03 (1.29, 375.40) |
| Albania | 10 to 14 | MS | Male | 17.18 (0.99, 296.64) |
| Albania | 15 to 19 | MS | Both | 182.25 (11.15, 2978.08) |
| Albania | 15 to 19 | MS | Female | 105.35 (6.33, 1752.87) |
| Albania | 15 to 19 | MS | Male | 78.10 (4.67, 1306.68) |
| Albania | 20 to 24 | MS | Both | 519.35 (31.83, 8473.48) |
| Albania | 20 to 24 | MS | Female | 307.16 (18.51, 5097.84) |
| Albania | 20 to 24 | MS | Male | 211.41 (12.68, 3524.77) |
| Albania | 25 to 29 | MS | Both | 1065.52 (65.31, 17384.42) |
| Albania | 25 to 29 | MS | Female | 633.30 (38.16, 10510.18) |
| Albania | 25 to 29 | MS | Male | 431.63 (25.89, 7196.18) |
| Albania | 30 to 34 | MS | Both | 1761.69 (107.95, 28748.86) |
| Albania | 30 to 34 | MS | Female | 1091.48 (65.75, 18117.98) |
| Albania | 30 to 34 | MS | Male | 661.26 (39.63, 11033.64) |
| Albania | 35 to 39 | MS | Both | 2827.99 (173.27, 46156.58) |
| Albania | 35 to 39 | MS | Female | 1893.84 (114.07, 31442.41) |
| Albania | 35 to 39 | MS | Male | 912.70 (54.66, 15239.31) |
| Albania | 40 to 44 | MS | Both | 4675.12 (286.44, 76303.99) |
| Albania | 40 to 44 | MS | Female | 3259.14 (196.30, 54110.41) |
| Albania | 40 to 44 | MS | Male | 1394.51 (83.52, 23283.40) |
| Albania | 45 to 49 | MS | Both | 7092.11 (434.49, 115764.14) |
| Albania | 45 to 49 | MS | Female | 4433.84 (266.99, 73632.69) |
| Albania | 45 to 49 | MS | Male | 2581.91 (154.65, 43105.64) |
| Albania | 5 to 9 | MS | Both | 1.00 (1.00, 1.00) |
| Albania | 5 to 9 | MS | Female | 1.00 (1.00, 1.00) |
| Albania | 5 to 9 | MS | Male | 1.00 (1.00, 1.00) |
| Albania | 50 to 54 | MS | Both | 9462.19 (579.61, 154471.39) |

| | | | | |
|---|---|---|---|---|
| Albania | 50 to 54 | MS | Female | 5052.56 (304.14, 83936.65) |
| Albania | 50 to 54 | MS | Male | 4132.10 (247.47, 68996.40) |
| Albania | 55 to 59 | MS | Both | 10293.47 (630.43, 168069.12) |
| Albania | 55 to 59 | MS | Female | 5134.21 (308.94, 85323.86) |
| Albania | 55 to 59 | MS | Male | 4754.94 (284.68, 79419.55) |
| Albania | 60 to 64 | MS | Both | 9643.77 (590.53, 157489.19) |
| Albania | 60 to 64 | MS | Female | 4774.09 (287.16, 79370.76) |
| Albania | 60 to 64 | MS | Male | 4481.69 (268.24, 74879.85) |
| Albania | 65 to 69 | MS | Both | 8653.07 (529.74, 141342.77) |
| Albania | 65 to 69 | MS | Female | 4267.80 (256.57, 70989.85) |
| Albania | 65 to 69 | MS | Male | 4047.90 (242.18, 67659.50) |
| Albania | 0 to 4 | Psoriasis | Both | 0.32 (0.30, 0.35) |
| Albania | 0 to 4 | Psoriasis | Female | 0.30 (0.26, 0.34) |
| Albania | 0 to 4 | Psoriasis | Male | 0.34 (0.31, 0.39) |
| Albania | 10 to 14 | Psoriasis | Both | 1.76 (1.67, 1.84) |
| Albania | 10 to 14 | Psoriasis | Female | 1.97 (1.84, 2.10) |
| Albania | 10 to 14 | Psoriasis | Male | 1.55 (1.44, 1.66) |
| Albania | 15 to 19 | Psoriasis | Both | 2.39 (2.28, 2.51) |
| Albania | 15 to 19 | Psoriasis | Female | 2.70 (2.53, 2.89) |
| Albania | 15 to 19 | Psoriasis | Male | 2.07 (1.93, 2.21) |
| Albania | 20 to 24 | Psoriasis | Both | 2.37 (2.26, 2.49) |
| Albania | 20 to 24 | Psoriasis | Female | 2.51 (2.34, 2.69) |
| Albania | 20 to 24 | Psoriasis | Male | 2.23 (2.08, 2.39) |
| Albania | 25 to 29 | Psoriasis | Both | 2.27 (2.16, 2.39) |
| Albania | 25 to 29 | Psoriasis | Female | 2.20 (2.04, 2.36) |
| Albania | 25 to 29 | Psoriasis | Male | 2.35 (2.19, 2.53) |
| Albania | 30 to 34 | Psoriasis | Both | 2.46 (2.34, 2.60) |
| Albania | 30 to 34 | Psoriasis | Female | 2.23 (2.06, 2.40) |
| Albania | 30 to 34 | Psoriasis | Male | 2.72 (2.53, 2.93) |
| Albania | 35 to 39 | Psoriasis | Both | 2.66 (2.52, 2.81) |
| Albania | 35 to 39 | Psoriasis | Female | 2.32 (2.14, 2.51) |
| Albania | 35 to 39 | Psoriasis | Male | 3.04 (2.82, 3.28) |
| Albania | 40 to 44 | Psoriasis | Both | 2.84 (2.68, 3.00) |

| | | | | |
|---|---|---|---|---|
| Albania | 40 to 44 | Psoriasis | Female | 2.40 (2.22, 2.60) |
| Albania | 40 to 44 | Psoriasis | Male | 3.32 (3.07, 3.59) |
| Albania | 45 to 49 | Psoriasis | Both | 3.06 (2.89, 3.24) |
| Albania | 45 to 49 | Psoriasis | Female | 2.68 (2.47, 2.90) |
| Albania | 45 to 49 | Psoriasis | Male | 3.50 (3.23, 3.78) |
| Albania | 5 to 9 | Psoriasis | Both | 1.00 (1.00, 1.00) |
| Albania | 5 to 9 | Psoriasis | Female | 1.00 (1.00, 1.00) |
| Albania | 5 to 9 | Psoriasis | Male | 1.00 (1.00, 1.00) |
| Albania | 50 to 54 | Psoriasis | Both | 3.35 (3.16, 3.54) |
| Albania | 50 to 54 | Psoriasis | Female | 3.19 (2.93, 3.46) |
| Albania | 50 to 54 | Psoriasis | Male | 3.55 (3.28, 3.85) |
| Albania | 55 to 59 | Psoriasis | Both | 3.59 (3.38, 3.81) |
| Albania | 55 to 59 | Psoriasis | Female | 3.52 (3.23, 3.83) |
| Albania | 55 to 59 | Psoriasis | Male | 3.71 (3.42, 4.03) |
| Albania | 60 to 64 | Psoriasis | Both | 3.72 (3.50, 3.95) |
| Albania | 60 to 64 | Psoriasis | Female | 3.60 (3.30, 3.93) |
| Albania | 60 to 64 | Psoriasis | Male | 3.90 (3.58, 4.25) |
| Albania | 65 to 69 | Psoriasis | Both | 3.54 (3.33, 3.78) |
| Albania | 65 to 69 | Psoriasis | Female | 3.31 (3.02, 3.63) |
| Albania | 65 to 69 | Psoriasis | Male | 3.85 (3.52, 4.20) |
| Albania | 0 to 4 | RHD | Both | 0.17 (0.16, 0.19) |
| Albania | 0 to 4 | RHD | Female | 0.17 (0.15, 0.19) |
| Albania | 0 to 4 | RHD | Male | 0.17 (0.15, 0.19) |
| Albania | 10 to 14 | RHD | Both | 2.01 (1.95, 2.09) |
| Albania | 10 to 14 | RHD | Female | 2.01 (1.92, 2.12) |
| Albania | 10 to 14 | RHD | Male | 2.01 (1.91, 2.11) |
| Albania | 15 to 19 | RHD | Both | 2.87 (2.78, 2.97) |
| Albania | 15 to 19 | RHD | Female | 2.88 (2.75, 3.02) |
| Albania | 15 to 19 | RHD | Male | 2.86 (2.72, 3.00) |
| Albania | 20 to 24 | RHD | Both | 3.30 (3.19, 3.42) |
| Albania | 20 to 24 | RHD | Female | 3.36 (3.20, 3.52) |
| Albania | 20 to 24 | RHD | Male | 3.24 (3.09, 3.41) |
| Albania | 25 to 29 | RHD | Both | 3.52 (3.40, 3.65) |

| | | | | |
|---|---|---|---|---|
| Albania | 25 to 29 | RHD | Female | 3.61 (3.43, 3.80) |
| Albania | 25 to 29 | RHD | Male | 3.42 (3.25, 3.60) |
| Albania | 30 to 34 | RHD | Both | 3.62 (3.49, 3.76) |
| Albania | 30 to 34 | RHD | Female | 3.74 (3.55, 3.94) |
| Albania | 30 to 34 | RHD | Male | 3.48 (3.30, 3.67) |
| Albania | 35 to 39 | RHD | Both | 3.62 (3.49, 3.77) |
| Albania | 35 to 39 | RHD | Female | 3.76 (3.56, 3.97) |
| Albania | 35 to 39 | RHD | Male | 3.46 (3.27, 3.66) |
| Albania | 40 to 44 | RHD | Both | 3.52 (3.38, 3.66) |
| Albania | 40 to 44 | RHD | Female | 3.66 (3.47, 3.88) |
| Albania | 40 to 44 | RHD | Male | 3.34 (3.15, 3.54) |
| Albania | 45 to 49 | RHD | Both | 3.23 (3.10, 3.37) |
| Albania | 45 to 49 | RHD | Female | 3.40 (3.20, 3.60) |
| Albania | 45 to 49 | RHD | Male | 3.03 (2.85, 3.22) |
| Albania | 5 to 9 | RHD | Both | 1.00 (1.00, 1.00) |
| Albania | 5 to 9 | RHD | Female | 1.00 (1.00, 1.00) |
| Albania | 5 to 9 | RHD | Male | 1.00 (1.00, 1.00) |
| Albania | 50 to 54 | RHD | Both | 2.80 (2.68, 2.93) |
| Albania | 50 to 54 | RHD | Female | 2.99 (2.82, 3.18) |
| Albania | 50 to 54 | RHD | Male | 2.57 (2.41, 2.74) |
| Albania | 55 to 59 | RHD | Both | 2.38 (2.27, 2.50) |
| Albania | 55 to 59 | RHD | Female | 2.59 (2.43, 2.77) |
| Albania | 55 to 59 | RHD | Male | 2.13 (1.99, 2.28) |
| Albania | 60 to 64 | RHD | Both | 2.01 (1.90, 2.11) |
| Albania | 60 to 64 | RHD | Female | 2.22 (2.07, 2.39) |
| Albania | 60 to 64 | RHD | Male | 1.75 (1.62, 1.89) |
| Albania | 65 to 69 | RHD | Both | 1.66 (1.56, 1.76) |
| Albania | 65 to 69 | RHD | Female | 1.87 (1.72, 2.03) |
| Albania | 65 to 69 | RHD | Male | 1.41 (1.29, 1.54) |
| Albania | 10 to 14 | RA | Both | 7.46 (4.69, 11.85) |
| Albania | 10 to 14 | RA | Female | 7.61 (4.29, 13.51) |
| Albania | 10 to 14 | RA | Male | 6.87 (3.13, 15.09) |
| Albania | 15 to 19 | RA | Both | 21.82 (13.88, 34.30) |

| | | | | |
|---|---|---|---|---|
| Albania | 15 to 19 | RA | Female | 22.85 (13.04, 40.02) |
| Albania | 15 to 19 | RA | Male | 19.27 (8.95, 41.49) |
| Albania | 20 to 24 | RA | Both | 43.46 (27.68, 68.22) |
| Albania | 20 to 24 | RA | Female | 45.95 (26.28, 80.37) |
| Albania | 20 to 24 | RA | Male | 38.10 (17.76, 81.75) |
| Albania | 25 to 29 | RA | Both | 69.65 (44.35, 109.38) |
| Albania | 25 to 29 | RA | Female | 73.10 (41.76, 127.95) |
| Albania | 25 to 29 | RA | Male | 61.88 (28.84, 132.78) |
| Albania | 30 to 34 | RA | Both | 101.13 (64.35, 158.94) |
| Albania | 30 to 34 | RA | Female | 104.01 (59.38, 182.21) |
| Albania | 30 to 34 | RA | Male | 91.28 (42.47, 196.16) |
| Albania | 35 to 39 | RA | Both | 140.89 (89.59, 221.57) |
| Albania | 35 to 39 | RA | Female | 143.08 (81.62, 250.82) |
| Albania | 35 to 39 | RA | Male | 129.94 (60.38, 279.64) |
| Albania | 40 to 44 | RA | Both | 190.19 (120.91, 299.17) |
| Albania | 40 to 44 | RA | Female | 191.01 (108.93, 334.92) |
| Albania | 40 to 44 | RA | Male | 179.32 (83.30, 386.02) |
| Albania | 45 to 49 | RA | Both | 249.42 (158.50, 392.48) |
| Albania | 45 to 49 | RA | Female | 246.11 (140.29, 431.77) |
| Albania | 45 to 49 | RA | Male | 242.63 (112.65, 522.60) |
| Albania | 5 to 9 | RA | Both | 1.00 (1.00, 1.00) |
| Albania | 5 to 9 | RA | Female | 1.00 (1.00, 1.00) |
| Albania | 5 to 9 | RA | Male | 1.00 (1.00, 1.00) |
| Albania | 50 to 54 | RA | Both | 317.90 (201.93, 500.46) |
| Albania | 50 to 54 | RA | Female | 306.05 (174.35, 537.25) |
| Albania | 50 to 54 | RA | Male | 321.77 (149.30, 693.48) |
| Albania | 55 to 59 | RA | Both | 389.86 (247.51, 614.10) |
| Albania | 55 to 59 | RA | Female | 361.91 (206.02, 635.78) |
| Albania | 55 to 59 | RA | Male | 413.74 (191.82, 892.43) |
| Albania | 60 to 64 | RA | Both | 459.42 (291.47, 724.15) |
| Albania | 60 to 64 | RA | Female | 409.91 (233.12, 720.78) |
| Albania | 60 to 64 | RA | Male | 514.76 (238.43, 1111.38) |
| Albania | 65 to 69 | RA | Both | 506.14 (320.82, 798.51) |

| | | | | |
|---|---|---|---|---|
| Albania | 65 to 69 | RA | Female | 431.82 (245.27, 760.26) |
| Albania | 65 to 69 | RA | Male | 600.19 (277.66, 1297.36) |
| Algeria | 0 to 4 | AA | Both | 0.71 (0.69, 0.73) |
| Algeria | 0 to 4 | AA | Female | 0.73 (0.71, 0.76) |
| Algeria | 0 to 4 | AA | Male | 0.67 (0.64, 0.70) |
| Algeria | 10 to 14 | AA | Both | 1.28 (1.25, 1.30) |
| Algeria | 10 to 14 | AA | Female | 1.26 (1.22, 1.29) |
| Algeria | 10 to 14 | AA | Male | 1.31 (1.26, 1.36) |
| Algeria | 15 to 19 | AA | Both | 2.13 (2.09, 2.18) |
| Algeria | 15 to 19 | AA | Female | 2.19 (2.13, 2.25) |
| Algeria | 15 to 19 | AA | Male | 2.04 (1.97, 2.11) |
| Algeria | 20 to 24 | AA | Both | 3.34 (3.27, 3.41) |
| Algeria | 20 to 24 | AA | Female | 3.42 (3.34, 3.52) |
| Algeria | 20 to 24 | AA | Male | 3.18 (3.07, 3.29) |
| Algeria | 25 to 29 | AA | Both | 4.68 (4.58, 4.78) |
| Algeria | 25 to 29 | AA | Female | 4.78 (4.66, 4.91) |
| Algeria | 25 to 29 | AA | Male | 4.48 (4.32, 4.64) |
| Algeria | 30 to 34 | AA | Both | 4.96 (4.85, 5.07) |
| Algeria | 30 to 34 | AA | Female | 5.06 (4.92, 5.20) |
| Algeria | 30 to 34 | AA | Male | 4.73 (4.56, 4.91) |
| Algeria | 35 to 39 | AA | Both | 4.65 (4.55, 4.76) |
| Algeria | 35 to 39 | AA | Female | 4.75 (4.62, 4.89) |
| Algeria | 35 to 39 | AA | Male | 4.41 (4.24, 4.58) |
| Algeria | 40 to 44 | AA | Both | 3.96 (3.87, 4.06) |
| Algeria | 40 to 44 | AA | Female | 4.02 (3.90, 4.15) |
| Algeria | 40 to 44 | AA | Male | 3.78 (3.62, 3.93) |
| Algeria | 45 to 49 | AA | Both | 3.42 (3.33, 3.51) |
| Algeria | 45 to 49 | AA | Female | 3.48 (3.37, 3.60) |
| Algeria | 45 to 49 | AA | Male | 3.24 (3.10, 3.38) |
| Algeria | 5 to 9 | AA | Both | 1.00 (1.00, 1.00) |
| Algeria | 5 to 9 | AA | Female | 1.00 (1.00, 1.00) |
| Algeria | 5 to 9 | AA | Male | 1.00 (1.00, 1.00) |
| Algeria | 50 to 54 | AA | Both | 3.05 (2.97, 3.14) |

| | | | | |
|---|---|---|---|---|
| Algeria | 50 to 54 | AA | Female | 3.11 (3.01, 3.23) |
| Algeria | 50 to 54 | AA | Male | 2.90 (2.76, 3.04) |
| Algeria | 55 to 59 | AA | Both | 2.87 (2.79, 2.96) |
| Algeria | 55 to 59 | AA | Female | 2.94 (2.82, 3.05) |
| Algeria | 55 to 59 | AA | Male | 2.73 (2.59, 2.88) |
| Algeria | 60 to 64 | AA | Both | 2.98 (2.89, 3.09) |
| Algeria | 60 to 64 | AA | Female | 3.06 (2.93, 3.19) |
| Algeria | 60 to 64 | AA | Male | 2.84 (2.68, 3.01) |
| Algeria | 65 to 69 | AA | Both | 2.79 (2.69, 2.90) |
| Algeria | 65 to 69 | AA | Female | 2.86 (2.73, 3.00) |
| Algeria | 65 to 69 | AA | Male | 2.68 (2.51, 2.86) |
| Algeria | 0 to 4 | IBD | Both | 0.05 (0.02, 0.13) |
| Algeria | 0 to 4 | IBD | Female | 0.01 (0.01, 0.19) |
| Algeria | 0 to 4 | IBD | Male | 0.05 (0.01, 0.27) |
| Algeria | 10 to 14 | IBD | Both | 4.40 (3.51, 5.50) |
| Algeria | 10 to 14 | IBD | Female | 4.59 (3.29, 6.40) |
| Algeria | 10 to 14 | IBD | Male | 4.48 (3.28, 6.11) |
| Algeria | 15 to 19 | IBD | Both | 14.98 (12.07, 18.59) |
| Algeria | 15 to 19 | IBD | Female | 17.97 (13.05, 24.74) |
| Algeria | 15 to 19 | IBD | Male | 13.20 (9.77, 17.83) |
| Algeria | 20 to 24 | IBD | Both | 33.97 (27.42, 42.09) |
| Algeria | 20 to 24 | IBD | Female | 40.96 (29.81, 56.28) |
| Algeria | 20 to 24 | IBD | Male | 29.77 (22.10, 40.11) |
| Algeria | 25 to 29 | IBD | Both | 60.66 (48.96, 75.16) |
| Algeria | 25 to 29 | IBD | Female | 73.60 (53.57, 101.13) |
| Algeria | 25 to 29 | IBD | Male | 52.72 (39.13, 71.02) |
| Algeria | 30 to 34 | IBD | Both | 90.51 (73.02, 112.19) |
| Algeria | 30 to 34 | IBD | Female | 110.14 (80.11, 151.41) |
| Algeria | 30 to 34 | IBD | Male | 78.33 (58.10, 105.59) |
| Algeria | 35 to 39 | IBD | Both | 119.86 (96.64, 148.66) |
| Algeria | 35 to 39 | IBD | Female | 145.84 (106.01, 200.64) |
| Algeria | 35 to 39 | IBD | Male | 103.62 (76.80, 139.82) |
| Algeria | 40 to 44 | IBD | Both | 147.63 (118.98, 183.19) |

| | | | | |
|---|---|---|---|---|
| Algeria | 40 to 44 | IBD | Female | 179.45 (130.37, 247.02) |
| Algeria | 40 to 44 | IBD | Male | 127.55 (94.47, 172.23) |
| Algeria | 45 to 49 | IBD | Both | 173.11 (139.44, 214.91) |
| Algeria | 45 to 49 | IBD | Female | 210.39 (152.74, 289.79) |
| Algeria | 45 to 49 | IBD | Male | 149.66 (110.75, 202.24) |
| Algeria | 5 to 9 | IBD | Both | 1.00 (1.00, 1.00) |
| Algeria | 5 to 9 | IBD | Female | 1.00 (1.00, 1.00) |
| Algeria | 5 to 9 | IBD | Male | 1.00 (1.00, 1.00) |
| Algeria | 50 to 54 | IBD | Both | 168.60 (135.69, 209.50) |
| Algeria | 50 to 54 | IBD | Female | 179.18 (129.91, 247.13) |
| Algeria | 50 to 54 | IBD | Male | 167.78 (124.03, 226.97) |
| Algeria | 55 to 59 | IBD | Both | 178.44 (143.47, 221.92) |
| Algeria | 55 to 59 | IBD | Female | 190.30 (137.80, 262.81) |
| Algeria | 55 to 59 | IBD | Male | 176.84 (130.55, 239.55) |
| Algeria | 60 to 64 | IBD | Both | 175.25 (140.72, 218.24) |
| Algeria | 60 to 64 | IBD | Female | 186.72 (134.97, 258.32) |
| Algeria | 60 to 64 | IBD | Male | 173.76 (128.03, 235.81) |
| Algeria | 65 to 69 | IBD | Both | 159.15 (127.52, 198.63) |
| Algeria | 65 to 69 | IBD | Female | 167.68 (120.83, 232.69) |
| Algeria | 65 to 69 | IBD | Male | 159.38 (117.09, 216.95) |
| Algeria | 10 to 14 | MS | Both | 9.56 (6.18, 14.78) |
| Algeria | 10 to 14 | MS | Female | 9.50 (5.50, 16.41) |
| Algeria | 10 to 14 | MS | Male | 7.79 (4.06, 14.96) |
| Algeria | 15 to 19 | MS | Both | 41.53 (27.11, 63.61) |
| Algeria | 15 to 19 | MS | Female | 43.14 (25.24, 73.74) |
| Algeria | 15 to 19 | MS | Male | 30.98 (16.37, 58.61) |
| Algeria | 20 to 24 | MS | Both | 120.66 (78.89, 184.54) |
| Algeria | 20 to 24 | MS | Female | 127.78 (74.91, 217.95) |
| Algeria | 20 to 24 | MS | Male | 85.43 (45.26, 161.23) |
| Algeria | 25 to 29 | MS | Both | 261.31 (170.88, 399.59) |
| Algeria | 25 to 29 | MS | Female | 277.21 (162.53, 472.80) |
| Algeria | 25 to 29 | MS | Male | 184.27 (97.65, 347.73) |
| Algeria | 30 to 34 | MS | Both | 421.10 (275.30, 644.11) |

| | | | | |
|---|---|---|---|---|
| Algeria | 30 to 34 | MS | Female | 441.14 (258.57, 752.62) |
| Algeria | 30 to 34 | MS | Male | 303.92 (160.98, 573.80) |
| Algeria | 35 to 39 | MS | Both | 568.08 (371.26, 869.25) |
| Algeria | 35 to 39 | MS | Female | 584.58 (342.50, 997.77) |
| Algeria | 35 to 39 | MS | Male | 422.95 (223.87, 799.08) |
| Algeria | 40 to 44 | MS | Both | 683.83 (446.78, 1046.65) |
| Algeria | 40 to 44 | MS | Female | 691.99 (405.29, 1181.51) |
| Algeria | 40 to 44 | MS | Male | 523.54 (276.95, 989.66) |
| Algeria | 45 to 49 | MS | Both | 770.71 (503.38, 1180.01) |
| Algeria | 45 to 49 | MS | Female | 772.03 (451.99, 1318.69) |
| Algeria | 45 to 49 | MS | Male | 601.83 (318.17, 1138.35) |
| Algeria | 5 to 9 | MS | Both | 1.00 (1.00, 1.00) |
| Algeria | 5 to 9 | MS | Female | 1.00 (1.00, 1.00) |
| Algeria | 5 to 9 | MS | Male | 1.00 (1.00, 1.00) |
| Algeria | 50 to 54 | MS | Both | 831.39 (542.79, 1273.44) |
| Algeria | 50 to 54 | MS | Female | 828.65 (484.89, 1416.12) |
| Algeria | 50 to 54 | MS | Male | 656.57 (346.84, 1242.87) |
| Algeria | 55 to 59 | MS | Both | 869.60 (567.45, 1332.64) |
| Algeria | 55 to 59 | MS | Female | 867.34 (507.21, 1483.17) |
| Algeria | 55 to 59 | MS | Male | 689.44 (363.85, 1306.36) |
| Algeria | 60 to 64 | MS | Both | 890.48 (580.72, 1365.46) |
| Algeria | 60 to 64 | MS | Female | 892.87 (521.76, 1527.94) |
| Algeria | 60 to 64 | MS | Male | 704.58 (371.41, 1336.62) |
| Algeria | 65 to 69 | MS | Both | 908.09 (591.73, 1393.58) |
| Algeria | 65 to 69 | MS | Female | 914.47 (533.86, 1566.43) |
| Algeria | 65 to 69 | MS | Male | 716.42 (377.05, 1361.23) |
| Algeria | 0 to 4 | Psoriasis | Both | 0.47 (0.46, 0.48) |
| Algeria | 0 to 4 | Psoriasis | Female | 0.47 (0.45, 0.48) |
| Algeria | 0 to 4 | Psoriasis | Male | 0.47 (0.46, 0.48) |
| Algeria | 10 to 14 | Psoriasis | Both | 1.16 (1.14, 1.18) |
| Algeria | 10 to 14 | Psoriasis | Female | 1.18 (1.16, 1.21) |
| Algeria | 10 to 14 | Psoriasis | Male | 1.13 (1.11, 1.16) |
| Algeria | 15 to 19 | Psoriasis | Both | 1.31 (1.29, 1.32) |

| | | | | |
|---|---|---|---|---|
| Algeria | 15 to 19 | Psoriasis | Female | 1.36 (1.33, 1.38) |
| Algeria | 15 to 19 | Psoriasis | Male | 1.25 (1.22, 1.27) |
| Algeria | 20 to 24 | Psoriasis | Both | 1.52 (1.50, 1.54) |
| Algeria | 20 to 24 | Psoriasis | Female | 1.56 (1.53, 1.59) |
| Algeria | 20 to 24 | Psoriasis | Male | 1.47 (1.44, 1.50) |
| Algeria | 25 to 29 | Psoriasis | Both | 1.79 (1.76, 1.81) |
| Algeria | 25 to 29 | Psoriasis | Female | 1.74 (1.71, 1.77) |
| Algeria | 25 to 29 | Psoriasis | Male | 1.83 (1.80, 1.87) |
| Algeria | 30 to 34 | Psoriasis | Both | 2.03 (2.00, 2.06) |
| Algeria | 30 to 34 | Psoriasis | Female | 1.91 (1.87, 1.94) |
| Algeria | 30 to 34 | Psoriasis | Male | 2.17 (2.12, 2.22) |
| Algeria | 35 to 39 | Psoriasis | Both | 2.17 (2.13, 2.20) |
| Algeria | 35 to 39 | Psoriasis | Female | 2.02 (1.98, 2.06) |
| Algeria | 35 to 39 | Psoriasis | Male | 2.34 (2.28, 2.39) |
| Algeria | 40 to 44 | Psoriasis | Both | 2.25 (2.21, 2.28) |
| Algeria | 40 to 44 | Psoriasis | Female | 2.10 (2.05, 2.15) |
| Algeria | 40 to 44 | Psoriasis | Male | 2.42 (2.36, 2.48) |
| Algeria | 45 to 49 | Psoriasis | Both | 2.33 (2.30, 2.37) |
| Algeria | 45 to 49 | Psoriasis | Female | 2.19 (2.14, 2.24) |
| Algeria | 45 to 49 | Psoriasis | Male | 2.50 (2.44, 2.56) |
| Algeria | 5 to 9 | Psoriasis | Both | 1.00 (1.00, 1.00) |
| Algeria | 5 to 9 | Psoriasis | Female | 1.00 (1.00, 1.00) |
| Algeria | 5 to 9 | Psoriasis | Male | 1.00 (1.00, 1.00) |
| Algeria | 50 to 54 | Psoriasis | Both | 2.46 (2.42, 2.51) |
| Algeria | 50 to 54 | Psoriasis | Female | 2.31 (2.26, 2.37) |
| Algeria | 50 to 54 | Psoriasis | Male | 2.64 (2.57, 2.71) |
| Algeria | 55 to 59 | Psoriasis | Both | 2.59 (2.54, 2.63) |
| Algeria | 55 to 59 | Psoriasis | Female | 2.41 (2.34, 2.47) |
| Algeria | 55 to 59 | Psoriasis | Male | 2.79 (2.71, 2.87) |
| Algeria | 60 to 64 | Psoriasis | Both | 2.57 (2.52, 2.62) |
| Algeria | 60 to 64 | Psoriasis | Female | 2.36 (2.30, 2.43) |
| Algeria | 60 to 64 | Psoriasis | Male | 2.81 (2.73, 2.89) |
| Algeria | 65 to 69 | Psoriasis | Both | 2.43 (2.38, 2.49) |

| | | | | |
|---|---|---|---|---|
| Algeria | 65 to 69 | Psoriasis | Female | 2.20 (2.14, 2.27) |
| Algeria | 65 to 69 | Psoriasis | Male | 2.70 (2.61, 2.78) |
| Algeria | 0 to 4 | RHD | Both | 0.17 (0.16, 0.18) |
| Algeria | 0 to 4 | RHD | Female | 0.17 (0.16, 0.18) |
| Algeria | 0 to 4 | RHD | Male | 0.17 (0.16, 0.18) |
| Algeria | 10 to 14 | RHD | Both | 1.93 (1.86, 1.99) |
| Algeria | 10 to 14 | RHD | Female | 1.92 (1.85, 1.99) |
| Algeria | 10 to 14 | RHD | Male | 1.93 (1.87, 2.00) |
| Algeria | 15 to 19 | RHD | Both | 2.84 (2.75, 2.94) |
| Algeria | 15 to 19 | RHD | Female | 2.84 (2.74, 2.94) |
| Algeria | 15 to 19 | RHD | Male | 2.84 (2.75, 2.94) |
| Algeria | 20 to 24 | RHD | Both | 3.62 (3.50, 3.75) |
| Algeria | 20 to 24 | RHD | Female | 3.66 (3.53, 3.79) |
| Algeria | 20 to 24 | RHD | Male | 3.58 (3.46, 3.70) |
| Algeria | 25 to 29 | RHD | Both | 4.17 (4.03, 4.32) |
| Algeria | 25 to 29 | RHD | Female | 4.25 (4.10, 4.41) |
| Algeria | 25 to 29 | RHD | Male | 4.08 (3.95, 4.23) |
| Algeria | 30 to 34 | RHD | Both | 4.52 (4.36, 4.69) |
| Algeria | 30 to 34 | RHD | Female | 4.64 (4.47, 4.82) |
| Algeria | 30 to 34 | RHD | Male | 4.39 (4.24, 4.55) |
| Algeria | 35 to 39 | RHD | Both | 4.69 (4.51, 4.87) |
| Algeria | 35 to 39 | RHD | Female | 4.84 (4.65, 5.03) |
| Algeria | 35 to 39 | RHD | Male | 4.53 (4.36, 4.70) |
| Algeria | 40 to 44 | RHD | Both | 4.64 (4.46, 4.83) |
| Algeria | 40 to 44 | RHD | Female | 4.81 (4.61, 5.01) |
| Algeria | 40 to 44 | RHD | Male | 4.46 (4.28, 4.64) |
| Algeria | 45 to 49 | RHD | Both | 4.23 (4.05, 4.42) |
| Algeria | 45 to 49 | RHD | Female | 4.42 (4.22, 4.62) |
| Algeria | 45 to 49 | RHD | Male | 4.03 (3.86, 4.20) |
| Algeria | 5 to 9 | RHD | Both | 1.00 (1.00, 1.00) |
| Algeria | 5 to 9 | RHD | Female | 1.00 (1.00, 1.00) |
| Algeria | 5 to 9 | RHD | Male | 1.00 (1.00, 1.00) |
| Algeria | 50 to 54 | RHD | Both | 3.55 (3.39, 3.73) |

| | | | | |
|---|---|---|---|---|
| Algeria | 50 to 54 | RHD | Female | 3.75 (3.57, 3.94) |
| Algeria | 50 to 54 | RHD | Male | 3.35 (3.20, 3.50) |
| Algeria | 55 to 59 | RHD | Both | 2.91 (2.76, 3.07) |
| Algeria | 55 to 59 | RHD | Female | 3.11 (2.95, 3.28) |
| Algeria | 55 to 59 | RHD | Male | 2.71 (2.57, 2.85) |
| Algeria | 60 to 64 | RHD | Both | 2.36 (2.22, 2.51) |
| Algeria | 60 to 64 | RHD | Female | 2.55 (2.39, 2.71) |
| Algeria | 60 to 64 | RHD | Male | 2.17 (2.04, 2.30) |
| Algeria | 65 to 69 | RHD | Both | 1.89 (1.76, 2.03) |
| Algeria | 65 to 69 | RHD | Female | 2.06 (1.91, 2.21) |
| Algeria | 65 to 69 | RHD | Male | 1.72 (1.60, 1.85) |
| Algeria | 10 to 14 | RA | Both | 7.42 (6.33, 8.69) |
| Algeria | 10 to 14 | RA | Female | 7.63 (6.31, 9.24) |
| Algeria | 10 to 14 | RA | Male | 7.12 (5.35, 9.46) |
| Algeria | 15 to 19 | RA | Both | 22.20 (19.02, 25.93) |
| Algeria | 15 to 19 | RA | Female | 23.59 (19.57, 28.45) |
| Algeria | 15 to 19 | RA | Male | 19.47 (14.72, 25.75) |
| Algeria | 20 to 24 | RA | Both | 45.45 (38.94, 53.04) |
| Algeria | 20 to 24 | RA | Female | 48.98 (40.65, 59.02) |
| Algeria | 20 to 24 | RA | Male | 37.82 (28.62, 49.98) |
| Algeria | 25 to 29 | RA | Both | 73.66 (63.11, 85.97) |
| Algeria | 25 to 29 | RA | Female | 80.11 (66.48, 96.54) |
| Algeria | 25 to 29 | RA | Male | 59.67 (45.14, 78.87) |
| Algeria | 30 to 34 | RA | Both | 106.90 (91.56, 124.80) |
| Algeria | 30 to 34 | RA | Female | 116.68 (96.79, 140.65) |
| Algeria | 30 to 34 | RA | Male | 84.94 (64.21, 112.36) |
| Algeria | 35 to 39 | RA | Both | 147.33 (126.14, 172.08) |
| Algeria | 35 to 39 | RA | Female | 160.69 (133.24, 193.79) |
| Algeria | 35 to 39 | RA | Male | 115.93 (87.56, 153.50) |
| Algeria | 40 to 44 | RA | Both | 194.38 (166.38, 227.10) |
| Algeria | 40 to 44 | RA | Female | 211.15 (175.03, 254.73) |
| Algeria | 40 to 44 | RA | Male | 153.14 (115.58, 202.90) |
| Algeria | 45 to 49 | RA | Both | 242.75 (207.70, 283.71) |

| | | | | |
|---|---|---|---|---|
| Algeria | 45 to 49 | RA | Female | 261.70 (216.84, 315.85) |
| Algeria | 45 to 49 | RA | Male | 196.40 (148.12, 260.41) |
| Algeria | 5 to 9 | RA | Both | 1.00 (1.00, 1.00) |
| Algeria | 5 to 9 | RA | Female | 1.00 (1.00, 1.00) |
| Algeria | 5 to 9 | RA | Male | 1.00 (1.00, 1.00) |
| Algeria | 50 to 54 | RA | Both | 290.10 (248.10, 339.21) |
| Algeria | 50 to 54 | RA | Female | 309.13 (256.01, 373.27) |
| Algeria | 50 to 54 | RA | Male | 244.14 (183.95, 324.00) |
| Algeria | 55 to 59 | RA | Both | 330.13 (282.19, 386.21) |
| Algeria | 55 to 59 | RA | Female | 347.54 (287.65, 419.91) |
| Algeria | 55 to 59 | RA | Male | 291.16 (219.16, 386.82) |
| Algeria | 60 to 64 | RA | Both | 361.07 (308.45, 422.65) |
| Algeria | 60 to 64 | RA | Female | 374.18 (309.48, 452.40) |
| Algeria | 60 to 64 | RA | Male | 334.67 (251.63, 445.12) |
| Algeria | 65 to 69 | RA | Both | 370.82 (316.53, 434.41) |
| Algeria | 65 to 69 | RA | Female | 377.70 (312.10, 457.08) |
| Algeria | 65 to 69 | RA | Male | 361.02 (271.04, 480.85) |
| American Samoa | 0 to 4 | AA | Both | 0.74 (0.44, 1.26) |
| American Samoa | 0 to 4 | AA | Female | 0.73 (0.37, 1.44) |
| American Samoa | 0 to 4 | AA | Male | 0.84 (0.35, 2.01) |
| American Samoa | 10 to 14 | AA | Both | 1.31 (0.82, 2.09) |
| American Samoa | 10 to 14 | AA | Female | 1.34 (0.74, 2.41) |
| American Samoa | 10 to 14 | AA | Male | 1.49 (0.69, 3.21) |
| American Samoa | 15 to 19 | AA | Both | 2.39 (1.53, 3.75) |
| American Samoa | 15 to 19 | AA | Female | 2.27 (1.29, 4.01) |
| American Samoa | 15 to 19 | AA | Male | 2.44 (1.14, 5.20) |
| American Samoa | 20 to 24 | AA | Both | 3.72 (2.36, 5.87) |
| American Samoa | 20 to 24 | AA | Female | 3.65 (2.06, 6.48) |
| American Samoa | 20 to 24 | AA | Male | 3.87 (1.80, 8.30) |
| American Samoa | 25 to 29 | AA | Both | 5.13 (3.22, 8.19) |
| American Samoa | 25 to 29 | AA | Female | 4.96 (2.76, 8.92) |
| American Samoa | 25 to 29 | AA | Male | 5.40 (2.47, 11.83) |
| American Samoa | 30 to 34 | AA | Both | 5.58 (3.45, 9.04) |

| | | | | |
|---|---|---|---|---|
| American Samoa | 30 to 34 | AA | Female | 5.26 (2.86, 9.65) |
| American Samoa | 30 to 34 | AA | Male | 5.70 (2.54, 12.81) |
| American Samoa | 35 to 39 | AA | Both | 5.26 (3.19, 8.68) |
| American Samoa | 35 to 39 | AA | Female | 5.00 (2.66, 9.38) |
| American Samoa | 35 to 39 | AA | Male | 5.44 (2.34, 12.63) |
| American Samoa | 40 to 44 | AA | Both | 4.50 (2.66, 7.61) |
| American Samoa | 40 to 44 | AA | Female | 4.05 (2.09, 7.86) |
| American Samoa | 40 to 44 | AA | Male | 4.48 (1.85, 10.83) |
| American Samoa | 45 to 49 | AA | Both | 3.83 (2.19, 6.67) |
| American Samoa | 45 to 49 | AA | Female | 3.70 (1.84, 7.43) |
| American Samoa | 45 to 49 | AA | Male | 3.91 (1.54, 9.93) |
| American Samoa | 5 to 9 | AA | Both | 1.00 (1.00, 1.00) |
| American Samoa | 5 to 9 | AA | Female | 1.00 (1.00, 1.00) |
| American Samoa | 5 to 9 | AA | Male | 1.00 (1.00, 1.00) |
| American Samoa | 50 to 54 | AA | Both | 3.48 (1.92, 6.32) |
| American Samoa | 50 to 54 | AA | Female | 3.25 (1.54, 6.88) |
| American Samoa | 50 to 54 | AA | Male | 3.55 (1.30, 9.71) |
| American Samoa | 55 to 59 | AA | Both | 3.39 (1.77, 6.47) |
| American Samoa | 55 to 59 | AA | Female | 3.04 (1.34, 6.89) |
| American Samoa | 55 to 59 | AA | Male | 3.32 (1.11, 9.94) |
| American Samoa | 60 to 64 | AA | Both | 3.49 (1.70, 7.15) |
| American Samoa | 60 to 64 | AA | Female | 3.16 (1.28, 7.79) |
| American Samoa | 60 to 64 | AA | Male | 3.58 (1.09, 11.74) |
| American Samoa | 65 to 69 | AA | Both | 3.07 (1.33, 7.09) |
| American Samoa | 65 to 69 | AA | Female | 2.86 (1.02, 8.02) |
| American Samoa | 65 to 69 | AA | Male | 3.63 (0.92, 14.36) |
| American Samoa | 0 to 4 | IBD | Both | 0.93 (0.02, 41.56) |
| American Samoa | 0 to 4 | IBD | Female | 0.92 (0.02, 41.38) |
| American Samoa | 0 to 4 | IBD | Male | 0.93 (0.02, 41.73) |
| American Samoa | 10 to 14 | IBD | Both | 1.08 (0.03, 44.26) |
| American Samoa | 10 to 14 | IBD | Female | 1.08 (0.03, 44.09) |
| American Samoa | 10 to 14 | IBD | Male | 1.08 (0.03, 44.42) |
| American Samoa | 15 to 19 | IBD | Both | 1.25 (0.03, 59.88) |

| | | | | |
|---|---|---|---|---|
| American Samoa | 15 to 19 | IBD | Female | 1.23 (0.03, 59.01) |
| American Samoa | 15 to 19 | IBD | Male | 1.26 (0.03, 60.71) |
| American Samoa | 20 to 24 | IBD | Both | 1.59 (0.03, 92.17) |
| American Samoa | 20 to 24 | IBD | Female | 1.53 (0.03, 88.84) |
| American Samoa | 20 to 24 | IBD | Male | 1.64 (0.03, 95.47) |
| American Samoa | 25 to 29 | IBD | Both | 1.84 (0.03, 133.47) |
| American Samoa | 25 to 29 | IBD | Female | 1.75 (0.02, 127.16) |
| American Samoa | 25 to 29 | IBD | Male | 1.93 (0.03, 139.85) |
| American Samoa | 30 to 34 | IBD | Both | 1.95 (0.02, 182.51) |
| American Samoa | 30 to 34 | IBD | Female | 1.85 (0.02, 172.66) |
| American Samoa | 30 to 34 | IBD | Male | 2.06 (0.02, 192.56) |
| American Samoa | 35 to 39 | IBD | Both | 2.08 (0.02, 251.86) |
| American Samoa | 35 to 39 | IBD | Female | 1.97 (0.02, 238.25) |
| American Samoa | 35 to 39 | IBD | Male | 2.19 (0.02, 265.70) |
| American Samoa | 40 to 44 | IBD | Both | 2.23 (0.01, 334.85) |
| American Samoa | 40 to 44 | IBD | Female | 2.11 (0.01, 317.69) |
| American Samoa | 40 to 44 | IBD | Male | 2.34 (0.02, 352.29) |
| American Samoa | 45 to 49 | IBD | Both | 2.42 (0.01, 449.87) |
| American Samoa | 45 to 49 | IBD | Female | 2.29 (0.01, 426.23) |
| American Samoa | 45 to 49 | IBD | Male | 2.54 (0.01, 473.76) |
| American Samoa | 5 to 9 | IBD | Both | 1.00 (1.00, 1.00) |
| American Samoa | 5 to 9 | IBD | Female | 1.00 (1.00, 1.00) |
| American Samoa | 5 to 9 | IBD | Male | 1.00 (1.00, 1.00) |
| American Samoa | 50 to 54 | IBD | Both | 2.68 (0.01, 615.76) |
| American Samoa | 50 to 54 | IBD | Female | 2.54 (0.01, 582.49) |
| American Samoa | 50 to 54 | IBD | Male | 2.83 (0.01, 649.81) |
| American Samoa | 55 to 59 | IBD | Both | 3.03 (0.01, 853.51) |
| American Samoa | 55 to 59 | IBD | Female | 2.84 (0.01, 799.25) |
| American Samoa | 55 to 59 | IBD | Male | 3.24 (0.01, 910.11) |
| American Samoa | 60 to 64 | IBD | Both | 3.63 (0.01, 1255.36) |
| American Samoa | 60 to 64 | IBD | Female | 3.32 (0.01, 1149.25) |
| American Samoa | 60 to 64 | IBD | Male | 3.95 (0.01, 1366.24) |
| American Samoa | 65 to 69 | IBD | Both | 4.49 (0.01, 1996.67) |

| | | | | |
|---|---|---|---|---|
| American Samoa | 65 to 69 | IBD | Female | 4.00 (0.01, 1776.54) |
| American Samoa | 65 to 69 | IBD | Male | 5.02 (0.01, 2231.86) |
| American Samoa | 10 to 14 | MS | Both | 1.08 (0.02, 48.18) |
| American Samoa | 10 to 14 | MS | Female | 1.07 (0.02, 48.00) |
| American Samoa | 10 to 14 | MS | Male | 1.08 (0.02, 48.36) |
| American Samoa | 15 to 19 | MS | Both | 1.24 (0.02, 66.74) |
| American Samoa | 15 to 19 | MS | Female | 1.22 (0.02, 65.78) |
| American Samoa | 15 to 19 | MS | Male | 1.25 (0.02, 67.68) |
| American Samoa | 20 to 24 | MS | Both | 1.57 (0.02, 102.67) |
| American Samoa | 20 to 24 | MS | Female | 1.51 (0.02, 98.94) |
| American Samoa | 20 to 24 | MS | Male | 1.62 (0.02, 106.34) |
| American Samoa | 25 to 29 | MS | Both | 1.82 (0.02, 146.78) |
| American Samoa | 25 to 29 | MS | Female | 1.73 (0.02, 139.82) |
| American Samoa | 25 to 29 | MS | Male | 1.90 (0.02, 153.80) |
| American Samoa | 30 to 34 | MS | Both | 1.93 (0.02, 197.22) |
| American Samoa | 30 to 34 | MS | Female | 1.83 (0.02, 186.55) |
| American Samoa | 30 to 34 | MS | Male | 2.04 (0.02, 208.08) |
| American Samoa | 35 to 39 | MS | Both | 2.06 (0.02, 272.23) |
| American Samoa | 35 to 39 | MS | Female | 1.95 (0.01, 257.49) |
| American Samoa | 35 to 39 | MS | Male | 2.17 (0.02, 287.19) |
| American Samoa | 40 to 44 | MS | Both | 2.20 (0.01, 360.61) |
| American Samoa | 40 to 44 | MS | Female | 2.09 (0.01, 342.09) |
| American Samoa | 40 to 44 | MS | Male | 2.32 (0.01, 379.41) |
| American Samoa | 45 to 49 | MS | Both | 2.39 (0.01, 483.35) |
| American Samoa | 45 to 49 | MS | Female | 2.26 (0.01, 457.88) |
| American Samoa | 45 to 49 | MS | Male | 2.52 (0.01, 509.02) |
| American Samoa | 5 to 9 | MS | Both | 1.00 (1.00, 1.00) |
| American Samoa | 5 to 9 | MS | Female | 1.00 (1.00, 1.00) |
| American Samoa | 5 to 9 | MS | Male | 1.00 (1.00, 1.00) |
| American Samoa | 50 to 54 | MS | Both | 2.65 (0.01, 659.34) |
| American Samoa | 50 to 54 | MS | Female | 2.51 (0.01, 623.63) |
| American Samoa | 50 to 54 | MS | Male | 2.80 (0.01, 695.81) |
| American Samoa | 55 to 59 | MS | Both | 3.00 (0.01, 909.79) |

| | | | | |
|---|---|---|---|---|
| American Samoa | 55 to 59 | MS | Female | 2.81 (0.01, 851.82) |
| American Samoa | 55 to 59 | MS | Male | 3.20 (0.01, 970.13) |
| American Samoa | 60 to 64 | MS | Both | 3.59 (0.01, 1330.49) |
| American Samoa | 60 to 64 | MS | Female | 3.29 (0.01, 1217.87) |
| American Samoa | 60 to 64 | MS | Male | 3.91 (0.01, 1448.02) |
| American Samoa | 65 to 69 | MS | Both | 4.45 (0.01, 2101.71) |
| American Samoa | 65 to 69 | MS | Female | 3.96 (0.01, 1869.77) |
| American Samoa | 65 to 69 | MS | Male | 4.98 (0.01, 2349.30) |
| American Samoa | 0 to 4 | Psoriasis | Both | 0.47 (0.25, 0.89) |
| American Samoa | 0 to 4 | Psoriasis | Female | 0.53 (0.23, 1.24) |
| American Samoa | 0 to 4 | Psoriasis | Male | 0.50 (0.21, 1.24) |
| American Samoa | 10 to 14 | Psoriasis | Both | 1.32 (0.83, 2.12) |
| American Samoa | 10 to 14 | Psoriasis | Female | 1.43 (0.75, 2.71) |
| American Samoa | 10 to 14 | Psoriasis | Male | 1.15 (0.57, 2.32) |
| American Samoa | 15 to 19 | Psoriasis | Both | 1.61 (1.00, 2.61) |
| American Samoa | 15 to 19 | Psoriasis | Female | 1.85 (0.97, 3.54) |
| American Samoa | 15 to 19 | Psoriasis | Male | 1.27 (0.62, 2.60) |
| American Samoa | 20 to 24 | Psoriasis | Both | 1.75 (1.05, 2.94) |
| American Samoa | 20 to 24 | Psoriasis | Female | 1.98 (0.99, 3.98) |
| American Samoa | 20 to 24 | Psoriasis | Male | 1.58 (0.74, 3.37) |
| American Samoa | 25 to 29 | Psoriasis | Both | 2.06 (1.20, 3.53) |
| American Samoa | 25 to 29 | Psoriasis | Female | 2.06 (0.98, 4.34) |
| American Samoa | 25 to 29 | Psoriasis | Male | 2.05 (0.94, 4.49) |
| American Samoa | 30 to 34 | Psoriasis | Both | 2.23 (1.27, 3.90) |
| American Samoa | 30 to 34 | Psoriasis | Female | 2.27 (1.05, 4.89) |
| American Samoa | 30 to 34 | Psoriasis | Male | 2.33 (1.03, 5.28) |
| American Samoa | 35 to 39 | Psoriasis | Both | 2.74 (1.54, 4.86) |
| American Samoa | 35 to 39 | Psoriasis | Female | 2.63 (1.20, 5.79) |
| American Samoa | 35 to 39 | Psoriasis | Male | 3.08 (1.35, 7.06) |
| American Samoa | 40 to 44 | Psoriasis | Both | 3.30 (1.84, 5.92) |
| American Samoa | 40 to 44 | Psoriasis | Female | 2.96 (1.31, 6.68) |
| American Samoa | 40 to 44 | Psoriasis | Male | 3.82 (1.64, 8.92) |
| American Samoa | 45 to 49 | Psoriasis | Both | 3.64 (1.99, 6.65) |

| | | | | |
|---|---|---|---|---|
| American Samoa | 45 to 49 | Psoriasis | Female | 3.02 (1.29, 7.04) |
| American Samoa | 45 to 49 | Psoriasis | Male | 4.39 (1.84, 10.45) |
| American Samoa | 5 to 9 | Psoriasis | Both | 1.00 (1.00, 1.00) |
| American Samoa | 5 to 9 | Psoriasis | Female | 1.00 (1.00, 1.00) |
| American Samoa | 5 to 9 | Psoriasis | Male | 1.00 (1.00, 1.00) |
| American Samoa | 50 to 54 | Psoriasis | Both | 3.94 (2.11, 7.37) |
| American Samoa | 50 to 54 | Psoriasis | Female | 3.01 (1.24, 7.34) |
| American Samoa | 50 to 54 | Psoriasis | Male | 4.88 (2.00, 11.93) |
| American Samoa | 55 to 59 | Psoriasis | Both | 3.97 (2.06, 7.66) |
| American Samoa | 55 to 59 | Psoriasis | Female | 3.11 (1.21, 7.98) |
| American Samoa | 55 to 59 | Psoriasis | Male | 5.19 (2.05, 13.16) |
| American Samoa | 60 to 64 | Psoriasis | Both | 4.02 (2.00, 8.08) |
| American Samoa | 60 to 64 | Psoriasis | Female | 3.14 (1.13, 8.71) |
| American Samoa | 60 to 64 | Psoriasis | Male | 4.88 (1.82, 13.09) |
| American Samoa | 65 to 69 | Psoriasis | Both | 4.00 (1.86, 8.59) |
| American Samoa | 65 to 69 | Psoriasis | Female | 3.51 (1.15, 10.70) |
| American Samoa | 65 to 69 | Psoriasis | Male | 4.68 (1.61, 13.57) |
| American Samoa | 0 to 4 | RHD | Both | 0.19 (0.14, 0.27) |
| American Samoa | 0 to 4 | RHD | Female | 0.21 (0.13, 0.33) |
| American Samoa | 0 to 4 | RHD | Male | 0.19 (0.12, 0.31) |
| American Samoa | 10 to 14 | RHD | Both | 1.40 (1.18, 1.68) |
| American Samoa | 10 to 14 | RHD | Female | 1.42 (1.11, 1.82) |
| American Samoa | 10 to 14 | RHD | Male | 1.40 (1.09, 1.81) |
| American Samoa | 15 to 19 | RHD | Both | 1.51 (1.25, 1.82) |
| American Samoa | 15 to 19 | RHD | Female | 1.52 (1.17, 1.98) |
| American Samoa | 15 to 19 | RHD | Male | 1.50 (1.15, 1.97) |
| American Samoa | 20 to 24 | RHD | Both | 1.53 (1.24, 1.88) |
| American Samoa | 20 to 24 | RHD | Female | 1.56 (1.17, 2.07) |
| American Samoa | 20 to 24 | RHD | Male | 1.51 (1.12, 2.04) |
| American Samoa | 25 to 29 | RHD | Both | 1.52 (1.21, 1.90) |
| American Samoa | 25 to 29 | RHD | Female | 1.57 (1.15, 2.14) |
| American Samoa | 25 to 29 | RHD | Male | 1.48 (1.06, 2.05) |
| American Samoa | 30 to 34 | RHD | Both | 1.55 (1.22, 1.97) |

| | | | | |
|---|---|---|---|---|
| American Samoa | 30 to 34 | RHD | Female | 1.61 (1.16, 2.23) |
| American Samoa | 30 to 34 | RHD | Male | 1.51 (1.06, 2.13) |
| American Samoa | 35 to 39 | RHD | Both | 1.57 (1.22, 2.02) |
| American Samoa | 35 to 39 | RHD | Female | 1.62 (1.15, 2.29) |
| American Samoa | 35 to 39 | RHD | Male | 1.54 (1.07, 2.24) |
| American Samoa | 40 to 44 | RHD | Both | 1.58 (1.21, 2.06) |
| American Samoa | 40 to 44 | RHD | Female | 1.63 (1.13, 2.34) |
| American Samoa | 40 to 44 | RHD | Male | 1.52 (1.02, 2.25) |
| American Samoa | 45 to 49 | RHD | Both | 1.48 (1.11, 1.98) |
| American Samoa | 45 to 49 | RHD | Female | 1.52 (1.02, 2.25) |
| American Samoa | 45 to 49 | RHD | Male | 1.43 (0.94, 2.20) |
| American Samoa | 5 to 9 | RHD | Both | 1.00 (1.00, 1.00) |
| American Samoa | 5 to 9 | RHD | Female | 1.00 (1.00, 1.00) |
| American Samoa | 5 to 9 | RHD | Male | 1.00 (1.00, 1.00) |
| American Samoa | 50 to 54 | RHD | Both | 1.26 (0.92, 1.75) |
| American Samoa | 50 to 54 | RHD | Female | 1.30 (0.84, 2.02) |
| American Samoa | 50 to 54 | RHD | Male | 1.21 (0.75, 1.94) |
| American Samoa | 55 to 59 | RHD | Both | 1.06 (0.73, 1.53) |
| American Samoa | 55 to 59 | RHD | Female | 1.08 (0.65, 1.80) |
| American Samoa | 55 to 59 | RHD | Male | 0.99 (0.57, 1.72) |
| American Samoa | 60 to 64 | RHD | Both | 0.90 (0.58, 1.41) |
| American Samoa | 60 to 64 | RHD | Female | 0.90 (0.49, 1.65) |
| American Samoa | 60 to 64 | RHD | Male | 0.81 (0.42, 1.59) |
| American Samoa | 65 to 69 | RHD | Both | 0.73 (0.41, 1.28) |
| American Samoa | 65 to 69 | RHD | Female | 0.71 (0.33, 1.51) |
| American Samoa | 65 to 69 | RHD | Male | 0.71 (0.31, 1.62) |
| American Samoa | 10 to 14 | RA | Both | 10.75 (0.60, 193.07) |
| American Samoa | 10 to 14 | RA | Female | 1.07 (0.02, 46.05) |
| American Samoa | 10 to 14 | RA | Male | 1.14 (0.03, 49.23) |
| American Samoa | 15 to 19 | RA | Both | 24.39 (1.39, 428.29) |
| American Samoa | 15 to 19 | RA | Female | 12.11 (0.57, 257.24) |
| American Samoa | 15 to 19 | RA | Male | 13.07 (0.61, 279.47) |
| American Samoa | 20 to 24 | RA | Both | 45.12 (2.57, 792.11) |

| | | | | |
|---|---|---|---|---|
| American Samoa | 20 to 24 | RA | Female | 29.20 (1.39, 613.59) |
| American Samoa | 20 to 24 | RA | Male | 16.74 (0.75, 375.18) |
| American Samoa | 25 to 29 | RA | Both | 61.05 (3.45, 1082.05) |
| American Samoa | 25 to 29 | RA | Female | 39.97 (1.87, 855.77) |
| American Samoa | 25 to 29 | RA | Male | 19.35 (0.83, 450.01) |
| American Samoa | 30 to 34 | RA | Both | 86.21 (4.85, 1533.57) |
| American Samoa | 30 to 34 | RA | Female | 55.37 (2.58, 1190.20) |
| American Samoa | 30 to 34 | RA | Male | 30.64 (1.31, 717.51) |
| American Samoa | 35 to 39 | RA | Both | 104.10 (5.81, 1864.12) |
| American Samoa | 35 to 39 | RA | Female | 71.54 (3.30, 1550.13) |
| American Samoa | 35 to 39 | RA | Male | 36.15 (1.51, 863.93) |
| American Samoa | 40 to 44 | RA | Both | 117.63 (6.53, 2117.90) |
| American Samoa | 40 to 44 | RA | Female | 83.52 (3.82, 1824.78) |
| American Samoa | 40 to 44 | RA | Male | 42.95 (1.77, 1042.64) |
| American Samoa | 45 to 49 | RA | Both | 135.64 (7.48, 2459.50) |
| American Samoa | 45 to 49 | RA | Female | 90.47 (4.10, 1996.67) |
| American Samoa | 45 to 49 | RA | Male | 51.87 (2.08, 1290.94) |
| American Samoa | 5 to 9 | RA | Both | 1.00 (1.00, 1.00) |
| American Samoa | 5 to 9 | RA | Female | 1.00 (1.00, 1.00) |
| American Samoa | 5 to 9 | RA | Male | 1.00 (1.00, 1.00) |
| American Samoa | 50 to 54 | RA | Both | 142.77 (7.80, 2614.76) |
| American Samoa | 50 to 54 | RA | Female | 98.36 (4.39, 2203.03) |
| American Samoa | 50 to 54 | RA | Male | 58.98 (2.31, 1508.10) |
| American Samoa | 55 to 59 | RA | Both | 155.54 (8.39, 2884.93) |
| American Samoa | 55 to 59 | RA | Female | 103.41 (4.52, 2365.24) |
| American Samoa | 55 to 59 | RA | Male | 61.02 (2.29, 1626.88) |
| American Samoa | 60 to 64 | RA | Both | 154.04 (8.15, 2912.45) |
| American Samoa | 60 to 64 | RA | Female | 108.69 (4.63, 2551.99) |
| American Samoa | 60 to 64 | RA | Male | 60.01 (2.12, 1700.38) |
| American Samoa | 65 to 69 | RA | Both | 149.95 (7.67, 2932.98) |
| American Samoa | 65 to 69 | RA | Female | 104.83 (4.26, 2582.09) |
| American Samoa | 65 to 69 | RA | Male | 78.53 (2.68, 2303.42) |
| Andorra | 0 to 4 | AA | Both | 0.74 (0.36, 1.51) |

| | | | | |
|---|---|---|---|---|
| Andorra | 0 to 4 | AA | Female | 0.63 (0.22, 1.85) |
| Andorra | 0 to 4 | AA | Male | 0.55 (0.19, 1.57) |
| Andorra | 10 to 14 | AA | Both | 1.33 (0.73, 2.41) |
| Andorra | 10 to 14 | AA | Female | 1.18 (0.49, 2.88) |
| Andorra | 10 to 14 | AA | Male | 0.97 (0.42, 2.23) |
| Andorra | 15 to 19 | AA | Both | 2.33 (1.32, 4.11) |
| Andorra | 15 to 19 | AA | Female | 2.25 (0.97, 5.21) |
| Andorra | 15 to 19 | AA | Male | 1.78 (0.83, 3.83) |
| Andorra | 20 to 24 | AA | Both | 3.82 (2.18, 6.69) |
| Andorra | 20 to 24 | AA | Female | 3.28 (1.42, 7.58) |
| Andorra | 20 to 24 | AA | Male | 3.05 (1.46, 6.40) |
| Andorra | 25 to 29 | AA | Both | 5.29 (3.03, 9.24) |
| Andorra | 25 to 29 | AA | Female | 4.70 (2.03, 10.86) |
| Andorra | 25 to 29 | AA | Male | 4.33 (2.07, 9.04) |
| Andorra | 30 to 34 | AA | Both | 5.53 (3.15, 9.72) |
| Andorra | 30 to 34 | AA | Female | 5.50 (2.37, 12.79) |
| Andorra | 30 to 34 | AA | Male | 4.13 (1.95, 8.72) |
| Andorra | 35 to 39 | AA | Both | 5.13 (2.89, 9.09) |
| Andorra | 35 to 39 | AA | Female | 5.57 (2.37, 13.07) |
| Andorra | 35 to 39 | AA | Male | 3.34 (1.55, 7.16) |
| Andorra | 40 to 44 | AA | Both | 4.35 (2.43, 7.79) |
| Andorra | 40 to 44 | AA | Female | 5.17 (2.18, 12.27) |
| Andorra | 40 to 44 | AA | Male | 2.60 (1.19, 5.68) |
| Andorra | 45 to 49 | AA | Both | 3.70 (2.04, 6.72) |
| Andorra | 45 to 49 | AA | Female | 4.68 (1.95, 11.26) |
| Andorra | 45 to 49 | AA | Male | 2.00 (0.89, 4.49) |
| Andorra | 5 to 9 | AA | Both | 1.00 (1.00, 1.00) |
| Andorra | 5 to 9 | AA | Female | 1.00 (1.00, 1.00) |
| Andorra | 5 to 9 | AA | Male | 1.00 (1.00, 1.00) |
| Andorra | 50 to 54 | AA | Both | 3.32 (1.80, 6.12) |
| Andorra | 50 to 54 | AA | Female | 4.55 (1.86, 11.13) |
| Andorra | 50 to 54 | AA | Male | 1.68 (0.72, 3.91) |
| Andorra | 55 to 59 | AA | Both | 3.29 (1.75, 6.18) |

| | | | | |
|---|---|---|---|---|
| Andorra | 55 to 59 | AA | Female | 4.62 (1.86, 11.53) |
| Andorra | 55 to 59 | AA | Male | 1.33 (0.54, 3.29) |
| Andorra | 60 to 64 | AA | Both | 3.34 (1.73, 6.46) |
| Andorra | 60 to 64 | AA | Female | 4.90 (1.92, 12.49) |
| Andorra | 60 to 64 | AA | Male | 1.25 (0.47, 3.35) |
| Andorra | 65 to 69 | AA | Both | 3.07 (1.52, 6.19) |
| Andorra | 65 to 69 | AA | Female | 4.76 (1.80, 12.59) |
| Andorra | 65 to 69 | AA | Male | 0.98 (0.33, 2.98) |
| Andorra | 0 to 4 | IBD | Both | 1.10 (0.03, 47.60) |
| Andorra | 0 to 4 | IBD | Female | 1.13 (0.03, 48.75) |
| Andorra | 0 to 4 | IBD | Male | 1.09 (0.03, 47.11) |
| Andorra | 10 to 14 | IBD | Both | 0.97 (0.02, 38.17) |
| Andorra | 10 to 14 | IBD | Female | 0.95 (0.02, 37.61) |
| Andorra | 10 to 14 | IBD | Male | 0.98 (0.02, 38.67) |
| Andorra | 15 to 19 | IBD | Both | 9.29 (0.51, 169.46) |
| Andorra | 15 to 19 | IBD | Female | 0.91 (0.02, 39.94) |
| Andorra | 15 to 19 | IBD | Male | 0.94 (0.02, 41.22) |
| Andorra | 20 to 24 | IBD | Both | 19.37 (1.08, 346.11) |
| Andorra | 20 to 24 | IBD | Female | 11.16 (0.53, 233.60) |
| Andorra | 20 to 24 | IBD | Male | 7.93 (0.37, 169.86) |
| Andorra | 25 to 29 | IBD | Both | 35.05 (1.97, 622.37) |
| Andorra | 25 to 29 | IBD | Female | 18.66 (0.89, 389.31) |
| Andorra | 25 to 29 | IBD | Male | 15.33 (0.72, 328.27) |
| Andorra | 30 to 34 | IBD | Both | 49.10 (2.76, 874.47) |
| Andorra | 30 to 34 | IBD | Female | 26.57 (1.26, 558.15) |
| Andorra | 30 to 34 | IBD | Male | 21.17 (0.98, 457.94) |
| Andorra | 35 to 39 | IBD | Both | 64.55 (3.61, 1152.99) |
| Andorra | 35 to 39 | IBD | Female | 33.03 (1.56, 698.05) |
| Andorra | 35 to 39 | IBD | Male | 28.10 (1.29, 611.80) |
| Andorra | 40 to 44 | IBD | Both | 68.65 (3.83, 1229.97) |
| Andorra | 40 to 44 | IBD | Female | 33.26 (1.56, 707.00) |
| Andorra | 40 to 44 | IBD | Male | 33.58 (1.54, 733.86) |
| Andorra | 45 to 49 | IBD | Both | 77.71 (4.32, 1396.65) |

| | | | | |
|---|---|---|---|---|
| Andorra | 45 to 49 | IBD | Female | 36.34 (1.70, 777.31) |
| Andorra | 45 to 49 | IBD | Male | 40.73 (1.86, 894.03) |
| Andorra | 5 to 9 | IBD | Both | 1.00 (1.00, 1.00) |
| Andorra | 5 to 9 | IBD | Female | 1.00 (1.00, 1.00) |
| Andorra | 5 to 9 | IBD | Male | 1.00 (1.00, 1.00) |
| Andorra | 50 to 54 | IBD | Both | 86.05 (4.77, 1552.64) |
| Andorra | 50 to 54 | IBD | Female | 37.42 (1.73, 807.43) |
| Andorra | 50 to 54 | IBD | Male | 44.14 (2.00, 975.81) |
| Andorra | 55 to 59 | IBD | Both | 90.02 (4.96, 1633.08) |
| Andorra | 55 to 59 | IBD | Female | 39.33 (1.80, 857.87) |
| Andorra | 55 to 59 | IBD | Male | 47.22 (2.12, 1053.23) |
| Andorra | 60 to 64 | IBD | Both | 91.34 (5.00, 1668.08) |
| Andorra | 60 to 64 | IBD | Female | 38.41 (1.73, 851.31) |
| Andorra | 60 to 64 | IBD | Male | 45.11 (2.00, 1018.16) |
| Andorra | 65 to 69 | IBD | Both | 81.30 (4.40, 1501.46) |
| Andorra | 65 to 69 | IBD | Female | 34.62 (1.53, 783.64) |
| Andorra | 65 to 69 | IBD | Male | 45.31 (1.98, 1039.35) |
| Andorra | 10 to 14 | MS | Both | 0.98 (0.02, 42.14) |
| Andorra | 10 to 14 | MS | Female | 0.99 (0.02, 42.79) |
| Andorra | 10 to 14 | MS | Male | 1.00 (0.02, 43.34) |
| Andorra | 15 to 19 | MS | Both | 0.94 (0.02, 46.49) |
| Andorra | 15 to 19 | MS | Female | 0.99 (0.02, 49.15) |
| Andorra | 15 to 19 | MS | Male | 0.96 (0.02, 48.40) |
| Andorra | 20 to 24 | MS | Both | 7.88 (0.31, 197.93) |
| Andorra | 20 to 24 | MS | Female | 8.45 (0.34, 212.52) |
| Andorra | 20 to 24 | MS | Male | 0.81 (0.01, 47.33) |
| Andorra | 25 to 29 | MS | Both | 16.39 (0.66, 409.23) |
| Andorra | 25 to 29 | MS | Female | 13.37 (0.53, 336.85) |
| Andorra | 25 to 29 | MS | Male | 6.42 (0.21, 200.45) |
| Andorra | 30 to 34 | MS | Both | 26.82 (1.07, 671.23) |
| Andorra | 30 to 34 | MS | Female | 21.51 (0.85, 545.69) |
| Andorra | 30 to 34 | MS | Male | 10.33 (0.32, 335.52) |
| Andorra | 35 to 39 | MS | Both | 36.20 (1.44, 912.00) |

| | | | | |
|---|---|---|---|---|
| Andorra | 35 to 39 | MS | Female | 28.09 (1.10, 720.34) |
| Andorra | 35 to 39 | MS | Male | 12.60 (0.38, 417.18) |
| Andorra | 40 to 44 | MS | Both | 42.77 (1.69, 1082.61) |
| Andorra | 40 to 44 | MS | Female | 34.35 (1.33, 886.40) |
| Andorra | 40 to 44 | MS | Male | 15.06 (0.45, 503.71) |
| Andorra | 45 to 49 | MS | Both | 47.44 (1.86, 1207.24) |
| Andorra | 45 to 49 | MS | Female | 35.06 (1.34, 914.94) |
| Andorra | 45 to 49 | MS | Male | 16.59 (0.49, 562.29) |
| Andorra | 5 to 9 | MS | Both | 1.00 (1.00, 1.00) |
| Andorra | 5 to 9 | MS | Female | 1.00 (1.00, 1.00) |
| Andorra | 5 to 9 | MS | Male | 1.00 (1.00, 1.00) |
| Andorra | 50 to 54 | MS | Both | 49.39 (1.93, 1266.78) |
| Andorra | 50 to 54 | MS | Female | 39.10 (1.48, 1030.61) |
| Andorra | 50 to 54 | MS | Male | 17.37 (0.51, 597.50) |
| Andorra | 55 to 59 | MS | Both | 52.10 (2.01, 1349.37) |
| Andorra | 55 to 59 | MS | Female | 38.94 (1.45, 1042.82) |
| Andorra | 55 to 59 | MS | Male | 16.67 (0.47, 588.56) |
| Andorra | 60 to 64 | MS | Both | 51.60 (1.97, 1353.28) |
| Andorra | 60 to 64 | MS | Female | 38.72 (1.42, 1057.81) |
| Andorra | 60 to 64 | MS | Male | 17.25 (0.47, 627.00) |
| Andorra | 65 to 69 | MS | Both | 50.42 (1.89, 1345.02) |
| Andorra | 65 to 69 | MS | Female | 37.74 (1.35, 1057.96) |
| Andorra | 65 to 69 | MS | Male | 14.30 (0.37, 557.39) |
| Andorra | 0 to 4 | Psoriasis | Both | 0.42 (0.31, 0.59) |
| Andorra | 0 to 4 | Psoriasis | Female | 0.42 (0.26, 0.66) |
| Andorra | 0 to 4 | Psoriasis | Male | 0.42 (0.26, 0.66) |
| Andorra | 10 to 14 | Psoriasis | Both | 1.42 (1.14, 1.77) |
| Andorra | 10 to 14 | Psoriasis | Female | 1.49 (1.10, 2.03) |
| Andorra | 10 to 14 | Psoriasis | Male | 1.38 (1.01, 1.89) |
| Andorra | 15 to 19 | Psoriasis | Both | 1.73 (1.39, 2.16) |
| Andorra | 15 to 19 | Psoriasis | Female | 1.87 (1.38, 2.54) |
| Andorra | 15 to 19 | Psoriasis | Male | 1.59 (1.16, 2.19) |
| Andorra | 20 to 24 | Psoriasis | Both | 2.04 (1.64, 2.54) |

| | | | | |
|---|---|---|---|---|
| Andorra | 20 to 24 | Psoriasis | Female | 2.17 (1.60, 2.96) |
| Andorra | 20 to 24 | Psoriasis | Male | 1.91 (1.39, 2.62) |
| Andorra | 25 to 29 | Psoriasis | Both | 2.49 (2.00, 3.10) |
| Andorra | 25 to 29 | Psoriasis | Female | 2.45 (1.80, 3.33) |
| Andorra | 25 to 29 | Psoriasis | Male | 2.53 (1.85, 3.46) |
| Andorra | 30 to 34 | Psoriasis | Both | 2.93 (2.35, 3.65) |
| Andorra | 30 to 34 | Psoriasis | Female | 2.73 (2.00, 3.72) |
| Andorra | 30 to 34 | Psoriasis | Male | 3.13 (2.29, 4.29) |
| Andorra | 35 to 39 | Psoriasis | Both | 3.28 (2.62, 4.10) |
| Andorra | 35 to 39 | Psoriasis | Female | 2.95 (2.15, 4.04) |
| Andorra | 35 to 39 | Psoriasis | Male | 3.60 (2.62, 4.95) |
| Andorra | 40 to 44 | Psoriasis | Both | 3.56 (2.84, 4.47) |
| Andorra | 40 to 44 | Psoriasis | Female | 3.14 (2.28, 4.33) |
| Andorra | 40 to 44 | Psoriasis | Male | 3.99 (2.89, 5.50) |
| Andorra | 45 to 49 | Psoriasis | Both | 3.86 (3.07, 4.85) |
| Andorra | 45 to 49 | Psoriasis | Female | 3.40 (2.46, 4.72) |
| Andorra | 45 to 49 | Psoriasis | Male | 4.31 (3.12, 5.97) |
| Andorra | 5 to 9 | Psoriasis | Both | 1.00 (1.00, 1.00) |
| Andorra | 5 to 9 | Psoriasis | Female | 1.00 (1.00, 1.00) |
| Andorra | 5 to 9 | Psoriasis | Male | 1.00 (1.00, 1.00) |
| Andorra | 50 to 54 | Psoriasis | Both | 4.21 (3.34, 5.32) |
| Andorra | 50 to 54 | Psoriasis | Female | 3.81 (2.73, 5.30) |
| Andorra | 50 to 54 | Psoriasis | Male | 4.64 (3.34, 6.45) |
| Andorra | 55 to 59 | Psoriasis | Both | 4.52 (3.57, 5.74) |
| Andorra | 55 to 59 | Psoriasis | Female | 4.11 (2.93, 5.76) |
| Andorra | 55 to 59 | Psoriasis | Male | 4.96 (3.55, 6.92) |
| Andorra | 60 to 64 | Psoriasis | Both | 4.68 (3.67, 5.97) |
| Andorra | 60 to 64 | Psoriasis | Female | 4.25 (3.01, 6.02) |
| Andorra | 60 to 64 | Psoriasis | Male | 5.13 (3.64, 7.21) |
| Andorra | 65 to 69 | Psoriasis | Both | 4.70 (3.66, 6.04) |
| Andorra | 65 to 69 | Psoriasis | Female | 4.30 (3.01, 6.15) |
| Andorra | 65 to 69 | Psoriasis | Male | 5.12 (3.60, 7.27) |
| Andorra | 0 to 4 | RHD | Both | 1.07 (0.02, 47.40) |

| | | | | |
|---|---|---|---|---|
| Andorra | 0 to 4 | RHD | Female | 1.10 (0.02, 48.82) |
| Andorra | 0 to 4 | RHD | Male | 1.13 (0.03, 50.38) |
| Andorra | 10 to 14 | RHD | Both | 0.97 (0.02, 39.55) |
| Andorra | 10 to 14 | RHD | Female | 0.98 (0.02, 40.20) |
| Andorra | 10 to 14 | RHD | Male | 0.99 (0.02, 40.32) |
| Andorra | 15 to 19 | RHD | Both | 0.90 (0.02, 42.99) |
| Andorra | 15 to 19 | RHD | Female | 0.95 (0.02, 45.55) |
| Andorra | 15 to 19 | RHD | Male | 1.00 (0.02, 47.76) |
| Andorra | 20 to 24 | RHD | Both | 0.74 (0.01, 42.56) |
| Andorra | 20 to 24 | RHD | Female | 0.82 (0.01, 47.09) |
| Andorra | 20 to 24 | RHD | Male | 0.87 (0.02, 50.25) |
| Andorra | 25 to 29 | RHD | Both | 0.55 (0.01, 38.37) |
| Andorra | 25 to 29 | RHD | Female | 0.64 (0.01, 46.15) |
| Andorra | 25 to 29 | RHD | Male | 0.64 (0.01, 46.08) |
| Andorra | 30 to 34 | RHD | Both | 2.56 (0.04, 148.62) |
| Andorra | 30 to 34 | RHD | Female | 0.58 (0.01, 53.17) |
| Andorra | 30 to 34 | RHD | Male | 0.55 (0.01, 50.93) |
| Andorra | 35 to 39 | RHD | Both | 4.20 (0.08, 221.89) |
| Andorra | 35 to 39 | RHD | Female | 0.60 (0.01, 67.73) |
| Andorra | 35 to 39 | RHD | Male | 0.52 (0.01, 61.08) |
| Andorra | 40 to 44 | RHD | Both | 4.22 (0.08, 236.63) |
| Andorra | 40 to 44 | RHD | Female | 4.48 (0.06, 317.84) |
| Andorra | 40 to 44 | RHD | Male | 1.87 (0.02, 195.46) |
| Andorra | 45 to 49 | RHD | Both | 6.91 (0.12, 386.72) |
| Andorra | 45 to 49 | RHD | Female | 6.88 (0.09, 533.23) |
| Andorra | 45 to 49 | RHD | Male | 4.77 (0.05, 421.56) |
| Andorra | 5 to 9 | RHD | Both | 1.00 (1.00, 1.00) |
| Andorra | 5 to 9 | RHD | Female | 1.00 (1.00, 1.00) |
| Andorra | 5 to 9 | RHD | Male | 1.00 (1.00, 1.00) |
| Andorra | 50 to 54 | RHD | Both | 9.25 (0.16, 525.12) |
| Andorra | 50 to 54 | RHD | Female | 7.64 (0.09, 628.19) |
| Andorra | 50 to 54 | RHD | Male | 5.19 (0.05, 496.94) |
| Andorra | 55 to 59 | RHD | Both | 14.45 (0.25, 828.20) |

| | | | | |
|---|---|---|---|---|
| Andorra | 55 to 59 | RHD | Female | 12.99 (0.16, 1076.99) |
| Andorra | 55 to 59 | RHD | Male | 7.17 (0.07, 737.51) |
| Andorra | 60 to 64 | RHD | Both | 22.25 (0.39, 1284.24) |
| Andorra | 60 to 64 | RHD | Female | 18.56 (0.22, 1559.52) |
| Andorra | 60 to 64 | RHD | Male | 9.61 (0.09, 1010.31) |
| Andorra | 65 to 69 | RHD | Both | 31.81 (0.55, 1844.66) |
| Andorra | 65 to 69 | RHD | Female | 26.33 (0.31, 2221.32) |
| Andorra | 65 to 69 | RHD | Male | 11.68 (0.11, 1249.82) |
| Andorra | 10 to 14 | RA | Both | 13.20 (0.76, 230.54) |
| Andorra | 10 to 14 | RA | Female | 9.80 (0.55, 175.91) |
| Andorra | 10 to 14 | RA | Male | 0.97 (0.02, 41.81) |
| Andorra | 15 to 19 | RA | Both | 30.18 (1.77, 513.25) |
| Andorra | 15 to 19 | RA | Female | 19.18 (1.09, 336.11) |
| Andorra | 15 to 19 | RA | Male | 9.40 (0.44, 199.86) |
| Andorra | 20 to 24 | RA | Both | 43.92 (2.59, 746.10) |
| Andorra | 20 to 24 | RA | Female | 27.79 (1.59, 484.39) |
| Andorra | 20 to 24 | RA | Male | 9.48 (0.43, 208.60) |
| Andorra | 25 to 29 | RA | Both | 62.99 (3.72, 1067.46) |
| Andorra | 25 to 29 | RA | Female | 38.26 (2.20, 665.25) |
| Andorra | 25 to 29 | RA | Male | 14.24 (0.66, 307.71) |
| Andorra | 30 to 34 | RA | Both | 82.86 (4.88, 1406.11) |
| Andorra | 30 to 34 | RA | Female | 49.60 (2.85, 863.95) |
| Andorra | 30 to 34 | RA | Male | 17.76 (0.82, 385.67) |
| Andorra | 35 to 39 | RA | Both | 108.55 (6.38, 1845.51) |
| Andorra | 35 to 39 | RA | Female | 67.30 (3.85, 1175.33) |
| Andorra | 35 to 39 | RA | Male | 25.19 (1.16, 549.27) |
| Andorra | 40 to 44 | RA | Both | 147.42 (8.66, 2509.53) |
| Andorra | 40 to 44 | RA | Female | 88.81 (5.08, 1553.64) |
| Andorra | 40 to 44 | RA | Male | 33.00 (1.51, 721.97) |
| Andorra | 45 to 49 | RA | Both | 197.64 (11.59, 3369.67) |
| Andorra | 45 to 49 | RA | Female | 116.23 (6.63, 2037.96) |
| Andorra | 45 to 49 | RA | Male | 45.55 (2.07, 1000.28) |
| Andorra | 5 to 9 | RA | Both | 1.00 (1.00, 1.00) |

| | | | | |
|---|---|---|---|---|
| Andorra | 5 to 9 | RA | Female | 1.00 (1.00, 1.00) |
| Andorra | 5 to 9 | RA | Male | 1.00 (1.00, 1.00) |
| Andorra | 50 to 54 | RA | Both | 258.10 (15.11, 4408.27) |
| Andorra | 50 to 54 | RA | Female | 148.10 (8.42, 2603.92) |
| Andorra | 50 to 54 | RA | Male | 61.07 (2.77, 1347.34) |
| Andorra | 55 to 59 | RA | Both | 330.81 (19.33, 5661.59) |
| Andorra | 55 to 59 | RA | Female | 185.27 (10.50, 3267.42) |
| Andorra | 55 to 59 | RA | Male | 82.36 (3.71, 1826.76) |
| Andorra | 60 to 64 | RA | Both | 417.19 (24.33, 7154.09) |
| Andorra | 60 to 64 | RA | Female | 231.63 (13.10, 4096.90) |
| Andorra | 60 to 64 | RA | Male | 106.77 (4.79, 2380.59) |
| Andorra | 65 to 69 | RA | Both | 496.03 (28.87, 8522.21) |
| Andorra | 65 to 69 | RA | Female | 268.44 (15.13, 4761.97) |
| Andorra | 65 to 69 | RA | Male | 132.06 (5.89, 2960.45) |
| Angola | 0 to 4 | AA | Both | 0.71 (0.69, 0.73) |
| Angola | 0 to 4 | AA | Female | 0.72 (0.69, 0.74) |
| Angola | 0 to 4 | AA | Male | 0.70 (0.67, 0.73) |
| Angola | 10 to 14 | AA | Both | 1.29 (1.26, 1.32) |
| Angola | 10 to 14 | AA | Female | 1.27 (1.23, 1.31) |
| Angola | 10 to 14 | AA | Male | 1.32 (1.26, 1.38) |
| Angola | 15 to 19 | AA | Both | 2.26 (2.20, 2.32) |
| Angola | 15 to 19 | AA | Female | 2.22 (2.15, 2.30) |
| Angola | 15 to 19 | AA | Male | 2.27 (2.18, 2.38) |
| Angola | 20 to 24 | AA | Both | 3.53 (3.43, 3.62) |
| Angola | 20 to 24 | AA | Female | 3.48 (3.37, 3.60) |
| Angola | 20 to 24 | AA | Male | 3.46 (3.31, 3.62) |
| Angola | 25 to 29 | AA | Both | 4.93 (4.80, 5.07) |
| Angola | 25 to 29 | AA | Female | 4.86 (4.70, 5.02) |
| Angola | 25 to 29 | AA | Male | 4.83 (4.61, 5.06) |
| Angola | 30 to 34 | AA | Both | 5.25 (5.10, 5.40) |
| Angola | 30 to 34 | AA | Female | 5.17 (4.99, 5.36) |
| Angola | 30 to 34 | AA | Male | 5.13 (4.88, 5.38) |
| Angola | 35 to 39 | AA | Both | 4.94 (4.79, 5.09) |

| | | | | |
|---|---|---|---|---|
| Angola | 35 to 39 | AA | Female | 4.85 (4.67, 5.04) |
| Angola | 35 to 39 | AA | Male | 4.81 (4.57, 5.07) |
| Angola | 40 to 44 | AA | Both | 4.20 (4.06, 4.34) |
| Angola | 40 to 44 | AA | Female | 4.10 (3.94, 4.27) |
| Angola | 40 to 44 | AA | Male | 4.12 (3.89, 4.35) |
| Angola | 45 to 49 | AA | Both | 3.62 (3.49, 3.76) |
| Angola | 45 to 49 | AA | Female | 3.53 (3.38, 3.70) |
| Angola | 45 to 49 | AA | Male | 3.52 (3.31, 3.74) |
| Angola | 5 to 9 | AA | Both | 1.00 (1.00, 1.00) |
| Angola | 5 to 9 | AA | Female | 1.00 (1.00, 1.00) |
| Angola | 5 to 9 | AA | Male | 1.00 (1.00, 1.00) |
| Angola | 50 to 54 | AA | Both | 3.26 (3.14, 3.40) |
| Angola | 50 to 54 | AA | Female | 3.18 (3.03, 3.34) |
| Angola | 50 to 54 | AA | Male | 3.12 (2.91, 3.34) |
| Angola | 55 to 59 | AA | Both | 3.10 (2.96, 3.24) |
| Angola | 55 to 59 | AA | Female | 2.98 (2.82, 3.15) |
| Angola | 55 to 59 | AA | Male | 2.99 (2.77, 3.23) |
| Angola | 60 to 64 | AA | Both | 3.26 (3.10, 3.43) |
| Angola | 60 to 64 | AA | Female | 3.12 (2.94, 3.32) |
| Angola | 60 to 64 | AA | Male | 3.10 (2.85, 3.39) |
| Angola | 65 to 69 | AA | Both | 3.05 (2.87, 3.24) |
| Angola | 65 to 69 | AA | Female | 2.87 (2.67, 3.09) |
| Angola | 65 to 69 | AA | Male | 2.90 (2.61, 3.22) |
| Angola | 0 to 4 | IBD | Both | 0.06 (0.01, 0.34) |
| Angola | 0 to 4 | IBD | Female | 0.03 (0.01, 0.53) |
| Angola | 0 to 4 | IBD | Male | 0.03 (0.01, 0.51) |
| Angola | 10 to 14 | IBD | Both | 3.52 (2.29, 5.42) |
| Angola | 10 to 14 | IBD | Female | 3.50 (1.90, 6.43) |
| Angola | 10 to 14 | IBD | Male | 3.36 (1.82, 6.20) |
| Angola | 15 to 19 | IBD | Both | 9.59 (6.27, 14.66) |
| Angola | 15 to 19 | IBD | Female | 10.57 (5.82, 19.18) |
| Angola | 15 to 19 | IBD | Male | 7.98 (4.34, 14.65) |
| Angola | 20 to 24 | IBD | Both | 18.61 (12.16, 28.48) |

| | | | | |
|---|---|---|---|---|
| Angola | 20 to 24 | IBD | Female | 20.40 (11.23, 37.07) |
| Angola | 20 to 24 | IBD | Male | 15.45 (8.40, 28.42) |
| Angola | 25 to 29 | IBD | Both | 30.80 (20.06, 47.29) |
| Angola | 25 to 29 | IBD | Female | 33.57 (18.43, 61.15) |
| Angola | 25 to 29 | IBD | Male | 26.10 (14.15, 48.13) |
| Angola | 30 to 34 | IBD | Both | 46.18 (29.96, 71.17) |
| Angola | 30 to 34 | IBD | Female | 49.35 (26.97, 90.31) |
| Angola | 30 to 34 | IBD | Male | 39.11 (21.09, 72.53) |
| Angola | 35 to 39 | IBD | Both | 63.92 (41.32, 98.87) |
| Angola | 35 to 39 | IBD | Female | 67.82 (36.88, 124.73) |
| Angola | 35 to 39 | IBD | Male | 55.09 (29.53, 102.78) |
| Angola | 40 to 44 | IBD | Both | 83.11 (53.54, 129.01) |
| Angola | 40 to 44 | IBD | Female | 87.43 (47.33, 161.50) |
| Angola | 40 to 44 | IBD | Male | 71.46 (38.10, 134.01) |
| Angola | 45 to 49 | IBD | Both | 102.33 (65.69, 159.42) |
| Angola | 45 to 49 | IBD | Female | 108.16 (58.29, 200.70) |
| Angola | 45 to 49 | IBD | Male | 87.64 (46.47, 165.30) |
| Angola | 5 to 9 | IBD | Both | 1.00 (1.00, 1.00) |
| Angola | 5 to 9 | IBD | Female | 1.00 (1.00, 1.00) |
| Angola | 5 to 9 | IBD | Male | 1.00 (1.00, 1.00) |
| Angola | 50 to 54 | IBD | Both | 106.41 (67.94, 166.66) |
| Angola | 50 to 54 | IBD | Female | 102.37 (54.74, 191.43) |
| Angola | 50 to 54 | IBD | Male | 101.30 (53.33, 192.38) |
| Angola | 55 to 59 | IBD | Both | 110.34 (70.02, 173.88) |
| Angola | 55 to 59 | IBD | Female | 102.27 (54.22, 192.91) |
| Angola | 55 to 59 | IBD | Male | 108.91 (56.84, 208.69) |
| Angola | 60 to 64 | IBD | Both | 112.01 (70.49, 177.96) |
| Angola | 60 to 64 | IBD | Female | 102.78 (53.87, 196.10) |
| Angola | 60 to 64 | IBD | Male | 110.38 (56.91, 214.11) |
| Angola | 65 to 69 | IBD | Both | 107.52 (66.72, 173.25) |
| Angola | 65 to 69 | IBD | Female | 99.45 (51.17, 193.30) |
| Angola | 65 to 69 | IBD | Male | 109.23 (55.19, 216.21) |
| Angola | 10 to 14 | MS | Both | 7.92 (3.40, 18.45) |

| | | | | |
|---|---|---|---|---|
| Angola | 10 to 14 | MS | Female | 6.27 (2.24, 17.60) |
| Angola | 10 to 14 | MS | Male | 5.94 (1.48, 23.85) |
| Angola | 15 to 19 | MS | Both | 25.08 (10.82, 58.17) |
| Angola | 15 to 19 | MS | Female | 20.62 (7.50, 56.64) |
| Angola | 15 to 19 | MS | Male | 19.09 (4.70, 77.59) |
| Angola | 20 to 24 | MS | Both | 54.41 (23.51, 125.92) |
| Angola | 20 to 24 | MS | Female | 44.24 (16.10, 121.53) |
| Angola | 20 to 24 | MS | Male | 39.67 (9.60, 163.99) |
| Angola | 25 to 29 | MS | Both | 96.61 (41.69, 223.83) |
| Angola | 25 to 29 | MS | Female | 79.80 (28.96, 219.91) |
| Angola | 25 to 29 | MS | Male | 68.37 (16.50, 283.19) |
| Angola | 30 to 34 | MS | Both | 142.75 (61.46, 331.58) |
| Angola | 30 to 34 | MS | Female | 118.05 (42.64, 326.80) |
| Angola | 30 to 34 | MS | Male | 100.58 (24.18, 418.42) |
| Angola | 35 to 39 | MS | Both | 186.12 (79.92, 433.49) |
| Angola | 35 to 39 | MS | Female | 153.76 (55.38, 426.91) |
| Angola | 35 to 39 | MS | Male | 131.32 (31.44, 548.53) |
| Angola | 40 to 44 | MS | Both | 222.08 (95.12, 518.52) |
| Angola | 40 to 44 | MS | Female | 181.97 (65.32, 506.94) |
| Angola | 40 to 44 | MS | Male | 157.51 (37.53, 661.01) |
| Angola | 45 to 49 | MS | Both | 245.31 (104.78, 574.33) |
| Angola | 45 to 49 | MS | Female | 199.71 (71.42, 558.44) |
| Angola | 45 to 49 | MS | Male | 174.71 (41.42, 736.92) |
| Angola | 5 to 9 | MS | Both | 1.00 (1.00, 1.00) |
| Angola | 5 to 9 | MS | Female | 1.00 (1.00, 1.00) |
| Angola | 5 to 9 | MS | Male | 1.00 (1.00, 1.00) |
| Angola | 50 to 54 | MS | Both | 255.24 (108.63, 599.72) |
| Angola | 50 to 54 | MS | Female | 207.53 (73.87, 582.99) |
| Angola | 50 to 54 | MS | Male | 178.68 (42.09, 758.47) |
| Angola | 55 to 59 | MS | Both | 244.71 (103.60, 578.03) |
| Angola | 55 to 59 | MS | Female | 200.33 (70.84, 566.47) |
| Angola | 55 to 59 | MS | Male | 169.99 (39.67, 728.38) |
| Angola | 60 to 64 | MS | Both | 217.81 (91.42, 518.97) |

| | | | | |
|---|---|---|---|---|
| Angola | 60 to 64 | MS | Female | 178.65 (62.53, 510.40) |
| Angola | 60 to 64 | MS | Male | 146.01 (33.51, 636.20) |
| Angola | 65 to 69 | MS | Both | 185.05 (76.38, 448.34) |
| Angola | 65 to 69 | MS | Female | 154.15 (52.96, 448.72) |
| Angola | 65 to 69 | MS | Male | 115.28 (25.52, 520.79) |
| Angola | 0 to 4 | Psoriasis | Both | 0.39 (0.38, 0.40) |
| Angola | 0 to 4 | Psoriasis | Female | 0.39 (0.38, 0.40) |
| Angola | 0 to 4 | Psoriasis | Male | 0.39 (0.38, 0.40) |
| Angola | 10 to 14 | Psoriasis | Both | 1.25 (1.24, 1.27) |
| Angola | 10 to 14 | Psoriasis | Female | 1.25 (1.23, 1.28) |
| Angola | 10 to 14 | Psoriasis | Male | 1.25 (1.23, 1.28) |
| Angola | 15 to 19 | Psoriasis | Both | 1.35 (1.33, 1.37) |
| Angola | 15 to 19 | Psoriasis | Female | 1.34 (1.31, 1.37) |
| Angola | 15 to 19 | Psoriasis | Male | 1.35 (1.32, 1.38) |
| Angola | 20 to 24 | Psoriasis | Both | 1.40 (1.38, 1.42) |
| Angola | 20 to 24 | Psoriasis | Female | 1.39 (1.36, 1.42) |
| Angola | 20 to 24 | Psoriasis | Male | 1.41 (1.38, 1.44) |
| Angola | 25 to 29 | Psoriasis | Both | 1.49 (1.47, 1.52) |
| Angola | 25 to 29 | Psoriasis | Female | 1.48 (1.44, 1.52) |
| Angola | 25 to 29 | Psoriasis | Male | 1.50 (1.47, 1.54) |
| Angola | 30 to 34 | Psoriasis | Both | 1.60 (1.57, 1.63) |
| Angola | 30 to 34 | Psoriasis | Female | 1.58 (1.54, 1.63) |
| Angola | 30 to 34 | Psoriasis | Male | 1.61 (1.56, 1.65) |
| Angola | 35 to 39 | Psoriasis | Both | 1.67 (1.63, 1.70) |
| Angola | 35 to 39 | Psoriasis | Female | 1.65 (1.61, 1.70) |
| Angola | 35 to 39 | Psoriasis | Male | 1.67 (1.63, 1.72) |
| Angola | 40 to 44 | Psoriasis | Both | 1.72 (1.68, 1.75) |
| Angola | 40 to 44 | Psoriasis | Female | 1.71 (1.66, 1.76) |
| Angola | 40 to 44 | Psoriasis | Male | 1.73 (1.67, 1.78) |
| Angola | 45 to 49 | Psoriasis | Both | 1.77 (1.73, 1.81) |
| Angola | 45 to 49 | Psoriasis | Female | 1.76 (1.70, 1.81) |
| Angola | 45 to 49 | Psoriasis | Male | 1.78 (1.72, 1.84) |
| Angola | 5 to 9 | Psoriasis | Both | 1.00 (1.00, 1.00) |

| | | | | |
|---|---|---|---|---|
| Angola | 5 to 9 | Psoriasis | Female | 1.00 (1.00, 1.00) |
| Angola | 5 to 9 | Psoriasis | Male | 1.00 (1.00, 1.00) |
| Angola | 50 to 54 | Psoriasis | Both | 1.86 (1.82, 1.91) |
| Angola | 50 to 54 | Psoriasis | Female | 1.85 (1.79, 1.91) |
| Angola | 50 to 54 | Psoriasis | Male | 1.87 (1.81, 1.94) |
| Angola | 55 to 59 | Psoriasis | Both | 1.94 (1.89, 1.99) |
| Angola | 55 to 59 | Psoriasis | Female | 1.92 (1.85, 2.00) |
| Angola | 55 to 59 | Psoriasis | Male | 1.95 (1.87, 2.02) |
| Angola | 60 to 64 | Psoriasis | Both | 1.92 (1.87, 1.98) |
| Angola | 60 to 64 | Psoriasis | Female | 1.91 (1.83, 1.98) |
| Angola | 60 to 64 | Psoriasis | Male | 1.93 (1.85, 2.01) |
| Angola | 65 to 69 | Psoriasis | Both | 1.82 (1.76, 1.88) |
| Angola | 65 to 69 | Psoriasis | Female | 1.80 (1.72, 1.88) |
| Angola | 65 to 69 | Psoriasis | Male | 1.83 (1.74, 1.92) |
| Angola | 0 to 4 | RHD | Both | 0.15 (0.15, 0.15) |
| Angola | 0 to 4 | RHD | Female | 0.15 (0.15, 0.16) |
| Angola | 0 to 4 | RHD | Male | 0.15 (0.15, 0.16) |
| Angola | 10 to 14 | RHD | Both | 2.31 (2.28, 2.33) |
| Angola | 10 to 14 | RHD | Female | 2.36 (2.33, 2.39) |
| Angola | 10 to 14 | RHD | Male | 2.26 (2.23, 2.28) |
| Angola | 15 to 19 | RHD | Both | 3.33 (3.30, 3.36) |
| Angola | 15 to 19 | RHD | Female | 3.55 (3.50, 3.60) |
| Angola | 15 to 19 | RHD | Male | 3.13 (3.09, 3.17) |
| Angola | 20 to 24 | RHD | Both | 3.61 (3.58, 3.65) |
| Angola | 20 to 24 | RHD | Female | 3.91 (3.86, 3.96) |
| Angola | 20 to 24 | RHD | Male | 3.34 (3.30, 3.39) |
| Angola | 25 to 29 | RHD | Both | 3.61 (3.57, 3.65) |
| Angola | 25 to 29 | RHD | Female | 3.93 (3.88, 3.99) |
| Angola | 25 to 29 | RHD | Male | 3.31 (3.26, 3.36) |
| Angola | 30 to 34 | RHD | Both | 3.48 (3.44, 3.52) |
| Angola | 30 to 34 | RHD | Female | 3.81 (3.76, 3.88) |
| Angola | 30 to 34 | RHD | Male | 3.18 (3.13, 3.23) |
| Angola | 35 to 39 | RHD | Both | 3.26 (3.22, 3.30) |

| | | | | |
|---|---|---|---|---|
| Angola | 35 to 39 | RHD | Female | 3.58 (3.52, 3.64) |
| Angola | 35 to 39 | RHD | Male | 2.96 (2.91, 3.01) |
| Angola | 40 to 44 | RHD | Both | 2.95 (2.91, 2.98) |
| Angola | 40 to 44 | RHD | Female | 3.25 (3.19, 3.31) |
| Angola | 40 to 44 | RHD | Male | 2.66 (2.62, 2.71) |
| Angola | 45 to 49 | RHD | Both | 2.53 (2.49, 2.56) |
| Angola | 45 to 49 | RHD | Female | 2.80 (2.75, 2.86) |
| Angola | 45 to 49 | RHD | Male | 2.27 (2.22, 2.32) |
| Angola | 5 to 9 | RHD | Both | 1.00 (1.00, 1.00) |
| Angola | 5 to 9 | RHD | Female | 1.00 (1.00, 1.00) |
| Angola | 5 to 9 | RHD | Male | 1.00 (1.00, 1.00) |
| Angola | 50 to 54 | RHD | Both | 2.05 (2.02, 2.08) |
| Angola | 50 to 54 | RHD | Female | 2.29 (2.24, 2.35) |
| Angola | 50 to 54 | RHD | Male | 1.82 (1.77, 1.86) |
| Angola | 55 to 59 | RHD | Both | 1.64 (1.61, 1.68) |
| Angola | 55 to 59 | RHD | Female | 1.86 (1.81, 1.91) |
| Angola | 55 to 59 | RHD | Male | 1.44 (1.40, 1.48) |
| Angola | 60 to 64 | RHD | Both | 1.32 (1.29, 1.36) |
| Angola | 60 to 64 | RHD | Female | 1.51 (1.46, 1.56) |
| Angola | 60 to 64 | RHD | Male | 1.14 (1.10, 1.19) |
| Angola | 65 to 69 | RHD | Both | 1.07 (1.03, 1.10) |
| Angola | 65 to 69 | RHD | Female | 1.22 (1.17, 1.28) |
| Angola | 65 to 69 | RHD | Male | 0.91 (0.87, 0.96) |
| Angola | 10 to 14 | RA | Both | 7.76 (6.25, 9.64) |
| Angola | 10 to 14 | RA | Female | 8.10 (6.20, 10.58) |
| Angola | 10 to 14 | RA | Male | 7.10 (4.91, 10.27) |
| Angola | 15 to 19 | RA | Both | 24.68 (19.92, 30.58) |
| Angola | 15 to 19 | RA | Female | 26.53 (20.36, 34.56) |
| Angola | 15 to 19 | RA | Male | 20.43 (14.16, 29.47) |
| Angola | 20 to 24 | RA | Both | 53.30 (43.02, 66.04) |
| Angola | 20 to 24 | RA | Female | 57.69 (44.29, 75.16) |
| Angola | 20 to 24 | RA | Male | 41.92 (29.06, 60.46) |
| Angola | 25 to 29 | RA | Both | 95.20 (76.81, 117.99) |

| | | | | |
|---|---|---|---|---|
| Angola | 25 to 29 | RA | Female | 103.07 (79.08, 134.33) |
| Angola | 25 to 29 | RA | Male | 74.26 (51.44, 107.19) |
| Angola | 30 to 34 | RA | Both | 154.86 (124.87, 192.04) |
| Angola | 30 to 34 | RA | Female | 165.50 (126.91, 215.83) |
| Angola | 30 to 34 | RA | Male | 123.86 (85.73, 178.96) |
| Angola | 35 to 39 | RA | Both | 245.82 (198.12, 305.00) |
| Angola | 35 to 39 | RA | Female | 253.07 (193.94, 330.25) |
| Angola | 35 to 39 | RA | Male | 213.00 (147.32, 307.98) |
| Angola | 40 to 44 | RA | Both | 376.34 (303.19, 467.13) |
| Angola | 40 to 44 | RA | Female | 369.15 (282.74, 481.99) |
| Angola | 40 to 44 | RA | Male | 358.91 (248.08, 519.26) |
| Angola | 45 to 49 | RA | Both | 566.99 (456.61, 704.06) |
| Angola | 45 to 49 | RA | Female | 517.31 (395.99, 675.79) |
| Angola | 45 to 49 | RA | Male | 607.63 (419.76, 879.59) |
| Angola | 5 to 9 | RA | Both | 1.00 (1.00, 1.00) |
| Angola | 5 to 9 | RA | Female | 1.00 (1.00, 1.00) |
| Angola | 5 to 9 | RA | Male | 1.00 (1.00, 1.00) |
| Angola | 50 to 54 | RA | Both | 809.38 (651.52, 1005.49) |
| Angola | 50 to 54 | RA | Female | 690.34 (528.13, 902.37) |
| Angola | 50 to 54 | RA | Male | 949.96 (655.81, 1376.04) |
| Angola | 55 to 59 | RA | Both | 1037.89 (835.04, 1290.02) |
| Angola | 55 to 59 | RA | Female | 848.66 (648.79, 1110.10) |
| Angola | 55 to 59 | RA | Male | 1281.53 (884.08, 1857.66) |
| Angola | 60 to 64 | RA | Both | 1230.66 (989.59, 1530.45) |
| Angola | 60 to 64 | RA | Female | 978.53 (747.49, 1280.97) |
| Angola | 60 to 64 | RA | Male | 1562.50 (1077.12, 2266.61) |
| Angola | 65 to 69 | RA | Both | 1338.78 (1075.75, 1666.13) |
| Angola | 65 to 69 | RA | Female | 1048.50 (800.09, 1374.03) |
| Angola | 65 to 69 | RA | Male | 1722.40 (1186.20, 2500.96) |
| Antigua and Barbuda | 0 to 4 | AA | Both | 0.76 (0.43, 1.36) |
| Antigua and Barbuda | 0 to 4 | AA | Female | 0.69 (0.34, 1.42) |
| Antigua and Barbuda | 0 to 4 | AA | Male | 0.50 (0.18, 1.43) |
| Antigua and Barbuda | 10 to 14 | AA | Both | 1.26 (0.77, 2.05) |

| | | | | |
|---|---|---|---|---|
| Antigua and Barbuda | 10 to 14 | AA | Female | 1.25 (0.69, 2.25) |
| Antigua and Barbuda | 10 to 14 | AA | Male | 1.11 (0.49, 2.50) |
| Antigua and Barbuda | 15 to 19 | AA | Both | 2.22 (1.40, 3.53) |
| Antigua and Barbuda | 15 to 19 | AA | Female | 2.11 (1.21, 3.69) |
| Antigua and Barbuda | 15 to 19 | AA | Male | 1.93 (0.89, 4.15) |
| Antigua and Barbuda | 20 to 24 | AA | Both | 3.51 (2.22, 5.55) |
| Antigua and Barbuda | 20 to 24 | AA | Female | 3.31 (1.90, 5.76) |
| Antigua and Barbuda | 20 to 24 | AA | Male | 3.00 (1.40, 6.44) |
| Antigua and Barbuda | 25 to 29 | AA | Both | 4.87 (3.07, 7.73) |
| Antigua and Barbuda | 25 to 29 | AA | Female | 4.57 (2.61, 8.00) |
| Antigua and Barbuda | 25 to 29 | AA | Male | 4.23 (1.96, 9.13) |
| Antigua and Barbuda | 30 to 34 | AA | Both | 5.28 (3.29, 8.48) |
| Antigua and Barbuda | 30 to 34 | AA | Female | 4.92 (2.77, 8.73) |
| Antigua and Barbuda | 30 to 34 | AA | Male | 4.51 (2.04, 9.96) |
| Antigua and Barbuda | 35 to 39 | AA | Both | 4.94 (3.03, 8.05) |
| Antigua and Barbuda | 35 to 39 | AA | Female | 4.66 (2.58, 8.41) |
| Antigua and Barbuda | 35 to 39 | AA | Male | 4.30 (1.90, 9.76) |
| Antigua and Barbuda | 40 to 44 | AA | Both | 4.14 (2.50, 6.86) |
| Antigua and Barbuda | 40 to 44 | AA | Female | 3.90 (2.12, 7.19) |
| Antigua and Barbuda | 40 to 44 | AA | Male | 3.72 (1.59, 8.68) |
| Antigua and Barbuda | 45 to 49 | AA | Both | 3.61 (2.13, 6.10) |
| Antigua and Barbuda | 45 to 49 | AA | Female | 3.42 (1.81, 6.48) |
| Antigua and Barbuda | 45 to 49 | AA | Male | 3.13 (1.28, 7.62) |
| Antigua and Barbuda | 5 to 9 | AA | Both | 1.00 (1.00, 1.00) |
| Antigua and Barbuda | 5 to 9 | AA | Female | 1.00 (1.00, 1.00) |
| Antigua and Barbuda | 5 to 9 | AA | Male | 1.00 (1.00, 1.00) |
| Antigua and Barbuda | 50 to 54 | AA | Both | 3.29 (1.89, 5.73) |
| Antigua and Barbuda | 50 to 54 | AA | Female | 3.01 (1.53, 5.92) |
| Antigua and Barbuda | 50 to 54 | AA | Male | 2.96 (1.15, 7.63) |
| Antigua and Barbuda | 55 to 59 | AA | Both | 2.99 (1.65, 5.44) |
| Antigua and Barbuda | 55 to 59 | AA | Female | 2.91 (1.41, 6.00) |
| Antigua and Barbuda | 55 to 59 | AA | Male | 2.75 (0.99, 7.61) |
[truncated: 614,237 more chars]
